# Response of human metabolism to ultra-low and high nicotine cigarettes based on urine metabolomics and bioinformatic analysis

**DOI:** 10.18332/tid/196677

**Published:** 2024-12-18

**Authors:** Mengyue Zhang, Chunting Yang, Lingling Gao, Yuanyuan Zhao, Hongzhi Shi

**Affiliations:** 1 College of Tobacco Science, Henan Agricultural University, Zhengzhou, China; 2 Henan Academy of Agricultural Sciences, Zhengzhou, China

**Keywords:** cigarette, nicotine, untargeted metabolomics, bioinformatics

## Abstract

**INTRODUCTION:**

This study aimed to evaluate the metabolomic profiles of urine samples obtained from smokers who smoked cigarettes with low and high nicotine content.

**METHODS:**

Three smokers participated in this study. They were given low-nicotine (LN) cigarettes, and urine was collected at the end of the third day for the LN group. After 1 week of not smoking, they were given high-nicotine (HN) cigarettes, and urine was collected for the HN group. Untargeted metabolomics and bioinformatic analysis methods were used for urine analysis.

**RESULTS:**

PCA showed a high degree of similarity between samples within the group and a large distance between samples between groups, indicating a significant difference between the two groups. A total of 1150 significantly differential metabolites were selected between the HN and LN groups, such as cotinine and 4-(methylnitrosamino)-1-(3-pyridyl)-1-butanol-N-glucuronide. Two-way hierarchical clustering analysis also suggested noticeable differences between the two comparison groups Enrichment analysis indicates that the differential metabolites between the two groups were mainly enriched in 19 pathways, such as the protein kinase G (cGMP)-protein kinase G (PKG) signaling pathway, adenosine monophosphate (AMP)-activated protein kinase signaling pathway, mammalian target of rapamycin signaling pathway, and Parkinson’s disease.

**CONCLUSIONS:**

Cigarettes with different nicotine content may alter the metabolism of smokers. A total of 1150 significantly different metabolites were identified between the HN and LN groups, which were mainly enriched in ABC transporters, protein kinase G (cGMP)-protein kinase G (PKG) signaling pathway, caffeine metabolism, and arginine biosynthesis pathways.

## INTRODUCTION

Alkaloids are a group of nitrogenous secondary metabolites widely distributed in the plant kingdom, particularly in *Nicotiana*. Among these alkaloids, nicotines are the most important, accounting for more than 90% of total alkaloids^1^. The nicotine released by tobacco leaves during smoking enters the brain cells through the blood and makes smoking pleasurable. Therefore, nicotine is considered the primary active ingredient in tobacco leaves. In 1994, Benowitz et al.^2^ first proposed that reducing the nicotine content of tobacco leaves below the critical value of addiction could effectively reduce the dependence of smokers on tobacco. The World Health Organization, to reduce smoking rates, recommends reducing the nicotine content of cigarette cuts to less than 0.4 mg/g (between 0.2 and 0.3 mg/g)^3^. Recently, the nicotine content in cigarettes has been reduced to even lower levels with the development of technology^4^.

It has been reported that the metabolism and uptake of nicotine and derived carcinogens may contribute to the occurrence of cancers^5^. Due to the different nicotine content of cigarettes, the metabolites of nicotine, such as cotinine, vary greatly after smoking. As a result, the impact of smoking on the human body may differ. To the best of our knowledge, this is the first study to evaluate the response of human metabolism to cigarette smoking with different nicotine concentrations.

Mass spectrometry-based metabolomics is an emerging technique for assessing the physiological responses related to metabolic disorders, toxic effects, and drug action^6^. Several analytical techniques have been used to detect different metabolites. With the development of more advanced bioinformatics, instrumentation platforms, and spectral databases, the applicability of metabolomics to address various biologically relevant problems is rapidly expanding^7^. For example, many metabolomics platforms have been used to study the patterns and changes in metabolite alterations between non-smokers and smokers^8^. Dator et al.^9^ performed metabolomic profiling of urine samples of smokers from two ethnic groups to characterize metabolite patterns and differentially regulated pathways. Their study found that African American smokers have lower glucuronidation ability compared to White smokers, and there are significant differences in the d-glucuronic acid degradation pathway between the two races. In this study, we aimed to elucidate the effect of cigarettes with different nicotine content on human metabolism through untargeted metabolomics.

## METHODS

### Study design

**Figure 1:**
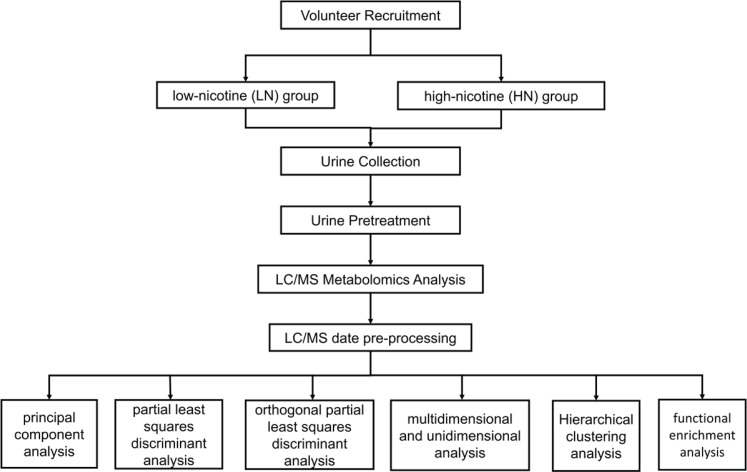
Study design and workflow

Figure 1 shows the overall design of the study and provides a detailed explanation of the analysis method.

### Cigarettes

All test cigarettes were hand-rolled using tobacco leaves of Zhongyan 100 that was obtained from a grafting test in the field. The nicotine contents in low-nicotine (LN) and high-nicotine (HN) cigarettes were 0.08% and 2.00%, respectively.

### Participants

The participants completed a brief questionnaire on smoking behavior. The questionnaire included: age, smoking years, daily number of cigarettes, dependence on nicotine, health status, with underlying diseases or not, with special hobbies or not, etc. Male smokers who smoked for >5 years, smoked about 10 cigarettes a day, had low nicotine dependence, were in good health, had no underlying diseases, and had no alcohol habit were selected as participants. Three were selected to participate in the study. All three participants were informed of the experimental requirements and signed an informed consent form. Information on the three participants is presented in Supplementary file Table S1. All three participants were males and had been smoking for >10 years. This study was approved by the Ethics Committee of Henan Agricultural University (ethics number: HNND20221118001).

### Smoking experiment

Before the experiment, the three participants were instructed not to smoke for 1 week. They were given low-nicotine cigarettes and asked to smoke only those cigarettes for the next 3 days (10 cigarettes per day: they smoked one cigarette every 1.5 hours starting at 7 am each day.). Urine was collected at the end of the third day at 5 p.m. for the LN group. After 1 week of not smoking, they were given high-nicotine cigarettes and asked to smoke only those cigarettes for the next 3 days (10 cigarettes per day). Urine was collected at 5 p.m. on the third day for the HN group. The weight of the tobacco shred was the same for each cigarette. Additionally, the number of puffs per person and the amount of smoke per puff remained the same (each cigarette was prescribed to be smoked in 8 puffs with a one-minute interval between two puffs and 2 seconds for each puff. The smoke needed to fill the mouth).

### Urine collection

The three participants were given guidelines regarding the method of urine collection. Urine (30 mL) was collected at 5 p.m. on the third day and was used for liquid chromatography-mass spectrometry (LC/MS) analysis. The samples were frozen in liquid nitrogen and stored at -80^o^C.

### Urine pretreatment

Prior to the urinary analysis, the stored urine was thawed; 100 μL of urine was added to a 1.5 mL Eppendorf tube with 20 μL L-2-chlorophenylalanine (0.06 mg/mL, methanol solution) (Shanghai Hengchuang Biotechnology Co. LTD, Shanghai, China) and 300 μL methanol-acetonitrile solution (2/1, v/v) (Thermo Fisher Scientific, Waltham, MA, USA). The mixture was vortexed for 1 min, followed by ultrasonic extraction for 10 min in an ice-water bath. The extract was placed at -20^o^C for 30 min. After centrifugation (4^o^C, 13000 rpm) for 10 min, 350 μL of the supernatant was collected and dried in a freeze-concentration centrifugal dryer (Taicang Huamei Biochemical instrument factory, Suzhou, China). Next, 300 μL of a methanol–water mixture (1/4, v/v) was added to each sample and re-dissolved (vortexed for 30 min and ultrasonicated for 3 min). The solution was placed at -20^o^C for 2 h. Next, the solutions were centrifuged again (4^o^C, 13000 rpm), and supernatants (150 μL) were collected, filtered, transferred to vials for liquid chromatography, and stored at -80^o^C.

### LC/MS metabolomics analysis

An ACQUITY UPLC I-Class Plus ultra-performance liquid tandem QE high-resolution mass spectrometer (Waters Corporation, Milford, MA, USA) was used for the metabolic profiling analysis. An ACQUITY UPLC HSS T3 column (100 mm × 2.1 nm, 1.8 μm) (Waters Corporation, Milford, MA, USA) was used. Solvent A was water (0.1% formic acid) (Thermo Fisher Scientific, Waltham, MA, USA). Solvent B was acetonitrile (0.1% formic acid) (Thermo Fisher Scientific, Waltham, MA, USA). The linear gradients were 0 min of 5% B, 2 min of 5% B, 4 min of 30% B, 8 min of 50% B, 10 min of 80% B, 14 min of 100% B, 15 min of 100% B, 15.1 min of 5% B, and 16 min of 5% B. The column temperature was 45^o^C, and the flow rate was 0.35 mL/min. The injection volume was 2 μL. Ion source electrospray ionization (ESI) was used; the sampling mass spectrum signal was collected by positive and negative ion scanning.

### Data pre-processing

Raw data were subjected to baseline filtering, peak identification, integration, retention time correction, peak alignment, and normalization using Progenesis QI^10^ version 2.3 (Waters Corporation, Milford, MA, USA)^11^. The parameters were as follows: precursor tolerance, 5 ppm/10 ppm; product tolerance, 10 ppm/20 ppm; and production threshold, 5%. The compounds were determined based on the isotopic distribution, accurate mass number, and secondary debris. Qualitative analysis was performed using the Human Metabolome Database^12^, lipid maps 2.3^13^, Metabolite Link (METLIN)^14^, and self-built databases.

The self-built database is an LC-MS/MS database established by Ouyi Mass Spectrometry through hardware system standardization and based on standard samples. The database comprehensively covers more than 10000 common and important metabolites, including more than 2000 standard samples (including RT, MS1, MS2 dimensional identification information), mainly including amino acids and their derivatives, organic acids and their derivatives, carbohydrates and their derivatives, organic heterocycles, nucleosides and their derivatives, indoles and their derivatives, steroids and their derivatives, bile acids and their derivatives, acylcarnitine, hemolytic phospholipids, benzene and exposure group related metabolites, totaling 13 major categories.

After processing the raw data, we removed measurement values with ion peak data >50% and replaced the missing values with half of the minimum value. Qualitative compounds were screened based on their qualitative results. The screening standard was 36 points (full score: 60), and qualitative results below 36 points were deemed inaccurate and deleted. Finally, the positive and negative ion data were combined in a data matrix.

### Data analysis

Unsupervised principal component analysis (PCA) was first used to observe the overall distribution of samples and the stability of the analysis, followed by supervised partial least-squares discriminant analysis (PLS-DA) and orthogonal partial least-squares discriminant analysis (OPLS-DA) used to distinguish the differences in metabolic profiles.

Differential metabolites were screened using multidimensional and unidimensional analysis.

In OPLS-DA, variable importance in projection (VIP) was used to detect the influence intensity and interpretation ability of the expression mode of each metabolite on the classification and discrimination of samples, as well as to investigate differential metabolites with biological significance. The significance of differential metabolites was determined using the Student’s t-test. The screening thresholds were VIP value >1 and p<0.05, and we performed multiple corrections on the results. The metabolites of low-nicotine cigarettes were detected by two-way hierarchical clustering performed based on the expression levels of differential metabolites. The specific methods and usage standards mainly include the following aspects.

*Data preparation*

Before drawing a heatmap, it is usually necessary to preprocess the raw data. For example, when conducting cluster analysis of differential protein expression levels, the data is standardized using Z-score to ensure that data of different magnitudes can be compared on the same scale.

*Clustering method*

Horizontal and vertical clustering: simultaneously clustering rows (such as genes, metabolites, etc.) and columns (such as samples) can help identify which features are commonly expressed in a specific set of samples; horizontal clustering: clustering only for rows (features) while keeping the positions of columns (samples) unchanged can help observe the changing patterns of each feature under specific conditions.

*Color coding*

The color changes in the heatmap reflect the differences in numerical values. For the display of differential gene fold changes, the degree of change in gene expression levels between different samples can be represented by the depth of color.

A volcano plot was used to display differential metabolites. The linear correlation between the two metabolites was determined using the Pearson correlation coefficient. The Kyoto Encyclopedia of Genes and Genomes (KEGG) IDs (Kanehisa, 2000 #5) of differential metabolites were used for pathway analysis. A hypergeometric test was used for the identification of significant pathways^15^.

## RESULTS

### Qualitative and quantitative data

The positive and negative ion data were combined into data matrix tables (Supplementary file Tables S2 and S3), which contained all the information extracted from the raw data that could be used for subsequent analysis. The total number of material peaks was 21092, including 11807 negative (Supplementary file Table S2) and 9285 positives (Supplementary file Table S3). There were 8821 metabolites, including 4634 negative and 4187 positive.

### Multivariate statistical analysis

**Figure 2:**
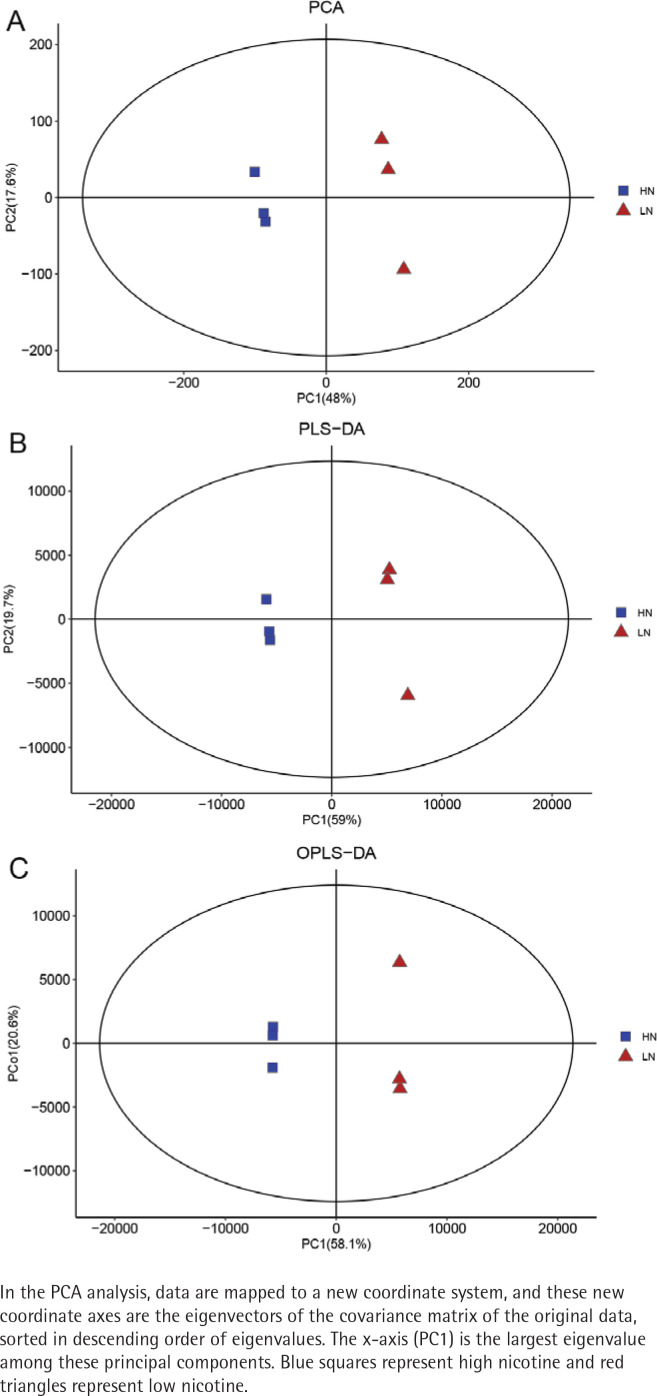
Results of multivariate statistical analysis: A) Results of principal component analysis (PCA); B) Results of partial least-squares discriminant analysis (PLS-DA); and C) Results of orthogonal partial least-squares discriminant analysis (OPLS-DA)

PCA revealed high similarity among the intra-group samples. In contrast, the distance between inter-group samples was large, indicating significant differences between the two groups (Figure 2A). Similar results were also identified using PLS-DA (Figure 2B) and OPLS-DA (Figure 2C). In PLS-DA, R2Y (cum) and Q2 (cum) were 1 and 0.999, respectively, indicating a good predictive power. In OPLS-DA, R2Y (cum) and Q2 (cum) were 1 and 0.992, respectively.

### Differential metabolite screening

Under the threshold, a total of 1150 significantly differential metabolites were identified between HN and LN groups, such as cotinine, 4-(methylnitrosamino)-1-(3-pyridyl)-1-butanol (NNAL)-N-glucuronide, 1-(5'-phosphoribosyl)-5-formamido-4-imidazolecarboxamide, 1-methylxanthine, 1,2-dihydroxy-3-keto-5-methylthiopentene, 11-dehydro-thromboxane B2, 2-hydroxyethanesulfonate, 2-hydroxyfelbamate, 2-methoxyestrone 3-glucuronide, deoxycholic acid 3-glucuronide, 2-phenylethanol glucuronide, and pregnanediol 3-O-glucuronide (Supplementary file Table S4). Therefore, 1-(5'-phosphoribosyl)-5-formamido-4-imidazolecarboxamide was associated with purine metabolism, 1-methylxanthine was associated with caffeine metabolism, 1,2-dihydroxy-3-keto-5-methylthiopentene was involved in cysteine and methionine metabolism, 11-dehydro-thromboxane B2 was related to arachidonic acid metabolism, and 2-hydroxyethanesulfonate was involved in taurine and hypotaurine metabolism. The last three metabolites (deoxycholic acid 3-glucuronide, 2-phenylethanol glucuronide, and pregnanediol 3-O-glucuronide) were associated with pentose and glucuronate interconversions.

**Figure 3:**
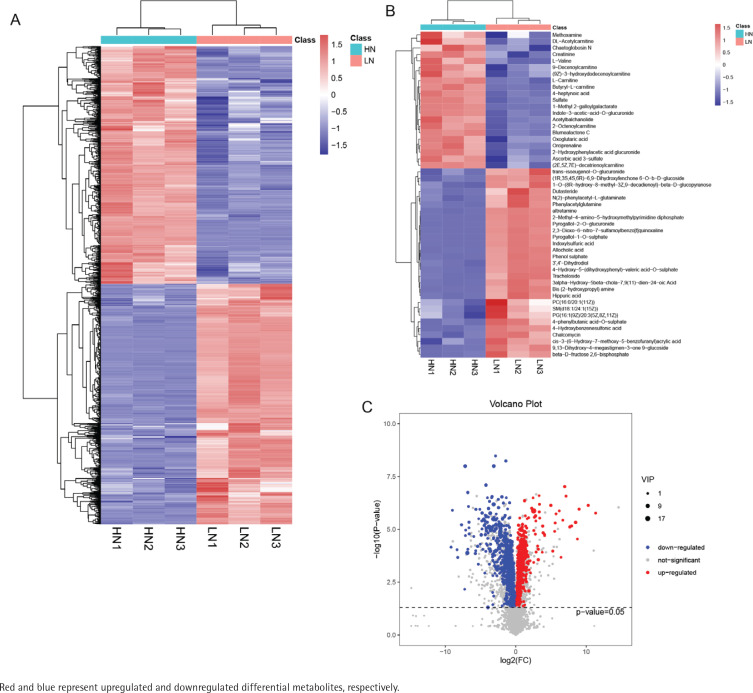
Differential metabolite screening between low-nicotine (LN) and high-nicotine (HN) groups: A) Heatmap of all significantly differential metabolites between LN and HN groups; B) Heatmap of top 50 significantly differential metabolites between LN and HN groups; and C) Volcano plot of all differential metabolites between LN and HN groups

Two-way hierarchical clustering analysis of all the differential metabolites and the top 50 differential metabolites showed noticeable differences between the two comparison groups (Figures 3A and 3B). A volcano plot of all differential metabolites is presented in Figure 3C. The correlation heatmap of the top 50 significantly differentially expressed metabolites is shown in Supplementary file Figure S1.

### Pathway enrichment analysis

**Figure 4:**
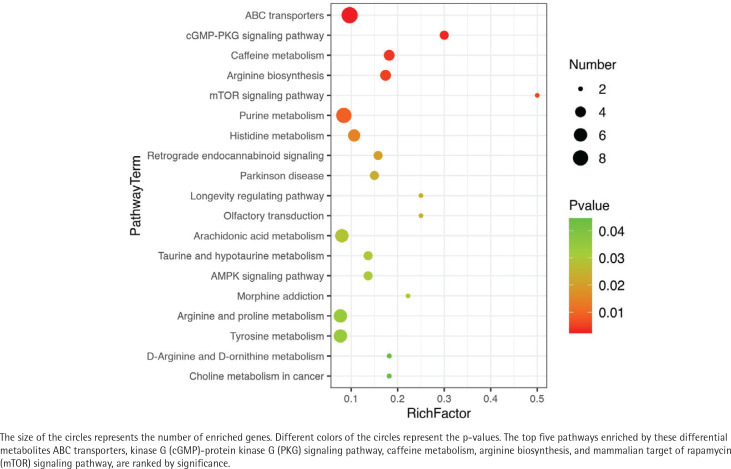
Bubble diagram of the significant pathways for KEGG enrichment of all significantly differential metabolites between LN and HN groups

A total of 19 KEGG pathways were identified with p<0.05, as shown in Figure 4. The 19 pathways were as follows: ABC transporters (sulfate, N-acetyl-D-glucosamine, and L-arginine), protein kinase G (cGMP)-protein kinase G (PKG) signaling pathway (adenosine monophosphate, guanosine monophosphate, and adenosine), caffeine metabolism (1-methylxanthine, 7-methylxanthine, theobromine, and caffeine), arginine biosynthesis (oxoglutaric acid, L-arginine, ornithine, and argininosuccinic acid), mammalian target of rapamycin (mTOR) signaling pathway (adenosine monophosphate, and L-arginine), purine metabolism (sulfate, guanosine monophosphate, and adenosine monophosphate), histidine metabolism (oxoglutaric acid, 3-methyl-L-histidine, and anserine), retrograde endocannabinoid signaling [PC (20:0/18:2 (9Z,12Z), PE (18:3 (9Z,12Z,15Z)/18:0), and PGH2], Parkinson’s disease (adenosine monophosphate, rotenone, and adenosine), longevity regulating pathway (resveratrol, and adenosine monophosphate), olfactory transduction (guanosine monophosphate, and adenosine monophosphate), arachidonic acid metabolism (2,3-dinor-8-iso-PGF2alpha, 11-dehydro-thromboxane B2, and 6-ketoprostaglandin E1), taurine and hypotaurine metabolism (2-hydroxyethanesulfonate, taurine, and oxoglutaric acid), adenosine monophosphate (AMP)-activated protein kinase (AMPK) signaling pathway (beta-D-fructose 2,6-bisphosphate, adenosine monophosphate, and AICAR), morphine addiction (adenosine monophosphate and adenosine), arginine and proline metabolism (L-arginine, ornithine, and creatine), tyrosine metabolism [gentisic acid, 5-(L-alanin-3-yl)-2-hydroxy-cis,cis-muconate 6-semialdehyde, and vanillylmandelic acid], D-arginine and D-ornithine metabolism (L-arginine and ornithine), and choline metabolism in cancer [choline and PC (20:0/18:2 (9Z,12Z)].

## DISCUSSION

To better understand the metabolic differences in smokers who smoke cigarettes with low and high nicotine content, untargeted metabolomics and bioinformatics analyses were used to assess the metabolomic profiles of urine samples. A total of 1150 significantly differential metabolites were identified between the HN and LN groups, such as cotinine, NNAL-N-glucuronide, and 11-dehydrothromboxane-B2, which were significantly enriched in some metabolism-related pathways, such as caffeine metabolism, arachidonic acid metabolism, arginine biosynthesis, and arginine and proline metabolism, as well as several disease-related signaling pathways, such as cGMP-PKG signaling pathway, AMPK signaling pathway, mTOR signaling pathway, and Parkinson’s disease.

Tobacco-specific nitrosamines in tobacco are potent carcinogens, among which 4-(methylnitrosamino)1-(3-pyridyl)-1-butanone (NNK) is the most potent and one of the most abundant. NNK is extensively metabolized to NNAL, a potent lung carcinogen. Additionally, NNAL can be glucuronidated and excreted in urine^16^. Carmella et al.^17^ confirmed the presence of NNAL-N-glucuronide in the urine of smokers using LC-ESI-MS/MS. In this study, NNAL-N-glucuronide was downregulated in the LN group, suggesting that low-nicotine cigarettes could decrease the levels of potent carcinogenic metabolites.

In the human body, approximately 70–80% of the absorbed nicotine is metabolized into cotinine in the liver by cytochrome P450 2A6^18^. There is a good dose-response relationship between plasma cotinine and daily cigarette consumption^19^. Plasma cotinine levels have been used as an exposure marker for cigarette smoking. Specifically, the concentration of cotinine is considerably higher in urine, compared to that in plasma^20^. In this study, cotinine levels decreased in the LN group compared with those in the HN group, suggesting that cotinine can serve as a biomarker of smoking with different nicotine content.

Caffeine is a natural stimulant, and cytochrome P450 family 1 subfamily A member 2 (CYP1A2) is the main enzyme responsible for caffeine metabolism^21^. CYP1A2 activity is upregulated by cigarette smoking, suggesting that cigarette smoking may promote caffeine metabolism^22^. Recently, Liu et al.^23^ reported that smokers exhibited significant changes in caffeine metabolism relative to non-tobacco consumers based on metabolomic analysis. Additionally, Liu et al.^23^ reported that some caffeine metabolites were significantly increased in the urine of smokers compared with that of non-tobacco consumers. In our study, the upregulated metabolites of 1-methylxanthine, 7-methylxanthine, and theobromine, as well as downregulated caffeine, in the HN versus LN groups, resulted in enriching caffeine metabolism, suggesting that caffeine metabolism exhibited marked changes in the LN group.

It has been reported that smoking is a key modifiable risk factor for cardiovascular diseases, such as stroke and sudden death^24^. Thromboxane is an important mediator of smoking-induced inflammation and has been implicated in the pathogenesis of cardiovascular disease^25^. 11-Dehydrothromboxane-B2 is a urinary metabolite of thromboxane^26^. Saareks et al.^27^ assessed the effect of smoking cessation^15^ on urinary 11-dehydrothromboxane-B2 levels and found that 11-dehydrothromboxane-B2 levels significantly decreased as early as 3 days after smoking cessation. In our study, 11-dehydrothromboxane-B2 levels increased in the LN group compared with those in the HN group, which may be due to the differences in metabolic levels between individuals. Our study also revealed that 11-dehydrothromboxane-B2 is associated with arachidonic acid metabolism. It has been reported that smoking is associated with alterations in arachidonic acid metabolism^28^, which is consistent with our results.

In addition to the above metabolism-related pathways and metabolites, arginine biosynthesis, as well as arginine and proline metabolism, were significant pathways enriched by some upregulated metabolites. Arginine is a building block for protein synthesis and is a precursor of small molecules, such as urea and nitric oxide. Arginine and proline metabolism are common pathways influenced by tobacco consumption^23^. Zhang et al.^29^ demonstrated that exposure to 10% cigarette smoke extract had a significant effect on endothelial arginine and proline metabolism, resulting in the reduction of intracellular arginine, N-hydroxy-l-arginine, and citrulline. In the present study, the upregulated arginine biosynthesis and arginine and proline metabolism in the HN group, suggested that compared to low-nicotine cigarettes, high-nicotine cigarettes may have a larger effect on arginine biosynthesis and metabolism.

Furthermore, several signaling pathways were identified. PKG is the main receptor of the cGMP second messenger. cGMP can regulate intracellular signaling pathways that control various intracellular processes, such as vasodilation, platelet activation, and memory formation, by binding to PKG^30^. In the present study, three differential metabolites (adenosine monophosphate, guanosine monophosphate, and adenosine) were found to be involved in the cGMP-PKG signaling pathway. A recent study reported that cigarette smoke exposure could enhance the expression of inducible nitric oxide synthase (iNOS) and activate nod-like receptor family pyrin domain containing 3 (NLRP3) inflammasome, both of which contributed to endothelial injury and vascular dysfunction. Additionally, this study linked iNOS to NLRP3 in cigarette smoke extract-stimulated human aortic endothelial cells via the soluble GC (sGC)/ cGMP/PKG/TNF-α converting enzyme (TACE)/ tumor necrosis factor (TNF)-α pathway^31^. Thus, we speculate that the metabolites of low-nicotine cigarettes might be beneficial for cardiovascular disease through the cGMP-PKG signaling pathway^32^.

Many pathological conditions associated with cigarette smoking are caused by the production of reactive oxygen species (ROS). Recently, Morsch et al.^33^ revealed the involvement of ROS, mTOR, and AMPK in the cigarette smoke-induced autophagic process in the lung, thereby increasing the risk of pulmonary diseases. In this study, both the mTOR and AMPK signaling pathways were identified. Therefore, we speculate that different nicotine contents might lead to different risks of pulmonary-related diseases by altering the mTOR and AMPK signaling pathways.

A previous study showed a correlation between cigarette smoking with a low risk of Parkinson’s disease^34^. Nicotine has been considered to prevent the risk of Parkinson’s disease, playing a critical role in the regulation of striatal activity mediated by the dopaminergic system^35^. Animal studies have shown that nicotine can regulate the transmission of dopamine and alleviate the dyskinesia caused by L-dopa^36^. In this study, three differential metabolites were involved in the pathway of Parkinson’s disease, suggesting that decreasing nicotine content may change the nerve conduction of Parkinson’s disease-related substances.

### Limitations

Despite these findings, this study has a few limitations. First, the results of the study are deemed as exploratory and preliminary. Second, this study recruited relatively few volunteers, and all participants were male, which limits the generalizability of the research results to the female population. Third, the identified differential metabolites and related pathways were not validated through *in vivo* and *in vitro* experiments. Further studies with larger sample sizes are necessary to confirm our results.

## CONCLUSIONS

Based on untargeted metabolomics and bioinformatics analyses, 1150 metabolites were identified to be differentially expressed between the HN and LN groups, such as cotinine, NNAL-N-glucuronide, and 11-dehydrothromboxane-B2. These differential metabolites were mainly enriched in ABC transporters, protein kinase G (cGMP)-protein kinase G (PKG) signaling pathway, caffeine metabolism, and arginine biosynthesis pathways.

## Supplementary Material



## Data Availability

The data supporting this research are available from the authors on reasonable request.
