# Supplementary material for: Response of human metabolism to ultra-low and high nicotine cigarettes based on urine metabolomics and bioinformatic analysis
Source: Tob Induc Dis. 2024 Dec 18;22:10.18332/tid/196677. doi: 10.18332/tid/196677 (PMC11653067; doi:10.18332/tid/196677)
Supplement: Supplementary file 1 [file TID-22-190-s1.pdf]

Supplementary file Table S1. Information of the three participations

Name	Sex	Age	Smoking years	Nation
Participation 1	Male	58	16	Han
Participation 2	Male	46	10	Han
Participation 3	Male	42	12	Han

Supplementary file Figure S1. Correlation analysis heatmap of the top 50 significantly differential expressed metabolites between LN and HN groups

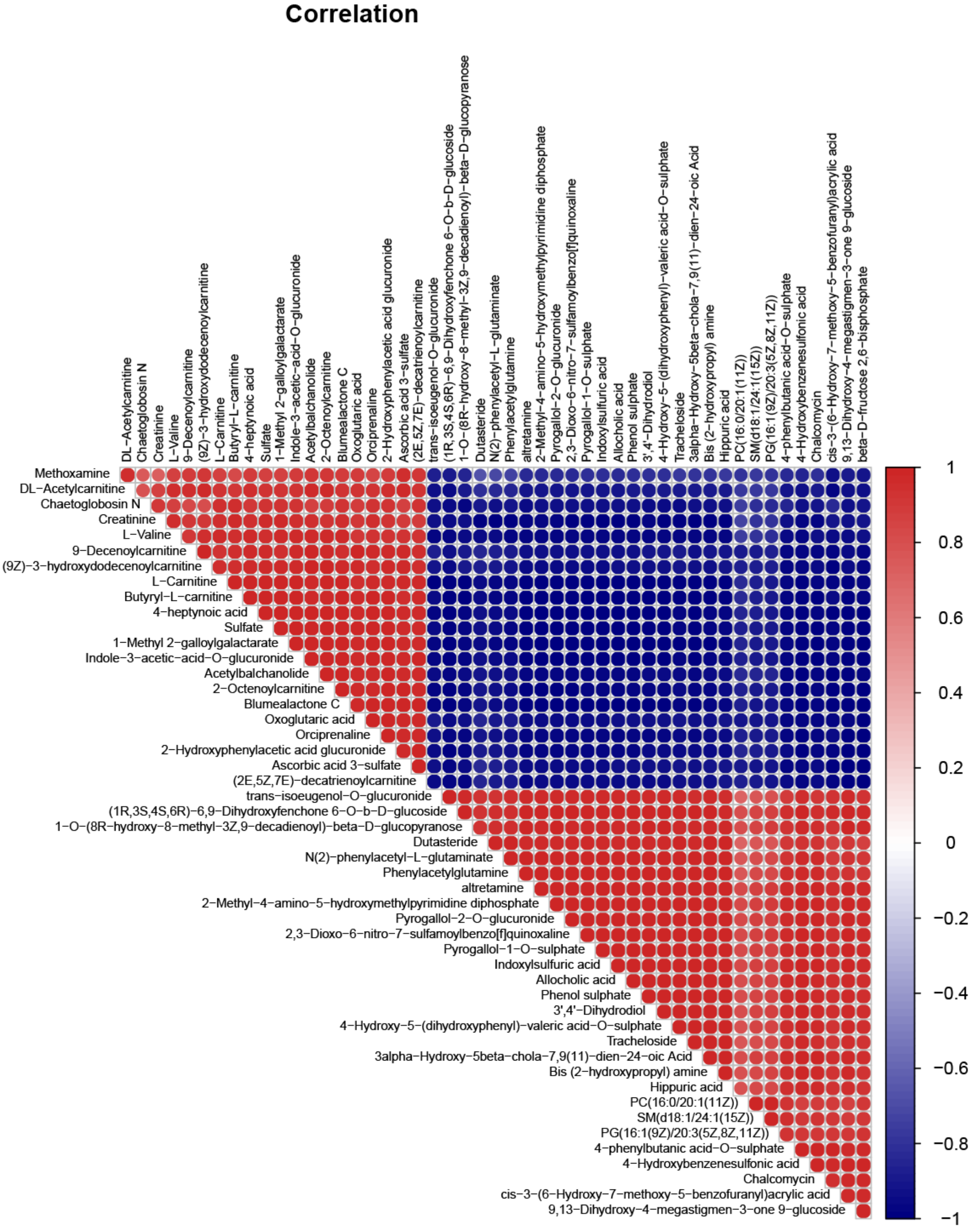

Red represents positive correlation, and blue represents negative correlation. There was a significant correlation among 50 differential metabolites.

Supplementary file Table S2. Raw negative data obtained from sequencing

ID	m/z	Retention time (min)	Ion mode	Metabolites	Compound ID	Super Class	Class	Sub Class	level	kegg	Score	Fragmentation Score	Adducts	Formula	Mass Error (ppm)	CK1	CK2	CK3	HN1	HN2	HN3	LN1	LN2	LN3
4.79_151.0401m/z	151.0401147	4.792316667	neg	3-Cresotinic acid	6661	Benzenoids	Benzene and substituted derivatives	Benzoic acids and derivatives		C14088	59	97.7	M-H	C8H8O3	0.309310423	3988.903632	4292.847518	4677.456248	4962.132168	4956.103801	5003.075784	4631.990888	4906.8231	5234.225656
5.22_167.0350m/z	167.0350416	5.222166667	neg	Vanillic acid	HMDB0000484	Benzenoids	Benzene and substituted derivatives	Benzoic acids and derivatives	***	C06672	58.7	96.5	M-H	C8H8O4	0.352842483	5472.604211	5730.643319	5914.011913	568.7488299	512.836852	686.763145	5061.884992	5215.861842	5471.473995
4.92_194.0460m/z	194.0459594	4.92485	neg	Salicyluric acid	617	Benzenoids	Benzene and substituted derivatives	Benzoic acids and derivatives	*	C07588	58.6	95.6	M-H	C9H9NO4	0.400099959	31219.45981	34804.0261	34938.75925	4255.661137	4286.678612	4416.639838	7151.178015	7937.769494	7730.460374
4.39_264.1110n	263.1037232	4.3893	neg	Phenylacetylglutamine	HMDB0006344	Organic acids and derivatives	Carboxylic acids and derivatives	Amino acids, peptides, and analogues	***	C04148	57.9	90.9	M-H, 2M-H, M-H2O-H	C13H16N2O4	-0.028048749	1296934.967	1401733.886	1353489.988	885556.1803	854312.3244	845983.9958	1427805.186	1689978.386	1536547.731
1.70_283.0686m/z	283.0685973	1.6992	neg	Xanthosine	3408	Nucleosides, nucleotides, and analogues	Purine nucleosides	Unclassified	**	C01762	57.6	92.6	M-H	C10H12N4O6	0.667546165	22195.50084	23849.25854	22938.99232	17596.77964	17616.69789	16586.69279	18699.98984	16637.35793	20917.84473
3.98_188.0354m/z	188.0354098	3.97985	neg	Kynurenic acid	HMDB0000715	Organoheterocyclic compounds	Quinolines and derivatives	Quinoline carboxylic acids	***	C01717	57.4	89.6	M-H	C10H7NO3	0.492515169	11416.75666	12639.53737	12327.92896	16534.81474	16041.80554	16033.48902	13424.18093	15402.98868	14731.3503
5.73_541.2660m/z	541.265998	5.727283333	neg	Cortolone-3-glucuronide	HMDB0010320	Lipids and lipid-like molecules	Steroids and steroid derivatives	Steroidal glycosides	*		57.2	90.3	M-H	C27H42O11	1.036962921	123329.377	139921.4341	109479.9291	130085.9335	179957.7227	156685.4862	142056.9616	182114.6084	181757.4575
5.71_539.2501m/z	539.2500672	5.709933333	neg	Tetrahydroaldosterone-3-glucuronide	HMDB0010357	Lipids and lipid-like molecules	Steroids and steroid derivatives	Steroidal glycosides	*		57	91.3	M-H	C27H40O11	0.521036279	344371.2964	349148.1809	292576.9232	309698.6487	414356.91	383306.9577	301396.3394	398647.2635	421864.7832
4.70_172.0980m/z	172.0980228	4.703166667	neg	N-Acetylleucine	HMDB0011756	Organic acids and derivatives	Carboxylic acids and derivatives	Amino acids, peptides, and analogues	***	C02710	56.8	87.1	M-H	C8H15NO3	0.611812152	11354.63742	10910.77498	11889.61897	14475.30345	13871.25213	13887.25413	15632.12355	16389.41836	15875.22343
4.37_159.0663m/z	159.0663257	4.367166667	neg	3-Methyladipic acid	HMDB0000555	Lipids and lipid-like molecules	Fatty Acyls	Fatty acids and conjugates	***		56.6	84.8	M-H	C7H12O4	0.270408869	27988.21385	31259.14337	33018.61132	44853.87341	30255.03439	32473.61499	23792.72381	26712.07358	26390.67325
0.90_168.0284n	167.0210934	0.904683333	neg	Uric acid	HMDB0000289	Organoheterocyclic compounds	Imidazopyrimidines	Purines and purine derivatives	***	C00366	56.5	86.4	M-H, 2M-H	C5H4N4O3	0.177727895	470483.5341	489558.9714	527552.7328	446759.0644	592840.7439	459363.8536	526170.4012	428046.3046	541895.5898
4.28_213.0097n	212.0024497	4.278183333	neg	Indoxylsulfuric acid	253	Organic acids and derivatives	Organic sulfuric acids and derivatives	Arylsulfates	*		56.2	84.8	M-H, 2M-H	C8H7NO4S	0.692021131	873409.2118	944600.9147	934250.8591	63673.55765	57224.44981	55912.32839	768364.9263	855781.0175	805114.1193
4.43_153.0194m/z	153.0193914	4.432683333	neg	Gentisic acid	618	Benzenoids	Benzene and substituted derivatives	Benzoic acids and derivatives	**	C00628	56.1	83.4	M-H	C7H6O4	0.384209097	13690.72623	14863.8246	15215.5791	3062.853938	2921.209785	3040.564189	11515.41546	13178.63626	12030.24058
4.00_172.9914m/z	172.9914257	4.002233333	neg	Phenol sulphate	HMDB0060015	Organic acids and derivatives	Organic sulfuric acids and derivatives	Arylsulfates	***	C02180	56	90.8	M-H	C6H6O4S	0.128824485	238836.0847	256838.9891	268094.1966	73576.74837	69812.46795	70739.06463	216549.9843	242558.9318	234102.8175
3.69_196.0597n	195.0524192	3.685866667	neg	1,7-Dimethyluric acid	61971	Organoheterocyclic compounds	Imidazopyrimidines	Purines and purine derivatives	**	C16356	55.7	85.9	M-H, 2M-H	C7H8N4O3	0.282946136	130418.4228	146418.8467	145458.1603	228664.9315	228872.8626	169391.3807	168980.5279	184748.3524	183741.8337
5.01_166.0631n	165.0557731	5.014716667	neg	Dihydro-3-coumaric acid	4152	Phenylpropanoids and polyketides	Phenylpropanoic acids	Unclassified	**	C11457	55.5	80.2	M-H2O-H, M-H	C9H10O3	0.818377092	12469.48333	13200.18014	13253.52929	14802.19873	16157.9845	17529.33799	15553.33462	14395.54644	14932.9251
1.20_173.0092m/z	173.0092182	1.202333333	neg	Aconitic acid	3300	Organic acids and derivatives	Carboxylic acids and derivatives	Tricarboxylic acids and derivatives	**	C00417	55.3	77.6	M-H	C6H6O6	0.325908687	136714.1647	148603.7076	163321.8151	153632.319	137198.6801	142722.9323	135889.2277	160432.6271	155530.7287
3.85_193.0619m/z	193.0619091	3.84605	neg	aminohippuric acid	3927	Benzenoids	Benzene and substituted derivatives	Benzoic acids and derivatives	*	D06890	55.3	79.1	M-H	C9H10N2O3	0.223105331	7879.605902	8518.476183	8972.860622	9743.968247	9256.198595	8938.010846	9470.023681	10138.26208	10286.16661
3.85_179.0575m/z	179.0575348	3.84605	neg	Theophylline	1458	Organoheterocyclic compounds	Imidazopyrimidines	Purines and purine derivatives	**	C07130	55.1	83.8	M-H	C7H8N4O2	0.476254429	37968.79315	40431.53772	43248.93508	45501.61693	46444.48607	45868.25177	45864.43434	49028.21345	48796.13821
4.00_158.0823m/z	158.0823066	4.002233333	neg	Acetyl-DL-Valine	73550	Unclassified	Unclassified	Unclassified			55.1	77.7	M-H	C7H13NO3	0.249879672	13429.13336	14291.72598	15129.43165	17545.26393	16797.04205	16655.49944	17853.74972	19470.78368	18667.09039
7.47_528.2641m/z	528.2641439	7.46525	neg	Glycochenodeoxycholate-3-sulfate	HMDB0002497	Lipids and lipid-like molecules	Steroids and steroid derivatives	Bile acids, alcohols and derivatives	***		55.1	84.3	M-H	C26H43NO8S	0.910740107	62031.67561	60857.95704	70116.04313	30358.71857	31124.48522	29591.54769	58415.22885	66032.61074	59982.30187
0.76_131.0463m/z	131.0462575	0.757316667	neg	L-Asparagine	14	Organic acids and derivatives	Carboxylic acids and derivatives	Amino acids, peptides, and analogues	**	C00152	55	78.6	M-H	C4H8N2O3	0.316560769	3039.754656	2818.375689	2944.7837	2669.396985	3973.07334	3946.41896	4097.654036	4321.946009	4393.177548
1.58_182.0459m/z	182.0459329	1.577833333	neg	4-Pyridoxic acid	HMDB0000017	Organoheterocyclic compounds	Pyridines and derivatives	Pyridinecarboxylic acids and derivatives	***	C00847	55	77.1	M-H	C8H9NO4	0.281810364	16567.11221	14056.41415	15198.66818	29983.24156	27379.10067	28988.40559	12387.33782	16612.91197	13968.70905
1.90_296.1002m/z	296.1001622	1.899016667	neg	2-Methylguanosine	58332	Nucleosides, nucleotides, and analogues	Purine nucleosides	Unclassified	*		54.9	79.4	M-H	C11H15N5O5	0.403990779	16558.6101	17689.20476	15061.06441	18454.05186	19208.27157	17927.11515	16555.68912	16638.43005	16287.90089
5.70_431.1925m/z	431.1924916	5.701283333	neg	Corchoionol C 9-glucoside	HMDB0029772	Lipids and lipid-like molecules	Fatty Acyls	Fatty acyl glycosides	*		54.8	77.4	M+FA-H	C19H30O8	0.571760666	182878.9711	179697.4995	180625.8571	180450.3764	187590.0186	186155.4492	192113.4457	199656.1053	219267.6518
6.48_200.1294m/z	200.1293813	6.477433333	neg	2,2,6,6-Tetramethyl-4-piperidinone	HMDB0031179	Organoheterocyclic compounds	Piperidines	Piperidinones	*		54.8	75.7	M+FA-H	C9H17NO	1.05913836	25031.64947	25904.75714	30729.5533	47455.53024	43594.81709	43430.87628	36450.75714	40297.37738	39197.98999
9.32_293.1761m/z	293.176076	9.321783333	neg	Furanofukinin	HMDB0036640	Lipids and lipid-like molecules	Prenol lipids	Sesquiterpenoids	*		54.7	76.6	M+FA-H	C16H24O2	0.979573425	123548.8785	118417.4059	90341.85133	114693.4052	139498.9823	124164.5435	102670.1346	101025.5454	124791.6545
4.18_137.0244m/z	137.0244351	4.181333333	neg	p-Salicylic acid	3263	Benzenoids	Benzene and substituted derivatives	Benzoic acids and derivatives	**	C00156	54.4	74.9	M-H	C7H6O3	0.126609109	7653.656046	6492.337561	6217.17795	4618.711867	4436.053052	5359.078174	7647.41548	5790.626146	6012.477203
4.41_179.0583n	178.0510155	4.41115	neg	Hippuric acid	1301	Benzenoids	Benzene and substituted derivatives	Benzoic acids and derivatives	**	C01586	54.4	82.2	M-H, 2M-H	C9H9NO3	0.272509782	394708.7497	447411.8227	417240.3151	175356.5311	170266.6304	173008.5625	314542.8832	361681.1797	338761.5757
3.87_182.0581n	181.050758	3.868533333	neg	Hydroxyphenyllactic acid	34515	Phenylpropanoids and polyketides	Phenylpropanoic acids	Unclassified	*	C03672	54.3	74.1	M-H2O-H, M-H	C9H10O4	0.851488422	54828.01894	58647.54207	61367.98467	23091.83562	21979.60411	22540.13542	61556.39728	66242.84984	66745.24444
3.94_183.0300m/z	183.0299862	3.9351	neg	Ethyl aconitate	HMDB0040275	Organic acids and derivatives	Carboxylic acids and derivatives	Tricarboxylic acids and derivatives			54.3	73.9	M-H2O-H	C8H10O6	0.441973222	10954.42023	11607.79973	12417.15675	10915.29934	10170.29317	10469.64399	8446.955098	9335.073331	9212.493043
4.75_202.1207n	201.1133927	4.747466667	neg	9,10-dihydroxy-2-decenoic acid	LMFA01050428	Lipids and lipid-like molecules	Fatty Acyls	Fatty Acids and Conjugates			54.3	73.8	M-H, 2M-H	C10H18O4	0.792025519	227738.334	243708.1469	246582.114	217390.5351	214558.6137	209027.9182	211194.5861	233910.0021	232501.3791
4.09_153.0194m/z	153.019406	4.091783333	neg	2,3-Dihydroxybenzoic acid	3276	Benzenoids	Benzene and substituted derivatives	Benzoic acids and derivatives	*	C00196	54.2	73.6	M-H	C7H6O4	0.479110894	4153.430221	4013.63588	4160.061425	2486.367644	2231.739442	2409.337049	2973.629448	3012.588404	2905.818119
4.75_208.0616m/z	208.061629	4.747466667	neg	3-Methyldioxyindole	HMDB0004186	Organoheterocyclic compounds	Indoles and derivatives	Indolines	**	C05834	53.9	71.1	M+FA-H	C9H9NO2	0.598634819	131521.9077	135502.8561	138175.5432	61243.54721	63137.36682	63153.37458	113118.08	131291.7169	121469.4627
4.88_173.0820m/z	173.0819524	4.880083333	neg	Suberic acid	4243	Lipids and lipid-like molecules	Fatty Acyls	Fatty acids and conjugates	*	C08278	53.9	70.3	M-H	C8H14O4	0.114229274	74095.94903	79153.31141	80759.75306	76765.24311	75831.94437	78047.96824	57292.17555	61030.73618	58664.34812
6.57_213.1134m/z	213.113397	6.570233333	neg	2,2'-(3-methylcyclohexane-1,1-diyl)diacetic acid	96295	Unclassified	Unclassified	Unclassified			53.9	72.9	M-H	C11H18O4	0.767727971	23084.75807	25230.54363	26697.35711	21589.77248	21428.5689	21196.40279	15225.6365	17618.62179	15909.06697
3.96_279.0987m/z	279.0987348	3.957583333	neg	Asp-Phe	64957	Organic acids and derivatives	Carboxylic acids and derivatives	Amino acids, peptides, and analogues			53.6	73	M-H	C13H16N2O5	0.319832325	3994.151568	4791.856291	4798.105546	5478.176138	5100.80732	5807.565536	4375.778704	4805.226978	4409.375703
4.16_175.0612m/z	175.0612271	4.15895	neg	2-Isopropylmalic acid	HMDB0000402	Lipids and lipid-like molecules	Fatty Acyls	Fatty acids and conjugates	***	C02504	53.6	69.9	M-H	C7H12O5	0.170430361	10427.41717	10545.93524	10892.50821	15438.96226	11873.72054	12802.30454	13530.07961	10625.21068	10239.2395
5.30_423.2028m/z	423.20279	5.302883333	neg	18-Hydroxycortisol	LMST02030193	Lipids and lipid-like molecules	Steroids and steroid derivatives	Hydroxysteroids	*		53.6	74.6	M+FA-H	C21H30O6	0.921446834	8614.354296	8063.724284	9140.62405	9356.660231	11388.39485	11507.54847	12186.19554	10436.51955	14920.70007
1.96_561.2040m/z	561.2039566	1.95955	neg	indoprofen	HMDB0253478	Unclassified	Unclassified	Unclassified			53.4	72.2	2M-H	C17H15NO3	1.505363482	25840.24389	24364.82044	20002.9153	206.1507667	0.001403199	96.6509771	21337.88107	21667.57476	19554.81778
0.76_146.0692n	145.0619439	0.757316667	neg	D-Glutamine	63630	Organic acids and derivatives	Carboxylic acids and derivatives	Amino acids, peptides, and analogues	**	C00819	53.3	72.9	M-H2O-H, M-H	C5H10N2O3	0.365811811	28488.04569	26836.55019	26041.88158	21194.14233	24995.80934	27542.44618	28474.96522	32851.70613	32601.1453
5.83_199.0977m/z	199.0977044	5.825	neg	Matsutakic acid A	HMDB0036715	Organic acids and derivatives	Hydroxy acids and derivatives	Medium-chain hydroxy acids and derivatives			53.2	68.1	M-H	C10H16O4	0.608928114	73973.98224	74424.52178	81590.52749	96979.4885	76761.91289	76706.18006	65127.31027	64309.798	62271.11868
6.21_203.1289m/z	203.128932	6.206233333	neg	Pelargonic acid	LMFA01010009	Lipids and lipid-like molecules	Fatty Acyls	Fatty acids and conjugates	*	C01601	53.2	69.1	M+FA-H	C9H18O2	0.31185941	3414.40651	3458.621648	3916.968264	5364.635338	4759.830036	5098.053254	2669.345705	2922.156056	3174.750976
1.16_151.0262m/z	151.0261847	1.160166667	neg	Xanthine	HMDB0000292	Organoheterocyclic compounds	Imidazopyrimidines	Purines and purine derivatives	***	C00385	53.1	71.9	M-H	C5H4N4O2	0.235174736	10417.71433	10666.42661	11877.45753	11598.24699	13853.10067	13777.4694	9960.235534	10811.8141	12028.0238
5.06_164.0355m/z	164.035473	5.05975	neg	N-formylanthranilic acid	34537	Benzenoids	Benzene and substituted derivatives	Benzoic acids and derivatives	**	C05653	53.1	72	M-H	C8H7NO3	0.947085273	3411.080989	3486.850998	3525.601463	5010.568553	4649.58683	5111.375369	4095.443555	4235.803733	5293.463472
1.20_180.0667m/z	180.0667375	1.202333333	neg	L-Tyrosine	34	Organic acids and derivatives	Carboxylic acids and derivatives	Amino acids, peptides, and analogues	**	C00082	53	68.3	M-H	C9H11NO3	0.666486829	8153.409991	8309.63602	8402.157393	11107.70907	10685.38542	10665.27209	9247.742009	9875.511231	10836.52294
2.71_131.0350m/z	131.0350158	2.709983333	neg	Glutaric acid	3254	Organic acids and derivatives	Carboxylic acids and derivatives	Dicarboxylic acids and derivatives	**	C00489	53	67.6	M-H	C5H8O4	0.2538414	4746.094822	4580.176767	4779.538329	4941.93651	4614.837546	4949.756313	4654.711006	4248.960266	4502.928766
3.80_195.0532n	194.0459171	3.801716667	neg	L-Dopaquinone	HMDB0039119	Organic acids and derivatives	Carboxylic acids and derivatives	Amino acids, peptides, and analogues			53	75.4	M-H, M+FA-H	C9H9NO4	0.18314937	33333.37354	28706.40352	28958.11846	4468.129983	3865.340166	3339.982406	16462.58124	16793.95743	17303.96378
6.10_201.1134m/z	201.1134043	6.101316667	neg	2-Hydroxy-2,6,6-trimethylcyclohexanone	HMDB0037023	Organic oxygen compounds	Organooxygen compounds	Carbonyl compounds	*		53	67.1	M+FA-H	C9H16O2	1.099881626	82087.55861	87273.48821	91105.16815	70282.21515	75898.59569	71693.51326	81348.82193	83000.38532	88364.64058
0.97_174.0408m/z	174.0408221	0.967616667	neg	N-acetylaspartate	3769	Organic acids and derivatives	Carboxylic acids and derivatives	Amino acids, peptides, and analogues	**	C01042	52.9	68	M-H	C6H9NO5	0.149569313	7303.734794	7457.247106	7772.256635	9093.376785	8815.55256	8202.921639	7887.737011	8999.637041	8364.551914
3.69_145.0507m/z	145.0507092	3.685866667	neg	Adipic acid	115	Lipids and lipid-like molecules	Fatty Acyls	Fatty acids and conjugates	**	C06104	52.9	67.9	M-H	C6H10O4	0.525903502	5213.771106	4944.319215	5033.483912	7436.686392	7682.014395	5590.765095	5520.501509	5290.157375	5709.80256
4.86_401.1441m/z	401.1441182	4.858383333	neg	xi-3-Hydroxy-5-phenylpentanoic acid O-beta-D-Glucopyranoside	HMDB0031695	Lipids and lipid-like molecules	Fatty Acyls	Fatty acyl glycosides	*		52.9	74.3	M+FA-H	C17H24O8	-3.376074141	57409.55663	59771.12496	62073.24997	81417.52548	77645.43448	78119.33506	70952.03245	77026.79613	75113.29539
7.65_269.1396m/z	269.1395695	7.65305	neg	3-Methyl-5-pentyl-2-furanpropanoic acid	HMDB0112084	Lipids and lipid-like molecules	Fatty Acyls	Fatty acids and conjugates			52.9	66.9	M+FA-H	C13H20O3	0.545065515	15862.9685	16854.48912	18611.46052	10233.86112	9319.22639	10030.4533	14469.42526	15113.9123	15950.8521
5.13_223.0846n	204.0667102	5.132616667	neg	2-hydroxyphenylpropionylglycine	HMDB0094723	Organic acids and derivatives	Carboxylic acids and derivatives	Amino acids, peptides, and analogues			52.7	65.9	M-H2O-H, M-H	C11H13NO4	0.418627781	33575.75573	37555.43459	28823.83101	52790.00269	51839.94259	50271.62157	48341.36969	51061.80616	64396.64394
8.07_405.2648m/z	405.2647973	8.074883333	neg	(23R)-3alpha,7alpha,17alpha,23-Tetrahydroxy-5beta-cholan-24-oic Acid	LMST04010421	Unclassified	Unclassified	Unclassified			52.7	68.7	M-H2O-H	C24H40O6	0.352239726	232710.9359	263745.2709	240438.357	44439.56986	41457.64625	39951.96879	111989.3075	136297.0466	131916.7707
4.52_287.0233m/z	287.0233001	4.521733333	neg	4-Hydroxy-5-(dihydroxyphenyl)-valeric acid-O-sulphate	HMDB0059978	Benzenoids	Phenols	Benzenediols	*		52.6	67.9	M-H2O-H	C11H14O8S	0.66257282	80598.8187	83071.97727	97276.0216	60281.27941	57366.007	55604.54391	149348.1402	165986.8798	165970.9427
3.98_222.0773m/z	222.0773102	3.97985	neg	Acetyl-L-tyrosine	5827	Organic acids and derivatives	Carboxylic acids and derivatives	Amino acids, peptides, and analogues	*	C01657	52.5	67.3	M-H	C11H13NO4	0.5768499	2588.693337	2544.729402	2821.758891	2988.883053	2761.932137	2566.339979	2919.072938	2966.928251	2799.849328
2.35_141.0194m/z	141.0193803	2.34905	neg	(2E,4Z)-2-hydroxy-6-oxohexa-2,4-dienoic acid	LMFA01030926	Lipids and lipid-like molecules	Fatty Acyls	Fatty Acids and Conjugates			52.4	65.9	M-H	C6H6O4	0.33827902	3517.129496	3387.291115	3273.555509	1699.181336	1755.53451	1552.150474	1052.008508	1160.480782	1213.550078
8.68_567.3179m/z	567.3178705	8.68065	neg	Deoxycholic acid 3-glucuronide	LMST05010046	Lipids and lipid-like molecules	Steroids and steroid derivatives	Steroidal glycosides	**	C03033	52.4	65.2	M-H	C30H48O10	0.702483295	189772.3091	214186.3825	202790.7427	29714.68254	27737.18346	23516.41574	98035.57394	102502.3008	107318.6989
3.94_125.0243m/z	125.0243332	3.9351	neg	4-HYDROXY-6-METHYLPYRAN-2-ONE	44653	Unclassified	Unclassified	Unclassified			52.2	66.3	M-H	C6H6O3	-0.670201253	1681.207329	1828.28364	1929.082415	838.2690928	736.5257261	730.0179336	1479.757184	1635.478997	1554.379517
7.04_271.1553m/z	271.1552843	7.044716667	neg	Riesling acetal	HMDB0037562	Organoheterocyclic compounds	Tetrahydrofurans	Unclassified	*		52.2	64.6	M+FA-H	C13H22O3	0.826385665	6991.08507	7361.528153	6773.807644	12025.88408	11795.99898	10737.47544	6477.273938	7046.356413	6027.747028
2.86_195.0524m/z	195.0524266	2.864816667	neg	1,3-Dimethyluric acid	2822	Organoheterocyclic compounds	Imidazopyrimidines	Purines and purine derivatives	*		52.1	69.8	M-H	C7H8N4O3	0.320714272	36151.70914	36374.74729	40510.58389	56204.74629	53604.75584	52793.74901	44183.22363	46660.30896	42426.37755
3.96_187.0613m/z	187.0612868	3.957583333	neg	3-acetyl-3-methyldihydrofuran-2(3H)-one	LMFA07040006	Lipids and lipid-like molecules	Fatty Acyls	Fatty esters			52.1	61.9	M+FA-H	C7H10O3	0.631997569	3797.344968	4099.567406	4516.659758	7096.362666	6881.080278	7415.108252	4287.120186	4935.713612	4678.607465
6.43_405.1921m/z	405.192126	6.433933333	neg	Aldosterone	LMST02030026	Lipids and lipid-like molecules	Steroids and steroid derivatives	Hydroxysteroids	**	C01780	52.1	77	M+FA-H	C21H28O5	0.691641832	26794.25673	24629.58257	24270.61069	25350.1856	23187.48122	25260.06698	35041.69705	35444.66902	30688.39531
0.93_146.0216n	191.0198685	0.925866667	neg	Oxoglutaric acid	119	Organic acids and derivatives	Keto acids and derivatives	Gamma-keto acids and derivatives	**	C00026	52	61	M-H, M+FA-H	C5H6O5	0.418166227	389591.3497	484469.8807	564170.5733	910328.1777	929902.2747	847668.5402	496383.3445	612087.1789	614000.3543
1.38_225.0631m/z	225.0631212	1.380283333	neg	5-Acetylamino-6-formylamino-3-methyluracil	HMDB0011105	Organoheterocyclic compounds	Diazines	Pyrimidines and pyrimidine derivatives	**	C16365	52	70.3	M-H	C8H10N4O4	0.852741347	235906.2323	192594.8402	202662.9284	340187.4979	314699.459	289608.5594	310562.4338	300043.2402	289906.9995
3.80_245.1144m/z	245.1143778	3.801716667	neg	Aspartyl-Isoleucine	HMDB0028756	Organic acids and derivatives	Carboxylic acids and derivatives	Amino acids, peptides, and analogues	*		52	64.6	M-H	C10H18N2O5	0.335485475	1797.545942	1865.516952	1945.392155	2514.628067	2594.789348	2277.755907	1699.605725	1883.984728	2038.193549
9.75_239.1290m/z	239.1290439	9.75225	neg	Cucujolide IV	LMFA07040036	Lipids and lipid-like molecules	Fatty Acyls	Fatty esters			52	63	M+FA-H	C12H18O2	0.830357353	27793.89759	24836.18659	23637.61877	24935.31547	24773.42692	26744.48042	28509.20962	26876.6283	26517.35847
0.88_243.0623m/z	243.0622965	0.884633333	neg	Uridine	HMDB0000296	Nucleosides, nucleotides, and analogues	Pyrimidine nucleosides	Unclassified	**	C00299	51.9	61.9	M-H	C9H12N2O6	0.150891894	197204.6924	191227.1145	194450.8088	174126.9827	166688.4432	181192.0634	193882.481	171959.8683	187540.2908
5.38_244.1312n	243.123801	5.384216667	neg	Polyethylene, oxidized	HMDB0032472	Organic acids and derivatives	Keto acids and derivatives	Medium-chain keto acids and derivatives	*		51.9	63.2	M-H2O-H, M-H	C12H20O5	0.580198357	49524.04738	58484.68139	54570.70578	47577.2074	47591.66649	45980.87861	35762.71807	41818.56292	40038.0341
5.45_234.0773m/z	234.0773196	5.451166667	neg	Phensuximide	HMDB0014970	Organoheterocyclic compounds	Pyrrolidines	Phenylpyrrolidines		C07437	51.9	61.6	M+FA-H	C11H11NO2	0.730419645	13316.76256	13099.80932	13836.55935	4756.75228	4451.621802	4737.084522	12079.96618	9478.218372	9785.999962
1.16_191.0199m/z	191.0198602	1.160166667	neg	Citric acid	HMDB0000094	Organic acids and derivatives	Carboxylic acids and derivatives	Tricarboxylic acids and derivatives	***	C00158	51.8	60.3	M-H	C6H8O7	0.698108218	715698.6009	784459.5944	1044995.386	1101801.76	1170032.927	1088361.696	954172.9242	988344.9313	1094971.387
4.57_423.2029m/z	423.2028883	4.5668	neg	Eremopetasitenin C1	HMDB0032770	Lipids and lipid-like molecules	Prenol lipids	Terpene lactones	*		51.8	63.8	M+FA-H	C21H30O6	1.181169419	5711.394146	6127.499799	6790.136444	9796.144992	9187.782676	8482.89143	6826.924493	6902.930925	7126.029065
1.55_103.0401m/z	103.0401011	1.553483333	neg	4-Hydroxybutyric acid	HMDB0000710	Lipids and lipid-like molecules	Fatty Acyls	Fatty acids and conjugates	*	C01991	51.7	60.2	M-H	C4H8O3	0.321648482	1842.499953	1585.977343	1476.038858	2726.437844	2780.372779	2497.641585	1777.629186	2609.956959	2091.075334
5.16_215.0022m/z	215.0022253	5.155116667	neg	Hydroxytyrosol 3'-sulfate	HMDB0240530	Organic acids and derivatives	Organic sulfuric acids and derivatives	Arylsulfates			51.7	66.4	M-H2O-H	C8H10O6S	1.099618929	58892.68463	72321.5565	62403.57038	1956.817148	1682.766792	1682.259188	48676.69844	53640.17441	55892.34014
3.94_302.0640n	301.0566972	3.9351	neg	Pyrogallol-2-O-glucuronide	HMDB0060017	Organic oxygen compounds	Organooxygen compounds	Carbohydrates and carbohydrate conjugates	*		51.6	60.5	M-H, 2M-H	C12H14O9	0.634343121	119205.7944	125814.8252	134400.7538	6444.97756	5937.848805	6566.436265	109930.7518	119228.2215	116168.2293
5.22_185.0820m/z	185.0820076	5.222166667	neg	5-(2-Methylpropyl)tetrahydro-2-oxo-3-furancarboxylic acid	HMDB0030988	Organoheterocyclic compounds	Lactones	Gamma butyrolactones	*		51.6	60.4	M-H	C9H14O4	0.403444583	15146.02347	15575.98431	15568.00878	17035.61354	15714.23385	16239.38115	12738.61715	11882.31395	13339.95975
0.80_209.0303m/z	209.0303238	0.800366667	neg	D-Glucarate	3343	Organic oxygen compounds	Organooxygen compounds	Carbohydrates and carbohydrate conjugates	**	C00818	51.5	65	M-H	C6H10O8	0.157090788	5838.865713	7303.214375	7270.863982	8060.047412	8537.142454	9423.167381	2948.009426	2949.37664	3061.965844
0.84_179.0561m/z	179.0561477	0.841733333	neg	myo-Inositol	144	Organic oxygen compounds	Organooxygen compounds	Alcohols and polyols	**	C00137	51.5	59.4	M-H	C6H12O6	0.200241019	102835.4116	107836.6902	123196.7006	92333.90697	95160.43442	91317.3411	89256.01726	92200.39209	91530.39825
4.11_293.1146m/z	293.114578	4.113883333	neg	gamma-Glutamylphenylalanine	HMDB0000594	Organic acids and derivatives	Carboxylic acids and derivatives	Amino acids, peptides, and analogues	*		51.5	62.5	M-H	C14H18N2O5	0.961396003	8498.741035	8914.711658	9039.118978	11408.59668	10361.76188	10882.44058	12299.97554	12245.99786	11720.02545
4.79_217.1082m/z	217.1081953	4.792316667	neg	cis-3-Hexenyl lactate	HMDB0036213	Organic acids and derivatives	Carboxylic acids and derivatives	Carboxylic acid derivatives			51.5	59.2	M+FA-H	C9H16O3	0.279448389	8669.086487	9023.889667	9084.061464	11704.22734	12022.37852	11408.00812	8681.222202	9202.532426	8802.79749
5.43_188.1048n	187.0976474	5.428733333	neg	Azelaic acid	LMFA01170054	Lipids and lipid-like molecules	Fatty Acyls	Fatty acids and conjugates	*	C08261	51.5	58.8	M-H2O-H, M-H	C9H16O4	-0.131241162	245189.3053	266811.0447	270206.0079	195582.4394	186152.9914	195423.3268	234265.0872	255762.2266	202716.61
2.11_197.0456m/z	197.0456353	2.114866667	neg	Vanillylmandelic acid	HMDB0000291	Benzenoids	Phenols	Methoxyphenols	***	C05584	51.4	59.3	M-H	C9H10O5	0.445963348	32844.69388	39241.49167	37409.80572	43957.58745	42752.88848	47250.2328	34411.47325	34859.76365	41261.32649
4.99_172.1101n	217.1082598	4.99225	neg	8-oxo-nonanoic acid	LMFA01060159	Lipids and lipid-like molecules	Fatty Acyls	Fatty Acids and Conjugates			51.4	59.4	M-H, M+FA-H	C9H16O3	0.779507684	10748.12432	10518.24226	11337.64294	12753.30249	13542.20922	14217.68823	13382.82371	11761.62745	11141.76076
6.22_273.1709m/z	273.1709433	6.215	neg	11-Hydroxy-9-tridecenoic acid	LMFA01050437	Lipids and lipid-like molecules	Fatty Acyls	Fatty acids and conjugates	*		51.4	59.1	M+FA-H	C13H24O3	0.858322116	57402.90815	55143.13898	60844.98857	49328.66605	48465.76948	49229.22695	45625.93069	48713.81041	50007.85127
1.59_219.0776m/z	219.0776486	1.5934	neg	Tyrosylglycine	HMDB0029105	Organic acids and derivatives	Carboxylic acids and derivatives	Amino acids, peptides, and analogues	*		51.3	59.5	M-H2O-H	C11H14N2O4	0.557669262	9796.544341	8913.215873	9677.882064	12197.85719	11728.23336	11840.6373	9774.604439	9998.462592	10468.75248
4.28_284.1504m/z	284.1504433	4.278183333	neg	Petasinine	HMDB0030516	Organoheterocyclic compounds	Pyrrolizidines	Unclassified	*		51.3	59.1	M+FA-H	C13H21NO3	0.405319954	31006.75698	33553.61066	32609.56881	17210.08721	16127.61265	16120.30472	28413.4949	31805.07853	29270.54297
4.84_199.0977m/z	199.0977257	4.836566667	neg	2-Hydroxydecanedioic acid	HMDB0000424	Organic acids and derivatives	Hydroxy acids and derivatives	Medium-chain hydroxy acids and derivatives	*		51.3	61.3	M-H2O-H	C10H18O5	0.656414566	32364.88424	33102.56312	34445.87694	35831.15706	34458.59554	35013.28648	34171.11412	34105.91265	34327.9648
6.25_481.2446m/z	481.2445571	6.248083333	neg	11-beta-hydroxyandrosterone-3-glucuronide	LMST05010030	Organoheterocyclic compounds	Indoles and derivatives	Hydroxyindoles	**	C05643	51.3	58.8	M-H	C25H38O9	0.51988486	287852.9367	216276.2248	276626.618	306107.5067	255487.4141	302822.0634	257032.0589	274228.4208	261468.6862
0.80_124.9914m/z	124.9913572	0.800366667	neg	2-Hydroxyethanesulfonate	HMDB0003903	Organic acids and derivatives	Organic sulfonic acids and derivatives	Organosulfonic acids and derivatives	**	C05123	51.2	63.3	M-H	C2H6O4S	-0.365462771	90605.58626	96042.1313	104573.036	146198.81	157523.436	151520.6524	109879.6601	101461.5861	110339.6135
5.20_447.0936m/z	447.0936396	5.20005	neg	Naringenin-7-O-β-D-Glucuronide	44838	Unclassified	Unclassified	Unclassified			51.2	59.6	M-H	C21H20O11	0.791225947	30617.23022	37442.28527	35271.96201	24043.51228	22853.03076	23655.87593	31382.34865	33755.80068	33358.50672
4.77_283.0824m/z	283.082419	4.769883333	neg	p-Cresol glucuronide	HMDB0011686	Organic oxygen compounds	Organooxygen compounds	Carbohydrates and carbohydrate conjugates	*		51.1	57.8	M-H	C13H16O7	0.325880646	27237.30107	27664.17109	28459.14355	23663.01402	24919.8449	24218.0501	27153.75917	31172.18715	28721.32361
6.20_213.1134m/z	213.1134094	6.195716667	neg	(3S)-3-hydroxycyclocitral	HMDB0303972	Unclassified	Unclassified	Unclassified			51.1	57.8	M+FA-H	C10H16O2	1.051724526	37097.51385	33631.58773	39757.4673	60041.26906	55703.90913	58707.95739	41554.13973	43235.48213	45880.70626
1.70_351.0562m/z	351.0561869	1.6992	neg	4-O-alpha-D-Galactopyranuronosyl-D-galacturonic acid	HMDB0039721	Organic oxygen compounds	Organooxygen compounds	Carbohydrates and carbohydrate conjugates	**	C02273	51	63.5	M-H2O-H	C12H18O13	-1.925462547	9757.265225	10858.67966	10589.43743	7971.72864	8079.112832	7280.843181	8140.347477	7119.994729	9560.590144
4.23_275.0232m/z	275.0232091	4.234483333	neg	3-Phenylpropionic acid sulfate	HMDB0240372	Benzenoids	Benzene and substituted derivatives	Unclassified			51	59.4	M+FA-H	C9H10O5S	0.486253543	35895.37075	37631.02992	39499.63651	30752.72772	29262.09119	30267.73125	24555.65566	29017.25513	26648.36822
6.84_457.2063m/z	457.2062868	6.842166667	neg	Abscisic alcohol 11-glucoside	HMDB0039636	Lipids and lipid-like molecules	Prenol lipids	Terpene glycosides	*		51	65.6	M+FA-H	C21H32O8	-3.964047106	35908.7997	36108.70658	33993.68391	43879.78475	43142.64711	42862.79765	38463.03338	40568.42111	36499.0125
4.26_273.0076m/z	273.0075951	4.2568	neg	Ferulic acid 4-O-sulfate	HMDB0029200	Phenylpropanoids and polyketides	Cinnamic acids and derivatives	Hydroxycinnamic acids and derivatives	*		50.8	58.5	M-H	C10H10O7S	0.539631874	170325.7566	177161.9096	175349.2861	83504.54611	79226.44389	76825.16834	137178.6078	148555.2024	133885.1998
5.85_211.0978m/z	211.097779	5.84575	neg	5-Hydroxy-p-mentha-6,8-dien-2-one	HMDB0037011	Lipids and lipid-like molecules	Prenol lipids	Monoterpenoids	*		50.8	58.2	M+FA-H	C10H14O2	1.182932067	1927.734565	1957.423808	2517.495725	4379.334239	3603.493121	3359.485115	2641.046472	2548.129232	2299.557897
8.07_451.2705m/z	451.2705207	8.074883333	neg	7-Ketodeoxycholic acid	HMDB0000391	Lipids and lipid-like molecules	Steroids and steroid derivatives	Bile acids, alcohols and derivatives	*		50.8	56.6	M+FA-H	C24H38O5	0.968668271	130194.9656	141420.5207	138419.9619	32126.03929	29802.7496	29522.29703	70425.24745	85365.46305	79913.47852
0.78_193.0354m/z	193.0354342	0.778533333	neg	D-Glucuronic acid	HMDB0000127	Organic oxygen compounds	Organooxygen compounds	Carbohydrates and carbohydrate conjugates	**	C00191	50.7	56.4	M-H	C6H10O7	0.299000104	65966.43239	71476.02615	87568.96726	68625.69835	79139.7376	71720.26688	60293.68875	75688.82623	73897.09927
3.91_157.0507m/z	157.0506696	3.912566667	neg	Hexadienic acid	HMDB0253115	Lipids and lipid-like molecules	Fatty Acyls	Fatty acids and conjugates	*		50.7	54.6	M+FA-H	C6H8O2	0.332602557	18266.23932	18624.86266	19977.53747	29048.5865	26523.40144	26625.60474	23952.81965	26104.46825	26159.13681
4.18_211.0614m/z	211.0613726	4.181333333	neg	3,4-Dihydroxyphenylacetone	HMDB0031132	Benzenoids	Benzene and substituted derivatives	Phenylpropanes	*		50.7	56	M+FA-H	C9H10O3	1.057266611	13485.63199	11464.68944	11782.67569	3751.594881	3520.407473	3973.110326	9813.984663	7160.384556	7635.53648
4.99_140.0838n	185.0820271	4.99225	neg	Alepramic acid	LMFA01140031	Lipids and lipid-like molecules	Fatty Acyls	Fatty Acids and Conjugates			50.7	55.1	M-H, M+FA-H, M-H2O-H	C8H12O2	0.622918772	23720.82288	23041.23139	24369.05181	32980.32456	35272.40304	36591.14638	33186.66197	27758.76579	26527.9173
0.54_239.0600m/z	239.0599912	0.535283333	neg	Propyl 1-(propylsulfinyl)propyl disulfide	HMDB0033071	Organosulfur compounds	Sulfoxides	Unclassified			50.6	61.1	M-H	C9H20OS3	-1.502257978	10213.21729	840.1528322	906.1129368	713.5957591	2133.433297	12014.21144	752.4986621	2072.456744	470.4024318
1.72_182.0441n	181.0368524	1.721216667	neg	1-Methyluric acid	2821	Organoheterocyclic compounds	Imidazopyrimidines	Purines and purine derivatives	**	C16359	50.6	60	M-H, 2M-H	C6H6N4O3	0.762074577	150498.2567	156328.2347	139955.184	172907.5222	173629.8594	172062.815	161079.1436	159315.0056	189541.8964
3.28_204.9813m/z	204.9812701	3.280233333	neg	Pyrogallol-1-O-sulphate	HMDB0060016	Organic acids and derivatives	Organic sulfuric acids and derivatives	Arylsulfates	*		50.6	58.3	M-H	C6H6O6S	0.182427092	2945850.428	3065593.986	3050080.986	603150.4824	569451.8993	569473.0582	2540766.164	2769388.06	2663755.572
3.77_203.0828m/z	203.082783	3.770416667	neg	Nicotyrine	HMDB0255591	Unclassified	Unclassified	Unclassified			50.6	55.1	M+FA-H	C10H10N2	1.150276808	12607.62077	10304.91056	11181.03285	22452.72687	21805.32029	14374.26283	12984.86441	14358.52502	14392.36081
3.80_211.0612m/z	211.061214	3.801716667	neg	Ethyl salicylate	HMDB0029817	Benzenoids	Benzene and substituted derivatives	Benzoic acids and derivatives	*		50.6	55.6	M+FA-H	C9H10O3	0.102147603	4204.477342	4113.443543	4412.474044	3679.732664	3792.107153	3521.893823	2873.600923	2989.124445	3142.881224
5.26_287.1502m/z	287.150229	5.263033333	neg	(3S,5R,6R,7E)-3,5,6-Trihydroxy-7-megastigmen-9-one	HMDB0038736	Lipids and lipid-like molecules	Prenol lipids	Sesquiterpenoids	*		50.6	55.8	M+FA-H	C13H22O4	0.895818903	13567.23024	11605.98284	10560.0697	13820.76224	13373.21943	13544.89425	10788.58434	8686.305835	11507.1857

3.85_227.1039m/z	227.1039364	3.84605	neg	gamma-Glutamylvaline	HMDB0011172	Organic acids and derivatives	Carboxylic acids and derivatives	Amino acids, peptides, and analogues	*		50.5	56.5	M-H2O-H	C10H18N2O5	0.836367785	4605.649543	5454.710621	5612.975448	5867.658231	5700.289706	5920.069828	4712.662217	5197.905651	4923.436495
5.24_199.0072m/z	199.0071767	5.244033333	neg	4-Vinylphenol sulfate	HMDB0062775	Organic acids and derivatives	Organic sulfuric acids and derivatives	Arylsulfates	*		50.5	58.8	M-H	C8H8O4S	0.616744442	107580.4039	94348.52716	97694.84126	6723.94277	6939.157812	7113.531819	91538.63601	79435.85399	96598.35132
0.84_132.0302m/z	132.030245	0.841733333	neg	L-Aspartic acid	HMDB0000191	Organic acids and derivatives	Carboxylic acids and derivatives	Amino acids, peptides, and analogues	**	C00049	50.4	55.5	M-H	C4H7NO4	0.102964292	5698.459666	6367.306582	6655.142355	6408.777146	7171.233727	6759.930989	6142.831924	6926.108499	7031.555656
3.73_343.0673m/z	343.0672742	3.730516667	neg	Isovanillic acid glucuronide	HMDB0240538	Unclassified	Unclassified	Unclassified			50.4	54.4	M-H	C14H16O10	0.592683993	73866.00472	67620.14538	73292.58588	21749.33942	22180.47524	16740.32788	47339.08267	53101.45362	53427.89718
5.85_433.2081m/z	433.2080936	5.84575	neg	9,13-Dihydroxy-4-megastigmen-3-one 9-glucoside	HMDB0036318	Lipids and lipid-like molecules	Fatty Acyls	Fatty acyl glycosides	*		50.4	53.6	M+FA-H	C19H32O8	0.445073667	444419.7061	482361.4146	496523.3796	396903.7542	400951.8953	401871.2899	607512.0878	565818.575	592007.6873
2.28_164.0717m/z	164.0717191	2.2828	neg	L-Phenylalanine	28	Organic acids and derivatives	Carboxylic acids and derivatives	Amino acids, peptides, and analogues	**	C00079	50.3	53.9	M-H	C9H11NO2	0.102749486	8200.4363	8369.920631	7636.046187	12348.22016	11894.54052	12378.52517	10199.95764	11199.78388	10950.26846
7.92_465.2495m/z	465.2495276	7.91555	neg	Androsterone glucuronide	HMDB0002829	Lipids and lipid-like molecules	Steroids and steroid derivatives	Steroidal glycosides	**	C11135	50.3	53.1	M-H	C25H38O8	0.291399507	401955.193	396258.5944	410100.2363	525221.6373	520812.4289	549603.2769	402272.8868	495106.831	462137.2615
4.14_261.0075m/z	261.0075018	4.136366667	neg	3-(3,5-dihydroxyphenyl)-1-propanoic acid sulphate	HMDB0061117	Benzenoids	Phenols	Benzenediols	*		50.2	56.2	M-H	C9H10O7S	0.20815897	13573.94282	13513.87579	14153.5679	31808.56298	26599.84009	29201.79838	15524.82518	13308.58703	13070.00924
6.39_213.1134m/z	213.1134455	6.386066667	neg	Aleprestic acid	LMFA01030188	Lipids and lipid-like molecules	Fatty Acyls	Fatty Acids and Conjugates			50.2	53.4	M+FA-H	C10H16O2	1.266446841	13193.68308	14355.65872	15468.82437	22541.85691	22895.3008	23693.58159	14792.8456	14847.24592	14823.9879
6.91_227.1291m/z	227.1290927	6.9072	neg	Traumatic acid	HMDB0000933	Lipids and lipid-like molecules	Fatty Acyls	Fatty acids and conjugates	***	C16308	50.2	56.5	M-H	C12H20O4	0.92077138	10655.31317	11174.45781	10488.4779	15166.09282	15319.44892	14981.94279	9271.121136	9984.440198	9748.669519
0.78_325.0945m/z	325.0944988	0.778533333	neg	6-O-p-Coumaroyl-D-glucose	HMDB0039169	Phenylpropanoids and polyketides	Cinnamic acids and derivatives	Hydroxycinnamic acids and derivatives			50.1	64.7	M-H	C15H18O8	4.93004813	23707.07588	22867.95562	27326.31157	8948.445922	14801.25167	11372.59467	22381.2879	31011.36841	39126.12904
1.16_111.0088m/z	111.0087811	1.160166667	neg	2-Hydroxyglutaric acid lactone	HMDB0059743	Organoheterocyclic compounds	Lactones	Gamma butyrolactones			50.1	52	M-H2O-H	C5H6O4	0.103953425	8400.751485	9724.54989	11944.08608	13235.9305	13126.88197	12983.67898	10524.6067	11625.85478	12979.63995
1.20_160.0616m/z	160.0616431	1.202333333	neg	Glucosamine	HMDB0001514	Organic oxygen compounds	Organooxygen compounds	Carbohydrates and carbohydrate conjugates	**	C00329	50.1	53.4	M-H2O-H	C6H13NO5	0.62354222	10264.28208	10514.0331	11184.6715	13128.53994	12792.05604	14008.6264	10787.97863	12198.20235	12944.87095
6.22_218.0824m/z	218.0823752	6.215	neg	Salsoline-1-carboxylate	HMDB0013067	Organoheterocyclic compounds	Tetrahydroisoquinolines	Unclassified	*		50.1	54.4	M-H2O-H	C12H15NO4	0.456959489	3523.143781	2656.671859	3544.82604	1199.508732	1190.95251	1224.402676	3344.03145	3406.034639	3480.945556
0.74_104.0354m/z	104.0353629	0.7359	neg	L-Serine	30	Organic acids and derivatives	Carboxylic acids and derivatives	Amino acids, peptides, and analogues	**	C00065	50	51.6	M-H	C3H7NO3	0.439937707	1366.171856	922.5363625	1308.425665	1137.615636	2460.224095	1920.508633	995.6570042	1836.225195	1991.374382
3.44_194.0459m/z	194.0459083	3.440016667	neg	3-Hydroxyhippuric acid	HMDB0006116	Benzenoids	Benzene and substituted derivatives	Benzoic acids and derivatives	*		50	51.8	M-H	C9H9NO4	0.138121075	119897.3707	138490.056	141535.6599	75191.84904	73125.97987	74809.90787	133252.6125	124871.8459	126518.4469
4.11_306.0411n	287.0232829	4.113883333	neg	4-hydroxy-5-[4-hydroxy-3-(sulfooxy)phenyl]pentanoic acid	HMDB0127746	Lipids and lipid-like molecules	Fatty Acyls	Fatty acids and conjugates	*		50	54.8	M-H2O-H, M+FA-H	C11H14O8S	0.606570723	17889.79418	18045.73118	18334.70527	24101.92597	19427.8595	21441.51468	81008.10851	79279.21749	78340.49457
4.48_210.0774m/z	210.0773799	4.477166667	neg	4-Hydroxy-1-(3-pyridinyl)-1-butanone	HMDB0062402	Organic oxygen compounds	Organooxygen compounds	Carbonyl compounds	**	C19565	50	53.4	M+FA-H	C9H11NO2	1.202253872	2645.109966	2869.718482	2987.123647	654.4181163	774.031023	616.2900998	662.218227	696.857234	859.5126291
4.66_259.1188m/z	259.1187904	4.660483333	neg	alpha-Carboxy-delta-decalactone	HMDB0030985	Organoheterocyclic compounds	Lactones	Delta valerolactones	*		50	51	M+FA-H	C11H18O4	0.366646188	36370.8902	35227.01946	38377.44439	79043.11901	74513.80584	81498.52511	48040.44445	52902.24469	38750.46819
3.75_204.0303m/z	204.03029	3.747833333	neg	Xanthurenic acid	HMDB0000881	Organoheterocyclic compounds	Quinolines and derivatives	Quinoline carboxylic acids	***	C02470	49.9	51.6	M-H	C10H7NO4	0.286232944	5428.831194	4681.661416	4873.727613	10249.02333	9811.385954	6861.216815	5396.180445	6290.300109	6123.390078
3.82_123.0452m/z	123.0452106	3.82415	neg	4-Methylcatechol	64945	Benzenoids	Phenols	Benzenediols	**	C06730	49.9	52	M-H	C7H8O2	0.463418848	1370.988083	1438.775208	1447.307223	1340.020192	1271.60479	1088.176006	1131.142937	1265.920171	1213.480222
4.54_217.1083m/z	217.1082548	4.54425	neg	(3R,6S)-3-Hydroxy-1,7-dioxaspiro[5.5]undecane	LMPK09000005	Unclassified	Unclassified	Unclassified			49.9	53.7	M+FA-H	C9H16O3	0.624856739	6337.28196	7268.16224	7012.111959	7394.520238	7267.678246	7349.920251	5306.127106	5755.158203	6357.289207
4.95_445.0782m/z	445.0781732	4.94705	neg	Genistein 5-O-glucuronide	HMDB0041738	Phenylpropanoids and polyketides	Isoflavonoids	Isoflavonoid O-glycosides	*		49.9	55.6	M-H	C21H18O11	1.206483317	347.4360035	246.3325995	366.5580062	19292.62244	17962.51835	18698.85734	65.46482111	44.50161153	0.001403199
5.49_194.0459m/z	194.0459339	5.489016667	neg	N-Acetyl-5-aminosalicylic acid	1146	Benzenoids	Benzene and substituted derivatives	Benzoic acids and derivatives	*		49.9	53.4	M-H	C9H9NO4	0.26936731	2177.763349	1471.545019	1500.802291	1653.52547	1629.778848	2094.132672	2251.748438	1728.706906	1742.615811
5.95_218.0823m/z	218.0823471	5.95155	neg	N-Deschlorobenzoyl indomethacin	HMDB0013988	Organoheterocyclic compounds	Indoles and derivatives	Indolyl carboxylic acids and derivatives	*		49.9	54.2	M-H	C12H13NO3	0.36651188	2854.611025	3079.289797	3158.30637	2877.663044	3712.608979	3683.985575	2555.784083	2984.646581	2780.068297
0.80_168.9813m/z	168.9812592	0.800366667	neg	(S)-3-Sulfonatolactate	HMDB0060176	Organic acids and derivatives	Hydroxy acids and derivatives	Alpha hydroxy acids and derivatives	**	C11499	49.8	54.5	M-H	C3H6O6S	0.156927299	20506.92171	23404.15355	24135.01251	24533.29258	27115.94696	25311.33098	22924.25645	23925.49369	24549.53431
3.89_261.0075m/z	261.0075174	3.8906	neg	Homovanillic acid sulfate	HMDB0011719	Organic acids and derivatives	Organic sulfuric acids and derivatives	Arylsulfates	*		49.8	56.8	M-H	C9H10O7S	0.267746155	24409.0463	26321.43859	27877.88681	21689.82754	20257.98889	20569.68819	20476.11205	22513.49363	22375.58169
5.08_236.0930m/z	236.0929915	5.080566667	neg	(E)-5-(3,4,5,6-Tetrahydro-3-pyridylidenemethyl)-2-furanmethanol	HMDB0033560	Organoheterocyclic compounds	Pyridines and derivatives	Hydropyridines			49.8	51.2	M+FA-H	C11H13NO2	0.837015484	8964.218789	8961.453116	9012.141125	9589.872229	7343.793527	8305.544135	12449.74149	10385.23396	10416.52953
5.83_185.1184m/z	185.1183806	5.825	neg	2,6-Nonadien-1-ol	HMDB0031262	Lipids and lipid-like molecules	Fatty Acyls	Fatty alcohols	*		49.8	54.3	M+FA-H	C9H16O	0.446416789	2215.39646	2334.095027	2274.205443	3852.137436	2700.101734	2940.084216	2808.705514	2629.115235	2390.857184
5.85_423.0759m/z	423.0759033	5.84575	neg	Enterolactone 3'-sulfate	HMDB0240505	Unclassified	Unclassified	Unclassified			49.8	59.8	M+FA-H	C18H18O7S	0.995961915	21989.63157	24944.76824	25426.78545	4370.40361	3157.112065	3134.16178	593.1187018	467.4948605	516.6968925
7.24_357.0620m/z	357.061962	7.237866667	neg	PSOROMIC ACID	43642	Unclassified	Unclassified	Unclassified			49.8	55.3	M-H	C18H14O8	1.036212885	20157.96753	21152.06238	18221.97191	20233.31128	23390.20336	21416.83135	23193.75293	21131.02085	21129.82529
7.35_567.3181m/z	567.3180629	7.346316667	neg	(3alpha,5beta,7alpha)-23-Carboxy-7-hydroxy-24-norcholan-3-yl-beta-D-Glucopy	HMDB0243600	Unclassified	Unclassified	Unclassified			49.8	57.9	M-H	C30H48O10	1.040921993	64317.47988	68173.64555	64764.49662	10859.35585	10950.524	10707.13907	27543.4456	33502.08998	35831.92942
1.27_189.0406m/z	189.0406411	1.26875	neg	2-O-Methylascorbic acid	HMDB0240294	Organoheterocyclic compounds	Dihydrofurans	Furanones			49.7	50.5	M-H	C7H10O6	0.944640243	24811.83448	31724.13936	35803.98697	51104.38158	48450.95728	47906.94783	28188.21664	33867.21759	32142.05495
2.07_245.1143m/z	245.1143356	2.070366667	neg	Glutamylvaline	HMDB0028832	Organic acids and derivatives	Carboxylic acids and derivatives	Amino acids, peptides, and analogues	*		49.7	51.4	M-H	C10H18N2O5	0.163842702	14765.89409	16589.19567	16965.14357	29668.87776	33083.56689	32526.51984	22596.50165	21657.7789	22226.00069
5.53_339.1087m/z	339.1086909	5.525416667	neg	trans-isoeugenol-O-glucuronide	HMDB0060021	Organic oxygen compounds	Organooxygen compounds	Carbohydrates and carbohydrate conjugates	*		49.7	52.4	M-H	C16H20O8	0.440173133	392049.6106	331039.0363	335670.0957	116019.7858	151205.7504	127937.5492	279872.9775	287912.8451	342341.8626
1.40_211.0475m/z	211.0474504	1.3958	neg	3-Methylxanthine	HMDB0001886	Organoheterocyclic compounds	Imidazopyrimidines	Purines and purine derivatives	**	C16357	49.6	51.7	M+FA-H	C6H6N4O2	1.036307941	6914.814439	5513.343357	6003.512418	13336.88384	11923.26463	10685.32526	10118.64975	9546.753281	9470.789657
4.16_269.0668m/z	269.0668427	4.15895	neg	Phenyl glucuronide	1825	Organic oxygen compounds	Organooxygen compounds	Carbohydrates and carbohydrate conjugates	*		49.6	51.3	M-H	C12H14O7	0.615794744	8575.477352	8526.911294	8741.51106	10921.78598	7498.758942	8906.922127	14724.72224	10730.04767	11203.00452
4.54_250.0723m/z	250.0722616	4.54425	neg	4-Hydroxy-3-methoxy-cinnamoylglycine	HMDB0059747	Organic acids and derivatives	Carboxylic acids and derivatives	Amino acids, peptides, and analogues		C02564	49.6	51.7	M-H	C12H13NO5	0.659250465	11238.2968	11552.96058	12515.51228	8826.726444	8478.750618	8155.089681	8489.983035	8529.660565	8984.627132
4.79_351.0957n	350.0884143	4.792316667	neg	Indole-3-acetic-acid-O-glucuronide	HMDB0060001	Organic oxygen compounds	Organooxygen compounds	Carbohydrates and carbohydrate conjugates	*		49.6	52.8	M-H, 2M-H	C16H17NO8	0.780925824	153191.7259	168458.3003	164084.6768	251882.5431	248890.0916	244770.1302	132049.5639	146328.5416	140893.4973
5.10_211.0614m/z	211.0614022	5.1031	neg	Eudesmic acid	44069	Benzenoids	Benzene and substituted derivatives	Benzoic acids and derivatives			49.6	52.2	M-H	C10H12O5	0.967209423	4782.974646	4591.583392	4815.616573	8325.130638	7772.108197	8599.81072	7338.991135	5607.795829	6231.360811
5.27_611.2690m/z	611.2690371	5.271716667	neg	Canescein	HMDB0029313	Organoheterocyclic compounds	Isobenzofurans	Unclassified	*		49.6	56.7	M+FA-H	C29H42O11	-3.316387417	34514.33445	24742.69372	26267.80721	20731.10172	22279.79604	21873.69358	30655.46515	23331.41981	29188.02042
6.11_211.0978m/z	211.0977732	6.113516667	neg	2-Hydroxy-p-mentha-1,8-dien-6-one	HMDB0037012	Lipids and lipid-like molecules	Prenol lipids	Monoterpenoids	*		49.6	51.9	M+FA-H	C10H14O2	1.14753723	7835.891613	8838.040323	8873.533104	9614.62622	10396.85321	10252.01781	6407.795375	7214.924624	7304.308206
8.29_489.2709m/z	489.2709052	8.294766667	neg	12-O-D-Glucuronoside-13-hydroxyoctadec-9Z-enoate	HMDB0060119	Lipids and lipid-like molecules	Saccharolipids	Unclassified	*		49.6	51.3	M-H	C24H42O10	0.783376903	143839.9667	164449.9818	173067.1998	157562.7095	136231.6618	142721.2539	132665.69	153326.2862	149992.8539
5.01_461.0727m/z	461.0726996	5.014716667	neg	Kaempferol 3-glucuronide	LMPK12111843	Phenylpropanoids and polyketides	Flavonoids	Flavonoid glycosides	*		49.5	52.7	M-H	C21H18O12	0.32463743	4958.840682	4942.929838	5238.018345	22421.7653	23544.92662	24924.92838	9289.659661	9102.632626	9777.456414
5.06_295.0825m/z	295.0825397	5.05975	neg	Tyrosol glucuronide	HMDB0240563	Organic oxygen compounds	Organooxygen compounds	Carbohydrates and carbohydrate conjugates			49.5	51.6	M-H2O-H	C14H18O8	0.679104541	21191.25059	21363.47514	21737.61412	4514.199778	4413.039106	4753.658228	25725.59266	25875.82332	33770.45794
5.30_617.1487m/z	617.1486645	5.302883333	neg	Rhamnazin 3-[6"-(3-hydroxy-3-methylglutaryl)glucoside]	LMPK12112636	Lipids and lipid-like molecules	Polyketides	Flavonoids			49.4	56.7	M-H2O-H	C29H32O16	-3.975806101	17144.27341	16062.18757	16997.62316	0.001403199	161.7381735	0.001403199	1670.059788	991.0997772	1710.467185
5.57_199.0977m/z	199.0977156	5.57225	neg	5,7-nonadienoic acid	LMFA01030450	Lipids and lipid-like molecules	Fatty Acyls	Fatty Acids and Conjugates			49.4	49.5	M+FA-H	C9H14O2	0.863043765	20292.42078	17951.85807	18502.78861	24289.56987	27692.81126	28130.14625	19145.14182	17209.69264	17965.4149
15.05_165.0409m/z	165.0408561	15.05453333	neg	S-Propyl 1-propanesulfinothioate	HMDB0034394	Organic acids and derivatives	Thiosulfinic acid esters	Unclassified			49.3	55.7	M-H	C6H14OS2	-2.857982706	32919.32838	158369.9305	18586.42073	103660.9678	51079.8735	23296.07163	135230.7162	16759.14953	18439.37852
4.16_252.0878m/z	252.0878353	4.15895	neg	N-lactoyl-Tyrosine	HMDB0062177	Organic acids and derivatives	Carboxylic acids and derivatives	Amino acids, peptides, and analogues			49.3	49.8	M-H	C12H15NO5	0.352063189	6211.59683	6104.384533	6381.488164	6022.01131	4272.24666	4364.404557	9384.11241	7022.73761	6785.789966
4.99_219.1239m/z	219.1239224	4.99225	neg	9-hydroxy pelargonic acid	LMFA01050028	Lipids and lipid-like molecules	Fatty Acyls	Fatty Acids and Conjugates			49.3	48.7	M+FA-H	C9H18O3	0.718590738	23064.17695	25247.03518	25913.6436	21730.75891	21641.26654	22431.7315	26142.18938	22686.52373	22671.41804
7.35_401.0880m/z	401.0880017	7.353466667	neg	Dihydrohydroxy-O-methylsterigmatocystin	HMDB0033659	Phenylpropanoids and polyketides	Sterigmatocystins	Unclassified			49.3	50.4	M+FA-H	C19H16O7	0.550406694	146522.3129	138084.6498	123400.4931	129718.5332	143945.5864	139235.4194	148882.5535	140822.6093	148197.8977
4.43_233.0126m/z	233.0125525	4.432683333	neg	o-Cresol sulfate	HMDB0240655	Organic acids and derivatives	Organic sulfuric acids and derivatives	Arylsulfates			49.2	48.3	M+FA-H	C7H8O4S	0.105755244	52211.68345	57564.1746	57804.07223	10701.17046	9908.602133	9751.624701	46830.68837	54565.73757	51742.09921
4.79_209.0457m/z	209.0456757	4.792316667	neg	5-Hydroxyferulic acid	HMDB0035484	Phenylpropanoids and polyketides	Cinnamic acids and derivatives	Hydroxycinnamic acids and derivatives	**	C05619	49.2	48.7	M-H	C10H10O5	0.612699398	90409.45052	86305.34597	85095.78388	90741.28399	98124.36781	100027.7923	107515.5967	93917.8733	94838.10348
4.88_163.0402m/z	163.0401989	4.880083333	neg	m-Coumaric acid	HMDB0001713	Phenylpropanoids and polyketides	Cinnamic acids and derivatives	Hydroxycinnamic acids and derivatives	***	C12621	49.2	50.2	M-H	C9H8O3	0.799640437	2435.745882	2556.879918	2707.911482	965.3991184	903.2983746	1003.625565	1703.926381	1771.001986	1905.701101
5.73_203.1289m/z	203.1288936	5.727283333	neg	3,10-dihydroxydecanoic acid	LMFA01050429	Lipids and lipid-like molecules	Fatty Acyls	Fatty Acids and Conjugates			49.2	48.8	M-H	C10H20O4	0.053261404	7088.377895	7593.054946	6457.270319	5233.767622	6780.543854	6005.671434	7435.584828	8718.314818	8335.670216
1.80_202.0723m/z	202.0722972	1.798266667	neg	N-Acetylproline	HMDB0094701	Organic acids and derivatives	Carboxylic acids and derivatives	Amino acids, peptides, and analogues			49.1	49	M+FA-H	C7H11NO3	1.280174662	5481.092695	5208.51339	5577.775119	5443.748521	5423.446702	6150.62695	5359.021262	6028.737625	5654.969429
5.89_138.0197m/z	138.019716	5.894166667	neg	4-Nitrophenol	4100	Benzenoids	Phenols	Nitrophenols	*	C00870	49.1	51	M-H	C6H5NO3	0.355529708	3710.479252	3230.668742	3508.065615	3148.381234	3874.524822	3604.376592	3920.886599	3822.793406	4251.525363
5.91_517.1355m/z	517.1354617	5.908066667	neg	Medicarpin 3-O-(6'-malonylglucoside)	HMDB0038776	Phenylpropanoids and polyketides	Isoflavonoids	Furanoisoflavonoids			49.1	48.9	M-H	C25H26O12	0.601914156	72705.22567	86448.46558	78156.5948	70297.94641	84553.46118	70920.11322	22345.39392	27090.89682	29164.73398
5.99_196.0253m/z	196.0252606	5.9916	neg	3-Hydroxy-2-methylpyridine-4,5-dicarboxylate	HMDB0006955	Organoheterocyclic compounds	Pyridines and derivatives	Pyridinecarboxylic acids and derivatives	**	C04604	49.1	49.5	M-H	C8H7NO5	0.581935985	8281.642657	2449.916802	4122.405554	803.5873279	1051.434516	4549.883523	1027.64032	1068.138013	4781.726302
4.54_429.0829m/z	429.0829237	4.54425	neg	Daidzein 4'-O-glucuronide	HMDB0041717	Organic acids and derivatives	Organic sulfuric acids and derivatives	Arylsulfates	*		49	50.2	M-H	C21H18O10	0.472898993	3406.343905	3572.748232	3421.445296	10876.49062	10416.65945	10041.70292	206.7175111	195.6644887	192.1196465
4.73_243.1350m/z	243.1350345	4.725416667	neg	Hydroxyprolyl-Leucine	HMDB0028867	Organic acids and derivatives	Carboxylic acids and derivatives	Amino acids, peptides, and analogues	*		49	54.9	M-H	C11H20N2O4	0.015468928	33009.68965	32710.08828	34895.4468	73305.36004	73673.82686	73166.07678	35410.53309	39096.534	38255.53727
5.36_255.1240m/z	255.1239718	5.361783333	neg	2-furan-octanoic acid	LMFA01150037	Lipids and lipid-like molecules	Fatty Acyls	Fatty Acids and Conjugates			49	49.8	M+FA-H	C12H18O3	0.830215591	7136.553418	7605.510597	8436.319678	6553.061083	8312.744883	7551.759302	4604.067606	5250.66685	5731.87619
6.46_435.2243m/z	435.2242673	6.458366667	neg	(3S,5R,6S,7E,9x)-7-Megastigmene-3,6,9-triol 9-glucoside	HMDB0041176	Lipids and lipid-like molecules	Fatty Acyls	Fatty acyl glycosides	*		49	49.1	M+FA-H	C19H34O8	1.784580559	16495.12038	14590.09054	16951.36484	18561.26141	15257.38949	16769.16714	19639.73183	20258.46369	19914.83155
6.69_267.1239m/z	267.1239432	6.69475	neg	3,4-dimethyl-5-carboxyethyl-2-furanpentanoic acid	LMFA01150055	Lipids and lipid-like molecules	Fatty Acyls	Fatty Acids and Conjugates			49	50.1	M-H	C14H20O5	0.544248201	5132.177139	6063.456119	6107.867302	7135.677701	6801.002593	6692.502722	4925.222363	6066.059598	6328.953304
0.86_254.9816m/z	254.9816395	0.86335	neg	Ascorbic acid 3-sulfate	HMDB0240641	Organoheterocyclic compounds	Dihydrofurans	Furanones			48.9	48.6	M-H	C6H8O9S	0.050992854	212868.6979	239984.9832	270811.4759	553953.4736	504464.9608	523868.1543	269466.4022	358784.6132	329150.5734
2.92_168.0303m/z	168.0302916	2.9156	neg	2-Furoylglycine	5428	Organic acids and derivatives	Carboxylic acids and derivatives	Amino acids, peptides, and analogues	*		48.9	47.1	M-H	C7H7NO4	0.356648469	29081.10344	29872.4391	32038.70456	21478.9165	20085.6907	21959.84151	20644.63308	21707.51133	20289.96443
3.48_378.1034m/z	378.1033598	3.484883333	neg	Hyaluronan biosynthesis, precursor 1	HMDB0060071	Organic oxygen compounds	Organooxygen compounds	Carbohydrates and carbohydrate conjugates		C04794	48.9	52.2	M-H	C14H21NO11	-2.174088528	17164.77403	21393.32552	21432.03262	27535.58374	23506.03747	24778.84578	22215.09817	22055.67371	21687.47634
3.91_214.1087m/z	214.1087043	3.912566667	neg	2-amino-8-oxo-9,10-epoxy-decanoic acid	LMFA01060176	Lipids and lipid-like molecules	Fatty Acyls	Fatty Acids and Conjugates		C12027	48.9	49.1	M-H	C10H17NO4	1.035248964	3797.35949	3876.262239	4319.295275	2628.860964	2351.164168	2617.947127	3447.597577	3650.584692	3675.620934
4.34_203.0927m/z	203.0926521	4.344733333	neg	Mycinonic acid I	LMFA01020402	Lipids and lipid-like molecules	Fatty Acyls	Fatty Acids and Conjugates			48.9	47.5	M+FA-H	C8H14O3	0.97977752	2040.828503	2343.359715	2415.882985	2931.387622	2398.504344	2462.446771	1733.040975	2297.327641	2087.032566
5.10_192.0668m/z	192.0668183	5.1031	neg	4-Methylhippuric acid	64936	Benzenoids	Benzene and substituted derivatives	Benzoic acids and derivatives	*		48.9	54	M-H	C10H11NO3	1.043491322	1890.960055	1785.953249	1766.333887	2398.003788	2657.637137	2883.232009	3294.753362	2792.740834	3110.361486
6.03_353.0339m/z	353.0339325	6.028183333	neg	cis-Resveratrol 4'-sulfate	HMDB0041714	Phenylpropanoids and polyketides	Stilbenes	Unclassified	*		48.9	51.9	M+FA-H	C14H12O6S	0.878324178	20010.20029	20510.88257	22400.38764	0.001403199	0.001403199	0.001403199	1078.722658	1193.87655	1268.148284
8.61_489.2709m/z	489.2708905	8.606866667	neg	9-Hydroxy-10-O-D-glucuronoside-12Z-octadecenoate	HMDB0060121	Lipids and lipid-like molecules	Saccharolipids	Unclassified	*		48.9	47.8	M-H	C24H42O10	0.75344944	154281.9657	158003.2577	152794.0425	180843.9289	167270.8278	176538.9785	161615.9533	168029.9507	156612.9262
4.37_369.0287m/z	369.0286842	4.367166667	neg	Catechin 3'-sulfate	HMDB0240485	Phenylpropanoids and polyketides	Flavonoids	Flavans			48.8	50.7	M-H	C15H14O9S	0.290882731	37447.26474	39477.30977	42121.52652	26505.2851	20377.53559	21939.4901	24175.59177	31001.43924	28711.19263
4.50_157.0507m/z	157.0506841	4.49925	neg	Parasorbic acid	HMDB0301801	Unclassified	Unclassified	Unclassified			48.8	46.9	M+FA-H	C6H8O2	0.461647467	881.5034398	1013.532098	945.2765219	691.3752443	589.711037	741.8413768	2144.640216	2236.771019	2390.535006
4.86_379.1613m/z	379.1612673	4.858383333	neg	Prenyl arabinosyl-(1->6)-glucoside	HMDB0041360	Lipids and lipid-like molecules	Fatty Acyls	Fatty acyl glycosides	*		48.8	48.6	M-H	C16H28O10	0.780375182	125159.0374	130439.6266	140318.8606	146529.9962	147958.431	148594.7331	150206.378	169549.9473	158787.4419
4.97_471.1852m/z	471.1852371	4.969683333	neg	11,13-Dihydrotaraxinic acid glucosyl ester	HMDB0035867	Lipids and lipid-like molecules	Saccharolipids	Unclassified	*		48.8	58.3	M+FA-H	C21H30O9	-4.571522953	8405.668114	8719.29363	8592.275911	7498.69011	7323.88535	7388.607514	11922.5095	10780.15262	10176.73699
6.66_255.1240m/z	255.1239524	6.663683333	neg	Sedanonic acid	HMDB0032871	Organic acids and derivatives	Keto acids and derivatives	Gamma-keto acids and derivatives			48.8	46.7	M+FA-H	C12H18O3	0.73805852	8970.312055	10213.57508	10233.11177	6063.582464	5850.431264	5388.214439	3411.701511	4153.645294	4270.030912
1.53_215.1038m/z	215.1038139	1.534483333	neg	Etiracetam	HMDB0252105	Organic acids and derivatives	Carboxylic acids and derivatives	Amino acids, peptides, and analogues		C07841	48.7	44.1	M+FA-H	C8H14N2O2	0.489713098	17618.44154	14709.26028	15515.00126	14524.70203	13432.93928	13841.11623	15146.88398	17965.48849	16252.28916
2.14_299.0635m/z	299.0635265	2.140766667	neg	beta-D-3-Ribofuranosyluric acid	HMDB0029920	Organoheterocyclic compounds	Imidazopyrimidines	Purines and purine derivatives	***	C05513	48.7	51	M-H	C10H12N4O7	0.68058657	5651.523378	6656.50064	6227.150256	7801.230006	7190.217307	7281.161942	5345.609345	6423.996401	6817.323946
3.94_302.0680m/z	302.0680097	3.9351	neg	10-Acetyl-3,7-dihydroxyphenoxazine	HMDB0244436	Unclassified	Unclassified	Unclassified			48.7	58.4	M+FA-H	C14H11NO4	3.886137658	5068.211566	4764.750871	5759.133713	8474.697657	7483.121763	7935.324825	10443.02081	11027.38466	10908.28834
4.11_301.1295m/z	301.1294871	4.113883333	neg	2-[4-(3-Hydroxypropyl)-2-methoxyphenoxy]-1,3-propanediol	HMDB0040352	Benzenoids	Phenol ethers	Anisoles			48.7	46.4	M+FA-H	C13H20O5	0.821615195	5811.825996	6165.793375	6414.799927	6980.133156	6358.378211	5967.087397	5892.261349	6472.625415	6119.846188
4.19_336.0728m/z	336.0728293	4.189783333	neg	2,8-Dihydroxyquinoline-beta-D-glucuronide	HMDB0011658	Organic oxygen compounds	Organooxygen compounds	Carbohydrates and carbohydrate conjugates	*		48.7	47.5	M-H	C15H15NO8	1.006679624	26902.84703	19854.58944	21427.65493	15731.94326	13729.91417	20696.63282	32227.9516	22955.36116	23212.7902
5.22_405.1768m/z	405.1768158	5.222166667	neg	1-O-(8R-hydroxy-8-methyl-3Z,9-decadienoyl)-beta-D-glucopyranose	LMFA13010039	Lipids and lipid-like molecules	Fatty Acyls	Fatty acyl glycosides			48.7	44.7	M+FA-H	C17H28O8	0.541646569	483737.5731	469903.3374	462489.0847	468108.4905	449736.7716	447511.9978	591105.6213	577291.2356	615746.2121
8.79_303.1451m/z	303.1451318	8.791166667	neg	Butyl (S)-3-hydroxybutyrate glucoside	HMDB0031694	Lipids and lipid-like molecules	Fatty Acyls	Fatty acyl glycosides	*		48.7	47.3	M-H2O-H	C14H26O8	0.636765093	25538.86602	26927.0671	29931.89057	1255.527861	1318.441932	1288.450336	23539.34938	26828.79453	26205.01173
1.16_147.0300m/z	147.0299729	1.160166667	neg	Acetoacetic acid	LMFA01060003	Organic acids and derivatives	Keto acids and derivatives	Short-chain keto acids and derivatives	**	C00164	48.6	44.7	M+FA-H	C4H6O3	0.745046826	4037.342626	4680.734014	5461.537364	4880.312955	5220.326911	4644.553085	5043.722343	4766.618608	5601.556214
5.01_163.0402m/z	163.0402287	5.014716667	neg	4-Hydroxycinnamic acid	HMDB0002035	Phenylpropanoids and polyketides	Cinnamic acids and derivatives	Hydroxycinnamic acids and derivatives	***	C00811	48.6	48.3	M-H	C9H8O3	0.981387001	2425.452415	2575.653851	2602.599188	1066.595277	1197.615493	1282.181786	1547.972908	1533.38155	1406.793693
1.59_145.0740n	144.0666977	1.5934	neg	L-cis-4-(Hydroxymethyl)-2-pyrrolidinecarboxylic acid	HMDB0029425	Organic acids and derivatives	Carboxylic acids and derivatives	Amino acids, peptides, and analogues			48.5	50.3	M-H2O-H, M-H	C6H11NO3	0.569683902	26995.52496	23592.81651	24572.62385	23536.91669	22594.15817	23029.43859	25479.18553	26248.3723	26669.1393
1.96_195.0524m/z	195.052433	1.95955	neg	3,7-Dimethyluric acid	58072	Organoheterocyclic compounds	Imidazopyrimidines	Purines and purine derivatives	**	C16360	48.5	47.3	M-H	C7H8N4O3	0.353473461	3341.900354	3307.212303	2600.653001	4221.722248	4481.369774	4563.981692	3622.971524	4054.756085	3473.578487
3.80_325.0800n	324.07272	3.801716667	neg	Dihyroxy-1H-indole glucuronide I	HMDB0059997	Organic oxygen compounds	Organooxygen compounds	Carbohydrates and carbohydrate conjugates	*		48.5	45.3	M-H, M+FA-H	C14H15NO8	0.707526693	83076.81336	81017.54481	83569.58685	9398.822824	9297.078737	7600.943559	67807.82128	75523.69666	73605.83843
4.16_303.0181m/z	303.0181372	4.15895	neg	5-(3',4',5'-Trihydroxyphenyl)-gamma-valerolactone-3'-O-sulphate	HMDB0059985	Organic acids and derivatives	Organic sulfuric acids and derivatives	Arylsulfates	*		48.5	50.1	M-H	C11H12O8S	0.412084723	7164.2996	7091.234446	7172.675695	7820.061427	5799.815487	6488.228403	9636.54147	6711.42497	7395.247455
4.92_229.1082m/z	229.1082437	4.92485	neg	(4S,8R)-8,9-Dihydroxy-p-menth-1(6)-en-2-one	HMDB0039052	Lipids and lipid-like molecules	Prenol lipids	Monoterpenoids	*		48.5	46.6	M+FA-H	C10H16O3	0.523955787	2729.352817	2985.763539	3320.538157	3153.558138	3263.628601	3131.520327	2542.209427	2498.494721	2398.892898
6.04_223.0975m/z	223.0974701	6.040433333	neg	Diplosporin	HMDB0030680	Organoheterocyclic compounds	Pyrans	Pyranones and derivatives			48.5	47.2	M-H	C12H16O4	-0.501743132	20636.44	21774.30257	23206.07759	25748.79558	27400.01174	25715.90261	20266.15932	21789.04942	22422.55859
4.19_139.0400m/z	139.040033	4.189783333	neg	2-METHOXYRESORCINOL	44540	Benzenoids	Phenols	Methoxyphenols	*		48.4	45.8	M-H	C7H8O3	-0.248009642	3663.181913	3542.498404	3620.173578	1724.030645	1627.516565	1647.505767	3627.522218	3391.308354	3398.229586
4.21_203.0021m/z	203.0021313	4.212116667	neg	O-methoxycatechol-O-sulphate	HMDB0060013	Organic acids and derivatives	Organic sulfuric acids and derivatives	Arylsulfates	*		48.4	46.8	M-H	C7H8O5S	0.800932988	16990.83518	18167.736	18343.7753	11945.99618	10907.43621	11562.23651	17059.43173	18901.84225	18235.01862
4.86_261.1344m/z	261.1344366	4.858383333	neg	Undecanedioic acid	HMDB0000888	Lipids and lipid-like molecules	Fatty Acyls	Fatty acids and conjugates	*		48.4	45.8	M+FA-H	C11H20O4	0.345236845	7475.193643	7707.202733	7936.101017	5133.637547	5262.570701	4778.093803	5119.807861	5466.39009	5032.337791
1.49_161.0457m/z	161.0456588	1.492933333	neg	D-Fructose	HMDB0000660	Organic oxygen compounds	Organooxygen compounds	Carbohydrates and carbohydrate conjugates	***	C02336	48.3	45.3	M-H2O-H	C6H12O6	0.621042444	20598.46274	20970.39877	20831.33835	22371.6945	22326.82773	21477.56854	22238.65559	19534.59929	20653.17341
2.72_158.0823m/z	158.0823159	2.72215	neg	Epsilon-caprolactam	HMDB0062769	Organoheterocyclic compounds	Lactams	Caprolactams	**	C06593	48.3	42.1	M+FA-H	C6H11NO	0.433688984	42983.66116	43312.23418	45370.34751	41230.65441	42538.73685	42330.15491	50986.07713	56233.38193	56140.74439
3.98_209.0681m/z	209.0680697	3.97985	neg	1,3,7-Trimethyluric acid	HMDB0002123	Organoheterocyclic compounds	Imidazopyrimidines	Purines and purine derivatives	**	C16361	48.3	51.1	M-H	C8H10N4O3	0.266344362	34350.29736	36231.65484	38189.30593	36976.74994	35041.06219	34625.76501	33709.08462	39272.13056	36962.28846
4.61_336.0729m/z	336.0728988	4.611883333	neg	3-Indole carboxylic acid glucuronide	HMDB0013189	Lipids and lipid-like molecules	Fatty Acyls	Fatty acyl thioesters	*		48.3	46.7	M-H	C15H15NO8	1.21285499	26034.75263	24030.03338	25939.112	39965.10823	41132.79951	33299.75691	31693.12635	25076.92543	38025.28306
5.16_257.1509m/z	257.1508771	5.155116667	neg	1,4'-Bipiperidine-1'-carboxylic acid	HMDB0060336	Organoheterocyclic compounds	Piperidines	Piperidinecarboxylic acids and derivatives		C16836	48.3	42.9	M+FA-H	C11H20N2O2	0.925392123	6784.947153	8203.288353	7302.482043	12780.53697	12002.04713	11950.29908	7431.148089	8233.68594	8314.452513
9.02_408.2878n	407.2805251	9.01635	neg	Allocholic acid	5491	Lipids and lipid-like molecules	Steroids and steroid derivatives	Bile acids, alcohols and derivatives	*	C17737	48.3	44.9	M-H, M+FA-H	C24H40O5	0.556417509	436453.658	547112.4376	530825.0244	50806.15671	52946.68209	51200.65397	216290.437	240034.4068	236706.5318
0.88_173.0568m/z	173.0567658	0.884633333	neg	N-Acetylasparagine	HMDB0006028	Organic acids and derivatives	Carboxylic acids and derivatives	Amino acids, peptides, and analogues	*		48.2	45	M-H	C6H10N2O4	-0.083538636	7762.993508	8329.668794	7471.793442	10533.58316	9211.285126	10763.81255	10377.08348	11431.9103	11398.4439
0.90_119.0350m/z	119.034978	0.904683333	neg	2,4-Dihydroxybutanoic acid	HMDB0000360	Organic acids and derivatives	Hydroxy acids and derivatives	Short-chain hydroxy acids and derivatives	*		48.2	41.8	M-H	C4H8O4	-0.036225786	49943.79861	53819.64441	55243.97634	82792.20115	76226.19658	79978.65847	55413.96107	59273.84474	58914.79236
2.54_285.0962n	284.0889383	2.5421	neg	N4-Acetylcytidine	HMDB0005923	Nucleosides, nucleotides, and analogues	Pyrimidine nucleosides	Unclassified	*		48.2	44.3	M-H, M+FA-H	C11H15N3O6	0.454252768	21945.21184	25630.43293	26804.94709	32247.92368	30822.38726	30540.73917	23762.26472	27062.22873	27958.35261
3.78_481.0991m/z	481.0990942	3.779133333	neg	Taxifolin 3-arabinoside	HMDB0041418	Phenylpropanoids and polyketides	Flavonoids	Flavonoid glycosides			48.2	43.3	M+FA-H	C20H20O11	0.756349306	25301.98866	18756.34429	18639.68082	11706.84897	11028.61668	9583.458302	13936.15027	14684.09823	14227.12803
4.61_222.0410m/z	222.0409858	4.611883333	neg	4-(2-Amino-3-hydroxyphenyl)-2,4-dioxobutanoic acid	HMDB0004083	Organic oxygen compounds	Organooxygen compounds	Carbonyl compounds	**	C05645	48.2	44.9	M-H	C10H9NO5	0.85103923	3563.412165	3459.376564	3351.715775	3096.662695	3258.490239	2493.410085	4122.229259	3451.970186	4999.374982
4.64_271.0284m/z	271.0283885	4.641416667	neg	5-(Hydroxyphenyl)-gamma-valerolactone-O-sulphate	HMDB0059993	Organic acids and derivatives	Organic sulfuric acids and derivatives	Arylsulfates	*		48.2	46.7	M-H	C11H12O6S	0.75678461	6561.8281	6199.28935	6337.426628	461.0070695	363.2634902	532.607058	24722.95374	26403.54757	20861.41525
5.20_381.1015m/z	381.1015445	5.20005	neg	Enterodiol sulfate	HMDB0240504	Unclassified	Unclassified	Unclassified			48.2	46.8	M-H	C18H22O7S	0.51536261	23405.65742	23007.41083	22400.90364	987.0911467	1001.68693	1161.611669	13504.58654	13819.37847	14218.96231
5.65_217.1082m/z	217.10824	5.650833333	neg	3-caproyl propionic acid	LMFA01060023	Lipids and lipid-like molecules	Fatty Acyls	Fatty Acids and Conjugates			48.1	43.3	M+FA-H	C9H16O3	0.538904294	2414.153549	2516.775414	3185.830953	3347.651592	2966.697197	2825.671972	2418.134176	2075.248956	2689.064183
1.47_243.9922m/z	243.992213	1.472133333	neg	2-Hydroxy-1,4-benzoxazin-3-one sulfate	HMDB0240439	Organoheterocyclic compounds	Benzoxazines	Benzoxazinones			48	43.9	M-H	C8H7NO6S	0.332762454	53933.02329	63565.72491	59308.61422	5729.598084	5219.421343	5631.019552	73703.84781	59827.08197	67066.61745

3.89_143.0350m/z	143.0349875	3.8906	neg	2-Hydroxyadipic acid	LMFA01170049	Lipids and lipid-like molecules	Fatty Acyls	Fatty acids and conjugates	*	C02360	48	43.2	M-H2O-H	C6H10O5	0.03204772	913.2680261	867.3355216	865.9581171	1188.893794	936.8255109	1064.228759	878.7353313	988.4924317	1035.889078	
3.96_156.0666m/z	156.0666373	3.957583333	neg	2-Aminoheptanedioic acid	HMDB0034252	Organic acids and derivatives	Carboxylic acids and derivatives	Amino acids, peptides, and analogues			48	41.9	M-H2O-H	C7H13NO4	0.117453058	8388.328799	9027.789199	9701.818008	11427.87198	11138.88882	11623.78479	10876.7695	11471.38424	11108.19579	
4.73_193.0354m/z	193.0354394	4.725416667	neg	α-D-Galacturonic acid	3333	Organic oxygen compounds	Organooxygen compounds	Carbohydrates and carbohydrate conjugates	**	C00470	48	44.1	M-H	C6H10O7	0.325509512	5564.892915	6088.111391	6282.837073	5863.050997	6137.379948	6057.279848	6782.425639	6806.192539	7102.264608	
5.41_245.1395m/z	245.1395228	5.406433333	neg	(S)-9-Hydroxy-10-undecenoic acid	HMDB0032662	Organic acids and derivatives	Hydroxy acids and derivatives	Medium-chain hydroxy acids and derivatives	*		48	42.6	M+FA-H	C11H20O3	0.376973438	19711.7987	21518.05982	20954.78842	18651.3963	18803.86046	19902.52639	19956.7708	20072.13217	19652.19438	
2.84_131.0350m/z	131.035019	2.83525	neg	3-hydroxy-3-methyl-2-oxo-butanoic acid	LMFA01020277	Lipids and lipid-like molecules	Fatty Acyls	Fatty Acids and Conjugates	**	C04181	47.9	41.9	M-H	C5H8O4	0.277834912	5239.4285	5160.819032	5491.021623	5690.392016	6247.248551	5850.632264	5256.693028	5785.078973	5432.583969	
3.80_354.0833m/z	354.0833195	3.801716667	neg	DIMBOA-Glc	HMDB0029710	Organic oxygen compounds	Organooxygen compounds	Carbohydrates and carbohydrate conjugates	*		47.9	46	M-H2O-H	C15H19NO10	0.709697088	3665.051991	4310.490527	4496.457997	1380.752587	1072.72803	1240.204213	3355.949808	3344.660221	3378.80337	
4.21_465.1039m/z	465.1038564	4.212116667	neg	(-)-Epicatechin 3'-O-glucuronide	HMDB0041728	Phenylpropanoids and polyketides	Isoflavonoids	O-methylated isoflavonoids	*		47.9	46.1	M-H	C21H22O12	0.01428064	12779.22845	12743.83614	12923.75266	5052.381194	4983.214392	6530.100962	9932.553383	10154.96774	9584.872189	
4.97_241.1084m/z	241.1083935	4.969683333	neg	5S,6S-epoxy-7-undecynoic acid	LMFA01070039	Lipids and lipid-like molecules	Fatty Acyls	Fatty Acids and Conjugates			47.9	45	M+FA-H	C11H16O3	1.255522058	10069.09852	10764.03606	11560.45603	13983.26248	14073.65675	14099.45736	11864.20854	11069.72696	10662.52687	
6.49_255.1240m/z	255.1239887	6.494283333	neg	(R)-8-Acetoxycarvotanacetone	HMDB0037104	Lipids and lipid-like molecules	Prenol lipids	Monoterpenoids	*		47.9	43.7	M+FA-H	C12H18O3	0.910753635	8716.357781	9288.794151	9239.597061	5751.275209	5305.925953	5434.805581	5090.665482	5342.317361	5803.3316	
7.44_369.1739m/z	369.1739049	7.440866667	neg	Androstenediol 3-sulfate	HMDB0248416	Lipids and lipid-like molecules	Steroids and steroid derivatives	Sulfated steroids			47.9	47.6	M-H	C19H30O5S	-0.577276141	29159.73834	28362.21264	32655.75115	39095.84252	38528.00023	38271.99086	31250.39333	33131.31906	30899.93999	
4.77_264.0878m/z	264.0878368	4.769883333	neg	2-(2-Phenylacetoxy)propionylglycine	HMDB0059732	Organic acids and derivatives	Carboxylic acids and derivatives	Amino acids, peptides, and analogues			47.8	41.8	M-H	C13H15NO5	0.341879388	6392.953876	6990.750562	7130.834266	4582.973077	4486.032997	4585.389255	6453.01395	7289.05275	6580.08847	
5.36_189.1133m/z	189.1133343	5.361783333	neg	(3R,5Z)-5-Octene-1,3-diol	LMFA05000556	Lipids and lipid-like molecules	Fatty Acyls	Fatty alcohols	*		47.8	40.8	M+FA-H	C8H16O2	0.70559238	10256.18321	10677.56816	10218.59979	9448.341699	10506.54485	10398.20265	11481.33392	12551.70396	12911.49638	
4.09_212.0930m/z	212.093007	4.091783333	neg	a-Methyldopamine	HMDB0060807	Benzenoids	Benzene and substituted derivatives	Phenethylamines	*		47.7	41.4	M+FA-H	C9H13NO2	1.049841844	1918.868281	2071.371283	1981.457739	1160.370521	957.6349521	1178.40754	1778.840635	1681.977896	1650.454776	
4.86_215.0926m/z	215.092562	4.858383333	neg	Dendryphiellic acid B	LMFA01020399	Lipids and lipid-like molecules	Fatty Acyls	Fatty Acids and Conjugates			47.7	40.3	M+FA-H	C9H14O3	0.381156636	17064.62012	17321.34162	18348.07399	25199.15728	25188.16662	24653.20922	18955.60119	19198.6077	17879.68721	
5.88_318.1380m/z	318.1380464	5.88025	neg	4-hydroxy Nonenal Mercapturic Acid	44812	Unclassified	Unclassified	Unclassified			47.7	48.6	M-H	C14H25NO5S	-0.065937806	15992.85786	19534.18513	19998.92171	15359.62113	13839.2167	15127.39837	23926.10276	25256.42158	27117.72728	
6.39_503.1929m/z	503.1929131	6.386066667	neg	Myricatomentoside I	HMDB0031536	Lignans, neolignans and related compounds	Lignan glycosides	Unclassified	*		47.7	44.2	M-H	C26H32O10	1.273949709	27456.84779	29580.97056	33060.3041	9615.985341	8643.855175	9677.518278	26763.74293	26551.52447	25916.27626	
4.19_242.9969m/z	242.996913	4.189783333	neg	(2E)-3-[3-(sulfooxy)phenyl]prop-2-enoic acid	HMDB0124975	Phenylpropanoids and polyketides	Cinnamic acids and derivatives	Hydroxycinnamic acids and derivatives	*		47.6	42.5	M-H	C9H8O6S	0.124726626	17056.57144	13984.14722	14541.23499	4738.561005	4649.880975	5907.527384	13328.89992	10743.97514	10542.38138	
4.97_255.1241m/z	255.1241143	4.969683333	neg	Isoamyl 2-furonpropionate	HMDB0037735	Lipids and lipid-like molecules	Fatty Acyls	Fatty acid esters	*		47.6	41.2	M+FA-H	C12H18O3	1.508696689	8744.361325	10269.65145	9193.918336	11538.42326	10556.10566	11064.55087	7780.102081	6723.907694	7303.574672	
5.13_445.0781m/z	445.0780671	5.125666667	neg	Genistein 7-O-glucuronide	HMDB0041739	Phenylpropanoids and polyketides	Isoflavonoids	Isoflavonoid O-glycosides	*		47.6	44.1	M-H	C21H18O11	0.968695165	1272.449081	1170.658292	1187.305958	27356.33157	28349.74788	28202.54412	185.7587956	204.6286683	289.4470342	
5.30_241.1084m/z	241.1083701	5.302883333	neg	11-oxo-undeca-5,8-dienoic acid	LMFA01060220	Lipids and lipid-like molecules	Fatty Acyls	Fatty Acids and Conjugates			47.6	41.6	M+FA-H	C11H16O3	1.13650824	15605.56133	14203.76528	14701.39782	23569.73235	27000.92517	26528.6166	16820.94432	15298.76814	16449.22987	
5.41_439.1612m/z	439.1611778	5.406433333	neg	Gibberellin A43	HMDB0035046	Lipids and lipid-like molecules	Prenol lipids	Diterpenoids	*		47.6	40.9	M+FA-H	C20H26O8	0.525477181	41167.56997	48800.36555	45164.96655	65048.1866	58126.33825	59892.65474	53393.78622	57545.71694	41641.20047	
6.15_397.1142m/z	397.1142145	6.154666667	neg	5-(3',4'-Dihydroxyphenyl)-gamma-valerolactone-4'-O-methyl-3'-O-glucuronide	HMDB0059990	Organic oxygen compounds	Organooxygen compounds	Carbohydrates and carbohydrate conjugates	*		47.6	39.6	M-H	C18H22O10	0.487261513	91788.5994	102179.6686	104419.7625	60717.22633	67981.47332	72029.89949	58569.87051	71137.94245	74188.25017	
6.30_455.1904m/z	455.1903872	6.302033333	neg	Pteroside A	HMDB0030758	Organic oxygen compounds	Organooxygen compounds	Carbohydrates and carbohydrate conjugates	*		47.6	47.8	M+FA-H	C21H30O8	-4.59200406	35520.86433	46524.13095	45656.1818	45048.39854	44991.97577	38617.36081	42960.78694	42919.22344	53477.23282	
6.51_411.2027m/z	411.202707	6.509966667	neg	Ingol	LMPR0104310002	Lipids and lipid-like molecules	Prenol lipids	Isoprenoids			47.6	42.2	M+FA-H	C20H30O6	0.724799291	78006.68067	83486.09135	89211.00642	36874.50924	36053.47845	35474.26491	70192.56707	76960.25643	81438.42024	
3.78_503.0813m/z	503.0812515	3.779133333	neg	3,4',5,6-Tetrahydroxy-3',7-dimethoxyflavone 3-glucuronide	HMDB0038262	Phenylpropanoids and polyketides	Flavonoids	Flavonoid glycosides	*		47.5	48.7	M-H2O-H	C23H22O14	-3.567882012	9535.143685	7298.211933	7264.756238	4081.561481	3997.054078	4166.084761	5435.858465	5470.931755	5314.675725	
5.20_227.1291m/z	227.1290992	5.20005	neg	3-Hydroxydodecanedioic acid	HMDB0000413	Organic acids and derivatives	Hydroxy acids and derivatives	Medium-chain hydroxy acids and derivatives	*		47.5	43.2	M-H2O-H	C12H22O5	0.879585732	19501.37063	20587.93567	20704.1436	22554.89292	24904.37828	24350.90217	19791.46479	18633.35947	19563.34449	
5.32_361.1508m/z	361.1507538	5.320233333	neg	Glucosyl (E)-2,6-Dimethyl-2,5-heptadienoate	HMDB0035136	Organic oxygen compounds	Organooxygen compounds	Carbohydrates and carbohydrate conjugates	*		47.5	42.1	M+FA-H	C15H24O7	1.100162696	75381.25945	77734.59291	82818.10728	82967.98274	98994.20357	96336.61276	84140.67248	78080.47348	99260.99425	
9.32_221.1548m/z	221.1547667	9.321783333	neg	(6E,8E)-4,6,8-Megastigmatriene	HMDB0035180	Hydrocarbons	Unsaturated hydrocarbons	Branched unsaturated hydrocarbons	*		47.5	39.4	M+FA-H	C13H20	0.358471811	4558.466744	4524.406359	3237.711028	4334.793473	5597.620452	4684.565834	3634.476903	3584.753879	4786.066624	
9.57_309.1710m/z	309.1709827	9.565266667	neg	12-Oxo-2,3-dinor-10,15-phytodienoic acid	HMDB0032090	Lipids and lipid-like molecules	Fatty Acyls	Eicosanoids	*		47.5	42.4	M+FA-H	C16H24O3	0.890313828	14554.23582	14497.58006	13418.37527	13106.68567	13484.73896	15510.55798	13679.19278	14281.89721	15212.3523	
0.86_267.1100m/z	267.1099563	0.86335	neg	Acetylcarnosine	HMDB0012881	Organic acids and derivatives	Peptidomimetics	Hybrid peptides	*		47.4	42.5	M-H	C11H16N4O4	0.28989598	24837.44592	22585.15832	25734.93687	17931.50423	18197.31393	19387.01119	22501.95574	25593.61151	24385.25013	
2.03_365.1356m/z	365.1355577	2.025516667	neg	Semilepidinoside B	HMDB0033108	Organic oxygen compounds	Organooxygen compounds	Carbohydrates and carbohydrate conjugates			47.4	42.3	M-H	C17H22N2O7	0.363397811	31136.19881	33088.45456	32607.20208	55355.07708	52403.27565	52243.0414	39229.54892	45836.88466	40057.51887	
4.52_239.0927m/z	239.092727	4.521733333	neg	3-methyl-5-carboxypropyl-2-furanpropanoic acid	LMFA01150049	Lipids and lipid-like molecules	Fatty Acyls	Fatty Acids and Conjugates			47.4	43.3	M-H	C12H16O5	0.957360422	5737.789779	6575.38142	7189.013397	6712.360463	6205.970187	5946.067366	7630.59002	7882.84589	9407.165757	
5.85_455.1902m/z	455.1902274	5.84575	neg	Flumethasone	HMDB0252336	Unclassified	Unclassified	Unclassified			47.4	44.2	M+FA-H	C22H28F2O5	3.764217594	140906.6279	149999.5688	160278.5913	128943.9115	128750.6302	128668.7782	195772.7687	184300.0231	193093.1027	
6.54_229.1446m/z	229.1446004	6.539816667	neg	(Z)-3-Methyl-4-decenoic acid	HMDB0031063	Lipids and lipid-like molecules	Fatty Acyls	Fatty acids and conjugates	*		47.4	39	M+FA-H	C11H20O2	0.367145176	20711.14405	21469.81282	22880.7897	17448.34491	17026.20668	17015.47841	20240.92332	21579.76245	20877.55289	
0.74_225.0617m/z	225.0617368	0.7359	neg	D-Glucose	HMDB0000122	Organic oxygen compounds	Organooxygen compounds	Carbohydrates and carbohydrate conjugates	**	C00221	47.3	41.5	M+FA-H	C6H12O6	0.809877278	13665.89826	11333.33705	17916.77074	21469.4153	34292.57536	23942.69103	14995.71352	21763.50541	24527.02443	
3.82_259.1300m/z	259.1299904	3.82415	neg	Glutamylisoleucine	HMDB0028822	Organic acids and derivatives	Carboxylic acids and derivatives	Amino acids, peptides, and analogues	*		47.3	39.1	M-H	C11H20N2O5	0.173214199	13449.90553	14623.94728	15592.93977	19248.655	18231.06899	18881.0516	17481.1186	19234.33353	19424.39557	
6.94_294.1350m/z	294.134988	6.939266667	neg	2-Pentanamido-3-phenylpropanoic acid	HMDB0094646	Organic acids and derivatives	Carboxylic acids and derivatives	Amino acids, peptides, and analogues			47.3	41.2	M+FA-H	C14H19NO3	1.170678997	4798.911727	5648.974805	4692.648778	9822.332712	9310.833682	8940.67064	5451.927972	6964.091849	7261.171625	
7.42_226.1451m/z	226.145059	7.41815	neg	N-OCTANOYL-L-HOMOSERINE LACTONE	HMDB0255213	Unclassified	Unclassified	Unclassified			47.3	41.2	M-H	C12H21NO3	0.844987596	6188.387498	6151.767493	6983.713377	11127.00777	10344.44434	10181.54671	6283.598038	6475.570206	6538.328717	
1.34_147.0300m/z	147.0300215	1.335933333	neg	3-hydroxybut-2-enoic acid	LMFA01030978	Lipids and lipid-like molecules	Fatty Acyls	Fatty Acids and Conjugates			47.2	38	M+FA-H	C4H6O3	1.220659295	10789.39765	10702.38188	11347.53776	10388.17267	9476.249816	9560.190181	8672.911078	9276.398547	9020.465617	
3.02_167.0462m/z	167.0462455	3.02	neg	2-Pyrroloylglycine	HMDB0059778	Organic acids and derivatives	Carboxylic acids and derivatives	Amino acids, peptides, and analogues			47.2	40.4	M-H	C7H8N2O3	0.17748317	3085.905172	2993.170867	3345.615819	4740.900531	5146.147711	4770.402259	3210.778993	3363.875681	3295.046488	
5.76_533.1668m/z	533.1667916	5.7632	neg	3,4-Dihydroxychalcone 4-beta-L-arabinopyranosyl-(1->4)-galactoside	LMPK12120196	Unclassified	Unclassified	Unclassified			47.2	39.9	M-H	C26H30O12	0.639532767	27316.15183	30740.21782	26653.78586	645.8549749	804.8488169	815.9495009	2047.006842	2266.807114	1845.166563	
7.16_429.1768m/z	429.1768201	7.1647	neg	Artesunate	HMDB0240267	Lipids and lipid-like molecules	Prenol lipids	Sesquiterpenoids			47.2	40.5	M+FA-H	C19H28O8	0.51890564	49730.53158	58084.30077	55404.72263	59394.76111	58580.16745	56973.13221	45316.1394	57325.23398	50903.75162	
0.86_105.0193m/z	105.019301	0.86335	neg	Glyceric acid	280	Organic oxygen compounds	Organooxygen compounds	Carbohydrates and carbohydrate conjugates	**	C00258	47.1	40.2	M-H	C3H6O4	-0.294659726	686.3767301	740.1429561	847.034335	938.349915	711.4630375	1086.125706	713.8576656	1031.606349	681.7141745	
0.97_111.0088m/z	111.0087749	0.967616667	neg	2-Furoic acid	HMDB0000617	Organoheterocyclic compounds	Furans	Furoic acid and derivatives	**	C01546	47.1	38.1	M-H	C5H4O3	0.065287527	2436.803175	3098.439766	3662.577996	6562.542862	6182.217735	5893.237878	3428.810797	4195.989509	4354.430399	
1.36_118.0267n	117.0193835	1.358266667	neg	Succinic acid	HMDB0000254	Organic acids and derivatives	Carboxylic acids and derivatives	Dicarboxylic acids and derivatives	***	C00042	47.1	38.4	M-H, M+FA-H	C4H6O4	0.434050456	1431.069321	1727.730703	1752.681138	1647.662799	2001.270391	3039.869501	1453.40238	1577.052281	1634.144318	
4.63_196.0616m/z	196.0616454	4.632666667	neg	3alpha,4,7,7alpha-Tetrahydro-1H-isoindole-1,3(2H)-dione	HMDB0033903	Organoheterocyclic compounds	Isoindoles and derivatives	Isoindolines			47.1	37.3	M+FA-H	C8H9NO2	0.75485711	3590.35846	3229.115328	3008.332121	490.3707508	406.4872224	540.8673275	855.3590603	790.5284447	700.0072934	
5.18_197.0821m/z	197.0820548	5.1777	neg	3,4-Methylene suberic acid	HMDB0059768	Lipids and lipid-like molecules	Fatty Acyls	Fatty acids and conjugates	*		47.1	38.8	M-H	C10H14O4	0.617593148	11554.37594	11991.41569	12317.91864	23418.87158	25813.49939	25421.32565	12753.06683	12301.45774	13673.81609	
5.88_273.1709m/z	273.1709249	5.88025	neg	10-keto tridecanoic acid	LMFA01060042	Lipids and lipid-like molecules	Fatty Acyls	Fatty Acids and Conjugates			47.1	38.5	M+FA-H	C13H24O3	0.777490416	16639.40923	19824.35587	19045.97578	14932.3477	14418.71124	14365.43305	9417.034259	9934.057724	11950.35591	
0.90_173.0092m/z	173.0091686	0.904683333	neg	5-Hydroxy-2-furoic acid	HMDB0059784	Organoheterocyclic compounds	Furans	Furoic acid and derivatives			47	35.8	M+FA-H	C5H4O4	0.055726043	18923.69378	23413.4407	25898.39265	35452.89126	36824.85412	35187.46768	20160.98464	23114.9589	23686.71358	
1.59_230.1148m/z	230.1147892	1.5934	neg	Asparaginyl-Valine	HMDB0028744	Organic acids and derivatives	Carboxylic acids and derivatives	Amino acids, peptides, and analogues	*		47	40.1	M-H	C9H17N3O4	0.690488341	3256.449854	3112.192077	3445.466007	1723.522907	1745.939934	1747.043517	2387.643197	2580.233539	2488.05427	
1.92_204.9813m/z	204.9813008	1.924916667	neg	Pyrogallol-2-O-sulphate	HMDB0060018	Organic acids and derivatives	Organic sulfuric acids and derivatives	Arylsulfates	*		47	41.2	M-H	C6H6O6S	0.33145368	122355.9562	129218.9549	101133.0985	22998.25004	23691.42378	23097.66515	101065.988	109311.4273	99440.21906	
4.86_202.1087m/z	202.1086756	4.858383333	neg	(S)-Homostachydrine	HMDB0033433	Organic acids and derivatives	Carboxylic acids and derivatives	Amino acids, peptides, and analogues		C08283	47	37.2	M+FA-H	C8H15NO2	1.234733714	11792.26465	12546.57444	13290.98356	10661.38472	10182.94099	10299.46634	13000.18237	13907.64982	13402.64263	
4.88_311.0775m/z	311.0775349	4.880083333	neg	Hydroxytyrosol 3'-glucuronide	HMDB0240529	Organic oxygen compounds	Organooxygen compounds	Carbohydrates and carbohydrate conjugates			47	41.8	M-H2O-H	C14H18O9	0.890304594	23018.70678	26216.13696	25505.0238	1769.354344	1622.492475	1299.231417	40818.99932	44027.22859	42825.13374	
7.14_183.1027m/z	183.1027054	7.138633333	neg	exo-2-Methyl-3-methylenebicyclo[2.2.1]heptan-2-ol	HMDB0036092	Lipids and lipid-like molecules	Prenol lipids	Monoterpenoids	*		47	36.2	M+FA-H	C9H14O	0.271256522	6509.100754	6955.153447	6859.804721	8429.870859	9448.750779	7851.990808	6467.862558	7468.951045	7248.272799	
3.54_277.0024m/z	277.0023942	3.540433333	neg	3,5-dimethoxy-4-(sulfooxy)benzoic acid	HMDB0131461	Benzenoids	Benzene and substituted derivatives	Benzoic acids and derivatives	*		46.9	44.2	M-H	C9H10O8S	0.116476004	7448.467775	8004.09966	8076.234041	4100.121154	4499.054829	4418.956169	4388.564604	4823.516391	4741.916367	
4.00_495.1149m/z	495.1148565	4.002233333	neg	4'-Methyl-(-)-epigallocatechin 7-glucuronide	HMDB0041676	Phenylpropanoids and polyketides	Flavonoids	Flavonoid glycosides	*		46.9	41.8	M-H	C22H24O13	0.891125175	17267.19556	17846.44855	19680.29056	14344.63556	13105.39269	12325.7069	16990.71662	19604.43681	17130.14661	
5.36_217.0177m/z	217.0176717	5.361783333	neg	Tyrosol 4-sulfate	HMDB0041785	Organic acids and derivatives	Organic sulfuric acids and derivatives	Arylsulfates	*		46.9	43.4	M-H	C8H10O5S	0.246556476	5744.073538	6832.578856	6506.190354	1015.438095	1050.654934	922.8460553	2496.496231	3224.029998	2800.300271	
6.68_547.1462m/z	547.1461782	6.680966667	neg	3,4,5-trihydroxy-6-{3-[(E)-2-(4-hydroxyphenyl)ethenyl]-5-{[3,4,5-trihydroxy-6-(hyc	HMDB0128508	Phenylpropanoids and polyketides	Stilbenes	Stilbene glycosides	*		46.9	37.8	M-H2O-H	C26H30O14	0.818984842	57089.37848	67391.315	65767.81545	64386.73894	63819.85447	55964.44412	39053.0013	51175.8505	51315.96286	
0.78_196.0583n	195.0510405	0.778533333	neg	Gluconic acid	345	Organic oxygen compounds	Organooxygen compounds	Carbohydrates and carbohydrate conjugates	**	C00257	46.8	34.9	M-H, 2M-H	C6H12O7	0.072259301	449811.2203	492793.2542	542748.7004	385237.9616	417033.7784	428493.8087	436250.4364	508817.2419	515412.8915	
0.78_632.2047m/z	632.2046643	0.778533333	neg	3'-Sialyllactose	3628	Organic oxygen compounds	Organooxygen compounds	Carbohydrates and carbohydrate conjugates	*		46.8	42.2	M-H	C23H39NO19	0.493831058	73716.66886	69574.1122	72843.95172	68777.03958	72664.58306	63686.27515	70981.80533	76920.49504	70333.89451	
1.14_173.0932m/z	173.093187	1.1382	neg	L-Theanine	HMDB0034365	Organic acids and derivatives	Carboxylic acids and derivatives	Amino acids, peptides, and analogues		C01047	46.8	39.3	M-H	C7H14N2O3	0.121487289	3161.643314	2748.325962	2499.606631	1553.01559	1259.304064	1288.013401	1297.632853	1831.128157	1774.660304	
4.48_165.0194m/z	165.0193629	4.477166667	neg	Phthalic acid	HMDB0002107	Benzenoids	Benzene and substituted derivatives	Benzoic acids and derivatives	**	C01606	46.8	36.4	M-H	C8H6O4	0.184443232	14223.85047	13826.34285	14027.22277	15387.38987	15493.24013	15276.60329	16420.6105	14955.40878	16473.2148	
0.80_136.0372n	135.029916	0.800366667	neg	Threonic acid	HMDB0000943	Organic oxygen compounds	Organooxygen compounds	Carbohydrates and carbohydrate conjugates	**	C01620	46.7	34	M-H, 2M-H	C4H8O5	0.140057002	324333.6278	315767.8019	361740.6903	256611.6347	253287.372	253679.1313	314383.6887	335192.9687	334744.3652	
4.88_367.2240m/z	367.2240395	4.880083333	neg	Perindopril	HMDB0014928	Organic acids and derivatives	Carboxylic acids and derivatives	Amino acids, peptides, and analogues		C07706	46.7	38.7	M-H	C19H32N2O5	0.526247535	13260.64922	15406.18215	16741.92687	11075.58355	10644.79828	11524.6007	9186.712134	11265.21183	10681.41399	
5.55_204.1362n	203.1289034	5.547916667	neg	(1alpha,2alpha,4betaH,6alpha,8R)-p-Menthane-2,6,8,9-tetrol	HMDB0039053	Lipids and lipid-like molecules	Prenol lipids	Monoterpenoids	*		46.7	35.8	M-H2O-H, M-H	C10H20O4	0.301695025	46409.59369	42661.33797	45739.95014	38041.73157	49886.68051	39553.5755	39216.78096	40828.48258	37458.49824	
5.97_213.0229m/z	213.0228623	5.965483333	neg	4-Allylphenol sulfate	HMDB0170765	Organic acids and derivatives	Organic sulfuric acids and derivatives	Arylsulfates			46.7	40	M-H	C9H10O4S	0.742470273	14100.63783	15544.51431	17110.35754	11485.65083	13451.20862	13056.32337	16578.16144	18147.43218	16823.54427	
6.63_545.1305m/z	545.1305384	6.63275	neg	Afrormosin 7-O-(6''-malonylglucoside)	LMPK12050099	Lipids and lipid-like molecules	Polyketides	Flavonoids			46.7	41.9	M-H	C26H26O13	0.867893053	65406.52647	69712.62824	64054.29081	45305.38256	43011.33933	42999.99868	996.3636868	1706.55353	1453.032065	
7.16_331.1765m/z	331.1764894	7.1647	neg	Neomenthol-glucuronide	HMDB0060012	Lipids and lipid-like molecules	Prenol lipids	Terpene glycosides	*		46.7	37.9	M-H	C16H28O7	0.790601971	34040.04248	38881.52821	38102.33572	46289.42904	45584.70169	45221.54392	39394.59365	51363.16966	45207.5926	
4.32_257.1031m/z	257.1030672	4.3224	neg	3,4-Methyleneazelaic acid	HMDB0059744	Lipids and lipid-like molecules	Fatty Acyls	Fatty acids and conjugates	*		46.6	37.8	M+FA-H	C11H16O4	0.025335344	4535.360259	4967.716712	5094.006807	6543.708616	5856.162566	5545.315423	5252.75846	5610.161655	5311.322958	
5.34_389.0915m/z	389.0914577	5.3394	neg	4-Methoxybenzenepropanol 1-(2-sulfoglucoside)	HMDB0034757	Lipids and lipid-like molecules	Fatty Acyls	Fatty acyl glycosides	*		46.6	40.7	M-H2O-H	C16H24O10S	0.688139087	60092.17094	68621.02966	72079.41221	60994.31238	65361.93964	57494.69718	53674.43375	61381.68242	60872.94824	
8.09_457.1873m/z	457.1872562	8.087016667	neg	2-Methoxyestrone 3-glucuronide	HMDB0004482	Lipids and lipid-like molecules	Steroids and steroid derivatives	Steroidal glycosides	**	C11132	46.6	38.4	M-H2O-H	C25H32O9	0.975793212	42777.00398	49752.64168	44209.55718	19194.5473	16646.54544	17246.3821	25867.82106	32216.19793	30328.96156	
4.90_281.1144m/z	281.1144171	4.902316667	neg	Alanylphenylalanine	HMDB0028694	Organic acids and derivatives	Carboxylic acids and derivatives	Amino acids, peptides, and analogues	*		46.5	53.2	M+FA-H	C12H16N2O3	0.516005318	18913.51331	20776.11066	21813.33873	23816.70203	24403.27039	24652.85463	19407.5058	22207.53732	20312.96002	
5.06_363.0704m/z	363.0703736	5.05975	neg	Xanthotoxol glucoside	HMDB0038626	Phenylpropanoids and polyketides	Coumarins and derivatives	Coumarin glycosides			46.5	41.4	M-H	C17H16O9	-4.894562559	7302.238115	7443.474103	7766.778695	1621.488047	1538.031182	1557.276806	9480.041953	9592.412762	12220.38694	
6.30_533.1303m/z	533.1302725	6.302033333	neg	Acacetin 7-(6''-acetylglucoside)	LMPK12110457	Lipids and lipid-like molecules	Polyketides	Flavonoids			46.5	34.7	M+FA-H	C24H24O11	0.426301114	79523.38356	86092.54363	96663.97038	73208.9	77639.56511	72776.72594	72933.74043	78222.16472	81051.22661	
10.46_449.2548m/z	449.2547927	10.45966667	neg	3alpha-androstanediol glucuronide	LMST05010004	Lipids and lipid-like molecules	Sterol Lipids	Steroid conjugates	**	C03033	46.4	34.5	M-H2O-H	C25H40O8	0.673989739	177709.8335	185593.7308	213437.4219	194750.8754	207032.1932	184472.2161	189169.382	206463.6902	197166.8755	
11.44_165.0409m/z	165.0408772	11.44468333	neg	2,3-Dihydroxybutanoic acid	HMDB0245394	Organic oxygen compounds	Organooxygen compounds	Carbohydrates and carbohydrate conjugates	*		46.4	37.8	M+FA-H	C4H8O4	3.461935207	2890.833699	2743.530235	2257.726703	2588.573557	2680.138072	2918.438935	3020.994023	2681.553785	2500.204874	
4.05_338.0885m/z	338.0884992	4.046666667	neg	6-Hydroxy-5-methoxyindole glucuronide	HMDB0010362	Organic oxygen compounds	Organooxygen compounds	Carbohydrates and carbohydrate conjugates	**	C03033	46.4	35.9	M-H	C15H17NO8	1.058959755	12123.8675	13612.21767	14092.00025	15287.49098	14650.91227	14384.74313	12773.63019	15149.66331	14078.81915	
4.39_261.0881m/z	261.0881157	4.3893	neg	Phenylalanylaspartic acid	HMDB0028991	Organic acids and derivatives	Carboxylic acids and derivatives	Amino acids, peptides, and analogues	*		46.4	33.1	M-H2O-H	C13H16N2O5	0.125575567	27796.6176	29712.25706	28825.51361	29563.28234	23883.02496	25319.34874	37087.15884	43147.22125	40350.87401	
4.73_171.0664m/z	171.0663784	4.725416667	neg	xi-4-Hydroxy-4-methyl-2-cyclohexen-1-one	HMDB0033629	Organic oxygen compounds	Organooxygen compounds	Carbonyl compounds			46.4	34.2	M+FA-H	C7H10O2	0.761629722	7567.075685	6733.178181	7644.318146	9977.861835	10546.0831	11231.37032	7823.352192	8055.804226	6815.627589	
4.73_187.1209n	186.1136447	4.725416667	neg	7-Keto-8-aminopelargonic acid	LMFA01100065	Lipids and lipid-like molecules	Fatty Acyls	Fatty acids and conjugates	**	C01092	46.4	42.8	M-H, M+FA-H	C9H17NO3	0.415378555	156547.6316	146269.5417	144734.0814	152036.0788	164490.0515	164957.3389	179333.3585	154209.97	162337.2187	
5.32_247.1552m/z	247.1551963	5.320233333	neg	omega-hydroxy hendecanoic acid	LMFA01050035	Lipids and lipid-like molecules	Fatty Acyls	Fatty Acids and Conjugates			46.4	33.8	M+FA-H	C11H22O3	0.489069854	27244.52499	27914.44725	31545.3874	29329.13573	33070.61298	31177.33814	19429.58956	18904.75978	22183.13837	
6.63_319.1400m/z	319.1399845	6.63275	neg	Valproic acid glucuronide	HMDB0000901	Organic oxygen compounds	Organooxygen compounds	Carbohydrates and carbohydrate conjugates	*		46.4	36.6	M-H	C14H24O8	0.447312973	16417.2653	17473.77035	16041.0136	8195.089068	8017.823363	7811.542644	19523.82477	22702.9125	22803.31917	
7.11_227.1291m/z	227.1290936	7.107783333	neg	9-undecynoic acid	LMFA01030617	Lipids and lipid-like molecules	Fatty Acyls	Fatty Acids and Conjugates			46.4	35.9	M+FA-H	C11H18O2	1.15825988	8206.399396	7831.627628	8212.472073	10382.39722	11663.33465	10106.02555	5986.686259	6570.368002	6313.154988	
0.84_191.0562m/z	191.0562207	0.841733333	neg	L-galacto-2-Heptulose	HMDB0029901	Organic oxygen compounds	Organooxygen compounds	Carbohydrates and carbohydrate conjugates	*		46.3	35.9	M-H2O-H	C7H14O7	0.518952694	14316.73883	18561.84515	21902.75348	22271.05034	14963.45022	19625.41275	22920.95395	13985.53398	17471.16152	
1.31_285.0619m/z	285.0618745	1.3134	neg	Diphenol glucuronide	HMDB0059998	Organic oxygen compounds	Organooxygen compounds	Carbohydrates and carbohydrate conjugates	*		46.3	34.8	M-H	C12H14O8	0.991276488	10368.98415	11883.35128	11292.61855	5254.966606	5308.595407	5494.881878	7702.348771	9017.727492	8828.042038	
3.48_310.1159m/z	310.1159121	3.484883333	neg	1,7-Dimethylguanosine	HMDB0001961	Nucleosides, nucleotides, and analogues	Purine nucleosides	Unclassified	*		46.3	36	M-H	C12H17N5O5	0.706778581	38182.41865	46382.16962	44722.41395	56576.82963	49331.80705	50788.25643	47553.14135	46908.62633	47013.48863	
4.21_239.0562m/z	239.0561518	4.212116667	neg	(-)-trans-3,4-Dihydro-4,8-dihydroxy-3-methyl-1H-2-benzopyran-1-one	HMDB0030765	Organoheterocyclic compounds	Benzopyrans	2-benzopyrans	*		46.3	34.3	M+FA-H	C10H10O4	0.206912176	4545.310198	4460.068662	4587.324521	4384.124042	4347.978864	4677.624121	2405.206399	2265.085838	2425.001783	
5.13_245.0933m/z	245.0933437	5.125666667	neg	N-Acetyl-DL-tryptophan	34535	Unclassified	Unclassified	Unclassified			46.3	36.8	M-H	C13H14N2O3	0.722705404	2810.196147	2705.936305	2347.998667	2328.902692	2592.973175	2397.009876	3025.686754	2709.786214	3306.491695	
5.18_507.1486m/z	507.1486234	5.1777	neg	Andrographidin A	LMPK12140652	Lipids and lipid-like molecules	Polyketides	Flavonoids			46.3	40.7	M+FA-H	C23H26O10	-4.709374772	26210.14961	28448.7699	27606.52916	29063.13572	28577.7699	27782.39081	27174.0211	26998.50057	29089.80412	
5.18_575.1362m/z	575.1362366	5.1777	neg	Kuwanon Z	HMDB0033297	Phenylpropanoids and polyketides	Flavonoids	Flavans			46.3	51.8	M-H2O-H	C34H26O10	2.492163937	10126.44097	11574.61393	10025.96711	11640.42446	10559.6358	10896.85776	9748.246377	11425.09675	9982.370536	
5.88_270.1350m/z	270.1349918	5.88025	neg	Prenalterol	HMDB0256762	Unclassified	Unclassified	Unclassified			46.3	38.4	M+FA-H	C12H19NO3	1.312135392	3796.034248	4973.379461	4935.112012	6274.097314	5925.990096	6311.235256	4990.588289	5128.273464	6072.900726	
2.00_215.1038m/z	215.1037666	2.004766667	neg	N2,N5-Diacetylornithine	HMDB0240345	Organic acids and derivatives	Carboxylic acids and derivatives	Amino acids, peptides, and analogues			46.2	42	M-H	C9H16N2O4	0.166799724	126579.5464	121842.9539	127288.131	83126.43444	74226.70166	77188.28564	84577.58191	103126.8501	88273.43263	
2.36_216.9806m/z	216.9805922	2.36465	neg	3-hydroxybenzoic acid-3-O-sulphate	HMDB0059968	Organic acids and derivatives	Organic sulfuric acids and derivatives	Arylsulfates	*		46.2	47.2	M-H	C7H6O6S	-2.937251987	4910.996792	4676.21791	4810.446968	6922.013731	6854.721765	5971.126624	5210.991788	6274.578182	6405.526076	
4.75_230.1035m/z	230.1034617	4.747466667	neg	5-Hepteneoylglycine	HMDB0094731	Organic acids and derivatives	Carboxylic acids and derivatives	Amino acids, peptides, and analogues			46.2	34.9	M+FA-H	C9H15NO3	0.353734803	1802.670584	2065.45824	2080.754976	3680.826074	3157.920503	3463.24417	2803.383969	3374.390342	2413.551961	
7.61_169.0871m/z	169.0871118	7.6091	neg	2,3-Dimethyl-2-cyclohexen-1-one	HMDB0031414	Organic oxygen compounds	Organooxygen compounds	Carbonyl compounds	*		46.2	33.9	M+FA-H	C8H12O	0.756913884	4892.122877	5132.690885	4828.775114	5110.701757	4913.017474	5570.681915	5260.354663	5177.833598	5335.026385	
4.16_186.0561m/z	186.0561315	4.15895	neg	3-amino-2-naphthoic acid	34509	Unclassified	Unclassified	Unclassified			46.1	42.9	M-H	C11H9NO2	0.424708807	458.4126999	415.946551	408.2678632	1563.467963	995.4678197	1177.929276	788.238516	592.0971598	659.5237798	
5.95_199.0977m/z	199.0976988	5.95155	neg	2-Nonenoic acid gamma-lactone	HMDB0032442	Organoheterocyclic compounds	Dihydrofurans	Furanones	*		46.1	32.3	M+FA-H	C9H14O2	0.754551839	42855.9391	46739.55247	51355.06164	35260.76578	42100.88383	41244.07452	40636.51748	47614.36579	44866.61137	
0.86_151.0071m/z	151.0070612	0.86335	neg	2-Mercaptopropanoic acid	HMDB0031520	Organic acids and derivatives	Carboxylic acids and derivatives	Carboxylic acids	*		46	33.1	M+FA-H	C3H6O2S	0.074648182	8615.250111	9911.45233	10595.64816	13921.06915	12772.46511	13618.56445	19459.5656	20134.40689	19168.38614	
2.20_285.0618m/z	285.0617824	2.203283333	neg	Quinol glucuronide	HMDB0259508	Unclassified	Unclassified	Unclassified			46	32	M-H	C12H14O8	0.669222055	10709.21303	10240.33146	10964.59592	1595.167741	1435.897988	1779.482148	28070.19069	30570.4397	27190.66413	
3.80_175.0976m/z	175.0976398	3.801716667	neg	6,8-dihydroxy-octanoic acid	LMFA01050228	Lipids and lipid-like molecules	Fatty Acyls	Fatty Acids and Conjugates			46	32.6	M-H	C8H16O4	0.325047509	3220.259508	3528.414202	3352.716142	6455.948416	5743.664991	5651.665274	3345.027105	3388.64957	3505.446895	
3.96_179.0575n	224.0556717	3.957583333	neg	Adrenochrome	HMDB0012884	Organoheterocyclic compounds	Indoles and derivatives	Unclassified	*		46	43.3	M+FA-H, 2M-H	C9H9NO3	-4.324595423	12772.95767	13513.54272	13671.24942	10714.53893	10053.74781	10052.2502	11609.28733	12626.46161	12047.12315	
4.18_369.0289m/z	369.0288689	4.181333333	neg	Catechin 7-sulfate	HMDB0240436	Unclassified	Unclassified	Unclassified			46	39.3	M-H	C15H14O9S	0.78988446	14255.89047	12459.7123	13850.22377	5097.018434	4536.255929	4983.800087	8367.577017	6753.945335	7235.855483	
4.32_233.1031m/z	233.1031226	4.3224	neg	2,4-Dimethylpimelic acid	HMDB0059760	Lipids and lipid-like molecules	Fatty Acyls	Fatty acids and conjugates	*		46	32.4	M+FA-H	C9H16O4	0.322861111	3212.581741	3372.504143	3296.8332	3983.299689	3781.047113	3935.196934	2633.442991	2842.922615	2979.528357	
5.61_225.0770m/z	225.0770273	5.610733333	neg	10-hydroxy-(2E,8E)-decadien-4-ynoic Acid	HMDB0062773	Lipids and lipid-like molecules	Fatty Acyls	Fatty acids and conjugates	*		46	32.8	M+FA-H	C10H12O3	1.000720664	7934.71354	8063.51704	9016.021571	8906.383964	8352.390647	7961.689935	9074.129321	8440.554512	7985.503316	
2.59_343.0672m/z	343.0672238	2.58585	neg	Benzoyl glucuronide (Benzoic acid)	HMDB0010324	Organic oxygen compounds	Organooxygen compounds	Carbohydrates and carbohydrate conjugates	*		45.9	31.9	M+FA-H	C13H14O8	0.515008064	55725.84026	58454.36355	65406.40572	10607.40885	10104.61199	9933.646579	42592.3082	46343.46428	47858.65995	
3.96_143.0714m/z	143.0714109	3.957583333	neg	5-hexenal	LMFA06000005	Lipids and lipid-like molecules	Fatty Acyls	Fatty aldehydes			45.9	30.4	M+FA-H	C6H10O	0.439500883	1629.955723	1773.544817	1817.400682	2822.842699	2882.167401	3023.54518	2010.512255	2037.254254	2079.053449	
4.16_138.0561m/z	138.0560936	4.15895	neg	Paramethadione	HMDB0014755	Organoheterocyclic compounds	Azolidines	Oxazolidines		C07411	45.9	31.9	M-H2O-H	C7H11NO3	0.264446326	2707.322151	2673.178276	2681.504505	3886.050899	2664.758035	2819.510638	3603.124584	2704.392975	2879.845836	
4.30_188.0929m/z	188.0929147	4.300033333	neg	1-Aminocyclohexanecarboxylic acid	HMDB0243822	Lipids and lipid-like molecules	Prenol lipids	Monoterpenoids	*		45.9	31.3	M+FA-H	C7H13NO2	0.5813035	10004.90374	11070.07809	10628.55154	8578.843343	8407.473265	8207.31398	10589.35562	11763.53354	11305.42962	
5.36_345.1556m/z	345.1556421	5.361783333	neg	(1R,3S,4S,6R)-6,9-Dihydroxyfenchone 6-O-b-D-glucoside	HMDB0033222	Lipids and lipid-like molecules	Prenol lipids	Terpene glycosides	*		45.9	33.2	M-H	C16H26O8	0.435583056	311597.9838	325943.6238	333017.8757	121351.0357	138791.8237	132462.9006	315444.2101	304962.7212	337734.9027	
0.78_230.0192n	229.0118816	0.778533333	neg	D-Ribulose 5-phosphate	HMDB0000618	Organic oxygen compounds	Organooxygen compounds	Carbohydrates and carbohydrate conjugates	**	C00199	45.8	36.5	M-H2O-H, M-H	C5H11O8P	0.383176989	12005.73975	12931.54547	12957.59521	15741.67063	14935.37985	15077.4256	13026.31106	13773.35138	11613.25388	

3.73_230.9970m/z	230.9969863	3.730516667	neg	2-[3-(sulfooxy)phenyl]acetic acid	HMDB0125163	Organic acids and derivatives	Organic sulfuric acids and derivatives	Arylsulfates	*		45.8	35.8	M-H	C8H8O6S	0.447414103	10310.16293	10447.38737	10480.83683	14551.17894	15005.91416	14278.00866	7992.768038	9314.650403	9314.540566
4.02_231.0204n	230.0129912	4.024283333	neg	HPAA sulfate	HMDB0240381	Organic acids and derivatives	Organic sulfuric acids and derivatives	Arylsulfates			45.8	36.8	M-H2O-H, M-H	C8H9NO5S	1.090326662	4545.413293	5079.880412	5230.745783	1509.617052	1513.207355	1568.281122	2302.821334	3002.59183	2538.441504
6.37_325.1296m/z	325.1295776	6.373833333	neg	8-Deoxy-11,13-dihydroxygrosheimin	HMDB0041144	Lipids and lipid-like molecules	Prenol lipids	Terpene lactones	*		45.8	32.9	M+FA-H	C15H20O5	1.074290459	18152.59421	20149.06241	21960.67641	8477.547076	9481.674384	8784.650062	18703.13921	20249.8852	19502.49098
1.63_167.0351m/z	167.0350642	1.631516667	neg	Dihydroxyphenylacetic acid	968	Benzenoids	Phenols	Benzenediols	**	C01161	45.7	30.9	M-H	C8H8O4	0.487299796	8343.41822	9098.726327	10980.90393	10385.94921	10260.08096	10410.61201	12715.14581	10649.07845	11358.0534
2.48_115.0401m/z	115.0400633	2.479216667	neg	3-keto valeric acid	LMFA01060005	Lipids and lipid-like molecules	Fatty Acyls	Fatty Acids and Conjugates			45.7	31.1	M-H	C5H8O3	-0.038141439	5357.669134	5398.923632	5776.928111	7169.127864	6696.014647	6606.873624	6026.580986	6086.945815	6032.433101
4.21_216.9813m/z	216.9813114	4.212116667	neg	4-Methylcatechol sulfate	HMDB0242166	Organic acids and derivatives	Organic sulfuric acids and derivatives	Arylsulfates			45.7	42.3	M-H	C7H6O6S	0.362131793	6421.773308	6692.613336	6718.885577	7891.109512	7593.175143	7750.668984	2294.807159	2554.297763	2672.683028
4.43_377.1455m/z	377.1455224	4.432683333	neg	Tryptophyl-Gamma-glutamate	HMDB0029097	Organic acids and derivatives	Carboxylic acids and derivatives	Amino acids, peptides, and analogues	*		45.7	37.7	M+FA-H	C16H20N4O4	-3.41897227	84126.55214	89331.29376	87061.90745	103688.4985	94551.86631	94101.863	92124.75847	102680.8427	96745.12981
4.54_383.0446m/z	383.0445885	4.54425	neg	4'-Methylepicatechin 5-sulfate	HMDB0240468	Phenylpropanoids and polyketides	Flavonoids	Flavans			45.7	36.9	M-H	C16H16O9S	0.942097504	45642.0264	49019.06699	50566.43823	28457.42989	26872.33115	26063.91967	35935.13143	36695.58825	38992.6492
5.01_357.0831m/z	357.0831077	5.014716667	neg	Furaneol 4-(6-malonylglucoside)	HMDB0029778	Organic oxygen compounds	Organooxygen compounds	Carbohydrates and carbohydrate conjugates	*		45.7	33.3	M-H2O-H	C15H20O11	1.029859525	5684.574677	5850.1668	6220.512347	1252.262364	1257.922778	1228.797778	5895.293673	4697.877498	4612.334911
5.10_325.0931m/z	325.0930995	5.1031	neg	Homovanillyl alcohol glucuronide	HMDB0240527	Organic oxygen compounds	Organooxygen compounds	Carbohydrates and carbohydrate conjugates			45.7	32.6	M-H2O-H	C15H20O9	0.605584323	9585.460342	9146.320334	8678.999269	6533.291506	7986.408546	8750.657204	16027.78761	12500.66557	14733.88025
1.20_129.0194m/z	129.019427	1.202333333	neg	Gamma-delta-Dioxovaleric acid	HMDB0013233	Organic acids and derivatives	Keto acids and derivatives	Gamma-keto acids and derivatives	**	C02800	45.6	30.3	M-H	C5H6O4	0.7287604	27380.71151	28390.8314	31017.35875	29009.91194	27012.57425	27622.62595	26043.14721	30718.30297	30175.83765
4.05_305.0339m/z	305.0338599	4.046666667	neg	4-Hydroxy-5-(3',4'-dihydroxyphenyl)-valeric acid 4'-sulfate	HMDB0240454	Unclassified	Unclassified	Unclassified			45.6	34.5	M-H	C11H14O8S	0.646885508	12959.4425	14485.23094	14715.39053	9408.367344	8532.629712	8486.474747	22841.1738	25705.29688	22363.84534
4.26_258.9918m/z	258.99179	4.2568	neg	Caffeic acid 3-O-sulfate	HMDB0041706	Phenylpropanoids and polyketides	Cinnamic acids and derivatives	Hydroxycinnamic acids and derivatives	*		45.6	32.8	M-H	C9H8O7S	-0.027513715	65640.35072	68965.32022	68936.0483	20630.19938	19095.36433	17853.51376	54560.07304	59503.54039	54279.10041
4.68_203.0021m/z	203.0021206	4.682483333	neg	4-Methylcatechol 2-sulfate	HMDB0240461	Organic acids and derivatives	Organic sulfuric acids and derivatives	Arylsulfates			45.6	34.4	M-H	C7H8O5S	0.748543762	7304.85178	7787.900399	8767.414162	3264.827411	2898.963305	3079.042662	3100.712861	3476.835282	3146.314662
4.68_431.0987m/z	431.0987066	4.682483333	neg	Phloretin 2'-O-glucuronide	HMDB0041768	Phenylpropanoids and polyketides	Flavonoids	Flavonoid glycosides	*		45.6	33.5	M-H2O-H	C21H22O11	0.746900455	6604.70515	6887.917838	7162.533609	58.53966002	0.001403199	192.3141794	224.4126582	65.88692692	125.5320278
4.99_184.0980m/z	184.0979923	4.99225	neg	2-Hepteneoylglycine	HMDB0094728	Organic acids and derivatives	Carboxylic acids and derivatives	Amino acids, peptides, and analogues			45.6	32.6	M-H	C9H15NO3	0.407329586	2418.313649	2286.52861	2446.065718	659.5310505	527.6169735	626.2337791	2782.121067	2221.116091	1947.717616
5.29_268.1555m/z	268.1555454	5.28555	neg	Dumetorine	HMDB0030268	Organoheterocyclic compounds	Pyrans	Pyranones and derivatives	*		45.6	30.2	M+FA-H	C13H21NO2	0.509066958	35306.34579	28637.53166	28661.10622	14823.59034	19422.61427	21855.98253	25185.2206	17554.36592	25242.55762
6.10_239.0927m/z	239.0927363	6.101316667	neg	3-Hydroxyphenyl-valeric acid	HMDB0041666	Lipids and lipid-like molecules	Fatty Acyls	Fatty acids and conjugates	*		45.6	30.8	M+FA-H	C11H14O3	1.23217111	5059.14558	6225.829084	6156.294092	9587.762793	11393.44803	9407.828143	7028.289531	8188.695362	8364.353703
7.34_417.2134m/z	417.2134196	7.33585	neg	Blumenol C glucoside	HMDB0040668	Lipids and lipid-like molecules	Fatty Acyls	Fatty acyl glycosides	*		45.6	32.2	M+FA-H	C19H32O7	1.110598595	41900.10215	45274.3358	40837.80614	51347.5043	55613.48968	50867.96956	35694.00996	40536.98919	40604.51235
7.77_465.2495m/z	465.2495474	7.770583333	neg	Etiocholanolone glucuronide	HMDB0004484	Lipids and lipid-like molecules	Steroids and steroid derivatives	Steroidal glycosides	**	C11136	45.6	30.1	M-H	C25H38O8	0.333853676	203056.48	218211.5737	232297.8878	333482.6022	302579.3343	299344.3625	226199.3152	242657.8182	240598.9171
1.92_165.0418m/z	165.0418092	1.924916667	neg	7-Methylxanthine	58075	Organoheterocyclic compounds	Imidazopyrimidines	Purines and purine derivatives	**	C16353	45.5	34.3	M-H	C6H6N4O2	0.061197568	48936.62769	51078.94269	35887.25332	50733.5833	57753.20557	60100.071	46940.04725	49406.74866	45033.11515
3.94_168.0065m/z	168.0065154	3.9351	neg	Glyphosate	HMDB0252891	Organic acids and derivatives	Carboxylic acids and derivatives	Amino acids, peptides, and analogues	**	C05672	45.5	31.7	M-H	C3H8NO5P	-1.285141508	2783.108554	2832.473663	3073.12067	2562.029087	2630.711343	2749.553161	2260.369747	2416.17516	2239.344004
4.23_258.0384m/z	258.0384243	4.234483333	neg	N-Lactoyl ethanolamine phosphate	HMDB0032357	Organic acids and derivatives	Organic phosphoric acids and derivatives	Phosphate esters	*		45.5	32.9	M+FA-H	C5H12NO6P	-0.011061397	5431.627956	5622.20143	5348.511967	6007.117112	6172.935194	5435.98242	5566.497814	6339.231136	6095.766735
5.34_519.1504m/z	519.1504153	5.3394	neg	Enterolactone 3'-glucuronide	HMDB0240377	Lignans, neolignans and related compounds	Lignan glycosides	Unclassified			45.5	31.7	M+FA-H	C24H26O10	-0.811084019	58391.85561	66879.80226	74796.13666	2132.696029	3090.468659	2563.665151	4183.677247	5179.754328	5925.76717
0.54_165.0410m/z	165.0409682	0.535283333	neg	4-Deoxythreonic acid	HMDB0002453	Organic oxygen compounds	Organooxygen compounds	Carbohydrates and carbohydrate conjugates	*		45.4	41.5	M+FA-H	C4H8O4	4.220072819	19927.73405	492.4808887	427.766928	507.754799	856.4597241	13545.88776	397.639844	1078.333608	333.0343289
0.78_190.0591n	171.0412187	0.778533333	neg	Aspartyl-Glycine	HMDB0028753	Organic acids and derivatives	Carboxylic acids and derivatives	Amino acids, peptides, and analogues	*		45.4	33.3	M-H2O-H, M-H	C6H10N2O5	0.464836925	8938.751973	8889.011292	10566.08689	8838.736122	8720.80372	8655.182149	8531.349604	8803.861298	8788.032092
3.34_253.0831m/z	253.0831451	3.3365	neg	L-Agaridoxin	HMDB0029445	Organic acids and derivatives	Carboxylic acids and derivatives	Amino acids, peptides, and analogues	*		45.4	32	M-H	C11H14N2O5	0.590262212	9754.859939	10459.02316	9650.818966	14418.81742	13668.64378	13588.11008	10057.15614	11698.87032	10671.83435
3.85_174.0893n	219.0875086	3.84605	neg	2-Propylglutaric acid	HMDB0060684	Lipids and lipid-like molecules	Fatty Acyls	Fatty acids and conjugates	**	C16658	45.4	29.7	M-H2O-H, M+FA-H	C8H14O4	0.438948645	2803.343182	2714.315776	3212.994516	3147.476649	3312.80965	3199.453825	2768.292776	2774.992288	2730.82846
4.48_284.1506m/z	284.15056	4.477166667	neg	Salbutamol	HMDB0001937	Benzenoids	Benzene and substituted derivatives	Benzyl alcohols		C11770	45.4	30.6	M+FA-H	C13H21NO3	0.893000399	4415.878371	5134.669506	4540.855155	2773.421343	2554.351071	2438.094049	3797.710835	4966.341662	4393.429993
5.59_229.1446m/z	229.1445802	5.5933	neg	Undecylenic acid	HMDB0033724	Lipids and lipid-like molecules	Fatty Acyls	Fatty acids and conjugates	*	C13910	45.4	30	M+FA-H	C11H20O2	0.257933854	13905.09235	13544.8051	13393.00847	12244.42238	13971.82618	13220.83919	13127.3398	12768.82475	12448.38588
5.76_243.0333m/z	243.0332993	5.7632	neg	4-phenylbutanic acid-O-sulphate	HMDB0059983	Benzenoids	Benzene and substituted derivatives	Unclassified	*		45.4	30	M-H	C10H12O5S	0.128104092	445016.1828	500990.6096	480699.6039	282601.6617	301779.7214	282457.3883	600388.9867	578872.5143	498573.7024
5.98_269.1032m/z	269.1031876	5.9829	neg	Dhelwangin	HMDB0030703	Organic oxygen compounds	Organooxygen compounds	Carbonyl compounds	*		45.4	30	M+FA-H	C12H16O4	0.561080618	8760.416306	9812.606673	11144.35558	7225.349089	7811.978792	6273.887158	6898.785261	8878.06436	7965.266212
5.98_361.1508m/z	361.15077	5.9829	neg	Prenyl apiosyl-(1->6)-glucoside	HMDB0031956	Lipids and lipid-like molecules	Fatty Acyls	Fatty acyl glycosides	*		45.4	32.7	M-H2O-H	C16H28O10	0.957430843	17380.42464	19977.85584	20053.64472	20093.62991	21871.36936	22761.50323	15457.39847	17209.03602	16674.4821
1.49_147.0664m/z	147.0663923	1.492933333	neg	Tetrahydro-2-furanmethanol	HMDB0031175	Organoheterocyclic compounds	Tetrahydrofurans	Unclassified			45.3	28.7	M+FA-H	C5H10O2	1.076120546	6627.856089	6790.954862	7009.375931	5265.528156	4797.59335	4666.137487	6114.903559	5667.748154	6645.94674
1.53_180.0529m/z	180.0528511	1.534483333	neg	8-Hydroxy-7-methylguanine	HMDB0006037	Organoheterocyclic compounds	Imidazopyrimidines	Purines and purine derivatives	*		45.3	34.3	M-H	C6H7N5O2	0.845370081	18151.88232	14479.78825	14329.56809	18133.6643	17916.31274	17448.54837	15020.6724	18755.59502	15408.42958
1.72_343.0670m/z	343.0670114	1.721216667	neg	Theogallin	HMDB0039287	Organic oxygen compounds	Organooxygen compounds	Alcohols and polyols			45.3	33	M-H	C14H16O10	-0.171161162	7605.461878	7183.562762	6354.217954	2482.966909	2078.369154	1734.552979	3149.669592	3436.139749	3133.068762
2.78_225.0883m/z	225.0882711	2.776216667	neg	1-Pyrroline-5-carboxylic acid	HMDB0001301	Organic acids and derivatives	Carboxylic acids and derivatives	Amino acids, peptides, and analogues	*	C04322	45.3	31.5	2M-H	C5H7NO2	0.84314971	26186.15844	26172.13875	27540.05749	32974.59142	32097.64208	31733.08395	27247.82991	31267.48858	28944.07052
3.96_303.0182m/z	303.0182002	3.957583333	neg	5-(3',4',5'-Trihydroxyphenyl)-gamma-valerolactone-4'-O-sulphate	HMDB0059987	Organic acids and derivatives	Organic sulfuric acids and derivatives	Arylsulfates	*		45.3	34.9	M-H	C11H12O8S	0.619401344	6596.651908	6765.337784	7787.757343	2775.530418	3003.204637	2878.964035	9086.552858	10573.09289	10404.66576
4.48_241.1196m/z	241.1195741	4.477166667	neg	Glutamylleucine	HMDB0028823	Organic acids and derivatives	Carboxylic acids and derivatives	Amino acids, peptides, and analogues	*		45.3	32.7	M-H2O-H	C11H20N2O5	0.743565072	57741.16704	63068.16024	63226.23078	115358.6742	101453.2906	103054.286	59381.56433	66682.25178	63087.50689
5.06_267.0513m/z	267.0512677	5.05975	neg	2-succinylbenzoate	HMDB0304087	Unclassified	Unclassified	Unclassified			45.3	31.2	M+FA-H	C11H10O5	1.087346427	3295.027979	2994.814247	3062.263203	3611.15968	3566.291775	3783.506203	4067.266881	3265.471258	4612.702387
1.36_219.0745n	218.0671356	1.358266667	neg	O-Succinyl-L-homoserine	HMDB0255868	Unclassified	Unclassified	Unclassified			45.2	31.9	M-H2O-H, M-H	C8H13NO6	0.968812838	6095.82875	5221.381938	5546.08627	3588.648276	3622.822916	3507.519	4990.726899	4760.511158	4865.759066
1.52_232.0286m/z	232.028637	1.515416667	neg	Dopamine 4-sulfate	HMDB0004148	Organic acids and derivatives	Organic sulfuric acids and derivatives	Arylsulfates	*	C13691	45.2	31	M-H	C8H11NO5S	0.514714488	10228.59861	9982.2059	11209.52064	8831.067415	8301.502144	8191.838323	8871.940175	10412.53605	9329.151072
3.40_262.9867m/z	262.9867286	3.40385	neg	Methylgallic acid-O-sulphate	HMDB0060005	Benzenoids	Benzene and substituted derivatives	Benzoic acids and derivatives	*		45.2	30.3	M-H	C8H8O8S	0.06364911	116686.3474	132106.2893	120182.697	137253.5857	134312.1786	129027.7454	105275.2588	113491.1874	109467.5126
3.52_189.0770m/z	189.0769718	3.52125	neg	2S-Hydroxy-2-isopropylbutano-3S-lactone	LMFA07040002	Lipids and lipid-like molecules	Fatty Acyls	Fatty esters			45.2	28.1	M+FA-H	C7H12O3	0.86537996	6440.581588	6449.472927	6596.581436	7432.260709	7616.925695	7264.869013	5064.987947	5872.544865	5700.186355
5.95_432.2361n	431.2288358	5.95155	neg	Glucosyl (2E,6E,10x)-10,11-dihydroxy-2,6-farnesadienoate	HMDB0037823	Lipids and lipid-like molecules	Prenol lipids	Terpene glycosides	*		45.2	29.9	M-H, M+FA-H	C21H36O9	0.415346249	24405.62903	27362.12755	29114.24771	12297.25778	15991.63945	14837.55437	29623.2891	32987.55431	31173.8038
5.99_198.1137m/z	198.1136909	5.9916	neg	2-octenoylglycine	HMDB0094801	Organic acids and derivatives	Carboxylic acids and derivatives	Amino acids, peptides, and analogues			45.2	28.9	M-H	C10H17NO3	0.622355401	20763.64089	22835.41059	22905.34293	33204.684	34631.35015	38018.72653	26166.1124	27205.23485	25938.62209
6.46_408.2879n	407.2806642	6.458366667	neg	Lagocholic Acid	LMST04010085	Lipids and lipid-like molecules	Steroids and steroid derivatives	Bile acids, alcohols and derivatives	*		45.2	32.5	M-H, M+FA-H	C24H40O5	0.897122493	24222.19895	22745.5544	26338.70323	14002.12255	10963.38712	12393.7964	24510.9174	27680.89745	26338.7179
1.14_198.0754n	197.0680811	1.1382	neg	6-amino-5[N-methylformylamino]-1-methyluracil	HMDB0059771	Organic nitrogen compounds	Organonitrogen compounds	N-arylamides			45.1	33.8	M-H, 2M-H	C7H10N4O3	0.339765386	51284.85952	40838.52594	39567.52772	64675.57669	58905.83112	74385.14881	42763.60929	60207.29418	52784.15538
1.46_181.0368m/z	181.0368396	1.459966667	neg	ronidazole	HMDB0257302	Unclassified	Unclassified	Unclassified			45.1	33.4	M-H2O-H	C6H8N4O4	0.629854789	18345.27383	24973.70981	20144.85952	28632.44099	23818.76609	26272.64566	28994.81758	20026.47396	24040.14226
3.42_303.0723m/z	303.0723312	3.417666667	neg	Lepidimoic acid	HMDB0041096	Organic oxygen compounds	Organooxygen compounds	Carbohydrates and carbohydrate conjugates			45.1	28.8	M-H2O-H	C12H18O10	0.545075619	63243.56634	73354.13289	67606.26463	32166.27621	31365.53243	31746.01637	37798.55945	37840.61586	36451.85813
4.52_171.0664m/z	171.0664118	4.521733333	neg	cis-4-Octenedioic acid	HMDB0004982	Lipids and lipid-like molecules	Fatty Acyls	Fatty acids and conjugates	*		45.1	28.4	M-H	C8H12O4	0.751756932	6324.330906	7501.945738	7367.469545	12183.4533	10974.77191	9946.768756	8286.746624	8795.343436	10354.67039
4.84_287.1503m/z	287.1502569	4.836566667	neg	Botryodiplodin	HMDB0036594	Organoheterocyclic compounds	Tetrahydrofurans	Unclassified			45.1	30.3	2M-H	C7H12O3	0.849590032	12153.52111	12228.13619	12557.90322	17269.45194	16986.90653	16633.19101	11927.78465	12710.16655	10578.52434
5.53_273.1709m/z	273.1709178	5.525416667	neg	3-hydroxy-tetradecanedioic acid	LMFA01170094	Lipids and lipid-like molecules	Fatty Acyls	Fatty Acids and Conjugates			45.1	29.2	M-H	C14H26O5	0.6212868	43062.18178	37849.33885	40520.60726	26705.32904	31698.66769	28042.17012	32003.04885	32574.21126	33668.14296
0.82_207.0147m/z	207.0146728	0.819566667	neg	Hydroxypyruvic acid	HMDB0001352	Organic acids and derivatives	Hydroxy acids and derivatives	Beta hydroxy acids and derivatives	**	C00168	45	26.2	2M-H	C3H4O4	0.154145415	8677.373025	9604.25735	10179.84353	17388.45254	18819.06651	17944.85483	9780.13444	10850.48512	11030.11839
1.27_195.0334m/z	195.0334001	1.26875	neg	(±)-2-Hydroxy-4-(methylthio)butanoic acid	HMDB0037115	Lipids and lipid-like molecules	Fatty Acyls	Fatty acids and conjugates	*		45	28	M+FA-H	C5H10O3S	0.880139236	11259.51305	12671.30692	12656.70717	4617.667533	4124.05001	3850.777664	10227.92649	11957.69497	10882.25261
1.86_154.9987m/z	154.9987469	1.86075	neg	2,5-Furandicarboxylic acid	HMDB0004812	Organoheterocyclic compounds	Furans	Furoic acid and derivatives	*	C20450	45	31.8	M-H	C6H4O5	0.962218526	2575.265536	2620.313498	2901.254699	1601.347315	1670.752687	1604.104783	1208.554784	999.2266515	1059.718833
2.63_233.0126m/z	233.0126029	2.634083333	neg	Hydroxytyrosol 4'-sulfate	HMDB0240532	Organic acids and derivatives	Organic sulfuric acids and derivatives	Arylsulfates			45	29.6	M-H	C8H10O6S	0.300401909	39862.92927	42107.58156	45047.93213	3159.716997	3093.592664	3047.956487	56908.35459	58192.82645	58923.25221
3.98_369.0824m/z	369.0824165	3.97985	neg	Feruloyl C1-glucuronide	HMDB0041734	Phenylpropanoids and polyketides	Isoflavonoids	Isoflavonoid O-glycosides	*		45	30.8	M-H	C16H18O10	-0.820929041	21158.71455	21753.18233	24084.86574	14034.45532	12103.31637	12252.60499	19720.6994	21669.94897	20306.58484
4.50_173.1053n	172.0980727	4.49925	neg	ACEXAMIC ACID	44105	Unclassified	Unclassified	Unclassified			45	29.2	M-H2O-H, M-H	C8H15NO3	0.469271274	2842.745075	3369.838251	3306.408068	1918.339553	1967.49744	1869.874598	2899.00509	2933.019965	3359.882948
4.75_164.0717m/z	164.0716863	4.747466667	neg	Methylnoradrenaline	HMDB0002832	Benzenoids	Benzene and substituted derivatives	Phenylpropanes	*	C17925	45	28.5	M-H2O-H	C9H13NO3	-0.086579039	2656.681236	2669.072068	2440.473135	1126.870754	1188.254084	1187.481739	2132.953386	2537.607816	2238.470686
4.81_187.0072m/z	187.0071534	4.814166667	neg	p-Toluenesulfonic acid	HMDB0059933	Benzenoids	Benzene and substituted derivatives	Benzenesulfonic acids and derivatives	***	C06677	45	30.9	M-H	C7H8O4S	0.532342117	202268.3165	219330.5039	225073.7022	93444.54891	90798.46672	93905.70191	111949.5662	123423.8502	118419.9353
4.92_170.0824m/z	170.0823893	4.92485	neg	4-Butyloxazole	HMDB0038288	Organoheterocyclic compounds	Azoles	Oxazoles			45	27.5	M+FA-H	C7H11NO	0.978592758	1834.567485	1954.05276	2030.033152	4032.895377	4103.848024	3868.788144	1798.334691	2041.934704	1898.375029
5.08_86.0368n	171.0664205	5.080566667	neg	But-2-enoic acid	HMDB0010720	Lipids and lipid-like molecules	Fatty Acyls	Fatty acids and conjugates	*	C01771	45	27.1	M+FA-H, 2M-H	C4H6O2	0.185397614	6059.301336	4961.980851	4548.885095	5230.678345	5540.176593	5713.036216	6542.369318	4955.04075	5508.100159
5.38_423.0759m/z	423.0759018	5.384216667	neg	Enterolactone 3''-sulfate	HMDB0240506	Unclassified	Unclassified	Unclassified			45	34.8	M+FA-H	C18H18O7S	0.992022979	11299.82316	14064.7274	12740.62428	18610.42195	17245.83495	16949.20375	2706.881372	3327.871949	2886.345674
6.26_181.0872m/z	181.0872089	6.261966667	neg	alpha-Carboxy-delta-nonalactone	HMDB0030990	Organoheterocyclic compounds	Lactones	Delta valerolactones	*		45	29.1	M-H2O-H	C10H16O4	0.954721756	2452.57883	1783.549731	2220.717972	3004.175176	2675.486407	3246.546332	2572.076503	2868.515111	2677.853661
1.03_284.8971m/z	284.8971243	1.033516667	neg	Trimetaphosphoric acid	HMDB0059921	Homogeneous non-metal compounds	Non-metal oxoanionic compounds	Non-metal phosphates		C02466	44.9	27	M+FA-H	H3O9P3	-0.292435395	26059.54214	25761.94239	24297.77972	30946.61216	33362.5985	32728.58009	32527.73905	29083.74616	32063.57461
3.66_246.9919m/z	246.9918692	3.6635	neg	Benzoic acid sulfate	HMDB0240374	Benzenoids	Benzene and substituted derivatives	Benzoic acids and derivatives			44.9	28.3	M+FA-H	C7H6O5S	0.356830728	59350.46659	64577.50019	66132.58034	32225.63637	33270.41588	29908.63501	59289.17556	65305.42995	66403.5253
4.41_146.0692n	145.061842	4.41115	neg	Alanylglycine	HMDB0006899	Organic acids and derivatives	Carboxylic acids and derivatives	Amino acids, peptides, and analogues	*		44.9	31.3	M-H2O-H, M-H	C5H10N2O3	0.130433096	16309.61388	16647.01028	15905.78218	12013.92538	11352.92807	11641.86291	17139.27506	20539.58891	19773.04432
4.52_273.0917m/z	273.0916792	4.521733333	neg	N-gamma-Glutamyl-S-propylcysteine	HMDB0039096	Organic acids and derivatives	Carboxylic acids and derivatives	Amino acids, peptides, and analogues			44.9	28.6	M-H2O-H	C11H20N2O5S	0.778954266	9883.532288	10630.59061	11300.46404	10439.64861	9789.155289	9551.35365	11020.28235	11820.26505	11330.85526
7.18_367.1587m/z	367.1587237	7.178516667	neg	Dehydroepiandrosterone sulfate	HMDB0001032	Lipids and lipid-like molecules	Steroids and steroid derivatives	Sulfated steroids	***	C04555	44.9	29.9	M-H	C19H28O5S	0.692885743	453356.5393	514350.5856	498555.7269	581164.0965	569078.304	586619.3315	453482.6038	534829.3864	494501.243
1.12_187.0726m/z	187.0725767	1.11925	neg	Nα-Acetyl-L-glutamine	58344	Unclassified	Unclassified	Unclassified			44.8	30.8	M-H	C7H12N2O4	0.777549793	4305.181495	4146.063382	4527.306755	4211.018413	3357.755604	4003.861965	4713.583711	4977.285644	4614.348643
3.77_407.0452m/z	407.0451734	3.770416667	neg	Daucic acid	HMDB0031665	Organic acids and derivatives	Hydroxy acids and derivatives	Beta hydroxy acids and derivatives	*		44.8	37	2M-H	C7H8O7	-3.811730464	17521.07717	15190.09279	15974.82707	29188.48514	28092.29338	18514.97167	18086.81009	18085.41874	18192.61945
4.43_222.0772m/z	222.0772175	4.432683333	neg	2-Propenyl 2-aminobenzoate	HMDB0037693	Benzenoids	Benzene and substituted derivatives	Benzoic acids and derivatives			44.8	27.5	M+FA-H	C10H11NO2	0.203441525	1168.774861	932.3224913	1031.886032	718.802523	704.7920987	552.7604911	950.9005087	977.2125626	1332.972113
5.24_243.0776m/z	243.0776107	5.244033333	neg	Indolylacryloylglycine	HMDB0006005	Organic acids and derivatives	Carboxylic acids and derivatives	Amino acids, peptides, and analogues	*		44.8	29.5	M-H	C13H12N2O3	0.388730782	1970.079048	1813.120956	2034.988277	1467.716396	1349.713837	1180.592382	389.2096844	451.3605059	578.2413634
5.30_549.1617m/z	549.161682	5.302883333	neg	Isoliquiritin apioside	HMDB0253655	Phenylpropanoids and polyketides	Flavonoids	Flavonoid glycosides			44.8	28.2	M-H	C26H30O13	0.576966052	93443.31229	88809.88016	94038.58417	1711.158249	2071.460709	2513.575438	17240.9908	14206.67951	19121.58763
1.38_205.0355m/z	205.0354962	1.380283333	neg	3-Oxoadipic acid	HMDB0000398	Organic acids and derivatives	Keto acids and derivatives	Medium-chain keto acids and derivatives	**	C00846	44.7	26.5	M+FA-H	C6H8O5	0.74955188	11418.46546	11595.30367	11913.36528	15896.72903	14850.39805	14610.00092	12125.28574	11979.5626	12382.67546
4.73_193.0482m/z	193.04818	4.725416667	neg	Temozolomide	HMDB0014991	Organoheterocyclic compounds	Imidazotetrazines	Unclassified			44.7	27.2	M-H	C6H6N6O2	1.200518707	4370.267632	4033.748613	4409.862244	6373.597199	6722.418624	6768.921663	4299.574407	4683.022655	4294.254853
4.99_229.0178m/z	229.0177505	4.99225	neg	Homovanillyl alcohol sulfate	HMDB0240528	Organic acids and derivatives	Organic sulfuric acids and derivatives	Arylsulfates			44.7	33.4	M-H2O-H	C9H12O6S	0.534077456	1983.74444	2139.305925	2338.020565	947.7746006	924.2411746	913.1443139	2308.422989	1876.939878	1663.729749
5.56_245.1395m/z	245.1395316	5.56185	neg	Hexyl glucoside	HMDB0031688	Lipids and lipid-like molecules	Fatty Acyls	Fatty acyl glycosides	*		44.7	28.7	M-H2O-H	C12H24O6	0.318999988	6340.869213	4910.017146	5273.229225	4417.691823	5189.354791	4518.795789	6571.732239	6110.3653	5442.220856
3.58_231.0875m/z	231.0874876	3.5753	neg	5-Butyltetrahydro-2-oxo-3-furancarboxylic acid	HMDB0030992	Organoheterocyclic compounds	Lactones	Gamma butyrolactones	*		44.6	26.1	M+FA-H	C9H14O4	0.407446587	2774.649094	2892.563662	3030.991572	3679.301525	3758.712896	3620.350224	3122.739182	3446.708088	3282.238386
3.80_347.0619n	346.0546633	3.801716667	neg	2'-Deoxyguanosine 5'-monophosphate	HMDB0001044	Nucleosides, nucleotides, and analogues	Purine nucleotides	Purine deoxyribonucleotides	**	C00362	44.6	29.9	M-H, M+FA-H	C10H14N5O7P	-3.298213002	30641.32794	29974.11739	31791.73727	2837.760141	3079.625658	2500.446595	26009.92312	27768.04542	28068.94979
4.28_277.0024m/z	277.0023756	4.278183333	neg	2-[4-(sulfooxy)phenyl]acetic acid	HMDB0132500	Organic acids and derivatives	Organic sulfuric acids and derivatives	Arylsulfates	*		44.6	31.3	M+FA-H	C8H8O6S	0.059261989	4327.120974	4484.171324	4467.558871	4290.999947	3900.54606	3952.598536	3116.700343	3693.875333	3171.258699
4.43_131.0714m/z	131.0714016	4.432683333	neg	6-Hydroxyhexanoic acid	HMDB0012843	Organic acids and derivatives	Hydroxy acids and derivatives	Medium-chain hydroxy acids and derivatives	*	C06103	44.6	26.9	M-H	C6H12O3	0.256185787	5602.064223	5874.803546	5960.722712	8216.305868	7810.925903	7864.474811	6963.634245	6764.025474	6442.032082
5.10_333.0081m/z	333.0080886	5.1031	neg	5,7,4'-Trihydroxyflavanone 7-sulfate	LMPK12140327	Unclassified	Unclassified	Unclassified			44.6	35.3	M-H2O-H	C15H12O8S	1.82185877	3976.200526	4065.948738	3743.757814	10721.50132	10712.39391	12413.64836	1397.743174	1305.922136	1222.30746
5.71_411.1662m/z	411.1661887	5.709933333	neg	Secoeremopetasitolide A	HMDB0041364	Lipids and lipid-like molecules	Prenol lipids	Terpene lactones	*		44.6	29.7	M+FA-H	C19H26O7	0.362165636	47883.40465	50557.70811	49590.29903	51479.26538	55344.57728	53198.51871	41216.07315	42740.06931	45149.76664
0.78_399.0950m/z	399.0949901	0.778533333	neg	Biflorin	HMDB0002336	Organic oxygen compounds	Organooxygen compounds	Carbohydrates and carbohydrate conjugates		C08996	44.5	33.6	M+FA-H	C16H18O9	4.815284609	105269.1614	121399.0455	108172.4613	146447.0787	242707.9267	170153.1751	147416.0711	152596.9356	153247.7843
1.22_171.0413m/z	171.0412932	1.224233333	neg	Hydantoin-5-propionic acid	6084	Organoheterocyclic compounds	Azolidines	Imidazolidines	**	C05565	44.5	31.4	M-H	C6H8N2O4	0.946740002	5596.194921	6117.144123	5987.345762	11441.46537	11353.77718	11348.1065	7555.411483	8478.634179	8002.908386
2.18_227.9974m/z	227.9974142	2.180683333	neg	HHPAA sulfate	HMDB0240380	Organic acids and derivatives	Organic sulfuric acids and derivatives	Arylsulfates			44.5	30.8	M-H2O-H	C8H9NO6S	0.798881807	49565.91968	46429.23039	52517.7224	3989.616709	3688.315632	3929.72773	43962.60534	49481.49992	46804.81774
4.00_248.0599m/z	248.059928	4.002233333	neg	N-Acetyl-S-allylcysteine	HMDB0240543	Organic acids and derivatives	Carboxylic acids and derivatives	Amino acids, peptides, and analogues			44.4	25	M+FA-H	C8H13NO3S	0.545774546	30871.92724	33519.14689	35246.52263	21307.83231	20608.63755	20369.7908	21532.85054	24709.98697	23380.68561
4.41_215.0925m/z	215.0925253	4.41115	neg	1,4-Ipomeadiol	HMDB0030471	Organoheterocyclic compounds	Heteroaromatic compounds	Unclassified	*		44.4	25.5	M+FA-H	C9H14O3	0.1655524	6452.395699	7584.822836	6876.97124	10275.58774	9482.513692	9908.156225	6553.187075	7498.497571	8039.526671
5.20_271.1553m/z	271.1553134	5.20005	neg	4,5-Dihydrovomifoliol	HMDB0040615	Lipids and lipid-like molecules	Prenol lipids	Sesquiterpenoids	*		44.4	25.3	M+FA-H	C13H22O3	0.954751725	11926.0427	12171.19115	11542.14823	9801.976094	9969.501824	10729.0279	9547.780995	9245.570397	9771.582456
5.47_237.1246m/z	237.1246162	5.471616667	neg	Secobarbital	43331	Organoheterocyclic compounds	Diazines	Pyrimidines and pyrimidine derivatives			44.4	26.5	M-H	C12H18N2O3	0.630729694	13865.18003	10263.61239	10583.07731	9796.016397	9267.826196	10905.89237	7805.151471	5325.416522	6093.202008
6.58_341.1244m/z	341.1243595	6.582416667	neg	Chavicol O-beta-glucopyranoside	HMDB0041617	Organic oxygen compounds	Organooxygen compounds	Carbohydrates and carbohydrate conjugates			44.4	25.3	M+FA-H	C15H20O6	0.568303193	38002.00763	40564.78499	40195.98609	71944.38222	73713.06474	72341.12792	46423.65143	55874.1432	55251.95197
0.71_112.9857m/z	112.985652	0.714216667	neg	Trifluoroacetic acid	HMDB0014118	Organic acids and derivatives	Carboxylic acids and derivatives	Alpha-halocarboxylic acids and derivatives	*		44.3	23.4	M-H	C2HF3O2	0.565553479	118985.5208	116622.6425	120843.6494	105314.361	105856.838	107626.792	125129.3499	116749.4378	124625.0912
0.80_267.0722m/z	267.0722144	0.800366667	neg	Ethyl glucuronide	HMDB0010325	Organic oxygen compounds	Organooxygen compounds	Carbohydrates and carbohydrate conjugates	*		44.3	29.3	M+FA-H	C8H14O7	0.26472859	16040.89847	16845.26317	18662.47244	16655.17534	18809.97253	17718.30937	16234.03385	19537.13549	18158.72317
2.07_189.1002n	188.0929083	2.070366667	neg	4-hydroxyhexanoylglycine	HMDB0094721	Organic acids and derivatives	Carboxylic acids and derivatives	Amino acids, peptides, and analogues			44.3	23.7	M-H2O-H, M-H	C8H15NO4	0.376853288	67317.8781	78042.26966	78337.94518	71936.6464	79159.33176	72502.08057	82858.40419	74891.98275	76759.71698
2.20_177.0228m/z	177.0227586	2.203283333	neg	2,3-Dihydroxy-5-methylthio-4-pentenoic acid	HMDB0240388	Lipids and lipid-like molecules	Fatty Acyls	Fatty acids and conjugates			44.3	27.8	M-H	C6H10O4S	0.310040445	32010.96737	29214.43678	33970.00192	45839.87206	41425.00759	42738.26448	33602.14274	36591.30082	32818.73583
4.88_333.0594m/z	333.0593732	4.880083333	neg	Glycerophosphoinositol	HMDB0011649	Lipids and lipid-like molecules	Glycerophospholipids	Glycerophosphoinositols	*	C01225	44.3	27.5	M-H	C9H19O11P	0.453748152	8271.346721	8719.100195	9232.835254	416.7098392	328.1841069	146.5449036	14381.07608	15271.9236	15260.0615
4.95_477.1770m/z	477.1769595	4.94705	neg	(7R*,8R*)-3-Methoxy-3',4,7,9,9'-pentahydroxy-8,4'-oxyneolignan 4-xyloside	HMDB0040325	Lignans, neolignans and related compounds	Lignan glycosides	Unclassified			44.3	24	M-H2O-H	C24H32O11	0.682735328	151135.0554	161164.9968	157542.4919	559.5341947	679.4385182	736.1580934	94127.34998	95085.13564	96220.88393
6.49_225.1135m/z	225.1134762	6.494283333	neg	Norecasantalic acid	HMDB0036817	Lipids and lipid-like molecules	Prenol lipids	Monoterpenoids	*		44.3	25.4	M+FA-H	C11H16O2	1.352508656	12777.06113	14276.77843	14745.84704	21066.73227	21877.71103	20972.0579	11816.88648	13618.04751	12792.85371
4.16_230.9970m/z	230.9969578	4.15895	neg	Methyl-4-hydroxybenzoate sulfate	HMDB0168668	Organic acids and derivatives	Organic sulfuric acids and derivatives	Arylsulfates			44.2	31.1	M-H	C8H8O6S	0.324441503	4574.54171	4361.282686	4529.755103	1706.002583	1275.510752	1588.41357	2182.734244	1789.709145	2024.253482
6.54_547.1463m/z	547.1462793	6.539816667	neg	5,7,4'-Trihydroxyflavonone 4'-O-xylosylglucoside	LMPK12140252	Unclassified	Unclassified	Unclassified			44.2	23.9	M-H2O-H	C26H30O14	0.997475324	25804.56056	27603.51066	26862.58799	39758.22355	45901.48868	44924.35409	18384.83747	19802.28286	17779.13816
7.26_483.2215m/z	483.2214904	7.262266667	neg	Eriojaposide A	HMDB0038028	Lipids and lipid-like molecules	Fatty Acyls	Fatty acyl glycosides	*		44.2	33.9	M-H2O-H	C24H38O11	-4.142543023	23445.82561	26304.63048	25841.48113	27838.40712	27185.42901	28309.24323	32355.441	34175.23915	36331.15806
1.22_286.1047m/z	286.1047499	1.224233333	neg	3'-Amino-3'-deoxythimidine	HMDB0060750	Nucleosides, nucleotides, and analogues	Pyrimidine nucleosides	Pyrimidine 2',3'-dideoxyribonucleosides	*		44.1	27.8	M+FA-H	C10H15N3O4	1.207075421	3005.108622	3537.817498	3461.79458	2967.895951	2955.013962	3161.636392	2514.006903	2937.377332	2895.197994
5.29_297.0982m/z	297.0982196	5.28555	neg	2-Phenylethanol glucuronide	HMDB0010350	Organic oxygen compounds	Organooxygen compounds	Carbohydrates and carbohydrate conjugates	**	C03033	44.1	27	M-H	C14H18O7	0.815644164	1056.17851	984.2645504	988.5758034	5904.030894	6585.640011	6573.16095	10832.32595	8581.659772	11065.3669
6.63_229.0541m/z	229.0541189	6.63275	neg	Thymol Sulfate	HMDB0062720	Organic acids and derivatives	Organic sulfuric acids and derivatives	Arylsulfates	*		44.1	27	M-H	C10H14O4S	0.501616679	15139.17758	15346.50255	14681.53888	7911.917634	7766.861472	7322.586416	16394.50105	19056.05063	19037.84692
2.69_355.0899m/z	355.0898546	2.692466667	neg	Acaciabiuronic acid	HMDB0029938	Organic oxygen compounds	Organooxygen compounds	Carbohydrates and carbohydrate conjugates	*		44	29.6	M-H	C12H20O12	4.647521561	15510.90705	15099.07514	15033.05781	22299.5462	21186.15874	21117.24221	16412.31294	18105.43105	17237.63911
3.55_253.1195m/z	253.1194949	3.549183333	neg	Aldosine	HMDB0037817	Organic acids and derivatives	Carboxylic acids and derivatives	Amino acids, peptides, and analogues			44	24.5	M-H	C12H18N2O4	0.44949943	7315.567637	8485.328339	7974.83216	4416.127503	5373.510709	4561.665763	3865.695905	4034.965021	4216.424731
4.05_179.0463m/z	179.0463152	4.046666667	neg	Mimosine	HMDB0015188	Organic acids and derivatives	Carboxylic acids and derivatives	Amino acids, peptides, and analogues		C04771	44	30.4	M-H2O-H	C8H10N2O4	0.502267583	5773.342258	6426.706976	6608.909005	9971.521873	10187.56292	9868.556885	7320.683281	8209.890797	7910.403003
4.86_469.1308m/z	469.1307973	4.858383333	neg	Khelmarin D	HMDB0041394	Phenylpropanoids and polyketides	Coumarins and derivatives	Pyranocoumarins			44	35.9	M-H2O-H	C28H24O8	3.115316783	18189.59746	18540.18885	19643.54708	22454.42631	21342.12247	21399.66829	22071.09356	24801.7819	23845.62205

1.25_206.0494m/z	206.0494358	1.246333333	neg	(2R,2'S)-Isobuteine	HMDB0030411	Organic acids and derivatives	Carboxylic acids and derivatives	Amino acids, peptides, and analogues	*		43.9	33.9	M-H	C7H13NO4S	0.885489524	6423.415602	7225.230486	7364.462417	4043.113891	4106.035122	3973.580248	4231.681904	4746.769715	4519.675073
6.10_433.2083m/z	433.2082724	6.101316667	neg	5a,6a-Epoxy-7E-megastigmene-3b,9e-diol 9-glucoside	HMDB0038306	Lipids and lipid-like molecules	Fatty Acyls	Fatty acyl glycosides	*		43.9	22.3	M+FA-H	C19H32O8	0.905612439	50665.39506	59285.8212	59092.62485	44136.45004	51840.6083	45684.12358	49668.58552	55721.0564	59748.59579
6.18_295.1665m/z	295.1664596	6.181866667	neg	3-Hydroxylidocaine	HMDB0060655	Organic acids and derivatives	Carboxylic acids and derivatives	Amino acids, peptides, and analogues	**	C16560	43.9	24.1	M+FA-H	C14H22N2O2	0.514894281	6025.511502	6321.552338	6753.640554	11106.62189	10829.54848	11965.57025	6084.822037	7493.93544	7891.542729
7.55_511.2915m/z	511.29146	7.547766667	neg	(S)-Nerolidol 3-O-[a-L-rhamnopyranosyl-(1->2)-b-D-glucopyranoside]	HMDB0040844	Lipids and lipid-like molecules	Fatty Acyls	Fatty acyl glycosides	*		43.9	21.7	M-H2O-H	C27H46O10	0.38372878	131498.8523	140210.663	137402.8048	182695.0597	179032.7558	176210.1816	120599.9756	129419.6845	128421.6907
1.16_173.0568m/z	173.0567732	1.160166667	neg	Formiminoglutamic acid	HMDB0000854	Organic acids and derivatives	Carboxylic acids and derivatives	Amino acids, peptides, and analogues	**	C00439	43.8	22.8	M-H	C6H10N2O4	-0.04147438	8829.580644	9517.586744	9909.143944	12196.13005	12009.28171	11359.21343	10689.8863	11163.60356	11181.32411
1.49_133.0507m/z	133.0507102	1.492933333	neg	2,3-Dihydroxy-2-methylbutanoic acid	HMDB0029576	Lipids and lipid-like molecules	Fatty Acyls	Fatty acids and conjugates	*		43.8	21.4	M-H	C5H10O4	0.580545007	4754.324036	4913.788779	5519.903912	3766.12204	3588.874925	3492.474607	4538.404342	4403.759094	4408.363775
1.80_143.0350m/z	143.0349764	1.798266667	neg	2(R)-Hydroxyadipic acid	HMDB0000368	Lipids and lipid-like molecules	Fatty Acyls	Fatty acids and conjugates	*		43.8	22.7	M-H2O-H	C6H10O5	-0.03632602	2602.716527	2417.377494	2776.50851	2481.068821	2489.165186	2644.007582	2506.83432	2858.836058	2618.297395
2.11_137.0244m/z	137.0244264	2.114866667	neg	Salicylic acid	HMDB0001895	Benzenoids	Benzene and substituted derivatives	Benzoic acids and derivatives	***	C00805	43.8	23.3	M-H	C7H6O3	0.063345876	3894.583517	4192.342972	4115.891671	4511.225736	4683.634672	5061.633986	4569.820046	4033.08078	4433.194358
4.19_325.0931m/z	325.093136	4.189783333	neg	1-O-p-Coumaroyl-beta-D-glucose	HMDB0036936	Phenylpropanoids and polyketides	Cinnamic acids and derivatives	Hydroxycinnamic acids and derivatives			43.8	24.6	M-H	C15H18O8	0.751020873	11519.89822	9244.436535	10019.54313	5990.557568	5490.820638	7547.094713	7761.325065	5994.683197	5923.403425
4.48_267.0866m/z	267.0866396	4.477166667	neg	2,3-Dihydro-3-hydroxy-6-methoxy-2,2-dimethyl-4H-1-benzopyran-4-one	HMDB0041379	Organoheterocyclic compounds	Benzopyrans	1-benzopyrans			43.8	27.1	M+FA-H	C12H14O4	-3.476768014	3090.342159	3548.459003	3394.700241	2583.405336	2448.94761	2652.954742	3443.445008	3887.170019	3692.214146
4.75_213.0770m/z	213.0770411	4.747466667	neg	Epoxyoxophorone	HMDB0032252	Organoheterocyclic compounds	Oxepanes	Unclassified	*		43.8	22.5	M+FA-H	C9H12O3	1.153912798	10834.12021	10771.38699	11331.01497	10628.69808	10480.94635	10183.41522	13011.03936	13294.55596	13598.33916
0.78_181.0719m/z	181.0718771	0.778533333	neg	Dulcitol	4179	Organic oxygen compounds	Organooxygen compounds	Carbohydrates and carbohydrate conjugates	**	C01697	43.7	24.8	M-H	C6H14O6	0.633622757	8880.484329	7517.730855	8747.000213	5871.709008	6332.41237	5372.455185	5443.815019	7997.13462	7273.93387
0.88_145.0375n	144.0302629	0.884633333	neg	2-Keto-glutaramic acid	HMDB0001552	Organic acids and derivatives	Keto acids and derivatives	Short-chain keto acids and derivatives	**	C00940	43.7	29.4	M-H2O-H, M-H	C5H7NO4	0.179755286	11555.46309	12314.86263	12316.72079	12235.08333	11168.47234	12108.29579	12063.9015	11929.29122	11963.70913
2.42_144.0667m/z	144.0666663	2.415216667	neg	2-Amino-4-ethoxy-3-hydroxybutanoic acid	HMDB0032862	Organic acids and derivatives	Carboxylic acids and derivatives	Amino acids, peptides, and analogues			43.7	21.8	M-H2O-H	C6H13NO4	0.303355657	5999.521334	6229.384536	7051.60923	7942.272316	7247.128581	7087.258834	6906.85086	7947.944324	6918.953188
3.13_117.0557m/z	117.0557412	3.131933333	neg	5-Hydroxypentanoic acid	HMDB0061927	Lipids and lipid-like molecules	Fatty Acyls	Fatty acids and conjugates		C02804	43.7	21.1	M-H	C5H10O3	0.198980337	10025.30076	10358.32205	10521.52435	16234.04895	15296.82727	14865.76481	12531.72825	13416.74358	12725.6896
3.15_216.9812m/z	216.9812451	3.153616667	neg	4-hydroxybenzoic acid-4-O-sulphate	HMDB0059982	Organic acids and derivatives	Organic sulfuric acids and derivatives	Arylsulfates	*		43.7	31.6	M-H	C7H6O6S	0.057767544	14107.23095	14388.26567	15116.66545	11653.34805	10589.63121	10737.86771	13863.23568	15788.88432	14610.53072
4.11_217.1082m/z	217.1082361	4.113883333	neg	(3S,6S)-3-Hydroxy-1,7-dioxaspiro[5.5]undecane	LMPK09000006	Unclassified	Unclassified	Unclassified			43.7	21.8	M+FA-H	C9H16O3	0.516482733	7803.486935	8117.630458	8848.139477	6515.593089	6277.338371	6353.418025	7076.170454	7003.33201	6802.050552
4.57_302.1149m/z	302.1148729	4.5668	neg	Carmofur	HMDB0249685	Unclassified	Unclassified	Unclassified			43.7	26.5	M+FA-H	C11H16FN3O3	-3.498690106	7964.169059	8275.284503	8136.068422	16243.33328	15849.06387	14980.12736	8707.24065	9334.390386	8994.297592
4.97_449.2033m/z	449.2032893	4.969683333	neg	Pisumionoside	HMDB0039947	Lipids and lipid-like molecules	Prenol lipids	Terpene glycosides	*		43.7	21.1	M+FA-H	C19H32O9	1.122752162	26172.10831	25940.56891	28852.39633	25401.79482	27026.43138	23334.32682	38272.04194	36339.27758	34618.07964
5.26_375.1663m/z	375.1663314	5.263033333	neg	6-Hydroxy-2-bornanone glucoside	HMDB0034558	Lipids and lipid-like molecules	Prenol lipids	Terpene glycosides	*		43.7	25.8	M+FA-H	C16H26O7	0.833929232	17406.89809	16313.7785	16147.56424	21457.23562	20217.67151	20455.1492	16836.20079	14931.04115	18440.29215
6.59_469.2082m/z	469.2082169	6.592933333	neg	4-(5'-((1E,3E,6Z,9Z)-dodeca-1,3,6,9-tetraen-1-yl)-[3,3'-bi(1,2-dioxolan)]-5-yl)-4-I	LMFA04060002	Lipids and lipid-like molecules	Fatty Acyls	Docosanoids			43.7	21.6	M+FA-H	C22H32O8	0.697920109	64353.29685	66883.89792	66408.34157	17278.93245	17838.65091	17007.76191	43258.40407	51836.14624	48927.3825
6.66_157.0870m/z	157.0870494	6.663683333	neg	trans-2-trans-4-Heptadien-1-ol	HMDB0032301	Lipids and lipid-like molecules	Fatty Acyls	Fatty alcohols	*		43.7	20	M+FA-H	C7H12O	0.281246149	9435.767095	9710.898141	9781.780724	14008.75896	13793.51373	11351.40831	11564.49646	12603.3359	12963.04978
6.68_198.1137m/z	198.11367	6.680966667	neg	psi-Pelletierine	HMDB0034580	Organoheterocyclic compounds	Piperidines	Piperidinones		C10865	43.7	21	M+FA-H	C9H15NO	0.67311386	2414.703969	3001.649179	2950.811227	6056.366102	6209.799266	5274.020849	2440.136467	2741.702493	2845.464059
4.54_230.0130m/z	230.0129955	4.54425	neg	Norepinephrine sulfate	HMDB0002062	Organic acids and derivatives	Organic sulfuric acids and derivatives	Arylsulfates	*		43.6	28.7	M-H2O-H	C8H11NO6S	0.51608552	1914.710463	2183.890781	2311.715652	1978.18693	2003.934314	1939.183989	2291.942269	2594.597349	2465.297915
4.61_467.1836m/z	467.1835805	4.611883333	neg	4'-Hydroxycarvedilol	HMDB0013947	Organoheterocyclic compounds	Indoles and derivatives	Carbazoles	*		43.6	29.5	M+FA-H	C24H26N2O5	2.855778188	17875.82405	19106.66739	18840.33246	14615.56966	14584.92458	12902.05322	18919.18626	17586.06006	21630.887
4.68_327.0723m/z	327.0723214	4.682483333	neg	6-[3-(carboxymethyl)phenoxy]-3,4,5-trihydroxyoxane-2-carboxylic acid	HMDB0125161	Organic oxygen compounds	Organooxygen compounds	Carbohydrates and carbohydrate conjugates	*		43.6	20.9	M-H	C14H16O9	0.505341911	9096.403031	10128.85071	11295.44063	6536.691317	5375.021964	6642.892212	12259.28337	12634.4477	11437.33527
5.04_203.1290m/z	203.1289685	5.037216667	neg	Isovaleric acid	LMFA01020181	Lipids and lipid-like molecules	Fatty Acyls	Fatty acids and conjugates	**	C08262	43.6	20.3	2M-H	C5H10O2	0.420199189	3413.456142	3621.010655	3512.229793	3762.042242	4001.622081	4416.186827	2910.92089	3293.655662	3239.267927
0.78_369.0845m/z	369.0845068	0.778533333	neg	Ferulic acid 4-O-glucuronide	HMDB0041733	Organic oxygen compounds	Organooxygen compounds	Carbohydrates and carbohydrate conjugates	*		43.5	30.5	M-H	C16H18O10	4.827045197	70045.41385	79092.94708	77765.30049	70161.52638	144212.2451	83529.37595	80043.70837	91700.72797	104232.0975
3.11_232.9762m/z	232.9762164	3.112733333	neg	2-hydroxy-3-(sulfooxy)benzoic acid	HMDB0134105	Organic acids and derivatives	Organic sulfuric acids and derivatives	Arylsulfates	*		43.5	25.2	M-H	C7H6O7S	0.296333699	15277.281	16524.63772	17880.29536	2350.235168	2249.645331	2102.132596	9615.997219	11256.15096	11011.0929
4.02_285.0618m/z	285.061781	4.024283333	neg	Catechol glucuronide	HMDB0240490	Organic oxygen compounds	Organooxygen compounds	Carbohydrates and carbohydrate conjugates			43.5	21.8	M-H	C12H14O8	0.664354047	1383.03799	1715.135022	1505.190697	381.0668397	626.1934907	610.5720455	2229.79767	2520.64855	2484.019844
4.26_228.1241m/z	228.1241137	4.2568	neg	Methyprylon	HMDB0015239	Organoheterocyclic compounds	Piperidines	Piperidinones			43.5	20.7	M+FA-H	C10H17NO2	-0.098301032	2196.322107	2327.937224	2410.712142	1535.345714	1464.386983	1191.937505	1945.567575	2243.301274	2078.520125
4.37_402.1164n	383.0985387	4.367166667	neg	4-Hydroxy-5-(3',4'-dihydroxyphenyl)-valeric acid-O-glucuronide	HMDB0059971	Organic oxygen compounds	Organooxygen compounds	Carbohydrates and carbohydrate conjugates	*		43.5	25.3	M-H2O-H, M-H	C17H22O11	0.418417142	17998.46433	17733.85437	20428.9433	7748.009734	6231.139157	6357.852154	21561.54838	25015.61152	23663.64887
5.18_485.1668m/z	485.1668176	5.1777	neg	(S)-Multifidol 2-[apiosyl-(1->6)-glucoside]	HMDB0039930	Organic oxygen compounds	Organooxygen compounds	Carbohydrates and carbohydrate conjugates			43.5	19.7	M-H2O-H	C22H32O13	0.72914138	86859.19093	101134.122	97198.85539	98303.59634	95398.3323	95111.24426	88699.67542	93831.21227	95876.27236
5.63_137.0244m/z	137.0244421	5.628083333	neg	Ascladiol	HMDB0029610	Organoheterocyclic compounds	Dihydrofurans	Furanones	*		43.5	22.2	M-H2O-H	C7H8O4	0.156772046	5523.926528	5614.68009	6331.520216	2894.577416	3191.656866	3116.644471	4139.403875	3478.949448	3758.607077
7.65_207.1392m/z	207.1391645	7.65305	neg	Hexylbenzene	HMDB0061815	Benzenoids	Benzene and substituted derivatives	Unclassified			43.5	20.3	M+FA-H	C12H18	0.68482069	4845.622765	5345.510878	5369.195458	2873.647632	2592.999638	2928.776421	4224.433248	4441.673147	4821.864489
0.76_118.0511m/z	118.0510711	0.757316667	neg	Hydroxyethyl glycine	HMDB0061148	Organic acids and derivatives	Carboxylic acids and derivatives	Amino acids, peptides, and analogues	*		43.4	20.2	M-H	C4H9NO3	0.876593994	1840.176029	808.6224215	439.8268575	914.7801085	446.1446918	1961.834741	519.1261346	1132.381835	279.994323
2.03_433.1231m/z	433.123095	2.025516667	neg	Sulfinpyrazone sulfide	HMDB0060943	Organosulfur compounds	Thioethers	Aryl thioethers	*		43.4	29.9	M+FA-H	C23H20N2O2S	0.884269391	11995.41765	13547.09633	13668.78395	23903.75904	22302.21334	22018.1962	16270.80402	19573.90045	15446.50236
2.14_165.0418m/z	165.0417952	2.140766667	neg	1-METHYLXANTHINE	44228	Organoheterocyclic compounds	Imidazopyrimidines	Purines and purine derivatives	**	C16358	43.4	23.6	M-H	C6H6N4O2	-0.023213068	51273.24253	56680.14291	56378.56024	105240.2951	98814.04251	101463.6685	82717.5778	85830.03288	96280.85199
4.02_313.0569m/z	313.0568691	4.024283333	neg	Beta-D-Glucopyranuronic acid	HMDB0010314	Organic oxygen compounds	Organooxygen compounds	Carbohydrates and carbohydrate conjugates	*		43.4	22.4	M-H	C13H14O9	1.157434477	5489.922348	5989.442159	5593.081348	805.5105748	667.1971869	596.8966549	959.5585935	1270.261435	995.6429831
4.16_371.0985m/z	371.0985268	4.15895	neg	Melilotoside	HMDB0033581	Organic oxygen compounds	Organooxygen compounds	Carbohydrates and carbohydrate conjugates		C05158	43.4	21.5	M+FA-H	C15H18O8	0.479508861	10294.36133	10584.65005	10450.38481	15085.35651	9318.556377	10944.05927	9960.839985	7696.946214	7919.387664
4.23_341.0880m/z	341.0880324	4.234483333	neg	rhamnetin 3-rhamninoside	HMDB0304579	Lipids and lipid-like molecules	Polyketides	Flavonoids			43.4	22.2	M+FA-H	C14H16O7	0.765628886	13108.6023	13135.0936	13887.81741	5232.86263	3895.807945	4203.142491	7197.712013	7442.166445	7006.980066
4.39_371.0985m/z	371.0984531	4.3893	neg	cis-Melilotoside	HMDB0060077	Organic oxygen compounds	Organooxygen compounds	Carbohydrates and carbohydrate conjugates		C05839	43.4	19.9	M+FA-H	C15H18O8	0.253731961	29952.03792	29650.14659	29976.60138	39184.4293	35422.86258	35771.4333	27994.5305	32694.43652	30979.18366
4.84_373.1143m/z	373.1142725	4.836566667	neg	Ethylvanillin glucoside	HMDB0037682	Organic oxygen compounds	Organooxygen compounds	Carbohydrates and carbohydrate conjugates			43.4	21.4	M+FA-H	C15H20O8	0.76797363	28150.76574	28712.81898	29363.27329	38029.36112	37127.53479	37549.50987	42136.63605	47152.97892	46256.97807
5.08_445.1866m/z	445.1865898	5.080566667	neg	Estrone 3-glucuronide	43173	Lipids and lipid-like molecules	Steroids and steroid derivatives	Steroidal glycosides	**	C11133	43.4	32.2	M-H	C24H30O8	-0.451959467	11065.50404	11524.9522	10976.20878	21792.16945	19280.85213	22529.35671	13067.83461	12029.16618	12900.53736
6.83_181.0871m/z	181.0871196	6.825	neg	5-Pentyltetrahydro-2-oxo-3-furancarboxylic acid	HMDB0030989	Organoheterocyclic compounds	Lactones	Gamma butyrolactones	*		43.4	20.6	M-H2O-H	C10H16O4	0.508443598	4337.809838	4049.529272	4057.99508	2274.971953	2322.591889	2462.024483	4355.656057	3855.170252	3409.405723
6.92_374.2551m/z	374.2550693	6.915966667	neg	Undecanoylcarnitine	LMFA07070110	Lipids and lipid-like molecules	Fatty Acyls	Fatty acid esters	*		43.4	27.7	M+FA-H	C18H35NO4	0.783142972	19808.44411	19915.58061	17148.19867	19128.96133	20266.36229	18163.25986	20654.70646	20558.32185	21392.06758
7.15_222.1137m/z	222.1137061	7.147366667	neg	2,3,6,7-Tetrahydro-6,7-dimethylcyclopent[b]azepin-8(1H)-one	HMDB0039667	Organoheterocyclic compounds	Azepines	Unclassified	*		43.4	18.9	M+FA-H	C11H15NO	0.785376023	27400.59579	30539.85932	29333.86851	46838.52195	50111.9762	44188.77651	30921.9169	38818.84294	36473.00671
2.65_230.0129m/z	230.0129497	2.651566667	neg	Paracetamol sulfate	HMDB0059911	Organic acids and derivatives	Organic sulfuric acids and derivatives	Arylsulfates	*		43.3	26.9	M-H	C8H9NO5S	0.357962998	1758.008711	1862.103739	1568.357355	13338.96973	12181.99593	11992.56684	1580.213146	1845.331528	2017.541088
3.28_125.0243m/z	125.024325	3.280233333	neg	Hydroxyhydroquinone	506	Benzenoids	Phenols	Benzenetriols and derivatives	**	C02814	43.3	19.3	M-H	C6H6O3	-0.734504503	32511.66545	34085.25674	33530.01502	6850.726892	6410.937877	6382.030993	27554.4884	30834.13889	30196.47889
3.89_282.9896m/z	282.989562	3.8906	neg	Ellagic acid	HMDB0002899	Phenylpropanoids and polyketides	Tannins	Hydrolyzable tannins		C10788	43.3	23.3	M-H2O-H	C14H6O8	3.761535793	6289.417604	6699.11656	7253.180814	6258.219111	6088.05905	5769.09578	5354.541897	6089.806952	5896.073833
4.92_150.0561m/z	150.0560748	4.92485	neg	Dopamine quinone	HMDB0012219	Organic oxygen compounds	Organooxygen compounds	Carbonyl compounds	**	C17755	43.3	19.8	M-H	C8H9NO2	0.150080519	3454.738279	3991.646515	3894.482268	483.3972946	524.3959369	394.1828136	703.4317862	834.8271216	874.3388482
6.07_387.1664m/z	387.1663848	6.07	neg	Jasmolone glucoside	HMDB0032870	Organic oxygen compounds	Organooxygen compounds	Carbohydrates and carbohydrate conjugates	*		43.3	20.7	M+FA-H	C17H26O7	0.960814137	79061.75547	88657.01106	87270.13834	66602.4001	69739.84113	60199.84959	80453.7123	81370.78071	81397.51738
7.55_579.2792m/z	579.279157	7.547766667	neg	Corchoroside A	HMDB0033846	Lipids and lipid-like molecules	Steroids and steroid derivatives	Steroid lactones	*		43.3	24.9	M+FA-H	C29H42O9	-3.60998084	35382.39076	38262.85878	37889.1655	48501.58506	48737.67972	47457.55453	33179.28221	36467.90853	34859.16807
0.80_339.0739m/z	339.0738984	0.800366667	neg	Dihydrocaffeic acid 3-O-glucuronide	HMDB0041720	Organic oxygen compounds	Organooxygen compounds	Carbohydrates and carbohydrate conjugates	*		43.2	27.7	M-H2O-H	C15H18O10	4.866807701	155997.5797	159458.3169	149706.4678	120019.4989	156170.278	137180.4701	150542.1599	199145.3807	188932.3735
4.88_375.1299m/z	375.1299398	4.880083333	neg	Pseudomonine	HMDB0041438	Organic acids and derivatives	Carboxylic acids and derivatives	Amino acids, peptides, and analogues			43.2	24.8	M+FA-H	C16H18N4O4	-3.235517044	33579.84726	35828.72204	36342.30331	47795.41484	47181.02819	48039.73995	37322.577	38244.59232	38104.78491
4.92_535.1467m/z	535.146682	4.92485	neg	Isoorientin 7,3',4'-trimethyl ether	LMPK12111058	Lipids and lipid-like molecules	Polyketides	Flavonoids			43.2	20.9	M+FA-H	C24H26O11	1.973769087	28821.11286	29748.16807	28286.84629	237.8718076	407.0859623	0.001403199	2384.380317	1862.794784	3106.028343
4.95_273.1346m/z	273.1345855	4.94705	neg	9,12-dioxo-dodecanoic acid	LMFA01060092	Lipids and lipid-like molecules	Fatty Acyls	Fatty Acids and Conjugates			43.2	19.2	M+FA-H	C12H20O4	0.97959946	6655.676165	6859.187054	6793.679297	6962.69508	7072.293765	7023.32462	5285.106138	5139.837601	5038.110471
5.83_462.1774m/z	462.1773806	5.825	neg	Etodolac acyl glucuronide	HMDB0060916	Organic oxygen compounds	Organooxygen compounds	Carbohydrates and carbohydrate conjugates	*		43.2	20.4	M-H	C23H29NO9	0.918602619	12720.82959	14385.24646	13658.68398	46214.40028	38385.92616	38839.62014	39379.43154	39692.44277	42252.41364
0.88_176.0321n	351.0570953	0.884633333	neg	D-Glucurono-6,3-lactone	HMDB0006355	Organoheterocyclic compounds	Furofurans	Isosorbides	**	C02670	43.1	17.9	M-H, 2M-H	C6H8O6	-0.029206325	17198.62015	19527.29994	23357.51689	38347.52069	37661.49611	38376.71974	19460.73374	23346.89741	19742.97452
1.31_174.0773m/z	174.0772651	1.3134	neg	Vigabatrin	HMDB0015212	Organic acids and derivatives	Carboxylic acids and derivatives	Amino acids, peptides, and analogues		C07500	43.1	18.8	M+FA-H	C6H11NO2	0.648217104	3284.70013	3620.398026	3471.552713	3196.598013	2898.788587	3038.605802	3110.643023	3371.641539	3143.392398
1.80_171.0664m/z	171.0664147	1.798266667	neg	3-(3-Ethyloxiranyl)-2-propenal	HMDB0034835	Organic oxygen compounds	Organooxygen compounds	Carbonyl compounds			43.1	19.6	M+FA-H	C7H10O2	1.049328559	3531.502434	3653.947549	3394.646349	3575.938603	3863.496825	3891.500124	4490.010496	4268.989525	4252.139194
4.28_483.9522m/z	483.9522285	4.278183333	neg	Gemcitabine triphosphate	HMDB0060638	Organoheterocyclic compounds	Diazines	Pyrimidines and pyrimidine derivatives	*		43.1	20.3	M-H2O-H	C9H14F2N3O13P3	-1.360314692	46022.79301	44365.57492	44742.75271	2958.139847	2864.473639	2761.190945	40159.94225	42879.175	41655.58606
4.30_189.0769m/z	189.0769433	4.300033333	neg	5-aceto valeric acid	LMFA01060015	Lipids and lipid-like molecules	Fatty Acyls	Fatty Acids and Conjugates			43.1	18.8	M+FA-H	C7H12O3	0.667661799	957.5796293	1135.423368	1179.741251	1561.410357	1394.866467	1414.546935	1235.216172	1201.659584	1170.200978
4.30_245.0126m/z	245.0126071	4.300033333	neg	3-[4-(sulfooxy)phenyl]propanoic acid	HMDB0125171	Organic acids and derivatives	Organic sulfuric acids and derivatives	Arylsulfates	*		43.1	21.3	M-H	C9H10O6S	0.302987616	12168.31969	12196.59008	13532.64283	1973.361287	2136.973261	1476.824555	6154.28584	7235.97294	6673.728439
4.66_215.1289m/z	215.1289097	4.660483333	neg	trans-p-Menth-2-ene-1,4-diol	HMDB0037021	Lipids and lipid-like molecules	Prenol lipids	Monoterpenoids	*		43.1	19.4	M+FA-H	C10H18O2	0.158611656	4224.013482	4013.917985	4794.628022	5699.445397	5366.466754	5911.304498	3797.272153	4015.658781	3226.363059
5.73_229.0177m/z	229.017694	5.727283333	neg	Polystyrene sulfonate	HMDB0015435	Benzenoids	Benzene and substituted derivatives	Benzenesulfonic acids and derivatives			43.1	22	M+FA-H	C8H8O3S	0.412920682	8956.847925	9990.317317	9599.31411	4965.2844	5422.727831	4821.210475	9029.767832	10788.94487	11079.05594
6.30_433.2082m/z	433.2082063	6.302033333	neg	Icariside B8	HMDB0036846	Lipids and lipid-like molecules	Prenol lipids	Terpene glycosides	*		43.1	17.9	M+FA-H	C19H32O8	0.73517941	117005.4128	147837.1791	148711.5256	152153.8227	148592.3732	125597.3101	142826.3614	144658.5942	175856.4664
3.14_186.0772m/z	186.0772493	3.142483333	neg	N-(3-hydroxy-butanoyl)-homoserine lactone	LMFA08030029	Lipids and lipid-like molecules	Fatty Acyls	Fatty amides			43	19.9	M-H	C8H13NO4	0.362487089	5656.860788	5978.04405	6561.539023	3600.466996	3339.354399	3334.282193	5366.905154	5456.913846	5552.714306
6.07_165.0921m/z	165.0921068	6.07	neg	Isopropylbenzene	HMDB0034029	Benzenoids	Benzene and substituted derivatives	Cumenes		C14396	43	16.2	M+FA-H	C9H12	0.029400249	3351.541278	3842.956352	3722.0436	5937.924736	5903.152632	5293.921947	4226.874486	4411.678426	4358.498485
6.82_357.1194m/z	357.1194432	6.817666667	neg	Moringyne	HMDB0031724	Organic oxygen compounds	Organooxygen compounds	Carbohydrates and carbohydrate conjugates	*		43	18.7	M+FA-H	C15H20O7	1.080914166	38160.63302	35322.0609	35340.0882	33900.5612	33992.95912	36857.56532	33249.31854	30864.86041	26219.61356
8.27_207.1028m/z	207.1027849	8.273616667	neg	Allixin	HMDB0040705	Organoheterocyclic compounds	Pyrans	Pyranones and derivatives			43	19.7	M-H2O-H	C12H18O4	0.517067754	6339.36441	6583.094235	6505.174218	6713.784133	6687.550189	6594.902599	7147.660349	6712.423936	6988.888472
1.45_160.0372n	319.0672734	1.4461	neg	3-oxo-adipic acid	LMFA01170096	Lipids and lipid-like molecules	Fatty Acyls	Fatty Acids and Conjugates			42.9	21.5	M-H, 2M-H	C6H8O5	0.053989462	74521.13728	93153.49111	75079.63401	28384.241	23856.74659	25214.66094	32508.16352	23341.79248	27208.50009
2.36_329.0881m/z	329.0880766	2.36465	neg	Hydroxytyrosol 4'-glucuronide	HMDB0240531	Organic acids and derivatives	Organic sulfuric acids and derivatives	Arylsulfates			42.9	19.7	M-H	C14H18O9	0.820681101	10246.85623	10321.34319	10491.79219	0.001403199	0.001403199	49.13984489	8387.494213	9978.067517	10128.09443
2.62_232.9762m/z	232.9762466	2.622366667	neg	3,5-Dihydroxybenzoic acid sulfate	HMDB0240373	Organic acids and derivatives	Organic sulfuric acids and derivatives	Arylsulfates			42.9	22.5	M-H	C7H6O7S	0.425083964	10286.03329	10752.04185	12005.13772	1185.398863	1007.405669	982.1942811	5189.953074	5560.297757	5516.802731
4.28_239.9974m/z	239.997401	4.278183333	neg	2,8-Quinolinediol 8-sulfate	HMDB0240658	Organoheterocyclic compounds	Quinolines and derivatives	Quinolones and derivatives			42.9	24.4	M-H	C9H7NO5S	0.763973802	3881.582682	4392.290616	4132.419317	5579.796588	4980.134092	4724.145904	3578.004403	4025.19513	3598.340275
4.63_361.1144m/z	361.1144068	4.632666667	neg	Vanilloloside	HMDB0032013	Organic oxygen compounds	Organooxygen compounds	Carbohydrates and carbohydrate conjugates	*		42.9	18.6	M+FA-H	C14H20O8	1.22201642	7098.961585	7720.567015	6727.836093	13177.50648	13583.52637	11751.77854	11834.4416	9717.309105	11837.86504
5.29_373.1506m/z	373.1506488	5.28555	neg	Tetranor-PGEM	HMDB0253059	Lipids and lipid-like molecules	Fatty Acyls	Eicosanoids			42.9	20.6	M+FA-H	C16H24O7	0.740094437	25618.73523	23621.09537	22944.89204	25608.05567	30229.19887	32336.08135	27584.75115	22312.59578	27426.10618
5.43_144.1150n	125.0971868	5.428733333	neg	Valeraldehyde propyleneglycol acetal	HMDB0032551	Organoheterocyclic compounds	Dioxolanes	1,3-dioxolanes	*		42.9	16.2	M-H2O-H, M-H	C8H16O2	-0.012619355	3896.129583	4445.018855	4392.945025	3417.38202	3202.161275	3325.265571	3873.339179	4280.992793	3534.49499
5.76_232.0980m/z	232.0979796	5.7632	neg	2,3-Dihydro-5-(5-methyl-2-furanyl)-1H-pyrrolizine	HMDB0040012	Organoheterocyclic compounds	Pyrrolizines	Unclassified			42.9	15.7	M+FA-H	C12H13NO	0.335175953	3178.332471	3955.594295	3250.29965	2423.356264	3529.456094	2820.409385	6279.735857	7247.956198	6069.595739
6.20_319.1401m/z	319.1400524	6.195716667	neg	Octanoylglucuronide	HMDB0010347	Organic oxygen compounds	Organooxygen compounds	Carbohydrates and carbohydrate conjugates	*	C03033	42.9	20.7	M-H	C14H24O8	0.659438544	14480.36171	14833.93133	16275.79479	15457.39152	15926.56232	16170.11517	15963.23759	17315.21144	18491.88125
0.78_383.1002m/z	383.1002497	0.778533333	neg	6-[4-(4-carboxy-2-hydroxybutyl)-2-hydroxyphenoxy]-3,4,5-trihydroxyoxane-2-carl	HMDB0127743	Lipids and lipid-like molecules	Saccharolipids	Unclassified	*		42.8	29	M-H2O-H	C17H22O11	4.67363322	44597.59705	52086.32545	57298.95159	68859.50828	87181.00267	77215.80565	59948.34377	55447.75873	69350.70466
2.72_249.0630m/z	249.0630428	2.72215	neg	Inosine	HMDB0000195	Nucleosides, nucleotides, and analogues	Purine nucleosides	Unclassified	**	C00294	42.8	18.7	M-H2O-H	C10H12N4O5	0.426767563	15434.51981	16085.08155	16494.39667	16129.49784	16091.96222	16407.49782	14397.23278	16651.50413	16694.31162
4.37_355.1037m/z	355.103687	4.367166667	neg	4-(4'-O-Acetyl-alpha-L-rhamnosyloxy)benzaldehyde	HMDB0039958	Organic oxygen compounds	Organooxygen compounds	Carbohydrates and carbohydrate conjugates			42.8	21.4	M+FA-H	C15H18O7	0.745604458	5587.771635	5975.636065	6001.304732	5181.559762	4068.796778	4022.223826	7638.723489	9322.030774	7398.130105
5.04_267.1352m/z	267.1351887	5.037216667	neg	3-Hydroxymonoethylglycinexylidide	HMDB0060678	Organic acids and derivatives	Carboxylic acids and derivatives	Amino acids, peptides, and analogues	**	C16572	42.8	30.2	M+FA-H	C12H18N2O2	0.711101157	15678.92159	16586.39087	18590.59713	28302.31003	32056.98415	33086.0117	18243.64056	17045.63814	17524.13186
5.27_342.1924m/z	342.192425	5.271716667	neg	N-(3-oxododecanoyl) homoserine lactone	LMFA08030001	Lipids and lipid-like molecules	Fatty Acyls	Fatty amides			42.8	18.3	M+FA-H	C16H27NO4	0.719247263	40522.71162	30895.09597	30352.13605	23851.52035	20424.79761	21170.52503	31932.08091	25921.01788	32478.22988
5.91_401.1455m/z	401.1455389	5.908066667	neg	Zingerone glucoside	HMDB0040558	Organic oxygen compounds	Organooxygen compounds	Carbohydrates and carbohydrate conjugates			42.8	15	M+FA-H	C17H24O8	0.612882158	79617.514	91507.94495	83570.05363	33919.08364	39537.16476	35486.68566	50360.1796	60581.74371	65959.86871
1.22_188.0566m/z	188.0566282	1.224233333	neg	6-Oxopiperidine-2-carboxylic acid	HMDB0061705	Organic acids and derivatives	Carboxylic acids and derivatives	Amino acids, peptides, and analogues			42.7	16.8	M+FA-H	C6H9NO3	1.273572188	7939.835499	8675.737741	8915.181707	10826.24644	10421.26877	10744.03651	9469.378552	10674.38161	10407.02924
1.93_147.0664m/z	147.0663627	1.933583333	neg	Tetrahydro-2-methyl-3-furanol	HMDB0039799	Organoheterocyclic compounds	Tetrahydrofurans	Unclassified			42.7	14.9	M+FA-H	C5H10O2	0.786126551	6904.246521	7308.834295	5451.957113	5980.021093	6688.000942	6992.482908	6662.516901	6867.086751	6013.981754
4.70_147.0686n	192.0667419	4.703166667	neg	4-(2-Furanylmethylene)-3,4-dihydro-2H-pyrrole	HMDB0040009	Organoheterocyclic compounds	Heteroaromatic compounds	Unclassified			42.7	18.4	M-H, M+FA-H	C9H9NO	0.988173952	5656.227599	5829.457632	6227.009628	2377.180218	2449.270302	2440.365794	6151.466411	6186.267433	6017.373838
5.06_285.1346m/z	285.1345887	5.05975	neg	Imiquimod	HMDB0014862	Organoheterocyclic compounds	Quinolines and derivatives	Imidazoquinolines			42.7	22.6	M+FA-H	C14H16N4	-4.625256142	18845.65055	19816.48843	19805.71867	23441.85278	22973.04622	24919.38788	17746.646	18213.00309	19510.03622
9.32_361.1636m/z	361.1635776	9.321783333	neg	Thyrotropin releasing hormone	HMDB0060080	Organic acids and derivatives	Carboxylic acids and derivatives	Amino acids, peptides, and analogues		C03958	42.7	18.6	M-H	C16H22N6O4	1.658884751	29343.55062	27803.64623	19661.04978	26601.87082	33876.84969	28508.19436	22277.801	23028.95527	29740.88644
0.78_262.1045m/z	262.1045042	0.778533333	neg	Glutaminylalanine	HMDB0028790	Organic acids and derivatives	Carboxylic acids and derivatives	Amino acids, peptides, and analogues	*		42.6	13.4	M+FA-H	C8H15N3O4	0.2087009	19975.23654	21574.50565	25496.49731	33332.68748	34316.4667	32423.20641	36073.10287	41547.19616	39303.90981
0.82_194.9275m/z	194.9275444	0.819566667	neg	Sulfate	HMDB0001448	Homogeneous non-metal compounds	Non-metal oxoanionic compounds	Non-metal sulfates	**	C00059	42.6	17.4	2M-H	H2O4S	0.315125643	279739.9389	325077.1222	343143.9538	845031.3369	846527.6026	805964.4365	434781.2753	466594.2038	476166.9011
1.12_246.0853n	245.0780146	1.11925	neg	5,6-Dihydrouridine	HMDB0000497	Organic oxygen compounds	Organooxygen compounds	Carbohydrates and carbohydrate conjugates	*		42.6	17.9	M-H, M+FA-H	C9H14N2O6	0.426101377	19660.17881	18407.95896	18607.48818	19343.03495	17709.81345	19457.38464	18215.23221	17008.47983	17055.06282
1.36_204.9814m/z	204.9813891	1.358266667	neg	5-Sulfoxymethylfurfural	HMDB0059752	Organic oxygen compounds	Organooxygen compounds	Carbonyl compounds			42.6	23.8	M-H	C6H6O6S	0.76049953	2195.711284	2228.045187	2136.487175	3960.35631	3774.464418	3723.761216	2089.555275	1909.747876	1822.114966
3.24_315.0724m/z	315.0724136	3.23635	neg	Phenylglucuronide	HMDB0059806	Organic oxygen compounds	Organooxygen compounds	Carbohydrates and carbohydrate conjugates	*		42.6	15.7	M+FA-H	C12H14O7	0.955035924	66074.1821	64710.74644	66337.88024	4374.211931	3838.696989	3994.633575	41274.18442	46202.02032	44631.69438
5.97_292.1557m/z	292.1556926	5.965483333	neg	Ketobemidone	HMDB0041913	Organoheterocyclic compounds	Piperidines	Phenylpiperidines		C11792	42.6	15.9	M+FA-H	C15H21NO2	1.05540496	13173.40635	14238.7206	14876.82224	6990.723288	8508.980836	8215.673344	13311.23441	15646.40683	14859.40869
7.93_487.2553m/z	487.2552734	7.9268	neg	Prostaglandin G2 2-glyceryl Ester	HMDB0062591	Lipids and lipid-like molecules	Fatty Acyls	Eicosanoids	*		42.6	17.5	M+FA-H	C23H38O8	0.909718875	42515.08864	41068.5867	43581.70396	54199.70867	52602.07426	55238.21848	40904.37818	52265.76871	46634.81794
2.19_296.1002m/z	296.1001808	2.194566667	neg	Cordycepin	HMDB0250451	Unclassified	Unclassified	Unclassified			42.5	17.5	M+FA-H	C10H13N5O3	0.552018874	12346.67131	10890.89006	11858.02311	14198.83554	13280.70928	13592.05207	10809.60581	12342.5803	11874.16008
2.36_117.0558m/z	117.0557621	2.36465	neg	5-hydroxy valeric acid	LMFA01050010	Lipids and lipid-like molecules	Fatty Acyls	Fatty Acids and Conjugates			42.5	14.4	M-H	C5H10O3	0.375457352	19675.52066	19461.83959	21512.66058	33137.30578	33150.52009	31505.49787	25613.33167	28322.65648	27854.02745
4.28_205.0119m/z	205.0119264	4.278183333	neg	Cysteinyl-Cysteine	HMDB0028772	Organic acids and derivatives	Carboxylic acids and derivatives	Amino acids, peptides, and analogues	*		42.5	28.7	M-H2O-H	C6H12N2O3S2	3.719662984	2335.675412	2643.912436	2411.8774	4349.751594	4261.112912	4182.290988	2666.629677	2874.175436	2645.892463
4.79_360.1422n	359.1350743	4.792316667	neg	Deoxyloganic acid	HMDB0037028	Lipids and lipid-like molecules	Prenol lipids	Terpene glycosides	*	C11647	42.5	16.8	M-H2O-H, M-H	C16H24O9	0.553677727	132597.5635	141584.2565	144005.592	117216.087	116063.9426	112659.0458	140074.5114	154166.9639	147884.4895
5.47_153.0922m/z	153.0921822	5.471616667	neg	6-Pentyl-1,4-dioxan-2-one	HMDB0059937	Organoheterocyclic compounds	Dioxanes	1,4-dioxanes			42.5	16.5	M-H2O-H	C9H16O3	0.458810976	2825.113269	2479.001278	2257.103221	2794.603549	2981.041376	3062.860784	2875.559108	2366.519413	2690.523829
7.51_345.1557m/z	345.1556943	7.50615	neg	Nepetaside	HMDB0038149	Lipids and lipid-like molecules	Prenol lipids	Terpene glycosides	*		42.5	16.4	M-H	C16H26O8	0.58619493	182872.03	188365.7688	198906.4095	223328.0233	238419.49	211227.6791	177700.5168	198797.1872	201806.4932
8.34_359.1714m/z	359.1714462	8.338466667	neg	(-)-trans-Carveol glucoside	HMDB0029848	Lipids and lipid-like molecules	Prenol lipids	Terpene glycosides	*		42.5	16	M+FA-H	C16H26O6	0.970179707	27957.93995	30059.72115	29991.23009	35814.3944	34229.51141	34315.29116	20164.46675	22738.35396	22654.43124
0.82_673.2310m/z	673.2310151	0.819566667	neg	3'-N-Acetylneuraminyl-N-acetyllactosamine	58470	Unclassified	Unclassified	Unclassified			42.4	21.3	M-H	C25H42N2O19	0.169710148	33266.08892	33076.60461	41729.54675	37308.18176	34077.03553	37432.75468	33502.76356	38065.3385	34010.01609
1.16_405.0289m/z	405.0288658	1.160166667	neg	Quercetin 7,3',4'-trimethyl ether 3-sulfate	LMPK12112657	Lipids and lipid-like molecules	Polyketides	Flavonoids			42.4	25.3	M-H2O-H	C18H16O10S	0.682100254	49474.34646	50836.65271	65716.24507	80460.97669	79263.76411	80191.34706	61811.08401	71640.34766	74696.17272
1.70_142.0510m/z	142.051015	1.6992	neg	4,5-Dimethyloxazole	HMDB0032970	Organoheterocyclic compounds	Azoles	Oxazoles			42.4	14.7	M+FA-H	C5H7NO	0.497601923	2317.060896	2482.449123	2519.05911	3250.140811	3066.210171	2769.125633	3087.259396	2799.141954	3354.723684
4.28_308.0778m/z	308.0777995	4.278183333	neg	Indoxyl glucuronide	HMDB0010319	Lipids and lipid-like molecules	Steroids and steroid derivatives	Steroidal glycosides	*		42.4	16.8	M-H	C14H15NO7	0.725150798	7009.674329	8455.690915	7832.430569	341.8934958	311.5014482	398.7211762	6729.597247	6748.572245	6083.12903
4.52_367.1037m/z	367.1037047	4.521733333	neg	3,4-Dihydroxyphenylvaleric acid 3 glucuronide	HMDB0240445	Unclassified	Unclassified	Unclassified			42.4	18.9	M-H2O-H	C17H22O10	0.644617981	4030.921122	4156.290635	4518.814329	1385.857394	1575.676704	1149.453554	6411.230466	6933.824817	6955.019251

5.41_609.2533m/z	609.2532805	5.406433333	neg	14-chlorotetracosane-1,15-disulfate	LMFA00000023	Lipids and lipid-like molecules	Fatty Acyls	Other Fatty Acyls			42.4	45.9	M+FA-H	C24H49ClO8S2	-1.170768235	40361.15835	40299.15172	44400.59263	43593.93767	49304.95685	46188.52195	45185.85076	47274.46258	43511.14985
5.50_201.0229m/z	201.0228669	5.497816667	neg	4-Ethylphenylsulfate	HMDB0062551	Organic acids and derivatives	Organic sulfuric acids and derivatives	Arylsulfates	*		42.4	19.5	M-H	C8H10O4S	0.809419451	5063.36604	4110.505681	4310.031468	3312.070292	3762.417419	3693.015636	6997.554301	6411.343847	7062.895688
5.70_273.1709m/z	273.1709165	5.701283333	neg	12-keto tridecanoic acid	LMFA01060043	Lipids and lipid-like molecules	Fatty Acyls	Fatty Acids and Conjugates			42.4	14.6	M+FA-H	C13H24O3	0.740731462	16781.81714	17182.92397	15427.69492	11881.3987	14670.22445	13835.30544	10411.75294	11947.98792	11934.03549
4.28_420.1255m/z	420.1255247	4.278183333	neg	loganate	HMDB0304409	Unclassified	Unclassified	Unclassified			42.3	23.1	M+FA-H	C16H23O10-	-4.798517258	6029.475702	5541.797339	6241.96887	3147.334727	3242.446102	3342.225808	5425.961074	5854.244431	5160.277616
4.61_180.0668m/z	180.0667939	4.611883333	neg	L-Threo-3-Phenylserine	HMDB0002184	Organic acids and derivatives	Carboxylic acids and derivatives	Amino acids, peptides, and analogues	*		42.3	13.8	M-H	C9H11NO3	0.978341391	1924.541552	1879.110663	1816.560526	786.5007724	759.7356307	746.7609881	1619.151821	1472.737842	1934.770099
5.70_453.1750m/z	453.1749532	5.701283333	neg	3-O-Methylniveusin A	HMDB0039084	Lipids and lipid-like molecules	Prenol lipids	Terpene lactones	*		42.3	21.9	M+FA-H	C21H28O8	-4.085196494	37032.05459	35218.95569	36445.89748	38967.87712	40258.70641	38695.434	42587.11557	42123.91322	45446.62593
6.48_433.2082m/z	433.2082471	6.477433333	neg	Dihydroroseoside	HMDB0040614	Lipids and lipid-like molecules	Fatty Acyls	Fatty acyl glycosides	*		42.3	16.5	M+FA-H	C19H32O8	0.840469991	34125.14379	32898.61684	38496.40728	44753.41968	38548.172	39643.16799	64523.86266	66740.40551	66065.64269
6.71_415.1978m/z	415.1978424	6.70695	neg	(3S,7E,9S)-9-Hydroxy-4,7-megastigmadien-3-one 9-glucoside	HMDB0036822	Lipids and lipid-like molecules	Fatty Acyls	Fatty acyl glycosides	*		42.3	17.1	M+FA-H	C19H30O7	1.313380673	55027.31277	58600.06225	56972.21753	61414.70447	61581.22127	57836.70841	46817.2741	55899.31415	52381.52116
8.00_408.2877n	453.2861554	7.99505	neg	Butaprost	HMDB0249457	Unclassified	Unclassified	Unclassified			42.3	14.8	M-H, M+FA-H	C24H40O5	0.383553051	42394.37765	40487.63221	46888.51389	7561.056966	6946.960641	7847.51129	17407.53071	17857.54762	19202.60092
1.42_324.0728m/z	324.0727564	1.421683333	neg	HHPAA glucuronide	HMDB0240379	Organic oxygen compounds	Organooxygen compounds	Carbohydrates and carbohydrate conjugates			42.2	16.5	M-H2O-H	C14H17NO9	0.776321604	21123.18577	18561.76554	14158.98845	1574.526652	1442.715401	1353.695108	15175.89154	11700.34359	14264.39444
13.84_109.0043m/z	109.0043141	13.83571667	neg	Barbituric acid	HMDB0041833	Organoheterocyclic compounds	Diazines	Pyrimidines and pyrimidine derivatives	**	C00813	42.2	13.9	M-H2O-H	C4H4N2O3	-0.28762832	161073.4604	156990.4895	166432.2638	146010.5411	157251.4346	150691.6718	189304.2896	161067.8134	165875.7649
2.84_117.0558m/z	117.0557692	2.83525	neg	Tetrahydrofuran	HMDB0000246	Organoheterocyclic compounds	Tetrahydrofurans	Unclassified	*		42.2	12.7	M+FA-H	C4H8O	0.714480183	5126.250191	5014.978765	5474.931392	7696.931983	7919.724043	7483.403345	6317.365401	6169.877127	6204.218143
4.57_107.0503m/z	107.05028	4.5668	neg	3-Ethyl-1,2-cyclopentanedione	HMDB0031222	Organic oxygen compounds	Organooxygen compounds	Carbonyl compounds	*		42.2	13.8	M-H2O-H	C7H10O2	0.330012069	422.2914967	434.3478659	483.0228654	179.4966013	179.5911956	168.3685001	290.0088519	322.4539491	301.1067858
5.68_433.1143m/z	433.1142939	5.678766667	neg	4'-Hydroxyfenoprofen glucuronide	HMDB0060772	Benzenoids	Benzene and substituted derivatives	Diphenylethers	*		42.2	15	M-H	C21H22O10	0.62983243	16344.65599	15547.83102	16385.33371	68.50550084	0.001403199	178.6428663	0.001403199	71.68621826	287.8957569
6.97_225.1134m/z	225.1134187	6.970716667	neg	Bovolide	HMDB0039159	Organoheterocyclic compounds	Dihydrofurans	Furanones			42.2	13.8	M+FA-H	C11H16O2	1.032860359	16201.44518	15433.06377	18832.1818	15490.44552	13227.9646	14767.55742	12687.6796	15863.11994	15730.4478
8.11_413.2184m/z	413.218392	8.110466667	neg	11-Dehydro-thromboxane B2	HMDB0004242	Lipids and lipid-like molecules	Fatty Acyls	Eicosanoids	**	C05964	42.2	14.7	M+FA-H	C20H32O6	0.815913983	60020.52322	62514.67696	63796.34759	29287.83578	27608.51913	28364.84221	43413.28523	50678.48698	47498.24992
0.78_355.1051m/z	355.1051011	0.778533333	neg	1-O-2'-Hydroxy-4'-methoxycinnamoyl-b-D-glucose	HMDB0040866	Phenylpropanoids and polyketides	Cinnamic acids and derivatives	Hydroxycinnamic acids and derivatives			42.1	25.7	M-H	C16H20O9	4.620167155	23916.69299	22295.55203	26774.3363	15055.31524	20750.19157	19760.31724	26271.56972	27208.31388	37213.22477
1.16_571.0403m/z	571.0403213	1.160166667	neg	Genistein 4'-glucuronide-7-sulfate	HMDB0240520	Phenylpropanoids and polyketides	Isoflavonoids	Isoflavonoid O-glycosides			42.1	25.2	M+FA-H	C21H18O14S	0.745541475	15962.67191	17445.45709	22486.95035	17224.6276	20152.67526	18641.97165	18797.00075	15077.35402	18120.52685
1.29_249.0077m/z	249.0076528	1.290916667	neg	3-Methylcatechol 1-sulfate	HMDB0240662	Organic acids and derivatives	Organic sulfuric acids and derivatives	Arylsulfates			42.1	15.8	M+FA-H	C7H8O5S	1.007687467	8862.642531	9755.621695	9845.457954	8642.866797	7924.697962	7997.130476	7374.091839	8377.551562	8279.923964
3.89_383.0986m/z	383.0986316	3.8906	neg	3-O-p-Coumaroylquinic acid	HMDB0029681	Organic oxygen compounds	Organooxygen compounds	Alcohols and polyols	*		42.1	14.4	M+FA-H	C16H18O8	0.772541947	3646.459359	4109.736561	4332.668562	2691.297872	2521.21865	2469.934021	14007.8502	14590.72925	14172.4295
4.00_357.0831m/z	357.0830704	4.002233333	neg	Homovanillic acid 4-glucuronide	HMDB0240526	Organic oxygen compounds	Organooxygen compounds	Carbohydrates and carbohydrate conjugates			42.1	20.8	M-H	C15H18O10	0.977540672	4852.348539	4670.109938	5580.338365	4871.978427	4399.609598	4477.990655	3469.644335	4039.15055	3904.89373
4.34_357.1194m/z	357.1193808	4.344733333	neg	3-Hydroxychavicol 1-glucoside	HMDB0034861	Organic oxygen compounds	Organooxygen compounds	Carbohydrates and carbohydrate conjugates			42.1	19.4	M+FA-H	C15H20O7	0.880832456	12989.03356	14552.7859	15094.1426	9020.588923	8462.096759	8182.900662	13662.19315	16056.61351	15404.53417
5.01_239.0904m/z	239.0903607	5.014716667	neg	CMPF	45041	Lipids and lipid-like molecules	Fatty Acyls	Fatty acids and conjugates			42.1	26.4	M-H	C12H16O5	-8.898346113	3399.137034	3629.315053	3879.737176	4485.82014	4341.81506	4843.372436	4304.974105	3694.429072	3598.105038
0.86_209.0668m/z	209.0667556	0.86335	neg	2-O-Methyl-D-xylose	HMDB0033821	Organic oxygen compounds	Organooxygen compounds	Carbohydrates and carbohydrate conjugates			42	15.5	M+FA-H	C6H12O5	0.483248781	10465.83746	9537.018093	11342.6081	10498.28095	9889.708041	10248.86321	10399.0404	10687.68396	9508.13706
3.64_188.9864m/z	188.9864165	3.640916667	neg	Pyrocatechol sulfate	HMDB0059724	Organic acids and derivatives	Organic sulfuric acids and derivatives	Arylsulfates	*		42	22.6	M-H	C6H6O5S	0.519382246	87391.97302	96068.65088	94776.39771	33507.91593	33817.76484	28679.39001	60256.23607	64928.98564	72714.09942
5.16_266.1399m/z	266.1399314	5.155116667	neg	N,N,O-Tridesmethyltramadol	HMDB0060850	Benzenoids	Benzene and substituted derivatives	Cyclohexylphenols	*		42	11.4	M+FA-H	C13H19NO2	0.676805414	9166.201654	11009.40062	9673.681397	6261.321666	5764.580225	6562.106131	7198.948064	7143.693283	7922.985108
3.96_121.0295m/z	121.0295429	3.957583333	neg	3-(2-Furanyl)-2-propenal	HMDB0032918	Organoheterocyclic compounds	Heteroaromatic compounds	Unclassified			41.9	13.2	M-H	C7H6O2	0.326774107	4597.847023	4852.981539	5340.121612	1797.596307	1665.935385	1814.025092	3603.352692	3521.362826	3622.599276
4.00_175.0976m/z	175.097614	4.002233333	neg	Heptanoic acid	HMDB0000666	Lipids and lipid-like molecules	Fatty Acyls	Fatty acids and conjugates	*	C17714	41.9	10.8	M+FA-H	C7H14O2	0.241454775	5050.812603	5407.429941	5630.482235	4906.331183	5005.992657	4750.692205	4725.748287	5190.856112	5271.042175
4.81_345.1557m/z	345.1556917	4.814166667	neg	(1S,3R,4R)-8,10-Dihydroxyfenchone 10-O-b-D-glucoside	HMDB0033223	Lipids and lipid-like molecules	Prenol lipids	Terpene glycosides	*		41.9	14.9	M-H	C16H26O8	0.578889247	46009.72199	52556.60884	51954.73281	28763.50113	27226.15819	26827.96557	51579.0782	56483.14154	56561.46947
5.06_453.1762m/z	453.1761997	5.05975	neg	Deoxycytidine	HMDB0000014	Nucleosides, nucleotides, and analogues	Pyrimidine nucleosides	Pyrimidine 2'-deoxyribonucleosides	**	C00881	41.9	23	2M-H	C9H13N3O4	4.985492041	3751.988331	3962.987821	4319.39374	12260.90151	10431.7508	11165.68742	3341.04828	3190.784341	3882.923724
5.41_431.1924m/z	431.1924188	5.406433333	neg	Citroside A	HMDB0030370	Lipids and lipid-like molecules	Prenol lipids	Terpene glycosides	*		41.9	13.8	M+FA-H	C19H30O8	0.383335587	64174.2033	64987.28308	63358.92343	65899.79505	74901.68142	68812.75705	63922.00808	63590.94282	63122.18437
5.61_309.0983m/z	309.0983124	5.610733333	neg	(E)-2-O-Cinnamoyl-beta-D-glucopyranose	HMDB0035880	Organic oxygen compounds	Organooxygen compounds	Carbohydrates and carbohydrate conjugates			41.9	14.4	M-H	C15H18O7	1.083119582	26869.42899	26274.34422	30194.43826	12552.36808	12421.57032	11763.23497	23551.92605	21928.18733	20711.49838
5.81_165.0921m/z	165.0921099	5.806016667	neg	Propylbenzene	HMDB0059877	Benzenoids	Benzene and substituted derivatives	Phenylpropanes			41.9	13.1	M+FA-H	C9H12	0.05509029	2439.285881	2623.273803	2517.113706	5475.037474	5093.965977	5164.242161	3555.643862	3767.201199	3552.806082
6.43_314.1413m/z	314.1413499	6.433933333	neg	SENECIPHYLLINE	HMDB0258231	Unclassified	Unclassified	Unclassified			41.9	17.7	M-H2O-H	C18H23NO5	4.706900387	5212.75071	4737.944763	5109.898384	7882.821673	7414.636279	8200.524963	6136.971929	6501.622662	6106.828462
6.88_343.1400m/z	343.1400282	6.8759	neg	8-Epiiridotrial glucoside	LMPR0102070023	Lipids and lipid-like molecules	Prenol lipids	Isoprenoids		C11652	41.9	13.4	M-H	C16H24O8	0.543036563	333146.3451	346509.9672	319476.359	357474.4456	371919.5782	348734.5373	304837.5928	336269.8029	298161.6569
4.05_137.0608m/z	137.0608268	4.046666667	neg	2-(4-Hydroxyphenyl)ethanol	7044	Benzenoids	Phenols	Tyrosols and derivatives	**	C06044	41.8	12.8	M-H	C8H10O2	0.171093572	532.1230892	601.7177007	528.0154953	371.5935124	360.6639458	357.4867542	610.2170957	640.1087268	637.2989745
4.09_374.0851n	373.0778327	4.091783333	neg	Ethyl gallate 4-glucuronide	HMDB0166847	Organic oxygen compounds	Organooxygen compounds	Carbohydrates and carbohydrate conjugates			41.8	13.7	M-H2O-H, M-H	C15H18O11	0.44921781	7470.332604	7850.382985	7856.953628	6445.633778	5605.213367	5831.121715	5488.41089	6377.202923	5955.139923
5.13_363.1665m/z	363.1664627	5.125666667	neg	1,2,10-Trihydroxydihydro-trans-linalyl oxide 7-O-beta-D-glucopyranoside	HMDB0033237	Organic oxygen compounds	Organooxygen compounds	Carbohydrates and carbohydrate conjugates	*		41.8	15	M-H2O-H	C16H30O10	1.064174983	37848.18951	36444.81129	33224.14544	32968.05375	33526.62102	35960.6607	46363.88046	37335.14132	44238.32562
7.42_183.1027m/z	183.1027098	7.41815	neg	(1'R)-Nepetalic acid	HMDB0036117	Lipids and lipid-like molecules	Prenol lipids	Monoterpenoids	*		41.8	12.2	M-H	C10H16O3	0.227457652	6340.541677	6322.81074	6804.990029	4368.958264	4212.551149	3887.728208	7626.338233	8084.941542	8308.900798
0.82_308.0988m/z	308.0987789	0.819566667	neg	N-Acetyl-a-neuraminic acid	HMDB0000773	Organic oxygen compounds	Organooxygen compounds	Carbohydrates and carbohydrate conjugates	*	C19909	41.7	12.2	M-H	C11H19NO9	0.239948971	65701.64371	68098.39513	69331.90944	61398.07397	59278.55524	58698.3484	60898.08891	65590.56101	66659.29849
2.07_149.0469m/z	149.0468747	2.070366667	neg	1-Methylhypoxanthine	HMDB0013141	Organoheterocyclic compounds	Imidazopyrimidines	Purines and purine derivatives	*		41.7	11.9	M-H	C6H6N4O	-0.064886822	2078.058628	2435.081614	2271.225844	3551.344274	3799.756282	3809.064132	2580.866859	2498.142942	2469.089192
3.42_211.0838m/z	211.0837862	3.417666667	neg	3-(Methylthio)-1-propanol	HMDB0031716	Organosulfur compounds	Thioethers	Dialkylthioethers	*		41.7	16.9	2M-H	C4H10OS	2.78528988	6983.220283	7362.930634	6511.963354	11947.79872	12985.29273	11497.70766	8944.36512	9952.920839	8717.490818
4.66_537.1982m/z	537.1982176	4.660483333	neg	Diosbulbinoside F	HMDB0036783	Lipids and lipid-like molecules	Prenol lipids	Terpene glycosides	*		41.7	14.1	M-H	C26H34O12	0.868708208	16115.96022	16550.20291	17207.386	16476.91283	16778.06452	18722.7212	6128.120338	6403.909342	5426.455938
7.16_242.1764m/z	242.1763781	7.1647	neg	N-Undecanoylglycine	HMDB0013286	Organic acids and derivatives	Carboxylic acids and derivatives	Amino acids, peptides, and analogues	*		41.7	12.2	M-H	C13H25NO3	0.866975737	18293.50347	20106.35559	17226.33558	21534.82673	20560.5986	20033.51889	19224.86594	21169.73019	19998.81941
7.59_417.2134m/z	417.2133881	7.58625	neg	9-Hydroxy-7-megastigmen-3-one glucoside	HMDB0040701	Lipids and lipid-like molecules	Fatty Acyls	Fatty acyl glycosides	*		41.7	13.5	M+FA-H	C19H32O7	1.025979545	56791.97821	61764.13328	61518.94915	77330.69636	75657.73665	75593.26901	57809.21231	64442.92271	62588.79412
4.02_129.0558m/z	129.0558024	4.024283333	neg	Adipate semialdehyde	HMDB0012882	Lipids and lipid-like molecules	Fatty Acyls	Fatty acids and conjugates	**	C06102	41.6	10.5	M-H	C6H10O3	0.650696722	4860.814752	5360.720479	5584.041743	8226.498545	7707.045605	7224.19738	6974.691284	7171.706877	7251.491527
4.70_390.0762m/z	390.0762415	4.703166667	neg	Thiamine monophosphate	HMDB0002666	Organoheterocyclic compounds	Diazines	Pyrimidines and pyrimidine derivatives	**	C01081	41.6	15.8	M+FA-H	C12H18N4O4PS+	-1.736130721	14497.65379	13774.37215	12983.53375	15698.38704	16562.88965	16024.93893	17584.48875	14162.92388	16151.23534
4.88_383.1347m/z	383.1347473	4.880083333	neg	15-Acetyl-4-deoxynivalenol	HMDB0033444	Nucleosides, nucleotides, and analogues	Purine nucleosides	Purine 2'-deoxyribonucleosides	*		41.6	11.7	M+FA-H	C17H22O7	-0.025493613	28250.31728	28917.72014	29018.61823	46618.89888	47172.52547	46706.29955	40548.88307	39107.71616	38979.92189
5.01_233.0433m/z	233.0433167	5.014716667	neg	Methionyl-Cysteine	HMDB0028970	Organic acids and derivatives	Carboxylic acids and derivatives	Amino acids, peptides, and analogues	*		41.6	22.8	M-H2O-H	C8H16N2O3S2	3.663569771	8927.122348	9555.051287	10008.20647	11048.51207	11299.32859	12322.1071	11495.91531	9825.032096	10123.09696
5.73_292.1557m/z	292.1556848	5.727283333	neg	Meperidine	HMDB0014597	Organoheterocyclic compounds	Piperidines	Phenylpiperidines		C07128	41.6	13	M+FA-H	C15H21NO2	1.023863025	5550.385773	5534.555068	4800.807519	2490.577381	3274.245494	3146.962139	4275.699883	5351.256588	5965.552241
6.37_527.2139m/z	527.213894	6.373833333	neg	16,17-Dihydro-16alpha,17-dihydroxygibberellin A4 17-glucoside	HMDB0040647	Lipids and lipid-like molecules	Prenol lipids	Terpene glycosides	*		41.6	14.1	M-H	C25H36O12	0.934852167	29790.72409	33252.43533	36436.41007	29950.91931	32718.71844	31424.34349	32068.51874	32489.17316	30560.25007
2.25_191.0563m/z	191.0562653	2.248516667	neg	Solerol	HMDB0002173	Organoheterocyclic compounds	Lactones	Unclassified			41.5	11.4	M+FA-H	C6H10O4	1.051669699	5758.992814	6460.074772	6543.688739	6205.154542	5749.443448	6109.064559	2140.451735	2309.001318	2130.683697
3.73_305.0339m/z	305.0338993	3.730516667	neg	4-Hydroxy-5-(3',5'-dihydroxyphenyl)-valeric acid sulfate	HMDB0240457	Unclassified	Unclassified	Unclassified			41.5	23	M-H	C11H14O8S	0.775376136	4564.845005	3986.786529	4170.737535	6643.196041	5901.664903	4196.750812	12376.91049	13192.13116	13327.32992
5.10_265.0717m/z	265.0716825	5.1031	neg	Hispolone	HMDB0041442	Benzenoids	Phenols	Benzenediols			41.5	10.3	M+FA-H	C12H12O4	-0.359854753	6255.786637	6688.44892	6584.977504	7723.401216	7200.190035	8281.265034	2770.045933	2554.233063	2297.991032
5.76_439.1611m/z	439.1610953	5.7632	neg	Gibberellin A39	HMDB0035045	Lipids and lipid-like molecules	Prenol lipids	Diterpenoids	*		41.5	11.1	M+FA-H	C20H26O8	0.316271523	14634.18193	16351.07513	14224.34211	19331.97533	25957.25155	19915.79669	22269.34286	23140.6825	19547.6114
5.81_245.1395m/z	245.139509	5.806016667	neg	3-Hydroxy-2,8-dimethyl-1,7-dioxaspiro[5.5]undecane	LMPK09000029	Unclassified	Unclassified	Unclassified			41.5	10.8	M+FA-H	C11H20O3	0.307995175	21289.54547	22068.83142	21733.21422	22496.44858	20607.71446	21083.67163	22522.11672	23786.74167	23287.24014
6.17_339.1088m/z	339.1088487	6.171316667	neg	Sweroside	HMDB0258643	Unclassified	Unclassified	Unclassified			41.5	12.5	M-H2O-H	C16H22O9	0.858652626	43771.77347	45391.43178	46362.95174	18795.81192	19234.78972	21816.08447	34066.45474	39646.67238	40328.33078
7.96_394.2722n	393.2649601	7.961816667	neg	Norcholic acid	LMST04060019	Lipids and lipid-like molecules	Sterol Lipids	Bile acids and derivatives			41.5	13.5	M-H, M+FA-H	C23H38O5	0.791827172	24563.49217	23712.4654	26309.34109	19219.76836	17258.17161	18578.42996	19114.51035	21332.72517	20364.49025
4.64_242.0131m/z	242.0131076	4.641416667	neg	2-Methoxyacetaminophen sulfate	HMDB0062550	Organic acids and derivatives	Organic sulfuric acids and derivatives	Arylsulfates	*		41.4	16.5	M-H2O-H	C9H11NO6S	0.921853138	11657.89014	10893.21997	10379.76198	17882.21987	17159.64585	16692.4964	14822.54568	14356.37556	13667.93338
4.79_126.0680n	107.0501371	4.792316667	neg	Enol-3-Ethyl-1,2-cyclopentanedione	HMDB0031223	Organic oxygen compounds	Organooxygen compounds	Carbonyl compounds	*		41.4	9.53	M-H2O-H, M-H	C7H10O2	-0.804073099	1772.078253	1842.110729	1883.375382	1439.458912	1511.163107	1589.508843	1355.055745	1603.366631	1385.605814
7.18_451.0708m/z	451.0708426	7.178516667	neg	Torvanol A	HMDB0038789	Phenylpropanoids and polyketides	Isoflavonoids	O-methylated isoflavonoids			41.4	15.8	M-H	C20H20O10S	0.887349418	191958.222	210840.1604	208819.9765	180685.3689	190417.4951	176851.6003	135218.3669	161394.6441	145022.9489
0.86_166.0180m/z	166.0179588	0.86335	neg	N-Acetyltaurine	HMDB0240253	Organic acids and derivatives	Organic sulfonic acids and derivatives	Organosulfonic acids and derivatives			41.3	15.6	M-H	C4H9NO4S	0.038855312	19528.97918	21222.58523	23145.5953	25516.61794	25041.84793	24777.02431	19946.61122	21376.96667	21315.18373
10.82_239.0675m/z	239.0675245	10.82006667	neg	2'-O-Methyluridine	HMDB0240328	Nucleosides, nucleotides, and analogues	Pyrimidine nucleosides	Unclassified			41.3	10.2	M-H2O-H	C10H14N2O6	0.695112736	42036.79365	41722.21119	40774.20148	42927.84921	42366.84743	42223.36367	46191.30196	43534.48694	45683.69193
3.91_170.1188m/z	170.1187848	3.912566667	neg	Gabapentin	HMDB0005015	Organic acids and derivatives	Carboxylic acids and derivatives	Amino acids, peptides, and analogues			41.3	10.8	M-H	C9H17NO2	0.773741803	1335.649554	1367.75099	1437.77622	742.8296761	876.9474407	772.3493821	1147.482105	1282.649158	1419.833982
4.57_371.1350m/z	371.1350346	4.5668	neg	Citrusin C	HMDB0038708	Organic oxygen compounds	Organooxygen compounds	Carbohydrates and carbohydrate conjugates			41.3	10.7	M+FA-H	C16H22O7	0.854497971	14848.54435	16553.91938	15772.85746	6958.438923	6629.96812	5555.000803	12867.08348	13655.3579	14559.80286
5.04_288.0916m/z	288.0915721	5.037216667	neg	N-(3-oxo-heptanoyl)-homoserine thiolactone	LMFA08020238	Lipids and lipid-like molecules	Fatty Acyls	Fatty amides			41.3	15.8	M+FA-H	C11H17NO3S	1.870838716	1316.882015	1529.910947	1312.367262	3030.072651	2997.693274	3412.691636	1891.022498	1850.794181	1987.026879
5.55_271.1165m/z	271.1164762	5.547916667	neg	Decarbamoylneosaxitoxin	HMDB0033663	Phenylpropanoids and polyketides	Saxitoxins, gonyautoxins, and derivatives	Unclassified			41.3	11.1	M-H	C9H16N6O4	1.652302657	19576.90942	18535.78568	18358.80282	15325.79625	21190.96323	15670.74497	16116.616	16707.04846	15895.24047
5.70_411.2027m/z	411.2026857	5.701283333	neg	20-hydroxy-Resolvin E1	LMFA03140008	Lipids and lipid-like molecules	Fatty Acyls	Eicosanoids			41.3	23.4	M+FA-H	C20H30O6	0.666802595	67451.27801	65480.67709	63555.44785	40826.77461	44883.59663	44447.8895	71466.75837	71123.11045	86855.8773
6.15_465.1016m/z	465.1016351	6.154666667	neg	(-)-Epicatechin 7-O-glucuronide	HMDB0041729	Phenylpropanoids and polyketides	Flavonoids	Flavonoid glycosides	*		41.3	15.8	M-H	C21H22O12	-4.751225796	31591.75678	35157.64088	35810.86535	20508.26732	22420.79805	24644.78742	20022.2727	23697.79578	25727.89216
15.05_297.0472m/z	297.0472199	15.05453333	neg	Phoxim	HMDB0256498	Unclassified	Unclassified	Unclassified			41.2	15.2	M-H	C12H15N2O3PS	1.329646191	35493.40935	66955.41757	19644.43285	27796.90249	14668.38899	9484.450894	52285.74717	9506.220147	11036.56797
2.38_188.9864m/z	188.9864277	2.380333333	neg	Hydroquinone sulfate	HMDB0240263	Organic acids and derivatives	Organic sulfuric acids and derivatives	Arylsulfates			41.2	12.8	M-H	C6H6O5S	0.578086937	27344.10544	26967.31617	29903.77464	4032.741007	3556.071781	3601.825134	82478.25717	90176.76184	87557.64713
2.72_161.0820m/z	161.0820176	2.72215	neg	4-hydroxy caproaldehyde	LMFA06000013	Lipids and lipid-like molecules	Fatty Acyls	Fatty aldehydes			41.2	7.62	M+FA-H	C6H12O2	0.732745881	6581.719613	6621.470902	7111.431458	8067.116711	8155.025508	8332.173223	6852.955728	7740.571003	7740.054778
4.52_341.0791m/z	341.07907	4.521733333	neg	Phenytoin methylcatechol	HMDB0060867	Organoheterocyclic compounds	Azolidines	Imidazolidines	*		41.2	13.2	M+FA-H	C16H12N2O4	3.918851387	3561.620908	3877.477426	3921.644678	3993.505084	3990.356705	3267.470283	4186.296205	3982.475202	4925.824871
4.63_242.1400m/z	242.1400208	4.632666667	neg	N-(3-hydroxy-octanoyl)-homoserine lactone	LMFA08030036	Lipids and lipid-like molecules	Fatty Acyls	Fatty amides			41.2	11.2	M-H	C12H21NO4	0.983114143	9645.302646	8724.11739	7918.092363	7332.43962	6946.982744	5886.13238	9223.087538	7098.092032	9022.223611
6.28_429.1765m/z	429.1764965	6.2811	neg	22-dimethylarsinoyl-(5Z,8Z, 11Z,14Z,17Z,20Z)-docosahexaenoic acid	LMFA00000029	Lipids and lipid-like molecules	Fatty Acyls	Other Fatty Acyls			41.2	19.1	M-H2O-H	C24H37AsO3	-3.411337967	21637.68466	23389.796	25792.46609	26293.15044	26731.69147	25041.03307	19449.30202	20770.75831	23239.25471
6.44_343.1400m/z	343.1400462	6.442716667	neg	Iridotrial glucoside	LMPR0102070024	Lipids and lipid-like molecules	Prenol lipids	Isoprenoids		C11653	41.2	10.4	M-H	C16H24O8	0.595496285	130613.2184	137200.5615	144042.7652	159997.0071	151995.9718	155376.5145	144081.8904	148796.609	146712.6478
3.58_308.0778m/z	308.0777967	3.5753	neg	Blepharin	HMDB0029344	Organic oxygen compounds	Organooxygen compounds	Carbohydrates and carbohydrate conjugates	*		41.1	11.3	M-H2O-H	C14H17NO8	0.676655129	9722.075541	10791.19964	9486.217425	466.2656609	412.9161686	509.141812	9265.566296	10128.12007	9291.814284
5.30_175.0976m/z	175.0976358	5.302883333	neg	Hexyl formate	HMDB0032874	Organic acids and derivatives	Carboxylic acids and derivatives	Carboxylic acid derivatives			41.1	6.94	M+FA-H	C7H14O2	0.408991858	2466.938625	2232.836262	2378.232275	1448.111396	2307.404209	2314.016773	3328.652833	2304.780022	3243.553816
5.38_277.1657m/z	277.1656584	5.384216667	neg	(x)-2-Heptanol glucoside	HMDB0035028	Lipids and lipid-like molecules	Fatty Acyls	Fatty acyl glycosides	*		41.1	9.04	M-H	C13H26O6	-0.013273844	5683.893541	6804.027857	7299.114866	5585.109632	6111.604851	5642.221322	7191.510241	8305.750445	7231.331367
6.16_341.1244m/z	341.1243912	6.161866667	neg	Citrusin D	HMDB0039334	Lipids and lipid-like molecules	Fatty Acyls	Fatty acyl glycosides	*		41.1	9.6	M-H	C16H22O8	0.584625911	211837.7696	224089.175	237397.7879	196646.4246	208900.3906	202141.1942	194451.4179	209968.0417	216068.8147
7.47_437.1432m/z	437.1431934	7.473983333	neg	Sec-o-Glucosylhamaudol	HMDB0258199	Unclassified	Unclassified	Unclassified			41.1	18.6	M-H	C21H26O10	-4.854859237	51122.03972	55166.51026	58002.62164	77563.46196	75675.47238	75301.80669	39506.31441	43267.55642	41214.64146
1.20_133.0507m/z	133.0507097	1.202333333	neg	Acetoin	HMDB0003243	Organic oxygen compounds	Organooxygen compounds	Carbonyl compounds		C00466	41	7.1	M+FA-H	C4H8O2	0.878533416	7345.889837	8285.640161	8727.919657	7821.772104	7505.807935	8002.929934	6850.864378	7116.952172	7770.78944
3.73_366.0564n	365.0491325	3.730516667	neg	5-formamido-1-(5-phospho-D-ribosyl)imidazole-4-carboxamide(2-)	HMDB0062708	Nucleosides, nucleotides, and analogues	Imidazole ribonucleosides and ribonucleotides	1-ribosyl-imidazolecarboxamides			41	10.4	M-H, M+FA-H	C10H15N4O9P	-3.430513917	29903.49167	27357.14737	29774.9671	7827.888168	8291.790964	6709.855147	20066.02392	21517.40771	21214.9184
3.77_329.0290m/z	329.0289839	3.770416667	neg	Inosinic acid	HMDB0000175	Nucleosides, nucleotides, and analogues	Purine nucleotides	Purine ribonucleotides	**	C00130	41	11.2	M-H2O-H	C10H13N4O8P	-0.789994818	3557.497735	3127.691479	3298.500851	6577.545166	6266.83274	4036.718001	3841.896148	4047.278812	4072.320255
3.85_291.1088m/z	291.1087842	3.84605	neg	Pantoyllactone glucoside	HMDB0041269	Lipids and lipid-like molecules	Fatty Acyls	Fatty acyl glycosides	*		41	12	M-H	C12H20O8	0.831925465	2347.978782	2952.65836	3389.652455	663.4280625	772.6827855	632.0246158	1653.637252	1746.62431	1780.120493
4.41_134.0612m/z	134.0611855	4.41115	neg	5-(2-Furanyl)-3,4-dihydro-2H-pyrrole	HMDB0040013	Organoheterocyclic compounds	Heteroaromatic compounds	Unclassified			41	6.74	M-H	C8H9NO	0.355184571	14408.65623	15727.5017	14615.92931	6475.32978	6318.930529	5575.255326	10933.93081	12793.80809	12437.93952
7.35_470.0887n	451.0708166	7.346316667	neg	(E)-Resveratrol 3-glucoside 4'-sulfate	HMDB0037073	Phenylpropanoids and polyketides	Stilbenes	Stilbene glycosides	*		41	14	M-H2O-H, M-H	C20H22O11S	0.798250293	290085.9584	301788.3393	284068.5967	222328.6944	230584.3731	219611.5043	191803.2408	210629.211	223259.7396
0.78_397.0795m/z	397.0795311	0.778533333	neg	Decarbamoylgonyautoxin III	HMDB0040137	Phenylpropanoids and polyketides	Saxitoxins, gonyautoxins, and derivatives	Unclassified			40.9	20.6	M+FA-H	C9H16N6O7S	3.437738754	26720.20881	31269.10593	21283.65504	53174.32463	75693.55467	60855.01222	32166.44807	43368.13922	38994.27824
1.14_183.0524m/z	183.0524454	1.1382	neg	8-Hydroxypurine	HMDB0012182	Organoheterocyclic compounds	Imidazopyrimidines	Purines and purine derivatives	*		40.9	8.18	M+FA-H	C5H6N4O	0.591780135	1882.646985	1304.994713	1184.580968	2830.219667	2547.920657	3130.2506	1718.279516	2160.578486	2211.866322
4.73_121.0295m/z	121.0295363	4.725416667	neg	Ethyl maltol	HMDB0031735	Organoheterocyclic compounds	Pyrans	Pyranones and derivatives	*	C20362	40.9	10.9	M-H2O-H	C7H8O3	0.238022725	4900.15871	4550.597935	4578.481824	5278.898216	5671.368302	5328.451213	5945.014452	5012.455144	5192.826818
4.75_337.0885m/z	337.0884884	4.747466667	neg	Edetic Acid	HMDB0015109	Organic acids and derivatives	Carboxylic acids and derivatives	Tetracarboxylic acids and derivatives		C00284	40.9	8.39	M+FA-H	C10H16N2O8	-1.30073343	51125.01213	54688.17488	54025.73125	50926.79524	51057.85241	48015.5198	51141.28561	52852.77826	56488.75099
5.38_387.1664m/z	387.166379	5.384216667	neg	7-Epi-12-hydroxyjasmonic acid glucoside	HMDB0040706	Lipids and lipid-like molecules	Fatty Acyls	Fatty acyl glycosides	*		40.9	10.4	M-H	C18H28O9	0.832002307	63554.28328	63182.33922	63358.36597	73484.68134	79331.17421	79348.29836	58637.3958	57248.99336	59593.29221
7.28_415.1979m/z	415.1978655	7.2779	neg	5-Megastigmen-7-yne-3,9-diol 3-glucoside	HMDB0032842	Organic oxygen compounds	Organooxygen compounds	Carbohydrates and carbohydrate conjugates	*		40.9	11.1	M+FA-H	C19H30O7	1.37577903	71034.63278	72971.68313	70944.39434	89407.51926	87013.37349	83949.26282	59652.27588	66832.46243	65611.78548
8.84_395.2077m/z	395.2077268	8.8368	neg	Andrographolide	HMDB0248395	Unclassified	Unclassified	Unclassified			40.9	8.45	M+FA-H	C20H30O5	0.570588215	147191.7964	153802.3639	151696.0084	66255.37591	63355.77078	66579.74914	101438.7769	111175.9764	108849.3819
1.25_319.0673m/z	319.0673493	1.246333333	neg	2-Methyl-4-oxopentanedioic acid	HMDB0039447	Organic acids and derivatives	Keto acids and derivatives	Gamma-keto acids and derivatives			40.8	6.45	2M-H	C6H8O5	0.871832052	42905.28633	48646.45175	46283.78085	19370.08972	19015.0937	19477.574	18847.14674	19864.38907	20452.2037
1.36_328.0456m/z	328.0455949	1.358266667	neg	3'-AMP	HMDB0003540	Nucleosides, nucleotides, and analogues	Ribonucleoside 3'-phosphates	Unclassified	**	C01367	40.8	9.32	M-H2O-H	C10H14N5O7P	1.013080499	15093.20939	14177.0196	15144.96998	15321.41805	15564.74129	14791.98963	15033.42348	15288.98686	15357.79323
1.52_197.0569m/z	197.0569484	1.515416667	neg	Succinimide	HMDB0240653	Organoheterocyclic compounds	Pyrrolidines	Pyrrolidones		C07273	40.8	7.72	2M-H	C4H5NO2	0.84815632	1270.29879	1365.143081	1537.380221	4413.060309	3961.328189	4011.849386	1720.964458	1779.284299	1638.995481
1.56_137.0357m/z	137.0356724	1.56225	neg	Nicotinamide N-oxide	HMDB0002730	Organoheterocyclic compounds	Pyridines and derivatives	Pyridinecarboxylic acids and derivatives	*		40.8	7.57	M-H	C6H6N2O2	0.154933826	2523.557831	2003.914124	1989.785577	2313.591704	2121.238651	2154.268432	1830.393937	2438.871306	1980.74098
4.64_259.1294m/z	259.1293711	4.641416667	neg	Dethiobiotin	HMDB0003581	Lipids and lipid-like molecules	Fatty Acyls	Fatty acids and conjugates	**	C01909	40.8	8.73	M+FA-H	C10H18N2O3	-2.681823254	2712.439779	2970.984066	3467.466578	0.001403199	0.001403199	0.001403199	230.7111714	56.22399398	150.0426252
4.88_459.1513m/z	459.1512798	4.880083333	neg	Ptelatoside A	HMDB0032600	Organic oxygen compounds	Organooxygen compounds	Carbohydrates and carbohydrate conjugates			40.8	10.2	M+FA-H	C19H26O10	1.158769021	12113.72111	13228.36068	13067.72965	13295.54423	12476.22955	12055.89286	11757.65598	13599.7928	12963.48623
5.71_675.2252m/z	675.2251923	5.709933333	neg	3,5,7-Trihydroxy-6-prenylflavone	LMPK12111640	Unclassified	Unclassified	Unclassified			40.8	15.9	2M-H	C20H18O5	2.397671166	34450.89223	33726.02666	31191.47862	31895.17632	43035.68411	38819.98627	30953.3773	41768.08095	47152.94834
7.65_487.2553m/z	487.2553253	7.6458	neg	4-Megastigmene-6a,9R-diol 9-[apiosyl-(1->6)-glucoside]	HMDB0041580	Lipids and lipid-like molecules	Fatty Acyls	Fatty acyl glycosides	*		40.8	10.4	M-H2O-H	C24H42O11	0.897277327	41798.33131	44906.20509	46821.35757	42970.55279	40652.55623	44999.46622	34846.45312	37247.01265	36669.14501
13.21_293.1794m/z	293.1793958	13.21166667	neg	Sodium Tetradecyl Sulfate	HMDB0014607	Organic acids and derivatives	Organic sulfuric acids and derivatives	Sulfuric acid esters			40.7	14.8	M-H	C14H30O4S	0.651817981	116793.1686	72778.25303	110897.972	62620.10171	77002.90526	58920.23611	82458.54265	96917.06087	69627.98449
2.26_402.9955m/z	402.9954979	2.26065	neg	Uridine diphosphate (UDP)	97	Unclassified	Unclassified	Unclassified			40.7	7.81	M-H	C9H14N2O12P2	1.428624513	104671.9808	108667.6122	111374.9868	12251.9983	11488.0168	12215.59167	83749.44398	90528.20762	85364.47982
3.00_279.0987m/z	279.0987252	3.004583333	neg	S-4-Hydroxymephenytoin	HMDB0013894	Organoheterocyclic compounds	Azolidines	Imidazolidines	*		40.7	6.52	M+FA-H	C12H14N2O3	0.341912858	14309.33497	13545.46074	14595.24991	25586.97184	26098.37942	25078.26859	20395.04877	21236.99526	20138.99882
4.28_129.0558m/z	129.0557966	4.278183333	neg	4-penten-2-one	LMFA12000034	Organic oxygen compounds	Organooxygen compounds	Carbonyl compounds	*		40.7	6.32	M+FA-H	C5H8O	0.937540116	2051.661412	2231.737062	2298.406213	2941.295391	2898.878201	2620.644962	2589.502003	2837.31403	2581.680008
7.18_213.1498m/z	213.1497897	7.178516667	neg	10-Undecenal	HMDB0031128	Organic oxygen compounds	Organooxygen compounds	Carbonyl compounds	*		40.7	7.53	M+FA-H	C11H20O	1.020485183	5271.999753	5582.902743	5417.647379	7895.979586	8048.300508	7997.928889	5325.160241	6153.016932	5359.535757
7.47_369.1557m/z	369.1556656	7.473983333	neg	Blumealactone C	HMDB0035357	Lipids and lipid-like molecules	Prenol lipids	Terpene lactones	*		40.7	6.8	M+FA-H	C17H24O6	0.537543887	163074.5472	175324.7673	180738.6269	234991.0835	227522.7352	234086.3795	121540.1093	136134.0407	131262.9889
1.16_381.0302m/z	381.0301769	1.160166667	neg	Hesperetin 5-sulfate	HMDB0240524	Unclassified	Unclassified	Unclassified			40.6	18.7	M-H	C16H14O9S	4.188866067	105806.0866	103354.897	141638.1393	118617.7869	137590.8887	132762.6988	117560.5321	113598.5891	147002.97
3.80_154.0147m/z	154.0146509	3.801716667	neg	4-Nitrocatechol	58136	Benzenoids	Phenols	Nitrophenols	*	C02235	40.6	8.82	M-H	C6H5NO4	0.449401024	953.328911	898.8860208	918.4160999	466.1031656	434.7938082	405.9701681	317.8382435	339.2836431	371.0409417

4.86_507.1874m/z	507.1873764	4.858383333	neg	13-Hydroxy-5'-O-methylmelledonal	HMDB0035864	Lipids and lipid-like molecules	Prenol lipids	Sesquiterpenoids	*		40.6	9.88	M+FA-H	C24H30O9	0.413302039	6965.276113	7888.173135	8246.13802	496.3915187	331.8233637	353.8553989	5775.864628	6360.53757	5676.89924
5.10_425.1453m/z	425.1452574	5.1031	neg	Methyl helianthenoate A glucoside	HMDB0040899	Lipids and lipid-like molecules	Fatty Acyls	Fatty acyl glycosides	*		40.6	8.75	M+FA-H	C19H24O8	-0.166322545	8715.786603	8776.539322	8488.913773	12330.5461	13564.0182	16430.93702	12160.04891	9963.23048	10329.17975
5.43_267.0383m/z	267.0382732	5.428733333	neg	Glycineamideribotide	HMDB0002022	Nucleosides, nucleotides, and analogues	Glycinamide ribonucleotides	Unclassified	**	C03838	40.6	7.65	M-H2O-H	C7H15N2O8P	-1.704943983	8245.678583	8633.93894	8108.234126	5856.922769	5627.985527	5296.435266	7380.924318	7712.160289	5967.598708
6.48_469.2084m/z	469.2083603	6.477433333	neg	8beta-Angeloyloxy-15-hydroxy-1alpha,10R-dimethoxy-3-oxo-11(13)-germacren-	HMDB0039013	Lipids and lipid-like molecules	Prenol lipids	Terpene lactones	*		40.6	9.19	M+FA-H	C22H32O8	1.035971178	22761.92873	22574.90334	26835.91153	8354.972445	7225.321371	7547.945018	20225.10393	21105.0638	21334.57764
6.84_435.2239m/z	435.2238604	6.842166667	neg	7,8-Dihydro-3b,6a-dihydroxy-alpha-ionol 9-glucoside	HMDB0041578	Lipids and lipid-like molecules	Fatty Acyls	Fatty acyl glycosides	*		40.6	4.68	M+FA-H	C19H34O8	0.741860159	104791.4925	106411.2586	104247.5791	125670.165	127892.9097	124328.9607	127664.3178	128379.3136	112992.1627
7.09_367.1402m/z	367.140208	7.086816667	neg	2-Hydroxyphenylacetic acid glucuronide	HMDB0240440	Unclassified	Unclassified	Unclassified			40.6	7.21	M+FA-H	C17H22O6	1.138188607	228040.8822	229748.906	226723.2324	320624.5545	320974.2633	300983.4703	197895.9135	225369.658	210060.5044
7.20_408.2879n	407.2805783	7.201166667	neg	1b,3a,12a-Trihydroxy-5b-cholanoic acid	HMDB0000326	Lipids and lipid-like molecules	Steroids and steroid derivatives	Bile acids, alcohols and derivatives	*		40.6	6.77	M-H, M+FA-H	C24H40O5	0.68668437	142276.911	159866.3355	157947.2431	118069.4594	115930.7084	121947.4188	93447.81275	107734.7762	94083.40198
7.26_461.2397m/z	461.2396521	7.262266667	neg	3-O-(alpha-L-rhamnopyranosyl-(1-2)-alpha-L-rhamnopyranosyl)-3-hydroxydecar	LMFA13030006	Lipids and lipid-like molecules	Fatty Acyls	Fatty acyl glycosides			40.6	24.4	M-H2O-H	C22H40O11	0.897684603	96587.10531	110529.9762	104853.8734	121209.8793	118122.3848	116511.4754	125841.77	136296.5961	149020.6581
1.90_263.0232m/z	263.0232041	1.899016667	neg	3-Methoxy-4-Hydroxyphenylglycol sulfate	HMDB0003332	Benzenoids	Phenols	Methoxyphenols	*		40.5	11	M-H	C9H12O7S	0.404582278	8118.282674	8350.755186	8089.45284	8902.663409	9008.772104	8385.290978	8497.48641	8920.119933	7874.322418
4.39_549.1971m/z	549.197051	4.3893	neg	Tracheloside	HMDB0030557	Lignans, neolignans and related compounds	Lignan glycosides	Unclassified	*		40.5	6.34	M-H	C27H34O12	-1.270532078	152789.4832	181775.3797	174237.0602	76206.53988	71445.88563	71186.47028	175099.9005	207070.4876	204564.3582
14.04_136.9992m/z	136.9992255	14.04215	neg	Orotic acid	HMDB0000226	Organoheterocyclic compounds	Diazines	Pyrimidines and pyrimidine derivatives	**	C00295	40.4	6.78	M-H2O-H	C5H4N2O4	-0.256394702	59618.80551	58226.03178	64017.53525	53050.35803	57705.22202	56464.23452	73169.40122	61493.21664	62743.10078
14.31_441.2031m/z	441.2031155	14.30818333	neg	Carbofuran	HMDB0031770	Organoheterocyclic compounds	Coumarans	Unclassified	*	C14291	40.4	10.7	2M-H	C12H15NO3	0.011753551	34904.49233	35940.74059	33443.8229	24245.92403	33458.08244	37715.23563	41511.07205	36640.82243	35803.38998
2.07_327.0948m/z	327.094755	2.070366667	neg	meta-O-Dealkylated flecainide lactam	HMDB0060832	Benzenoids	Benzene and substituted derivatives	Benzoic acids and derivatives	*		40.4	13	M-H2O-H	C15H17F3N2O4	-4.176705947	23857.1372	27031.87963	25875.82275	40662.63849	43553.38157	43722.21631	29529.13278	27017.82238	28246.18757
3.98_415.1249m/z	415.1249287	3.97985	neg	4-Hydroxy-5-(phenyl)-valeric acid-O-glucuronide	HMDB0059980	Organic oxygen compounds	Organooxygen compounds	Carbohydrates and carbohydrate conjugates	*		40.4	9.49	M+FA-H	C17H22O9	0.928325255	2210.18051	2419.406517	2893.526248	2228.798173	2105.827196	2060.163991	5035.448799	5410.802629	5187.085308
4.39_467.0662m/z	467.0661547	4.3893	neg	Acetyl citrate	HMDB0059808	Organic acids and derivatives	Carboxylic acids and derivatives	Tetracarboxylic acids and derivatives			40.4	13.9	2M-H	C8H10O8	-3.639294184	85463.50531	85750.99582	81496.09507	79559.3636	76497.83936	78458.55773	100503.1391	93534.64407	97657.16826
6.33_369.1744m/z	369.1743914	6.331216667	neg	5alpha-androstane-3alpha-ol-17-one sulfate	LMST05020001	Lipids and lipid-like molecules	Steroids and steroid derivatives	Sulfated steroids	*		40.4	7.82	M-H	C19H30O5S	0.736979431	73859.185	88859.17754	85564.96188	105280.3625	101474.3337	93444.67746	75854.81307	76942.54442	83721.62125
6.60_220.0980m/z	220.098016	6.6024	neg	Metaxalone	HMDB0014798	Benzenoids	Phenol ethers	Unclassified		C07934	40.4	6.69	M-H	C12H15NO3	0.448131114	5857.816831	5943.425375	5665.873999	9292.196193	9531.054424	9688.779155	6470.838815	7791.305815	7601.500451
6.73_329.1608m/z	329.1608135	6.72945	neg	(1S,2S,4S)-1,8-Epoxy-p-menthane-2,7-diol 2-O-b-D-glucoside	HMDB0034786	Organic oxygen compounds	Organooxygen compounds	Carbohydrates and carbohydrate conjugates	*		40.4	6.98	M-H2O-H	C16H28O8	0.679974122	34485.53941	37253.14932	37459.59669	23679.4934	22898.92205	19938.90177	34599.08302	41760.9266	40855.81935
8.40_495.2967m/z	495.2967177	8.40065	neg	Pregnanediol 3-O-glucuronide	HMDB0010318	Lipids and lipid-like molecules	Steroids and steroid derivatives	Steroidal glycosides	**	C03033	40.4	4.8	M-H	C27H44O8	0.757192334	78176.31582	79367.60255	80155.09141	108196.4594	112734.3236	106199.3676	72932.86794	85836.23672	84082.43094
1.72_238.9956m/z	238.9955584	1.721216667	neg	alpha-D-glucose 6-phosphate	HMDB0304534	Organic oxygen compounds	Organooxygen compounds	Carbohydrates and carbohydrate conjugates	**	C00092	40.3	7.96	M-H2O-H	C6H11O9P-2	-2.593058102	5240.505347	5510.859135	5100.213138	5661.438582	5795.216647	5437.961663	5781.4999	5488.79349	6886.247081
4.73_127.0765m/z	127.0765023	4.725416667	neg	epsilon-heptenoic acid	LMFA01030016	Lipids and lipid-like molecules	Fatty Acyls	Fatty Acids and Conjugates			40.3	4.51	M-H	C7H12O2	0.383153245	2512.952067	2630.92424	2659.231308	3440.078578	3354.254478	3215.983868	2488.816648	2626.521557	2357.661976
5.08_175.0977m/z	175.0976771	5.080566667	neg	2-Methylhexanoic acid	HMDB0031594	Lipids and lipid-like molecules	Fatty Acyls	Fatty acids and conjugates	*		40.3	3.17	M+FA-H	C7H14O2	0.726560934	2351.380216	2271.839055	2281.534294	2149.748679	1719.087906	1919.507865	2569.930703	2182.515759	2182.848988
5.65_469.1330m/z	469.1330134	5.650833333	neg	Plumieride	HMDB0256647	Unclassified	Unclassified	Unclassified			40.3	13.8	M-H	C21H26O12	-4.544250278	31122.18257	30969.6964	36994.49317	11321.84955	10133.03921	10061.28185	26264.99111	21201.49558	26651.4261
6.49_329.1608m/z	329.1608451	6.494283333	neg	cis-10-Hydroxylinalyl oxide 7-glucoside	HMDB0033236	Organic oxygen compounds	Organooxygen compounds	Carbohydrates and carbohydrate conjugates	*		40.3	7.13	M-H2O-H	C16H28O8	0.770636168	55438.96754	56977.65201	62001.8357	25809.5416	24053.92892	24916.94777	56406.35053	58140.45236	60760.33874
6.56_457.1696m/z	457.1696226	6.55905	neg	Fluocinolone	HMDB0252347	Unclassified	Unclassified	Unclassified			40.3	10.2	M+FA-H	C21H26F2O6	4.063269062	22875.15904	23918.09116	24575.01424	27998.21626	30048.65226	30156.04573	17813.02754	21826.79659	19593.6285
7.53_443.1562m/z	443.1562409	7.527033333	neg	Methyl (3x,10R)-dihydroxy-11-dodecene-6,8-diynoate 10-glucoside	HMDB0040896	Lipids and lipid-like molecules	Fatty Acyls	Fatty acyl glycosides	*		40.3	3.89	M+FA-H	C19H26O9	0.893083828	78145.1302	79621.20551	84054.84337	139390.1366	131935.5983	126063.6985	99395.15379	112676.4238	114198.3488
8.38_193.0870m/z	193.0870358	8.383816667	neg	Cuminaldehyde	HMDB0002214	Lipids and lipid-like molecules	Prenol lipids	Monoterpenoids	**	C06577	40.3	2.54	M+FA-H	C10H12O	0.120947475	10441.02493	9172.212644	8821.475345	9229.370141	10280.0743	9631.536862	9918.316388	9578.532041	9629.167648
0.82_396.1147m/z	396.1147208	0.819566667	neg	N-Acetyl-9-O-acetylneuraminic acid	HMDB0000794	Organic oxygen compounds	Organooxygen compounds	Carbohydrates and carbohydrate conjugates	*		40.2	6.88	M+FA-H	C13H21NO10	-0.079725332	19386.09033	19708.44865	21436.97762	16200.44808	16005.88992	14659.53975	20592.37828	20805.7634	19333.76817
11.58_250.1450m/z	250.1449638	11.58276667	neg	Tetranor-5-NO2-CLA	LMFA01120010	Lipids and lipid-like molecules	Fatty Acyls	Unclassified			40.2	5.44	M-H2O-H	C14H23NO4	0.359440051	60931.82356	53395.00897	59532.49456	61164.36133	62569.42981	61135.25665	65929.87059	51729.29395	62603.93671
4.66_281.1008m/z	281.1007905	4.660483333	neg	S-aminomethyldihydrolipoamide	HMDB0006239	Lipids and lipid-like molecules	Fatty Acyls	Fatty amides	*		40.2	14.4	M+FA-H	C9H20N2OS2	3.737179769	13652.32522	13660.92352	14421.87795	31303.65749	28213.8804	32435.02499	18823.80219	20435.30544	15132.62157
5.20_383.1714m/z	383.1713924	5.20005	neg	Pinolidoxin	HMDB0301836	Unclassified	Unclassified	Unclassified			40.2	7.18	M+FA-H	C18H26O6	0.742076388	17305.22944	17279.11873	17572.95407	10460.8125	11017.22394	11694.24307	18967.57964	17301.90056	18246.48089
5.22_141.0922m/z	141.0922023	5.222166667	neg	(R)-2-Hydroxycaprylic acid	HMDB0002264	Lipids and lipid-like molecules	Fatty Acyls	Fatty acids and conjugates	*		40.2	4.58	M-H2O-H	C8H16O3	0.618791207	4119.307721	4334.793398	4100.268298	4504.382271	4408.585784	4376.988586	3232.273523	3319.020759	3324.252901
5.36_413.1433m/z	413.1432861	5.361783333	neg	Benzyl gentiobioside	HMDB0041515	Organic oxygen compounds	Organooxygen compounds	Carbohydrates and carbohydrate conjugates			40.2	12.4	M-H2O-H	C19H28O11	-4.707711314	95017.37593	98853.77391	99200.06583	35553.88052	40694.29716	40599.71547	96020.87314	88505.99214	102922.7729
5.43_141.0921m/z	141.0921479	5.428733333	neg	Hydroxyoctanoic acid	HMDB0000711	Lipids and lipid-like molecules	Fatty Acyls	Fatty acids and conjugates	*		40.2	5.92	M-H2O-H	C8H16O3	0.278774121	860.9458247	1016.739514	1039.254799	1773.057456	1808.97707	1921.824841	1454.357288	1536.239564	1217.9283
5.56_677.2407m/z	677.2407245	5.56185	neg	Mulberrofuran E	HMDB0041423	Phenylpropanoids and polyketides	2-arylbenzofuran flavonoids	Unclassified			40.2	15.2	M+FA-H	C39H36O8	2.378120688	68290.71826	56480.4766	58092.27557	81073.35162	98455.37673	83277.3762	60676.82136	63304.59604	47885.79889
6.07_327.1452m/z	327.1451753	6.07	neg	Cynaratriol	HMDB0034983	Lipids and lipid-like molecules	Prenol lipids	Terpene lactones	*		40.2	4.73	M+FA-H	C15H22O5	0.881289759	40268.03852	45436.71874	47054.24434	17896.18655	19694.33618	17930.73109	40384.97716	42976.26496	43520.98045
1.49_229.0331m/z	229.0330795	1.492933333	neg	Dibenzothiophene	HMDB0251165	Unclassified	Unclassified	Unclassified			40.1	13.5	M+FA-H	C12H8S	1.115942448	3072.348021	3184.965111	3445.6552	3286.398281	3251.959655	2934.679177	3210.773861	2774.834211	3237.414181
12.26_311.1689m/z	311.1688794	12.26416667	neg	N-Undecylbenzenesulfonic acid	HMDB0032549	Benzenoids	Benzene and substituted derivatives	Benzenesulfonic acids and derivatives			40.1	8.4	M-H	C17H28O3S	0.769119081	379643.2787	276614.1179	273349.109	236644.1985	253461.324	245741.244	315804.1203	442473.3337	284613.4634
3.98_144.0455m/z	144.0455264	3.97985	neg	4-(3-Pyridyl)-3-butenoic acid	HMDB0001424	Organoheterocyclic compounds	Pyridines and derivatives	Unclassified	*		40.1	3.48	M-H2O-H	C9H9NO2	0.23894215	4291.32976	4650.224275	4830.974158	5996.045795	5751.36504	6064.35838	5174.579556	5653.638037	5505.186649
4.90_255.1352m/z	255.1351718	4.902316667	neg	L,L-Cyclo(leucylprolyl)	HMDB0034276	Organic acids and derivatives	Carboxylic acids and derivatives	Amino acids, peptides, and analogues			40.1	5.92	M+FA-H	C11H18N2O2	0.671478487	27557.93883	29858.30963	31185.00795	48276.09766	46461.74219	48844.34174	31975.24945	35193.17089	32845.46431
5.13_431.1541m/z	431.1540628	5.125666667	neg	Methylsyringin	HMDB0039570	Organic oxygen compounds	Organooxygen compounds	Carbohydrates and carbohydrate conjugates			40.1	12.4	M+FA-H	C18H26O9	-4.71939887	10228.9792	9635.66985	9947.764735	8585.737342	8869.763123	9321.292336	12960.09954	9906.727824	11831.22501
5.65_349.1506m/z	349.1506209	5.650833333	neg	(2S)-2-Butanol O-[b-D-Apiofuranosyl-(1->6)-b-D-glucopyranoside]	HMDB0041255	Organic oxygen compounds	Organooxygen compounds	Carbohydrates and carbohydrate conjugates			40.1	5.7	M-H2O-H	C15H28O10	0.583640595	10254.29033	9893.767847	12032.67886	15264.1802	13833.40303	13880.1825	10089.76107	7704.842071	9450.697443
6.27_373.1507m/z	373.1507294	6.272366667	neg	3-Hydroxy-4-isopropylbenzyl alcohol 3-glucoside	HMDB0039385	Lipids and lipid-like molecules	Prenol lipids	Terpene glycosides	*		40.1	6	M+FA-H	C16H24O7	0.985437508	12063.71662	10648.17709	13173.96425	5350.384199	5526.407863	5799.05806	10334.41936	10674.96663	12370.09891
6.36_407.1927m/z	407.1927438	6.3617	neg	2-hydroxy-7-methyl-Octanedioic acid	LMFA01170136	Lipids and lipid-like molecules	Fatty Acyls	Fatty Acids and Conjugates			40.1	10.4	2M-H	C9H16O5	1.158889066	8750.834143	10963.15882	11578.17279	14350.05121	15696.00977	15195.49165	11898.57491	10807.71344	14382.94529
6.57_169.1235m/z	169.1234883	6.570233333	neg	2-Hexyl-1,3-dioxolane-4-methanol	HMDB0039669	Organoheterocyclic compounds	Dioxolanes	1,3-dioxolanes	*		40.1	4.99	M-H2O-H	C10H20O3	0.451509629	2908.061782	3294.857297	3429.835265	2727.350539	2664.618175	2702.503771	1860.585403	2215.304346	2006.64945
9.75_195.1391m/z	195.1391481	9.75225	neg	Dihydro-5-(2-octenyl)-2(3H)-furanone	HMDB0030867	Organoheterocyclic compounds	Lactones	Gamma butyrolactones	*		40.1	4.24	M-H	C12H20O2	0.482460017	5085.748885	4644.561116	4519.829204	4576.155648	4558.322297	5029.188252	5607.277036	5282.420375	5039.145826
0.76_236.0778m/z	236.0777548	0.757316667	neg	2-amino-2,3,7-trideoxy-D-lyxo-hept-6-ulosonic acid	LMFA01050473	Lipids and lipid-like molecules	Fatty Acyls	Fatty Acids and Conjugates			40	4.81	M+FA-H	C7H13NO5	0.938981358	3141.620706	3252.737737	3457.23861	1594.103219	3054.376736	1993.000948	2645.238924	3355.560027	4251.748096
0.84_227.1038m/z	227.1038117	0.841733333	neg	Valylglutamic acid	HMDB0029126	Organic acids and derivatives	Carboxylic acids and derivatives	Amino acids, peptides, and analogues	*		40	8.97	M-H2O-H	C10H18N2O5	0.329524706	2726.853508	3375.822876	3066.246848	2578.599449	2706.115136	3030.488811	2926.35921	2915.518659	3455.535579
1.38_312.0953m/z	312.0953491	1.380283333	neg	Vidarabine	HMDB0014340	Nucleosides, nucleotides, and analogues	Purine nucleosides	Unclassified		C07195	40	17.4	M+FA-H	C10H13N5O4	1.468905024	5696.148624	5542.462068	5578.659625	7116.049861	6540.094249	6243.279493	6193.223757	6465.344964	5507.918913
4.16_407.1496m/z	407.1495855	4.15895	neg	Odyssic acid	LMFA01031114	Lipids and lipid-like molecules	Fatty Acyls	Fatty Acids and Conjugates			40	3.93	2M-H	C12H12O3	-1.045153319	2650.855608	2978.154831	3202.03847	4914.037464	3258.908189	3298.24098	3800.54174	3135.429398	3172.721379
4.54_295.1301m/z	295.1300954	4.54425	neg	Heptabarbital	HMDB0015443	Organoheterocyclic compounds	Diazines	Pyrimidines and pyrimidine derivatives		C17725	40	3.08	M+FA-H	C13H18N2O3	0.600138212	3564.597689	3771.274523	4308.793246	2448.135938	2550.810078	2457.748778	1957.601295	2267.511422	2409.220616
7.56_461.2397m/z	461.2396829	7.556516667	neg	xi-Linalool 3-[rhamnosyl-(1->6)-glucoside]	HMDB0030422	Lipids and lipid-like molecules	Fatty Acyls	Fatty acyl glycosides	*		40	5.16	M-H	C22H38O10	0.999288284	46147.37962	50501.54088	48630.47652	58540.10595	52687.9846	55704.23226	66962.13579	71378.1778	67068.05665
9.02_475.2680m/z	475.267994	9.01635	neg	Lucidenic acid G	HMDB0035599	Lipids and lipid-like molecules	Prenol lipids	Triterpenoids	*		40	9.26	M-H	C27H40O7	-4.478882489	134418.5851	168877.0444	164247.8026	14898.91263	15054.732	15326.50433	66348.88903	74109.08292	71320.58501
9.74_411.2027m/z	411.2027451	9.7401	neg	11-dehydro-TXB3	LMFA03030009	Lipids and lipid-like molecules	Fatty Acyls	Eicosanoids			40	3.74	M+FA-H	C20H30O6	0.828972474	55762.54668	57374.72866	58491.86256	73540.66995	72398.22882	80032.26155	49104.20041	54742.21441	51747.37638
1.29_275.0710m/z	275.0710284	1.290916667	neg	gamma-L-Glutamyl-L-methionine sulfoxide	HMDB0038613	Organic acids and derivatives	Carboxylic acids and derivatives	Amino acids, peptides, and analogues			39.9	8.47	M-H2O-H	C10H18N2O6S	1.061634984	3428.170194	3498.819632	3356.430988	636.4847067	501.7951635	531.6119806	1919.975905	1994.913538	1853.555146
1.58_138.0561m/z	138.0561255	1.577833333	neg	Ethyl 2-pyrrolecarboxylate	HMDB0059856	Organoheterocyclic compounds	Pyrroles	Pyrrole carboxylic acids and derivatives			39.9	3.14	M-H	C7H9NO2	0.527607666	1449.317649	1224.759527	1272.944929	2573.345106	2296.455681	2526.744655	1154.896921	1463.232803	1184.864722
2.03_198.0754n	197.0681034	2.025516667	neg	Cymoxanil	72304	Unclassified	Unclassified	Unclassified			39.9	3.51	M-H, 2M-H	C7H10N4O3	0.452547507	24081.10409	25696.81255	26900.16359	42213.44161	39650.03878	40766.85316	31741.95045	36303.95649	32791.09444
3.56_268.0626m/z	268.0626183	3.564783333	neg	Citrusinine II	HMDB0030373	Organoheterocyclic compounds	Quinolines and derivatives	Benzoquinolines	*		39.9	7.84	M-H2O-H	C15H13NO5	3.786031969	3492.285758	4048.619799	3662.335159	4897.99971	5240.029397	4893.016253	4105.917058	4124.326795	3797.12564
5.18_359.1351m/z	359.1351254	5.1777	neg	1-(3,4-Dimethoxyphenyl)-1,2-ethanediol 2-O-b-D-glucoside	HMDB0034627	Organic oxygen compounds	Organooxygen compounds	Carbohydrates and carbohydrate conjugates			39.9	4.81	M-H	C16H24O9	1.025933557	56995.83023	63483.1384	58810.52025	50233.08268	48450.08441	54358.13707	55880.56841	54758.95686	57386.65649
5.65_401.1456m/z	401.1455635	5.650833333	neg	Lamioside	LMPR0102070018	Lipids and lipid-like molecules	Prenol lipids	Isoprenoids		C11645	39.9	5.66	M-H2O-H	C18H28O11	0.578024854	119541.9665	113847.3963	133030.0105	60305.63544	57346.69875	58825.84924	102444.0798	83297.82604	103542.9233
5.69_359.1351m/z	359.1351095	5.6875	neg	1-(3,4-Dimethoxyphenyl)-1,2-ethanediol 1-O-b-D-glucoside	HMDB0034626	Organic oxygen compounds	Organooxygen compounds	Carbohydrates and carbohydrate conjugates			39.9	4.4	M-H	C16H24O9	0.981914852	43051.6589	38376.57203	39521.69638	36750.91728	40634.3882	37582.18513	38616.21984	37613.74875	41549.68634
7.79_369.1743m/z	369.1743116	7.793283333	neg	5alpha-Dihydrotestosterone sulfate	LMST05020023	Lipids and lipid-like molecules	Steroids and steroid derivatives	Sulfated steroids	*		39.9	2.85	M-H	C19H30O5S	0.521180159	298068.6637	315848.3164	334286.9354	411124.0858	383526.8496	394098.4607	309518.5791	364326.873	348268.7861
8.74_413.2183m/z	413.2183452	8.735716667	neg	6-Ketoprostaglandin E1	HMDB0004241	Lipids and lipid-like molecules	Fatty Acyls	Eicosanoids	**	C05962	39.9	4.28	M+FA-H	C20H32O6	0.688550603	31728.86298	28823.70538	33978.98787	17887.23225	15410.34024	18359.11831	24523.15689	26268.52459	25786.10444
0.69_145.0983m/z	145.0982737	0.692166667	neg	3-Aminopropionaldehyde	HMDB0001106	Organic oxygen compounds	Organooxygen compounds	Carbonyl compounds	**	C05665	39.8	0	2M-H	C3H7NO	0.153864718	347.8055	395.6076563	318.3927879	1284.851677	1334.38765	1123.074795	709.0752899	744.6059187	672.9257264
0.78_413.1108m/z	413.1108293	0.778533333	neg	Apiosylglucosyl 4-hydroxybenzoate	HMDB0035318	Organic oxygen compounds	Organooxygen compounds	Carbohydrates and carbohydrate conjugates			39.8	15.6	M-H2O-H	C18H24O12	4.383525917	8917.992271	9077.114879	9539.977372	8803.151249	12574.33256	11474.86076	11933.31907	13024.5124	14139.66997
0.80_120.0422n	165.0404203	0.800366667	neg	(S)-3,4-Dihydroxybutyric acid	HMDB0000337	Organic acids and derivatives	Hydroxy acids and derivatives	Beta hydroxy acids and derivatives	*		39.8	0	M+FA-H, 2M-H	C4H8O4	-0.344237894	141381.65	150368.5076	181491.3771	98366.77052	100686.2915	100897.4895	126051.9937	133887.1387	155517.2486
1.16_174.0408m/z	174.040805	1.160166667	neg	(3R,5S)-1-pyrroline-3-hydroxy-5-carboxylic Acid	HMDB0062585	Organic acids and derivatives	Carboxylic acids and derivatives	Amino acids, peptides, and analogues	**	C04281	39.8	0	M+FA-H	C5H7NO3	0.07037397	9528.886424	12050.19686	13461.74573	8779.033211	10577.59167	8502.057802	12480.32782	10667.50333	12328.88892
1.78_157.0507m/z	157.0506503	1.778766667	neg	trans-1,2-Dihydrobenzene-1,2-diol	6046	Organic oxygen compounds	Organooxygen compounds	Alcohols and polyols	*	C04221	39.8	0	M+FA-H	C6H8O2	0.160249963	400.9415731	371.7831325	387.2330791	361.0231306	380.0232251	440.4368599	448.553184	422.7016173	418.0771353
3.87_135.0452m/z	135.0452399	3.868533333	neg	3-Butyn-1-al	66349	Organic oxygen compounds	Organooxygen compounds	Carbonyl compounds	**	C06145	39.8	0	2M-H	C4H4O	0.638534253	1040.507572	1196.777412	1154.081547	614.8647497	577.1077652	666.2393852	1244.755774	1359.776262	1447.875061
3.94_205.0718m/z	205.0718167	3.9351	neg	2-Hydroxyisobutyrate	HMDB0242161	Organic acids and derivatives	Hydroxy acids and derivatives	Alpha hydroxy acids and derivatives			39.8	0	2M-H	C4H7O3-	0.266743086	1038.589653	1106.376132	1323.211718	33.94457223	32.08385547	39.21645906	411.5977553	401.170887	356.0328332
4.16_337.0545m/z	337.0545271	4.15895	neg	5-Amino-6-(5'-phosphoribitylamino)uracil	HMDB0003841	Organic oxygen compounds	Organooxygen compounds	Carbohydrates and carbohydrate conjugates	**	C04454	39.8	4.87	M-H2O-H	C9H17N4O9P	-2.658349715	4768.336126	5023.436013	5145.43963	4512.696548	3564.957019	3928.407831	7463.004368	5938.061845	5942.350661
4.66_203.1289m/z	203.1289459	4.660483333	neg	Ethylmethylacetic acid	HMDB0002176	Lipids and lipid-like molecules	Fatty Acyls	Fatty acids and conjugates	**	C18319	39.8	0	2M-H	C5H10O2	0.309727141	2999.773613	3203.572433	3392.454554	2639.305212	2272.938432	2556.059261	2435.93175	2588.409228	2596.520113
4.70_260.0541m/z	260.0540891	4.703166667	neg	Glycerylphosphorylethanolamine	HMDB0000114	Lipids and lipid-like molecules	Glycerophospholipids	Glycerophosphoethanolamines	**	C01233	39.8	3.17	M+FA-H	C5H14NO6P	0.058011931	3053.78008	3460.819838	3201.234288	1205.474133	1088.03589	1083.220809	3456.908291	3465.220886	3522.844294
4.73_256.0947n	255.0874335	4.725416667	neg	3-carboxy-4-methyl-5-(1-hydroxypropyl)-2-furanpropionic acid	LMFA01150060	Lipids and lipid-like molecules	Fatty Acyls	Fatty Acids and Conjugates			39.8	5.65	M-H, M+FA-H	C12H16O6	0.084865333	3588.752342	3463.525492	3330.652319	5729.881994	5538.604013	5285.675502	3565.69524	3667.66613	3100.498795
5.16_200.1294m/z	200.1294188	5.155116667	neg	Miglustat	HMDB0014563	Organoheterocyclic compounds	Piperidines	Unclassified			39.8	5.21	M-H2O-H	C10H21NO4	0.920459476	1700.171094	1939.706497	1243.209917	1538.992572	1679.689521	1507.232865	1880.042845	1578.154597	1623.970908
5.22_428.1661n	427.1588558	5.222166667	neg	rosavin	HMDB0257308	Unclassified	Unclassified	Unclassified			39.8	8.17	M-H, 2M-H	C20H28O10	-4.939279881	154099.7706	142838.6948	145187.5248	150176.1567	144754.8046	140159.3182	188483.299	183687.3324	195676.8694
6.23_387.1664m/z	387.1663876	6.230633333	neg	2-[4-(3-Hydroxypropyl)-2-methoxyphenoxy]-1,3-propanediol 1-xyloside	HMDB0041552	Organic oxygen compounds	Organooxygen compounds	Carbohydrates and carbohydrate conjugates			39.8	5.75	M-H	C18H28O9	0.854037724	22871.42655	18591.24332	23229.2408	31111.5719	26843.33196	28816.84526	16640.49058	16741.34702	18022.83454
6.81_387.1664m/z	387.1664107	6.809016667	neg	Tuberonic acid glucoside	LMFA13010032	Lipids and lipid-like molecules	Fatty Acyls	Fatty acyl glycosides		C08558	39.8	6.31	M-H	C18H28O9	0.913586858	39246.68722	40693.60379	40475.49954	66406.1567	64039.88069	64580.4204	33414.56518	35816.34747	33695.30661
7.20_543.2557m/z	543.2557279	7.201166667	neg	Perindopril Acyl-beta-D-glucuronide	HMDB0060623	Organoheterocyclic compounds	Pyridines and derivatives	Unclassified	*		39.8	3.25	M-H	C25H40N2O11	-0.378126462	22214.66889	24674.56231	24107.7115	17431.16013	17294.33087	19681.46372	14577.79356	16425.25804	14794.76251
7.36_613.3597m/z	613.3597432	7.36395	neg	Cuscutic resinoside A	LMSL05000008	Lipids and lipid-like molecules	Saccharolipids	Other acyl sugars			39.8	0.708	M-H2O-H	C32H56O12	0.643813217	74822.38709	78942.39777	79398.21121	68578.4931	70667.10348	64976.78378	70612.56208	82676.71551	82408.83543
8.61_511.2526m/z	511.2526471	8.606866667	neg	5-alpha-Dihydrotestosterone glucuronide	HMDB0006203	Lipids and lipid-like molecules	Steroids and steroid derivatives	Steroidal glycosides	*		39.8	8.75	M+FA-H	C25H38O8	-4.76986673	42567.3748	44449.05697	43787.00933	52062.33082	47835.3379	48700.88547	45446.6265	49509.76203	44537.37585
9.08_373.1870m/z	373.1870488	9.077466667	neg	6-Epi-7-isocucurbic acid glucoside	HMDB0029782	Lipids and lipid-like molecules	Fatty Acyls	Fatty acyl glycosides	*		39.8	4.44	M-H	C18H30O8	0.687699446	31077.27242	31439.0287	32917.6781	23390.35693	21837.97702	21745.25214	19111.78983	18172.04085	21297.18878
0.80_295.0841m/z	295.0840827	0.800366667	neg	4-Carboxynevirapine	HMDB0060759	Lipids and lipid-like molecules	Steroids and steroid derivatives	Estrane steroids	*		39.7	10.5	M-H	C15H12N4O3	1.414805557	23576.6846	19689.10402	22493.42858	9541.002654	15914.55211	11596.11422	20386.81016	34174.31949	34615.02756
11.46_118.9182m/z	118.9182228	11.45843333	neg	Permanganate	HMDB0001417	Miscellaneous inorganic compounds	Inorganic oxides	Unclassified	*		39.7	0	M-H	HMnO4	-0.244780221	13632.5886	12465.66639	11397.19042	12661.5553	12391.86745	12692.47912	13495.41984	11919.03518	12654.71883
14.66_284.2715n	283.2642525	14.6619	neg	Palmitic Acid ethyl ester	45008	Lipids and lipid-like molecules	Fatty Acyls	Fatty acid esters	*		39.7	1.73	M-H, M+FA-H	C18H36O2	-0.005313174	155450.3855	181817.5998	215735.0006	144699.5956	201253.4611	187225.7402	208895.8262	202660.4183	210593.0164
2.15_281.1508m/z	281.1508245	2.154666667	neg	Guvacoline	71368	Unclassified	Unclassified	Unclassified			39.7	0	2M-H	C7H11NO2	0.5094012	1033.510969	1342.146623	1293.519698	794.7705859	802.02058	727.414594	2143.88195	2741.503605	2511.760452
2.48_229.1195m/z	229.1194587	2.479216667	neg	L-Proline	HMDB0000162	Organic acids and derivatives	Carboxylic acids and derivatives	Amino acids, peptides, and analogues	**	C00148	39.7	0	2M-H	C5H9NO2	0.339334749	2957.515668	2822.485196	3006.828544	3716.085948	3192.426572	3428.781822	3198.409004	2993.718856	2785.294121
3.06_218.1035m/z	218.1034897	3.05735	neg	Swainsonine	44848	Unclassified	Unclassified	Unclassified			39.7	0	M+FA-H	C8H15NO3	0.539977944	35553.5839	36496.01378	39521.17633	76729.77899	82623.51055	74895.95782	59147.20671	59427.50684	55992.58738
3.46_124.0404m/z	124.0403511	3.455516667	neg	5-ethyl-5-methyl-2,4-oxazolidinedione	HMDB0061082	Organoheterocyclic compounds	Azolidines	Oxazolidines	*		39.7	0	M-H2O-H	C6H9NO3	-0.355889484	479.3624599	537.5144423	464.5129065	527.5470786	510.9563387	619.1354328	585.2757567	625.0267406	556.643048
3.66_307.1037m/z	307.103684	3.6635	neg	6,7-Dimethyl-8-(1-D-ribityl)lumazine	HMDB0003826	Organoheterocyclic compounds	Pteridines and derivatives	Unclassified	**	C04332	39.7	9.71	M-H2O-H	C13H18N4O6	-3.401305959	3906.673478	4690.46463	4396.002082	7312.193799	7963.40852	6182.009277	4882.817437	5450.69041	5720.219529
3.85_247.0449m/z	247.0449395	3.84605	neg	Succinylacetoacetate	HMDB0240258	Organic acids and derivatives	Keto acids and derivatives	Medium-chain keto acids and derivatives			39.7	5.96	M+FA-H	C8H10O6	-4.956208442	10257.25387	10792.0005	11028.76491	11299.92262	12155.57785	11703.10382	12062.65653	13072.78217	12911.34367
3.91_163.0766m/z	163.0765657	3.912566667	neg	2,4-Pentadienal	HMDB0031597	Organic oxygen compounds	Organooxygen compounds	Carbonyl compounds	*		39.7	0	2M-H	C5H6O	0.685769061	30.55840607	55.79199027	64.59812609	21.0102452	44.90464887	71.63563271	528.8856765	561.1211198	641.0269374
4.09_148.0616m/z	148.06162	4.091783333	neg	Dimethylglycine	277	Organic acids and derivatives	Carboxylic acids and derivatives	Amino acids, peptides, and analogues	**	C01026	39.7	0	M+FA-H	C4H9NO2	0.859201316	959.6950226	861.1740951	1063.565182	1010.221622	892.7177691	910.3994457	1225.91932	914.7713903	1045.984977
4.14_165.0426n	329.0755995	4.136366667	neg	4-Pyridoxolactone	HMDB0003454	Organoheterocyclic compounds	Pyridines and derivatives	Pyridinecarboxylic acids and derivatives	**	C00971	39.7	0	M-H2O-H, 2M-H	C8H7NO3	0.019429824	992.2252602	1096.196402	993.8049886	1053.367784	1047.568462	1221.835201	999.8371358	932.9964961	1170.421068
4.16_405.1771m/z	405.1770713	4.15895	neg	Dexnorfenfluramine	1957	Unclassified	Unclassified	Unclassified			39.7	0	2M-H	C10H12F3N	-0.050193117	490.0314159	674.8724881	661.1069893	2389.659798	1834.922394	1852.649054	850.4800546	729.9701157	696.664711
4.19_113.0608m/z	113.0608439	4.189783333	neg	ISOPRENE	HMDB0253673	Unclassified	Unclassified	Unclassified			39.7	0	M+FA-H	C5H8	0.598403863	1097.072714	960.3605204	1013.933381	1205.343476	1166.59428	1539.477725	1216.587514	914.1534386	1001.671234
4.26_193.0506m/z	193.0505723	4.2568	neg	trans-Cinnamic acid	HMDB0000930	Phenylpropanoids and polyketides	Cinnamic acids and derivatives	Cinnamic acids	***	C10438	39.7	0	M+FA-H	C9H8O2	-0.40560008	4610.193236	4817.98731	5023.764497	2497.359526	2294.536864	2189.143512	3614.762707	4010.531226	3532.47073
4.30_405.1772m/z	405.177178	4.300033333	neg	Norfenfluramine	2681	Unclassified	Unclassified	Unclassified			39.7	0	2M-H	C10H12F3N	0.21256424	600.3384968	759.7430739	394.6329742	2401.408458	2457.184279	2168.849122	793.7554611	1137.621189	773.1314756
4.63_295.1399m/z	295.1399188	4.632666667	neg	Mevalonic acid	127	Lipids and lipid-like molecules	Fatty Acyls	Fatty acids and conjugates	**	C00418	39.7	0	2M-H	C6H12O4	0.261722601	1085.547072	933.3385162	781.7159533	972.2838069	938.7396895	770.4661233	1153.05355	971.6631429	1034.429495
4.73_261.0357m/z	261.0357218	4.725416667	neg	Iminoaspartic acid	HMDB0001131	Organic acids and derivatives	Carboxylic acids and derivatives	Dicarboxylic acids and derivatives	**	C05840	39.7	3.6	2M-H	C4H5NO4	-2.736297484	3132.481614	2637.086004	2721.73014	4777.474726	4611.768397	4417.202919	3056.105702	2921.731147	2634.66266
4.95_263.1502m/z	263.1501676	4.94705	neg	2-hydroxy-3-methyl-pentanoic acid	45843	Lipids and lipid-like molecules	Fatty Acyls	Fatty acids and conjugates	*		39.7	0	2M-H	C6H12O3	0.588690904	682.250009	475.7262073	524.6290937	452.6381428	372.9224881	446.5837844	279.7237458	557.8806705	190.2977208
5.06_259.0284m/z	259.0283598	5.05975	neg	Fluoruracil	HMDB0257930	Organoheterocyclic compounds	Diazines	Pyrimidines and pyrimidine derivatives	**	C07649	39.7	0	2M-H	C4H3FN2O2	-0.28801388	779.9273069	863.5288824	668.6014447	1137.108253	1091.542023	1270.597976	1235.596544	1328.729757	1319.05811
5.16_135.0452m/z	135.0452138	5.155116667	neg	Furan	HMDB0013785	Organoheterocyclic compounds	Heteroaromatic compounds	Unclassified	*	C14275	39.7	0	2M-H	C4H4O	0.446538365	2139.893783	2510.419199	2109.556643	520.4213822	516.3272771	591.2770119	2001.41774	2050.242064	2105.695217
5.98_255.1604m/z	255.1604154	5.9829	neg	Isopentenyl acetate	86180	Organic acids and derivatives	Carboxylic acids and derivatives	Carboxylic acid derivatives	*		39.7	0	2M-H	C7H12O2	0.907750123	4723.400328	5279.580912	5596.752818	5277.144523	6128.710269	6022.541498	3591.20622	4164.094146	4177.309078
6.06_213.1498m/z	213.1497865	6.056116667	neg	10-Undecen-2-one	LMFA12000157	Organic oxygen compounds	Organooxygen compounds	Carbonyl compounds			39.7	2.19	M+FA-H	C11H20O	1.001462632	3275.040226	3439.396756	3771.646634	4604.350906	4308.523584	3959.759776	3543.22772	3387.659097	3330.563683
6.23_145.0507m/z	145.0506976	6.230633333	neg	Senecioic acid	HMDB0000509	Lipids and lipid-like molecules	Fatty Acyls	Fatty acids and conjugates	*		39.7	0	M+FA-H	C5H8O2	0.652167383	442.5160643	346.3133987	488.987214	467.1784188	457.3023527	474.6729452	485.3768246	451.0604761	459.7140698
6.86_167.1076m/z	167.1076022	6.86375	neg	Methyl propenyl ketone	HMDB0001184	Organic oxygen compounds	Organooxygen compounds	Carbonyl compounds	*		39.7	0	2M-H	C5H8O	-0.89861458	2725.628894	2757.456878	2547.154556	2953.684426	2950.935	3110.058188	2503.743041	2868.759892	2392.665434
6.96_413.1438m/z	413.1438301	6.96195	neg	6-Amino-9H-purine-9-propanoic acid	HMDB0029939	Organoheterocyclic compounds	Imidazopyrimidines	Purines and purine derivatives	*		39.7	0	2M-H	C8H9N5O2	-0.344356912	1457.207081	1316.026241	1477.181886	1075.089222	707.8014076	723.2352182	690.7854881	992.5728012	1143.360186
7.15_255.1604m/z	255.1603821	7.147366667	neg	Cyclohexane-1-carboxylate	67955	Organic acids and derivatives	Carboxylic acids and derivatives	Carboxylic acids	**	C09822	39.7	0	2M-H	C7H12O2	0.777822271	453.5627013	508.1833188	434.3208368	554.3867624	635.5356693	703.6271242	380.5211458	454.9962896	348.1733839
7.74_215.1289m/z	215.1289299	7.738233333	neg	Butyl butyryllactate	92106	Lipids and lipid-like molecules	Fatty Acyls	Fatty acid esters	*		39.7	0	M-H	C11H20O4	0.218509964	415.1640536	296.2307088	400.9510547	607.711165	637.7384763	433.392945	295.9754454	403.7325442	397.2267324

7.74_437.1433m/z	437.1433079	7.738233333	neg	7-Hydroxy-5-(4-hydroxy-2-oxopentyl)-2-methylchromone 7-glucoside	HMDB0034698	Organic oxygen compounds	Organooxygen compounds	Carbohydrates and carbohydrate conjugates			39.7	10.8	M-H	C21H26O10	-4.593601873	28325.67334	29456.27396	29543.79491	42727.23776	45115.35812	41276.76861	20867.03748	22630.87505	25866.11329
8.71_227.1291m/z	227.1290714	8.713883333	neg	delta-Hexanolactone	HMDB0000453	Organoheterocyclic compounds	Lactones	Delta valerolactones	*		39.7	0	2M-H	C6H10O2	0.827127156	1991.048426	1967.628321	1916.924667	2905.989612	2792.647098	2620.586256	1563.980749	1736.715736	1755.094641
0.78_155.0463m/z	155.0462827	0.778533333	neg	Imidazole-4-acetaldehyde	HMDB0003905	Organoheterocyclic compounds	Azoles	Imidazoles	**	C05130	39.6	0	M+FA-H	C5H6N2O	0.608923134	9605.468125	9051.148462	11881.80768	4627.287214	6920.642761	4478.800301	7899.0826	11249.38313	10411.45681
0.82_323.0792m/z	323.0791671	0.819566667	neg	Quindoxin	HMDB0032927	Organoheterocyclic compounds	Diazanaphthalenes	Benzodiazines			39.6	1.04	2M-H	C8H6N2O2	1.816389831	146083.3682	145212.9014	138721.1211	78711.31125	80084.07834	77004.55143	134423.3151	118127.8781	125030.1379
0.84_177.0405m/z	177.0404972	0.841733333	neg	Methylsuccinic acid	HMDB0001844	Lipids and lipid-like molecules	Fatty Acyls	Fatty acids and conjugates	***		39.6	0	M+FA-H	C5H8O4	0.269437456	20645.65093	21957.33507	25165.35976	19364.39689	19858.60968	19582.84031	20507.95466	22685.16996	22287.66493
1.16_166.0147m/z	166.0146929	1.160166667	neg	Quinolinic acid	HMDB0000232	Organoheterocyclic compounds	Pyridines and derivatives	Pyridinecarboxylic acids and derivatives	***	C03722	39.6	0	M-H	C7H5NO4	0.66886182	4440.789175	4352.182365	4579.783834	5174.349063	5391.846792	5857.742335	4628.307462	4589.868055	5192.771103
1.22_137.0357m/z	137.0357312	1.224233333	neg	Urocanic acid	298	Organoheterocyclic compounds	Azoles	Imidazoles	**	C00785	39.6	3.87	M-H	C6H6N2O2	0.581088601	766.8749016	856.0610862	1128.788535	736.7974261	766.4818965	878.8530899	957.8945014	903.0860464	899.6141413
1.93_216.0878m/z	216.0878127	1.933583333	neg	Crotanecine	68218	Unclassified	Unclassified	Unclassified			39.6	0	M+FA-H	C8H13NO3	0.389152979	12196.39331	13076.94935	9249.404943	11653.25283	12933.55776	13317.99929	11182.75648	11830.59112	10407.76023
10.56_243.1967m/z	243.1966788	10.56396667	neg	2-Hydroxymyristic Acid	6579	Lipids and lipid-like molecules	Fatty Acyls	Fatty acids and conjugates	*		39.6	0	M-H	C14H28O3	0.452477989	769.0421011	620.0190275	913.340176	967.4287941	806.4594609	931.5673399	830.9041598	634.6866248	826.5032966
11.04_247.1704m/z	247.1704473	11.0408	neg	6-Methyl-3,5-heptadien-2-one	87784	Organic oxygen compounds	Organooxygen compounds	Carbonyl compounds	*		39.6	0	2M-H	C8H12O	0.377751702	2224.179306	2186.408652	1989.324034	2149.419148	2298.909096	2604.89741	2448.991069	2339.884104	2345.758003
13.27_325.1844m/z	325.1843878	13.26855	neg	2-Dodecylbenzenesulfonic acid	HMDB0031031	Benzenoids	Benzene and substituted derivatives	Benzenesulfonic acids and derivatives	*		39.6	5.4	M-H	C18H30O3S	0.301609344	838809.9496	651590.5871	628639.1194	544575.8449	607216.0948	575663.0427	709184.5997	880609.9243	651334.7367
13.77_381.1740m/z	381.1740099	13.77343333	neg	4-Hydroxycitrulline	HMDB0029384	Organic acids and derivatives	Carboxylic acids and derivatives	Amino acids, peptides, and analogues	*		39.6	0	2M-H	C6H13N3O4	0.195025378	2505.354323	3239.221816	3052.280988	1839.266945	1086.462398	2568.057818	3398.374046	3310.988371	2453.658042
2.00_167.0351m/z	167.0351106	2.004766667	neg	3-Hydroxybenzaldehyde	65898	Unclassified	Unclassified	Unclassified	***		39.6	0	M+FA-H	C7H6O2	1.051710018	460.6105407	399.2197084	413.9003325	570.6710947	564.2803765	576.7551308	525.9499532	604.1908941	482.0097634
2.09_347.0986m/z	347.098606	2.092433333	neg	Shikimic acid	HMDB0003070	Organic oxygen compounds	Organooxygen compounds	Alcohols and polyols	***	C00493	39.6	0	2M-H	C7H10O5	0.676900697	822.0173315	1110.851249	1097.100916	1383.721221	1400.17745	1718.025816	1010.28008	846.2586587	1122.495668
2.18_147.0664m/z	147.066379	2.180683333	neg	Propionic acid	3215	Organic acids and derivatives	Carboxylic acids and derivatives	Carboxylic acids	**	C00163	39.6	0	2M-H	C3H6O2	0.652371417	1611.763609	1629.48649	1979.193742	2199.474043	2088.390723	2031.16456	2012.131753	2208.945083	2230.657528
2.89_124.0404m/z	124.0404171	2.8859	neg	Pyridine	HMDB0000926	Organoheterocyclic compounds	Pyridines and derivatives	Unclassified		C00747	39.6	0	M+FA-H	C5H5N	0.19024631	718.1932107	591.7774815	686.5759691	554.4411055	484.1792962	615.650446	596.8046068	545.480121	486.4853104
3.13_143.0349m/z	143.0349356	3.131933333	neg	Pyruvaldehyde	HMDB0001167	Organic oxygen compounds	Organooxygen compounds	Carbonyl compounds	**	C00546	39.6	0	2M-H	C3H4O2	-0.323918324	1600.250841	1658.500591	1741.496835	1581.223105	1542.657763	1546.776823	2059.774068	2378.994542	2238.583754
3.35_382.1007m/z	382.1007202	3.350266667	neg	(S)-malyl N-acetyl-alpha-D-glucosaminide	LMFA13010059	Lipids and lipid-like molecules	Fatty Acyls	Fatty acyl glycosides			39.6	6.05	M+FA-H	C12H19NO10	4.810239215	35185.28817	38105.24831	33758.63399	45766.82722	43635.29306	41961.55692	33910.71531	41703.83	34405.21196
3.40_143.0349m/z	143.0349039	3.40385	neg	2-Furanmethanol	HMDB0013742	Organoheterocyclic compounds	Heteroaromatic compounds	Unclassified	*	C20441	39.6	0	M+FA-H	C5H6O2	-0.799963224	183.1832638	279.5904339	121.1857897	305.8243608	277.9647513	260.5302389	219.4352715	155.8695773	108.1373892
3.54_175.0977m/z	175.0976641	3.540433333	neg	Butyric acid	107	Lipids and lipid-like molecules	Fatty Acyls	Fatty acids and conjugates	**	C00246	39.6	0	2M-H	C4H8O2	0.4627412	1255.95053	1356.546065	1289.224138	1022.92892	1142.676479	1246.502499	818.8978658	940.1104803	1027.928513
3.85_149.0720m/z	149.0720327	3.84605	neg	Pyridoxamine	HMDB0001431	Organoheterocyclic compounds	Pyridines and derivatives	Pyridoxamines	**	C00534	39.6	0	M-H2O-H	C8H12N2O2	-0.02247048	324.4751767	370.8854407	337.1749964	311.9788649	358.6338626	373.9389233	388.168715	463.9562764	411.859656
4.02_220.0841m/z	220.0841482	4.024283333	neg	2-Benzimidazolylguanidine	68668	Unclassified	Unclassified	Unclassified			39.6	0	M+FA-H	C8H9N5	0.856577867	255.5043175	287.7183873	236.4513432	454.6822151	489.8977649	446.8608557	300.4300274	373.4497237	334.5237334
4.11_375.1299m/z	375.1299329	4.113883333	neg	(±)-threo-1-(p-Hydroxyphenyl)propylene glycol 4'-glucoside	HMDB0033068	Organic oxygen compounds	Organooxygen compounds	Carbohydrates and carbohydrate conjugates			39.6	4.17	M+FA-H	C15H22O8	0.794741004	12352.81807	13788.45022	13578.79367	18493.87247	15699.26415	16073.63026	16473.41148	17774.32782	17261.25651
4.43_246.0384m/z	246.0384121	4.432683333	neg	m-Trifluoromethylhippuric acid	2682	Unclassified	Unclassified	Unclassified			39.6	0.0556	M-H	C10H8F3NO3	0.24602284	176692.0371	188759.3652	181289.1598	96111.81478	90585.3411	96077.56704	149446.8786	160048.5635	157240.3169
4.52_145.0508m/z	145.0507508	4.521733333	neg	Methylglutaric acid	HMDB0000752	Lipids and lipid-like molecules	Fatty Acyls	Fatty acids and conjugates	***		39.6	0	M-H	C6H10O4	0.811202826	573.9399351	583.4740283	555.3716933	555.9371444	540.2759854	655.6789346	657.4827528	613.9494508	742.204012
4.90_183.0300m/z	183.030017	4.902316667	neg	3,4-Dihydroxybenzaldehyde	HMDB0059965	Organic oxygen compounds	Organooxygen compounds	Carbonyl compounds	***	C16700	39.6	0	M+FA-H	C7H6O3	0.869611268	2026.784047	2114.127294	2207.225191	2329.727462	2251.916328	2064.779469	1887.167688	2103.887781	2261.897502
4.92_209.0821m/z	209.0821143	4.92485	neg	4-(4-Hydroxyphenyl)-2-butanone	HMDB0033723	Benzenoids	Phenols	1-hydroxy-2-unsubstituted benzenoids	***		39.6	0	M+FA-H	C10H12O2	1.108282644	1163.694044	1153.140142	1237.688978	946.7302327	926.9271034	1009.611747	866.6570029	851.9892206	858.6029906
4.95_265.0951n	264.0878357	4.94705	neg	CAY10487	44933	Unclassified	Unclassified	Unclassified			39.6	2.49	M-H2O-H, M-H	C13H15NO5	0.363015022	2676.315593	3058.404564	2982.314876	5302.849765	4817.069045	4953.074094	3492.189973	3651.900371	3001.667407
5.41_251.1290m/z	251.1290076	5.406433333	neg	Methyl sorbate	86362	Lipids and lipid-like molecules	Fatty Acyls	Fatty acid esters	*		39.6	0	2M-H	C7H10O2	0.495558402	2247.505341	2649.410034	2458.163314	1537.702266	1592.396738	1584.025012	2573.379871	2636.264505	2230.254598
5.45_259.1188m/z	259.1187857	5.451166667	neg	Ketoleucine	121	Organic acids and derivatives	Keto acids and derivatives	Short-chain keto acids and derivatives	**	C00233	39.6	0	2M-H	C6H10O3	0.283656588	1925.266055	1995.187276	1603.605348	2300.682914	2517.866782	2983.441816	2729.618	2370.304946	2482.566019
5.47_144.0455m/z	144.0455368	5.471616667	neg	4-Oxo-1-(3-pyridyl)-1-butanone	HMDB0062406	Organic oxygen compounds	Organooxygen compounds	Carbonyl compounds	**	C19567	39.6	4.85	M-H2O-H	C9H9NO2	0.302690199	1698.453455	1450.125507	1500.632466	1673.093528	1680.076078	1699.187066	2304.552824	1667.597045	1655.121094
5.55_148.0616m/z	148.0616398	5.547916667	neg	Lyxosylamine	44771	Unclassified	Unclassified	Unclassified			39.6	0	M-H	C5H11NO4	0.727305842	1501.083283	1298.175774	1289.907634	1117.837438	1435.816192	1310.315699	1615.877031	1344.848523	1518.653303
5.69_481.2054m/z	481.2054332	5.6875	neg	5-Methyldeoxycytidine	HMDB0002224	Nucleosides, nucleotides, and analogues	Pyrimidine nucleosides	Pyrimidine 2'-deoxyribonucleosides	*	C03592	39.6	0	2M-H	C10H15N3O4	0.410029439	1801.118136	1914.187944	1533.901169	463.5371723	540.1209261	214.7536566	877.8223796	1063.50969	1065.0461
5.83_156.1150n	155.1078387	5.825	neg	4-hydroxynonenal	75330	Lipids and lipid-like molecules	Fatty Acyls	Fatty alcohols	*		39.6	0	M-H2O-H, M-H	C9H16O2	0.08329772	18682.45265	18254.90476	19607.31939	22465.84791	15851.76686	16454.3364	15096.99066	14460.85797	14313.96329
5.91_469.1330m/z	469.1330497	5.908066667	neg	Ginkgolide J	LMPR0104540004	Lipids and lipid-like molecules	Prenol lipids	Terpene lactones	*	C07604	39.6	8.25	M+FA-H	C20H24O10	-4.951462586	23414.66407	26833.19895	25292.64098	9836.731223	10711.92312	9333.213186	14990.33255	16867.40183	18799.60838
6.04_339.1538m/z	339.1537828	6.040433333	neg	N-Nitroso-N-morpholinoaminoacetonitrile	1338	Unclassified	Unclassified	Unclassified			39.6	0	2M-H	C6H10N4O2	0.90565	500.0676487	445.4201414	362.5885853	1580.359568	824.7456016	1137.736048	657.9902404	491.7888426	361.3246622
6.21_227.1291m/z	227.1291307	6.206233333	neg	trans-Hex-2-enoic acid	HMDB0010719	Lipids and lipid-like molecules	Fatty Acyls	Fatty acids and conjugates	*		39.6	0	2M-H	C6H10O2	1.087126411	3062.849706	2808.247227	3163.755521	3281.920466	3582.945855	3513.731317	2852.692899	2722.441516	3313.097594
6.28_287.1502m/z	287.1502371	6.2811	neg	4-Hydroxycyclohexylcarboxylic acid	HMDB0001988	Organic oxygen compounds	Organooxygen compounds	Alcohols and polyols	*		39.6	0	2M-H	C7H12O3	0.781037968	1309.832917	1469.13581	1784.485716	2821.335478	2384.021106	2558.014405	1540.773188	1906.887108	1863.500706
6.43_203.1290m/z	203.1289828	6.433933333	neg	Valeric acid	110	Lipids and lipid-like molecules	Fatty Acyls	Fatty acids and conjugates	*	C00803	39.6	0	2M-H	C5H10O2	0.490432333	1938.171189	1930.565677	2007.258119	1994.618639	1543.810768	1744.448787	1789.833647	1544.409407	1447.677551
6.80_183.0664m/z	183.0663792	6.799583333	neg	Vanylglycol	HMDB0001490	Benzenoids	Phenols	Methoxyphenols	**	C05594	39.6	0	M-H	C9H12O4	0.525493452	874.1115425	808.3152376	837.6095469	962.6048095	955.9309922	902.5178404	918.0267907	824.6020625	939.5262981
7.54_343.2128m/z	343.2127562	7.536516667	neg	9-Oxo-nonanoic acid	HMDB0094711	Lipids and lipid-like molecules	Fatty Acyls	Fatty acids and conjugates	**	C16322	39.6	0	2M-H	C9H16O3	0.417971681	2704.565614	2951.987725	3379.352814	3372.264743	3078.854718	3217.184444	1554.528959	1903.114126	1675.835305
7.78_227.1291m/z	227.129111	7.7828	neg	Allyl propionate	HMDB0029487	Organic acids and derivatives	Carboxylic acids and derivatives	Carboxylic acid derivatives	*		39.6	0	2M-H	C6H10O2	1.000939405	3796.2013	4247.224981	4287.553079	5369.314579	5360.714838	5378.568333	3745.416181	3883.973232	4134.848002
7.93_227.1291m/z	227.129099	7.9268	neg	2-Methyl-4-pentenoic acid	87452	Lipids and lipid-like molecules	Fatty Acyls	Fatty acids and conjugates	*		39.6	0	2M-H	C6H10O2	0.947973244	1539.375292	1339.915582	1372.409361	1656.579352	1695.154037	1936.427455	1109.577256	1519.245078	1324.230853
8.07_473.2524m/z	473.252394	8.074883333	neg	2-Angeloyl-9-(3-methyl-2E-pentenoyl)-2b,9a-dihydroxy-4Z,10(14)-oplopadien-3-	HMDB0034064	Lipids and lipid-like molecules	Prenol lipids	Sesquiterpenoids	*		39.6	6.57	M+FA-H	C26H36O5	-4.864215647	124706.3721	139097.4914	128341.074	27412.49902	25644.75158	24188.93204	63439.08182	77004.16418	72569.11406
8.43_347.1713m/z	347.1712976	8.430033333	neg	Ethyladipic acid	HMDB0002023	Lipids and lipid-like molecules	Fatty Acyls	Fatty acids and conjugates	*		39.6	0	2M-H	C8H14O4	0.448546998	1756.824638	1940.985974	1776.582315	83.56023835	48.57226608	116.5749319	1026.95168	1262.878013	1089.304649
8.84_243.1603m/z	243.1603082	8.843116667	neg	5Z-Dodecenoic acid	HMDB0000529	Lipids and lipid-like molecules	Fatty Acyls	Fatty acids and conjugates	*		39.6	0	M+FA-H	C12H22O2	0.632621795	359.7032271	687.9644651	402.4594104	518.2737845	495.5881405	832.6471549	360.2559841	301.0946463	410.8803488
9.22_255.1605m/z	255.1604532	9.2201	neg	1-Methoxy-3-methylene-2-pentanone	86893	Organic oxygen compounds	Organooxygen compounds	Carbohydrates and carbohydrate conjugates	*		39.6	0	2M-H	C7H12O2	1.055425838	1736.167051	1617.651622	1726.398837	2956.108728	3009.122799	2878.340703	1794.217979	1867.095172	1742.387336
9.75_360.2759m/z	360.2759165	9.75225	neg	Dehydrophytosphingosine	LMSP01030002	Organic nitrogen compounds	Organonitrogen compounds	Amines			39.6	3.01	M+FA-H	C18H37NO3	1.17234539	67919.23564	64157.77239	59919.5993	61029.53343	66524.65317	68453.37587	73720.18843	69009.7904	68660.01371
0.67_191.0352m/z	191.0351662	0.6701	neg	2-Furancarboxaldehyde	HMDB0032914	Organic oxygen compounds	Organooxygen compounds	Carbonyl compounds	**	C14279	39.5	0	2M-H	C5H4O2	0.957583545	2152.002251	1772.709506	1786.605733	1304.279152	1443.087939	1447.388973	1719.183756	1472.408054	1729.964437
0.76_184.0617m/z	184.0617015	0.757316667	neg	3,4-Dihydroxybenzylamine	HMDB0012153	Benzenoids	Phenols	Benzenediols	*		39.5	0	M+FA-H	C7H9NO2	1.222932596	729.0479437	1010.823994	655.0035136	417.3676263	521.86739	594.1476132	407.6516373	605.3186488	448.6795536
0.78_245.0433m/z	245.0432924	0.778533333	neg	Phosphatidyl glycerol	186	Unclassified	Unclassified	Unclassified			39.5	0	M-H	C6H15O8P	0.466578417	33402.3945	35919.0729	39475.51686	23610.36139	25861.52417	23542.55409	32681.24927	38658.27099	38629.5715
0.82_157.0367m/z	157.0366808	0.819566667	neg	1,2,4-Triazole-3-carboxamide	2299	Unclassified	Unclassified	Unclassified			39.5	0	M+FA-H	C3H4N4O	-0.292776131	7438.426719	7652.300864	7428.786204	3599.074966	3974.656837	3523.912567	7282.893536	7045.939379	6913.717225
0.82_185.0222m/z	185.0222475	0.819566667	neg	2-Hydroxypropylphosphonate	63585	Unclassified	Unclassified	Unclassified			39.5	0	M+FA-H	C3H9O4P	1.423027216	18281.75964	16012.32118	18320.31075	15199.16669	14873.17645	15255.24955	18088.11139	18896.15228	18143.89406
1.18_115.0037m/z	115.0036793	1.180366667	neg	Glyoxal	HMDB0303107	Unclassified	Unclassified	Unclassified			39.5	0	2M-H	C2H2O2	-0.02509575	855.8978617	973.2420848	1081.930134	915.2760549	945.7047977	840.88243	1011.666856	1121.619016	841.2617884
1.22_130.0510m/z	130.0510097	1.224233333	neg	Acetone cyanohydrin	HMDB0060427	Organic oxygen compounds	Organooxygen compounds	Alcohols and polyols	**	C02659	39.5	0	M+FA-H	C4H7NO	0.504786345	3232.663925	3530.762038	3679.048422	3024.569993	3319.681538	2953.261133	3402.345228	3691.704484	3842.853032
1.25_178.0372m/z	178.037223	1.246333333	neg	Isoxanthopterin	44874	Organoheterocyclic compounds	Pteridines and derivatives	Pterins and derivatives	*	C03975	39.5	0	M-H	C6H5N5O2	0.977294304	421.6260446	511.5366157	489.4423351	543.0666827	485.5531936	570.4701617	409.1256095	435.2372004	482.7819223
1.31_175.0249m/z	175.0249388	1.3134	neg	Pyruvic acid	HMDB0000243	Organic acids and derivatives	Keto acids and derivatives	Alpha-keto acids and derivatives	***	C00022	39.5	0	2M-H	C3H4O3	0.723007969	838.244327	916.5507354	950.7094662	1250.383682	1237.432817	1128.698196	1127.572355	1265.659182	1175.432721
1.38_283.0685m/z	283.0685401	1.380283333	neg	5-Hydroxymethyluracil	HMDB0000469	Organoheterocyclic compounds	Diazines	Pyrimidines and pyrimidine derivatives	*	C03088	39.5	0	2M-H	C5H6N2O3	0.466117728	1304.920181	778.4060339	935.905616	1312.327849	1194.163341	1380.174302	985.5283273	943.994175	881.6131955
1.52_165.0194m/z	165.0194334	1.515416667	neg	Demethylphosphinothricin	63600	Unclassified	Unclassified	Unclassified			39.5	0	M-H	C4H9NO4P	-1.262458457	1133.192531	1120.547628	1180.795721	1262.517232	1203.138201	1192.586357	1242.745461	1259.162574	1207.079892
2.59_287.0773m/z	287.0773457	2.58585	neg	2,3-Dimethylmaleate	63874	Lipids and lipid-like molecules	Fatty Acyls	Fatty acids and conjugates	**	C00922	39.5	0	2M-H	C6H8O4	0.363161259	738.9293911	773.6076956	1146.004039	1709.146174	1632.113602	1524.624144	2758.398486	3440.584925	3583.424308
2.68_247.0225m/z	247.0224924	2.680233333	neg	D-Erythritol 4-phosphate	65986	Unclassified	Unclassified	Unclassified			39.5	0	M+FA-H	C4H11O7P	0.248459519	4598.105466	4517.175969	4588.160837	4154.654657	3948.085541	2823.283011	4844.997295	5274.844871	5098.133955
3.58_253.0694m/z	253.0694367	3.5753	neg	1-Methyl-4-nitroimidazole	HMDB0060659	Organoheterocyclic compounds	Azoles	Imidazoles	**	C16621	39.5	0	2M-H	C4H5N3O2	1.418040431	1007.63464	1130.981938	955.9196661	1448.843777	1489.376197	1521.875077	1167.46748	1247.637381	1222.358472
3.91_225.0770m/z	225.0770229	3.912566667	neg	Coniferyl alcohol	HMDB0012915	Benzenoids	Phenols	Methoxyphenols	**	C00590	39.5	0	M+FA-H	C10H12O3	0.976385252	268.6105222	274.1806079	354.6578224	339.007613	349.2912775	287.3135974	1640.180799	1865.812491	1740.608937
4.00_231.0875m/z	231.0874957	4.002233333	neg	alpha-Ketoisovaleric acid	HMDB0000019	Organic acids and derivatives	Keto acids and derivatives	Short-chain keto acids and derivatives	**	C00141	39.5	0	2M-H	C5H8O3	0.361582778	2136.020868	2268.110662	2343.663193	2944.079867	2798.549208	2783.673984	2199.261733	2418.842518	2215.88863
4.34_220.0617m/z	220.0616605	4.344733333	neg	5-Hydroxyindoleacetaldehyde	58202	Organoheterocyclic compounds	Indoles and derivatives	Hydroxyindoles	**	C05634	39.5	0	M+FA-H	C10H9NO2	0.737263207	2498.022222	2786.784299	2873.885278	1547.50587	1210.431304	1174.124206	2062.902026	2262.088556	2298.947236
4.34_259.1187m/z	259.118739	4.344733333	neg	3S-methyl-2-oxo-pentanoic acid	LMFA01020275	Lipids and lipid-like molecules	Fatty Acyls	Fatty Acids and Conjugates	**	C00671	39.5	0	2M-H	C6H10O3	0.104236436	8783.217024	10002.53495	10375.82606	16897.74318	13573.05612	13353.04183	11394.54893	12294.32799	11544.95615
4.37_375.1300m/z	375.1299596	4.367166667	neg	Ethyl Oxalacetate	6422	Unclassified	Unclassified	Unclassified			39.5	0	2M-H	C8H12O5	0.768479999	7245.937562	8697.280541	8461.82101	8932.581726	8167.677023	8121.471731	7933.525194	10454.27643	8589.706159
4.45_130.0874m/z	130.0873679	4.454616667	neg	Piperidine	64457	Organoheterocyclic compounds	Piperidines	Unclassified	**	C01746	39.5	0	M+FA-H	C5H11N	0.184180575	769.2497789	746.655404	745.3660351	344.2005416	315.6519282	275.1155181	630.7973629	790.2848662	698.9963436
4.45_243.1715m/z	243.1714777	4.454616667	neg	Cycluron	72851	Unclassified	Unclassified	Unclassified			39.5	0	M+FA-H	C11H22N2O	0.310127317	313.0583944	456.322877	334.5632771	258.3615254	362.8945842	279.417922	318.7680787	502.437341	378.560477
4.54_219.1239m/z	219.1239144	4.54425	neg	(±)-3-Hydroxynonanoic acid	HMDB0031513	Organic acids and derivatives	Hydroxy acids and derivatives	Medium-chain hydroxy acids and derivatives	*		39.5	0	M+FA-H	C9H18O3	0.672438743	291.292128	403.4870019	349.8495877	317.7586222	257.4519397	254.0532265	313.6042443	306.4783645	424.4745245
4.70_190.0511m/z	190.0511187	4.703166667	neg	Isoquinoline N-oxide	4111	Unclassified	Unclassified	Unclassified			39.5	0	M+FA-H	C9H7NO	1.048042918	3504.462312	3406.74087	3610.232115	572.2237683	545.3877136	624.8003793	829.1848761	913.7984009	839.0442254
4.70_258.0384m/z	258.0383966	4.703166667	neg	Glucosamine 6-phosphate	HMDB0001254	Organic oxygen compounds	Organooxygen compounds	Carbohydrates and carbohydrate conjugates	**	C00352	39.5	0	M-H	C6H14NO8P	-0.115879459	908.0826037	838.7800015	962.7882236	75.23397291	87.32894653	89.97523237	159.493856	178.6962051	185.11344
5.13_607.0849m/z	607.0849308	5.125666667	neg	Thymidine 3',5'-cyclic monophosphate	HMDB0001570	Organoheterocyclic compounds	Diazines	Pyrimidines and pyrimidine derivatives	**	C00364	39.5	0	2M-H	C10H13N2O7P	0.217843692	1713.656523	1419.401737	1301.468331	2039.671237	2008.344218	1511.762865	1815.191504	1516.394917	1612.870723
5.24_119.0502m/z	119.0501692	5.244033333	neg	3-Ethyl-1,2-benzenediol	HMDB0040177	Benzenoids	Phenols	Benzenediols	**	C06728	39.5	0.807	M-H2O-H	C8H10O2	-0.501428412	2554.291247	2121.727568	2247.876577	134.4234271	148.2516192	150.8602306	2006.180601	1859.557235	2259.181528
5.34_285.1821m/z	285.182118	5.3394	neg	Proline betaine	HMDB0004827	Organic acids and derivatives	Carboxylic acids and derivatives	Amino acids, peptides, and analogues	*	C10172	39.5	0	2M-H	C7H13NO2	0.479064706	460.9244883	669.3563199	764.3967813	497.9233181	599.6347362	487.2916483	681.5877533	718.4989624	875.1896266
5.38_315.1087m/z	315.1086642	5.384216667	neg	2-Isopropylmaleate	3279	Lipids and lipid-like molecules	Fatty Acyls	Fatty acids and conjugates	**	C02631	39.5	0	2M-H	C7H10O4	0.389301144	1405.238849	1523.209889	1512.200286	4025.838196	3308.417375	3252.632252	1574.83288	1726.946227	1564.206592
5.45_335.0774m/z	335.0774474	5.451166667	neg	Homogentisic acid	331	Benzenoids	Benzene and substituted derivatives	Phenylacetic acids	**	C00544	39.5	0	2M-H	C8H8O4	0.614130893	1131.912015	1324.251416	1239.204763	230.3751929	223.8364884	274.4460256	292.5635663	233.8139973	326.9083509
5.60_243.1238m/z	243.1238259	5.602	neg	Triethylene glycol diglycidyl ether	73214	Unclassified	Unclassified	Unclassified			39.5	0	M-H2O-H	C12H22O6	0.109003447	31128.96619	26092.35551	30973.58321	57881.73682	59794.37657	58574.90112	30718.48052	29774.25366	24612.97847
5.60_255.1604m/z	255.1603522	5.602	neg	2,3-Heptanedione	LMFA12000013	Organic oxygen compounds	Organooxygen compounds	Carbonyl compounds	*		39.5	0	2M-H	C7H12O2	0.661169756	4577.234773	4522.159191	4596.833836	5073.966409	5546.989883	5060.476121	4547.843223	3884.090593	4119.218645
5.68_233.1396m/z	233.13959	5.678766667	neg	3-Hydroxycapric acid	6544	Organic acids and derivatives	Hydroxy acids and derivatives	Medium-chain hydroxy acids and derivatives	*		39.5	0	M+FA-H	C10H20O3	0.758022468	608.2145144	555.6577475	606.3991906	389.8967239	507.8946	416.7588093	516.276686	572.1205442	606.7234144
5.68_311.1505m/z	311.1504679	5.678766667	neg	8-Hydroxy-5,6-octadienoic acid	HMDB0031101	Organic acids and derivatives	Hydroxy acids and derivatives	Medium-chain hydroxy acids and derivatives	*		39.5	0	2M-H	C8H12O3	1.460164431	1537.1537	1339.570817	1234.489769	1291.674993	1469.728046	1448.14739	577.583893	865.8240169	1063.118094
5.70_214.1451m/z	214.145052	5.701283333	neg	Nitramine	68167	Unclassified	Unclassified	Unclassified			39.5	0	M+FA-H	C10H19NO	1.093163481	9068.417703	7682.730372	7128.049096	5934.756584	8164.344583	7889.511501	8348.93687	8131.461208	10533.2972
6.09_179.1079m/z	179.1078933	6.087383333	neg	4-(Butoxymethyl)phenol	HMDB0039957	Benzenoids	Benzene and substituted derivatives	Benzylethers			39.5	2.28	M-H	C11H16O2	0.777165509	3734.275839	4358.648217	4459.890922	6158.781212	7358.430699	6241.09242	4744.511613	5272.768451	5535.80703
6.16_371.1713m/z	371.1712742	6.161866667	neg	cis-2-Carboxycyclohexyl-acetic acid	69806	Unclassified	Unclassified	Unclassified			39.5	0	2M-H	C9H14O4	0.35688611	1876.233267	2028.925321	2017.833436	2330.628078	2240.053399	2780.711402	1564.151672	1986.63329	1867.981687
6.17_175.0249m/z	175.0248617	6.171316667	neg	Malonic semialdehyde	HMDB0011111	Organic oxygen compounds	Organooxygen compounds	Carbonyl compounds	**	C00222	39.5	0	2M-H	C3H4O3	0.285054969	928.4738374	918.9823971	997.3736358	641.4592815	620.9933316	726.6060066	751.8616149	876.4499912	779.2748768
6.42_223.0978m/z	223.0978012	6.41995	neg	Cyclohexane-1,2-dione	66348	Organic oxygen compounds	Organooxygen compounds	Carbonyl compounds	*	C06105	39.5	0	2M-H	C6H8O2	0.975465549	1386.90668	1127.469234	1191.731357	2398.538179	2518.95794	2386.913227	1669.269563	1433.905399	1529.086537
6.62_331.1401m/z	331.140113	6.61635	neg	Tryptophyl-Glutamine	HMDB0029081	Organic acids and derivatives	Carboxylic acids and derivatives	Amino acids, peptides, and analogues	*		39.5	6.73	M-H	C16H20N4O4	-3.208470741	36011.30784	38556.37537	35610.028	71201.3527	67727.26097	69007.643	53696.64328	63363.23195	63057.07597
6.71_255.1604m/z	255.1604024	6.70695	neg	Cyclohexyl formate	HMDB0031351	Organic acids and derivatives	Carboxylic acids and derivatives	Carboxylic acid derivatives	*		39.5	0	2M-H	C7H12O2	0.857083425	1393.263727	1310.321826	1550.290862	1722.67617	1448.040211	1597.256682	799.4961687	1208.30818	1092.599709
6.78_745.3660m/z	745.3660072	6.776866667	neg	Chalcomycin	71013	Unclassified	Unclassified	Unclassified			39.5	0	M+FA-H	C35H56O14	1.139218365	285618.2745	268316.9024	295981.9538	82097.35639	71170.14783	83538.5094	195365.2868	197057.222	168900.6895
7.03_145.0507m/z	145.0507224	7.02545	neg	Rhamnose	HMDB0000849	Organic oxygen compounds	Organooxygen compounds	Carbohydrates and carbohydrate conjugates	**	C00507	39.5	0	M-H2O-H	C6H12O5	0.549058678	520.7448536	392.9296917	446.822743	540.9423523	475.9153535	406.1482604	614.0236842	565.3955388	505.8849701
7.09_435.1276m/z	435.1275742	7.086816667	neg	Phenethyl 6-galloylglucoside	HMDB0034573	Benzenoids	Benzene and substituted derivatives	Benzoic acids and derivatives			39.5	9.86	M-H	C21H24O10	-4.8066464	74496.16905	74167.21653	72728.12192	103600.2087	104902.5712	96942.4075	63561.34213	72247.67966	67758.4674
7.11_249.1109m/z	249.110853	7.107783333	neg	5-Methylcytosine	3247	Organoheterocyclic compounds	Diazines	Pyrimidines and pyrimidine derivatives	**	C02376	39.5	0	2M-H	C5H7N3O	1.222444651	1840.8236	2042.199815	1764.591568	2548.308466	2829.873759	2320.5952	1223.803073	1347.739149	1373.886614
7.44_251.1290m/z	251.1290052	7.440866667	neg	Allyl crotonate	HMDB0032067	Lipids and lipid-like molecules	Fatty Acyls	Fatty acid esters	*		39.5	0	2M-H	C7H10O2	0.485774384	2276.674207	2328.81284	2576.742986	2942.524074	2786.892075	2803.244704	1393.326966	1449.653952	1278.639913
7.74_369.1560m/z	369.1560454	7.738233333	neg	AF Toxin II	HMDB0033504	Lipids and lipid-like molecules	Fatty Acyls	Fatty acids and conjugates	*		39.5	2.4	M+FA-H	C17H24O6	1.70903185	88509.15878	91997.50903	91993.95438	132817.1414	139331.8877	129736.6668	67512.28067	72414.78571	80078.87019
8.25_183.1391m/z	183.1391009	8.254283333	neg	xi-p-Menth-3-ene	HMDB0037213	Lipids and lipid-like molecules	Prenol lipids	Monoterpenoids	*		39.5	0	M+FA-H	C10H18	0.343253233	1612.632593	1794.936421	2053.170003	3203.042867	2761.057234	2721.680117	1327.872881	1756.602527	1706.868642
8.46_228.1605m/z	228.1605466	8.463816667	neg	Tecostanine	68068	Unclassified	Unclassified	Unclassified			39.5	0	M+FA-H	C11H21NO	0.160717812	5737.811442	6505.636489	6307.829782	7132.494118	6976.181681	6736.085294	4200.436279	4685.130595	4878.297073
8.65_243.1602m/z	243.1602329	8.645533333	neg	trans-Dodec-2-enoic acid	HMDB0010729	Lipids and lipid-like molecules	Fatty Acyls	Fatty acids and conjugates	*		39.5	0	M+FA-H	C12H22O2	0.252667142	6508.004848	6955.527329	7623.994256	7998.170016	7142.302102	7260.667895	6680.653663	6171.560878	5809.388179
9.13_171.0664m/z	171.066445	9.1257	neg	Diacetyl	HMDB0003407	Organic oxygen compounds	Organooxygen compounds	Carbonyl compounds	**	C00741	39.5	0	2M-H	C4H6O2	0.944977804	374.6912829	363.0336965	333.774344	325.1875562	304.3206399	321.3687431	386.7251899	332.9030665	553.4522934
9.38_255.1604m/z	255.1604017	9.37835	neg	3,4-Heptanedione	87708	Organic oxygen compounds	Organooxygen compounds	Carbonyl compounds	*		39.5	0	2M-H	C7H12O2	0.854638531	1031.252927	940.3633493	751.7669472	1224.353472	1580.827537	1247.84381	731.4973677	864.6534685	844.6038982
9.58_360.2759m/z	360.2758602	9.575683333	neg	4-hydroxy-8cis-sphingenine	LMSP01080009	Lipids and lipid-like molecules	Sphingolipids	Sphingoid bases			39.5	2.39	M+FA-H	C18H37NO3	0.993568081	23546.57114	24077.8275	21851.4278	21685.96643	23087.16483	23481.28551	24539.2501	22298.29142	26832.92304
0.52_109.0044m/z	109.0043927	0.51805	neg	1-Methylparabanic acid	68857	Unclassified	Unclassified	Unclassified			39.4	0	M-H2O-H	C4H4N2O3	0.326510059	97.07818848	88.79426047	75.53438188	89.78665788	67.1558086	84.46882216	111.4461442	88.75686777	65.29249142
0.54_229.0509m/z	229.0508549	0.535283333	neg	Dibenzo-p-dioxin	66733	Unclassified	Unclassified	Unclassified			39.4	0	M+FA-H	C12H8O2	1.20907256	335.99079	304.4963688	552.0184313	100.2084648	217.6482886	144.5122002	272.2281832	188.8373346	203.5508006
0.84_149.0092m/z	149.0091559	0.841733333	neg	Tartronate semialdehyde	58597	Organic oxygen compounds	Organooxygen compounds	Carbohydrates and carbohydrate conjugates	**	C01146	39.4	0	M+FA-H	C3H4O4	-0.053468267	4470.572292	5348.988541	5968.545346	304.0196502	89.04118717	328.270041	1394.786333	1545.930986	1445.700475
0.84_199.0379m/z	199.037947	0.841733333	neg	Diethylphosphate	62856	Organic acids and derivatives	Organic phosphoric acids and derivatives	Phosphate esters	*	C06608	39.4	0	M+FA-H	C4H11O4P	1.614509583	10506.94436	10459.81182	12721.68641	10210.74402	10539.69827	10481.81693	9910.298676	10978.87618	11137.2165
0.88_161.0456m/z	161.0456297	0.884633333	neg	Glutarate semialdehyde	HMDB0012233	Lipids and lipid-like molecules	Fatty Acyls	Fatty acids and conjugates	**	C03273	39.4	0	M+FA-H	C5H8O3	0.712458525	9405.122033	9949.880764	9842.058594	12458.70698	12101.50806	12055.7812	10142.34706	8698.152939	9672.914916
1.22_418.0300m/z	418.0299576	1.224233333	neg	beta-D-Glucopyranose, 1-thio-, 1-(5-(methylsulfinyl)-N-(sulfooxy)pentanimidate)	HMDB0259647	Organic oxygen compounds	Organooxygen compounds	Carbohydrates and carbohydrate conjugates		C08419	39.4	18	M-H2O-H	C12H23NO10S3	-1.396207139	5337.765641	6385.996656	7627.088624	10306.42797	9395.926043	9866.92245	3745.287742	5011.408344	5205.621783
1.31_219.0777m/z	219.0777155	1.3134	neg	Glycyl-Tyrosine	85762	Organic acids and derivatives	Carboxylic acids and derivatives	Amino acids, peptides, and analogues	*		39.4	0	M-H2O-H	C11H14N2O4	0.838838952	305.542667	317.5109422	277.894058	485.61351	264.721204	376.8183713	379.6076409	272.9115944	399.5151238
1.49_117.0194m/z	117.0193671	1.492933333	neg	Malonaldehyde	73135	Organic oxygen compounds	Organooxygen compounds	Carbonyl compounds	*	C19440	39.4	0	M+FA-H	C3H4O2	0.484310872	871.0251155	876.4467491	903.8234537	1102.041838	1026.900899	1077.861084	1072.83658	1045.690057	1023.063429
1.53_351.0789m/z	351.0789304	1.534483333	neg	Ureidosuccinic acid	HMDB0000828	Organic acids and derivatives	Carboxylic acids and derivatives	Amino acids, peptides, and analogues	**	C00438	39.4	0	2M-H	C5H8N2O5	-1.238054768	2346.810236	1922.560792	1915.575504	1823.101042	1862.042344	1680.162485	1819.013117	2547.851234	2138.919945
1.55_379.1613m/z	379.1612614	1.553483333	neg	3-Hydroxysuberic acid	HMDB0000325	Organic acids and derivatives	Hydroxy acids and derivatives	Medium-chain hydroxy acids and derivatives	*		39.4	0	2M-H	C8H14O5	0.764653748	1224.814214	888.8877583	844.5162087	304.4185653	353.1114154	150.0277206	705.3464205	1157.403695	841.5354061

1.56_270.0854n	315.0838029	1.56225	neg	Chloramphenicol alcohol	638	Unclassified	Unclassified	Unclassified			39.4	0	M-H, M+FA-H	C11H14N2O6	0.744962468	19544.87234	15752.46069	16024.01589	17927.85109	17555.96151	16803.36341	14343.31047	19136.08375	15448.83529
1.70_381.0358m/z	381.0358494	1.6992	neg	5,6-Indolequinone-2-carboxylic acid	63501	Unclassified	Unclassified	Unclassified			39.4	0	2M-H	C9H5NO4	-1.543005958	534.9047754	1015.149701	917.2943752	796.6665743	700.7414141	427.9914196	814.8420529	622.6319319	813.3544361
10.48_315.2541m/z	315.254102	10.48043333	neg	Oenanthic ether	HMDB0000798	Lipids and lipid-like molecules	Fatty Acyls	Fatty acid esters	*		39.4	0	2M-H	C9H18O2	0.0594052	1508.524119	1625.903658	1215.128418	1084.967695	740.7185887	1107.309582	1309.246331	2028.912502	1115.975316
10.93_221.1548m/z	221.1548165	10.92908333	neg	1-Phenylheptane	HMDB0061825	Benzenoids	Benzene and substituted derivatives	Unclassified			39.4	0	M+FA-H	C13H20	0.64146356	5394.736451	4585.202698	5020.601277	4579.25788	4966.740829	4901.802673	5319.986164	4711.442025	5583.68937
14.95_255.2331m/z	255.2330529	14.95471667	neg	1-Pentadecene	HMDB0031082	Hydrocarbons	Unsaturated hydrocarbons	Unsaturated aliphatic hydrocarbons	*		39.4	0	M+FA-H	C15H30	0.471163901	43646.7672	36937.18595	46496.9612	31267.81368	42107.38717	41232.23562	41957.56339	46422.92209	48247.46983
15.17_227.2018m/z	227.2017658	15.16735	neg	1-Tridecene	87295	Hydrocarbons	Unsaturated hydrocarbons	Unsaturated aliphatic hydrocarbons	*		39.4	0	M+FA-H	C13H26	0.615040595	613.6684005	392.0531557	695.5005922	304.2869499	503.865964	479.6892477	424.5173868	736.5404209	652.3723097
2.66_171.0664m/z	171.0663921	2.661	neg	Oxolan-3-one	HMDB0002523	Organoheterocyclic compounds	Dihydrofurans	Furanones	*		39.4	0	2M-H	C4H6O2	0.637312292	2460.027017	2468.776565	2335.226297	2583.709616	2562.137541	2629.569247	2791.547554	2485.993906	2797.952821
3.85_229.1195m/z	229.1194573	3.84605	neg	D-Proline	58150	Organic acids and derivatives	Carboxylic acids and derivatives	Amino acids, peptides, and analogues	**	C00763	39.4	0	2M-H	C5H9NO2	0.333144479	1668.078241	1964.955759	1926.891812	2704.717047	2687.082155	2623.717586	2360.089007	2692.684158	2669.001365
3.89_113.0608m/z	113.0608493	3.8906	neg	2,3-Hexanedione	87717	Organic oxygen compounds	Organooxygen compounds	Carbonyl compounds	*		39.4	0	M-H	C6H10O2	0.404930243	354.2969508	387.2897233	431.1336705	591.3398656	547.8836048	577.3930373	498.8802778	508.3360415	582.2722185
4.02_189.0770m/z	189.0769538	4.024283333	neg	Tetrahydrofurfuryl acetate	87469	Organoheterocyclic compounds	Tetrahydrofurans	Unclassified	*		39.4	0	M+FA-H	C7H12O3	0.740576385	1096.328857	1160.833288	1225.900483	1411.991661	1452.666452	1436.825309	1043.036247	1215.296354	1179.15975
4.14_245.1297m/z	245.1296846	4.136366667	neg	para-Anisidine	73035	Benzenoids	Phenol ethers	Aminophenyl ethers	*	C19326	39.4	0	2M-H	C7H9NO	0.541058828	216.9283443	176.1674246	199.9837289	628.3200737	461.5438145	528.0169824	302.980808	319.2508945	267.0938896
4.19_171.0664m/z	171.0664005	4.189783333	neg	gamma-Butyrolactone	HMDB0000549	Organoheterocyclic compounds	Lactones	Gamma butyrolactones	*	C01770	39.4	0	2M-H	C4H6O2	0.686278586	2527.823242	2354.695624	2509.152464	2586.367653	2479.27839	2468.369607	2593.912656	2148.775241	2398.022673
4.23_190.0511m/z	190.0510898	4.234483333	neg	OXYQUINOLINE	44006	Unclassified	Unclassified	Unclassified			39.4	0	M+FA-H	C9H7NO	0.848602877	1208.281764	1341.442898	1558.188685	1571.452794	1472.837084	1491.428806	1388.18207	1575.399162	1499.826392
4.26_427.2341m/z	427.2340605	4.2568	neg	5-Hexyltetrahydro-2-oxo-3-furancarboxylic acid	HMDB0030984	Organoheterocyclic compounds	Lactones	Gamma butyrolactones	*		39.4	0	2M-H	C11H18O4	0.744534999	0.001403199	0.001403199	0.001403199	1163.100498	805.4942954	1184.236968	129.2277922	0.001403199	0.001403199
4.43_183.0663m/z	183.0663458	4.432683333	neg	3-Acetyl-2,5-dimethylfuran	86345	Organic oxygen compounds	Organooxygen compounds	Carbonyl compounds	*		39.4	0	M+FA-H	C8H10O2	0.459250661	13619.57983	13678.92743	13912.45086	14409.84686	14564.93205	14140.69509	15236.96384	13805.45575	14193.65631
4.48_303.0122m/z	303.0122316	4.477166667	neg	D-fructose 6-phosphate	HMDB0304311	Organic oxygen compounds	Organooxygen compounds	Carbohydrates and carbohydrate conjugates	**	C00085	39.4	0	M+FA-H	C6H11O9P-2	-0.154387435	1013.369391	1004.539212	836.2390776	1110.317522	1461.388109	1209.311952	1204.352995	1136.861467	1074.199748
4.52_253.1196m/z	253.119627	4.521733333	neg	D-1-Piperideine-2-carboxylic acid	5992	Organoheterocyclic compounds	Pyridines and derivatives	Hydropyridines	**	C04092	39.4	0	2M-H	C6H9NO2	0.969506042	371.6070328	504.4197573	444.2180212	554.4135308	519.4457051	481.3883326	297.8834544	310.7499287	223.737767
4.75_225.1134m/z	225.1134466	4.747466667	neg	Dihydroactinidiolide	HMDB0035240	Organoheterocyclic compounds	Benzofurans	Unclassified			39.4	4	M+FA-H	C11H16O2	1.187804314	3607.316489	3922.372843	4065.804906	3345.340691	3202.133419	3234.181855	2627.365587	2566.328944	2914.51342
4.75_327.0596m/z	327.0596079	4.747466667	neg	Lumazine	HMDB0254198	Unclassified	Unclassified	Unclassified			39.4	0	2M-H	C6H4N4O2	0.102352736	5331.00431	5190.93748	5427.353831	4467.595415	4689.231516	4593.289161	5178.669721	5469.582954	5527.112924
4.75_416.1311n	397.113254	4.747466667	neg	2-Hydroxybenzaldehyde O-[xylosyl-(1->6)-glucoside]	HMDB0034157	Organic oxygen compounds	Organooxygen compounds	Carbohydrates and carbohydrate conjugates			39.4	7.45	M-H2O-H, M-H	C18H24O11	-1.841870881	17389.77348	17938.62015	18402.12184	29793.32426	30254.1049	29464.57203	24832.69614	27338.19288	24974.06233
4.75_609.0384m/z	609.0384005	4.747466667	neg	UDP-alpha-D-galactose	HMDB0304527	Nucleosides, nucleotides, and analogues	Pyrimidine nucleotides	Pyrimidine nucleotide sugars	**	C00052	39.4	5.69	M+FA-H	C15H22N2O17P2-2	1.466340699	10477.45247	10939.98548	11377.58608	10521.38887	10838.0399	10654.56716	10971.24159	10818.00846	11585.91888
4.77_333.1194m/z	333.1194383	4.769883333	neg	Piromidic acid	HMDB0256609	Unclassified	Unclassified	Unclassified			39.4	8.11	M+FA-H	C14H16N4O3	-3.487951394	8735.501586	8644.216928	9351.688555	13033.29035	12882.31893	12510.5069	8317.582283	9361.063245	8481.470338
4.79_495.1101m/z	495.1100833	4.792316667	neg	Tryptanthrine	68542	Unclassified	Unclassified	Unclassified			39.4	0	2M-H	C15H8N2O2	0.412741362	14532.99849	16156.01706	16676.36583	12332.25233	13971.30085	11515.01011	16538.44789	18059.50991	16377.05455
4.92_250.1086m/z	250.1086086	4.92485	neg	Normeperidinic acid	1133	Unclassified	Unclassified	Unclassified			39.4	0	M+FA-H	C12H15NO2	0.619013926	359.9683451	581.7778008	512.0516173	638.2617001	636.6340584	592.7164022	390.8700664	388.5709372	518.8788532
5.10_333.0594m/z	333.0593743	5.1031	neg	2-(alpha-D-Galactosyl)-sn-glycerol 3-phosphate	66232	Unclassified	Unclassified	Unclassified			39.4	0	M-H	C9H19O11P	0.457134809	2717.709511	2539.798213	2713.03857	3388.223036	2797.715114	3485.253562	1247.461602	1118.430189	1059.053813
5.13_335.1352m/z	335.135191	5.132616667	neg	Trimethoprim	HMDB0014583	Benzenoids	Phenol ethers	Anisoles		C01965	39.4	5.83	M+FA-H	C14H18N4O3	-3.109926297	25737.46039	26476.42395	21936.97636	32577.62287	29635.47561	31360.45994	22063.65042	22487.2231	28850.82239
5.24_145.0507m/z	145.0506513	5.244033333	neg	Tiglic acid	HMDB0001470	Lipids and lipid-like molecules	Fatty Acyls	Fatty acids and conjugates	*	C08279	39.4	0	M+FA-H	C5H8O2	0.18953109	325.8077403	372.8937879	356.7895977	311.8787451	370.7274027	362.9192353	420.7031397	335.6245147	477.5025486
5.30_279.0875m/z	279.0874522	5.302883333	neg	(S)-2,3-Dihydro-6-hydroxy-5-(hydroxyacetyl)-2-isopropenylbenzofuran	HMDB0030486	Organoheterocyclic compounds	Coumarans	Unclassified	*		39.4	0	M+FA-H	C13H14O4	0.172607797	841.8222234	746.0804259	982.1546445	299.4337778	363.9325203	430.46141	458.4176995	394.0727692	515.2868669
5.49_473.1458m/z	473.1458297	5.489016667	neg	Dicrotophos	72442	Unclassified	Unclassified	Unclassified			39.4	0	2M-H	C8H16NO5P	-0.236909754	1040.374453	998.3862811	800.7943157	27.41840724	130.9129604	17.58344927	1270.534994	819.846359	818.5213235
5.55_385.1507m/z	385.1506826	5.547916667	neg	8-Acetyl-T2 tetrol	HMDB0036160	Lipids and lipid-like molecules	Prenol lipids	Sesquiterpenoids	*		39.4	1.32	M+FA-H	C17H24O7	0.813270253	52222.64374	49481.68247	45765.77154	53182.87767	69073.10515	58199.39763	48608.9675	48351.10336	41002.22975
5.65_242.0437m/z	242.0436915	5.650833333	neg	1-alkyl-2-acylglycerophosphoethanolamine	HMDB0062185	Lipids and lipid-like molecules	Glycerophospholipids	Glycerophosphoethanolamines	*		39.4	0	M-H	C6H14NO7P	0.738463323	909.0177477	831.2828201	1208.841456	1563.875896	1323.263781	1397.728802	1057.078508	851.9515991	1092.024534
5.73_148.0616m/z	148.0616123	5.727283333	neg	(R)-beta-Aminoisobutyric acid	HMDB0002299	Organic acids and derivatives	Carboxylic acids and derivatives	Amino acids, peptides, and analogues	**	C01205	39.4	0	M+FA-H	C4H9NO2	0.784826297	1108.955741	1147.277602	888.5691065	1047.983982	1206.857512	1148.769904	1564.276457	1196.015105	1333.793013
5.89_391.1616m/z	391.1616455	5.894166667	neg	3-Amino-L-tyrosine	HMDB0245811	Unclassified	Unclassified	Unclassified			39.4	0	2M-H	C9H12N2O3	-1.689513085	775.5404369	642.9789858	827.691312	1197.659272	1580.865793	1328.831681	405.472524	703.8424625	986.162452
6.15_231.1603m/z	231.1603198	6.154666667	neg	Caproic acid	LMFA01010006	Lipids and lipid-like molecules	Fatty Acyls	Fatty acids and conjugates	*	C01585	39.4	0	2M-H	C6H12O2	0.590183981	3292.111773	3159.303931	3487.784698	4774.556214	4949.144631	5316.566142	1933.475184	2194.153223	2291.65044
6.23_329.1242m/z	329.1242411	6.230633333	neg	2-Phenylethyl beta-D-glucopyranoside	HMDB0029819	Organic oxygen compounds	Organooxygen compounds	Carbohydrates and carbohydrate conjugates			39.4	0	M+FA-H	C14H20O6	0.175363186	744.0867356	593.0022649	842.8574886	898.2344886	703.7267187	858.6404491	640.0669296	615.3545584	674.3584707
6.23_523.1410m/z	523.141014	6.230633333	neg	Indigo	HMDB0240742	Organoheterocyclic compounds	Indoles and derivatives	Indolines			39.4	0	2M-H	C16H10N2O2	-0.314261497	1734.124373	1266.951427	1777.912924	2318.245521	1812.404105	2222.254708	1011.585412	1010.302113	1017.195543
6.28_272.1294m/z	272.1294236	6.2811	neg	N,N-Didemethyldiphenhydramine	2318	Unclassified	Unclassified	Unclassified			39.4	0	M+FA-H	C15H17NO	0.909604583	385.3780926	396.9076778	513.8300218	921.2193028	765.3131853	865.064438	952.6391726	1240.285374	1180.563547
6.54_183.0663m/z	183.0663282	6.539816667	neg	3-(1-Hydroxymethyl-1-propenyl)pentanedioic acid	88984	Lipids and lipid-like molecules	Fatty Acyls	Fatty acids and conjugates	*		39.4	0	M-H2O-H	C9H14O5	0.22671529	886.4460679	864.9699765	817.1244691	682.7915701	872.0278667	863.6381205	1157.997057	907.78672	958.8694784
6.63_227.1291m/z	227.1290942	6.63275	neg	3,4-Hexanedione	HMDB0031492	Organic oxygen compounds	Organooxygen compounds	Carbonyl compounds	*		39.4	0	2M-H	C6H10O2	0.927125781	5289.605293	5644.155534	5102.541102	5657.167383	5639.980294	5478.741866	4628.346015	5292.563263	5212.832085
6.84_255.1604m/z	255.1604417	6.842166667	neg	α-heptenoic acid	34703	Lipids and lipid-like molecules	Fatty Acyls	Fatty acids and conjugates	*		39.4	0	2M-H	C7H12O2	1.010529752	2605.04718	2357.774079	2369.23106	2424.645131	2545.720352	2489.441772	1808.263438	1834.253077	1582.960094
6.88_169.1235m/z	169.123476	6.884583333	neg	2,6-Dimethyl-2,4-heptadiene	HMDB0031415	Hydrocarbons	Unsaturated hydrocarbons	Branched unsaturated hydrocarbons	*		39.4	0	M+FA-H	C9H16	0.584825763	3598.592236	3764.147773	3497.892614	5193.834753	5511.978856	5364.742305	3600.85705	3856.821366	3521.958546
6.88_215.1289m/z	215.1289375	6.884583333	neg	8-Methylnonenoate	LMFA01030994	Lipids and lipid-like molecules	Fatty Acyls	Fatty acids and conjugates	**	C18202	39.4	0	M+FA-H	C10H18O2	0.322375335	3695.431609	4628.563297	4420.353637	4206.904654	4840.99001	4099.168681	3889.683819	3891.41076	3525.098926
7.09_223.0978m/z	223.0977758	7.086816667	neg	(E)-4-Oxo-2-hexen-1-al	HMDB0031759	Organic oxygen compounds	Organooxygen compounds	Carbonyl compounds	*		39.4	0	2M-H	C6H8O2	0.862428878	1561.862679	1247.924096	1282.93674	1299.614066	1519.360716	1302.574176	1523.612212	1574.23694	1434.350383
7.26_325.1636m/z	325.1635754	7.262266667	neg	6-Dimethylaminopurine	5460	Organoheterocyclic compounds	Imidazopyrimidines	Purines and purine derivatives	*		39.4	0	2M-H	C7H9N5	-2.265105797	833.7462857	1028.538471	961.9300449	979.3261577	1007.09385	699.96496	934.0150198	695.5928349	732.9886289
7.43_433.1574m/z	433.1574134	7.426916667	neg	Glutamyl-Alanine	85726	Organic acids and derivatives	Carboxylic acids and derivatives	Amino acids, peptides, and analogues	*		39.4	0	2M-H	C8H13N2O5-	-0.468046022	678.2084276	717.3155907	819.8363341	2306.340503	1830.470871	1952.63399	427.339896	700.9511996	495.7662268
9.02_543.2556m/z	543.2556233	9.01635	neg	Perindopril glucuronide	1796	Unclassified	Unclassified	Unclassified			39.4	0	M-H	C25H40N2O11	-0.570175861	69201.03635	88778.19983	83054.16585	7627.431056	7409.153071	6622.180729	33302.00762	37198.93586	35951.06458
9.37_239.1291m/z	239.1290542	9.367783333	neg	4-Hydroxypropofol	HMDB0014018	Benzenoids	Benzene and substituted derivatives	Cumenes	*		39.4	0	M+FA-H	C12H18O2	0.883357728	11178.26837	5578.578979	6061.261181	6226.642947	5161.732917	4804.18385	6449.272373	5939.631142	6012.488341
0.53_250.1451m/z	250.1450805	0.529533333	neg	N-Desthienylethyl-rotigotine	HMDB0060843	Benzenoids	Tetralins	Unclassified	*		39.3	0	M+FA-H	C13H19NO	1.040263906	340.7370672	358.5674794	231.7996873	368.3719943	294.3977912	265.8924046	388.6151565	298.4413326	363.7875638
0.54_255.2332m/z	255.2331993	0.535283333	neg	Caprylaldehyde	LMFA06000028	Organic oxygen compounds	Organooxygen compounds	Carbonyl compounds	*	C01545	39.3	0	2M-H	C8H16O	0.957772657	647.9649377	760.7856665	775.5217319	671.4624792	656.8353661	517.1244022	905.9499424	647.8491899	758.186262
0.76_146.0459m/z	146.0459402	0.757316667	neg	1-Aminocyclopropanecarboxylic acid	HMDB0036458	Organic acids and derivatives	Carboxylic acids and derivatives	Amino acids, peptides, and analogues	***	C01234	39.3	0	M+FA-H	C4H7NO2	0.582104299	3264.813921	3695.040836	4117.549084	4912.697657	5330.38443	4461.464841	2930.893467	2782.161129	4382.831363
0.78_171.0065m/z	171.0064528	0.778533333	neg	Ethylphosphate	HMDB0012228	Organic acids and derivatives	Organic phosphoric acids and derivatives	Phosphate esters	*		39.3	0	M+FA-H	C2H7O4P	0.433016788	2869.485944	2488.087132	3208.982326	5725.270524	5073.715081	6157.783497	4158.392269	4517.396445	4482.003892
0.78_215.0329m/z	215.0329035	0.778533333	neg	2-C-Methyl-D-erythritol 4-phosphate	64013	Unclassified	Unclassified	Unclassified			39.3	0	M-H	C5H13O7P	1.344818158	144313.7683	177972.7031	222986.9213	301342.6352	310020.4604	291186.6808	262454.8895	216535.8259	242351.8396
0.78_259.0129m/z	259.0129319	0.778533333	neg	(E)-C-HDMAPP	64798	Unclassified	Unclassified	Unclassified			39.3	7.72	M-H	C6H14O7P2	-4.875341535	17549.79574	18110.2698	19580.37341	22621.89744	25119.47047	24477.61417	16689.69391	18008.30089	19370.67831
0.78_355.0883m/z	355.0883289	0.778533333	neg	Gluconolactone	HMDB0000150	Organic oxygen compounds	Organooxygen compounds	Carbohydrates and carbohydrate conjugates	**	C00198	39.3	2.06	2M-H	C6H10O6	0.362983537	17521.80843	17246.78484	16906.47718	18742.34964	17111.6979	17277.51908	15382.87608	16649.17437	16722.34023
0.90_245.0779m/z	245.0778761	0.904683333	neg	Barbituric acid, 5-ethyl-5-(2-hydroxyethyl)-	493	Unclassified	Unclassified	Unclassified			39.3	0	M+FA-H	C8H12N2O4	-0.167910704	8245.489838	7652.386185	8046.252048	8274.59513	10004.81885	8766.43893	8893.052597	8288.862753	10282.12061
1.08_430.9991m/z	430.999144	1.078266667	neg	4-Phospho-D-erythronate	63867	Unclassified	Unclassified	Unclassified			39.3	0	2M-H	C4H9O8P	-1.359450958	6264.727998	5575.360768	5827.622709	5795.199887	4957.787446	6308.788377	5943.953487	5849.821249	5114.857901
1.22_368.8600m/z	368.8600095	1.224233333	neg	iodoacetamide	HMDB0253517	Unclassified	Unclassified	Unclassified			39.3	0	2M-H	C2H4INO	-0.642249745	1109.321674	1110.560912	885.5154848	730.665873	325.3439478	623.8206793	1032.462762	705.1015129	425.1051207
1.27_257.0782m/z	257.078181	1.26875	neg	Levoamine (Chloramphenicol D base)	639	Unclassified	Unclassified	Unclassified			39.3	0	M+FA-H	C9H12N2O4	1.279268584	5905.963224	6479.591766	6126.413722	6609.338126	6391.994669	6518.921866	5890.167725	6659.778773	6664.398578
1.41_357.0692m/z	357.0692388	1.40785	neg	5-FLUOROINDOLE-2-CARBOXYLIC ACID	44300	Unclassified	Unclassified	Unclassified			39.3	0	2M-H	C9H6FNO2	0.005363927	2653.258381	2027.685346	1542.645818	3843.056965	3447.189552	3381.113418	2503.710713	2209.443285	2241.223435
1.91_200.0566m/z	200.0566266	1.911116667	neg	(5S,6S)-6-Amino-5-hydroxycyclohexane-1,3-diene-1-carboxyate	73384	Unclassified	Unclassified	Unclassified			39.3	0	M+FA-H	C7H9NO3	1.164857127	297.5482883	278.5036168	312.0579011	569.4332826	633.9821123	576.3988886	507.3506562	510.9034035	509.4659775
1.99_131.0350m/z	131.0349913	1.9892	neg	Vinyl acetate	73020	Organic acids and derivatives	Carboxylic acids and derivatives	Carboxylic acid derivatives	*	C19309	39.3	0	M+FA-H	C4H6O2	0.105125218	1246.927792	1101.043401	1120.446088	1192.163456	1295.541259	1428.938692	1365.596499	1226.875516	1271.712348
10.03_461.2398m/z	461.2398471	10.02501667	neg	(±)Fenfluramine	2680	Unclassified	Unclassified	Unclassified			39.3	0	2M-H	C12H16F3N	0.335794384	3218.071249	3648.963308	3854.758084	4232.20367	4231.418487	3604.419294	1422.88972	1417.312305	1367.472555
13.41_212.9992m/z	212.9992202	13.40935	neg	Desflurane	HMDB0015320	Organohalogen compounds	Organofluorides	Unclassified		C07519	39.3	0	M+FA-H	C3H2F6O	0.198406282	462.8854694	521.1839264	913.4997	986.6470725	953.9378562	680.2600729	907.6847506	960.1800643	1461.698825
13.83_212.9991m/z	212.9991456	13.8256	neg	Hexafluoroisopropanol	HMDB0060648	Organohalogen compounds	Halohydrins	Fluorohydrins	*		39.3	0	M+FA-H	C3H2F6O	-0.245513926	1112.911622	1123.642959	629.4499107	948.7943657	806.6436417	1220.951569	995.4991829	1107.153482	1033.258397
14.65_423.1822m/z	423.1822021	14.6516	neg	6alpha-Fluoroprednisolone	70208	Unclassified	Unclassified	Unclassified			39.3	0	M+FA-H	C21H27FO5	-0.668863845	16595.03842	17422.42323	18607.87284	14501.54072	16798.9287	15951.41082	18412.6652	15727.35876	16565.58376
14.91_281.2486m/z	281.2486371	14.9138	neg	4Z,8Z-Heptadecadiene	LMFA11000132	Lipids and lipid-like molecules	Fatty Acyls	Hydrocarbons			39.3	0	M+FA-H	C17H32	0.140686994	31354.57952	28499.78337	33958.54746	23907.78629	32304.75498	30274.19817	32365.55444	34741.09035	35998.6205
2.29_504.0094m/z	504.0094262	2.294983333	neg	alpha,beta-Methylene ATP	HMDB0248246	Unclassified	Unclassified	Unclassified			39.3	0	M-H	C11H18N5O12P3	0.438940559	8733.256398	9016.622613	7762.246003	13912.68701	12786.13723	13404.00139	12232.85853	12036.9923	11718.13948
2.58_309.1095m/z	309.1095145	2.57535	neg	(3S,5S)-Carbapenam-3-carboxylic acid	71641	Unclassified	Unclassified	Unclassified			39.3	0	2M-H	C7H9NO3	0.982173824	3881.973177	4335.13333	5098.047732	5713.363037	5600.801511	5240.054367	5202.414069	6026.845889	6403.86429
2.68_179.0351m/z	179.0351069	2.680233333	neg	Phenylglyoxal	HMDB0061916	Benzenoids	Benzene and substituted derivatives	Phenylacetaldehydes			39.3	0	M+FA-H	C8H6O2	0.929290023	7242.610177	6820.397074	7576.697111	6487.629328	6081.126973	4775.260362	7608.440233	8290.329326	8045.622871
3.13_335.0386m/z	335.0386491	3.131933333	neg	Sedoheptulose 7-phosphate	3418	Organic oxygen compounds	Organooxygen compounds	Carbohydrates and carbohydrate conjugates	**	C05382	39.3	0	M+FA-H	C7H15O10P	0.561699158	1219.599838	1640.816374	1400.199608	696.8650203	514.6610491	515.1888166	1017.303744	1271.148018	1214.698365
3.82_189.0171m/z	189.0170746	3.82415	neg	Sodium benzoate	HMDB0303720	Benzenoids	Benzene and substituted derivatives	Benzoic acids and derivatives		C00539	39.3	0	M+FA-H	C7H5NaO2	1.027667732	332.2100536	202.2231749	330.7163101	47.18022732	110.9701108	191.7758871	187.2814738	118.7001362	142.5531509
3.85_191.0584n	381.1043953	3.84605	neg	5-Phenyl-1,3-oxazinane-2,4-dione	HMDB0060400	Benzenoids	Benzene and substituted derivatives	Unclassified	**	C16596	39.3	0	M+FA-H, 2M-H	C10H9NO3	0.921522051	6676.414154	9254.812231	9731.502639	11217.96237	10721.28171	10726.72729	8819.177245	11793.88756	10528.76811
3.94_253.1196m/z	253.1196475	3.9351	neg	Descarbonyl-lacosamide	HMDB0060813	Organic acids and derivatives	Carboxylic acids and derivatives	Amino acids, peptides, and analogues	*		39.3	0	M+FA-H	C11H16N2O2	1.28228982	385.5823547	325.5609184	449.0048159	348.5790154	343.9484351	351.9533872	237.6525388	355.3535322	279.8476061
4.00_228.0880m/z	228.0880213	4.002233333	neg	DL-Adrenaline	HMDB0062515	Benzenoids	Phenols	Benzenediols	***		39.3	0	M+FA-H	C9H13NO3	1.503064379	318.3570975	409.5389861	537.2605243	618.376348	613.558312	584.0566029	668.864787	718.4708099	686.0852478
4.18_243.0850m/z	243.0849747	4.181333333	neg	1H-Imidazole-4-carboxamide, 5-[3-(hydroxymethyl)-3-methyl-1-triazenyl]-	627	Unclassified	Unclassified	Unclassified			39.3	0	M+FA-H	C6H10N6O2	1.253015481	1103.900783	905.888022	1143.746358	1116.00676	1091.164394	1519.569097	1355.796918	929.16717	1101.945937
4.18_435.0545m/z	435.0545193	4.181333333	neg	1-Phosphatidyl-D-myo-inositol	58600	Unclassified	Unclassified	Unclassified			39.3	0	M+FA-H	C11H19O13P	-0.027934059	1391.642531	1127.088564	1232.682952	93.83462128	102.1447793	81.77912479	1823.049097	1900.040537	1674.158468
4.23_467.1194m/z	467.1194225	4.234483333	neg	Plicatic acid	HMDB0256643	Unclassified	Unclassified	Unclassified			39.3	2.59	M+FA-H	C20H22O10	-0.182984786	6835.194868	6595.422594	5538.416034	1576.604529	1222.089701	1507.999407	8249.446967	9496.789	8646.89323
4.37_115.0765m/z	115.0764715	4.367166667	neg	Acetone	HMDB0001659	Organic oxygen compounds	Organooxygen compounds	Carbonyl compounds	**	C00207	39.3	0	2M-H	C3H6O	0.15784577	211.6461618	218.1184385	193.8773412	318.5188117	209.9611043	213.7074128	137.5599773	181.0010204	197.3279182
4.45_399.1663m/z	399.1663196	4.454616667	neg	Propofol glucuronide	HMDB0060933	Organic oxygen compounds	Organooxygen compounds	Carbohydrates and carbohydrate conjugates	*		39.3	3.99	M+FA-H	C18H26O7	0.744103528	24901.81823	25048.79349	24072.79131	12629.03997	12187.13642	10943.73948	16468.11711	18921.58328	18607.2898
4.45_457.0860m/z	457.085953	4.454616667	neg	Flonicamid	72269	Unclassified	Unclassified	Unclassified			39.3	0	2M-H	C9H6F3N3O	1.389471667	608.0763733	367.9241477	381.2874024	1196.988069	621.1406962	1098.808781	1016.257024	902.3102722	510.3584764
4.50_241.1086m/z	241.1086125	4.49925	neg	11-oxo-undeca-5,9-dienoic acid	LMFA01060221	Lipids and lipid-like molecules	Fatty Acyls	Fatty Acids and Conjugates			39.3	3.47	M+FA-H	C11H16O3	2.372435427	6757.943111	7329.909894	8017.799004	7513.546194	6847.150802	7338.549841	6454.664303	7309.115928	7912.808165
4.50_255.0875m/z	255.0874855	4.49925	neg	C11354	LMFA01031001	Lipids and lipid-like molecules	Fatty Acyls	Fatty Acids and Conjugates	**	C11354	39.3	0	2M-H	C6H8O3	0.287917706	1693.155493	1965.106582	2001.846036	1866.41992	1836.147293	1871.65527	1724.605813	1929.735383	2032.479851
4.52_127.0766m/z	127.0765515	4.521733333	neg	3-Methylcyclopentene	87753	Hydrocarbons	Unsaturated hydrocarbons	Olefins	*		39.3	0	M+FA-H	C6H10	1.198107951	212.5983264	237.8047696	210.9589739	398.082332	359.9776265	315.2393065	314.5155608	292.3108315	257.4762798
4.52_139.0765m/z	139.0765425	4.521733333	neg	3-Butyn-1-ol	66350	Unclassified	Unclassified	Unclassified			39.3	0	2M-H	C4H6O	0.637325715	1136.759442	1087.601874	1028.799481	1195.240188	1063.511764	1305.788954	1252.542874	1204.14742	1238.060803
4.54_315.1086m/z	315.1085835	4.54425	neg	Succinylacetone	HMDB0000635	Organic acids and derivatives	Keto acids and derivatives	Medium-chain keto acids and derivatives	*		39.3	0	2M-H	C7H10O4	0.133989134	1557.5817	1536.774029	1540.990429	2204.154941	1951.224943	1883.186638	3661.106467	3416.568747	4315.201393
4.64_363.1089m/z	363.1088536	4.641416667	neg	Homovanillic acid	HMDB0000118	Benzenoids	Phenols	Methoxyphenols	***	C05582	39.3	0	2M-H	C9H10O4	0.858138136	448.6630447	67.049958	247.6776688	138.2319537	95.94038326	276.292425	429.9524244	329.507882	0.001403199
4.75_425.2161m/z	425.216065	4.747466667	neg	Cinnzeylanine	HMDB0036011	Lipids and lipid-like molecules	Prenol lipids	Diterpenoids	*		39.3	10.2	M-H	C22H34O8	-4.754841323	6980.157877	7820.616337	8917.179681	5708.941764	5497.996049	4385.519688	5007.283292	6928.04071	6712.96299
4.79_189.0770m/z	189.0769501	4.792316667	neg	Methyl (R)-3-methyl-2-oxopentanoate	LMFA07010933	Lipids and lipid-like molecules	Fatty Acyls	Fatty acid esters	*		39.3	0	M+FA-H	C7H12O3	0.714949609	2880.874251	3110.497372	3153.78603	2281.093896	2282.29783	2264.916743	2002.646941	2361.050391	2038.437654
4.84_403.0602m/z	403.0602042	4.836566667	neg	5-Hydroxy-2-oxo-4-ureido-2,5-dihydro-1H-imidazole-5-carboxylate	63396	Organic acids and derivatives	Carboxylic acids and derivatives	Amino acids, peptides, and analogues	**	C12248	39.3	0	2M-H	C5H6N4O5	-0.391065246	2168.479726	1631.385465	1825.401037	2319.372938	2295.19214	1865.695426	1614.253573	2026.194187	1543.351427
4.86_215.1289m/z	215.1289349	4.858383333	neg	Obtusilic acid	34721	Lipids and lipid-like molecules	Fatty Acyls	Fatty acids and conjugates	*		39.3	0	M+FA-H	C10H18O2	0.306903227	3202.290116	3268.09435	3531.136664	3094.760822	3079.014758	3132.105358	2089.792116	2146.451691	2075.736672
4.88_182.0823m/z	182.0823073	4.880083333	neg	4-Hydroxy-2,6-dimethylaniline	HMDB0060681	Benzenoids	Phenols	Cresols	**	C16570	39.3	0	M+FA-H	C8H11NO	0.295045907	1497.510709	1579.02204	1686.812054	342.7509228	268.6969974	388.2668619	1061.942064	1207.934354	1100.01072
5.01_206.0824m/z	206.0823994	5.014716667	neg	(R)-Boschniakine	HMDB0030267	Organoheterocyclic compounds	Pyridines and derivatives	Pyridine carboxaldehydes	*	C09915	39.3	0	M+FA-H	C10H11NO	0.823115874	3896.926796	4119.833401	4226.152404	3237.84567	3052.458655	3553.05396	4025.775676	3838.309052	3727.667511
5.01_330.0999m/z	330.0999064	5.014716667	neg	Letrozole	HMDB0015141	Benzenoids	Benzene and substituted derivatives	Diphenylmethanes		C08163	39.3	0	M+FA-H	C17H11N5	0.905435242	914.8620013	1057.396107	1124.564176	793.7170403	1103.393914	1097.973834	1557.039972	1784.859453	1296.810172
5.01_507.1486m/z	507.148624	5.014716667	neg	L-Arginine phosphate	63475	Organic acids and derivatives	Carboxylic acids and derivatives	Amino acids, peptides, and analogues	**	C05945	39.3	0	2M-H	C6H15N4O5P	-0.220453459	1219.203725	1147.437488	1767.206814	540.1506075	816.3966131	886.0051087	1222.473649	1200.795166	921.9665446
5.08_146.0944n	127.0765162	5.080566667	neg	3-Hydroxyisoheptanoic acid	6547	Organic acids and derivatives	Hydroxy acids and derivatives	Medium-chain hydroxy acids and derivatives	*		39.3	0	M-H2O-H, 2M-H	C7H14O3	0.431205009	1273.346269	1195.406193	923.2324295	1080.18904	1049.750538	1193.427664	1476.353553	1125.878716	1249.425656
5.10_260.0542m/z	260.0542039	5.1031	neg	sn-glycero-3-phosphoethanolamine	96256	Lipids and lipid-like molecules	Glycerophospholipids	Glycerophosphoethanolamines			39.3	0	M+FA-H	C5H14NO6P	0.591456209	585.1649844	575.0543302	465.5719152	895.5137655	672.8985103	1082.803909	1266.042738	987.957962	1296.642294
5.22_273.1248m/z	273.1248337	5.222166667	neg	Phenylbutyrylglutamine	HMDB0011687	Organic acids and derivatives	Carboxylic acids and derivatives	Amino acids, peptides, and analogues	*		39.3	0	M-H2O-H	C15H20N2O4	1.258556067	440.468026	571.5667648	391.3778134	549.80827	418.023981	667.8728866	413.7517177	408.4460933	467.8518051
5.24_229.1446m/z	229.1446021	5.244033333	neg	(Z)-3-Methyl-3-decenoic acid	87382	Lipids and lipid-like molecules	Fatty Acyls	Fatty acids and conjugates	*		39.3	0	M+FA-H	C11H20O2	0.376585176	6398.34764	6135.882819	6062.759797	8816.215964	8982.625155	9510.185563	6101.542451	5533.76448	5767.871748
5.24_279.1237m/z	279.1237413	5.244033333	neg	(10S,11S)-Pterosin C	HMDB0030763	Benzenoids	Indanes	Indanones	*		39.3	0	M+FA-H	C14H18O3	-0.239232668	2154.934595	1515.815387	1885.414796	523.5739171	568.2461804	619.4574276	873.4147468	619.4568287	814.5660345
5.41_327.1094m/z	327.1093946	5.406433333	neg	3-Hydroxynevirapine	HMDB0013914	Organic nitrogen compounds	Organonitrogen compounds	Amines	*		39.3	3.83	M+FA-H	C15H14N4O2	-1.715649069	8443.015143	9575.164959	9953.853026	17332.52081	16997.62185	16217.79606	14078.07036	14133.81426	11953.97635
5.58_297.1322m/z	297.1321876	5.584533333	neg	6-Methyladenine	HMDB0002099	Organoheterocyclic compounds	Imidazopyrimidines	Purines and purine derivatives	*	C08434	39.3	0	2M-H	C6H7N5	-2.772142352	2291.43695	2045.987519	2215.030144	2073.228763	2006.40471	2164.471822	2193.901769	2054.484221	1749.295654
5.58_513.1953m/z	513.1953336	5.584533333	neg	5-Methylcytidine	HMDB0000982	Nucleosides, nucleotides, and analogues	Pyrimidine nucleosides	Unclassified	*		39.3	0	2M-H	C10H15N3O5	0.5228915	2132.788364	1998.312325	2065.166434	3209.614734	3298.622595	3263.8738	2206.588327	2852.907173	2139.802522
5.67_309.1323m/z	309.1323262	5.666566667	neg	3-(Pyrazol-1-yl)-L-alanine	65565	Unclassified	Unclassified	Unclassified			39.3	0	2M-H	C6H9N3O2	2.094359625	688.1717197	529.5475291	898.5673056	1311.053767	1103.573735	1120.304167	549.728403	469.4393253	600.6086232
5.93_359.1351m/z	359.1350623	5.927283333	neg	2-(4-Hydroxy-3,5-dimethoxyphenyl)ethanol 4'-glucoside	HMDB0038381	Organic oxygen compounds	Organooxygen compounds	Carbohydrates and carbohydrate conjugates			39.3	3.51	M-H	C16H24O9	0.850670835	20152.2599	22411.12641	23010.35149	27779.54494	33133.52782	30079.09445	24114.16269	27607.52267	27124.98745
6.07_457.1700m/z	457.1699589	6.07	neg	Benz[a]acridine	73043	Unclassified	Unclassified	Unclassified			39.3	0	2M-H	C17H11N	-2.320972235	13275.45899	15388.46966	15725.1652	16332.42623	17620.55556	14450.40643	10968.53607	11159.0445	11521.88531
6.10_479.2290m/z	479.2289519	6.101316667	neg	Phorbol 13-butanoate	65885	Unclassified	Unclassified	Unclassified			39.3	0	M+FA-H	C24H34O7	0.680747135	64528.15399	78743.46897	80133.91968	59280.57794	70963.03051	59489.29704	56188.76677	63629.99705	66027.02526
6.44_479.2289m/z	479.2289489	6.442716667	neg	4-Propanoyl-HT2 toxin	HMDB0036161	Lipids and lipid-like molecules	Prenol lipids	Sesquiterpenoids	*		39.3	1.74	M-H	C25H36O9	0.609333509	28514.92191	26524.71995	26797.8939	31424.00497	26516.67524	28147.01674	33492.13199	32785.51633	31591.55542
6.83_183.1027m/z	183.1027001	6.832183333	neg	(3Z,6Z)-3,6-Nonadienal	HMDB0031152	Organic oxygen compounds	Organooxygen compounds	Carbonyl compounds	**	C16323	39.3	0	M+FA-H	C9H14O	0.233210765	4656.345187	4504.597915	4310.508697	2092.319519	2184.894678	2261.743393	4527.96555	4412.406314	4278.914912
6.88_283.1916m/z	283.1915696	6.8759	neg	4-ene-Valproic acid	HMDB0013897	Lipids and lipid-like molecules	Fatty Acyls	Fatty acids and conjugates	**	C16648	39.3	0	2M-H	C8H14O2	0.30499818	1185.667769	1428.165341	1046.097102	1002.685003	1119.597926	1152.043553	822.1144998	1031.694794	830.9785249
6.92_589.1627m/z	589.1626829	6.915966667	neg	Piperolactam D	93250	Alkaloids and derivatives	Aristolactams	Unclassified			39.3	0	2M-H	C17H13NO4	1.768204419	38544.82137	8117.576441	114618.506	8602.272246	7728.259693	7561.290727	9124.17512	8296.314758	20391.67509

6.97_533.2371m/z	533.2370932	6.970716667	neg	4-amino-MX	HMDB0061009	Benzenoids	Benzene and substituted derivatives	Aniline and substituted anilines	*		39.3	0	2M-H	C12H17N3O4	1.043607409	1140.675386	1477.835866	1041.359264	1810.561613	1724.738107	1901.071869	978.4933004	1174.030663	1391.179518
7.23_438.2136m/z	438.2135878	7.225666667	neg	Parsonsine	68273	Unclassified	Unclassified	Unclassified			39.3	0	M-H	C22H33NO8	0.562741919	100563.3034	103173.0068	99254.92408	64763.62338	70670.42924	62238.46267	69259.43764	79691.48451	74394.55807
7.25_251.1290m/z	251.1290039	7.248333333	neg	3,4-Dimethyl-1,2-cyclopentanedione	HMDB0037026	Organic oxygen compounds	Organooxygen compounds	Carbonyl compounds	*		39.3	0	2M-H	C7H10O2	0.480711859	3906.714071	4368.742837	4249.299601	6534.812719	6428.741096	5779.754822	4186.798559	4317.107393	4407.986438
7.28_257.1760m/z	257.1760327	7.2779	neg	10-Undecenyl acetate	87350	Lipids and lipid-like molecules	Fatty Acyls	Fatty alcohol esters	*		39.3	0	M+FA-H	C13H24O2	0.941986739	4315.227532	4978.030004	4591.57425	4754.87036	4031.487214	4117.997011	3087.724704	3130.689561	3626.468089
7.28_343.1401m/z	343.1400712	7.2779	neg	2-octenedioic acid	LMFA01170085	Lipids and lipid-like molecules	Fatty Acyls	Fatty acids and conjugates	*		39.3	0	2M-H	C8H12O4	0.667930562	3337.305496	3749.641402	3456.423711	4856.837859	5137.403102	4312.226204	2122.740866	2481.283854	2868.217965
7.75_299.1866m/z	299.1866358	7.747083333	neg	Lubiminol	HMDB0029604	Lipids and lipid-like molecules	Prenol lipids	Sesquiterpenoids	*		39.3	0	M+FA-H	C15H26O3	0.937323317	660.6820221	993.0958436	701.4895223	1368.278499	1422.424048	1259.018222	770.9177182	920.6899757	795.8078905
8.26_251.0926m/z	251.0926363	8.264766667	neg	Ethyl trans-p-methoxycinnamate	HMDB0030762	Phenylpropanoids and polyketides	Cinnamic acids and derivatives	Cinnamic acid esters	*	C10476	39.3	0	M+FA-H	C12H14O3	0.675139278	7090.166283	7829.581155	7765.069445	7478.421607	7035.02447	7599.7393	8292.835117	7676.007547	7606.217548
8.68_343.2127m/z	343.2126713	8.68065	neg	Tetrahydrofurfuryl butyrate	91337	Lipids and lipid-like molecules	Fatty Acyls	Fatty acid esters	*		39.3	0	2M-H	C9H16O3	0.17133332	1295.669058	1412.158121	1304.496779	3113.77969	2234.08151	2017.982963	720.0963279	633.9858058	611.271952
9.05_255.1605m/z	255.1604752	9.047766667	neg	Methyl 2E-hexenoate	HMDB0031500	Lipids and lipid-like molecules	Fatty Acyls	Fatty acid esters	*		39.3	0	2M-H	C7H12O2	1.141196775	994.3554964	1147.770301	1130.5069	1543.080291	1384.777357	1583.690439	882.8511838	795.7472201	984.9558637
9.37_195.1391m/z	195.1391497	9.367783333	neg	Cystophorene	HMDB0030944	Hydrocarbons	Unsaturated hydrocarbons	Olefins	*		39.3	0	M+FA-H	C11H18	0.640879714	4496.838745	2254.412948	2412.652865	2404.226066	2292.574717	2001.760542	2600.676952	2240.426414	2368.796255
9.82_179.1079m/z	179.1078758	9.815033333	neg	p-Cymene	LMPR0102090014	Lipids and lipid-like molecules	Prenol lipids	Monoterpenoids	**	C06575	39.3	0	M+FA-H	C10H14	0.91328791	2186.591203	2199.098239	2174.484573	2126.807139	2426.020802	2395.78888	2320.911593	2336.894348	2142.542251
0.54_305.0823m/z	305.0822937	0.535283333	neg	Dehydrowarfarin	HMDB0060575	Phenylpropanoids and polyketides	Coumarins and derivatives	Hydroxycoumarins	*		39.2	0	M-H	C19H14O4	1.179939808	874.1076327	978.2884563	1140.803592	221.7982744	494.477019	372.9435275	931.080766	409.6690559	448.9117345
0.80_160.0615m/z	160.061545	0.800366667	neg	Acetamidopropanal	HMDB0012880	Organic oxygen compounds	Organooxygen compounds	Carbonyl compounds	*	C18170	39.2	0	M+FA-H	C5H9NO2	0.117763853	4968.262413	4892.08486	5219.847021	4729.203161	4709.882139	4991.16957	4816.519497	5860.231493	5994.868059
0.82_117.0193m/z	117.0193385	0.819566667	neg	Acrylic acid	HMDB0031647	Organic acids and derivatives	Carboxylic acids and derivatives	Acrylic acids and derivatives	*	C19501	39.2	0	M+FA-H	C3H4O2	0.087583588	879.2518669	927.1574946	1099.934149	1280.422112	1010.063926	1164.462145	815.8907726	1061.952096	1049.36891
1.16_217.0831m/z	217.083121	1.160166667	neg	Glycylproline	4185	Organic acids and derivatives	Carboxylic acids and derivatives	Amino acids, peptides, and analogues	*		39.2	0	M+FA-H	C7H12N2O3	0.731487636	1493.973717	2056.762353	1989.60281	1279.98303	351.62276	1123.011282	803.9521458	1576.739639	491.4064983
1.16_289.0679m/z	289.0678944	1.160166667	neg	4-Oxoglutaramate	63497	Unclassified	Unclassified	Unclassified			39.2	0	2M-H	C5H7NO4	0.535674364	5205.506483	5102.263755	6095.744686	4504.892162	5789.240137	5947.74287	4199.444264	5617.177475	5798.419163
1.22_279.0723m/z	279.0722921	1.224233333	neg	(R)-(Homo)3-citrate	71255	Unclassified	Unclassified	Unclassified			39.2	0	M+FA-H	C9H14O7	0.582948107	2406.910742	2796.223504	3158.283065	4136.065588	3467.225232	3959.910119	2167.464117	2750.926909	2578.232257
1.31_131.0351m/z	131.0350562	1.3134	neg	Isocrotonic acid	34860	Lipids and lipid-like molecules	Fatty Acyls	Fatty acids and conjugates	*		39.2	0	M+FA-H	C4H6O2	0.858978796	992.5423566	1136.861337	1144.211498	1134.15246	1181.398835	1000.576095	1187.216688	1175.269075	1072.576499
1.31_205.0719m/z	205.0719389	1.3134	neg	3,3-Dimethylglutaric acid	HMDB0002441	Lipids and lipid-like molecules	Fatty Acyls	Fatty acids and conjugates	*		39.2	0	M+FA-H	C7H12O4	1.106780458	171.0079973	199.9732359	192.8410212	548.1742626	551.0746051	493.1898647	124.0571138	146.5651631	128.5082746
1.31_353.0495m/z	353.0494757	1.3134	neg	5-Amino-6-(5'-phosphoribosylamino)uracil	3497	Unclassified	Unclassified	Unclassified			39.2	0	M-H	C9H15N4O9P	-2.577445923	2843.199079	3046.539514	2916.187976	1253.392838	1287.282644	1390.202195	1927.384156	2513.210403	2094.691985
1.44_310.0343m/z	310.0342583	1.43735	neg	3',5'-Cyclic dGMP	3486	Unclassified	Unclassified	Unclassified			39.2	0	M-H2O-H	C10H12N5O6P	-1.277426691	2171.331566	1668.473545	1274.854461	3738.992694	2362.574219	2440.394033	1256.507903	768.4951897	1488.831297
1.53_345.1308m/z	345.130819	1.534483333	neg	N-Acetyl-L-glutamate 5-semialdehyde	3298	Organic acids and derivatives	Carboxylic acids and derivatives	Amino acids, peptides, and analogues	**	C01250	39.2	0	2M-H	C7H11NO4	1.385915542	2021.395697	1728.490439	1896.583167	2166.571143	2073.676272	2253.831252	1563.31335	2091.322781	1669.22299
1.56_201.0883m/z	201.0883029	1.56225	neg	Homoserine lactone	65863	Organic acids and derivatives	Carboxylic acids and derivatives	Amino acids, peptides, and analogues	*	C02926	39.2	0	2M-H	C4H7NO2	1.100257903	663.4215756	595.8553795	414.3494564	335.4375098	283.9514958	243.2701114	445.4472312	576.2002238	503.2970504
1.65_145.0507m/z	145.0507452	1.65405	neg	2-Ethylacrylic acid	4141	Lipids and lipid-like molecules	Fatty Acyls	Fatty acids and conjugates	*		39.2	0	M+FA-H	C5H8O2	1.127380066	1384.492469	1421.088789	1704.456476	1423.85118	1523.215427	1263.485526	1625.894066	1396.131332	1488.63978
1.81_211.0726m/z	211.072587	1.808733333	neg	Nicotinamide, N-(2-hydroxyethyl)-	1524	Unclassified	Unclassified	Unclassified			39.2	0	M+FA-H	C8H10N2O2	0.942717461	1882.579123	1816.505859	1948.212285	1674.172089	1752.69521	2057.892844	1825.835477	1931.450983	1822.991048
1.93_144.0303m/z	144.0302642	1.933583333	neg	(R)-Dihydromaleimide	HMDB0030276	Organoheterocyclic compounds	Pyrrolines	Unclassified	*		39.2	0	M+FA-H	C4H5NO2	0.332396233	1153.998508	1282.672204	981.4647553	1201.979571	1270.654633	1459.371812	1139.252693	1126.394112	1157.818902
1.98_257.0781m/z	257.0781121	1.980516667	neg	N-Acryloylglycine	6343	Organic acids and derivatives	Carboxylic acids and derivatives	Amino acids, peptides, and analogues	*		39.2	0	2M-H	C5H7NO3	0.783889495	1000.797225	906.2418353	1026.470549	1455.261564	1486.483728	1558.748009	1228.69803	1344.888612	1269.144937
10.47_275.1654m/z	275.1654427	10.46831667	neg	Ginsenoyne C	LMFA05000662	Lipids and lipid-like molecules	Fatty Acyls	Fatty alcohols	*		39.2	0.378	M-H	C17H24O3	0.631923625	16477.25588	15806.88093	16089.795	15586.07488	16087.70888	15959.16533	17537.76431	16113.34916	16570.15208
11.65_276.1605m/z	276.160536	11.65171667	neg	Metazocine	69220	Unclassified	Unclassified	Unclassified			39.2	0	M+FA-H	C15H21NO	0.081266047	4471.441122	4216.831577	3899.926905	3960.828574	4451.969807	3956.509536	4789.251284	4587.805435	4380.396376
15.10_250.1449m/z	250.1449393	15.09626667	neg	Heptopargil	72875	Unclassified	Unclassified	Unclassified			39.2	0	M+FA-H	C13H19NO	0.351930719	21790.63389	21636.86998	20354.23707	18827.7605	19178.87717	17864.17939	22553.17826	21105.08034	20988.45713
2.52_205.0719m/z	205.0718562	2.5212	neg	Pimelic acid	3280	Lipids and lipid-like molecules	Fatty Acyls	Fatty acids and conjugates	**	C02656	39.2	0	M+FA-H	C7H12O4	0.59002469	2571.085341	3009.125258	3109.386711	3320.517989	3108.182638	3312.7135	2103.83246	2241.599718	2411.219832
2.72_297.0570m/z	297.0570176	2.72215	neg	d,l-threo-3-HYDROXYASPARTIC ACID	44611	Unclassified	Unclassified	Unclassified			39.2	0	2M-H	C4H7NO5	-1.847326037	2741.038333	2774.875181	2690.280526	3331.374516	3421.169776	3406.64104	2651.954282	3163.841554	2992.678851
2.97_157.0506m/z	157.050635	2.971633333	neg	2,5-Dimethyl-3(2H)-furanone	HMDB0032233	Organoheterocyclic compounds	Dihydrofurans	Furanones	*		39.2	0	M+FA-H	C6H8O2	0.023094662	538.8480987	534.563109	471.8593576	578.1926916	784.9027493	671.5033517	480.3985126	580.7042446	473.2259459
3.16_157.0506m/z	157.050626	3.16405	neg	2-Hydroxy-3-methyl-2-cyclopenten-1-one	HMDB0303163	Unclassified	Unclassified	Unclassified			39.2	0	M+FA-H	C6H8O2	-0.056891281	489.6417225	537.4293551	538.4179905	466.1707828	548.7326661	567.2924492	485.2648111	505.6531125	620.34254
3.69_238.0723m/z	238.0722727	3.685866667	neg	3-Carbamoyl-2-phenylpropionaldehyde	HMDB0060366	Benzenoids	Benzene and substituted derivatives	Phenylacetaldehydes	**	C16587	39.2	0	M+FA-H	C10H11NO3	0.914925542	639.870995	642.0775052	715.7031994	369.7785612	305.951514	332.1741443	258.0139389	231.5626335	289.342456
3.77_155.0350m/z	155.0350339	3.770416667	neg	3-Carboxy-4-methyl-2-oxopentanoate	3301	Organic acids and derivatives	Keto acids and derivatives	Short-chain keto acids and derivatives	**	C04236	39.2	0	M-H2O-H	C7H10O5	0.29637932	154.7512634	153.9479851	202.5316984	190.8449975	166.2919825	125.9400148	187.5694399	150.6699514	187.301204
3.89_124.0404m/z	124.0403968	3.8906	neg	3,6-Dihydronicotinic acid	64432	Unclassified	Unclassified	Unclassified			39.2	0	M-H	C6H7NO2	-0.04148847	286.5987211	235.4010293	221.6963504	243.9823743	239.5146928	223.0723342	332.7174455	229.0831889	306.5875474
3.91_215.0926m/z	215.092551	3.912566667	neg	cis-3-Hexenyl pyruvate	HMDB0038276	Organic acids and derivatives	Keto acids and derivatives	Alpha-keto acids and derivatives			39.2	0	M+FA-H	C9H14O3	0.316511823	4378.958578	4723.080855	4999.883631	3583.578555	3322.742578	3142.368984	4345.565881	4759.473718	4636.081397
3.91_282.0961m/z	282.0960765	3.912566667	neg	7-Methylinosine	HMDB0003950	Nucleosides, nucleotides, and analogues	Purine nucleosides	Unclassified	*		39.2	6.93	M-H	C11H15N4O5+	-3.149771373	2962.653845	2933.211439	3236.873392	2007.117937	1965.5445	1794.858485	2688.989618	2819.232178	2659.827876
3.94_522.9673m/z	522.9672628	3.9351	neg	Xanthosine 5-triphosphate	HMDB0000293	Nucleosides, nucleotides, and analogues	Purine nucleotides	Purine ribonucleotides	**	C00700	39.2	0	M-H	C10H15N4O15P3	-0.260331633	1107.428311	1519.412953	1427.987311	1065.192515	1498.274254	1179.804199	1045.396432	803.4740526	801.1171082
4.05_135.0565m/z	135.0564711	4.046666667	neg	2-Acetyl-3-methylpyrazine	HMDB0030001	Organic oxygen compounds	Organooxygen compounds	Carbonyl compounds	*		39.2	0	M-H	C7H8N2O	0.622430356	486.3560754	591.26296	594.5940513	982.514981	930.3922042	922.0828178	678.8282158	766.7333767	721.6755436
4.07_232.1192m/z	232.1191723	4.069216667	neg	N-n-Hexanoylglycine methyl ester	5799	Unclassified	Unclassified	Unclassified			39.2	0	M+FA-H	C9H17NO3	0.673639035	176.7892683	184.189572	288.1272395	114.0548954	251.4211779	198.5964098	221.4534686	302.466273	245.426277
4.11_347.0986m/z	347.0985638	4.113883333	neg	6,7-dihydroxy-4-oxo-2-heptenoic acid	LMFA01050242	Lipids and lipid-like molecules	Fatty Acyls	Fatty Acids and Conjugates			39.2	3.44	2M-H	C7H10O5	0.555648595	8868.377968	8481.948571	8638.942687	8279.699611	6689.933986	7039.130706	7587.280646	7908.256382	7877.991239
4.21_215.0827m/z	215.0826897	4.212116667	neg	2-Amino-5-phenylpyridine	86463	Organoheterocyclic compounds	Pyridines and derivatives	Phenylpyridines	*		39.2	0	M+FA-H	C11H10N2	0.520156144	704.8852352	726.6577034	883.7913388	468.2090141	568.3867235	599.8742095	566.8403512	732.244771	740.7271637
4.52_157.0871m/z	157.0870767	4.521733333	neg	4-heptenal	75303	Organic oxygen compounds	Organooxygen compounds	Carbonyl compounds	*		39.2	0	M+FA-H	C7H12O	0.524707115	1486.388851	1567.388114	1629.031755	1943.122023	1798.004302	1963.886442	1662.880724	1760.805619	1949.282168
4.52_266.1036m/z	266.1035859	4.521733333	neg	(3-Phenylpropionyl)glycine methyl ester	6387	Unclassified	Unclassified	Unclassified			39.2	0	M+FA-H	C12H15NO3	0.8580447	451.4078081	290.2047823	361.7703744	650.2314235	757.2322028	819.2860617	434.0235018	575.8496207	561.4392455
4.57_238.1087m/z	238.1087121	4.5668	neg	(S)-N-Methylsalsolinol	58182	Organoheterocyclic compounds	Tetrahydroisoquinolines	Unclassified	*		39.2	0	M+FA-H	C11H15NO2	1.193657491	767.1517429	789.0069531	783.0776438	1371.689517	1249.701627	1291.615141	956.9348664	1085.532374	984.441132
4.59_246.0984m/z	246.0984282	4.589283333	neg	Valinopine	73463	Unclassified	Unclassified	Unclassified			39.2	0	M-H	C10H17NO6	0.474962672	846.9977352	940.0770934	1018.739963	1365.514777	1351.728167	991.3897063	1133.567035	999.2627063	1405.758323
4.66_325.0567m/z	325.0566744	4.660483333	neg	6-[4-(carboxymethyl)-2-hydroxyphenoxy]-3,4,5-trihydroxyoxane-2-carboxylic acid	HMDB0125148	Organic oxygen compounds	Organooxygen compounds	Carbohydrates and carbohydrate conjugates	*		39.2	0	M-H2O-H	C14H16O10	0.490571441	4433.734134	3939.907626	4396.267026	5172.077496	5249.917515	6216.161579	6113.027501	4895.215198	4320.302493
4.75_247.1552m/z	247.1551694	4.747466667	neg	3-Hydroxynonyl acetate	LMFA07010753	Lipids and lipid-like molecules	Fatty Acyls	Fatty alcohol esters	*		39.2	0	M+FA-H	C11H22O3	0.355970766	582.6623296	620.7320797	738.3587546	492.2676912	370.9001116	462.7876753	267.5714733	290.6848552	345.1793071
4.75_275.1037m/z	275.1037283	4.747466667	neg	1,7-Dihydroxytacrine	2625	Unclassified	Unclassified	Unclassified			39.2	0	M+FA-H	C13H14N2O2	-0.009867205	1659.242375	1747.335726	1495.857005	1859.46092	1821.871598	1772.714843	1642.942524	2202.14596	1641.128456
4.88_111.0816m/z	111.0815873	4.880083333	neg	Ethyl isovalerate	69416	Lipids and lipid-like molecules	Fatty Acyls	Fatty acid esters	*	C12290	39.2	0	M-H2O-H	C7H14O2	0.374214495	292.658469	367.3047199	340.5228804	338.8704842	340.8475112	355.6249887	203.1853031	274.2878836	286.8981817
4.92_145.0619m/z	145.0618977	4.92485	neg	N-Nitrosopyrrolidine	73003	Organoheterocyclic compounds	Pyrrolidines	Unclassified	*	C19285	39.2	0	M+FA-H	C4H8N2O	0.31928901	218.5978424	298.242594	303.0571392	464.2214705	438.1409744	457.2160146	267.7985112	329.5317421	340.2128235
4.97_359.1826m/z	359.1826091	4.969683333	neg	E-64c	44982	Unclassified	Unclassified	Unclassified			39.2	0	M+FA-H	C15H26N2O5	0.745854809	6012.688129	6650.729709	7219.159248	4619.424695	4634.580934	4645.148753	5956.923511	6452.423246	5555.79016
5.01_119.0502m/z	119.0501936	5.014716667	neg	2-Methylbenzaldehyde	66609	Benzenoids	Benzene and substituted derivatives	Benzoyl derivatives	**	C07214	39.2	0	M-H	C8H8O	-0.37357357	673.0609706	662.7402918	702.3406192	417.8344734	456.232947	447.8770915	633.081845	585.2135271	613.4715584
5.04_291.1089m/z	291.108907	5.037216667	neg	2-oxo-4-hydroxy-hexanoic acid	LMFA01050343	Lipids and lipid-like molecules	Fatty Acyls	Fatty Acids and Conjugates	**	C06762	39.2	0	2M-H	C6H10O4	1.252507843	1446.492084	1388.808563	1746.273074	2350.301862	2465.013669	2799.203287	2256.796743	2187.110583	2548.722378
5.06_335.0389m/z	335.0389099	5.05975	neg	D-glycero-D-manno-Heptose 7-phosphate	63657	Unclassified	Unclassified	Unclassified			39.2	0	M+FA-H	C7H15O10P	1.460851176	1624.534772	1387.471531	1320.646885	1456.104845	1670.803969	1894.421906	1713.516835	1465.334063	1954.422921
5.08_261.0358m/z	261.0357528	5.080566667	neg	2-Oxosuccinamate	65753	Organic acids and derivatives	Keto acids and derivatives	Short-chain keto acids and derivatives	**	C02362	39.2	0	2M-H	C4H5NO4	-2.618238313	1352.772872	1076.630814	991.1648803	1222.388198	1102.190854	1195.439843	1556.767117	938.6439141	1070.119852
5.16_311.1494m/z	311.1494161	5.155116667	neg	2,5-Dimethyl-4-ethoxy-3(2H)-furanone	88281	Organoheterocyclic compounds	Dihydrofurans	Furanones	*		39.2	0	2M-H	C8H12O3	-1.909178844	835.5282641	1065.954298	763.1081793	978.1652019	1170.764614	954.0071654	826.1929414	691.1562099	698.3439228
5.36_204.0667m/z	204.0666811	5.361783333	neg	Indoleacetaldehyde	HMDB0001190	Organoheterocyclic compounds	Indoles and derivatives	Indoles	**	C00637	39.2	0	M+FA-H	C10H9NO	0.404162489	1209.030511	1381.62925	1487.424469	1562.259645	1594.104564	1449.997242	1276.586004	1571.039973	1487.45712
5.43_321.0569m/z	321.0569428	5.428733333	neg	N-Formyl-L-aspartate	63246	Organic acids and derivatives	Carboxylic acids and derivatives	Amino acids, peptides, and analogues	**	C01044	39.2	0	2M-H	C5H7NO5	-1.941901935	2122.816039	1986.959828	1925.548144	1126.766112	1314.566049	1048.739096	1653.145045	1872.735933	1351.900512
5.53_189.1134m/z	189.1133582	5.525416667	neg	Caprylic acid	112	Lipids and lipid-like molecules	Fatty Acyls	Fatty acids and conjugates	**	C06423	39.2	0	M+FA-H	C8H16O2	0.871310342	1077.110082	1071.142554	1141.456845	877.2396071	1168.236912	841.0069535	1019.263503	1043.110644	1179.328439
5.69_145.0871m/z	145.0870835	5.6875	neg	4-Methylpentanal	6155	Organic oxygen compounds	Organooxygen compounds	Carbonyl compounds	**	C02373	39.2	0	M+FA-H	C6H12O	0.655499049	1602.92325	1465.472581	1328.398048	1009.03703	1079.85587	1303.858344	1503.009984	1327.211098	1591.145547
5.79_255.1238m/z	255.1238492	5.785316667	neg	1-Methoxy-1-(2,4,5-trimethoxyphenyl)-2-propanol	87929	Benzenoids	Benzene and substituted derivatives	Phenylpropanes	*		39.2	0	M-H	C13H20O5	0.202422645	526.0146021	462.3139085	415.6726175	608.8068254	548.6298457	566.3814341	564.3591976	360.8868314	552.2265458
5.81_233.1396m/z	233.1396252	5.806016667	neg	(1R,2R,4R,8R)-p-Menthane-2,8,9-triol	HMDB0033574	Lipids and lipid-like molecules	Prenol lipids	Monoterpenoids	*		39.2	0	M+FA-H	C10H20O3	0.945160141	1859.801041	1765.872412	2041.494787	3058.506053	2346.535521	2556.892995	2859.575687	2717.941108	2504.638395
6.09_313.0673m/z	313.0673004	6.087383333	neg	2-Aminomuconic acid	HMDB0001241	Organic acids and derivatives	Carboxylic acids and derivatives	Amino acids, peptides, and analogues	**	C02220	39.2	0	2M-H	C6H7NO4	-1.396386693	3696.743134	4011.619067	4266.930422	6045.145496	7120.638789	6039.064215	4866.128399	4690.602925	4757.675295
6.10_297.1322m/z	297.1321963	6.101316667	neg	1-Methyladenine	62374	Organoheterocyclic compounds	Imidazopyrimidines	Purines and purine derivatives	*	C02216	39.2	0	2M-H	C6H7N5	-2.742797954	4193.238367	4638.668847	4656.232441	4702.601136	4779.592429	4323.458411	2826.144005	2906.621249	3121.881584
6.12_193.0354m/z	193.0354137	6.123483333	neg	D-2-Hydroxyglutaric acid	HMDB0000606	Organic acids and derivatives	Hydroxy acids and derivatives	Short-chain hydroxy acids and derivatives	***	C01087	39.2	0	M+FA-H	C5H8O5	0.253098933	2301.529385	2675.748799	2481.490433	2856.709654	3139.911934	3189.021288	2303.111889	2506.005163	2385.623153
6.12_385.1507m/z	385.150736	6.123483333	neg	4-Deacetylneosolaniol	HMDB0036158	Lipids and lipid-like molecules	Prenol lipids	Sesquiterpenoids	*	C19748	39.2	0.779	M+FA-H	C17H24O7	0.970150485	69268.91249	76572.71245	79165.01137	91039.8997	99781.27028	94950.38451	63351.23371	71521.49385	74553.74247
6.20_303.0828m/z	303.0828167	6.195716667	neg	Imidazoleacetic acid riboside	HMDB0002331	Nucleosides, nucleotides, and analogues	Imidazole ribonucleosides and ribonucleotides	Unclassified	**	C05131	39.2	0.418	M+FA-H	C10H14N2O6	-2.217670024	7565.483948	6710.584434	8062.368092	12476.94068	11801.75471	12257.02333	8314.606529	9354.815239	9515.861449
6.26_137.0972m/z	137.0972317	6.261966667	neg	3,5,5-Trimethyl-2-cyclohexen-1-one	HMDB0031195	Organic oxygen compounds	Organooxygen compounds	Carbonyl compounds	*	C14743	39.2	0	M-H	C9H14O	0.311677863	946.6293294	729.1562289	792.6555486	1064.346747	982.6601771	1177.261461	903.2113384	974.7926263	955.2512596
6.41_363.1666m/z	363.1665958	6.408833333	neg	Soman	66663	Unclassified	Unclassified	Unclassified			39.2	0	2M-H	C7H16FO2P	-1.419581447	577.0337251	626.5375895	754.4251469	989.8429868	1094.039425	1202.544194	534.6296057	324.0284139	448.8891561
6.41_379.2130m/z	379.2130271	6.408833333	neg	5-Methoxytryptamine	7015	Organoheterocyclic compounds	Indoles and derivatives	Tryptamines and derivatives	**	C05659	39.2	0	2M-H	C11H14N2O	-2.426596435	3541.750214	3323.789954	3948.578439	4159.580173	3743.626949	4303.96767	2636.150384	2747.231358	2475.325776
6.44_157.1235m/z	157.1234542	6.442716667	neg	(Z)-2-Octene	HMDB0061903	Hydrocarbons	Unsaturated hydrocarbons	Unsaturated aliphatic hydrocarbons			39.2	1.72	M+FA-H	C8H16	0.453564521	1683.040646	1347.39874	1603.776931	1984.85164	1740.817258	1907.790288	2203.390446	2180.505276	2097.169909
6.48_183.1027m/z	183.1026829	6.477433333	neg	2-Pentylfuran	85277	Organoheterocyclic compounds	Heteroaromatic compounds	Unclassified	*		39.2	0	M+FA-H	C9H14O	0.108135588	963.1745305	985.3334798	1046.912136	577.4717327	491.0493418	414.0140056	1283.762021	1154.055379	1126.127113
6.52_255.1604m/z	255.160384	6.520516667	neg	Methyl (Z)-3-hexenoate	LMFA07010936	Lipids and lipid-like molecules	Fatty Acyls	Fatty acid esters	*		39.2	0	2M-H	C7H12O2	0.785430904	3043.80207	3325.601883	3169.562561	3236.523434	3049.664744	3108.162609	2148.85369	2307.343312	2416.90089
6.58_173.0820m/z	173.0819502	6.582416667	neg	5-Methyl-2,3-hexanedione	HMDB0031593	Organic oxygen compounds	Organooxygen compounds	Carbonyl compounds	*		39.2	0	M+FA-H	C7H12O2	0.138182355	557.6172732	410.5619012	430.8610079	484.6296539	505.8946885	552.4058175	514.2050841	449.6991892	337.7684254
6.71_159.1027m/z	159.1027198	6.70695	neg	2-Heptanone	HMDB0003671	Organic oxygen compounds	Organooxygen compounds	Carbonyl compounds	*	C08380	39.2	0	M+FA-H	C7H14O	0.45456381	1322.423789	1383.227178	1341.184004	1839.135118	1796.531681	1667.573053	1301.126808	1636.645162	1754.739699
6.78_213.0155m/z	213.0155112	6.776866667	neg	1,2-Dinitrobenzene	70273	Unclassified	Unclassified	Unclassified			39.2	0	M+FA-H	C6H4N2O4	1.200276658	10006.70314	8568.773252	6451.149965	5354.759777	3320.975532	10984.41385	5594.304069	5275.381639	9549.138328
6.88_175.0248m/z	175.0248483	6.8759	neg	3-Hydroxypropenoate	69288	Unclassified	Unclassified	Unclassified			39.2	0	2M-H	C3H4O3	0.208742522	1188.097896	1234.099839	953.2705143	1107.338633	1256.519228	1119.709236	1047.219719	1140.223793	1042.550829
6.96_297.1821m/z	297.182136	6.96195	neg	LUPANYL ACID	44429	Unclassified	Unclassified	Unclassified			39.2	0	M+FA-H	C14H24N2O2	0.615084671	27449.51012	26858.63704	27559.84128	51075.23965	44614.30853	50458.60628	25289.66995	30498.1942	31218.99292
7.24_339.1815m/z	339.1814523	7.237866667	neg	Furfural diethyl acetal	69937	Unclassified	Unclassified	Unclassified			39.2	0	2M-H	C9H14O3	0.41179941	2544.970219	2848.432339	3099.234566	4656.199572	4741.49675	4492.4015	2258.81382	2548.865938	2540.561326
7.24_391.1980m/z	391.1980109	7.237866667	neg	Eflornithine	2531	Unclassified	Unclassified	Unclassified			39.2	0	2M-H	C7H14F2N2O2	1.578385556	1333.138835	2025.148906	1652.258715	2936.24642	2816.795705	2374.755538	1960.454387	2489.88433	2233.90975
7.26_273.1110m/z	273.111004	7.262266667	neg	Isoniazid	HMDB0015086	Organoheterocyclic compounds	Pyridines and derivatives	Pyridinecarboxylic acids and derivatives	**	C07054	39.2	0	2M-H	C6H7N3O	1.666153872	806.7610068	1004.448546	824.4664798	1531.328095	1250.777202	1176.843414	870.6568048	891.9704876	1027.258423
7.36_285.1709m/z	285.1709428	7.36395	neg	L-Menthyl acetoacetate	HMDB0032369	Lipids and lipid-like molecules	Prenol lipids	Monoterpenoids	*		39.2	0	M+FA-H	C14H24O3	0.813097809	1143.054022	1375.897141	1206.591419	2672.868672	2462.330329	2556.45249	896.5652177	1178.353596	1040.763351
7.59_287.1502m/z	287.1502432	7.58625	neg	Hydroxycyclohexane-carboxylic Acid	44761	Unclassified	Unclassified	Unclassified			39.2	0	2M-H	C7H12O3	0.802175744	5517.078765	6340.791059	6254.744678	6743.564845	6439.315577	6117.681699	4481.987119	5275.731603	4792.351694
7.72_238.1087m/z	238.1086786	7.719916667	neg	(R)-N-Methylsalsolinol	58166	Organoheterocyclic compounds	Tetrahydroisoquinolines	Unclassified	*		39.2	0	M+FA-H	C11H15NO2	1.020232742	3916.594022	4353.961049	4414.312977	3709.368345	4368.306951	3835.028047	4213.356994	4593.572511	5252.530192
7.89_183.1027m/z	183.1027049	7.89215	neg	Pulegenone	HMDB0036069	Organic oxygen compounds	Organooxygen compounds	Carbonyl compounds	*		39.2	0	M+FA-H	C9H14O	0.267608175	3486.956029	3211.721067	3584.007043	5288.684317	5123.995142	4274.731202	3633.09986	4508.104767	4081.738553
8.01_253.1446m/z	253.144589	8.009683333	neg	(S,E)-Lyratol propanoate	HMDB0031869	Organic acids and derivatives	Carboxylic acids and derivatives	Carboxylic acid derivatives	*		39.2	0	M+FA-H	C13H20O2	0.27006288	782.555827	649.1876145	560.0864506	1163.778998	853.5829511	1236.937261	613.383489	639.2601918	685.5464394
8.13_227.1291m/z	227.1291029	8.129733333	neg	Hydrosorbic acid	LMFA01030009	Lipids and lipid-like molecules	Fatty Acyls	Fatty acids and conjugates	*		39.2	0	2M-H	C6H10O2	0.965431216	3206.685687	3104.060603	2982.802704	2827.997846	3067.492979	3212.668317	2969.318813	2847.228101	2904.194598
8.66_395.1713m/z	395.1713226	8.6638	neg	Calonectrin	HMDB0034915	Lipids and lipid-like molecules	Prenol lipids	Sesquiterpenoids	*		39.2	0	M+FA-H	C19H26O6	0.517256907	14593.28139	15173.04874	13470.61936	15848.61405	14092.06991	14215.37621	8134.48654	7439.198464	7081.025851
0.65_124.9836m/z	124.9836492	0.647833333	neg	Peroxynitrite	HMDB0002179	Homogeneous non-metal compounds	Non-metal oxoanionic compounds	Non-metal peroxynitrites	**	C16845	39.1	0	2M-H	HNO3	-2.858681402	710.9796642	500.3323764	583.3900302	576.2294076	228.3112991	580.272549	510.9405435	401.2629585	283.633603
0.67_323.1462m/z	323.1462396	0.6701	neg	Galactosylhydroxylysine	HMDB0000600	Organic acids and derivatives	Carboxylic acids and derivatives	Amino acids, peptides, and analogues	*	C05547	39.1	2.5	M-H	C12H24N2O8	0.772004702	2206.566427	2264.385646	2360.216595	2772.95238	2383.616032	2733.299423	1822.281444	2145.688984	1869.527177
0.74_415.1839m/z	415.1839015	0.7359	neg	8-METHYLCAFFEINE	84980	Unclassified	Unclassified	Unclassified			39.1	0	2M-H	C9H12N4O2	-2.098381252	1241.83405	1649.935499	1810.159274	1792.533014	1699.154712	1755.214598	2268.776919	2201.469444	2917.391329
0.76_112.0517m/z	112.0516639	0.757316667	neg	Beta-Guanidinopropionic acid	HMDB0013222	Organic nitrogen compounds	Organonitrogen compounds	Guanidines	*	C03065	39.1	0.983	M-H2O-H	C4H9N3O2	0.217184286	4232.195328	3681.627586	3710.563181	4076.44606	4875.251243	4174.426776	3734.579572	3904.553744	3933.582855
0.80_152.9959m/z	152.9958562	0.800366667	neg	Hydroxyacetone phosphate	65991	Unclassified	Unclassified	Unclassified			39.1	0	M-H	C3H7O5P	0.146975689	4351.77867	4209.603161	4364.743451	5464.640458	5781.889732	5555.539663	6045.589634	5535.255008	5896.398046
0.88_147.0300m/z	147.029964	0.884633333	neg	2-Ketobutyric acid	HMDB0000005	Organic acids and derivatives	Keto acids and derivatives	Short-chain keto acids and derivatives	**	C00109	39.1	0	M+FA-H	C4H6O3	0.657475258	2708.235624	3194.525796	3825.509476	4402.827878	4372.819201	4473.997441	2839.64913	3557.800421	3689.406045
0.90_133.0143m/z	133.0142924	0.904683333	neg	Glucosereductone	HMDB0040261	Organic oxygen compounds	Organooxygen compounds	Carbonyl compounds			39.1	0	M+FA-H	C3H4O3	0.517366338	1550.356068	2018.765401	2302.057034	3587.483479	3388.217784	3259.805827	2560.503799	2357.068765	2542.704813
0.93_101.0244m/z	101.0243749	0.925866667	neg	Acrolein	HMDB0041822	Organic oxygen compounds	Organooxygen compounds	Carbonyl compounds	**	C01471	39.1	0	M+FA-H	C3H4O	-0.761704019	681.5048775	795.3818999	784.0850614	805.6132995	712.5999954	816.7706589	698.1401139	781.3875978	758.4181366
1.01_131.0462m/z	131.0462407	1.0111	neg	N-Nitrosomethylvinylamine	73000	Unclassified	Unclassified	Unclassified			39.1	0	M+FA-H	C3H6N2O	0.290927521	144.1748941	258.5901679	135.5241329	125.1625588	43.4297422	72.59107586	233.1699436	178.494804	124.7191848
1.18_258.0620m/z	258.0620061	1.180366667	neg	N,N-Dihydroxy-L-tyrosine	63627	Unclassified	Unclassified	Unclassified			39.1	0	M+FA-H	C9H11NO5	0.37911056	3363.300248	3524.205657	3610.06493	3608.293904	3489.877574	3756.492979	3207.250057	3617.71466	3518.861758
1.20_116.0353m/z	116.035343	1.202333333	neg	Acrylamide	65628	Organic acids and derivatives	Carboximidic acids and derivatives	Carboximidic acids	*	C01659	39.1	0	M+FA-H	C3H5NO	0.371561737	3473.326546	3171.808751	3041.525322	3531.920937	3699.77249	3811.702747	3792.060824	3448.774175	3428.072343
1.20_306.0129m/z	306.0128945	1.202333333	neg	5-Azacytidine 5'-monophosphate	1177	Unclassified	Unclassified	Unclassified			39.1	0	M-H2O-H	C8H12N3O9P	-1.169096046	570.3522077	503.780351	311.3344199	444.347192	733.8254557	578.8851774	522.4294865	536.6916596	887.3445232
1.34_202.0835m/z	202.0835131	1.335933333	neg	Alanylasparagine	HMDB0028682	Organic acids and derivatives	Carboxylic acids and derivatives	Amino acids, peptides, and analogues	*		39.1	0	M-H	C7H13N3O4	0.904165911	1119.528334	984.4306015	853.61593	144.5991272	194.1670554	135.8797242	887.3961792	856.7497845	909.4657362
1.44_172.0617m/z	172.0616769	1.43735	neg	L-Baikiain	66876	Organoheterocyclic compounds	Pyridines and derivatives	Pyridinecarboxylic acids and derivatives	**	C10164	39.1	0	M+FA-H	C6H9NO2	1.145236331	2045.006417	2074.633956	1421.860042	1985.183876	1901.648594	1833.976318	1658.187411	1321.258612	1569.619639
1.44_216.0337m/z	216.0336879	1.43735	neg	o-Toluenesulfamide	3056	Unclassified	Unclassified	Unclassified			39.1	0	M+FA-H	C7H9NO2S	0.500031566	3279.03863	3628.223445	2799.154493	8245.800842	7052.671777	7382.627193	2903.206578	2498.621036	2788.047626
1.53_157.0507m/z	157.0506902	1.534483333	neg	3-Isopropylmalic acid	HMDB0012156	Lipids and lipid-like molecules	Fatty Acyls	Fatty acids and conjugates	**	C04411	39.1	0	M-H2O-H	C7H12O5	0.328707449	647.9172437	529.5563434	436.2191438	656.2594396	535.1261554	596.5454258	554.799445	424.7109491	616.2168917
1.59_184.0616m/z	184.0616135	1.5934	neg	Gabaculine	69310	Unclassified	Unclassified	Unclassified			39.1	0	M+FA-H	C7H9NO2	0.590312061	1736.673681	1357.846365	1514.760923	586.2861954	608.5639845	669.401763	610.8622225	699.3938207	666.0367911
1.59_287.0652m/z	287.0651991	1.5934	neg	5-phosphonooxy-L-lysine	HMDB0059600	Organic acids and derivatives	Carboxylic acids and derivatives	Amino acids, peptides, and analogues	**	C03366	39.1	1.17	M+FA-H	C6H15N2O6P	0.92282115	5772.375386	5295.589897	5614.317843	6922.650087	6814.577411	7462.304845	5963.89108	6164.525465	5711.86144
1.70_210.0386m/z	210.0385721	1.6992	neg	L-Xylonate	HMDB0242183	Organic oxygen compounds	Organooxygen compounds	Carbohydrates and carbohydrate conjugates	**	C05411	39.1	0	M+FA-H	C5H9O6-	2.764537318	1115.408387	1403.946534	1237.327651	1718.51471	1461.831604	1335.008164	1385.002219	1440.488992	1663.277924

1.74_365.0493m/z	365.0492757	1.736816667	neg	2,4-Dinitroaniline	70282	Unclassified	Unclassified	Unclassified			39.1	0	2M-H	C6H5N3O4	1.47745136	1830.867282	1662.392471	1352.970553	602.4387149	703.631306	459.2980651	727.4130355	938.019835	965.740677
1.84_142.0510m/z	142.0509988	1.841666667	neg	2,4-Dimethyloxazole	HMDB0032968	Organoheterocyclic compounds	Azoles	Oxazoles			39.1	0	M+FA-H	C5H7NO	0.330272017	1900.565503	1892.32933	2057.158026	1984.258724	1922.788796	1905.200626	2007.866779	1800.327261	1809.425442
10.63_323.2231m/z	323.2230535	10.63316667	neg	L-Nicotine	1526	Organoheterocyclic compounds	Pyridines and derivatives	Pyrrolidinylpyridines	**	C00745	39.1	0	2M-H	C10H14N2	-3.290755744	1087.344707	2717.275947	1047.984759	333.0599119	800.2084335	454.8599699	342.186619	1632.56839	197.235536
11.05_837.1883m/z	837.1882613	11.05113333	neg	Cyanidin 3-arabinoside	92761	Phenylpropanoids and polyketides	Flavonoids	Flavonoid glycosides			39.1	0	2M-H	C20H19O10+	-0.126278259	11364.64518	11267.75999	9651.912597	10450.62059	11161.12781	12345.58254	11984.35826	11852.15047	11543.90654
14.66_487.2266m/z	487.2265666	14.6619	neg	Guanidinoproclavaminic acid	64566	Unclassified	Unclassified	Unclassified			39.1	0	2M-H	C9H16N4O4	-0.956406817	5663.297365	4126.676701	4451.589643	5079.813761	5143.253951	4610.494099	5388.881265	4525.229497	4189.614316
2.00_173.0932m/z	173.0931838	2.004766667	neg	D-2-Amino-hexano-6-lactam	66307	Unclassified	Unclassified	Unclassified			39.1	0	M+FA-H	C6H12N2O	0.139567052	1219.798974	1215.282414	1289.993755	674.7044303	614.3573458	746.2285058	766.4204226	927.4563951	845.9782797
2.07_112.0404m/z	112.0404031	2.070366667	neg	3-Butenenitrile	HMDB0031323	Organic nitrogen compounds	Organonitrogen compounds	Organic cyanides	*		39.1	0	M+FA-H	C4H5N	0.015956377	179.7152214	156.2305189	163.9355356	183.6712668	177.9105711	189.9327157	276.5867988	232.135683	195.3196836
2.27_142.0510m/z	142.0509996	2.27405	neg	1-Cyano-2-hydroxy-3-butene	HMDB0031339	Organic oxygen compounds	Organooxygen compounds	Alcohols and polyols	*		39.1	0	M+FA-H	C5H7NO	0.338975063	1528.635194	1577.017341	1540.382821	2239.613738	2296.198776	2143.625976	1787.862041	1697.779945	1793.649403
3.04_139.0399m/z	139.0398524	3.041866667	neg	3,5-Dihydroxyanisole	HMDB0132905	Benzenoids	Phenols	Methoxyphenols	*		39.1	0	M-H	C7H8O3	-1.536940054	2384.883432	2503.487511	2584.336908	726.385136	622.7594802	725.2846751	2006.542735	2080.751441	2049.111252
3.22_103.0400m/z	103.040028	3.215616667	neg	Propionaldehyde	64615	Organic oxygen compounds	Organooxygen compounds	Carbonyl compounds	**	C00479	39.1	0	M+FA-H	C3H6O	-0.683091016	198.9966429	206.885123	124.0762777	204.5579758	203.4527253	252.838303	201.7213679	157.7433036	176.4101231
3.42_116.0717m/z	116.0717118	3.417666667	neg	Pyrrolidine	87832	Organoheterocyclic compounds	Pyrrolidines	Unclassified	*		39.1	0	M+FA-H	C4H9N	0.135786633	353.7918442	381.6155269	385.8772115	641.2939447	631.556658	583.8401944	604.7464626	570.964295	487.9717991
3.65_519.1359m/z	519.1358643	3.64965	neg	trans-Caffeic acid [apiosyl-(1->6)-glucosyl] ester	89479	Phenylpropanoids and polyketides	Isoflavonoids	Furanoisoflavonoids			39.1	0	M+FA-H	C20H26O13	0.67613303	3446.476987	4248.737582	3955.705554	12825.56289	14223.11344	12255.35335	6052.23549	5312.839056	6473.147363
3.78_188.0686n	187.0613041	3.779133333	neg	2-Oxosuberate	71247	Unclassified	Unclassified	Unclassified			39.1	0	M-H2O-H, M-H	C8H12O5	0.461423988	3322.511129	2782.318266	2852.292212	4472.674662	4221.915727	3531.593276	2861.284947	2779.094017	2876.82013
3.78_343.0964m/z	343.0964338	3.779133333	neg	Drosophilin C	LMFA01030753	Lipids and lipid-like molecules	Fatty Acyls	Fatty Acids and Conjugates			39.1	1.1	2M-H	C11H8O2	-3.338392964	5412.941373	4515.297028	5357.138528	1161.150531	1149.368123	660.6489034	1232.887162	1220.473237	890.2002011
3.80_150.0561m/z	150.0560549	3.801716667	neg	2-Amino-3-methylbenzoate	71241	Benzenoids	Benzene and substituted derivatives	Benzoic acids and derivatives	**	C16571	39.1	0	M-H	C8H9NO2	0.018578818	2004.908369	1625.495086	1660.361602	237.5244563	190.9311021	217.0002548	863.7920548	992.3568678	938.1726503
3.85_189.0770m/z	189.0769646	3.84605	neg	7-Oxoheptanoic acid	71248	Unclassified	Unclassified	Unclassified			39.1	0	M+FA-H	C7H12O3	0.815554122	1224.38746	1188.83299	1407.70577	1864.58712	1758.224452	1782.641179	1363.473696	1528.508732	1362.918551
3.89_172.0980m/z	172.0980315	3.8906	neg	N-Valerylglycine methyl ester	5650	Unclassified	Unclassified	Unclassified			39.1	0	M-H	C8H15NO3	0.66189727	1983.427298	2270.279672	2150.107607	526.5601401	409.1666652	543.9406048	1501.913485	1677.161641	1649.021419
3.96_118.0510m/z	118.0510134	3.957583333	neg	Aminoacetone	6502	Organic oxygen compounds	Organooxygen compounds	Carbonyl compounds	**	C01888	39.1	0	M+FA-H	C3H7NO	0.638854776	25.40418878	0.001403199	16.78899684	27.14392	20.97786368	31.73457802	35.78459754	25.84331098	19.62847146
4.00_256.1193m/z	256.1192519	4.002233333	neg	Isoproterenol	852	Benzenoids	Phenols	Benzenediols	**	C07056	39.1	0	M+FA-H	C11H17NO3	0.973731329	769.1076129	810.7188093	833.7232964	1335.286451	1299.816612	1324.20861	949.5479135	1114.022627	939.5539756
4.02_240.0516m/z	240.0515585	4.024283333	neg	Dopaquinone	354	Organic acids and derivatives	Carboxylic acids and derivatives	Amino acids, peptides, and analogues	**	C00822	39.1	0	M+FA-H	C9H9NO4	1.014438806	2272.181116	2068.18752	2336.885428	2200.433821	2141.062568	2247.256123	2435.431889	2487.511004	2500.588177
4.11_203.0927m/z	203.0926723	4.113883333	neg	4-Oxovalproic acid	2995	Organic acids and derivatives	Keto acids and derivatives	Gamma-keto acids and derivatives	**	C16655	39.1	0	M+FA-H	C8H14O3	1.107784786	1544.695108	1584.468167	1755.315538	1632.517201	1762.216433	1811.399541	1546.101973	1601.299954	1652.996806
4.11_391.0991m/z	391.0991475	4.113883333	neg	B-(4-Fluorobenzoyl)propionic acid	1287	Unclassified	Unclassified	Unclassified			39.1	0	2M-H	C10H9FO3	-1.838243117	1094.428763	882.5557534	1166.783338	1233.899835	1196.701433	980.1856906	850.5485385	996.7363358	928.4556868
4.18_196.0616m/z	196.0616271	4.181333333	neg	Acetaminophen	HMDB0001859	Benzenoids	Phenols	1-hydroxy-2-unsubstituted benzenoids	**	C06804	39.1	0	M+FA-H	C8H9NO2	0.63373492	990.4285911	794.4000524	869.3218081	356.2142424	204.1337984	302.6018006	603.0713189	371.7654507	433.2532424
4.18_367.0672m/z	367.0672407	4.181333333	neg	Elephantorrhizol	LMPK12020151	Lipids and lipid-like molecules	Polyketides	Flavonoids			39.1	0	M+FA-H	C15H14O8	0.529103755	3303.21052	3427.972266	2928.143347	432.4496328	343.9585696	292.3278701	5581.841996	4290.089359	4406.015256
4.19_143.0714m/z	143.0714171	4.189783333	neg	(Z)-3-Hexenal	HMDB0031498	Organic oxygen compounds	Organooxygen compounds	Carbonyl compounds	**	C16310	39.1	0	M+FA-H	C6H10O	0.502603184	1002.577824	881.8458366	941.2461796	957.1323835	978.7061008	1079.543404	1176.860357	970.5987854	1048.605663
4.26_197.0821m/z	197.0821104	4.2568	neg	3-(4-Hydroxyphenyl)-1-propanol	HMDB0032599	Benzenoids	Phenols	1-hydroxy-2-unsubstituted benzenoids	*		39.1	0	M+FA-H	C9H12O2	1.169986039	404.7071713	406.4069842	474.246751	456.9830912	404.2085914	500.3718178	361.5815318	300.880857	390.5021945
4.28_183.0300m/z	183.0299779	4.278183333	neg	2-Hydroxy-5-methylquinone	72151	Unclassified	Unclassified	Unclassified			39.1	0	M+FA-H	C7H6O3	0.586911071	761.2846102	655.3701771	760.8026424	1073.929249	993.2798432	965.6780063	776.5415432	864.9652902	913.2201709
4.28_189.1134m/z	189.1133598	4.278183333	neg	Hexyl acetate	HMDB0029980	Organic acids and derivatives	Carboxylic acids and derivatives	Carboxylic acid derivatives	*		39.1	0	M+FA-H	C8H16O2	0.882389367	2509.360685	2682.661077	2610.253306	2840.697243	2673.941879	2649.938012	2585.267205	2565.123904	2521.732276
4.32_301.0907m/z	301.0906778	4.3224	neg	Guanine	315	Organoheterocyclic compounds	Imidazopyrimidines	Purines and purine derivatives	**	C00242	39.1	0	2M-H	C5H5N5O	-2.864533622	1183.016395	1663.635878	1859.365663	1633.572793	1849.725976	1421.352805	984.9243708	1109.600315	1053.808509
4.34_127.0401m/z	127.0401042	4.344733333	neg	2-Methylglutaric acid	HMDB0000422	Lipids and lipid-like molecules	Fatty Acyls	Fatty acids and conjugates	*		39.1	0	M-H2O-H	C6H10O4	0.249904842	487.0023804	599.5462822	539.9309842	792.6878021	592.9007843	581.551331	536.7517105	622.5862865	670.819963
4.34_355.0779m/z	355.0778997	4.344733333	neg	Acromelic acid A	73445	Unclassified	Unclassified	Unclassified			39.1	0	M+FA-H	C13H14N2O7	-1.302732908	1328.717261	1717.677834	2003.256458	3199.061805	1726.870339	2078.399365	998.6210428	1513.65733	1421.842143
4.41_287.1502m/z	287.1501804	4.41115	neg	(S)-2-aceto-2-hydroxy-butanoic acid	45845	Unclassified	Unclassified	Unclassified			39.1	0	2M-H	C7H12O3	0.584342721	17544.11813	18672.8163	18054.05603	15991.7598	14409.95684	15288.9472	12546.1055	14379.56857	14244.31306
4.50_189.1134m/z	189.113412	4.49925	neg	Valproic acid	HMDB0001877	Lipids and lipid-like molecules	Fatty Acyls	Fatty acids and conjugates	**	C07185	39.1	0	M+FA-H	C8H16O2	1.244485086	457.579124	613.2253863	555.5321481	597.5313164	494.6826971	716.2915611	418.5119672	412.7571076	634.673076
4.54_318.0597m/z	318.0597043	4.54425	neg	N-Gluconyl ethanolamine phosphate	HMDB0032294	Organic acids and derivatives	Organic phosphoric acids and derivatives	Phosphate esters	*		39.1	0	M-H	C8H18NO10P	0.464733419	4337.201063	4570.418954	4737.927441	3121.213771	3310.210223	2911.784193	2739.870892	3304.315318	3320.433303
4.54_335.0387m/z	335.038692	4.54425	neg	D-glycero-D-manno-Heptose 1-phosphate	63659	Unclassified	Unclassified	Unclassified			39.1	0	M+FA-H	C7H15O10P	0.709516539	3357.521073	3532.487297	3628.244805	419.2886362	413.0251528	464.8971652	586.0825745	715.3532022	669.3612311
4.66_393.0802m/z	393.0801503	4.660483333	neg	4-Amino-2,6-dinitrotoluene	HMDB0246344	Unclassified	Unclassified	Unclassified			39.1	0	2M-H	C7H7N3O4	0.292558399	701.4814003	567.5365074	629.5356964	2583.01179	2103.765605	2375.250762	1583.632396	1598.512644	1136.037655
4.73_379.0650m/z	379.0649506	4.725416667	neg	alpha-D-Galactosyl-(1,1')-sn-glycerol 3-phosphate	66243	Unclassified	Unclassified	Unclassified			39.1	0	M+FA-H	C9H19O11P	0.747443186	7143.688167	7164.017371	7201.625091	5278.363711	5573.743196	5290.646398	8959.994284	9670.260768	9003.552407
4.75_247.1188m/z	247.1187945	4.747466667	neg	R-2-Hydroxy-3-methylbutanoic acid 3-Methylbutanoyl	LMFA07011009	Lipids and lipid-like molecules	Fatty Acyls	Fatty acid esters	*		39.1	0	M+FA-H	C10H18O4	0.40851939	5301.92835	4891.189541	5017.930566	4224.403082	4519.53172	4153.891375	3182.794142	3358.256863	3456.44992
4.79_175.0249m/z	175.0248795	4.792316667	neg	2-hydroxyacrylic Acid	HMDB0062676	Organic acids and derivatives	Carboxylic acids and derivatives	Carboxylic acids			39.1	0	2M-H	C3H4O3	0.385919373	596.6209221	716.5726025	759.3177417	980.8615152	939.6556559	985.7547642	688.1161579	804.0664483	730.7528069
4.79_563.0974m/z	563.097427	4.792316667	neg	3-O-Methylcoumestrol	HMDB0029584	Phenylpropanoids and polyketides	Isoflavonoids	Coumestans	*		39.1	0	2M-H	C16H10O5	-1.672462465	6857.229083	8478.617118	8904.980931	6228.586847	6530.21257	5676.830938	9132.017745	8802.737192	8817.910734
4.84_117.0193m/z	117.0192912	4.836566667	neg	beta-propiolactone	HMDB0249149	Unclassified	Unclassified	Unclassified			39.1	0	M+FA-H	C3H4O2	-0.569600647	223.7290586	179.2790994	210.5691477	213.3857033	207.1978263	194.7037561	299.3330338	227.6924626	225.5350716
4.88_192.0668m/z	192.0667793	4.880083333	neg	Phenylacetylglycine	4237	Organic acids and derivatives	Carboxylic acids and derivatives	Amino acids, peptides, and analogues	**	C05598	39.1	0	M-H	C10H11NO3	0.841869573	2442.980638	2695.148524	2543.345305	1110.425383	954.9798923	811.201543	2041.167984	1855.471882	2071.767803
4.88_210.1501m/z	210.1501044	4.880083333	neg	N-Butylscopolamine metabolite	2399	Unclassified	Unclassified	Unclassified			39.1	0	M-H	C12H21NO2	0.71928506	530.7527628	538.6116078	514.3996911	300.1752748	281.6836205	220.5074433	495.3715859	500.5944773	513.9542129
4.92_159.1027m/z	159.1027409	4.92485	neg	4-Heptanone	7078	Organic oxygen compounds	Organooxygen compounds	Carbonyl compounds	*		39.1	0	M+FA-H	C7H14O	0.639282053	816.2679524	801.4015138	893.0104445	1319.247192	1432.138498	1355.101292	1058.210615	1094.200646	1015.421418
5.01_425.0707m/z	425.0707019	5.014716667	neg	4-hydroxylamino-2,6-dinitrotoluene	HMDB0304181	Unclassified	Unclassified	Unclassified			39.1	0	2M-H	C7H7N3O5	1.965894772	944.78647	1020.077736	1449.967086	203.4162337	144.7976811	0.001403199	1405.785586	1208.882841	808.6081424
5.06_220.0981m/z	220.0980573	5.05975	neg	4-(Dimethylamino)cinnamaldehyde	85013	Unclassified	Unclassified	Unclassified			39.1	0	M+FA-H	C11H13NO	0.80164528	980.0704688	978.4092071	1006.897788	1264.101877	1032.511587	1126.026188	678.4644182	757.2813293	942.4887786
5.08_222.1502m/z	222.1501584	5.080566667	neg	Dihydrodioscorine	86533	Organoheterocyclic compounds	Azaspirodecane derivatives	Unclassified	*		39.1	0	M-H	C13H21NO2	0.922620729	267.2735057	288.584906	418.5643326	146.6612498	141.6623124	213.0902513	175.9765878	184.2346427	226.1844905
5.13_265.1195m/z	265.1194961	5.132616667	neg	Phenylalanylthreonine	HMDB0029005	Organic acids and derivatives	Carboxylic acids and derivatives	Amino acids, peptides, and analogues	*		39.1	2.54	M-H	C13H18N2O4	0.433816494	16948.94376	17229.3326	13857.23607	11066.11297	10414.58663	10880.23773	17033.15381	16318.68044	21189.70389
5.13_269.1507m/z	269.1507264	5.125666667	neg	Aspergillic acid	68394	Unclassified	Unclassified	Unclassified			39.1	0	M+FA-H	C12H20N2O2	0.203461382	2792.513734	2504.43724	1991.597113	3999.291092	4210.945659	4092.119152	2388.921134	2111.432868	3251.578054
5.20_245.1032m/z	245.1031605	5.20005	neg	Decenedioic acid	5578	Lipids and lipid-like molecules	Fatty Acyls	Fatty acids and conjugates	*		39.1	0	M+FA-H	C10H16O4	0.493085231	929.061459	908.6659494	813.4372027	1062.720213	1125.694125	1022.529733	1235.493451	1179.819444	1326.514431
5.26_377.1198m/z	377.1197684	5.263033333	neg	Glutarylglycine	5569	Organic acids and derivatives	Carboxylic acids and derivatives	Amino acids, peptides, and analogues	*		39.1	0	2M-H	C7H11NO5	-1.058022458	1266.16352	1189.909324	900.2352559	1554.524025	1633.513813	1510.69077	936.5561611	766.8088722	1065.007064
5.29_509.1256m/z	509.1256015	5.28555	neg	1-(Indol-3-yl)propanol 3-phosphate	66142	Unclassified	Unclassified	Unclassified			39.1	0	2M-H	C11H14NO4P	1.54637156	2584.714598	2222.978045	2049.788052	2419.241246	3012.358238	3230.600201	2704.708961	1881.504608	2779.245962
5.34_347.1714m/z	347.171381	5.3394	neg	(2R,6x)-7-Methyl-3-methylene-1,2,6,7-octanetetrol 2-glucoside	HMDB0033216	Lipids and lipid-like molecules	Fatty Acyls	Fatty acyl glycosides	*		39.1	0	M-H2O-H	C16H30O9	0.65432668	270.7649206	598.7456815	677.1169288	831.8713687	1013.874028	1228.535704	754.4557065	772.1957017	1194.559462
5.41_541.1009m/z	541.1009062	5.406433333	neg	Herbacetin 8-(2",3",4"-triacetylxyloside)	LMPK12113138	Lipids and lipid-like molecules	Polyketides	Flavonoids			39.1	12.3	M-H2O-H	C26H24O14	3.823905728	15880.29732	16537.80209	16917.44163	28942.12976	30642.19924	26914.3251	23172.28984	22857.27307	18281.94603
5.50_279.1351m/z	279.1351431	5.497816667	neg	2H-Indol-2-one, 1,3-dihydro-7-hydroxy-4-[2-(propylamino)ethyl]-	2371	Unclassified	Unclassified	Unclassified			39.1	0	M+FA-H	C13H18N2O2	0.480154665	3330.550573	2422.298985	2224.143686	1411.926441	1587.420023	2002.361607	1520.519047	1261.989276	1494.457013
5.53_148.0529m/z	148.0529151	5.525416667	neg	2-Fluoro-3-guanidinopropionic acid	689	Unclassified	Unclassified	Unclassified			39.1	0	M-H	C4H8FN3O2	0.918086225	699.2229008	579.8403736	595.6713991	161.0019941	217.9331853	191.758103	420.2132128	517.2089783	633.4120393
5.56_269.1008m/z	269.1008093	5.56185	neg	Adenine	85	Organoheterocyclic compounds	Imidazopyrimidines	Purines and purine derivatives	**	C00147	39.1	0	2M-H	C5H5N5	-3.349324798	4619.553622	3869.448981	4652.360069	3441.637872	4000.642482	3473.294949	5009.736557	4465.332629	4384.633917
5.59_347.1713m/z	347.1712728	5.5933	neg	Diethyl succinate	HMDB0033838	Lipids and lipid-like molecules	Fatty Acyls	Fatty acid esters	*		39.1	0	2M-H	C8H14O4	0.377321022	4702.390607	4523.118619	4501.101535	3089.734108	3223.569699	3404.988899	5261.893406	4911.999077	4122.654545
5.63_259.1551m/z	259.1551445	5.628083333	neg	3-Oxododecanoic acid	HMDB0010727	Organic acids and derivatives	Keto acids and derivatives	Medium-chain keto acids and derivatives	*	C02367	39.1	0	M+FA-H	C12H22O3	0.219876601	2066.914183	2139.157463	2442.989396	1865.053275	1825.205545	1763.104066	2039.327461	1389.727659	1928.70471
5.64_197.0821m/z	197.0820619	5.6386	neg	2-Isopropyl-1,4-benzenediol	HMDB0032132	Benzenoids	Benzene and substituted derivatives	Cumenes	*		39.1	0	M+FA-H	C9H12O2	0.851012744	2909.353665	2733.962277	3340.365738	2071.227585	1799.455229	1808.124699	2507.694143	2113.401351	2519.723521
5.65_159.1027m/z	159.102716	5.650833333	neg	Ethyl isobutyl ketone	HMDB0005841	Organic oxygen compounds	Organooxygen compounds	Carbonyl compounds	*		39.1	0	M+FA-H	C7H14O	0.420869781	510.5571567	305.2644721	492.8922475	449.7562843	391.677926	394.0393967	588.1589924	449.7845573	566.3155117
5.67_287.1502m/z	287.1502342	5.666566667	neg	trans-4-Hydroxycyclohexanecarboxylate	66175	Unclassified	Unclassified	Unclassified			39.1	0	2M-H	C7H12O3	0.770801803	3470.335029	3229.738953	3810.684704	5515.184232	5083.78165	5562.799344	3273.70942	3063.169299	3509.825754
5.69_223.0978m/z	223.0977605	5.6875	neg	Cyclohexane-1,3-dione	65556	Unclassified	Unclassified	Unclassified			39.1	0	2M-H	C6H8O2	0.794051378	933.0861169	875.1202722	730.7839538	644.8639604	779.8197394	675.780984	740.7174973	735.5985909	897.1001978
5.70_183.1027m/z	183.1026596	5.701283333	neg	exo-5,6-Dimethylbicyclo[2.2.1]hept-5-en-2-ol	HMDB0036091	Organic oxygen compounds	Organooxygen compounds	Alcohols and polyols	*		39.1	0	M+FA-H	C9H14O	-0.060640986	1877.136238	1872.591523	1925.492425	1653.767608	1791.665153	1784.24596	1916.572885	1801.964697	2182.578542
5.73_405.1257m/z	405.1257022	5.727283333	neg	Glutamyl-Glycine	85733	Organic acids and derivatives	Carboxylic acids and derivatives	Amino acids, peptides, and analogues	*		39.1	0	2M-H	C7H11N2O5-	-1.512577545	1103.659601	1289.404089	1162.263515	2540.657184	4154.460993	2993.479181	1053.15499	2099.283448	1967.624943
5.87_407.1930m/z	407.1929576	5.866533333	neg	Diethyl (2R,3R)-2-methyl-3-hydroxysuccinate	66216	Unclassified	Unclassified	Unclassified			39.1	0	2M-H	C9H16O5	1.68246981	1259.488417	1475.753135	1593.684054	1919.953945	1714.23041	1523.200772	2064.274371	1439.354649	2315.138141
5.92_357.1558m/z	357.1557538	5.9168	neg	Artelinic acid	1116	Unclassified	Unclassified	Unclassified			39.1	0	M+FA-H	C16H24O6	0.840735006	5883.292657	6202.649697	6167.44704	7406.269917	8347.797559	7201.816146	5577.381489	6771.785495	6862.191568
6.03_505.2088m/z	505.2088014	6.028183333	neg	Triamterene	2908	Organoheterocyclic compounds	Pteridines and derivatives	Unclassified		D00386	39.1	0	2M-H	C12H11N7	1.760196992	2984.212758	3702.666348	4622.811576	1252.993978	1308.847114	1309.980806	3174.458852	2902.306854	3144.172563
6.04_267.1240m/z	267.1239888	6.040433333	neg	Annuionone B	88204	Organoheterocyclic compounds	Oxepanes	Unclassified	*		39.1	0	M+FA-H	C13H18O3	0.861878478	4387.837692	4757.495489	5238.90458	6351.659473	6570.52855	6420.818336	4876.822387	5028.211678	5231.650445
6.17_347.1714m/z	347.1713818	6.171316667	neg	Tributyrin	69736	Lipids and lipid-like molecules	Glycerolipids	Triradylcglycerols	*	C13870	39.1	0	M+FA-H	C15H26O6	0.795631788	7151.054367	6512.558992	6995.413007	4653.591142	4441.312964	5184.600706	4735.934648	5376.611127	5771.312202
6.23_464.3021m/z	464.3020594	6.230633333	neg	Stearidonyl carnitine	LMFA07070106	Lipids and lipid-like molecules	Fatty Acyls	Fatty acid esters	*		39.1	0	M+FA-H	C25H41NO4	0.709950326	22142.21856	18922.98154	22688.89102	14385.61903	13126.84966	14741.28133	18331.84892	20154.92547	20477.96201
6.28_225.1134m/z	225.1134446	6.2811	neg	3-tert-Butyl-5-methylcatechol	66083	Unclassified	Unclassified	Unclassified			39.1	0	M+FA-H	C11H16O2	1.177093286	3900.767642	3600.435985	4298.061487	5805.599074	5550.131117	6127.669827	3849.143365	4052.118987	4768.072435
6.28_352.0863m/z	352.0863118	6.2811	neg	NAC-Diketide	69301	Unclassified	Unclassified	Unclassified			39.1	0	M+FA-H	C15H17NO4S	0.911615151	114621.0029	117049.2547	131121.074	192125.0476	199836.0114	194464.6534	212354.7111	234866.0278	238183.554
6.32_297.1322m/z	297.132211	6.320716667	neg	3-Methyladenine	HMDB0011600	Organoheterocyclic compounds	Imidazopyrimidines	Purines and purine derivatives	*	C00913	39.1	0	2M-H	C6H7N5	-2.693673771	2043.966752	2859.805872	2590.339038	2248.920487	2434.271133	2234.070857	1949.857543	1901.894161	2411.748735
6.32_419.1096m/z	419.1095904	6.320716667	neg	Portulacaxanthin II	64473	Organic acids and derivatives	Carboxylic acids and derivatives	Amino acids, peptides, and analogues	*		39.1	0	M+FA-H	C18H18N2O7	-0.03585499	825.6275447	1254.888501	965.144891	1051.654236	1389.180821	1122.599357	738.5541209	500.4795437	874.2760932
6.37_272.1292m/z	272.1292318	6.373833333	neg	4'-O-Methylnorbelladine	64406	Unclassified	Unclassified	Unclassified	***		39.1	0	M-H	C16H19NO3	0.054133834	347.3582565	376.413311	348.726251	791.0689945	649.6584173	816.955747	715.4365358	790.5198892	782.1288352
6.54_489.1745m/z	489.1744519	6.539816667	neg	Debromohymenialdisine	73418	Unclassified	Unclassified	Unclassified			39.1	0	2M-H	C11H11N5O2	-1.674582055	1846.827612	1372.595651	1800.121526	2305.48174	2573.615444	2477.692526	1657.166577	2362.888404	2213.349008
6.57_157.1234m/z	157.1234149	6.570233333	neg	(E)-2-Octene	HMDB0061904	Hydrocarbons	Unsaturated hydrocarbons	Unsaturated aliphatic hydrocarbons			39.1	0	M+FA-H	C8H16	0.102318676	574.4877469	642.6636479	661.3528906	681.6733138	650.9968381	640.754528	501.0375007	555.8940775	487.046203
6.57_517.0990m/z	517.099033	6.570233333	neg	Tetracenomycin C	63815	Unclassified	Unclassified	Unclassified			39.1	0	M+FA-H	C23H20O11	0.569086412	35316.40281	32052.78688	30273.97128	31223.98708	33503.14301	29456.9545	35116.94684	33640.34938	37625.70763
6.62_237.1134m/z	237.1133978	6.61635	neg	2-Methyl-1-phenyl-2-propanyl acetate	87776	Benzenoids	Benzene and substituted derivatives	Phenylpropanes	*		39.1	0	M+FA-H	C12H16O2	0.859925984	1596.748907	1674.031929	1529.214986	2279.869413	2191.695164	2290.047376	1773.44345	2082.063764	2004.766187
6.83_505.2661m/z	505.266097	6.825	neg	(3b,9R)-5-Megastigmene-3,9-diol 9-[apiosyl-(1->6)-glucoside]	LMFA13010056	Lipids and lipid-like molecules	Fatty Acyls	Fatty acyl glycosides	*		39.1	2.56	M-H	C24H42O11	1.306067619	33730.18137	31566.37916	32120.10274	29449.5573	28213.48285	28717.32648	41721.25934	39815.10494	35888.39097
6.90_193.0354m/z	193.0354078	6.896766667	neg	D-Xylono-1,5-lactone	62415	Organoheterocyclic compounds	Lactones	Delta valerolactones	**	C02266	39.1	0	M+FA-H	C5H8O5	0.213241724	2370.136908	2595.13289	2422.852996	3399.928742	3551.102889	3062.908227	2190.72872	2575.076426	2481.846624
6.94_269.1396m/z	269.1395502	6.939266667	neg	Methyl epijasmonate	HMDB0301800	Lipids and lipid-like molecules	Fatty Acyls	Lineolic acids and derivatives	**	C11512	39.1	0	M+FA-H	C13H20O3	0.458688704	4279.331128	5026.77544	4751.275111	4828.144157	4408.552201	4193.626415	3298.051772	3960.979502	4100.054036
6.97_355.1406m/z	355.1405544	6.970716667	neg	4-Oxo-4-(3-pyridyl)-butanamide	6122	Organic oxygen compounds	Organooxygen compounds	Carbonyl compounds	*		39.1	0	2M-H	C9H10N2O2	-1.752934392	733.5080329	695.7799722	738.5255689	1134.690997	906.3648577	1053.626191	585.1725673	636.993337	751.0618279
7.10_201.1134m/z	201.1134335	7.097333333	neg	2,4-Nonanedione	87536	Organic oxygen compounds	Organooxygen compounds	Carbonyl compounds	*		39.1	0	M+FA-H	C9H16O2	1.286654342	4771.095754	4787.572774	5194.133361	3799.598147	3891.162113	3670.40924	4650.805165	5064.063339	4568.454209
7.16_467.1514m/z	467.1514185	7.1647	neg	4,4'-methanol-bisbenzonitrile	HMDB0061037	Benzenoids	Benzene and substituted derivatives	Diphenylmethanes	*		39.1	0	2M-H	C15H10N2O	0.147445766	4943.674978	5503.048087	5162.160413	6618.62238	6142.106051	6243.169456	5582.946722	7471.887284	6065.077331
7.20_475.2680m/z	475.2680489	7.201166667	neg	2-(2-Methylbutanoyl)-9-(3-methyl-2E-pentenoyl)-2b,9a-dihydroxy-4Z,10(14)-oplo	HMDB0040994	Lipids and lipid-like molecules	Prenol lipids	Sesquiterpenoids	*		39.1	4.16	M+FA-H	C26H38O5	-4.830098099	45422.83583	48280.32159	47791.85333	35398.65355	36763.13569	37625.93751	28346.20614	34281.19636	28829.6259
7.42_411.2027m/z	411.2026883	7.41815	neg	Phenylethylmalonamide (PEMA)	2084	Unclassified	Unclassified	Unclassified			39.1	0	2M-H	C11H14N2O2	-2.645864487	6691.677152	6610.495651	7937.902094	2751.652739	2958.122234	3025.636286	5187.863998	5280.918178	5251.918105
7.47_245.1396m/z	245.139586	7.473983333	neg	3-Nonanon-1-yl acetate	HMDB0037179	Lipids and lipid-like molecules	Fatty Acyls	Fatty alcohol esters	*		39.1	0	M+FA-H	C11H20O3	0.692544439	2417.858815	2494.739545	2700.19561	3638.569699	4197.45008	3790.408991	1878.781563	2484.59046	2126.722867
7.49_446.1222n	491.1199141	7.4852	neg	Prunitrin	HMDB0034149	Phenylpropanoids and polyketides	Isoflavonoids	Isoflavonoid O-glycosides			39.1	0.705	M-H, M+FA-H	C22H22O10	2.121241636	156459.5524	144526.312	141660.722	140179.6972	158286.4043	145523.2277	165071.6148	159628.9093	162841.4446
7.59_183.0664m/z	183.0663916	7.58625	neg	4-Formyl-3-(formylmethyl)-4-hexenoic acid	LMFA01030984	Lipids and lipid-like molecules	Fatty Acyls	Fatty acids and conjugates	*		39.1	0	M-H	C9H12O4	0.593043829	372.3303433	327.3571826	347.7535274	392.433134	393.6340853	360.7093495	533.1606421	370.0223394	370.4495111
7.65_343.1401m/z	343.1401077	7.65305	neg	trans-3-Octenedioic acid	HMDB0013312	Lipids and lipid-like molecules	Fatty Acyls	Fatty acids and conjugates	*		39.1	0	2M-H	C8H12O4	0.774171309	3695.333812	3846.911516	3761.168661	2568.091198	2323.222382	2308.935565	2845.971318	3020.792278	3082.426695
7.72_145.0507m/z	145.0507093	7.719916667	neg	(R)-4-Dehydropantoate	63892	Lipids and lipid-like molecules	Fatty Acyls	Fatty Acids and Conjugates	**	C01053	39.1	0	M-H	C6H10O4	0.526712188	269.9172146	331.9289914	281.0748094	241.1295435	295.1219982	257.0100655	275.733292	269.1434308	331.6074168
7.75_229.1446m/z	229.1445586	7.747083333	neg	trans-2-Hexyl-1-cyclopropaneacetic acid	HMDB0031087	Organic acids and derivatives	Carboxylic acids and derivatives	Carboxylic acids	*		39.1	0	M+FA-H	C11H20O2	0.140233889	2068.01989	2367.54588	2038.692416	2592.893429	2722.024231	2631.067231	2237.04425	2118.198349	2307.858899
7.76_226.1450m/z	226.1450438	7.756566667	neg	N-Ethyl trans-2-cis-6-nonadienamide	HMDB0032273	Lipids and lipid-like molecules	Fatty Acyls	Fatty amides	**	C06551	39.1	0	M+FA-H	C11H19NO	0.975667144	3220.807305	3740.403577	3591.585683	4643.240315	4585.967646	4324.085103	3079.065865	3078.166349	3293.971376
7.78_427.1326m/z	427.1325836	7.7828	neg	ETANIDAZOLE	44130	Unclassified	Unclassified	Unclassified			39.1	0	2M-H	C7H10N4O4	-1.283752937	1114.707456	1157.538071	1613.336395	1815.083304	1302.749589	2102.977878	1595.431938	1343.51492	1632.885653
7.98_543.2556m/z	543.2556066	7.984516667	neg	Sempervirine	67546	Unclassified	Unclassified	Unclassified			39.1	0	2M-H	C19H16N2	0.341636109	5594.880262	5565.553908	6498.121344	545.8820049	242.5672516	368.0932715	2182.389734	2100.324195	2119.62967
8.02_221.0821m/z	221.0820716	8.01695	neg	Cinnamyl acetate	HMDB0029699	Benzenoids	Benzene and substituted derivatives	Styrenes	*	C12299	39.1	0	M+FA-H	C11H12O2	0.790277844	6363.26484	4847.341234	4648.671918	6063.029316	7139.867817	7331.434843	5981.485211	6102.051035	6107.199717
8.09_395.1713m/z	395.1712689	8.087016667	neg	5-(3E-Pentenyl)tetrahydro-2-oxo-3-furancarboxylic acid	87335	Organoheterocyclic compounds	Lactones	Gamma butyrolactones	*		39.1	0	2M-H	C10H14O4	0.321708667	1974.402926	2051.34733	2265.947166	2180.451442	2354.163359	2001.433332	1184.530097	1919.905369	1761.644657
8.42_395.2077m/z	395.2076591	8.421216667	neg	8-iso-15-keto-PGE2	HMDB0002341	Lipids and lipid-like molecules	Fatty Acyls	Eicosanoids	*		39.1	0	M+FA-H	C20H30O5	0.377345433	33481.10717	36519.76312	36243.23276	14553.21637	14710.44894	13754.0928	23142.29525	27249.48126	26368.89311
9.25_397.1870m/z	397.1869961	9.248216667	neg	Idebenone Metabolite (QS-10)	758	Unclassified	Unclassified	Unclassified			39.1	0	M+FA-H	C19H28O6	0.580915444	32326.24751	32987.95873	33581.49731	51622.82014	50866.34138	51686.65975	20846.57995	22282.66793	21576.96339
9.76_397.2233m/z	397.2233304	9.7627	neg	Prostaglandin E2	HMDB0001220	Lipids and lipid-like molecules	Fatty Acyls	Eicosanoids	***	C00584	39.1	0	M+FA-H	C20H32O5	0.435620374	41104.18803	44930.93785	45518.00194	37095.74335	36887.49201	36883.84949	36247.01319	41682.71399	38901.907
0.54_283.2645m/z	283.2644643	0.535283333	neg	2-Methyl-4-heptanone	85275	Organic oxygen compounds	Organooxygen compounds	Carbonyl compounds	*		39	0	2M-H	C9H18O	0.739825071	909.8688183	1292.247523	1121.536943	1074.340251	927.8646988	590.0291746	1250.492145	868.5634184	1002.436456
0.74_152.0579m/z	152.0579302	0.7359	neg	FAPy-adenine	7080	Organoheterocyclic compounds	Diazines	Pyrimidines and pyrimidine derivatives	*	C06502	39	0	M-H	C5H7N5O	0.958949046	519.9140688	424.2284718	992.1934236	695.4261401	963.449523	583.9943917	828.1421556	675.6395721	1026.336855
0.74_417.0122m/z	417.0121963	0.7359	neg	4-Fluoro-3-nitrobenzotrifluoride	85169	Unclassified	Unclassified	Unclassified			39	0	2M-H	C7H3F4NO2	-1.219318207	1884.126877	1615.049042	5564.470432	457.2367437	2223.60833	535.2203967	4363.02241	1994.517063	5629.073012
0.76_251.0522m/z	251.0522232	0.757316667	neg	Cysteinyl-Methionine	HMDB0028781	Organic acids and derivatives	Carboxylic acids and derivatives	Amino acids, peptides, and analogues	*		39	7.25	M-H	C8H16N2O3S2	-2.914883852	3286.941606	2833.529409	3651.404646	12174.25653	13174.81471	10755.13235	3604.408515	3890.084729	4143.065122
0.80_439.0758m/z	439.0758213	0.800366667	neg	5'-Butyrylphosphouridine	66401	Unclassified	Unclassified	Unclassified			39	0	M+FA-H	C13H19N2O10P	-0.286839405	114939.4487	138867.7306	145819.2194	98254.22996	97631.90733	94486.34244	123999.1424	100214.4861	114097.4074
0.82_151.0612m/z	151.0612053	0.819566667	neg	Trihydroxybutane	HMDB0061944	Organic acids and derivatives	Orthocarboxylic acid derivatives	Unclassified			39	0	M+FA-H	C4H10O3	0.077825239	892.037401	1053.403542	935.7132485	1489.707263	1102.568016	1395.899139	743.3977926	815.4515878	722.592956
0.84_143.0462m/z	143.0462197	0.841733333	neg	4-Hydroxymethylpyrazole	HMDB0060768	Organoheterocyclic compounds	Azoles	Pyrazoles	*		39	0	M+FA-H	C4H6N2O	0.040881762	526.979657	439.5635609	661.5017522	529.2483176	374.8995874	576.1821622	643.7340056	635.2502342	579.1392555
0.84_295.0731m/z	295.0730865	0.841733333	neg	3,3'-Dimethoxybenzidine-4,4'-diisocyanate	73106	Unclassified	Unclassified	Unclassified			39	0	M-H	C16H12N2O4	2.215896571	150.9037422	406.0465424	1134.275861	553.4823243	1220.247738	838.5155395	116.5328366	851.5966777	1308.214804

0.86_291.0955n	290.088224	0.86335	neg	2,7-Anhydro-alpha-N-acetylneuraminic acid	66198	Unclassified	Unclassified	Unclassified			39	0	M-H, M+FA-H	C11H17NO8	0.288335946	27463.58005	26985.38488	31866.34511	27676.98164	29890.81065	29359.9314	27270.15337	29359.1314	28336.59478
0.88_112.0404m/z	112.0404263	0.884633333	neg	Pyrrole	HMDB0035924	Organoheterocyclic compounds	Heteroaromatic compounds	Unclassified		C19907	39	0	M+FA-H	C4H5N	0.362302921	548.2468433	657.4590725	710.3518716	796.6738649	577.514754	725.0311406	815.0057644	848.5315068	607.1492709
0.88_155.0098m/z	155.0098425	0.884633333	neg	Uracil 5-carboxylate	65886	Unclassified	Unclassified	Unclassified			39	0	M-H	C5H4N2O4	0.079164073	2041.852054	2136.919008	2843.349211	2305.686312	2613.103892	2467.477062	1963.133403	2592.844548	2308.172326
0.90_187.0725m/z	187.0725278	0.904683333	neg	L-glycyl-L-hydroxyproline	62021	Unclassified	Unclassified	Unclassified			39	0	M-H	C7H12N2O4	0.517603979	5177.852582	5656.056101	6003.843599	4822.093843	6080.915968	5706.856549	8004.263435	8007.175789	9304.830605
1.03_129.0306m/z	129.0306307	1.033516667	neg	Imidazolone	HMDB0004363	Organic acids and derivatives	Carboxylic acids and derivatives	Amino acids, peptides, and analogues	**	C06195	39	0	M+FA-H	C3H4N2O	0.774035234	28.64620636	20.49221084	0.001403199	39.42196933	64.92206444	55.13915037	99.81050847	105.0975631	75.89143305
1.16_132.0666m/z	132.06663	1.160166667	neg	4-Aminobutyraldehyde	HMDB0001080	Organic oxygen compounds	Organooxygen compounds	Carbonyl compounds	*	C02903	39	0	M+FA-H	C4H9NO	0.151705188	200.9577186	310.3394966	272.0583169	441.6868921	266.1244557	91.75201263	430.2395	186.2452602	223.5212755
1.16_290.0883m/z	290.0882979	1.160166667	neg	9-O-Acetylneuraminic acid	3320	Unclassified	Unclassified	Unclassified			39	0	M-H2O-H	C11H19NO9	0.510549272	3412.450199	3921.054073	4912.421705	2391.321816	3451.775213	2386.873093	2723.634878	2112.969271	2975.782197
1.20_159.0300m/z	159.0299927	1.202333333	neg	Norfuraneol	HMDB0031859	Organoheterocyclic compounds	Dihydrofurans	Furanones	*		39	0	M+FA-H	C5H6O3	0.839525709	1444.490726	1689.075405	1951.349008	1512.836413	1437.470759	1534.561544	1566.524234	1909.86872	1733.168811
1.20_359.0988m/z	359.0988078	1.202333333	neg	Vanilloside	HMDB0029664	Organic oxygen compounds	Organooxygen compounds	Carbohydrates and carbohydrate conjugates	*		39	0	M+FA-H	C14H18O8	1.392628789	7137.137181	7578.671321	7132.745181	7079.949334	7201.21672	7590.452427	6876.742902	7225.997654	7089.485998
1.45_339.0812m/z	339.0811783	1.4461	neg	5-Ureido-4-imidazole carboxylate	63403	Unclassified	Unclassified	Unclassified			39	0	2M-H	C5H6N4O3	1.395395816	734.9221816	1100.350585	633.0170575	873.1592788	575.6315111	707.1483075	707.2319528	643.5325784	620.2692447
1.74_204.0879m/z	204.0878639	1.736816667	neg	5-Acetamidovalerate	62836	Lipids and lipid-like molecules	Fatty Acyls	Fatty acids and conjugates	**	C03087	39	0	M+FA-H	C7H13NO3	0.740209746	2000.442495	2089.823234	1826.339124	1252.52671	1225.681815	1575.292819	1806.515035	2126.747277	2146.193884
1.82_326.0885m/z	326.0885446	1.820883333	neg	4-Hydroxyphenylacetylglutamic acid	HMDB0006061	Organic acids and derivatives	Carboxylic acids and derivatives	Amino acids, peptides, and analogues	**	C05595	39	0	M+FA-H	C13H15NO6	1.439135774	45.07481319	69.0649272	57.28674213	4113.429533	4176.468891	4614.834783	0.001403199	0.001403199	0.001403199
10.21_459.2028m/z	459.2027976	10.20653333	neg	Armillaripin	HMDB0030404	Lipids and lipid-like molecules	Prenol lipids	Sesquiterpenoids	*		39	0	M+FA-H	C24H30O6	0.859485837	11241.52285	9290.016823	9509.159213	11804.07735	15516.82795	16687.84496	12167.40743	12236.48477	11124.8799
10.78_491.1202m/z	491.1202057	10.78066667	neg	2-Hydroxy-4-imino-2,5-cyclohexadienone	87887	Organic nitrogen compounds	Organonitrogen compounds	Quinonimines	*		39	0	2M-H	C12H10N2O4	-1.283221257	1900.633401	1757.5818	1740.513621	1513.657599	1704.434082	1767.51121	3033.150672	1685.119333	2379.093942
11.00_233.1548m/z	233.1548219	10.99596667	neg	3(4->5)-Abeo-4,11:4,12-diepoxy-3-eudesmanol	88205	Organoheterocyclic compounds	Oxepanes	Unclassified	*		39	0	M-H2O-H	C15H24O3	0.469453811	27721.63796	23189.30418	23127.33307	25151.79482	25122.06473	27151.75116	27575.39332	24055.17802	24775.12724
11.82_838.5604m/z	838.5603683	11.81565	neg	PC(22:5(4Z,7Z,10Z,13Z,16Z)/15:0)	HMDB0008658	Lipids and lipid-like molecules	Glycerophospholipids	Glycerophosphocholines	**	C00157	39	0	M+FA-H	C45H80NO8P	0.012934913	2764.063581	1947.245197	2723.791804	2197.511552	2042.098412	745.417309	6445.545346	3725.728582	3547.877065
15.19_297.2798m/z	297.2798252	15.19468333	neg	3-Cyclohexyldodecane	HMDB0036345	Hydrocarbons	Saturated hydrocarbons	Cycloalkanes			39	0	M+FA-H	C18H36	-0.312631014	760.7558546	563.3758066	706.545576	369.1637164	275.0918809	565.4030544	438.7089793	528.9425279	624.3610355
2.00_351.0789m/z	351.0788653	2.004766667	neg	L-2-Amino-3-(oxalylamino)propanoic acid	HMDB0029402	Organic acids and derivatives	Carboxylic acids and derivatives	Amino acids, peptides, and analogues	*	C04209	39	0.377	2M-H	C5H8N2O5	-1.422959196	22701.28116	22806.09942	23112.12916	16538.3806	13341.81945	14261.85849	15964.55516	19737.30809	16300.55473
2.07_129.0194m/z	129.019368	2.070366667	neg	2(5H)-Furanone	71806	Organoheterocyclic compounds	Dihydrofurans	Furanones	*	C17601	39	0	M+FA-H	C4H4O2	0.425109445	769.8270062	918.6242807	832.8463397	990.3707423	866.50095	955.3750253	948.6076085	853.3160296	816.1974123
2.23_187.0613m/z	187.061328	2.227683333	neg	5-Ethyl-3-hydroxy-4-methyl-2(5H)-furanone	89361	Organoheterocyclic compounds	Dihydrofurans	Furanones			39	0	M+FA-H	C7H10O3	0.921468267	1474.356434	1439.92596	1682.837566	1624.137254	1738.469461	1835.817883	1351.966263	1166.290503	1297.230123
2.26_125.0357m/z	125.0356589	2.26065	neg	Pyrimidine	HMDB0003361	Organoheterocyclic compounds	Diazines	Pyrimidines and pyrimidine derivatives	*	C00396	39	0	M+FA-H	C4H4N2	0.099222342	236.7293497	221.8557683	189.6699973	374.4590045	322.2062669	242.8938841	290.143381	232.2587334	215.8064442
2.59_152.0354m/z	152.0353857	2.594616667	neg	Nitrosobenzene	HMDB0255658	Unclassified	Unclassified	Unclassified			39	0	M+FA-H	C6H5NO	0.644929029	1561.494589	1751.671035	1940.733942	2116.167103	2116.75419	2152.368755	1380.347553	1281.037338	1608.623198
2.73_116.0717m/z	116.0717341	2.734316667	neg	3-Buten-1-amine	69390	Unclassified	Unclassified	Unclassified			39	0	M+FA-H	C4H9N	0.449839346	213.502433	213.1662218	267.3671181	256.0824372	308.0757345	275.3748134	348.1079912	397.548082	322.8497525
2.76_145.0507m/z	145.0507288	2.75705	neg	3-Methylbutyrolactone	6521	Organoheterocyclic compounds	Lactones	Gamma butyrolactones	*		39	0	M+FA-H	C5H8O2	0.963513886	2852.557089	2582.65741	2705.375967	2947.266313	2729.914321	2847.390243	2638.514073	2710.896199	2812.71115
2.89_531.2244m/z	531.2244178	2.8859	neg	(±)-Goniothalesdiol	44860	Unclassified	Unclassified	Unclassified			39	0	2M-H	C14H18O5	1.591162015	1069.344169	1406.24022	1348.364034	1367.491779	1158.842977	1703.699026	1754.900338	1244.139544	1477.133543
2.93_143.0348m/z	143.0348267	2.926066667	neg	Glycidaldehyde	72967	Unclassified	Unclassified	Unclassified			39	0	2M-H	C3H4O2	-1.080179374	251.7848228	208.7276913	209.0838591	355.6347498	258.3135014	312.4301703	313.1051565	292.296537	286.0737322
3.11_232.0827m/z	232.0827212	3.112733333	neg	2-Keto-6-acetamidocaproate	62815	Organic acids and derivatives	Keto acids and derivatives	Medium-chain keto acids and derivatives	**	C05548	39	0	M+FA-H	C8H13NO4	0.32289822	3364.348465	3469.120411	3788.932803	4332.37731	3853.407955	3780.847536	3521.670684	3794.255752	4210.665382
3.13_233.0044m/z	233.0044312	3.131933333	neg	3-Nitroacrylate	65733	Unclassified	Unclassified	Unclassified			39	0	2M-H	C3H3NO4	-3.023281725	1714.019394	1666.058198	1982.859853	1689.424813	1630.998157	1643.957667	2390.041157	2524.835329	2475.79877
3.47_271.0937m/z	271.093737	3.47105	neg	Carbidopa	1500	Phenylpropanoids and polyketides	Phenylpropanoic acids	Unclassified			39	0	M+FA-H	C10H14N2O4	0.783659978	345.9060465	528.0343974	699.2800558	872.6343557	698.1395835	689.2057058	696.8666612	678.8823991	715.7279583
3.55_187.0613m/z	187.0613192	3.549183333	neg	5-Heptynoic acid, 7-hydroxy-; 7-Hydroxy-5-heptynoic acid	74626	Unclassified	Unclassified	Unclassified			39	0	M+FA-H	C7H10O3	0.859959604	1577.041588	1666.668993	1566.480866	2526.761681	2813.148618	2575.341919	1797.462413	1880.64558	1868.35721
3.56_184.0615m/z	184.0615378	3.564783333	neg	Ammonium benzoate	89228	Unclassified	Unclassified	Unclassified			39	0	M+FA-H	C7H9NO2	0.04588888	1151.55675	1327.935446	1123.181446	943.1836953	1105.376059	1005.989174	1057.265679	1118.187505	976.9760156
3.69_253.0111m/z	253.0110851	3.685866667	neg	Penem	HMDB0256222	Alkaloids and derivatives	Camptothecins	Unclassified			39	0	2M-H	C5H5NOS	-0.03183614	4750.890676	5393.845499	5050.025895	8175.334447	8084.115898	6002.492901	6190.030945	6563.761134	6281.788981
3.71_327.0723m/z	327.0722588	3.708366667	neg	6-[4-(carboxymethyl)phenoxy]-3,4,5-trihydroxyoxane-2-carboxylic acid	HMDB0132498	Organic oxygen compounds	Organooxygen compounds	Carbohydrates and carbohydrate conjugates	*		39	0	M-H	C14H16O9	0.314346685	3237.335667	3253.549714	3210.821173	4600.06321	4847.426084	3855.698449	3489.113902	3589.405821	3973.501291
3.75_272.0181m/z	272.018062	3.747833333	neg	L-Glutamyl 5-phosphate	63480	Organic acids and derivatives	Carboxylic acids and derivatives	Amino acids, peptides, and analogues	**	C03287	39	0	M+FA-H	C5H10NO7P	1.63337145	595.7934824	645.4930067	538.8722146	1482.258154	1279.317955	683.3637588	550.4683983	916.9596187	641.8120379
3.85_302.1612m/z	302.1612027	3.84605	neg	3-Methyladipoylcarnitine	HMDB0240666	Lipids and lipid-like molecules	Fatty Acyls	Fatty acid esters			39	0	M-H	C14H25NO6	0.962031705	476.9156934	519.6778944	681.6617136	512.0394344	493.4446074	640.2176632	458.1388052	601.063983	558.9826299
3.85_371.0985m/z	371.0985173	3.84605	neg	Erinapyrone C	HMDB0041026	Organoheterocyclic compounds	Pyrans	Pyranones and derivatives			39	0	2M-H	C8H10O5	0.394688614	794.6219884	985.3569971	1169.847466	770.7927085	655.517339	747.2088921	984.8297688	804.3946114	812.102419
3.85_385.1143m/z	385.1142519	3.84605	neg	Linocinnamarin	87136	Organic oxygen compounds	Organooxygen compounds	Carbohydrates and carbohydrate conjugates	*		39	0	M+FA-H	C16H20O8	0.680525203	776.9899383	1041.449638	975.9056649	225.2078173	229.5408986	277.3047465	2353.891541	2974.861118	3082.622073
3.96_285.1458m/z	285.1458005	3.957583333	neg	Pirbuterol	66744	Organoheterocyclic compounds	Pyridines and derivatives	Hydroxypyridines		C07807	39	0	M+FA-H	C12H20N2O3	0.854246007	1316.775328	1317.421521	1401.947065	3143.363058	2604.322812	3018.249064	1460.973527	1515.376709	1692.345407
4.00_471.1509m/z	471.1508698	4.002233333	neg	N'-Formylkynurenine	HMDB0001200	Organic oxygen compounds	Organooxygen compounds	Carbonyl compounds	*	C02406	39	0	2M-H	C11H12N2O4	-2.68443922	1189.658616	1127.437308	1511.125991	1163.660169	1065.004584	791.5395327	1178.305999	1375.492683	1204.578033
4.02_199.0615m/z	199.0614956	4.024283333	neg	Sylvopinol	87067	Benzenoids	Phenols	Methoxyphenols	*		39	0	M+FA-H	C8H10O3	1.937698693	836.9058053	875.2852548	891.7418013	286.7963623	437.0820288	459.1981607	719.7561727	911.2385261	747.9080105
4.02_335.0387m/z	335.038745	4.024283333	neg	Sedoheptulose 1-phosphate	66357	Organic oxygen compounds	Organooxygen compounds	Carbohydrates and carbohydrate conjugates		C06222	39	0	M+FA-H	C7H15O10P	0.892503261	2194.936565	2619.166721	2586.479824	175.2157626	301.6910692	174.8068728	398.1907221	393.1020498	257.8647445
4.11_261.0880m/z	261.0879677	4.113883333	neg	desethyletomidate	2645	Unclassified	Unclassified	Unclassified			39	0	M+FA-H	C12H12N2O2	-0.521992286	2657.759014	2912.362818	3137.523127	3684.822779	3379.981408	3550.19966	2744.21774	3097.412374	2805.080109
4.14_411.1511m/z	411.151117	4.136366667	neg	Diethyl tartrate	89393	Organic acids and derivatives	Hydroxy acids and derivatives	Beta hydroxy acids and derivatives			39	0	2M-H	C8H14O6	0.769325515	1956.730297	2055.259374	2446.411233	1143.712383	1024.107532	1279.929706	2098.651992	2055.519504	1976.473717
4.16_268.0828m/z	268.0827571	4.15895	neg	bendiocarb	HMDB0248947	Unclassified	Unclassified	Unclassified			39	0	M+FA-H	C11H13NO4	0.431617936	1611.372254	1718.560765	1799.890541	2003.512779	1292.963976	1173.768841	1824.616841	1387.375544	1165.788117
4.19_218.9970m/z	218.9969615	4.189783333	neg	4-Hydroxybenzenesulfonic acid	34512	Unclassified	Unclassified	Unclassified			39	0	M+FA-H	C6H6O4S	0.45390928	605136.269	561371.8432	586053.7396	114771.9275	107625.7739	131495.33	556284.4509	523309.2714	508905.2372
4.19_339.0722m/z	339.0721862	4.189783333	neg	Cyclic de-hypoxanthine futalosine	63996	Unclassified	Unclassified	Unclassified			39	0	M+FA-H	C14H14O7	0.104002114	4237.927401	3060.390629	3522.035525	837.0042094	820.8325869	1058.916558	2419.98956	2140.453323	2201.531506
4.30_230.1035m/z	230.1034759	4.300033333	neg	Pseudoecgonine	HMDB0006348	Alkaloids and derivatives	Tropane alkaloids	Unclassified	*	C12449	39	0	M+FA-H	C9H15NO3	0.430367855	1844.744912	2047.024112	2005.765581	3137.724857	2585.224786	2495.196841	2258.405661	2787.415896	2066.588234
4.37_445.1603m/z	445.1603225	4.367166667	neg	Salsolinol 1-carboxylate	HMDB0013068	Organoheterocyclic compounds	Tetrahydroisoquinolines	Unclassified	*		39	0	2M-H	C11H13NO4	-2.951403434	792.4145136	1045.254223	866.0133342	1727.50709	1175.704707	1352.48253	1103.695934	1038.438515	859.5211232
4.41_532.1316m/z	532.1316189	4.41115	neg	cyclo-Dopa-glucuronylglucoside	64477	Unclassified	Unclassified	Unclassified			39	0	M-H	C21H27NO15	1.549640638	50187.88365	50931.28599	51018.02747	21237.04885	17602.14014	16312.06893	44540.25673	47088.96534	47608.69692
4.48_281.0305m/z	281.0304993	4.477166667	neg	5-hydroxymethyl-2-furanoate	86075	Organoheterocyclic compounds	Furans	Furoic acid and derivatives	*	C20448	39	0	2M-H	C6H5O4-	0.739035686	603.7804289	425.230709	396.4651454	469.2476212	550.0422533	553.5686855	565.1813104	642.2451229	683.4145196
4.52_226.0841n	225.0745228	4.521733333	neg	3,4,5-Trimethoxyphenyl acetate	87894	Benzenoids	Phenol esters	Unclassified	*		39	0	M-H2O-H, M-H	C11H14O5	0.054749909	1161.889803	1274.278817	1220.358337	1145.793539	1149.155958	1153.901943	1824.506366	1940.294249	2130.630897
4.61_535.1709m/z	535.1708528	4.611883333	neg	Benzo[a]pyrene-9,10-oxide	HMDB0060090	Benzenoids	Pyrenes	Unclassified	**	C14849	39	0	2M-H	C20H12O	0.931160575	7738.769629	8034.800098	7822.586779	6561.56894	6367.382064	5588.701783	7855.175037	7996.288967	9928.865795
4.66_311.1615m/z	311.1614683	4.660483333	neg	Trimetazidine	85225	Unclassified	Unclassified	Unclassified			39	0	M+FA-H	C14H22N2O3	0.837136611	472.1159892	268.696295	371.0972024	1551.42755	1090.57419	1467.586073	703.5636013	680.6328678	559.1207111
4.66_407.0469m/z	407.0468815	4.660483333	neg	2-O-Galloylgalactaric acid	HMDB0036932	Organic oxygen compounds	Organooxygen compounds	Carbohydrates and carbohydrate conjugates			39	0	M+FA-H	C13H14O12	0.422028977	445.0133362	338.6183403	562.5124549	1073.5371	1154.683623	1154.751863	927.7604798	837.505192	439.2688143
4.68_175.0976m/z	175.0976079	4.682483333	neg	Isobutyric acid	106	Organic acids and derivatives	Carboxylic acids and derivatives	Carboxylic acids	**	C02632	39	0	2M-H	C4H8O2	0.143813688	592.1588156	558.2252601	601.3444855	703.4735309	721.7873359	715.6393179	649.6857351	624.965753	552.7997579
4.70_130.0874m/z	130.0873547	4.703166667	neg	L-Leucine	HMDB0000687	Organic acids and derivatives	Carboxylic acids and derivatives	Amino acids, peptides, and analogues	**	C00123	39	0	M-H	C6H13NO2	0.018819194	577.707879	526.0753392	609.4512435	704.9668444	663.8424602	762.5787303	769.8015437	793.5238771	815.661073
4.73_234.0885m/z	234.0884947	4.725416667	neg	N'-Nitrosoanatabine	73172	Unclassified	Unclassified	Unclassified			39	0	M+FA-H	C10H11N3O	0.422217045	3095.93789	3098.727095	3001.514754	3304.84537	3450.759549	3372.913366	3960.959466	3101.620837	3337.45187
4.79_431.2221m/z	431.2221409	4.792316667	neg	2-Naphthalenepropanol, 6-hydroxy-a-methyl-	1369	Unclassified	Unclassified	Unclassified			39	0	2M-H	C14H16O2	-1.485760847	522.5990563	628.0333757	532.0185063	1222.850301	1217.359455	1215.644293	463.4557791	533.0439652	709.541744
4.81_238.1087m/z	238.1086878	4.814166667	neg	(-)-Salsoline	HMDB0012469	Organoheterocyclic compounds	Tetrahydroisoquinolines	Unclassified	*	C09640	39	0	M+FA-H	C11H15NO2	1.067899689	1378.912611	1655.855527	1302.680295	2168.130433	2180.870376	2235.954619	1517.998313	1919.403328	1790.709278
4.88_291.0184m/z	291.0183558	4.880083333	neg	Vanillin 4-sulfate	96141	Organic acids and derivatives	Organic sulfuric acids and derivatives	Arylsulfates	*		39	0	M+FA-H	C9H10O6S	1.397779362	3098.65848	3708.298735	3610.990108	448.0731336	418.065614	498.7312955	2467.549347	2812.934227	2700.628726
4.95_145.0871m/z	145.0871089	4.94705	neg	Caproaldehyde	LMFA06000109	Organic oxygen compounds	Organooxygen compounds	Carbonyl compounds	**	C02373	39	0	M+FA-H	C6H12O	0.909868791	1455.63208	1542.732824	1497.122455	1778.900398	1815.931154	1893.244429	1835.324164	1700.644433	1668.04103
4.95_447.2236m/z	447.2235927	4.94705	neg	Butalbital	HMDB0014386	Organoheterocyclic compounds	Diazines	Pyrimidines and pyrimidine derivatives			39	0	2M-H	C11H16N2O3	-2.93506545	3836.142992	3803.163993	3314.361775	1283.558206	1526.330341	1241.587984	3206.596976	3254.043211	3172.559515
4.95_500.1662n	545.1645739	4.94705	neg	Ikarisoside A	LMPK12111710	Lipids and lipid-like molecules	Polyketides	Flavonoids			39	3.74	M-H, M+FA-H	C26H28O10	-4.013840909	45383.34918	46869.78467	47749.5018	162.2370989	188.5147615	404.9157687	28750.32558	27505.06077	28130.44495
4.97_341.1245m/z	341.124542	4.969683333	neg	Vomitoxin	LMPR0103180002	Lipids and lipid-like molecules	Prenol lipids	Sesquiterpenoids	*	C09747	39	0	M+FA-H	C15H20O6	1.184397332	3373.823436	3681.184423	3408.445275	1785.483188	1950.864154	1977.448126	4441.856539	3998.544202	3909.495078
4.97_535.1826m/z	535.1825687	4.969683333	neg	5-Hydroxy-7,4'-dimethoxy-6,8-di-C-prenylflavanone 5-O-galactoside	LMPK12140572	Unclassified	Unclassified	Unclassified			39	1.78	M+FA-H	C25H30O10	0.956013579	8720.72766	9807.64049	9277.891636	3358.435162	3794.750061	3538.322564	9067.671498	7629.383214	6503.07477
5.01_265.0227m/z	265.0226866	5.014716667	neg	Imidazole acetol-phosphate	HMDB0012236	Organic acids and derivatives	Organic phosphoric acids and derivatives	Phosphate esters	**	C01267	39	0	M+FA-H	C6H9N2O5P	-1.928566554	538.9543556	617.3108107	683.4658087	1013.674434	1053.743682	983.2990637	1035.367592	735.1254416	810.6467276
5.06_172.0981m/z	172.0980659	5.05975	neg	Methyl N-(a-methylbutyryl)glycine	5709	Unclassified	Unclassified	Unclassified			39	0	M-H	C8H15NO3	0.860799269	2826.597787	2717.871825	2749.232999	5060.010769	4438.340958	4612.072412	3247.302362	2801.648258	3287.979759
5.10_167.0715m/z	167.0714527	5.1031	neg	2-Phenylethanol	64740	Benzenoids	Benzene and substituted derivatives	Unclassified	**	C05853	39	0	M+FA-H	C8H10O	0.695036487	706.800692	631.2410926	612.4533749	997.6375649	970.687704	1103.794927	821.825992	690.0900079	876.239935
5.10_217.1447m/z	217.1446585	5.1031	neg	Capric acid	HMDB0000511	Lipids and lipid-like molecules	Fatty Acyls	Fatty acids and conjugates	***	C01571	39	0	M+FA-H	C10H20O2	0.730605347	724.2604954	724.9757903	717.8079588	1141.822107	1060.870954	1195.400498	940.1850242	777.4801601	798.8243205
5.10_240.1244m/z	240.1243743	5.1031	neg	2-hydroxymexiletine	HMDB0060953	Benzenoids	Phenol ethers	Unclassified	*		39	0	M+FA-H	C11H17NO2	1.243503753	2766.952655	2551.72003	2186.38211	2491.844924	3063.860399	3410.127774	3286.733515	2558.898693	2872.275995
5.10_343.1400m/z	343.1400272	5.1031	neg	3-Isopropenylpentanedioic acid	HMDB0032352	Lipids and lipid-like molecules	Fatty Acyls	Fatty acids and conjugates	*		39	0	2M-H	C8H12O4	0.540106672	19775.79495	19392.49559	20720.93455	9861.540578	9998.734057	10297.0635	21183.29368	20397.18345	19253.76962
5.13_471.1102m/z	471.1101789	5.132616667	neg	11-Hydroxycanthin-6-one	67521	Unclassified	Unclassified	Unclassified			39	0	2M-H	C14H8N2O2	0.63615006	4366.692892	4739.511743	4158.479365	4805.885675	4463.540201	5082.082924	3776.837175	3251.508721	4468.947334
5.18_153.0922m/z	153.092192	5.1777	neg	4-Vinylcyclohexene	73021	Unclassified	Unclassified	Unclassified			39	0	M+FA-H	C8H12	0.820891558	470.8791815	596.1791834	509.9991748	758.4975751	763.6334058	663.1457395	549.4034046	496.6607812	586.2436685
5.36_333.0935m/z	333.0934623	5.361783333	neg	(S)-a-Amino-2,5-dihydro-5-oxo-4-isoxazolepropanoic acid N2-glucoside	86247	Organic oxygen compounds	Organooxygen compounds	Carbohydrates and carbohydrate conjugates	*		39	0	M-H	C12H18N2O9	-1.470783233	7479.455896	9051.940677	8451.582819	6721.325614	7041.767786	6410.570804	5336.808809	6280.506522	5720.57674
5.41_461.1432m/z	461.1432136	5.406433333	neg	Cyrtopterin	LMPK12140232	Lipids and lipid-like molecules	Polyketides	Flavonoids			39	6.77	M-H	C23H26O10	-4.559198941	12090.21348	14225.42783	14577.49341	18572.1103	18044.06319	17744.0671	15515.2053	17378.84627	12675.49761
5.43_277.0669m/z	277.0669342	5.428733333	neg	Kifunensine	HMDB0253800	Unclassified	Unclassified	Unclassified			39	0.329	M+FA-H	C8H12N2O6	-3.467910337	55335.69348	59620.50766	57806.4602	42226.54572	40213.16983	41608.91907	52148.95063	55850.31272	44022.52982
5.50_253.1447m/z	253.1447232	5.497816667	neg	Theaspirone A	HMDB0035228	Organic oxygen compounds	Organooxygen compounds	Carbonyl compounds	*		39	0	M+FA-H	C13H20O2	0.915103172	3427.534765	2927.873283	2549.51116	2557.988993	3522.759584	3986.878281	3830.649804	3145.055657	3487.816572
5.52_521.1619m/z	521.1618996	5.516816667	neg	Dihydromaleimide beta-D-glucoside	86854	Organic oxygen compounds	Organooxygen compounds	Carbohydrates and carbohydrate conjugates	*		39	0	2M-H	C10H15NO7	-1.010509653	1849.369237	2052.476232	1638.267824	1651.513156	2259.269949	1854.73998	1991.965069	1393.909272	1942.573624
5.61_250.1085m/z	250.1085253	5.610733333	neg	Bellendine	68626	Unclassified	Unclassified	Unclassified			39	0	M+FA-H	C12H15NO2	0.213249921	465.1468177	682.3395172	754.291152	1344.10595	1150.991467	1196.01086	832.9641507	774.2136562	660.4349756
5.70_355.1402m/z	355.1401553	5.701283333	neg	BOX B	HMDB0004145	Organoheterocyclic compounds	Pyrrolines	Unclassified	*		39	0	2M-H	C9H10N2O2	-2.873632241	842.9824238	753.7380149	682.7040076	601.7915476	734.2979725	939.6864893	548.1583273	871.2215989	1332.330297
5.98_403.1612m/z	403.1612431	5.9829	neg	Diethyl Oxalpropionate	44744	Organic acids and derivatives	Keto acids and derivatives	Beta-keto acids and derivatives	*	C04067	39	0	2M-H	C9H14O5	0.673999061	8025.147198	7960.850071	7912.503145	9472.07072	10092.48682	10286.07956	6660.676557	6855.372089	6645.667391
6.16_155.1079m/z	155.10787	6.161866667	neg	2-(Methylenebutyl)-cyclopropane	HMDB0061899	Hydrocarbons	Saturated hydrocarbons	Unclassified			39	0	M+FA-H	C8H14	1.059945228	497.8077982	586.316215	523.4201999	815.1617234	866.3209679	989.5111281	590.9644117	632.4255872	604.9444192
6.30_234.1137m/z	234.1137383	6.302033333	neg	1-Methyl-4-phenyl-1,2,3,6-tetrahydropyridine N-oxide	66251	Unclassified	Unclassified	Unclassified			39	0	M+FA-H	C12H15NO	0.905852602	917.7024598	974.9205197	1005.455914	938.6418806	987.1386852	760.5471254	1382.814427	1424.437706	1583.197929
6.35_255.1604m/z	255.1604437	6.348783333	neg	Ethyl 4-pentenoate	HMDB0031603	Lipids and lipid-like molecules	Fatty Acyls	Fatty acid esters	*		39	0	2M-H	C7H12O2	1.018383903	4769.879896	5670.867321	5841.176635	7338.017124	7209.414086	6610.274273	4127.905493	4055.367687	4129.048548
6.37_155.1079m/z	155.1078705	6.373833333	neg	1,3-Octadiene	95410	Hydrocarbons	Unsaturated hydrocarbons	Olefins			39	0	M+FA-H	C8H14	1.064562442	1092.175223	1040.442252	1196.422944	1769.255832	1992.338213	1992.705084	1020.914649	1014.702735	964.5724656
6.37_273.1710m/z	273.1710139	6.373833333	neg	Menthyl lactate	92179	Lipids and lipid-like molecules	Prenol lipids	Monoterpenoids	*		39	0	M+FA-H	C13H24O3	1.167667069	7870.677843	8514.188065	8769.66981	8072.125254	8618.025972	8201.576404	6498.431553	7128.414538	7010.51446
6.40_445.1909m/z	445.1908558	6.39835	neg	N-Fluorenylacetamide	516	Unclassified	Unclassified	Unclassified			39	0	2M-H	C15H13NO	-2.904205305	1091.776848	1037.09377	1235.026082	1098.78043	1277.657447	1634.818375	751.6917667	726.9183037	857.5048971
6.46_331.2130m/z	331.2129918	6.458366667	neg	3-Isopropyl-2-methoxy-5-methylpyrazine	86460	Organoheterocyclic compounds	Diazines	Pyrazines	*		39	0	2M-H	C9H14N2O	-2.88354313	1906.688015	1608.643358	1787.90448	1584.737049	1223.771392	1305.568838	1658.166683	1855.949108	1855.01941
6.54_297.1321m/z	297.1321266	6.539816667	neg	7-Methyladenine	62383	Organoheterocyclic compounds	Imidazopyrimidines	Purines and purine derivatives	*	C02241	39	0	2M-H	C6H7N5	-2.976679753	5568.873242	5913.172489	6306.566663	4372.918561	3911.285041	3937.742581	5632.418872	6477.15284	5541.195945
6.62_167.1077m/z	167.1076798	6.61635	neg	Ethyl (3R,5Z)-3-hydroxy-5-octenoate	HMDB0030367	Lipids and lipid-like molecules	Fatty Acyls	Fatty alcohols	*		39	0	M-H2O-H	C10H18O3	-0.394974077	1927.900519	2070.231858	2044.598523	2520.90085	2658.7675	2446.253431	2278.427461	2322.079316	2092.37884
7.04_183.0664m/z	183.0663682	7.035966667	neg	3,4-Methylenepimelic acid	HMDB0059730	Lipids and lipid-like molecules	Fatty Acyls	Fatty acids and conjugates	*		39	0	M-H	C9H12O4	0.466219999	849.4021607	605.6603096	723.3278498	612.9476922	690.2389779	724.9398049	799.1827977	770.603089	737.8108608
7.16_399.1640m/z	399.1639512	7.1647	neg	Valganciclovir	HMDB0015548	Organic acids and derivatives	Carboxylic acids and derivatives	Amino acids, peptides, and analogues			39	0.295	M+FA-H	C14H22N6O5	1.638985792	9658.130826	11981.92271	10597.16006	14456.49061	13194.62115	13610.59768	12042.73929	15454.92063	13723.01696
7.35_229.1446m/z	229.1445642	7.346316667	neg	Ethyl (E)-2-nonenoate	87542	Lipids and lipid-like molecules	Fatty Acyls	Fatty acid esters	*		39	0	M+FA-H	C11H20O2	0.1708964	2516.544246	2789.683094	2438.490136	3270.27031	3276.935449	3023.720832	2127.666487	2199.553347	2538.805639
7.40_331.2129m/z	331.2129243	7.404266667	neg	Hexadecanedioic acid	5642	Lipids and lipid-like molecules	Fatty Acyls	Fatty acids and conjugates	**	C19615	39	0	M+FA-H	C16H30O4	1.090173562	10756.94627	10911.38229	11256.75766	8909.299364	7906.311511	8248.320322	8593.336186	9168.373812	9318.977349
7.43_183.0664m/z	183.0663842	7.426916667	neg	cis-3-(Carboxy-ethyl)-3,5-cyclo-hexadiene-1,2-diol	63531	Unclassified	Unclassified	Unclassified			39	0	M-H	C9H12O4	0.55263908	552.8471892	529.0720531	527.4662359	519.6743132	551.6997225	601.9536512	533.5190243	505.9840188	557.947297
7.51_169.1233m/z	169.1232908	7.50615	neg	Isobutylene-isoprene copolymer	88379	Unclassified	Unclassified	Unclassified			39	0	M+FA-H	C9H16	-0.907408552	1456.925319	1419.37308	1635.822205	1443.992387	1642.82357	1421.182637	1351.670002	1535.47531	1638.660495
7.54_229.1446m/z	229.1445775	7.536516667	neg	cis-3-Hexenyl pentanoate	87867	Lipids and lipid-like molecules	Fatty Acyls	Fatty acid esters	*		39	0	M+FA-H	C11H20O2	0.243091489	3435.654523	3485.273709	3733.01968	2333.560232	2300.472613	2411.45913	2063.551178	2290.83155	2187.247348
7.63_146.0248m/z	146.0247856	7.631783333	neg	Noradrenochrome	HMDB0013030	Organoheterocyclic compounds	Indoles and derivatives	Unclassified	*		39	0	M-H2O-H	C8H7NO3	0.204042412	210.976458	320.0088873	265.080402	241.0393621	214.1215537	208.2432116	266.1922403	278.8422427	195.2138076
7.74_253.1446m/z	253.1446001	7.738233333	neg	(5R,6S)-5,6-Epoxy-7-megastigmen-9-one	HMDB0034984	Organoheterocyclic compounds	Oxepanes	Unclassified	*		39	0	M+FA-H	C13H20O2	0.323668727	495.5946027	481.598053	542.8999102	985.259413	1194.68751	876.0116929	557.5437353	478.9742606	512.0134398
8.00_611.2427m/z	611.2427473	7.99505	neg	(1aalpha,2beta,3alpha,11calpha)-1a,2,3,11c-Tetrahydro-6,11-dimethylbenzo[6,	HMDB0062223	Benzenoids	Phenanthrenes and derivatives	Phenanthrols	**	C19559	39	0	2M-H	C20H18O3	-1.903041145	3323.21095	2474.10801	3354.037811	0.001403199	0.001403199	109.6305534	1206.224081	924.2675901	887.3309929
8.25_229.1445m/z	229.1445342	8.254283333	neg	Ethyl 3-cyclohexylpropionate	LMFA07010862	Lipids and lipid-like molecules	Fatty Acyls	Fatty acid esters	*		39	0	M+FA-H	C11H20O2	0.007815183	547.5462271	617.2756403	773.5429208	936.2602072	587.6768254	830.5227952	426.0197765	460.4979439	452.3288089
8.38_433.2444m/z	433.2444138	8.375033333	neg	5,6-Dihydroxyprostaglandin F1a	HMDB0012109	Lipids and lipid-like molecules	Fatty Acyls	Eicosanoids	*	C06475	39	0	M+FA-H	C20H36O7	0.276787633	927.8994014	1028.497698	960.9302123	927.382724	907.8186722	1111.315418	396.0515763	588.9389631	690.3671269
8.46_395.1713m/z	395.171315	8.463816667	neg	Millefin	HMDB0036689	Lipids and lipid-like molecules	Prenol lipids	Terpene lactones	*		39	0	M+FA-H	C19H26O6	0.495745553	40094.63625	40887.0839	42671.28663	44289.99166	42176.88468	42884.73252	24413.8638	27692.59409	28224.94817
8.50_471.2601m/z	471.2600531	8.503866667	neg	Prostaglandin PGE2 1-glyceryl ester	85231	Lipids and lipid-like molecules	Fatty Acyls	Eicosanoids	*		39	0	M+FA-H	C23H38O7	0.226707764	1560.755179	1387.319244	1481.976765	3591.613437	2975.62805	3030.363076	859.1188512	947.8751887	1129.749488
8.79_439.1200m/z	439.1199811	8.791166667	neg	SP 600125	45266	Unclassified	Unclassified	Unclassified			39	0	2M-H	C14H8N2O	-0.155140887	4060.331755	3885.885265	4699.159318	54.2732494	0.001403199	61.89223348	3609.489998	3858.074497	4057.99954
8.99_413.2184m/z	413.2183724	8.9919	neg	Prostaglandin G2	HMDB0003235	Lipids and lipid-like molecules	Fatty Acyls	Eicosanoids	**	C05956	39	0	M+FA-H	C20H32O6	0.762604507	18593.99308	19769.77034	19525.61257	7182.923342	7454.097502	8085.312809	10678.21587	11637.69321	11865.09843
9.41_399.2027m/z	399.202661	9.414	neg	Idebenone Metabolite (Benzenedecanoic acid, 2,5-dihydroxy-3,4-dimethoxy-6-n	771	Unclassified	Unclassified	Unclassified			39	0	M+FA-H	C19H30O6	0.619543296	10883.87474	10704.98451	12829.25189	13301.01419	12080.18171	12207.62753	8057.657201	8536.162246	8746.313531
0.54_281.2489m/z	281.2488766	0.535283333	neg	6Z,9Z-Heptadecadiene	LMFA11000141	Lipids and lipid-like molecules	Fatty Acyls	Hydrocarbons			38.9	0	M+FA-H	C17H32	1.154103276	327.0208252	376.9723956	418.9148866	398.6561098	295.9331797	329.5333336	553.249261	320.9369323	391.7064931
0.71_316.9480m/z	316.9479797	0.714216667	neg	beta-D-fructose 2,6-bisphosphate	HMDB0304539	Organic oxygen compounds	Organooxygen compounds	Carbohydrates and carbohydrate conjugates	**	C00665	38.9	1.09	M-H2O-H	C6H10O12P2-4	3.190143696	559332.7075	535370.9307	537703.6256	373536.9504	376294.2457	380444.3797	545438.6779	493915.978	512491.4604
0.82_233.0779m/z	233.0778938	0.819566667	neg	2-Amino-3-oxobutanoate	65931	Organic acids and derivatives	Carboxylic acids and derivatives	Amino acids, peptides, and analogues	**	C03508	38.9	0	2M-H	C4H7NO3	-0.068306869	1759.851453	1196.839061	2177.064922	1599.60944	1499.32849	1379.711808	1722.724185	1621.640642	1848.506543

0.82_287.0521m/z	287.0520741	0.819566667	neg	Orotidine	5754	Nucleosides, nucleotides, and analogues	Pyrimidine nucleosides	Unclassified	**	C01103	38.9	0	M-H	C10H12N2O8	-0.051485689	12437.91784	12847.20178	12364.14212	11176.46296	10020.03076	10338.82334	11230.75181	14368.64102	12285.08324	
0.84_163.0249m/z	163.0248978	0.841733333	neg	Erythrono-1,4-lactone	HMDB0000349	Organoheterocyclic compounds	Lactones	Gamma butyrolactones	*		38.9	0	M+FA-H	C4H6O4	0.730515823	4453.51535	5254.057959	6077.613467	3498.918315	3113.014321	3500.312991	3602.16059	3788.817263	4020.393047	
0.88_196.0286m/z	196.0285656	0.884633333	neg	(S)C(S)S-S-Methylcysteine sulfoxide	86264	Organic acids and derivatives	Carboxylic acids and derivatives	Amino acids, peptides, and analogues	*		38.9	0	M+FA-H	C4H9NO3S	0.32142227	10731.63399	12465.06859	10245.37006	14286.99509	11191.15752	13935.93895	10069.88709	13044.01688	11655.60051	
0.93_132.0667m/z	132.0666664	0.925866667	neg	N-Ethylacetamide	87500	Organic acids and derivatives	Carboximidic acids and derivatives	Carboximidic acids	*		38.9	0	M+FA-H	C4H9NO	0.569831922	259.2328389	314.3119354	356.7127323	480.224367	334.772214	365.1551406	411.6674304	555.1485006	246.8975046	
0.93_158.0460m/z	158.0459587	0.925866667	neg	1-Pyrroline-2-carboxylic acid	58576	Organoheterocyclic compounds	Pyrrolines	Unclassified	**	C03564	38.9	0	M+FA-H	C5H7NO2	0.684748286	1779.746046	2304.318194	2318.557459	1251.594558	1187.679219	1272.09084	1182.122318	1429.433357	1204.677279	
1.06_359.0988m/z	359.0987894	1.055866667	neg	Isonicotinylglycine	HMDB0041912	Organic acids and derivatives	Carboxylic acids and derivatives	Amino acids, peptides, and analogues	**	C16623	38.9	0	2M-H	C8H8N2O3	-2.550478026	1217.872778	1364.320818	1617.92969	1586.341992	1150.655939	1125.926483	1300.236116	1368.247867	1361.293698	
1.16_164.0579m/z	164.0579057	1.160166667	neg	1-Methylguanine	HMDB0003282	Organoheterocyclic compounds	Imidazopyrimidines	Purines and purine derivatives	*	C04152	38.9	0	M-H	C6H7N5O	0.740709614	1428.249082	1653.689808	1876.308593	1364.058814	1976.508469	1186.331268	1520.57072	1423.72854	2299.474026	
1.16_359.0483m/z	359.0483064	1.160166667	neg	5'-Phosphoribosyl-N-formylglycinamide	HMDB0001308	Nucleosides, nucleotides, and analogues	Glycinamide ribonucleotides	Unclassified	**	C04376	38.9	2.59	M+FA-H	C8H15N2O9P	-4.499832027	46882.28435	47283.67508	84163.55206	82903.38641	92714.24548	89647.157	62561.22371	77881.85743	81756.1544	
1.18_216.0990m/z	216.0989834	1.180366667	neg	N-alpha-Acetyl-L-citrulline	HMDB0000856	Organic acids and derivatives	Carboxylic acids and derivatives	Amino acids, peptides, and analogues	**	C15532	38.9	0	M-H	C8H15N3O4	0.017574068	1878.151805	1722.0611	1961.723745	3074.411553	2682.926834	3089.922967	2593.744019	2739.07473	2994.995495	
1.29_204.0879m/z	204.0879001	1.290916667	neg	N-isovalerylglycine	4128	Organic acids and derivatives	Carboxylic acids and derivatives	Amino acids, peptides, and analogues	*		38.9	0	M+FA-H	C7H13NO3	0.96782614	736.2215135	967.3407295	1145.920441	695.76138	870.8707062	983.2734688	998.9613957	1182.478675	950.4189575	
1.38_145.0507m/z	145.0507428	1.380283333	neg	Glutaral	HMDB0029599	Organic oxygen compounds	Organooxygen compounds	Carbonyl compounds	*	C12518	38.9	0	M+FA-H	C5H8O2	1.104052092	1534.690804	1407.647149	1318.191711	1657.172671	1334.11502	1348.667926	1502.849436	1473.688467	1423.834134	
1.47_142.0510m/z	142.0510323	1.472133333	neg	2,5-Dimethyloxazole	HMDB0032969	Organoheterocyclic compounds	Azoles	Oxazoles			38.9	0	M+FA-H	C5H7NO	0.676136719	1079.663625	1065.778899	1029.182859	1048.538106	905.0499412	957.330918	941.2335786	804.412874	928.3957647	
1.62_127.0150m/z	127.0149953	1.615883333	neg	5-N-Methyloxaluric acid	68858	Unclassified	Unclassified	Unclassified			38.9	0	M-H2O-H	C4H6N2O4	0.54587732	2784.663652	2482.390726	2468.214329	3331.859625	3130.029928	3052.123649	3320.843361	2842.505519	3244.607755	
1.72_188.0025m/z	188.0024567	1.721216667	neg	2-aminophenol sulphate	HMDB0061116	Organic acids and derivatives	Organic sulfuric acids and derivatives	Arylsulfates	*		38.9	4.31	M-H	C6H7NO4S	0.817270022	2843.670176	3088.781425	2718.337114	1236.908763	1167.219188	1328.63053	1625.343641	1849.277819	1817.336038	
1.75_243.1351m/z	243.1351333	1.74555	neg	Slaframine	64439	Unclassified	Unclassified	Unclassified			38.9	0	M+FA-H	C10H18N2O2	0.51803344	555.2643159	608.7003642	494.517983	690.5980452	764.1714119	838.003286	671.3246619	552.1122971	586.6950467	
1.76_169.0144m/z	169.0144416	1.7577	neg	2-Hydroxy-1,4-benzoquinone	66588	Unclassified	Unclassified	Unclassified			38.9	0	M+FA-H	C6H4O3	1.570328025	1355.977577	1425.123823	1344.30806	1228.151438	1316.378846	1074.045775	1076.991731	1429.916477	1190.104024	
10.52_311.2231m/z	311.2231218	10.51685	neg	α-nonylenic acid	34716	Lipids and lipid-like molecules	Fatty Acyls	Fatty acids and conjugates	*		38.9	0	2M-H	C9H16O2	1.085015158	1132.817281	1649.24124	1128.805636	488.1882029	753.7028812	713.2010768	581.9085246	1048.854055	687.072272	
11.14_297.2436m/z	297.2436246	11.13858333	neg	13-heptadecyn-1-ol	85269	Lipids and lipid-like molecules	Fatty Acyls	Fatty alcohols	*		38.9	0	M+FA-H	C17H32O	0.420495191	13376.3944	13496.43148	11794.73861	11516.0615	10820.12265	10880.5689	14437.4268	15543.1234	11860.48715	
11.77_299.2019m/z	299.2018965	11.77491667	neg	4-Phenyl-2-butanol	87808	Benzenoids	Benzene and substituted derivatives	Unclassified	*		38.9	0	2M-H	C10H14O	0.808719651	858.0300253	585.0824152	566.9357781	853.8702504	1073.769847	666.1791534	1002.44064	849.0558584	789.1491939	
13.52_174.0156m/z	174.0155776	13.52308333	neg	Cyanuric acid	HMDB0041861	Organoheterocyclic compounds	Triazines	1,3,5-triazines	**	C06554	38.9	0	M+FA-H	C3H3N3O3	-0.513235896	116.6786766	396.6188302	253.6865051	401.9525269	167.3114169	218.1501303	504.2493122	461.4191111	367.3205964	
14.93_297.2435m/z	297.2435277	14.93063333	neg	8-Heptadecenal	95743	Lipids and lipid-like molecules	Fatty Acyls	Fatty aldehydes	*		38.9	0	M+FA-H	C17H32O	0.036565624	8172.799506	6220.973966	8348.735805	5961.903461	7973.570687	6615.387207	8470.749551	8657.98041	9222.580499	
2.07_260.0776m/z	260.0776122	2.070366667	neg	Succinylproline	69170	Unclassified	Unclassified	Unclassified			38.9	0	M+FA-H	C9H13NO5	0.171280069	1471.287123	1780.526513	1712.435619	2721.294553	2764.799146	2937.638314	1969.496963	1625.272348	1774.621807	
2.07_399.1750m/z	399.1749668	2.070366667	neg	3,6,8-Trimethylallantoin	64464	Unclassified	Unclassified	Unclassified			38.9	0	2M-H	C7H12N4O3	0.906326104	1105.548956	1173.681869	1324.138773	724.9338423	551.3907894	634.9556844	1060.604447	1029.094514	875.9383239	
2.12_161.0820m/z	161.0819986	2.12355	neg	Isocaproic acid	HMDB0000689	Lipids and lipid-like molecules	Fatty Acyls	Fatty acids and conjugates	*		38.9	0	M+FA-H	C6H12O2	0.569291872	513.241907	558.5355177	560.2495929	591.8010799	481.4266697	468.6891684	458.8908513	602.3185897	578.8560457	
2.31_411.1096m/z	411.109599	2.3142	neg	EUGENITOL	44043	Organoheterocyclic compounds	Benzopyrans	1-benzopyrans	*		38.9	0	2M-H	C11H10O4	2.566931478	9258.961825	8780.610866	8282.637709	13840.70339	14526.16682	15016.90199	11014.37809	12293.39362	11411.82042	
2.40_355.0899m/z	355.0898636	2.399566667	neg	Melizame	HMDB0029684	Organic oxygen compounds	Organooxygen compounds	Ethers	*		38.9	0	2M-H	C7H6N4O2	-2.838724689	5685.005177	6401.329958	6293.212384	8854.128546	8259.128591	7199.265867	5633.282978	6501.817196	5437.52409	
2.43_219.0776m/z	219.0776203	2.429083333	neg	1H-Indole-3-acetamide	HMDB0029739	Organoheterocyclic compounds	Indoles and derivatives	Indoles	***	C02693	38.9	0	M+FA-H	C10H10N2O	0.600376052	3935.505583	4216.981556	4660.050933	3644.740978	3367.782199	3279.503628	4711.328843	5547.215893	4941.414332	
2.45_173.0456m/z	173.0455653	2.451266667	neg	Osmundalactone	HMDB0031303	Organoheterocyclic compounds	Pyrans	Pyranones and derivatives	*		38.9	0	M+FA-H	C6H8O3	0.142749253	518.0557413	530.9424461	631.5293062	557.221105	501.582719	488.1127092	452.0079734	374.7059746	307.4061274	
3.00_145.0619m/z	145.0619009	3.004583333	neg	Gyromitrin	HMDB0033952	Organic nitrogen compounds	Organonitrogen compounds	Hydrazines and derivatives		C08305	38.9	0	M+FA-H	C4H8N2O	0.350890097	0.001403199	13.76304459	0.001403199	98.63810214	48.66357504	75.04037483	24.23353925	49.57483661	19.22032099	
3.02_181.0507m/z	181.050742	3.02	neg	Phenylacetic acid	129	Benzenoids	Benzene and substituted derivatives	Unclassified	**	C07086	38.9	0	M+FA-H	C8H8O2	0.806112908	2775.345652	2963.729866	3289.87585	1586.226801	1678.930005	1561.984997	2386.68492	2483.389325	2417.253654	
3.06_161.0820m/z	161.0819549	3.05735	neg	Methyl (S)-2-Methylbutanoate	HMDB0029762	Lipids and lipid-like molecules	Fatty Acyls	Fatty acid esters	*		38.9	0	M+FA-H	C6H12O2	0.193362987	556.1893184	574.4266463	589.6684836	636.7522869	581.528013	542.2541555	593.471999	722.0804895	657.4155	
3.50_300.0726m/z	300.0726228	3.498716667	neg	beta-D-ribosylnicotinate	HMDB0304540	Organic oxygen compounds	Organooxygen compounds	Carbohydrates and carbohydrate conjugates	**	C05841	38.9	0	M+FA-H	C11H13NO6	0.520704826	4808.962811	5662.494086	5586.322338	1003.286955	923.8637392	736.1941106	3963.615373	4285.516814	4099.510107	
3.60_240.0515m/z	240.0515113	3.601516667	neg	Leucodopachrome	58201	Organoheterocyclic compounds	Indoles and derivatives	Indolecarboxylic acids and derivatives	**	C05604	38.9	0	M+FA-H	C9H9NO4	0.772300395	1397.817667	1713.731657	1558.651884	1272.844369	1358.906922	1536.842824	1034.445173	1285.163073	1134.463815	
3.64_233.1032m/z	233.1031732	3.640916667	neg	Nonate	HMDB0011717	Lipids and lipid-like molecules	Fatty Acyls	Fatty acids and conjugates	*		38.9	0	M+FA-H	C9H16O4	0.591931881	1295.34324	1328.497442	1133.513954	2071.9669	2151.888394	2073.664097	1612.876016	1487.14381	1813.56672	
3.69_279.0987m/z	279.0987283	3.685866667	neg	5-Methoxy-DL-tryptophan	103475	Unclassified	Unclassified	Unclassified			38.9	0	M+FA-H	C12H14N2O3	0.355074678	922.5691165	858.852395	1004.254624	556.4969691	504.268952	320.0080358	589.5242066	494.9620136	585.1855066	
4.02_324.0726m/z	324.0726421	4.024283333	neg	N-[4'-hydroxy-(E)-cinnamoyl]-L-aspartic acid	HMDB0029234	Organic acids and derivatives	Carboxylic acids and derivatives	Amino acids, peptides, and analogues	*		38.9	0	M+FA-H	C13H13NO6	0.54482071	956.1369213	1310.533685	1085.213589	38.18225304	0.001403199	0.001403199	832.2909571	1195.737947	797.6251985	
4.09_295.1300m/z	295.1299954	4.091783333	neg	Paucine	HMDB0029876	Benzenoids	Phenols	Benzenediols	*	C03002	38.9	0	M+FA-H	C13H18N2O3	0.20018217	1129.831681	1085.225171	1123.250592	1839.971315	1462.214361	1665.139913	948.3375391	1049.836564	1189.792367	
4.19_163.0401m/z	163.0401454	4.189783333	neg	3,5,7-Octatriyn-1-ol	HMDB0038998	Lipids and lipid-like molecules	Fatty Acyls	Fatty alcohols	*		38.9	0	M+FA-H	C8H6O	0.658536061	1422.605793	1024.661541	1047.95665	726.7734789	665.0860305	821.1499731	1104.32041	809.4634985	894.7340002	
4.19_176.1049n	175.097621	4.189783333	neg	L-Cladinose	69247	Unclassified	Unclassified	Unclassified			38.9	0	M-H2O-H, M-H	C8H16O4	0.154254723	2332.10493	2040.219217	2127.063246	2411.235327	2386.421021	3142.150193	2749.657778	2196.742358	2318.604324	
4.21_307.0435m/z	307.0435263	4.212116667	neg	D-Mannitol 1-phosphate	3393	Organic oxygen compounds	Organooxygen compounds	Carbohydrates and carbohydrate conjugates	**	C00644	38.9	0	M+FA-H	C6H15O9P	-0.172833719	1220.705537	1142.667488	1378.26349	1287.521708	1116.755097	1762.624391	751.399879	632.5418818	658.9226075	
4.23_146.0612m/z	146.0611671	4.234483333	neg	5-(3-Pyridyl)-2-hydroxytetrahydrofuran	HMDB0062407	Organoheterocyclic compounds	Pyridines and derivatives	Unclassified	**	C19578	38.9	0	M-H2O-H	C9H11NO2	0.179633182	257.6463507	317.1779534	279.2614469	310.5430457	313.7260164	285.0054558	206.1466371	337.4441688	339.2727783	
4.23_379.1614m/z	379.1613882	4.234483333	neg	6-(2-Hydroxyethoxy)-6-oxohexanoic acid	HMDB0061681	Lipids and lipid-like molecules	Fatty Acyls	Fatty acids and conjugates	*		38.9	0	2M-H	C8H14O5	1.098415389	5534.199285	5918.213043	5188.876931	5210.334917	4103.280334	4556.149151	5737.922636	5770.842415	5024.374688	
4.23_481.1352m/z	481.1352448	4.234483333	neg	Phlorizin	HMDB0036634	Phenylpropanoids and polyketides	Flavonoids	Flavonoid glycosides	***	C01604	38.9	0	M+FA-H	C21H24O10	0.217720224	1585.415168	1400.326632	1658.885333	493.7463169	412.2878464	320.2562959	1280.185955	2053.714738	1687.719602	
4.32_131.0349m/z	131.0349438	4.3224	neg	2-Butyne-1,4-diol	HMDB0245045	Unclassified	Unclassified	Unclassified			38.9	0	M+FA-H	C4H6O2	-0.447323914	101.0247389	138.6472052	120.0547416	179.0519401	122.6947579	150.1752027	179.2191602	201.5438382	190.6995425	
4.34_286.1662m/z	286.1662052	4.344733333	neg	Dioscoretine	93255	Lipids and lipid-like molecules	Fatty Acyls	Fatty acids and conjugates	*		38.9	0	M+FA-H	C13H23NO3	0.865520638	1376.106725	1572.836484	1717.8832	954.6558942	663.8366086	876.0808389	1290.118548	1359.744643	1260.854786	
4.34_321.1194m/z	321.1194005	4.344733333	neg	Triethyl citrate	HMDB0034263	Organic acids and derivatives	Carboxylic acids and derivatives	Tricarboxylic acids and derivatives			38.9	0	M+FA-H	C12H20O7	1.06703915	861.427075	591.5196173	1057.89695	1821.854706	1211.292539	1729.525021	877.6758297	923.9331164	1009.282964	
4.41_338.0883m/z	338.0883382	4.41115	neg	(+)-2,7-Dideoxypancratistatin	69398	Unclassified	Unclassified	Unclassified			38.9	0	M+FA-H	C14H15NO6	0.676084468	6151.26007	7217.073559	7268.595553	4186.974184	4664.405916	5347.826413	7208.147955	8718.557442	7464.994632	
4.41_425.0706m/z	425.0705857	4.41115	neg	2-Hydroxylamino-4,6-dinitrotoluene	71181	Unclassified	Unclassified	Unclassified			38.9	0	2M-H	C7H7N3O5	1.693339188	3008.388314	3483.042089	2896.272787	761.5170069	481.8796702	871.594068	2480.419789	2590.231003	2978.202316	
4.48_367.0659m/z	367.0659342	4.477166667	neg	3-O-Methylgallate	66325	Unclassified	Unclassified	Unclassified			38.9	0	2M-H	C8H8O5	-3.086441514	564.080728	775.9943784	904.4921188	1684.350794	1328.035994	1386.461339	667.1881348	910.3473437	851.9529333	
4.50_275.1502m/z	275.1502193	4.49925	neg	4-Hydroxymethylphenylhydrazine	66095	Unclassified	Unclassified	Unclassified			38.9	0	2M-H	C7H10N2O	-4.092278204	544.9872087	654.6558814	644.6140268	637.2550332	632.8790785	547.4626183	429.2727459	687.1077805	655.0611168	
4.57_203.0927m/z	203.0927354	4.5668	neg	4-Hydroxycyclohexylacetic acid	5440	Organic oxygen compounds	Organooxygen compounds	Alcohols and polyols	*		38.9	0	M+FA-H	C8H14O3	1.506976061	1008.851962	1097.532605	1268.66483	1194.566142	1281.737538	1214.582285	1091.266376	1155.307834	1020.603513	
4.57_252.0879m/z	252.087896	4.5668	neg	N-Acetyl-L-phenylalanine	34536	Organic acids and derivatives	Carboxylic acids and derivatives	Amino acids, peptides, and analogues	**	C03519	38.9	0	M+FA-H	C11H13NO3	0.723682054	1024.389055	1271.975062	1315.374334	1045.150583	870.5368561	825.1250303	958.7064526	933.0226135	1189.406605	
4.57_283.0825m/z	283.0824815	4.5668	neg	2-[(Ethylthio)methyl]furan	94421	Organoheterocyclic compounds	Heteroaromatic compounds	Unclassified			38.9	0	2M-H	C7H10OS	-2.512968002	1153.692731	1184.70154	1194.383529	659.7351227	451.04791	484.3318167	1077.218565	986.5963601	1188.278488	
4.66_393.0441m/z	393.0440802	4.660483333	neg	2-O-(6-Phospho-alpha-mannosyl)-D-glycerate	HMDB0012152	Organic oxygen compounds	Organooxygen compounds	Carbohydrates and carbohydrate conjugates	*	C16699	38.9	0	M+FA-H	C9H17O12P	0.329599649	5835.879316	3947.727078	5047.759518	7425.276951	7541.608553	8480.645089	8909.729361	7052.563858	5001.256789	
4.66_473.1646m/z	473.1646027	4.660483333	neg	Sepiapterin	62978	Organoheterocyclic compounds	Pteridines and derivatives	Pterins and derivatives	**	C00835	38.9	0	2M-H	C9H11N5O3	-1.053052481	649.926172	571.4978295	749.5884495	2062.5906	2175.210193	2163.887766	981.2566924	979.285992	679.3325768	
4.68_170.0824m/z	170.0823615	4.682483333	neg	6-Acetyl-1,2,3,4-tetrahydropyridine	HMDB0030345	Organoheterocyclic compounds	Pyridines and derivatives	Hydropyridines	*		38.9	0	M+FA-H	C7H11NO	0.756729033	569.4082881	622.7846764	710.2592909	1174.171635	960.1927776	1152.863742	958.3838033	971.0276394	747.3267479	
4.70_523.1826m/z	523.1825568	4.703166667	neg	Bidenoside B	52350	Lipids and lipid-like molecules	Polyketides	Flavonoids			38.9	0	M+FA-H	C24H30O10	0.955181561	2878.774625	2759.48786	3082.670372	4084.883102	3653.295489	3622.755813	1381.292794	1056.360039	834.134952	
4.73_125.0972m/z	125.0971991	4.725416667	neg	2-Octenal	HMDB0030961	Organic oxygen compounds	Organooxygen compounds	Carbonyl compounds	*		38.9	0	M-H	C8H14O	0.082919742	792.3932824	706.0462166	625.0738775	710.5527912	776.0061478	804.8924645	885.0064955	755.587973	777.8711581	
4.73_387.1297m/z	387.1297074	4.725416667	neg	Pyridylacetylglycine	HMDB0059723	Organic acids and derivatives	Carboxylic acids and derivatives	Amino acids, peptides, and analogues			38.9	0	2M-H	C9H10N2O3	-3.350717489	2946.260083	2955.039274	2632.998872	3248.818263	3100.572008	3030.09965	2890.29133	3280.078058	3097.490448	
4.77_275.1503m/z	275.1502586	4.769883333	neg	1-Methyl-1,6-dihydropyridine-2-carbaldoxime	69204	Unclassified	Unclassified	Unclassified			38.9	0	2M-H	C7H10N2O	-3.950117288	2302.445711	2586.039756	2789.715862	2981.313866	2805.729576	2722.368875	2305.577415	3025.234736	2663.831715	
4.79_427.1224m/z	427.1224164	4.792316667	neg	Lucuminic acid	HMDB0035007	Organic oxygen compounds	Organooxygen compounds	Carbohydrates and carbohydrate conjugates			38.9	8.79	M-H2O-H	C19H26O12	-4.861092646	36386.53755	37607.69838	39036.72014	30534.48568	32944.37445	30156.08599	37118.29424	40953.33192	39416.86916	
4.84_227.0928m/z	227.092833	4.836566667	neg	Iridotrial	LMPR0102070004	Lipids and lipid-like molecules	Prenol lipids	Isoprenoids	**	C06070	38.9	0	M+FA-H	C10H14O3	1.844353225	371.4142735	443.0919803	610.1022255	681.7947517	620.0390992	652.8528218	541.696325	400.4171699	435.5693126	
4.88_254.1400m/z	254.1399531	4.880083333	neg	3,4-DMMA	96606	Unclassified	Unclassified	Unclassified			38.9	0	M+FA-H	C12H19NO2	0.819517914	10829.65694	11987.02195	11683.6595	7246.915615	6680.310919	7222.574857	11484.32882	11949.95675	12379.25671	
4.90_369.1534m/z	369.1534111	4.902316667	neg	Secnidazole	HMDB0258202	Unclassified	Unclassified	Unclassified			38.9	0	2M-H	C7H11N3O3	1.63466774	1261.082657	1401.621129	1552.206978	1133.560255	1191.143477	1298.092177	656.3884806	856.3980275	638.4629822	
4.92_487.9779m/z	487.9778698	4.92485	neg	Adenosine triphosphate	HMDB0000538	Nucleosides, nucleotides, and analogues	Purine nucleotides	Purine ribonucleotides	***	C00002	38.9	0	M-H2O-H	C10H16N5O13P3	-0.068092162	2966.441216	2664.66226	2936.663469	263.6562741	201.217366	200.7954763	549.166159	623.2916809	351.8157651	
4.95_439.1614m/z	439.1613762	4.94705	neg	Gein	HMDB0029909	Organic oxygen compounds	Organooxygen compounds	Carbohydrates and carbohydrate conjugates		C17058	38.9	2.39	M-H2O-H	C21H30O11	0.885123081	13318.82556	14399.72309	14160.55311	13025.76761	12672.23473	13457.41772	14669.93716	14306.02563	14028.5607	
4.97_252.1607m/z	252.1607119	4.969683333	neg	N-Desmethyl tapentadol	HMDB0060611	Benzenoids	Benzene and substituted derivatives	Phenylpropanes	*		38.9	0	M+FA-H	C13H21NO	0.939993553	923.3806834	897.5022225	924.1082565	729.2855429	627.228219	549.8713156	846.6516008	805.5994161	754.3675557	
5.08_313.0932m/z	313.0932241	5.080566667	neg	Idebenone Metabolite (QS-4)	761	Unclassified	Unclassified	Unclassified			38.9	0	M+FA-H	C13H16O6	1.242032003	1310.421778	1527.315429	1357.039608	849.3740909	667.6835352	830.4924225	1050.976639	991.7613966	998.4719542	
5.08_391.1403m/z	391.1403482	5.080566667	neg	XANTHOXYLIN	43856	Organic oxygen compounds	Organooxygen compounds	Carbonyl compounds	*	C10726	38.9	0	2M-H	C10H12O4	1.29254355	1512.260263	1350.620699	1430.949223	538.9799121	454.5185555	394.1345287	2570.729587	2014.660597	2153.413791	
5.08_433.2085m/z	433.2085139	5.080566667	neg	5a,6a-Epoxy-7E-megastigmene-3a,9e-diol 3-glucoside	87854	Organic oxygen compounds	Organooxygen compounds	Carbohydrates and carbohydrate conjugates			38.9	0	M+FA-H	C19H32O8	1.527715132	791.5567874	985.4736501	1010.520848	1388.767325	1932.850433	1813.429358	1229.724873	1220.942028	1149.510866	
5.16_393.1262m/z	393.1261536	5.155116667	neg	N2-(2-Carboxymethyl-2-hydroxysuccinoyl)arginine	HMDB0032766	Organic acids and derivatives	Carboxylic acids and derivatives	Amino acids, peptides, and analogues			38.9	0	M+FA-H	C12H20N4O8	-0.467760549	857.1872394	1193.699955	769.8664064	1895.80723	1733.641594	1705.646098	1004.407587	1138.872918	1155.72988	
5.22_427.2335m/z	427.23355	5.222166667	neg	Sarcostin	71890	Unclassified	Unclassified	Unclassified			38.9	0	M+FA-H	C21H34O6	-0.501538246	105.0427387	658.9006582	428.2538183	1010.677699	481.1679182	881.1500828	198.6979172	107.9039688	563.9248363	
5.24_292.1192m/z	292.1191826	5.244033333	neg	Oxomethylphenidate	1229	Unclassified	Unclassified	Unclassified			38.9	0	M+FA-H	C14H17NO3	0.551820919	692.5472333	491.5385196	768.8193024	1349.016239	1473.95513	1201.119791	630.8413992	751.9994517	774.6809935	
5.29_185.1184m/z	185.1183522	5.28555	neg	1-nonen-3-one	HMDB0303853	Organic oxygen compounds	Organooxygen compounds	Carbonyl compounds	*		38.9	0	M+FA-H	C9H16O	0.243769793	1422.953561	1241.982164	1324.610442	1733.18831	2024.080063	1994.8831	1969.338036	1526.144836	2209.764461	
5.34_411.0734m/z	411.0733538	5.3394	neg	Methyl (Z)-5-(5-methyl-2-thienyl)-2-penten-4-ynoate	HMDB0033995	Lipids and lipid-like molecules	Fatty Acyls	Fatty acid esters	*		38.9	0.919	2M-H	C11H10O2S	0.798646745	11031.92916	13821.44466	14894.08789	12394.9845	13506.1538	11631.9976	11011.90721	12121.06908	11919.84542	
5.43_330.1924m/z	330.192388	5.428733333	neg	(2E)-octenoylcarnitine	LMFA07070014	Lipids and lipid-like molecules	Fatty Acyls	Fatty esters			38.9	0	M+FA-H	C15H27NO4	0.619839353	1459.797314	1572.9242	1870.26306	3763.356898	3417.656614	3401.134079	2479.938702	2653.237007	2113.895197	
5.50_343.1764m/z	343.176367	5.497816667	neg	13,14-dihydro-15-keto-tetranor Prostaglandin D2	45459	Unclassified	Unclassified	Unclassified			38.9	0	M+FA-H	C16H26O5	0.47005311	3459.704787	2741.37852	2896.39475	3090.147919	3458.159987	4395.037692	3188.046132	2933.451738	3982.862824	
5.52_183.1027m/z	183.1026869	5.516816667	neg	(3E,6E)-nona-3,6-dienal	75326	Unclassified	Unclassified	Unclassified			38.9	0	M+FA-H	C9H14O	0.137093468	1795.534837	1479.303794	1401.721751	1437.20846	1787.034171	1618.247171	1804.509377	1622.53751	1798.104852	
5.53_331.1299m/z	331.1298848	5.525416667	neg	Schradan	72766	Unclassified	Unclassified	Unclassified			38.9	0	M+FA-H	C8H24N4O3P2	-2.383579765	714.0072767	864.1163648	757.4231118	478.4879391	569.1493006	419.2264303	496.9563571	451.1191063	573.1652734	
5.57_433.2090m/z	433.2089587	5.57225	neg	5-hydroxysebacate	86076	Organic acids and derivatives	Hydroxy acids and derivatives	Medium-chain hydroxy acids and derivatives			38.9	0	2M-H	C10H17O5-	2.39025403	5076.856795	4922.086619	4832.735798	5097.3892	5793.566014	5118.306093	4994.018289	5031.369577	4374.362185	
5.61_439.1613m/z	439.161327	5.610733333	neg	p-Hydroxyl-ethotoin	HMDB0061174	Organoheterocyclic compounds	Azolidines	Imidazolidines	*		38.9	0	2M-H	C11H12N2O3	-2.228761073	1858.681874	2084.489916	2444.177951	2376.011008	2018.855845	1733.456476	2457.446911	1980.686706	2165.582303	
5.63_327.1448m/z	327.1448267	5.628083333	neg	Artabsinolide D	90319	Organoheterocyclic compounds	Lactones	Gamma butyrolactones	*		38.9	0	M+FA-H	C15H22O5	-0.354229264	1230.920035	1319.429234	1397.136482	1853.059934	1670.983275	1716.674604	1239.855365	1042.313027	1150.785207	
5.69_257.1760m/z	257.1759964	5.6875	neg	Methyl (E)-2-dodecenoate	LMFA07010931	Lipids and lipid-like molecules	Fatty Acyls	Fatty acid esters	*		38.9	0	M+FA-H	C13H24O2	0.770684851	1783.744154	1658.438762	1718.95499	1296.556987	1551.253404	1545.24436	1147.942043	1359.485114	1751.180015	
5.73_198.1137m/z	198.1136651	5.727283333	neg	2-Methyl-4-pentyloxazole	92662	Organoheterocyclic compounds	Azoles	Oxazoles			38.9	0	M+FA-H	C9H15NO	0.640635734	1269.056582	1173.115518	1063.233657	1421.119653	2162.609959	1859.423333	1697.457833	2371.939999	2364.412254	
5.76_163.0763m/z	163.0763227	5.7632	neg	(3-Methylphenyl)methyl acetate	HMDB0041473	Benzenoids	Benzene and substituted derivatives	Benzyloxycarbonyls	**	C07216	38.9	0	M-H	C10H12O2	-0.79530721	1645.717424	1714.136729	1690.075046	968.1284992	950.1816049	1125.178962	2164.840701	2096.672864	1839.924166	
5.93_281.1032m/z	281.1031598	5.927283333	neg	3-Dimethylallyl-4-hydroxymandelic acid	64596	Benzenoids	Phenols	1-hydroxy-2-unsubstituted benzenoids	*	C11527	38.9	0	M+FA-H	C13H16O4	0.414882305	281.0843778	260.2430871	196.4597013	433.6208144	427.2545554	466.9822188	415.824348	592.0078447	678.194209	
5.95_635.1915m/z	635.1914597	5.95155	neg	Isopentyl pyrophosphate	373	Unclassified	Unclassified	Unclassified			38.9	0	2M-H	C10H24O7P2	-1.126378225	1289.665997	1349.01644	2050.518307	440.3704561	447.8978602	618.260094	1494.913439	1899.185204	2425.298589	
5.98_313.1197m/z	313.1197229	5.9829	neg	Lysergic acid	66676	Unclassified	Unclassified	Unclassified			38.9	0	M+FA-H	C16H16N2O2	1.27656778	4541.611666	4816.82179	5645.029542	4516.964737	5454.634826	5974.125036	4258.191752	4994.185343	4728.357796	
5.99_173.1183m/z	173.1183402	5.9916	neg	(R)-Sulcatol	HMDB0030030	Lipids and lipid-like molecules	Fatty Acyls	Fatty alcohols	*	C07288	38.9	0	M+FA-H	C8H16O	0.173126565	571.7483518	557.7489005	675.3501317	551.2282743	644.6811355	696.3173221	685.1043281	536.8101351	504.0086123	
6.01_271.1189m/z	271.1189463	6.007266667	neg	3-Aminobenzamide	HMDB0245814	Unclassified	Unclassified	Unclassified			38.9	0	2M-H	C7H8N2O	-4.053520499	2275.325235	2443.773584	2859.954451	2873.720642	3370.239177	3206.270362	2762.070843	2754.549593	2874.112703	
6.07_183.1027m/z	183.1026895	6.07	neg	(2E,4E)-nona-2,4,-dienal	75324	Unclassified	Unclassified	Unclassified			38.9	0	M+FA-H	C9H14O	0.155812058	1929.721671	2156.735389	2127.292715	2019.083332	2078.927343	1911.505031	1905.731288	1965.261219	2051.181748	
6.10_229.1446m/z	229.1445928	6.101316667	neg	3-Hexenyl 2-methylbutyrate	88361	Lipids and lipid-like molecules	Fatty Acyls	Fatty acid esters	*		38.9	0	M+FA-H	C11H20O2	0.325928246	6829.381304	7375.586393	7755.3617	7047.556269	7473.27973	6757.169815	5139.232469	4863.165708	5056.400988	
6.20_293.0541m/z	293.0541487	6.195716667	neg	Pyridoxamine 5'-phosphate	HMDB0001555	Organoheterocyclic compounds	Pyridines and derivatives	Pyridoxamines	**	C00647	38.9	0	M+FA-H	C8H13N2O5P	-1.057693135	1088.343525	903.6238934	1237.039457	1764.650436	1619.027613	1985.266561	1135.820824	1482.858024	1381.22451	
6.22_311.1503m/z	311.15033	6.215	neg	Humulinic acid A	86723	Organic acids and derivatives	Vinylogous acids	Unclassified	*		38.9	0	M+FA-H	C15H22O4	1.194630627	7596.285408	6834.891745	8204.309386	12596.23474	11927.63169	12254.32564	7267.937898	7283.906305	7751.956332	
6.27_259.1551m/z	259.1551418	6.272366667	neg	4-Acetoxy-2-hexyltetrahydrofuran	HMDB0037398	Organoheterocyclic compounds	Tetrahydrofurans	Unclassified			38.9	0	M+FA-H	C12H22O3	0.207229593	2186.477874	2058.987075	2355.252358	2663.337357	2562.274342	2582.03889	1733.316381	2083.165493	2088.507087	
6.28_271.1553m/z	271.1553086	6.2811	neg	Methyl dihydrojasmonate	HMDB0031740	Lipids and lipid-like molecules	Fatty Acyls	Lineolic acids and derivatives	*		38.9	0	M+FA-H	C13H22O3	0.933748437	7904.779381	8254.957775	9343.734578	6966.105729	7450.323915	7234.129366	6188.799829	6161.870735	6610.226599	
6.32_331.2130m/z	331.2129904	6.320716667	neg	2-Methoxy-3-(2-methylpropyl)pyrazine	87979	Organoheterocyclic compounds	Diazines	Pyrazines	*		38.9	0	2M-H	C9H14N2O	-2.887627974	927.6614339	1487.824268	1195.569507	1044.327227	756.6328646	998.1840172	967.9334734	1046.217306	1315.015002	
6.42_173.1184m/z	173.1183637	6.41995	neg	2,5-Diethyltetrahydrofuran	HMDB0029574	Organoheterocyclic compounds	Tetrahydrofurans	Unclassified	*		38.9	0	M+FA-H	C8H16O	0.356981745	1308.10968	1334.089805	1422.655779	1455.825206	1499.93932	1574.746379	1804.319827	1615.425443	1547.783779	
6.42_259.1552m/z	259.1551739	6.41995	neg	10-hydroxy-11-dodecenoic acid	74547	Lipids and lipid-like molecules	Fatty Acyls	Fatty Acids and Conjugates			38.9	0	M+FA-H	C12H22O3	0.357124623	3045.819172	3273.309226	3397.351625	3598.818735	3465.347861	3880.568243	3330.107401	3294.156851	3178.470455	
6.44_471.2245m/z	471.2244809	6.442716667	neg	Phenylalanyl-Alanine	85890	Organic acids and derivatives	Carboxylic acids and derivatives	Amino acids, peptides, and analogues	*		38.9	0	2M-H	C12H16N2O3	-0.90517911	2190.126538	2207.45531	2550.162087	1301.20609	913.3630155	1356.155874	2092.715828	2192.46114	1752.269016	
6.44_615.2040m/z	615.2040094	6.442716667	neg	Kanzonol U	86496	Phenylpropanoids and polyketides	2-arylbenzofuran flavonoids	Unclassified	*		38.9	0	2M-H	C19H16O4	2.544296024	2182.636788	2779.85444	2756.080576	2740.026319	2409.710826	3300.006586	3441.740149	3413.818729	2982.743011	
6.56_593.1444m/z	593.1443702	6.55905	neg	3-Hydroxynitrazepam	1568	Unclassified	Unclassified	Unclassified			38.9	0	2M-H	C15H11N3O4	2.919990999	6219.776675	7299.440791	7530.775245	8793.199907	8635.045678	8665.334351	4968.238782	6299.897331	5745.467453	
6.63_273.1710m/z	273.1709577	6.63275	neg	3-oxo-tridecanoic acid	LMFA01060097	Lipids and lipid-like molecules	Fatty Acyls	Fatty Acids and Conjugates			38.9	0	M+FA-H	C13H24O3	0.921289195	2500.845203	2388.570266	2221.964328	2858.513661	2901.273937	2737.270539	2109.470976	2281.299407	2339.595856	
6.74_343.2128m/z	343.2127844	6.739933333	neg	(4R,6S)-4-Hydroxy-1,7-dioxaspiro[5.5]undecane	102846	Unclassified	Unclassified	Unclassified			38.9	0	2M-H	C9H16O3	0.499908333	8447.867583	9036.49258	8928.413863	11033.10175	10565.06564	9923.218372	5224.024221	6235.031968	5602.87444	
6.75_215.1289m/z	215.1289478	6.747166667	neg	5Z-Octenyl acetate	86219	Lipids and lipid-like molecules	Fatty Acyls	Fatty alcohol esters	*		38.9	0	M+FA-H	C10H18O2	0.382921843	1773.471472	1751.57489	1821.896385	2951.331652	2478.575004	2409.909069	1915.686287	2201.951658	2005.847031	
6.78_193.0354m/z	193.035397	6.776866667	neg	2-Hydroxyglutarate	HMDB0059655	Organic acids and derivatives	Hydroxy acids and derivatives	Short-chain hydroxy acids and derivatives	**	C02630	38.9	0	M+FA-H	C5H8O5	0.140335671	1503.049182	1404.911994	1567.877485	2456.443239	2163.119435	2459.255579	1635.24719	1616.312584	1651.804341	
6.86_461.2399m/z	461.2399199	6.86375	neg	Dexfenfluramine	1956	Benzenoids	Benzene and substituted derivatives	Phenethylamines			38.9	0	2M-H	C12H16F3N	0.493281991	7036.352861	7605.389182	7174.476246	6151.844617	6555.689574	6305.881873	5984.087245	6954.424303	5865.253467	
6.88_181.1235m/z	181.123528	6.884583333	neg	(-)-Limonene	HMDB0004321	Lipids and lipid-like molecules	Prenol lipids	Monoterpenoids	**	C00521	38.9	0	M+FA-H	C10H16	0.915378502	1464.449377	1470.734763	1320.99229	1544.388892	1646.839505	1352.249062	1143.299323	1310.165122	1401.561609	
7.02_372.2394m/z	372.2393866	7.018266667	neg	(9E)-9-nitrooctadecenoic Acid	HMDB0062738	Lipids and lipid-like molecules	Fatty Acyls	Fatty acids and conjugates	*		38.9	0	M+FA-H	C18H33NO4	0.688260123	26528.76667	24654.94189	22141.79352	23718.66025	24791.40394	22936.59365	27627.64284	25847.92362	24868.17619	
7.07_411.2025m/z	411.2024669	7.0658	neg	Pheneturide	HMDB0256403	Unclassified	Unclassified	Unclassified			38.9	0	2M-H	C11H14N2O2	-3.183017612	2822.39662	2619.43487	3004.614386	1164.233667	1195.922679	1131.234894	1989.231207	2422.964092	2202.883602	

7.11_237.1134m/z	237.1133858	7.114116667	neg	4-Phenyl-2-butyl acetate	HMDB0031614	Benzenoids	Benzene and substituted derivatives	Unclassified	*		38.9	0	M+FA-H	C12H16O2	0.79725889	1772.2169	1695.260449	1776.336125	1676.60414	2120.772168	1518.36795	981.0588826	1210.358114	1163.746738
7.20_611.2432m/z	611.2431554	7.201166667	neg	Lonchocarpin	LMPK12120012	Lipids and lipid-like molecules	Polyketides	Flavonoids			38.9	0	2M-H	C20H18O3	-1.23646579	12228.12238	13506.9885	12888.94607	9644.083413	9291.157817	10190.52525	7470.217254	9381.122212	7270.822635
7.39_549.1976m/z	549.1975841	7.3938	neg	Ethenodeoxyadenosine	6339	Organoheterocyclic compounds	Imidazopyrimidines	Purines and purine derivatives	*		38.9	0	2M-H	C12H13N5O3	2.1481593	3155.100661	3342.232347	3061.085343	638.0323558	659.279187	489.4116995	1763.576527	2064.829955	2206.678728
7.47_415.0676m/z	415.0675597	7.46525	neg	Fraxetin	44399	Unclassified	Unclassified	Unclassified	***		38.9	0	2M-H	C10H8O5	1.176212568	4131.097136	3915.365153	4100.438439	3693.467811	4200.482722	4226.743051	4628.094905	3952.39148	4483.374231
7.55_433.2083m/z	433.2083191	7.547766667	neg	Rehmaionoside C	71699	Unclassified	Unclassified	Unclassified			38.9	0	M+FA-H	C19H32O8	1.025773343	9645.074756	10519.59196	10910.05186	12163.06527	11722.17247	11546.50396	5141.76224	5686.372672	5698.700266
7.59_439.1958m/z	439.1957716	7.58625	neg	1,2-Benzisoxazole, 6-fluoro-3-(4-piperidinyl)-	2351	Unclassified	Unclassified	Unclassified			38.9	0	2M-H	C12H13FN2O	1.511886699	11412.20687	13768.56504	12783.6552	13178.27882	13081.61232	13601.87042	11320.13128	13400.92366	12458.27277
7.67_464.3020m/z	464.3020225	7.66825	neg	Myxalamid S	69328	Unclassified	Unclassified	Unclassified			38.9	0	M+FA-H	C25H41NO4	0.622173203	17929.46211	19426.80862	21626.92079	5943.513866	6099.477072	6417.019327	10346.72922	11604.05925	11857.5825
7.72_457.1697m/z	457.1696675	7.719916667	neg	Benz[c]acridine	73044	Unclassified	Unclassified	Unclassified			38.9	0	2M-H	C17H11N	-2.956955843	10445.30731	12175.19804	11459.4674	12139.34724	13415.98592	12061.30565	8782.600845	9115.169948	10012.6797
7.83_173.1183m/z	173.1183366	7.8311	neg	2-Octanone	HMDB0031294	Organic oxygen compounds	Organooxygen compounds	Carbonyl compounds	*		38.9	0	M+FA-H	C8H16O	0.145451871	1258.51395	1143.719498	1351.725774	1179.207794	1058.384729	1345.147248	1128.080701	1097.063193	1083.497734
7.95_229.1445m/z	229.1445489	7.949583333	neg	2,3-Undecanedione	HMDB0031843	Organic oxygen compounds	Organooxygen compounds	Carbonyl compounds	*		38.9	0	M+FA-H	C11H20O2	0.08769355	1642.197823	1610.940904	1630.904387	3146.826186	2876.027615	3124.329928	1258.386472	1560.181956	1531.464478
8.11_146.0248m/z	146.0247776	8.110466667	neg	Agrocybin	LMFA08010027	Organic acids and derivatives	Carboxylic acids and derivatives	Carboxylic acid derivatives	*		38.9	0	M-H	C8H5NO2	0.17458338	207.467257	178.1883612	136.7498907	174.6506224	297.6599216	192.7211048	186.2206748	221.0360785	206.4691176
8.16_395.2078m/z	395.2077671	8.16495	neg	PGE3	3494	Lipids and lipid-like molecules	Fatty Acyls	Eicosanoids	*	C06439	38.9	0	M+FA-H	C20H30O5	0.685661185	13315.85668	12037.0764	13261.49569	5775.65645	5564.86415	5807.832292	9062.574011	10488.88882	10250.31552
8.17_273.1710m/z	273.1710498	8.173766667	neg	2-keto tridecanoic acid	LMFA01060041	Lipids and lipid-like molecules	Fatty Acyls	Fatty Acids and Conjugates			38.9	0	M+FA-H	C13H24O3	1.32500875	842.5805199	634.5919618	800.8227756	1814.554084	1671.583609	1689.564948	461.2652022	445.5482111	501.0084177
8.50_465.2497m/z	465.2496654	8.495033333	neg	Dimethyl 3-methoxy-4-oxo-5-(8,11,14-pentadecatrienyl)-2-hexenedioate	HMDB0032099	Organic acids and derivatives	Keto acids and derivatives	Beta-keto acids and derivatives	*		38.9	0	M+FA-H	C24H36O6	0.651059691	12854.5767	14031.83411	13923.19603	16884.08745	15553.27333	16652.59153	12103.63329	14342.66047	13962.72219
8.50_533.2371m/z	533.2371298	8.495033333	neg	Agaritine	65610	Unclassified	Unclassified	Unclassified			38.9	0	2M-H	C12H17N3O4	1.112134421	2324.714743	3075.905767	2582.512738	3453.287107	3321.774657	3848.48742	2301.700306	2535.430138	2797.054551
8.74_209.1548m/z	209.1548495	8.7445	neg	5-Ethyl-3-methyl-2E,4E,6E-nonatriene	LMFA11000051	Lipids and lipid-like molecules	Fatty Acyls	Hydrocarbons			38.9	0	M+FA-H	C12H20	0.889035924	511.8153969	470.0115623	530.5884579	679.4779915	937.3570196	845.3825535	394.4939702	507.5392303	475.109944
8.80_271.1552m/z	271.155206	8.803433333	neg	DIHYDROJASMONIC ACID, METHYL ESTER	43916	Unclassified	Unclassified	Unclassified			38.9	0	M+FA-H	C13H22O3	0.480148198	1697.409709	1994.314965	1809.341066	3769.489066	3747.297715	3959.939192	1188.947502	1548.078835	1355.273686
8.82_453.2134m/z	453.2133815	8.8245	neg	Cascarillin	HMDB0036836	Lipids and lipid-like molecules	Prenol lipids	Diterpenoids	*	C09071	38.9	0	M+FA-H	C22H32O7	0.919357134	23577.09273	24166.3076	22300.41784	10366.05111	10177.11064	10351.95793	18210.88241	20655.26686	19095.07234
9.02_679.2304m/z	679.2304402	9.01635	neg	Lactodifucotetraose	58473	Lipids and lipid-like molecules	Fatty Acyls	Fatty acyl glycosides	*		38.9	0	M+FA-H	C24H42O19	0.328356414	24686.1141	32650.76639	29161.00602	1517.282353	1856.83696	1692.251744	11113.1172	13431.24827	12583.29069
9.24_395.2078m/z	395.2077739	9.2377	neg	PGD3	LMFA03010142	Lipids and lipid-like molecules	Fatty Acyls	Eicosanoids	*	C13802	38.9	0	M+FA-H	C20H30O5	0.705264719	47217.06353	50109.5376	51298.92966	25042.52564	25141.22323	24153.83905	35428.59696	40215.88369	37667.12581
9.28_171.0664m/z	171.0664025	9.278033333	neg	Cyclopropanecarboxylate	71167	Unclassified	Unclassified	Unclassified			38.9	0	2M-H	C4H6O2	0.697716363	650.0162112	535.9761785	471.2311832	571.7472127	638.128625	710.3176282	772.746428	740.5613355	566.8812882
0.67_225.0994m/z	225.0994261	0.6701	neg	1-(5-Hydroxy-2-pyrimidinyl)piperazine	1453	Unclassified	Unclassified	Unclassified			38.8	0	M+FA-H	C8H12N4O	0.623071422	1207.357857	1220.911813	1159.801811	991.7843506	1122.807526	858.5533516	1362.245159	1709.394315	1537.22138
0.67_259.0787m/z	259.0786639	0.6701	neg	4-Fluorocyclohexadiene-cis,cis-1,2-diol	71224	Unclassified	Unclassified	Unclassified			38.8	0	2M-H	C6H7FO2	-0.28835384	125.702444	171.2244947	119.1834603	810.3563481	290.8749029	658.0068392	439.3739069	497.8288174	167.3511833
0.74_147.0413m/z	147.041252	0.7359	neg	3-Amino-2-oxazolidone	HMDB0060739	Organoheterocyclic compounds	Azolidines	Oxazolidines	*		38.8	0	M+FA-H	C3H6N2O2	1.192058949	1333.515471	1223.034845	1355.692117	1278.818341	1614.733906	1629.900121	1265.781785	1057.548172	1385.41197
0.76_201.1358m/z	201.1358055	0.757316667	neg	Symmetric dimethylarginine	HMDB0003334	Organic acids and derivatives	Carboxylic acids and derivatives	Amino acids, peptides, and analogues	*		38.8	0	M-H	C8H18N4O2	0.524853486	3718.265327	3078.59201	3727.490672	1340.096453	2811.632956	1891.743084	2859.841826	4312.896648	3908.185414
0.76_203.0207m/z	203.0206819	0.757316667	neg	Dihydro-3(2H)-thiophenone	87518	Organoheterocyclic compounds	Thiolanes	Unclassified	*		38.8	0	2M-H	C4H6OS	0.425124883	5884.798679	3713.9807	1532.791134	784.2123502	0.001403199	475.2316108	2407.792739	3820.928116	1035.729803
0.76_258.0385m/z	258.0384846	0.757316667	neg	Glucosamine-1P	HMDB0001109	Organic oxygen compounds	Organooxygen compounds	Carbohydrates and carbohydrate conjugates	*		38.8	0	M-H	C6H14NO8P	0.223969957	1973.106848	1720.455886	1001.85526	1976.599437	1615.131273	1725.395992	1333.699144	2046.152748	1138.678915
0.86_162.0409m/z	162.040882	0.86335	neg	L-Aspartate-semialdehyde	HMDB0012249	Organic acids and derivatives	Carboxylic acids and derivatives	Amino acids, peptides, and analogues	**	C00441	38.8	0	M+FA-H	C4H7NO3	0.73493971	85.39614759	139.6995565	260.9011643	104.7208591	197.5393563	215.9153636	119.383035	231.1139417	237.1754398
0.86_326.0649m/z	326.0649499	0.86335	neg	Flufenamic acid	HMDB0252331	Unclassified	Unclassified	Unclassified			38.8	0.755	M+FA-H	C14H10F3NO2	1.365691236	10389.58388	10994.06114	12916.05654	7292.980501	7587.366674	7101.224056	9957.26173	11230.41814	9971.24067
0.88_312.0590m/z	312.0589627	0.884633333	neg	2-Amino-4-oxo-6-(1',2'-dioxoprolyl)-7,8-dihydroxypteridine	HMDB0001410	Organoheterocyclic compounds	Pteridines and derivatives	Pterins and derivatives	*	C05255	38.8	0	M+FA-H	C9H9N5O5	1.465589186	5454.42756	6100.217835	7106.775667	4390.94331	6641.057991	4167.666313	6647.466481	4527.429705	5621.62912
1.22_290.9907m/z	290.9907374	1.224233333	neg	Flupropanate	72616	Unclassified	Unclassified	Unclassified			38.8	0	2M-H	C3H2F4O2	-0.584105718	3897.725278	3869.736209	3873.989023	9895.907499	8313.777649	8980.542234	5541.653263	6595.471268	6517.07535
1.42_190.0723m/z	190.0722894	1.421683333	neg	Allysine	HMDB0001263	Organic acids and derivatives	Carboxylic acids and derivatives	Amino acids, peptides, and analogues	**	C04076	38.8	0	M+FA-H	C6H11NO3	1.332580852	1503.966676	1216.386058	1058.917117	1562.177456	1377.130011	1349.554565	1429.142335	1156.637719	1406.085933
1.42_392.0603m/z	392.060277	1.421683333	neg	Adenosine monophosphate	HMDB0000045	Nucleosides, nucleotides, and analogues	Purine nucleotides	Purine ribonucleotides	***	C00020	38.8	0	M+FA-H	C10H14N5O7P	-2.911118211	6294.88835	5817.692876	4454.418098	326.337021	163.0987255	85.31693974	4778.274303	3195.728342	4065.520207
1.44_383.1198m/z	383.1197755	1.43735	neg	Quinic acid	HMDB0003072	Organic oxygen compounds	Organooxygen compounds	Alcohols and polyols	***	C00296	38.8	0	2M-H	C7H12O6	0.717848031	3461.500055	3782.274401	2521.517156	578.3149896	330.3961605	387.5742224	468.1592312	210.2683449	421.8518824
1.46_203.0563m/z	203.0563239	1.459966667	neg	2-Methyl-3-oxopropanoic acid	6053	Organic oxygen compounds	Organooxygen compounds	Carbonyl compounds	**	C00349	38.8	0	2M-H	C4H6O3	1.03984615	437.8230356	558.6543808	512.4423304	707.784226	578.0153539	592.2140445	567.4206919	353.3215656	518.048195
1.55_267.1353m/z	267.1352759	1.553483333	neg	Miotine	69202	Unclassified	Unclassified	Unclassified			38.8	0	M+FA-H	C12H18N2O2	1.103899321	1153.617273	927.8599003	942.6117931	1019.562289	878.2262029	1154.317317	673.72313	1200.402212	878.8658843
1.59_277.0363m/z	277.0362917	1.5934	neg	Thorium	HMDB0029215	Homogeneous metal compounds	Homogeneous actinide compounds	Unclassified		C19157	38.8	0	M+FA-H	Th	0.169431049	581.4233094	591.8581554	673.0853884	710.2113506	603.5821511	724.7632129	574.0662184	639.8171614	594.4692652
1.80_145.0507m/z	145.0507226	1.798266667	neg	2,3-Pentanedione	87796	Organic oxygen compounds	Organooxygen compounds	Carbonyl compounds	*		38.8	0	M+FA-H	C5H8O2	0.902163133	1122.816646	1023.509308	1033.833262	868.9062515	807.1373547	1020.990533	1042.223181	1012.816577	1024.617482
1.86_111.0088m/z	111.0087778	1.86075	neg	3-Furoic acid	HMDB0000444	Organoheterocyclic compounds	Furans	Furoic acid and derivatives	*		38.8	0	M-H	C5H4O3	0.091544999	1330.101286	1291.107251	1352.432491	1103.909071	1094.948625	946.8643233	788.3977774	678.1565446	734.9860424
1.92_125.0244m/z	125.0243621	1.924916667	neg	5-Methylfuran-2-carboxylic acid	HMDB0059735	Organoheterocyclic compounds	Furans	Furoic acid and derivatives			38.8	0	M-H	C6H6O3	-0.44088601	1637.667134	1709.577497	1339.12779	628.9895308	681.3432774	722.2141728	1498.994108	1523.099879	1371.144795
11.76_367.1891m/z	367.1891038	11.76466667	neg	LysoPA(i-13:0/0:0)	HMDB0114764	Lipids and lipid-like molecules	Glycerophospholipids	Glycerophosphates	*		38.8	0	M-H	C16H33O7P	-0.026596935	2269.202146	1663.515406	1973.000991	2103.033562	2124.464943	2065.812817	3131.186815	2450.249167	2571.400635
2.15_300.0844n	299.0771327	2.154666667	neg	4-(β-D-Glucosyloxy)benzoate	64012	Unclassified	Unclassified	Unclassified			38.8	0	M-H, M+FA-H	C13H16O8	-0.360958911	6282.479622	6920.678181	6186.803945	10234.6306	9068.766077	9739.703073	6251.568716	6570.252885	7221.79057
2.18_125.0469m/z	125.0468582	2.180683333	neg	5-Aminoimidazole-4-carboxamide	629	Organoheterocyclic compounds	Azoles	Imidazoles	**	C04051	38.8	0	M-H	C4H6N4O	-0.208219803	325.8360521	226.697979	292.9852195	478.4489248	421.5988164	352.6907254	246.1920859	360.6822028	371.8200702
2.35_235.0727m/z	235.072656	2.34905	neg	6-Hydroxy-1H-indole-3-acetamide	87466	Organoheterocyclic compounds	Indoles and derivatives	Hydroxyindoles	*		38.8	0	M+FA-H	C10H10N2O2	1.186640506	302.9172363	189.9520694	267.9903914	523.5022391	548.7293591	511.9177321	386.6859869	442.0937958	583.5658393
2.51_145.0507m/z	145.050711	2.510633333	neg	Allyl acetic acid	LMFA01030007	Lipids and lipid-like molecules	Fatty Acyls	Fatty acids and conjugates	*		38.8	0	M+FA-H	C5H8O2	0.786293264	1389.602526	1327.57268	1554.639482	1603.160884	1577.381878	1670.60792	1589.540654	1214.367895	1516.361196
2.52_161.0820m/z	161.0820043	2.5212	neg	Methyl 3-methylbutanoate	LMFA07010950	Lipids and lipid-like molecules	Fatty Acyls	Fatty acid esters	*		38.8	0	M+FA-H	C6H12O2	0.618587962	1268.089746	1470.073097	1494.055816	1341.95504	1213.504702	1353.484376	1282.871013	1151.029779	1249.015186
2.54_273.0594m/z	273.0594293	2.5421	neg	2-Azahypoxanthine	625	Unclassified	Unclassified	Unclassified			38.8	0	2M-H	C4H3N5O	-2.969156381	2210.620884	2357.685794	2629.47157	2783.162326	2485.176896	2525.196209	1664.320815	1703.911143	1808.095949
2.67_186.0409m/z	186.0408851	2.6698	neg	2-aminomuconic acid semialdehyde	LMFA01060191	Organic acids and derivatives	Carboxylic acids and derivatives	Amino acids, peptides, and analogues	**	C03824	38.8	0	M+FA-H	C6H7NO3	0.631948936	1337.317867	1466.965839	1454.846243	2109.570243	1898.738338	1826.454851	1489.550345	1724.947977	1643.66701
2.96_182.0459m/z	182.0458671	2.962916667	neg	Trigonelline	HMDB0000875	Alkaloids and derivatives	Unclassified	Unclassified	**	C01004	38.8	0	M+FA-H	C7H7NO2	-0.103925856	1868.929762	1747.56906	1637.951325	787.2979076	795.0119726	721.7995502	653.6042841	687.2345433	655.8308595
3.00_415.0737m/z	415.073675	3.004583333	neg	2-(2'-Methylthio)ethylmalic acid	64491	Unclassified	Unclassified	Unclassified			38.8	0	2M-H	C7H12O5S	-0.330589592	1117.23244	1119.055989	1265.916655	2567.392891	3143.38827	3047.177217	2271.575461	2033.870116	1763.681904
3.06_124.0165m/z	124.0164659	3.05735	neg	D-(1-Aminoethyl)phosphonate	66044	Unclassified	Unclassified	Unclassified			38.8	0	M-H	C2H8NO3P	-3.498764146	8058.557961	8117.598834	8863.794565	618.6806749	663.4886139	638.3068662	6677.794456	6985.498377	6921.095274
3.06_146.0823m/z	146.0822809	3.05735	neg	5-Aminopentanal	64437	Organic oxygen compounds	Organooxygen compounds	Carbonyl compounds	**	C12455	38.8	0	M+FA-H	C5H11NO	0.139224092	121.248201	150.4554525	132.3495771	313.4086642	302.9579896	331.867105	180.9623768	248.989688	269.4672335
3.32_277.1194m/z	277.1194142	3.324533333	neg	5-Acetyl-2,4-dimethyloxazole	92910	Organoheterocyclic compounds	Azoles	Oxazoles			38.8	0	2M-H	C7H9NO2	0.120752173	1213.19166	1472.794904	943.7599378	1741.159877	1796.789675	1703.441082	1141.014189	1654.627785	1250.67356
3.71_254.1147m/z	254.1147176	3.708366667	neg	4-(Methylnitrosamino)-1-(3-pyridyl)-1-butanol	HMDB0041809	Organoheterocyclic compounds	Pyridines and derivatives	Unclassified	**	C19574	38.8	0	M+FA-H	C10H15N3O2	0.420609986	1160.952778	1012.493671	1014.03102	734.1196267	470.5291173	525.048909	580.5312543	767.5777836	672.3622408
3.73_301.0568m/z	301.0567703	3.730516667	neg	PISCIDIC ACID	43637	Phenylpropanoids and polyketides	Phenylpropanoic acids	Unclassified	*		38.8	0	M+FA-H	C11H12O7	1.033668084	1014.555903	887.9591943	929.57486	412.2490309	555.4675921	431.7013402	359.3623786	305.3616295	385.798113
3.78_639.0561m/z	639.0561149	3.779133333	neg	Erosnin	48335	Phenylpropanoids and polyketides	Isoflavonoids	Coumestans	*		38.8	0	2M-H	C18H8O6	-1.225764484	3065.134962	1896.66473	2044.27631	723.4587791	541.7926155	1056.782796	1608.720371	1595.472059	1880.994864
3.85_219.1239m/z	219.1239203	3.84605	neg	Nonanoic acid, 8-hydroxy-, (R)-; (-)-(R)-8-Hydroxynonanoic acid	74639	Unclassified	Unclassified	Unclassified			38.8	0	M+FA-H	C9H18O3	0.706580454	2764.634346	3045.855028	3204.043102	2860.720578	2656.74467	2636.299895	2456.170546	2673.206022	2547.770735
3.94_323.0386m/z	323.038639	3.9351	neg	Ribavirin 5'-phosphate	2297	Unclassified	Unclassified	Unclassified			38.8	0	M-H	C8H13N4O8P	-3.655605716	46681.37565	47675.85443	51098.60892	1918.64563	2118.466784	2170.046675	40642.59004	44669.26591	42827.78182
4.02_301.0930m/z	301.0929984	4.024283333	neg	Phenylgalactoside	65793	Unclassified	Unclassified	Unclassified			38.8	0	M+FA-H	C12H16O6	0.418984149	182.6507508	292.8605207	496.5775008	1098.51989	759.0530854	894.2767114	2720.265576	2565.471844	2726.592367
4.05_299.0775m/z	299.0774948	4.046666667	neg	Dimethyl fukiic acid	HMDB0029497	Benzenoids	Phenols	Benzenediols	*		38.8	0	M-H	C13H16O8	0.845627595	554.1635711	790.8477743	854.6048289	318.965399	221.5866289	284.3001998	280.6534112	460.057148	331.8690694
4.07_227.1040m/z	227.1039775	4.069216667	neg	Hydroxyprolyl-Proline	85777	Organic acids and derivatives	Carboxylic acids and derivatives	Amino acids, peptides, and analogues	*		38.8	0	M-H	C10H16N2O4	1.08243655	400.8789354	465.2460104	510.3923704	413.0068186	375.3955416	374.3192734	390.3760391	412.5473471	403.9566637
4.07_276.1566m/z	276.1566061	4.069216667	neg	gamma-Aminobutyryllysine	HMDB0001959	Organic acids and derivatives	Peptidomimetics	Hybrid peptides	*		38.8	0.344	M+FA-H	C10H21N3O3	0.483107005	331.4112839	390.1224123	198.7253541	379.8855669	2562.729657	931.3173232	192.8301481	266.755291	884.1652296
4.11_477.8656m/z	477.865564	4.113883333	neg	3,5-Diiodo-L-tyrosine	HMDB0003474	Organic acids and derivatives	Carboxylic acids and derivatives	Amino acids, peptides, and analogues	**	C01060	38.8	0	M+FA-H	C9H9I2NO3	0.397232122	3046.251507	3188.561698	3101.465292	3162.171857	3583.022648	3352.9606	4322.911047	2779.780361	3425.921262
4.16_218.0460m/z	218.0460179	4.15895	neg	1-nitronaphthalene	HMDB0062188	Benzenoids	Naphthalenes	Nitronaphthalenes	**	C14040	38.8	0	M+FA-H	C10H7NO2	0.789300975	431.8075084	415.8939345	421.3455716	547.1056226	298.7057535	258.4648012	662.9831948	413.8271253	499.6379825
4.16_272.0155m/z	272.0154587	4.15895	neg	Trinitrotoluene	71179	Unclassified	Unclassified	Unclassified			38.8	0	M+FA-H	C7H5N3O6	-2.550596125	3434.09807	2986.763552	3341.177639	4615.053869	3212.090791	3547.205406	4280.962125	3122.223734	3316.474771
4.18_145.0507m/z	145.0507321	4.181333333	neg	Angelic acid	LMFA01020029	Lipids and lipid-like molecules	Fatty Acyls	Fatty acids and conjugates	*		38.8	0	M+FA-H	C5H8O2	0.996959358	1246.510509	1179.372275	1136.528894	1176.648199	1203.162198	1142.392176	1386.706492	985.7871908	1263.866629
4.18_155.0351m/z	155.0351027	4.181333333	neg	Hydroquinone	HMDB0002434	Benzenoids	Phenols	Benzenediols	**	C00530	38.8	0	M+FA-H	C6H6O2	1.094042731	327.4445615	400.2230946	376.1991022	446.8670953	347.7552712	381.7390773	534.8679943	353.1561756	423.8600494
4.21_124.0404m/z	124.040378	4.212116667	neg	N-ethylmaleimide	HMDB0255137	Unclassified	Unclassified	Unclassified			38.8	0	M-H	C6H7NO2	-0.192112915	113.1166353	153.3384553	233.1396347	178.5085764	176.1609961	202.8981555	210.1311235	110.324016	128.2824784
4.26_152.0474n	151.0401028	4.2568	neg	p-Hydroxyphenylacetic acid	HMDB0000020	Benzenoids	Phenols	1-hydroxy-2-unsubstituted benzenoids	***	C00642	38.8	0	M-H, M+FA-H	C8H8O3	0.231249958	2137.619386	2062.542471	2059.412415	2948.279965	2731.418779	2625.818608	2518.629591	2472.18187	2515.926645
4.28_279.9898m/z	279.9897939	4.278183333	neg	2-Methyl-4-amino-5-hydroxymethylpyrimidine diphosphate	3426	Unclassified	Unclassified	Unclassified			38.8	0	M-H2O-H	C6H11N3O7P2	1.378133209	170284.689	179972.3857	178700.3623	13866.23991	12551.34563	12582.05835	154097.5251	165811.4207	159895.5685
4.37_180.0788n	225.0759114	4.367166667	neg	4-Ethoxy-3-methoxybenzaldehyde	HMDB0029685	Benzenoids	Benzene and substituted derivatives	Benzoyl derivatives	*		38.8	0	M-H, M+FA-H	C10H12O3	0.733184788	1431.532923	1633.646988	1751.10595	2175.447954	1593.672623	1889.730585	1193.171789	1654.851839	1428.932643
4.50_298.1663m/z	298.1662513	4.49925	neg	O-Demethylmetoprolol	1252	Unclassified	Unclassified	Unclassified			38.8	0	M+FA-H	C14H23NO3	1.006433965	2076.530328	2104.585479	2104.088857	956.5683589	1027.847757	1043.905772	1712.098636	1761.760457	2139.895349
4.52_409.0664m/z	409.0663617	4.521733333	neg	6-Hydroxykynurenate	66328	Organoheterocyclic compounds	Quinolines and derivatives	Quinoline carboxylic acids		C08480	38.8	0	2M-H	C10H7NO4	-3.358559277	1234.83081	1340.61818	1353.094047	1449.185629	1393.242592	1179.47405	1543.08053	1569.689311	1414.045132
4.54_145.0871m/z	145.0871111	4.54425	neg	3-Hexen-1-ol	HMDB0030003	Lipids and lipid-like molecules	Fatty Acyls	Fatty alcohols	**	C08492	38.8	0	M+FA-H	C6H12O	0.931714573	659.306689	704.2852269	694.7081356	777.4616008	707.1075547	828.272335	916.5317029	820.3493218	943.9363292
4.57_490.9777m/z	490.9777227	4.5668	neg	2'-Deoxyinosine triphosphate	HMDB0003537	Nucleosides, nucleotides, and analogues	Purine nucleotides	Purine deoxyribonucleotides	**	C01345	38.8	0	M-H	C10H15N4O13P3	0.310423241	3072.626674	3107.582662	2593.039983	1110.541221	1074.388334	1170.730536	1668.655961	2098.395984	1660.774677
4.61_300.1818m/z	300.1817766	4.611883333	neg	N-decanoyl-L-Homoserine lactone	45310	Unclassified	Unclassified	Unclassified			38.8	0	M+FA-H	C14H25NO3	0.509583615	298.6216161	366.9614619	435.3788338	315.8136653	332.0033772	270.8344788	287.6252557	426.8941189	592.9020766
4.63_477.8660m/z	477.8659525	4.632666667	neg	3,5-diiodo-L-tyrosinate(1-)	HMDB0062622	Organic acids and derivatives	Carboxylic acids and derivatives	Amino acids, peptides, and analogues			38.8	0	M+FA-H	C9H9I2NO3	1.294855762	1858.338603	1781.705784	1578.222891	2316.59095	2044.866164	2715.128099	2718.999125	1786.798837	1993.410458
4.68_387.0939m/z	387.0938764	4.682483333	neg	Caffeic acid 3-glucoside	68321	Unclassified	Unclassified	Unclassified			38.8	0	M+FA-H	C15H18O9	1.728765849	1014.987362	980.6226092	1243.058093	614.6232964	671.8903462	368.3496604	817.0152127	484.1138725	846.3282617
4.70_141.0558m/z	141.0558203	4.703166667	neg	2-Methyl-2-cyclopenten-1-one	HMDB0031545	Organic oxygen compounds	Organooxygen compounds	Carbonyl compounds	*		38.8	0	M+FA-H	C6H8O	1.06747151	2563.683561	2476.967537	2526.404665	2758.334426	2853.454749	2780.482854	3153.908206	2662.840848	2987.415205
4.70_457.2019m/z	457.2019177	4.703166667	neg	Adapalene	64832	Lipids and lipid-like molecules	Prenol lipids	Retinoids			38.8	0	M+FA-H	C28H28O3	-0.315274764	929.7903684	798.3861349	932.9596857	996.9819502	1086.11572	1236.530937	653.3376213	622.7786384	289.9077839
4.84_377.1197m/z	377.1197203	4.836566667	neg	Mytilin A	93608	Organic acids and derivatives	Carboxylic acids and derivatives	Amino acids, peptides, and analogues			38.8	0	M+FA-H	C13H20N2O8	-1.34941615	608.7090608	491.4188669	557.0454933	838.2996135	995.2603055	844.8283637	351.9515195	408.2116776	650.2970194
4.84_421.1867m/z	421.1867363	4.836566667	neg	11,12,14-Trihydroxy-7-methoxy-8,11,13-abietatrien-20,6-olide	88046	Lipids and lipid-like molecules	Prenol lipids	Terpene lactones	*		38.8	0	M+FA-H	C21H28O6	-0.146620602	3279.418609	3230.173863	3253.568481	5053.690551	4719.776763	5013.851241	3583.640013	3669.416788	3692.089619
4.90_139.0401m/z	139.0401371	4.902316667	neg	3-METHOXYCATECHOL	44614	Unclassified	Unclassified	Unclassified			38.8	0	M-H	C7H8O3	0.495617564	170.3525629	168.3400142	191.2282917	195.6385918	175.9159324	208.5159212	216.8183039	181.2785673	219.3875821
5.10_515.1151m/z	515.1150706	5.1031	neg	Streptamine 4-phosphate	65978	Unclassified	Unclassified	Unclassified			38.8	0	2M-H	C6H15N2O7P	-1.991507824	516.4092776	742.0885805	563.7375023	1157.769981	1553.174654	1537.292399	1236.547718	651.3034681	1059.014145
5.13_421.1129m/z	421.1129471	5.132616667	neg	Nicorandil	HMDB0255584	Unclassified	Unclassified	Unclassified			38.8	0	2M-H	C8H9N3O4	3.818844894	4805.774234	4982.52867	4450.20979	5601.995231	5646.060943	5834.261799	5585.936934	4873.336295	4979.128054
5.13_499.1419m/z	499.1419303	5.125666667	neg	LY83583	63044	Unclassified	Unclassified	Unclassified			38.8	0	2M-H	C15H10N2O2	1.502767348	4195.169809	2963.274006	2826.861073	3602.625616	3225.291173	3375.813628	4242.381071	3447.673647	4373.247132
5.18_193.0507m/z	193.0506895	5.1777	neg	4-Hydroxycinnamyl aldehyde	44643	Phenylpropanoids and polyketides	Cinnamaldehydes	Unclassified	**	C05608	38.8	0	M+FA-H	C9H8O2	0.3861107	477.6815334	541.8868647	507.0948827	308.4760176	249.6857977	236.672087	388.8266629	334.9938531	401.3643508
5.18_563.0976m/z	563.0975862	5.1777	neg	3-Hydroxy-8,9-methylenedioxypterocarp-6a-ene	48246	Unclassified	Unclassified	Unclassified			38.8	0	2M-H	C16H10O5	-1.390209698	3219.588278	3563.775973	2887.172859	1853.616636	1364.518225	1875.252739	2376.061615	2017.435684	2168.547648
5.27_201.0228m/z	201.0228299	5.271716667	neg	3-(Acetylthio)-2-methylfuran	88237	Organosulfur compounds	Thioethers	Aryl thioethers	*		38.8	0	M+FA-H	C7H8O2S	0.810846553	842.8880601	870.112178	773.2269255	3772.159724	3881.369597	3797.628505	5995.26891	4861.843236	6258.985554
5.30_425.1455m/z	425.1455148	5.302883333	neg	Gibberellin A75	91891	Lipids and lipid-like molecules	Prenol lipids	Diterpenoids	*		38.8	0	M+FA-H	C19H24O8	0.510778905	5188.472066	5442.408177	6238.727466	6861.315645	10256.21898	10363.9346	6711.379681	4978.73808	7241.58279
5.34_181.0871m/z	181.0871275	5.3394	neg	2,3,6-Trimethylphenol	86411	Benzenoids	Phenols	Cresols	*		38.8	0	M+FA-H	C9H12O	0.805897437	296.9317255	342.6491901	368.5950177	463.8076423	575.3410583	517.0264861	409.5669214	422.0477216	496.2614896
5.36_325.0880m/z	325.0879786	5.361783333	neg	4-Hydroxy-L-glutamic acid	327	Organic acids and derivatives	Carboxylic acids and derivatives	Amino acids, peptides, and analogues	*	C03079	38.8	0	2M-H	C5H9NO5	-2.728576115	2221.520278	2043.654008	2278.893716	1830.740073	1689.09554	2245.298554	2061.93642	2626.935672	2814.219869
5.38_193.0354m/z	193.035391	5.384216667	neg	Citramalic acid	3273	Lipids and lipid-like molecules	Fatty Acyls	Fatty acids and conjugates	*	C00815	38.8	0	M+FA-H	C5H8O5	0.099972662	1361.93869	1469.007373	1566.615482	1873.661935	1891.420865	1949.650444	1414.596193	1577.197993	1601.199895
5.57_445.2081m/z	445.2081408	5.57225	neg	Cincassiol B	HMDB0036855	Lipids and lipid-like molecules	Prenol lipids	Diterpenoids	*		38.8	0	M+FA-H	C20H32O8	0.549604838	9966.806649	8954.84154	9062.894975	13336.26856	14024.08283	14534.84728	11976.02382	11505.56522	9292.605147
5.58_267.1239m/z	267.1239325	5.584533333	neg	(6S)-dehydrovomifoliol	53371	Lipids and lipid-like molecules	Prenol lipids	Sesquiterpenoids	*	C04223	38.8	0	M+FA-H	C13H18O3	0.6084195	2943.935728	2547.484942	2961.918444	3520.837396	3695.67476	3657.438121	3775.930935	3613.353229	2806.902944
5.59_398.1279m/z	398.1279252	5.5933	neg	N-Acetyl gemifloxacin	HMDB0060633	Benzenoids	Benzene and substituted derivatives	Diphenylmethanes	*		38.8	0	M-H2O-H	C19H20FN5O5	2.203857388	1934.346695	1236.298999	1968.790398	1494.919064	2101.500167	1049.480498	2656.971926	2377.712897	2239.439635
5.59_457.1697m/z	457.1697329	5.5933	neg	Ergothioneine	HMDB0003045	Organic acids and derivatives	Carboxylic acids and derivatives	Amino acids, peptides, and analogues	**	C05570	38.8	0	2M-H	C9H15N3O2S	0.030201961	3944.431552	3420.404802	3426.376076	4046.776195	4436.529451	4347.156772	3540.167264	3103.261738	3050.512735
5.60_479.2288m/z	479.2288085	5.602	neg	Ajugarin I	67391	Unclassified	Unclassified	Unclassified			38.8	0	M+FA-H	C24H34O7	0.350464028	12928.01687	12460.13567	14053.19305	19235.42365	19802.90773	18164.86298	16505.63397	15263.49646	14826.92192
5.74_317.1508m/z	317.150843	5.744566667	neg	Ergoline-8-methanol, 10-methoxy-, (8b)-	1516	Unclassified	Unclassified	Unclassified			38.8	0	M+FA-H	C16H20N2O2	0.596240762	453.7596529	606.2565602	519.568034	434.8535377	837.4540663	628.8062386	529.0684568	587.724575	651.7589567
5.76_155.0715m/z	155.0714647	5.7632	neg	2,3,5-Trimethylfuran	86445	Organoheterocyclic compounds	Heteroaromatic compounds	Unclassified	*		38.8	0	M+FA-H	C7H10O	0.880043671	1641.771551	1468.24755	1569.394737	1891.647554	2228.58515	1594.100458	2630.349341	2702.126848	2710.202336
5.81_259.1552m/z	259.1551511	5.806016667	neg	12-hydroxy-3Z-dodecenoic acid	74548	Lipids and lipid-like molecules	Fatty Acyls	Fatty Acids and Conjugates			38.8	0	M+FA-H	C12H22O3	0.250482214	1924.868344	1917.212201	2017.341468	2707.674931	2287.50502	2061.210096	2847.458607	2659.57829	2225.172718
5.85_263.1290m/z	263.1289839	5.84575	neg	Indoleamine	65657	Unclassified	Unclassified	Unclassified			38.8	0	2M-H	C8H8N2	-4.680088875	1025.152728	1359.486074	1691.832473	840.9469546	492.2128061	572.3822774	1416.756168	1190.630979	1035.155631
5.88_311.1505m/z	311.1504962	5.88025	neg	7-Octenoic acid, 5-oxo-	74797	Lipids and lipid-like molecules	Fatty Acyls	Fatty Acids and Conjugates			38.8	0	2M-H	C8H12O3	1.550924272	2324.91612	2689.275123	2874.12588	2536.251951	2312.677565	2274.51256	1037.322588	1375.886849	1459.405526
5.89_415.1611m/z	415.161137	5.894166667	neg	4-hydroxy-2,6-bis(1-methylethyl)phenyl glucuronide	2158	Unclassified	Unclassified	Unclassified			38.8	0	M+FA-H	C18H26O8	0.449401711	886.3051876	938.829952	1189.615929	1295.930832	1318.346622	1181.253529	707.5472264	727.1555727	1033.837983
5.94_303.1816m/z	303.1815675	5.937716667	neg	2-Methoxy-(3 or 5 or 6)-isopropylpyrazine	88408	Organic oxygen compounds	Organooxygen compounds	Ethers	*		38.8	0	2M-H	C8H12N2O	-3.557179317	1206.43319	1078.355638	1466.682522	875.6824047	983.9369842	1148.679977	899.1554779	1169.266972	1055.544438
5.98_299.1502m/z	299.1502337	5.9829	neg	6-methylnicotinamide	85256	Organoheterocyclic compounds	Pyridines and derivatives	Pyridinecarboxylic acids and derivatives	*		38.8	0	2M-H	C8H10N2O	-3.717371531	631.475768	688.4673877	716.9147629	1064.594943	1313.781001	1472.122704	618.3668748	597.4747448	521.635558
6.06_297.1709m/z	297.1708642	6.056116667	neg	(4R,5S,7R,11R)-11,12-Dihydroxy-1(10)-spirovetiven-2-one	HMDB0033149	Lipids and lipid-like molecules	Prenol lipids	Sesquiterpenoids	*		38.8	0	M+FA-H	C15H24O3	0.46287631	2248.250192	2636.979848	2576.329954	3174.787168	3190.220024	2916.380925	1606.195341	1624.582003	1648.474741
6.07_591.1292m/z	591.1291743	6.07	neg	2',5'-Dihydroxyflavone 5'-acetate	LMPK12110092	Unclassified	Unclassified	Unclassified			38.8	0	2M-H	C17H12O5	-0.838019709	5630.655408	5534.954858	6069.474713	3717.508943	4016.712392	3451.348628	5859.130928	6510.892778	5899.891504
6.09_253.1447m/z	253.1446777	6.087383333	neg	4-Heptyloxyphenol	HMDB0246434	Unclassified	Unclassified	Unclassified			38.8	0	M+FA-H	C13H20O2	0.69630643	4614.317653	5100.964889	5283.160704	5169.548115	5980.17751	5967.74369	4515.668503	4465.691623	4696.079798
6.11_167.1441m/z	167.1441197	6.113516667	neg	Undecanoic acid	HMDB0000947	Lipids and lipid-like molecules	Fatty Acyls	Fatty acids and conjugates	***	C17715	38.8	0	M-H2O-H	C11H22O2	-0.102678609	380.9831687	426.0157887	400.1064537	355.5232823	352.1881705	343.2102384	224.6614033	235.1039835	282.0425968

6.14_263.1290m/z	263.1289632	6.143466667	neg	1-Methylpyrrolo[1,2-a]pyrazine	89048	Organoheterocyclic compounds	Pyrrolopyrazines	Unclassified			38.8	0	2M-H	C8H8N2	-4.758511403	1328.726433	1537.955648	1426.786913	449.1013256	364.4049563	682.5040306	690.3394489	550.7647371	704.9608229
6.14_443.1926m/z	443.1926002	6.143466667	neg	3'-Hydroxy-T2-triol	90039	Lipids and lipid-like molecules	Prenol lipids	Sesquiterpenoids	*		38.8	0	M+FA-H	C20H30O8	0.827261133	12708.90917	14237.47784	14623.00445	6559.393466	7611.001593	7551.280752	9478.839024	11241.1412	12015.86619
6.17_397.0680m/z	397.0680317	6.171316667	neg	2-(4-Nitrobenzylidene)malononitrile	HMDB0245723	Organic oxygen compounds	Organooxygen compounds	Carbohydrates and carbohydrate conjugates	**	C06397	38.8	0	2M-H	C10H5N3O2	-2.62444918	1283.05483	1127.196325	1057.085738	340.6343916	326.9719495	300.9362932	828.8525018	841.9952672	1116.008559
6.22_309.1460m/z	309.1460289	6.215	neg	Subaphylline	HMDB0033463	Phenylpropanoids and polyketides	Cinnamic acids and derivatives	Hydroxycinnamic acids and derivatives	**	C10497	38.8	0	M+FA-H	C14H20N2O3	1.641217898	4096.300005	3286.187791	4086.293579	4643.046293	4174.234372	4464.090005	2922.428906	3185.194389	3298.174944
6.22_333.1327m/z	333.1327074	6.215	neg	(S)-Spinacine	86559	Organic acids and derivatives	Carboxylic acids and derivatives	Amino acids, peptides, and analogues	*		38.8	0	2M-H	C7H9N3O2	3.084802554	1052.057647	928.2342988	1178.21499	2047.435942	1700.327328	1820.771351	1187.621622	1222.924938	1190.795606
6.37_411.1658m/z	411.1658312	6.373833333	neg	Diacetoxyscirpenol	HMDB0035104	Lipids and lipid-like molecules	Prenol lipids	Sesquiterpenoids	*	C09662	38.8	0	M+FA-H	C19H26O7	-0.613985573	37721.85519	39236.00785	42312.58549	44422.00429	44581.72625	43586.5592	35515.2617	36264.23148	35826.83801
6.48_376.2133m/z	376.2132852	6.477433333	neg	2-[Octahydro-4,7-dimethyl-1-oxocyclopenta[c]pyran-3-yl]nepetalactam	HMDB0038596	Lipids and lipid-like molecules	Prenol lipids	Terpene lactones	*		38.8	0	M+FA-H	C20H29NO3	1.022141516	505.9519428	365.5814189	484.4508411	577.2852792	506.4358314	577.4310146	948.6745763	1002.905098	1210.965228
6.52_261.1708m/z	261.1708247	6.520516667	neg	beta-hydroxylauric acid	74493	Organic acids and derivatives	Hydroxy acids and derivatives	Medium-chain hydroxy acids and derivatives	*		38.8	0	M+FA-H	C12H24O3	0.356940164	2731.517466	3184.167481	3094.479224	2129.054632	2177.887679	1973.362991	2221.227771	2271.006987	2356.426888
6.57_212.1294m/z	212.129378	6.570233333	neg	Myrtine	HMDB0030371	Organoheterocyclic compounds	Quinolizines	Unclassified	*		38.8	0	M+FA-H	C10H17NO	0.962803493	3811.251861	4400.117692	4364.287212	4045.820924	4277.984299	3963.737668	3324.584429	3565.582523	3262.059898
6.58_464.3020m/z	464.3019821	6.582416667	neg	N-palmitoyl tyrosine	LMFA08020098	Organic acids and derivatives	Carboxylic acids and derivatives	Amino acids, peptides, and analogues			38.8	0	M+FA-H	C25H41NO4	0.525698594	9018.16988	9929.3319	9064.988238	2336.375569	2234.503929	2606.221453	7809.746101	9606.850797	8779.151238
6.62_389.0987m/z	389.0987433	6.61635	neg	4-Carboxyphenylglycine	6439	Organic acids and derivatives	Carboxylic acids and derivatives	Amino acids, peptides, and analogues	*		38.8	0	2M-H	C9H9NO4	-0.758284249	687.2282153	353.2235685	538.94792	1406.768184	1131.238696	1052.4539	906.9458578	1232.762003	1168.450792
6.63_352.0863m/z	352.0862744	6.63275	neg	(1R)-Hydroxy-(2R)-N-acetyl-L-cysteinyl-1,2-dihydronaphthalene	70328	Unclassified	Unclassified	Unclassified			38.8	0	M+FA-H	C15H17NO4S	0.789521113	30650.70796	31312.91362	30039.76936	58869.17938	53766.11829	53378.53395	51193.03058	55449.23061	56037.90444
6.76_302.1037m/z	302.1037467	6.759433333	neg	Mefenamic acid Metabolite (Anthranilic acid, N-(a3-hydroxy-2,3-xylyl)-)	1099	Unclassified	Unclassified	Unclassified			38.8	0	M+FA-H	C15H15NO3	1.36317595	599.7254509	568.966797	925.4070872	800.6986458	465.889171	670.5826418	517.1936605	469.1884067	353.6868895
6.95_463.2338m/z	463.2337508	6.951516667	neg	Melatonin	HMDB0001389	Organoheterocyclic compounds	Indoles and derivatives	Indoles	***	C01598	38.8	0	2M-H	C13H16N2O2	-2.861114609	1297.319475	1743.756095	1270.668709	2265.305267	2032.382686	1958.940171	1735.364333	2273.193729	2010.143449
7.20_273.1345m/z	273.1345358	7.201166667	neg	Pralidoxime	HMDB0014871	Organoheterocyclic compounds	Pyridines and derivatives	Methylpyridines		C07400	38.8	0	2M-H	C7H9N2O+	-4.244688113	1789.887356	2040.180642	2091.54294	2670.015491	2619.524735	2691.151813	1926.679182	2321.59528	1867.144619
7.24_239.1290m/z	239.1290284	7.237866667	neg	(S,Z)-Lyratol acetate	HMDB0031840	Organic acids and derivatives	Carboxylic acids and derivatives	Carboxylic acid derivatives	*		38.8	0	M+FA-H	C12H18O2	0.750557998	753.7738567	914.6701157	851.7848616	1145.724516	1210.855214	1032.525803	853.2962458	1158.377605	858.1128183
7.47_193.0354m/z	193.0354214	7.473983333	neg	3-Hydroxyglutaric acid	5417	Organic acids and derivatives	Hydroxy acids and derivatives	Beta hydroxy acids and derivatives	*		38.8	0	M+FA-H	C5H8O5	0.304964301	1724.23324	1770.135307	2014.846188	2377.122844	2728.026766	2460.927531	1470.600317	1607.421527	1626.503888
7.61_183.1391m/z	183.1391112	7.6091	neg	7-Methyl-3-methylene-1,6-octadiene	97899	Lipids and lipid-like molecules	Prenol lipids	Monoterpenoids	*	C06074	38.8	0	M+FA-H	C10H18	0.418333155	522.6504837	580.6798692	546.6581625	845.6131136	684.714482	806.9849797	513.7849125	492.3719916	475.5461177
7.73_485.2377m/z	485.2376874	7.729433333	neg	Glutaminyl-Proline	85719	Organic acids and derivatives	Carboxylic acids and derivatives	Amino acids, peptides, and analogues	*		38.8	0	2M-H	C10H17N3O4	2.368600796	1638.385939	2148.291471	1771.297476	1906.56251	2242.311593	2152.079929	1186.180219	2253.918204	1925.23333
7.77_297.1709m/z	297.1708798	7.770583333	neg	Neryl glucoside	86207	Lipids and lipid-like molecules	Prenol lipids	Terpene glycosides	*		38.8	0	M-H2O-H	C16H28O6	0.418552767	468.369782	373.4132251	483.9848664	968.5003779	1183.801408	515.0551798	502.1773678	608.2024946	509.5749863
7.78_311.1869m/z	311.1868534	7.7828	neg	4-Hydroxy-3-methoxy-2,10-bisaboladien-9-one	HMDB0033917	Lipids and lipid-like molecules	Prenol lipids	Sesquiterpenoids	*		38.8	0	M+FA-H	C16H26O3	1.712575107	1407.509738	1323.735866	1535.702329	4347.419319	3710.43852	3838.849899	812.0525996	625.9165685	956.5000414
7.94_285.1709m/z	285.1708551	7.9373	neg	(1S,2S)-3-oxo-2-pentyl-cyclopentanebutanoic acid	74961	Lipids and lipid-like molecules	Fatty Acyls	Octadecanoids	*		38.8	0	M+FA-H	C14H24O3	0.447907494	569.2330469	464.9627745	447.6523359	786.4484466	861.8739133	959.3717333	548.1618391	564.8777079	522.2387669
8.07_609.2274m/z	609.2273599	8.074883333	neg	Aerobactin	HMDB0004051	Organic acids and derivatives	Carboxylic acids and derivatives	Amino acids, peptides, and analogues	**	C05554	38.8	0	M+FA-H	C22H36N4O13	2.250533329	37799.81589	44943.03717	39736.14323	7304.38261	6803.981538	6571.888182	19503.04778	23808.20742	21360.52022
8.09_593.1622m/z	593.1622309	8.087016667	neg	Hexahydro-6,7-dihydroxy-5-(hydroxymethyl)-3-(2-hydroxyphenyl)-2H-pyrano[2,3	HMDB0037297	Benzenoids	Phenols	1-hydroxy-4-unsubstituted benzenoids			38.8	0	2M-H	C13H15NO7	-0.330405179	4447.433974	5506.316019	4401.929238	1647.228102	1265.874349	1737.183433	2668.75184	3264.453021	3233.931534
8.20_419.2291m/z	419.229106	8.200183333	neg	Tsangane L 3-glucoside	95286	Lipids and lipid-like molecules	Prenol lipids	Terpene glycosides	*		38.8	0	M+FA-H	C19H34O7	1.201619856	8393.971346	7019.688206	7824.076799	9026.987978	7617.804074	8718.342181	9023.705089	10098.24473	9085.87248
8.38_241.1448m/z	241.1447527	8.383816667	neg	α-Terpinyl acetate	41143	Lipids and lipid-like molecules	Prenol lipids	Monoterpenoids	*	C12300	38.8	0	M+FA-H	C12H20O2	1.121387417	388.1184346	359.9825748	261.4747358	603.3682417	379.7548257	394.2832799	196.0975631	252.8031936	307.921567
8.39_253.1446m/z	253.1446357	8.393383333	neg	Carvyl propionate	92819	Lipids and lipid-like molecules	Prenol lipids	Monoterpenoids	*		38.8	0	M+FA-H	C13H20O2	0.494798729	633.7190144	651.547899	652.5082099	983.6947352	920.9436616	1057.74562	561.5921448	569.8785158	802.7624989
8.48_241.1447m/z	241.1447059	8.484433333	neg	cis-3-Hexenyl trans-4-hexenoate	HMDB0031692	Lipids and lipid-like molecules	Fatty Acyls	Fatty acid esters	*		38.8	0	M+FA-H	C12H20O2	0.882858085	2287.663211	2541.558422	2371.861894	3542.627982	3628.280781	3633.808793	1726.192685	1835.305188	1819.610472
8.80_223.1342m/z	223.1341597	8.803433333	neg	3-Methyl-1-phenyl-3-pentanol	87902	Benzenoids	Benzene and substituted derivatives	Unclassified	*		38.8	0	M+FA-H	C12H18O	1.07604574	1735.313342	1798.835439	1833.363384	1675.291432	2094.859079	1927.200834	2100.908952	1978.203122	2276.6804
8.85_171.0201m/z	171.0200867	8.851966667	neg	4-Nitroquinoline-1-oxide	24073	Unclassified	Unclassified	Unclassified			38.8	0	M-H2O-H	C9H6N2O3	0.451450029	792.5185555	853.979476	850.0932625	962.4799733	762.7127775	1003.993953	1004.598851	752.0755438	995.1914175
9.02_405.1557m/z	405.1556749	9.01635	neg	Quassinol	92200	Lipids and lipid-like molecules	Prenol lipids	Terpene lactones	*		38.8	0	M+FA-H	C20H24O6	0.509610181	6156.020462	6525.517974	6421.106314	9211.72248	8499.991708	9359.463718	12430.79921	12791.24451	13703.9029
9.06_471.2598m/z	471.2598056	9.056483333	neg	Prostaglandin PGE2 glyceryl ester	HMDB0013045	Lipids and lipid-like molecules	Fatty Acyls	Eicosanoids	*		38.8	0	M+FA-H	C23H38O7	-0.353940118	10252.89041	11694.88705	11568.68975	16597.40368	16348.91574	16177.31979	7155.596843	6601.444337	8015.043255
9.16_413.2184m/z	413.218392	9.155683333	neg	19(R)-hydroxy-PGE2	3819	Lipids and lipid-like molecules	Fatty Acyls	Eicosanoids	*		38.8	0	M+FA-H	C20H32O6	0.815703363	13049.4746	14385.10504	14539.44583	6918.452068	7514.152237	7113.222671	10089.39892	11235.07901	9450.939006
9.63_224.1657m/z	224.1656671	9.633466667	neg	Rimantadine	HMDB0014621	Organic nitrogen compounds	Organonitrogen compounds	Amines		C07236	38.8	0	M+FA-H	C12H21N	0.360439432	595.2142613	988.4542734	361.0372657	2900.155852	0.001403199	57.87320953	86.9078918	651.9973615	80.13593514
9.91_194.0823m/z	194.0823421	9.906316667	neg	Venoterpine	HMDB0030175	Organoheterocyclic compounds	Pyridines and derivatives	Unclassified	*		38.8	0	M+FA-H	C9H11NO	0.504800071	1173.831509	1054.443864	1123.85464	993.8741893	1072.355739	1054.550976	1114.81972	978.3764791	1124.076614
0.69_154.0623m/z	154.0622651	0.692166667	neg	Brunfelsamidine	68140	Unclassified	Unclassified	Unclassified			38.7	0	M+FA-H	C5H7N3	0.59563511	44127.02896	45756.48264	49739.26543	67998.47463	71404.02468	67522.1065	68964.20685	70412.89916	74212.87395
0.71_765.3105m/z	765.3105115	0.714216667	neg	Niazimin A	93291	Organic oxygen compounds	Organooxygen compounds	Carbohydrates and carbohydrate conjugates			38.7	0	2M-H	C18H25NO8	2.28934688	2575.796067	1874.937464	2652.234286	3396.44307	4586.033403	1968.671654	3124.298706	1422.393799	1014.573867
0.74_289.1158m/z	289.1157725	0.7359	neg	GlcNAcβ-Sp	3605	Unclassified	Unclassified	Unclassified			38.7	0	M-H	C10H18N4O6	1.429020848	1581.185771	1388.969895	1378.473974	1038.013201	1202.18047	1065.17765	873.8988066	714.9510002	996.2547201
0.74_322.1138m/z	322.1137744	0.7359	neg	N-(1-Deoxy-1-fructosyl)proline	HMDB0038493	Organoheterocyclic compounds	Benzodioxanes	Phenylbenzodioxanes			38.7	0	M+FA-H	C11H19NO7	-2.09435784	79.08425791	0.001403199	475.1138617	0.001403199	931.7470908	185.4522453	260.3226021	917.1574821	941.0993417
0.76_148.0617m/z	148.0616606	0.757316667	neg	(S)-beta-Aminoisobutyric acid	HMDB0002166	Organic acids and derivatives	Carboxylic acids and derivatives	Amino acids, peptides, and analogues	**	C03284	38.7	0	M+FA-H	C4H9NO2	1.253520711	531.5059802	489.2352369	361.3667328	670.3893812	463.7916235	571.0684152	629.6281731	397.0477793	610.0123625
0.76_162.0409m/z	162.0409202	0.757316667	neg	Aceturic acid	4230	Organic acids and derivatives	Carboxylic acids and derivatives	Amino acids, peptides, and analogues	*		38.7	0	M+FA-H	C4H7NO3	1.061647825	190.7372906	228.7396091	282.612146	110.6026499	49.7661158	251.8482906	109.7347562	66.94326019	220.854217
0.88_187.1089m/z	187.1089115	0.884633333	neg	Glycylleucine	HMDB0000759	Organic acids and derivatives	Carboxylic acids and derivatives	Amino acids, peptides, and analogues	***	C02155	38.7	0	M-H	C8H16N2O3	0.507823897	2887.916049	3011.416775	2248.821063	2735.836051	2329.42887	3131.105247	2544.13176	3292.654822	2866.680663
0.97_145.0507m/z	145.0506816	0.967616667	neg	Pentane-2,4-dione	LMFA12000075	Organic oxygen compounds	Organooxygen compounds	Carbonyl compounds	*	C15499	38.7	0	M+FA-H	C5H8O2	0.491730521	209.7123019	151.2417197	289.9628806	96.95322769	156.2757948	104.9109363	265.0085226	204.5423363	136.2735309
1.18_128.0354m/z	128.0353831	1.180366667	neg	dimethadione	HMDB0061093	Organoheterocyclic compounds	Azolidines	Oxazolidines	*		38.7	0	M-H	C5H7NO3	0.514828349	42392.76307	43025.62084	45322.12818	41138.05575	41968.25415	42376.41435	41726.33478	44750.70181	44451.46687
1.31_201.0883m/z	201.0883253	1.3134	neg	(S)-2-Azetidinecarboxylic acid	HMDB0029615	Organic acids and derivatives	Carboxylic acids and derivatives	Amino acids, peptides, and analogues	*		38.7	0	2M-H	C4H7NO2	1.211139724	249.187907	345.3524212	317.6798838	368.2362983	354.7205511	270.5661459	400.1701768	347.8540025	303.9827642
1.36_267.0737m/z	267.0736848	1.358266667	neg	Allopurinol riboside	HMDB0000481	Nucleosides, nucleotides, and analogues	Pyrazolo[3,4-d]pyrimidine glycosides	Unclassified	*		38.7	20.2	M-H	C10H12N4O5	0.715036403	5139.209228	4565.664576	4440.889131	4690.581432	4134.931108	4351.689652	3687.079843	3864.55229	3867.319863
1.45_328.9830m/z	328.9829502	1.4461	neg	Deamino-α-keto-demethylphosphinothricin	63599	Unclassified	Unclassified	Unclassified			38.7	0	2M-H	C4H6O5P	-1.040042841	41.81885474	0.001403199	0.001403199	156.077849	138.4105576	47.12340819	742.9455537	206.8972931	370.1044354
1.47_166.0509m/z	166.0509122	1.472133333	neg	Noradrenochrome o-semiquinone	HMDB0013029	Organoheterocyclic compounds	Indoles and derivatives	Indolines	*		38.7	0	M-H	C8H9NO3	-0.326060151	493.9722054	405.059229	383.0103499	312.1931843	228.2319947	343.3401813	456.0037049	345.2802711	438.7370006
1.56_210.0774m/z	210.0773704	1.56225	neg	4-(3-Pyridyl)-butanoic acid	5937	Organoheterocyclic compounds	Pyridines and derivatives	Unclassified	*		38.7	0	M+FA-H	C9H11NO2	1.144535033	771.3509365	523.3036906	551.0344919	636.4863416	480.2351613	573.1452797	559.1368268	814.7681508	633.6723236
1.90_205.0354m/z	205.0354305	1.899016667	neg	Oxoadipic acid	HMDB0000225	Organic acids and derivatives	Keto acids and derivatives	Medium-chain keto acids and derivatives	**	C00322	38.7	0	M+FA-H	C6H8O5	0.338909828	1933.21095	1939.481565	1817.767202	2027.947584	1903.552332	1914.762682	1671.312442	1515.244766	1471.586674
10.29_256.1921m/z	256.1920565	10.29078333	neg	Ethyl menthane carboxamide	92642	Lipids and lipid-like molecules	Prenol lipids	Monoterpenoids	*		38.7	0	M+FA-H	C13H25NO	1.132511192	2712.937098	2860.367464	2911.199981	1902.731472	2911.492831	2340.313323	1933.133664	2484.471335	2450.806287
15.06_166.0442m/z	166.0441559	15.0628	neg	Zapotidine	HMDB0030383	Organoheterocyclic compounds	Diazines	Pyrimidines and pyrimidine derivatives	*		38.7	0	M-H	C7H9N3S	-1.711576495	129.5825111	4266.163032	91.39494838	1498.852958	157.5254038	124.5710982	80.21251186	31.62554151	61.21259461
2.07_127.0401m/z	127.0400969	2.070366667	neg	Monomethyl glutaric acid	HMDB0000858	Lipids and lipid-like molecules	Fatty Acyls	Fatty acid esters	*		38.7	0	M-H2O-H	C6H10O4	0.200246261	498.6702844	593.1787605	575.9768863	669.1212698	721.7897275	708.9051569	607.3980818	543.5926576	458.6095677
2.32_198.0409m/z	198.0409423	2.322933333	neg	Mesalazine	HMDB0014389	Benzenoids	Benzene and substituted derivatives	Benzoic acids and derivatives	*		38.7	0	M+FA-H	C7H7NO3	0.956363261	1012.696999	966.7092362	966.5139735	542.5986053	474.6897907	559.9332759	257.2540566	349.0134724	318.6126281
2.35_317.0516m/z	317.0515582	2.34905	neg	Fukiic acid	86306	Phenylpropanoids and polyketides	Phenylpropanoic acids	Unclassified	*		38.7	0	M+FA-H	C11H12O8	0.507283097	3614.362945	3626.823597	3217.718037	1177.878781	1442.850318	1254.910088	1423.371616	2250.044896	2208.285583
2.53_343.1037m/z	343.1036614	2.531716667	neg	4-Ethylphenol glucuronide	HMDB0240654	Organic oxygen compounds	Organooxygen compounds	Carbohydrates and carbohydrate conjugates			38.7	0	M+FA-H	C14H18O7	0.689759791	796.653345	766.8574308	881.0526482	2178.100015	2209.10805	2263.959272	1270.447333	1576.399668	1367.332913
2.79_286.0571m/z	286.057064	2.790116667	neg	2,4-Dihydroxy-6,7-dimethoxy-2H-1,4-benzoxazin-3(4H)-one	HMDB0037548	Organoheterocyclic compounds	Benzoxazines	Benzoxazinones			38.7	0	M+FA-H	C10H11NO6	0.929357287	2570.840613	2830.225623	3195.125524	4077.756765	4151.679437	3884.940458	2807.485026	2850.379583	2672.331334
2.83_214.0723m/z	214.0723135	2.826466667	neg	Pyridoxine	HMDB0000239	Organoheterocyclic compounds	Pyridines and derivatives	Pyridoxines	**	C00314	38.7	0	M+FA-H	C8H11NO3	1.285683017	2319.410964	2492.586352	2452.001463	1795.540829	1969.820708	1678.61024	2952.878753	3342.451404	3132.638717
3.12_153.0194m/z	153.0193723	3.123216667	neg	N-methylethanolamine phosphate	HMDB0304429	Unclassified	Unclassified	Unclassified			38.7	0	M-H	C3H9NO4P-	-1.757589606	498.1183744	486.9680085	549.1521799	376.543513	292.9505902	264.825293	359.486897	444.7963829	548.9609536
3.54_202.1087m/z	202.1086681	3.540433333	neg	N,N-diethyl-3-hydroxybut-2-enamide	LMFA08020190	Lipids and lipid-like molecules	Fatty Acyls	Fatty amides			38.7	0	M+FA-H	C8H15NO2	1.187139965	1566.583112	1630.080941	1737.770728	1040.626412	1071.256522	1071.988654	1254.275035	1615.923136	1354.993485
3.58_221.0569m/z	221.0568944	3.5753	neg	Pyrrole-2-carboxylic acid	HMDB0004230	Organoheterocyclic compounds	Pyrroles	Pyrrole carboxylic acids and derivatives	**	C05942	38.7	0	2M-H	C5H5NO2	0.51345531	715.9684264	640.3101737	700.3077612	249.4968283	258.2068439	303.2894828	125.8291565	136.116842	72.61001528
3.64_109.0294m/z	109.0293731	3.640916667	neg	Hexa-2,4-dienedial	HMDB0254943	Organic oxygen compounds	Organooxygen compounds	Carbonyl compounds	*		38.7	0	M-H	C6H6O2	-1.180691545	747.7978644	814.0141015	781.2270174	306.9032936	313.8669915	264.9410939	574.8272777	597.229734	647.4343572
3.64_219.0875m/z	219.0875344	3.640916667	neg	Dimethyl adipate	70165	Lipids and lipid-like molecules	Fatty Acyls	Fatty acid esters	*	C14570	38.7	0	M+FA-H	C8H14O4	0.704158591	1141.296295	1264.989739	1136.344386	1131.432673	1125.129604	1159.053333	752.7291593	648.1372948	903.6634084
3.73_252.9790m/z	252.9790176	3.730516667	neg	Zoledronate	HMDB0014543	Organic acids and derivatives	Organic phosphonic acids and derivatives	Bisphosphonates			38.7	0	M-H2O-H	C5H10N2O7P2	1.966215968	1635.834707	1677.521058	1447.493094	2886.817578	3148.552109	2838.960658	1284.279307	1487.399929	1394.737862
3.75_205.0177m/z	205.0176789	3.747833333	neg	Thiacremonone	91104	Organic oxygen compounds	Organooxygen compounds	Carbonyl compounds			38.7	0	M+FA-H	C6H8O3S	0.38052156	653.143841	586.0169338	552.530214	1531.996776	1553.679581	1099.952161	2339.388103	2635.03388	2518.952099
3.75_212.0025m/z	212.0024543	3.747833333	neg	7-Hydroxyindole sulfate	HMDB0240659	Organic acids and derivatives	Organic sulfuric acids and derivatives	Arylsulfates			38.7	0	M-H	C8H7NO4S	0.713618953	1982.680628	2035.106444	1986.710551	387.1860197	310.4161327	210.4986994	1839.135076	1817.046234	1869.299565
3.87_284.1254m/z	284.1253918	3.868533333	neg	Eldoral	HMDB0251741	Organoheterocyclic compounds	Benzazepines	Dibenzazepines			38.7	0	M+FA-H	C11H17N3O3	0.826065182	4463.78362	4767.286797	5253.562264	5773.290005	5523.311302	5149.031752	4776.438258	4982.312968	4619.729779
3.94_319.0580m/z	319.0579672	3.9351	neg	cis,trans-5'-Hydroxythalidomide	HMDB0013870	Organoheterocyclic compounds	Isoindoles and derivatives	Isoindolines	*		38.7	0	M+FA-H	C13H10N2O5	2.893099115	0.001403199	73.29408914	120.6769882	167.2080493	450.0398785	473.0025673	549.0114781	628.3826037	647.8005743
3.96_144.0301m/z	144.0301108	3.957583333	neg	Hymexazol	68844	Unclassified	Unclassified	Unclassified			38.7	0	M+FA-H	C4H5NO2	-1.216964207	116.7742322	115.4356363	143.9328385	170.320328	161.1337426	163.8466716	126.1750489	147.1070175	138.7666266
4.02_116.0717m/z	116.0717498	4.024283333	neg	5-amino-pentanoic acid	LMFA01100040	Lipids and lipid-like molecules	Fatty Acyls	Fatty Acids and Conjugates	**	C00431	38.7	0	M-H	C5H11NO2	0.407192208	201.9746794	226.4515639	236.437331	168.0147008	280.8695397	292.6040801	223.6828142	282.6543245	278.6841504
4.05_327.1087m/z	327.1086891	4.046666667	neg	3-carboxy-4-methyl-5-(4-oxopentyl)-2-furanpropanoic acid	LMFA01150067	Lipids and lipid-like molecules	Fatty Acyls	Fatty Acids and Conjugates			38.7	0	M+FA-H	C14H18O6	0.524442533	2513.109131	2737.8432	2914.827141	3273.742666	2862.183635	2825.317854	2973.634435	3360.060793	2900.774112
4.07_328.1516m/z	328.1515734	4.069216667	neg	Valclavam	66481	Unclassified	Unclassified	Unclassified			38.7	0	M-H	C14H23N3O6	0.499215326	4794.301144	5320.853368	5593.939943	7601.809544	7004.584621	7290.483437	4469.967572	5615.469792	5126.343431
4.09_216.0879m/z	216.0878513	4.091783333	neg	N-butanoyl-homoserine lactone	36732	Lipids and lipid-like molecules	Fatty Acyls	Fatty amides		C11837	38.7	0	M+FA-H	C8H13NO3	0.614341601	277.2176543	295.1155939	259.0778421	539.0732736	452.6546315	508.0368133	502.8201099	371.7129967	455.8629222
4.09_317.1121m/z	317.1120902	4.091783333	neg	fluvoxamino acid	HMDB0060950	Benzenoids	Benzene and substituted derivatives	Trifluoromethylbenzenes	*		38.7	0	M-H	C14H17F3N2O3	0.753035298	516.8448947	353.7575146	586.1233191	811.1563959	805.7358776	815.2276054	486.2891419	523.1367769	679.6921813
4.09_386.1095m/z	386.10945	4.091783333	neg	Diethylpropion(metabolite VIII-glucuronide)	2023	Unclassified	Unclassified	Unclassified			38.7	0	M+FA-H	C15H19NO8	0.529462102	3253.805753	3646.831954	3809.143267	1199.99297	1009.424547	1181.684405	775.5898843	1065.784401	1021.055867
4.11_519.0588m/z	519.0588225	4.113883333	neg	Demethylbellidifolin	68100	Unclassified	Unclassified	Unclassified			38.7	0	2M-H	C13H8O6	3.69766339	890.0654813	870.6865718	707.2233002	965.3998714	1083.039121	926.1866626	817.9189349	1045.898003	1175.683006
4.16_475.9778m/z	475.9778839	4.15895	neg	Dihydroneopterin triphosphate	HMDB0000980	Organoheterocyclic compounds	Pteridines and derivatives	Pterins and derivatives	**	C04895	38.7	0	M-H2O-H	C9H16N5O13P3	-0.041239781	1222.065297	1401.899782	1179.525354	1364.389756	1456.736467	1279.287488	1796.336681	1290.123375	1419.441556
4.18_393.0806m/z	393.0806146	4.181333333	neg	2-Amino-4,6-dinitrotoluene	HMDB0245219	Unclassified	Unclassified	Unclassified			38.7	0	2M-H	C7H7N3O4	1.470779808	8437.633652	7840.136809	7901.399979	8085.949366	6182.818474	7639.708795	6871.627173	5365.29295	5138.931701
4.30_244.1555m/z	244.1555454	4.300033333	neg	Valerylcarnitine	LMFA07070111	Lipids and lipid-like molecules	Fatty Acyls	Fatty acid esters	*		38.7	0	M-H	C12H23NO4	0.463413087	1262.836323	1354.525192	1501.178025	1354.844717	1347.930123	1493.89256	1354.933998	1555.438523	1474.05333
4.30_463.0887m/z	463.0886777	4.300033333	neg	5'-Butyrylphosphoinosine	63405	Unclassified	Unclassified	Unclassified			38.7	0	M+FA-H	C14H19N4O9P	3.611769358	1199.432942	1534.171064	1641.372903	2286.209653	1958.363914	2039.168848	1009.371791	1138.803208	1035.125757
4.34_363.1201m/z	363.1201373	4.344733333	neg	2-iodo-hexadecanoic acid	LMFA01090037	Lipids and lipid-like molecules	Fatty Acyls	Fatty Acids and Conjugates			38.7	0	M-H2O-H	C16H31IO2	2.879054008	2141.467481	2557.610925	2687.493864	3211.457819	2808.021339	2914.823066	2167.008639	2692.102726	2374.146934
4.34_535.1824m/z	535.1824453	4.344733333	neg	diethyl 2,6-dimethyl-4-oxo-4h-pyran-3,5-dicarboxylate	96355	Unclassified	Unclassified	Unclassified			38.7	0	2M-H	C13H16O6	0.643853564	2559.038532	2010.77642	2557.530664	2295.311711	1991.745871	1901.802092	1396.205353	1534.199142	1375.419551
4.37_351.0577m/z	351.0576774	4.367166667	neg	Ascorbic acid	HMDB0000044	Organoheterocyclic compounds	Dihydrofurans	Furanones	***	C01041	38.7	0	2M-H	C6H8O6	2.209460411	3054.440263	3233.627398	3262.134668	3910.39539	3780.781514	3617.083997	3774.885056	4257.771476	3767.07495
4.43_325.2135m/z	325.2134685	4.432683333	neg	Phygrine	HMDB0039754	Alkaloids and derivatives	Unclassified	Unclassified			38.7	0	M+FA-H	C16H28N2O2	0.669068144	3273.149422	3728.833128	4395.462066	2426.138333	2187.730794	2300.456848	2557.111032	3104.409986	2785.857321
4.50_203.0022m/z	203.0021665	4.49925	neg	3-Methylcatechol 2-sulfate	HMDB0240663	Organic acids and derivatives	Organic sulfuric acids and derivatives	Arylsulfates			38.7	0	M-H	C7H8O5S	0.973591145	1004.74103	1077.789422	1120.782188	265.3462986	298.4329667	261.6966396	3358.77086	3866.757635	3759.879499
4.54_293.0622m/z	293.0622354	4.54425	neg	Aspartyl-Aspartate	85664	Organic acids and derivatives	Carboxylic acids and derivatives	Amino acids, peptides, and analogues	*		38.7	0	M-H	C8H12N2O7	-1.686057751	441.5782909	409.6658991	457.9417698	551.0295928	588.2410556	572.83045	420.682673	507.2927619	485.4641724
4.57_220.0616m/z	220.0616447	4.5668	neg	N-Acetylindoxyl	63544	Unclassified	Unclassified	Unclassified			38.7	0	M+FA-H	C10H9NO2	0.647212965	672.1802044	668.0579023	619.1618468	24.68722084	29.30748642	58.66451885	400.9764152	435.9660644	442.1782078
4.57_507.1099m/z	507.1099216	4.5668	neg	Daidzein	HMDB0003312	Phenylpropanoids and polyketides	Isoflavonoids	Isoflav-2-enes	***	C10208	38.7	0	2M-H	C15H10O4	2.716860719	1922.615841	1936.92213	1713.736056	615.1875743	866.0834999	573.8328572	1609.318345	1872.622196	1844.222072
4.59_153.0559m/z	153.0558694	4.589283333	neg	o-Cresol	HMDB0002055	Benzenoids	Phenols	Cresols	**	C01542	38.7	0	M+FA-H	C7H8O	1.403058836	234.9330976	354.4328311	290.6245046	277.9875159	334.1309336	350.8123892	293.460011	256.2985833	308.3598371
4.68_499.1825m/z	499.1825422	4.682483333	neg	N-Acetylkynurenine	HMDB0240342	Organic oxygen compounds	Organooxygen compounds	Carbonyl compounds			38.7	0	2M-H	C12H14N2O4	-1.789733568	3571.69088	3207.984426	3771.629145	1989.272415	1943.760288	1868.138833	4041.459999	3599.353828	3527.07449
4.77_183.1027m/z	183.1026774	4.769883333	neg	(2Z,4E)-nona-2,4,-dienal	87862	Unclassified	Unclassified	Unclassified			38.7	0	M+FA-H	C9H14O	0.068264107	1266.126226	1249.103438	1454.389739	1488.807855	1507.610266	1354.252014	1380.392413	1498.133454	1433.507908
4.81_236.0930m/z	236.092961	4.814166667	neg	5-Methoxytryptophol	58062	Organoheterocyclic compounds	Indoles and derivatives	Indoles	*		38.7	0	M+FA-H	C11H13NO2	0.677560545	732.3917732	778.7498862	880.6065115	365.8871521	569.1197874	594.3541414	871.7625409	1065.542125	719.6306812
4.86_314.1150m/z	314.1149775	4.858383333	neg	Disperse Yellow 3	73115	Unclassified	Unclassified	Unclassified			38.7	0	M+FA-H	C15H15N3O2	1.292760484	1096.871301	1304.279313	1321.793445	1144.18747	1280.397367	1427.954495	1302.626954	1144.121225	1259.012774
4.88_345.1195m/z	345.1194889	4.880083333	neg	Salidroside	HMDB0257463	Unclassified	Unclassified	Unclassified			38.7	0	M+FA-H	C14H20O7	1.276335071	816.1476199	789.2208748	888.8801302	977.7985576	1024.893064	821.2557395	665.6671474	795.3792081	454.3929698
4.95_244.1193m/z	244.1192862	4.94705	neg	Ecgonine methyl ester	64430	Alkaloids and derivatives	Tropane alkaloids	Unclassified	**	C12448	38.7	0	M+FA-H	C10H17NO3	1.204755824	925.8450743	1119.805097	1089.955959	1160.572112	1128.925519	1211.428611	1328.813251	1369.316201	1144.472038
5.08_183.1027m/z	183.1026699	5.080566667	neg	2,4-Nonadienal	HMDB0031685	Organic oxygen compounds	Organooxygen compounds	Carbonyl compounds			38.7	0	M+FA-H	C9H14O	0.014311053	2165.582519	2409.716381	1975.98559	1932.966768	1820.741408	2164.660466	2257.461488	1961.933186	1891.624854
5.08_387.2028m/z	387.2028326	5.080566667	neg	2,3-Dinor-6-keto-prostaglandin F1 a	HMDB0002277	Lipids and lipid-like molecules	Fatty Acyls	Eicosanoids	*		38.7	0	M+FA-H	C18H30O6	1.142769608	4707.715454	4688.808201	4663.149052	2272.428699	1804.32108	2069.282104	4489.210031	3902.031156	4815.10812
5.08_405.0758m/z	405.0758208	5.080566667	neg	4-AMINOETHYLBENZENESULFONYL FLUORIDE	43987	Unclassified	Unclassified	Unclassified			38.7	0	2M-H	C8H10FNO2S	-0.389993531	1826.262284	1865.974521	1829.52216	2027.913486	1594.872613	2193.582251	1742.329051	1513.249735	1702.469387
5.08_455.1910m/z	455.1909795	5.080566667	neg	Propyl propane thiosulfonate	HMDB0032496	Organic acids and derivatives	Carboxylic acids and derivatives	Dicarboxylic acids and derivatives	*		38.7	0	2M-H	C11H16O5	-2.830565128	1654.847002	1636.007126	1635.327974	581.6739906	851.3652182	730.8275976	1415.758366	1430.603684	1604.452346
5.13_159.1027m/z	159.1026797	5.132616667	neg	2,4-Dimethyl-3-pentanone	HMDB0031429	Organic oxygen compounds	Organooxygen compounds	Carbonyl compounds	*		38.7	0	M+FA-H	C7H14O	0.103140067	678.9945504	753.6666319	563.5153586	764.3064192	624.0655046	697.4248715	859.6790311	609.1227697	913.4288518
5.13_269.1397m/z	269.139685	5.132616667	neg	Annuionone A	88618	Organoheterocyclic compounds	Oxepanes	Unclassified	*		38.7	0	M+FA-H	C13H20O3	1.05998456	2751.556673	3519.172576	1835.134582	2698.77593	1924.476482	1825.681782	2160.45512	1436.21208	2271.191711
5.13_403.1228m/z	403.1227954	5.132616667	neg	Gardenoside	HMDB0252642	Unclassified	Unclassified	Unclassified			38.7	4.17	M-H	C17H24O11	-4.428755297	8712.15362	8738.882556	7228.595623	10863.36229	9723.652601	10038.44856	7294.851769	7146.911218	9551.921795
5.13_539.0974m/z	539.0973852	5.132616667	neg	Apigenin	HMDB0002124	Phenylpropanoids and polyketides	Flavonoids	Flavones	***	C01477	38.7	0	2M-H	C15H10O5	-1.824164578	3172.494542	2628.869991	2150.678961	3606.451232	2986.045635	3933.455619	2776.788185	2542.158284	2636.257882
5.13_567.1294m/z	567.1294396	5.125666667	neg	Biochanin A	HMDB0002338	Phenylpropanoids and polyketides	Isoflavonoids	O-methylated isoflavonoids	***	C00814	38.7	0	2M-H	C16H12O5	-0.406494702	2040.763003	1642.131967	1453.026294	1822.620654	1364.491119	2301.228405	2238.628075	1648.992252	2299.525074
5.18_286.1096m/z	286.1096447	5.1777	neg	Zaleplon	HMDB0015097	Organoheterocyclic compounds	Diazines	Pyrimidines and pyrimidine derivatives		C07484	38.7	0	M-H2O-H	C17H15N5O	-0.571234973	893.0577828	987.2170128	978.5266674	1546.79228	1567.781569	1737.648952	937.2560067	878.006286	767.7411698
5.30_286.1299m/z	286.1298618	5.302883333	neg	1,2-Propanediol, 3-[[2-(2-methoxyphenoxy)ethyl]amino]	1573	Unclassified	Unclassified	Unclassified			38.7	0	M+FA-H	C12H19NO4	1.040067512	2157.967068	1823.067392	2262.527771	1112.151997	1578.147388	1439.619768	1800.910806	1674.722399	2227.264231
5.45_567.2040m/z	567.2039511	5.451166667	neg	Cimetidine sulfoxide	1757	Unclassified	Unclassified	Unclassified			38.7	0	2M-H	C10H16N6O2S	0.242820727	976.8692427	1215.325817	1111.717287	725.397594	669.9970445	634.493615	2499.142568	1876.627781	2103.448306
5.52_269.1397m/z	269.1397212	5.516816667	neg	Epoxyeremopetasinorol	HMDB0029670	Organoheterocyclic compounds	Oxanes	Unclassified	*		38.7	0	M+FA-H	C13H20O3	1.221565688	10057.30081	10212.29117	10240.02154	10829.01937	12747.08836	12426.9262	9966.213115	9350.46524	9390.497016
5.55_453.1384m/z	453.1383742	5.547916667	neg	Wybutoxine	93724	Organic acids and derivatives	Carboxylic acids and derivatives	Amino acids, peptides, and analogues			38.7	0	M+FA-H	C16H20N6O7	2.019794763	18037.41501	17097.30971	19320.51746	21105.75065	25053.69397	20640.58817	17910.09416	16974.11347	16673.10589
5.60_199.1341m/z	199.1340954	5.602	neg	eucalyptol	4020	Organoheterocyclic compounds	Oxanes	Unclassified	**	C09844	38.7	0	M+FA-H	C10H18O	0.825960611	1288.09317	1068.201728	1129.746383	2217.191191	2427.233225	2404.884116	1236.811721	1109.273577	963.2530595

5.65_225.1134m/z	225.1133726	5.650833333	neg	formyl 2E,4Z,6Z-decatrienoate	97204	Unclassified	Unclassified	Unclassified			38.7	0	M+FA-H	C11H16O2	0.777336475	3436.657396	2987.226316	3251.627737	4219.311359	3997.049371	3793.959161	3343.302939	2772.251717	3276.920666
5.67_537.2347m/z	537.2346784	5.666566667	neg	rac-5,6-Epoxy-retinoyl-beta-D-glucuronide	HMDB0060123	Lipids and lipid-like molecules	Prenol lipids	Terpene glycosides	*		38.7	0	M+FA-H	C26H36O9	1.102706172	11470.98506	11250.9891	13135.59851	9557.390842	9915.776694	9549.357281	9758.687083	8889.380165	11714.80647
5.71_387.2026m/z	387.2025766	5.709933333	neg	6-Hydroxypseudooxynicotine	HMDB0240264	Organic oxygen compounds	Organooxygen compounds	Carbonyl compounds	**	C01297	38.7	0	2M-H	C10H14N2O2	-3.097249566	1996.450494	1927.485813	1241.128795	530.0825612	615.9128066	666.9582687	1213.513768	1760.809297	2315.854469
5.73_145.0619m/z	145.0618738	5.727283333	neg	Isoglutamine	2819	Unclassified	Unclassified	Unclassified			38.7	0	M-H	C5H10N2O3	0.054820129	17.07007289	0.001403199	19.01049058	85.18589984	138.93206	80.91861543	21.35905151	40.43429953	24.23687799
5.73_229.1082m/z	229.1081814	5.727283333	neg	(S)-Oleuropeic acid	HMDB0036998	Lipids and lipid-like molecules	Prenol lipids	Monoterpenoids	*		38.7	0	M+FA-H	C10H16O3	0.185368369	1127.856823	1205.592668	1034.230732	639.2954442	1050.28397	746.6794054	1512.204982	2175.432514	2117.378482
5.76_407.2076m/z	407.2076492	5.7632	neg	11-Epicortisol	HMDB0244354	Lipids and lipid-like molecules	Steroids and steroid derivatives	Hydroxysteroids	**	C00735	38.7	0	M+FA-H	C21H30O5	0.337689675	3153.547834	3944.646076	3340.214586	3762.100712	5446.165992	4526.513739	5391.839546	5396.329093	4416.04777
5.83_347.0258m/z	347.0257979	5.825	neg	2,5-Dimethyl-3-(methyldithio)furan	HMDB0040200	Organoheterocyclic compounds	Heteroaromatic compounds	Unclassified			38.7	0	2M-H	C7H10OS2	-4.423549103	3136.021429	2395.61948	3085.505899	3587.989779	2708.390086	2605.701405	2063.117292	2290.087389	2039.767805
5.85_459.1514m/z	459.1513871	5.84575	neg	Hydralazine pyruvate hydrazone	HMDB0061143	Organoheterocyclic compounds	Diazanaphthalenes	Benzodiazines	*		38.7	0	2M-H	C11H10N4O2	-4.536665387	2278.594687	2101.447046	2364.595361	4046.666915	3901.080137	3935.153643	4147.345601	3402.419383	3333.051544
5.87_337.0934m/z	337.0934146	5.866533333	neg	(S)-Angelicain	86735	Organoheterocyclic compounds	Benzopyrans	1-benzopyrans	*		38.7	0	M+FA-H	C15H16O6	1.792097389	3400.823865	4034.854412	4306.648611	2189.806115	1672.790373	1848.241842	6321.969341	5215.124531	6058.260896
5.97_199.1341m/z	199.134083	5.965483333	neg	beta-Geraniol	HMDB0035155	Lipids and lipid-like molecules	Prenol lipids	Monoterpenoids	**	C01500	38.7	0	M+FA-H	C10H18O	0.745551668	2579.586815	2879.060057	2934.251047	959.3732775	1130.136465	1171.015869	729.4947268	829.0790299	725.8641745
6.03_354.1020m/z	354.1019823	6.028183333	neg	Ethacrynic acid M1	2585	Unclassified	Unclassified	Unclassified			38.7	0	M-H	C16H21NO6S	0.845633451	31497.82018	32465.0027	36952.49507	14520.50074	14898.92697	13501.13769	21923.61399	21417.59877	22671.39374
6.06_139.1129m/z	139.112912	6.056116667	neg	xi-2-Ethylheptanoic acid	HMDB0030331	Lipids and lipid-like molecules	Fatty Acyls	Fatty acids and conjugates	*		38.7	0	M-H2O-H	C9H18O2	0.463740425	890.3935297	986.3240085	993.7539057	728.140958	782.8742669	716.0274358	795.6626092	817.5333445	745.7633803
6.07_591.2428m/z	591.242763	6.07	neg	6,6'-(1,2-phenylene)bis(1,3,5-triazine-2,4-diamine)	96304	Unclassified	Unclassified	Unclassified			38.7	0	2M-H	C12H12N10	1.280812498	1105.40301	1542.426824	1331.035391	1892.584417	2718.094283	1842.417694	1074.860923	1367.330856	1143.487993
6.10_159.1027m/z	159.1027393	6.101316667	neg	(Z)-4-Hepten-1-ol	HMDB0031405	Lipids and lipid-like molecules	Fatty Acyls	Fatty alcohols	*		38.7	0	M+FA-H	C7H14O	0.625768587	968.1832589	974.6816041	915.4771135	954.8384375	1074.913083	958.3120356	1064.303196	984.1925559	1021.471226
6.10_171.1028m/z	171.1027877	6.101316667	neg	2,4,4-Trimethylcyclopentanone	HMDB0031197	Organic oxygen compounds	Organooxygen compounds	Carbonyl compounds	*		38.7	0	M+FA-H	C8H14O	0.949397073	4382.178327	4178.980294	4198.542199	3707.788195	4096.192465	4323.436466	4676.514456	3865.372801	4155.268751
6.13_255.1237m/z	255.1237239	6.133983333	neg	(±)7-epi Jasmonic Acid	36071	Unclassified	Unclassified	Unclassified			38.7	0	M+FA-H	C12H18O3	-0.349479506	576.8369256	879.9570484	717.9709567	596.2981207	581.4540426	481.4473226	494.337403	394.8250898	490.8653535
6.23_267.1240m/z	267.1239998	6.230633333	neg	1-Hydroxyibuprofen	HMDB0060565	Phenylpropanoids and polyketides	Phenylpropanoic acids	Unclassified	*		38.7	0	M+FA-H	C13H18O3	0.911748515	1575.9069	1160.371843	1535.842355	3046.618832	2763.998633	3131.272548	1555.55394	1558.202381	1625.7336
6.23_359.1716m/z	359.1715654	6.230633333	neg	5-Hydroxykynurenamine	58203	Organic oxygen compounds	Organooxygen compounds	Carbonyl compounds	**	C05638	38.7	0	2M-H	C9H12N2O2	-2.536163563	4754.390337	3835.210437	4785.149965	6541.453763	6118.912886	6995.937674	4093.944695	4598.082588	4411.342966
6.25_549.2322m/z	549.2322445	6.248083333	neg	Crocin 4	HMDB0039122	Lipids and lipid-like molecules	Prenol lipids	Diterpenoids	**	C00744	38.7	0.919	M+FA-H	C27H36O9	-3.750503377	78342.15959	60061.78908	77597.27566	85044.96279	70905.62714	84708.88055	72022.65601	75821.111	71095.65587
6.37_288.0883m/z	288.0882779	6.373833333	neg	N-Desmethyltolmetin	2872	Unclassified	Unclassified	Unclassified			38.7	0	M+FA-H	C14H13NO3	2.187473014	5656.505466	5587.496923	7067.246173	8426.839778	9465.067132	8791.464374	9212.497954	11322.30547	10615.68799
6.37_301.1659m/z	301.1658853	6.373833333	neg	(6R,8Z)-6-Hydroxy-3-oxotetradecenoic acid	HMDB0062363	Unclassified	Unclassified	Unclassified			38.7	0	M+FA-H	C14H24O4	0.871314534	850.4823334	786.7182832	880.2398085	1807.804195	1668.598645	1686.925887	825.0281087	787.4861546	739.5869763
6.40_327.1449m/z	327.144863	6.39835	neg	Anguidol	91063	Lipids and lipid-like molecules	Prenol lipids	Sesquiterpenoids	*		38.7	0	M+FA-H	C15H22O5	-0.225784871	4590.594483	5105.529022	5450.471387	2971.445351	2834.831385	2685.772503	4893.791026	5301.430007	5027.630621
6.42_411.2392m/z	411.2392438	6.41995	neg	Tetrahydrocortisol	1914	Lipids and lipid-like molecules	Steroids and steroid derivatives	Bile acids, alcohols and derivatives	**	C05465	38.7	0	M+FA-H	C21H34O5	1.137805044	3962.292362	4044.152807	4181.663085	3546.550729	3147.030502	3729.483532	4169.633015	3766.437507	3740.503754
6.56_503.1927m/z	503.1927357	6.55905	neg	1-(2,4,6-Trimethoxyphenyl)-1,3-butanedione	87129	Organic oxygen compounds	Organooxygen compounds	Carbonyl compounds	*		38.7	0	2M-H	C13H16O5	0.922172386	4335.920716	5061.21681	5599.185896	1473.748236	1528.027582	1541.910877	3381.461787	4038.354372	3562.872386
6.59_145.0507m/z	145.0506808	6.592933333	neg	Isopropenyl acetate	88386	Organic acids and derivatives	Carboxylic acids and derivatives	Carboxylic acid derivatives	*		38.7	0	M+FA-H	C5H8O2	0.48413841	220.3050555	163.9926843	153.4948296	194.9791827	239.7415406	211.0470166	248.3680831	213.7285599	221.624992
6.62_155.1077m/z	155.1077137	6.61635	neg	2,4-Octadiene	HMDB0061900	Hydrocarbons	Unsaturated hydrocarbons	Olefins			38.7	0	M+FA-H	C8H14	-0.359946649	225.449866	224.84476	262.627418	504.9789037	438.6550204	475.1638515	438.3843868	414.9519835	431.2006821
6.65_193.0354m/z	193.0353874	6.653683333	neg	D-Ribonolactone	44751	Organic oxygen compounds	Organooxygen compounds	Carbohydrates and carbohydrate conjugates	*	C02674	38.7	0	M+FA-H	C5H8O5	0.075728911	1948.896126	2128.668013	1940.180202	2068.446567	2212.189819	1992.764606	2151.989	2266.540127	2214.292545
6.65_355.1763m/z	355.1763357	6.653683333	neg	3-Hydroxy-6,8-dimethoxy-7(11)-eremophilen-12,8-olide	95221	Lipids and lipid-like molecules	Prenol lipids	Terpene lactones	*		38.7	0	M+FA-H	C17H26O5	0.350932713	812.5041226	1144.454146	1048.799776	2947.5332	3029.366058	2340.653575	537.8225453	613.4376083	738.4945303
6.93_419.1927m/z	419.1926995	6.926366667	neg	2,6-Dihydroxypseudooxynicotine	63883	Unclassified	Unclassified	Unclassified			38.7	0	2M-H	C10H14N2O3	-2.162552461	6914.324529	7131.987816	6233.18286	11052.38308	10254.41658	9585.056499	4445.3498	5732.687774	5811.869346
6.96_433.1573m/z	433.1573183	6.96195	neg	Alanyl-Glutamate	85602	Organic acids and derivatives	Carboxylic acids and derivatives	Amino acids, peptides, and analogues	*		38.7	0	2M-H	C8H13N2O5-	-0.687102602	3392.562288	3730.783339	3668.230553	6763.633917	5479.552933	7121.263514	2942.868833	3400.019738	3662.846884
6.98_283.1553m/z	283.1552716	6.981183333	neg	Geranyl acetoacetate	92981	Lipids and lipid-like molecules	Fatty Acyls	Fatty alcohol esters	*		38.7	0	M+FA-H	C14H22O3	0.731407651	2100.580333	2153.773874	2136.168574	1796.444006	1627.229498	1861.008899	911.5925407	1091.253084	1142.102775
6.99_222.1137m/z	222.1137332	6.993516667	neg	Phenmetrazine	43306	Organoheterocyclic compounds	Oxazinanes	Morpholines	*	C07432	38.7	0	M+FA-H	C11H15NO	0.938294838	441.2185273	461.8211449	502.3780306	542.3895573	426.3668847	523.1249144	478.5642282	501.6244526	582.180217
7.04_427.1975m/z	427.1974631	7.044716667	neg	12-Oxo-20-trihydroxy-leukotriene B4	HMDB0012553	Lipids and lipid-like molecules	Fatty Acyls	Eicosanoids	*		38.7	0	M+FA-H	C20H30O7	0.279829535	9833.691706	9765.033078	9567.964901	7851.508046	8556.160474	7289.980686	6510.770471	8175.207495	7086.713277
7.08_273.1710m/z	273.1709874	7.076316667	neg	7(S)-methoxy-dodedec-4E-enoic acid	LMFA01080033	Lipids and lipid-like molecules	Fatty Acyls	Fatty Acids and Conjugates			38.7	0	M+FA-H	C13H24O3	1.051204644	2616.413166	2314.256029	2574.691828	2388.677058	2474.697758	2167.538199	1817.175068	1816.597207	1840.767398
7.09_333.1558m/z	333.1558079	7.086816667	neg	1,5-Dibutyl methyl hydroxycitrate	HMDB0040461	Organic acids and derivatives	Carboxylic acids and derivatives	Tricarboxylic acids and derivatives			38.7	0	M-H	C15H26O8	0.947232061	16380.04099	15125.23985	15951.48342	14272.18866	14347.93797	12880.56987	11445.88299	13272.95513	11787.59041
7.09_435.2240m/z	435.2239529	7.086816667	neg	Rehmaionoside A	71697	Unclassified	Unclassified	Unclassified			38.7	0	M+FA-H	C19H34O8	0.978754155	4676.727449	4221.326271	4507.674808	6185.11405	5924.897198	5253.312	6619.875737	6883.013493	6252.8463
7.11_317.0983m/z	317.0982544	7.107783333	neg	4-Methylene-L-glutamate	63273	Organic acids and derivatives	Carboxylic acids and derivatives	Amino acids, peptides, and analogues	*	C00651	38.7	0	2M-H	C6H9NO4	-2.466933384	701.4470065	743.766359	692.7222537	1235.58779	1385.667638	1011.476102	591.4229572	615.1056862	749.562131
7.13_377.1821m/z	377.1821229	7.1264	neg	Unshuoside A	86491	Lipids and lipid-like molecules	Prenol lipids	Terpene glycosides	*		38.7	0	M+FA-H	C16H28O7	1.254632746	5715.863907	5564.648413	5763.615524	10675.62198	12523.87155	9621.104281	4846.702268	5535.847721	5776.155626
7.15_173.1184m/z	173.1183592	7.147366667	neg	Octan-3-one	LMFA12000055	Organic oxygen compounds	Organooxygen compounds	Carbonyl compounds	*	C17145	38.7	0	M+FA-H	C8H16O	0.321376157	1429.518419	1448.413535	1343.127554	1532.439492	1689.775219	1695.434384	1743.653203	1492.635147	1479.744351
7.16_535.1390m/z	535.1389512	7.1647	neg	Formononetin	HMDB0005808	Phenylpropanoids and polyketides	Isoflavonoids	O-methylated isoflavonoids	***	C00858	38.7	0	2M-H	C16H12O4	-1.660197146	2954.48334	3573.395571	3061.301814	3655.788808	3827.118344	4026.054855	3751.758456	4205.606575	4187.205535
7.28_495.2241m/z	495.2240582	7.2779	neg	6-Hydroxymelatonin	7013	Organoheterocyclic compounds	Indoles and derivatives	Hydroxyindoles	**	C05643	38.7	0	2M-H	C13H16N2O3	-1.713121125	2470.399596	2164.263852	3104.970332	2045.618453	1686.879874	1521.526387	2775.85897	2864.046831	2786.192567
7.43_257.1760m/z	257.1760495	7.426916667	neg	Coniferan	HMDB0031741	Organic acids and derivatives	Carboxylic acids and derivatives	Carboxylic acid derivatives	*		38.7	0	M+FA-H	C13H24O2	1.02114008	2430.918438	2316.461415	2509.483435	2795.425848	2639.492271	2471.478106	2138.850456	1964.52282	2021.666416
7.63_463.2338m/z	463.23375	7.631783333	neg	Phyllanthin	68545	Unclassified	Unclassified	Unclassified			38.7	0	M+FA-H	C24H34O6	0.020015273	2558.451938	2940.42938	3038.100457	3063.159196	2399.209291	3054.026485	1996.020393	2420.766044	2241.842132
7.77_411.2027m/z	411.2027205	7.770583333	neg	12-oxo-10,11-dihydro-20-COOH-LTB4	HMDB0012549	Lipids and lipid-like molecules	Fatty Acyls	Fatty acids and conjugates	*		38.7	0	M+FA-H	C20H30O6	0.761623114	16875.47784	18879.68953	18272.33204	8122.915254	7597.418	6793.568431	12853.99415	13314.70463	13646.46996
7.80_413.2184m/z	413.2184319	7.802066667	neg	TXB3	LMFA03030006	Lipids and lipid-like molecules	Fatty Acyls	Eicosanoids	*		38.7	0	M+FA-H	C20H32O6	0.924226487	34257.36557	36061.51395	35376.59304	17345.95169	15807.55751	16177.27375	23661.84085	28787.46876	28798.16763
7.95_250.1450m/z	250.1449946	7.949583333	neg	4-Ethylethcathinone	96612	Unclassified	Unclassified	Unclassified			38.7	0	M+FA-H	C13H19NO	0.62169438	1489.452486	1400.423899	1228.202763	2815.484124	2742.828372	2939.19141	1513.94381	1988.202383	1813.939255
8.05_177.1649m/z	177.1649109	8.053916667	neg	(Z)-4-Dodecenal	LMFA06000256	Lipids and lipid-like molecules	Fatty Acyls	Fatty aldehydes	*		38.7	0	M-H2O-H	C13H24O	0.186713103	458.5983974	604.7469276	559.747572	822.3157987	782.3013895	584.7831256	397.4097983	424.9788207	484.1234982
8.07_204.1362n	407.2710899	8.074883333	neg	3,7-Dimethyl-3-octene-1,2,6,7-tetrol	HMDB0035133	Lipids and lipid-like molecules	Fatty Acyls	Fatty alcohols	*		38.7	0	M-H2O-H, 2M-H	C10H20O4	0.360869098	9239.60087	10476.39971	9897.533499	4367.716984	4143.375107	3833.295231	5336.597586	6993.67914	6009.493953
8.12_305.0822m/z	305.0821918	8.11925	neg	S-6-Hydroxywarfarin	85292	Phenylpropanoids and polyketides	Homoisoflavonoids	Homoisoflavones	*		38.7	0	M-H2O-H	C19H16O5	0.800144712	15362.75446	14602.07466	13177.51833	13957.6754	14592.02819	15389.55819	14674.18852	14216.89248	14027.71878
8.12_333.2286m/z	333.2286109	8.11925	neg	MG(a-13:0/0:0/0:0)[rac]	HMDB0072841	Lipids and lipid-like molecules	Glycerolipids	Monoradylglycerols	*		38.7	0	M+FA-H	C16H32O4	1.209249758	3881.378007	4017.760248	3950.62502	3114.763669	2900.025649	2755.279456	2681.633732	3405.782167	3467.918736
8.33_413.2183m/z	413.2182569	8.3263	neg	6,15-Diketo,13,14-dihydro-PGF1a	HMDB0001979	Lipids and lipid-like molecules	Fatty Acyls	Eicosanoids	*		38.7	0	M+FA-H	C20H32O6	0.448977352	14847.1765	15303.67371	16024.57624	7493.718814	6397.515763	6680.754754	11063.10248	12342.73916	12210.28911
8.50_446.2916m/z	446.2916218	8.503866667	neg	Myxalamid B	69330	Unclassified	Unclassified	Unclassified			38.7	0	M+FA-H	C25H39NO3	1.058620684	10861.81473	11393.00031	12272.92603	770.6315663	478.5215863	923.3962843	5692.331685	6788.95117	6841.307222
8.65_252.1608m/z	252.1607525	8.654333333	neg	1-Phenyl-2-(diethylamino)-1-propanol	2014	Unclassified	Unclassified	Unclassified			38.7	0	M+FA-H	C13H21NO	1.136166483	665.6617263	779.0278731	857.6255792	2075.98266	1510.937583	1592.973117	884.585229	996.9632177	876.900982
8.74_253.1446m/z	253.1445817	8.7445	neg	(5alpha,8beta,9beta)-5,9-Epoxy-3,6-megastigmadien-8-ol	HMDB0034672	Organoheterocyclic compounds	Benzopyrans	Unclassified			38.7	0	M+FA-H	C13H20O2	0.234979732	548.2765162	477.1231733	535.1469109	664.8932147	1003.317118	678.720852	421.969834	431.3983554	552.0970472
8.82_531.1828m/z	531.1827831	8.8245	neg	METHOXYVONE	43575	Unclassified	Unclassified	Unclassified			38.7	0	2M-H	C17H14O3	2.763807278	16262.02718	17106.52922	16881.17625	6583.29755	5679.334359	6761.947544	10023.81127	12449.03713	11788.82811
8.97_335.1868m/z	335.186785	8.97085	neg	(9Z,11E,13E,15Z)-4-Oxo-9,11,13,15-octadecatetraenoic acid	HMDB0031098	Lipids and lipid-like molecules	Fatty Acyls	Lineolic acids and derivatives	*		38.7	0	M+FA-H	C18H26O3	1.335127508	2037.109648	2174.036027	2200.858656	2488.828333	2715.007934	2634.520348	1237.575265	1498.149418	1715.253343
9.02_635.3055m/z	635.3055087	9.01635	neg	Fluvoxamine	2949	Benzenoids	Benzene and substituted derivatives	Trifluoromethylbenzenes		C07571	38.7	0	2M-H	C15H21F3N2O2	2.765902904	1965.207474	2921.261411	2426.891156	0.001403199	91.95780725	104.0752748	2211.415517	1825.191898	1903.04759
9.32_297.1345m/z	297.1345264	9.321783333	neg	QH(2)	96257	Lipids and lipid-like molecules	Prenol lipids	Quinone and hydroquinone lipids	*		38.7	0	M+FA-H	C14H20O4	0.652102421	2018.86031	1976.509853	1968.448808	3920.296643	4599.236774	3999.155621	3126.492244	3619.880244	3000.291993
9.36_267.1240m/z	267.1239578	9.36055	neg	3-Hydroxyibuprofen	HMDB0060921	Phenylpropanoids and polyketides	Phenylpropanoic acids	Unclassified	*		38.7	0	M+FA-H	C13H18O3	0.722666542	1898.944501	1791.529689	1995.629763	3156.8416	2686.449908	2932.097449	2406.530793	3041.05202	2593.428772
9.45_273.1711m/z	273.171054	9.447183333	neg	7-methoxy-dodec-4-enoic acid	96773	Lipids and lipid-like molecules	Fatty Acyls	Fatty Acids and Conjugates			38.7	0	M+FA-H	C13H24O3	1.343492787	629.9591936	732.1991501	713.4427791	1730.740628	1637.806853	1612.675884	541.1004009	541.6829533	463.984915
9.46_327.1815m/z	327.1814854	9.455966667	neg	Lactapiperanol C	89430	Lipids and lipid-like molecules	Prenol lipids	Diterpenoids	*		38.7	0	M+FA-H	C16H26O4	0.613897186	2230.776881	2385.236336	2603.069707	2746.25805	2419.981566	2317.975603	1866.792311	2042.353748	1709.744862
9.76_229.1809m/z	229.1809038	9.7627	neg	2-Dodecanone	HMDB0031019	Organic oxygen compounds	Organooxygen compounds	Carbonyl compounds	*	C14996	38.7	0	M+FA-H	C12H24O	-0.078582647	776.8508036	736.4288488	658.6588203	917.0137001	710.9134781	732.4323705	1005.00877	898.0284157	767.4247079
0.54_150.0169n	149.0096391	0.535283333	neg	Tartaric acid	HMDB0000956	Organic oxygen compounds	Organooxygen compounds	Carbohydrates and carbohydrate conjugates	**	C00898	38.6	0	M-H, 2M-H	C4H6O6	3.183598351	3194.87694	528.4655336	180.4012339	93.68572827	143.4229514	1619.066414	203.5511711	175.7224297	85.13625121
0.67_161.0932m/z	161.0932365	0.6701	neg	5-Aminopentanamide	HMDB0012176	Organic acids and derivatives	Carboxylic acids and derivatives	Amino acids, peptides, and analogues	**	C00990	38.6	0	M+FA-H	C5H12N2O	0.608503614	67.11921174	78.71816115	47.56212154	281.0130538	161.9508145	192.0675949	120.4443713	125.0548134	87.72412603
0.74_207.0448m/z	207.0447971	0.7359	neg	1-Tridecene-3,5,7,9,11-pentayne	HMDB0035400	Hydrocarbons	Unsaturated hydrocarbons	Enynes		C08464	38.6	0	M+FA-H	C13H6	-2.196357121	1122.20942	1547.847867	1477.445493	2052.11062	2086.706008	2157.412789	1252.542706	1457.157348	1319.68716
0.76_219.0623m/z	219.0622914	0.757316667	neg	Serinyl-Aspartate	85928	Organic acids and derivatives	Carboxylic acids and derivatives	Amino acids, peptides, and analogues	*		38.6	0	M-H	C7H12N2O6	0.144280471	1341.836712	1577.168197	1726.467785	1572.576684	906.1041547	1616.751	1279.784497	1522.636805	861.3583462
0.78_219.0455m/z	219.0454921	0.778533333	neg	Cystathionine sulfoxide	HMDB0002399	Organic acids and derivatives	Carboxylic acids and derivatives	Amino acids, peptides, and analogues	*		38.6	6.75	M-H2O-H	C7H14N2O5S	4.161440677	24282.47342	24537.1947	32279.25899	12060.20871	14478.9559	11216.05516	16879.00308	25128.62492	22812.9635
0.78_443.1213m/z	443.1213485	0.778533333	neg	Metoxadiazone	71763	Unclassified	Unclassified	Unclassified			38.6	0	2M-H	C10H10N2O4	1.151342643	5239.816591	4795.368187	2836.13838	4535.851023	9831.351428	6956.573944	3987.591364	5418.110167	6193.398733
0.82_174.0884m/z	174.0883695	0.819566667	neg	(R)-Piperazine-2-carboxamide	73352	Unclassified	Unclassified	Unclassified			38.6	0	M+FA-H	C5H11N3O	-0.351644344	471.3276697	231.1324042	352.5013886	356.2786781	159.6094393	132.3725369	348.840419	272.3154755	354.5201725
0.86_213.0173m/z	213.017296	0.86335	neg	Butanoylphosphate	65782	Unclassified	Unclassified	Unclassified			38.6	0	M+FA-H	C4H9O5P	1.982783627	3443.754318	3085.939749	3481.218483	2372.303317	2526.137866	2457.617363	2766.642037	3061.935478	3363.412491
0.88_110.9758m/z	110.9757713	0.884633333	neg	Monomethyl sulfate	65814	Unclassified	Unclassified	Unclassified			38.6	0	M-H	CH4O4S	0.161444889	333.5200622	373.7441554	438.774713	412.9205772	85.57262419	275.0536788	442.8778777	299.3793611	115.1629169
0.88_163.0613m/z	163.0613023	0.884633333	neg	(2S,3R)-3-Hydroxy-2-methylbutanoic acid	HMDB0000351	Lipids and lipid-like molecules	Fatty Acyls	Fatty acids and conjugates	*		38.6	0	M+FA-H	C5H10O3	0.89164194	4362.593674	4352.331059	4557.858976	3960.436472	3632.067381	3631.60386	3869.144313	3696.978472	3420.579361
0.88_183.0066m/z	183.0066372	0.884633333	neg	3,4-Dihydroxy-2-butanone 4-phosphate	63860	Unclassified	Unclassified	Unclassified			38.6	0	M-H	C4H9O6P	1.298741686	811.0917545	1204.611105	1117.905992	306.7938136	373.2476901	687.4790428	638.0378702	881.6397553	498.7333064
0.88_194.9580m/z	194.9580098	0.884633333	neg	Trifluoromethanesulfonic acid	91649	Organic acids and derivatives	Organic sulfonic acids and derivatives	Organosulfonic acids and derivatives			38.6	0	M+FA-H	CHF3O3S	-0.284357192	4649.928523	4005.696643	4579.661734	2646.28829	2120.315691	2956.522713	3789.809189	2902.347386	2500.540054
0.88_203.0675m/z	203.0674784	0.884633333	neg	4-Methylene-L-glutamine	63274	Unclassified	Unclassified	Unclassified			38.6	0	M+FA-H	C6H10N2O3	0.843521741	3064.731495	3017.459754	3331.465098	5320.88736	5112.585966	5509.269085	3362.371933	3380.473372	3362.711
0.88_277.0737m/z	277.0737288	0.884633333	neg	N-Monomethyl-2-aminoethylphosphonate	63593	Unclassified	Unclassified	Unclassified			38.6	0	2M-H	C3H10NO3P	4.83888728	3460.593729	5551.301554	4434.028948	12007.49834	7238.053096	9953.402653	3493.433195	7695.031959	4943.279771
0.93_175.0725m/z	175.0725072	0.925866667	neg	D-1-Amino-2-pyrrolidinecarboxylic acid	HMDB0030405	Organic acids and derivatives	Carboxylic acids and derivatives	Amino acids, peptides, and analogues	*		38.6	0	M+FA-H	C5H10N2O2	0.589923651	988.2487973	1080.066647	1108.935489	1419.941896	936.8753985	1815.038587	1114.676751	1481.806555	838.246591
1.31_289.0567m/z	289.0567465	1.3134	neg	1-O-Galloylglycerol	93771	Benzenoids	Benzene and substituted derivatives	Benzoic acids and derivatives			38.6	0	M+FA-H	C10H12O7	0.986956469	787.8224946	835.2103052	592.636402	291.2053138	383.1604272	343.6221697	329.5641118	304.3404999	304.1604264
1.34_344.0404m/z	344.0404233	1.335933333	neg	cGMP	3485	Nucleosides, nucleotides, and analogues	Purine nucleotides	Cyclic purine nucleotides	**	C00942	38.6	0	M-H	C10H12N5O7P	0.769066821	2114.826004	2356.22027	2206.330024	1421.124386	1499.968236	1631.863435	2045.813992	2098.827644	1910.859389
1.40_274.1298m/z	274.129821	1.3958	neg	N-(3-hydroxy-heptanoyl)-homoserine lactone	LMFA08030034	Lipids and lipid-like molecules	Fatty Acyls	Fatty amides			38.6	0	M+FA-H	C11H19NO4	0.916840511	2067.388807	1380.612919	1225.113543	1868.875214	1866.371676	1625.847509	1777.5044	1714.946652	1618.299161
1.44_243.0543n	242.0470503	1.43735	neg	Maculine	68520	Unclassified	Unclassified	Unclassified			38.6	0	M-H, M+FA-H	C13H9NO4	4.809320382	2543.056701	2431.984932	1667.712661	4310.063393	3572.332909	3496.329711	1999.456784	1624.608364	1839.029429
1.63_123.0452m/z	123.0452006	1.631516667	neg	Benzene	6285	Benzenoids	Benzene and substituted derivatives	Unclassified	**	C01407	38.6	0	M+FA-H	C6H6	0.609473455	77.70307317	81.79855672	95.92911528	96.43226674	89.56449228	96.82885185	101.7697545	74.10027827	93.76602253
1.91_319.0675m/z	319.0674537	1.911116667	neg	3D-(3,5/4)-Trihydroxycyclohexane-1,2-dione	63287	Unclassified	Unclassified	Unclassified			38.6	0	2M-H	C6H8O5	1.197804109	3374.143226	3865.951227	3284.587383	2301.647011	2621.215854	2392.978944	2367.458847	2528.283093	2426.931229
1.97_233.1032m/z	233.1031698	1.9683	neg	(+/-)-Ethyl 3-acetoxy-2-methylbutyrate	HMDB0032258	Lipids and lipid-like molecules	Fatty Acyls	Fatty acid esters	*		38.6	0	M+FA-H	C9H16O4	0.573923746	1696.296755	1843.943892	1443.46511	1445.160159	1377.494731	1435.583684	1295.689556	1227.535222	1208.761282
10.62_291.1605m/z	291.1604767	10.62453333	neg	5,6-Dimethylbenzimidazole	4161	Organoheterocyclic compounds	Benzimidazoles	Unclassified	**	C03114	38.6	0	2M-H	C9H10N2	-3.571554642	1710.208785	1490.054071	1593.504341	1626.247078	1617.670258	1651.749702	1552.984589	1442.37606	1693.363716
10.69_767.7869m/z	767.7868822	10.6877	neg	erythro-6,8-Pentacosanediol	95508	Lipids and lipid-like molecules	Fatty Acyls	Fatty alcohols	*		38.6	0	2M-H	C25H52O2	0.906374089	2943.997969	3186.792003	2677.544524	3809.150809	3417.813086	3653.290961	3183.394002	3440.346957	3829.934889
2.14_288.0525m/z	288.0525163	2.140766667	neg	Narciclasine	HMDB0255458	Unclassified	Unclassified	Unclassified			38.6	3.93	M-H2O-H	C14H13NO7	3.763508435	13200.42649	15089.4529	14343.74281	8976.969041	8406.585923	8615.365567	10856.69902	11931.94567	13380.29684
2.17_281.0892m/z	281.0891888	2.172	neg	Didanosine	HMDB0015037	Nucleosides, nucleotides, and analogues	Purine nucleosides	Purine 2',3'-dideoxyribonucleosides		C06953	38.6	0	M+FA-H	C10H12N4O3	0.193463001	2575.640222	2208.228363	2274.25235	2684.358745	2618.670428	2558.516752	2195.414727	2819.692395	2765.04068
2.19_207.0776m/z	207.0775959	2.194566667	neg	Norcotinine	6142	Organoheterocyclic compounds	Pyridines and derivatives	Unclassified	*		38.6	0	M+FA-H	C9H10N2O	0.494205184	518.2442934	489.5655556	542.5525136	803.2493158	659.1277091	784.0434658	527.469737	650.9428711	737.7109602
2.67_142.0510m/z	142.0510073	2.6698	neg	Acrylamide-acrylic acid resin	88238	Unclassified	Unclassified	Unclassified			38.6	0	M-H	C6H9NO3	0.283500743	841.8185593	825.0321501	716.5884874	921.5575138	885.1877689	821.4580208	1010.544435	877.8427362	962.2394841
2.72_114.0924m/z	114.0924409	2.72215	neg	Trimethylaminoacetone	62917	Organic oxygen compounds	Organooxygen compounds	Carbonyl compounds	*		38.6	0	M-H	C6H13NO	0.028924647	183.7195008	155.8355578	181.7584041	220.0191892	215.8345091	204.9442367	252.146055	301.7343004	229.631405
2.76_135.0314m/z	135.0313666	2.75705	neg	Allopurinol	HMDB0014581	Organoheterocyclic compounds	Pyrazolopyrimidines	Pyrazolo[3,4-d]pyrimidines			38.6	0	M-H	C5H4N4O	0.972131598	1198.291603	1073.370178	1101.012112	1045.740934	987.6436681	1030.143605	1138.392266	1058.446744	1224.340035
2.81_373.1143m/z	373.1142884	2.8089	neg	Paeonoside	68523	Phenylpropanoids and polyketides	Flavonoids	Flavonoid glycosides			38.6	0	M+FA-H	C15H20O8	0.816560306	3403.094863	3373.368908	3501.589671	1622.664719	1578.212228	1874.240687	4223.423688	4903.0601	4622.771306
3.07_145.0870m/z	145.0870422	3.067683333	neg	Methyl isobutyl ketone	HMDB0002939	Organic oxygen compounds	Organooxygen compounds	Carbonyl compounds	*	C19263	38.6	0	M+FA-H	C6H12O	0.24352551	374.0078696	396.1583363	403.5900388	465.7178522	534.9334505	457.0382468	407.6300557	486.8364613	523.834905
3.19_174.0772m/z	174.0771725	3.186766667	neg	Nipecotic acid	HMDB0255618	Unclassified	Unclassified	Unclassified			38.6	0	M+FA-H	C6H11NO2	-0.069095673	168.640967	227.527431	208.0500635	132.4586536	147.2608196	135.7862847	182.9991757	164.5260821	220.0889115
3.24_338.0616n	337.0543147	3.23635	neg	AICAR	HMDB0001517	Nucleosides, nucleotides, and analogues	Imidazole ribonucleosides and ribonucleotides	1-ribosyl-imidazolecarboxamides	**	C04677	38.6	0	M-H, M+FA-H	C9H15N4O8P	-3.428101393	22493.7921	23089.22901	23118.10767	1095.384549	883.0225365	823.5962928	14344.32671	15682.01379	15451.5498
3.34_145.0619m/z	145.0619212	3.3365	neg	B-ureidoisobutyric acid	304	Unclassified	Unclassified	Unclassified			38.6	0	M-H	C5H10N2O3	0.379725543	43.5290719	36.96136593	62.05313117	170.8125531	151.9651078	129.2337817	82.1058789	127.6311376	18.80462547
3.40_159.0776m/z	159.0775789	3.40385	neg	3-Amino-2-piperidone	5312	Organic acids and derivatives	Carboxylic acids and derivatives	Amino acids, peptides, and analogues	*		38.6	0	M+FA-H	C5H10N2O	0.553076044	394.2863894	469.1955774	453.926743	741.3952233	721.5666998	722.411299	604.3638924	663.2298442	670.1052974
3.42_149.0244m/z	149.0244147	3.417666667	neg	(E)-8-Hydroxy-2-octene-4,6-diynoic acid	87731	Lipids and lipid-like molecules	Fatty Acyls	Fatty acids and conjugates	*		38.6	0	M-H	C8H6O3	-0.019229875	826.4384595	1010.569853	866.8107893	1579.045913	1424.969054	1340.030931	1342.848061	1299.283257	1277.806195
3.47_174.0772m/z	174.0771747	3.47105	neg	DL-pipecolic acid	50	Organic acids and derivatives	Carboxylic acids and derivatives	Amino acids, peptides, and analogues	**	C00408	38.6	0	M+FA-H	C6H11NO2	-0.052677273	398.9246775	549.434396	588.4916921	898.3400204	784.5400305	910.0079105	572.3480437	664.4007711	745.6157604
3.62_171.0122m/z	171.0121981	3.624166667	neg	5-Methyl-2-thiophenecarboxaldehyde	HMDB0032431	Organoheterocyclic compounds	Thiophenes	2,5-disubstituted thiophenes	*		38.6	0	M+FA-H	C6H6OS	0.471288246	0.001403199	0.001403199	112.3759963	180.3179417	0.001403199	64.06417938	315.1865381	0.001403199	0.001403199
3.71_193.0353m/z	193.0353231	3.708366667	neg	3-methylmalate(2-)	HMDB0062739	Lipids and lipid-like molecules	Fatty Acyls	Fatty acids and conjugates	*		38.6	0	M+FA-H	C5H8O5	-0.358799912	635.1979053	558.0382456	710.0663053	724.7638745	687.7480128	335.2859659	672.4007019	400.4335704	624.3332342
3.73_263.1137m/z	263.1137207	3.730516667	neg	Ilicifolinoside A	95608	Lipids and lipid-like molecules	Fatty Acyls	Fatty acyl glycosides	*		38.6	0	M-H	C11H20O7	0.356394389	852.8280393	738.36999	748.8241	1385.103993	1412.932801	872.9528181	423.6509824	569.3200442	469.6559414
3.77_467.1194m/z	467.1194156	3.770416667	neg	2',4',3,4,alpha-Pentahydroxydihydrochalcone 3'-C-xyloside	LMPK12120568	Unclassified	Unclassified	Unclassified			38.6	0	M+FA-H	C20H22O10	-0.199479616	917.5306676	695.0022189	719.4025915	0.001403199	80.28447433	94.75861651	1121.410108	1417.450052	1202.130216
3.80_341.0879m/z	341.0878714	3.801716667	neg	de-Hypoxanthine futalosine	63995	Unclassified	Unclassified	Unclassified			38.6	0	M+FA-H	C14H16O7	0.221922279	268.6048188	399.7449599	414.0002186	195.7448985	187.5227764	322.2108949	371.8383758	485.3203613	462.1002066
3.85_153.0194m/z	153.0193786	3.84605	neg	1,2-Benzoquinone	62804	Organic oxygen compounds	Organooxygen compounds	Carbonyl compounds	*	C02351	38.6	0	M+FA-H	C6H4O2	0.429554105	198.9457897	166.2790732	215.3022831	82.18393636	83.15066989	74.7002381	77.90657261	163.1043311	118.0804789
3.85_161.0819m/z	161.0819354	3.84605	neg	Isopropyl propionate	HMDB0030056	Organic acids and derivatives	Carboxylic acids and derivatives	Carboxylic acid derivatives	*		38.6	0	M+FA-H	C6H12O2	0.025317456	280.2568894	218.4344325	330.8402134	123.0972187	178.8965167	300.8755329	249.0105598	252.8076652	245.0387766
3.85_188.0930m/z	188.0929662	3.84605	neg	L-2-Amino-3-methylenehexanoic acid	86954	Organic acids and derivatives	Carboxylic acids and derivatives	Amino acids, peptides, and analogues	*		38.6	0	M+FA-H	C7H13NO2	0.940908549	655.4903596	704.7475937	752.3870574	1431.773817	1334.478709	1219.50214	628.8771683	785.3548117	818.5358273
3.94_201.9937n	402.9770135	3.9351	neg	4-Sulfobenzoate	65735	Unclassified	Unclassified	Unclassified			38.6	0	M-H, 2M-H	C7H6O5S	0.716935472	539.0639557	632.7882862	615.8305127	1203.344893	1426.192352	1189.784362	693.4062989	576.8411765	643.5149968
3.96_124.0165m/z	124.0165169	3.957583333	neg	2-Aminoethylphosphonic acid	4137	Organic acids and derivatives	Organic phosphonic acids and derivatives	Organic phosphonic acids	**	C03557	38.6	0	M-H	C2H8NO3P	-3.091049394	362.2110246	250.3141813	476.2326182	37.07220445	162.848021	293.0334123	277.5884628	283.6547804	319.0587823

3.98_277.0555m/z	277.055496	3.97985	neg	D-Erythroascorbic acid 1'-a-D-xylopyranoside	HMDB0033626	Organic oxygen compounds	Organooxygen compounds	Carbohydrates and carbohydrate conjugates			38.6	0.468	M-H	C10H14O9	-3.63063551	9049.615863	9357.354611	9698.085504	9394.153876	9024.490822	9125.1061	9030.960491	10409.84047	9338.176238
4.23_439.1359m/z	439.1359182	4.234483333	neg	Trifluoromethylphenylpropanediol	1958	Unclassified	Unclassified	Unclassified			38.6	0	2M-H	C10H11F3O2	2.194994349	584.4887394	558.6194607	610.4998742	398.2590778	439.7917804	534.9357487	630.4018454	995.3735778	863.8522116
4.23_535.1072m/z	535.1071529	4.234483333	neg	3-O-(6-O-alpha-D-Xylosylphospho-alpha-D-mannopyranosyl)-alpha-D-mannopy	73349	Unclassified	Unclassified	Unclassified			38.6	0	M-H2O-H	C17H31O18P	0.348694175	2060.945893	1928.623562	2256.340743	532.4984425	364.4863033	322.893575	2783.587514	3691.95943	3167.169896
4.30_229.1195m/z	229.1195383	4.300033333	neg	1-AMINOCYCLOBUTANE CARBOXYLIC ACID	44305	Unclassified	Unclassified	Unclassified			38.6	0	2M-H	C5H9NO2	0.685103455	60.07101581	138.7596052	86.42764451	184.4768148	156.0481178	169.419738	85.47207805	49.97304153	0.001403199
4.32_427.0411m/z	427.0411244	4.3224	neg	2-Deoxy-D-ribose 5-phosphate	3349	Organic oxygen compounds	Organooxygen compounds	Carbohydrates and carbohydrate conjugates	**	C00673	38.6	0	2M-H	C5H11O7P	-0.181735732	553.5378126	1021.069375	868.7014345	90.42228899	97.7248254	103.619584	345.7032393	583.0763111	450.8359331
4.43_258.0077m/z	258.0077348	4.432683333	neg	6-Hydroxyindole sulfate	HMDB0240651	Organic acids and derivatives	Organic sulfuric acids and derivatives	Arylsulfates			38.6	0	M+FA-H	C8H7NO4S	-0.219428283	497.5297011	962.2609607	625.7050976	513.4682964	492.6568644	340.275765	647.9640213	757.8222314	795.4645318
4.43_329.0879m/z	329.0878723	4.432683333	neg	2-O-Benzoyl-D-glucose	HMDB0034618	Organic oxygen compounds	Organooxygen compounds	Carbohydrates and carbohydrate conjugates			38.6	0	M+FA-H	C13H16O7	0.234310818	6050.490512	6857.219329	6666.53869	1342.890537	1334.826466	1129.758287	5145.667985	6907.067279	6331.373961
4.45_297.0620m/z	297.0619833	4.454616667	neg	(Z)-Tamarindienal	88592	Organic oxygen compounds	Organooxygen compounds	Carbonyl compounds	*		38.6	0	M+FA-H	C12H12O6	1.556275989	589.9990994	389.4607772	508.2337334	387.9851791	265.6058644	457.07604	659.2868227	654.9404697	606.0431952
4.50_113.0608m/z	113.0608356	4.49925	neg	4-Methyl-2,3-pentanedione	87764	Organic oxygen compounds	Organooxygen compounds	Carbonyl compounds	*		38.6	0	M-H	C6H10O2	0.28437346	116.3580076	104.0036306	101.5424134	130.2443509	118.1620572	116.1889737	221.0783665	238.0970154	235.2066277
4.50_301.0752m/z	301.0752429	4.49925	neg	Benfuresate	72258	Unclassified	Unclassified	Unclassified			38.6	0	M+FA-H	C12H16O4S	0.429862798	225.2594749	303.1787692	289.1188045	750.2468949	502.9597289	880.9169327	310.2992515	355.69673	298.1721342
4.50_427.2340m/z	427.2339548	4.49925	neg	2-Carboxy-5,7-dimethyl-4-octanolide	87330	Organoheterocyclic compounds	Lactones	Gamma butyrolactones	*		38.6	0	2M-H	C11H18O4	0.497709626	824.3261266	1012.851526	1003.922773	1773.259848	1616.50092	1738.896231	1032.066081	806.4727517	1022.582946
4.52_242.0922n	241.0849134	4.521733333	neg	Thymidine	3375	Nucleosides, nucleotides, and analogues	Pyrimidine nucleosides	Pyrimidine 2'-deoxyribonucleosides	**	C00214	38.6	5.09	M-H, M+FA-H	C10H14N2O5	7.923977152	55748.32774	9155.897278	196446.5743	14888.74482	11656.9325	12738.03762	13759.98024	13169.77429	30822.96872
4.54_269.1396m/z	269.139582	4.54425	neg	13-Oxo-9,11-tridecadienoic acid	90028	Lipids and lipid-like molecules	Fatty Acyls	Fatty acids and conjugates	*		38.6	0	M+FA-H	C13H20O3	0.600718773	576.9722385	629.7986447	358.3237917	504.0204365	439.4172087	537.0719484	611.2056305	601.6466061	919.670528
4.59_459.0938m/z	459.0938306	4.589283333	neg	Pyrazosulfuron-ethyl	72250	Unclassified	Unclassified	Unclassified			38.6	0	M+FA-H	C14H18N6O7S	-0.338663058	2015.46911	2215.467525	2068.391738	4336.868424	4329.775026	3414.622736	0.001403199	0.001403199	140.9241996
4.64_194.0460m/z	194.0459863	4.641416667	neg	5,6-Dihydroxyindole	7009	Organoheterocyclic compounds	Indoles and derivatives	Hydroxyindoles	**	C05578	38.6	0	M+FA-H	C8H7NO2	0.704213979	1131.221327	1039.572469	929.0206142	1378.023327	1458.683128	1355.419541	920.9269879	899.8135333	868.5592363
4.64_209.0822m/z	209.0821538	4.641416667	neg	4-Isopropylbenzoic acid	44724	Lipids and lipid-like molecules	Prenol lipids	Monoterpenoids	**	C06578	38.6	0	M+FA-H	C10H12O2	1.349001767	508.6834557	480.8511514	442.122663	132.7171374	154.5138181	90.70780806	1323.706141	1420.160211	1099.154725
4.70_312.0599m/z	312.0599487	4.703166667	neg	5'-Phosphoribosyl-N-formylglycinamidine	HMDB0002305	Organic oxygen compounds	Organooxygen compounds	Carbohydrates and carbohydrate conjugates	**	C04640	38.6	0	M-H	C8H16N3O8P	-0.881428895	2586.019719	2190.464476	2598.029342	2554.522069	2872.775049	2715.240681	3400.707977	2325.327588	2856.111548
4.75_259.0718m/z	259.0718325	4.747466667	neg	1,2,3,4-Tetrahydro-b-carboline-1,3-dicarboxylic acid	88186	Alkaloids and derivatives	Harmala alkaloids	Unclassified	*		38.6	0	M-H	C13H12N2O4	-2.299248675	7979.121273	8127.883806	9024.895338	6605.993331	7013.419845	6418.339811	7328.811406	7690.141462	7680.756599
4.77_351.0701m/z	351.0700714	4.769883333	neg	4-Methylumbelliferyl β-D-glucuronide	4080	Unclassified	Unclassified	Unclassified			38.6	4.78	M-H	C16H16O9	-5.919777275	8518.184934	8630.681116	9651.622584	7796.644834	8233.509011	7755.170565	8991.123991	10399.91947	9411.558957
4.86_223.0250m/z	223.0249838	4.858383333	neg	2,3-Dihydroxy-2,4-cyclopentadien-1-one	HMDB0039437	Organic acids and derivatives	Vinylogous acids	Unclassified			38.6	0	2M-H	C5H4O3	0.768809764	716.9214888	666.1267252	570.1884538	616.5154399	767.9153046	698.0392881	849.1041852	715.1828213	730.2584559
4.88_493.1308m/z	493.1308369	4.880083333	neg	N-Succinyl-L-glutamate	63479	Unclassified	Unclassified	Unclassified			38.6	0	2M-H	C9H13NO7	-0.587306523	1356.432689	1562.344642	2223.276076	1698.368244	1836.542	1811.762699	1811.448439	1775.004233	2248.816857
4.92_230.1519n	229.144597	4.92485	neg	Diisopropyl adipate	70124	Unclassified	Unclassified	Unclassified			38.6	0	M-H, M+FA-H	C12H22O4	0.279059106	3214.872585	3409.7694	4145.635919	4002.880056	4083.267739	4229.111977	3023.065643	3405.04687	3466.170766
4.95_315.1815m/z	315.181491	4.94705	neg	3-Oxooctanoic acid	HMDB0010721	Organic acids and derivatives	Keto acids and derivatives	Medium-chain keto acids and derivatives	*		38.6	0	2M-H	C8H14O3	0.56547141	1719.844504	1466.291491	1881.019995	1648.944459	1735.414349	1656.762274	1343.0933	1192.755148	930.3475929
5.01_307.0777m/z	307.0777312	5.014716667	neg	Glutamylaspartic acid	HMDB0028815	Organic acids and derivatives	Peptidomimetics	Hybrid peptides	*		38.6	0	M+FA-H	C9H14N2O7	-2.18413651	1183.717198	1185.211555	1651.514	1829.558388	2217.551732	1753.763341	2008.450129	1240.861287	1545.725862
5.04_278.1036m/z	278.1035669	5.037216667	neg	Phenethylamine glucuronide	HMDB0010323	Organic oxygen compounds	Organooxygen compounds	Carbohydrates and carbohydrate conjugates	**	C03033	38.6	0	M-H2O-H	C14H19NO6	0.574306212	1520.531512	1740.371827	1806.222571	2349.844294	2518.55751	2654.182817	1791.020782	1890.223086	2529.153132
5.06_380.0603m/z	380.0602923	5.05975	neg	2-Amino-5-formylamino-6-(5-phospho-D-ribosylamino)pyrimidin-4(3H)-one	70967	Unclassified	Unclassified	Unclassified			38.6	0	M-H	C10H16N5O9P	-2.611163305	542.9672879	537.0581584	648.7946109	146.0716467	257.4930233	251.6535204	680.464264	505.4688181	724.3414351
5.10_303.1086m/z	303.1085558	5.1031	neg	N1-Methyl-2-pyridone-5-carboxamide	HMDB0004193	Organoheterocyclic compounds	Pyridines and derivatives	Pyridinecarboxylic acids and derivatives	**	C05842	38.6	0	2M-H	C7H8N2O2	-4.349436018	2135.144234	2409.100898	2051.593213	2301.687684	3036.333432	3145.82845	3189.256756	2739.110284	2700.733376
5.10_377.1457m/z	377.1456924	5.1031	neg	1-(3-Methylbutanoyl)-6-apiosylglucose	94495	Lipids and lipid-like molecules	Fatty Acyls	Fatty acyl glycosides	*		38.6	0	M-H2O-H	C16H28O11	0.938496606	6234.474607	5996.332782	6553.955184	8165.826091	7089.440049	8350.000174	8467.927194	6825.390041	7380.634565
5.16_289.1659m/z	289.1659318	5.155116667	neg	Menthyl ethylene glycol carbonate	91297	Lipids and lipid-like molecules	Prenol lipids	Monoterpenoids	*		38.6	0	M+FA-H	C13H24O4	1.104562643	6432.388619	7160.495174	6550.436929	5856.747698	5847.293003	5758.808847	6720.2744	5950.203788	6034.59139
5.18_417.1191m/z	417.1191272	5.1777	neg	Tangeritin	HMDB0030539	Phenylpropanoids and polyketides	Flavonoids	O-methylated flavonoids	*	C10190	38.6	0	M+FA-H	C20H20O7	0.05747256	918.5491895	1107.763038	1257.170157	249.6801697	178.6097646	233.4372042	483.1369549	389.0345001	317.9394796
5.22_389.1457m/z	389.1456651	5.222166667	neg	(-)-erythro-Anethole glycol 1-glucoside	88958	Organic oxygen compounds	Organooxygen compounds	Carbohydrates and carbohydrate conjugates			38.6	0	M+FA-H	C16H24O8	1.001028148	5183.633997	5210.841872	5551.45669	8661.149767	8685.129278	9154.377125	6082.552539	4943.598461	6340.118677
5.24_328.1203m/z	328.1202553	5.244033333	neg	N-Phenylacetylphenylalanine	HMDB0002372	Organic acids and derivatives	Carboxylic acids and derivatives	Amino acids, peptides, and analogues	*		38.6	0	M+FA-H	C17H17NO3	4.270383267	1763.541886	1794.342772	1550.925677	2215.832148	2506.85438	2083.42013	1706.244117	1794.717722	2024.495545
5.29_285.0805m/z	285.0805179	5.28555	neg	5-Tetrazolyl-glycine	69671	Unclassified	Unclassified	Unclassified			38.6	0	2M-H	C3H5N5O2	-2.986726085	3446.193322	2926.840108	2951.705056	1161.902642	1377.996156	1628.605453	3956.14931	2787.626896	3455.029332
5.30_493.1332m/z	493.1331954	5.302883333	neg	N2-Succinylglutamic acid	6246	Unclassified	Unclassified	Unclassified			38.6	0	2M-H	C9H13NO7	4.185680786	1025.27342	841.3320901	1198.143219	1555.378558	2358.994402	2712.703878	1552.172341	1268.987673	1733.831375
5.32_137.0608m/z	137.0607796	5.320233333	neg	1,4-Dimethoxybenzene	HMDB0029671	Benzenoids	Benzene and substituted derivatives	Methoxybenzenes	*		38.6	0	M-H	C8H10O2	-0.170250935	163.718434	198.5606874	261.9104921	76.64824361	160.2238959	193.2268735	177.2346213	185.7032995	232.8021195
5.34_425.1821m/z	425.1820942	5.3394	neg	Pteroside B	87180	Organic oxygen compounds	Organooxygen compounds	Carbohydrates and carbohydrate conjugates	*		38.6	0	M+FA-H	C20H28O7	1.020815535	5478.615274	6295.577866	6386.585386	6075.114043	8774.739654	7269.954986	5529.018536	6386.980415	7028.894352
5.43_471.0005m/z	471.0005354	5.428733333	neg	Methazolamide	43249	Organoheterocyclic compounds	Azoles	Thiadiazoles		C07764	38.6	0	2M-H	C5H8N4O3S2	0.52443493	6185.63845	6229.673786	6483.836653	4278.711223	4326.745437	4052.201915	5788.467257	5145.670683	4839.234559
5.45_423.1278m/z	423.1277657	5.451166667	neg	Danielone	68483	Organic oxygen compounds	Organooxygen compounds	Carbonyl compounds	*	C10674	38.6	0	2M-H	C10H12O5	-4.490977382	926.9712098	897.8706508	977.4821881	1539.922374	1259.549316	1067.776441	1032.906796	1005.932833	1009.678937
5.47_373.0996m/z	373.0995956	5.471616667	neg	7-Hydroxy-6-methyl-8-ribityl lumazine	58232	Organoheterocyclic compounds	Pteridines and derivatives	Unclassified	**	C05995	38.6	0	M+FA-H	C12H16N4O7	-1.542599957	2053.534377	1639.591402	1579.270676	1369.744016	1454.109529	2011.451715	1043.929183	857.6267373	766.7446443
5.47_535.2190m/z	535.2189847	5.471616667	neg	Aldosterone 18-glucuronide	HMDB0010345	Lipids and lipid-like molecules	Steroids and steroid derivatives	Steroidal glycosides	*		38.6	0	M-H	C27H36O11	0.930853282	4654.970082	3956.59748	3326.198848	4403.03561	3814.991167	4437.255284	6280.567737	4147.200302	4768.779696
5.53_409.1472m/z	409.1472185	5.525416667	neg	N-Acetylmuramoyl-Ala	65878	Organic oxygen compounds	Organooxygen compounds	Carbohydrates and carbohydrate conjugates		C02999	38.6	0	M+FA-H	C14H24N2O9	2.293739053	10953.19834	10062.34152	9548.620005	9888.301669	11268.64323	10189.84681	9809.734767	9150.944094	10713.4229
5.61_145.0507m/z	145.0507368	5.610733333	neg	Methyl methacrylate	HMDB0032385	Organic acids and derivatives	Carboxylic acids and derivatives	Carboxylic acid derivatives	*	C19504	38.6	0	M+FA-H	C5H8O2	1.043308042	355.4576515	254.6820783	420.9524989	305.8452918	350.4995137	328.6776153	409.0557354	333.3143195	413.9806664
5.63_213.0559m/z	213.0558712	5.628083333	neg	Dibenzofuran	HMDB0251164	Unclassified	Unclassified	Unclassified			38.6	0	M+FA-H	C12H8O	0.913161705	1263.599637	1162.682113	1418.202434	1382.218372	1455.82926	1414.317318	1575.186298	1079.914992	1343.330076
5.64_325.1406m/z	325.1406173	5.6386	neg	Tyrosyl-Valine	86010	Organic acids and derivatives	Carboxylic acids and derivatives	Amino acids, peptides, and analogues	*		38.6	0	M+FA-H	C14H20N2O4	0.382892206	1195.504278	1237.209822	1475.592188	2769.091338	2680.75779	2592.431579	2087.262623	1361.158347	2062.775657
5.65_462.1775m/z	462.1774562	5.650833333	neg	Dihydroisomorphine-6-glucuronide	HMDB0061137	Alkaloids and derivatives	Morphinans	Unclassified	*		38.6	0	M-H	C23H29NO9	1.081843094	3111.960671	3038.583551	3661.591866	8114.158636	7778.397404	7445.861193	8002.512067	6552.515471	7948.355477
5.76_300.9920m/z	300.9920456	5.7632	neg	Erythrityl Tetranitrate	85532	Organic oxygen compounds	Organic oxoanionic compounds	Organic nitrates			38.6	0	M-H	C4H6N4O12	3.643824678	6720.007427	6963.495579	7655.478205	3966.097683	4558.439598	3825.349638	9174.844055	9200.157863	7781.440526
5.79_419.1355m/z	419.1355423	5.785316667	neg	Thiolactomycin	68888	Unclassified	Unclassified	Unclassified			38.6	0	2M-H	C11H14O2S	-0.19665347	364.5906851	394.3200534	288.3390732	270.132242	409.2921217	524.1298003	1166.859285	1254.970828	880.7128402
5.83_431.2287m/z	431.228702	5.825	neg	Oleandolide	LMPK04000031	Lipids and lipid-like molecules	Polyketides	Macrolides and lactone polyketides	**	C11990	38.6	0	M+FA-H	C20H34O7	0.118279192	5687.399898	5436.908751	5347.05698	2994.802706	1587.367116	1926.463704	6875.496753	6524.407339	5648.204274
5.98_473.1645m/z	473.1644657	5.9829	neg	Dyspropterin	6071	Organoheterocyclic compounds	Pteridines and derivatives	Pterins and derivatives	**	C03684	38.6	0	2M-H	C9H11N5O3	-1.342033988	8664.807679	8943.627777	8481.177559	7378.060482	8987.101141	9291.315199	5290.697124	5795.798029	5914.392996
6.09_187.1341m/z	187.1340706	6.087383333	neg	Dihydroisophorol	HMDB0029557	Organic oxygen compounds	Organooxygen compounds	Alcohols and polyols	*		38.6	0	M+FA-H	C9H18O	0.721545357	552.2058771	648.1735528	598.2922118	651.1572098	739.7956077	622.865652	623.7940487	753.815546	671.9627779
6.12_391.1979m/z	391.1979332	6.123483333	neg	(3S,4S)-3-hydroxytetradecane-1,3,4-tricarboxylic acid	45918	Lipids and lipid-like molecules	Fatty Acyls	Fatty Acids and Conjugates		C04529	38.6	0	M+FA-H	C17H30O7	1.666709231	5469.357133	6145.814385	5816.258179	5818.519888	5356.440371	5876.914323	4633.073946	5457.817462	5279.144426
6.18_415.1602m/z	415.1602426	6.181866667	neg	L-Kynurenine	HMDB0000684	Organic oxygen compounds	Organooxygen compounds	Carbonyl compounds	***	C00328	38.6	0	2M-H	C10H12N2O3	-4.963043593	4084.969139	3334.550669	3738.493721	3878.568774	3682.507531	3615.511565	2283.692556	2587.061875	2551.469134
6.20_151.1129m/z	151.1128899	6.195716667	neg	3-Decenoic acid	HMDB0031002	Lipids and lipid-like molecules	Fatty Acyls	Fatty acids and conjugates	*		38.6	0	M-H2O-H	C10H18O2	0.300727182	322.0168905	291.2852275	337.3560383	573.7623394	517.5017535	545.0356717	360.1122772	393.4896478	479.2456227
6.26_179.1079m/z	179.1078872	6.261966667	neg	1-Methyl-2-propylbenzene	88576	Benzenoids	Benzene and substituted derivatives	Phenylpropanes	*		38.6	0	M+FA-H	C10H14	0.998174353	425.139153	290.2317764	379.5913397	813.9372658	631.3160027	584.660092	391.5287026	512.8866893	492.0895989
6.43_269.1396m/z	269.1396074	6.433933333	neg	Annuionone C	88627	Organoheterocyclic compounds	Oxepanes	Unclassified	*		38.6	0	M+FA-H	C13H20O3	0.714000428	8337.099516	9085.479788	9724.832341	12142.63048	11118.00784	12057.20418	7999.771637	8149.618803	8587.657059
6.49_523.2551m/z	523.2551217	6.494283333	neg	Prolylphenylalanine	HMDB0011179	Organic acids and derivatives	Carboxylic acids and derivatives	Amino acids, peptides, and analogues	*		38.6	0	2M-H	C14H18N2O3	-2.072935437	4033.945791	3850.203987	4327.813197	5153.925295	5033.320783	5156.270857	3512.72097	4792.164917	3909.696564
6.59_415.1612m/z	415.1612421	6.592933333	neg	Formyl-5-hydroxykynurenamine	63540	Organic oxygen compounds	Organooxygen compounds	Carbonyl compounds	**	C05647	38.6	0	2M-H	C10H12N2O3	-2.561328277	2293.828195	2197.422535	2252.797484	2318.86386	2219.563094	2541.641408	1764.180538	2244.29896	2182.543219
6.59_605.1832m/z	605.1831993	6.592933333	neg	Gynocardin	66910	Organic oxygen compounds	Organooxygen compounds	Carbohydrates and carbohydrate conjugates	*	C08331	38.6	0	2M-H	C12H17NO8	-0.589325804	5857.034306	5996.33667	6389.420545	1093.87873	1158.800799	1480.74086	3954.920889	5380.732278	3718.39945
6.64_308.1504m/z	308.1503913	6.644916667	neg	N-(Heptan-4-yl)benzo[d][1,3]dioxole-5-carboxamide	88349	Organoheterocyclic compounds	Benzodioxoles	Unclassified	*		38.6	0	M+FA-H	C15H21NO3	0.17065651	583.7351422	635.5183705	551.1275557	1337.646138	1064.83188	1083.377006	1176.912479	1237.973663	1365.861296
6.64_459.2241m/z	459.2240659	6.644916667	neg	methyl 6,8-epidioxy-5,15-dihydroperoxy-9,11,13-eicosatrienoate	74993	Lipids and lipid-like molecules	Fatty Acyls	Eicosanoids			38.6	0	M+FA-H	C21H34O8	1.195015898	18921.37815	20357.69108	20638.69215	25943.87236	24174.17471	23379.28922	21462.3876	25707.21143	25631.59612
6.69_325.1658m/z	325.1658362	6.69475	neg	Prehumulinic acid	86756	Organic acids and derivatives	Vinylogous acids	Unclassified	*		38.6	0	M+FA-H	C16H24O4	0.62148343	1076.452554	1713.462422	1563.796753	2880.672871	2931.540497	2753.54342	761.538113	1284.75633	1315.137566
6.71_295.1187m/z	295.1187112	6.70695	neg	Ubiquinone-1	45118	Lipids and lipid-like molecules	Prenol lipids	Quinone and hydroquinone lipids	**	C00399	38.6	0	M+FA-H	C14H18O4	-0.003090777	2849.53368	2852.928707	3310.496047	2759.743216	2334.801271	1682.850833	2253.159792	2984.041683	3265.92266
6.76_253.1083m/z	253.1083131	6.759433333	neg	gamma-Asarone	HMDB0029872	Benzenoids	Phenol ethers	Anisoles	*	C17821	38.6	0	M+FA-H	C12H16O3	0.79677241	3964.031873	4541.846416	4645.526495	4750.56273	4111.54683	4295.750188	4537.196314	4896.338417	4789.885613
6.78_643.3704m/z	643.3703609	6.776866667	neg	Perindoprilat lactam A	1800	Unclassified	Unclassified	Unclassified			38.6	0	2M-H	C17H26N2O4	-1.361487721	3019.994225	3039.859597	3953.207745	3576.07403	3412.666031	3974.907661	3639.486825	4159.438033	3134.714476
6.83_595.2356m/z	595.2356449	6.832183333	neg	7C-aglycone	HMDB0004808	Lipids and lipid-like molecules	Prenol lipids	Quinone and hydroquinone lipids	*		38.6	0	2M-H	C18H18O4	3.192002798	2150.731123	2773.926693	1885.912068	2146.214672	1991.386056	1975.108435	3572.945221	3132.531145	2697.067792
6.88_183.0664m/z	183.0664029	6.884583333	neg	7-Methyl-2-hydroxy-6-oxoocta-2,4-dienoate	66201	Unclassified	Unclassified	Unclassified			38.6	0	M-H	C9H12O4	0.654549288	346.5067657	447.0976654	313.9982739	302.4595142	436.1796013	301.6793464	434.326455	461.0096016	408.1315104
6.94_503.1903m/z	503.1903371	6.939266667	neg	Anatoxin a(s)	73483	Unclassified	Unclassified	Unclassified			38.6	0	2M-H	C7H17N4O4P	0.258236641	1804.466733	2335.394156	1949.679199	2332.866038	2045.803776	2082.74632	2264.014258	3164.752774	3205.941139
6.97_465.2498m/z	465.2497655	6.970716667	neg	6'β-Hydroxylovastatin	1038	Unclassified	Unclassified	Unclassified			38.6	0	M+FA-H	C24H36O6	0.889411189	6272.719475	7352.459216	7330.495233	12058.04946	9899.250282	11808.77414	5795.453925	7265.263398	6977.416668
6.99_331.2130m/z	331.2129536	6.993516667	neg	2,3-Dihydroxycyclopentaneundecanoic acid	70485	Unclassified	Unclassified	Unclassified			38.6	0	M+FA-H	C16H30O4	1.192546052	2561.670572	2124.313936	2357.572784	1967.201982	1635.562514	1857.74423	1790.265618	2381.948571	1880.621611
7.01_385.1510m/z	385.1510459	7.01105	neg	15-Deacetylneosolaniol	HMDB0036157	Lipids and lipid-like molecules	Prenol lipids	Sesquiterpenoids	*	C02951	38.6	0	M+FA-H	C17H24O7	1.881295072	6864.962877	6825.047751	6439.597291	7284.882418	6083.620104	6591.446993	5953.230686	6245.947557	6039.875283
7.10_152.0354m/z	152.0353832	7.097333333	neg	3-Pyridinaldehyde	66624	Unclassified	Unclassified	Unclassified			38.6	0	M+FA-H	C6H5NO	0.621671502	1042.80721	906.240238	921.7710423	879.2394213	1013.070097	993.3603861	1133.432509	985.6115136	1016.349904
7.30_215.1289m/z	215.128922	7.29525	neg	Caproleic acid	LMFA01030033	Lipids and lipid-like molecules	Fatty Acyls	Fatty acids and conjugates	*		38.6	0	M+FA-H	C10H18O2	0.230934683	1419.392859	1546.188184	1611.28525	2860.41604	2535.153707	2319.467123	1588.079494	1480.70678	1614.67284
7.59_459.0938m/z	459.0938008	7.58625	neg	Premithramycinone	63775	Unclassified	Unclassified	Unclassified			38.6	0	M+FA-H	C21H18O9	1.245466359	9842.212934	10132.94149	9632.708484	9660.330192	8849.52003	10746.05524	10804.45093	10797.3625	10526.6195
7.79_236.1294m/z	236.1293565	7.793283333	neg	N,N-Diethylphenylacetamide	68704	Benzenoids	Benzene and substituted derivatives	Phenylacetamides	*	C10938	38.6	0	M+FA-H	C12H17NO	0.729691263	637.6688796	828.9489931	997.1801235	1137.864011	951.6807237	1028.654555	664.3296203	796.775078	696.2854897
7.97_439.1973m/z	439.1973276	7.9723	neg	Pteroside Z	88545	Organic oxygen compounds	Organooxygen compounds	Carbohydrates and carbohydrate conjugates	*		38.6	0	M+FA-H	C21H30O7	-0.072575165	4244.545072	3824.210243	3810.729076	8016.363565	6555.06672	7758.768304	3592.013459	4197.365615	3941.864517
8.12_549.1936m/z	549.193647	8.11925	neg	Sarmentosin	HMDB0030697	Lipids and lipid-like molecules	Fatty Acyls	Fatty acyl glycosides	*	C08340	38.6	0	2M-H	C11H17NO7	-0.146091672	6689.674963	6771.031852	7542.718682	2992.147266	2837.738656	2569.400537	4873.275933	5715.571438	5625.117384
8.29_311.1867m/z	311.1866832	8.294766667	neg	Tetranor-12(R)-HETE	35513	Unclassified	Unclassified	Unclassified			38.6	0	M+FA-H	C16H26O3	1.072945581	1926.090651	2133.162967	2408.419925	3238.346012	2649.372768	3055.98197	1454.63018	1604.938362	1635.523851
8.29_448.3072m/z	448.3071709	8.294766667	neg	CB-25	45213	Unclassified	Unclassified	Unclassified			38.6	0	M+FA-H	C25H41NO3	0.803076549	7912.419441	9172.54351	9926.985798	0.001403199	0.001403199	0.001403199	3615.446414	3994.314787	4076.914157
8.46_171.0201m/z	171.0200731	8.463816667	neg	Nitroxoline	85514	Organoheterocyclic compounds	Quinolines and derivatives	Nitroquinolines and derivatives			38.6	0	M-H2O-H	C9H6N2O3	0.379479247	770.6457936	833.9948581	792.3907635	817.5479139	1020.809961	746.2122767	1005.858126	725.6725101	788.5536086
8.58_197.0205m/z	197.0204583	8.57575	neg	DNOC	72439	Unclassified	Unclassified	Unclassified			38.6	0	M-H	C7H6N2O5	0.320531621	2072.887219	1718.363673	1894.594447	1765.407849	2006.645753	2124.442312	2090.636417	2068.770912	1977.085162
8.65_311.1867m/z	311.1866831	8.654333333	neg	Tetranor 12-HETE	HMDB0060055	Lipids and lipid-like molecules	Fatty Acyls	Fatty acids and conjugates	*		38.6	0	M+FA-H	C16H26O3	1.072688898	1985.452836	2233.035424	1837.785003	5084.448233	4780.756565	4069.833162	1161.557255	1081.998355	1115.43449
8.81_270.2076m/z	270.2076272	8.814016667	neg	Tridecanoylglycine	HMDB0013317	Organic acids and derivatives	Carboxylic acids and derivatives	Amino acids, peptides, and analogues	*		38.6	0	M-H	C15H29NO3	0.589196896	1040.452496	1075.654736	935.3567946	938.7599207	808.909308	993.5859179	1013.488671	870.0889259	1154.988337
8.94_358.2603m/z	358.2602505	8.944866667	neg	3-hydroxydodecanoylcarnitine	LMFA07070039	Lipids and lipid-like molecules	Fatty Acyls	Fatty esters			38.6	0	M-H	C19H37NO5	0.984364498	6902.362631	5980.724984	6867.106332	5800.483372	6807.995816	6776.849059	6775.920411	5948.925814	6829.638043
9.47_409.1871m/z	409.1870687	9.466483333	neg	Phorbol	41249	Lipids and lipid-like molecules	Prenol lipids	Isoprenoids	**	C09155	38.6	0	M+FA-H	C20H28O6	0.761077888	3827.296826	4340.94827	4501.713713	3647.639199	3543.75729	3606.596413	4217.440223	4699.01353	4240.402503
9.69_355.1766m/z	355.1765918	9.6906	neg	Valdiate	HMDB0040980	Lipids and lipid-like molecules	Prenol lipids	Monoterpenoids	*		38.6	0	M+FA-H	C17H26O5	1.17670323	4495.71802	5320.710174	4939.303498	4590.657695	5831.025417	5174.522942	5489.938976	4913.919522	4941.557369
9.84_565.0481m/z	565.0480716	9.835916667	neg	Uridine diphosphate glucose	HMDB0000286	Nucleosides, nucleotides, and analogues	Pyrimidine nucleotides	Pyrimidine nucleotide sugars	**	C00029	38.6	0	M-H	C15H24N2O17P2	0.578483001	1622.397995	1154.228941	1626.977079	1842.401463	2478.249459	1865.462322	1876.290897	2190.867867	1750.202508
9.94_397.1083m/z	397.108324	9.943016667	neg	pamoate	4070	Benzenoids	Naphthalenes	Naphthalenecarboxylic acids and derivatives			38.6	0	M-H2O-H	C25H20O6	0.424767314	10395.53135	8656.214067	7965.925241	8841.575219	9586.193	9865.214202	8976.272809	8780.699089	8809.562009
0.69_137.0357m/z	137.0356518	0.692166667	neg	5-Oxo-2(5H)-isoxazolepropanenitrile	87595	Organoheterocyclic compounds	Azoles	Isoxazoles	*		38.5	0	M-H	C6H6N2O2	0.005449854	239.9337342	174.7839724	289.4542462	371.7520086	402.0506995	449.8678352	331.3559345	364.3214645	312.6625457
0.69_719.4041m/z	719.4041331	0.692166667	neg	O-Acetylcypholophine	68387	Unclassified	Unclassified	Unclassified			38.5	0	2M-H	C20H28N2O4	2.213637013	3395.354444	2393.843552	2405.148855	1956.061003	2201.81352	3047.291609	6540.809052	4448.639954	4216.429725
0.82_138.9707m/z	138.9706736	0.819566667	neg	Sulfoacetic acid	HMDB0258590	Unclassified	Unclassified	Unclassified			38.5	0	M-H	C2H4O5S	0.041484713	14322.77852	14436.77978	17212.99092	24945.42701	25857.64661	24721.66231	18240.68914	17476.64552	18332.69293
0.84_299.0986m/z	299.0986238	0.841733333	neg	D-Xylose	HMDB0000098	Organic oxygen compounds	Organooxygen compounds	Carbohydrates and carbohydrate conjugates	**	C00181	38.5	0	2M-H	C5H10O5	0.844287843	1281.595455	1591.361807	1857.555295	1577.033758	725.3828343	1501.328883	1134.027064	1465.138664	1648.032905
0.86_165.0768m/z	165.0768231	0.86335	neg	1-Deoxy-D-glucitol	95885	Organic oxygen compounds	Organooxygen compounds	Carbohydrates and carbohydrate conjugates			38.5	0	M-H	C6H14O5	-0.144851737	1130.826726	1027.464752	1151.779948	792.9802665	331.5103404	616.5385153	869.5615003	831.1402891	315.4131628
0.86_233.0473m/z	233.0473202	0.86335	neg	F-Honaucin A	65489	Unclassified	Unclassified	Unclassified			38.5	0	M+FA-H	C8H9FO4	3.352170251	8590.426809	9695.703907	10664.31756	7222.379303	8663.284225	7323.700781	6809.280587	7709.489835	8177.022615
0.86_345.0247m/z	345.0247044	0.86335	neg	Polyvidone	HMDB0033843	Organoheterocyclic compounds	Pyrrolidines	N-alkylpyrrolidines			38.5	0	2M-H	C6H9NOP2	0.307738701	984.5472741	584.7909084	1720.896008	3384.709099	2399.589721	1999.750759	1227.16046	1478.699326	1369.468954
0.97_142.0510m/z	142.050986	0.967616667	neg	4-Methyl-L-glutamate	63279	Unclassified	Unclassified	Unclassified			38.5	0	M-H2O-H	C6H11NO4	0.119900495	458.1982412	446.5685999	435.4609629	492.18649	472.4529563	486.8605195	549.2915605	423.6278541	511.4219488
1.14_339.0925m/z	339.092474	1.1382	neg	6-O-(3R,4-dihydroxy-2-methylene-butanoyl)-beta-D-glucopyranose	LMSL05000003	Unclassified	Unclassified	Unclassified			38.5	0.319	M+FA-H	C11H18O9	-2.757769184	1100.524563	423.1055211	270.4156968	1544.026233	1045.901602	2049.940678	165.9416677	1009.713019	740.96771
1.18_204.0513m/z	204.0513368	1.180366667	neg	L-trans-alpha-Amino-2-carboxycyclopropaneacetic acid	HMDB0034442	Organic acids and derivatives	Carboxylic acids and derivatives	Amino acids, peptides, and analogues			38.5	0	M+FA-H	C6H9NO4	-0.15008513	1291.303992	1297.016076	1740.880894	674.5882104	931.3904035	1126.780071	1086.264219	1237.961282	836.6460674
1.22_127.0514m/z	127.0514081	1.224233333	neg	L-Cyclo(alanylglycyl)	87756	Organic acids and derivatives	Carboxylic acids and derivatives	Amino acids, peptides, and analogues	*		38.5	0	M-H	C5H8N2O2	0.835823511	102.5974283	108.5439692	85.5821099	221.1222066	175.4713246	271.0199123	135.6024035	165.1687899	118.3074488
1.46_225.0383m/z	225.0382577	1.459966667	neg	Azomycin	70132	Unclassified	Unclassified	Unclassified			38.5	0	2M-H	C3H3N3O2	2.130020815	338.6147629	434.5827473	350.6208162	532.8155115	465.4347642	443.1713709	494.5693073	267.7461121	332.5460454
1.52_232.1062n	231.0987801	1.515416667	neg	N2-Succinyl-L-ornithine	63478	Organic acids and derivatives	Carboxylic acids and derivatives	Amino acids, peptides, and analogues	**	C03415	38.5	0	M-H2O-H, M-H	C9H16N2O5	1.214527565	1831.757109	1748.749833	1860.994327	3177.34677	2774.258821	2656.85763	1818.839534	1763.982354	1805.353928
1.76_159.0776m/z	159.0776362	1.7577	neg	N-Mononitrosopiperazine	HMDB0061162	Organoheterocyclic compounds	Piperidines	Nitrosopiperidines	*		38.5	0	M+FA-H	C5H10N2O	1.055608595	84.78041221	115.4240653	66.26536131	58.23959836	16.24491087	118.9026966	58.0906654	87.31316987	97.04497869
1.90_177.0769m/z	177.0768955	1.899016667	neg	(5R)-5-Hydroxyhexanoic acid	5398	Lipids and lipid-like molecules	Fatty Acyls	Fatty acids and conjugates	*		38.5	0	M+FA-H	C6H12O3	0.366552677	470.3008221	426.1649918	375.628434	617.2926982	549.4897589	519.9296361	526.026771	356.5797558	455.7750923
1.93_123.0088m/z	123.008754	1.933583333	neg	2,3-Methylenesuccinic acid	HMDB0059762	Lipids and lipid-like molecules	Fatty Acyls	Fatty acids and conjugates	*		38.5	0	M-H2O-H	C6H6O4	-0.095488901	4865.492832	4803.299994	5004.410145	1413.480632	1356.796401	1501.869597	3841.170529	3878.136485	3841.216681
10.46_517.2421m/z	517.2420562	10.45966667	neg	Lucidenic acid K	HMDB0035890	Lipids and lipid-like molecules	Prenol lipids	Triterpenoids	*		38.5	1.63	M+FA-H	C27H36O7	-4.76476168	36040.81048	37675.18786	44754.05835	38666.13483	42241.83283	36173.61334	37586.24568	43643.61813	41599.87496
10.89_295.2279m/z	295.2279233	10.88813333	neg	(Z)-9-Cycloheptadecen-1-one	87593	Organic oxygen compounds	Organooxygen compounds	Carbonyl compounds	*		38.5	0	M+FA-H	C17H30O	0.219006602	884.5281367	1565.265724	897.8514363	837.5080125	676.28533	869.7087725	859.0691463	2011.079459	866.5782886
11.27_837.1883m/z	837.1883079	11.26556667	neg	Cyanidin 3-xyloside	HMDB0037984	Phenylpropanoids and polyketides	Flavonoids	Flavonoid glycosides			38.5	0	2M-H	C20H19O10+	-0.070773065	12422.79356	12168.79483	9826.420642	9514.202666	11150.10777	10770.47426	11216.41027	10732.43578	11159.64402
14.70_313.0786m/z	313.0785543	14.69558333	neg	O-Ethyl-O-(5-methyl-4-nitrophenyl)(1-methylpropyl)phosphoramidothioate	68780	Unclassified	Unclassified	Unclassified			38.5	0	M-H2O-H	C13H21N2O4PS	1.29680076	175079.4466	11194.36397	8779.31002	103927.6911	17360.70018	11646.3627	8012.829635	12555.79397	8484.408244
2.00_174.0772m/z	174.0771956	2.004766667	neg	trans-3-Aminocyclopentane-1-carboxylic acid	69643	Unclassified	Unclassified	Unclassified			38.5	0	M+FA-H	C6H11NO2	0.10962909	592.1101761	577.7187754	626.0485916	794.1092525	829.0462599	894.630204	658.5683717	982.7790909	780.2702606
2.29_161.0820m/z	161.0819973	2.294983333	neg	propyl propionate	LMFA07010412	Organic acids and derivatives	Carboxylic acids and derivatives	Carboxylic acid derivatives	*		38.5	0	M+FA-H	C6H12O2	0.558072952	762.9197647	668.9903186	586.765788	504.1652711	439.7126495	423.0796664	716.063537	767.0409779	645.4077121
2.31_277.0831m/z	277.0830866	2.3142	neg	Phenobarbital	HMDB0015305	Organoheterocyclic compounds	Diazines	Pyrimidines and pyrimidine derivatives	*	C07434	38.5	0	M+FA-H	C12H12N2O3	0.393941545	1422.22698	1170.958868	1028.01968	1344.103168	1300.686047	1214.82104	971.6360185	1388.190873	1095.292825

2.56_315.0725m/z	315.072533	2.56315	neg	S-(4,5-Dihydro-2-methyl-3-furanyl) ethanethioate	92601	Organoheterocyclic compounds	Dihydrofurans	Unclassified			38.5	0	2M-H	C7H10O2S	-1.555555894	3402.555993	3703.207691	4070.611993	2079.165668	2250.267766	2329.448721	1668.324326	2244.098627	2269.197682
2.62_153.0557m/z	153.0557274	2.622366667	neg	m-Cresol	HMDB0002048	Benzenoids	Phenols	Cresols	*	C01467	38.5	0	M+FA-H	C7H8O	0.089744497	331.2171202	349.2978592	360.3227485	132.7625561	56.14128936	138.9248187	340.044868	324.0398559	284.9882992
3.04_278.9525m/z	278.9525385	3.041866667	neg	(alpha-D-mannosyl)7-beta-D-mannosyl-diacetylchitobiosyl-L-asparagine, isoforn	HMDB0062252	Benzenoids	Phenol ethers	Anisoles			38.5	0	M+FA-H	C7H7IO	0.738412318	4312.250002	4148.772437	5044.956675	274.8289397	226.0140061	241.0460605	4091.138568	3899.868588	3993.787106
3.10_214.0723m/z	214.0722565	3.10055	neg	6-Hydroxydopamine	HMDB0001537	Benzenoids	Phenols	Benzenediols	*		38.5	0	M+FA-H	C8H11NO3	0.948964444	1300.368803	1275.772946	1322.436894	2136.576476	1799.104039	1584.315944	1450.835551	1629.507851	1642.637705
3.46_269.1144m/z	269.1143737	3.455516667	neg	Hypoglycin B	66881	Organic acids and derivatives	Carboxylic acids and derivatives	Amino acids, peptides, and analogues	*	C08280	38.5	0	M-H	C12H18N2O5	0.290544056	2386.570578	3218.632194	3135.509073	2971.620637	2800.902326	2990.120385	2147.847125	1920.580552	1968.299513
3.64_231.1351m/z	231.1350609	3.640916667	neg	Leucyl-Threonine	85841	Organic acids and derivatives	Carboxylic acids and derivatives	Amino acids, peptides, and analogues	*		38.5	0	M-H	C10H20N2O4	0.130021005	175.9598687	161.4909888	156.0980657	289.0884752	327.3067871	271.3297239	936.9986841	732.6447877	750.4201017
3.69_342.9806m/z	342.980612	3.685866667	neg	Bismuth Subsalicylate	HMDB0015408	Benzenoids	Unclassified	Unclassified		C07870	38.5	0	M-H2O-H	C7H5BiO4	-2.014515436	3294.547726	3638.428297	3632.51973	5684.693765	5506.909617	4286.141196	4631.789195	4273.542824	4129.610272
3.73_252.0879m/z	252.0878912	3.730516667	neg	Phenylpropionylglycine	HMDB0000860	Organic acids and derivatives	Carboxylic acids and derivatives	Amino acids, peptides, and analogues	*		38.5	0	M+FA-H	C11H13NO3	0.700615826	1032.39243	874.4985633	950.3605125	1311.478871	1198.019773	900.9390419	649.225436	746.2538476	873.2442704
3.75_253.1195m/z	253.119491	3.747833333	neg	(S)-2,3,4,5-Tetrahydropiperidine-2-carboxylate	62803	Organic acids and derivatives	Carboxylic acids and derivatives	Amino acids, peptides, and analogues	**	C00450	38.5	0	2M-H	C6H9NO2	0.434102371	399.4563018	303.9018432	296.007656	348.1766033	340.6682475	184.3633752	199.5278135	247.0604443	244.2688352
3.85_283.0012n	564.9854043	3.84605	neg	Risedronic acid	66852	Organic acids and derivatives	Organic phosphonic acids and derivatives	Bisphosphonates		C08233	38.5	0	M-H, 2M-H	C7H11NO7P2	0.380016056	1207.670165	1651.287943	1374.19004	1358.946084	2037.829011	1881.992016	2328.807171	2251.946038	2364.302108
3.91_291.0990m/z	291.0990379	3.912566667	neg	N-acetyltryptophan	85257	Organic acids and derivatives	Carboxylic acids and derivatives	Amino acids, peptides, and analogues	*		38.5	0	M+FA-H	C13H14N2O3	1.595788382	1288.466067	1219.455818	1167.662059	1105.924519	1008.427313	1357.4312	1273.575861	1387.904649	1285.939523
4.05_352.0112m/z	352.0112299	4.046666667	neg	Zalcitabine diphosphate	3064	Unclassified	Unclassified	Unclassified			38.5	0	M-H2O-H	C9H15N3O9P2	1.937044507	2376.863509	2684.580119	2938.309859	1663.744829	2014.42132	1828.633964	1939.032445	2013.39415	1841.461261
4.09_213.0771m/z	213.0770896	4.091783333	neg	Ethyl 2-furanpropionate	HMDB0032921	Lipids and lipid-like molecules	Fatty Acyls	Fatty acid esters	*		38.5	0	M+FA-H	C9H12O3	1.442628547	1248.327721	1256.921653	1309.770243	1504.857942	1332.279503	1208.818083	1321.892723	1426.857288	1249.002145
4.09_355.1038m/z	355.1038446	4.091783333	neg	1-Pentadecanecarboxylic acid	94500	Lipids and lipid-like molecules	Fatty Acyls	Fatty acids and conjugates	**	C00249	38.5	0	M+FA-H	C15H18O7	1.253974431	1329.629237	1356.263696	1399.228174	802.5294594	612.4119199	604.8957964	1149.461786	1308.088289	1389.983291
4.16_421.0751m/z	421.0750605	4.15895	neg	Lactose 6-phosphate	58544	Organic oxygen compounds	Organooxygen compounds	Carbohydrates and carbohydrate conjugates	**	C05396	38.5	0	M-H	C12H23O14P	-0.485875924	885.3082611	1249.18266	1080.26089	927.5256372	502.1363642	561.2775438	2188.040163	889.2881331	1058.95869
4.21_303.1451m/z	303.1451246	4.212116667	neg	Butyl 3-O-beta-D-glucopyranosyl-butanoate	98328	Lipids and lipid-like molecules	Fatty Acyls	Fatty acyl glycosides			38.5	0	M-H2O-H	C14H26O8	0.614231906	8102.759866	8717.661074	8934.960331	12114.11736	10559.52545	11158.74811	8083.881444	9130.527483	9063.659485
4.23_331.1040m/z	331.1039946	4.234483333	neg	Salicin	HMDB0003546	Organic oxygen compounds	Organooxygen compounds	Carbohydrates and carbohydrate conjugates	***	C01451	38.5	0	M+FA-H	C13H18O7	1.883211092	444.0398134	711.4091895	786.1725359	498.3045189	446.2295981	340.9230097	517.1370436	603.7890504	396.9837536
4.30_261.1244m/z	261.1244475	4.300033333	neg	Nb-Acetyl-Nb-methyltryptamine	86541	Organoheterocyclic compounds	Indoles and derivatives	Indoles	*		38.5	0	M+FA-H	C13H16N2O	-0.085450853	3609.93999	3038.035041	3225.712569	3622.270621	3668.290207	3904.509362	4532.875692	4176.002523	4580.064924
4.30_425.2186m/z	425.2185568	4.300033333	neg	[6]-Gingerdiol 3,5-diacetate	95053	Lipids and lipid-like molecules	Fatty Acyls	Fatty alcohol esters	*		38.5	0	M+FA-H	C21H32O6	1.223470115	1552.164437	1131.231869	1614.337552	2288.968352	2154.973358	1838.412473	1619.168274	1363.990311	1481.289102
4.37_249.0358m/z	249.0357775	4.367166667	neg	Diflunisal	HMDB0014999	Benzenoids	Benzene and substituted derivatives	Biphenyls and derivatives		C01691	38.5	0	M-H	C13H8F2O3	-4.385837425	8770.655554	10222.17751	10285.03656	16586.87694	11879.00552	7911.70487	7323.529508	8814.135088	8471.233195
4.37_296.0938m/z	296.0938294	4.367166667	neg	Citpressine II	86188	Organoheterocyclic compounds	Quinolines and derivatives	Benzoquinolines	*		38.5	0	M-H2O-H	C17H17NO5	3.166702007	178.3682924	269.2582914	410.58926	1024.707418	541.9863257	684.2487377	269.1552846	346.3707428	335.5606432
4.39_321.0625m/z	321.0624865	4.3893	neg	Aesculin	HMDB0030820	Phenylpropanoids and polyketides	Coumarins and derivatives	Coumarin glycosides	*	C09264	38.5	1.23	M-H2O-H	C15H16O9	2.633328676	34480.75436	34440.45091	31584.20435	27558.74502	25337.15254	26573.57331	39132.11259	39540.85085	38945.75022
4.52_412.1077m/z	412.107662	4.521733333	neg	SB 228357	69191	Unclassified	Unclassified	Unclassified			38.5	0	M-H2O-H	C22H17F4N3O2	-0.43261201	1674.532432	1888.052549	1709.212635	1220.486002	1412.41333	1518.928358	1934.419331	2109.142861	2147.047206
4.54_329.1609m/z	329.1608892	4.54425	neg	Dihydroartemisinin	1119	Lipids and lipid-like molecules	Prenol lipids	Sesquiterpenoids	*		38.5	0	M+FA-H	C15H24O5	1.099583897	927.3296588	1005.084772	800.408886	1316.907057	1095.315853	1235.451965	920.3797463	1014.530275	1137.2907
4.57_293.1146m/z	293.1146446	4.5668	neg	2-Oxomelatonin	HMDB0060721	Organoheterocyclic compounds	Indoles and derivatives	Indolines	*		38.5	0	M+FA-H	C13H16N2O3	1.407872387	410.4188495	506.7780135	349.1813791	523.7707207	487.2600304	526.6762521	485.6791089	574.0877634	589.4751839
4.63_425.1670m/z	425.1669861	4.632666667	neg	3-Methyl-3-butenyl apiosyl-(1->6)-glucoside	HMDB0031954	Lipids and lipid-like molecules	Fatty Acyls	Fatty acyl glycosides	*		38.5	0	M+FA-H	C16H28O10	1.410201051	4129.23541	3549.442358	3182.410938	4021.013099	4219.044328	3668.723483	3373.094537	2748.304785	3135.646338
4.64_113.0609m/z	113.0608731	4.641416667	neg	5-hydroxy caproic acid	LMFA01050014	Lipids and lipid-like molecules	Fatty Acyls	Fatty acids and conjugates	*		38.5	0	M-H2O-H	C6H12O3	0.529681861	350.1693075	271.7005501	326.3528869	551.1436413	484.2395894	534.9710499	383.7849586	389.5886288	324.003178
4.64_221.0457m/z	221.0456901	4.641416667	neg	4-Methylumbelliferone	HMDB0059622	Phenylpropanoids and polyketides	Coumarins and derivatives	Hydroxycoumarins	***	C03081	38.5	0	M+FA-H	C10H8O3	0.813062439	1104.304841	956.4627729	1048.700173	495.4311406	463.0829926	525.776284	1069.804641	1112.263402	738.4589774
4.68_490.1725m/z	490.1724596	4.682483333	neg	Narceine	67831	Alkaloids and derivatives	Aporphines	Unclassified	*	C09591	38.5	0	M+FA-H	C23H27NO8	1.325196525	512.2981581	263.9303268	847.2821248	0.001403199	0.001403199	0.001403199	1262.785173	1105.539169	1497.628685
4.73_357.1194m/z	357.1193861	4.725416667	neg	Nivalenol	64093	Lipids and lipid-like molecules	Prenol lipids	Sesquiterpenoids	*	C06080	38.5	0	M+FA-H	C15H20O7	0.897887529	26496.50375	27846.39498	27368.55176	23024.44483	21644.94851	22781.17217	27547.97859	28892.38554	27448.06765
4.73_469.1331m/z	469.1330771	4.725416667	neg	6-Succinoaminopurine	HMDB0013204	Organoheterocyclic compounds	Imidazopyrimidines	Purines and purine derivatives	*		38.5	0	2M-H	C9H9N5O3	-1.541731566	307.0747133	164.1095096	185.0846167	1132.301959	1166.396953	752.2305255	830.6129311	1092.745772	606.372991
4.75_261.0690m/z	261.0689737	4.747466667	neg	Diphenylmethylphosphine oxide	519	Unclassified	Unclassified	Unclassified			38.5	0	M+FA-H	C13H13OP	1.708617339	2629.798634	2670.496846	2793.97002	2112.113673	2064.030286	2192.424505	2140.205646	2357.262139	2375.400592
4.75_277.0692m/z	277.0692385	4.747466667	neg	5-Hydroxypyrazinamide	HMDB0001978	Organoheterocyclic compounds	Diazines	Pyrazines	*	C01956	38.5	0	2M-H	C5H5N3O2	0.582824927	471.8793213	215.2952773	500.9744612	436.949258	671.1455844	412.8118771	381.8087805	557.4003905	279.8317229
4.79_429.0832m/z	429.0832154	4.792316667	neg	5,6-Dimethoxysterigmatocystin	HMDB0030592	Phenylpropanoids and polyketides	Sterigmatocystins	Unclassified	*		38.5	0	M+FA-H	C20H16O8	1.289035768	2302.938848	2451.769021	2353.884906	7012.359149	6462.003629	6530.309729	194.7666059	0.001403199	0.001403199
4.81_254.9947m/z	254.9946686	4.814166667	neg	4-Methylumbelliferyl sulfate	4083	Phenylpropanoids and polyketides	Coumarins and derivatives	Unclassified		C11585	38.5	8.01	M-H	C10H8O6S	-8.647990092	22738.37557	22939.20965	25063.60536	9795.900786	9926.333158	9496.849241	11443.63161	12631.03206	12075.37062
4.86_507.0943m/z	507.0942947	4.858383333	neg	farnesyl triphosphate	LMPR0103010023	Lipids and lipid-like molecules	Prenol lipids	Isoprenoids		C03115	38.5	0	M+FA-H	C15H29O10P3	-2.738022397	355.0020927	215.6065869	208.0065163	2529.318339	2327.744248	2288.948174	0.001403199	0.001403199	113.1028602
4.90_129.0922m/z	129.0922151	4.902316667	neg	Butyl propionate	86689	Organic acids and derivatives	Carboxylic acids and derivatives	Carboxylic acid derivatives	*		38.5	0	M-H	C7H14O2	0.859665818	252.7630625	290.4316035	331.6133245	315.1523523	310.0185063	285.5646349	285.9686863	271.7889692	191.1915393
4.95_145.0508m/z	145.0507562	4.94705	neg	β-ethyl acrylic acid	34696	Lipids and lipid-like molecules	Fatty Acyls	Fatty acids and conjugates	*		38.5	0	M+FA-H	C5H8O2	1.237469584	320.5304907	234.8059437	260.1497869	265.1754888	322.7019437	249.7098276	360.9913528	290.7072758	256.3365568
5.13_471.1852m/z	471.1851782	5.132616667	neg	Carbamazepine	1489	Organoheterocyclic compounds	Benzazepines	Dibenzazepines	**	C06868	38.5	0	2M-H	C15H12N2O	5.355052411	8392.911592	9586.774554	7655.272743	8239.331762	7664.345068	9096.273001	9836.968163	9500.839819	10584.41253
5.13_507.2084m/z	507.2084274	5.132616667	neg	Patrinoside	69143	Unclassified	Unclassified	Unclassified			38.5	0	M+FA-H	C21H34O11	0.243637485	3428.393037	4035.681621	2663.133346	825.1898097	752.6102488	664.5398879	1439.791089	1686.803662	1922.741933
5.16_423.1664m/z	423.1664392	5.155116667	neg	S-2,5-Dimethyl-3-furanyl 3-methylbutanethioate	92121	Organosulfur compounds	Thioethers	Aryl thioethers			38.5	0	2M-H	C11H16O2S	-1.145374253	1091.077804	1320.143632	831.0899276	222.5085326	146.9650744	296.4743779	432.6355239	583.9758072	795.3307318
5.18_551.1774m/z	551.1773802	5.1777	neg	Diosbulbinoside D	HMDB0030084	Organoheterocyclic compounds	Naphthopyrans	Unclassified	*		38.5	0	M+FA-H	C25H30O11	0.722121782	2134.777188	2224.332981	3021.755834	0.001403199	0.001403199	120.5077616	4331.874538	4043.020573	4958.990199
5.24_421.0754m/z	421.0753648	5.244033333	neg	Trehalose 6-phosphate	HMDB0001124	Organic oxygen compounds	Organooxygen compounds	Carbohydrates and carbohydrate conjugates	**	C00689	38.5	0	M-H	C12H23O14P	0.235002003	1200.990432	1182.137499	1247.842277	642.3314282	492.4778174	638.1208601	820.9563554	757.408265	949.6391914
5.32_181.0719m/z	181.071896	5.320233333	neg	2-Methylerythritol	62404	Organic oxygen compounds	Organooxygen compounds	Carbohydrates and carbohydrate conjugates	*		38.5	0	M+FA-H	C5H12O4	0.986476331	263.8760766	143.0650522	264.2830746	225.5452365	252.7664014	318.7955155	324.3703323	239.3705441	319.3159761
5.36_272.0542m/z	272.054207	5.361783333	neg	Adefovir	68958	Organoheterocyclic compounds	Imidazopyrimidines	Purines and purine derivatives			38.5	0	M-H	C8H12N5O4P	-4.420608623	466.2425828	580.0767761	621.4987076	607.124502	528.0147828	482.0963389	391.367791	433.3021091	553.7765172
5.36_277.1057m/z	277.1057481	5.361783333	neg	2-Methylaminoadenosine	65933	Unclassified	Unclassified	Unclassified			38.5	0	M-H2O-H	C11H16N6O4	0.966545874	1988.333671	2210.974722	2272.666417	1783.652984	2190.509387	1996.274496	1161.075142	1115.352413	1702.651699
5.36_393.2399m/z	393.2398551	5.361783333	neg	5-Ethyl-4-methyl-2-pentylthiazole	94631	Organoheterocyclic compounds	Azoles	Thiazoles			38.5	0	2M-H	C11H19NS	-1.292864568	2334.246195	3426.160467	4003.156134	2848.469235	2417.782306	2313.863041	2394.345002	2927.585814	2719.394743
5.36_527.2862m/z	527.2862424	5.361783333	neg	N-Valylphenylalanine	HMDB0255244	Organic acids and derivatives	Carboxylic acids and derivatives	Amino acids, peptides, and analogues	*		38.5	0	2M-H	C14H20N2O3	-2.396657561	720.3443241	682.6566447	933.5806396	1404.654663	1542.051768	1019.128664	575.9099591	487.0679944	788.2574784
5.41_395.0968m/z	395.0968177	5.406433333	neg	Syringic acid	HMDB0002085	Benzenoids	Benzene and substituted derivatives	Benzoic acids and derivatives	***	C10833	38.5	0	2M-H	C9H10O5	-3.919989388	4873.356852	5349.127968	4901.178255	8980.253333	9402.116541	8252.501453	7018.69969	7050.579603	6443.387698
5.49_205.0905m/z	205.090544	5.489016667	neg	(+/-)-3-(Methylthio)heptanal	HMDB0032426	Organic oxygen compounds	Organooxygen compounds	Carbonyl compounds	*		38.5	0	M+FA-H	C8H16OS	0.967953113	1443.128183	1149.982357	1002.55145	260.42547	253.9530042	363.4548819	1034.178129	834.3276443	1004.308771
5.50_259.1551m/z	259.1550996	5.497816667	neg	3R-hydroxy-5Z-dodecenoic acid	35609	Lipids and lipid-like molecules	Fatty Acyls	Fatty Acids and Conjugates			38.5	0	M+FA-H	C12H22O3	0.010343169	1549.966745	1094.434063	1146.188473	1211.290447	1512.294371	1278.843096	879.354626	827.6369171	957.2253524
5.52_375.0855m/z	375.0854692	5.516816667	neg	1-Hydroxy-2-naphthoic acid	2389	Unclassified	Unclassified	Unclassified			38.5	0	2M-H	C11H8O3	-5.165061695	1264.928415	1185.614661	1123.273507	202.0306143	445.3679598	219.0094662	723.106557	946.6224824	1012.989231
5.52_407.1927m/z	407.1926973	5.516816667	neg	Diethyl (2S,3R)-2-methyl-3-hydroxysuccinate	66217	Unclassified	Unclassified	Unclassified			38.5	0	2M-H	C9H16O5	1.044784149	3229.320395	2847.651276	2575.070352	2678.397712	3722.302765	3305.930021	3237.429877	3166.911094	3394.940977
5.53_511.1624m/z	511.1623879	5.525416667	neg	Fluorofelbamate	70814	Unclassified	Unclassified	Unclassified			38.5	0	2M-H	C11H13FN2O4	-4.307069287	5227.37201	5524.304741	5562.318485	6536.696327	8902.495761	6893.096903	5404.657654	5688.193561	4969.825504
5.53_545.1213m/z	545.121258	5.525416667	neg	Flindersiamine	68487	Unclassified	Unclassified	Unclassified			38.5	0	2M-H	C14H11NO5	1.995022669	2229.830074	2198.257818	1660.456036	1532.782329	2279.701574	1618.988449	1568.025949	1518.519822	2209.538473
5.56_145.0871m/z	145.0870869	5.56185	neg	2-Oxohexane	HMDB0005842	Organic oxygen compounds	Organooxygen compounds	Carbonyl compounds	*		38.5	0	M+FA-H	C6H12O	0.689847387	430.9964963	282.730181	234.8248849	269.920741	330.0768253	344.9523561	309.9340059	297.1271267	289.3856578
5.59_350.0833m/z	350.0833138	5.5933	neg	Convicine	HMDB0250445	Unclassified	Unclassified	Unclassified			38.5	0	M+FA-H	C10H15N3O8	-2.633645699	315.9738174	346.5702996	273.8359002	746.325735	858.567107	812.2387613	348.8691164	414.6076404	185.2945026
5.61_415.1603m/z	415.1602709	5.610733333	neg	4-Aminobenzoyl-(beta)-alanine	HMDB0060757	Organic acids and derivatives	Carboxylic acids and derivatives	Amino acids, peptides, and analogues	*		38.5	0	2M-H	C10H12N2O3	-4.894974592	2215.806734	1993.159709	1998.440057	1794.749498	1792.206276	1833.371773	2485.520822	2034.130886	1912.764837
5.63_337.0936m/z	337.0935543	5.628083333	neg	Dihydromikanolide	67660	Unclassified	Unclassified	Unclassified			38.5	0	M+FA-H	C15H16O6	2.270496525	5640.116511	6163.25258	6613.145052	5298.877373	4740.783421	4636.131489	5544.188999	4947.024043	5143.813504
5.73_257.1397m/z	257.1396532	5.727283333	neg	12-oxo-10E-dodecenoic acid	35783	Lipids and lipid-like molecules	Fatty Acyls	Fatty acids and conjugates	**	C16309	38.5	0	M+FA-H	C12H20O3	0.9701095	1129.451978	1328.647562	1094.282831	819.922298	923.0601336	953.2706102	612.6407169	835.8877312	1024.350872
5.76_381.1380m/z	381.1380417	5.7632	neg	L-Thiocitrulline (hydrochloride)	44816	Unclassified	Unclassified	Unclassified			38.5	0	2M-H	C6H13N3O2S	-0.987129314	2299.931943	2839.080303	2641.034832	3805.5508	4369.275696	3801.274456	4941.082888	4466.611879	3991.91538
5.79_253.1447m/z	253.1446701	5.785316667	neg	5,9:6,9-Diepoxy-4-megastigmene	HMDB0038529	Organoheterocyclic compounds	Oxepanes	Unclassified			38.5	0	M+FA-H	C13H20O2	0.66010876	2299.387046	2495.817265	2625.701854	2946.528797	2944.089119	2928.683471	2777.6019	2682.475842	2631.008336
5.79_589.3036m/z	589.303645	5.785316667	neg	Urobilin	HMDB0004160	Organoheterocyclic compounds	Tetrapyrroles and derivatives	Bilirubins	**	C05794	38.5	0	M-H	C33H42N4O6	0.82380264	15777.66539	15944.99249	15416.93838	11739.62451	13004.84993	12780.47212	21257.56565	20786.125	18350.48608
5.81_141.0920m/z	141.0919787	5.806016667	neg	3-Hydroxyvalproic acid	HMDB0013899	Lipids and lipid-like molecules	Fatty Acyls	Fatty acids and conjugates	**	C16651	38.5	0	M-H2O-H	C8H16O3	-0.777677501	351.1491225	378.8923072	444.0330896	805.2637765	728.111521	723.2345374	638.867037	535.2669568	550.7307555
5.81_354.1925m/z	354.1924642	5.806016667	neg	Metipranolol	66765	Benzenoids	Phenol esters	Unclassified		C07915	38.5	0	M+FA-H	C17H27NO4	0.818105436	2101.636654	2060.35867	2024.774767	6507.919174	5581.248247	5613.769308	3637.016401	3474.146906	3259.883794
5.81_453.0994m/z	453.0993729	5.806016667	neg	Hydroxynicorandil	1522	Unclassified	Unclassified	Unclassified			38.5	0	2M-H	C8H9N3O5	-3.944940558	8129.806423	8377.453108	8021.619892	23858.62292	18970.22046	19735.94093	14840.9285	12347.32806	12893.87094
5.87_311.1139m/z	311.1138889	5.866533333	neg	(+)-2,5-epi Goniothalesdiol	64661	Unclassified	Unclassified	Unclassified			38.5	0	M+FA-H	C14H18O5	0.985874118	930.8812179	1315.616734	1438.104285	1678.164816	1738.312589	1710.316083	3777.451971	3810.129611	4161.899698
5.94_469.1714m/z	469.1714017	5.937716667	neg	Taraxinic acid glucosyl ester	90559	Lipids and lipid-like molecules	Prenol lipids	Terpene lactones	*		38.5	0	M+FA-H	C21H28O9	-0.315071455	2882.158204	3142.590041	3646.482044	7122.49062	9006.616903	8013.067547	3033.002171	3453.452406	3868.465866
5.97_368.2200n	413.2183145	5.965483333	neg	5(6)-Epoxy Prostaglandin E1	LMFA03020068	Lipids and lipid-like molecules	Fatty Acyls	Eicosanoids	*	C04741	38.5	0	M-H2O-H, M+FA-H	C20H32O6	0.311822759	14707.91504	16610.6276	17357.41368	5861.873122	7233.38629	6517.068802	13378.26708	13975.05404	14535.29521
5.99_329.1244m/z	329.124446	5.9916	neg	N2-Methylguanine	HMDB0006040	Organoheterocyclic compounds	Imidazopyrimidines	Purines and purine derivatives	*	C04153	38.5	0	2M-H	C6H7N5O	4.854595644	1916.066235	2236.574907	2288.811009	3340.716848	3214.799373	3683.241876	2779.588451	2797.635634	2841.307249
6.07_463.1202m/z	463.1202278	6.07	neg	Sampangine	89826	Alkaloids and derivatives	1-azaoxoaporphines	Unclassified			38.5	0	2M-H	C15H8N2O	0.38448013	3551.728729	4234.779816	3532.501848	1004.155838	1324.202341	810.704132	3397.062894	3648.371384	3573.978952
6.07_523.2552m/z	523.2552041	6.07	neg	3β,5β-Tetrahydronorethindrone glucuronide	1055	Unclassified	Unclassified	Unclassified			38.5	0	M+FA-H	C26H38O8	0.696341829	6347.298593	7822.445296	7727.655933	9630.969812	10575.01258	8368.00298	5970.03584	6267.808263	5376.072722
6.11_290.1434m/z	290.1434399	6.113516667	neg	S-3-oxodecanoyl cysteamine	HMDB0059773	Lipids and lipid-like molecules	Fatty Acyls	Fatty acyl thioesters	*		38.5	0	M+FA-H	C12H23NO2S	1.170945206	1463.024814	1773.571344	1913.003658	2345.799168	2040.501433	2053.063339	1434.999425	1563.366837	1546.984486
6.12_299.1866m/z	299.1866298	6.123483333	neg	8-Hydroxy-4(6)-lactarene-5,14-diol	HMDB0035780	Organic oxygen compounds	Organooxygen compounds	Alcohols and polyols	*		38.5	0	M+FA-H	C15H26O3	0.913648341	2541.807756	3022.288197	2884.088577	2488.6289	2567.239153	2511.723742	1956.604588	2139.889729	2317.853793
6.12_462.1774m/z	462.1774051	6.123483333	neg	Ritodrine glucuronide	2356	Unclassified	Unclassified	Unclassified			38.5	0	M-H	C23H29NO9	0.971642872	3804.80027	4814.123197	4581.533465	13672.39363	14109.84782	14589.32321	8937.681713	9967.92465	10525.52073
6.16_163.0764m/z	163.0764033	6.161866667	neg	2-Phenylbutyric acid	HMDB0000329	Benzenoids	Benzene and substituted derivatives	Phenylpropanes	*		38.5	0	M-H	C10H12O2	-0.304227404	915.8107006	870.0592865	914.2158176	393.1624926	313.6863168	404.9595084	741.7996852	801.6533866	880.6224789
6.22_377.1335m/z	377.133534	6.215	neg	meta-O-Dealkylated flecainide	HMDB0060831	Benzenoids	Benzene and substituted derivatives	Benzoic acids and derivatives	*		38.5	0	M+FA-H	C15H19F3N2O3	1.668069138	917.1421536	578.9097499	950.437428	994.5184663	949.5741693	890.0376919	621.146343	595.7964079	431.1662603
6.23_651.3870m/z	651.3870187	6.230633333	neg	D-Phosphatidylglycerol	4194	Unclassified	Unclassified	Unclassified			38.5	0	M-H2O-H	C32H63O12P	-1.27427709	16558.25583	12041.14841	15660.59224	3734.664406	3671.954221	3005.817523	10075.9414	10899.2702	10775.19384
6.23_719.3745m/z	719.3744636	6.230633333	neg	Arginyltryptophan	HMDB0028720	Organic acids and derivatives	Carboxylic acids and derivatives	Amino acids, peptides, and analogues	*		38.5	0	2M-H	C17H24N6O3	-0.329365253	2899.443225	1996.98251	3093.28978	0.001403199	223.5166487	361.197597	1763.988058	1423.415955	1201.498179
6.36_289.1659m/z	289.1659403	6.3617	neg	(±)-1,4-Nonanediol diacetate	HMDB0037184	Lipids and lipid-like molecules	Fatty Acyls	Fatty alcohol esters	*		38.5	0	M+FA-H	C13H24O4	1.139405654	2232.623901	2688.21623	2772.560374	4610.55555	4628.629073	4582.087906	1572.297664	1634.43562	1727.99098
6.43_422.1825m/z	422.1825181	6.433933333	neg	Neopetasitenine	HMDB0030329	Organoheterocyclic compounds	Azaspirodecane derivatives	Unclassified	*		38.5	0	M-H	C21H29NO8	1.128670856	6643.043012	6238.914671	6526.688465	6118.487515	5422.567116	5390.6209	8358.662249	7064.144632	7589.824464
6.43_461.1075m/z	461.1075123	6.433933333	neg	Trovafloxacin	66709	Organoheterocyclic compounds	Diazanaphthalenes	Naphthyridines		C07664	38.5	0	M+FA-H	C20H15F3N4O3	-0.758324726	10078.42558	9425.462413	9374.515739	874.3924622	1131.185581	957.3296058	3513.461484	3748.481228	3419.586294
6.43_473.1815m/z	473.1815367	6.433933333	neg	1-Isomangostin hydrate	HMDB0029984	Organoheterocyclic compounds	Benzopyrans	1-benzopyrans	*		38.5	0	M+FA-H	C24H28O7	-0.395524654	11521.16536	12113.06416	12338.80682	5802.832451	5852.193976	6156.267554	12301.18733	11164.4416	12053.76288
6.54_467.1924m/z	467.1923958	6.539816667	neg	Valtratum	LMPR0102070015	Lipids and lipid-like molecules	Prenol lipids	Monoterpenoids	*	C09801	38.5	0	M+FA-H	C22H30O8	0.296188829	6886.805948	7074.727508	7326.289721	8431.480965	9002.507519	8951.223781	8357.567291	8819.484325	7985.477493
6.60_302.1036m/z	302.1036465	6.6024	neg	Ismine	69348	Unclassified	Unclassified	Unclassified			38.5	0	M+FA-H	C15H15NO3	0.973576575	1254.570138	1353.671513	1304.679955	979.7582114	954.9787719	1010.993001	1234.956647	1590.323922	1411.151269
6.71_341.1028m/z	341.102822	6.70695	neg	Calophyllin B	68098	Organoheterocyclic compounds	Benzopyrans	1-benzopyrans	*	C10054	38.5	0	M+FA-H	C18H16O4	-0.809959849	1501.684211	1658.467228	1893.337836	1706.608116	2007.652448	1742.118621	74.71729607	233.2785015	116.8499037
6.83_137.0972m/z	137.0971641	6.825	neg	4-Acetyl-1-methylcyclohexene	89268	Organic oxygen compounds	Organooxygen compounds	Carbonyl compounds			38.5	0	M-H	C9H14O	-0.177662817	551.5469326	564.7904539	521.2082852	356.0937332	417.5466472	439.5438297	569.2381377	512.4899336	388.7743942
6.84_297.1709m/z	297.1709044	6.842166667	neg	alpha-Carissanol	HMDB0035597	Lipids and lipid-like molecules	Prenol lipids	Sesquiterpenoids	*		38.5	0	M+FA-H	C15H24O3	0.622132324	1009.524314	977.7029773	807.3506761	1102.013055	1616.295545	1151.471534	812.9354485	610.7602692	671.3586135
6.88_249.1127m/z	249.1126808	6.8759	neg	alpha,alpha-Dimethylanisalacetone	HMDB0030904	Benzenoids	Phenol ethers	Anisoles	*		38.5	0	M+FA-H	C13H16O2	-2.703641502	4859.402889	5454.477368	4324.686026	4980.106837	5905.872141	4967.524809	3018.590573	3521.687307	3577.800187
6.88_462.2866m/z	462.2865911	6.884583333	neg	Latanoprost ethyl amide	45716	Unclassified	Unclassified	Unclassified			38.5	0	M+FA-H	C25H39NO4	1.149127691	7412.336136	7952.844841	7135.036673	3139.8333	3682.963955	2775.315074	5128.726288	5832.997717	5374.000211
6.88_473.2032m/z	473.2031525	6.8759	neg	Taraxacolide 1-O-b-D-glucopyranoside	HMDB0035610	Lipids and lipid-like molecules	Prenol lipids	Terpene lactones	*		38.5	0	M+FA-H	C21H32O9	0.740391368	14841.58593	16361.89625	13454.40149	9042.3772	10574.40168	9211.290321	9760.80262	12528.36881	11594.82587
6.91_411.2391m/z	411.2391181	6.9072	neg	Cortolone	63340	Lipids and lipid-like molecules	Steroids and steroid derivatives	Hydroxysteroids	**	C05481	38.5	0	M+FA-H	C21H34O5	0.794630631	4779.93454	5760.638344	4953.603768	6379.651812	5881.997341	5170.954887	3773.905279	4990.830583	4826.995395
6.92_211.1340m/z	211.1340262	6.915966667	neg	Limonene aldehyde	HMDB0030252	Lipids and lipid-like molecules	Prenol lipids	Monoterpenoids	*		38.5	0	M+FA-H	C11H18O	0.350103849	551.7193522	644.1973051	596.4336377	1012.207091	946.2644314	829.0738569	332.4522866	566.5479426	497.219617
7.00_222.9782m/z	222.9781593	7.0023	neg	4-thiodimethylarsenobutanoic acid	LMFA00000041	Lipids and lipid-like molecules	Fatty Acyls	Other Fatty Acyls			38.5	0	M-H	C6H13AsO2S	0.953327481	20994.13385	19120.91629	18774.63167	20332.43649	19775.25913	20670.21241	24123.53095	21004.6706	21540.41457
7.02_285.1710m/z	285.1709887	7.018266667	neg	(1R,2R)-3-oxo-2-pentyl-cyclopentanebutanoic acid	LMFA02010017	Lipids and lipid-like molecules	Fatty Acyls	Octadecanoids			38.5	0	M+FA-H	C14H24O3	1.004214489	1374.139938	1509.060191	1431.470377	1779.115902	1604.439132	1547.591497	1135.216064	1287.43732	1222.902795
7.18_435.1462m/z	435.1461674	7.178516667	neg	Chidamide	64835	Unclassified	Unclassified	Unclassified			38.5	0	M+FA-H	C22H19FN4O2	-3.177056297	78610.02353	88758.02375	87826.99679	101110.1099	100824.1446	104699.6515	79368.273	94927.71655	86587.46679
7.20_259.1551m/z	259.1550871	7.201166667	neg	4-keto lauric acid	35733	Lipids and lipid-like molecules	Fatty Acyls	Fatty Acids and Conjugates			38.5	0	M+FA-H	C12H22O3	-0.048405296	1068.99237	1103.65244	1089.547538	1435.517184	1463.594882	1447.981552	1111.47792	1266.906945	954.9697158
7.32_182.1308n	227.1290954	7.324733333	neg	2,2,7,7-Tetramethyl-1,6-dioxaspiro[4.4]non-3-ene	86658	Organic oxygen compounds	Organooxygen compounds	Ethers	*		38.5	0	M-H, M+FA-H	C11H18O2	0.669752221	4201.57116	4899.386685	4453.96649	4744.15577	4362.018766	4337.657157	3354.776653	3380.438293	3706.349762
7.34_507.1829m/z	507.182938	7.33585	neg	3,6-Dimethoxy-4-phenanthrenol	86425	Benzenoids	Phenanthrenes and derivatives	Phenanthrols	*		38.5	0	2M-H	C16H14O3	3.199238055	3865.17824	4546.366987	3981.77352	5139.772219	4481.717775	4997.495231	3315.507118	3778.117581	4126.095386
7.40_399.2004m/z	399.2003923	7.404266667	neg	(±)-Camphoric acid	89970	Organic acids and derivatives	Carboxylic acids and derivatives	Dicarboxylic acids and derivatives	*		38.5	0	2M-H	C10H16O4	-5.120512161	2761.79506	3079.348416	2654.166842	2202.579304	2087.15884	1651.892236	2267.102743	2611.591955	2294.043011
7.47_201.1135m/z	201.1134585	7.473983333	neg	6-Butyltetrahydro-2H-pyran-2-one	87730	Organoheterocyclic compounds	Lactones	Delta valerolactones	*		38.5	0	M+FA-H	C9H16O2	1.446955825	1354.653133	1392.962907	1436.493501	1366.667018	1688.642249	1527.246588	1249.085945	1331.966342	1386.062152
7.47_508.1100m/z	508.1100398	7.473983333	neg	Carboxyltolmetin Glucuronide	2875	Unclassified	Unclassified	Unclassified			38.5	0	M+FA-H	C21H21NO11	0.812908448	5558.564882	5020.641212	4910.558241	4727.297688	5056.458748	4040.5803	5606.441457	5134.759308	5776.320198
7.49_142.0510m/z	142.0509582	7.4852	neg	L-Glutamate methylester	66284	Unclassified	Unclassified	Unclassified			38.5	0	M-H2O-H	C6H11NO4	-0.052665553	300.7589859	319.4059693	346.2716492	211.2248138	356.7541987	357.9610599	403.1575308	268.3142284	355.7947452
7.51_413.1433m/z	413.1433018	7.50615	neg	Zizybeoside I	HMDB0034954	Organic oxygen compounds	Organooxygen compounds	Carbohydrates and carbohydrate conjugates			38.5	2.88	M-H2O-H	C19H28O11	-4.671438849	62199.54679	63169.68333	65287.45459	75698.38124	80972.56194	70189.19965	59529.57923	66344.94782	67877.64866
7.66_183.0664m/z	183.0663739	7.659433333	neg	Genipic acid	91245	Organoheterocyclic compounds	Dihydrofurans	Unclassified	*		38.5	0	M-H	C9H12O4	0.497084759	556.8434499	627.4854329	526.9749306	471.822669	429.5482297	473.075199	399.0299112	336.8279066	576.9479496
7.66_404.2566n	403.249283	7.659433333	neg	Lucidone C	HMDB0038152	Lipids and lipid-like molecules	Steroids and steroid derivatives	Oxosteroids	*		38.5	0	M-H, M+FA-H	C24H36O5	0.705468355	11580.36667	13259.30472	13314.73089	11416.98803	10730.98136	11052.15504	12476.02978	13269.24329	14052.86817
7.72_448.3073m/z	448.3072553	7.719916667	neg	N-Palmitoyl phenylalanine	HMDB0062339	Organic acids and derivatives	Carboxylic acids and derivatives	Amino acids, peptides, and analogues			38.5	0	M+FA-H	C25H41NO3	1.012291268	7323.46685	8100.340669	8507.107549	1810.272033	1643.238309	1886.35554	6191.968788	6996.972017	7248.567466
7.74_341.1972m/z	341.197192	7.738233333	neg	(1(10)E,4a,5E)-1(10),5-Germacradiene-12-acetoxy-4,11-diol	HMDB0038798	Lipids and lipid-like molecules	Prenol lipids	Sesquiterpenoids	*		38.5	0	M+FA-H	C17H28O4	0.775629711	3795.937471	4205.073004	3789.00197	7732.665726	7752.401477	6992.908087	2473.899821	2765.257829	2752.415067
7.80_183.1391m/z	183.1391317	7.802066667	neg	(S)-3,7-Dimethyl-1,6-octadiene	HMDB0061795	Lipids and lipid-like molecules	Prenol lipids	Monoterpenoids	*		38.5	0	M+FA-H	C10H18	0.566309776	854.7791341	802.308692	973.5866104	1154.909189	1104.265527	1152.446745	880.1179105	780.3392111	798.3864471
7.93_509.2370m/z	509.2370176	7.9268	neg	HC Blue No.1	72968	Unclassified	Unclassified	Unclassified			38.5	0	2M-H	C11H17N3O4	0.944618576	11061.58926	10292.85045	10521.25857	14342.08593	13424.16099	13552.8151	9740.826196	13750.02837	12012.31067

8.07_677.2149m/z	677.2148646	8.074883333	neg	Rutagravine	89278	Organoheterocyclic compounds	Quinolines and derivatives	Benzoquinolines			38.5	0	2M-H	C19H17NO5	1.173302103	22410.97671	26602.27619	23375.76562	3862.83781	3529.961485	3597.684663	11280.37135	13938.06106	12836.90766
8.62_223.0285m/z	223.0284642	8.6174	neg	3,3'-Thiobispropanoic acid	87455	Organic acids and derivatives	Carboxylic acids and derivatives	Dicarboxylic acids and derivatives	*		38.5	0	M+FA-H	C6H10O4S	1.581373799	1154.801536	951.7996716	961.1394407	823.522879	829.0669138	975.8231563	1243.877537	944.0704907	936.1769195
8.79_507.1074m/z	507.1073617	8.791166667	neg	5,7-Dihydroxyflavone	HMDB0036619	Phenylpropanoids and polyketides	Flavonoids	Flavones	***	C10028	38.5	0	2M-H	C15H10O4	-2.321247924	2504.563843	2618.593315	2606.33266	0.001403199	0.001403199	0.001403199	1857.810221	2092.748609	2474.220495
8.95_406.2720n	405.2647708	8.953233333	neg	3,7-Dihydroxy-12-oxocholanoic acid	5389	Lipids and lipid-like molecules	Steroids and steroid derivatives	Bile acids, alcohols and derivatives	*		38.5	0	M-H, M+FA-H	C24H38O5	0.302543695	12935.55725	13205.74182	16766.8906	1547.461816	1840.643985	1783.766944	6007.737745	6446.905543	7902.527812
9.02_297.1707m/z	297.1707286	9.01635	neg	6alpha-Carissanol	HMDB0035309	Lipids and lipid-like molecules	Prenol lipids	Sesquiterpenoids	*		38.5	0	M+FA-H	C15H24O3	-0.075087257	671.6923158	775.4434697	498.865767	1144.384345	918.5762352	817.7656933	313.1661884	399.2528801	377.5578367
9.02_409.2868m/z	409.2867743	9.01635	neg	3-Ethylethcathinone	96613	Unclassified	Unclassified	Unclassified			38.5	0	2M-H	C13H19NO	1.760441125	8019.245933	10499.29141	10084.34441	629.2734001	674.1884482	654.4969314	3815.269987	4329.82991	4085.804807
9.08_441.1746m/z	441.1746266	9.077466667	neg	2'-Deoxysepiapterin	HMDB0000389	Organoheterocyclic compounds	Pteridines and derivatives	Pterins and derivatives	*		38.5	0	2M-H	C9H11N5O2	-1.461354412	8585.098809	7858.773203	8706.947829	5856.429457	5074.294992	5431.088958	4719.737337	4717.247655	5829.172355
9.66_211.0361m/z	211.0361432	9.662666667	neg	N-phosphocreatinate(2-)	HMDB0062567	Organic acids and derivatives	Carboxylic acids and derivatives	Amino acids, peptides, and analogues			38.5	0	M-H	C4H11N3O5P+	-1.001915684	911.8082753	811.7308762	737.8248455	853.9944949	1040.066083	827.7601839	1105.364224	886.5817566	951.5616053
0.67_307.1514m/z	307.1513881	0.6701	neg	Fructoselysine	71227	Unclassified	Unclassified	Unclassified			38.4	0	M-H	C12H24N2O7	1.017162284	1991.957348	1911.277614	1942.560484	3053.557954	2992.60959	3388.700505	1894.404819	1936.340032	2064.934376
0.76_298.1147m/z	298.1147112	0.757316667	neg	Penciclovir	HMDB0014444	Organoheterocyclic compounds	Imidazopyrimidines	Purines and purine derivatives		C07417	38.4	0	M+FA-H	C10H15N5O3	-3.87561606	600.7587819	216.3076255	0.001403199	656.637708	0.001403199	1225.386755	0.001403199	224.6443371	0.001403199
0.80_155.0020m/z	155.0019752	0.800366667	neg	METHYLMETHANE SULFONATE	44135	Unclassified	Unclassified	Unclassified			38.4	0	M+FA-H	C2H6O3S	0.06650584	1432.799857	1618.921395	1402.154014	227.7954096	93.8957605	0.001403199	0.001403199	0.001403199	0.001403199
0.82_331.0456m/z	331.0455734	0.819566667	neg	4-Hydroxyphenylglyoxylate	63853	Unclassified	Unclassified	Unclassified			38.4	0	2M-H	C8H6O4	-1.106880019	8816.186497	11060.5046	10796.59691	11768.60656	10321.21597	11977.64087	11320.31278	11772.21089	12303.08979
0.86_423.0814m/z	423.0813682	0.86335	neg	7-Methylguanosine 5'-phosphate	HMDB0059612	Organic acids and derivatives	Carboxylic acids and derivatives	Amino acids, peptides, and analogues		C03998	38.4	1.07	M+FA-H	C11H17N5O8P+	4.47296083	31047.44039	33867.70034	38137.9189	37352.36225	36955.33084	37007.2217	40662.11921	37352.39299	39077.92489
0.90_153.0306m/z	153.0306074	0.904683333	neg	2-Amino-4-nitrophenol	HMDB0244990	Unclassified	Unclassified	Unclassified			38.4	0	M-H	C6H6N2O3	0.271467111	4771.803008	4488.356133	5085.317218	4305.29668	4006.667349	4325.555828	4699.449984	4340.685197	4674.306632
0.90_154.9988m/z	154.998803	0.904683333	neg	trans-Aconitate	3771	Organic acids and derivatives	Carboxylic acids and derivatives	Tricarboxylic acids and derivatives	**	C02341	38.4	0	M-H2O-H	C6H6O6	1.184774541	1216.928665	1679.463775	2052.658146	3261.469439	1497.787712	2681.947259	1696.070794	1700.540343	989.6247591
0.90_337.0778m/z	337.0777726	0.904683333	neg	3-O-beta-D-Galactopyranuronosyl-D-galactose	HMDB0039726	Organic oxygen compounds	Organooxygen compounds	Carbohydrates and carbohydrate conjugates			38.4	0	M-H2O-H	C12H20O12	0.386478172	3072.138654	2933.896811	2933.736873	504.0743119	0.001403199	519.074857	1247.831715	658.1541748	0.001403199
1.08_159.0664m/z	159.0663964	1.078266667	neg	4-Methyl-2-pentenoic acid	LMFA01030985	Lipids and lipid-like molecules	Fatty Acyls	Fatty acids and conjugates	*		38.4	0	M+FA-H	C6H10O2	0.999549389	528.2472897	377.3652405	302.3356737	317.2735018	473.911653	484.4490444	561.9716767	350.7311379	520.8960673
1.14_294.9936m/z	294.9936495	1.1382	neg	Dihydroxyfumarate	6460	Organic acids and derivatives	Keto acids and derivatives	Short-chain keto acids and derivatives	**	C00975	38.4	0	2M-H	C4H4O6	-2.195144878	677.5503303	833.4697131	751.2645972	631.1795202	833.8029135	922.7527938	634.0593746	1040.012819	1004.546735
1.34_215.0175m/z	215.0174865	1.335933333	neg	Sodium alginate	HMDB0303715	Organic oxygen compounds	Organooxygen compounds	Carbohydrates and carbohydrate conjugates	**	C01768	38.4	0	M-H	C6H9NaO7	0.768507621	1382.816571	1427.182926	1563.838976	1057.553514	1217.952531	1068.51397	974.3911782	1159.853945	1062.804956
1.62_188.9865m/z	188.9864721	1.615883333	neg	4-Sulfocatechol	66486	Unclassified	Unclassified	Unclassified			38.4	0	M-H	C6H6O5S	0.811732391	14370.0411	14410.78279	15483.2374	7440.071053	7112.850502	7239.379065	13221.86872	12821.86398	12972.28589
1.80_250.0756m/z	250.0756245	1.798266667	neg	N-Propionylmethionine	HMDB0094704	Organic acids and derivatives	Carboxylic acids and derivatives	Amino acids, peptides, and analogues			38.4	0	M+FA-H	C8H15NO3S	0.767127362	2021.188036	1923.336574	2035.595213	1735.97862	1905.477977	2006.384358	2041.699656	2128.722722	2017.971119
10.76_269.2123m/z	269.2123271	10.76333333	neg	(+/-)-Dihydrofarnesol	HMDB0032218	Lipids and lipid-like molecules	Prenol lipids	Sesquiterpenoids	*		38.4	0	M+FA-H	C15H28O	0.48470864	2001.850067	2735.77721	1342.349602	1602.200884	1355.877003	1524.05973	1971.303061	1455.712502	1400.565698
10.99_269.2123m/z	269.2123275	10.98771667	neg	5,10-Pentadecadien-1-ol	HMDB0031111	Lipids and lipid-like molecules	Fatty Acyls	Fatty alcohols	*		38.4	0	M+FA-H	C15H28O	0.486571798	3198.776364	3402.600155	2986.720513	2825.005148	2846.21901	2743.913731	3115.30331	3462.821921	3384.922416
2.19_209.0569m/z	209.0568934	2.194566667	neg	5-aminosalicyluric acid	1147	Unclassified	Unclassified	Unclassified			38.4	0	M-H	C9H10N2O4	0.538092243	1480.40852	1341.397931	1645.060513	2648.511867	2313.932543	2552.25059	1412.145144	1592.311099	1538.305764
2.24_168.0303m/z	168.0303021	2.236416667	neg	Nicotinic acid	HMDB0001488	Organoheterocyclic compounds	Pyridines and derivatives	Pyridinecarboxylic acids and derivatives	***	C00253	38.4	0	M+FA-H	C6H5NO2	0.575374829	904.8276683	740.3430792	791.3202684	544.2793029	624.0751159	463.0586824	290.1632308	375.2059302	434.5892683
2.28_270.1098m/z	270.1097676	2.2828	neg	4-(Methylnitrosamino)-1-(3-pyridyl-N-oxide)-1-butanol	73258	Organoheterocyclic compounds	Pyridines and derivatives	Pyridinium derivatives	**	C19603	38.4	0	M+FA-H	C10H15N3O3	0.992502744	894.8129124	850.2056637	694.6246029	982.8305256	986.2041913	983.0829938	729.2255759	873.0971238	852.1784253
2.39_109.0293m/z	109.029318	2.390833333	neg	3-Hydroxy-4,5-dimethyl-2(5H)-furanone	HMDB0031306	Organoheterocyclic compounds	Dihydrofurans	Furanones	*		38.4	0	M-H2O-H	C6H8O3	-1.444739397	126.5964247	102.0100204	130.160183	24.2352165	34.77590991	55.83434331	238.4251141	328.0082296	250.596845
2.67_499.0710m/z	499.0710046	2.6698	neg	SAICAR	HMDB0000797	Nucleosides, nucleotides, and analogues	Imidazole ribonucleosides and ribonucleotides	1-ribosyl-imidazolecarboxamides	**	C04823	38.4	0	M+FA-H	C13H19N4O12P	-1.997440264	1431.725762	1512.371959	1656.456085	0.001403199	0.001403199	0.001403199	1254.486998	1541.136536	1150.247899
2.78_303.0725m/z	303.0724728	2.776216667	neg	Isosorbide-2-glucuronide	864	Unclassified	Unclassified	Unclassified			38.4	0	M-H2O-H	C12H18O10	0.984774746	4624.232628	4196.960615	4474.150436	2881.492859	2434.538082	2734.76362	2844.718561	3420.357723	2989.166137
3.44_150.0560m/z	150.0560461	3.440016667	neg	5-Hydroxydopamine	HMDB0004817	Benzenoids	Phenols	Benzenediols	*		38.4	0	M-H2O-H	C8H11NO3	-0.035504585	1549.984956	1932.935727	1947.610313	1125.690984	1056.267788	1040.845743	1906.838554	1969.302725	1721.894439
3.60_449.0681m/z	449.068106	3.601516667	neg	Dinitolmide	88528	Benzenoids	Benzene and substituted derivatives	Toluenes	*		38.4	0	2M-H	C8H7N3O5	-3.906433692	1114.773522	1506.939862	1209.345959	915.0438604	990.3081153	1289.29316	914.155951	1322.434214	1385.921092
3.73_569.0118m/z	569.0117771	3.730516667	neg	trans-zeatin riboside triphosphate	HMDB0304509	Unclassified	Unclassified	Unclassified			38.4	0	M-H2O-H	C15H21N5O14P3-3	-0.284069626	5290.324817	4861.509274	5042.663159	1318.272894	1435.97579	1277.706594	3736.776595	3357.486306	4143.688627
3.75_333.0832m/z	333.0831601	3.747833333	neg	8-hydroxy Guanine	34520	Unclassified	Unclassified	Unclassified			38.4	0	2M-H	C5H5N5O2	5.350888917	283.4116611	285.6951776	246.7657575	1013.851646	909.9841252	476.7978709	425.7317199	494.1699982	525.2274323
3.75_448.1721n	895.3294761	3.747833333	neg	2,3,4,5,2',3',4',6'-Octamethoxychalcone	52155	Unclassified	Unclassified	Unclassified			38.4	0	M-H2O-H, 2M-H	C23H28O9	-2.720802617	8224.667178	7134.267282	8062.428026	12627.12547	12236.77886	7485.601702	6998.821323	9344.231846	7277.143787
3.82_349.0995m/z	349.0995376	3.82415	neg	Vicine	66974	Organic oxygen compounds	Organooxygen compounds	Carbohydrates and carbohydrate conjugates		C08437	38.4	0	M+FA-H	C10H16N4O7	-1.855216386	3043.169749	3666.390829	3385.698947	4586.87992	4578.67296	4496.786916	4007.084476	4372.909662	4646.506469
3.89_378.0243m/z	378.0242862	3.8906	neg	beta-nicotinate D-ribonucleotide	HMDB0304543	Nucleosides, nucleotides, and analogues	Pyridine nucleotides	Nicotinic acid nucleotides	**	C01185	38.4	0	M+FA-H	C11H12NO9P-2	3.350328663	1016.837426	823.508667	1160.458766	287.0525138	248.5860315	321.0995682	331.4327605	388.0892969	249.1942073
3.91_467.1196m/z	467.1196161	3.912566667	neg	Rumexoside	91540	Organic oxygen compounds	Organooxygen compounds	Carbohydrates and carbohydrate conjugates			38.4	0	M+FA-H	C20H22O10	0.27568301	1506.964665	1354.6429	1612.387022	0.001403199	0.001403199	186.4933298	2419.259989	2394.71576	2527.493439
4.02_467.1192m/z	467.1192498	4.024283333	neg	1,8-Naphthyridine-3-carboxylic acid, 1-ethyl-1,4-dihydro-7-hydroxy-4-oxo-	1413	Unclassified	Unclassified	Unclassified			38.4	0	2M-H	C11H10N2O4	-3.390853273	585.1324972	637.4656124	340.747485	74.34347339	0.001403199	0.001403199	911.9314496	1033.856094	800.7362035
4.07_125.0608m/z	125.0607644	4.069216667	neg	Toluene-cis-dihydrodiol	66219	Unclassified	Unclassified	Unclassified			38.4	0	M-H	C7H10O2	-0.306970798	187.4706571	153.7514859	175.8598815	56.29800423	57.89939425	96.61143001	124.3814889	171.9505813	128.3763207
4.11_343.1036m/z	343.1036439	4.113883333	neg	Thalassemine	65686	Unclassified	Unclassified	Unclassified			38.4	0	M+FA-H	C8H19N4O6P	4.092729013	1024.386601	793.4452052	1061.072418	1804.851042	1636.468248	1678.079128	1165.33528	993.5008773	966.7379641
4.11_407.2191m/z	407.2190723	4.113883333	neg	Triethylenemelamine	HMDB0259195	Organoheterocyclic compounds	Triazines	1,3,5-triazines		C08737	38.4	0	2M-H	C9H12N6	4.066269904	890.1829072	1433.639362	1464.683173	1112.285097	1128.295164	890.4198361	969.0409204	1094.36491	1296.937201
4.19_123.0087m/z	123.0086906	4.189783333	neg	Cis,trans-muconate	HMDB0062661	Lipids and lipid-like molecules	Fatty Acyls	Fatty acids and conjugates	*		38.4	0	M-H2O-H	C6H6O4	-0.542040522	298.8313763	291.1610609	290.7595019	128.8580924	116.1132297	132.3268455	365.422171	310.8515544	295.2646617
4.19_127.0765m/z	127.0764572	4.189783333	neg	4-Pentenyl acetate	88478	Organic acids and derivatives	Carboxylic acids and derivatives	Carboxylic acid derivatives	*		38.4	0	M-H	C7H12O2	0.03091581	198.9681023	171.8831251	202.8722025	184.1651087	196.245262	289.5395066	250.3602011	203.9085713	193.2398934
4.21_344.0789m/z	344.0789476	4.212116667	neg	Avenanthramide 1c	86155	Phenylpropanoids and polyketides	Cinnamic acids and derivatives	Hydroxycinnamic acids and derivatives	*		38.4	0	M+FA-H	C16H13NO5	4.588011477	84.03242968	321.1600993	420.4805646	653.4627642	767.0500054	873.8164604	542.9445224	441.7721192	537.7924821
4.34_507.9681m/z	507.9680521	4.344733333	neg	Sulfluramid	HMDB0258587	Unclassified	Unclassified	Unclassified			38.4	0	M-H2O-H	C10H6F17NO2S	2.114015392	1139.777514	1236.223617	1182.49016	1755.20665	1493.158195	1488.924861	1290.193605	1824.305199	1400.661783
4.41_435.1412m/z	435.1412344	4.41115	neg	1-Hydroxypyrene	64943	Benzenoids	Pyrenes	Unclassified	*	C14519	38.4	0	2M-H	C16H10O	5.000482448	374.8885375	566.9868061	0.001403199	903.5318675	462.0199567	1215.908771	370.8237764	659.3944663	297.6463688
4.43_441.1039m/z	441.1039257	4.432683333	neg	L-Histidinol phosphate	3355	Unclassified	Unclassified	Unclassified			38.4	0	2M-H	C6H12N3O4P	-4.258788561	3501.395311	4067.34705	3540.842242	311.2379141	562.4099924	385.0316878	0.001403199	267.2463303	222.0749909
4.48_241.0699m/z	241.0698532	4.477166667	neg	Glutamyl-Asparagine	85728	Organic acids and derivatives	Carboxylic acids and derivatives	Amino acids, peptides, and analogues	*		38.4	0	M-H2O-H	C9H14N3O6-	-2.175464083	400.5168818	224.2028444	187.666699	236.3807143	0.001403199	260.7006388	406.7501078	448.0169535	458.3141604
4.52_292.1303m/z	292.1302631	4.521733333	neg	Glutaminylphenylalanine	HMDB0028804	Organic acids and derivatives	Carboxylic acids and derivatives	Amino acids, peptides, and analogues	*		38.4	0	M-H	C14H19N3O4	-0.056489919	469.8947408	429.6962505	401.2906132	425.5745741	487.2610379	442.2985349	548.6068874	704.9518427	804.3886878
4.63_309.0982m/z	309.0982408	4.632666667	neg	1'-Acetoxyeugenol acetate	HMDB0036544	Benzenoids	Phenol esters	Unclassified			38.4	0	M+FA-H	C14H16O5	1.000950656	1114.865193	1064.88778	1072.723445	1497.001757	1254.695454	1282.635979	1629.840456	1469.325576	1291.750945
4.63_327.1089m/z	327.108873	4.632666667	neg	Flutrimazole	HMDB0252420	Unclassified	Unclassified	Unclassified			38.4	0	M-H2O-H	C22H16F2N2	-4.162883734	1833.852259	1853.787212	1851.188704	1130.236844	1204.141645	1194.114107	1840.040833	2333.436795	2125.711324
4.66_485.0631m/z	485.0631142	4.660483333	neg	2-(Methylthio)-3H-phenoxazin-3-one	HMDB0035996	Organoheterocyclic compounds	Benzoxazines	Phenoxazines			38.4	0	2M-H	C13H9NO2S	-0.840181942	3038.092002	3066.814492	3256.288475	7573.115156	6493.230188	7761.725127	4481.544663	5031.34776	3084.967146
4.68_123.0451m/z	123.0451241	4.682483333	neg	Guaiacol	HMDB0001398	Benzenoids	Phenols	Methoxyphenols	*	C15572	38.4	0	M-H	C7H8O2	-0.233156911	115.3179631	82.7283272	119.8663131	41.77721078	26.70613026	0.001403199	48.73872453	42.23167278	24.60904658
4.68_499.0864m/z	499.0863574	4.682483333	neg	2-Hydroxy-6-oxo-6-(2-hydroxyphenoxy)-hexa-2,4-dienoate	66735	Unclassified	Unclassified	Unclassified			38.4	0	2M-H	C12H10O6	-3.683839598	1708.675443	1905.846677	1678.951239	0.001403199	0.001403199	0.001403199	0.001403199	0.001403199	0.001403199
4.73_227.0497m/z	227.0496629	4.725416667	neg	Ethionamide sulfoxide	2594	Organoheterocyclic compounds	Pyridines and derivatives	Unclassified	*		38.4	0	M+FA-H	C8H10N2OS	0.417645619	399.0973073	448.5066878	392.3313666	835.0957609	849.5708555	728.7925252	956.4268602	1006.944501	966.9377107
4.79_524.1178m/z	524.1177687	4.792316667	neg	Pulchellidin 3-glucoside	LMPK12010413	Unclassified	Unclassified	Unclassified			38.4	0	M+FA-H	C22H23O12+	1.282963991	442.8093053	167.5268558	284.0649834	1030.348545	640.6789644	629.9967554	213.086518	657.08969	0.001403199
4.88_472.2373m/z	472.2372509	4.880083333	neg	20-hydroxy N-Arachidonoyl Taurine	45183	Unclassified	Unclassified	Unclassified			38.4	0	M+FA-H	C22H37NO5S	-0.459310542	5131.614692	5321.657531	5425.308327	4093.354664	4432.411724	4646.096411	5922.167057	6295.569647	4732.389216
4.92_443.1560m/z	443.1560017	4.92485	neg	Methyl 3,4-dihydroxy-5-prenylbenzoate 3-glucoside	HMDB0040121	Phenylpropanoids and polyketides	Tannins	Hydrolyzable tannins			38.4	0	M+FA-H	C19H26O9	0.292338501	5194.75213	6257.276185	5772.251974	8293.558604	8264.821092	8624.933898	6776.395569	7843.594868	6663.801064
5.04_441.1776m/z	441.1776303	5.037216667	neg	2-Deoxysepiapterin	5378	Unclassified	Unclassified	Unclassified			38.4	0	2M-H	C9H11N5O2	5.331505799	1199.986952	753.2091365	1182.46848	369.2591807	541.978785	477.2649153	528.2020312	1145.452214	685.3519411
5.08_313.1660m/z	313.166023	5.080566667	neg	4,11,13,15-Tetrahydroridentin B	HMDB0036150	Lipids and lipid-like molecules	Prenol lipids	Terpene lactones	*		38.4	0	M+FA-H	C15H24O4	1.345592859	9648.953407	10711.94148	10364.23174	11556.57011	11480.37822	12424.02292	9049.783862	7835.799075	8657.121864
5.08_411.2027m/z	411.2026905	5.080566667	neg	12-oxo-20-dihydroxy-leukotriene B4	LMFA03020048	Lipids and lipid-like molecules	Fatty Acyls	Eicosanoids	*		38.4	0	M+FA-H	C20H30O6	0.679769557	2153.930921	2357.397461	2196.903604	1499.894704	1219.515148	1566.986705	2837.427226	2587.474685	2432.195382
5.08_535.1461m/z	535.1461477	5.080566667	neg	7-Hydroxy-2',4',5'-trimethoxyisoflavone 7-O-glucoside	LMPK12050064	Unclassified	Unclassified	Unclassified			38.4	0	M+FA-H	C24H26O11	0.88386913	12960.03032	13062.37198	13147.27964	851.1156634	734.7610743	628.2462622	4920.993745	4981.44133	5180.145367
5.18_577.1819m/z	577.18192	5.1777	neg	Acronycidine	68446	Unclassified	Unclassified	Unclassified			38.4	0	2M-H	C15H15NO5	-1.46790935	9478.692472	9556.35176	9945.22116	6184.392596	5181.79264	5130.877021	10116.90377	7879.552414	9182.534738
5.24_353.0882m/z	353.0882071	5.244033333	neg	Allamandin	67925	Unclassified	Unclassified	Unclassified			38.4	0	M+FA-H	C15H16O7	1.302644869	4055.099289	4206.570028	3857.648523	2402.652384	1969.496925	2180.23505	3557.96959	3469.491638	3746.762936
5.26_273.1344m/z	273.1344398	5.263033333	neg	(-)-11-hydroxy-9,10-dihydrojasmonic acid	45942	Lipids and lipid-like molecules	Fatty Acyls	Octadecanoids			38.4	0	M+FA-H	C12H20O4	0.340855286	1116.853054	827.2928221	878.8710214	1134.702907	942.9734984	1117.306035	696.671696	668.0560405	883.729865
5.30_263.0902m/z	263.0901549	5.302883333	neg	CAY10574	45317	Unclassified	Unclassified	Unclassified			38.4	0	M+FA-H	C9H10N6O	1.57313781	2661.144853	2680.428668	2529.213763	4823.38626	5535.668262	5863.188593	3226.322046	3074.895293	3624.556697
5.34_224.1294m/z	224.1293994	5.3394	neg	3,4-Dimethyl-2-(1-pyrrolidinyl)-2-cyclopenten-1-one	94232	Organoheterocyclic compounds	Pyrrolidines	Unclassified	*		38.4	0	M+FA-H	C11H17NO	1.017910804	530.6559012	674.7932602	605.376416	740.0784336	965.3170966	845.7452927	836.6174047	828.8903776	997.0817505
5.38_347.2077m/z	347.2077014	5.384216667	neg	xi-7-Hydroxyhexadecanedioic acid	HMDB0037830	Lipids and lipid-like molecules	Fatty Acyls	Fatty acids and conjugates	*		38.4	0	M+FA-H	C16H30O5	0.57718996	9816.855832	8687.463987	8635.138469	5325.177954	5397.172572	5436.938257	7873.887369	7063.442888	7887.40273
5.43_537.2344m/z	537.2344172	5.428733333	neg	4-Hydroxyretinoic acid glucuronide	HMDB0061688	Lipids and lipid-like molecules	Prenol lipids	Terpene glycosides	*		38.4	0	M+FA-H	C26H36O9	0.572068729	736.97758	996.3864811	1688.928724	1439.973029	730.4879335	1307.006586	901.5237578	1000.958992	1340.420437
5.45_355.1038m/z	355.1037662	5.451166667	neg	(E)-1-O-Cinnamoyl-beta-D-glucose	86865	Phenylpropanoids and polyketides	Cinnamic acids and derivatives	Cinnamic acid esters	*		38.4	0	M+FA-H	C15H18O7	1.001019403	3339.016861	2830.027211	2994.388738	1222.629345	1186.134268	1287.086346	3558.302463	2490.452342	2402.553545
5.53_163.0764m/z	163.076405	5.525416667	neg	Benzenebutanoic acid	5528	Benzenoids	Benzene and substituted derivatives	Unclassified	*		38.4	0	M-H	C10H12O2	-0.293424127	633.6779253	524.3529196	523.3587443	166.6519155	216.4880168	180.3669631	504.7402012	500.4510313	490.2157925
5.53_289.1659m/z	289.1658917	5.525416667	neg	2-methyl-dodecanedioic acid	LMFA01170010	Lipids and lipid-like molecules	Fatty Acyls	Fatty Acids and Conjugates			38.4	0	M+FA-H	C13H24O4	0.940345381	4263.676521	3802.174958	4083.563125	3043.870727	3336.113994	3328.804101	3731.216601	3777.867952	3822.669676
5.53_383.1713m/z	383.1712704	5.525416667	neg	Oxoamide	HMDB0001004	Organic oxygen compounds	Organooxygen compounds	Carbonyl compounds	*		38.4	0	2M-H	C10H12N2O2	-3.145473299	10013.40664	8163.303984	8532.16077	4928.587704	6663.734887	5468.062292	8438.03403	8247.071027	7599.040785
5.53_579.1494m/z	579.1493589	5.525416667	neg	Epicatechin	HMDB0001871	Phenylpropanoids and polyketides	Flavonoids	Flavans	***	C09727	38.4	0	2M-H	C15H14O6	-2.483836248	851.8081394	859.2269572	779.9984929	1529.605033	2188.354104	1270.131541	907.8068486	862.0934073	1141.638988
5.61_433.0553m/z	433.0553111	5.610733333	neg	5-Hydroxythiabendazole	HMDB0247680	Unclassified	Unclassified	Unclassified			38.4	0	2M-H	C10H7N3OS	1.432662333	805.1208071	877.1812762	1140.278828	1259.338028	934.9100946	1032.262519	919.1396698	812.6881457	1057.586889
5.65_611.2693m/z	611.2692717	5.650833333	neg	O-Isopentenylhalfordinol	86879	Organoheterocyclic compounds	Azoles	Oxazoles	*		38.4	0	2M-H	C19H18N2O2	4.724242392	3770.245529	3365.401636	5020.9043	4392.693692	4186.071881	4142.993028	4299.470709	2456.303481	4151.862173
5.70_479.1901m/z	479.1901439	5.701283333	neg	Melledonol	HMDB0035884	Lipids and lipid-like molecules	Prenol lipids	Sesquiterpenoids	*		38.4	2.66	M+FA-H	C23H30O8	-4.89848227	15010.52868	13411.42166	12962.59872	6937.160891	8523.100843	9145.745436	13711.42224	14335.28141	18664.61328
5.81_422.1799m/z	422.1799495	5.806016667	neg	Deaminohydroxyblasticidin S	66067	Unclassified	Unclassified	Unclassified			38.4	0	M-H	C17H25N7O6	1.404523469	4063.085339	4729.075448	5247.744825	13843.11414	12882.40845	11383.59295	8211.167614	7682.677764	7523.098019
5.83_530.1646m/z	530.1646045	5.825	neg	Fluoxetine glucuronide	HMDB0060607	Organic oxygen compounds	Organooxygen compounds	Carbohydrates and carbohydrate conjugates	*		38.4	0	M+FA-H	C23H26F3NO7	0.545834375	2900.513834	3518.286083	2568.89744	11671.66329	9735.833236	9285.829131	9392.302573	9615.654425	10694.38431
5.97_533.1666m/z	533.1666448	5.965483333	neg	Egonol glucoside	HMDB0034280	Phenylpropanoids and polyketides	2-arylbenzofuran flavonoids	Unclassified			38.4	0	M+FA-H	C25H28O10	0.399048066	3811.024853	4068.838868	4894.494866	0.001403199	0.001403199	0.001403199	0.001403199	0.001403199	0.001403199
6.02_242.1520n	287.1502367	6.01945	neg	2-Carboxy-4-dodecanolide	HMDB0030987	Organoheterocyclic compounds	Lactones	Gamma butyrolactones	*		38.4	0	M-H, M+FA-H	C13H22O4	0.942795154	2922.853732	2647.290792	3051.448217	4331.242206	4282.853342	4540.84321	2538.523624	2480.967712	2402.951278
6.09_427.1973m/z	427.1972814	6.087383333	neg	Cinncassiol A	HMDB0035164	Lipids and lipid-like molecules	Prenol lipids	Terpene lactones	*		38.4	0	M+FA-H	C20H30O7	-0.19565902	13941.64707	16220.17453	17960.61438	5768.536375	5795.340571	6559.354723	12454.10225	13763.0657	14163.77735
6.10_615.2041m/z	615.2040623	6.101316667	neg	(+)-Warfarin	45556	Unclassified	Unclassified	Unclassified			38.4	0	2M-H	C19H16O4	2.630121577	7112.908979	9936.084827	9341.822968	6126.469117	7789.808654	6713.54202	6640.96949	7100.105605	8243.392844
6.32_473.2157m/z	473.2157251	6.320716667	neg	PG(12:0/0:0)	79999	Lipids and lipid-like molecules	Glycerophospholipids	Glycerophosphoglycerols			38.4	0	M+FA-H	C18H37O9P	0.006661295	161.6315797	296.8949622	437.6741826	1687.657098	1898.595455	1210.109888	557.2148754	516.1185532	723.7832505
6.33_385.1694m/z	385.1693676	6.331216667	neg	2-Anthramine	70045	Unclassified	Unclassified	Unclassified			38.4	0	2M-H	C14H11N	-4.28467681	5001.266515	5719.023116	6044.169202	8352.06778	8210.796954	7509.318085	6138.236401	5910.192432	6828.99676
6.33_478.2242m/z	478.2241676	6.331216667	neg	Hericenone B	91629	Organoheterocyclic compounds	Isoindoles and derivatives	Isoindolines			38.4	0	M+FA-H	C27H31NO4	1.51492083	1064.258528	1424.854574	1427.076528	1680.972824	2006.0848	1838.422081	1269.982648	1300.411761	1299.650161
6.44_212.1414n	211.1341337	6.442716667	neg	DIHYDROJASMONIC ACID	44409	Lipids and lipid-like molecules	Fatty Acyls	Lineolic acids and derivatives	*		38.4	0	M-H, M+FA-H	C12H20O3	0.780934488	1612.415508	1663.749564	1703.239951	2353.705962	1996.344745	1932.962009	1952.602579	1891.101035	1855.072748
6.51_313.1660m/z	313.1660375	6.509966667	neg	1-(3-Furanyl)-6,7-dihydroxy-4,8-dimethyl-1-nonanone	HMDB0038153	Lipids and lipid-like molecules	Fatty Acyls	Fatty alcohols	*		38.4	0	M+FA-H	C15H24O4	1.399633953	4616.796672	4666.614413	4840.045443	7820.019554	7402.237115	7174.476987	3511.766268	3871.837101	3866.857997
6.64_464.2413n	463.2340311	6.644916667	neg	Dehydroisoandrosterone 3-glucuronide	LMST05010064	Lipids and lipid-like molecules	Steroids and steroid derivatives	Steroidal glycosides	**	C03033	38.4	0	M-H, M+FA-H	C25H36O8	0.623418667	18043.38227	20074.86042	18997.97541	25821.30654	23303.45844	23444.95801	18086.50468	21818.27207	22247.98885
6.66_441.1767m/z	441.1767369	6.663683333	neg	Pteroside P	91668	Organic oxygen compounds	Organooxygen compounds	Carbohydrates and carbohydrate conjugates			38.4	0	M+FA-H	C20H28O8	0.293205284	4379.823729	4909.312838	4766.902422	9195.044453	8074.013637	7517.006271	4008.829374	5143.537911	5862.985568
6.83_325.1658m/z	325.1658304	6.832183333	neg	3-Ethyl-2-hydroxy-4-methyl-2-cyclopenten-1-one	91325	Organic oxygen compounds	Organooxygen compounds	Carbonyl compounds	*		38.4	0	M+FA-H	C16H24O4	0.60052518	1815.649924	1648.30933	1451.508919	2963.500026	3161.282625	3246.255353	1659.814392	1534.572406	1437.303824
6.90_177.1649m/z	177.1649287	6.896766667	neg	4-(2,6,6-Trimethyl-1-cyclohexenyl)-2-butanol	91321	Lipids and lipid-like molecules	Prenol lipids	Sesquiterpenoids	*		38.4	0	M-H2O-H	C13H24O	0.277217595	447.4461865	456.4283262	401.86427	517.7913189	578.7819612	422.8191	294.1346503	324.0248823	240.3506512
6.92_441.1762m/z	441.1762079	6.915966667	neg	4,5-Dihydroniveusin A	88764	Lipids and lipid-like molecules	Prenol lipids	Terpene lactones	*		38.4	0	M+FA-H	C20H28O8	-1.041909034	5102.104069	5820.097027	4641.985557	9508.054175	8935.692319	7625.896853	4054.180939	4793.912481	5029.630525
6.93_173.0819m/z	173.0819376	6.926366667	neg	Ethyl tiglate	LMFA07010884	Lipids and lipid-like molecules	Fatty Acyls	Fatty acid esters	*	C08487	38.4	0	M+FA-H	C7H12O2	0.040213003	318.7702752	379.1790227	441.9140019	397.4001793	395.9690746	385.1219991	374.8113393	422.8732	405.8954029
6.99_511.2913m/z	511.29129	6.993516667	neg	(25S)-11alpha,20,26-trihydroxyecdysone	LMST01010158	Lipids and lipid-like molecules	Sterol Lipids	Sterols			38.4	0	M-H	C27H44O9	0.065365888	969.4990587	1344.496924	1166.231032	1233.589676	1175.10099	1609.010864	1070.632002	1140.01218	1808.060244
7.01_433.2085m/z	433.2084723	7.01105	neg	methyl 9-hydroperoxy-10,12,13,15-bisepidioxy-16E-octadecenoate	74476	Lipids and lipid-like molecules	Fatty Acyls	Fatty Acids and Conjugates			38.4	0	M+FA-H	C19H32O8	1.420363746	5488.092667	5114.636746	5132.879652	5774.585564	5037.789997	5511.667899	5002.666573	5058.932446	4441.338635
7.10_429.2132m/z	429.2132028	7.097333333	neg	20-Trihydroxy-leukotriene-B4	HMDB0012643	Lipids and lipid-like molecules	Fatty Acyls	Eicosanoids	*		38.4	0	M+FA-H	C20H32O7	0.511496073	16973.49547	16569.87228	17506.31666	16177.41128	16769.01252	16165.80104	11981.36248	14884.40089	13890.25611
7.13_269.1007m/z	269.1007453	7.1264	neg	(+)-threo-2-Amino-3,4-dihydroxybutanoic acid	86235	Organic acids and derivatives	Carboxylic acids and derivatives	Amino acids, peptides, and analogues	*		38.4	0	2M-H	C4H9NO4	6.316755723	935.7848416	931.7081363	886.7051113	543.7931533	546.5162319	515.7878193	767.2407949	756.1550274	655.4753074
7.13_467.2655m/z	467.2654695	7.1264	neg	p-Coumaroylputrescine	72157	Unclassified	Unclassified	Unclassified			38.4	0	2M-H	C13H18N2O2	-1.942692276	5584.399272	4809.774098	5024.020772	6650.123602	7276.155164	6164.804259	4746.593609	5534.125111	5081.301953
7.16_310.1640m/z	310.1639669	7.1647	neg	Histidinyl-Arginine	85785	Organic acids and derivatives	Carboxylic acids and derivatives	Amino acids, peptides, and analogues	*		38.4	0	M-H	C12H21N7O3	2.107425747	4216.564773	4710.397999	4098.405064	4925.678696	4683.259875	4584.154112	4497.21137	5161.089696	4793.810805
7.18_245.1396m/z	245.1395578	7.178516667	neg	Clarycet	HMDB0036029	Organoheterocyclic compounds	Oxanes	Unclassified			38.4	0	M+FA-H	C11H20O3	0.551704296	518.2084497	609.3303365	564.7206911	1493.862806	1470.26034	1595.650221	496.2641138	557.2391087	436.8997637
7.24_329.1973m/z	329.1973169	7.237866667	neg	(10S)-Juvenile hormone III diol	64605	Unclassified	Unclassified	Unclassified			38.4	0	M+FA-H	C16H28O4	1.247869336	5372.603494	5983.193453	5404.680271	2408.56241	2697.056059	2292.707959	2378.48156	3005.762798	3132.816357
7.25_467.2653m/z	467.2653466	7.248333333	neg	Lenacil	68909	Unclassified	Unclassified	Unclassified			38.4	0	2M-H	C13H18N2O2	-2.205203938	3018.052831	3843.550008	3520.882227	5426.66164	4827.205566	4740.194739	3495.10259	3833.546025	4302.483458
7.30_169.1233m/z	169.1232849	7.29525	neg	2-Isopropyl-1,4-hexadiene	HMDB0037777	Hydrocarbons	Unsaturated hydrocarbons	Branched unsaturated hydrocarbons			38.4	0	M+FA-H	C9H16	-0.954239791	552.2804062	613.1861345	652.0371645	945.0283838	895.8878301	728.8172304	396.8738636	464.508812	512.1972807
7.30_505.0428m/z	505.0427584	7.303966667	neg	Cefditoren	HMDB0015199	Organoheterocyclic compounds	Tetrahydroisoquinolines	Unclassified			38.4	8.45	M-H	C19H18N6O5S3	-0.090692526	141143.0513	156900.6283	148505.061	125142.5332	121299.3303	120938.5489	105718.0595	114730.1623	125058.8923
7.34_225.1134m/z	225.1133787	7.33585	neg	3-tert-Butyl-4-hydroxyanisole	HMDB0059925	Benzenoids	Phenols	Methoxyphenols			38.4	0	M+FA-H	C11H16O2	0.810924308	847.2403162	933.0025864	833.3302384	927.2742833	841.0089784	815.4008055	850.6033338	789.7299764	872.8837715
7.37_239.1291m/z	239.1290685	7.372733333	neg	(1-Methylethenyl)pyrazine	92103	Organoheterocyclic compounds	Diazines	Pyrazines			38.4	0	2M-H	C7H8N2	-4.795683965	2187.148094	2421.202181	2252.709689	3578.664455	3582.301463	3280.023649	1822.400025	1972.578776	1962.530837
7.40_236.9162m/z	236.91616	7.404266667	neg	5-Iodouracil	HMDB0246818	Unclassified	Unclassified	Unclassified			38.4	0	M-H	C4H3IN2O2	-2.055024386	1104.209806	1169.958571	929.9550832	1139.294909	1431.511194	1271.332721	1240.895127	1078.978725	1093.689262
7.47_463.2341m/z	463.2340515	7.473983333	neg	21-Acetoxy-11beta,17-dihydroxy-6alpha-methylpregn-4-ene-3,20-dione	70751	Unclassified	Unclassified	Unclassified			38.4	0	M+FA-H	C24H34O6	0.740891165	6833.150781	7572.628609	8077.638104	8231.767991	8515.431993	8070.961476	7272.662095	8550.130681	7911.544701
7.47_531.2221m/z	531.2220505	7.473983333	neg	3',4',5'-Trimethoxycinnamyl alcohol acetate	95344	Benzenoids	Phenol ethers	Anisoles			38.4	0	2M-H	C14H18O5	-2.856641874	920.9181298	1016.863465	1057.322258	1075.160621	1175.107028	1397.628942	852.6871164	1116.341942	1118.051203

7.71_433.2084m/z	433.2083725	7.710433333	neg	methyl 10,12,13,15-bisepidioxy-16-hydroperoxy-8E-octadecenoate	LMFA01040052	Lipids and lipid-like molecules	Fatty Acyls	Fatty Acids and Conjugates			38.4	0	M+FA-H	C19H32O8	1.163535286	7120.861344	8327.786457	7538.058329	8763.070366	9836.126205	8083.451768	3988.297187	3782.64728	4305.338594
7.73_442.2601m/z	442.2601419	7.729433333	neg	17-phenyl trinor Prostaglandin F2α serinol amide	44883	Unclassified	Unclassified	Unclassified			38.4	0	M-H2O-H	C26H39NO6	0.531200331	1347.893453	1831.010341	2046.607704	917.1088264	958.6089211	904.6798617	1439.266926	1916.728569	2033.624136
7.81_241.1448m/z	241.1447746	7.814316667	neg	Artemisia alcohol acetate	HMDB0036048	Organic acids and derivatives	Carboxylic acids and derivatives	Carboxylic acid derivatives	*		38.4	0	M+FA-H	C12H20O2	1.232877363	1445.966346	1865.821704	1631.607889	2651.544964	2253.331128	2476.155829	1401.930929	1810.488651	1457.299144
7.90_251.1290m/z	251.1289733	7.899366667	neg	1-Cyclohexenecarboxylic acid	69306	Unclassified	Unclassified	Unclassified			38.4	0	2M-H	C7H10O2	0.359558113	893.6501076	1041.467024	940.1439327	1243.11194	1270.169426	1332.61445	886.9205349	1124.970175	1273.97779
8.03_213.1134m/z	213.1134161	8.033866667	neg	Ethyl (4Z)-4,7-octadienoate	87450	Lipids and lipid-like molecules	Fatty Acyls	Fatty acid esters	*		38.4	0	M+FA-H	C10H16O2	1.091474284	1205.920568	1219.758701	1167.78045	538.4493358	533.1372588	510.4397369	926.5986494	819.7502136	892.7255075
8.03_393.1558m/z	393.1557781	8.033866667	neg	Gibberellin A1	LMPR0104170001	Lipids and lipid-like molecules	Prenol lipids	Isoprenoids	**	C00859	38.4	0	M+FA-H	C19H24O6	0.823561204	2647.911733	2688.257561	2761.794827	5641.420684	5196.390573	5050.820946	2735.864732	3189.200997	3035.513084
8.07_541.2400m/z	541.2400338	8.074883333	neg	PI(14:1(9Z)/0:0)	81196	Lipids and lipid-like molecules	Glycerophospholipids	Glycerophosphoinositols			38.4	0	M-H	C23H43O12P	-3.509863329	69532.67784	75553.56375	69843.53073	14150.05265	13670.65512	13433.86209	34844.23285	42627.95134	39700.03556
8.67_463.1590m/z	463.1589514	8.67015	neg	Fluometuron	72615	Unclassified	Unclassified	Unclassified			38.4	0	2M-H	C10H11F3N2O	3.30177151	3226.190481	3314.955868	3285.003661	3560.133348	2735.16739	3185.799072	1242.351284	1590.672802	1372.975564
8.74_549.1937m/z	549.1936663	8.735716667	neg	Cardiospermin	66907	Unclassified	Unclassified	Unclassified			38.4	0	2M-H	C11H17NO7	-0.110887486	2942.380648	3558.480057	3457.179619	1652.530248	1326.49208	1704.879455	1866.006566	2698.822807	2705.021782
8.76_441.2133m/z	441.2133077	8.759533333	neg	Isopetasoside	HMDB0029622	Lipids and lipid-like molecules	Prenol lipids	Terpene glycosides	*		38.4	0	M+FA-H	C21H32O7	0.760755005	4510.496121	4685.595079	5884.923392	7865.593091	8576.105417	9693.845353	4200.550432	5310.562118	4694.165902
9.08_409.1871m/z	409.1870907	9.077466667	neg	12-Oxo-20-carboxy-leukotriene B4	HMDB0012550	Lipids and lipid-like molecules	Fatty Acyls	Fatty acids and conjugates	*		38.4	0	M+FA-H	C20H28O6	0.821561967	6398.526226	6396.428744	7146.54749	3313.543074	2716.08851	3294.588281	4163.818718	4162.220579	5049.192746
9.25_356.2446m/z	356.2446233	9.248216667	neg	N-tetradecanoyl-L-Homoserine lactone	64716	Unclassified	Unclassified	Unclassified			38.4	0	M+FA-H	C18H33NO3	1.209628271	19003.87524	16597.16051	16668.64543	16000.58402	18127.83867	17785.27859	18917.58204	17580.90585	17956.40225
9.32_397.2233m/z	397.2233326	9.321783333	neg	Prostaglandin I2	778	Lipids and lipid-like molecules	Fatty Acyls	Eicosanoids	**	C01312	38.4	0	M+FA-H	C20H32O5	0.44188341	9849.672711	10125.63251	10256.83706	3414.663025	3588.126498	4102.477102	6344.758922	8237.663348	7203.320153
9.92_213.0671m/z	213.0671467	9.922166667	neg	6-Iminocyclohexa-2,4-dienone	71740	Unclassified	Unclassified	Unclassified			38.4	0	2M-H	C6H5NO	0.913667517	1075.230378	1102.253485	956.3146172	1117.865695	1044.552659	1189.630052	1084.728718	1061.775254	1099.656597
9.97_447.2393m/z	447.239261	9.969833333	neg	Lucidone A	91061	Lipids and lipid-like molecules	Prenol lipids	Sesquiterpenoids	*		38.4	0	M+FA-H	C24H34O5	1.078716272	14958.22069	14123.82516	14686.76538	12970.55709	13018.76979	13734.3432	10873.45858	12345.5153	12463.82502
0.54_213.0560m/z	213.0559685	0.535283333	neg	Cassiachromone	87209	Organoheterocyclic compounds	Benzopyrans	1-benzopyrans	*		38.3	0	M-H2O-H	C13H12O4	1.080351579	738.9638957	717.0667736	1324.018639	375.733614	665.795622	407.9658353	808.315846	531.9656868	674.327681
0.71_173.1044m/z	173.1043726	0.714216667	neg	D-Arginine	HMDB0003416	Organic acids and derivatives	Carboxylic acids and derivatives	Amino acids, peptides, and analogues	**	C00792	38.3	0	M-H	C6H14N4O2	-0.153265368	418.6777795	467.2625851	477.9646699	422.3525515	453.2159955	534.0133184	342.3841883	199.748642	307.2442036
0.74_281.0870m/z	281.0870048	0.7359	neg	Xylobiose	65624	Organic oxygen compounds	Organooxygen compounds	Carbohydrates and carbohydrate conjugates	**	C01630	38.3	0	M-H	C10H18O9	-2.839362379	731.1885189	177.4271778	1009.485766	302.1093109	2176.236164	458.8089075	1042.436187	2009.28175	2117.253551
0.76_265.0208m/z	265.0208261	0.757316667	neg	2-hydroxy-4-oxobutane-1,2,4-tricarboxylic acid	LMFA01050481	Lipids and lipid-like molecules	Fatty Acyls	Fatty Acids and Conjugates			38.3	4.15	M+FA-H	C7H8O8	3.208833397	2402.08928	1238.893363	918.9412349	3357.074561	3092.844089	4392.79829	1195.370423	3981.477865	1742.116391
0.78_291.0030m/z	291.0030058	0.778533333	neg	Dimethylallyl pyrophosphate	LMPR01010001	Lipids and lipid-like molecules	Prenol lipids	Isoprenoid phosphates	**	C00235	38.3	0.413	M+FA-H	C5H12O7P2	-4.158633371	9080.578673	10748.72688	11934.89856	32827.78898	39760.21146	34418.74689	11327.32909	11599.58235	12806.98906
0.80_189.0882m/z	189.088175	0.800366667	neg	4-Amino-1-piperidinecarboxylic acid	HMDB0060385	Organoheterocyclic compounds	Piperidines	Piperidinecarboxylic acids and derivatives		C16837	38.3	0	M+FA-H	C6H12N2O2	0.65604648	2810.414808	3096.082355	2840.486114	1205.637001	1309.81161	1447.940378	1647.905351	2021.176556	2062.735488
0.80_196.0728m/z	196.0727701	0.800366667	neg	Phenicarbazide	73190	Unclassified	Unclassified	Unclassified			38.3	0	M+FA-H	C7H9N3O	0.035212666	2586.330277	2528.066254	2621.33583	1672.482442	1222.831447	1496.695569	2584.974177	2465.174519	2356.513542
0.80_235.0572m/z	235.0572192	0.800366667	neg	N-Carbamylglutamate	44787	Organic acids and derivatives	Carboxylic acids and derivatives	Amino acids, peptides, and analogues	**	C05829	38.3	0	M+FA-H	C6H10N2O5	0.23650457	2050.200322	1721.103723	1810.300124	2125.384085	1752.379728	2432.662148	2256.239306	1778.246941	1339.136043
0.82_122.9758m/z	122.9757815	0.819566667	neg	Sulfoacetaldehyde	63298	Unclassified	Unclassified	Unclassified			38.3	0	M-H	C2H4O4S	0.228378029	634.0559766	975.8759506	982.5902052	1423.808253	1263.742704	1288.656097	808.4401603	725.2845078	672.3450648
0.82_170.9969m/z	170.9969452	0.819566667	neg	Ethyl hydrogen sulfate	87510	Organic acids and derivatives	Organic sulfuric acids and derivatives	Sulfuric acid esters	*		38.3	0	M+FA-H	C2H6O4S	0.497125732	2119.082354	2025.390249	2207.392831	2556.869214	2283.723512	2600.704626	2072.218356	2105.531537	1706.685091
0.82_227.0441m/z	227.0440901	0.819566667	neg	L-2-Aminoethyl seryl phosphate	HMDB0031950	Organic acids and derivatives	Carboxylic acids and derivatives	Amino acids, peptides, and analogues	**	C03872	38.3	0	M-H	C5H13N2O6P	1.068979741	963.9234805	816.3633512	760.0152987	822.8161168	608.2859197	557.7581855	1007.422606	1147.953703	692.236557
0.82_494.0822m/z	494.082183	0.819566667	neg	6-(Methylthio)hexyl glucosinolate	HMDB0038409	Organic oxygen compounds	Organooxygen compounds	Carbohydrates and carbohydrate conjugates			38.3	13.4	M+FA-H	C14H27NO9S3	-1.813256974	21437.75809	20931.66338	24084.52915	31368.87173	30294.42218	30362.70829	23837.82894	25281.47551	23681.8098
0.82_601.1292m/z	601.1291564	0.819566667	neg	1-Guanidino-1-deoxy-scyllo-inositol 4-phosphate	64554	Unclassified	Unclassified	Unclassified			38.3	0	2M-H	C7H16N3O8P	2.375907077	38879.99897	47226.11433	50281.99326	53436.4402	49786.94727	55479.28632	21307.40791	24660.87462	23919.17733
0.86_159.0107m/z	159.0106847	0.86335	neg	4-Fluoromuconolactone	71217	Organoheterocyclic compounds	Dihydrofurans	Furanones	**	C16476	38.3	0	M-H	C6H5FO4	4.838901395	3752.423584	4897.305031	10824.44173	2199.024425	4911.982677	2214.318734	2993.540436	3777.706524	5483.314844
0.86_263.0581m/z	263.0581039	0.86335	neg	Dihydro-5-fluorouracil	687	Unclassified	Unclassified	Unclassified			38.3	0	2M-H	C4H5FN2O2	-6.176278029	9402.202794	11724.6616	12256.60329	17574.68843	16908.81665	15988.57051	11041.52206	13483.57173	12416.27359
0.86_371.1196m/z	371.1195789	0.86335	neg	Pyroglutamylglycine	HMDB0061890	Organic acids and derivatives	Carboxylic acids and derivatives	Amino acids, peptides, and analogues			38.3	0	2M-H	C7H10N2O4	-3.381302438	5146.755909	5382.543863	6844.882593	5688.111142	5453.077862	5790.367312	5586.480933	5554.168865	5070.189288
0.88_172.0728m/z	172.0728076	0.884633333	neg	2-Oxoarginine	7030	Organic acids and derivatives	Keto acids and derivatives	Short-chain keto acids and derivatives	**	C03771	38.3	0	M-H	C6H11N3O3	0.247210247	457.9410801	411.3271608	615.7108393	623.4605143	299.8728385	316.9254771	396.910296	697.8240014	484.2027117
0.90_359.0486m/z	359.0485904	0.904683333	neg	5'-Phosphoribosyl-N-formylglycinamide (FGAR)	3443	Unclassified	Unclassified	Unclassified			38.3	0	M+FA-H	C8H15N2O9P	-3.595512396	4175.474107	5050.483086	6301.350225	5522.146205	7321.043171	6836.005545	4894.762952	3370.826272	4638.085096
0.93_217.0830m/z	217.082999	0.925866667	neg	5-δ-Hydroxybutyl Hydantoin	44728	Unclassified	Unclassified	Unclassified			38.3	0	M+FA-H	C7H12N2O3	0.022608993	1657.395471	1505.990966	1477.383228	745.0407404	591.8911683	889.6998511	1337.944917	1632.410663	833.0112912
1.14_454.9986m/z	454.998565	1.1382	neg	3,5-Dinitrosalicylic acid	68986	Unclassified	Unclassified	Unclassified			38.3	0	2M-H	C7H4N2O7	4.693905455	4456.298578	7170.513564	10255.1836	5127.315499	6641.090064	5087.065246	6974.423485	4772.989514	8643.761161
1.16_173.9867m/z	173.9866852	1.160166667	neg	3-Hydroxypyridine sulfate	HMDB0240652	Organic acids and derivatives	Organic sulfuric acids and derivatives	Arylsulfates			38.3	0	M-H	C5H5NO4S	0.188382295	8907.02017	8526.051595	9962.028618	4341.425808	4505.531284	4499.13018	5811.394877	5102.324207	5990.236944
1.27_236.0600m/z	236.0600473	1.26875	neg	N-Acetyl-L-methionine	HMDB0011745	Organic acids and derivatives	Carboxylic acids and derivatives	Amino acids, peptides, and analogues	*	C02712	38.3	0	M+FA-H	C7H13NO3S	1.204451768	1264.309642	1495.744497	1361.672246	1259.917896	1465.924889	1333.263494	1248.526638	1574.958315	1348.619413
1.46_139.0071m/z	139.0071198	1.459966667	neg	Dimethyl sulfone	HMDB0004983	Organosulfur compounds	Sulfonyls	Sulfones	**	C11142	38.3	0	M+FA-H	C2H6O2S	0.707600413	587.3202576	510.5778089	378.1729959	805.6197498	546.0867581	669.3720648	659.0715507	532.2496791	817.3955455
1.58_307.0519m/z	307.051862	1.577833333	neg	Diethyl sulfate	HMDB0251240	Unclassified	Unclassified	Unclassified			38.3	0	2M-H	C4H10O4S	-2.665524653	533.6321575	369.3546565	384.0162434	511.984974	475.8075111	544.0038455	362.259211	450.2352228	437.2665263
1.59_229.0443m/z	229.0443012	1.5934	neg	Dapsone	HMDB0014395	Benzenoids	Benzene and substituted derivatives	Benzenesulfonyl compounds		C07666	38.3	0	M-H2O-H	C12H12N2O2S	0.780855778	1868.849753	1671.467751	1684.561259	1534.952498	1476.33678	1409.648073	1570.734361	1737.810463	1762.081701
1.82_172.0617m/z	172.0616544	1.820883333	neg	(±)-4-Methylene-2-pyrrolidinecarboxylic acid	86265	Organic acids and derivatives	Carboxylic acids and derivatives	Amino acids, peptides, and analogues	*		38.3	0	M+FA-H	C6H9NO2	0.967975424	150.9554357	171.1914214	155.6294599	141.3205357	167.5405316	107.7038388	65.61176026	120.3337793	173.6901065
1.93_223.0006m/z	223.0005756	1.933583333	neg	6-Deoxy-5-ketofructose 1-phosphate	63568	Unclassified	Unclassified	Unclassified			38.3	0	M-H2O-H	C6H11O8P	-3.046197083	1740.599253	1771.622598	1356.615502	1793.87661	2037.49749	2157.082499	1679.234699	1757.685258	1571.21518
10.63_277.1447m/z	277.1446934	10.63316667	neg	1(10),11-Eremophiladiene-2,9-dione	90703	Lipids and lipid-like molecules	Prenol lipids	Sesquiterpenoids	*		38.3	0	M+FA-H	C15H20O2	0.692038379	1443.234652	1348.435562	1396.769155	1723.795347	1980.740924	1832.544556	1689.1249	1584.864807	1764.472675
11.05_381.1135m/z	381.1134592	11.05113333	neg	Lophirone J	52539	Lipids and lipid-like molecules	Polyketides	Flavonoids			38.3	0	M-H2O-H	C25H20O5	0.566350465	6697.374958	7528.291023	5995.756351	7388.963134	7866.869198	8100.279905	7152.573886	7677.703254	7062.700261
11.16_269.2124m/z	269.2123626	11.15923333	neg	3-Pentadecenal	94889	Lipids and lipid-like molecules	Fatty Acyls	Fatty aldehydes	*		38.3	0	M+FA-H	C15H28O	0.643261838	995.3911618	898.032069	730.2886738	802.8755272	1026.650369	758.0789911	1043.076907	867.0096247	782.5683096
11.33_901.2009m/z	901.2008673	11.3321	neg	Flumazenil glucoronide	2727	Unclassified	Unclassified	Unclassified			38.3	0	2M-H	C19H18FN3O9	3.0247946	15801.52461	13992.04239	11611.75749	13544.20328	10971.46246	12762.06809	15622.02916	11385.3147	11313.36665
11.79_297.2436m/z	297.2436173	11.78693333	neg	2,6,8,12-Tetramethyl-2,4-tridecadien-1-ol	46121	Lipids and lipid-like molecules	Fatty Acyls	Fatty alcohols			38.3	0	M+FA-H	C17H32O	0.391721417	3280.503361	2895.115261	3041.081635	2818.257624	2618.443987	2567.104194	4577.898111	3039.219935	3957.119708
14.57_211.0360m/z	211.0360399	14.57003333	neg	4-Hydroxy-3-nitrosobenzamide	69313	Unclassified	Unclassified	Unclassified			38.3	0	M+FA-H	C7H6N2O3	-0.030183523	324.7785464	443.5993605	384.0115625	444.2268919	252.7222499	283.6358473	438.2543961	279.5408516	456.8139955
15.01_491.1197m/z	491.119749	15.0096	neg	Obtusifolin 2-glucoside	68292	Benzenoids	Anthracenes	Anthraquinones	*	C10381	38.3	0	M+FA-H	C22H22O10	0.558685246	20678.80807	17526.50443	19808.71904	14615.68189	17925.58178	16762.79758	17206.43868	20997.69808	20257.83527
15.05_149.0095m/z	149.0095324	15.05453333	neg	D-Tartaric acid	HMDB0029878	Organic oxygen compounds	Organooxygen compounds	Carbohydrates and carbohydrate conjugates	*	C02107	38.3	0	M-H	C4H6O6	2.472273924	2149.449385	19720.33091	1370.959969	7900.157268	1837.891883	1368.571013	1097.93123	1071.613999	1089.394096
15.05_239.0599m/z	239.0598634	15.05453333	neg	Histidinyl-Cysteine	85788	Organic acids and derivatives	Carboxylic acids and derivatives	Amino acids, peptides, and analogues	*		38.3	0	M-H2O-H	C9H14N4O3S	-3.707265788	99314.71939	689392.5251	53718.76594	264080.8587	90931.33845	48314.07024	255186.4702	36452.22391	42014.77328
2.32_175.0614m/z	175.061369	2.322933333	neg	Ethyl acetoacetate	87501	Organic acids and derivatives	Keto acids and derivatives	Beta-keto acids and derivatives	*	C03500	38.3	0	M+FA-H	C6H10O3	1.322374317	442.6010396	413.1707835	332.5530586	674.4965551	665.4466932	483.6999345	316.9772899	548.3784065	519.2435588
2.74_111.0452m/z	111.0451789	2.743066667	neg	5-Ethoxy-4,5-dihydro-2(3H)furanone	HMDB0031214	Organoheterocyclic compounds	Lactones	Gamma butyrolactones	*		38.3	0	M-H2O-H	C6H10O3	0.198552998	185.5874018	202.2595291	247.0646665	128.6912771	80.90125301	87.70248013	69.76433055	112.082383	132.4043427
2.92_209.0669m/z	209.0668504	2.9156	neg	L-Fucose	63169	Organic oxygen compounds	Organooxygen compounds	Carbohydrates and carbohydrate conjugates	**	C01019	38.3	0	M+FA-H	C6H12O5	1.060784133	523.6558717	371.2162845	489.7630565	607.1066981	628.6189104	566.4609505	577.4722605	470.4505257	484.3451521
3.13_313.0568m/z	313.0567967	3.131933333	neg	6-(3-carboxyphenoxy)-3,4,5-trihydroxyoxane-2-carboxylic acid	HMDB0124994	Organic oxygen compounds	Organooxygen compounds	Carbohydrates and carbohydrate conjugates	*		38.3	0	M-H	C13H14O9	0.926872357	4551.674434	4801.908075	5190.434318	2804.1763	2355.253428	2372.639646	3852.535715	4562.582141	3833.686202
3.56_295.1664m/z	295.1663982	3.564783333	neg	Rivastigmine	HMDB0015124	Benzenoids	Benzene and substituted derivatives	Phenoxy compounds		C11766	38.3	0	M+FA-H	C14H22N2O2	0.269244158	1056.792534	1315.089515	1239.152211	1202.130368	1296.762792	1297.181682	956.3161777	1012.718824	883.0384484
3.58_311.1254m/z	311.1253608	3.5753	neg	N5-(4-Methoxybenzyl)glutamine	89401	Organic acids and derivatives	Carboxylic acids and derivatives	Amino acids, peptides, and analogues			38.3	0	M+FA-H	C13H18N2O4	1.882104602	458.9708961	543.7982094	436.4941425	980.3471335	901.7804054	905.1996678	804.6523296	574.3494783	626.7696969
3.58_471.0587m/z	471.0587109	3.5753	neg	2-Hydroxy-3-carboxybenzalpyruvate	69790	Unclassified	Unclassified	Unclassified			38.3	0	2M-H	C11H8O6	3.837186256	834.2536371	1019.184572	885.3648316	1216.524004	1515.898885	1422.185229	989.3666545	906.1823199	890.9993383
3.61_347.0984m/z	347.0984269	3.611966667	neg	2-Oxopimelate	71246	Unclassified	Unclassified	Unclassified			38.3	0	2M-H	C7H10O5	0.162379372	1207.076691	1264.655163	1128.797665	569.4653409	641.2859692	851.2610189	778.9803644	905.074464	1037.331134
3.65_261.0075m/z	261.0075012	3.64965	neg	2-Hydroxy-4-methoxyacetophenone 5-sulfate	HMDB0041648	Organic oxygen compounds	Organooxygen compounds	Carbonyl compounds	*		38.3	0	M-H	C9H10O7S	0.205905365	8128.190124	8892.540601	8749.306981	7301.770811	7894.136575	7502.316734	7710.404284	7832.158311	8426.564221
3.66_355.1148m/z	355.1148435	3.6635	neg	Glutamyltyrosine	HMDB0028831	Organic acids and derivatives	Carboxylic acids and derivatives	Amino acids, peptides, and analogues	*		38.3	0	M+FA-H	C14H18N2O6	0.497763103	52.92371112	227.7176036	215.0426299	2072.799872	1847.527746	2018.729087	707.1870391	622.4357834	780.2818107
3.75_383.0481m/z	383.0480737	3.747833333	neg	Imidazoleacetic acid ribotide	HMDB0006032	Organic oxygen compounds	Organooxygen compounds	Carbohydrates and carbohydrate conjugates	**	C04437	38.3	0	M+FA-H	C10H15N2O9P	-4.868793939	398.8562428	401.1402255	390.8401844	1145.923208	796.4613668	522.9208683	550.5946054	539.9209094	489.6506212
3.78_346.1622m/z	346.162206	3.779133333	neg	(Ac)2-L-Lys-D-Ala	65775	Unclassified	Unclassified	Unclassified			38.3	0	M+FA-H	C13H23N3O5	0.77131414	479.28074	373.6525454	319.335712	506.6342158	591.8845544	483.9532327	288.329612	643.7286657	545.2617592
3.78_366.0831m/z	366.0831301	3.779133333	neg	Zeanoside B	93479	Organic oxygen compounds	Organooxygen compounds	Carbohydrates and carbohydrate conjugates			38.3	0	M-H	C16H17NO9	0.205510325	874.0780469	695.3399979	640.0605393	1065.109638	857.7196403	870.4455024	697.1672886	762.8071559	749.7359593
3.78_392.1057m/z	392.1057173	3.779133333	neg	Profluralin	72813	Unclassified	Unclassified	Unclassified			38.3	0	M+FA-H	C14H16F3N3O4	-5.117246987	1367.069001	1145.875443	978.0062848	2553.56323	2172.513794	1744.86042	1006.306512	1239.921421	1118.259207
3.85_367.2239m/z	367.2239277	3.84605	neg	BW 245C	45416	Unclassified	Unclassified	Unclassified			38.3	0	M-H	C19H32N2O5	0.222744707	644.0096168	760.3274891	751.4788563	316.7181239	421.6600142	684.8663238	769.2891018	776.1599846	779.0078775
3.91_259.0758m/z	259.0758246	3.912566667	neg	Methyl bisnorbiotinyl ketone	7084	Organoheterocyclic compounds	Thienoimidazolidines	Unclassified	*		38.3	0	M+FA-H	C9H14N2O2S	0.107403227	1110.661237	1232.778974	1173.513455	1399.044404	1357.896117	1143.580211	1242.795253	1450.948007	1215.543712
3.96_119.0502m/z	119.0502368	3.957583333	neg	Phenylacetaldehyde	HMDB0006236	Benzenoids	Benzene and substituted derivatives	Phenylacetaldehydes	**	C00601	38.3	0	M-H	C8H8O	-0.013609846	320.0349856	373.4659371	372.1236649	97.25791761	89.79605988	101.6411512	332.1665219	300.4843906	332.9119269
3.96_297.0551m/z	297.0550656	3.957583333	neg	Hydroxyzileuton	HMDB0013968	Organoheterocyclic compounds	Benzothiophenes	1-benzothiophenes	*		38.3	0	M+FA-H	C11H12N2O3S	-0.002089456	579.5615104	600.980939	627.2593945	469.8248984	424.183139	505.1844883	413.8136518	308.1542244	286.8843297
3.96_319.1037m/z	319.1037371	3.957583333	neg	Dikegulac	72590	Unclassified	Unclassified	Unclassified			38.3	0	M+FA-H	C12H18O7	1.026300009	6452.406906	7257.560236	7289.717942	15242.32337	13958.63264	15046.82701	9146.635813	10064.40244	10109.21799
4.00_216.0410m/z	216.0409731	4.002233333	neg	2,4,6-trimethyl-3,5-dinitrobenzonitrile	34473	Unclassified	Unclassified	Unclassified			38.3	0	M-H2O-H	C10H9N3O4	-2.091310017	806.4584006	881.1745045	807.9516535	1045.231008	925.3198838	1120.230742	1060.755717	1108.552888	1044.162016
4.02_336.1205m/z	336.1205104	4.024283333	neg	Tryptophyl-Serine	85985	Organic acids and derivatives	Carboxylic acids and derivatives	Amino acids, peptides, and analogues	*		38.3	0	M+FA-H	C14H17N3O4	1.379199799	1825.245701	2024.781398	2311.155613	2030.337808	2206.567397	2348.2245	2007.522653	2266.544787	2033.642076
4.19_358.0545m/z	358.0545478	4.189783333	neg	Cyclic dAMP	3441	Unclassified	Unclassified	Unclassified			38.3	0	M+FA-H	C10H12N5O5P	-4.025453385	11636.58439	7486.244588	9174.229771	7088.329705	6408.565877	8874.453847	12445.3672	9926.130602	10039.17096
4.26_430.9643m/z	430.9643436	4.2568	neg	S-Methyl-3-phospho-1-thio-D-glycerate	66174	Unclassified	Unclassified	Unclassified			38.3	0	2M-H	C4H9O6PS	0.298113376	8853.411784	9424.913746	9097.157797	5410.086359	5012.75333	4818.548953	7111.357403	8432.795055	7290.282653
4.28_135.0452m/z	135.045203	4.278183333	neg	4-Hydroxyphenylacetaldehyde	63506	Benzenoids	Benzene and substituted derivatives	Phenylacetaldehydes	**	C03765	38.3	0	M-H	C8H8O2	0.367073652	792.610602	1000.567138	1010.171515	545.5547965	606.566179	582.7257986	718.9831682	1014.430352	729.5116756
4.30_345.1192m/z	345.1191953	4.300033333	neg	2-(3-Hydroxyphenyl)ethanol 1'-glucoside	HMDB0038332	Organic oxygen compounds	Organooxygen compounds	Carbohydrates and carbohydrate conjugates			38.3	0	M+FA-H	C14H20O7	0.297903501	3053.185964	2741.476435	2649.904212	3968.125929	3963.188845	3857.032315	1910.771168	2058.48659	1724.741802
4.32_359.0988m/z	359.0988177	4.3224	neg	Picolinoylglycine	HMDB0059766	Organic acids and derivatives	Carboxylic acids and derivatives	Amino acids, peptides, and analogues			38.3	0	2M-H	C8H8N2O3	-2.47186337	1507.674235	1309.539334	1173.90002	1290.773079	1053.243112	1340.790993	1468.938673	1862.729155	1655.952957
4.43_511.1457m/z	511.1457366	4.432683333	neg	Agnuside	67924	Lipids and lipid-like molecules	Prenol lipids	Terpene glycosides	*		38.3	0	M+FA-H	C22H26O11	0.047470437	1956.159305	1430.465395	1935.328478	0.001403199	0.001403199	148.6460026	345.6106274	885.7642544	866.6670787
4.45_397.1142m/z	397.1141518	4.454616667	neg	5-(3',5'-Dihydroxyphenyl)-gamma-valerolactone-O-glucuronide-O-methyl	HMDB0060030	Organic oxygen compounds	Organooxygen compounds	Carbohydrates and carbohydrate conjugates	*		38.3	0	M-H	C18H22O10	0.329889875	2360.862133	2673.456196	2864.852684	2247.666797	1904.993761	2534.221417	4138.90496	4977.249011	4247.30971
4.45_467.1533m/z	467.1533383	4.454616667	neg	Bis-(4-cyanophenyl)-methanol	85080	Unclassified	Unclassified	Unclassified			38.3	0	2M-H	C15H10N2O	4.248254897	3760.520656	3935.350559	2816.556267	1651.142671	1345.279781	1774.196405	1866.199533	2550.658071	2491.812025
4.50_483.1781m/z	483.1781132	4.49925	neg	Suxibuzone	HMDB0042019	Lipids and lipid-like molecules	Fatty Acyls	Fatty acid esters			38.3	0	M+FA-H	C24H26N2O6	1.879869829	259.4185993	422.6837534	1113.45982	158.2734993	101.2359495	252.4929859	387.5067072	386.085895	260.6323208
4.54_242.1037m/z	242.1036608	4.54425	neg	Desglymidodrine	HMDB0060558	Benzenoids	Benzene and substituted derivatives	Methoxybenzenes	*		38.3	0	M+FA-H	C10H15NO3	1.342486046	90.54707299	159.8998856	182.751539	275.5203885	276.1389317	307.1675265	161.3632846	261.1099254	225.6199993
4.57_282.1470n	327.1452729	4.5668	neg	Epidihydrophaseic acid	HMDB0038661	Lipids and lipid-like molecules	Prenol lipids	Sesquiterpenoids	*		38.3	0	M-H, M+FA-H	C15H22O5	0.924355901	1423.484482	1319.794385	1568.135073	1725.834866	1650.76714	1441.510802	1194.715158	1162.49864	1107.431622
4.59_419.0624m/z	419.062377	4.589283333	neg	Gossypetin 7-methyl ether 8-acetate	LMPK12113256	Lipids and lipid-like molecules	Polyketides	Flavonoids			38.3	0	M+FA-H	C18H14O9	1.048106728	0.001403199	69.72298713	0.001403199	0.001403199	0.001403199	0.001403199	2967.520908	2929.08468	3061.725465
4.63_332.2082m/z	332.2081689	4.632666667	neg	Octanoylcarnitine	HMDB0000791	Lipids and lipid-like molecules	Fatty Acyls	Fatty acid esters	*	C02838	38.3	0	M+FA-H	C15H29NO4	1.070996364	310.8115794	401.7719969	409.721406	705.4796967	676.6695619	618.1145994	459.3528243	430.2158393	680.96599
4.64_327.1175m/z	327.1174515	4.641416667	neg	Phenylheptatriyne	66990	Unclassified	Unclassified	Unclassified			38.3	0	2M-H	C13H8	-1.440392143	1141.738033	1005.043779	1235.221254	357.0783133	162.4858128	172.1825269	329.0850188	407.7628982	301.5654798
4.64_471.0552m/z	471.0552488	4.641416667	neg	2-Carboxy-2-hydroxy-8-carboxychromene	69789	Unclassified	Unclassified	Unclassified			38.3	0	2M-H	C11H8O6	-3.496865874	390.0109128	257.5169099	445.3289877	478.2365898	369.4275037	807.2778672	2167.446253	2068.382719	1003.291405
4.75_329.0562m/z	329.0561982	4.747466667	neg	6-Thioinosinic acid	HMDB0060791	Nucleosides, nucleotides, and analogues	Purine nucleosides	Unclassified	*		38.3	0	M+FA-H	C10H12N4O4S	0.244359495	1232.289461	1512.312184	2097.607653	1177.154699	1321.713381	1280.098143	1281.837102	1532.778979	1390.721259
4.75_363.1299m/z	363.1298787	4.747466667	neg	Tryptophyl-Asparagine	HMDB0029078	Organic acids and derivatives	Carboxylic acids and derivatives	Amino acids, peptides, and analogues	*		38.3	1.27	M+FA-H	C15H18N4O4	-3.549504719	5002.631216	5599.761858	5342.20747	8256.770483	8006.682188	7980.442786	5914.116403	6631.623969	6476.828962
4.75_410.1817m/z	410.1816632	4.747466667	neg	Senkirkine	68302	Unclassified	Unclassified	Unclassified			38.3	0	M+FA-H	C19H27NO6	-1.03293918	2396.946575	3159.721557	2958.213395	1332.103214	1096.261018	970.8474139	1590.398522	2526.744307	2058.752323
4.81_549.1187m/z	549.11874	4.814166667	neg	3-beta-D-Glucopyranuronosyloxy-5-methylisoxazole	68848	Unclassified	Unclassified	Unclassified			38.3	0	2M-H	C10H13NO8	-4.028783416	2364.281903	3488.501654	3387.198006	2401.87452	1672.016651	1526.397335	3126.825816	3250.758162	3666.194141
4.84_442.2199m/z	442.2199301	4.836566667	neg	PC(0:0/9:0)[U]	40364	Unclassified	Unclassified	Unclassified			38.3	0	M+FA-H	C17H36NO7P	-3.050905559	4823.96354	4601.39897	4407.32107	2008.02581	2002.550819	2156.935714	2914.861635	3261.620826	2680.294567
4.88_119.0502m/z	119.0502265	4.880083333	neg	3-Methylbenzaldehyde	HMDB0029637	Benzenoids	Benzene and substituted derivatives	Benzoyl derivatives	**	C07209	38.3	0	M-H	C8H8O	-0.099198025	183.6267495	195.3217538	189.0621124	49.19652856	69.42648314	46.57850334	108.5304699	124.3441963	108.438909
4.88_551.1775m/z	551.1774752	4.880083333	neg	4,6,2',4'-Tetramethoxychalcone 2'-beta-glucoside	52083	Unclassified	Unclassified	Unclassified			38.3	0	M+FA-H	C25H30O11	0.90994369	1166.025039	1473.440104	1257.323025	144.3459607	119.9440571	0.001403199	1916.187858	1779.378792	1524.399457
4.90_459.0997m/z	459.0997328	4.902316667	neg	GW 590735	45205	Unclassified	Unclassified	Unclassified			38.3	0	M-H2O-H	C23H21F3N2O4S	0.33685319	5101.433777	5215.526506	5022.472632	7598.213751	7676.647985	7645.038886	6120.405434	5940.628296	5368.920203
4.95_704.1286n	703.1212986	4.94705	neg	a-L-threo-4-Hex-4-enopyranuronosyl-D-galacturonic acid	94275	Organic oxygen compounds	Organooxygen compounds	Carbohydrates and carbohydrate conjugates			38.3	0	M-H, M+FA-H	C24H32O24	0.31691918	6289.924803	6949.188806	6428.277539	0.001403199	92.75269376	0.001403199	4335.417898	3955.329809	4459.532367
4.99_181.0872m/z	181.0872017	4.99225	neg	p-Isopropylphenol	HMDB0031446	Benzenoids	Benzene and substituted derivatives	Cumenes	*		38.3	0	M+FA-H	C9H12O	1.350610545	708.5911543	887.2554419	923.6548519	817.725119	839.5730023	894.2494985	1083.669489	865.8089549	853.952218
5.01_273.1710m/z	273.1709915	5.014716667	neg	6-hydroxy-4-tridecanolide	97399	Lipids and lipid-like molecules	Fatty Acyls	Fatty esters			38.3	0	M+FA-H	C13H24O3	1.069371973	1009.853973	800.0372356	1030.319842	1161.19639	1094.83088	1379.584329	1016.315652	991.5288442	802.1188392
5.06_120.0455m/z	120.0455432	5.05975	neg	3-Acetylpyridine	HMDB0033131	Organic oxygen compounds	Organooxygen compounds	Carbonyl compounds			38.3	0	M-H	C7H7NO	0.460551612	284.0180704	298.8007088	328.0362308	419.6207339	404.9037047	448.8783669	329.180438	376.5122843	445.1377159
5.10_473.1659m/z	473.1659074	5.1031	neg	Furcatin	68339	Unclassified	Unclassified	Unclassified			38.3	0	M+FA-H	C20H28O10	-1.267231465	1000.893723	1269.013216	843.8630412	1150.788502	722.899851	1419.60267	1028.296116	736.1594887	1135.9569
5.13_539.1727m/z	539.1727125	5.132616667	neg	3,5-Dimethoxy-2,7-phenanthrenediol	87807	Benzenoids	Phenanthrenes and derivatives	Phenanthrols	*		38.3	0	2M-H	C16H14O4	2.908384555	2572.458386	3191.721338	1894.985877	2654.435736	1798.57643	2769.693039	3102.315128	2927.992655	3149.637106
5.29_195.1028m/z	195.1027661	5.28555	neg	4-tert-Butylphenol	HMDB0032063	Benzenoids	Benzene and substituted derivatives	Phenylpropanes	*	C14200	38.3	0	M+FA-H	C10H14O	0.654132086	445.677421	345.9287484	362.2084499	401.9733141	615.0083989	526.2429963	426.4826411	337.6242434	451.8069248
5.32_259.0101m/z	259.0100999	5.320233333	neg	4-Hydroxy-5-methyl-3(2H)-thiophenone	89358	Organic acids and derivatives	Vinylogous thioesters	Unclassified			38.3	0	2M-H	C5H6O2S	-1.248042197	33857.32237	35461.57474	35735.99028	44917.41371	48715.42326	48762.1213	40512.11038	40505.35153	42871.6109
5.36_255.1603m/z	255.1602907	5.361783333	neg	Allyl butyrate	95074	Lipids and lipid-like molecules	Fatty Acyls	Fatty acid esters	*		38.3	0	2M-H	C7H12O2	0.421024698	326.2689151	433.9765742	261.786668	308.0835422	308.78184	396.4683815	379.5473424	267.5398159	342.3038689
5.36_537.0557m/z	537.055668	5.361783333	neg	N-Acetyl-L-glutamate 5-phosphate	3396	Organic acids and derivatives	Carboxylic acids and derivatives	Amino acids, peptides, and analogues	**	C04133	38.3	0	2M-H	C7H12NO8P	5.275394385	2224.842977	2811.320301	2699.930855	1829.927923	2446.088344	1475.901724	1149.98669	1847.480889	1920.761192
5.41_473.1132m/z	473.1132316	5.406433333	neg	PD 146176	45273	Unclassified	Unclassified	Unclassified			38.3	0	2M-H	C15H11NS	-4.076438572	24273.40705	26905.91725	25122.43754	47159.75404	46963.11392	43367.01176	35366.65109	37272.01502	29279.47085
5.41_529.1305m/z	529.1304959	5.406433333	neg	N-[[3-(b-D-Glucopyranosyloxy)-2,3-dihydro-2-oxo-1H-indol-3-yl]acetyl]aspartic a	93984	Benzenoids	Benzene and substituted derivatives	Phenylacetamides			38.3	0	M+FA-H	C20H24N2O12	-1.303787498	4844.77325	4438.915315	3953.754783	6657.391997	6131.48294	5942.350549	4780.818718	4790.668688	4348.652311
5.43_377.1820m/z	377.1819742	5.428733333	neg	7-Hydroxyterpineol 8-glucoside	HMDB0033019	Organic oxygen compounds	Organooxygen compounds	Carbohydrates and carbohydrate conjugates	*		38.3	0	M+FA-H	C16H28O7	0.807092724	4878.602189	5035.545119	5740.522827	4854.098152	4783.812539	4933.107828	6028.346564	5356.344529	5300.438307
5.43_403.0132m/z	403.0132137	5.428733333	neg	4-carboxy-2-hydroxy-cis,cis-muconic acid	45915	Lipids and lipid-like molecules	Fatty Acyls	Fatty Acids and Conjugates	**	C04324	38.3	0	2M-H	C7H6O7	-5.482073636	6024.718403	6198.762905	5998.751465	4128.085792	4185.926301	4156.530481	5736.703118	5794.752818	4651.238448
5.50_179.0715m/z	179.0714981	5.497816667	neg	(4-Methylphenyl)acetaldehyde	HMDB0029639	Benzenoids	Benzene and substituted derivatives	Phenylacetaldehydes	*		38.3	0	M+FA-H	C9H10O	0.971668636	447.4802375	348.6323581	347.4137742	208.5140397	350.2644662	329.8196364	503.7500325	320.5694323	442.3775703

5.50_435.1874m/z	435.1874192	5.497816667	neg	2-Oxo-9-methylthiononanoic acid	64507	Unclassified	Unclassified	Unclassified			38.3	0	2M-H	C10H18O3S
5.50_547.1459m/z	547.145905	5.497816667	neg	Pueraria glycoside 2	LMPK12050009	Lipids and lipid-like molecules	Polyketides	Flavonoids			38.3	0	M-H	C26H28O13
5.52_495.1106m/z	495.1105508	5.516816667	neg	S-ethyl N-[4-(trifluoromethyl)phenyl] Isothiourea	64868	Unclassified	Unclassified	Unclassified			38.3	0	2M-H	C10H11F3N2S
5.52_675.2250m/z	675.2250224	5.516816667	neg	Yinyanghuo D	LMPK12110415	Phenylpropanoids and polyketides	Flavonoids	Flavans	*		38.3	0	2M-H	C20H18O5
5.67_523.2553m/z	523.2553355	5.666566667	neg	Methohexital	43252	Organoheterocyclic compounds	Diazines	Pyrimidines and pyrimidine derivatives		C07844	38.3	0	2M-H	C14H18N2O3
5.71_519.1287m/z	519.1286714	5.709933333	neg	2,3,4'-TRIHYDROXY-4-METHOXYBENZOPHENONE	43634	Unclassified	Unclassified	Unclassified			38.3	0	2M-H	C14H12O5
5.81_490.1676m/z	490.1676058	5.806016667	neg	Tetrahydrofolic acid	714	Organoheterocyclic compounds	Imidazopyrimidines	Purines and purine derivatives	**	C00101	38.3	0	M+FA-H	C19H23N7O6
5.92_269.1396m/z	269.1396069	5.9168	neg	3-Methylbutyl 2-furanbutanoate	HMDB0037736	Lipids and lipid-like molecules	Fatty Acyls	Fatty acid esters	*		38.3	0	M+FA-H	C13H20O3
5.92_294.1711m/z	294.1711478	5.9168	neg	N,O-Didesmethylvenlafaxine	HMDB0060852	Organic oxygen compounds	Organooxygen compounds	Alcohols and polyols	*		38.3	0	M+FA-H	C15H23NO2
5.94_239.0021m/z	239.0020754	5.937716667	neg	1,2-Dihydroxynaphthalene-6-sulfonate	71140	Unclassified	Unclassified	Unclassified			38.3	0	M-H	C10H8O5S
5.94_369.1919m/z	369.1919087	5.937716667	neg	Lactapiperanol D	HMDB0033631	Lipids and lipid-like molecules	Prenol lipids	Diterpenoids	*		38.3	0	M+FA-H	C18H28O5
5.95_567.2040m/z	567.2040194	5.95155	neg	Desmethoxymatteucinol	52681	Phenylpropanoids and polyketides	Flavonoids	Flavans	*		38.3	0	2M-H	C17H16O4
6.09_257.1397m/z	257.1397075	6.087383333	neg	12-oxo-9(Z)-dodecenoic acid	35852	Unclassified	Unclassified	Unclassified			38.3	0	M+FA-H	C12H20O3
6.32_185.1184m/z	185.1183587	6.320716667	neg	(±)-2,6-Dimethyl-5-heptenal	HMDB0031834	Organic oxygen compounds	Organooxygen compounds	Carbonyl compounds	*		38.3	0	M+FA-H	C9H16O
6.37_423.1664m/z	423.1663902	6.373833333	neg	Carinol	HMDB0030657	Lignans, neolignans and related compounds	Dibenzylbutane lignans	Dibenzylbutanediol lignans	*		38.3	0	M+FA-H	C20H26O7
6.37_479.1525m/z	479.1525386	6.373833333	neg	2',5'-Dihydroxychalcone	88211	Phenylpropanoids and polyketides	Linear 1,3-diarylpropanoids	Chalcones and dihydrochalcones	*		38.3	0	2M-H	C15H12O3
6.39_163.0402m/z	163.0402016	6.386066667	neg	Benzofuran	70116	Organoheterocyclic compounds	Benzofurans	Unclassified		C14512	38.3	0	M+FA-H	C8H6O
6.39_364.2265m/z	364.226522	6.386066667	neg	PC(O-8:0/O-1:0)[U]	40254	Unclassified	Unclassified	Unclassified			38.3	0	M-H2O-H	C17H38NO6P
6.39_391.2454m/z	391.2453586	6.386066667	neg	1,3-Diphenylpropane	70176	Phenylpropanoids and polyketides	Linear 1,3-diarylpropanoids	Unclassified	*	C14581	38.3	0	2M-H	C15H16
6.39_461.2184m/z	461.2183551	6.386066667	neg	(1H-Indol-4-yl)-Carbamic Acid tert-butyl ester	44983	Unclassified	Unclassified	Unclassified			38.3	0	2M-H	C13H15N2O2
6.41_545.1309m/z	545.1308602	6.408833333	neg	Apiin	HMDB0030843	Phenylpropanoids and polyketides	Flavonoids	Flavonoid glycosides	***	C04858	38.3	0	M-H2O-H	C26H28O14
6.42_304.1227m/z	304.1226847	6.41995	neg	zeleplon	85038	Unclassified	Unclassified	Unclassified			38.3	7.43	M-H	C17H15N5O
6.46_473.0724m/z	473.0723688	6.458366667	neg	Theaflavic acid	HMDB0040609	Phenylpropanoids and polyketides	Flavonoids	Flavans			38.3	0	M+FA-H	C21H16O10
6.52_289.1659m/z	289.165942	6.520516667	neg	3-methyl-dodecanedioic acid	74903	Lipids and lipid-like molecules	Fatty Acyls	Fatty Acids and Conjugates			38.3	0	M+FA-H	C13H24O4
6.57_363.1547m/z	363.1546665	6.570233333	neg	Lathyrine	66888	Unclassified	Unclassified	Unclassified			38.3	0	2M-H	C7H10N4O2
6.68_523.2552m/z	523.2551687	6.680966667	neg	Physovenine	67539	Unclassified	Unclassified	Unclassified			38.3	0	2M-H	C14H18N2O3
6.73_427.1977m/z	427.1976727	6.72945	neg	T2 Triol	90690	Lipids and lipid-like molecules	Prenol lipids	Sesquiterpenoids	*		38.3	0	M+FA-H	C20H30O7
6.74_313.1660m/z	313.1660297	6.739933333	neg	3,11,12-Trihydroxy-1(10)-spirovetiven-2-one	92895	Lipids and lipid-like molecules	Prenol lipids	Sesquiterpenoids	*		38.3	0	M+FA-H	C15H24O4
6.81_213.1135m/z	213.1134697	6.809016667	neg	Methyl octynecarboxylate	LMFA07010964	Lipids and lipid-like molecules	Fatty Acyls	Fatty acid esters	*		38.3	0	M+FA-H	C10H16O2
6.84_593.1809m/z	593.1808828	6.842166667	neg	Charine	94423	Organic oxygen compounds	Organooxygen compounds	Carbohydrates and carbohydrate conjugates			38.3	0	M+FA-H	C18H28N8O12
6.86_527.2868m/z	527.286807	6.86375	neg	Fusicoccin H	67422	Unclassified	Unclassified	Unclassified			38.3	0	M+FA-H	C26H42O8
6.91_511.2187m/z	511.2187317	6.9072	neg	Echothiophate	HMDB0015190	Organic nitrogen compounds	Organonitrogen compounds	Quaternary ammonium salts		C06975	38.3	0	2M-H	C9H23NO3PS+
6.93_224.1293m/z	224.1293488	6.926366667	neg	2,3,4,5,6,7-Hexahydro-6,7-dimethylcyclopent[b]azepin-8(1H)-one	94239	Organoheterocyclic compounds	Azepines	Unclassified	*		38.3	0	M+FA-H	C11H17NO
7.16_169.0871m/z	169.0870922	7.1647	neg	(E,E)-2,6-Octadienal	HMDB0039844	Organooxygen compounds	Carbonyl compounds	Aldehydes			38.3	0	M+FA-H	C8H12O
7.16_565.1516m/z	565.1516336	7.1647	neg	Cucumopine	67006	Unclassified	Unclassified	Unclassified			38.3	0	2M-H	C11H13N3O6
7.18_340.1567m/z	340.1567027	7.178516667	neg	(E)-Herclavine	HMDB0030242	Phenylpropanoids and polyketides	Cinnamic acids and derivatives	Unclassified	*		38.3	0	M+FA-H	C19H21NO2
7.21_313.1197m/z	313.1197358	7.211683333	neg	N-Butyl-beta-carboline-3-carboxylate	69712	Unclassified	Unclassified	Unclassified			38.3	0	M+FA-H	C16H16N2O2
7.29_300.1819m/z	300.1818681	7.286583333	neg	N-decanoyl-homoserine lactone	LMFA08030026	Lipids and lipid-like molecules	Fatty Acyls	Fatty amides			38.3	0	M+FA-H	C14H25NO3
7.31_321.1821m/z	321.1820608	7.314366667	neg	Noralfentanil	HMDB0061010	Benzenoids	Benzene and substituted derivatives	Anilides	*		38.3	0	M+FA-H	C16H24N2O2
7.42_294.1326m/z	294.132624	7.41815	neg	3-Oxooctanoic acid glycerides	94392	Unclassified	Unclassified	Unclassified			38.3	0	M+FA-H	C11H21O6-
7.47_269.1392m/z	269.1392468	7.46525	neg	(8alpha,10beta,11beta)-3-Hydroxy-4,15-dinor-1(5)-xanthen-12,8-olide	92205	Organoheterocyclic compounds	Lactones	Gamma butyrolactones	*		38.3	0	M+FA-H	C13H20O3
7.59_507.1828m/z	507.1828042	7.58625	neg	p-Coumaryl benzoate	265094	Unclassified	Unclassified	Unclassified			38.3	0	2M-H	C16H14O3
7.65_360.1161n	405.1146069	7.65305	neg	Acalyphin	66904	Unclassified	Unclassified	Unclassified			38.3	0	M-H, M+FA-H	C14H20N2O9
7.77_479.1901m/z	479.1900538	7.770583333	neg	3-(3,4,5-Trimethoxyphenyl)propanoic acid	HMDB0030254	Phenylpropanoids and polyketides	Phenylpropanoic acids	Unclassified	*		38.3	0	2M-H	C12H16O5
7.79_437.1617m/z	437.1617467	7.793283333	neg	Mulberranol	HMDB0030797	Phenylpropanoids and polyketides	Flavonoids	Flavones	*		38.3	3.67	M-H	C25H26O7
7.87_294.9536m/z	294.9535533	7.87455	neg	(2R)-O-Phospho-3-sulfolactate	69090	Unclassified	Unclassified	Unclassified			38.3	0	M+FA-H	C3H7O9PS
7.96_461.2527m/z	461.252657	7.961816667	neg	Alfentanil	HMDB0014940	Benzenoids	Benzene and substituted derivatives	Anilides		C08005	38.3	0	M+FA-H	C21H32N6O3
8.22_241.1448m/z	241.144787	8.218516667	neg	Ethyl(E,Z)-decadienoate	67014	Lipids and lipid-like molecules	Fatty Acyls	Fatty acid esters	*	C08486	38.3	0	M+FA-H	C12H20O2
8.62_265.1422m/z	265.1421523	8.6174	neg	L-2-Amino-5-hydroxypentanoic acid	87840	Organic acids and derivatives	Carboxylic acids and derivatives	Amino acids, peptides, and analogues	*		38.3	0	2M-H	C5H11NO3
8.63_239.0598m/z	239.0598485	8.6262	neg	Cysteinyl-Histidine	85691	Organic acids and derivatives	Carboxylic acids and derivatives	Amino acids, peptides, and analogues	*		38.3	0	M-H2O-H	C9H14N4O3S
8.79_493.2810m/z	493.2809675	8.791166667	neg	16-Feruloyloxypalmitate	72077	Unclassified	Unclassified	Unclassified			38.3	0	M+FA-H	C26H40O6
9.00_567.3179m/z	567.3179213	9.004166667	neg	6α-Glucuronosylhyodeoxycholate	43177	Unclassified	Unclassified	Unclassified			38.3	0	M-H	C30H48O10
9.14_397.2234m/z	397.223378	9.14175	neg	PGH2	LMFA03010010	Lipids and lipid-like molecules	Fatty Acyls	Eicosanoids	**	C00427	38.3	0	M+FA-H	C20H32O5
9.16_463.1954m/z	463.1953985	9.155683333	neg	Cryptolepine	67463	Unclassified	Unclassified	Unclassified			38.3	0	2M-H	C16H12N2
9.27_409.1870m/z	409.1870367	9.267516667	neg	Eremopetasitenin A1	87267	Organoheterocyclic compounds	Naphthofurans	Unclassified	*		38.3	0	M+FA-H	C20H28O6
9.47_371.2079m/z	371.2078913	9.473733333	neg	cis- and trans-L-Mercapto-p-menthan-3-one	88259	Lipids and lipid-like molecules	Prenol lipids	Monoterpenoids	*		38.3	0	2M-H	C10H18OS
9.93_295.1552m/z	295.1551648	9.932583333	neg	1-Hydroxyacorenone	87285	Organic oxygen compounds	Organooxygen compounds	Carbonyl compounds	*		38.3	0	M+FA-H	C15H22O3
9.94_459.2028m/z	459.2028056	9.943016667	neg	2,4-Dihydroxytacrine	2626	Unclassified	Unclassified	Unclassified			38.3	0	2M-H	C13H14N2O2
0.67_173.0821m/z	173.0820772	0.6701	neg	1-Oxa-2-oxo-3-methylcycloheptane	68738	Unclassified	Unclassified	Unclassified			38.2	0	M+FA-H	C7H12O2
0.67_625.1871m/z	625.1870745	0.6701	neg	Avenanthramide 1f	86154	Phenylpropanoids and polyketides	Cinnamic acids and derivatives	Hydroxycinnamic acids and derivatives	*		38.2	0	2M-H	C17H15NO5
0.69_239.0399m/z	239.0399383	0.692166667	neg	Pectin	HMDB0003402	Organic oxygen compounds	Organooxygen compounds	Carbohydrates and carbohydrate conjugates	*	C08348	38.2	0	M+FA-H	C6H10O7
0.71_168.0779m/z	168.0778593	0.714216667	neg	Nalpha-Methylhistidine	65951	Unclassified	Unclassified	Unclassified			38.2	0	M-H	C7H11N3O2
0.71_871.3523m/z	871.3522583	0.714216667	neg	Flupenthixol	HMDB0015013	Organoheterocyclic compounds	Benzothiopyrans	1-benzothiopyrans			38.2	0	2M-H	C23H27F3N2OS
0.84_367.1053m/z	367.1052536	0.841733333	neg	(+/-)-3-[(2-methyl-3-furyl)thio]-2-butanone	HMDB0032401	Organosulfur compounds	Thioethers	Aryl thioethers	*		38.2	0.0787	2M-H	C9H12O2S
0.86_222.0984m/z	222.098357	0.86335	neg	Anthopleurine	71488	Unclassified	Unclassified	Unclassified			38.2	0	M+FA-H	C7H15NO4
0.86_388.9444m/z	388.9443579	0.86335	neg	Phosphoribosyl pyrophosphate	HMDB0000280	Organic oxygen compounds	Organooxygen compounds	Carbohydrates and carbohydrate conjugates	**	C00119	38.2	0	M-H	C5H13O14P3
0.90_188.0566m/z	188.056615	0.904683333	neg	2-Hydroxymethylclavam	71631	Unclassified	Unclassified	Unclassified			38.2	0	M+FA-H	C6H9NO3
0.93_340.0675m/z	340.0675178	0.925866667	neg	Caffeoyl aspartic acid	86165	Organic acids and derivatives	Carboxylic acids and derivatives	Amino acids, peptides, and analogues			38.2	0	M+FA-H	C13H13NO7
1.06_173.1184m/z	173.1183773	1.055866667	neg	(R)-1-Octen-3-ol	HMDB0031299	Lipids and lipid-like molecules	Fatty Acyls	Fatty alcohols	*	C14272	38.2	0	M+FA-H	C8H16O
1.14_265.0556m/z	265.0556192	1.1382	neg	1-Hydroxypentane-1,2,5-tricarboxylate	71254	Unclassified	Unclassified	Unclassified			38.2	0	M+FA-H	C8H12O7
1.18_202.0722m/z	202.0721671	1.180366667	neg	Tiglylglycine	HMDB0000959	Organic acids and derivatives	Carboxylic acids and derivatives	Amino acids, peptides, and analogues	*		38.2	0	M+FA-H	C7H11NO3
1.18_217.0353m/z	217.0353117	1.180366667	neg	2-Heptenedioic acid, 4-oxo-	2194	Unclassified	Unclassified	Unclassified			38.2	0	M+FA-H	C7H8O5
1.20_427.0861m/z	427.0861117	1.202333333	neg	2-Hydroxy-6-oxonona-2,4-diene-1,9-dioate	63524	Unclassified	Unclassified	Unclassified			38.2	0	2M-H	C9H10O6
1.25_410.9399m/z	410.939895	1.246333333	neg	Etidronic acid	HMDB0015210	Organic acids and derivatives	Organic phosphonic acids and derivatives	Bisphosphonates		C07736	38.2	0	2M-H	C2H8O7P2
1.29_170.0580n	151.0401369	1.290916667	neg	3,4-Dihydroxyphenylglycol	HMDB0000318	Benzenoids	Phenols	Benzenediols	**	C05576	38.2	0	M-H2O-H, M-H	C8H10O4
1.46_249.0242m/z	249.0242494	1.459966667	neg	Oxaloglutarate	3339	Unclassified	Unclassified	Unclassified			38.2	0	M+FA-H	C7H8O7
1.47_164.0354m/z	164.0354438	1.472133333	neg	1,2-Diacylglycerol-LD-PI-pool	HMDB0062270	Benzenoids	Benzene and substituted derivatives	Unclassified			38.2	0	M+FA-H	C7H5NO
1.53_186.0773m/z	186.0773078	1.534483333	neg	Ethosuximide	HMDB0014731	Organoheterocyclic compounds	Pyrrolidines	Pyrrolidones		C07505	38.2	0	M+FA-H	C7H11NO2
1.55_306.0824m/z	306.0823809	1.553483333	neg	Hymexazol N-glucoside	68845	Unclassified	Unclassified	Unclassified			38.2	0	M+FA-H	C10H15NO7
1.55_355.1240m/z	355.1239744	1.553483333	neg	Galactopinitol B	90621	Organic oxygen compounds	Organooxygen compounds	Carbohydrates and carbohydrate conjugates			38.2	0	M-H	C13H24O11
1.72_300.9863m/z	300.9863318	1.721216667	neg	Ascorbic acid 2-sulfate	HMDB0060649	Organoheterocyclic compounds	Dihydrofurans	Furanones	*		38.2	0.965	M+FA-H	C6H8O9S
1.81_220.9763m/z	220.9763022	1.808733333	neg	3-Sulfomuconate	66487	Unclassified	Unclassified	Unclassified			38.2	0	M-H	C6H6O7S
1.86_304.1517m/z	304.1516987	1.86075	neg	Glutaminylisoleucine	HMDB0028800	Organic acids and derivatives	Carboxylic acids and derivatives	Amino acids, peptides, and analogues	*		38.2	0	M+FA-H	C11H21N3O4
1.91_177.0228m/z	177.0227798	1.911116667	neg	THTC	68817	Unclassified	Unclassified	Unclassified			38.2	0	M+FA-H	C5H8O2S
10.02_393.2499m/z	393.2499049	10.0168	neg	4-ethylamino-6-isopropylamino-1,3,5-triazin-2-ol	HMDB0062766	Organoheterocyclic compounds	Triazines	1,3,5-triazines	**	C06552	38.2	0	2M-H	C8H15N5O
10.46_589.1725m/z	589.1725458	10.45966667	neg	4-(4-Hydroxyphenyl)-2-butanone O-[2-galloyl-6-cinnamoylglucoside]	HMDB0039313	Organic oxygen compounds	Organooxygen compounds	Carbohydrates and carbohydrate conjugates			38.2	0	M-H2O-H	C32H32O12
10.58_315.1968m/z	315.1967535	10.583	neg	17beta-Methylestra-1,3,5(10)-trien-3-ol	70515	Unclassified	Unclassified	Unclassified			38.2	0	M+FA-H	C19H26O
13.57_255.2331m/z	255.2330572	13.5693	neg	(E)-2-Octen-1-ol	HMDB0031296	Lipids and lipid-like molecules	Fatty Acyls	Fatty alcohols	*		38.2	0	2M-H	C8H16O
13.77_281.2486m/z	281.2485607	13.77343333	neg	7Z,9Z-Heptadecadiene	97561	Lipids and lipid-like molecules	Fatty Acyls	Hydrocarbons			38.2	0	M+FA-H	C17H32
13.90_407.1873m/z	407.1872906	13.89845	neg	Dimethylenetriurea	66392	Unclassified	Unclassified	Unclassified			38.2	0	2M-H	C5H12N6O3
2.03_155.0827m/z	155.0826792	2.025516667	neg	Glycyl-Valine	85763	Organic acids and derivatives	Carboxylic acids and derivatives	Amino acids, peptides, and analogues	*		38.2	0	M-H2O-H	C7H14N2O3
2.07_187.0613m/z	187.0613008	2.070366667	neg	Mesifurane	HMDB0034230	Organoheterocyclic compounds	Dihydrofurans	Furanones			38.2	0	M+FA-H	C7H10O3
2.69_187.0613m/z	187.0613138	2.692466667	neg	Homofureanol	89506	Organoheterocyclic compounds	Dihydrofurans	Furanones			38.2	0	M+FA-H	C7H10O3
2.93_166.0146m/z	166.0146365	2.926066667	neg	2-Amino-3-carboxymuconic acid 6-semialdehyde	350	Unclassified	Unclassified	Unclassified			38.2	0	M-H2O-H	C7H7NO5
3.42_238.9690m/z	238.9689809	3.417666667	neg	3-mercaptopyruvate	4200	Organic acids and derivatives	Keto acids and derivatives	Alpha-keto acids and derivatives	**	C00957	38.2	0	2M-H	C3H4O3S
3.42_309.1094m/z	309.109434	3.417666667	neg	(1R,6S)-6-Amino-5-oxocyclohex-2-ene-1-carboxylate	73385	Unclassified	Unclassified	Unclassified			38.2	0	2M-H	C7H9NO3
3.58_376.0652m/z	376.065186	3.5753	neg	Deoxyadenosine monophosphate	3461	Nucleosides, nucleotides, and analogues	Purine nucleotides	Purine deoxyribonucleotides	**	C00360	38.2	0	M+FA-H	C10H14N5O6P
3.71_355.0673m/z	355.067273	3.708366667	neg	Mono-trans-p-coumaroylmesotartaric acid	HMDB0029783	Phenylpropanoids and polyketides	Cinnamic acids and derivatives	Hydroxycinnamic acids and derivatives			38.2	0	M+FA-H	C14H14O8
3.87_383.0601m/z	383.0601367	3.868533333	neg	2-O-Feruloylhydroxycitric acid	95056	Organic acids and derivatives	Carboxylic acids and derivatives	Tetracarboxylic acids and derivatives			38.2	0	M-H	C16H16O11
3.94_625.1018m/z	625.1017631	3.9351	neg	Isoxathion	70175	Unclassified	Unclassified	Unclassified			38.2	0	2M-H	C13H16NO4PS
3.96_459.1968m/z	459.1968235	3.957583333	neg	N- (3-TRIFLUOROMETHYLPHENYL)PIPERAZINE (TFMPP)	44359	Unclassified	Unclassified	Unclassified			38.2	0	2M-H	C11H13F3N2
3.96_469.0858m/z	469.0858078	3.957583333	neg	3,4-Dihydro-7-methoxy-2-methylene-3-oxo-2H-1,4-benzoxazine-5-carboxylic aci	69053	Unclassified	Unclassified	Unclassified			38.2	0	2M-H	C11H9NO5
4.00_314.1614m/z	314.1613926	4.002233333	neg	N-(3-oxo-decanoyl)-homoserine lactone	LMFA08030017	Lipids and lipid-like molecules	Fatty Acyls	Fatty amides			38.2	0	M+FA-H	C14H23NO4
4.00_497.1311m/z	497.1311256	4.002233333	neg	(-)-epicatechin-3'-O-glucuronide	HMDB0029164	Phenylpropanoids and polyketides	Flavonoids	Flavonoid glycosides	*		38.2	0	M+FA-H	C21H24O11
4.07_384.1763m/z	384.1763195	4.069216667	neg	Xamoterol	69212	Unclassified	Unclassified	Unclassified			38.2	0	M+FA-H	C16H25N3O5
4.09_182.0282m/z	182.0282451	4.091783333	neg	Thiobenzamide	71171	Unclassified	Unclassified	Unclassified			38.2	0	M+FA-H	C7H7NS
4.11_411.1874m/z	411.1873946	4.113883333	neg	(1S,2S,4R,8S)-p-Menthane-1,2,8,9-tetrol 2-glucoside	HMDB0039975	Lipids and lipid-like molecules	Prenol lipids	Terpene glycosides	*		38.2	0	M+FA-H	C16H30O9
4.16_403.1976m/z	403.197552	4.15895	neg	1-Octen-3-ol-3-o-beta-D-xylopyranosyl(1->6)-beta-D-glucopyranoside	71817	Unclassified	Unclassified	Unclassified			38.2	0	M-H2O-H	C19H34O10
4.18_284.1253m/z	284.1253147	4.181333333	neg	HC Blue No. 2	73129	Unclassified	Unclassified	Unclassified			38.2	0	M-H	C12H19N3O5
4.32_443.1466m/z	443.1465807	4.3224	neg	cis-ACCP	45355	Unclassified	Unclassified	Unclassified			38.2	0	2M-H	C7H15N2O4P

5.50_435.1874m/z	-1.456400067	7051.527406	5603.872967	6637.695732	6090.74519	6005.686156	7041.420462	7598.115452	7602.903992	7006.187348
5.50_547.1459m/z	0.347557041	10134.25729	9104.328386	7525.185939	3340.302407	3538.791835	3955.099198	327.0441502	453.6307288	556.5138214
5.52_495.1106m/z	-2.380026803	2139.520543	1877.867141	1715.5332	905.3490386	1224.878578	776.0635339	2053.116499	1700.531794	1861.593726
5.52_675.2250m/z	2.146378069	1713.609179	1190.509902	2052.433714	1719.996015	3052.42913	2080.78733	1131.82145	1574.594523	1970.43972
5.67_523.2553m/z	-1.665177887	2347.335357	2032.626962	2234.115013	2578.344239	2891.581427	2812.497198	2154.22481	1857.240487	2404.478358
5.71_519.1287m/z	-1.920967787	4597.409956	4157.174565	3798.344415	8951.743987	8180.201837	8507.798559	5576.780993	6517.473651	7832.983574
5.81_490.1676m/z	-3.545979956	2699.483756	2695.311858	2714.085102	7708.128632	7767.812867	6836.006677	5539.736683	4734.964588	4900.646387
5.92_269.1396m/z	0.711764897	5971.327596	6620.092451	6549.716665	5946.360733	7042.327938	6332.061229	4206.855242	5072.077884	4870.841942
5.92_294.1711m/z	0.264650732	1662.931594	1708.976577	1841.056976	718.5059073	870.4358896	560.2220231	739.8943645	1003.773097	1220.502813
5.94_239.0021m/z	0.447739122	3837.744369	4566.036322	5377.355569	206.9674569	278.1108866	209.2524694	491.7164992	450.3590238	529.6448718
5.94_369.1919m/z	0.098059	414.1680171	381.7611283	673.3494975	1063.571951	1497.614937	1100.108361	124.3054614	365.5415143	493.3888745
5.95_567.2040m/z	2.776824398	2871.041449	3389.140132	3216.923357	915.0668376	1685.115224	1387.741992	3212.062454	3942.474268	3661.317381
6.09_257.1397m/z	1.226193708	2122.279394	2579.412269	2509.606241	2203.458702	2155.666613	2048.64979	1676.172134	1696.045922	1860.993519
6.32_185.1184m/z	0.290149633	1914.155641	2253.003723	2143.885252	2857.975825	2974.055473	2675.054946	2019.094982	2087.25423	2219.493339
6.37_423.1664m/z	0.883725066	1578.719792	1811.866109	1715.37532	528.5942413	626.8900432	483.5908242	799.6370504	1104.221037	853.6502731
6.37_479.1525m/z	5.261844723	9456.331974	9429.958873	10401.04235	11091.23423	11042.6435	11385.40434	8484.288154	9581.642602	9155.440127
6.39_163.0402m/z	1.134789608	970.8977202	953.3911389	1061.548397	839.9877209	988.7406381	905.8630718	1090.894019	1044.943705	1002.268262
6.39_364.2265m/z	1.796616923	6483.252285	6546.583845	6668.307906	6114.036741	6980.561375	6666.649294	7846.877905	6589.553974	6753.511843
6.39_391.2454m/z	5.695281882	4714.420819	4725.277848	4688.430332	4741.85698	5220.049762	5157.705336	5988.6388	4818.227753	4861.554033
6.39_461.2184m/z	-2.323279871	4054.38436	4775.674086	5601.478899	5586.19168	6110.768503	5944.873754	4691.858416	4961.259336	4798.250243
6.41_545.1309m/z	1.410450367	16522.62489	18337.17342	19168.59424	11287.88016	10966.09369	10318.34897	4389.141002	4092.45281	3668.226272
6.42_304.1227m/z	7.541289866	10306.0666	11596.14394	11838.01862	18929.47826	18091.53806	17125.49814	11872.31378	11242.19223	12007.19897
6.46_473.0724m/z	-0.422340217	6575.396365	6047.079929	5905.771126	6023.867342	5801.318277	6901.381939	8904.383847	7147.318192	7841.259512
6.52_289.1659m/z	1.146363358	1191.134246	1237.015758	1199.13665	1999.535643	1897.129728	2002.630975	587.8331361	721.0418251	764.5608475
6.57_363.1547m/z	3.272746191	604.8465792	899.3196585	769.3888915	2076.998716	1718.461744	2075.673327	733.1058324	736.1727064	946.8844853
6.68_523.2552m/z	-1.983200905	2072.984439	2413.197236	2042.157907	2358.826121	1872.908691	1687.258067	1765.835272	2443.613758	2044.198335
6.73_427.1977m/z	0.828071121	12648.14739	14398.61314	13299.95369	15925.4246	15257.83318	14497.13546	13683.98619	15613.28804	15285.76865
6.74_313.1660m/z	1.370842527	2481.350864	2735.746362	3228.818539	4458.735851	4673.119404	4117.952511	1882.808567	2528.074644	2341.489507
6.81_213.1135m/z	1.410109976	1359.823731	1312.19835	1209.775688	1274.43075	1351.166771	1550.094237	1502.66698	1216.580917	1176.009669
6.84_593.1809m/z	0.021084413	6394.768398	5770.998172	5418.758116	7839.629923	8551.305989	7974.707263	8509.744825	7554.154573	6961.841938
6.86_527.2868m/z	1.318429296	6408.445313	6550.468481	5466.112961	6315.76189	8348.3861	6699.108948	5894.574478	7196.915531	5771.368344
6.91_511.2187m/z	-2.429243712	348.4583878	435.7176406	454.8007057	4066.01651	3386.875544	3189.25116	303.9374672	658.8837117	309.4957638
6.93_224.1293m/z	0.735356501	893.1936031	997.8773326	893.3077659	1150.735274	1208.285306	959.1767925	697.5519355	978.2824327	925.6277524
7.16_169.0871m/z	0.598757152	636.7128535	764.240172	659.3164034	619.7089699	630.4461061	671.5101231	756.6188771	904.9492366	576.9227675
7.16_565.1516m/z	-3.462306784	3043.432612	4159.391726	3915.768147	4617.689687	3675.904382	4617.31411	3433.963084	4531.558791	3295.117832
7.18_340.1567m/z	4.306035348	4023.149045	4902.051396	4892.920214	7711.792251	7630.530381	7788.780584	3673.04878	5365.785152	4155.365039
7.21_313.1197m/z	1.324801828	1683.295496	2195.619618	1983.692424	2544.850976	2433.316033	2487.424871	2279.61339	2683.003219	2312.829997
7.29_300.1819m/z	0.868221503	676.7411804	646.1374329	682.5314584	1374.990037	1295.486357	1203.817673	742.786585	760.4375561	958.565576
7.31_321.1821m/z	0.289168493	1092.723882	1636.95061	1332.647105	1653.182064	1471.701904	1349.038853	1025.778699	1139.85932	1364.437466
7.42_294.1326m/z	2.439323955	1937.655317	1696.638332	2080.940545	3462.692482	2927.84102	3099.047587	1731.43004	1819.500095	1894.130187
7.47_269.1392m/z	-0.894642449	482.5552154	585.0839519	451.2645546	983.2485351	1103.688924	878.3809795	424.4015612	419.0285858	487.1247502
7.59_507.1828m/z	2.936024669	3556.338423	3448.255971	3667.429863	4465.214358	3811.905981	3962.226885	3927.112925	4443.467747	3809.704456
7.65_360.1161n	-2.120926328	7976.596332	8732.808308	8909.041193	4314.4134	3752.668095	4518.720231	6877.183898	7189.85859	7246.045095
7.77_479.1901m/z	-4.616715222	4241.329247	4259.84475	4676.305637	1934.416049	1878.31896	2067.694732	2727.255595	3397.15017	2934.995686
7.79_437.1617m/z	2.670115969	58735.87204	63518.96606	66513.48903	83376.31044	78195.37465	79727.47424	60346.2567	72921.15936	68346.39286
7.87_294.9536m/z	2.044227161	2070.045392	2162.079925	2280.136369	2165.90039	2336.655954	2278.475256	2430.945495	2303.886902	2159.683156
7.96_461.2527m/z	2.078670223	6765.640178	6280.759832	7685.822274	5301.008491	4677.719106	5305.310626	5155.553829	5659.190673	5307.287833
8.22_241.1448m/z	1.296419833	838.4490992	865.2404334	1042.010152	1342.590744	1436.126272	1409.617048	820.2150171	551.5097899	737.296977
8.62_265.1422m/z	6.17045865	1670.601626	1765.149868	2047.677639	1879.339615	1769.291328	1887.193206	1993.73752	1638.182476	1416.82766
8.63_239.0598m/z	-3.764971032	3629.065663	3155.672974	3079.31656	3067.168175	3064.881457	3458.208575	3472.432663	3246.341393	3259.189959
8.79_493.2810m/z	0.614820691	4780.02975	5013.426703	5773.218129	6765.012526	7362.59894	8280.145374	5843.620274	6817.150145	6400.002401
9.00_567.3179m/z	0.791722943	13931.46708	17511.38698	18691.55088	1670.71076	1793.292684	2077.382528	15098.14842	15739.63061	16268.69153
9.14_397.2234m/z	0.570593284	11377.69582	11946.77555	12871.47271	3805.689113	3816.167609	3963.366775	7530.97067	7745.346494	7829.949752
9.16_463.1954m/z	5.553936232	3766.81619	4537.072166	4110.888392	1967.882514	1717.66287	1754.547659	2708.034685	2621.018768	3207.162455
9.27_409.1870m/z	0.673257088	8999.322434	8881.686993	8828.70227	5367.630333	5207.977687	4994.811923	6655.345122	7959.132601	7366.177467
9.47_371.2079m/z	-1.355914935	885.0583835	920.4032217	999.2862008	1310.837981	653.5246199	1037.686364	413.6690702	611.9870896	526.0646363
9.93_295.1552m/z	0.269304842	1362.081761	1428.342113	1279.568868	1358.540355	1259.102628	1394.966383	1343.756697	1365.041719	1329.228276
9.94_459.2028m/z	-2.115079448	3116.197081	1794.87057	2922.775302	5132.245939	1721.992562	2154.095955	1963.93666	1732.301147	3945.61643
0.67_173.0821m/z	1.129703384	22.88398198	93.26463683	87.83967833	21.79335465	46.69697112	79.0710792	153.4285614	39.1131115	68.48304522
0.67_625.1871m/z	6.876194133	2863.463888	3248.831211	3696.37223	4038.250159	3163.189698	2643.816188	2456.426038	1644.584915	2318.469514
0.69_239.0399m/z	-4.727018293	1080.383974	943.426616	1749.269362	2079.790709	3676.735694	2697.559209	2322.980195	2530.390656	3385.457632
0.71_168.0779m/z	0.053932109	932.573196	669.5155909	831.6566879	1068.207022	859.2372253	993.5106626	841.6312957	696.3342508	551.0102814
0.71_871.3523m/z	0.339835032	8971.009685	7667.833655	3899.058554	3627.47182	7478.10398	6184.683617	13912.19011	8326.233657	9493.873341
0.84_367.1053m/z	2.523218086	15453.01876	14530.36523	17526.06828	12430.19326	14818.36523	11900.74058	13752.35788	13999.3653	14406.70021
0.86_222.0984m/z	0.260622168	1004.765876	905.8257391	1172.371021	1334.519159	1060.666562	1514.232515	1351.965657	1283.163382	810.015965
0.86_388.9444m/z	-0.463280708	2929.621001	2439.66343	3234.134331	1477.693093	2208.16949	2343.909527	2565.598212	1751.222321	2359.33435
0.90_188.0566m/z	1.180982302	1254.200564	1715.199441	1574.167554	1951.173756	1239.863082	2006.378974	1485.01465	1662.956705	1175.861883
0.93_340.0675m/z	0.383560886	3314.449834	2886.138577	2706.526668	0.001403199	0.001403199	0.001403199	2742.971103	3334.357708	2230.692053
1.06_173.1184m/z	0.463127878	256.8774725	210.0328788	165.3478936	149.8813747	110.1895797	190.9252321	237.7401844	238.3278895	142.4258925
1.14_265.0556m/z	-4.028114959	6119.989583	3580.613333	3861.965137	5042.110557	5142.694041	5933.241207	3257.416324	5220.861896	5364.441756
1.18_202.0722m/z	0.45241955	1158.270821	1015.541618	1212.088455	752.3798445	416.3549724	1166.173682	650.4077714	883.9964613	544.8920837
1.18_217.0353m/z	-0.375087952	475.8501284	372.6304369	283.1169337	378.7673354	193.0955387	443.727233	159.3481667	322.1891555	95.737474
1.20_427.0861m/z	-4.877268872	992.0011803	959.7077371	1247.175492	997.3494046	835.1038012	988.8477592	1215.864549	1099.550743	1088.457095
1.25_410.9399m/z	-4.564256437	817.2482828	836.3933115	663.8185339	2249.934706	1400.009322	1974.723818	1218.900663	1566.364391	1089.24356
1.29_170.0580n	0.407291106	5794.759786	5577.993062	5692.761278	5758.826305	5771.174683	6185.232821	6324.311353	5567.212738	5792.532606
1.46_249.0242m/z	-4.68613817	2626.591723	3624.862869	3043.185322	4214.673702	3674.844055	3877.100444	4465.24495	2722.28268	3671.44419
1.47_164.0354m/z	1.067982887	1329.204267	1292.367758	1280.767641	1120.513494	1002.89336	1063.912049	1558.804986	1331.874971	1562.149349
1.53_186.0773m/z	0.895501419	1664.170261	1761.743919	1725.487666	1734.110797	1275.437845	1393.002496	1400.352128	1576.826031	1531.266706
1.55_306.0824m/z	-2.580674405	346.3047629	307.5034342	182.1736899	743.1585054	508.7091007	500.9273032	215.6092363	433.4820328	239.5899601
1.55_355.1240m/z	-1.715013414	422.2792954	272.6760859	347.7861043	615.8547853	537.9699408	694.1608969	307.591512	574.4186249	395.0990556
1.72_300.9863m/z	-3.023042388	10449.32513	11518.48924	10403.89756	14229.72631	13377.84354	13221.01721	12575.59049	11652.70454	14428.72785
1.81_220.9763m/z	0.698518272	5354.419883	5143.553928	5652.149994	2090.00716	2116.50518	2209.339015	3049.770829	3545.438523	3186.174575
1.86_304.1517m/z	1.117839971	266.9725905	254.6839059	383.2602307	1937.665765	1923.639517	1502.41075	995.0215044	778.9176152	856.4058144
1.91_177.0228m/z	0.579277341	561.2453713	607.7658018	379.2088655	462.9929167	601.4656944	586.5156492	567.3188458	511.6733389	464.3389585
10.02_393.2499m/z	4.720565041	963.4371001	304.9105227	405.8103697	587.2402447	1986.927118	252.4176226	356.025977	276.6627332	454.4444334
10.46_589.1725m/z	1.661393528	7729.949498	7140.440596	9217.143682	8238.368757	8652.120601	6710.502759	7508.784869	9416.310862	9198.746297
10.58_315.1968m/z	0.685462401	1358.138794	1682.535645	1815.831394	1223.720372	1985.92026	1461.684455	1379.572259	1079.431418	1320.142639
13.57_255.2331m/z	0.403315696	2759.092525	2601.76998	2847.689754	1852.632728	1843.72052	2151.36072	2200.717238	2681.274927	2396.550236
13.77_281.2486m/z	-0.182659859	7796.818393	8536.516137	7007.021464	6287.360543	5479.841891	6049.849349	8255.818344	7878.837524	7111.488785
13.90_407.1873m/z	0.956581859	2627.518549	1561.510578	1699.925656	991.7731819	1073.220722	775.0706924	1534.278543	1739.009064	1700.520978
2.03_155.0827m/z	0.448140596	676.3453515	540.7710101	637.2740883	413.33442	384.5719668	381.4543864	387.3158616	473.487541	350.0973011
2.07_187.0613m/z	0.730621823	368.817294	359.0049773	411.035762	662.1997465	624.5973617	689.7404613	558.2164646	440.8661768	539.0420936
2.69_187.0613m/z	0.821862019	743.4034447	702.0159027	545.7521481	383.3378633	340.5146783	274.29124	214.3878191	372.2127711	309.9900109
2.93_166.0146m/z	0.29869692	93.65214388	89.67035132	130.7502866	163.0676065	179.8872051	138.8426121	142.6012906	101.3845734	85.84723728
3.42_238.9690m/z	0.113836674	1700.283214	1651.548176	1760.846235	947.5453452	894.2447403	897.1977997	1240.432855	1345.877779	1029.753155
3.42_309.1094m/z	0.722828229	147.4235236	132.7642059	234.148865	368.3318813	199.9222888	264.4377585	221.5363445	301.0126008	309.9025848
3.58_376.0652m/z	-3.584423525	2951.511374	3183.797058	3323.202789	50.04798838	41.10062984	80.06998241	2779.396729	3359.794045	3131.146316
3.71_355.0673m/z	0.653936669	1361.746766	1390.075087	1375.672118	557.6752202	547.6399476	382.7859883	837.5483214	1009.877456	1022.600079
3.87_383.0601m/z	-4.812242115	410.6582573	608.6718077	662.241358	257.0555158	185.0629288	337.495198	367.5871299	459.506962	328.6536381
3.94_625.1018m/z	2.408966934	859.968849	592.9938556	1543.40469	0.001403199	0.001403199	0.001403199	776.6227737	1183.995084	688.1014189
3.96_459.1968m/z	-4.489746011	532.5980989	670.840161	599.7173922	1062.250748	1024.282435	806.2892402	837.8115212	905.3830872	554.6639061
3.96_469.0858m/z	-6.510458558	1049.380041	930.1824618	1009.813522	1303.925485	1271.981909	1230.287773	1265.340884	974.2109375	992.2626093
4.00_314.1614m/z	1.789000549	251.5478214	320.4092047	323.406587	744.7355193	637.6778871	679.2408438	363.8569967	481.9275511	417.9580412
4.00_497.1311m/z	2.346963769	4151.7763	4436.532353	4243.822733	625.2288273	369.7238152	240.1235024	3045.011278	3302.715983	3627.038173
4.07_384.1763m/z	-3.845540219	303.672697	235.1111063	335.1758136	871.5614046	670.9589632	812.6509347	338.1792909	353.7853317	372.0823636
4.09_182.0282m/z	0.890007725	0.001403199	0.001403199	15.95829874	614.0945338	485.949336	512.2397759	0.001403199	18.89599587	20.13074303
4.11_411.1874m/z	0.571179648	650.9403843	728.5799482	729.3504866	456.6127126	503.8113248	403.1307238	623.3061829	578.5846825	580.9171121
4.16_403.1976m/z	0.463887882	4617.000715	5303.863022	5130.246043	1705.504091	1358.979004	1487.923355	3853.192009	3513.501545	3389.155026
4.18_284.1253m/z	0.422341329	1398.422444	984.8460188	819.7545222	996.8020311	855.5618811	1066.891253	1345.282116	750.4430542	1086.473035
4.32_443.1466m/z	-0.067761968	421.6665527	207.1879478	398.9829796	797.2264693	618.1112238	669.7642677	436.2622656	438.1890297	544.4157409

4.34_373.1139m/z	373.1139125	4.344733333	neg	1-(2-Hydroxyethyl)-2-hydroxymethyl-5-nitroimidazole	693	Unclassified	Unclassified	Unclassified			38.2	0	2M-H	C6H9N3O4	6.889206214	639.7816	682.2969831	920.3573531	1130.085321	833.7967517	1043.901907	909.2298925	860.4125811	868.0102578
4.39_299.0791m/z	299.0791289	4.3893	neg	Fluoroureidopropionic acid	688	Unclassified	Unclassified	Unclassified			38.2	0	2M-H	C4H7FN2O3	-5.782581976	4261.985131	5566.633796	5920.95718	6987.970863	6285.892325	5865.076682	6220.586432	6879.094794	7172.035141
4.39_547.1814m/z	547.1813505	4.3893	neg	(1xi,3S)-1,2,3,4-Tetrahydro-1-methyl-beta-carboline-1,3-dicarboxylic acid	90453	Alkaloids and derivatives	Harmala alkaloids	Unclassified			38.2	0	2M-H	C14H14N2O4	-3.806908928	3181.533607	3144.717805	4358.004624	2671.914404	2204.772118	1528.146509	2676.115524	5397.713263	4573.034917
4.41_179.0350m/z	179.0350234	4.41115	neg	6E-Octene-2,4-diynoic acid	LMFA01030743	Lipids and lipid-like molecules	Fatty Acyls	Fatty Acids and Conjugates			38.2	0	M+FA-H	C8H6O2	0.306518656	383.5658412	299.2846445	328.0196682	213.4784777	153.0845544	218.6790784	245.2636642	237.3092808	256.2926668
4.41_417.1647m/z	417.1647433	4.41115	neg	Clavamycin F	71660	Unclassified	Unclassified	Unclassified			38.2	0	M+FA-H	C15H24N4O7	5.484820519	676.6594688	976.3409047	601.3821685	1595.502365	1627.419508	1086.837739	1018.158223	644.4216919	1005.299669
4.41_421.1366m/z	421.1366308	4.41115	neg	Riboflavin	HMDB0000244	Organoheterocyclic compounds	Pteridines and derivatives	Alloxazines and isoalloxazines	***	C00255	38.2	0	M+FA-H	C17H20N4O6	0.381572632	1838.704858	2277.607034	1795.946766	3723.099216	4016.23433	4155.590999	1552.814526	1886.676007	1869.465108
4.43_169.0144m/z	169.0143645	4.432683333	neg	Gallic acid	HMDB0005807	Benzenoids	Benzene and substituted derivatives	Benzoic acids and derivatives	***	C01424	38.2	0	M-H	C7H6O5	0.692180649	691.1949664	433.7365778	330.3852303	0.001403199	16.83022328	0.001403199	462.5162563	373.498703	303.3204572
4.48_445.0822m/z	445.0822389	4.477166667	neg	(4aS,5aS,12aS)-7-chloro-4-(dimethylamino)-3,6,10,12,12a-pentahydroxy-1,11-	HMDB0144089	Phenylpropanoids and polyketides	Tetracyclines	Unclassified	*		38.2	28.3	M-H2O-H	C21H21ClN2O8	3.095726755	6721.333085	6133.991418	6369.475275	13932.03175	11940.76401	12623.52535	6792.127216	7035.958624	6595.579388
4.48_581.0573m/z	581.0573406	4.477166667	neg	Parathion	58039	Organic acids and derivatives	Organic thiophosphoric acids and derivatives	Thiophosphoric acid esters	*	C06604	38.2	0	2M-H	C10H14NO5PS	-2.479602244	1567.899442	1944.790472	1692.9897	5030.153368	4411.187264	4405.921597	1715.892175	1427.143513	2294.690659
4.52_289.1659m/z	289.1659206	4.521733333	neg	4-methyl-dodecanedioic acid	LMFA01170012	Lipids and lipid-like molecules	Fatty Acyls	Fatty Acids and Conjugates			38.2	0	M+FA-H	C13H24O4	1.058486218	5819.346305	6036.122101	5697.249021	6079.975025	5455.373455	5943.271863	4465.325091	5063.621924	5214.299936
4.52_341.1243m/z	341.1243351	4.521733333	neg	Marasmal	HMDB0036867	Organoheterocyclic compounds	Naphthofurans	Unclassified	*		38.2	0	M+FA-H	C15H20O6	0.485881377	798.5435428	783.4233513	809.5392482	620.9871271	486.057178	534.4052791	748.7324707	873.7312832	928.8204945
4.54_197.0821m/z	197.0820985	4.54425	neg	2-Propenyl 2,4-hexadienoate	LMFA07010740	Lipids and lipid-like molecules	Fatty Acyls	Fatty acid esters	*		38.2	0	M+FA-H	C9H12O2	1.091554568	675.2223201	795.9279966	812.706524	735.6000599	737.9334968	723.3380259	858.542249	811.0971637	929.3197607
4.59_197.1185m/z	197.1185082	4.589283333	neg	(-)-trans-Carveol	HMDB0003450	Lipids and lipid-like molecules	Prenol lipids	Monoterpenoids	*	C00964	38.2	0	M+FA-H	C10H16O	1.25059803	287.0169625	295.3409369	239.9635541	413.2607388	393.9339438	418.2778118	324.2968403	298.6024714	343.1456524
4.59_311.1134m/z	311.1133989	4.589283333	neg	3,4-dimethyl-5-carboxyethylene-2-furanpentanoic acid	LMFA01150064	Lipids and lipid-like molecules	Fatty Acyls	Fatty Acids and Conjugates			38.2	0	M+FA-H	C14H18O5	-0.855453527	1835.367765	2235.899907	1834.097292	2440.431819	1955.259396	2262.644164	1933.085073	2020.967627	2132.271608
4.68_463.0884m/z	463.0884041	4.682483333	neg	Quercetin 3-galactoside	HMDB0030775	Phenylpropanoids and polyketides	Flavonoids	Flavonoid glycosides	***	C10073	38.2	0	M-H	C21H20O12	0.440547631	1100.475445	840.5355514	1072.406399	4103.026163	3328.884178	4119.842683	2074.062876	2681.857494	1588.356828
4.75_268.0172m/z	268.0172242	4.747466667	neg	FR	HMDB0303046	Organic acids and derivatives	Carboxylic acids and derivatives	Amino acids, peptides, and analogues	*		38.2	0	M+FA-H	Fr	-3.204174784	1801.341871	1915.435573	1728.503264	739.754133	806.6915638	767.7158232	1440.821641	1795.099975	1738.797721
4.75_377.1821m/z	377.182057	4.747466667	neg	(1S,2S,4R)-1,8-Epoxy-p-menthan-2-ol glucoside	HMDB0033110	Organic oxygen compounds	Organooxygen compounds	Carbohydrates and carbohydrate conjugates	*		38.2	0	M+FA-H	C16H28O7	1.0562993	1252.007113	1128.685104	1615.913116	776.1545905	971.7364468	789.8251633	1288.341308	1550.34586	1136.169776
4.79_699.0726m/z	699.0725571	4.792316667	neg	N-Phospho-D-lombricine	63424	Unclassified	Unclassified	Unclassified			38.2	0	2M-H	C6H16N4O9P2	1.900961922	2461.238267	2357.489451	2751.900923	2351.523638	1498.402841	1576.6711	3428.021551	2338.656268	3030.518573
4.81_322.1095m/z	322.1095496	4.814166667	neg	2H-1-Benzopyran-2-one, 3-[1-(4-aminophenyl)-3-oxobutyl]-4-hydroxy-, (S)- (Ace	576	Unclassified	Unclassified	Unclassified			38.2	0	M-H	C19H17NO4	3.305347926	271.7379097	347.4530453	442.8891817	825.1097768	587.1272253	827.3183142	552.2021661	607.6108385	190.9106549
4.81_329.1609m/z	329.1608878	4.814166667	neg	Dihydroartemisinin (DHA)	HMDB0061088	Organic oxygen compounds	Organooxygen compounds	Carbohydrates and carbohydrate conjugates	*		38.2	0	M+FA-H	C15H24O5	1.094533113	1368.788804	1496.511798	1695.162384	1576.913696	1615.290713	1627.665655	829.4370928	1111.644211	797.4195946
4.86_431.0991m/z	431.0991168	4.858383333	neg	1-Methyl-4-nitro-5-(S-Gluctathionyl) Imidazole	1199	Unclassified	Unclassified	Unclassified			38.2	0	M-H	C14H20N6O8S	0.140171534	1759.388285	2053.195286	2218.792074	101.7163515	243.1531828	180.4384127	614.5116802	409.1452637	829.1931567
4.86_464.1386m/z	464.1385659	4.858383333	neg	O-Ethyl-4-[(2',3',4'-tri-O-acetyl-alpha-L-rhamnosyloxy)benzyl]thiocarbamate	HMDB0031946	Organic oxygen compounds	Organooxygen compounds	Carbohydrates and carbohydrate conjugates	*		38.2	0	M-H2O-H	C22H29NO9S	0.21636588	1417.643513	1511.670573	1557.549303	1381.069525	1589.346377	1375.626841	1514.024774	1570.4606	1685.504611
4.90_391.1103m/z	391.1103344	4.902316667	neg	S 26191	2703	Unclassified	Unclassified	Unclassified			38.2	0	M+FA-H	C15H17F3N2O4	-5.518791061	3063.260431	3390.781129	3410.326121	6185.874687	6655.27834	6388.992129	3666.454073	3626.984064	3897.91089
5.01_336.1325n	335.123084	5.014716667	neg	Semilepidinoside A	HMDB0033107	Organic oxygen compounds	Organooxygen compounds	Carbohydrates and carbohydrate conjugates			38.2	3.05	M-H2O-H, M-H	C16H20N2O6	1.015835435	5078.466239	5495.739321	5545.872136	9442.075434	9456.320321	10045.47189	5651.555239	5315.088342	5681.230833
5.08_211.1341m/z	211.1341006	5.080566667	neg	2,4-Undecadienal	HMDB0031359	Organic oxygen compounds	Organooxygen compounds	Carbonyl compounds	*		38.2	0	M+FA-H	C11H18O	0.797623286	342.4857863	355.0234804	431.8122945	594.6830308	563.9497942	604.0481154	319.3876225	239.9017185	323.1282454
5.16_371.1351m/z	371.135056	5.155116667	neg	1-Methoxy-3-(4-hydroxyphenyl)-2E-propenal 4'-glucoside	HMDB0034755	Organic oxygen compounds	Organooxygen compounds	Carbohydrates and carbohydrate conjugates			38.2	0	M+FA-H	C16H22O7	0.92016915	687.5928349	857.7117167	737.5947087	536.2682649	429.7885271	536.7926077	662.6242788	599.6895494	567.2902898
5.18_335.0235m/z	335.0234667	5.1777	neg	Dihydronaringenin-O-sulphate	HMDB0059996	Phenylpropanoids and polyketides	Linear 1,3-diarylpropanoids	Chalcones and dihydrochalcones	*		38.2	0	M-H2O-H	C15H14O8S	1.043408217	6477.729496	6940.007757	7297.023128	0.001403199	0.001403199	0.001403199	143.5597062	0.001403199	0.001403199
5.22_527.2133m/z	527.2133379	5.222166667	neg	Acetyl-N-formyl-5-methoxykynurenamine	HMDB0004259	Organic oxygen compounds	Organooxygen compounds	Carbonyl compounds	**	C05642	38.2	0	2M-H	C13H16N2O4	-2.649825066	1404.083961	1407.337971	1488.067908	416.5247661	659.705331	677.9553117	649.6844354	1025.210258	952.3670736
5.24_537.2347m/z	537.2346773	5.244033333	neg	Caryoptin	67402	Unclassified	Unclassified	Unclassified			38.2	0	M+FA-H	C26H36O9	1.100446388	3481.357982	3662.717374	3219.260875	4507.919751	4509.012248	4038.97739	3158.712709	3438.918348	3871.992364
5.26_317.1972m/z	317.1971825	5.263033333	neg	7(14)-Bisabolene-2,3,10,11-tetrol	91117	Lipids and lipid-like molecules	Prenol lipids	Sesquiterpenoids	*		38.2	0	M+FA-H	C15H28O4	0.809257652	1529.389971	1176.578036	1158.616309	1203.03622	1134.843493	1158.016749	1324.109453	1079.426805	1517.77365
5.27_747.2442m/z	747.2442338	5.271716667	neg	N-(3-Benzooxazol-2-yl-4-hydroxy-phenyl)-2-p-tolyloxyacetamide	69097	Unclassified	Unclassified	Unclassified			38.2	0	2M-H	C22H18N2O4	-2.41082016	6811.107409	4129.696127	4226.682073	3325.145605	3043.222167	3423.919244	6293.684742	4063.280263	5216.369586
5.41_184.0979m/z	184.0979209	5.406433333	neg	9-Azabicyclo[3.3.1]nonan-3-one	HMDB0029613	Organoheterocyclic compounds	Piperidines	Piperidinones	*		38.2	0	M+FA-H	C8H13NO	0.028952003	96.35791596	89.22789841	128.3926667	183.5619936	159.032145	131.3902503	216.8196528	124.4418335	137.8911108
5.41_405.1257m/z	405.1256949	5.406433333	neg	Glycyl-Glutamate	85753	Organic acids and derivatives	Carboxylic acids and derivatives	Amino acids, peptides, and analogues	*		38.2	0	2M-H	C7H11N2O5-	-1.53048281	37747.44678	38115.49468	36994.05102	72442.09119	70184.51623	65485.25493	53945.49334	55309.78544	42940.3289
5.41_629.2781m/z	629.2781341	5.406433333	neg	Clavamycin D	71638	Unclassified	Unclassified	Unclassified			38.2	0	2M-H	C13H21N3O6	-1.047548424	1112.013565	1491.996965	1386.665478	6427.555556	5697.867449	4788.922609	1950.209606	2402.669671	1762.651831
5.43_472.2918m/z	472.2917888	5.428733333	neg	PGF2α serinol amide	45400	Unclassified	Unclassified	Unclassified			38.2	0	M+FA-H	C23H41NO6	0.463184291	987.0143349	1233.088434	1564.064559	2832.477984	2050.941754	2178.319707	1017.543464	2134.188105	1618.268207
5.45_499.2167m/z	499.2167036	5.451166667	neg	Glycophymoline	68495	Unclassified	Unclassified	Unclassified			38.2	0	2M-H	C16H14N2O	5.505361115	3323.96351	3189.10589	3635.115538	3436.513856	2858.83277	2972.037007	7311.437408	4750.383984	5214.690541
5.53_293.1510m/z	293.1510013	5.525416667	neg	2-Piperidinecarboxamide, N-(3-hydroxy-2,6-dimethylphenyl)-, (S)-	2384	Unclassified	Unclassified	Unclassified			38.2	0	M+FA-H	C14H20N2O2	1.291804741	818.2273099	565.6430959	608.5431696	1109.475612	1525.763816	1108.218369	596.6681862	729.0454313	684.094431
5.53_407.0961m/z	407.0961314	5.525416667	neg	5,7,3'-Trihydroxy-6,4',5'-trimethoxyflavanone	53114	Unclassified	Unclassified	Unclassified			38.2	0	M+FA-H	C18H18O8	-6.183263678	129798.914	111853.3753	109629.9458	40084.95579	51880.88769	43299.12891	92571.49745	94990.10199	114551.8804
5.53_451.0861m/z	451.0860621	5.525416667	neg	Chorismic acid	368	Organic acids and derivatives	Carboxylic acids and derivatives	Dicarboxylic acids and derivatives	**	C00251	38.2	0	2M-H	C10H10O6	-4.728002694	1455.767278	1502.538397	1569.283714	297.5926965	552.7138077	383.4000843	785.441131	980.7018006	1308.124038
5.64_167.0714m/z	167.0714048	5.6386	neg	2-Methylbenzyl alcohol	66608	Benzenoids	Benzene and substituted derivatives	Benzyloxycarbonyls	**	C07213	38.2	0	M+FA-H	C8H10O	0.302907098	301.9817215	229.785375	310.2776927	351.8149446	267.4798544	288.6778169	299.916536	332.1297069	263.6438227
5.64_227.0928m/z	227.0928213	5.6386	neg	3-Carboxy-2,4,4-trimethyl-2-cyclohexenone	2292	Unclassified	Unclassified	Unclassified			38.2	0	M+FA-H	C10H14O3	1.78022671	1077.221417	1008.271864	1154.725496	1053.422668	763.8052392	732.1167298	1267.080628	765.4370079	864.1934816
5.68_437.0914m/z	437.0913988	5.678766667	neg	L-Oxalylalbizziine	93758	Organic acids and derivatives	Carboxylic acids and derivatives	Amino acids, peptides, and analogues			38.2	0	2M-H	C6H9N3O6	0.924817979	6551.308301	6540.42437	6432.122196	1713.866146	1909.588127	1709.734596	178.580348	96.26556397	233.6949662
5.69_413.2184m/z	413.2184431	5.6875	neg	20-COOH-10,11-dihydro-LTB4	HMDB0012633	Lipids and lipid-like molecules	Fatty Acyls	Fatty acids and conjugates	*		38.2	0	M+FA-H	C20H32O6	0.954578333	5817.199951	5406.067688	5295.364566	860.8691553	1012.546247	1161.240031	2904.754438	3769.95372	4848.009169
5.70_521.1619m/z	521.1618524	5.701283333	neg	Hymexazol	68843	Unclassified	Unclassified	Unclassified			38.2	0	2M-H	C10H15NO7	-1.10082912	12557.23581	12183.37295	11796.7695	10402.67115	12726.22066	11389.72597	15237.34544	15776.44469	18568.57128
5.70_683.1530m/z	683.1530182	5.701283333	neg	2-NBDG	96480	Unclassified	Unclassified	Unclassified			38.2	0	2M-H	C12H14N4O8	-2.970417918	1656.804972	2166.759945	2120.189675	691.7904957	719.581964	1076.55144	1653.717531	2143.5562	3588.223242
5.83_233.0796m/z	233.0795756	5.825	neg	2S-amino-3-oxo-butanoic acid	35855	Lipids and lipid-like molecules	Fatty Acyls	Fatty Acids and Conjugates	**	C03508	38.2	0	2M-H	C4H7NO3	7.116562236	1149.627206	1287.955398	1056.623352	3027.701102	2587.106974	2378.438145	1828.289077	1494.909861	1397.27896
5.85_483.2605m/z	483.2604604	5.84575	neg	(-)-Fusicoplagin A	53690	Lipids and lipid-like molecules	Prenol lipids	Isoprenoids			38.2	0	M+FA-H	C24H38O7	1.149878989	5129.149547	5094.997705	5447.258014	6568.201456	4595.065115	5055.948921	6137.636579	5255.513133	5127.850101
5.88_464.3019m/z	464.3018935	5.88025	neg	Glycohyocholic acid	HMDB0240607	Lipids and lipid-like molecules	Steroids and steroid derivatives	Bile acids, alcohols and derivatives			38.2	0	M-H	C26H43NO6	0.283333993	3043.588346	3770.749176	3265.862471	547.3250653	747.1232565	483.4496321	2393.161592	2683.666411	3938.734508
5.94_429.2131m/z	429.2131031	5.937716667	neg	Cinnzeylanol	HMDB0036010	Lipids and lipid-like molecules	Prenol lipids	Diterpenoids	*		38.2	0	M+FA-H	C20H32O7	0.252123859	10648.31834	12045.86935	11737.25346	4285.715218	5267.483086	4510.115768	7970.873412	10046.91172	10687.07786
5.95_499.2166m/z	499.2165716	5.95155	neg	Arborine	68457	Organoheterocyclic compounds	Diazanaphthalenes	Benzodiazines	*	C10642	38.2	0	2M-H	C16H14N2O	5.241465326	6753.942288	7447.355442	8691.772515	3606.447155	5184.518126	4706.203694	8494.624205	9257.545305	9788.324514
6.01_441.1594m/z	441.1594079	6.007266667	neg	b-D-Xylopyranosyl-(1->4)-a-L-rhamnopyranosyl-(1->2)-D-fucose	95644	Organic oxygen compounds	Organooxygen compounds	Carbohydrates and carbohydrate conjugates			38.2	0	M-H	C17H30O13	-4.425282937	4012.650713	4359.859655	4237.516445	4264.13532	4638.274221	4167.892367	4749.707868	5158.591993	5484.980677
6.02_211.1341m/z	211.1340746	6.01945	neg	Dihydrojasmone	87770	Organic oxygen compounds	Organooxygen compounds	Carbonyl compounds	*		38.2	0	M+FA-H	C11H18O	0.641467576	531.3062775	538.3446624	577.3217351	734.5131924	810.5688277	876.6093194	459.1728295	453.7942924	439.2100623
6.07_455.1542m/z	455.1541792	6.07	neg	Bruceine D	HMDB0249404	Unclassified	Unclassified	Unclassified			38.2	2.34	M+FA-H	C20H26O9	-4.159675115	20260.31539	22336.27017	21704.8652	16279.61152	16986.1498	14211.72785	20559.867	20242.45301	19743.19393
6.10_377.1972m/z	377.1971756	6.101316667	neg	Gibberellin A12	HMDB0303453	Lipids and lipid-like molecules	Prenol lipids	Isoprenoids	**	C11857	38.2	0	M+FA-H	C20H28O4	0.642210314	2535.206928	2837.96281	2840.358656	2192.833069	2456.631034	1944.225948	1726.589541	1950.804657	2217.622751
6.16_413.2184m/z	413.2184385	6.161866667	neg	20-dihydroxyleukotriene B4	LMFA03020061	Lipids and lipid-like molecules	Fatty Acyls	Eicosanoids	*		38.2	0	M+FA-H	C20H32O6	0.942190636	11133.26845	10976.90344	11971.94523	3091.038486	3010.351407	2991.863758	6364.424583	7365.152828	7820.670425
6.18_359.1351m/z	359.1350509	6.181866667	neg	2'-Methoxy-3-(2,4-dihydroxyphenyl)-1,2-propanediol 4'-glucoside	94045	Organic oxygen compounds	Organooxygen compounds	Carbohydrates and carbohydrate conjugates			38.2	0	M-H	C16H24O9	0.819156815	1399.257396	1225.929554	1453.72723	1660.565897	1484.623586	2009.14104	1265.746294	1027.243755	1218.99759
6.20_297.1709m/z	297.1708879	6.195716667	neg	Epioxylubimin	HMDB0035613	Lipids and lipid-like molecules	Prenol lipids	Sesquiterpenoids	*		38.2	0	M+FA-H	C15H24O3	0.556945837	6329.633902	6364.277834	6972.667374	9641.615298	9271.92008	9120.301376	4959.449159	5575.840126	6293.150459
6.36_399.1278m/z	399.1278095	6.3617	neg	Laurencione diacetate	65391	Unclassified	Unclassified	Unclassified			38.2	0	2M-H	C9H12O5	-4.651044978	7608.212482	7938.564506	8001.911095	8042.263023	8125.924159	8594.485576	6967.092515	6985.646497	7169.551706
6.41_629.3548m/z	629.3548194	6.408833333	neg	Goshonoside F4	93085	Lipids and lipid-like molecules	Prenol lipids	Terpene glycosides	*		38.2	0	M-H	C32H54O12	0.902057479	7612.741426	7146.201508	8686.003582	7157.781988	6749.954244	6120.502883	9759.162626	8477.701369	9648.727353
6.48_158.1187m/z	158.1187296	6.477433333	neg	Pregabalin	HMDB0014375	Organic acids and derivatives	Carboxylic acids and derivatives	Amino acids, peptides, and analogues			38.2	0.806	M-H	C8H17NO2	0.485432476	962.4260151	1024.184791	1140.210714	1984.33685	1660.190561	1780.866676	1289.9553	1534.548856	1574.347724
6.48_537.1959m/z	537.195908	6.477433333	neg	p-Hydroxytriamterene	2909	Unclassified	Unclassified	Unclassified			38.2	0	2M-H	C12H11N7O	-3.403186298	6563.829518	5963.96087	7125.172089	1830.711789	1728.748068	1595.111005	5317.971203	5665.815875	5576.841807
6.48_608.0546m/z	608.0545767	6.477433333	neg	UDP-L-Ara4FN	63219	Unclassified	Unclassified	Unclassified			38.2	0	M+FA-H	C15H23N3O16P2	1.80954988	1230.254319	1471.539556	1732.305941	2660.915681	2769.29502	2393.251035	2023.197662	2685.849133	2366.129536
6.60_341.1736m/z	341.1735919	6.6024	neg	(3-Methylcrotonyl)glycine methyl ester	6386	Unclassified	Unclassified	Unclassified			38.2	0	2M-H	C8H13NO3	5.2070585	507.581748	0.001403199	2876.560998	0.001403199	0.001403199	65.41464974	0.001403199	0.001403199	232.064527
6.79_399.1275m/z	399.1275489	6.7874	neg	(Z)-3-(1-Formyl-1-propenyl)pentanedioic acid	88983	Lipids and lipid-like molecules	Fatty Acyls	Fatty acids and conjugates	*		38.2	0	2M-H	C9H12O5	-5.302187642	996.9946083	777.7378753	1226.330557	2528.970433	1885.093921	2616.581087	1633.3112	1485.016914	1496.818347
6.81_413.2182m/z	413.2182166	6.809016667	neg	Cinncassiol D2	71842	Lipids and lipid-like molecules	Prenol lipids	Sesquiterpenoids	*		38.2	0	M+FA-H	C20H32O6	0.339562773	10029.33605	10394.22548	9549.557704	4595.436499	3776.167279	5215.086072	8530.650005	9752.188897	8266.705052
6.84_269.0456m/z	269.0456496	6.842166667	neg	Hydroxyanthraquinone	65876	Unclassified	Unclassified	Unclassified			38.2	0	M+FA-H	C14H8O3	0.45785097	0.001403199	0.001403199	0.001403199	1784.931362	1688.453721	1770.180557	0.001403199	0.001403199	0.001403199
6.95_481.2073m/z	481.2073127	6.951516667	neg	Polhovolide	67781	Unclassified	Unclassified	Unclassified			38.2	0	M+FA-H	C23H32O8	-1.394136077	16454.24354	17253.36654	15868.28314	15675.84438	13646.93632	14076.80448	16292.08708	19686.37754	18670.70609
7.04_787.8458m/z	787.8458048	7.044716667	neg	Ioversol	HMDB0253553	Unclassified	Unclassified	Unclassified			38.2	0	M-H2O-H	C18H24I3N3O9	-1.273367763	6444.584804	4876.014151	5381.007321	8502.701704	8054.303304	8481.310734	8748.833769	6125.671458	7999.283444
7.06_397.1868m/z	397.1867798	7.055233333	neg	γ-Glutamyl-β-aminopropiononitrile	63620	Organic acids and derivatives	Carboxylic acids and derivatives	Amino acids, peptides, and analogues		C06114	38.2	0	2M-H	C8H13N3O3	6.714432348	3791.534722	4104.193405	3962.125976	2701.354487	2844.616245	2425.901186	3579.253164	4443.416982	3632.155235
7.20_445.1143m/z	445.1142587	7.201166667	neg	(E)-Monocrotophos	HMDB0031805	Organic acids and derivatives	Organic phosphoric acids and derivatives	Phosphate esters	*	C18663	38.2	0	2M-H	C7H14NO5P	-0.858953175	4998.213474	5064.231338	4226.312898	4895.997838	5564.472037	5245.138887	7031.403927	5936.841938	5429.742757
7.23_463.2339m/z	463.2339447	7.225666667	neg	11beta,17,21-Trihydroxy-2alpha-methylpregn-4-ene-3,20-dione 21-acetate	70776	Unclassified	Unclassified	Unclassified			38.2	0	M+FA-H	C24H34O6	0.485328763	1616.230735	1924.903751	1982.353295	4206.26961	4912.773225	5014.436144	3336.483256	3150.894807	2400.517401
7.25_511.2189m/z	511.2188539	7.248333333	neg	T2 Toxin	HMDB0036600	Lipids and lipid-like molecules	Prenol lipids	Sesquiterpenoids	*	C09738	38.2	0	M+FA-H	C24H34O9	0.790115257	7028.966044	8637.294077	8075.448637	11162.12793	10933.86409	11448.95086	6796.044619	8140.777507	8876.35115
7.25_513.2708m/z	513.2708187	7.248333333	neg	3-alpha-Androstanediol glucuronide	HMDB0010339	Alkaloids and derivatives	Morphinans	Unclassified	*		38.2	0	M+FA-H	C25H40O8	0.635449441	216.7665545	0.001403199	247.6130516	0.001403199	0.001403199	0.001403199	980.3722593	1562.642099	1505.595849
7.35_519.0583m/z	519.0583186	7.346316667	neg	Norathyriol	68120	Unclassified	Unclassified	Unclassified			38.2	0	2M-H	C13H8O6	2.728607464	41201.17036	40000.92067	40513.68816	29563.48103	30457.61906	28530.29034	24220.55289	27704.82806	29761.29213
7.44_437.1614m/z	437.1613799	7.440866667	neg	Asparaginyl-Serine	85655	Organic acids and derivatives	Carboxylic acids and derivatives	Amino acids, peptides, and analogues	*		38.2	0	2M-H	C7H13N3O5	-5.442508885	3039.102699	2910.089032	3624.24566	4588.335301	3705.27392	4009.029118	2796.688888	3808.986476	2788.258829
7.45_214.1451m/z	214.1450571	7.453016667	neg	Lupinine	43880	Unclassified	Unclassified	Unclassified			38.2	0	M+FA-H	C10H19NO	1.123160824	999.6974591	994.3259664	994.0434163	1162.63948	1178.92734	1118.940494	1052.977962	961.936601	880.9627757
7.54_455.1906m/z	455.1906046	7.536516667	neg	dimethyl 2-(3-oxocyclohexyl)propanedioate	88496	Unclassified	Unclassified	Unclassified			38.2	0	2M-H	C11H16O5	-3.652247727	2840.277756	3681.621111	3605.882657	4215.385245	3653.717624	3697.616193	1796.451779	1665.678596	1744.019958
7.61_125.0972m/z	125.0972195	7.6091	neg	2-Ethylhexanoic acid	HMDB0031230	Lipids and lipid-like molecules	Fatty Acyls	Fatty acids and conjugates	*		38.2	0	M-H2O-H	C8H16O2	0.21431474	351.6193326	313.3907332	292.524875	368.2158632	294.5238579	323.4797562	317.71069	335.4784738	341.8772022
7.65_471.2382m/z	471.2381923	7.65305	neg	DIHYDRO-7-DESACETYLDEOXYGEDUNIN	43755	Unclassified	Unclassified	Unclassified			38.2	0	M+FA-H	C26H34O5	-1.48923353	7715.457474	8367.807721	8062.896343	3523.545342	2881.790238	3453.454259	5882.25725	6112.57472	5973.681515
7.68_787.8459m/z	787.8459132	7.67705	neg	Ioglunide	HMDB0253538	Benzenoids	Benzene and substituted derivatives	Halobenzenes			38.2	0	M-H2O-H	C18H24I3N3O9	-1.139033935	15784.92666	17049.16516	18425.01394	18849.53273	20711.27008	17978.59612	22509.47885	19343.7821	19159.51475
7.72_344.1837n	389.1821216	7.719916667	neg	Granisetron metabolite 1	3143	Unclassified	Unclassified	Unclassified			38.2	0	M-H, M+FA-H	C18H24N4O3	-3.37872136	44913.4891	49883.60483	47306.08952	44153.5094	46522.81445	42107.41617	31704.48833	34847.11133	38497.58175
7.84_408.2880n	407.2806769	7.838516667	neg	3a,4b,7a-Trihydroxy-5b-cholanoic acid	HMDB0000320	Lipids and lipid-like molecules	Steroids and steroid derivatives	Bile acids, alcohols and derivatives	*		38.2	0	M-H, M+FA-H	C24H40O5	0.928248331	9367.637224	10106.70417	11548.51678	1999.281278	1772.986216	1731.817459	4738.626507	5740.022321	5008.918608
8.29_511.2527m/z	511.2527208	8.294766667	neg	3α-hydroxy-5α-androstane-17-one 3-D-glucuronide	43165	Unclassified	Unclassified	Unclassified			38.2	0	M+FA-H	C25H38O8	-4.611818958	41781.79816	49565.84918	52591.72508	46071.01432	39320.46951	41220.9109	39525.98186	47024.6819	45790.19571
8.41_463.1952m/z	463.1951725	8.411166667	neg	2,3-Diphenylpyrazine	88880	Organoheterocyclic compounds	Diazines	Pyrazines			38.2	0	2M-H	C16H12N2	5.067115528	7822.408711	7891.975825	7812.739112	2973.436081	2514.626248	2500.319124	4525.949504	5464.015267	5445.85819
8.47_463.1590m/z	463.158977	8.473333333	neg	Parafluron	72831	Unclassified	Unclassified	Unclassified			38.2	0	2M-H	C10H11F3N2O	3.356812012	8965.452605	9589.45395	9407.257125	9850.390978	9719.968597	9219.222346	4859.492913	5612.913336	5338.907399
8.50_315.1815m/z	315.1814578	8.495033333	neg	6-Ethyl-1-methyl-2,7,8-trioxabicyclo[3.2.1]octane	HMDB0030303	Organoheterocyclic compounds	Dioxepanes	1,3-dioxepanes	*		38.2	0	2M-H	C8H14O3	0.460416948	3019.497349	2639.007366	2832.091025	3302.655431	3768.523273	3416.094577	2458.6646	2366.431929	2040.986884
8.56_413.2183m/z	413.2182538	8.560966667	neg	Cinncassiol D3	HMDB0036854	Lipids and lipid-like molecules	Prenol lipids	Sesquiterpenoids	*		38.2	0	M+FA-H	C20H32O6	0.440539715	1234.054122	1235.759894	1287.613855	682.8292355	574.8084332	947.2000333	671.9963845	1048.583248	957.9987112
8.69_611.3806m/z	611.3805563	8.6894	neg	W123	64710	Unclassified	Unclassified	Unclassified			38.2	0	2M-H	C17H26N2O3	-1.392425082	2557.905153	3855.025164	2425.585506	3763.82023	3460.673482	2590.258729	1792.330806	2611.186302	2926.587427
9.02_611.2430m/z	611.2429788	9.01635	neg	(-)-Isolonchocarpin	LMPK12140015	Lipids and lipid-like molecules	Polyketides	Flavonoids			38.2	0	2M-H	C20H18O3	-1.524966291	40035.41067	50139.6682	46951.54524	3108.951828	3454.010142	3826.576217	19417.18902	22074.67802	20702.53728
9.37_307.1165m/z	307.1165096	9.367783333	neg	Vanillyl alcohol	66362	Benzenoids	Phenols	Methoxyphenols	*	C06317	38.2	0	2M-H	C8H10O3	-7.147564493	2722.850483	1233.532442	1309.537712	1315.458565	1106.228485	1016.74295	1314.978653	1403.049234	1286.373518
0.58_173.1184m/z	173.1184027	0.58075	neg	2-Methyl-3-heptanone	HMDB0032402	Organic oxygen compounds	Organooxygen compounds	Carbonyl compounds	*		38.1	0	M+FA-H	C8H16O	0.660734843	143.0809592	82.65764697	122.4141411	130.1766867	192.4793522	107.2179073	63.91696969	44.82337379	68.33564323
0.67_169.0871m/z	169.087121	0.6701	neg	5,7-octadienal	75315	Lipids and lipid-like molecules	Fatty Acyls	Fatty aldehydes			38.1	0	M+FA-H	C8H12O	0.830758147	153.1955136	112.1526177	107.9808824	80.85792072	88.30052611	131.1854037	166.7312796	144.8192827	94.40317605
0.67_395.0536m/z	395.0536216	0.6701	neg	Naphthalic anhydride	72867	Unclassified	Unclassified	Unclassified			38.1	0	2M-H	C12H6O3	-6.287099383	169.1071315	228.58807	107.2023047	890.9866077	481.9055551	829.9156368	670.5582843	545.1983757	395.4769168
0.71_895.7726m/z	895.7725656	0.714216667	neg	(3beta,5alpha,6beta,24R)-Stigmastane-3,5,6-triol	LMST01040240	Lipids and lipid-like molecules	Steroids and steroid derivatives	Stigmastanes and derivatives	*		38.1	0	2M-H	C29H52O3	-3.846042105	969.0072451	0.001403199	1628.052399	2745.432901	4481.495282	3320.863312	0.001403199	6200.111023	4904.25578
0.76_893.2472m/z	893.2472362	0.757316667	neg	Peonidin 3-rhamnoside	HMDB0038091	Phenylpropanoids and polyketides	Flavonoids	Flavonoid glycosides			38.1	0	2M-H	C22H23O10+	-4.172404473	6020.701455	5188.540389	3057.943964	16967.52621	7464.458654	13676.73051	7334.036805	6366.488221	9827.665125
0.78_217.0298m/z	217.0298306	0.778533333	neg	Aspartyl-Cysteine	85665	Organic acids and derivatives	Carboxylic acids and derivatives	Amino acids, peptides, and analogues	*		38.1	0	M-H2O-H	C7H12N2O5S	4.148241498	50575.45044	60241.87891	77732.60324	103199.6495	109698.5401	101961.1633	93451.37263	77422.79998	88187.62171
0.80_229.0475m/z	229.0474862	0.800366667	neg	MALEAMIC ACID	HMDB0254310	Unclassified	Unclassified	Unclassified			38.1	0	2M-H	C4H5NO3	3.810160172	3009.008439	1689.038674	2647.580714	2148.39538	2038.701968	1767.034427	1479.43241	1708.526253	1692.011684
0.80_321.0498m/z	321.0497911	0.800366667	neg	5-Thymidylic acid	HMDB0001227	Nucleosides, nucleotides, and analogues	Pyrimidine nucleotides	Pyrimidine deoxyribonucleotides	**	C00364	38.1	1.62	M-H	C10H15N2O8P	1.445297006	9592.592651	14184.7512	17070.52985	14386.18577	21937.72227	14048.60891	14425.80179	11974.50561	15849.32293
0.82_152.0023m/z	152.0023347	0.819566667	neg	Cysteine sulfinic acid	HMDB0250705	Organic acids and derivatives	Carboxylic acids and derivatives	Amino acids, peptides, and analogues	**	C00606	38.1	0	M-H	C3H7NO4S	0.211820695	1668.171049	1726.367671	1666.967507	1108.298043	895.7414357	1185.786947	1187.811734	1595.244789	981.6381934
0.86_237.0617m/z	237.0617091	0.86335	neg	2D-5-O-Methyl-2,3,5/4,6-pentahydroxycyclohexanone	65578	Unclassified	Unclassified	Unclassified			38.1	0	M+FA-H	C7H12O6	0.615251832	1287.604832	1493.695157	1348.67432	1530.216061	955.0200243	1201.197958	744.8276201	344.1104736	351.3212419
0.88_159.0300m/z	159.0299732	0.884633333	neg	2-Dehydro-3-deoxy-D-galactonate	63177	Organic acids and derivatives	Keto acids and derivatives	Medium-chain keto acids and derivatives	**	C01216	38.1	0	M-H2O-H	C6H10O6	0.428260679	459.1865462	519.003484	769.1414943	356.8775696	377.3442528	397.0981292	296.0269186	244.2356876	312.1096332
0.88_203.0370m/z	203.0369904	0.884633333	neg	1,4-Dihydroxy-2-naphthoic acid	64746	Unclassified	Unclassified	Unclassified			38.1	10.8	M-H	C11H8O4	9.841692217	4777.877493	7493.813966	6140.876862	13420.18121	7934.351427	12930.11544	3630.452589	7214.030357	5480.360303
0.88_341.0298m/z	341.0297874	0.884633333	neg	Medicagol	LMPK12090025	Phenylpropanoids and polyketides	Isoflavonoids	Coumestans			38.1	0	M+FA-H	C16H8O6	-1.700528093	83175.18376	90581.04114	85810.6025	120825.8742	103261.75	116843.7004	94320.84075	89223.81733	93326.64422
0.90_224.0236m/z	224.0235613	0.904683333	neg	S-Carboxymethyl-L-cysteine	86253	Organic acids and derivatives	Carboxylic acids and derivatives	Amino acids, peptides, and analogues	*		38.1	0	M+FA-H	C5H9NO4S	0.724049458	4029.17056	3855.929022	4943.962567	7188.147295	8224.663141	7011.215254	5763.293037	5175.79912	6720.276817
0.95_202.0722m/z	202.0722261	0.946033333	neg	2-Ethyl-2-methyl-3-hydroxysuccinimide	2596	Unclassified	Unclassified	Unclassified			38.1	0	M+FA-H	C7H11NO3	0.827603284	394.2919666	355.219537	471.5087592	570.7291645	424.9520897	489.125829	439.3478926	591.671418	463.2028239
0.95_247.0631m/z	247.0631234	0.946033333	neg	Phenylmethanethiol	HMDB0029633	Benzenoids	Benzene and substituted derivatives	Unclassified	*		38.1	0	2M-H	C7H8S	4.262263422	8520.284752	16392.84826	19110.42387	80604.50037	65197.341	66800.80675	15900.55977	33375.66253	26477.94231
1.14_287.0634m/z	287.0633929	1.1382	neg	2-Deoxystreptamine phospahte	66053	Unclassified	Unclassified	Unclassified			38.1	0	M+FA-H	C6H15N2O6P	-6.538546175	1994.910509	5855.568297	4689.617347	6588.293953	2335.902288	2992.409932	4735.86548	2014.840251	1859.364803
1.27_162.0423m/z	162.0422933	1.26875	neg	Pterin	HMDB0000802	Organoheterocyclic compounds	Pteridines and derivatives	Pterins and derivatives	*	C00715	38.1	0	M-H	C6H5N5O	0.980959157	263.1798481	308.5518891	318.2200103	326.8803739	352.5864642	278.9612974	254.5889364	367.4871244	299.369131
1.40_230.0670m/z	230.0670426	1.3958	neg	N-(3-oxo-butanoyl)-homoserine lactone	LMFA08030028	Lipids and lipid-like molecules	Fatty Acyls	Fatty amides			38.1	0	M+FA-H	C8H11NO4	0.172336218	796.0380537	507.0019395	578.0969555	862.3790276	867.4230059	750.188832	833.7077053	637.5733414	725.7067673
1.53_173.0932m/z	173.0932464	1.534483333	neg	N5-Acetylornithine	HMDB0240589	Organic acids and derivatives	Carboxylic acids and derivatives	Amino acids, peptides, and analogues			38.1	0	M-H	C7H14N2O3	0.46243243	194.8747296	138.8774775	171.7156271	154.4764416	194.9039027	178.6946843	167.8855591	189.11656	202.3020228

1.53_198.0715m/z	198.0715273	1.534483333	neg	1-aminopyrene	96142	Benzenoids	Pyrenes	Unclassified	*		38.1	0	M-H2O-H	C16H11N	1.008993769	354.9895669	399.9263745	454.972446	622.3964146	666.6899449	745.391983	391.7029453	721.3176482	542.3999183
1.72_179.0212m/z	179.0211876	1.721216667	neg	2,4-Dinitrophenylhydrazine	HMDB0245463	Unclassified	Unclassified	Unclassified			38.1	0	M-H2O-H	C6H6N4O4	0.626136686	525.4146585	566.1939803	276.2567709	597.6657853	638.0042666	310.030318	617.986916	379.6275864	798.7872958
10.29_449.2549m/z	449.2549329	10.29078333	neg	3α-Hydroxy-7,12-dioxo-5β-cholan-24-oic Acid	42778	Unclassified	Unclassified	Unclassified			38.1	0	M+FA-H	C24H36O5	1.127475369	2905.228911	2866.418526	4191.440722	3625.456355	3697.688679	3985.574408	2530.071503	3211.816896	2986.869302
13.60_313.0786m/z	313.0786131	13.5994	neg	Arabinosylhypoxanthine	3022	Nucleosides, nucleotides, and analogues	Purine nucleosides	Unclassified	*		38.1	0	M+FA-H	C10H12N4O5	-1.340102655	1165.970298	275.1521572	364.9675139	1852.395195	316.9114682	609.4339196	372.5287417	368.565979	145.7830288
13.80_212.9793m/z	212.9792939	13.7953	neg	2-Oxo-3-hydroxy-4-phosphobutanoate	3347	Organic acids and derivatives	Keto acids and derivatives	Short-chain keto acids and derivatives	**	C06054	38.1	0	M-H	C4H7O8P	-5.997907196	612417.1135	591104.489	577193.1185	568058.1	598244.3659	586531.3258	667143.2158	597158.175	600350.5209
2.03_463.1029m/z	463.102887	2.025516667	neg	(2E,11Z)-5-[5-(Methylthio)-4-penten-2-ynyl]-2-furanacrolein	87387	Lipids and lipid-like molecules	Fatty Acyls	Fatty acids and conjugates	*		38.1	0	2M-H	C13H12O2S	-3.097979712	413.3274442	316.5907083	653.3075661	1269.419343	1029.681245	723.7459858	645.9693275	1150.528986	753.4328486
2.07_324.0678m/z	324.0678453	2.070366667	neg	N-(7-Mercaptoheptanoyl)threonine 3-O-phosphate	63904	Unclassified	Unclassified	Unclassified			38.1	0	M-H2O-H	C11H22NO7PS	0.660914319	18063.01988	19253.04014	19797.9387	19233.86511	20513.87897	18909.0834	21858.66095	19624.9393	19762.48663
2.26_381.0137m/z	381.0136579	2.26065	neg	2,3-Dioxo-6-nitro-7-sulfamoylbenzo[f]quinoxaline	69621	Unclassified	Unclassified	Unclassified			38.1	0	M+FA-H	C12H8N4O6S	-2.976034588	236450.4908	249328.8075	255126.6494	29938.74647	28297.0227	27790.94363	191896.9538	209574.5704	195815.5416
2.31_479.0971m/z	479.097069	2.3142	neg	1,4-DPCA	63078	Unclassified	Unclassified	Unclassified			38.1	0	2M-H	C13H8N2O3	-5.496270657	3107.936628	3258.09796	2703.189567	5285.364863	5090.149966	5993.640869	3854.18728	4969.42312	4017.33422
3.19_371.0846m/z	371.0846465	3.186766667	neg	mafenide	43232	Unclassified	Unclassified	Unclassified			38.1	0	2M-H	C7H10N2O2S	-1.81189727	1807.575941	2045.272202	2155.157437	3327.927534	3030.634116	2933.974399	2139.016906	2433.071755	2442.279704
3.75_472.1590m/z	472.1589775	3.747833333	neg	10-Formyltetrahydrofolate	HMDB0000972	Organoheterocyclic compounds	Dithiolanes	Lipoamides	**	C00234	38.1	0	M-H	C20H23N7O7	0.756262772	714.7365335	555.1506963	712.3434824	1752.132064	1285.862584	1123.931333	363.9508333	437.250515	312.0725164
3.77_552.9758m/z	552.9757781	3.770416667	neg	Inosine triphosphate	HMDB0000189	Nucleosides, nucleotides, and analogues	Purine nucleotides	Purine ribonucleotides	**	C00081	38.1	0	M+FA-H	C10H15N4O14P3	-4.302963471	3512.613796	3218.752997	2931.099757	5649.728572	5297.822304	3297.714144	3223.57231	2814.026399	3152.407361
3.80_203.0926m/z	203.0925686	3.801716667	neg	Tetrahydrofurfuryl propionate	87470	Organoheterocyclic compounds	Tetrahydrofurans	Unclassified	*		38.1	0	M+FA-H	C8H14O3	0.451999566	465.1147929	424.3588046	369.4172913	183.6849206	287.1199079	218.9158927	202.2404721	228.4445133	146.6897544
3.80_221.0491m/z	221.0491065	3.801716667	neg	2-Oxo-6-methylthiohexanoic acid	64495	Unclassified	Unclassified	Unclassified			38.1	0	M+FA-H	C7H12O3S	1.070233538	521.391647	492.1866974	548.8746058	786.1975253	752.3619192	836.850871	581.6167676	616.7755985	598.1975317
3.87_195.0775m/z	195.0775445	3.868533333	neg	2-Acetyl-3-ethylpyrazine	HMDB0029410	Organic oxygen compounds	Organooxygen compounds	Carbonyl compounds	*		38.1	0	M+FA-H	C8H10N2O	0.190992091	623.7938905	692.7147974	638.1480625	125.6548449	151.4295222	164.4228956	193.6163399	111.420949	122.2538159
3.87_331.0608m/z	331.0607633	3.868533333	neg	Tetracenomycin B1	63753	Unclassified	Unclassified	Unclassified			38.1	0	M-H2O-H	C20H14O6	-1.239127411	8159.203032	8020.086488	8651.101524	9789.45264	9495.170538	9330.310347	8615.895984	9821.534239	9899.056369
4.05_380.0602m/z	380.0602494	4.046666667	neg	Dihydroneopterin phosphate	HMDB0006824	Organoheterocyclic compounds	Pteridines and derivatives	Pterins and derivatives	**	C05925	38.1	0	M+FA-H	C9H14N5O7P	-3.097533797	1281.636746	1576.520414	1241.360208	2048.098629	1891.035997	1902.677986	1529.113597	1793.83897	1582.409848
4.07_239.1039m/z	239.1039418	4.069216667	neg	3-Hydroxy-N-glycyl-2,6-xylidine (3-Hydroxyglycinexylidide)	1003	Unclassified	Unclassified	Unclassified			38.1	0	M+FA-H	C10H14N2O2	1.088276379	926.2386076	1044.711073	1016.360206	871.4036904	828.8252643	983.4601157	792.8529644	971.538729	976.2557126
4.09_162.0561m/z	162.0561364	4.091783333	neg	Benzeneacetonitrile	HMDB0034171	Benzenoids	Benzene and substituted derivatives	Benzyl cyanides	**	C16074	38.1	0	M+FA-H	C8H7N	0.719918548	170.249585	207.0140477	253.9028193	233.9939476	349.9481123	275.754053	212.1291808	277.9951868	239.004705
4.19_415.1245m/z	415.1244811	4.189783333	neg	Linusitamarin	95321	Phenylpropanoids and polyketides	Cinnamic acids and derivatives	Hydroxycinnamic acids and derivatives			38.1	0	M+FA-H	C17H22O9	-0.281251675	1548.196886	1179.0417	898.0880558	1720.360734	2170.680423	2563.191151	1326.561894	1253.487198	1106.084265
4.23_325.1271m/z	325.1271225	4.234483333	neg	Homomethionine	64484	Organic acids and derivatives	Carboxylic acids and derivatives	Amino acids, peptides, and analogues	**	C17213	38.1	0	2M-H	C6H13NO2S	3.065093912	1239.164491	1490.644134	1488.209031	2370.815859	2147.829146	2028.510936	1434.654549	1779.8625	1669.773253
4.32_200.0929m/z	200.0928684	4.3224	neg	ARECOLINE	43570	Alkaloids and derivatives	Unclassified	Unclassified	*	C10129	38.1	0	M+FA-H	C8H13NO2	0.237399797	126.6292278	146.7200824	76.68371562	267.3871269	61.53303019	136.8399546	155.1789912	45.58888817	179.6095258
4.39_265.1096m/z	265.109554	4.3893	neg	SB 200646	69188	Unclassified	Unclassified	Unclassified			38.1	0	M-H	C15H14N4O	0.260663711	14052.06376	14648.61004	14206.97873	9423.822108	9884.04705	9611.981606	15026.49596	18161.73827	16037.39818
4.43_328.1040m/z	328.1040083	4.432683333	neg	Dopamine glucuronide	HMDB0010329	Organic oxygen compounds	Organooxygen compounds	Carbohydrates and carbohydrate conjugates	*		38.1	0	M-H	C14H19NO8	0.662995873	2545.686587	920.3188008	702.9776504	2108.482688	617.6097836	2605.889057	2144.620815	1872.691504	1751.003472
4.50_384.0715m/z	384.0714593	4.49925	neg	Propinol adenylate	58551	Organic acids and derivatives	Carboxylic acids and derivatives	Amino acids, peptides, and analogues	**	C05983	38.1	0	M-H2O-H	C13H18N5O8P	0.003067108	1329.547192	1327.575774	1269.921674	3190.133425	2810.324896	2759.766279	2963.539869	3048.193245	3082.978482
4.54_313.0570m/z	313.0570315	4.54425	neg	6-(4-carboxyphenoxy)-3,4,5-trihydroxyoxane-2-carboxylic acid	HMDB0125174	Organic oxygen compounds	Organooxygen compounds	Carbohydrates and carbohydrate conjugates	*		38.1	0	M-H	C13H14O9	1.674389308	1639.892985	1689.036165	1693.859972	205.6963276	213.2970842	221.2713823	348.3503109	416.5737994	303.1088853
4.54_335.1352m/z	335.1351504	4.54425	neg	Isopropyl apiosylglucoside	HMDB0041513	Organic oxygen compounds	Organooxygen compounds	Carbohydrates and carbohydrate conjugates	***		38.1	0	M-H2O-H	C14H26O10	1.113880091	1199.197435	1209.885439	1029.239866	1542.318159	1391.716229	1313.310645	1308.591954	1292.288786	1561.923342
4.57_170.0580n	151.0401291	4.5668	neg	3,4-Methyleneadipic acid	HMDB0059756	Lipids and lipid-like molecules	Fatty Acyls	Fatty acids and conjugates	*		38.1	0	M-H2O-H, M-H	C8H10O4	0.361281566	2759.934664	3165.977638	2751.551666	2834.690639	2757.143081	2557.048939	2702.065381	2617.311021	2914.658409
4.64_521.1662m/z	521.1662035	4.641416667	neg	Matteucinol 7-O-glucoside	LMPK12140328	Lipids and lipid-like molecules	Polyketides	Flavonoids			38.1	0	M+FA-H	C24H28O10	-0.517647547	7416.306092	6968.188618	6400.496593	894.3211526	728.7510244	688.2791804	6484.603646	6911.153963	5285.366919
4.68_487.1803m/z	487.1803128	4.682483333	neg	Lusitanicoside	68347	Organic oxygen compounds	Organooxygen compounds	Carbohydrates and carbohydrate conjugates		C10474	38.1	0	M+FA-H	C21H30O10	-4.041863475	994.1394962	793.5619657	1092.811954	919.7856524	974.7103089	712.9228871	1256.936764	1734.358203	1435.288233
4.70_244.0722m/z	244.0722405	4.703166667	neg	3-Hydroxymethyltriazolopthalazinone	3167	Unclassified	Unclassified	Unclassified			38.1	0	M+FA-H	C11H9N3O	-2.633560894	3323.56673	2926.366874	3085.487657	3201.17331	3121.014987	3062.183789	3445.072919	2820.798695	3527.812036
4.75_169.0871m/z	169.0871179	4.747466667	neg	Methyl-1-cyclopenten-1-yl)-ethanone	HMDB0061898	Organic oxygen compounds	Organooxygen compounds	Carbonyl compounds			38.1	0	M+FA-H	C8H12O	0.806402304	2299.12284	2172.942899	2219.195135	2591.78938	2556.723235	2522.689238	2541.949956	2239.166216	2160.43099
4.77_155.1079m/z	155.1078676	4.769883333	neg	Dimethylhexa-1,4-diene	HMDB0061901	Hydrocarbons	Unsaturated hydrocarbons	Branched unsaturated hydrocarbons			38.1	0	M+FA-H	C8H14	1.037755448	297.8641093	301.6582188	256.7799988	239.8038727	379.6194061	385.5449139	294.0038278	343.6850096	131.8337812
4.88_496.2357m/z	496.2356755	4.880083333	neg	14alpha-Hydroxypaxilline	95419	Organoheterocyclic compounds	Naphthopyrans	Unclassified			38.1	0	M+FA-H	C27H33NO5	3.544597961	595.9282429	453.4763815	805.7137885	483.7210131	401.3348519	578.8278209	1016.206397	932.1690079	980.6091385
4.99_447.2063m/z	447.206331	4.99225	neg	1-Methyl-6-phenyl-1H-imidazo[4,5-b]pyridin-2-amine	HMDB0041008	Organoheterocyclic compounds	Pyridines and derivatives	Phenylpyridines	**	C16038	38.1	0	2M-H	C13H12N4	2.709869766	1549.800601	1923.500359	2048.387373	1057.983611	1127.744058	1540.684484	2101.886557	1971.24536	1817.192069
4.99_487.1942m/z	487.1942029	4.99225	neg	PG(6:0/6:0)[U]	40849	Unclassified	Unclassified	Unclassified			38.1	0	M+FA-H	C18H35O10P	-1.772788539	793.6997497	944.6289359	579.2700084	1760.316345	1731.263391	1811.23077	1047.481966	1133.655692	695.2764103
5.01_199.1342m/z	199.134162	5.014716667	neg	(R)-(+)-alpha-Terpineol	LMPR0102090028	Lipids and lipid-like molecules	Prenol lipids	Monoterpenoids	*	C09902	38.1	0	M+FA-H	C10H18O	1.258496321	403.0724601	342.070592	418.0311401	462.1140133	453.6278453	444.3923112	509.8475987	489.4321732	366.361977
5.04_377.1821m/z	377.1821388	5.037216667	neg	(1R,2R,4S,6R)-2,6-Fenchanediol 2-O-b-D-glucoside	HMDB0033220	Lipids and lipid-like molecules	Prenol lipids	Terpene glycosides	*		38.1	0	M+FA-H	C16H28O7	1.302722093	2556.918954	2610.204188	2689.817913	1399.158916	1247.127005	1589.923012	1945.224322	2135.44381	1998.486183
5.06_353.0415m/z	353.0414723	5.05975	neg	Brassicanal A	HMDB0038591	Organoheterocyclic compounds	Indoles and derivatives	Indoles		C11048	38.1	0	2M-H	C9H7NOS	-2.601349116	503.4499024	615.5658489	627.1666592	92.63459362	0.001403199	50.68484143	1108.331422	727.3827095	1073.87426
5.08_505.1475m/z	505.1474911	5.080566667	neg	Thiorphan	65621	Unclassified	Unclassified	Unclassified			38.1	0	2M-H	C12H15NO3S	0.471899969	690.3161899	499.4308116	440.3838406	1093.449809	152.0023362	1124.003341	682.7577332	462.9477854	633.2913955
5.10_177.0557m/z	177.0557123	5.1031	neg	Cinnamaldehyde	6931	Phenylpropanoids and polyketides	Cinnamaldehydes	Unclassified	**	C00903	38.1	0	M+FA-H	C9H8O	-0.041114864	171.1809979	165.7014243	54.66540945	69.25153971	86.0923984	126.6073065	240.0478102	145.9075136	204.4383687
5.10_371.0964m/z	371.0964163	5.1031	neg	Bilobalide	73567	Lipids and lipid-like molecules	Prenol lipids	Terpene lactones	*	C07605	38.1	0	M+FA-H	C15H18O8	-5.992198447	476.4870513	575.8284121	390.7337647	943.3174325	641.6252829	1031.387962	711.4795305	775.1725697	707.3964777
5.16_495.1103m/z	495.1103274	5.155116667	neg	Tazobactam-M1	695	Unclassified	Unclassified	Unclassified			38.1	0	2M-H	C7H12N4O4S	3.531499506	6492.152352	7093.836442	6199.100489	5055.988277	4747.189851	5584.178226	6108.422408	6318.715436	6639.975842
5.20_451.1590m/z	451.1589866	5.20005	neg	4-Hydroxy-(3',4'-dihydroxyphenyl)-valeric acid	HMDB0041679	Organic acids and derivatives	Hydroxy acids and derivatives	Medium-chain hydroxy acids and derivatives	*		38.1	0	2M-H	C11H14O5	-4.387926725	5693.457474	5867.340972	5801.033883	4097.269775	3941.688159	4837.104282	6762.083241	5876.915089	6343.905592
5.30_257.1398m/z	257.1397501	5.302883333	neg	Putaminoxin	67030	Unclassified	Unclassified	Unclassified			38.1	0	M+FA-H	C12H20O3	1.427146397	804.4219952	802.7005243	1081.933877	1066.124881	1194.153492	1543.744914	545.1085989	802.2152933	803.9066944
5.30_433.2085m/z	433.2085167	5.302883333	neg	methyl 10,12,13,16-bisepidioxy-9-hydroperoxy-14-octadecenoate	74482	Lipids and lipid-like molecules	Fatty Acyls	Fatty Acids and Conjugates			38.1	0	M+FA-H	C19H32O8	1.534845617	3081.55702	3172.293758	3052.963457	4270.66784	5938.179798	6140.57636	4283.617862	3374.817443	4599.853182
5.34_533.1666m/z	533.1666134	5.3394	neg	Phellatin	50125	Lipids and lipid-like molecules	Polyketides	Flavonoids			38.1	0	M-H	C26H30O12	0.30602106	7224.664251	9174.685603	9799.659912	2757.858788	3650.150412	3948.733185	2578.322675	2838.550044	3141.158265
5.49_396.1123m/z	396.1123075	5.489016667	neg	Ethacrynic acid M2	2586	Unclassified	Unclassified	Unclassified			38.1	0	M-H	C18H23NO7S	0.153360339	2367.190448	2329.965907	2544.164855	1035.660914	665.0619808	960.1028062	1792.897711	1545.036252	1674.28978
5.49_527.2866m/z	527.286607	5.489016667	neg	2H-Indol-2-one, 1,3-dihydro-4-[2-hydroxy-3-[(1-methylethyl)amino]propoxy]-	1944	Unclassified	Unclassified	Unclassified			38.1	0	2M-H	C14H20N2O3	-1.70663549	3101.553624	2546.680944	2229.666428	3517.279063	3198.461178	4381.402867	2540.129215	2238.809883	2925.614884
5.55_312.1820m/z	312.181993	5.547916667	neg	Metoprolol	1250	Benzenoids	Phenols	Tyrosols and derivatives		C07202	38.1	0	M+FA-H	C15H25NO3	1.296864674	1386.059805	1298.676576	1111.956239	1693.426566	1939.967832	1679.720365	1429.298271	1711.308391	1814.789
5.56_377.1821m/z	377.1821415	5.56185	neg	(1R,2S,4R,5S)-2,5-Fenchanediol 2-O-b-D-glucoside	89088	Lipids and lipid-like molecules	Prenol lipids	Terpene glycosides	*		38.1	0	M+FA-H	C16H28O7	1.310777179	2038.330877	1930.709505	1890.355845	1870.187854	2605.53153	2241.852783	2401.482077	2251.435333	2156.884495
5.60_146.0248m/z	146.0247816	5.602	neg	3,4-Dihydroxymandelonitrile	66038	Unclassified	Unclassified	Unclassified			38.1	0	M-H2O-H	C8H7NO3	0.179758456	199.3774347	185.2012161	197.4283425	194.956179	146.3953079	128.2265363	230.5093096	163.1683565	161.6025616
5.61_345.2283m/z	345.2282714	5.610733333	neg	Indospicine	3299	Unclassified	Unclassified	Unclassified			38.1	0	2M-H	C7H15N3O2	7.781806903	1327.383409	1421.6011	1641.035737	880.4567296	792.1070457	654.8553618	843.3091696	982.6582832	862.3969022
5.63_549.1614m/z	549.1614471	5.628083333	neg	6-Hydroxy-5-methyl-3',4',5'-trimethoxyaurone-4-O-alpha-L-rhamnopyranoside	LMPK12130045	Unclassified	Unclassified	Unclassified			38.1	0	M+FA-H	C25H28O11	0.163713045	6205.465686	6013.113228	7542.107458	2969.583068	2418.765915	3055.347469	8915.855254	7746.675546	9004.557217
5.68_375.1664m/z	375.1664309	5.678766667	neg	Portuloside A	HMDB0031382	Lipids and lipid-like molecules	Fatty Acyls	Fatty acyl glycosides	*		38.1	0	M+FA-H	C16H26O7	1.13547462	1686.342821	1795.07257	1697.48096	2108.901638	2085.747329	2067.307523	1590.383382	1752.138448	1854.555741
5.68_377.1821m/z	377.1821336	5.678766667	neg	1,8-Epoxy-p-menthan-4-ol glucoside	HMDB0033215	Lipids and lipid-like molecules	Prenol lipids	Terpene glycosides	*		38.1	0	M+FA-H	C16H28O7	1.286903775	4306.643766	4043.736167	3844.855453	2484.04219	2665.138545	2666.495186	2269.645109	2672.022098	2887.168354
5.83_499.2168m/z	499.2168184	5.825	neg	3-(m-Aminophenyl)-2-(p-methoxyphenyl)acrylonitrile	70533	Unclassified	Unclassified	Unclassified			38.1	0	2M-H	C16H14N2O	5.734806195	1981.506008	2106.6561	2268.643293	1764.845019	1051.750824	1352.818794	2555.878999	2731.413865	2104.533836
5.85_409.2233m/z	409.2233424	5.84575	neg	Dihydrocortisol	HMDB0003259	Lipids and lipid-like molecules	Steroids and steroid derivatives	Hydroxysteroids	**	C05471	38.1	0	M+FA-H	C21H32O5	0.454057463	1860.700182	2176.783147	2401.185466	2109.699963	1742.821024	1470.63796	3072.202157	2805.501312	2166.077961
5.88_357.1018m/z	357.1018308	5.88025	neg	Lactobionic acid	44767	Unclassified	Unclassified	Unclassified			38.1	0	M-H	C12H22O12	-5.637556406	3513.01929	4371.11462	4162.709038	6302.354767	5698.561646	6368.467478	3304.535199	3607.633903	4546.863056
5.93_495.1101m/z	495.1100617	5.927283333	neg	Yuccaol A	91745	Phenylpropanoids and polyketides	2-arylbenzofuran flavonoids	Unclassified			38.1	0	M-H	C29H20O8	3.064798896	3132.183098	2928.021919	3269.506468	3216.709687	4453.38144	3836.523298	3537.02168	4369.39983	4329.232098
5.97_257.1397m/z	257.1396866	5.965483333	neg	12-hydroxy-3Z,6Z-dodecadienoic acid	LMFA01050171	Lipids and lipid-like molecules	Fatty Acyls	Fatty Acids and Conjugates			38.1	0	M+FA-H	C12H20O3	1.127830869	794.1060614	704.1224559	1002.360806	755.4268686	997.2043755	1139.183421	635.8257834	720.2148685	527.932021
5.99_661.2459m/z	661.2459294	5.9916	neg	5'-O-beta-D-Glucosylpyridoxine	66097	Unclassified	Unclassified	Unclassified			38.1	0	2M-H	C14H21NO8	-0.34347707	4354.82359	4606.160273	5031.121502	5598.385823	6432.112805	6539.260627	3636.014424	4114.469948	4167.997246
6.01_400.2344m/z	400.2344374	6.007266667	neg	3'-Hydroxybuspirone	HMDB0061108	Organoheterocyclic compounds	Diazinanes	Piperazines	*		38.1	0	M-H	C21H31N5O3	-2.432622612	613.3671126	546.5305177	537.7514072	1129.198815	879.8518947	1363.875191	530.8954577	494.0981531	490.7600885
6.01_471.1991m/z	471.1991113	6.007266667	neg	Carboxyibuprofen	692	Phenylpropanoids and polyketides	Phenylpropanoic acids	Unclassified	*		38.1	0	2M-H	C13H16O4	-7.052404305	2041.549914	1964.567505	2117.685158	1362.870341	982.1531428	990.5960854	1341.048644	1526.217071	1491.195992
6.04_155.1078m/z	155.1078427	6.040433333	neg	2,5-Dimethyl-2,4-hexadiene	HMDB0061902	Hydrocarbons	Unsaturated hydrocarbons	Branched unsaturated hydrocarbons			38.1	0	M+FA-H	C8H14	0.812050236	393.0508058	428.674139	313.6858444	441.1439318	397.4862547	471.5353477	266.5264959	219.6938626	259.1606238
6.10_751.1786m/z	751.1785723	6.101316667	neg	Resistomycin	69295	Unclassified	Unclassified	Unclassified			38.1	0	2M-H	C22H16O6	-4.689929843	2205.354407	2940.684574	2413.171413	1633.47236	1461.815793	1848.037732	2385.518209	2224.22065	1829.165386
6.11_373.1508m/z	373.1507931	6.113516667	neg	Perilloside B	94965	Lipids and lipid-like molecules	Prenol lipids	Terpene glycosides	*		38.1	0	M+FA-H	C16H24O7	1.179653819	2070.255742	2842.521228	2779.8028	1757.259792	1843.425902	1412.705159	2245.690109	2471.041843	2349.205992
6.15_455.0730m/z	455.0730094	6.154666667	neg	Phosphomevalonic acid	HMDB0256482	Organic acids and derivatives	Organic phosphoric acids and derivatives	Phosphate esters	**	C01107	38.1	0	2M-H	C6H13O7P	1.111815148	1742.188491	1808.184478	1814.013722	1103.148752	1170.925587	1366.197971	1089.313362	1128.462662	1263.824358
6.20_531.1510m/z	531.1510194	6.195716667	neg	Aquayamycin	63762	Unclassified	Unclassified	Unclassified			38.1	0	M+FA-H	C25H26O10	0.45153445	4096.282968	3080.894814	3648.865034	4738.513957	4629.551207	4799.831295	813.0004515	837.6703512	917.9072959
6.36_375.1666m/z	375.1666186	6.3617	neg	Epijasminoside A	89002	Organic oxygen compounds	Organooxygen compounds	Carbohydrates and carbohydrate conjugates	*		38.1	0	M+FA-H	C16H26O7	1.703797082	1244.2344	1514.165101	1380.883549	2043.279753	2634.211059	2507.644606	1349.098783	854.1662704	1524.546766
6.37_331.1402m/z	331.1401889	6.373833333	neg	Glutaminyl-Tryptophan	85722	Organic acids and derivatives	Carboxylic acids and derivatives	Amino acids, peptides, and analogues	*		38.1	0	M-H	C16H20N4O4	-2.979882116	22959.52813	24851.67656	26759.65681	25812.33457	26138.05443	25918.86432	23186.24018	23451.87083	22913.41723
6.39_365.1431m/z	365.1430501	6.386066667	neg	3b,16a-Dihydroxyandrostenone sulfate	57974	Lipids and lipid-like molecules	Steroids and steroid derivatives	Sulfated steroids	*		38.1	0	M-H2O-H	C19H28O6S	0.602987138	5711.981394	6384.161966	7467.441346	6517.640468	6939.002339	7083.684961	6421.821261	6696.893019	6396.054961
6.49_413.2183m/z	413.2182955	6.494283333	neg	19-hydroxyprostaglandin H2(1-)	HMDB0062683	Lipids and lipid-like molecules	Fatty Acyls	Eicosanoids	*		38.1	0	M+FA-H	C20H32O6	0.553817066	12588.94488	12623.87508	14093.51178	3170.513031	3136.490617	3007.724809	9830.298167	13017.86788	11058.22206
6.51_460.2708m/z	460.2708044	6.509966667	neg	15(R)-17-phenyl trinor Prostaglandin F2α ethyl amide	45000	Unclassified	Unclassified	Unclassified			38.1	0	M+FA-H	C25H37NO4	0.825589157	3228.645888	3765.406339	3891.488745	2621.751389	2731.594901	3032.227975	3375.983425	3847.494549	3819.174695
6.58_232.1014m/z	232.1014099	6.582416667	neg	Molinate	68828	Unclassified	Unclassified	Unclassified			38.1	0	M+FA-H	C9H17NOS	0.65150528	629.9083531	689.1174279	715.7710727	560.8959486	566.4425988	474.3599769	522.0917915	645.3416054	529.8173678
6.58_409.1119m/z	409.1118617	6.582416667	neg	Lipoamide	HMDB0000962	Organoheterocyclic compounds	Dithiolanes	Lipoamides	*	C00248	38.1	3.67	2M-H	C8H15NOS2	0.307069547	12335.83938	12897.41578	12049.14604	23939.85116	24646.22015	23752.98321	15651.03544	18231.05016	18174.19305
6.66_365.1245m/z	365.1245018	6.663683333	neg	Mycophenolic acid	HMDB0015159	Organoheterocyclic compounds	Isocoumarans	Isobenzofuranones	*	C20380	38.1	0	M+FA-H	C17H20O6	0.970010027	5161.631499	5502.893447	5474.574365	12028.36088	11860.77155	10413.73083	7497.634015	9093.638407	9269.784415
6.66_443.1361m/z	443.1360955	6.663683333	neg	S-Adenosylmethionine	3289	Nucleosides, nucleotides, and analogues	5'-deoxyribonucleosides	5'-deoxy-5'-thionucleosides	**	C00019	38.1	0	M+FA-H	C15H22N6O5S	1.642025106	2092.503886	2550.946394	2548.19159	589.9496921	574.9178676	702.94632	1626.197043	1312.76057	1878.3149
6.66_581.2973m/z	581.2972978	6.663683333	neg	Corchorosol A	95566	Lipids and lipid-like molecules	Steroids and steroid derivatives	Steroid lactones	*		38.1	0	M+FA-H	C29H44O9	1.047898126	17613.19439	20094.97828	20591.16712	5232.823533	5178.938332	4800.524984	9451.240555	12242.03406	13719.62277
6.75_207.1392m/z	207.1391543	6.747166667	neg	1,3-Diisopropylbenzene	HMDB0013806	Benzenoids	Benzene and substituted derivatives	Cumenes	*		38.1	0	M+FA-H	C12H18	0.621780542	1097.148088	1142.981586	1396.996636	249.5005163	208.5829896	175.3719851	809.669692	1042.655014	934.2248597
6.88_411.1276m/z	411.1276112	6.8759	neg	7-beta-D-Glucopyranosyloxybutylidenephthalide	90178	Organic oxygen compounds	Organooxygen compounds	Carbohydrates and carbohydrate conjugates			38.1	0	M+FA-H	C18H22O8	-5.624672717	116798.1354	121181.0529	108974.3808	122917.5126	132816.5831	121464.1159	110801.8049	117551.7163	106622.5926
6.90_237.1109m/z	237.1108526	6.896766667	neg	DL-Homoserine	288	Unclassified	Unclassified	Unclassified			38.1	0	2M-H	C4H9NO3	6.898920861	1537.189897	1878.142749	1847.137788	1710.334351	1736.617443	1548.936766	1334.253715	1756.249374	1295.842326
6.91_358.2239m/z	358.2238648	6.9072	neg	(4Z)-decenoylcarnitine	LMFA07070017	Lipids and lipid-like molecules	Fatty Acyls	Fatty esters			38.1	0	M+FA-H	C17H31NO4	1.128531126	4614.320061	5225.07303	3972.058884	6779.351375	7189.067694	6376.777608	4511.702231	4881.349226	4568.184304
6.96_285.0408m/z	285.0407677	6.96195	neg	ALIZARIN	43807	Unclassified	Unclassified	Unclassified			38.1	0	M+FA-H	C14H8O4	1.275199262	286.3412047	240.1873562	299.9903038	1624.580041	1192.08466	1433.608509	243.1914337	459.518066	352.6329832
6.99_469.2268m/z	469.2268477	6.993516667	neg	(R)-1-O-[b-D-Glucopyranosyl-(1->6)-b-D-glucopyranoside]-1,3-octanediol	HMDB0032799	Lipids and lipid-like molecules	Fatty Acyls	Fatty acyl glycosides	*		38.1	0	M-H	C20H38O12	-4.683837004	12331.81625	11837.95998	12496.09297	11228.02631	9542.163915	10443.36053	10121.75373	12349.92466	11963.96995
7.13_317.1607m/z	317.1607337	7.1264	neg	Eseramine	67484	Unclassified	Unclassified	Unclassified			38.1	0	M-H	C16H22N4O3	-3.710109992	16083.65545	16518.20295	16444.0161	14456.06675	16397.74625	12932.97541	12851.89161	15748.57906	15239.68188
7.14_409.2234m/z	409.2233592	7.138633333	neg	3a,11b,21-Trihydroxy-20-oxo-5b-pregnan-18-al	57884	Lipids and lipid-like molecules	Steroids and steroid derivatives	Hydroxysteroids	**	C05474	38.1	0	M+FA-H	C21H32O5	0.500176332	4663.923278	4358.718368	4720.832067	3661.093238	3146.417869	3185.315516	3053.981919	3526.764389	3509.814044
7.18_408.1440m/z	408.143981	7.178516667	neg	δ-(L-2-Aminoadipyl)-L-cysteinyl-D-valine	64549	Unclassified	Unclassified	Unclassified			38.1	0	M+FA-H	C14H25N3O6S	-1.730362727	2249.842277	2414.46229	2553.628592	3929.768862	3844.767216	4075.607152	1847.341413	2674.652767	1828.486088
7.18_571.1212m/z	571.1211797	7.178516667	neg	3-Hydroxy-L-tyrosyl-AMP	69049	Unclassified	Unclassified	Unclassified			38.1	0	M+FA-H	C19H23N6O10P	3.134742625	18456.63023	23208.44412	22121.57378	25245.20337	24529.93412	26102.62227	20092.60182	23290.39207	20355.1324
7.25_553.2297m/z	553.2297388	7.248333333	neg	Acetyl-T2 Toxin	HMDB0033164	Lipids and lipid-like molecules	Prenol lipids	Sesquiterpenoids	*		38.1	0	M+FA-H	C26H36O10	1.354928852	3852.357301	4549.556562	4079.41736	3101.492209	2940.808671	3233.609771	2639.54852	2163.284282	2826.990539
7.26_411.1655m/z	411.1654569	7.262266667	neg	Orizabin	67773	Unclassified	Unclassified	Unclassified			38.1	0	M+FA-H	C19H26O7	-1.636291241	5890.79305	6448.19135	6428.622399	8426.20984	8025.601654	7145.843686	6194.805972	6490.607299	6714.937385
7.30_673.2979m/z	673.2978548	7.29525	neg	Desmethylpirenzepine	1975	Unclassified	Unclassified	Unclassified			38.1	0	2M-H	C18H19N5O2	-3.883201903	3165.590501	3924.263643	3858.427226	147.0899572	0.001403199	0.001403199	1325.897426	1421.47216	2561.150674
7.55_647.2668m/z	647.2668003	7.547766667	neg	Glabranin	52653	Lipids and lipid-like molecules	Fatty Acyls	Fatty alcohols	*	C09752	38.1	0	2M-H	C20H20O4	2.712620008	16153.55097	17007.5147	16599.11949	20674.19451	21227.02929	20952.0892	14195.37535	16268.10159	14895.08713
7.55_783.2415m/z	783.2415242	7.547766667	neg	Dehydrorotenone	44627	Lipids and lipid-like molecules	Polyketides	Flavonoids			38.1	0	2M-H	C23H20O6	-4.04979466	5302.406519	4485.295412	5052.731503	6724.340942	6416.230228	6402.726261	3653.294927	4261.007224	5203.849754
7.65_279.1604m/z	279.160363	7.6458	neg	2,4-Hexadienyl acetate	HMDB0032311	Organic acids and derivatives	Carboxylic acids and derivatives	Carboxylic acid derivatives	*		38.1	0	2M-H	C8H12O2	0.642997903	702.3136138	819.6477694	548.0782333	145.4889785	147.6592896	145.1327868	323.2931986	542.6008445	451.7367607
7.81_311.1977m/z	311.1977121	7.814316667	neg	APHYLLIC ACID	44024	Unclassified	Unclassified	Unclassified			38.1	0	M+FA-H	C15H26N2O2	0.304672953	2685.098198	3026.702128	3450.028521	3083.528041	2266.732087	2943.086678	1843.502725	2461.975767	2384.702134
7.86_323.1978m/z	323.1977724	7.855233333	neg	Lycocernuine	67980	Unclassified	Unclassified	Unclassified			38.1	0	M+FA-H	C16H26N2O2	0.508483599	1146.298566	1491.16289	1463.884258	1829.60947	1821.420599	1812.895994	742.4559765	1259.57115	978.3728444
7.95_342.1738m/z	342.1738204	7.949583333	neg	2-(4-Methyl-5-thiazolyl)ethyl decanoate	88443	Organoheterocyclic compounds	Azoles	Thiazoles	*		38.1	0	M+FA-H	C16H27NO2S	-2.128631194	5918.721392	5732.024945	5908.09822	8055.601259	7751.864866	8029.899846	4080.256586	4820.681422	4421.687267
8.07_787.8457m/z	787.845678	8.066183333	neg	Amipaque	HMDB0259655	Organic oxygen compounds	Organooxygen compounds	Carbohydrates and carbohydrate conjugates			38.1	0	M-H	C18H22I3N3O8	-1.463133801	11012.96164	8495.081616	9061.880742	11512.86975	12410.16548	12807.42446	13197.07666	11438.91243	13089.45874
8.23_183.1027m/z	183.1026768	8.234666667	neg	(2E,6Z)-nona-2,6-dienal	LMFA06000117	Organic oxygen compounds	Organooxygen compounds	Carbonyl compounds		C08499	38.1	0	M+FA-H	C9H14O	0.063902079	495.7366217	588.3681813	689.8124412	629.3260237	654.7654241	690.9280545	563.4767513	545.1307658	594.758937
8.26_479.1898m/z	479.1897767	8.264766667	neg	2-Hydroxy-3-(4-methoxyethylphenoxy)-propanoic acid	1255	Unclassified	Unclassified	Unclassified			38.1	0	2M-H	C12H16O5	-5.1938076	834.4230046	1279.712099	1030.139823	1372.713621	809.2013178	997.5128816	838.8453837	945.1492074	548.2223085
8.43_457.2083m/z	457.2082615	8.430033333	neg	Prolyl-Asparagine	85910	Organic acids and derivatives	Carboxylic acids and derivatives	Amino acids, peptides, and analogues	*		38.1	0	2M-H	C9H15N3O4	6.60395096	1500.999381	1827.723402	1657.966441	1708.615513	1757.132243	1488.253025	851.9085034	1291.126999	1271.775053
8.89_313.2025m/z	313.2024931	8.888766667	neg	10,11-Epoxy-7Z-hexadecenoic acid	LMFA01070026	Lipids and lipid-like molecules	Fatty Acyls	Fatty Acids and Conjugates			38.1	0	M+FA-H	C16H28O3	1.66079442	1247.36817	1141.998247	1223.998135	940.244878	754.3432643	807.0245815	701.612784	945.3016682	851.9408151
9.02_615.1861m/z	615.1861101	9.01635	neg	Candesartan N2-glucuronide	HMDB0013843	Organic oxygen compounds	Organooxygen compounds	Carbohydrates and carbohydrate conjugates	*		38.1	0	M-H	C30H28N6O9	2.61280514	2423.921038	3887.524336	3668.837007	0.001403199	102.5449794	0.001403199	857.2846238	1049.656988	1165.238362
9.40_549.1044m/z	549.104399	9.396466667	neg	BL II	94124	Organic acids and derivatives	Carboxylic acids and derivatives	Pentacarboxylic acids and derivatives			38.1	0	M-H	C28H22O12	0.998588145	6391.828721	6239.92919	6040.541505	5342.242103	6009.929892	5778.800141	7392.394987	6470.496867	6891.710449
9.96_229.1810m/z	229.1809972	9.960433333	neg	2-Methylundecanal	HMDB0031734	Organic oxygen compounds	Organooxygen compounds	Carbonyl compounds	*		38.1	0	M+FA-H	C12H24O	0.428358556	1030.732684	1148.473327	917.3158869	760.5445437	707.2440133	968.4668868	680.852934	952.9943714	1023.631198
0.67_327.0664m/z	327.0664396	0.6701	neg	6-sulfatoxymelatonin	96149	Organic acids and derivatives	Carboxylic acids and derivatives	Carboxylic acid derivatives	*		38	0	M-H	C13H16N2O6S	2.465216282	142.8120078	156.2662112	112.228919	880.385023	777.6005044	760.7713484	534.3109401	544.9543076	393.1184361
0.78_360.0720m/z	360.0720039	0.778533333	neg	Gonyautoxin V	HMDB0033513	Phenylpropanoids and polyketides	Saxitoxins, gonyautoxins, and derivatives	Unclassified			38	12.1	M-H2O-H	C10H17N7O7S	-3.091341316	6938.787906	6550.378845	7747.448724	29378.96304	28151.70497	29258.22175	12766.60242	15280.94271	15497.7952
0.82_203.1038m/z	203.1037878	0.819566667	neg	Serylvaline	HMDB0029052	Organic acids and derivatives	Carboxylic acids and derivatives	Amino acids, peptides, and analogues	*		38	0	M-H	C8H16N2O4	0.280340871	1737.092787	1469.609151	1259.287069	693.315363	463.3099308	508.2853918	1439.917183	1525.969242	1185.624781
0.86_309.0458m/z	309.0457592	0.86335	neg	Cabbage identification factor 2	HMDB0033622	Organic acids and derivatives	Carboxylic acids and derivatives	Amino acids, peptides, and analogues			38	5.25	M-H2O-H	C15H12N4O3S	1.795694646	8363.27	8524.288296	8883.950613	7987.993777	10519.26057	9512.984953	10412.60607	11177.69654	12653.87818

1.22_151.0072m/z	151.0071706	1.224233333	neg	S-Methylthioglycolate	44691	Organic acids and derivatives	Carboxylic acids and derivatives	Carboxylic acid derivatives	*	C03173	38	0	M+FA-H	C3H6O2S	1.106896748	320.2951068	426.3586743	361.10999	408.2356221	81.95174335	225.1932208	347.4666625	264.7738335	81.09976645
1.53_273.0623m/z	273.0623258	1.534483333	neg	5-Diazoimidazole-4-carboxamide	624	Unclassified	Unclassified	Unclassified			38	0	2M-H	C4H3N5O	7.599366037	982.1959286	991.1894101	819.9885418	837.0987394	781.1899658	842.1218662	907.2281922	870.0664005	1082.36491
1.55_188.0930m/z	188.0930234	1.553483333	neg	4-(Trimethylammonio)but-2-enoate	66118	Unclassified	Unclassified	Unclassified			38	0	M+FA-H	C7H13NO2	1.340921751	93.66196062	164.4742875	131.4008201	187.9373929	199.3520954	224.1415633	117.0161985	171.8797054	111.1755312
1.59_329.0882m/z	329.0882218	1.5934	neg	D-Vacciniin	HMDB0038492	Benzenoids	Benzene and substituted derivatives	Benzoic acids and derivatives			38	0	M+FA-H	C13H16O7	1.464581904	2009.53604	1774.348398	1940.834955	1399.595602	1581.352385	1587.946855	1886.830596	1844.346281	1792.038467
1.65_116.0176m/z	116.0175929	1.65405	neg	DL-Homocysteine	63636	Organic acids and derivatives	Carboxylic acids and derivatives	Amino acids, peptides, and analogues	**	C00155	38	0	M-H2O-H	C4H9NO2S	0.25541536	119.0289527	130.8923942	151.7923552	32.08570094	20.52155473	21.59636688	111.5225086	142.3730311	107.6175545
1.72_293.0882m/z	293.0881724	1.721216667	neg	Serotinose	HMDB0029897	Organic oxygen compounds	Organooxygen compounds	Carbohydrates and carbohydrate conjugates	*		38	0	M-H2O-H	C11H20O10	1.174777884	1115.403244	1097.28135	785.00791	1539.451004	1531.653005	1415.891649	1144.761134	1135.965214	1261.941514
1.72_367.1248m/z	367.1247593	1.721216667	neg	isoflurophate	43220	Organic acids and derivatives	Organic phosphoric acids and derivatives	Phosphate esters		C00202	38	0	2M-H	C6H14FO3P	-2.397503845	318.0928234	332.3754972	226.0753977	325.7418853	304.9554912	265.0900269	251.4675348	361.171196	411.5590611
1.72_392.1205m/z	392.1205052	1.721216667	neg	Dide-O-methylsimmondsin	HMDB0041207	Organic oxygen compounds	Organooxygen compounds	Carbohydrates and carbohydrate conjugates			38	0	M+FA-H	C14H21NO9	1.933162967	648.2733432	1117.890467	642.7346573	1414.540619	1268.851299	1281.851697	925.2618318	1033.248909	1104.481701
10.36_243.1967m/z	243.1966598	10.3649	neg	tridecanal	LMFA06000074	Lipids and lipid-like molecules	Fatty Acyls	Fatty aldehydes	*		38	0	M+FA-H	C13H26O	0.461536045	1304.750586	1485.16267	1212.608627	1578.282006	1450.055575	1597.074656	988.361955	1661.814131	1336.679257
10.71_271.2281m/z	271.228063	10.70671667	neg	2-Pentadecanone	HMDB0031081	Organic oxygen compounds	Organooxygen compounds	Carbonyl compounds	*		38	0	M+FA-H	C15H30O	0.859761642	5369.718337	5536.102903	5040.074304	4614.227438	5176.427887	4895.899552	5373.546432	4889.295765	4930.98409
11.52_323.1716m/z	323.1715751	11.51641667	neg	beta-Cymaropyranose	66853	Unclassified	Unclassified	Unclassified			38	0	2M-H	C7H14O4	1.337877995	120.3685894	211.4095701	738.4776975	135.962641	78.65545417	150.8539929	130.617378	0.001403199	366.54892
11.95_271.2280m/z	271.2280492	11.95381667	neg	Pentadecanal	HMDB0031078	Lipids and lipid-like molecules	Fatty Acyls	Fatty aldehydes	*	C01948	38	0	M+FA-H	C15H30O	0.798841352	2100.166099	1905.383395	2419.640876	1625.822309	2232.50857	1858.725699	2344.531209	2351.888236	2265.918955
13.89_479.1176m/z	479.1176486	13.88881667	neg	(1R,6R)-6-Hydroxy-2-succinylcyclohexa-2,4-diene-1-carboxylate	63986	Unclassified	Unclassified	Unclassified			38	0	2M-H	C11H12O6	-3.855537413	12847.07145	7861.300944	6652.170254	5481.701509	5476.587776	5332.770424	6606.72281	11404.23781	6554.646782
13.94_168.9894m/z	168.9893596	13.93851667	neg	Thiazole	HMDB0029713	Organoheterocyclic compounds	Azoles	Thiazoles	*	C02740	38	0	2M-H	C3H3NS	-3.553844595	715579.1768	699330.7185	684104.8655	676370.1536	715754.8219	695046.6321	793039.504	705941.7907	718651.9829
14.70_149.0095m/z	149.0094894	14.69558333	neg	meso-Tartaric acid	63253	Unclassified	Unclassified	Unclassified			38	0	M-H	C4H6O6	2.185599681	40301.58295	812.1822368	607.4811405	7859.765353	1179.270239	857.9477953	413.0555738	739.5462499	490.6198354
2.00_273.0625m/z	273.0625387	2.004766667	neg	2-Hydroxy-3-carboxy-6-oxo-7-methylocta-2,4-dienoate	66438	Unclassified	Unclassified	Unclassified			38	0	M+FA-H	C10H12O6	4.155513818	5783.921686	5555.045884	5919.613971	3570.068426	3114.859453	3248.914162	3772.800216	4758.979309	4000.035953
2.15_359.0628m/z	359.062768	2.154666667	neg	Dantrolene	HMDB0015350	Organoheterocyclic compounds	Azolidines	Imidazolidines		C06939	38	0	M+FA-H	C14H10N4O5	-1.764856565	3837.957978	3775.629692	4062.861254	4093.301124	3700.91195	3730.401783	2748.392807	3325.132111	3183.188862
2.15_367.0646m/z	367.0645646	2.154666667	neg	Methyl 2,4,6-trihydroxybenzoate	86398	Benzenoids	Benzene and substituted derivatives	Benzoic acids and derivatives	*		38	0	2M-H	C8H8O5	-6.807541929	2334.151918	2812.544214	2892.769868	3817.145418	4603.49865	4553.154813	2338.239052	3013.675847	3264.792155
2.79_181.0984m/z	181.0983689	2.790116667	neg	N,N-Dimethyl-1,4-phenylenediamine	66134	Unclassified	Unclassified	Unclassified			38	0	M+FA-H	C8H12N2	0.864548209	463.0301001	497.2734406	332.111362	481.6771051	427.8201137	454.3021453	493.694904	477.1248054	636.7047217
2.89_299.0345m/z	299.0345177	2.894666667	neg	N-acetyl zonisamide	HMDB0060603	Organoheterocyclic compounds	Benzisoxazoles	Unclassified	*		38	0	M+FA-H	C10H10N2O4S	0.736280448	3228.238683	3276.114182	3474.632828	5511.175686	5647.586994	5675.309942	3779.882151	3847.177578	3531.295305
3.13_261.0075m/z	261.0074833	3.131933333	neg	Dihydrocaffeic acid 3-sulfate	HMDB0041721	Organic acids and derivatives	Organic sulfuric acids and derivatives	Arylsulfates	*		38	0	M-H	C9H10O7S	0.13762556	5158.86412	5839.591603	6279.285496	6845.767356	6237.16845	5694.706387	5098.041586	6074.292513	5923.330154
3.46_291.0964m/z	291.0964404	3.455516667	neg	2-methyl-1,3-thiazolidine-2-carboxamide	HMDB0062599	Organic acids and derivatives	Carboxylic acids and derivatives	Amino acids, peptides, and analogues			38	0	2M-H	C5H10N2OS	3.248750608	1096.645272	1173.363299	1478.490636	1489.353966	1262.817453	1407.287324	731.5351441	875.4405462	786.9778154
3.60_314.1006n	359.0986992	3.601516667	neg	6-O-Acetylarbutin	HMDB0034060	Organic oxygen compounds	Organooxygen compounds	Carbohydrates and carbohydrate conjugates			38	0	M-H, M+FA-H	C14H18O8	1.399539419	3680.23253	5047.393049	4495.457823	3081.810674	3142.386321	4189.233731	3254.619089	4285.484316	4029.726362
3.63_193.0541m/z	193.0541082	3.630433333	neg	3-(Methylthio)propyl acetate	87890	Organic acids and derivatives	Carboxylic acids and derivatives	Carboxylic acid derivatives	*		38	0	M+FA-H	C6H12O2S	0.707035178	619.6046114	726.3632595	608.4422667	223.4872912	194.8759242	236.3715513	726.6697551	614.1090307	737.258703
3.73_180.0668m/z	180.0667656	3.730516667	neg	N-Methylformamide	58023	Organic acids and derivatives	Carboximidic acids and derivatives	Carboximidic acids	*	C11489	38	0	M+FA-H	C8H9NO	1.101614529	156.51974	133.0216375	111.2629177	151.5838321	179.824177	122.8476371	86.4325174	101.4936781	113.8996726
3.75_633.1174m/z	633.1173632	3.747833333	neg	Nilutamide	HMDB0014803	Organoheterocyclic compounds	Azolidines	Imidazolidines		C08164	38	0	2M-H	C12H10F3N3O4	-0.065128899	763.9631534	875.0777469	966.0660095	3287.612078	1997.540931	1536.216573	1608.990499	1322.370285	1136.377517
3.82_274.0393m/z	274.0393137	3.82415	neg	L-Tyrosine methyl ester 4-sulfate	66133	Unclassified	Unclassified	Unclassified			38	0	M-H	C10H13NO6S	0.843562443	9078.239238	9534.910161	9331.261407	7804.739341	7392.019833	6976.333299	7646.116727	8325.35926	8949.542378
3.85_529.1204m/z	529.1204061	3.84605	neg	Diospyrin	LMPK12020205	Phenylpropanoids and polyketides	Flavonoids	Flavonoid glycosides			38	0	M+FA-H	C21H24O13	1.058522045	4319.725948	4409.908552	5113.427856	3024.965257	2497.12726	2798.660786	3947.285029	3883.450905	3654.565441
3.91_380.9975m/z	380.9974537	3.912566667	neg	Octulose-1,8-bisphosphate	157	Unclassified	Unclassified	Unclassified			38	0	M-H2O-H	C8H18O14P2	-4.709125061	2065.91275	2213.216927	2701.258288	0.001403199	70.75993698	0.001403199	2095.45461	1991.508188	1784.66209
3.98_232.0828m/z	232.0827567	3.97985	neg	dimethyl (R)-pyrrolidine-1,2-dicarboxylate	265049	Unclassified	Unclassified	Unclassified			38	0	M+FA-H	C8H13NO4	0.512934063	151.6306414	67.72466791	141.3644407	69.32303424	181.0015154	125.1911497	112.1915517	49.15202141	175.5883951
4.02_198.0504n	197.0431552	4.024283333	neg	(E)-2-(hexa-3,5-dien-1-yn-1-yl)-5-(prop-1-yn-1-yl)thiophene	LMFA12000346	Lipids and lipid-like molecules	Fatty Acyls	Oxygenated hydrocarbons			38	3.27	M-H, 2M-H	C13H10S	0.556772721	3081.0252	3701.780326	3721.897785	5169.914638	5790.550081	5050.568641	4796.431132	4836.521035	4665.658037
4.05_288.0550m/z	288.0550261	4.046666667	neg	Mono-N-depropylprobenecid	2091	Unclassified	Unclassified	Unclassified			38	0	M+FA-H	C10H13NO4S	1.211018021	825.6358623	931.8945043	902.1442892	251.6512928	287.3347472	177.588362	441.9026488	333.6390076	335.4218873
4.07_180.0125m/z	180.0125381	4.069216667	neg	Pifithrin-μ	96447	Unclassified	Unclassified	Unclassified			38	0	M-H	C8H7NO2S	0.35967127	227.4454656	223.6382245	234.5942025	24.9928497	46.37124363	16.62185519	339.2121252	289.8407544	246.7895836
4.09_417.1406m/z	417.1405829	4.091783333	neg	Syringin	64181	Unclassified	Unclassified	Unclassified			38	0	M+FA-H	C17H24O9	0.934167084	1024.62173	1038.756504	935.1427748	1341.692445	987.1500864	965.2004882	1070.359472	1230.413676	994.257898
4.11_285.1458m/z	285.1457903	4.113883333	neg	2-Ethyl-2,5-dihydro-4,5-dimethylthiazole	92144	Organoheterocyclic compounds	Azolines	Thiazolines			38	0	2M-H	C7H13NS	-2.355761866	1435.228514	1183.536458	1354.03722	3237.005033	2607.401277	3115.191255	1718.877443	1854.599881	1629.896053
4.11_443.1176m/z	443.1176333	4.113883333	neg	CBHA	44839	Unclassified	Unclassified	Unclassified			38	0	2M-H	C10H10N2O4	-7.213849592	2508.038779	3232.21278	3254.110075	3064.616736	3095.763173	2974.883791	2892.728726	2615.477835	2612.920509
4.18_289.0389m/z	289.0388535	4.181333333	neg	4-Hydroxy-5-(4'-hydroxyphenyl)-valeric acid-4'-O-sulphate	HMDB0059976	Lipids and lipid-like molecules	Fatty Acyls	Fatty acids and conjugates	*		38	0	M-H	C11H14O7S	0.365886784	2182.502002	1505.572181	1911.987331	143.0673868	129.0796911	177.1099296	6934.535827	5058.321181	5826.952535
4.23_194.0460m/z	194.0459893	4.234483333	neg	2-Formylaminobenzaldehyde	6090	Unclassified	Unclassified	Unclassified			38	0	M+FA-H	C8H7NO2	0.724531569	1139.965457	997.6648535	1103.99003	989.6609665	879.6419006	894.4606142	844.040153	882.8306206	683.5300104
4.26_144.0455m/z	144.0455261	4.2568	neg	Quinolin-2-ol	66376	Unclassified	Unclassified	Unclassified			38	0	M-H	C9H7NO	0.266408726	304.0847795	176.5549848	138.1124598	268.7795987	306.4278661	420.5563518	468.9478354	203.3620959	375.8098911
4.28_241.1678n	222.1499268	4.278183333	neg	Valeroidine	68643	Unclassified	Unclassified	Unclassified			38	0	M-H2O-H, M-H	C13H23NO3	-0.106545321	1367.617441	1406.282693	1551.196867	875.8357356	804.5439403	791.0412079	1249.097874	1447.68755	1285.448484
4.37_328.1768m/z	328.1767849	4.367166667	neg	α-Hydroxymetoprolol	1253	Benzenoids	Phenol ethers	Unclassified	*		38	0	M+FA-H	C15H25NO4	0.790058725	394.9521291	350.0206081	421.8514968	1059.412803	709.1303587	775.3068699	316.4591152	420.5961914	333.7142802
4.52_401.1819m/z	401.1819142	4.521733333	neg	Methyl 7-epi-12-hydroxyjasmonate glucoside	HMDB0031763	Lipids and lipid-like molecules	Fatty Acyls	Fatty acyl glycosides	*		38	0	M-H	C19H30O9	0.517419165	15686.58002	17370.49693	15949.35711	10364.09725	10613.90486	10581.39974	13583.53101	14002.56958	14282.27103
4.59_364.1883m/z	364.1882921	4.589283333	neg	PC(O-6:0/2:0)[U]	40153	Unclassified	Unclassified	Unclassified			38	0	M-H2O-H	C16H34NO7P	-3.016201564	683.4346681	634.9250246	754.048482	1915.874727	1912.418598	1491.708106	994.4991218	945.9409669	1149.852722
4.63_163.0403m/z	163.0402641	4.632666667	neg	cis-p-Coumaric acid	HMDB0030677	Phenylpropanoids and polyketides	Cinnamic acids and derivatives	Hydroxycinnamic acids and derivatives	*	C06738	38	0	M-H	C9H8O3	1.197168022	494.9367893	483.1995847	516.3809401	698.4341357	677.8276895	724.1923572	822.3145574	673.6819264	735.9651634
4.63_379.1612m/z	379.1611647	4.632666667	neg	Diethyl L-malate	94751	Organic acids and derivatives	Hydroxy acids and derivatives	Beta hydroxy acids and derivatives			38	0	2M-H	C8H14O5	0.510468588	703.6299317	351.0656891	690.1960301	1183.740876	1179.684459	1098.087556	848.5871558	709.306325	398.1399368
4.70_231.1238m/z	231.1238113	4.703166667	neg	3-Hydroxy-5Z-octenyl acetate	86921	Lipids and lipid-like molecules	Fatty Acyls	Fatty alcohol esters	*		38	0	M+FA-H	C10H18O3	0.07509355	1062.976563	1114.231061	1222.884973	1606.040168	1480.655405	1434.898736	869.2031462	826.8989057	1004.132427
4.70_512.1752m/z	512.1751857	4.703166667	neg	4-Hydroxytamoxifen sulfate	HMDB0061123	Phenylpropanoids and polyketides	Stilbenes	Unclassified	*		38	0	M+FA-H	C26H29NO5S	0.725302259	610.2906638	1147.042541	1221.106143	741.1280687	529.528967	745.0594948	206.7574191	562.1250458	137.6921699
4.73_230.0566m/z	230.0566132	4.725416667	neg	Cyometrinil	72859	Unclassified	Unclassified	Unclassified			38	0	M+FA-H	C10H7N3O	-2.710201703	351.1752763	367.3408747	401.0810654	515.4086395	456.2922411	529.2817439	607.1138018	605.0386954	684.4114811
4.73_515.0851m/z	515.0851434	4.725416667	neg	dTDP-β-L-rhodinose	63787	Unclassified	Unclassified	Unclassified			38	0	M-H	C16H26N2O13P2	2.727593202	1884.609965	2177.574068	2172.045206	5765.779858	4762.738182	4343.526523	2013.454686	2692.48798	2119.383572
4.75_197.0457m/z	197.0456993	4.747466667	neg	4-ACETOXYPHENOL	44202	Benzenoids	Phenol esters	Unclassified	**	C13636	38	0	M+FA-H	C8H8O3	1.001755606	614.8881121	606.5406349	719.2797063	581.1805652	607.9714304	442.622166	485.8504137	626.3992512	595.5904284
4.81_537.1611m/z	537.1610749	4.814166667	neg	4,2'-Dihydroxy-3,4',6'-trimethoxychalcone 4-glucoside	52129	Unclassified	Unclassified	Unclassified			38	0	M+FA-H	C24H28O11	-0.588541061	4761.811913	4983.912855	5168.906835	0.001403199	175.1341621	135.7439157	3595.649484	3581.43469	3445.325605
4.86_217.9766m/z	217.9765871	4.858383333	neg	4-Nitrophenyl sulfate	58427	Organic acids and derivatives	Organic sulfuric acids and derivatives	Arylsulfates	*		38	0	M-H	C6H5NO6S	0.482277938	1457.841415	1490.550729	1547.237897	567.9506024	619.2781039	483.2463589	705.877023	814.6237381	738.3097246
4.88_263.0514m/z	263.0514478	4.880083333	neg	bis(4-fluorophenyl)-Methanone	2735	Unclassified	Unclassified	Unclassified			38	0	M+FA-H	C13H8F2O	-4.936411042	21800.90427	23293.27772	23799.56378	22882.05325	23807.50721	23687.27411	18162.50135	17731.07027	17564.67666
4.90_383.0987m/z	383.0986675	4.902316667	neg	1-Caffeoyl-4-deoxyquinic acid	68322	Unclassified	Unclassified	Unclassified			38	0	M+FA-H	C16H18O8	0.8788082	1629.952593	1871.237976	2043.489822	1256.048534	1197.464124	1207.466312	899.0587948	898.7174439	966.309285
4.95_185.1184m/z	185.1183614	4.94705	neg	trans-4-Nonenal	HMDB0032535	Organic oxygen compounds	Organooxygen compounds	Carbonyl compounds	*		38	0	M+FA-H	C9H16O	0.309411102	658.3720569	461.3550626	499.1894428	546.4282637	556.7125245	664.247428	454.5571869	692.1292032	336.1514808
4.97_447.0933m/z	447.0932583	4.969683333	neg	7-Hydroxy-5,6,8,3'-tetramethoxy-4',5'-methylenedioxyflavone	49918	Unclassified	Unclassified	Unclassified			38	0	M+FA-H	C20H18O9	-0.066429951	1959.510354	2045.86377	1682.938449	182.4880924	136.0215252	311.9504353	79.07241786	87.51530457	101.1275402
4.97_539.1727m/z	539.1726617	4.969683333	neg	DALBERGIONE, 4-METHOXY-4'-HYDROXY-	43919	Unclassified	Unclassified	Unclassified			38	0	2M-H	C16H14O4	2.814380836	2132.237901	2181.603408	2384.977738	2035.027519	1941.334672	1921.608368	3885.258367	3497.915144	2720.071744
4.99_253.0711m/z	253.0710785	4.99225	neg	5-Amino-4-imidazole carboxylate	63404	Unclassified	Unclassified	Unclassified			38	0	2M-H	C4H5N3O2	7.879877309	1448.087869	1402.273714	1395.459538	1554.961388	1574.778189	1812.504898	2116.933347	1765.061716	1507.888762
5.01_555.2452m/z	555.2452058	5.014716667	neg	N1-(alpha-D-ribosyl)-5,6-dimethyl-benzimidazole	61979	Nucleosides, nucleotides, and analogues	Benzimidazole ribonucleosides and ribonucleotides	Unclassified	**	C05775	38	0	2M-H	C14H18N2O4	-1.495609182	7732.143036	8123.434097	8520.137188	7697.736186	8961.946231	9762.648152	6338.459289	7600.923135	7584.527197
5.04_385.1871m/z	385.1870534	5.037216667	neg	2,3-Dinor-6-keto-PGF1a	2560	Lipids and lipid-like molecules	Fatty Acyls	Eicosanoids	*		38	0	M+FA-H	C18H28O6	0.76992779	2768.176865	3020.757525	3186.07773	1468.156273	1276.434042	1248.793113	2544.499883	2199.005083	3348.404805
5.06_521.1635m/z	521.1635003	5.05975	neg	Pisatoside	93725	Organic oxygen compounds	Organooxygen compounds	Carbohydrates and carbohydrate conjugates			38	0	2M-H	C10H15NO7	2.055013071	1017.998575	1043.862182	754.492746	3545.067174	2541.788439	2746.137178	832.7476041	712.9233451	746.9914093
5.13_333.1071m/z	333.1071187	5.132616667	neg	Isopyridoxal	63863	Organoheterocyclic compounds	Pyridines and derivatives	Pyridine carboxaldehydes	**	C06051	38	0	2M-H	C8H9NO3	-6.258732677	5476.158822	5273.248398	4188.289044	3106.345178	3068.082453	3059.286311	5407.327741	5046.97164	6898.231898
5.26_311.0651m/z	311.0651448	5.263033333	neg	Thio-THIP	69652	Unclassified	Unclassified	Unclassified			38	0	2M-H	C6H8N2OS	3.05520563	958.088616	619.0333235	742.1706596	525.5185472	323.902745	369.2777569	341.6288414	147.5341273	231.0355691
5.27_679.2565m/z	679.2565296	5.271716667	neg	Dolichin B	LMPK12070015	Phenylpropanoids and polyketides	Isoflavonoids	Furanoisoflavonoids	*		38	0	2M-H	C20H20O5	2.438169517	13940.23583	8749.367365	9104.037704	7886.740498	8443.46925	7423.068265	13280.97136	8015.735238	12249.76218
5.29_577.1123m/z	577.112321	5.28555	neg	Dianthramine	67009	Unclassified	Unclassified	Unclassified			38	0	2M-H	C14H11NO6	4.018723002	2695.347799	2238.667067	2568.325365	1369.141877	1428.069948	2189.279957	1689.997286	799.8596954	1498.722246
5.41_381.1285m/z	381.1284798	5.406433333	neg	3-iodo-hexadecanoic acid	LMFA01090038	Lipids and lipid-like molecules	Fatty Acyls	Fatty Acids and Conjugates			38	0	M-H	C16H31IO2	-2.936051458	1112.31961	1722.578929	1592.78624	2611.993238	2502.498205	2730.321273	1882.773412	1864.950318	2283.912001
5.43_245.0562m/z	245.0562213	5.428733333	neg	Niacin (Nicotinic acid)	240	Unclassified	Unclassified	Unclassified			38	0	2M-H	C6H5NO2	-2.272182205	1339.474025	1134.003752	1096.961267	1002.784021	888.7263099	537.7678638	634.0196543	973.3691013	1081.064519
5.50_615.1336m/z	615.1336112	5.497816667	neg	3',4'-Methylenedioxy-[2",3":7,8]furanoflavanone	52558	Unclassified	Unclassified	Unclassified			38	0	2M-H	C18H12O5	6.395816845	2751.811866	2274.579065	1589.13065	538.7283894	753.0516003	949.8247065	0.001403199	0.001403199	0.001403199
5.61_505.0988m/z	505.0988104	5.610733333	neg	Glycitein 4'-O-glucuronide	96098	Phenylpropanoids and polyketides	Isoflavonoids	Isoflavonoid O-glycosides	*		38	0	M+FA-H	C22H20O11	0.100111725	1262.609255	1409.665021	1716.746504	831.4908104	685.8687246	709.9593559	1325.324702	1412.607346	1032.775421
5.63_405.0804m/z	405.0804104	5.628083333	neg	Nitrovin	73181	Unclassified	Unclassified	Unclassified			38	0	M+FA-H	C14H12N6O6	1.042490916	2097.725573	2131.512293	2508.15156	1981.328887	1549.410844	1453.946367	1766.44787	1811.643198	1954.596816
5.63_499.2167m/z	499.216695	5.628083333	neg	Methaqualone	66682	Organoheterocyclic compounds	Diazanaphthalenes	Benzodiazines		C07560	38	0	2M-H	C16H14N2O	5.488133018	2794.551891	3183.148754	3940.192072	3528.670218	2994.489235	3237.575058	3520.637452	3354.88086	3275.944688
5.71_318.1680n	363.166451	5.709933333	neg	LY171883	43454	Unclassified	Unclassified	Unclassified			38	0	M-H2O-H, M+FA-H	C16H22N4O3	-3.881086147	12104.32855	11896.08377	10751.28771	12304.58596	15582.8041	15502.5863	10557.58917	12445.16021	15156.93391
5.73_473.1137m/z	473.1137071	5.727283333	neg	Cartap	HMDB0249690	Unclassified	Unclassified	Unclassified			38	0	2M-H	C7H15N3O2S2	-0.324646789	514.0040807	692.1493714	517.2710645	1760.62226	2506.042028	1921.850075	551.3775255	1179.438046	1611.402179
5.74_413.1437m/z	413.143749	5.744566667	neg	4-Hydroxy-3-prenylbenzoic acid glucoside	HMDB0039891	Organic oxygen compounds	Organooxygen compounds	Carbohydrates and carbohydrate conjugates			38	0	M+FA-H	C18H24O8	-4.268894057	23849.66747	23443.94281	24844.37765	8539.428952	10944.87881	9419.275837	23096.98819	22706.92813	23314.3704
5.74_527.2865m/z	527.2864625	5.744566667	neg	SAHA	45206	Benzenoids	Benzene and substituted derivatives	Unclassified			38	0	2M-H	C14H20N2O3	-1.98013512	4908.892828	5032.065484	4361.533857	3887.157328	5427.261883	4672.744524	5998.289713	6884.021356	6073.633533
5.76_311.0209m/z	311.0209433	5.7632	neg	Blighinone	HMDB0030643	Benzenoids	Phenanthrenes and derivatives	Hydrophenanthrenes	*		38	0.662	M-H2O-H	C16H10O8	3.687785259	68771.93274	76549.86651	75104.06333	41916.1501	46461.25943	42647.20795	95467.44097	88572.34685	77513.56183
5.83_561.0170m/z	561.0169705	5.825	neg	Uridine diphosphate glucuronic acid	HMDB0000935	Organoheterocyclic compounds	Lactones	Gamma butyrolactones	**	C00167	38	0	M-H2O-H	C15H22N2O18P2	0.907749295	5863.039353	5759.464571	6742.804951	9381.66054	5806.868448	5884.982806	5874.475533	4908.585395	4626.967752
5.91_265.1058m/z	265.1058358	5.908066667	neg	4-Methylthiobutanaldoxime	64485	Unclassified	Unclassified	Unclassified			38	0	2M-H	C5H11NOS	3.165293861	5314.485453	5987.918583	6120.648525	2882.391463	3469.196889	3232.851352	2570.969294	3039.830024	3131.598611
5.99_811.2003m/z	811.2002644	5.9916	neg	Rotenonone	LMPK12060070	Lipids and lipid-like molecules	Polyketides	Flavonoids			38	0	2M-H	C23H18O7	-3.650516174	7614.474139	6992.675194	7088.842902	6626.608149	6268.223419	6790.387215	6017.355935	6463.436071	5659.033697
6.01_222.0774m/z	222.0774104	6.007266667	neg	1,2-Dehydrosalsolinol	HMDB0012490	Organoheterocyclic compounds	Dihydroisoquinolines	Unclassified	*		38	0	M+FA-H	C10H11NO2	1.292673647	617.1238299	684.6908169	818.1695762	491.4474658	519.6041862	559.7531113	552.7596293	576.4850512	430.5872089
6.01_879.1869m/z	879.1868971	6.007266667	neg	3,5-Dihydroxyphenyl 1-O-(6-O-galloyl-beta-D-glucopyranoside)	93891	Organic oxygen compounds	Organooxygen compounds	Carbohydrates and carbohydrate conjugates			38	0	2M-H	C19H20O12	3.659852949	3572.293233	4005.792345	4140.042079	3924.607647	4602.658072	4224.138025	2385.168996	3392.441686	2715.834225
6.02_298.1121m/z	298.1120754	6.01945	neg	Fenothiocarb	68826	Unclassified	Unclassified	Unclassified			38	0	M+FA-H	C13H19NO2S	0.87974707	32.29212504	84.06049549	77.19301686	1185.375775	1240.349443	1138.795477	349.5704108	289.0179303	352.0672368
6.07_295.1553m/z	295.1552782	6.07	neg	Blennin A	88017	Lipids and lipid-like molecules	Prenol lipids	Terpene lactones	*		38	0	M+FA-H	C15H22O3	0.722537167	1146.170187	1216.712022	1357.418345	1373.374522	1683.390929	1397.034392	937.1560706	883.0201958	768.1150585
6.10_365.1195m/z	365.1195275	6.101316667	neg	4-Amino-2-hydroxylamino-6-nitrotoluene	71191	Unclassified	Unclassified	Unclassified			38	0	2M-H	C7H9N3O3	-5.403412185	1833.431625	2092.050877	1677.922564	1658.699373	1842.602015	1466.40346	1067.264859	1249.633065	1061.743023
6.10_407.2807m/z	407.2806879	6.101316667	neg	24-Nor-5β-chol-22-ene-3α,7α,12α-triol	43158	Unclassified	Unclassified	Unclassified			38	0	M+FA-H	C23H38O3	1.076300817	1964.827339	2495.395054	2241.004437	0.001403199	0.001403199	0.001403199	499.2648446	753.7184178	695.2896865
6.12_479.1910m/z	479.1909502	6.123483333	neg	Ethyl 3,4,5-trimethoxybenzoate	HMDB0038627	Benzenoids	Benzene and substituted derivatives	Benzoic acids and derivatives			38	0	2M-H	C12H16O5	-2.750129909	2716.64386	3005.517606	3233.112839	1189.084608	1623.81109	1396.870819	2467.36765	3208.875646	2971.421785
6.14_297.1473m/z	297.1473053	6.143466667	neg	Olomoucine	45229	Organoheterocyclic compounds	Imidazopyrimidines	Purines and purine derivatives			38	0	M-H	C15H18N6O	1.249399132	1435.019259	85.51862453	5669.775082	46.87380605	0.001403199	52.97189508	89.43269215	160.3514347	698.765305
6.16_459.0939m/z	459.0938936	6.161866667	neg	Adriamycinone	657	Unclassified	Unclassified	Unclassified			38	0	M+FA-H	C21H18O9	1.469700357	0.001403199	13.20439715	36.8079056	3814.913656	3656.880769	4269.044066	0.001403199	13.08874131	14.12761756
6.30_149.0608m/z	149.0608354	6.302033333	neg	3-Methylphenylacetic acid	HMDB0002222	Benzenoids	Benzene and substituted derivatives	Toluenes	*		38	0	M-H	C9H10O2	0.215076994	38.91195837	93.80484524	75.68681816	35.55698648	64.12876691	59.10520127	90.92406765	96.97086753	55.98130912
6.36_427.1976m/z	427.1976339	6.3617	neg	Cinncassiol C3	71837	Lipids and lipid-like molecules	Prenol lipids	Sesquiterpenoids	*		38	0	M+FA-H	C20H30O7	0.726555211	3597.076583	3615.796567	4003.744416	3038.058236	2920.524075	2538.639764	3464.089435	3341.546542	3767.937231
6.44_491.2139m/z	491.2138502	6.442716667	neg	CAY10603	64797	Unclassified	Unclassified	Unclassified			38	0	M+FA-H	C22H30N4O6	-1.988634397	9562.684778	8466.521597	9088.724702	728.0151176	523.9897194	500.9524522	3823.521649	4127.424252	3776.300587
6.48_285.1070m/z	285.1070135	6.477433333	neg	N-Acrylylglycine methyl ester	5743	Unclassified	Unclassified	Unclassified			38	0	2M-H	C6H9NO3	-7.676443311	763.35697	758.609613	1068.506253	1415.337953	1346.279585	1404.039003	940.5885169	1209.733862	1136.232676
6.69_305.1607m/z	305.1607442	6.69475	neg	1-(beta-D-Glucopyranosyloxy)-3-octanone	HMDB0031315	Lipids and lipid-like molecules	Fatty Acyls	Fatty acyl glycosides	*		38	0	M-H	C14H26O7	0.54711935	1461.542438	1903.554953	1445.845146	716.0762624	808.298968	626.6496367	882.7589207	1390.437303	1324.292854
6.78_269.1008m/z	269.1007802	6.776866667	neg	4-Hydroxy-L-threonine	3259	Unclassified	Unclassified	Unclassified			38	0	2M-H	C4H9NO4	6.445936382	1235.192356	1054.565911	1177.636674	1803.196098	1519.842348	1803.386625	1413.388486	1452.547699	1245.485314
6.88_185.1184m/z	185.1183938	6.8759	neg	(±)-2,2,6-Trimethylcyclohexanone	89536	Organic oxygen compounds	Organooxygen compounds	Carbonyl compounds	*		38	0	M+FA-H	C9H16O	0.540666495	531.0526394	629.5881966	394.3552107	843.1365424	934.9175499	902.2280319	826.113083	837.8175007	784.8937237
6.91_427.1608m/z	427.1608031	6.9072	neg	4,8-Diacetyl-T2-tetrol	90520	Lipids and lipid-like molecules	Prenol lipids	Sesquiterpenoids	*		38	0	M+FA-H	C19H26O8	-0.438420193	1239.80467	1455.020753	1556.542625	1736.853567	1932.284292	1519.829009	1392.799235	1947.893897	1680.128706
7.15_321.1822m/z	321.1821771	7.147366667	neg	2H-Indol-2-one, 4-[2-(dipropylamino)ethyl]-1,3-dihydro-7-hydroxy-	2363	Unclassified	Unclassified	Unclassified			38	0	M+FA-H	C16H24N2O2	0.710473378	1590.125303	1633.768072	1480.534034	3453.874501	3233.810589	2880.313931	1196.127766	1849.746983	1553.10983
7.18_378.1019m/z	378.1019322	7.178516667	neg	Chondroitin	5602	Organic oxygen compounds	Organooxygen compounds	Carbohydrates and carbohydrate conjugates	**	C00401	38	0	M-H	C14H21NO11	-5.939835784	6040.034206	7444.520958	7443.235641	9577.526417	10132.46452	10195.84752	7946.049699	11265.3116	9787.623947
7.18_497.1644m/z	497.1644167	7.178516667	neg	Eucaglobulin	HMDB0036742	Lipids and lipid-like molecules	Saccharolipids	Unclassified	*		38	0	M-H	C23H30O12	-4.081411634	8479.376938	10063.52789	9345.124359	9282.728055	10031.435	10183.06699	8538.591683	10789.61806	10000.77952
7.18_519.0583m/z	519.0582948	7.178516667	neg	Norswertianin	68122	Unclassified	Unclassified	Unclassified			38	0	2M-H	C13H8O6	2.682932406	32514.956	35431.90201	32949.70948	29278.8241	31694.31176	28313.01453	21340.57176	25366.57887	23229.37989
7.32_589.2998m/z	589.2997695	7.324733333	neg	Butralin	HMDB0249470	Unclassified	Unclassified	Unclassified			38	0	2M-H	C14H21N3O4	1.073424871	2640.202618	3231.058839	2640.857491	585.339669	0.001403199	147.588916	984.0242534	1005.320143	1131.693646
7.43_392.2085m/z	392.2084737	7.426916667	neg	S-4-benzyl-3-((S)-3-hydroxy-2,2-dimethyloctanoyl)oxazolidin-2-one	65461	Unclassified	Unclassified	Unclassified			38	0	M+FA-H	C20H29NO4	1.763836892	1659.826127	1549.027811	1818.016617	3229.95297	2720.624339	2694.87864	2302.206722	2530.280005	2366.740618
7.44_787.2753m/z	787.2753272	7.440866667	neg	Rotenone	48020	Phenylpropanoids and polyketides	Isoflavonoids	Rotenoids	**	C07593	38	0	2M-H	C23H22O6	-0.854037	32651.89921	30884.82058	37796.59933	4988.900831	5104.092215	4448.497377	15138.48788	17397.95784	14401.41314
7.47_427.1146m/z	427.1146112	7.46525	neg	xi-2,3-Dihydro-2-oxo-1H-indole-3-acetic acid	90795	Organoheterocyclic compounds	Indoles and derivatives	Indolyl carboxylic acids and derivatives			38	0	M+FA-H	C20H18N2O6	-0.204120899	1339.855148	1444.427318	1967.919895	2097.249483	2567.99084	2462.623265	1040.714761	1117.316633	1037.186389
7.54_741.2702m/z	741.2702288	7.536516667	neg	N-Methyl-14-O-demethylepiporphyroxine	86771	Lipids and lipid-like molecules	Glycerolipids	Triradylcglycerols	*		38	0	2M-H	C20H21NO6	5.02564659	2276.309308	2393.165686	3166.070293	0.001403199	0.001403199	0.001403199	715.7668842	765.779541	562.7584186
7.83_565.3022m/z	565.3021505	7.8311	neg	24,25-Diacetylvulgaroside	HMDB0041366	Lipids and lipid-like molecules	Prenol lipids	Sesterterpenoids	*		38	0	M+FA-H	C29H44O8	0.632745163	13539.58889	14149.09187	15560.73746	7440.607889	6987.809631	7747.417985	7088.355441	8375.787861	7616.154562
7.88_410.1597m/z	410.1597108	7.883383333	neg	JWH 250 N-(5-carboxyxypentyl) metabolite	85087	Unclassified	Unclassified	Unclassified			38	0	M+FA-H	C22H23NO4	-3.286970979	1092.620903	1378.098612	1229.661865	1199.904727	1267.492914	1213.738497	557.8049805	748.5845817	529.0392305
8.01_489.2717m/z	489.2716752	8.009683333	neg	1(3)-glyceryl-6-keto-PGF1α	36234	Unclassified	Unclassified	Unclassified			38	0	M+FA-H	C23H40O8	2.597640558	9086.003509	8463.648657	9431.130064	5795.282092	4934.234895	5173.710223	6499.075501	7217.80273	6681.484576
8.02_311.1867m/z	311.1867228	8.01695	neg	tetranor-12(S)-HETE	44857	Unclassified	Unclassified	Unclassified			38	0	M+FA-H	C16H26O3	1.221946034	2714.663368	2343.7555	2400.187183	4326.320922	4010.473326	4016.239223	1755.069379	1904.775981	1613.407831
8.07_745.2025m/z	745.2024789	8.074883333	neg	Deferasirox	85530	Organoheterocyclic compounds	Azoles	Triazoles			38	0	2M-H	C21H15N3O4	-3.69408824	13888.93624	16643.32535	13495.0683	1904.939437	1186.750658	778.9288177	6828.707719	8688.863381	7108.866623

8.40_621.3283m/z	621.3282502	8.40065	neg	OLEANDRIN	44218	Unclassified	Unclassified	Unclassified			38	0	M+FA-H	C32H48O9	0.371761618	3202.496331	3661.216766	3493.359443	3006.168113	3246.430297	3164.0446	2687.041662	2606.659299	3664.009347
8.41_631.2717m/z	631.2716852	8.411166667	neg	PtdIns-(1,2-dioctanoyl) (sodium salt)	44997	Unclassified	Unclassified	Unclassified			38	0	M+FA-H	C25H47O13P	-3.318962367	7143.393715	7558.862239	7444.93925	11843.53998	11986.68743	10685.38559	7267.735028	9173.212859	8994.242009
9.04_491.2866m/z	491.2865739	9.039016667	neg	Dibutyl malate	87872	Organic acids and derivatives	Hydroxy acids and derivatives	Beta hydroxy acids and derivatives	*		38	0	2M-H	C12H22O5	0.818041311	9480.687573	11863.34475	11510.19765	12480.06296	12434.26002	12731.74782	9441.462742	10507.08347	10788.24026
9.07_325.2024m/z	325.2023741	9.065233333	neg	12-hydroxy-5,8,10-heptadecatrienoic acid	74573	Lipids and lipid-like molecules	Fatty Acyls	Fatty acids and conjugates	*	C20388	38	0	M+FA-H	C17H28O3	1.165025691	1927.873996	1680.351399	1909.745859	1811.716033	1953.528588	1699.681522	1917.894146	1074.031615	1420.343721
9.13_405.1064m/z	405.1063542	9.1257	neg	Anibine	68135	Unclassified	Unclassified	Unclassified			38	0	2M-H	C11H9NO3	-7.031714913	1241.371401	1129.6661	1052.938067	965.1953981	892.1530615	892.9556621	1122.003347	944.8359411	1343.535142
9.33_378.1534m/z	378.1533508	9.332316667	neg	2-(Arabinosylamino)-3-(glucosylamino)propanenitrile	HMDB0039505	Organic oxygen compounds	Organooxygen compounds	Carbohydrates and carbohydrate conjugates			38	0	M-H	C14H25N3O9	4.08223748	1508.78031	1489.384039	881.0120573	1570.892864	1878.446629	1831.846181	1095.427283	1293.48695	1942.225488
9.41_467.1902m/z	467.19018	9.414	neg	p-Hydroxyprimidone	2086	Unclassified	Unclassified	Unclassified			38	0	2M-H	C12H14N2O3	-7.322141287	2130.61565	2128.921026	2753.598233	2263.979557	2324.410275	2695.715436	1304.240343	1605.882199	1359.527818
9.53_443.2076m/z	443.2076176	9.52575	neg	Galbanic acid	86766	Lipids and lipid-like molecules	Prenol lipids	Terpene lactones	*		38	0	M+FA-H	C24H30O5	0.227770444	2616.954914	3288.397691	3350.945304	1440.323729	2255.278471	2064.676143	2383.235482	3274.985679	2677.368793
9.75_307.1165m/z	307.1165491	9.75225	neg	Ornaline	65631	Organic acids and derivatives	Carboxylic acids and derivatives	Amino acids, peptides, and analogues	*	C01683	38	0	M+FA-H	C10H18N2O6	7.095717707	8401.276048	8244.727109	7073.046688	7233.818643	7715.332229	8091.40281	9486.891562	8703.813673	8089.609583
0.54_699.4979m/z	699.4978684	0.535283333	neg	PA(18:0/18:2(9Z,12Z))	LMGP10010036	Lipids and lipid-like molecules	Glycerophospholipids	Glycerophosphates	**	C00416	37.9	0	M-H	C39H73O8P	1.197597325	805.3482391	712.4233034	774.6688457	0.001403199	166.4676482	0.001403199	643.4588422	0.001403199	0.001403199
0.69_154.9743m/z	154.9742958	0.692166667	neg	Formylphosphonate	63591	Unclassified	Unclassified	Unclassified			37.9	0	M+FA-H	CH3O4P	-7.295114545	5456.885389	2718.121455	3617.934908	3731.033912	2386.461596	5858.044673	4094.052374	3313.212378	4213.222926
0.71_943.2642m/z	943.2642194	0.714216667	neg	Formononetin 7-O-(6''-acetylglcoside)	47546	Lipids and lipid-like molecules	Polyketides	Flavonoids			37.9	0	2M-H	C24H24O10	-2.539642979	2816.83041	0.001403199	3716.708453	0.001403199	4477.615727	1347.707372	0.001403199	1179.515259	2341.261705
0.80_309.0635m/z	309.0635433	0.800366667	neg	N-Methylethanolaminium phosphate	HMDB0060173	Organic acids and derivatives	Organic phosphoric acids and derivatives	Phosphate esters	**	C01210	37.9	0	2M-H	C3H10NO4P	4.291993378	140893.8452	135141.5623	114950.0625	93541.21756	97880.8164	104134.5421	115567.935	137970.4824	132643.7697
0.86_219.0318m/z	219.0317897	0.86335	neg	3-Fluorocyclohexadiene-cis,cis-1,2-diol-1-carboxylate	71219	Unclassified	Unclassified	Unclassified			37.9	0	M+FA-H	C7H7FO4	4.308941781	4178.147665	5313.120752	5422.933552	3806.62674	523.128894	2238.174889	3776.794309	3456.580031	190.3306428
0.86_247.0936m/z	247.093639	0.86335	neg	Alanylhydroxyproline	HMDB0028688	Organic acids and derivatives	Carboxylic acids and derivatives	Amino acids, peptides, and analogues	*		37.9	0	M+FA-H	C8H14N2O4	0.391860232	1585.746756	1629.305405	1915.169558	1647.978432	520.0521436	1167.86978	1478.472426	1834.566943	656.5961139
1.12_166.0511m/z	166.0511328	1.11925	neg	3-Hydroxyphenylglycine	6616	Unclassified	Unclassified	Unclassified			37.9	0	M-H	C8H9NO3	0.994013283	128.7737267	42.52484704	55.33734445	57.32531997	81.87509445	74.42079208	64.84531874	112.1850273	142.6264888
1.18_200.1041m/z	200.1041353	1.180366667	neg	Dimethylguanidino valeric acid	HMDB0240212	Organic acids and derivatives	Keto acids and derivatives	Short-chain keto acids and derivatives			37.9	0	M-H	C8H15N3O3	0.350108572	844.3795295	830.6924364	768.1203118	957.9789752	1096.771942	936.6625182	916.0641108	865.1418514	677.531975
1.18_210.0442m/z	210.0442354	1.180366667	neg	Ethiin	HMDB0037057	Organic acids and derivatives	Carboxylic acids and derivatives	Amino acids, peptides, and analogues			37.9	0	M+FA-H	C5H11NO3S	0.414059129	1690.425458	1995.259509	1673.349786	2569.902733	2265.993283	2622.739917	1750.188108	2347.282772	2103.619602
1.20_383.1227m/z	383.1227165	1.202333333	neg	Alamarine	67604	Unclassified	Unclassified	Unclassified			37.9	0	M+FA-H	C19H18N2O4	-6.339211759	1027.100631	2206.629823	1877.122385	1644.218602	1265.509177	1602.569424	1100.016269	1816.04781	1316.040551
1.25_265.0930m/z	265.0930409	1.246333333	neg	2,4-Diamino-6,7-dimethoxyquinazoline	2461	Organoheterocyclic compounds	Diazanaphthalenes	Benzodiazines	*		37.9	0	M+FA-H	C10H12N4O2	-5.396028089	471.0237343	691.8309918	502.5848003	1096.14065	634.3539991	853.6537655	508.491196	511.1848164	330.4684029
1.27_178.9658m/z	178.9657796	1.26875	neg	(±)-Sulfobutanedioic acid	HMDB0040585	Lipids and lipid-like molecules	Fatty Acyls	Fatty acids and conjugates	*		37.9	0	M-H2O-H	C4H6O7S	0.996222872	1206.960412	1613.196171	1639.920868	1469.342003	1477.800408	1502.716607	1316.661968	1523.185457	1428.669777
1.29_227.0596m/z	227.0595829	1.290916667	neg	Diisopropyl sulfate	72953	Unclassified	Unclassified	Unclassified			37.9	0	M+FA-H	C6H14O4S	0.549760745	609.3236001	567.9767716	818.7474541	690.9889756	617.9883925	533.3393217	672.3040191	826.5999849	742.1649534
1.41_243.0986m/z	243.0986481	1.40785	neg	Metharbital	85385	Organoheterocyclic compounds	Diazines	Pyrimidines and pyrimidine derivatives			37.9	0	M+FA-H	C9H14N2O3	0.014534286	675.8838992	568.3792042	512.3707783	691.0908731	583.1269874	587.6656528	574.5368728	546.7075672	453.5895117
1.59_187.1090m/z	187.1089593	1.5934	neg	N-alpha-Acetyl-L-lysine	HMDB0000446	Organic acids and derivatives	Carboxylic acids and derivatives	Amino acids, peptides, and analogues	*	C12989	37.9	0	M-H	C8H16N2O3	0.761772404	58.96517009	22.69915427	75.65464298	37.72366548	27.58239322	25.74932678	0.001403199	34.86356354	67.95352223
1.63_148.9915m/z	148.9914545	1.631516667	neg	Thiodiacetic acid	70376	Organic acids and derivatives	Carboxylic acids and derivatives	Dicarboxylic acids and derivatives	**	C14872	37.9	0	M-H	C4H6O4S	0.341902	258.72174	236.2956884	316.0884287	471.2395331	387.2940044	394.9960614	284.5456927	321.751809	289.1563396
1.72_307.0662m/z	307.0662197	1.721216667	neg	Imidazol-5-yl-pyruvate	63492	Unclassified	Unclassified	Unclassified			37.9	0	2M-H	C6H6N2O3	-7.102069746	392.5810099	528.4564472	678.1722532	914.7421041	935.9024249	933.1612065	836.9326728	651.4533482	1065.531507
1.77_231.0988m/z	231.0988203	1.769966667	neg	Aspartyl-Valine	HMDB0028766	Organic acids and derivatives	Carboxylic acids and derivatives	Amino acids, peptides, and analogues	*		37.9	0	M-H	C9H16N2O5	0.754528945	708.2087157	749.0726476	636.8015475	1058.876705	1081.980966	1194.033535	723.5415747	803.6926029	733.5521488
10.55_409.1084m/z	409.1084327	10.54533333	neg	PHENETHICILLIN	44261	Unclassified	Unclassified	Unclassified			37.9	0	M+FA-H	C17H20N2O5S	2.573573875	1122.358864	985.5986709	928.9907175	743.1653348	1422.81468	1127.769269	980.3425223	1546.206601	1033.513148
10.85_540.3313m/z	540.3312689	10.8529	neg	PC(16:0/0:0)	40284	Lipids and lipid-like molecules	Glycerophospholipids	Glycerophosphocholines	**	C04230	37.9	0	M+FA-H	C24H50NO7P	1.16369598	3702.929926	3670.03157	3323.273386	2728.212869	3350.472231	3475.057362	2747.583277	3234.537943	3291.749706
11.09_265.1480m/z	265.1480186	11.08726667	neg	6,7-Dihydro-4-(hydroxymethyl)-2-(p-hydroxyphenethyl)-7-methyl-5H-2-pyrindiniu	89304	Benzenoids	Phenols	1-hydroxy-2-unsubstituted benzenoids			37.9	0	M-H2O-H	C18H22NO2+	2.835601069	128924.0375	84984.07585	199124.6498	83583.05911	88102.14413	77845.71499	126126.053	92480.55387	77062.3279
11.11_271.2281m/z	271.2280563	11.11088333	neg	Heptyl ketone	HMDB0059813	Organic oxygen compounds	Organooxygen compounds	Carbonyl compounds			37.9	0	M+FA-H	C15H30O	0.83051232	1508.082887	2105.323984	1482.760549	1366.387745	1643.596204	1196.524011	2011.131928	1419.49733	1351.99023
11.27_271.2281m/z	271.2280642	11.27416667	neg	7-Ethyltridecan-6-one	LMFA12000255	Unclassified	Unclassified	Unclassified			37.9	0	M+FA-H	C15H30O	0.865251477	2148.328267	2028.69505	1746.353309	1964.813281	1580.938574	1617.41943	2513.664962	1503.363544	2164.870672
11.38_269.2123m/z	269.2122533	11.38441667	neg	2-Pentadecenal	265104	Unclassified	Unclassified	Unclassified			37.9	0	M+FA-H	C15H28O	0.155518791	1732.135931	1718.385492	1980.70034	1595.712818	1799.12027	1570.607132	1958.447452	1704.355448	1362.772628
11.56_299.2594m/z	299.2593884	11.56388333	neg	Heptadecanal	HMDB0031039	Lipids and lipid-like molecules	Fatty Acyls	Fatty aldehydes	*		37.9	0	M+FA-H	C17H34O	0.864718636	3205.541172	3069.873469	2879.78358	3076.484937	2726.106824	3039.576322	3588.019084	3661.081325	3659.169343
12.59_297.2436m/z	297.2435587	12.5935	neg	14-Methyl-8E-hexadecenal	46484	Lipids and lipid-like molecules	Fatty Acyls	Fatty aldehydes			37.9	0	M+FA-H	C17H32O	0.159369744	1610.376461	1923.268911	1739.674795	1366.86344	1746.690754	1090.124066	1860.194837	1621.97005	2035.713286
13.94_439.1253m/z	439.1252943	13.93851667	neg	Dehydroxyzyleuton	85324	Organoheterocyclic compounds	Benzothiophenes	1-benzothiophenes	*		37.9	0	2M-H	C11H12N2OS	-3.401815388	25233.91893	14983.61255	16519.2959	12451.85378	13052.63638	12711.94916	16073.40112	26462.73517	14002.52928
14.70_166.0481n	165.0408134	14.69558333	neg	Apionic acid	35481	Unclassified	Unclassified	Unclassified			37.9	0	M-H, M+FA-H	C5H10O6	2.118570805	255781.7639	6515.279655	5339.037255	73608.72733	10479.00168	8465.619477	4689.604068	6119.143222	4736.766534
2.22_254.0672m/z	254.0671828	2.215433333	neg	Methylsalicyluric acid	5670	Unclassified	Unclassified	Unclassified			37.9	0	M+FA-H	C10H11NO4	0.823057822	1403.212919	1311.86427	1530.259204	794.3577026	720.8247391	712.7806813	341.6871984	500.4210968	471.2942771
2.38_232.0828m/z	232.0828301	2.380333333	neg	N2-Acetyl-L-aminoadipate semialdehyde	63444	Unclassified	Unclassified	Unclassified			37.9	0	M+FA-H	C8H13NO4	0.905054246	469.8907319	489.7310672	485.8880105	890.7120186	940.0586218	664.4980885	497.4313465	705.0236208	565.4779281
2.43_220.9763m/z	220.9762883	2.429083333	neg	4-Sulfolactone	66488	Unclassified	Unclassified	Unclassified			37.9	0	M-H	C6H6O7S	0.63608285	6609.03813	7382.673338	8537.594759	2995.507814	2719.406896	2486.831074	3471.72246	3706.140908	3206.663509
2.74_186.0773m/z	186.0773003	2.743066667	neg	6-Acetamido-3-oxohexanoate	66023	Unclassified	Unclassified	Unclassified			37.9	0	M-H	C8H13NO4	0.635051919	269.4693695	204.0038076	257.7095434	204.0286166	142.1018288	198.5529671	254.3156823	269.7279725	259.4098866
2.85_213.0882m/z	213.0882319	2.852583333	neg	4-PIOL	69651	Unclassified	Unclassified	Unclassified			37.9	0	M+FA-H	C8H12N2O2	0.900794646	670.7171569	625.5398555	747.6436368	851.9490819	838.0947337	863.0447655	767.3359284	691.0935234	659.6205027
2.86_197.0457m/z	197.0457396	2.864816667	neg	4-Hydroxy-3-methylbenzoic acid	58250	Benzenoids	Benzene and substituted derivatives	Benzoic acids and derivatives	*		37.9	0	M+FA-H	C8H8O3	1.266508933	5635.034251	5462.44564	6390.430134	58.21454187	83.72561895	54.18931962	2974.116888	3113.355505	3007.291206
3.60_381.0807m/z	381.0807372	3.601516667	neg	O-Oxalylhomoserine	66891	Unclassified	Unclassified	Unclassified			37.9	0	2M-H	C6H9NO6	5.338166416	1841.612954	2630.43462	2689.161549	1854.100903	1946.567497	2430.333833	2217.791208	2553.923374	2458.440945
3.65_609.1055m/z	609.105519	3.64965	neg	L-Tyrosine, 3-methoxy-a-methyl-, hydrogen sulfate	1220	Unclassified	Unclassified	Unclassified			37.9	0	2M-H	C11H15NO7S	-1.721384667	145.0292027	352.712179	390.0921583	1920.835055	2218.308566	1807.985362	600.8895135	835.0109634	804.1194071
3.75_117.0379m/z	117.0379459	3.747833333	neg	4-(Methylthio)-2-butanone	HMDB0031316	Organic oxygen compounds	Organooxygen compounds	Carbonyl compounds	*		37.9	0	M-H	C5H10OS	-0.115148633	0.001403199	0.001403199	0.001403199	41.7799474	44.22470562	23.74361847	22.50329745	39.05181503	41.00959063
3.77_160.0405m/z	160.0404654	3.770416667	neg	4,8-Dihydroxyquinoline	63549	Organoheterocyclic compounds	Quinolines and derivatives	Quinolones and derivatives	**	C05637	37.9	0	M-H	C9H7NO2	0.393174285	587.7388786	456.8856737	524.0235722	784.2635243	861.7922166	651.2720458	542.2323376	559.9390812	576.8784973
3.78_571.0688m/z	571.0687562	3.779133333	neg	3-carboxy-1-hydroxypropylthiamine diphosphate	58539	Organoheterocyclic compounds	Diazines	Pyrimidines and pyrimidine derivatives	**	C05381	37.9	0	M+FA-H	C16H24N4O10P2S	3.262114456	4003.510354	3224.713378	2972.979809	1655.249509	1428.772633	1496.734371	2245.119828	2686.390066	2073.09762
3.85_403.9920m/z	403.9920113	3.84605	neg	5-Azacytidine 5'-diphosphate	1181	Unclassified	Unclassified	Unclassified			37.9	0	M-H	C8H13N3O12P2	4.547312719	12133.71323	12580.72993	12932.67593	12814.91173	14919.14294	14160.13146	16009.66	14367.29444	15829.30006
3.87_321.1071m/z	321.1070864	3.868533333	neg	5-Amino-6-ribitylamino uracil	61974	Organic oxygen compounds	Organooxygen compounds	Carbohydrates and carbohydrate conjugates	**	C04732	37.9	0	M+FA-H	C9H16N4O6	6.878951575	810.7531082	989.2671778	864.7666712	3132.812516	2968.94249	2563.992529	1887.868682	1867.384603	2028.310735
3.87_400.0555n	399.0481776	3.868533333	neg	Bis(glycerophospho)-glycerol	66064	Unclassified	Unclassified	Unclassified			37.9	0	M-H, M+FA-H	C9H22O13P2	4.724445519	2864.384955	3897.593278	3447.928351	4813.375781	4359.140177	4247.811673	3130.38342	3205.499594	3665.71513
3.89_200.0930m/z	200.0929797	3.8906	neg	SCOPOLINE	43998	Unclassified	Unclassified	Unclassified			37.9	0	M+FA-H	C8H13NO2	0.955130737	1771.394585	1697.19389	1761.163259	1664.861517	1645.129633	1457.878973	1872.461824	1564.363945	1724.093592
3.89_232.0650m/z	232.065036	3.8906	neg	N-butyryl-L-Homocysteine thiolactone	45311	Unclassified	Unclassified	Unclassified			37.9	0	M+FA-H	C8H13NO2S	0.713269383	238.9576906	274.7276996	263.0846219	739.0052531	687.1178211	730.2506431	735.5077682	999.3421216	967.1544219
3.89_336.0518n	335.0445297	3.8906	neg	4-Hydroxy-5-(dihydroxyphenyl)-valeric acid-O-methyl-O-sulphate	HMDB0059977	Benzenoids	Phenols	Benzenediols	*		37.9	0	M-H, M+FA-H	C12H16O9S	0.901745408	2742.251306	3089.973754	2954.134879	2999.298908	2376.266834	2173.231486	2737.140438	3522.845533	3272.366636
3.98_117.0379m/z	117.0379316	3.97985	neg	S-Methyl butanethioate	87482	Lipids and lipid-like molecules	Fatty Acyls	Fatty acyl thioesters	*		37.9	0	M-H	C5H10OS	-0.236293323	0.001403199	0.001403199	0.001403199	58.42534743	29.50328739	46.46035676	93.25018245	85.35371971	73.03648825
3.98_214.0254m/z	214.0254102	3.97985	neg	AG-126	45522	Unclassified	Unclassified	Unclassified			37.9	0	M-H	C10H5N3O3	-1.880752517	559.8277691	669.8908573	408.9439567	779.4133939	623.7521705	703.9596365	662.7230748	666.0165851	674.3444377
4.00_196.0617m/z	196.0617069	4.002233333	neg	p-Acetamidophenol (Acetaminophen, Tylenol)	487	Unclassified	Unclassified	Unclassified			37.9	0	M+FA-H	C8H9NO2	1.161773723	407.3476145	272.4700968	384.4319452	219.5397375	242.1987242	307.1378981	373.2463436	419.0667887	411.0314505
4.05_247.0337m/z	247.0337433	4.046666667	neg	Fludioxonil	72268	Unclassified	Unclassified	Unclassified			37.9	0.0981	M-H	C12H6F2N2O2	5.184023897	8350.148433	9262.17345	9364.116037	14538.41628	14226.45906	13754.11679	10559.83167	11529.20681	10537.30818
4.05_393.1335m/z	393.1334971	4.046666667	neg	DL-Dopa	64960	Organic acids and derivatives	Carboxylic acids and derivatives	Amino acids, peptides, and analogues	**	C00355	37.9	0	2M-H	C9H11NO4	8.012099009	556.1078815	977.9485994	701.0305441	881.6097831	675.3746185	720.5655595	571.9624064	738.2033777	712.2667637
4.09_298.1409m/z	298.1409092	4.091783333	neg	PC(O-2:0/O-1:0)	40239	Unclassified	Unclassified	Unclassified			37.9	0	M-H	C11H26NO6P	-5.310071337	254.6343558	295.5923154	244.7525863	218.8667276	286.673148	289.2499881	267.0735181	329.9219646	313.9671176
4.09_389.1094m/z	389.1093661	4.091783333	neg	Domesticoside	HMDB0029647	Organic oxygen compounds	Organooxygen compounds	Carbohydrates and carbohydrate conjugates	*		37.9	0	M+FA-H	C15H20O9	1.252586772	1242.057016	1166.490732	1317.371725	268.6814512	186.3731745	0.001403199	553.1079761	865.853404	723.4350854
4.11_319.0495m/z	319.0494716	4.113883333	neg	4-Hydroxy-5-(phenyl)-valeric acid-O-sulphate	HMDB0059981	Benzenoids	Benzene and substituted derivatives	Unclassified	*		37.9	0	M+FA-H	C11H14O6S	0.582258774	739.7840845	762.0727228	863.3875473	668.6125586	527.3111422	504.5044359	1050.783875	1474.289429	1405.287608
4.11_447.1877m/z	447.1877067	4.113883333	neg	D-Linalool 3-(6''-malonylglucoside)	95199	Lipids and lipid-like molecules	Fatty Acyls	Fatty acyl glycosides	*		37.9	0	M+FA-H	C19H30O9	1.296218853	2354.440645	3045.893435	2596.181988	5564.167113	4961.125399	4968.870578	3065.755859	3134.17845	3116.954358
4.14_205.1083m/z	205.1083289	4.136366667	neg	4-Hydroxyvalproic acid	LMFA01050496	Lipids and lipid-like molecules	Fatty Acyls	Fatty acids and conjugates	**	C16649	37.9	0	M+FA-H	C8H16O3	1.134621006	210.5799217	206.9730826	257.9187963	175.5206559	230.8041664	184.6151456	294.2394289	288.2489814	185.9289966
4.14_258.1347m/z	258.1347471	4.136366667	neg	2-nonenoylglycine	HMDB0094807	Organic acids and derivatives	Carboxylic acids and derivatives	Amino acids, peptides, and analogues			37.9	0	M+FA-H	C11H19NO3	0.238180751	165.9851993	221.4662327	326.9501414	276.900893	218.6550418	266.6946875	249.5308646	319.3417287	301.1283984
4.32_279.0851m/z	279.0851239	4.3224	neg	Aspartyl-Threonine	85677	Organic acids and derivatives	Carboxylic acids and derivatives	Amino acids, peptides, and analogues	*		37.9	0	M+FA-H	C8H14N2O6	7.411303505	903.1979739	1100.153482	1061.097184	1719.436784	1358.936725	1625.211682	1220.539339	1038.391969	1070.949519
4.37_452.9983m/z	452.9983071	4.367166667	neg	Nitroglycerin	HMDB0014865	Organic oxygen compounds	Organic oxoanionic compounds	Organic nitrates		C07455	37.9	0	2M-H	C3H5N3O9	0.938386198	3747.445757	3537.173629	3431.175748	6774.579341	3758.072305	4517.395933	2557.239809	3048.158069	3518.49833
4.39_675.1424m/z	675.142417	4.3893	neg	CMP-N-glycoloylneuraminate	62854	Nucleosides, nucleotides, and analogues	Pyrimidine nucleotides	Pyrimidine ribonucleotides	**	C03691	37.9	0	M+FA-H	C20H31N4O17P	3.224528739	7264.410544	6712.185806	7615.396516	5031.463868	4341.195478	4791.469101	9115.392168	7051.431385	7676.381019
4.41_132.0453m/z	132.0453236	4.41115	neg	2-Methyl-4,5-benzoxazole	HMDB0032390	Organoheterocyclic compounds	Benzoxazoles	Unclassified	*		37.9	0	M-H	C8H7NO	-1.231008497	1809.747105	1948.502809	1982.178387	406.849337	295.0889847	431.6754061	1354.183892	1688.653368	1643.898838
4.48_230.9970m/z	230.9969704	4.477166667	neg	4-Sulfobenzaldehyde	66490	Unclassified	Unclassified	Unclassified			37.9	0	M+FA-H	C7H6O4S	0.472281812	1539.092426	1864.520286	1987.681442	2160.824507	2251.508189	2420.201706	2244.064571	2508.668983	2423.95945
4.63_455.1562m/z	455.15621	4.632666667	neg	Butyl 3-O-caffeoylquinate	90051	Organic oxygen compounds	Organooxygen compounds	Alcohols and polyols			37.9	0	M+FA-H	C20H26O9	0.791738053	933.9317479	848.0763952	1054.604672	818.7474977	716.5500391	646.208021	899.0504832	808.1922188	674.9386374
4.66_435.1411m/z	435.1411285	4.660483333	neg	Pyrene-4,5-oxide	72098	Unclassified	Unclassified	Unclassified			37.9	0	2M-H	C16H10O	4.757795579	765.444793	881.148646	1104.037294	844.3993279	542.2633438	989.5074141	630.2786875	709.5314026	307.8010696
4.75_158.1308n	157.1235046	4.747466667	neg	Methyl caprylate	87561	Lipids and lipid-like molecules	Fatty Acyls	Fatty acid esters	*		37.9	0	M-H2O-H, M-H	C9H18O2	0.515078172	360.4477166	360.3740345	364.7417567	305.711697	255.8360626	371.4720646	263.8854995	341.6260327	196.0746222
4.77_493.1720m/z	493.1719568	4.769883333	neg	Dichotosin	LMPK12020272	Lipids and lipid-like molecules	Polyketides	Flavonoids			37.9	0	M+FA-H	C23H28O9	0.94040771	6543.17339	6394.288925	6398.130053	93.79549832	146.6152097	0.001403199	2673.859313	3351.751671	2706.715287
4.81_280.0826m/z	280.0825713	4.814166667	neg	4-Hydroxyphenylacetylglutamine	58355	Unclassified	Unclassified	Unclassified			37.9	0	M-H	C13H15NO6	-0.318194163	445.8028392	499.0096179	492.356721	401.5218087	360.5964766	432.0859806	430.4588672	699.850562	497.8568036
4.88_494.2199m/z	494.2199032	4.880083333	neg	ZM 447439	45566	Unclassified	Unclassified	Unclassified			37.9	0	M-H2O-H	C29H31N5O4	0.272433847	1767.732777	2181.794078	1730.342236	1708.167284	1584.300017	1715.767516	1921.609987	2027.775566	2040.032064
4.90_305.1011m/z	305.1010929	4.902316667	neg	1,4-Diamino-2-nitrobenzene	73088	Unclassified	Unclassified	Unclassified			37.9	0	2M-H	C6H7N3O2	2.340248333	904.762281	1000.453432	1032.472963	1115.678008	1368.018305	1245.436957	859.0045802	952.9403121	1195.268314
4.97_389.1268m/z	389.126816	4.969683333	neg	9H-Carbazole-3-carboxaldehyde	86461	Organoheterocyclic compounds	Indoles and derivatives	Carbazoles	*		37.9	0	2M-H	C13H9NO	-7.011303591	674.5990372	868.6091536	897.967025	90.02778766	126.279688	0.001403199	1157.813394	1084.702493	883.2278201
4.99_423.0869m/z	423.0868997	4.99225	neg	Urolithin B	HMDB0013696	Phenylpropanoids and polyketides	Coumarins and derivatives	Unclassified	*		37.9	0	2M-H	C13H8O3	-1.20758158	2819.863029	3373.974157	3394.050811	2890.599258	2865.89691	3075.038518	3118.255539	2970.793276	2790.651651
5.01_403.1105m/z	403.1104826	5.014716667	neg	Sulfinpyrazone	521	Benzenoids	Benzene and substituted derivatives	Phenyl sulfoxides		C07317	37.9	0	M-H	C23H20N2O3S	-4.21769231	1825.57327	2074.551295	2309.731596	4023.566756	3767.853693	4652.323957	1687.335815	1625.899983	1849.220155
5.04_226.9663m/z	226.9663267	5.037216667	neg	5-Sulfo-1,3-benzenedicarboxylic acid	88744	Benzenoids	Benzene and substituted derivatives	Benzenesulfonic acids and derivatives			37.9	0	M-H2O-H	C8H6O7S	3.025955616	451.1412657	379.2317609	431.6549072	449.0530563	628.2722103	556.242857	463.6667761	577.9580869	473.4028037
5.06_307.1166m/z	307.1165857	5.05975	neg	2,6-DIHYDROXY-4-METHOXYTOLUENE	44513	Unclassified	Unclassified	Unclassified			37.9	0	2M-H	C8H10O3	-6.900457422	3276.912834	3364.145371	3465.295131	4260.792791	4636.340886	4657.480987	3056.191112	3082.297094	3896.849141
5.08_324.1263m/z	324.1263072	5.080566667	neg	Nornantenine	86830	Alkaloids and derivatives	Aporphines	Unclassified	*		37.9	0	M-H	C19H19NO4	6.691311768	270.9416142	267.2882052	249.9545575	242.8913585	66.00986906	280.7738717	203.4490142	154.2395134	78.44488852
5.08_478.1692n	459.1513783	5.080566667	neg	Gentiobiosyl 2-methyl-6-oxo-2E,4E-heptadienoate	88215	Organic oxygen compounds	Organooxygen compounds	Carbohydrates and carbohydrate conjugates	*		37.9	0	M-H2O-H, M-H	C20H30O13	1.209642597	6671.015547	6660.169557	6519.821935	12998.53664	12505.46145	12827.2766	14277.18691	12026.97997	14493.4026
5.10_491.0839m/z	491.083862	5.1031	neg	Genistein 4'-O-glucuronide	96095	Phenylpropanoids and polyketides	Isoflavonoids	Isoflavonoid O-glycosides	*		37.9	0	M+FA-H	C21H18O11	1.676328032	132.2425489	109.6392047	223.2819078	907.0167582	299.3985419	1053.729906	233.2569094	278.5640335	101.8578195
5.13_263.0538m/z	263.053795	5.125666667	neg	Diammonium hydrogen phosphate ((NH4)2HPO4)	90213	Unclassified	Unclassified	Unclassified			37.9	0	2M-H	H9N2O4P	4.107488939	1476.224225	1293.493226	1189.808724	664.8300147	460.1087312	663.8956619	1570.439878	1175.114337	1402.069799
5.18_196.0075m/z	196.0075406	5.1777	neg	1,2-Benzisothiazol-3(2H)-one	HMDB0034413	Organoheterocyclic compounds	Benzothiazoles	Unclassified			37.9	0	M+FA-H	C7H5NOS	1.013033647	359.3335361	428.0952823	460.8305161	827.997921	664.6553508	713.1748081	740.9296614	775.515336	707.3892497
5.18_219.0640m/z	219.0639612	5.1777	neg	Aspartyl-Serine	85676	Organic acids and derivatives	Carboxylic acids and derivatives	Amino acids, peptides, and analogues	*		37.9	0	M-H	C7H12N2O6	7.731882591	3301.987066	3883.434688	3782.976526	9119.560186	9835.082765	10047.45193	4485.437545	4438.546087	4599.328036
5.20_235.1011m/z	235.1011132	5.20005	neg	(+/-)-Isobutyl 3-methylthiobutyrate	LMFA07010696	Lipids and lipid-like molecules	Fatty Acyls	Fatty acid esters	*		37.9	0	M+FA-H	C9H18O2S	0.839258212	1200.058077	1276.551795	1315.460627	898.5257615	771.5215356	709.063411	1195.260766	1170.030289	1210.89094
5.20_280.1192m/z	280.1191805	5.20005	neg	Lophophorine	67815	Unclassified	Unclassified	Unclassified			37.9	0	M+FA-H	C13H17NO3	0.570692833	1129.969071	1316.783698	1013.221103	1488.55031	1524.697049	1678.465406	1800.444015	1607.371859	2017.883643
5.22_767.0962m/z	767.0961737	5.222166667	neg	4'-Methylepicatechin 3'-sulfate	HMDB0240466	Phenylpropanoids and polyketides	Flavonoids	Flavans			37.9	0	2M-H	C16H16O9S	0.57798448	12177.29719	10760.84284	9778.4706	10181.65202	10467.31329	9801.636432	15403.98288	14198.26546	14345.14829
5.27_815.2317m/z	815.2317189	5.271716667	neg	3,6-Dimethoxy-6'',6''-dimethyl-3',4'-methylenedioxypyrano[2,3:7,8]flavone	LMPK12111591	Unclassified	Unclassified	Unclassified			37.9	0	2M-H	C23H20O7	-3.443413961	3805.147416	2287.583611	1545.194661	1112.028322	1392.81953	1860.855911	3097.966644	2311.675892	3649.361186
5.29_198.1136m/z	198.1136253	5.28555	neg	5-Butyl-2-ethyloxazole	92668	Organoheterocyclic compounds	Azoles	Oxazoles			37.9	0	M+FA-H	C9H15NO	0.380876711	276.9924823	129.1430819	190.3648296	224.2841095	173.6237156	240.4962465	172.0782911	254.5709448	229.1603762
5.34_385.1869m/z	385.1868986	5.3394	neg	2,3-Dinor-6,15-diketo-13,14-dihydro-PGF1a	2561	Unclassified	Unclassified	Unclassified			37.9	0	M+FA-H	C18H28O6	0.314739732	5165.476969	5327.723554	6242.656169	1449.352102	1671.704799	1393.422915	4043.159141	3775.135712	5832.536193
5.38_813.0506m/z	813.0505684	5.384216667	neg	O-Desmethyloxotolrestat sulfate	2884	Unclassified	Unclassified	Unclassified			37.9	0	2M-H	C15H12F3NO7S	0.651079968	4999.265391	4080.75174	3950.176742	9488.242302	7882.902743	6726.908784	6034.970201	5167.435096	6219.525722
5.43_210.0869n	209.0795938	5.428733333	neg	N4-Phosphoagmatine	65821	Unclassified	Unclassified	Unclassified			37.9	0	M-H, 2M-H, M+FA-H	C5H15N4O3P	-6.220227331	108606.9666	121942.8558	119479.067	83222.73489	78153.38427	81042.86312	99199.50268	109221.6903	88041.75597
5.49_421.1871m/z	421.1870992	5.489016667	neg	Zalcitabine	3061	Nucleosides, nucleotides, and analogues	Pyrimidine nucleosides	Pyrimidine 2',3'-dideoxyribonucleosides	**	C07207	37.9	0	2M-H	C9H13N3O3	7.089344634	1580.617787	1399.843921	1226.36169	1242.642192	1636.07219	1360.245302	1678.403295	1309.286448	1575.303567
5.55_261.0876m/z	261.087554	5.547916667	neg	L-1,2,3,4-Tetrahydro-beta-carboline-3-carboxylic acid	HMDB0035665	Organoheterocyclic compounds	Indoles and derivatives	Pyridoindoles			37.9	0	M+FA-H	C12H12N2O2	-2.436496982	1325.841938	1157.862306	1389.802007	1079.433436	1371.550286	980.9373709	1108.41034	1213.814689	1112.558882
5.57_421.1144m/z	421.1143662	5.57225	neg	5,3'-Dihydroxy-6,7,4',5'-tetramethoxyflavanone	LMPK12140638	Unclassified	Unclassified	Unclassified			37.9	0	M+FA-H	C19H20O8	0.919347772	4203.873702	3524.330717	4150.21515	1159.07191	940.8938568	1402.021462	2799.568877	3442.724838	2574.449783
5.60_317.1510m/z	317.1509658	5.602	neg	4-(3-Methylbut-2-enyl)-L-tryptophan	66155	Unclassified	Unclassified	Unclassified			37.9	0	M+FA-H	C16H20N2O2	1.047379889	1430.448313	1156.463897	1258.74201	1329.986067	1224.357418	1273.371074	1201.38033	1133.859592	1092.017374
5.83_451.0708m/z	451.0708102	5.825	neg	(Z)-Resveratrol 3-glucoside 5-sulfate	HMDB0037074	Phenylpropanoids and polyketides	Stilbenes	Stilbene glycosides	*		37.9	0.499	M-H2O-H	C20H22O11S	0.784536318	28574.87472	30985.59708	33778.15354	35654.43732	31427.93366	33933.18396	1894.392579	2468.463958	2434.709285
5.85_409.1498m/z	409.149819	5.84575	neg	Gibberellin A55	HMDB0033420	Lipids and lipid-like molecules	Prenol lipids	Diterpenoids	*		37.9	0	M+FA-H	C19H24O7	-1.611839593	8815.499712	9652.860842	10552.28812	9163.786806	9752.832411	9648.176814	7738.947317	8195.127018	8445.258616
5.88_408.2879n	407.2805832	5.88025	neg	3a,4b,12a-Trihydroxy-5b-cholanoic acid	5319	Lipids and lipid-like molecules	Steroids and steroid derivatives	Bile acids, alcohols and derivatives	*		37.9	0	M-H, M+FA-H	C24H40O5	0.698671624	3218.988364	3905.316394	4185.982254	196.6450855	261.8834763	231.8759474	724.6018984	681.5885448	1090.347462
5.89_299.1865m/z	299.1864682	5.894166667	neg	(3beta,9beta)-7-Drimene-3,11,12-triol	91231	Organic oxygen compounds	Organooxygen compounds	Alcohols and polyols	*		37.9	0	M+FA-H	C15H26O3	0.278021624	710.232788	708.2890043	796.6011924	625.0768832	724.2047221	752.4505964	245.9431766	460.1169438	623.4108316
6.06_354.1926m/z	354.1925884	6.056116667	neg	2-{2-[4[(2-Hydroxy-3-isopropylaminopropoxy)-benzyloxy]ethoxy}-propionic acid	1357	Unclassified	Unclassified	Unclassified			37.9	0	M-H	C18H29NO6	1.061959261	1698.39834	1466.481359	1488.429094	3679.250914	3186.522159	3208.92166	2343.456239	2131.799534	2118.392773
6.10_455.1903m/z	455.1902533	6.101316667	neg	Tafluprost free acid	HMDB0014256	Lipids and lipid-like molecules	Fatty Acyls	Eicosanoids	*		37.9	0	M+FA-H	C22H28F2O5	3.827592145	14270.73994	15962.40812	15420.47099	13064.24715	14197.25778	13112.95484	14394.26287	15721.05507	16888.88609
6.13_465.1745m/z	465.1744569	6.133983333	neg	Hirsutin	HMDB0035923	Organosulfur compounds	Sulfoxides	Unclassified			37.9	0	2M-H	C10H19NOS2	0.25927812	3330.04502	3356.076217	3492.616107	1399.816983	1544.206908	1629.371282	2427.011716	2887.195251	2550.015373
6.16_343.2128m/z	343.212757	6.161866667	neg	3-Methylbutyl 3-oxobutanoate	HMDB0036396	Organic acids and derivatives	Keto acids and derivatives	Beta-keto acids and derivatives			37.9	0	2M-H	C9H16O3	0.420351061	955.1930346	1015.824833	839.3689857	969.7086748	1060.675339	1119.794393	641.1616086	628.9517876	572.8751119
6.17_366.1019m/z	366.1018985	6.171316667	neg	Ritodrine sulfate	2359	Unclassified	Unclassified	Unclassified			37.9	0	M-H	C17H21NO6S	0.589822267	7826.083146	8020.866089	9386.96248	14640.90646	13503.73878	14356.17048	14521.24119	16211.07114	15916.53688
6.33_325.2023m/z	325.2022904	6.331216667	neg	Fauronyl acetate	91558	Lipids and lipid-like molecules	Prenol lipids	Sesquiterpenoids	*		37.9	0	M+FA-H	C17H28O3	0.866409148	1044.934968	1591.119594	1181.602896	1349.590803	1262.270254	1045.540003	1145.516867	867.3742948	1335.543494

6.34_456.2430m/z	456.2429654	6.339983333	neg	N-arachidonoyl taurine	LMFA08020075	Lipids and lipid-like molecules	Fatty Acyls	Fatty amides			37.9	0	M+FA-H	C22H37NO4S	1.052451248	4424.473007	5446.220507	5323.743508	5731.641884	6656.921631	5546.279434	4804.634358	4770.98557	5541.41975
6.34_595.2356m/z	595.235632	6.339983333	neg	Enterolactone	58357	Lignans, neolignans and related compounds	Furanoid lignans	Tetrahydrofuran lignans	*	C18165	37.9	0	2M-H	C18H18O4	3.170358523	2217.048092	2790.664873	2086.128744	1657.608779	1699.788116	1732.803986	2646.046831	2540.901441	2385.359764
6.37_623.0998m/z	623.099832	6.373833333	neg	XAV939	45564	Unclassified	Unclassified	Unclassified			37.9	0	2M-H	C14H11F3N2OS	-2.770143848	5591.958229	6812.92615	6240.274006	5799.962966	5189.632124	4488.584868	4553.40946	4549.772067	5231.979978
6.43_329.1757m/z	329.1757111	6.433933333	neg	Boldione	HMDB0003422	Lipids and lipid-like molecules	Steroids and steroid derivatives	Androstane steroids	**	C20144	37.9	0	M+FA-H	C19H24O2	-0.428611796	3007.174099	3005.418925	2691.194191	2743.025762	2225.218746	2775.755507	4166.19582	3233.526221	3507.99549
6.43_382.1288m/z	382.128764	6.433933333	neg	5-Methoxynoracronycine	HMDB0029332	Organoheterocyclic compounds	Quinolines and derivatives	Benzoquinolines	*		37.9	0	M+FA-H	C20H19NO4	-2.512144419	2831.027424	2970.977064	2829.68369	4986.533802	4916.172805	4878.809047	3071.656885	3368.955744	3153.484084
6.46_429.2132m/z	429.2132066	6.458366667	neg	Acuminoside	HMDB0029347	Lipids and lipid-like molecules	Prenol lipids	Terpene glycosides	*		37.9	0	M-H2O-H	C21H36O10	0.446927051	8817.612146	9761.369099	10068.92607	8577.014825	7715.054167	8193.230642	9512.294976	9685.41924	10812.67188
6.49_315.0830m/z	315.0830198	6.494283333	neg	(S)(+)-Allantoin	63392	Unclassified	Unclassified	Unclassified			37.9	0	2M-H	C4H6N4O3	7.327413886	998.8829382	901.0843086	1374.008077	1932.839675	2002.223785	2012.802612	899.9914601	989.2702465	1026.654296
6.51_325.2023m/z	325.2023394	6.509966667	neg	Dihydropanaxacol	88614	Lipids and lipid-like molecules	Fatty Acyls	Fatty alcohols	*		37.9	0	M+FA-H	C17H28O3	1.041183692	1319.98486	1647.257268	1716.926708	1420.873393	1302.746709	1453.242363	1241.783863	1510.643187	1459.769714
6.57_293.0543m/z	293.0543103	6.570233333	neg	Pyridoxamine-5'-Phosphate	236	Unclassified	Unclassified	Unclassified			37.9	0	M+FA-H	C8H13N2O5P	-0.405861055	695.7561714	815.705042	857.008638	707.3262948	626.872375	689.0083133	371.0659994	667.8799026	336.3944261
6.59_377.1972m/z	377.1971926	6.592933333	neg	7'-Carboxy-gamma-tocotrienol	HMDB0012851	Organoheterocyclic compounds	Benzopyrans	1-benzopyrans	*		37.9	0	M+FA-H	C20H28O4	0.693303396	1888.466713	1856.275642	1814.914102	1608.411169	1438.384669	1711.830439	1220.732281	1894.483635	1386.791747
6.60_408.2879n	407.2806071	6.6024	neg	omega-muricholic acid	LMST04010065	Lipids and lipid-like molecules	Steroids and steroid derivatives	Bile acids, alcohols and derivatives	*	C17727	37.9	0	M-H, M+FA-H	C24H40O5	0.757077878	4668.465997	5316.488039	4956.184052	52.33588376	0.001403199	0.001403199	1280.085307	1865.909438	2022.102879
6.85_525.1935m/z	525.1935212	6.851566667	neg	(-)-Neplanocin A	96433	Unclassified	Unclassified	Unclassified			37.9	0	2M-H	C11H13N5O3	-5.475080969	14043.30099	13731.85827	12126.61528	15968.04346	16261.33679	15552.18879	16419.16012	15408.88217	14254.47825
6.88_479.1771m/z	479.177092	6.884583333	neg	11H-Benz[bc]aceanthrylene	73041	Unclassified	Unclassified	Unclassified			37.9	0	2M-H	C19H12	-7.147965109	27184.55781	30512.341	25849.85523	753.4451785	514.7105377	452.3092255	22299.78444	26984.07786	24067.49706
6.94_187.1341m/z	187.1340525	6.939266667	neg	2-Nonanone	87538	Organic oxygen compounds	Organooxygen compounds	Carbonyl compounds	*		37.9	0	M+FA-H	C9H18O	0.593914771	961.2802266	925.5015553	871.8926518	1059.603243	874.5493087	969.6788747	1093.160234	948.5141448	1119.866109
6.97_411.1662m/z	411.1662235	6.970716667	neg	4-Acetyl-2-prenylphenol glucoside	HMDB0038282	Organic oxygen compounds	Organooxygen compounds	Carbohydrates and carbohydrate conjugates			37.9	0	M+FA-H	C19H26O7	0.457442079	1798.085685	1622.667478	1609.440374	1715.947802	1597.456737	1380.462111	990.8044123	1061.301576	1189.923465
6.98_155.1079m/z	155.1078738	6.981183333	neg	Hept-trans-2-en-1-yl acetate	HMDB0032307	Organic acids and derivatives	Carboxylic acids and derivatives	Carboxylic acid derivatives	*		37.9	0	M-H	C9H16O2	0.771724235	347.5978688	352.7124378	375.9165139	534.5231631	427.4083727	499.0994094	282.5013722	353.072209	356.5957789
7.06_561.1252m/z	561.1251608	7.055233333	neg	1,3-Dicaffeoylquinic acid	HMDB0029279	Organic oxygen compounds	Organooxygen compounds	Alcohols and polyols	***		37.9	0	M+FA-H	C25H24O12	0.352156325	2618.889703	2812.114642	3126.362546	677.4336194	976.1489131	699.9766254	151.3335399	88.74676451	0.001403199
7.08_387.1664m/z	387.1663947	7.076316667	neg	W140	64665	Unclassified	Unclassified	Unclassified			37.9	0	M+FA-H	C16H27N2O4P	-7.75088162	16804.72363	16670.30486	16529.47132	28467.99669	30178.71286	26664.41442	14606.23274	15774.68449	15250.0704
7.09_425.0989m/z	425.0988523	7.086816667	neg	S-(N-Hydroxy-N-methylcarbamoyl)glutathione	66211	Unclassified	Unclassified	Unclassified			37.9	0	M+FA-H	C12H20N4O8S	1.22281439	3113.462958	3134.962824	2904.223928	3943.124838	3742.694725	3918.16897	2460.070258	2366.234983	2366.771409
7.14_251.0902m/z	251.0901898	7.138633333	neg	Cycasin	65593	Lipids and lipid-like molecules	Fatty Acyls	Fatty acyl glycosides			37.9	0	M-H	C8H16N2O7	6.804612133	2323.548086	2584.786796	2431.977245	3133.65801	3656.495713	2863.144257	2238.04634	2784.311239	2472.926473
7.28_479.1524m/z	479.1524283	7.2779	neg	3',4'-Dihydroxychalcone	88212	Phenylpropanoids and polyketides	Linear 1,3-diarylpropanoids	Chalcones and dihydrochalcones	*		37.9	0	2M-H	C15H12O3	5.032172616	975.3104604	1353.072023	1267.569826	1744.059998	1587.345121	1509.020471	1601.070126	1495.089549	1437.736651
7.39_407.2807m/z	407.2806671	7.3938	neg	24-Nor-5beta-chol-22-ene-3alpha,7alpha,12alpha-triol	LMST04060012	Unclassified	Unclassified	Unclassified			37.9	0	M+FA-H	C23H38O3	1.019030031	1532.309505	1770.665943	1883.91093	412.7852857	378.1817291	484.3448643	1193.605473	1444.800613	1537.343655
7.43_365.1697m/z	365.1696913	7.426916667	neg	Levonordefrin	43228	Benzenoids	Benzene and substituted derivatives	Phenylpropanes		C11768	37.9	0	2M-H	C9H13NO3	-5.786182023	1806.97423	1584.797548	1963.923096	5077.012743	4369.565444	4243.32011	1725.183273	1820.848034	1702.060654
7.44_187.1341m/z	187.1340941	7.440866667	neg	(Z)-3-Nonen-1-ol	HMDB0030959	Lipids and lipid-like molecules	Fatty Acyls	Fatty alcohols	*		37.9	0	M+FA-H	C9H18O	0.886695332	504.6407948	542.9708527	475.2035739	513.6272867	623.3868746	502.1038745	570.7006599	482.6169651	574.3400846
7.51_481.1308m/z	481.130767	7.50615	neg	Fluconazole glucuronide	2709	Unclassified	Unclassified	Unclassified			37.9	0	M-H	C19H20F2N6O7	3.920043805	30384.20357	31464.25387	32696.2455	37039.91236	40566.90377	34093.28967	29543.06831	34120.47339	35148.83939
7.65_297.1708m/z	297.1708484	7.6458	neg	(S)-alpha-Terpinyl glucoside	86550	Organic oxygen compounds	Organooxygen compounds	Carbohydrates and carbohydrate conjugates	*		37.9	0	M-H2O-H	C16H28O6	0.319098307	2444.446079	2761.707292	2677.883906	3018.417654	2515.992876	2669.293284	1807.495602	2218.275523	2234.021888
7.65_528.2641m/z	528.2640564	7.6458	neg	N-[(3a,5b,7b)-7-hydroxy-24-oxo-3-(sulfooxy)cholan-24-yl]-Glycine	6670	Lipids and lipid-like molecules	Steroids and steroid derivatives	Bile acids, alcohols and derivatives	*		37.9	0	M-H	C26H43NO8S	0.745372893	35120.65755	40282.40358	41145.78255	15660.65197	14784.64505	15853.70599	39996.44873	42500.34902	43407.81831
7.68_387.1665m/z	387.166483	7.67705	neg	Methiuron	72828	Unclassified	Unclassified	Unclassified			37.9	0	2M-H	C10H14N2S	-4.584187403	4380.463119	4668.28535	4761.299872	5948.071289	6659.35128	6226.183432	3426.938071	3651.535442	4012.702785
7.98_475.2680m/z	475.2680391	7.984516667	neg	15-keto Latanoprost	45712	Unclassified	Unclassified	Unclassified			37.9	0	M+FA-H	C26H38O5	-4.852901937	12206.82044	11088.77625	14109.93355	1759.108823	1750.856462	1929.586696	4473.474394	4929.494291	6019.325947
8.24_187.1340m/z	187.1340474	8.244766667	neg	Nonan-3-one	LMFA12000053	Organic oxygen compounds	Organooxygen compounds	Carbonyl compounds	*		37.9	0	M+FA-H	C9H18O	0.558266442	345.3926933	394.6894324	353.1876344	370.3425195	289.6545411	328.397379	351.7570478	222.2628813	296.6483371
8.39_439.1588m/z	439.1588363	8.393383333	neg	Jasminine	68064	Unclassified	Unclassified	Unclassified			37.9	0	2M-H	C11H12N2O3	-7.887248547	3680.986102	3459.844079	3478.035848	4458.617848	4467.464626	4383.052649	1266.934235	1749.173693	1803.11731
8.86_465.2497m/z	465.2496976	8.860733333	neg	3alpha-hydroxy-5alpha-androstane-17-one 3-D-glucuronide	84920	Unclassified	Unclassified	Unclassified			37.9	0	M-H	C25H38O8	0.65604319	5422.802142	6000.210844	5550.214143	8534.816217	7886.64407	7629.711798	6236.347666	6509.934284	6351.948254
9.17_313.2025m/z	313.202522	9.167966667	neg	(1R,2R)-3-oxo-2-pentyl-cyclopentanehexanoic acid	LMFA02010014	Lipids and lipid-like molecules	Fatty Acyls	Octadecanoids			37.9	0	M+FA-H	C16H28O3	1.768573891	837.0551951	898.4817309	977.7241716	786.7459679	943.2337074	829.8748019	1056.078506	903.8762989	821.2976788
9.32_236.1056m/z	236.105564	9.321783333	neg	4-Hydroxydebrisoquine	58417	Organoheterocyclic compounds	Tetrahydroisoquinolines	Unclassified	*		37.9	0	M+FA-H	C10H13N3O	7.844042348	6583.398507	6300.098214	4473.720686	6005.234256	8052.803043	6634.142376	5005.101836	5169.101159	6800.072556
0.67_485.1992m/z	485.1991823	0.6701	neg	Biotin amide	HMDB0001458	Organoheterocyclic compounds	Biotin and derivatives	Unclassified	*	C01893	37.8	0	2M-H	C10H17N3O2S	-3.777848804	6777.114334	7026.847054	6378.381267	8429.778764	6827.455295	7485.03625	7333.801637	6323.911562	6331.765191
0.74_232.0674n	213.0495719	0.7359	neg	Metsulfovax	72694	Unclassified	Unclassified	Unclassified			37.8	0	M-H2O-H, M-H	C12H12N2OS	1.632997532	7431.162943	3939.730584	14954.43151	3600.737973	20462.87054	5251.035404	8388.004319	17295.58067	22929.63401
0.82_182.0130m/z	182.0129654	0.819566667	neg	L-Homocysteic acid	HMDB0002205	Organic acids and derivatives	Carboxylic acids and derivatives	Amino acids, peptides, and analogues	**	C16511	37.8	0	M-H	C4H9NO5S	0.537627766	1191.127016	1458.301585	1338.518981	1118.383245	688.8557537	722.6767369	469.0599637	595.3506699	451.3626544
0.84_112.9373m/z	112.9373005	0.841733333	neg	Thiosulfate	HMDB0000257	Homogeneous non-metal compounds	Non-metal oxoanionic compounds	Non-metal thiosulfates	*	C05529	37.8	0	M-H	H2O3S2	0.359702565	212.9846739	461.702757	712.221444	1342.367765	812.3591391	593.9884491	1098.30451	1634.833036	990.0922573
0.88_201.1246m/z	201.1245551	0.884633333	neg	Alanylisoleucine	HMDB0028690	Organic acids and derivatives	Carboxylic acids and derivatives	Amino acids, peptides, and analogues	*		37.8	0	M-H	C9H18N2O3	0.440975406	1260.877212	1086.272397	1099.96552	703.3624192	608.8433774	775.5468146	1005.515224	937.9777229	772.3507083
1.03_216.0991m/z	216.0990901	1.033516667	neg	Alanyl-Gamma-glutamate	HMDB0028701	Organic acids and derivatives	Carboxylic acids and derivatives	Amino acids, peptides, and analogues	*		37.8	0	M-H	C8H15N3O4	0.509284776	259.6057114	251.0107546	282.0734536	419.9396605	495.5227043	497.2604836	489.923255	475.2194405	486.2649068
1.14_365.0964m/z	365.096363	1.1382	neg	Methyl(2-furoylamino)acetic acid	5824	Unclassified	Unclassified	Unclassified			37.8	0	2M-H	C8H9NO4	-7.309560262	5466.023049	10786.9326	8064.487732	12639.80262	5979.052048	6365.874187	8575.349557	5547.432655	6887.918631
1.29_201.1134m/z	201.1134352	1.290916667	neg	Heptylmalonic acid	96283	Lipids and lipid-like molecules	Fatty Acyls	Fatty acids and conjugates	*		37.8	0	M-H	C10H18O4	1.0022145	322.0297782	248.6071086	346.8972971	429.409536	498.0720334	377.3910234	445.3157661	388.4616904	347.9271285
1.59_433.1104m/z	433.1103737	1.5934	neg	2-(Hydroxymethyl)-3-(acetamidomethylene)succinate	63868	Unclassified	Unclassified	Unclassified			37.8	0	2M-H	C8H11NO6	0.866101787	460.6025509	504.9570719	351.1676459	628.8638286	777.3933078	423.1329767	314.610777	374.9243835	442.1200739
1.86_216.0337m/z	216.0337076	1.86075	neg	2-(4-Methyl-5-thiazolyl)ethyl formate	88444	Organoheterocyclic compounds	Azoles	Thiazoles	*		37.8	0	M+FA-H	C7H9NO2S	0.614799812	1241.077637	1118.111649	1640.790507	1687.326207	1484.306919	1495.185093	1703.327905	1372.363391	1705.880513
10.47_549.1802m/z	549.1801841	10.46831667	neg	Genipin 1-beta-gentiobioside	71467	Unclassified	Unclassified	Unclassified			37.8	0	M-H	C23H34O15	-4.198363268	6285.46592	7706.626376	8618.373411	8459.585016	8023.728115	7684.903864	7901.631383	8439.727438	8084.221586
11.26_269.2124m/z	269.212379	11.25696667	neg	3,7,11-Trimethyl-6E,10-dodecadien-1-ol	LMFA05000145	Lipids and lipid-like molecules	Fatty Acyls	Fatty alcohols			37.8	0	M+FA-H	C15H28O	0.716256221	1771.684385	1842.042717	1802.94816	1407.846089	1312.592996	1669.155416	1971.611701	1303.000522	1885.335272
2.03_293.0880m/z	293.0880489	2.025516667	neg	Vicianose	65623	Unclassified	Unclassified	Unclassified			37.8	0	M-H2O-H	C11H20O10	0.779310737	707.2816542	713.2384204	979.8369502	1610.447584	1478.183894	1743.050597	913.3634605	986.7322493	686.5451353
2.03_423.0945m/z	423.0944608	2.025516667	neg	Azimsulfuron	72244	Organic nitrogen compounds	Organonitrogen compounds	Sulfonylureas		C18438	37.8	0	M-H	C13H16N10O5S	-1.99827541	1371.287122	1539.072305	1734.984072	2704.227878	2461.918211	2229.079238	2030.766312	2184.818439	1797.993955
2.11_265.0330m/z	265.0330082	2.114866667	neg	Thioproline	HMDB0062164	Organic acids and derivatives	Carboxylic acids and derivatives	Amino acids, peptides, and analogues			37.8	0	2M-H	C4H7NO2S	2.953200688	17780.48526	21030.77799	20446.62511	24045.5344	23940.32734	25285.60455	18636.7271	19107.18949	21775.80987
2.19_190.0511m/z	190.0510884	2.194566667	neg	4-Hydroxyquinoline	66379	Unclassified	Unclassified	Unclassified			37.8	0	M+FA-H	C9H7NO	0.839171462	419.4670474	424.0224687	556.9528997	664.9724553	667.9549122	755.9599642	481.5578136	594.9221652	645.7370091
2.48_246.9919m/z	246.9918983	2.479216667	neg	Vanillic acid 4-sulfate	96140	Benzenoids	Benzene and substituted derivatives	Benzoic acids and derivatives	*		37.8	0	M-H	C8H8O7S	0.407679165	6030.855146	6065.947034	6887.474949	2745.291726	2446.42822	2449.675297	3601.356117	3954.104711	3555.270386
2.93_215.0386m/z	215.0385539	2.926066667	neg	2,5-Dimethyl-3-furanthiol acetate	88283	Organosulfur compounds	Thioethers	Aryl thioethers	*		37.8	0	M+FA-H	C8H10O2S	1.178983952	824.6596848	773.5983229	854.196513	310.7813918	433.437946	437.7172493	850.5376047	917.3787815	746.362983
3.02_366.0832m/z	366.0832258	3.02	neg	Zeanoside C	93570	Organoheterocyclic compounds	Benzoxazines	Unclassified			37.8	0	M-H2O-H	C16H19NO10	0.444435275	730.1782373	671.9728507	851.3918858	2189.577777	2743.365466	2243.595866	1532.418736	1444.735689	854.0428625
3.04_235.0337m/z	235.0337012	3.041866667	neg	3-Nitrosothiazolidine	68860	Unclassified	Unclassified	Unclassified			37.8	0	2M-H	C3H6N2OS	3.431628962	1431.632285	1517.224272	1689.745112	2253.229716	2492.801903	2385.724482	1747.389659	1619.913068	1401.551864
3.07_303.0811m/z	303.081124	3.067683333	neg	Cystamine	HMDB0250701	Unclassified	Unclassified	Unclassified			37.8	0	2M-H	C4H12N2S2	0.06454759	777.0697918	829.574274	1071.440669	1973.146565	1948.851757	2207.583555	1726.758156	1417.946384	1403.794573
3.42_507.0346m/z	507.0345803	3.417666667	neg	Shikimate-3-phosphate	3384	Unclassified	Unclassified	Unclassified			37.8	0	2M-H	C7H11O8P	6.985366714	4744.466488	5703.642775	5663.374596	2375.318299	2420.966224	2693.758513	3022.028417	2928.598961	2591.420662
3.51_201.1134m/z	201.1133894	3.509133333	neg	Dihydro-5-pentyl-2(3H)-furanone	87740	Organoheterocyclic compounds	Lactones	Gamma butyrolactones	*		37.8	0	M+FA-H	C9H16O2	1.004294534	298.2599772	326.6994824	359.2509393	344.7199618	459.0351307	373.8636161	551.0991082	410.4841833	481.4046232
3.77_259.0758m/z	259.0758096	3.770416667	neg	Glutamylmethionine	HMDB0028825	Organic acids and derivatives	Carboxylic acids and derivatives	Amino acids, peptides, and analogues	*		37.8	0	M-H2O-H	C10H18N2O5S	0.02893622	1675.66009	1484.135482	1766.60411	552.1850212	366.4065042	257.8327081	1067.517339	872.1535797	976.0083316
3.82_300.1299n	281.1120262	3.82415	neg	Promazine 5-sulfoxide	HMDB0013938	Organoheterocyclic compounds	Benzothiazines	Phenothiazines	*		37.8	3.03	M-H2O-H, M-H	C17H20N2OS	0.776626911	8885.034611	9153.813449	9615.743967	17952.08035	16822.59013	16500.54154	13830.11544	14299.7271	14512.68677
3.87_444.1851m/z	444.1850869	3.868533333	neg	Diethylpropion (metabolite VII Glucuronide)	2026	Unclassified	Unclassified	Unclassified			37.8	0	M+FA-H	C19H29NO8	-6.094595411	553.7249591	784.8833082	721.5255015	698.3251874	666.9073422	1016.937991	720.7413191	859.1515492	745.2026899
3.89_306.9968m/z	306.9967804	3.8906	neg	(±)-Mevalonic acid 5-pyrophosphate tetralithium salt	397	Unclassified	Unclassified	Unclassified			37.8	0	M-H	C6H14O10P2	-7.022819378	1469.260205	1804.835808	1659.480294	316.7150348	426.0441956	442.3690045	1583.057277	1712.948818	1568.766782
3.94_369.0686m/z	369.0685507	3.9351	neg	1,2-beta-D-Glucuronosyl-D-glucuronate	66167	Unclassified	Unclassified	Unclassified			37.8	0	M-H	C12H18O13	2.935900744	0.001403199	291.538899	298.4462053	662.3761379	1066.29772	672.250426	149.2697665	530.1216849	248.6659843
4.07_307.0941m/z	307.0940552	4.069216667	neg	Dinobuton	72497	Unclassified	Unclassified	Unclassified			37.8	0	M-H2O-H	C14H18N2O7	1.519097889	794.0190703	775.7753878	672.7441075	1217.277963	1174.367894	1011.115637	616.17972	674.6671384	622.4450683
4.09_417.2067m/z	417.2067479	4.091783333	neg	PROPOXUR	44243	Unclassified	Unclassified	Unclassified			37.8	0	2M-H	C11H15NO3	8.698115634	3951.412377	4085.94104	4230.230416	2667.185415	2607.069271	2914.963783	4436.806763	4353.352077	4479.521306
4.11_307.1038m/z	307.1038201	4.113883333	neg	2-O-a-L-Fucopyranosyl-galactose	HMDB0006590	Lipids and lipid-like molecules	Fatty Acyls	Fatty acyl glycosides	*		37.8	0	M-H2O-H	C12H22O10	1.11699756	726.4424522	544.3288253	659.0419377	1294.360763	762.3588238	918.2828361	711.0386032	748.6695043	704.4013815
4.11_315.0966m/z	315.0966279	4.113883333	neg	Aspartyl-Histidine	85669	Organic acids and derivatives	Carboxylic acids and derivatives	Amino acids, peptides, and analogues	*		37.8	0	M+FA-H	C10H14N4O5	7.425044958	3340.755711	3646.317132	3847.923983	4878.116739	4400.484592	4524.183814	5361.24246	5473.477407	5075.991295
4.11_399.0935m/z	399.0934726	4.113883333	neg	Cis-5-Caffeoylquinic acid	HMDB0029278	Organic oxygen compounds	Organooxygen compounds	Alcohols and polyols	***		37.8	0	M+FA-H	C16H18O9	0.529833305	1508.269034	1622.123976	1655.239476	908.6681398	1010.767544	1030.895603	1705.394367	1786.347934	1501.463044
4.14_142.0663m/z	142.0662741	4.136366667	neg	1-Naphthylamine	70324	Unclassified	Unclassified	Unclassified			37.8	0	M-H	C10H9N	0.357896937	329.5204237	331.0389484	362.87739	1142.690394	806.3750724	926.2306848	603.4394301	466.658424	484.3980496
4.16_290.0647m/z	290.0647485	4.15895	neg	5-azauridine	686	Unclassified	Unclassified	Unclassified			37.8	0	M+FA-H	C8H11N3O6	7.184082138	423.9115327	499.7443711	524.1629208	631.1830996	324.9312446	376.6249722	578.7562237	574.2580109	377.457249
4.16_388.0630m/z	388.0630427	4.15895	neg	N-Acetylneuraminic acid 9-phosphate	58238	Organic oxygen compounds	Organooxygen compounds	Carbohydrates and carbohydrate conjugates	**	C06241	37.8	0	M-H	C11H20NO12P	-5.121481727	844.2950059	1090.650693	850.663867	892.3702591	566.6105249	712.8953514	1656.09277	905.3802075	945.5912978
4.18_245.0125m/z	245.0125006	4.181333333	neg	3-[3-(Sulfooxy)phenyl]propanoic acid	HMDB0094710	Organic acids and derivatives	Organic sulfuric acids and derivatives	Arylsulfates			37.8	0	M-H	C9H10O6S	-0.130256631	1664.550516	1311.052579	1446.048146	635.4407257	596.1423379	668.8719638	1269.407052	950.9566488	988.67817
4.19_160.0402m/z	160.0402389	4.189783333	neg	2-Indolecarboxylic acid	HMDB0002285	Organoheterocyclic compounds	Indoles and derivatives	Indolecarboxylic acids and derivatives	*		37.8	0	M-H	C9H7NO2	-1.012871776	604.5596039	470.1811251	455.3512856	445.4814934	519.0089637	543.2628733	634.3212614	593.8985339	485.3143633
4.19_467.0268m/z	467.0267741	4.189783333	neg	5-(4-Hydroxybut-1-ynyl)-2,2'-bithiophene	66193	Organoheterocyclic compounds	Bi- and oligothiophenes	Unclassified		C04486	37.8	0	2M-H	C12H10OS2	-1.203717745	4514.22042	3319.251674	3109.053315	2554.26956	2187.785594	2691.563088	3079.349285	3189.205602	3080.922961
4.39_348.0814m/z	348.0813712	4.3893	neg	Ulifloxacin	HMDB0259395	Unclassified	Unclassified	Unclassified			37.8	1.44	M-H	C16H16FN3O3S	-2.844325357	37296.19932	40982.79763	38916.34737	26953.85922	24713.4798	25087.5139	43426.85206	44206.81333	46213.64176
4.39_617.1843m/z	617.184273	4.3893	neg	Linoside B	49486	Lipids and lipid-like molecules	Polyketides	Flavonoids			37.8	0	M-H2O-H	C30H36O15	-5.196956988	56431.76931	62662.75681	57668.48182	33786.55485	31071.13289	29063.00355	65953.04266	68797.72405	71911.06136
4.41_218.0459m/z	218.045914	4.41115	neg	Quinaldic acid	HMDB0000842	Organoheterocyclic compounds	Quinolines and derivatives	Quinoline carboxylic acids	*	C06325	37.8	0	M+FA-H	C10H7NO2	0.188472508	448.2639306	528.3025084	349.4157157	1595.313283	1309.008098	1669.018796	536.985736	880.7646893	766.1294027
4.41_447.0788m/z	447.0787822	4.41115	neg	N-Adenylylanthranilate	65950	Unclassified	Unclassified	Unclassified			37.8	0	M-H2O-H	C17H19N6O8P	-7.669740607	29925.34116	32008.64353	32210.1147	7769.73895	7678.76893	6908.085726	18696.15082	20996.2574	17987.33679
4.50_316.0838m/z	316.0838295	4.49925	neg	Koeniginequinone B	88192	Organoheterocyclic compounds	Indoles and derivatives	Carbazoles	*		37.8	0	M+FA-H	C15H13NO4	4.311289142	2967.751107	3201.74702	3484.036985	6874.233445	6192.620872	6437.992295	7717.283631	7711.587134	7761.103191
4.52_213.1135m/z	213.1134984	4.521733333	neg	(+/-)-Dihydromintlactone	HMDB0032219	Organoheterocyclic compounds	Benzofurans	Unclassified	*		37.8	0	M+FA-H	C10H16O2	1.580929483	920.3927597	1111.157151	1173.084233	1359.575723	1426.415874	1464.898	1053.100134	959.077695	1096.465572
4.52_431.1926m/z	431.1926208	4.521733333	neg	Sonchuionoside C	HMDB0035212	Lipids and lipid-like molecules	Prenol lipids	Terpene glycosides	*		37.8	0	M+FA-H	C19H30O8	0.906308764	537.8103532	910.1417639	988.5842445	1475.845662	722.759291	1059.20871	939.9894377	722.3867789	688.7784869
4.57_388.1804m/z	388.1803711	4.5668	neg	Roquefortine	86932	Organoheterocyclic compounds	Indoles and derivatives	Pyrroloindoles	*		37.8	0	M-H	C22H23N5O2	6.353047756	343.7918382	475.7437317	265.2006903	547.6015998	338.575424	228.8805193	723.7117757	737.7647244	1054.172278
4.57_481.1352m/z	481.1352044	4.5668	neg	Trilobatin	HMDB0037505	Phenylpropanoids and polyketides	Flavonoids	Flavonoid glycosides	***		37.8	0	M+FA-H	C21H24O10	0.125121374	794.9983101	1057.878583	825.8580468	423.4460308	389.0858408	165.4466949	1541.839061	1516.259566	1720.209934
4.59_285.1111m/z	285.1111367	4.589283333	neg	Trimethadione	85367	Organoheterocyclic compounds	Azolidines	Oxazolidines			37.8	0	2M-H	C6H9NO3	6.734429735	894.1157175	332.6999013	2795.632551	367.6063434	358.7556895	403.1643653	438.7673118	302.4261008	918.6860727
4.59_289.0389m/z	289.0389322	4.589283333	neg	3,4-Dihydroxyphenylvaleric acid 4 sulfate	HMDB0240447	Unclassified	Unclassified	Unclassified			37.8	0	M-H	C11H14O7S	0.637344377	3447.510412	3766.222724	3601.75468	2717.924162	2683.118198	2293.44501	3998.296268	3794.824208	4270.820704
4.61_442.1722m/z	442.1722062	4.611883333	neg	Diethylpropion(metabolite V-glucuronide)	2021	Unclassified	Unclassified	Unclassified			37.8	0	M+FA-H	C19H27NO8	0.847201992	1507.103736	1637.966343	1322.348436	2335.984335	2477.721837	1970.592391	1556.741166	1376.061024	2116.658661
4.68_206.0460m/z	206.0460123	4.682483333	neg	4,6-Dihydroxyquinoline	58204	Organoheterocyclic compounds	Quinolines and derivatives	Quinolones and derivatives	**	C05639	37.8	0	M+FA-H	C9H7NO2	0.813209831	732.7385042	546.1091083	862.2676788	759.229463	765.6033558	712.6443037	906.2274865	877.9409928	486.5400212
4.73_417.1299m/z	417.1299087	4.725416667	neg	2-N,6-N-Bis(2,3-dihydroxybenzoyl)-L-lysine	66403	Unclassified	Unclassified	Unclassified			37.8	0	M-H	C20H22N2O8	-1.029659487	780.5804947	924.8988207	465.5292888	230.6798111	214.010143	234.8830987	487.3828891	604.1044847	608.7347901
4.84_361.0603m/z	361.0603188	4.836566667	neg	fosphenytoin	4050	Benzenoids	Benzene and substituted derivatives	Diphenylmethanes		C07840	37.8	0	M-H	C16H15N2O6P	2.271255844	5651.221163	5882.159171	5572.569615	4193.546378	4287.019468	4371.084206	5037.195933	5084.299631	5506.859199
4.86_282.1349m/z	282.1348729	4.858383333	neg	3-O-Methylrimiterol	2336	Unclassified	Unclassified	Unclassified			37.8	0	M+FA-H	C13H19NO3	0.744564967	776.2622556	828.9269561	880.6329644	2054.819792	2049.805263	2039.001176	744.7551235	1048.058488	851.2700247
4.86_454.1737m/z	454.1736822	4.858383333	neg	Tryptophol [xylosyl-(1->6)-glucoside]	95287	Organic oxygen compounds	Organooxygen compounds	Carbohydrates and carbohydrate conjugates			37.8	0	M-H	C21H29NO10	3.981963462	1015.116954	991.8661458	1335.718516	1170.824682	1438.071519	1278.792666	839.0556014	851.3239485	1006.624231
4.90_323.1227m/z	323.1227131	4.902316667	neg	L-phenylalanyl-L-hydroxyproline	62023	Organic acids and derivatives	Carboxylic acids and derivatives	Amino acids, peptides, and analogues	*		37.8	0	M+FA-H	C14H18N2O4	-7.719049896	7204.41022	7558.975742	7583.655539	13326.94286	13150.58112	13019.43031	8053.049059	8545.470382	8772.977902
4.92_284.1507m/z	284.1507064	4.92485	neg	Macrophylline	68265	Unclassified	Unclassified	Unclassified			37.8	0	M+FA-H	C13H21NO3	1.505412486	1006.637707	1132.772983	973.8516039	655.928227	641.9792408	603.5074238	661.8176599	585.7574081	632.0786087
4.97_472.2193m/z	472.2192784	4.969683333	neg	NBD-FTY720 phenoxy	64844	Unclassified	Unclassified	Unclassified			37.8	0	M-H	C23H31N5O6	-1.857227806	7689.777616	7952.076905	7969.734266	5951.382886	5006.131071	5571.085308	8614.853067	7850.171467	6653.127597
5.01_292.0418m/z	292.0418361	5.014716667	neg	Emtricitabine	69459	Nucleosides, nucleotides, and analogues	Nucleoside and nucleotide analogues	3'-thia pyrimidine nucleosides		C12599	37.8	0	M+FA-H	C8H10FN3O3S	3.816383299	408.8634991	331.9450266	391.1205461	686.2417834	732.3246294	672.2515013	719.9516523	468.2895393	428.8481922
5.04_271.1175m/z	271.1175338	5.037216667	neg	4-Acetyl-2-methylpyrimidine	92630	Organic oxygen compounds	Organooxygen compounds	Carbonyl compounds			37.8	0	2M-H	C7H8N2O	-9.244022208	1208.660815	1185.768463	1210.146528	1553.926516	1708.24197	1889.76051	1267.250733	1111.482758	999.0743754
5.06_529.2297m/z	529.2296704	5.05975	neg	Plantaricin BN	92965	Organic acids and derivatives	Carboxylic acids and derivatives	Tetracarboxylic acids and derivatives			37.8	0	M+FA-H	C24H36O10	1.280794233	1316.391734	1392.552011	1523.895981	662.8185145	625.8838161	667.987769	1003.485838	1013.848328	1583.102457
5.08_286.0912m/z	286.0912242	5.080566667	neg	Methocarbamol	43251	Benzenoids	Phenol ethers	Anisoles			37.8	0	M+FA-H	C11H15NO5	-8.300695606	362.1171304	290.2323325	493.1514974	353.4260118	277.5364884	409.6132259	444.2241683	255.4485665	324.8525455
5.08_391.1103m/z	391.1103453	5.080566667	neg	D-Altronate	63146	Unclassified	Unclassified	Unclassified			37.8	0	2M-H	C6H12O7	2.591818636	437.1931333	637.0047975	447.3009134	663.2981226	217.890191	778.99548	687.4454848	389.4314528	433.6237188
5.08_557.1277m/z	557.1277476	5.080566667	neg	ZLJ-6	45512	Unclassified	Unclassified	Unclassified			37.8	0	2M-H	C12H13N3O3S	-0.896787168	1453.033153	1606.948601	1122.062734	0.001403199	0.001403199	0.001403199	416.7282018	1235.640823	543.9653357
5.13_397.0972m/z	397.0972234	5.125666667	neg	Tenitramine	71877	Unclassified	Unclassified	Unclassified			37.8	0	M-H2O-H	C10H20N6O12	2.750218849	1945.907387	1533.244683	1568.011086	76.49965992	46.56905036	72.66604925	677.0941083	244.1011643	682.9368246
5.24_533.1305m/z	533.1305234	5.244033333	neg	Phrymarolin I	68537	Unclassified	Unclassified	Unclassified			37.8	0	M+FA-H	C24H24O11	0.940265386	4488.255922	5230.564034	4321.937645	130.80236	4.758075069	267.6884532	11.15463344	0.001403199	0.001403199
5.29_197.1184m/z	197.1183535	5.28555	neg	3-Methyl-5-propyl-2-cyclohexen-1-one	87974	Organic oxygen compounds	Organooxygen compounds	Carbonyl compounds	*		37.8	0	M+FA-H	C10H16O	0.233129453	555.0402101	548.1360117	495.1121767	620.1814299	608.5805576	753.7554973	389.4219507	521.0636911	630.8218591
5.32_497.2396m/z	497.2396392	5.320233333	neg	19-Norandrosterone glucuronide	1438	Unclassified	Unclassified	Unclassified			37.8	0	M+FA-H	C24H36O8	0.924767503	306.0062281	544.8083574	749.1628215	833.9120453	1151.071802	951.7704857	469.1717261	438.1211319	604.8618315
5.34_196.0980m/z	196.0980291	5.3394	neg	1,2,3,4,5,6-Hexahydro-5-methyl-7H-cyclopenta[b]pyridin-7-one	94213	Organoheterocyclic compounds	Pyridines and derivatives	Hydropyridines	*		37.8	0	M+FA-H	C9H13NO	0.742772781	268.3075981	318.3197487	372.6871405	475.020762	520.1688445	440.9579769	306.8051851	343.777113	393.6564082
5.36_199.1341m/z	199.1340838	5.361783333	neg	2-hydroxy-10-undecenoic acid	74542	Lipids and lipid-like molecules	Fatty Acyls	Fatty Acids and Conjugates			37.8	0	M-H	C11H20O3	0.57834865	303.4740784	514.8564894	423.3685733	410.8926014	395.8686644	402.3170992	316.9816088	240.1750736	308.4125272
5.36_547.0482m/z	547.0481757	5.361783333	neg	Bis(1L-myo-inositol)-3,1'-phosphate 1-phosphate	73367	Unclassified	Unclassified	Unclassified			37.8	0	M+FA-H	C12H24O17P2	2.191476177	1451.160702	1739.167435	2120.42592	1424.78605	1768.158609	1953.158107	1259.673621	1615.397023	1604.350509
5.36_821.0679m/z	821.0679207	5.361783333	neg	Deoxyadenosine diphosphate (dADP)	3526	Unclassified	Unclassified	Unclassified			37.8	0	2M-H	C10H15N5O9P2	7.537570751	2080.654528	2264.465449	2627.035716	661.5761149	1011.694277	446.0326485	2485.029774	2411.600561	3684.72205
5.43_337.0228m/z	337.0228015	5.428733333	neg	Alanylphosphate	69258	Unclassified	Unclassified	Unclassified			37.8	0	2M-H	C3H8NO5P	6.093914905	2052.032241	2185.496389	2089.679318	1151.50333	1445.145026	1320.539459	1791.498511	1701.935112	1324.011702
5.56_626.2437m/z	626.2437353	5.56185	neg	Tri-N-acetylchitotriose	HMDB0006698	Organic oxygen compounds	Organooxygen compounds	Carbohydrates and carbohydrate conjugates	*		37.8	0	M-H	C24H41N3O16	3.71384585	7713.31312	6810.988181	7243.61621	9791.917285	12389.36346	10977.51577	5918.734794	7242.226441	5772.562281
5.57_425.1819m/z	425.181939	5.57225	neg	Cinncassiol C	90494	Organic oxygen compounds	Organooxygen compounds	Carbohydrates and carbohydrate conjugates	*		37.8	0	M+FA-H	C20H28O7	0.612610616	3144.026485	2451.450184	3143.499201	1049.172475	1384.698591	1023.927094	3322.137192	2688.337389	2614.922234

5.58_228.1242m/z	228.1242003	5.584533333	neg	3-Oxa-9-azoniatricyclo[3.3.1.02,4]nonane, 9-ethyl-7-hydroxy-9-methyl-	1680	Unclassified	Unclassified	Unclassified			37.8	0	M+FA-H	C10H17NO2	0.375011775	555.5825788	482.9780961	656.1147869	1204.326951	1277.018483	1208.396446	644.2927279	795.5502624	594.2764702
5.65_530.1648m/z	530.1647816	5.650833333	neg	1-deoxy-1-[methyl[3-phenyl-3-[4-(trifluoromethyl)phenoxy]propyl]amino]-b-D-Glu	2770	Unclassified	Unclassified	Unclassified			37.8	0	M+FA-H	C23H26F3NO7	0.910891815	358.9728286	545.2127153	729.1351075	1915.663441	1860.264713	1629.784387	1994.440382	1734.599172	2088.219444
5.79_611.2849m/z	611.284897	5.785316667	neg	PHHdiA-PA	LMGP20070011	Lipids and lipid-like molecules	Glycerophospholipids	Oxidized glycerophospholipids			37.8	0	M+FA-H	C26H47O11P	1.934032538	1789.261153	2034.788973	2216.098536	2334.793214	2959.050171	2455.761348	4051.603456	3425.850838	2686.353241
5.81_375.0830m/z	375.0830202	5.806016667	neg	Desmethyldehydronifedipine	1537	Unclassified	Unclassified	Unclassified			37.8	0	M+FA-H	C16H14N2O6	-1.117487832	1839.159636	1887.429637	1908.692814	6232.085444	5108.12243	4556.708459	3524.06094	3168.922829	3020.599723
5.88_215.1289m/z	215.1288811	5.88025	neg	Linalool oxide (trans-pyranoid)	87682	Organoheterocyclic compounds	Oxanes	Unclassified	*		37.8	0	M+FA-H	C10H18O2	-0.00925537	817.9914164	942.4051522	1026.400574	784.2063087	792.8292851	765.0189889	793.4802533	771.0457573	898.0676386
5.89_591.1298m/z	591.1297709	5.894166667	neg	5-Methoxy-6,7-methylenedioxyflavone	LMPK12111101	Unclassified	Unclassified	Unclassified			37.8	0	2M-H	C17H12O5	0.169516626	1397.20452	1591.925143	1729.999397	2096.766744	2351.110259	1727.567146	863.6673307	1329.713209	1340.575898
5.91_200.1294m/z	200.129366	5.908066667	neg	Methylisopelletierine	HMDB0030326	Organoheterocyclic compounds	Piperidines	Unclassified	*	C06184	37.8	0	M+FA-H	C9H17NO	0.960253131	540.7881665	525.2725351	458.5488644	359.9268908	469.404331	322.2743168	407.1185499	466.2926906	501.5720275
5.99_593.2586m/z	593.2585864	5.9916	neg	Archangelolide	67582	Unclassified	Unclassified	Unclassified			37.8	0	M+FA-H	C29H40O10	-3.21735401	13544.0488	13885.38995	13864.41681	16387.4066	18125.06019	18664.3605	10275.9208	11075.73159	12188.69971
6.02_293.1510m/z	293.1509849	6.01945	neg	Pindolol	1943	Organoheterocyclic compounds	Indoles and derivatives	Indoles		C07445	37.8	0	M+FA-H	C14H20N2O2	1.225784026	292.9683065	413.1567243	264.5273988	1119.115229	790.0125932	1020.881916	488.5833905	486.8541002	507.7347866
6.07_659.1167m/z	659.1167382	6.07	neg	5-Hydroxydantrolene	1927	Organoheterocyclic compounds	Azolidines	Imidazolidines	*		37.8	0	2M-H	C14H10N4O6	5.978524445	3117.543345	3555.540489	3289.395764	1948.801119	2603.958009	2197.072274	3575.90458	3448.037541	3476.297915
6.20_235.0953m/z	235.0953029	6.195716667	neg	DIHYDRO-β-TUBAIC ACID	44213	Unclassified	Unclassified	Unclassified			37.8	4.04	M-H	C13H16O4	-9.655230148	12874.032	11728.26918	14209.84795	21890.52051	20526.04854	21908.65878	13960.27884	15442.21056	16214.28415
6.22_481.1876m/z	481.1875737	6.215	neg	Artocarpin	HMDB0030849	Phenylpropanoids and polyketides	Flavonoids	Flavones	*		37.8	0	M+FA-H	C26H28O6	1.793286603	1488.426728	1362.860325	1642.029523	2157.560663	1950.797797	1926.737449	1332.302956	960.680194	1331.071388
6.25_753.1945m/z	753.1944501	6.248083333	neg	SB 203580	45450	Unclassified	Unclassified	Unclassified			37.8	0	2M-H	C21H16FN3OS	2.789378061	13420.08708	10542.74109	12344.76642	15745.57079	12352.56569	14626.90916	13239.98845	13393.88769	12079.73736
6.26_249.0745m/z	249.0745379	6.261966667	neg	Alanyl-Aspartate	85599	Organic acids and derivatives	Peptidomimetics	Hybrid peptides	*		37.8	0	M+FA-H	C7H12N2O5	8.396464564	1869.144675	1395.270571	1684.002762	2241.51789	1848.757073	2161.918976	1818.321056	1937.449411	2051.196964
6.26_743.1675m/z	743.1674765	6.261966667	neg	Malvidin 3-glucoside-5-(6-acetylglucoside)	47177	Unclassified	Unclassified	Unclassified			37.8	0	M+FA-H	C30H34O19	-0.222399427	2284.516238	1846.732168	1967.395271	2271.928708	1682.288464	1980.307273	2218.002125	2473.050667	2193.294976
6.28_247.0953m/z	247.0953272	6.2811	neg	Orcinol	44798	Unclassified	Unclassified	Unclassified			37.8	0	2M-H	C7H8O2	-9.090493253	566.0498435	495.4444016	315.76411	1065.160484	1095.76039	904.1436302	489.1730721	560.5172355	725.38666
6.28_451.1591m/z	451.1590754	6.2811	neg	3-(4-Hydroxy-3-methoxyphenyl)-2-methyllactic acid	HMDB0060736	Phenylpropanoids and polyketides	Phenylpropanoic acids	Unclassified	*		37.8	0	2M-H	C11H14O5	-4.191518505	4114.5877	4644.385314	5086.024293	4030.453954	4149.238132	4052.182261	2750.141733	3092.341603	3792.929192
6.33_573.1366m/z	573.1365744	6.331216667	neg	Sulmazole	HMDB0258615	Unclassified	Unclassified	Unclassified			37.8	0	2M-H	C14H13N3O2S	-3.212630242	1216.685326	1505.909282	1756.43293	2142.669011	1761.327662	1822.123024	1221.633555	1362.685425	1334.121698
6.35_314.1978m/z	314.1977771	6.348783333	neg	N-[(Ethoxycarbonyl)methyl)-p-menthane-3-carboxamide	HMDB0032255	Organic acids and derivatives	Carboxylic acids and derivatives	Amino acids, peptides, and analogues	*		37.8	0	M+FA-H	C15H27NO3	1.784995028	603.0132257	653.1124816	665.4941772	648.2091271	765.469981	841.8199577	878.769769	1231.784728	991.0495156
6.42_394.0923m/z	394.0922549	6.41995	neg	S-(Formylmethyl)glutathione	HMDB0060507	Organic acids and derivatives	Carboxylic acids and derivatives	Amino acids, peptides, and analogues	**	C14871	37.8	0	M+FA-H	C12H19N3O7S	-0.91349976	757.3620508	686.9704879	927.7432454	1640.697292	1502.108259	1590.710017	1099.126711	953.8460026	966.7411237
6.44_425.1459m/z	425.1459416	6.442716667	neg	Gibberellin A86	HMDB0038450	Lipids and lipid-like molecules	Prenol lipids	Diterpenoids	*		37.8	0	M+FA-H	C19H24O8	1.633524141	342.2999794	397.9019444	247.4078856	4084.439238	3286.977467	3621.917433	579.9899123	620.298642	582.4323032
6.44_497.1647m/z	497.1646721	6.442716667	neg	S-Acetyldihydrolipoamide-E	LMFA08020202	Lipids and lipid-like molecules	Fatty Acyls	Fatty amides	**	C16255	37.8	0	2M-H	C10H19NO2S2	1.017421562	3862.769246	3177.18497	3641.826306	3486.337513	3240.447384	3279.992042	3151.190378	3181.980324	3043.12631
6.46_442.1910m/z	442.1910167	6.458366667	neg	CAY10622	64836	Unclassified	Unclassified	Unclassified			37.8	0	M-H	C25H25N5O3	5.761356294	484.580568	233.6349208	470.8536549	3144.331775	2731.808151	2826.251779	1850.53583	1597.939213	1934.060777
6.52_335.1481m/z	335.1480582	6.520516667	neg	2-Furanylmethyl butanoate	92552	Lipids and lipid-like molecules	Fatty Acyls	Fatty acid esters	*		37.8	0	2M-H	C9H12O3	-5.81217961	749.2574678	764.4434038	946.6600456	792.3377082	859.4395491	1315.359051	750.8397271	827.346944	685.9872114
6.59_478.2084m/z	478.2083896	6.592933333	neg	Dextrorphan O-glucuronide	HMDB0010341	Alkaloids and derivatives	Morphinans	Unclassified	*		37.8	0	M+FA-H	C23H31NO7	0.310211237	3676.210377	3822.282439	3872.159736	2145.733825	2540.104154	2511.419715	3457.257292	3982.700712	4088.302467
6.62_399.1275m/z	399.1274995	6.61635	neg	Methyl helianthenoate F glucoside	95262	Lipids and lipid-like molecules	Fatty Acyls	Fatty acyl glycosides	*		37.8	0	M+FA-H	C17H22O8	-6.130474575	11226.44937	12882.84689	10558.8421	21925.81287	20678.82582	20914.13834	16409.37289	20137.1792	20779.38572
6.66_649.2848m/z	649.2847608	6.663683333	neg	Amdinocillin	HMDB0015294	Organic acids and derivatives	Carboxylic acids and derivatives	Amino acids, peptides, and analogues			37.8	0	2M-H	C15H23N3O3S	0.018394214	2967.74939	3714.159355	3656.672255	330.5443786	308.3091973	298.532067	1707.307483	2027.42978	2262.225641
6.92_385.1999m/z	385.1999443	6.915966667	neg	17-phenyl-trinor-PGE2	36138	Lipids and lipid-like molecules	Fatty Acyls	Eicosanoids			37.8	2.78	M-H	C23H30O5	-5.446211114	697105.6611	172571.1683	1944941.992	192084.4095	181881.6051	168174.833	193758.7935	185204.887	402979.6353
6.93_647.1213m/z	647.1212617	6.926366667	neg	Grevilline A	86324	Organoheterocyclic compounds	Pyrans	Pyranones and derivatives	*		37.8	0	2M-H	C18H12O6	2.718504101	2218.682779	122.3594868	9099.272656	0.001403199	107.6440082	142.3948037	123.3208602	124.497406	1176.434287
7.10_453.0758m/z	453.0757528	7.097333333	neg	5-(2'-Carboxyethyl)-4,6-Dihydroxypicolinate	58545	Organoheterocyclic compounds	Pyridines and derivatives	Hydropyridines	**	C05655	37.8	0	2M-H	C9H9NO6	-6.485082355	2237.481268	2250.035074	2172.492148	2022.444223	2662.747935	2173.971529	1065.140851	1403.129501	1197.749678
7.18_333.1817m/z	333.1817408	7.178516667	neg	4-Methoxytyramine	HMDB0012162	Benzenoids	Phenols	Methoxyphenols	*		37.8	0	2M-H	C9H13NO2	-0.718341673	348.0761081	401.8674378	359.1312576	495.5756046	394.9572903	372.2555828	397.9021036	477.6848393	414.9209002
7.20_679.2306m/z	679.2306138	7.201166667	neg	Hydroxypropyl methyl cellulose	89213	Organic oxygen compounds	Organooxygen compounds	Carbohydrates and carbohydrate conjugates			37.8	0	M-H	C25H44O21	0.56124025	7142.332768	8227.785212	7965.925544	5646.009467	5378.187503	5871.2406	4282.688912	5156.325987	3845.683378
7.31_340.1567m/z	340.1566569	7.314366667	neg	Doxepin N-oxide	HMDB0060594	Organoheterocyclic compounds	Benzoxepines	Dibenzoxepines	*		37.8	0	M+FA-H	C19H21NO2	4.150777425	3417.82613	3957.141032	3515.309451	4913.109371	4687.911105	4446.827337	2390.824645	2771.260197	3326.620696
7.35_359.1173m/z	359.1172746	7.353466667	neg	Galactinol dihydrate	62462	Unclassified	Unclassified	Unclassified			37.8	0	M-H2O-H	C12H26O13	-5.884492824	1094.256997	1074.544813	822.4638121	38.45057223	0.001403199	71.20978974	0.001403199	114.9393902	274.3067129
7.36_681.3474m/z	681.3474035	7.36395	neg	JWH 018	45443	Unclassified	Unclassified	Unclassified			37.8	0	2M-H	C24H23NO	-1.830073222	16178.58808	18609.07806	16631.01593	14902.34915	14135.88295	14814.8418	15771.39944	19241.21853	19485.00331
7.40_493.2247m/z	493.2247273	7.404266667	neg	Glutaminyl-Threonine	85721	Organic acids and derivatives	Carboxylic acids and derivatives	Amino acids, peptides, and analogues	*		37.8	0	2M-H	C9H17N3O5	-3.313362361	1237.997604	1721.966	1447.55855	82.77723215	79.80019039	72.18010002	502.8761173	536.985466	287.147973
7.56_483.2218m/z	483.2218132	7.556516667	neg	Ouabagenin	1671	Unclassified	Unclassified	Unclassified			37.8	0	M+FA-H	C23H34O8	-4.01095493	17082.7145	19157.39663	18263.05764	17627.67271	16613.47094	17316.85738	21853.01141	24308.23656	21668.65698
7.58_331.1765m/z	331.1764736	7.5758	neg	(1R,3S,4S)-1,8-Epoxy-p-menthan-3-ol glucoside	90196	Organic oxygen compounds	Organooxygen compounds	Carbohydrates and carbohydrate conjugates	*		37.8	0	M-H	C16H28O7	0.742852514	2410.909383	2570.717774	2708.462656	2069.380498	1654.462975	1603.737308	1672.763807	2211.078226	1560.224501
7.59_429.1768m/z	429.1768223	7.58625	neg	FK-409	62986	Unclassified	Unclassified	Unclassified			37.8	0	2M-H	C8H13N3O4	6.71080896	1375.632114	1346.156321	1229.123313	3250.743047	3033.300755	2945.647444	1113.087795	1415.560047	1107.513213
7.63_431.2291m/z	431.2291018	7.631783333	neg	10,11-dihydro-20-trihydroxy-leukotriene B4	HMDB0012503	Lipids and lipid-like molecules	Fatty Acyls	Eicosanoids	*		37.8	0	M+FA-H	C20H34O7	1.153526183	4652.278309	5511.592528	4548.500248	4068.339938	3027.947258	3881.0755	4131.312173	3828.892223	3842.71289
7.87_367.1589m/z	367.1588553	7.87455	neg	Testosterone sulfate	3558	Lipids and lipid-like molecules	Steroids and steroid derivatives	Sulfated steroids	*		37.8	0	M-H	C19H28O5S	1.050530865	654.5450181	753.6987664	891.5952867	962.2281627	1021.574647	765.7006856	382.8242354	606.48242	491.2626485
7.91_331.1765m/z	331.1764756	7.906766667	neg	(1S,2S,4S,5S)-2,4-Thujanediol 4-O-beta-D-Glucopyranoside	89443	Lipids and lipid-like molecules	Prenol lipids	Terpene glycosides	*		37.8	0	M-H	C16H28O7	0.7489133	659.659375	682.2928678	698.1540197	1452.731369	1505.868208	1533.291838	841.3640391	1059.152906	1014.55813
7.92_511.2913m/z	511.2913425	7.91555	neg	(24R)-11alpha,20,24-trihydroxyecdysone	LMST01010177	Lipids and lipid-like molecules	Sterol Lipids	Sterols			37.8	0	M-H	C27H44O9	0.167667831	2293.848531	2677.108826	2613.520036	1703.372741	1486.541215	2271.158808	1875.405733	2816.157688	2412.028831
7.93_539.2501m/z	539.2500914	7.9268	neg	7,11-Bisdeacetylvaltrate 7-(3-methylpentanoate) 11-(3-hydroxy-3-methylbutano	89454	Lipids and lipid-like molecules	Prenol lipids	Monoterpenoids	*		37.8	0	M+FA-H	C26H38O9	0.618498204	1445.566444	1216.351744	1168.883836	11302.24085	10693.99801	11088.28196	1043.899809	1125.570903	1028.466949
7.94_323.1478m/z	323.1478333	7.9373	neg	Valaciclovir	HMDB0014716	Organic acids and derivatives	Carboxylic acids and derivatives	Amino acids, peptides, and analogues	**	C07184	37.8	0	M-H	C13H20N6O4	1.562660306	1688.355711	1537.132304	1465.612142	2149.701618	2131.253494	2404.067747	1097.371655	1352.786062	1280.547436
8.40_563.2840m/z	563.2840154	8.40065	neg	Corchoroside B	86904	Lipids and lipid-like molecules	Steroids and steroid derivatives	Steroidal glycosides	*		37.8	0	M+FA-H	C29H42O8	-4.159437677	19074.45118	19738.60072	19568.27934	27325.11631	27848.82706	25065.28568	18566.66287	22772.93849	20975.71435
8.93_187.1341m/z	187.1341072	8.925416667	neg	Amyl propyl ketone	HMDB0031268	Organic oxygen compounds	Organooxygen compounds	Carbonyl compounds	*		37.8	0	M+FA-H	C9H18O	0.978887017	301.4767959	250.0770972	230.059882	235.7673203	287.3750382	389.3442373	235.1397901	276.688936	249.9063922
8.99_337.1189m/z	337.1188547	8.9919	neg	(-)-Blebbistatin	45431	Unclassified	Unclassified	Unclassified			37.8	0	M+FA-H	C18H16N2O2	-1.800558108	24079.66045	22094.64075	22670.99	22897.31451	24382.10194	25004.12595	26199.07057	23543.33344	24444.14585
9.02_951.1793m/z	951.1793354	9.01635	neg	Luteolin 4'-methyl ether 7-glucuronide	LMPK12110811	Phenylpropanoids and polyketides	Flavonoids	Flavonoid glycosides	*		37.8	0	2M-H	C22H20O12	-4.558291337	4967.57335	6863.093011	7375.994883	0.001403199	0.001403199	0.001403199	1986.983306	1560.770898	1597.932366
9.32_491.2866m/z	491.2865609	9.321783333	neg	3-hydroxy-dodecanedioic acid	LMFA01170089	Lipids and lipid-like molecules	Fatty Acyls	Fatty Acids and Conjugates			37.8	0	2M-H	C12H22O5	0.791574904	13098.37551	14731.1818	15765.61365	16447.3398	18245.287	17111.49459	14311.14029	15816.11608	15270.99211
9.84_509.0165m/z	509.0164927	9.835916667	neg	Sulfathiazole	43338	Benzenoids	Benzene and substituted derivatives	Benzenesulfonamides		C11169	37.8	0	2M-H	C9H9N3O2S2	-6.799534154	5672.625612	4343.442439	5213.00334	5189.258852	6189.608871	5799.746285	5773.744671	4879.228857	4970.95941
0.52_225.0131m/z	225.0130693	0.51805	neg	2-Nitrofuran	70043	Unclassified	Unclassified	Unclassified			37.7	0	2M-H	C4H3NO3	-9.911220132	274.1505471	356.7878433	373.5147318	303.1471392	365.9574739	259.6641464	373.2809611	329.2379925	446.8500335
0.74_402.9963m/z	402.9963329	0.7359	neg	Benzoyl phosphate	66355	Benzenoids	Benzene and substituted derivatives	Benzoic acids and derivatives		C06206	37.7	0	2M-H	C7H7O5P	-6.461708423	1862.249488	1362.131057	2217.982374	588.9081317	916.8155142	435.1574923	2779.5424	636.7129111	2162.236365
0.76_246.0733m/z	246.0733053	0.757316667	neg	Aspartyl-Asparagine	85663	Organic acids and derivatives	Carboxylic acids and derivatives	Amino acids, peptides, and analogues	*		37.7	0	M-H	C8H13N3O6	0.593385816	1093.132993	1111.405229	861.301086	969.618054	656.2017375	1103.920213	532.0997665	786.346132	930.9729371
0.78_250.9941m/z	250.9941223	0.778533333	neg	3,5-Dinitro-4-hydroxyphenylpyruvate	66154	Unclassified	Unclassified	Unclassified			37.7	0	M-H2O-H	C9H6N2O8	-1.672909801	7141.680196	7925.893106	6901.897886	2568.465108	1016.709028	2399.314075	5276.827387	5688.241692	4585.286582
0.78_357.1039m/z	357.1038704	0.778533333	neg	Arabino-galactose	66619	Unclassified	Unclassified	Unclassified			37.7	0	M+FA-H	C11H20O10	0.066345704	8195.668084	7699.955804	9236.427167	9727.033639	10046.06003	9127.405282	8564.441477	8350.459762	7465.310681
0.82_771.1986m/z	771.1986037	0.819566667	neg	8-8'-Dehydrodiferulic acid	86149	Lignans, neolignans and related compounds	Unclassified	Unclassified	*		37.7	0	2M-H	C20H18O8	7.180836984	18310.22658	21477.85093	21990.46278	31443.93464	25541.27051	26439.97959	19418.70106	20749.16797	21334.51946
0.84_363.0878m/z	363.0877746	0.841733333	neg	PHENOLPHTHALEIN	44257	Unclassified	Unclassified	Unclassified			37.7	0	M+FA-H	C20H14O4	1.140490586	16389.36725	15764.40005	18445.72816	9941.432591	12952.71695	10573.99826	15387.73372	16237.43391	16497.3216
0.86_201.0350m/z	201.0350301	0.86335	neg	SNAP	44297	Unclassified	Unclassified	Unclassified			37.7	0	M-H2O-H	C7H12N2O4S	4.968399531	3110.661241	3181.320645	3538.813291	3388.648062	3485.0896	2998.28819	3712.488876	3697.32133	3365.399891
0.88_235.0396m/z	235.039591	0.884633333	neg	5-L-Glutamyl-taurine	HMDB0004195	Organic acids and derivatives	Carboxylic acids and derivatives	Amino acids, peptides, and analogues	**	C05844	37.7	0	M-H2O-H	C7H14N2O6S	0.688477796	3517.77543	3213.84252	3254.480948	3275.431527	3850.765905	3795.272882	2995.993215	3189.536912	3015.29587
0.88_454.9981m/z	454.9981301	0.884633333	neg	Thiolutin	HMDB0034228	Organic nitrogen compounds	Organonitrogen compounds	N-arylamides			37.7	0	2M-H	C8H8N2O2S2	-0.071268811	1363.23835	1933.338924	3458.757019	1593.836996	2689.463241	1896.856298	2349.76713	1208.72052	3338.193281
0.90_192.0335m/z	192.0335186	0.904683333	neg	Thiomorpholine 3-carboxylate	HMDB0059611	Organic acids and derivatives	Carboxylic acids and derivatives	Amino acids, peptides, and analogues		C03901	37.7	0	M+FA-H	C5H9NO2S	-0.569925702	461.3851737	676.8046248	851.176858	665.4988424	685.5222456	731.7586771	792.6132935	718.2733154	832.758691
0.99_405.0289m/z	405.0288735	0.989233333	neg	Mikanin 3-O-sulfate	51296	Lipids and lipid-like molecules	Polyketides	Flavonoids			37.7	0	M-H2O-H	C18H16O10S	0.700187285	808.5365117	1145.240325	1608.426007	1914.821641	1260.975088	1671.648689	624.4586058	1417.229233	1194.707048
1.08_482.9609m/z	482.9609112	1.078266667	neg	Uridine triphosphate	HMDB0000285	Nucleosides, nucleotides, and analogues	Pyrimidine nucleotides	Pyrimidine ribonucleotides	**	C00075	37.7	0	M-H	C9H15N2O15P3	-0.702628282	5987.456987	6338.866094	6901.11762	8526.037423	6656.915436	7743.940687	6607.969702	7462.727376	6412.853872
1.16_279.0347m/z	279.0347099	1.160166667	neg	5-Hydroxypyrazinoic acid	2198	Unclassified	Unclassified	Unclassified			37.7	0	2M-H	C5H4N2O3	-8.561788342	4305.871017	4236.491386	4669.196113	2375.6422	523.5986863	2135.762594	1525.324331	3312.421218	644.2016591
1.16_403.0121m/z	403.0120848	1.160166667	neg	(1E)-4-Oxobut-1-ene-1,2,4-tricarboxylate	66182	Unclassified	Unclassified	Unclassified			37.7	0	2M-H	C7H6O7	-8.276343571	17433.52772	19447.16972	22266.98852	14751.80764	16408.6691	14656.6247	23811.22466	15872.34621	19289.2429
1.18_213.0130m/z	213.0130488	1.180366667	neg	Cyclobrassinone	HMDB0034209	Organoheterocyclic compounds	Indoles and derivatives	Indoles			37.7	3.19	M-H2O-H	C11H8N2O2S	1.04063969	4196.068895	3529.514787	2827.618505	3284.103972	2874.031571	3589.189774	2670.206316	3164.546452	3032.371557
1.18_231.0987m/z	231.0987211	1.180366667	neg	Threonylhydroxyproline	HMDB0029062	Organic acids and derivatives	Carboxylic acids and derivatives	Amino acids, peptides, and analogues	*		37.7	0	M-H	C9H16N2O5	0.326944395	883.8149274	1096.241873	1325.798172	1007.726803	1008.704575	1103.918286	1020.829065	1281.150563	796.1340156
1.38_145.0871m/z	145.0870766	1.380283333	neg	(+/-)-Ethyl 2-hydroxy-2-methylbutyrate	88315	Lipids and lipid-like molecules	Fatty Acyls	Fatty acid esters	*		37.7	0	M-H	C7H14O3	0.402042462	316.9279964	257.3835168	256.7413447	247.7207421	269.3166153	221.9499893	317.5240633	218.0003305	278.7606568
1.40_124.0404m/z	124.0404491	1.3958	neg	N-Furfurylformamide	65850	Unclassified	Unclassified	Unclassified			37.7	0	M-H	C6H7NO2	0.37619535	187.6719648	108.2669613	129.5699415	151.8867728	195.3545163	155.2850669	168.185214	165.0553154	153.0281421
1.46_238.9955m/z	238.9955149	1.459966667	neg	2-Dehydro-3-deoxy-6-phospho-D-gluconate	5064	Organic acids and derivatives	Keto acids and derivatives	Medium-chain keto acids and derivatives	**	C04442	37.7	0	M-H2O-H	C6H11O9P	-2.761800899	457.0692144	631.0907923	469.6087992	820.4673254	656.0447142	627.0725793	699.0521038	574.1017728	680.0761902
1.62_157.0772m/z	157.0771928	1.615883333	neg	1-Benzylimidazole	63058	Unclassified	Unclassified	Unclassified			37.7	0	M-H	C10H10N2	0.448732425	797.1129609	674.8947062	618.4305927	778.5420425	1076.529264	731.4108847	676.4047138	678.7664709	710.9242537
1.84_567.9424m/z	567.9423631	1.841666667	neg	Adenosine tetraphosphate	HMDB0001364	Lipids and lipid-like molecules	Fatty Acyls	Fatty acyl thioesters	**	C03483	37.7	0	M-H2O-H	C10H17N5O16P4	-3.188901625	3283.102972	3051.082189	3983.775985	1741.866849	1761.998978	1833.231579	4178.347761	3214.489694	3895.480833
1.96_629.1916m/z	629.1915506	1.95955	neg	Flusilazole	HMDB0039815	Benzenoids	Benzene and substituted derivatives	Halobenzenes		C18733	37.7	0	2M-H	C16H15F2N3Si	-2.91010767	8501.930267	7598.696058	6198.96253	0.001403199	0.001403199	0.001403199	7036.119019	7485.49411	6238.053933
11.63_759.5182m/z	759.5182435	11.6333	neg	PA(15:0/22:2(13Z,16Z))	HMDB0114826	Lipids and lipid-like molecules	Glycerophospholipids	Glycerophosphates	*		37.7	0	M+FA-H	C40H75O8P	0.118498012	37429.67883	31877.14759	31147.90591	15369.85742	11953.31667	9483.570538	38629.07252	27573.87724	25460.59248
13.02_743.5236m/z	743.523615	13.0167	neg	Cervonoyl ethanolamide	HMDB0013627	Lipids and lipid-like molecules	Fatty Acyls	Fatty acid esters	*	C13828	37.7	0	2M-H	C24H36O3	-2.684301909	216492.1481	217632.1442	216142.1542	160546.6665	190161.6547	150256.6988	235296.2676	197642.576	208236.8148
14.43_699.4965m/z	699.496545	14.43406667	neg	PA(18:1(11Z)/18:1(11Z))	59313	Lipids and lipid-like molecules	Glycerophospholipids	Glycerophosphates	**	C00416	37.7	0	M-H	C39H73O8P	-0.691590172	1193.738123	2137.021476	2014.577302	474.5594148	820.9233188	1575.740997	2278.479986	3073.165682	1694.932933
2.03_265.0555m/z	265.0555257	2.025516667	neg	Monoglyceride citrate	88460	Organic acids and derivatives	Carboxylic acids and derivatives	Tricarboxylic acids and derivatives	*		37.7	0	M-H	C9H14O9	-3.682835482	2399.386983	2502.246921	2541.896034	4668.031482	4244.556936	4458.021633	3244.849382	4058.09067	3381.011222
2.14_223.0005m/z	223.0004657	2.140766667	neg	SC-514	45240	Unclassified	Unclassified	Unclassified			37.7	0	M-H	C9H8N2OS2	-0.280060972	1697.415164	2054.344259	1979.135853	4015.059685	3719.453473	3646.004551	3073.253942	3221.913374	3487.854768
3.56_336.0502m/z	336.0501784	3.564783333	neg	1-(6-Oxo-6H-dibenzo[b,d]pyran-3-yl)-1H-pyrrole-2,5-dione	70902	Unclassified	Unclassified	Unclassified			37.7	0	M+FA-H	C17H9NO4	-4.062004301	2601.584335	3164.037427	2783.113412	3779.718688	3892.174264	3645.713984	2987.381245	2838.687717	2916.211308
3.61_354.1302m/z	354.1301659	3.611966667	neg	Glutaminyl-Tyrosine	85723	Organic acids and derivatives	Carboxylic acids and derivatives	Amino acids, peptides, and analogues	*		37.7	0	M+FA-H	C14H19N3O5	-1.642386745	978.2140716	1253.247431	938.8586083	1076.851626	1087.343343	1539.631921	1027.182604	1039.780904	1279.573935
3.71_256.0287m/z	256.0287466	3.708366667	neg	Methylthiobenzoylglycine	2540	Unclassified	Unclassified	Unclassified			37.7	0	M+FA-H	C9H9NO3S	1.087969393	767.040836	773.2101818	930.0209988	1073.463699	1175.934412	761.3801481	868.2221353	805.4063683	847.6778279
3.73_201.0770m/z	201.0769732	3.730516667	neg	2-Amino-1,6-dimethylfuro[3,2-e]imidazo[4,5-b]pyridine	90502	Organoheterocyclic compounds	Imidazopyridines	Unclassified			37.7	0	M-H	C10H10N4O	-5.994293409	660.2641291	616.2842408	581.5095485	754.2132627	689.7412694	524.2753868	518.4839037	482.9623055	540.4572335
3.85_295.0912m/z	295.0912396	3.84605	neg	beta-L-Aspartylhydroxamate	65909	Organic acids and derivatives	Carboxylic acids and derivatives	Amino acids, peptides, and analogues	*	C03124	37.7	0	2M-H	C4H8N2O4	5.749843398	1631.365439	1689.645624	1864.160585	1921.395259	2080.543622	1650.419075	1606.057531	1676.387297	1688.325297
3.85_363.0789m/z	363.0788717	3.84605	neg	9-Methyluric acid	58071	Organoheterocyclic compounds	Imidazopyrimidines	Purines and purine derivatives	*		37.7	0	2M-H	C6H6N4O3	-5.031706351	665.6087802	368.9370499	545.2406329	588.7148179	780.0225773	783.843463	459.4842191	593.2071571	468.2724933
3.85_400.0796m/z	400.0796077	3.84605	neg	Cyanidin 7-arabinoside	HMDB0037989	Phenylpropanoids and polyketides	Flavonoids	Hydroxyflavonoids			37.7	0	M-H2O-H	C20H19O10+	-0.889883681	2287.618835	3082.470844	3621.048954	3728.464718	3864.389267	3913.156613	3864.463114	4406.130047	4374.571896
3.85_575.0717m/z	575.0717344	3.84605	neg	dTDP-3-oxo-4,6-dideoxy-D-glucose	63832	Unclassified	Unclassified	Unclassified			37.7	0	M+FA-H	C16H24N2O14P2	6.140431649	4482.475063	4871.956055	4747.614608	4754.988016	4335.190577	4345.409174	4575.43972	5425.776703	5029.616051
3.94_370.0558m/z	370.0557749	3.9351	neg	Acetyl adenylate	HMDB0006880	Lipids and lipid-like molecules	Steroids and steroid derivatives	Bile acids, alcohols and derivatives	**	C05993	37.7	0	M-H2O-H	C12H16N5O8P	-0.085002984	1094.258087	947.0051505	1203.040965	4077.925011	3615.47647	3300.152855	3926.035999	4214.687612	3946.174466
4.16_243.0486m/z	243.0486098	4.15895	neg	4-Methyldibenzothiophene	HMDB0040164	Organoheterocyclic compounds	Benzothiophenes	Dibenzothiophenes			37.7	0	M+FA-H	C13H10S	0.432215248	1616.961826	1919.519235	1817.470724	2631.195448	2104.727397	2165.820471	2347.34289	1722.622003	1764.006448
4.18_311.0209m/z	311.0208678	4.181333333	neg	Inosine 2',3'-cyclic phosphate	62419	Organoheterocyclic compounds	Tetrapyrroles and derivatives	Porphyrins	**	C05768	37.7	0	M-H2O-H	C10H11N4O7P	6.585735017	446.8279981	318.6016534	445.2103785	0.001403199	0.001403199	0.001403199	1610.027564	1209.138054	1258.458986
4.21_317.0337m/z	317.0337196	4.212116667	neg	nicotinate beta-D-ribonucleotide	96249	Unclassified	Unclassified	Unclassified			37.7	0	M-H2O-H	C11H15NO9P+	9.278573757	11058.26433	12195.20125	12660.06311	4158.315259	3919.531483	4440.322835	12803.43652	14017.33201	12961.17849
4.23_228.0879m/z	228.0879407	4.234483333	neg	3,4,5,6-Tetrahydrohippuric acid	HMDB0061679	Organic acids and derivatives	Carboxylic acids and derivatives	Amino acids, peptides, and analogues			37.7	0	M+FA-H	C9H13NO3	1.062337957	69.25314401	118.1005866	250.2156087	495.6599378	523.4328771	516.252798	268.1128876	104.1738129	180.7777594
4.23_364.9926m/z	364.992567	4.234483333	neg	Saccharin	43328	Organoheterocyclic compounds	Benzothiazoles	Unclassified	**	C12283	37.7	0	2M-H	C7H5NO3S	4.960171479	2785.109331	3089.211617	3146.60582	2696.648726	3070.05592	3054.193459	2089.16892	2096.401646	2043.92534
4.30_413.1074m/z	413.1074229	4.300033333	neg	S-(2-Hydroxyethyl)-N-acetyl-L-cysteine	70379	Unclassified	Unclassified	Unclassified			37.7	0	2M-H	C7H13NO4S	3.964091168	784.8412477	1004.174539	535.3401194	954.9274761	1187.172254	1656.328513	710.9835286	550.2846031	772.514419
4.37_471.1493m/z	471.1492584	4.367166667	neg	N,N-Dihydroxy-L-tryptophan	73315	Unclassified	Unclassified	Unclassified			37.7	0	2M-H	C11H12N2O4	-6.097423836	627.4158026	2108.224487	1489.939501	3639.485704	3450.899291	2205.934715	1278.316349	1123.234726	812.3577194
4.45_451.1245m/z	451.1244823	4.454616667	neg	Afzelechin 7-O-beta-D-apiofuranoside	47247	Phenylpropanoids and polyketides	Flavonoids	Flavans	*		37.7	0	M+FA-H	C20H22O9	-0.253213232	751.432553	551.2237206	856.0892614	678.2654963	170.4762955	295.5252657	608.9670983	992.8597922	626.579835
4.48_309.1071m/z	309.1070782	4.477166667	neg	Bluensidine	71963	Unclassified	Unclassified	Unclassified			37.7	0	M+FA-H	C8H16N4O6	7.160285658	18648.08965	20602.36335	20121.03174	41043.59988	35572.90262	36428.28355	20256.17716	22276.88831	21807.11237
4.48_377.0947m/z	377.0946988	4.477166667	neg	Butamifos	68779	Unclassified	Unclassified	Unclassified			37.7	0	M+FA-H	C13H21N2O4PS	1.599344837	9739.849296	8880.681815	9527.81789	20125.09385	17867.62417	17274.50088	11107.39434	10442.58563	10405.06578
4.52_261.0358m/z	261.0358333	4.521733333	neg	Sorbitol-6-phosphate	58327	Organic oxygen compounds	Organooxygen compounds	Carbohydrates and carbohydrate conjugates	*	C02810	37.7	0	M-H	C6H15O9P	-8.620331795	1364.39363	1733.939183	1835.71252	3158.573177	3194.591558	2932.985302	2170.721289	2174.956387	3093.912006
4.54_461.1671m/z	461.1671196	4.54425	neg	Phenylethyl primeveroside	95688	Organic oxygen compounds	Organooxygen compounds	Carbohydrates and carbohydrate conjugates			37.7	0	M+FA-H	C19H28O10	1.608994088	2174.639958	2097.315286	1900.380869	1767.55179	1640.542051	1795.78707	2750.14086	2664.537908	3691.715481
4.57_276.9863m/z	276.9862762	4.5668	neg	2-C-Methyl-D-erythritol 2,4-cyclodiphosphate	64016	Unclassified	Unclassified	Unclassified			37.7	0	M-H	C5H12O9P2	-7.56350857	829.3566263	802.1575743	948.0292377	260.8003916	245.1649531	361.5664941	564.2979016	578.2251675	573.5593031
4.57_493.1718m/z	493.1718301	4.5668	neg	Harpagoside	HMDB0253053	Lipids and lipid-like molecules	Prenol lipids	Isoprenoids		C09783	37.7	0	M-H	C24H30O11	0.596459439	3360.6309	3895.594677	3948.782545	346.8164882	167.1953119	91.43546751	1858.341484	2237.912577	1912.758525
4.61_235.0589m/z	235.0589171	4.611883333	neg	Glycyl-Aspartate	85750	Organic acids and derivatives	Carboxylic acids and derivatives	Amino acids, peptides, and analogues	*		37.7	0	M+FA-H	C6H10N2O5	9.169717309	3258.15913	3692.293447	3516.781126	3480.630194	3422.236929	3159.788126	3961.103087	4277.823882	4582.991346
4.61_307.0798m/z	307.0797673	4.611883333	neg	Ng-L-Glutamyl-L-aspartic acid	86960	Unclassified	Unclassified	Unclassified			37.7	0	M+FA-H	C9H14N2O7	5.584600661	511.6758867	353.908788	391.4921711	588.5026543	579.1331893	731.3110214	1195.716181	1045.498348	1444.082261
4.70_257.0757m/z	257.0756592	4.703166667	neg	thymidine glycol	96212	Nucleosides, nucleotides, and analogues	Pyrimidine nucleosides	Pyrimidine 2'-deoxyribonucleosides	*		37.7	0	M-H2O-H	C10H16N2O7	-8.151124885	282.3976723	394.725057	538.3045836	474.9831515	533.5977162	513.4354835	391.5982695	675.579814	477.4246189
4.84_363.0759m/z	363.0759236	4.836566667	neg	Sulfometuron methyl	68719	Unclassified	Unclassified	Unclassified			37.7	0	M-H	C15H16N4O5S	-2.583546756	14374.98774	16163.093	16341.06491	14615.48326	13584.77714	13865.30941	14012.87963	15911.40772	14690.8722
4.88_505.0628m/z	505.0627763	4.880083333	neg	Rhein glucuronide 2	2415	Unclassified	Unclassified	Unclassified			37.7	0	M+FA-H	C21H16O12	0.863993014	737.0494841	708.8006812	564.4378368	881.2675364	1302.049369	1227.872617	994.3409871	1158.923396	1057.89558
4.92_275.0233m/z	275.0233426	4.92485	neg	Dihydroferulic acid 4-sulfate	96082	Organic acids and derivatives	Organic sulfuric acids and derivatives	Arylsulfates	*		37.7	0	M-H	C10H12O7S	0.888782429	788.9589872	896.8166894	870.4651069	261.3379434	273.9789503	259.3921558	701.7997575	858.3353717	542.2418308
4.95_513.0655m/z	513.0655024	4.94705	neg	Cromoglicic acid	HMDB0015138	Organoheterocyclic compounds	Benzopyrans	1-benzopyrans		C06928	37.7	0	M+FA-H	C23H16O11	-4.19119794	122.6350983	0.001403199	0.001403199	5264.588251	5667.852521	5625.871074	0.001403199	0.001403199	0.001403199
4.99_427.1076m/z	427.1076381	4.99225	neg	1-Methyl-3-(2-thiazolyl)-1H-indole	HMDB0034965	Organoheterocyclic compounds	Indoles and derivatives	N-alkylindoles			37.7	0	2M-H	C12H10N2S	4.61525396	1520.177555	1637.838278	1784.284998	4967.581011	4966.901911	5106.139118	590.7616263	473.2582591	442.771589

5.01_359.0968m/z	359.0967526	5.014716667	neg	2-Hydroxyphenylacetic acid O-b-D-glucoside	93053	Organic oxygen compounds	Organooxygen compounds	Carbohydrates and carbohydrate conjugates			37.7	0	M+FA-H	C14H18O8	-5.150661314	257.2181282	329.6304747	436.3237082	702.876584	852.4050735	851.3847541	771.9719811	638.3690338	851.4221136
5.06_242.1401m/z	242.1400893	5.05975	neg	hydroxyisovaleroyl carnitine	LMFA07070073	Lipids and lipid-like molecules	Fatty Acyls	Fatty acid esters	*		37.7	0	M-H2O-H	C12H23NO5	1.177893606	336.9417407	358.5865111	299.6213799	537.3486676	428.5367675	412.1683661	310.0879747	347.7017656	215.0600372
5.06_305.1513m/z	305.1512653	5.05975	neg	Octopamine (p-Hydroxyphenylethanolamine)	61	Unclassified	Unclassified	Unclassified			37.7	0	2M-H	C8H11NO2	1.909157869	572.4941404	568.6065358	694.1004585	528.8817082	492.1334671	514.5853075	580.1234599	688.575186	730.2631705
5.08_148.0617m/z	148.0616536	5.080566667	neg	4-amino-butanoic acid	LMFA01100039	Lipids and lipid-like molecules	Fatty Acyls	Fatty Acids and Conjugates	**	C00334	37.7	0	M+FA-H	C4H9NO2	1.185732939	1453.958391	1313.862912	1240.172765	1325.727738	1396.46053	1297.570271	1656.183188	1357.103655	1474.143766
5.10_393.0810m/z	393.080962	5.1031	neg	alpha-(1,2-Dihydroxyethyl)-1,2,3,4-tetrahydro-7-hydroxy-9-methoxy-3,4-dioxocyc	73253	Phenylpropanoids and polyketides	Coumarins and derivatives	Unclassified	**	C19592	37.7	0	M+FA-H	C17H16O8	-5.05136628	2262.383803	1824.031849	1871.559834	1664.560029	1998.628491	2408.444642	4138.667268	3584.788519	4326.39895
5.10_447.1275m/z	447.1274936	5.1031	neg	Sinapic acid	HMDB0032616	Phenylpropanoids and polyketides	Cinnamic acids and derivatives	Hydroxycinnamic acids and derivatives	***	C00482	37.7	0	2M-H	C11H12O5	-4.857635697	1972.227474	2179.558305	1999.495543	3670.497301	4221.027677	4531.019968	3157.033734	2119.382098	2661.277571
5.13_479.2289m/z	479.2288582	5.132616667	neg	Glutathionylspermidine	63639	Unclassified	Unclassified	Unclassified			37.7	0	M+FA-H	C17H34N6O5S	-1.114342218	2015.542417	2053.293283	1449.35133	1925.914503	1476.496506	1856.326607	1518.144894	1482.922695	1908.34938
5.16_158.0612m/z	158.0612314	5.155116667	neg	6-Methoxyquinoline	34519	Unclassified	Unclassified	Unclassified			37.7	0	M-H	C10H9NO	0.590355186	405.7384022	399.9947693	241.6106266	663.4383306	360.5539373	462.3975576	503.4319018	393.8099565	711.022507
5.16_265.1060m/z	265.1059973	5.155116667	neg	Threoninyl-Threonine	85964	Organic acids and derivatives	Carboxylic acids and derivatives	Amino acids, peptides, and analogues	*		37.7	0	M+FA-H	C8H16N2O5	8.508614016	3257.000329	3459.080851	3462.297652	5962.610794	5474.040212	5445.025795	3654.463799	3757.207489	3953.330984
5.16_326.1516n	325.1382866	5.155116667	neg	5,5-Diisopropyl-2,2'-dimethylbiphenyl-3,3',4,4'-tetrone	HMDB0040762	Lipids and lipid-like molecules	Prenol lipids	Monoterpenoids	*		37.7	0.603	M-H2O-H, M-H	C20H22O4	-0.714256602	2835.721611	3609.494075	3159.701641	5011.822532	5095.215554	5229.459692	3157.951995	3023.955936	3113.814714
5.18_367.2128m/z	367.2127507	5.1777	neg	4,5-Dihydro-5,5-dimethyl-4-(3-oxobutyl)furan-2(3H)-one	64050	Unclassified	Unclassified	Unclassified			37.7	0	2M-H	C10H16O3	0.375815701	108.2033401	112.0313406	196.3196907	132.0277665	57.91100662	61.05346828	61.55372483	161.286705	167.7206495
5.22_317.1144m/z	317.1144101	5.222166667	neg	(2S,4R)-4-(9H-Pyrido[3,4-b]indol-1-yl)-1,2,4-butanetriol	90512	Alkaloids and derivatives	Harmala alkaloids	Unclassified			37.7	0	M+FA-H	C15H16N2O3	0.422064459	1407.551478	1546.163632	1297.120911	1197.66226	1289.326704	1281.270596	1267.654757	1485.035118	1557.262807
5.22_413.2183m/z	413.2183196	5.222166667	neg	20-hydroxylipoxin A4	HMDB0062748	Lipids and lipid-like molecules	Fatty Acyls	Eicosanoids	*		37.7	0	M+FA-H	C20H32O6	0.619250758	3231.171845	2747.388573	2957.112031	1185.90913	898.4610026	814.3955777	2095.355242	1752.162826	1853.323395
5.32_483.1047m/z	483.1047063	5.320233333	neg	ETHOPROP	44576	Unclassified	Unclassified	Unclassified			37.7	0	2M-H	C8H19O2PS2	-1.723151851	347.035491	427.2812024	291.870531	1100.286093	1773.516873	1711.727379	516.1776168	535.1122601	872.5469278
5.36_265.1058m/z	265.1058021	5.361783333	neg	N-Acetyl-beta-D-glucosaminylamine	65570	Organic oxygen compounds	Organooxygen compounds	Carbohydrates and carbohydrate conjugates	*	C01239	37.7	0	M+FA-H	C8H16N2O5	7.62203086	15334.03828	18375.5704	17708.11689	14514.69998	15011.80543	13840.60501	11010.56638	13080.02283	12106.70034
5.43_705.1818m/z	705.181849	5.428733333	neg	4-Hydroxybenzyl isothiocyanate 4''-acetylrhamnoside	88724	Unclassified	Unclassified	Unclassified			37.7	0	2M-H	C16H19NO6S	3.55249191	1905.562016	1938.934807	1730.536992	3230.523698	1999.225857	1987.490814	1981.000853	2015.679016	1123.961506
5.45_190.0875m/z	190.0874601	5.451166667	neg	2,4-Dimethyl-1H-indole	88866	Organoheterocyclic compounds	Indoles and derivatives	Indoles			37.7	0	M+FA-H	C10H11N	0.743420327	370.1034315	340.8826468	365.640349	120.5068852	117.5722868	92.93966762	285.8538895	269.2605343	212.9186391
5.47_379.1493m/z	379.1492584	5.471616667	neg	Aldicarb	HMDB0248124	Unclassified	Unclassified	Unclassified			37.7	0	2M-H	C7H14N2O2S	3.518242797	557.3643795	345.4363693	504.4902335	725.5159507	702.1868425	701.9381681	567.2742831	317.8033161	294.6817178
5.47_381.1381m/z	381.1381478	5.471616667	neg	Steroid O-sulfate	65795	Unclassified	Unclassified	Unclassified			37.7	0	M+FA-H	C18H24O4S	1.233613701	5003.662821	4902.490801	5075.419525	4555.865178	5070.693702	5335.271019	8259.776792	6787.548725	7341.537545
5.50_282.1349m/z	282.134869	5.497816667	neg	L-Tyrosine n-butyl ester	3540	Unclassified	Unclassified	Unclassified			37.7	0	M+FA-H	C13H19NO3	0.727948452	256.2437347	123.9676154	197.3416397	339.8896943	465.3172939	380.9969312	186.77231	146.9173832	114.2935721
5.53_439.0334m/z	439.0334326	5.525416667	neg	4-Carboxy-4-hydroxy-2-oxoadipate	66119	Unclassified	Unclassified	Unclassified			37.7	0	2M-H	C7H8O8	-7.102538419	1534.718148	1218.59571	1066.883709	178.0687556	187.7724065	98.25527359	733.2087398	445.2841228	869.8425995
5.60_383.0844m/z	383.0844079	5.602	neg	6-(Allylthio)purine	70821	Unclassified	Unclassified	Unclassified			37.7	0	2M-H	C8H8N4S	-5.858613606	4037.024492	2752.238957	3821.991109	3670.737839	3650.081636	2712.395589	4588.637669	4512.015041	3346.990063
5.61_344.1153m/z	344.1152797	5.610733333	neg	N-[(diphenylmethoxy)acetyl]-Glycine	2320	Unclassified	Unclassified	Unclassified			37.7	0	M+FA-H	C17H17NO4	4.409124934	2210.095464	1989.345158	1991.41777	3746.009094	3203.145464	3374.666302	3046.443468	3220.114788	2971.197494
5.65_307.1164m/z	307.1164415	5.650833333	neg	3,4-Dihydroxyphenyl ethanol	58314	Unclassified	Unclassified	Unclassified			37.7	0	2M-H	C8H10O3	-7.36842437	604.8507357	631.3429923	812.8850554	794.3015445	718.5652462	696.2398596	702.1289389	573.3372707	538.1763807
5.67_376.2101n	357.1922803	5.666566667	neg	2,3-di-0-hexanoyl-alpha-glucopyranose	53893	Unclassified	Unclassified	Unclassified			37.7	0	M-H2O-H, M-H	C18H32O8	1.072476521	1617.190634	1602.076718	2094.140872	3588.490995	3367.056459	3465.163575	1162.339341	1291.854774	1533.780564
5.67_423.2027m/z	423.2027204	5.666566667	neg	Isohumulinone A	HMDB0030045	Lipids and lipid-like molecules	Prenol lipids	Terpene glycosides	*		37.7	0	M+FA-H	C21H30O6	0.73726082	1645.373002	1728.68451	2066.779848	1971.724881	2098.093635	1705.871605	2070.702223	1872.47731	1982.753023
5.69_231.1239m/z	231.1238926	5.6875	neg	(1R,2R,4R)-1,8-Epoxy-p-menthane-2,4-diol	95928	Lipids and lipid-like molecules	Prenol lipids	Monoterpenoids	*		37.7	0	M+FA-H	C10H18O3	0.512132024	710.4531833	674.1259462	679.1067997	602.2104311	602.1200557	550.9529556	607.7370174	675.6454303	776.4866884
5.71_479.1538m/z	479.1538476	5.709933333	neg	Lettucenin A	91765	Organoheterocyclic compounds	Cycloheptafurans	Unclassified	*		37.7	0	2M-H	C15H12O3	7.988025498	4795.297849	4397.173804	3862.300482	3734.676945	4706.439062	4266.920392	3244.886691	3368.441471	3217.659859
5.71_665.1964m/z	665.1963539	5.709933333	neg	Dihydrosanguinarine	64356	Organoheterocyclic compounds	Quinolines and derivatives	Benzoquinolines	**	C05191	37.7	0	2M-H	C20H15NO4	5.125120394	5143.840251	4209.258902	3982.221762	4338.34305	6295.990882	4605.636205	3888.250539	5200.184407	5741.661081
5.73_337.1383m/z	337.1382774	5.727283333	neg	HYCANTHONE	43996	Lipids and lipid-like molecules	Steroids and steroid derivatives	Physalins and derivatives	*		37.7	0	M-H2O-H	C20H24N2O2S	0.756665623	3359.992053	4032.867198	2700.029984	6296.173792	9004.739517	7333.340835	4380.187069	5249.484713	5493.33494
5.74_381.0287m/z	381.028668	5.744566667	neg	hesperetin 3'-O-sulfate	86085	Phenylpropanoids and polyketides	Flavonoids	O-methylated flavonoids	*		37.7	0	M-H	C16H14O9S	0.239225752	160.4780643	310.470002	359.7770207	1358.391226	1841.93752	1529.268169	0.001403199	66.20752174	59.78722605
5.79_245.0380m/z	245.0379824	5.785316667	neg	2',2'-Difluorodeoxyuridine	HMDB0060727	Nucleosides, nucleotides, and analogues	Pyrimidine nucleosides	Pyrimidine 2'-deoxyribonucleosides	*		37.7	0	M-H2O-H	C9H10F2N2O5	0.17284992	3259.794248	3482.995064	3266.521803	1735.567816	2058.39866	1729.648577	3990.822449	4421.790637	3769.862796
5.83_425.1820m/z	425.1819887	5.825	neg	Deacetylisovaltrate	HMDB0035840	Lipids and lipid-like molecules	Prenol lipids	Monoterpenoids	*		37.7	0	M+FA-H	C20H28O7	0.743439195	3377.547736	3287.017619	3841.536398	3422.727718	2461.846721	2523.477292	5854.830093	5575.581796	5530.071499
5.87_299.0960m/z	299.0959892	5.866533333	neg	D-Ribose	313	Organic oxygen compounds	Organooxygen compounds	Carbohydrates and carbohydrate conjugates	**	C00121	37.7	0	2M-H	C5H10O5	-7.934470282	2826.913858	3135.378824	3136.288629	1321.551652	1109.522701	1180.379116	3884.933549	3006.553205	3122.121213
5.98_381.1065m/z	381.1065309	5.9829	neg	1H-Indole-4-acetic acid, 2,3-dihydro-2-oxo-	2373	Unclassified	Unclassified	Unclassified			37.7	0	2M-H	C10H9NO3	-7.010797872	502.1813805	638.6917721	691.5037966	752.255391	684.6308165	1066.326166	439.4977936	631.088665	716.5474046
5.99_266.1011m/z	266.1011033	5.9916	neg	1-Guanidino-1-deoxy-scyllo-inositol	64555	Unclassified	Unclassified	Unclassified			37.7	0	M+FA-H	C7H15N3O5	7.823841465	9103.933179	9319.488298	10033.12	14556.16599	15103.33523	16389.89008	11280.06175	11783.16829	10966.94298
6.01_607.2378m/z	607.2378083	6.007266667	neg	Eriocarpin	67232	Unclassified	Unclassified	Unclassified			37.7	0	M+FA-H	C29H38O11	-3.213260495	46038.28987	46088.18915	49276.8691	46610.58657	45496.75381	46396.87512	38958.49529	39300.84914	37461.83521
6.07_233.0796m/z	233.0796064	6.07	neg	Threoninyl-Aspartate	85950	Organic acids and derivatives	Carboxylic acids and derivatives	Amino acids, peptides, and analogues	*		37.7	0	M-H	C8H14N2O6	7.24801957	4412.939578	4897.602135	5042.887646	6476.751518	6747.763281	6526.839833	4048.632948	4508.853894	4254.721635
6.07_445.1251m/z	445.1250777	6.07	neg	Carbamazepine N-Glucuronide	1491	Unclassified	Unclassified	Unclassified			37.7	0	M+FA-H	C20H20N2O7	-0.440240804	1275.922076	1523.160725	1156.484213	784.5935998	1079.199602	872.0418341	1394.805567	1536.078656	1249.098754
6.10_261.0745m/z	261.0745081	6.101316667	neg	Na-L-Glutamyl-L-aspartic acid	86956	Unclassified	Unclassified	Unclassified			37.7	0	M-H	C9H14N2O7	6.424534215	1420.698791	2077.505639	1986.051707	3251.568232	3704.071486	2994.364266	2353.509116	2656.174018	2953.952444
6.10_531.1511m/z	531.1511498	6.101316667	neg	Castavinol	86515	Phenylpropanoids and polyketides	Flavonoids	Flavans	*		37.7	0	M-H2O-H	C26H30O13	0.636022464	6438.955945	7686.01045	7135.675377	5275.92632	6547.282452	5878.124786	398.1279234	390.840768	512.3525807
6.23_455.1537m/z	455.1537495	6.230633333	neg	Benzyl salicylate	86523	Benzenoids	Benzene and substituted derivatives	Benzoic acids and derivatives	*		37.7	0	2M-H	C14H12O3	8.193390887	4057.662743	2719.884852	4708.572244	5031.151808	4223.152572	5505.527217	2817.778109	2798.652001	2905.779436
6.33_343.2130m/z	343.2130315	6.331216667	neg	6-propionyl n-caproic acid	LMFA01060026	Lipids and lipid-like molecules	Fatty Acyls	Fatty Acids and Conjugates			37.7	0	2M-H	C9H16O3	1.217682297	424.1819645	594.1421249	588.4265407	530.6241067	621.8734831	510.258309	354.8938852	259.807848	400.6454372
6.46_475.2682m/z	475.2681723	6.458366667	neg	15(R)-17-phenyl trinor PGF2α isopropyl ester	45718	Unclassified	Unclassified	Unclassified			37.7	0	M+FA-H	C26H38O5	-4.54343015	7063.768266	6489.032157	7750.870595	3445.554509	3206.097788	3207.845594	7326.341398	7337.902007	7264.014636
6.48_591.1654m/z	591.1653982	6.477433333	neg	Penicillamine disulfide	HMDB0246004	Unclassified	Unclassified	Unclassified			37.7	0	2M-H	C10H20N2O4S2	-0.377693845	2335.091913	2374.43375	2391.798712	3115.083849	2698.975192	1868.961137	4977.362133	5303.830128	5593.574144
6.51_247.0953m/z	247.0952965	6.509966667	neg	2,3-Dihydroxytoluene	65862	Unclassified	Unclassified	Unclassified			37.7	0	2M-H	C7H8O2	-9.214283621	3098.387782	3612.987038	3552.580553	6108.693626	5955.205482	6212.139276	2864.097613	3232.982616	3033.561803
6.52_624.3394m/z	624.339434	6.520516667	neg	(3a,5b,7b)-24-[(carboxymethyl)amino]-7-hydroxy-24-oxocholan-3-yl-b-D-glucop	6724	Unclassified	Unclassified	Unclassified			37.7	0	M-H	C32H51NO11	0.797957658	9029.773429	9693.585664	9730.969833	2891.192294	3228.963325	2598.159447	7714.884452	9191.338928	9508.137624
6.53_383.1536m/z	383.1535592	6.531066667	neg	(R)-Shinanolone	87075	Benzenoids	Tetralins	Unclassified	*		37.7	0	2M-H	C11H12O3	9.233622457	1304.170376	1279.552643	1189.246875	1018.091393	1284.815709	1235.077974	1349.817627	1477.507407	1586.238704
6.53_615.1337m/z	615.1336879	6.531066667	neg	Neodulin	48125	Lipids and lipid-like molecules	Polyketides	Flavonoids			37.7	0	2M-H	C18H12O5	6.520176485	6620.107774	7365.876019	7941.08398	12585.42428	13161.15023	12491.59167	4368.002564	4623.833617	3920.1384
6.57_640.3346m/z	640.3345955	6.570233333	neg	(3a,5b,7a,12a)-24-[(carboxymethyl)amino]-1,12-dihydroxy-24-oxocholan-3-yl-b-l	HMDB0002472	Organic nitrogen compounds	Organonitrogen compounds	Oximes	*		37.7	0	M-H	C32H51NO12	1.162900039	3721.677215	4099.487068	3742.594436	300.6876987	444.9289101	826.943698	1277.892289	1345.669604	1528.315741
6.59_643.3704m/z	643.3704356	6.592933333	neg	Kurilensoside G	84936	Lipids and lipid-like molecules	Sterol Lipids	Steroid conjugates			37.7	0	M+FA-H	C32H54O10	0.893782951	1769.352359	2232.984072	2516.131954	2772.807597	2916.594979	2418.044007	2263.413283	2479.16014	2283.480227
6.66_277.1058m/z	277.1058171	6.663683333	neg	Hydroxyprolyl-Threonine	85779	Organic acids and derivatives	Carboxylic acids and derivatives	Amino acids, peptides, and analogues	*		37.7	0	M+FA-H	C9H16N2O5	7.292351091	2950.651144	3359.117201	3267.195233	2014.763398	1721.48889	1600.913662	935.053918	1430.049156	1229.728606
6.68_615.1332m/z	615.1331852	6.680966667	neg	3,5-Dicaffeoyl-4-succinoylquinic acid	HMDB0029903	Organic acids and derivatives	Carboxylic acids and derivatives	Pentacarboxylic acids and derivatives	*		37.7	0	M-H	C29H28O15	-3.827916587	15845.21814	17854.77797	18663.20693	17953.24011	16517.87957	14925.84059	10495.10456	13654.17288	13863.03036
6.69_421.0603m/z	421.0602718	6.69475	neg	1-Methyl 2-galloylgalactarate	91922	Organic oxygen compounds	Organooxygen compounds	Carbohydrates and carbohydrate conjugates			37.7	0	M+FA-H	C14H16O12	-5.602940506	1249508.747	1394392.061	1353927.74	1467918.863	1453157.8	1396173.755	219772.7802	265174.6615	255623.0122
6.82_455.1539m/z	455.1539409	6.817666667	neg	Resveratrol	HMDB0003747	Phenylpropanoids and polyketides	Stilbenes	Unclassified	***	C03582	37.7	0	2M-H	C14H12O3	8.612940912	5701.73259	6583.284559	5936.944425	11099.97766	10424.61665	11754.24996	4869.78799	4905.058851	4648.82201
6.90_459.2241m/z	459.2241042	6.896766667	neg	Distigmine	2344	Unclassified	Unclassified	Unclassified			37.7	0	M+FA-H	C22H30N4O4	-1.941309243	12178.24437	12347.71866	11135.4026	15514.52142	14719.47089	13475.42197	13344.01913	15617.59002	14539.64611
7.04_380.1177m/z	380.1177051	7.044716667	neg	Niaziminin	HMDB0031945	Organic oxygen compounds	Organooxygen compounds	Carbohydrates and carbohydrate conjugates	*		37.7	0	M-H2O-H	C18H25NO7S	0.934639856	9750.432008	11424.58904	10090.45237	2545.119235	2306.075388	2372.163613	4601.38101	5110.140536	4501.854688
7.09_181.1235m/z	181.1235321	7.086816667	neg	(-)-α-Pinene	64044	Lipids and lipid-like molecules	Prenol lipids	Monoterpenoids	**	C06308	37.7	0	M+FA-H	C10H16	0.945509319	505.2926647	550.9725818	437.1045077	706.9582101	766.2612095	627.9757277	392.141453	371.594828	353.3269111
7.13_385.1486m/z	385.148618	7.1264	neg	Fortimicin FU-10	71967	Unclassified	Unclassified	Unclassified			37.7	0	M+FA-H	C12H24N2O9	6.56990733	5083.092181	5054.592225	5016.320799	4258.639113	5060.81423	4313.995575	4094.065649	4311.288779	4239.398059
7.18_373.1329m/z	373.1328798	7.178516667	neg	7-hydroxyolanzapine	HMDB0060958	Benzenoids	Phenol ethers	Unclassified	*		37.7	0	M+FA-H	C17H20N4OS	-3.368458889	64965.82342	73026.74573	70960.87217	23486.30094	23384.55986	23428.41674	44178.67574	53305.36119	49957.64278
7.21_405.2648m/z	405.264844	7.211683333	neg	O-Arachidonoyl Glycidol	44872	Unclassified	Unclassified	Unclassified			37.7	0	M+FA-H	C23H36O3	0.544227211	2554.467142	3000.728079	2719.288885	1458.443683	943.16801	1290.602634	3093.680877	3698.793563	3350.549573
7.23_485.2402m/z	485.2402177	7.225666667	neg	3''-HydroxyPravastatin	2054	Unclassified	Unclassified	Unclassified			37.7	0	M+FA-H	C23H36O8	2.264114589	3692.005397	4458.46073	4059.168584	2736.537191	2770.862532	2761.855462	2469.88353	3030.572067	3115.108084
7.30_685.0936m/z	685.093607	7.29525	neg	O-Desmethyltolrestat	2886	Unclassified	Unclassified	Unclassified			37.7	0	2M-H	C15H12F3NO3S	4.205775654	1710.463152	2406.697414	2500.94417	3366.625406	3725.742057	3047.49867	1514.381879	2626.774419	2064.589688
7.53_465.1381m/z	465.1380575	7.527033333	neg	Melampodin A	67753	Unclassified	Unclassified	Unclassified			37.7	0	M+FA-H	C21H24O9	-5.18330346	22921.67771	25253.39613	24416.54378	43466.70514	38711.57917	37867.08409	30722.90737	33036.24093	33973.18418
7.70_455.1908m/z	455.1908166	7.699916667	neg	Depdecin	LMFA05000013	Lipids and lipid-like molecules	Fatty Acyls	Fatty alcohols			37.7	0	2M-H	C11H16O5	-3.187550425	2223.088165	2467.727664	2339.549462	2638.137418	3132.324211	2443.269524	1024.397159	1397.067965	1414.107866
7.75_371.2441m/z	371.244096	7.747083333	neg	(1beta,2beta,5beta)-p-Menth-3-ene-1,2,5-triol	93586	Lipids and lipid-like molecules	Prenol lipids	Monoterpenoids	*		37.7	0	2M-H	C10H18O3	0.493140769	881.8866855	703.212201	1083.633051	1924.206801	1949.486825	1926.414843	299.1908137	651.9908478	713.4395596
7.92_669.2125m/z	669.2124904	7.91555	neg	4-O-beta-D-Galactopyranosyl-D-xylose	93493	Organic oxygen compounds	Organooxygen compounds	Carbohydrates and carbohydrate conjugates			37.7	0	M+FA-H	C22H40O20	4.79619943	33339.70585	33507.72839	36223.25393	43008.07365	46520.90498	46967.41642	33409.20284	44170.36152	40512.90153
8.13_577.2636m/z	577.2635722	8.129733333	neg	Calotropin	3590	Unclassified	Unclassified	Unclassified			37.7	0	M+FA-H	C29H40O9	-3.501044372	13600.15266	12783.23472	13372.80357	19295.87848	18975.96073	18754.40033	12237.41939	13898.91397	13111.40988
8.82_471.2430m/z	471.2430413	8.8245	neg	3-Sulfodeoxycholic acid	LMST05020038	Lipids and lipid-like molecules	Sphingolipids	Ceramides	*		37.7	1.8	M-H	C24H40O7S	1.78520799	26100.14636	32389.50142	29429.37297	4846.635515	4347.531226	4355.528035	14174.95172	16168.86725	15286.02049
9.02_815.2057m/z	815.2057344	9.01635	neg	3-(a-Naphthoxy)lactic acid glucuronide	2173	Unclassified	Unclassified	Unclassified			37.7	0	2M-H	C19H20O10	2.103808579	10108.12885	14619.66581	13293.40466	0.001403199	198.1942642	186.4332474	4457.138598	4350.226204	3779.481196
9.36_209.1548m/z	209.154761	9.36055	neg	Neryl propionate	92983	Lipids and lipid-like molecules	Fatty Acyls	Fatty alcohol esters	*		37.7	0	M-H	C13H22O2	0.273415257	187.2162947	411.5715365	314.4793587	574.0265445	0.001403199	76.78103419	209.2257581	245.1344389	127.9758982
9.75_350.2469m/z	350.2469493	9.75225	neg	Dodecylphosphocholine	34530	Unclassified	Unclassified	Unclassified			37.7	0	M-H	C17H38NO4P	1.083011198	1525.994373	1417.156071	1379.893063	1460.006271	1305.077143	1512.380593	1809.225133	1620.100775	1435.792346
0.53_351.2521m/z	351.2521301	0.529533333	neg	N-Methylnicotinium	1533	Organoheterocyclic compounds	Pyridines and derivatives	Pyrrolidinylpyridines	*		37.6	0	2M-H	C11H16N2	-9.3410739	160.0485026	211.3032391	25.94399352	251.3210813	197.534126	118.7970442	324.8972322	175.7555856	288.3879407
0.67_463.0413m/z	463.0412696	0.6701	neg	6-Cyano-7-nitroquinoxaline-2,3-dione	69622	Unclassified	Unclassified	Unclassified			37.6	0	2M-H	C9H4N4O4	4.389083881	190.9806455	331.5458086	237.597152	743.4941138	1113.062622	837.5498152	612.8046212	443.9270172	810.8276749
0.74_354.9813m/z	354.9812673	0.7359	neg	Ribose 1,5-bisphosphate	62426	Organic oxygen compounds	Organooxygen compounds	Carbohydrates and carbohydrate conjugates	**	C01151	37.6	0	M+FA-H	C5H12O11P2	-7.806910513	16.36939203	0.001403199	1406.974588	268.1098797	1646.157775	400.6436646	217.6083907	1969.701283	1877.586707
0.80_347.0751m/z	347.0751	0.800366667	neg	Quinoxaline-2-carboxylic acid	72418	Unclassified	Unclassified	Unclassified			37.6	0	2M-H	C9H6N2O2	-9.993120433	33929.52883	33990.06047	34301.56685	27109.25415	25014.20576	23523.68462	33185.53005	36147.67432	37762.0943
0.80_349.0720m/z	349.072006	0.800366667	neg	BIOCHANIN A DIACETATE	43569	Unclassified	Unclassified	Unclassified			37.6	0	M-H2O-H	C20H16O7	0.663597102	12577.69155	14094.06817	13545.85944	7306.274265	9935.587143	9486.493836	12488.7196	11699.73627	11347.74765
0.80_352.0425n	351.0376913	0.800366667	neg	4-[2-(5-Carboxy-2-hydroxy-3-methoxyphenyl)-2-oxoethylidene]-2-hydroxy-2-pent	72169	Unclassified	Unclassified	Unclassified			37.6	0	M-H2O-H, M-H	C15H12O10	-1.41306667	16779.61572	16612.0457	17305.27355	34927.39313	39262.7715	31723.02461	25866.91784	22184.2243	24222.00719
0.82_176.0387m/z	176.0386677	0.819566667	neg	N-Formylmethionine	4233	Organic acids and derivatives	Carboxylic acids and derivatives	Amino acids, peptides, and analogues	**	C03145	37.6	0	M-H	C6H11NO3S	-0.113308872	1263.887844	1741.374005	1797.3543	877.6160876	780.9124384	822.4267299	2171.989493	2486.254768	2896.50282
0.82_258.0564m/z	258.0563771	0.819566667	neg	PPM-18	64815	Unclassified	Unclassified	Unclassified			37.6	0	M-H2O-H	C17H11NO3	1.173050744	16139.70328	17081.20241	20088.00038	16002.84621	15963.86679	14976.22546	16530.41799	18175.18755	18587.93844
0.82_315.0935m/z	315.093522	0.819566667	neg	6-Hydroxyl-1,6-dihydropurine ribonucleoside	66214	Unclassified	Unclassified	Unclassified			37.6	0	M+FA-H	C10H14N4O5	-4.074291593	3689.636458	4226.061911	6500.41705	3019.750986	2072.700482	3092.594245	3367.614505	3484.646866	5032.555388
0.84_168.0634n	149.0455424	0.841733333	neg	N-Cyclopropylammelide	69836	Unclassified	Unclassified	Unclassified			37.6	0	M-H2O-H, M-H	C6H8N4O2	-7.985079457	67470.32189	71393.97936	83498.72732	69847.12542	68673.95018	70218.02464	64753.94202	75986.81867	72294.7049
0.84_307.0481m/z	307.0481459	0.841733333	neg	Sinapoyltartronate	65825	Unclassified	Unclassified	Unclassified			37.6	0	M-H2O-H	C14H14O9	6.762503223	4748.694111	6506.416205	5602.015063	3469.026672	4027.824941	2742.628488	5622.536	5180.105755	5445.231191
0.88_172.0253m/z	172.0252603	0.884633333	neg	gamma-carboxyglutamic acid	96169	Organic acids and derivatives	Carboxylic acids and derivatives	Amino acids, peptides, and analogues	*		37.6	0	M-H2O-H	C6H9NO6	0.599063893	280.8382283	335.1105632	314.8068935	522.3478007	259.0346854	625.2373429	298.4932136	465.0399217	307.9404672
0.88_190.0181m/z	190.0181216	0.884633333	neg	3,4-Dehydrothiomorpholine-3-carboxylate	66183	Unclassified	Unclassified	Unclassified			37.6	0	M+FA-H	C5H7NO2S	1.166950168	5366.884726	5945.738262	6322.995521	7589.981767	6627.389499	7225.851381	6439.108081	7808.782478	5898.498664
0.88_323.0380m/z	323.0379756	0.884633333	neg	(2R,2'R)-3,3'-disulfanediylbis(2-acetamidopropanoic acid)	65468	Unclassified	Unclassified	Unclassified			37.6	0	M-H	C10H16N2O6S2	0.844948974	5016.689541	4615.327298	2457.752209	3366.242183	2253.261754	3821.36571	2739.770572	2362.616007	2185.20046
0.90_197.0681m/z	197.0680724	0.904683333	neg	L-Arabitol	141	Organic oxygen compounds	Organooxygen compounds	Carbohydrates and carbohydrate conjugates	**	C00532	37.6	0	M+FA-H	C5H12O5	9.180178866	31025.66778	33630.57655	34161.44766	36821.88683	39403.44026	36831.54966	34798.03087	36369.62568	37636.65743
1.16_256.0941m/z	256.0941198	1.160166667	neg	N-Acetyl-1-methylhistidine	HMDB0240340	Organic acids and derivatives	Carboxylic acids and derivatives	Amino acids, peptides, and analogues			37.6	0	M+FA-H	C9H13N3O3	1.068925621	703.9952823	978.2069248	1202.300125	902.9554723	323.0483621	857.6462594	602.9574796	887.7733489	532.9456212
1.16_340.0674m/z	340.0674386	1.160166667	neg	Triglochinin	66921	Organic oxygen compounds	Organooxygen compounds	Carbohydrates and carbohydrate conjugates			37.6	0	M-H2O-H	C14H17NO10	0.094644633	5520.17684	6976.169454	6661.348413	0.001403199	754.7137974	0.001403199	6828.653145	5869.039837	6648.661294
1.22_221.0062m/z	221.0062088	1.224233333	neg	Cysteine Dimer	5006	Unclassified	Unclassified	Unclassified			37.6	0	M-H2O-H	C6H12N2O4S2	0.837780267	2311.980202	2680.516623	3047.765318	3189.386481	3135.950303	3242.345934	2654.009359	3095.818692	2878.234381
1.42_276.0177m/z	276.0177387	1.421683333	neg	N'-Phosphoguanidinoethyl methyl phosphate	66196	Unclassified	Unclassified	Unclassified			37.6	0	M-H	C4H13N3O7P2	7.732633807	1939.892504	1616.920505	1326.604737	3129.481859	2738.432529	2532.530662	2267.373192	1847.935198	1965.20492
1.55_229.0695m/z	229.0695343	1.553483333	neg	3-keto-2-methylbutyrate	86063	Organic acids and derivatives	Keto acids and derivatives	Short-chain keto acids and derivatives	*		37.6	0	2M-H	C5H7O3-	-9.680988135	610.1398401	514.505151	450.5413009	1762.814848	1408.922874	1669.681778	610.4794101	1225.108902	703.7076477
1.55_967.3133m/z	967.3132991	1.553483333	neg	7-Methyl-1,4,5-naphthalenetriol 4-[xylosyl-(1->6)-glucoside]	HMDB0034909	Organic oxygen compounds	Organooxygen compounds	Carbohydrates and carbohydrate conjugates			37.6	0	2M-H	C22H28O12	4.567636551	5331.259284	4320.100688	2794.546214	3007.878549	2390.83203	2691.956127	2185.584424	5035.09335	4563.975147
1.59_145.0701m/z	145.0700813	1.5934	neg	S-Methyl hexanethioate	94037	Lipids and lipid-like molecules	Fatty Acyls	Fatty acyl thioesters	*		37.6	0	M-H	C7H14OS	5.625158444	1827.639182	1568.951062	1617.482996	1568.573177	1605.365801	1562.46742	1579.388624	1722.966213	1822.437687
1.99_234.9919m/z	234.9919245	1.9892	neg	3-Sulfocatechol	66375	Unclassified	Unclassified	Unclassified			37.6	0	M+FA-H	C6H6O5S	0.670311707	1966.075967	1702.310963	1915.840586	201.8853878	187.8068847	135.6347138	1556.355466	1605.981626	1554.078564
10.46_653.2174m/z	653.2174042	10.45966667	neg	Benorilate	1261	Unclassified	Unclassified	Unclassified			37.6	0	2M-H	C18H17NO5	5.098212042	8873.228248	9209.787328	10222.77203	9220.421107	9884.219258	8346.738313	10275.62525	10851.10982	9546.520734
12.49_239.0600m/z	239.0599804	12.48888333	neg	AG-370	45281	Unclassified	Unclassified	Unclassified			37.6	0	M-H2O-H	C15H8N5	-0.576461513	3198.372973	2006.574008	2160.702946	3387.313067	2403.485428	1984.552243	2402.251079	2155.209261	2120.395045
14.65_563.0997m/z	563.0997042	14.6516	neg	Mutisifurocoumarin	48352	Unclassified	Unclassified	Unclassified			37.6	0	2M-H	C16H10O5	2.364365709	1514.294202	3720.254366	3194.537083	2741.238072	3613.974022	2891.718386	3524.831912	2970.725346	3235.844033
2.07_403.0712m/z	403.0712452	2.070366667	neg	AG-82	45247	Unclassified	Unclassified	Unclassified			37.6	0	2M-H	C10H6N2O3	7.022328316	1961.700654	2500.380741	2581.803775	4411.640957	5483.814221	5290.100213	3254.522103	3049.971271	3254.504929
2.86_253.0101m/z	253.0100823	2.864816667	neg	Imidazole citrate	HMDB0253417	Organoheterocyclic compounds	Azoles	Imidazoles		C01589	37.6	0	M-H	C9H6N2O7	-0.558419662	2266.21669	1998.954971	2499.273974	2562.849758	2726.350086	2563.211758	2230.432502	2429.009003	1883.60479
3.65_541.1179m/z	541.1179311	3.64965	neg	Luteolinidin	86126	Phenylpropanoids and polyketides	Flavonoids	Hydroxyflavonoids	*	C08652	37.6	0	2M-H	C15H11O5+	7.213574222	1238.829335	1362.441307	1113.294095	5089.792958	5705.760372	4880.876913	1977.729639	2074.933572	2259.533278
3.75_261.0414m/z	261.0413775	3.747833333	neg	Maclurin	HMDB0032644	Benzenoids	Benzene and substituted derivatives	Benzophenones	*	C09951	37.6	0.336	M-H	C13H10O6	3.495098681	2273.681417	2007.129301	2048.804119	4365.33748	4308.933032	2571.960482	2608.7909	2809.765526	2905.039448
3.82_329.0813m/z	329.0812983	3.82415	neg	Cysteinyl-Tyrosine	HMDB0028787	Organic acids and derivatives	Carboxylic acids and derivatives	Amino acids, peptides, and analogues	*		37.6	0	M+FA-H	C12H16N2O4S	0.061197735	1375.698415	1468.778102	1587.925445	354.8045376	391.082362	708.2561673	847.3585554	1000.034149	1027.44009
3.85_285.0075m/z	285.0075122	3.84605	neg	Sinapic acid 4-O-sulfate	HMDB0060020	Phenylpropanoids and polyketides	Cinnamic acids and derivatives	Hydroxycinnamic acids and derivatives	*		37.6	0	M-H2O-H	C11H12O8S	0.213794722	1581.805583	1743.396027	1662.580255	1701.268368	2268.182047	1989.588381	2056.63129	2122.157708	2048.488407
3.98_193.0504m/z	193.0503538	3.97985	neg	Di-2-furanylmethane	HMDB0032947	Organoheterocyclic compounds	Heteroaromatic compounds	Unclassified			37.6	0	M+FA-H	C9H8O2	-1.881493927	140.7945582	210.5347061	157.2666795	65.57039961	84.4879129	107.9205843	165.7365394	140.5945688	93.8600672
3.98_256.0229m/z	256.0229278	3.97985	neg	NITROMIDE	44168	Unclassified	Unclassified	Unclassified			37.6	0	M+FA-H	C7H5N3O5	8.552087461	3761.922761	4092.460338	4700.153409	6283.484793	6201.443397	6310.258095	5065.2906	5679.555992	5175.637918
3.98_391.2090m/z	391.2089739	3.97985	neg	2,6-Dimethoxy-4-propylphenol	91372	Benzenoids	Phenols	Methoxyphenols			37.6	0	2M-H	C11H16O3	-9.276448173	1970.461318	2200.631039	2649.352027	3099.033997	2819.543166	2594.640335	2871.994263	3232.338459	3208.026223
4.02_397.1115m/z	397.1114751	4.024283333	neg	Taraxacoside	86692	Organic oxygen compounds	Organooxygen compounds	Carbohydrates and carbohydrate conjugates	*		37.6	0	M-H	C18H22O10	-6.393391541	372.9069255	199.9529383	253.4475467	52.79072857	97.90492848	85.06107616	598.834559	970.0661279	654.414614
4.05_586.9720m/z	586.9720196	4.046666667	neg	2-Deoxy-D-ribose 1,5-bisphosphate	66127	Unclassified	Unclassified	Unclassified			37.6	0	2M-H	C5H12O10P2	-3.135572491	1189.346576	1251.627198	1497.781473	2818.080301	2830.962849	2458.549719	1675.773364	2013.901773	2291.417788
4.14_409.2349m/z	409.2348755	4.136366667	neg	PA(0:0/16:0)	LMGP10050042	Lipids and lipid-like molecules	Glycerophospholipids	Glycerophosphates	**	C00416	37.6	0	M-H	C19H39O7P	-2.896513891	1243.893477	1518.608013	1815.256628	1467.994738	1399.762709	1313.257511	1250.059889	1659.612852	1514.514944

4.34_383.0445m/z	383.0445001	4.344733333	neg	4'-Methylepicatechin 7-sulfate	HMDB0240470	Phenylpropanoids and polyketides	Flavonoids	Flavans			37.6	0	M-H	C16H16O9S	0.712076254	2596.570433	4603.344505	4955.982236	2873.338528	2767.684477	1826.038421	2150.317568	2946.483761	2723.657695
4.37_223.0469m/z	223.0469133	4.367166667	neg	3-Keto-beta-D-galactose	66316	Organic oxygen compounds	Organooxygen compounds	Carbohydrates and carbohydrate conjugates	*	C05394	37.6	0	M+FA-H	C6H10O6	5.461185597	691.9414873	775.1753307	834.6712392	1088.203428	687.1380567	721.3419739	795.2327259	833.7309875	828.8517276
4.37_431.1538m/z	431.1538436	4.367166667	neg	Artemidinol	87115	Phenylpropanoids and polyketides	Isocoumarins and derivatives	Unclassified	*		37.6	0	2M-H	C13H12O3	8.865985632	1070.91705	1113.510031	912.1322081	1316.438131	907.683173	1110.755552	692.2874803	865.1348	1188.00402
4.41_549.1223m/z	549.1222531	4.41115	neg	Peonidin 3-(6''-acetylglucoside)	47009	Unclassified	Unclassified	Unclassified			37.6	0	M+FA-H	C24H24O12	-5.407354582	3371.624928	2626.24636	3310.635683	1362.615777	1247.649218	1083.325255	2415.090283	3775.344288	1356.56836
4.48_513.0696m/z	513.0696397	4.477166667	neg	dTDP-L-olivose	63721	Unclassified	Unclassified	Unclassified			37.6	0	M-H2O-H	C16H26N2O14P2	2.920620527	2110.908121	2831.321024	2852.231326	6479.549853	5835.555716	5998.045001	2600.846485	2938.463215	3167.398103
4.50_367.0309m/z	367.0309287	4.49925	neg	Digallate	65616	Phenylpropanoids and polyketides	Depsides and depsidones	Unclassified	*	C01572	37.6	0	M+FA-H	C14H10O9	0.757557629	455.4000386	0.001403199	2514.252881	0.001403199	0.001403199	0.001403199	0.001403199	0.001403199	163.0208786
4.50_469.0813m/z	469.0813395	4.49925	neg	(Z)-Resveratrol 3-(2''-sulfoglucoside)	92051	Phenylpropanoids and polyketides	Stilbenes	Stilbene glycosides			37.6	0	M-H	C20H22O11S	0.709257563	480.2317809	702.6938472	685.9002482	429.5563326	584.6617829	473.5511454	279.4108349	564.522255	721.2328911
4.57_491.1905m/z	491.1904503	4.5668	neg	Nigellicine	86848	Organoheterocyclic compounds	Benzopyrazoles	Indazoles	*		37.6	0	2M-H	C13H14N2O3	-6.415941974	803.4932609	803.107568	1021.592051	1616.108089	1487.50798	1249.891733	777.2784373	801.9280712	915.9287291
4.61_265.1196m/z	265.119564	4.611883333	neg	Phenylacetylglycine dimethylamide	69515	Unclassified	Unclassified	Unclassified			37.6	0	M+FA-H	C12H16N2O2	0.832851959	1347.987138	1461.99268	1419.771133	1485.652565	1718.591986	1437.880641	813.065727	799.9909193	1070.264316
4.64_224.0567m/z	224.0567287	4.641416667	neg	N-Acetylanthranilate	66371	Benzenoids	Benzene and substituted derivatives	Benzoic acids and derivatives	*	C06332	37.6	0	M+FA-H	C9H9NO3	1.578722805	357.7046293	367.722011	431.8343636	626.7134246	651.546788	571.0272728	524.5576736	520.0953585	357.5831767
4.70_188.0354m/z	188.035427	4.703166667	neg	α-Cyano-4-hydroxycinnamic acid	4116	Unclassified	Unclassified	Unclassified			37.6	0	M-H	C10H7NO3	0.583902772	600.0474393	561.418404	642.8113587	286.7692427	251.1081034	289.9798632	422.2322728	403.3342648	413.1688403
4.75_432.1643m/z	432.164255	4.747466667	neg	4-Hydroxypropranolol glucuronide	HMDB0247679	Unclassified	Unclassified	Unclassified			37.6	0.584	M-H2O-H	C22H29NO9	-4.732947394	6260.159989	7232.601686	8159.591327	2713.694931	2612.180911	2060.722829	3910.536586	5497.806658	5425.269105
4.75_463.0346m/z	463.0345948	4.747466667	neg	Chrycolide	90481	Organoheterocyclic compounds	Benzofurans	Benzofuranones			37.6	0	2M-H	C12H8O3S	6.553255826	5448.030322	5454.218746	5121.622966	4103.194879	5266.72067	4359.259905	4931.138096	5226.389819	4625.256184
4.77_337.0391m/z	337.039094	4.769883333	neg	S-sulfanylglutathione	HMDB0304477	Unclassified	Unclassified	Unclassified			37.6	0	M-H	C10H16N3O6S2-	-4.975119571	2530.64586	2798.780463	2881.324359	0.001403199	279.6550061	100.0974527	83.68350497	100.5383043	0.001403199
4.84_395.0962m/z	395.0962392	4.836566667	neg	6-[2,3-Dihydroxy-1-(hydroxymethyl)propyl]-1,2-dihydro-7-hydroxy-9-methoxy-cyc	73251	Phenylpropanoids and polyketides	Coumarins and derivatives	Unclassified	**	C19590	37.6	0	M+FA-H	C17H18O8	-6.08733923	6675.470239	6861.134484	6858.924689	8250.297433	7928.176725	8334.500213	11242.71424	12080.84323	11248.20324
4.86_439.1072m/z	439.1071737	4.858383333	neg	Hallactone B	67431	Unclassified	Unclassified	Unclassified			37.6	0	M-H	C20H24O9S	0.787968512	2665.219135	2352.284859	2701.20362	13931.7522	12337.56939	13858.08093	783.098203	886.757442	684.0374548
4.97_357.1540m/z	357.1539648	4.969683333	neg	D-Galactosamine	63181	Unclassified	Unclassified	Unclassified			37.6	0	2M-H	C6H13NO5	6.969474677	554.340962	753.9878666	661.2808947	746.8775517	818.4157916	449.3158485	753.3715811	733.5537435	526.3866059
4.97_469.1694m/z	469.1694455	4.969683333	neg	Loxoprofen Metabolite cis-glucuronide	1042	Unclassified	Unclassified	Unclassified			37.6	0	M+FA-H	C21H28O9	-4.926853118	2081.583313	2034.963208	2212.290247	1788.654433	1991.332037	1723.82688	2220.891837	2529.022369	2098.795204
5.01_337.0569m/z	337.0568851	5.014716667	neg	Caffeic acid 4-O-glucuronide	96065	Organic oxygen compounds	Organooxygen compounds	Carbohydrates and carbohydrate conjugates	*		37.6	0	M-H2O-H	C15H16O10	1.065717867	1126.015255	1353.08187	1440.05232	0.001403199	0.001403199	0.001403199	0.001403199	0.001403199	0.001403199
5.01_497.1648m/z	497.1648231	5.014716667	neg	S-Acetyldihydrolipoamide	LMFA08010023	Lipids and lipid-like molecules	Fatty Acyls	Fatty amides	*	C01136	37.6	0	2M-H	C10H19NO2S2	1.320444427	1486.743041	1438.395669	1830.237833	2715.271564	2809.938993	3230.186401	1800.648079	2236.18839	1820.495913
5.13_413.1094m/z	413.10943	5.125666667	neg	3-O-Caffeoyl-1-O-methylquinic acid	HMDB0039959	Organic oxygen compounds	Organooxygen compounds	Alcohols and polyols			37.6	0	M+FA-H	C17H20O9	1.344462235	1454.48137	1521.15057	1095.416565	939.337035	522.0410433	826.2993519	1110.29804	877.9043291	1603.045792
5.13_457.1357m/z	457.1357376	5.125666667	neg	2'-(E)-Feruloyl-3-(arabinosylxylose)	HMDB0030230	Phenylpropanoids and polyketides	Cinnamic acids and derivatives	Hydroxycinnamic acids and derivatives	*		37.6	0	M-H	C20H26O12	1.282976632	2116.312672	1432.50947	1581.883055	2172.702214	2674.774205	2135.31698	3422.859014	3095.154465	3839.583423
5.16_387.0759m/z	387.0759451	5.155116667	neg	5-Keto-D-gluconate	62457	Organic acids and derivatives	Hydroxy acids and derivatives	Medium-chain hydroxy acids and derivatives	*	C01062	37.6	0	2M-H	C6H10O7	-5.369522178	2878.338355	3698.953983	3454.104852	2228.584576	2271.091654	1991.315969	2619.638911	3032.63742	3102.912232
5.22_405.1544m/z	405.1543686	5.222166667	neg	(5-Phenyl-1,2,4-triazol-3-yl)urea	70853	Unclassified	Unclassified	Unclassified			37.6	0	2M-H	C9H9N5O	0.554449215	0.001403199	257.1014912	110.6620759	90.91165721	0.001403199	0.001403199	256.4211212	268.8177889	0.001403199
5.22_407.1383m/z	407.1382911	5.222166667	neg	TETRAMISOLE	HMDB0258921	Lipids and lipid-like molecules	Fatty Acyls	Fatty alcohols	*		37.6	0	2M-H	C11H12N2S	3.255589048	1291.843037	1204.83554	1289.896922	513.0518053	578.909701	373.0345957	734.6674493	643.4583631	1085.420748
5.24_201.0029m/z	201.0029019	5.244033333	neg	2-Amino-1,3,4-thiadiazole	HMDB0244972	Organic acids and derivatives	Carboxylic acids and derivatives	Carboxylic acid derivatives			37.6	0	2M-H	C2H3N3S	3.178653809	4388.332679	3919.324384	3917.409024	317.1352538	331.030573	364.4034327	3874.096381	3246.632787	3900.249074
5.41_477.1042m/z	477.1041754	5.406433333	neg	Cosmosiin	HMDB0037340	Phenylpropanoids and polyketides	Flavonoids	Flavonoid glycosides	***	C04608	37.6	0	M+FA-H	C21H20O10	0.753843464	1788.814756	1877.375715	1669.102502	1008.755446	915.1789689	656.2168433	1023.643236	1739.595905	915.8245758
5.43_272.0753m/z	272.0752858	5.428733333	neg	Modafinil	HMDB0014883	Benzenoids	Benzene and substituted derivatives	Diphenylmethanes			37.6	0	M-H	C15H15NO2S	0.778302194	1261.89035	1583.384558	1503.45855	729.1530987	681.8301398	865.9625745	1027.0941	1674.787388	1149.394762
5.45_257.1397m/z	257.1396575	5.451166667	neg	8-Hydroxy-4,8-dimethyl-4E,9-decadienoic acid	LMFA01050373	Lipids and lipid-like molecules	Fatty Acyls	Fatty Acids and Conjugates			37.6	0	M+FA-H	C12H20O3	0.9903975	1708.907333	1697.633926	1535.995156	1872.414282	1948.858871	1999.302794	1745.064474	1460.200891	1315.667677
5.50_450.1774m/z	450.1773927	5.497816667	neg	Clivoline	68216	Unclassified	Unclassified	Unclassified			37.6	0	M+FA-H	C21H27NO7	1.079997714	4090.434711	3079.52908	3196.451683	924.9417627	1173.387164	1541.048294	2881.534472	2378.063942	3636.224372
5.53_240.0435m/z	240.043473	5.525416667	neg	p-Nitrophenyl-O-ethyl ethylphosphonate	69527	Unclassified	Unclassified	Unclassified			37.6	0	M-H2O-H	C10H14NO5P	1.370253685	2170.516769	1879.75452	1958.301332	1993.384832	3146.350455	2449.334324	2696.615121	2138.643755	2920.452954
5.53_341.1141m/z	341.1140859	5.525416667	neg	Dityrosine	58352	Lignans, neolignans and related compounds	Unclassified	Unclassified	*		37.6	0	M-H2O-H	C18H20N2O6	-0.581214935	9483.40191	7377.330871	7789.586818	2443.544743	3068.639173	3021.310546	6321.476361	6873.920451	8403.737309
5.53_387.1848m/z	387.1848107	5.525416667	neg	(R)-3-Hydroxy-5-phenylpentanoic acid	87733	Organic acids and derivatives	Hydroxy acids and derivatives	Medium-chain hydroxy acids and derivatives	*		37.6	0	2M-H	C11H14O3	9.012349521	5541.405446	4826.078347	5304.736414	5404.837838	8197.849409	5766.471798	4967.454121	5621.772777	5901.528469
5.53_607.2378m/z	607.2378023	5.525416667	neg	YC-1	64877	Unclassified	Unclassified	Unclassified			37.6	0	2M-H	C19H16N2O2	4.477224406	6562.13021	4730.496934	4765.783827	6135.745425	9084.09963	7857.332125	3898.898357	4756.399789	5832.969891
5.56_388.1418m/z	388.1417545	5.56185	neg	N-trans-Feruloyl-4-O-methyldopamine	94306	Phenylpropanoids and polyketides	Cinnamic acids and derivatives	Hydroxycinnamic acids and derivatives			37.6	0	M+FA-H	C19H21NO5	4.60115545	1921.606521	1868.987933	1607.122113	2615.182645	3260.987909	2789.825955	2218.347045	2396.033553	2132.195605
5.58_403.1612m/z	403.1612245	5.584533333	neg	Metamitron	68697	Unclassified	Unclassified	Unclassified			37.6	0	2M-H	C10H10N4O	-5.99000011	23691.37856	22520.83022	23029.93984	29494.6003	33744.7811	31191.50727	22533.2839	23404.26754	21131.24197
5.63_349.0026m/z	349.002618	5.628083333	neg	Apigenin 7-sulfate	LMPK12110431	Phenylpropanoids and polyketides	Flavonoids	Flavones			37.6	0	M-H	C15H10O8S	0.731890386	310.6160741	253.4990809	60.13271982	8469.954729	9507.653538	8817.228207	0.001403199	0.001403199	0.001403199
5.69_427.1224m/z	427.1224447	5.6875	neg	Phenyl salicylate	69843	Phenylpropanoids and polyketides	Depsides and depsidones	Unclassified	*	C14163	37.6	0	2M-H	C13H10O3	8.718914595	9963.835609	9195.193724	9677.867627	9959.86706	10653.22609	10641.97562	8595.916334	8371.035207	10284.94
5.69_507.2086m/z	507.2086254	5.6875	neg	Thiamylal	66752	Organoheterocyclic compounds	Diazines	Pyrimidines and pyrimidine derivatives		C07846	37.6	0	2M-H	C12H18N2O2S	-3.730234876	1888.864428	1328.448943	1246.397305	1845.146665	1938.044864	1862.104497	1408.433501	2045.284197	2005.425814
5.76_226.1086m/z	226.1086409	5.7632	neg	(R)-2-Nitro-p-mentha-1,5-diene	91256	Lipids and lipid-like molecules	Prenol lipids	Monoterpenoids	*		37.6	0	M+FA-H	C10H15NO2	0.879530364	82.31374915	41.76701748	151.8229305	0.001403199	0.001403199	0.001403199	35.28897306	40.69198418	84.92359424
5.79_213.1134m/z	213.1134195	5.785316667	neg	2,4-Hexadienyl butyrate	LMFA07010639	Lipids and lipid-like molecules	Fatty Acyls	Fatty acid esters	*		37.6	0	M+FA-H	C10H16O2	1.11164306	633.4836726	573.8638199	526.9977761	547.008261	733.0361009	795.3705833	863.2142033	665.8511496	787.2312868
5.83_381.1145m/z	381.1145199	5.825	neg	3-Epihydroxymugineic acid	70949	Unclassified	Unclassified	Unclassified			37.6	0	M+FA-H	C12H20N2O9	-1.675649222	2458.146547	2511.892633	1931.257313	2282.015984	1997.799634	1806.064946	2540.617966	2511.994444	2565.535465
5.85_591.1652m/z	591.1651623	5.84575	neg	PtdIns-(5)-P1 (1,2-dihexanoyl) (sodium salt)	44996	Unclassified	Unclassified	Unclassified			37.6	0	M-H2O-H	C21H40O16P2	6.301357344	33151.86926	38182.08338	38892.93054	31550.49131	32459.86383	30489.96596	49991.58616	47318.83624	48909.40183
5.88_329.1244m/z	329.1243717	5.88025	neg	3-(3,4-Dihydroxyphenyl)-1-propanol 3'-glucoside	HMDB0038304	Organic oxygen compounds	Organooxygen compounds	Carbohydrates and carbohydrate conjugates			37.6	0	M-H	C15H22O8	0.546669924	593.4828151	716.0059097	947.128914	1132.820851	984.1649137	1081.831787	933.1991073	1042.993042	1105.01707
5.88_427.1605m/z	427.1605037	5.88025	neg	7alpha,8alpha-Dihydroxycalonectrin	HMDB0038916	Lipids and lipid-like molecules	Prenol lipids	Sesquiterpenoids	*		37.6	0	M+FA-H	C19H26O8	-1.221976191	4592.545298	5875.926336	5674.987647	7295.681691	8605.818669	8107.44234	5035.680193	5564.563444	6100.127925
5.95_463.1242m/z	463.1242041	5.95155	neg	Liquiritin	52511	Phenylpropanoids and polyketides	Flavonoids	Flavonoid glycosides	*	C16989	37.6	0	M+FA-H	C21H22O9	-0.911240365	7306.889591	8612.219628	9446.773873	155.9081019	221.3086719	576.4550673	1183.981333	1487.210024	1663.452059
5.99_256.0724m/z	256.0723783	5.9916	neg	Milrinone	1312	Organoheterocyclic compounds	Pyridines and derivatives	Bipyridines and oligopyridines		C07224	37.6	0	M+FA-H	C12H9N3O	-1.831295132	786.0420573	893.8537586	894.3486268	1420.819134	1225.417006	1174.663978	1007.874805	1181.323414	1041.051175
6.01_675.2253m/z	675.2253064	6.007266667	neg	Otobanone	87100	Lignans, neolignans and related compounds	Aryltetralin lignans	Unclassified	*		37.6	0	2M-H	C20H18O5	2.566450933	21023.96696	20807.50505	22529.84218	21149.37236	21555.52396	21235.55643	18554.80197	17735.17731	18317.18106
6.02_323.1140m/z	323.1139626	6.01945	neg	Isomarasmone	91230	Organoheterocyclic compounds	Furofurans	Unclassified	*		37.6	0	M+FA-H	C15H18O5	1.208373894	798.2562049	897.9165514	935.4713063	627.1223699	492.2342919	485.3072031	1219.869967	1002.467445	1128.238236
6.07_226.1450m/z	226.1450441	6.07	neg	2-Butyl-4,5-diethyloxazole	HMDB0037891	Organoheterocyclic compounds	Azoles	Oxazoles			37.6	0	M+FA-H	C11H19NO	0.977113094	576.9649218	543.1155636	516.2747373	652.6447156	866.68637	549.9355887	627.3350528	540.7374595	524.5597414
6.10_547.2166m/z	547.2165797	6.101316667	neg	Isocyclocalamin	HMDB0035222	Lipids and lipid-like molecules	Steroids and steroid derivatives	Steroid lactones	*		37.6	0	M+FA-H	C27H34O9	-3.794808601	18208.52563	19814.63815	20150.08502	15171.75871	18035.88358	15374.42509	15013.18513	16165.90482	18089.31607
6.17_427.1610m/z	427.1609515	6.171316667	neg	Cyclocalopin B	HMDB0039811	Organoheterocyclic compounds	Furopyrans	Unclassified	*		37.6	0	M+FA-H	C19H26O8	-0.050011672	465.7337965	441.5387549	339.5555069	972.7196149	645.8110386	1152.708187	415.9882485	163.5274128	324.6715949
6.20_413.2549m/z	413.2548515	6.195716667	neg	alpha-Cortol	LMST02030228	Lipids and lipid-like molecules	Steroids and steroid derivatives	Hydroxysteroids	**	C05482	37.6	0	M+FA-H	C21H36O5	1.016614019	2609.05377	2051.686454	1960.327775	3378.901864	3090.586065	3399.03695	2160.910907	2045.543218	2579.042271
6.27_356.1175m/z	356.1174682	6.272366667	neg	Niazimicin	HMDB0031944	Organic oxygen compounds	Organooxygen compounds	Carbohydrates and carbohydrate conjugates			37.6	0	M-H	C16H23NO6S	0.381229585	2833.065387	2573.653532	2871.540318	705.5471146	861.052354	1026.247796	5054.114434	6212.896944	6924.739759
6.30_417.1772m/z	417.1772095	6.302033333	neg	2-[4-(3-Hydroxypropyl)-2-methoxyphenoxy]-1,3-propanediol 1-glucoside	HMDB0040353	Organic oxygen compounds	Organooxygen compounds	Carbohydrates and carbohydrate conjugates			37.6	0	M-H	C19H30O10	1.408019486	2289.215571	2389.471178	2668.72817	4863.348607	4195.055111	4152.393323	2011.796917	2043.038297	2511.158516
6.30_528.2649m/z	528.2649105	6.302033333	neg	N-[(3a,5b,7a)-3-hydroxy-24-oxo-7-(sulfooxy)cholan-24-yl]-Glycine	HMDB0002496	Lipids and lipid-like molecules	Steroids and steroid derivatives	Bile acids, alcohols and derivatives	*	C15559	37.6	0	M-H	C26H43NO8S	2.359147252	14748.3112	19278.11121	19665.55645	7176.058351	6595.679163	5097.15074	17741.60596	18823.32285	24105.54827
6.39_235.0953m/z	235.0953377	6.386066667	neg	Alanyl-Threonine	85612	Organic acids and derivatives	Carboxylic acids and derivatives	Amino acids, peptides, and analogues	*		37.6	0	M+FA-H	C7H14N2O4	9.352454232	4295.012029	4854.754075	5224.911604	7692.519349	7958.005276	8257.526294	4896.251182	4946.693065	4810.782572
6.48_455.1905m/z	455.1904647	6.477433333	neg	Scorzoside	95187	Lipids and lipid-like molecules	Prenol lipids	Terpene lactones	*		37.6	0	M+FA-H	C21H30O8	-4.402971492	10746.43806	10983.33771	12374.41128	13959.48672	12384.86433	12760.2631	19903.30214	21468.69824	21244.35346
6.54_411.2390m/z	411.2389807	6.539816667	neg	3b-Allotetrahydrocortisol	HMDB0000314	Lipids and lipid-like molecules	Steroids and steroid derivatives	Hydroxysteroids	*		37.6	0	M+FA-H	C21H34O5	0.419481628	2204.354666	2225.697454	2125.697309	1377.03542	1641.243975	1443.228013	1936.504304	2166.685056	1821.480573
6.56_447.1408m/z	447.1407628	6.55905	neg	desmethyldehydronimodipine	1544	Unclassified	Unclassified	Unclassified			37.6	0	M+FA-H	C20H22N2O7	-0.350907517	1606.259627	1810.088157	1902.192687	2140.650402	2314.19611	2131.809673	1205.025267	1553.826403	1180.656245
6.59_537.1957m/z	537.1957175	6.592933333	neg	Palmatoside G	71744	Unclassified	Unclassified	Unclassified			37.6	0	M+FA-H	C25H32O10	-4.129622792	14789.84589	14113.28612	14586.60607	2926.010453	3540.362193	3127.410652	9199.862506	11339.55106	10512.20205
6.60_439.1974m/z	439.1973714	6.6024	neg	Secoeremopetasitolide B	HMDB0041363	Lipids and lipid-like molecules	Prenol lipids	Terpene lactones	*		37.6	0	M+FA-H	C21H30O7	0.038627103	1920.347813	2070.897079	2219.015278	2160.177669	1939.979977	1989.682717	1803.889504	1935.43044	2057.64183
6.66_211.1340m/z	211.1340493	6.663683333	neg	2-Hexylidenecyclopentanone	87899	Organic oxygen compounds	Organooxygen compounds	Carbonyl compounds	*		37.6	0	M+FA-H	C11H18O	0.489127184	546.6295365	511.7090545	551.6697468	611.6010313	652.0327562	497.6647105	494.1164851	498.8638079	358.2195042
6.74_211.1340m/z	211.1340249	6.739933333	neg	Norecasantalol	91829	Lipids and lipid-like molecules	Prenol lipids	Monoterpenoids	*		37.6	0	M+FA-H	C11H18O	0.342064802	336.396057	292.9195601	285.4955843	467.719253	380.4606901	486.8265896	96.50432218	258.7432785	269.3489473
6.78_431.1386m/z	431.1386333	6.776866667	neg	8-Hydroxyalanylclavam	71633	Unclassified	Unclassified	Unclassified			37.6	0	2M-H	C8H12N2O5	-7.713129107	1582.227091	1557.830577	1597.656613	1225.772479	1172.461096	1163.135687	1294.24429	1756.18774	1111.597723
6.82_493.0947m/z	493.0946533	6.817666667	neg	Phenylephrine 3-O-sulfate	711	Unclassified	Unclassified	Unclassified			37.6	0	2M-H	C9H13NO5S	-1.937433383	6482.354378	5862.176329	5719.080378	6005.813402	6064.285144	6638.155824	6532.754196	5507.377935	5020.784326
6.92_453.1875m/z	453.1875426	6.915966667	neg	Fluprostenol methyl ester	45218	Unclassified	Unclassified	Unclassified			37.6	0	M-H2O-H	C24H31F3O6	-4.001787243	153638.7546	36854.73056	431597.5584	43481.99814	40105.18095	38384.52625	40612.62711	39596.26124	86969.24084
6.94_407.1174m/z	407.1173636	6.939266667	neg	Antirrhinoside	67927	Unclassified	Unclassified	Unclassified			37.6	0	M+FA-H	C15H22O10	-5.898959844	3309.921323	3251.61619	3038.823887	743.9074879	757.4490871	885.2882439	1246.512424	1814.462271	2134.127186
6.95_673.2821m/z	673.2821483	6.951516667	neg	Linezolid	66834	Organoheterocyclic compounds	Oxazinanes	Morpholines		C08146	37.6	0	2M-H	C16H20FN3O4	2.752598788	8144.58394	8234.726178	8240.683799	3128.902906	3384.999362	3160.166458	4289.476892	4882.758062	5306.092137
6.99_352.1202m/z	352.1202473	6.993516667	neg	N-Acetylanonaine	HMDB0030349	Organic acids and derivatives	Carboxylic acids and derivatives	Carboxylic acids	*		37.6	0	M+FA-H	C19H17NO3	3.910545836	2359.201957	2318.360196	2746.420705	4269.649166	3699.333811	4409.19389	2740.447269	2980.198657	3011.262975
7.04_439.1975m/z	439.1974694	7.035966667	neg	Morantel	43265	Unclassified	Unclassified	Unclassified			37.6	0	2M-H	C12H16N2S	-4.755015177	2488.641202	2921.86708	2446.12009	4418.586215	4571.81876	3552.621548	3123.492156	3497.59917	3089.25283
7.08_455.1540m/z	455.1539645	7.076316667	neg	2,4-Difurfurylfuran	88268	Organoheterocyclic compounds	Heteroaromatic compounds	Unclassified	*		37.6	0	2M-H	C14H12O3	8.66475944	4998.202351	4656.520954	4655.077615	8558.589524	8632.73174	7365.867503	4060.1482	4345.209677	4091.540153
7.16_451.1590m/z	451.1590373	7.1647	neg	ETHYL SYRINGATE	84963	Unclassified	Unclassified	Unclassified			37.6	0	2M-H	C11H14O5	-4.275700222	3632.966242	4210.328766	3914.153239	4141.65706	4098.47018	3568.891976	2980.043444	3866.568732	3528.110968
7.18_707.0978m/z	707.0978085	7.178516667	neg	Diguanosine diphosphate	HMDB0001507	Nucleosides, nucleotides, and analogues	(5'->5')-dinucleotides	Unclassified	*		37.6	0	M-H	C20H26N10O15P2	-0.492132739	3858.802989	4950.685961	4477.83721	5525.20293	5593.405398	6026.302262	4701.310297	6094.116203	4846.082328
7.20_465.2391m/z	465.239071	7.201166667	neg	2-Heptylbenzothiazole	HMDB0040033	Organoheterocyclic compounds	Benzothiazoles	Unclassified			37.6	0	2M-H	C14H19NS	-2.774833836	4592.667951	5214.871839	5300.170866	3350.105794	3993.821973	3947.211748	2483.095963	2987.222924	2652.708611
7.24_447.0314m/z	447.0314227	7.237866667	neg	2-(alpha-hydroxyethyl)thiamine diphosphate	HMDB0304055	Organoheterocyclic compounds	Diazines	Pyrimidines and pyrimidine derivatives	**	C05125	37.6	0	M-H2O-H	C14H20N4O8P2S-2	3.338690664	1700.814296	1655.972598	1730.052292	1392.956355	2101.032493	1892.250085	1853.565011	1782.976069	1871.341708
7.25_457.1698m/z	457.1698392	7.248333333	neg	endalin	96164	Nucleosides, nucleotides, and analogues	Pyrimidine nucleosides	Pyrimidine 2',3'-dideoxyribonucleosides	*		37.6	0	2M-H	C9H12FN3O3	9.988965452	1136.506443	1382.124275	1370.013786	2154.355332	2192.793577	2331.788242	1099.650388	1089.154119	1227.114907
7.34_212.1294m/z	212.1293551	7.33585	neg	Buccoxime	HMDB0036030	Organic nitrogen compounds	Organonitrogen compounds	Oximes	*		37.6	0	M+FA-H	C10H17NO	0.82624459	730.3666196	840.7513523	710.5460784	964.1585864	994.5777777	938.6823947	737.6949181	837.888996	1053.387485
7.34_439.1955m/z	439.1955008	7.33585	neg	Nigakilactone M	71514	Unclassified	Unclassified	Unclassified			37.6	0	M+FA-H	C21H30O7	-4.706585948	11360.61581	12233.38662	10770.53452	14620.36381	14896.04888	13253.39206	9865.5984	10789.07032	11904.33148
7.37_382.1332m/z	382.1332404	7.372733333	neg	Streptobiosamine	66705	Unclassified	Unclassified	Unclassified			37.6	0	M+FA-H	C13H23NO9	-6.655274433	3541.06683	4151.337301	3586.219815	1246.000593	1067.924213	1165.213667	2174.305138	2358.688175	2496.674175
7.74_427.1145m/z	427.1144827	7.738233333	neg	Phenytoin-N-glucuronide	1852	Unclassified	Unclassified	Unclassified			37.6	0	M-H	C21H20N2O8	-0.482390724	1367.870952	1220.102939	1506.825194	2265.908592	2631.356844	2393.95447	1256.088389	1055.115987	1289.387882
7.77_601.2249m/z	601.2249316	7.770583333	neg	Triphenylpropargylphosphonium	85162	Unclassified	Unclassified	Unclassified			37.6	0	2M-H	C21H18P+	4.954359632	25794.68273	29060.17413	30244.63827	46581.97782	41622.66307	41588.9804	30400.08701	32287.65496	32776.0987
7.77_669.2125m/z	669.2125444	7.770583333	neg	benfluralin	HMDB0248950	Unclassified	Unclassified	Unclassified			37.6	0	2M-H	C13H16F3N3O4	1.849378436	17714.5534	18346.44024	18759.02638	27638.19971	25897.09709	25234.30125	18217.98607	19783.85768	20673.85833
8.26_251.1266m/z	251.1265979	8.264766667	neg	2-methyl-1,3-Cyclohexanedione	96394	Unclassified	Unclassified	Unclassified			37.6	0	2M-H	C7H10O2	-9.061852136	550.5243585	667.3586891	536.8372326	1094.62221	889.5799179	1024.988276	511.707014	482.6288722	445.6562856
8.50_197.1548m/z	197.1547574	8.495033333	neg	4,8-Dimethyl-1,3(E),7-nonatriene	LMFA11000425	Lipids and lipid-like molecules	Prenol lipids	Monoterpenoids	*		37.6	0	M+FA-H	C11H20	0.354204822	430.3950317	575.297549	511.3254667	566.065216	634.4622391	541.3511146	378.6484846	378.3912821	284.4654999
8.82_463.1954m/z	463.1953729	8.8245	neg	Asteltoxin	HMDB0029464	Organoheterocyclic compounds	Furofurans	Unclassified	*		37.6	0	M+FA-H	C23H30O7	-4.742390561	38516.61225	40988.12772	38184.23616	16061.18602	16381.98441	16742.8485	25558.76396	30015.06075	28470.74953
8.82_477.2611m/z	477.2611043	8.8245	neg	PG(17:1(9Z)/0:0)	80024	Lipids and lipid-like molecules	Glycerophospholipids	Glycerophosphoglycerols			37.6	0	M-H2O-H	C23H45O9P	-2.366035594	2731.048414	3648.361739	3827.971411	2647.683151	2221.500317	2356.007404	2637.384924	3613.894983	2896.499781
8.82_493.2249m/z	493.2248673	8.8245	neg	Threoninyl-Glutamine	85952	Organic acids and derivatives	Carboxylic acids and derivatives	Amino acids, peptides, and analogues	*		37.6	0	2M-H	C9H17N3O5	-3.030075268	3919.507497	5674.454431	4630.395299	192.0729476	173.5490858	163.3888216	1497.307644	2054.28415	1987.15204
9.03_473.1432m/z	473.1431765	9.03025	neg	Lacosamide-glucuronide	HMDB0060829	Organic oxygen compounds	Organooxygen compounds	Carbohydrates and carbohydrate conjugates	*		37.6	0	M+FA-H	C18H24N2O10	4.387861178	908.2801999	1590.98338	1311.303767	2216.385049	2129.501915	2047.723788	3120.043522	3350.071339	3203.982069
9.74_479.1901m/z	479.1901429	9.7401	neg	Myristicanol A	95723	Lignans, neolignans and related compounds	Unclassified	Unclassified			37.6	0	M+FA-H	C23H30O8	-4.900842141	13220.0251	14256.40662	14131.52471	17726.18741	17796.42915	19301.26595	12208.5378	12924.78649	12010.05841
9.94_444.1215n	443.1141802	9.943016667	neg	Artomunoxanthentrione	LMPK12111528	Organoheterocyclic compounds	Benzopyrans	1-benzopyrans			37.6	0	M-H2O-H, M-H	C26H20O7	1.277727185	3480.755227	3716.941949	3020.950794	3170.202035	2964.143845	3516.95173	3275.440256	3546.050218	3330.618683
0.65_176.9606m/z	176.9606183	0.647833333	neg	1-Carboxyvinyl carboxyphosphonate	63582	Unclassified	Unclassified	Unclassified			37.5	0	M-H2O-H	C4H5O7P	5.971673797	793.8624591	1029.312026	798.1587994	749.334933	729.1842423	938.1996257	1025.833686	754.86228	1010.234045
0.74_221.0604m/z	221.0604038	0.7359	neg	Allocystathionine	HMDB0000455	Organic acids and derivatives	Carboxylic acids and derivatives	Amino acids, peptides, and analogues	*		37.5	0	M-H	C7H14N2O4S	1.136106787	1225.446254	963.2653855	819.5805049	1463.437999	1560.057072	1653.447449	1595.920055	1549.995595	1464.520028
0.74_260.9986m/z	260.9986324	0.7359	neg	Salithion	72740	Unclassified	Unclassified	Unclassified			37.5	0	M+FA-H	C8H9O3PS	-2.6494723	4083.143463	3226.139894	3518.115016	4361.701551	5451.984254	4956.477936	4813.589418	3658.074424	4200.12606
0.76_241.0925m/z	241.0924576	0.757316667	neg	D-erythro-D-galacto-octitol	86611	Organic oxygen compounds	Organooxygen compounds	Carbohydrates and carbohydrate conjugates	*		37.5	0	M-H	C8H18O8	-1.790645899	623.9810226	359.192504	658.0795985	408.9947186	221.2307414	348.5838415	535.5123377	396.3209112	144.171696
0.76_242.0778m/z	242.0778292	0.757316667	neg	Methyl5-(but-3-en-1-yl)amino-1,3,4-oxadiazole-2-carboxylate	69391	Unclassified	Unclassified	Unclassified			37.5	0	M+FA-H	C8H11N3O3	-2.105282264	686.6407252	918.5824509	441.531622	736.7515364	682.2698801	564.1169191	288.4175977	799.5125504	643.1886253
0.80_434.9487m/z	434.948652	0.800366667	neg	5-O-phosphonato-alpha-D-ribofuranosyl Diphosphate(5-)	HMDB0062643	Organic oxygen compounds	Organooxygen compounds	Carbohydrates and carbohydrate conjugates			37.5	0	M+FA-H	C5H13O14P3	-3.502722947	2873.512354	4366.612438	4360.003977	631.2327461	293.5050074	548.8509989	2426.862125	2866.754639	4281.110738
0.84_201.0536m/z	201.0535516	0.841733333	neg	4,5-Dihydro-2-methylthiazole	HMDB0029555	Organoheterocyclic compounds	Azolines	Thiazolines	*		37.5	0	2M-H	C4H7NS	4.88773793	5279.231267	5572.300358	6625.854185	2890.85944	2727.662758	2933.960973	4695.043381	4680.90023	3867.202863
0.84_226.9868m/z	226.9867742	0.841733333	neg	DMAPP (ammonium salt)	372	Unclassified	Unclassified	Unclassified			37.5	0	M-H2O-H	C5H12O7P2	-4.9211851	1179.807383	985.6228189	923.5539265	817.8519066	696.972254	785.3389053	870.3399126	1019.779816	570.1391019
0.84_335.0793m/z	335.0792633	0.841733333	neg	2,6-Dihydroxyphenylacetate	63521	Unclassified	Unclassified	Unclassified			37.5	0	2M-H	C8H8O4	6.017212233	8976.13833	9353.696641	9456.014334	9295.860556	8647.20365	8263.686324	13106.63247	9538.792897	10519.56182
0.84_361.0909m/z	361.0908543	0.841733333	neg	Phosphinothricin	63589	Unclassified	Unclassified	Unclassified			37.5	0	2M-H	C5H12NO4P	-7.341289325	37482.71465	35068.59812	43603.58554	21683.47996	21622.21659	21633.47852	39057.06632	39710.90556	40134.28634
0.88_317.0741m/z	317.0740615	0.884633333	neg	Pyridine N-oxide glucuronide	HMDB0061177	Organic oxygen compounds	Organooxygen compounds	Carbohydrates and carbohydrate conjugates	*		37.5	0	M+FA-H	C11H14NO7+	-4.293625825	2775.711271	2234.083303	2401.458269	1465.877971	1077.500769	1608.29873	2244.860779	1934.734604	1658.059068
0.88_805.2288m/z	805.2288216	0.884633333	neg	Azoxystrobin	HMDB0248818	Unclassified	Unclassified	Unclassified			37.5	0	2M-H	C22H17N3O5	3.047152975	2479.505025	4748.534524	1153.806582	3408.931802	3321.610615	2633.189354	4561.718151	3937.184102	3316.118675
0.90_167.0813m/z	167.0813011	0.904683333	neg	Amitrole	68946	Unclassified	Unclassified	Unclassified			37.5	0	2M-H	C2H4N4	8.241009252	477.1302123	468.3728858	368.7750434	227.9891704	518.4131184	100.9547569	507.9622106	264.380722	279.0756201
0.95_319.0478m/z	319.0478372	0.946033333	neg	5-Aminoimidazole-4-carboxamide-1-β-D-ribofuranosyl 5'-monophosphate	3474	Unclassified	Unclassified	Unclassified			37.5	0	M-H2O-H	C9H15N4O8P	8.661815975	2837.663999	7128.15439	10396.85321	92365.9951	65847.01951	66213.30753	8180.069231	31857.607	21694.21427
0.95_728.6451m/z	728.6451131	0.946033333	neg	Tetrac	HMDB0258867	Unclassified	Unclassified	Unclassified			37.5	0	M-H2O-H	C14H8I4O4	3.749629106	4961.9738	6597.518264	8640.97999	18603.74998	15706.51247	14697.50228	10619.0289	12385.37852	11078.83507
0.97_377.0534m/z	377.0534051	0.967616667	neg	Patuletin	51398	Phenylpropanoids and polyketides	Flavonoids	Flavones	*	C10118	37.5	0	M+FA-H	C16H12O8	5.977566562	0.001403199	0.001403199	133.1295544	4839.768056	3070.266852	3228.735993	0.001403199	1498.040324	430.792532
1.20_160.0440m/z	160.0439635	1.202333333	neg	1-Isothiocyanatobutane	87589	Organosulfur compounds	Isothiocyanates	Unclassified	*		37.5	0	M+FA-H	C5H9NS	1.65433472	186.2846969	332.0150761	263.2139553	0.001403199	0.001403199	0.001403199	292.1624907	297.2794992	443.4889097
1.25_253.0931m/z	253.0931365	1.246333333	neg	(2R)-1-O-beta-D-Galactopyranosylglycerol	HMDB0038664	Lipids and lipid-like molecules	Glycerolipids	Glycosylglycerols	*		37.5	0	M-H	C9H18O8	0.965879613	1127.435775	1236.804074	1209.602164	246.1162982	209.923421	223.1977019	344.4398775	409.9891757	312.1421159
1.31_250.0758m/z	250.0757809	1.3134	neg	5'-Deoxy-5'-fluoroadenosine	73343	Unclassified	Unclassified	Unclassified			37.5	0	M-H2O-H	C10H12FN5O3	4.476224236	439.1545258	444.5404719	539.5354987	1242.585124	1275.748311	1434.581356	1417.426143	1378.994663	1314.177009

1.58_330.0770m/z	330.0769914	1.577833333	neg	SANGUINARINE	44023	Alkaloids and derivatives	Benzophenanthridine alkaloids	Quaternary benzophenanthridine alkaloids	*	C06162	37.5	0	M-H	C20H13NO4	-0.574167299	525.4473756	461.2284239	466.6183179	410.0655005	646.701702	576.7460583	365.128218	513.5735375	352.8928595
1.58_395.0292m/z	395.0292218	1.577833333	neg	3,4-Dihydroxyphthalate	65934	Unclassified	Unclassified	Unclassified			37.5	0	2M-H	C8H6O6	9.146802286	4546.158207	4189.566586	3818.153028	0.001403199	56.5597042	0.001403199	2245.754868	2735.795277	2205.134708
1.80_178.0543m/z	178.0543483	1.798266667	neg	(R)C(R)S-S-Propylcysteine sulfoxide	HMDB0029442	Organic acids and derivatives	Carboxylic acids and derivatives	Amino acids, peptides, and analogues	*		37.5	0	M-H	C6H13NO3S	0.058288425	2279.060058	2018.031669	2218.949554	549.9213206	610.1224518	611.9849979	750.2737309	785.1292552	650.1188683
1.82_275.0710m/z	275.0709568	1.820883333	neg	BENZANTHRONE	44054	Unclassified	Unclassified	Unclassified			37.5	0	M+FA-H	C17H10O	-1.786296705	8105.240603	7975.791315	8927.669271	1714.817311	1838.080511	1677.736253	4696.333008	4800.851367	4963.660245
1.93_246.9915m/z	246.9914802	1.933583333	neg	2-[4-hydroxy-3-(sulfooxy)phenyl]acetic acid	HMDB0125151	Organic acids and derivatives	Organic sulfuric acids and derivatives	Arylsulfates	*		37.5	0	M-H	C8H8O7S	-1.278044065	799.3854164	1002.744802	736.7412272	716.8079265	739.148726	903.1084435	865.5166856	687.6642547	535.1346091
10.46_759.5182m/z	759.5181774	10.45966667	neg	1-tetradecanyl-2-(8-[3]-ladderane-octanyl)-sn-glycero-3-phospho-(1'-sn-glycerol)	LMGP04040003	Unclassified	Unclassified	Unclassified			37.5	0	M+FA-H	C40H75O8P	0.025953671	207602.3422	187420.0616	173520.5953	128474.6915	119773.8193	104253.1988	191149.8106	155505.1211	145338.5452
11.48_1093.2371m/z	1093.237118	11.4752	neg	Coumermic acid	64582	Unclassified	Unclassified	Unclassified			37.5	0	2M-H	C27H21N3O10	-0.908215027	7772.103786	6956.062669	7339.135893	4814.357326	6028.151813	8612.416823	8418.509584	8067.957265	7982.03826
12.44_759.5180m/z	759.5179923	12.4444	neg	PA(22:2(13Z,16Z)/15:0)	HMDB0115298	Lipids and lipid-like molecules	Glycerophospholipids	Glycerophosphates	*		37.5	0	M+FA-H	C40H75O8P	-0.233141431	5256.047992	4612.219617	4253.202995	839.8659569	2308.071233	822.9481341	5325.005553	4048.977489	3157.545155
13.92_339.2000m/z	339.1999535	13.91825	neg	NORETHINDRONE ACETATE	44171	Unclassified	Unclassified	Unclassified			37.5	2.22	M-H	C22H28O3	9.950469304	854354.3898	635259.6373	606029.3693	534165.5612	574590.6495	571555.9944	656377.2916	901846.1151	644738.2939
14.65_491.1695m/z	491.1695413	14.6516	neg	Corticrocin	HMDB0034384	Lipids and lipid-like molecules	Fatty Acyls	Fatty acids and conjugates	*		37.5	0	2M-H	C14H14O4	-3.251136339	14166.8808	15985.70288	16512.52485	15255.15704	16204.37712	14790.62362	17465.10696	14204.86878	13563.26267
2.36_198.9781m/z	198.978094	2.36465	neg	Tetrafluoroethylene	73011	Unclassified	Unclassified	Unclassified			37.5	0	2M-H	C2F4	-9.277104314	943.7648524	832.5446799	793.0742333	397.7699121	368.9408379	219.2008995	98.9040248	230.9969378	153.8623985
2.59_288.0525m/z	288.0524993	2.58585	neg	PMEG	68899	Unclassified	Unclassified	Unclassified			37.5	0	M-H	C8H12N5O5P	7.509351812	1940.175751	2031.837704	2514.485795	5624.136833	5631.81965	5247.949098	5644.962778	6209.359665	6432.510701
3.15_199.0977m/z	199.0976626	3.153616667	neg	3,7-Dimethyl-2E-octene-1,8-dioic acid	96876	Unclassified	Unclassified	Unclassified			37.5	0	M-H	C10H16O4	0.399779706	635.6715831	739.9991696	608.6963094	623.7458404	554.119679	506.0701942	757.7009556	687.3928643	886.2160845
3.47_239.9974m/z	239.9973795	3.47105	neg	Indole-3-carboxilic acid-O-sulphate	HMDB0060002	Organoheterocyclic compounds	Indoles and derivatives	Indolecarboxylic acids and derivatives	*		37.5	0	M-H	C9H7NO5S	0.674650469	1592.4309	2082.538125	2239.219397	2883.476494	2637.970159	3006.961974	2600.536262	2640.219897	2496.901427
3.78_254.0672m/z	254.0672236	3.779133333	neg	N-Benzyloxycarbonylglycine	66027	Unclassified	Unclassified	Unclassified			37.5	0	M+FA-H	C10H11NO4	1.018276693	431.4140396	342.086077	401.1314772	39.67037406	51.73442982	103.1121931	122.8261099	69.81464767	71.17377829
3.80_279.0485m/z	279.0484985	3.801716667	neg	zaltoprofen	HMDB0259983	Organoheterocyclic compounds	Benzopyrans	1-benzopyrans			37.5	2.06	M-H2O-H	C17H14O3S	-0.086142379	2881.383219	2492.938454	2828.487569	2533.034659	2265.532011	2182.948614	2003.831304	2024.731461	2065.72582
3.85_423.0419m/z	423.0418739	3.84605	neg	Zonisamide	66666	Organoheterocyclic compounds	Benzisoxazoles	Unclassified		C07504	37.5	0	2M-H	C8H8N2O3S	-4.659635695	3892.246335	4819.349449	4545.862723	4408.583685	4648.076282	5091.17605	4074.927393	4825.4662	5206.826295
3.85_557.0268m/z	557.0267949	3.84605	neg	Quercetin 3-glucuronide-7-sulfate	LMPK12112276	Lipids and lipid-like molecules	Polyketides	Flavonoids			37.5	0	M-H	C21H18O16S	4.508463254	4427.367644	5888.265528	5053.425674	5712.106427	6190.552458	6734.853513	7044.896371	7537.292521	6121.843592
3.89_258.1348m/z	258.1347564	3.8906	neg	3-nonenoylglycine	HMDB0094808	Organic acids and derivatives	Carboxylic acids and derivatives	Amino acids, peptides, and analogues			37.5	0	M+FA-H	C11H19NO3	0.281609862	248.5996899	338.0983997	290.2301592	204.5285567	209.8122047	356.9790602	394.917727	386.8885527	366.2662035
3.91_503.0788m/z	503.0788105	3.912566667	neg	Morindaparvin A	68287	Unclassified	Unclassified	Unclassified			37.5	0	2M-H	C15H8O4	3.113588278	700.7299118	653.1322515	1240.715196	0.001403199	0.001403199	0.001403199	812.4068222	879.8011904	708.0560917
4.02_358.1028m/z	358.102825	4.024283333	neg	Quinoline-3-carboxamides	69160	Unclassified	Unclassified	Unclassified			37.5	0	M-H	C21H14FN3O2	8.622708961	687.558621	1028.018263	1000.028417	350.0575854	550.8364599	713.1234854	681.9897851	682.7436941	688.6034464
4.09_234.0410m/z	234.0409707	4.091783333	neg	α-CYANO-3-HYDROXYCINNAMIC ACID	44302	Unclassified	Unclassified	Unclassified			37.5	0	M+FA-H	C10H7NO3	0.924604044	162.3036073	161.500979	162.5629653	313.9867488	346.6052968	276.0450317	148.6664724	213.7660428	236.2397056
4.21_219.0875m/z	219.0875363	4.212116667	neg	Pyrimidin-4-yl-Methanol	44994	Unclassified	Unclassified	Unclassified			37.5	0	2M-H	C5H6N2O	-5.510680952	566.1753734	333.9399723	239.9965646	72.72571366	39.60675837	81.21691591	405.4823984	317.9271611	315.2142832
4.32_326.0884m/z	326.0884149	4.3224	neg	Fenitropan	72698	Unclassified	Unclassified	Unclassified			37.5	0	M+FA-H	C13H15NO6	0.977726037	2462.021754	2897.349534	2827.402911	3963.698524	3985.418577	3567.228056	3237.309144	4066.537392	3983.751755
4.39_331.0913m/z	331.0912942	4.3893	neg	3',4'-Dihydrodiol	85300	Organoheterocyclic compounds	Azolidines	Imidazolidines	*		37.5	0	M+FA-H	C15H14N2O4	-7.919199621	317023.8803	319517.7376	304855.9854	251884.5429	247346.2027	242382.8114	354594.4769	371630.1986	368962.3834
4.41_379.0913m/z	379.0912959	4.41115	neg	RG-108	64811	Unclassified	Unclassified	Unclassified			37.5	0	M+FA-H	C19H14N2O4	-6.776204947	69523.23381	84918.49144	81240.01248	16538.27154	13953.31088	14625.46154	42300.11628	54420.07463	46919.18369
4.43_383.1351m/z	383.1350765	4.432683333	neg	Acetyldeoxynivalenol	91293	Lipids and lipid-like molecules	Prenol lipids	Sesquiterpenoids	*		37.5	0	M+FA-H	C17H22O7	0.948029307	1362.904557	1179.302287	1570.405546	3063.251187	2383.712415	2432.052007	2248.570651	2018.967483	2293.933949
4.45_267.1352m/z	267.135198	4.454616667	neg	Mexacarbate	72712	Unclassified	Unclassified	Unclassified			37.5	0	M+FA-H	C12H18N2O2	0.753373057	731.1323811	538.9953459	513.5111348	855.7089058	348.7615603	679.3697103	1220.382394	1152.005859	1244.226677
4.50_309.1570m/z	309.1570464	4.49925	neg	Sinapine	HMDB0029379	Alkaloids and derivatives	Morphinans	Unclassified		C00933	37.5	0	M-H	C16H24NO5+	-3.627111483	604.1355621	685.018813	540.1036061	605.1459029	487.4431929	571.9879955	591.4348676	554.9454961	593.1943323
4.61_471.1869m/z	471.1869276	4.611883333	neg	Abscisic acid glucose ester	64113	Unclassified	Unclassified	Unclassified			37.5	0	M+FA-H	C21H30O9	-0.604870209	1191.563861	1209.322715	1234.412964	2283.125385	1920.593559	2103.382498	1453.144503	1167.921684	1210.210111
4.61_549.1620m/z	549.1619741	4.611883333	neg	6-Hydroxy-7-methyl-3',4',5'-trimethoxyaurone 4-O-rhamnoside	LMPK12130046	Unclassified	Unclassified	Unclassified			37.5	0	M+FA-H	C25H28O11	1.208897127	3049.582467	2996.826776	2332.83914	2887.900467	2150.528375	2251.858296	851.5188302	508.9452782	1113.844967
4.66_327.1562m/z	327.1561977	4.660483333	neg	Spenolimycin	71966	Unclassified	Unclassified	Unclassified			37.5	0	M-H2O-H	C15H26N2O7	0.108714001	991.5858301	1218.266829	1082.346533	4321.339921	3913.3238	4658.073437	2085.742419	2130.123615	1581.040436
4.73_191.0339m/z	191.0338668	4.725416667	neg	Protoanemonin	66577	Organoheterocyclic compounds	Dihydrofurans	Furanones		C07090	37.5	0	2M-H	C5H4O2	-5.808838352	497.7062064	670.5356811	722.4418954	606.7621122	547.4470139	609.6485699	631.3971444	577.8513412	698.356275
4.73_615.9858m/z	615.9857975	4.725416667	neg	N-desethylamiodarone	HMDB0060528	Organic oxygen compounds	Organooxygen compounds	Carbonyl compounds	*		37.5	0	M-H	C23H25I2NO3	1.109021311	5179.242656	6653.887125	6533.429658	3211.139569	2754.059408	2488.042577	4307.578586	6275.873122	5524.725921
4.75_500.1518m/z	500.1518223	4.747466667	neg	5,10-Methenyltetrahydrofolic acid	HMDB0001354	Organoheterocyclic compounds	Pteridines and derivatives	Pterins and derivatives	**	C00445	37.5	0	M+FA-H	C20H21N7O6	-3.761338913	4711.921253	5398.214635	5736.86691	2151.624451	1842.079628	1702.902742	4421.825753	4785.912538	4873.284001
4.79_239.0914m/z	239.0914113	4.792316667	neg	Butylparaben	88536	Benzenoids	Benzene and substituted derivatives	Benzoic acids and derivatives	*		37.5	0	M+FA-H	C11H14O3	-5.594437824	6741.733857	7011.518174	7167.946998	6048.522166	6135.829321	6353.07825	5437.740785	5463.177341	5326.090519
4.79_345.0207m/z	345.0206596	4.792316667	neg	Benzenamine sulfate	63303	Unclassified	Unclassified	Unclassified			37.5	0	2M-H	C6H7NO3S	-4.023228329	10038.5359	10094.15192	9904.634261	10799.31476	11587.39605	10652.9042	12418.01122	11111.26156	11790.64965
4.86_281.1043n	280.0970319	4.858383333	neg	Annolobine	67612	Unclassified	Unclassified	Unclassified			37.5	0	M-H, M+FA-H	C17H15NO3	-3.148226236	1391.69826	1161.867383	1537.910865	2335.810577	1887.920962	1521.406765	1554.449866	1623.027628	1702.828396
4.86_377.1453m/z	377.145295	4.858383333	neg	Glycyl-Asparagine	85749	Organic acids and derivatives	Carboxylic acids and derivatives	Amino acids, peptides, and analogues	*		37.5	0	2M-H	C6H11N3O4	7.033490056	2886.208638	3362.802842	3407.289677	3120.093451	3433.842337	3406.998714	3448.582362	4222.307206	3924.933751
4.88_195.1027m/z	195.1026589	4.880083333	neg	Hexyl 2-furoate	HMDB0037725	Organoheterocyclic compounds	Furans	Furoic acid and derivatives			37.5	0	M-H	C11H16O3	-0.046182203	197.9956206	261.0584744	266.8005721	95.15881075	216.3392616	218.4569642	149.4350848	84.75921822	94.79032379
4.88_413.1175m/z	413.1175391	4.880083333	neg	trans-3-Hydroxycotinine glucuronide	6080	Organic oxygen compounds	Organooxygen compounds	Carbohydrates and carbohydrate conjugates	*		37.5	0	M+FA-H	C16H20N2O8	-7.14266934	1734.467503	2318.457984	2473.711122	1620.477263	1989.685385	1443.417468	1233.048931	1023.377749	1126.87614
4.88_469.0342m/z	469.0342371	4.880083333	neg	Thiamine pyrophosphate	2832	Organoheterocyclic compounds	Diazines	Pyrimidines and pyrimidine derivatives	**	C00068	37.5	0	M+FA-H	C12H18N4O7P2S	-2.615227918	2361.993119	1974.266394	2650.03321	10.40511103	0.001403199	16.31911591	4544.267152	4665.79467	4335.958258
4.88_597.2145m/z	597.2145435	4.880083333	neg	E3040	69116	Unclassified	Unclassified	Unclassified			37.5	0	2M-H	C16H17N3OS	5.605875	1076.994233	878.5100148	1386.502194	501.7554359	1841.021456	956.3243388	603.0711146	1407.201576	449.410313
4.90_313.0935m/z	313.0934883	4.902316667	neg	Leonuriside A	87893	Organic oxygen compounds	Organooxygen compounds	Carbohydrates and carbohydrate conjugates	*		37.5	0	M-H2O-H	C14H20O9	1.798265629	1244.751699	1546.036361	1631.628113	2528.065557	2626.656152	2237.26213	2120.804757	2180.348768	2555.142804
4.92_228.1244m/z	228.1243803	4.92485	neg	Acetyltropine	64445	Unclassified	Unclassified	Unclassified			37.5	0	M+FA-H	C10H17NO2	1.357683738	142.2765408	88.25155631	161.4125579	145.2426765	175.0867521	205.92745	153.8621477	139.4356982	56.87980677
4.95_258.0204m/z	258.0204286	4.94705	neg	Omethoate	72448	Unclassified	Unclassified	Unclassified			37.5	0	M+FA-H	C5H12NO4PS	-1.125641564	5080.207832	5818.274814	5645.403404	8017.875985	7426.299381	7731.819593	1059.727734	926.5440959	1220.760508
5.01_223.0146m/z	223.0145881	5.014716667	neg	3-(Phosphoacetylamido)-L-alanine	66116	Unclassified	Unclassified	Unclassified			37.5	0	M-H2O-H	C5H11N2O7P	8.436680378	574.953327	656.0848687	645.0997469	817.9604304	797.0529173	1035.85328	735.7437609	722.5574089	817.8136711
5.01_274.0432n	273.0359759	5.014716667	neg	1-Deoxy-D-altro-heptulose 7-phosphate	66168	Unclassified	Unclassified	Unclassified			37.5	0	M-H2O-H, M-H	C7H15O9P	-7.902693137	2100.529389	2335.728743	2597.305474	1770.307609	1653.157277	1570.132005	1831.146026	1717.801944	1214.860278
5.01_623.2327m/z	623.2327174	5.014716667	neg	4-Hydroxycinnamyl alcohol 4-D-glucoside	64179	Unclassified	Unclassified	Unclassified			37.5	0	2M-H	C15H20O7	-2.902968082	1186.250899	1881.307385	1600.316668	1788.325154	1964.824496	1498.423915	1348.513891	1922.665052	1209.453375
5.26_309.1322m/z	309.1322289	5.263033333	neg	Mannopine	71286	Unclassified	Unclassified	Unclassified			37.5	0	M-H	C11H22N2O8	6.092850656	3684.932284	2980.69998	2684.894595	3860.831176	3754.568437	3714.313929	2959.371441	2158.313558	2777.630165
5.26_579.1283m/z	579.1283474	5.263033333	neg	TEPP	72768	Unclassified	Unclassified	Unclassified			37.5	0	2M-H	C8H20O7P2	-2.117849603	382.8437707	0.001403199	137.138556	931.2553558	508.3073812	611.7870533	555.8550755	717.0958099	844.2362618
5.29_441.1383m/z	441.1383418	5.28555	neg	Methyl (3x,4E,10R)-3,10-dihydroxy-4,11-dodecadiene-6,8-diynoate 10-glucosid	95350	Lipids and lipid-like molecules	Fatty Acyls	Fatty acyl glycosides	*		37.5	0	M+FA-H	C19H24O9	-4.779554932	6078.533554	5148.785149	4932.110737	5534.928138	6761.179455	8010.001067	6442.477973	4964.903329	6477.253769
5.32_565.1129m/z	565.1129435	5.320233333	neg	Ginkgetin	HMDB0033762	Phenylpropanoids and polyketides	Flavonoids	Biflavonoids and polyflavonoids		C10048	37.5	0	M-H	C32H22O10	-1.902389866	3718.584656	3317.344476	4584.205751	3145.394917	4765.019802	4777.949922	4913.616861	4036.88588	5476.428358
5.41_353.0881m/z	353.0880642	5.406433333	neg	4R,5R,6S-Trihydroxy-2-hydroxymethyl-2-cyclohexen-1-one 6-(2-hydroxy-6-meth	95167	Benzenoids	Benzene and substituted derivatives	Benzoic acids and derivatives			37.5	0	M+FA-H	C15H16O7	0.838995611	2039.528361	2049.82302	2097.847661	979.9892982	784.8914809	872.6294784	1491.006451	1698.624062	1425.093665
5.45_370.0522m/z	370.0522475	5.451166667	neg	N-Acetylneuraminate 9-phosphate	58378	Organic oxygen compounds	Organooxygen compounds	Carbohydrates and carbohydrate conjugates	**	C06241	37.5	0	M-H2O-H	C11H20NO12P	-5.71381437	2252.24273	2390.51644	2259.325351	693.011757	561.1905545	913.4767883	2058.053515	1639.003546	1691.051049
5.50_489.1982m/z	489.1981534	5.497816667	neg	dihydrophaseic acid 4-O-beta-D-glucoside	LMPR0103050006	Lipids and lipid-like molecules	Prenol lipids	Isoprenoids			37.5	0	M+FA-H	C21H32O10	0.907929374	1453.499629	1221.058495	1313.162106	275.971243	491.2698157	631.6483458	0.001403199	223.787997	3.507278799
5.68_293.1512m/z	293.1511578	5.678766667	neg	Leucyl-Tyrosine	85843	Organic acids and derivatives	Carboxylic acids and derivatives	Amino acids, peptides, and analogues	*		37.5	0	M-H	C15H22N2O4	1.621551724	938.5466696	1022.837875	1093.781577	1653.723958	1840.476086	1380.621765	792.2916032	964.254988	960.8766994
5.73_340.0577m/z	340.0577242	5.727283333	neg	5-Aminoimidazole ribonucleotide	6097	Organic oxygen compounds	Organooxygen compounds	Carbohydrates and carbohydrate conjugates	**	C03373	37.5	0	M+FA-H	C8H14N3O7P	8.760690177	1082.137548	1134.744537	1173.241742	1897.587975	2584.607855	1941.365319	1278.889209	2204.408106	2261.666039
5.76_327.1089m/z	327.1089156	5.7632	neg	Aucubin	41151	Lipids and lipid-like molecules	Prenol lipids	Terpene glycosides	*	C09771	37.5	0	M-H2O-H	C15H22O9	1.081920502	1161.237613	1463.137965	1344.670072	1369.746381	1904.259674	1408.386133	1842.176339	2012.550708	1704.19725
5.83_269.1007m/z	269.1006863	5.825	neg	2-Hydroxymethylserine	65894	Unclassified	Unclassified	Unclassified			37.5	0	2M-H	C4H9NO4	6.098296804	1081.281562	922.1601439	911.0502081	883.9533307	670.3240647	715.1324963	1024.309161	817.5420185	881.71324
5.88_359.0687m/z	359.0687087	5.88025	neg	Geranyl diphosphate	400	Lipids and lipid-like molecules	Prenol lipids	Isoprenoid phosphates	**	C05847	37.5	0	M+FA-H	C10H20O7P2	6.621584132	1251.615292	1618.981905	1612.852303	1658.693257	1519.719873	1251.383672	1469.336733	1341.626388	1713.490348
5.89_526.2486m/z	526.2486487	5.894166667	neg	N-[(3a,5b,7a,12a)-3,12-dihydroxy-24-oxo-7-(sulfooxy)cholan-24-yl]-Glycine	HMDB0002640	Lipids and lipid-like molecules	Steroids and steroid derivatives	Bile acids, alcohols and derivatives	*		37.5	0	M-H2O-H	C26H43NO9S	1.167938506	2673.418269	3876.886879	3275.199598	2871.007389	2788.062384	3122.315643	2656.137327	3165.365505	3807.391373
5.91_213.1497m/z	213.1497354	5.908066667	neg	(+/-)-trans- and cis-4,8-Dimethyl-3,7-nonadien-2-ol	HMDB0032238	Lipids and lipid-like molecules	Prenol lipids	Monoterpenoids	*		37.5	0	M+FA-H	C11H20O	0.697499578	898.7084343	978.4275102	881.9078298	1156.923705	1419.242587	1256.238756	722.4280121	1044.184599	1085.885487
5.94_445.2078m/z	445.207784	5.937716667	neg	Linalool oxide D 3-[apiosyl-(1->6)-glucoside]	87620	Organic oxygen compounds	Organooxygen compounds	Carbohydrates and carbohydrate conjugates	*		37.5	0	M-H2O-H	C21H36O11	-0.294786533	1073.047515	1475.902328	993.8751072	451.6047806	552.0156598	771.1455791	563.2674368	897.1922647	984.0622222
5.99_459.2240m/z	459.2239686	5.9916	neg	Tentoxin	66978	Unclassified	Unclassified	Unclassified			37.5	0	M+FA-H	C22H30N4O4	-2.268529235	7210.639399	7994.084783	7937.986154	8062.410284	8485.059676	8047.627233	4734.878221	5544.959563	5671.853791
6.07_411.1851m/z	411.1850519	6.07	neg	(2RS,5RS)-(E)-2-(2-Phenylethenyl)-1,3-dioxan-5-ol	88084	Benzenoids	Benzene and substituted derivatives	Styrenes	*		37.5	0	2M-H	C12H14O3	9.07278337	7602.267251	8818.474095	8126.393741	8773.141866	9673.549454	8313.800597	6924.503556	7932.446446	7689.985216
6.09_395.1326m/z	395.1326079	6.087383333	neg	p-Hydroxybenzophenone	1760	Unclassified	Unclassified	Unclassified			37.5	0	2M-H	C13H10O2	9.403756115	10122.59168	11675.27474	11673.7778	3681.662923	4046.567786	3677.11579	10093.02347	10044.0703	10808.76371
6.27_276.1576n	257.1397128	6.272366667	neg	4-Hydroxycinnamoylagmatine	HMDB0033460	Phenylpropanoids and polyketides	Cinnamic acids and derivatives	Hydroxycinnamic acids and derivatives	**	C04498	37.5	0	M-H2O-H, M-H	C14H20N4O2	-3.881933676	2072.451217	1885.882486	2099.175972	3580.801124	3452.647952	3534.528181	2257.483026	2701.790606	2461.034026
6.27_381.1386m/z	381.1386218	6.272366667	neg	2-Methoxyestradiol-17beta 3-sulfate	LMST05020007	Lipids and lipid-like molecules	Sterol Lipids	Steroid conjugates	**	C08359	37.5	0	M-H	C19H26O6S	2.325583456	671.3655233	502.7166184	404.0776705	884.2783182	875.9166304	675.1387806	668.9365001	780.3024802	920.4086969
6.27_425.1820m/z	425.1820287	6.272366667	neg	6b-Angeloyl-3b,8b,9b-trihydroxy-7(11)-eremophilen-12,8-olide	HMDB0041228	Lipids and lipid-like molecules	Prenol lipids	Terpene lactones	*		37.5	0	M+FA-H	C20H28O7	0.84852249	1763.714651	1869.621876	2413.440415	1731.412257	1415.798652	1396.635931	1748.230954	1737.798689	2093.048596
6.30_601.1183m/z	601.118334	6.302033333	neg	4-Aminophenyl 1-thio-β-D-glucuronide	4090	Unclassified	Unclassified	Unclassified			37.5	0	2M-H	C12H15NO6S	2.647407061	4899.816516	5439.383266	6085.027536	5469.524887	4712.020346	4301.236917	4127.109729	4901.151651	5254.876616
6.35_383.1536m/z	383.1536114	6.348783333	neg	Allyl phenoxyacetate	87805	Benzenoids	Benzene and substituted derivatives	Phenoxyacetic acid derivatives	*		37.5	0	2M-H	C11H12O3	9.36953894	2991.176494	4045.966133	3673.17521	4544.074703	4486.826079	4162.952213	3588.534935	3467.589718	3455.790554
6.37_555.1124m/z	555.1124345	6.373833333	neg	4-Hydroxyfurano[2",3":6,7]aurone	52439	Unclassified	Unclassified	Unclassified			37.5	0	2M-H	C17H10O4	7.000968903	15829.55864	18016.07325	18668.51274	14313.75757	15351.74061	14450.56336	14014.4749	15403.37995	15475.40126
6.57_387.1090m/z	387.1090453	6.570233333	neg	CRESOPYRINE	43914	Unclassified	Unclassified	Unclassified	***		37.5	0	2M-H	C10H10O4	1.298907337	708.6795216	833.6624976	783.2692743	0.001403199	0.001403199	0.001403199	68.82640668	160.6117096	126.5702591
6.59_431.1532m/z	431.1532491	6.592933333	neg	O-Desmethylnaproxen	1463	Benzenoids	Naphthalenes	Naphthols and derivatives	*		37.5	0	2M-H	C13H12O3	7.490514778	880.5041922	960.0826815	790.1276325	1850.226317	1924.810235	1780.826546	1025.548804	1109.482911	890.7144636
6.63_435.0758m/z	435.0757893	6.63275	neg	Dimethyl 2-galloylgalactarate	91924	Organic oxygen compounds	Organooxygen compounds	Carbohydrates and carbohydrate conjugates			37.5	0	M+FA-H	C15H18O12	-5.741232865	41733.75037	46830.17599	42398.46943	49457.48619	46680.63454	46840.07106	5864.24955	7053.593687	7460.995566
6.63_613.1178m/z	613.1178044	6.63275	neg	Neobignonoside	HMDB0033006	Phenylpropanoids and polyketides	Flavonoids	Flavonoid glycosides			37.5	0	M+FA-H	C28H24O13	-3.677483428	17038.15416	20320.64354	17509.19244	11346.69234	11608.27651	11850.28292	0.001403199	0.001403199	247.5276792
6.66_299.1866m/z	299.1865631	6.663683333	neg	7-Drimene-11,12,14-triol	HMDB0036866	Organic oxygen compounds	Organooxygen compounds	Alcohols and polyols	*		37.5	0	M+FA-H	C15H26O3	0.651145325	1314.057148	1336.175386	1340.439746	1577.969131	1722.172552	1420.861104	1056.23627	1271.969305	1260.306884
6.73_495.1853m/z	495.1853451	6.72945	neg	Sudan I	73204	Unclassified	Unclassified	Unclassified			37.5	0	2M-H	C16H12N2O	5.432349225	1315.121957	1456.455095	1179.256628	3212.23909	2444.513237	2604.729935	1707.07162	2332.32678	2389.081494
6.75_383.1539m/z	383.1539343	6.747166667	neg	16alpha-Hydroxy DHEA 3-sulfate	HMDB0062544	Lipids and lipid-like molecules	Steroids and steroid derivatives	Sulfated steroids	*		37.5	0	M-H	C19H28O6S	1.434591569	842.6140286	1028.68062	1167.765336	1165.928356	1029.271236	745.2944635	264.763003	690.1061132	899.587782
6.81_443.1361m/z	443.1361321	6.809016667	neg	D 4476	45527	Unclassified	Unclassified	Unclassified			37.5	0	M+FA-H	C23H18N4O3	0.097380376	4630.763637	4770.91365	4649.189737	1394.446561	1031.015496	1479.225595	3229.913705	2849.488113	2636.959859
6.82_383.1538m/z	383.1538078	6.817666667	neg	Methyl trans-p-methoxycinnamate	87175	Phenylpropanoids and polyketides	Cinnamic acids and derivatives	Cinnamic acid esters	*		37.5	0	2M-H	C11H12O3	9.880750716	815.0462851	819.2498023	808.0654073	948.2515872	1281.911884	1287.175813	997.4171975	904.8300515	825.4242106
6.88_271.0953m/z	271.0952844	6.8759	neg	Toluate	65599	Benzenoids	Benzene and substituted derivatives	Benzoic acids and derivatives	**	C01454	37.5	0	2M-H	C8H8O2	-8.445868876	862.8968612	1050.29152	663.4655126	535.1849191	907.6392857	639.1769913	606.4885152	607.2182507	413.8072188
6.88_547.1642m/z	547.1641626	6.884583333	neg	1,4-Benzenediol, 2,6-bis(1-methylethyl)-, 4-(hydrogen sulfate)	2160	Unclassified	Unclassified	Unclassified			37.5	0	2M-H	C12H18O5S	-6.476600175	6943.965304	8149.214909	6678.397735	776.3308758	1172.905933	739.4161636	5825.591041	7002.791689	6436.687602
6.90_382.1674m/z	382.1673892	6.896766667	neg	RCS-4 N-(5-hydroxypentyl) metabolite	85217	Unclassified	Unclassified	Unclassified			37.5	0	M+FA-H	C21H23NO3	4.130590061	3057.522166	3517.1142	2630.220533	3527.742897	4298.934137	3123.89704	1602.744681	2204.677896	2477.954035
7.03_373.1329m/z	373.132903	7.02545	neg	2-hydroxymethylolanzapine	HMDB0060987	Benzenoids	Benzene and substituted derivatives	Benzenesulfonamides	*		37.5	0	M+FA-H	C17H20N4OS	-3.298017797	20703.69227	20127.25501	21563.47574	8500.676269	7724.040388	7794.613096	13191.26785	14080.97251	13115.02065
7.03_567.3179m/z	567.3179085	7.02545	neg	6alpha-Glucuronosylhyodeoxycholate	LMST05010016	Lipids and lipid-like molecules	Sterol Lipids	Steroid conjugates		C11246	37.5	0	M-H	C30H48O10	0.769309312	2025.847511	1856.618371	1561.354493	1516.933268	1336.103432	1274.015585	2013.250816	1804.328603	1428.17973
7.11_483.2218m/z	483.2217924	7.107783333	neg	Diphenylcarbazide	68930	Unclassified	Unclassified	Unclassified			37.5	0	2M-H	C13H14N4O	-9.196735402	4619.335101	4128.352052	4140.766814	3754.810815	4667.244714	3856.001773	3203.577945	3652.495834	4117.103498
7.15_280.0724m/z	280.0724334	7.147366667	neg	Nitrazepam	1559	Organoheterocyclic compounds	Benzodiazepines	1,4-benzodiazepines		C07487	37.5	0	M-H	C15H11N3O3	-1.178882285	739.1290133	835.0448177	757.7455756	1508.621221	1595.170755	1290.486917	886.6088617	1158.960769	1001.795283
7.18_775.0847m/z	775.0846782	7.178516667	neg	Repenol	48089	Lipids and lipid-like molecules	Polyketides	Flavonoids			37.5	0	2M-H	C18H12O10	7.552603437	2737.019657	3724.373481	2687.204285	3406.963718	2960.312112	3148.655589	2562.743557	4037.042821	2170.404004
7.24_379.0439m/z	379.0438914	7.237866667	neg	Dehydrodolineone	48071	Lipids and lipid-like molecules	Polyketides	Flavonoids			37.5	0	M+FA-H	C19H10O6	-6.135416826	4207.318384	4614.283299	3647.268085	4214.930489	5075.102836	4555.220024	5084.724507	4408.37634	5089.43126
7.29_403.1144m/z	403.1144199	7.286583333	neg	Penmacric acid	86267	Organic acids and derivatives	Carboxylic acids and derivatives	Amino acids, peptides, and analogues	*		37.5	0	2M-H	C7H10N2O5	9.288004719	1228.892786	1394.011416	1363.913628	1823.496268	1916.653099	1670.147551	829.5318984	1010.282127	1069.14761
7.30_565.3020m/z	565.3020228	7.29525	neg	Digoxigenin monodigitoxoside	HMDB0060819	Organic oxygen compounds	Organooxygen compounds	Ethers	*		37.5	0	M+FA-H	C29H44O8	0.387324259	4731.639826	5330.836872	5940.476478	4540.198004	4150.565936	5092.548433	4832.084708	6106.797901	6575.311602
7.32_599.2094m/z	599.2094199	7.324733333	neg	5-NITRO-2-PHENYLPROPYLAMINOBENZOIC ACID [NPPB]	44306	Unclassified	Unclassified	Unclassified			37.5	0	2M-H	C16H16N2O4	-8.859471947	1445.942921	2043.968166	2073.122988	2699.23665	2758.216473	2061.470694	1985.428622	2502.479721	2081.568991
7.35_403.0938m/z	403.0937722	7.353466667	neg	Sodium cyclamate	HMDB0303168	Organic acids and derivatives	Sulfamic acid derivatives	Cyclamates	*	C02824	37.5	0	2M-H	C6H13NNaO3S+	-4.252573895	2612.645019	2632.810843	2071.632141	2372.118425	3013.373025	2370.220987	2852.556351	2462.669113	2987.945853
7.77_533.2372m/z	533.2372134	7.770583333	neg	L-Agaritine	HMDB0033758	Organic acids and derivatives	Carboxylic acids and derivatives	Amino acids, peptides, and analogues			37.5	1.48	2M-H	C12H17N3O4	1.268718152	56354.85589	60953.10126	62954.38627	92421.31623	84433.95709	84077.36027	62814.89866	67192.16458	65926.15645
7.79_454.1520m/z	454.1519962	7.793283333	neg	Acetylcorynoline	73548	Unclassified	Unclassified	Unclassified			37.5	0	M+FA-H	C23H23NO6	3.069459544	1609.092027	2062.987245	2554.398045	2688.564603	2442.817969	2477.60194	1666.500655	2424.212229	1930.961366
7.93_625.3606m/z	625.3605866	7.9268	neg	Convallasaponin A	67250	Lipids and lipid-like molecules	Prenol lipids	Monoterpenoids	*		37.5	0	M+FA-H	C32H52O9	2.154733151	4305.207432	4682.144158	3854.302988	5725.11596	5316.653158	5727.327115	3690.288141	4995.92273	5002.477493
7.95_415.1978m/z	415.197822	7.949583333	neg	5-Megastigmen-7-yne-3,9-diol 9-glucoside	HMDB0032841	Lipids and lipid-like molecules	Fatty Acyls	Fatty acyl glycosides	*		37.5	0	M+FA-H	C19H30O7	1.258357299	9331.402857	8993.622634	9962.829624	13032.84569	12022.93497	13235.64387	6026.190941	7630.206018	5949.336774
7.95_529.2403m/z	529.2403154	7.949583333	neg	PI(13:0/0:0)	LMGP06050001	Lipids and lipid-like molecules	Glycerophospholipids	Glycerophosphoinositols			37.5	0	M-H	C22H43O12P	-3.058249717	3210.58186	3798.381959	4424.612658	2260.097115	2360.534296	2667.753793	2823.086922	3637.299968	3098.786664
8.04_411.2025m/z	411.2025344	8.042716667	neg	Anhydrocinnzeylanol	91866	Lipids and lipid-like molecules	Prenol lipids	Terpene lactones	*		37.5	0	M+FA-H	C20H30O6	0.253425992	1593.609196	1845.981533	2661.596968	559.6110939	470.779219	398.5879447	1161.790762	1140.019211	1181.027109
8.26_341.0619m/z	341.0618982	8.264766667	neg	4-Toluenesulfonamide	70041	Unclassified	Unclassified	Unclassified			37.5	0	2M-H	C7H9NO2S	-4.748965518	1592.734391	1977.446201	1514.315352	1778.784585	1466.941106	1513.330649	1703.155902	1613.326269	1461.501014
8.79_371.1326m/z	371.1325598	8.791166667	neg	1-Naphthylacetic acid	69522	Benzenoids	Naphthalenes	Unclassified	*	C13014	37.5	0	2M-H	C12H10O2	9.881161304	8953.597856	9656.096118	10209.46462	377.0109337	249.2518442	343.0656674	8369.905487	9422.299321	8912.743977
8.79_409.1870m/z	409.1869786	8.791166667	neg	Gibberellin A37	LMPR0104170016	Lipids and lipid-like molecules	Prenol lipids	Diterpenoids	*	C14161	37.5	0	M+FA-H	C20H28O6	0.513720144	1468.652497	1458.453652	2001.264143	457.0509614	609.7241531	810.2575359	1142.762442	1260.693394	1250.15049
8.96_269.1314m/z	269.1313564	8.962016667	neg	PHENACYLAMINE	44663	Organic oxygen compounds	Organooxygen compounds	Carbonyl compounds	*		37.5	0	2M-H	C8H9NO	6.681764639	360789.3235	340337.7622	332522.0523	339375.8227	361796.2339	360155.049	389555.389	347074.4239	363712.497
9.26_373.1870m/z	373.1870171	9.258733333	neg	7-hydroxygranisetron	HMDB0061062	Organoheterocyclic compounds	Benzopyrazoles	Indazoles	*		37.5	0	M+FA-H	C18H24N4O2	-3.38769548	2780.450978	3024.083303	2888.185352	2224.654352	2072.802426	2233.772361	1700.744103	2186.0455	1843.016145
0.54_223.0286m/z	223.0285673	0.535283333	neg	1-Phenyl-5-mercaptotetrazole	70884	Unclassified	Unclassified	Unclassified			37.4	0	M+FA-H	C7H6N4S	-5.351986827	5884.4316	645.6984037	513.7701455	463.7290269	1036.672023	6005.34265	577.6722492	1208.75933	340.0723386
0.69_239.1151m/z	239.1151054	0.692166667	neg	Balenine	HMDB0005769	Organic acids and derivatives	Peptidomimetics	Hybrid peptides	*		37.4	0	M-H	C10H16N4O3	0.5891991	558.9119733	639.8355589	650.8365758	1005.637182	570.4279607	642.6399041	533.8056855	815.4980332	604.0123828
0.69_621.1745m/z	621.174515	0.692166667	neg	Icilin	64735	Unclassified	Unclassified	Unclassified			37.4	0	2M-H	C16H13N3O4	0.931633417	2070.450465	2023.199069	3173.306531	3189.376511	1991.281283	1724.020932	2208.918625	1748.179062	2221.176281
0.74_199.0339m/z	199.0338788	0.7359	neg	3-(Isothiocyanatomethyl)-1-methoxy-1H-indole	HMDB0038463	Organoheterocyclic compounds	Indoles and derivatives	Indoles			37.4	0.913	M-H2O-H	C11H10N2OS	1.540931532	2138.125456	1422.986961	3797.272231	1225.153922	4096.642755	1310.196333	3471.628252	3471.100622	5899.92401

0.82_344.0756m/z	344.0756155	0.819566667	neg	D-4'-Phosphopantothenate	3427	Organic acids and derivatives	Carboxylic acids and derivatives	Amino acids, peptides, and analogues	**	C03492	37.4	0	M+FA-H	C9H18NO8P	1.36899491	1595.921845	1031.782853	1348.012322	1178.280313	326.5276053	1111.414551	553.8023109	836.1574927	695.9872438
0.88_175.0612m/z	175.0612001	0.884633333	neg	Sequoyitol	87689	Organic oxygen compounds	Organooxygen compounds	Alcohols and polyols	*	C03365	37.4	0	M-H2O-H	C7H14O6	0.01592202	1939.388507	2075.352146	2173.664872	703.1599022	497.0608247	525.0947223	1641.964239	1633.617674	1628.967143
0.93_321.0828m/z	321.0827733	0.925866667	neg	Ranunculin	67033	Unclassified	Unclassified	Unclassified			37.4	0	M+FA-H	C11H16O8	0.19191639	115.6780744	262.5258909	158.4010486	171.6535752	208.217057	0.001403199	257.3940226	0.001403199	202.1254788
0.97_191.0129m/z	191.0128815	0.967616667	neg	ZAPA	69642	Unclassified	Unclassified	Unclassified			37.4	0	M+FA-H	C4H6N2O2S	-2.190284312	209.6886922	17.24794584	105.2094609	152.0958086	479.7514934	465.9811876	91.5112601	142.170783	0.001403199
1.16_243.0988m/z	243.0988321	1.160166667	neg	gamma-Glutamylproline	HMDB0029157	Organic acids and derivatives	Carboxylic acids and derivatives	Amino acids, peptides, and analogues	*		37.4	0	M-H	C10H16N2O5	0.765737457	116.1370836	306.8973349	223.7322384	193.6087814	202.8436781	198.5990884	250.4968062	153.1174297	222.7799388
1.20_347.0598m/z	347.0597839	1.202333333	neg	5-Fluorocyclohexadiene-cis,cis-1,2-diol-1-carboxylate	71220	Unclassified	Unclassified	Unclassified			37.4	0	2M-H	C7H7FO4	3.983334349	221.8158946	419.1246778	194.9868483	283.2004726	139.8832252	283.0658115	103.2035089	240.4109571	253.6680839
1.49_188.0024m/z	188.0023924	1.492933333	neg	Lanthionine ketimine	7085	Organic acids and derivatives	Carboxylic acids and derivatives	Amino acids, peptides, and analogues	*		37.4	0	M-H	C6H7NO4S	0.476920915	575.8972887	747.0832101	691.3294977	145.4176865	153.295771	120.0211703	298.8453614	239.473392	296.2858948
1.49_346.0580m/z	346.0579649	1.492933333	neg	Adenosine 2'-phosphate	62385	Organic oxygen compounds	Organooxygen compounds	Carbohydrates and carbohydrate conjugates	*	C00946	37.4	0	M-H	C10H14N5O7P	6.214570501	1035.429434	1005.41895	784.4884152	1088.479399	961.9280405	1081.569638	648.2483283	764.8106732	895.9798767
1.62_491.0278m/z	491.0277767	1.615883333	neg	(5'-(but-3-en-1-yn-1-yl)-[2,2'-bithiophen]-5-yl)methanol	LMFA12000361	Lipids and lipid-like molecules	Fatty Acyls	Oxygenated hydrocarbons			37.4	0	2M-H	C13H10OS2	0.892713558	697.742715	908.3518214	901.9514857	1102.113686	1120.481872	1388.949714	1074.548715	1097.077049	1045.363528
1.63_303.0101m/z	303.010118	1.631516667	neg	D-Glucono-1,5-lactone 6-phosphate	3388	Organic oxygen compounds	Organooxygen compounds	Carbohydrates and carbohydrate conjugates	**	C01236	37.4	0	M+FA-H	C6H11O9P	-8.346212698	2022.939343	2293.817861	2631.497128	2534.194022	2555.526	2528.555465	3418.540518	2740.430635	2577.745069
1.68_240.0186m/z	240.0185752	1.68365	neg	Carbocysteine sulfoxide	1527	Unclassified	Unclassified	Unclassified			37.4	0	M+FA-H	C5H9NO5S	1.173969284	0.001403199	46.52421378	83.39754583	139.3228328	137.9096409	67.82733988	29.20481829	37.68671799	68.71229738
1.98_327.0852m/z	327.085165	1.980516667	neg	Phenylpyruvic acid	HMDB0000205	Benzenoids	Benzene and substituted derivatives	Phenylpyruvic acid derivatives	***	C00166	37.4	0	2M-H	C9H8O3	-6.848050195	2056.249577	2784.758415	2670.217946	2542.998571	3130.777403	4372.648236	2670.20282	2183.231395	1843.328682
10.46_585.2297m/z	585.2297233	10.45966667	neg	Desethylenenorfloxacin	1604	Unclassified	Unclassified	Unclassified			37.4	0	2M-H	C14H16FN3O3	3.173597807	14041.1393	15959.87232	19286.3372	16621.79598	18335.76288	15280.17398	15025.42977	18453.16851	17153.83952
12.32_379.1563m/z	379.1563006	12.31543333	neg	9-Fluoro-16alpha-hydroxyandrost-4-ene-3,11,17-trione	70598	Unclassified	Unclassified	Unclassified			37.4	0	M+FA-H	C19H23FO4	0.180539479	11471.42475	7651.931951	7827.770369	6350.220937	6073.732479	6462.574186	8966.27532	16002.64376	7549.109534
13.42_269.1312m/z	269.1312017	13.42113333	neg	2'-Aminoacetophenone	88578	Organic oxygen compounds	Organooxygen compounds	Carbonyl compounds	*		37.4	0	2M-H	C8H9NO	6.108998065	1566.065913	1301.634846	1291.752099	1571.612146	1551.702947	1240.85919	1900.671695	2074.767214	1308.898492
14.66_351.2518m/z	351.251782	14.6619	neg	5,8,11-eicosatrienoic acid	34827	Lipids and lipid-like molecules	Fatty Acyls	Fatty acids and conjugates	*		37.4	0	M+FA-H	C20H34O2	-7.514079156	71935.30716	62757.61335	67240.54177	47565.04356	58792.3698	55655.67928	64126.16304	57746.67707	59946.89703
15.09_141.0208m/z	141.0207672	15.08646667	neg	2-Formylglutarate	63877	Unclassified	Unclassified	Unclassified			37.4	0	M-H2O-H	C6H8O5	8.966491059	196.3827625	175.3239103	208.9960283	152.2390938	128.2652492	102.0299308	166.6291283	150.1164902	212.9883608
2.00_216.1071m/z	216.1070926	2.004766667	neg	2,5-Dihydro-4,5-dimethyl-2-(2-methylpropyl)thiazole	HMDB0037159	Organoheterocyclic compounds	Azolines	Thiazolines			37.4	0	M+FA-H	C9H17NS	4.202896155	13698.13811	12614.7905	13749.16329	8744.705704	7755.471886	7848.493601	8981.800627	11103.99013	9508.687684
2.00_361.0343m/z	361.0343341	2.004766667	neg	CAY10562	45271	Unclassified	Unclassified	Unclassified			37.4	0	2M-H	C8H7NO2S	5.832504608	5342.245921	4496.414032	4687.877732	2483.663473	2191.829026	2300.681441	2561.808571	3344.198944	2873.78068
2.07_222.9920m/z	222.9919567	2.070366667	neg	Allitridin	HMDB0031154	Organosulfur compounds	Organic trisulfides	Unclassified	*		37.4	6.51	M+FA-H	C6H10S3	-3.986151455	7674.336537	9259.940219	9808.067469	2599.555947	2849.098546	2379.42334	7930.832756	7943.637556	7896.513119
2.07_392.0555m/z	392.0554703	2.070366667	neg	5'-Hydroxypiroxicam	HMDB0014096	Organoheterocyclic compounds	Benzothiazines	Unclassified	*		37.4	0	M+FA-H	C15H13N3O5S	-0.933806728	13311.79634	14836.90392	14159.46426	13995.94488	15720.78584	14178.33245	16510.45033	14665.04649	14282.93508
2.30_547.0847m/z	547.0846914	2.303733333	neg	Athyriol	68096	Unclassified	Unclassified	Unclassified			37.4	0	2M-H	C14H10O6	-6.400804875	1214.516854	1096.447491	1380.809665	1990.297281	2571.253802	2339.353349	1265.084076	1899.782367	1661.309018
2.36_456.9933m/z	456.9932838	2.36465	neg	DIMETHOATE	44564	Unclassified	Unclassified	Unclassified			37.4	0	2M-H	C5H12NO3PS2	2.874322364	7518.617379	7498.233907	7323.441721	13042.02585	13651.65998	14015.62527	10230.75514	11892.57914	10918.44879
2.68_365.0186m/z	365.0186341	2.680233333	neg	ACEPHATE	44455	Unclassified	Unclassified	Unclassified			37.4	0	2M-H	C4H10NO3PS	5.761756754	2020.408475	1983.672979	2120.377467	298.2753641	368.7995046	240.7480561	911.6744179	1230.458299	954.8207266
2.86_263.0399m/z	263.0398956	2.864816667	neg	3-methyl sulfolene	96259	Organoheterocyclic compounds	Dihydrothiophenes	Unclassified			37.4	0	2M-H	C5H8O2S	-6.926670685	10324.85496	10433.05314	11592.235	16911.21022	15731.30157	16082.31538	13071.57322	13617.75879	12497.04763
3.44_411.0812m/z	411.0812435	3.440016667	neg	Homoisocitrate	3341	Unclassified	Unclassified	Unclassified			37.4	0	2M-H	C7H10O7	7.800736805	2395.804704	2841.728037	3349.542775	729.2979477	437.229032	653.9222099	1856.738465	2328.352727	1985.370171
3.69_180.0289m/z	180.0289028	3.685866667	neg	2-Methyl-3-hydroxy-5-formylpyridine-4-carboxylate	58601	Organoheterocyclic compounds	Pyridines and derivatives	Pyridinecarboxylic acids and derivatives	**	C06050	37.4	0	M-H	C8H7NO4	-7.338258421	1427.566599	1628.519154	1630.917232	2719.230545	2495.121178	1799.379994	1848.759628	2247.325406	2119.986921
3.87_385.0910m/z	385.0910433	3.868533333	neg	Vulgaxanthin-II	67072	Organic acids and derivatives	Carboxylic acids and derivatives	Amino acids, peptides, and analogues	*	C08569	37.4	0	M+FA-H	C14H16N2O8	6.395320113	614.6490481	245.3216652	592.4265439	0.001403199	18.87577667	78.35066733	1179.909811	1265.502161	1137.893813
3.89_357.0262m/z	357.0262335	3.8906	neg	Distemonanthin	51801	Lipids and lipid-like molecules	Polyketides	Flavonoids			37.4	0	M-H	C17H10O9	2.871419881	522.7440495	568.8881485	650.5905046	134.863871	72.56584863	260.3457139	418.8524821	683.3319721	498.1740499
3.96_277.0308m/z	277.0308057	3.957583333	neg	D-Ribitol 5-phosphate	63137	Unclassified	Unclassified	Unclassified			37.4	0	M+FA-H	C5H13O8P	-9.486313294	4504.213954	4791.827335	5042.154648	8539.748186	9058.404035	8921.102998	4977.804805	5554.66714	5576.930366
3.96_480.9934m/z	480.9933833	3.957583333	neg	Fosthietan	72750	Unclassified	Unclassified	Unclassified			37.4	0	2M-H	C6H12NO3PS2	2.937782263	1748.667367	1496.398679	1743.570602	3222.741234	4093.318397	3393.057327	1869.477829	1871.206249	2127.798408
4.00_563.1022m/z	563.10223	4.002233333	neg	5,6,7-Trihydroxyflavone 7-(6''-malonylglucoside)	LMPK12111089	Unclassified	Unclassified	Unclassified			37.4	0	M+FA-H	C24H22O13	-3.886606889	5238.374907	5186.963906	5795.154168	4695.580747	3741.736561	4196.994706	5804.382181	5757.670217	5113.72578
4.09_280.0810m/z	280.0809929	4.091783333	neg	7-Methylxanthosine	HMDB0301918	Unclassified	Unclassified	Unclassified			37.4	0	M-H2O-H	C11H15N4O6+	-1.08727011	1206.224745	1173.649738	1354.289247	1021.018728	974.5834285	1154.160702	1171.968277	1347.817993	1076.809493
4.14_643.0924m/z	643.0924194	4.136366667	neg	7,7'-Dihydroxy-6,8'-bicoumarin	89369	Phenylpropanoids and polyketides	Coumarins and derivatives	Hydroxycoumarins			37.4	0	2M-H	C18H10O6	6.551378052	205.5433914	453.5789056	373.0628218	3865.734742	2863.675867	2859.053281	0.001403199	0.001403199	0.001403199
4.16_321.1194m/z	321.1193999	4.15895	neg	3'-Hydroxytrimethoprim	2961	Unclassified	Unclassified	Unclassified			37.4	0	M+FA-H	C13H16N4O3	-3.778627979	859.9695692	915.5963085	890.3725709	1335.210048	1302.237732	1246.633272	1313.856919	858.9767675	848.0508079
4.18_543.1223m/z	543.1222876	4.181333333	neg	5-Carboxy-2'-deoxyuridine	HMDB0060774	Nucleosides, nucleotides, and analogues	Pyrimidine nucleosides	Pyrimidine 2'-deoxyribonucleosides	*		37.4	0	2M-H	C10H12N2O7	1.217579858	1103.560294	810.2368215	884.5158454	470.2296198	540.4210372	629.702552	882.5100743	634.4987335	772.0143085
4.30_457.1151m/z	457.1150679	4.300033333	neg	N-Tris[hydroxymethyl]methyl-2-aminoethanesulfonic acid [TES]	44793	Unclassified	Unclassified	Unclassified			37.4	0	2M-H	C6H15NO6S	-3.649860794	3539.886209	2942.901667	2816.797357	3734.209355	3128.768751	3367.572063	2816.37262	3684.070015	2840.186818
4.32_380.0789m/z	380.0788564	4.3224	neg	4-Hydroxy-6-methylpretetramide	63774	Unclassified	Unclassified	Unclassified			37.4	0	M-H	C20H15NO7	3.361433188	1224.735145	1173.369153	1380.042364	1248.838346	870.9867128	1321.396726	1164.339078	1581.39275	1042.734357
4.34_392.0722n	391.0649537	4.344733333	neg	5,7,3',4',5'-Pentahydroxy-3,6,8-trimethoxyflavone	51769	Unclassified	Unclassified	Unclassified			37.4	0	M-H, M+FA-H	C18H16O10	-5.399350386	16379.90531	17068.20945	17211.53006	6398.746331	5347.094175	5566.352038	13564.09284	14513.0285	14769.08673
4.39_394.0877n	393.0804589	4.3893	neg	9-Hydroxy-4-methoxypsoralen 9-glucoside	93653	Phenylpropanoids and polyketides	Coumarins and derivatives	Coumarin glycosides			37.4	0	M-H, M+FA-H	C18H18O10	-5.738333162	5265.760768	4916.598093	4628.995528	8261.803975	8397.713207	8034.973363	3416.667081	5290.722035	4647.94447
4.41_263.0287m/z	263.028706	4.41115	neg	Oxalureate	63388	Unclassified	Unclassified	Unclassified			37.4	0	2M-H	C3H4N2O4	6.700818884	31590.98474	36407.16676	35856.5987	16280.84971	14937.87342	15658.68672	28649.25634	29491.2565	29115.54474
4.41_375.0813m/z	375.0813331	4.41115	neg	SOTS-1	64678	Unclassified	Unclassified	Unclassified			37.4	0	M+FA-H	C16H14N2O6	-6.228452633	10006.31177	9982.047707	8294.450567	7028.098287	4985.629269	6968.972734	9881.409021	10991.40762	10458.49019
4.43_287.1251m/z	287.1251071	4.432683333	neg	N-Hydroxypentobarbital	1765	Unclassified	Unclassified	Unclassified			37.4	0	M+FA-H	C11H18N2O4	1.020798118	2219.990029	2708.458799	2287.457313	3131.416259	3214.59801	3921.24626	2078.779066	2544.085104	2349.494068
4.45_465.1013m/z	465.101325	4.454616667	neg	benfotiamine	HMDB0248951	Unclassified	Unclassified	Unclassified			37.4	0	M-H	C19H23N4O6PS	2.165472494	257.3515081	273.0530338	251.3571221	735.9299923	247.7759647	492.0796659	902.3215951	1290.908652	1154.815983
4.50_347.1255m/z	347.1254509	4.49925	neg	1-(1,2,3,4,5-Pentahydroxypent-1-yl)-1,2,3,4-tetrahydro-beta-carboline-3-carboxy	HMDB0012492	Alkaloids and derivatives	Harmala alkaloids	Unclassified	*		37.4	0	M-H2O-H	C17H22N2O7	1.614041176	603.5203259	620.7139156	479.7963915	734.7389839	525.4499805	462.5755021	709.6534286	725.6437419	734.0776033
4.52_363.1181m/z	363.1181333	4.521733333	neg	16-iodo-hexadecanoic acid	LMFA01090039	Lipids and lipid-like molecules	Fatty Acyls	Fatty Acids and Conjugates			37.4	0	M-H2O-H	C16H31IO2	-2.365177459	1217.728221	1571.236332	1516.375634	1085.67522	865.0469913	914.5684794	820.0740311	935.9213848	838.8239585
4.52_405.2134m/z	405.2134179	4.521733333	neg	Kiwiionoside	93346	Lipids and lipid-like molecules	Prenol lipids	Terpene glycosides	*		37.4	0	M-H	C19H34O9	1.013477634	1309.225606	1125.884995	1322.861894	65.22519688	77.78831771	0.001403199	798.1477498	1056.185592	768.6920891
4.54_497.0705m/z	497.0704783	4.54425	neg	5-Carboxypyranocyanidin 3-O-beta-glucopyranoside	47199	Unclassified	Unclassified	Unclassified			37.4	0	M-H2O-H	C24H20O13	-4.013342329	1016.613685	980.7170805	1001.209189	3904.097188	3259.56219	3377.494218	34.77190363	112.1238602	102.1867951
4.57_242.0171n	287.0155368	4.5668	neg	Inositol cyclic phosphate	6020	Organic acids and derivatives	Organic phosphoric acids and derivatives	Unclassified	*	C04299	37.4	0	M-H, M+FA-H	C6H11O8P	-8.576921295	4041.044011	5138.089622	4728.730111	918.9781702	1250.923886	1177.239457	778.8651417	551.3961331	565.4927709
4.61_399.1961m/z	399.1961248	4.611883333	neg	Fluorometholone 17-acetate	70218	Unclassified	Unclassified	Unclassified			37.4	0	M-H2O-H	C24H31FO5	-3.793301335	63113.33485	63225.37564	65134.24773	49085.18273	49447.06297	42055.96318	64778.29671	59276.72313	72780.47369
4.61_403.1977m/z	403.1977119	4.611883333	neg	1-Octen-3-yl primeveroside	88859	Lipids and lipid-like molecules	Fatty Acyls	Fatty acyl glycosides	*		37.4	0	M-H2O-H	C19H34O10	0.842638505	1834.50673	2134.197211	1642.187325	721.0736987	656.302454	640.2712969	1793.535409	1556.174175	1994.472973
4.63_531.1360m/z	531.1360408	4.632666667	neg	Carbamazepine-o-quinone	71260	Organoheterocyclic compounds	Benzazepines	Dibenzazepines	**	C16606	37.4	0	2M-H	C15H10N2O3	9.457863231	1310.339122	1006.078794	1005.131082	377.6574596	606.5650674	511.2046749	1184.99355	1045.130334	788.9740922
4.75_293.1008m/z	293.1008169	4.747466667	neg	Deoxyanisatin	69293	Unclassified	Unclassified	Unclassified			37.4	0	M-H2O-H	C15H20O7	-7.192533355	1174.024945	879.3203954	797.8126354	825.1531094	810.7558927	691.9422561	437.5715191	568.335044	250.3245665
4.75_493.2034m/z	493.2034052	4.747466667	neg	Fluocinonide	2747	Lipids and lipid-like molecules	Steroids and steroid derivatives	Pregnane steroids		C07007	37.4	0	M-H	C26H32F2O7	-1.878104103	5013.781123	5991.256114	6915.851553	4660.58542	4053.816884	3627.71848	4644.566769	5609.88805	5152.88454
4.79_313.1660m/z	313.165984	4.792316667	neg	Bisacurone epoxide	93181	Lipids and lipid-like molecules	Prenol lipids	Sesquiterpenoids	*		37.4	0	M+FA-H	C15H24O4	1.200283855	1919.99786	1663.463766	2146.411223	2378.933284	2165.511768	2177.196712	1636.524189	1959.162834	2120.926564
4.79_352.0938m/z	352.093774	4.792316667	neg	CI Pigment Red 3	73076	Unclassified	Unclassified	Unclassified			37.4	0	M+FA-H	C17H13N3O3	-0.391382716	3128.649275	3181.129022	3732.538036	5960.780439	5765.143382	5676.909386	2722.86142	3066.796467	3009.323451
4.79_413.0845m/z	413.084518	4.792316667	neg	Vitexin	LMPK12110194	Lipids and lipid-like molecules	Polyketides	Flavonoids	***		37.4	0	M-H2O-H	C21H20O10	-7.608551005	377.58206	631.2109744	581.4311934	1193.846826	1053.88643	1089.009709	500.382422	567.2133408	553.6684388
4.88_335.1350m/z	335.1349914	4.880083333	neg	Benomyl	HMDB0031767	Organoheterocyclic compounds	Benzimidazoles	2-benzimidazolylcarbamic acid esters	*	C10896	37.4	0	M+FA-H	C14H18N4O3	-3.798007675	1960.10272	2082.776131	1988.189287	3041.017512	2793.268662	2973.288407	2201.251204	2407.319899	2094.074854
4.88_527.0875m/z	527.0875394	4.880083333	neg	alpha-D-Galactopyranuronosyl-(1->4)-alpha-D-galactopyranuronosyl-(1->4)-D-g	HMDB0039751	Organic oxygen compounds	Organooxygen compounds	Carbohydrates and carbohydrate conjugates			37.4	0	M-H2O-H	C18H26O19	-2.651637688	2951.476275	3600.195565	3511.652529	5678.739961	5063.023798	4185.554832	4740.910159	5183.262044	3738.246148
4.95_771.1084m/z	771.1084184	4.94705	neg	Lithol Rubine	HMDB0041567	Benzenoids	Naphthalenes	Naphthalenecarboxylic acids and derivatives			37.4	0	2M-H	C18H14N2O6S	1.52883033	2395.093625	2240.480257	2371.332177	0.001403199	0.001403199	0.001403199	726.7642849	1442.958457	408.6700602
4.97_332.1637m/z	332.1636713	4.969683333	neg	Zolmitriptan	44026	Organoheterocyclic compounds	Indoles and derivatives	Tryptamines and derivatives		C07218	37.4	0	M+FA-H	C16H21N3O2	7.283389319	6280.036405	6013.548526	6539.244185	4158.457409	3831.448051	4064.321185	6002.410672	5655.862474	5054.026603
5.01_453.1391m/z	453.1390659	5.014716667	neg	Ginkgolide A	41244	Lipids and lipid-like molecules	Prenol lipids	Sesquiterpenoids	*		37.4	0	M+FA-H	C20H24O9	-2.864920119	531.1486383	1319.164197	1228.129742	1902.308144	1706.474672	1806.502792	968.2209345	1228.842854	810.6967125
5.06_551.0490m/z	551.0490138	5.05975	neg	5-(4-Acetoxybut-1-ynyl)-2,2'-bithiophene	66192	Organoheterocyclic compounds	Bi- and oligothiophenes	Unclassified		C04485	37.4	0	2M-H	C14H12O2S2	0.990802089	3524.133591	3249.781849	3429.014658	6066.722921	6053.936987	6218.972424	4840.546215	4841.887747	5342.848926
5.10_499.2189m/z	499.2188754	5.1031	neg	6S,9R-Dihydroxy-4,7E-megastigmadien-3-one 9-[apiosyl-(1->6)-glucoside]	HMDB0029773	Lipids and lipid-like molecules	Fatty Acyls	Fatty acyl glycosides	*		37.4	0	M-H2O-H	C24H38O12	0.752261332	1120.795898	982.2094091	851.3538428	1110.184011	1474.178848	1766.982343	1071.889825	1015.802508	1049.473946
5.16_323.0777m/z	323.0776891	5.155116667	neg	Glucocaffeic acid	HMDB0034313	Organic oxygen compounds	Organooxygen compounds	Carbohydrates and carbohydrate conjugates			37.4	0	M-H2O-H	C15H18O9	1.309661398	1193.50018	1848.600988	1573.890717	703.5710626	792.1837344	774.1913488	1603.251005	1563.843853	1707.890215
5.16_515.0811m/z	515.0811313	5.155116667	neg	4',5-Dihydroxy-3,3'-dimethoxy-6,7-methylenedioxyflavone 4'-glucuronide	92577	Phenylpropanoids and polyketides	Flavonoids	Flavonoid glycosides	*		37.4	0	M-H2O-H	C24H22O14	-3.712727023	3989.883056	5586.27193	4662.61173	2540.634174	2446.384862	2220.865548	3838.070034	4412.613056	3967.95392
5.20_209.0822m/z	209.0822233	5.20005	neg	3-Hydroxy-4-phenylbutan-2-one	HMDB0032336	Benzenoids	Benzene and substituted derivatives	Unclassified	*		37.4	0	M+FA-H	C10H12O2	1.772308041	405.8506349	390.3962293	366.4586146	247.8080958	210.3834322	281.4154046	421.5374993	236.0703432	430.4982561
5.22_533.1666m/z	533.1666387	5.222166667	neg	PD 98059	64632	Unclassified	Unclassified	Unclassified			37.4	0	2M-H	C16H13NO3	-9.681082087	1213.940057	1764.590139	1215.744879	3473.853423	3165.677719	3441.938273	1099.60821	1032.271475	1156.415487
5.22_570.0661m/z	570.0660885	5.222166667	neg	Guanosine diphosphofucose	4187	Nucleosides, nucleotides, and analogues	Purine nucleotides	Purine nucleotide sugars	**	C00325	37.4	0	M-H2O-H	C16H25N5O15P2	2.87091311	744.8984946	780.6604566	963.8348145	970.3616143	592.6076344	1231.031926	1162.139903	1669.546361	1376.170095
5.30_383.1536m/z	383.153577	5.302883333	neg	Dehydrozingerone	71922	Phenylpropanoids and polyketides	Cinnamic acids and derivatives	Hydroxycinnamic acids and derivatives	*		37.4	0	2M-H	C11H12O3	9.28002833	7637.534851	6614.435043	6402.081274	10074.043	13085.25584	12703.51435	10379.19243	7511.433048	10685.466
5.30_535.2163m/z	535.2162982	5.302883333	neg	EUGENYL BENZOATE	44605	Phenylpropanoids and polyketides	Depsides and depsidones	Unclassified	*		37.4	0	2M-H	C17H16O3	6.873767113	1900.481249	1348.01366	1446.522602	1660.30796	2115.259259	2697.719205	1752.746968	1558.863174	1785.703561
5.32_345.1203m/z	345.1202958	5.320233333	neg	Diaziquone	HMDB0251150	Unclassified	Unclassified	Unclassified			37.4	0	M-H2O-H	C16H20N4O6	-0.404841707	1159.646846	1185.197619	1232.344399	1165.076408	1736.106749	1588.508483	1489.816029	1299.672399	1964.797226
5.34_477.2140m/z	477.2139689	5.3394	neg	Melleolide B	HMDB0038787	Lipids and lipid-like molecules	Prenol lipids	Sesquiterpenoids	*		37.4	0	M+FA-H	C24H32O7	2.227247439	1703.90621	2133.929526	1973.70716	2916.252107	2801.32268	2316.440406	1767.529244	2245.57052	2728.140439
5.41_337.1383m/z	337.1383254	5.406433333	neg	Norepinephrine (noradrenaline)	63	Unclassified	Unclassified	Unclassified			37.4	0	2M-H	C8H11NO3	-6.460428137	71692.58356	81884.25695	77415.72144	146909.0946	136738.2326	136593.8848	105367.9693	112587.5384	89006.21445
5.43_494.2738m/z	494.2737677	5.428733333	neg	Condelphine	67106	Unclassified	Unclassified	Unclassified			37.4	0	M+FA-H	C25H39NO6	-4.837075234	1599.496344	1128.056857	1599.356282	2702.011845	2213.567838	1858.415749	1165.318256	2217.996846	1141.737186
5.53_611.0583m/z	611.0582925	5.525416667	neg	UDP-alpha-D-galacto-1,4-furanose	66033	Unclassified	Unclassified	Unclassified			37.4	0	M+FA-H	C15H24N2O17P2	8.954980799	28867.62264	25368.16944	23993.73686	9491.220046	11958.89987	9436.268931	20304.85786	20285.22805	26801.20294
5.56_599.2247m/z	599.2247344	5.56185	neg	23-Hydroxyphysalolactone	HMDB0031388	Lipids and lipid-like molecules	Steroids and steroid derivatives	Steroid lactones	*		37.4	0	M+FA-H	C28H39ClO9	-3.119584299	18104.89307	15153.63636	15402.39422	22307.68665	26193.42493	23665.20664	15041.54045	17889.15498	14466.29584
5.60_323.0646m/z	323.0646065	5.602	neg	2-Oxo-5-methylthiopentanoic acid	64490	Unclassified	Unclassified	Unclassified			37.4	0	2M-H	C6H10O3S	5.407967361	668.9324811	639.5145832	895.609612	1681.42593	1822.450275	1600.47826	542.839147	748.208839	635.0536309
5.70_725.1244m/z	725.1243892	5.701283333	neg	Flufenacet	HMDB0252330	Unclassified	Unclassified	Unclassified			37.4	0	2M-H	C14H13F4N3O2S	-1.728958814	850.9429017	1000.405225	1031.080514	833.9180988	717.9088818	428.7364001	1417.726292	1796.515657	2135.767964
5.71_263.0926m/z	263.0926048	5.709933333	neg	Dihydroeuparin	67164	Unclassified	Unclassified	Unclassified			37.4	0	M+FA-H	C13H14O3	0.493638934	872.1355554	955.59829	722.8666186	611.6578242	984.7639927	966.7608865	846.0194407	957.4219742	1593.809828
5.74_313.1660m/z	313.1659679	5.744566667	neg	Deoxydihydro-artemisinin	HMDB0060570	Organoheterocyclic compounds	Dioxolopyrans	Unclassified	*		37.4	0	M+FA-H	C15H24O4	1.140446258	709.7269901	825.7011556	690.2155541	535.1899117	575.7590619	630.6479154	438.6545874	441.6762261	601.6581544
5.81_553.1026m/z	553.1025571	5.806016667	neg	Fluorescein monoglucuronide	2756	Unclassified	Unclassified	Unclassified			37.4	0	M+FA-H	C26H20O11	7.464571882	33358.40639	34673.12826	35613.34234	56636.10359	44673.46449	47018.23617	5417.407661	5334.605726	5388.229599
5.83_279.0382m/z	279.0382307	5.825	neg	Uracil 5-carbaldehyde	71607	Unclassified	Unclassified	Unclassified			37.4	0	2M-H	C5H4N2O3	4.010639979	3402.625289	3284.38187	4051.958489	4215.813928	3282.16923	3389.725482	2633.145365	2617.433875	2413.471278
5.98_349.1872m/z	349.1872139	5.9829	neg	Citrulline	16	Organic acids and derivatives	Carboxylic acids and derivatives	Amino acids, peptides, and analogues	**	C00327	37.4	0	2M-H	C6H13N3O3	8.874456704	1206.938241	1302.058146	1719.87559	900.6826032	998.2905478	1206.41254	1390.413565	1484.195606	1209.731688
6.17_407.0962m/z	407.0962484	6.171316667	neg	Khellol glucoside	67349	Unclassified	Unclassified	Unclassified			37.4	0	M-H	C19H20O10	-5.19966462	17321.48485	18383.28161	18332.48851	7152.87252	7607.936639	8419.687259	13420.33838	15484.32901	16479.61274
6.26_569.0976m/z	569.0975985	6.261966667	neg	IRIGINOL HEXAACEATATE	43809	Unclassified	Unclassified	Unclassified			37.4	0	M-H	C27H22O14	6.875242902	70526.85347	64550.66996	71364.84397	83306.91321	74402.19563	84017.49468	54631.29199	60980.81257	59030.40335
6.32_413.2550m/z	413.2549541	6.320716667	neg	beta-Cortol	HMDB0005821	Organic oxygen compounds	Organooxygen compounds	Carbohydrates and carbohydrate conjugates	**	C01235	37.4	0	M+FA-H	C21H36O5	1.295122532	1043.523727	1477.177083	1488.657514	536.5312397	526.2158055	567.1345862	995.9425941	1036.425437	876.1939299
6.33_437.1620m/z	437.1619683	6.331216667	neg	Kanzonol T	86579	Phenylpropanoids and polyketides	Isoflavonoids	Isoflavans	*		37.4	0	M-H	C25H26O7	3.175959915	8005.584115	9798.669935	8582.656146	12004.55862	11272.12811	10135.89106	7527.798387	8216.558168	8749.871043
6.49_277.1059m/z	277.1058563	6.494283333	neg	Boc-Asn	65591	Unclassified	Unclassified	Unclassified			37.4	0	M+FA-H	C9H16N2O5	7.461470847	2035.607413	2224.026926	2306.00419	1481.769948	1308.965471	1395.535437	931.244115	1307.36158	1226.701031
6.49_397.1482m/z	397.1482159	6.494283333	neg	Coriandrone D	86629	Organoheterocyclic compounds	Benzopyrans	2-benzopyrans	*		37.4	0	M+FA-H	C18H24O7	-6.219198138	13803.97288	14644.66816	15873.93617	6094.58071	5832.342577	6056.097109	14717.69023	16105.56603	15723.72044
6.51_518.2609m/z	518.2608945	6.509966667	neg	Ximelagatran	HMDB0015603	Organic acids and derivatives	Carboxylic acids and derivatives	Amino acids, peptides, and analogues			37.4	0	M+FA-H	C24H35N5O5	-2.382695519	610.1613972	703.2423969	892.5917756	1137.31142	1410.186721	1359.041692	3261.404444	2803.424867	2950.577081
6.57_235.0953m/z	235.0952975	6.570233333	neg	Threoninyl-Alanine	85947	Organic acids and derivatives	Carboxylic acids and derivatives	Amino acids, peptides, and analogues	*		37.4	0	M+FA-H	C7H14N2O4	9.140949979	10120.56719	10571.32578	11147.93929	9254.94653	9192.14343	9022.852992	5973.752876	7321.994712	6545.658329
6.62_535.1025m/z	535.1024501	6.61635	neg	Homocystine	4189	Organic acids and derivatives	Carboxylic acids and derivatives	Amino acids, peptides, and analogues	**	C01817	37.4	0	2M-H	C8H16N2O4S2	-1.065943996	3376.490965	3623.016521	2855.642852	6815.860122	6371.992552	6413.109605	5125.91934	6386.195406	6058.340385
6.63_387.1280m/z	387.1279551	6.63275	neg	Sphalleroside A	88694	Organic oxygen compounds	Organooxygen compounds	Carbohydrates and carbohydrate conjugates			37.4	0	M+FA-H	C16H22O8	-5.0140377	5791.647438	5955.269413	5228.977164	2598.696943	2637.881618	2241.705887	6508.828838	7683.540085	7919.769789
6.64_411.1854m/z	411.1853586	6.644916667	neg	Isoeugenyl acetate	88383	Benzenoids	Phenol esters	Unclassified	*		37.4	0	2M-H	C12H14O3	9.816828359	1956.661096	2027.331311	1785.158635	2411.5329	2206.786405	1657.04895	1808.903545	2240.339608	2637.512411
6.66_463.1589m/z	463.1589239	6.663683333	neg	Euparotin acetate	67694	Unclassified	Unclassified	Unclassified			37.4	0	M+FA-H	C22H26O8	-4.894589746	786.1009569	783.5476223	929.9822095	2107.305887	1781.618822	1645.856169	977.5350377	1151.254539	1053.800157
6.69_289.1060m/z	289.1059638	6.69475	neg	Hydroxyprolyl-Hydroxyproline	85771	Organic acids and derivatives	Carboxylic acids and derivatives	Amino acids, peptides, and analogues	*		37.4	0	M+FA-H	C10H16N2O5	7.534716661	1830.047037	2365.941788	2258.493687	2818.949666	2771.518344	2348.611868	1877.995366	2318.731312	2549.900937
6.69_357.0936m/z	357.0936338	6.69475	neg	Pteroic acid	66690	Unclassified	Unclassified	Unclassified			37.4	0	M+FA-H	C14H12N6O3	-5.31038938	596.2383873	1076.330858	675.8941448	1259.231442	1096.582357	1036.062882	820.6901549	830.7657318	868.0607879
6.73_261.1708m/z	261.1707568	6.72945	neg	xi-5-Hydroxydodecanoic acid	94705	Lipids and lipid-like molecules	Fatty Acyls	Fatty acids and conjugates	*		37.4	0	M+FA-H	C12H24O3	0.042822793	178.4081319	119.8414856	297.1725914	152.8135729	167.8946912	31.79274988	36.27764463	196.588841	79.67381859
6.79_1017.3157m/z	1017.31573	6.7874	neg	Alpha-cyclodextrin	85154	Unclassified	Unclassified	Unclassified			37.4	0	M+FA-H	C36H60O30	0.603520953	9317.96138	8836.856338	10950.0629	1503.053586	556.1360861	1885.675781	5524.538	7187.360109	6559.204779
6.80_375.1486m/z	375.1486383	6.799583333	neg	Glycyl-H-1152	45671	Unclassified	Unclassified	Unclassified			37.4	0	M-H	C18H24N4O3S	-2.650227423	34622.92953	32570.68721	33657.05344	12401.07441	11153.53483	13364.76086	22346.55696	23135.2465	21574.6575
6.86_819.0528m/z	819.0527884	6.86375	neg	6-Hydroxyluteolin 6,3'-dimethyl ether 7-sulfate	49682	Lipids and lipid-like molecules	Polyketides	Flavonoids			37.4	0	2M-H	C17H14O10S	-1.79313984	5236.456044	5525.055335	3647.022293	4512.938315	5555.926176	4161.689014	4398.382353	4898.974621	4327.715279
6.88_316.1589m/z	316.1588805	6.8759	neg	1-NA-PP1	96473	Unclassified	Unclassified	Unclassified			37.4	0	M-H	C19H19N5	6.65667529	514.6043066	942.8043531	702.2046425	1042.962899	1061.507776	818.9102708	691.4371195	914.6174605	495.833326
6.97_316.1590m/z	316.1590029	6.970716667	neg	Pitrazepin	69707	Unclassified	Unclassified	Unclassified			37.4	0	M-H	C19H19N5	7.042743448	612.5421496	895.6137634	701.9369551	1472.394647	1099.555417	1316.73433	570.7759292	667.9957765	810.1728889
7.09_226.1450m/z	226.1450271	7.086816667	neg	2-Butyl-5-methyl-4-propyloxazole	92700	Organoheterocyclic compounds	Azoles	Oxazoles			37.4	0	M+FA-H	C11H19NO	0.883212488	700.5958766	701.5762985	653.1943785	558.8442968	542.0738022	453.3393866	955.0613066	1075.30504	1056.013388
7.10_451.1954m/z	451.1954168	7.097333333	neg	3-[(2-Methyl-3-furanyl)thio]-4-heptanone	92126	Organosulfur compounds	Thioethers	Aryl thioethers			37.4	0	2M-H	C12H18O2S	-6.210351955	2320.007808	2295.321412	2480.663917	2202.274963	2404.281648	1783.749464	1538.099284	1894.868622	1738.267619
7.10_503.1153m/z	503.1153019	7.097333333	neg	2H-1-Benzopyran-2-one, 6-(1,2-dihydroxyethyl)-7-hydroxy-8-methoxy-	1212	Unclassified	Unclassified	Unclassified			37.4	0	2M-H	C12H12O6	-8.326933064	21358.40439	21147.07874	19990.02432	28420.04797	28982.48808	26065.02155	18074.34355	20117.68754	18531.19242
7.11_461.2398m/z	461.2397817	7.107783333	neg	Forasartan	85498	Organoheterocyclic compounds	Pyridines and derivatives	Phenylpyridines			37.4	0	M+FA-H	C23H28N8	-5.078907221	15483.35833	15117.01321	14709.21142	14597.82997	16307.06912	12766.92088	13029.85402	15375.92473	14096.64558
7.34_455.1542m/z	455.1541758	7.33585	neg	4'-Hydroxy-4-biphenylacetic acid	2678	Unclassified	Unclassified	Unclassified			37.4	0	2M-H	C14H12O3	9.127954178	1030.478928	1368.550766	951.5126071	1980.250588	1583.439362	1532.794212	714.9635893	993.2136768	963.0040708
7.35_405.2647m/z	405.2646738	7.353466667	neg	17beta-Hydroxy-6beta-methyl-5alpha-androstan-3-one propionate	70804	Unclassified	Unclassified	Unclassified			37.4	0	M+FA-H	C23H36O3	0.071847633	2111.085666	2065.994635	2483.561003	1147.942443	1029.92094	773.920841	1005.396958	1536.845152	1556.317756

7.40_471.2424m/z	471.2423647	7.404266667	neg	Chenodeoxycholic acid sulfate	LMST05020026	Lipids and lipid-like molecules	Steroids and steroid derivatives	Bile acids, alcohols and derivatives	*		37.4	0	M-H	C24H40O7S	0.352576065	11808.9387	12605.4002	12390.20473	1712.344915	1528.70258	1741.142179	5463.901963	6246.447197	5990.42265
7.51_347.1611m/z	347.1611133	7.50615	neg	Ergoline-1,8-dimethanol, 10-methoxy-, (8b)-	1514	Unclassified	Unclassified	Unclassified			37.4	0	M+FA-H	C17H22N2O3	-0.437375923	3578.582049	3709.779141	3941.419077	4074.741	4560.871945	3825.789755	3146.430096	3846.856138	4038.898381
7.62_239.1290m/z	239.1289622	7.621333333	neg	(R)-2-Acetoxy-p-mentha-1,8-diene	HMDB0037389	Lipids and lipid-like molecules	Prenol lipids	Monoterpenoids	*		37.4	0	M+FA-H	C12H18O2	0.409410701	858.4769708	747.5686004	936.1036073	1175.35559	1052.796658	1128.291613	751.0377869	873.7882986	639.688249
7.65_337.1273m/z	337.1272526	7.65305	neg	Coriandrone A	92589	Organoheterocyclic compounds	Benzopyrans	2-benzopyrans			37.4	0	M+FA-H	C16H20O5	-6.928503554	11555.26861	11560.27515	12547.1322	6523.35022	5884.946372	6450.001883	10029.55426	10844.34569	10764.56404
7.73_306.0962m/z	306.0962409	7.729433333	neg	Aspartyl-Glutamine	85666	Organic acids and derivatives	Carboxylic acids and derivatives	Amino acids, peptides, and analogues	*		37.4	0	M+FA-H	C9H15N3O6	7.479296235	1227.372329	1205.782007	1260.145324	1099.006468	1273.155464	952.2571998	1317.127469	1390.655808	1424.328731
7.79_573.1364m/z	573.1363615	7.793283333	neg	Nodinitib-1	96479	Unclassified	Unclassified	Unclassified			37.4	0	2M-H	C14H13N3O2S	-3.583424319	9137.226253	8270.346334	10592.3047	13721.27287	12988.52563	12802.72819	10086.85725	9882.130539	10354.78444
7.94_302.1731n	347.1713378	7.9373	neg	Gamma-glutamyl-Arginine	86035	Unclassified	Unclassified	Unclassified			37.4	0	M-H, M+FA-H	C11H22N6O4	9.43784676	43561.83711	40563.81571	44286.44987	46570.41694	41704.23861	45711.85108	30124.54761	32761.04348	32415.39768
7.97_483.1462m/z	483.146243	7.9723	neg	Genipinic acid	92874	Organic acids and derivatives	Carboxylic acids and derivatives	Dicarboxylic acids and derivatives			37.4	0	2M-H	C11H14O6	-9.41200893	3897.773544	4183.691019	4151.107149	4078.642071	3508.30971	3632.429689	2504.351722	2576.992354	2628.591162
8.01_491.2503m/z	491.2502774	8.009683333	neg	Ammonium citrate	94825	Unclassified	Unclassified	Unclassified			37.4	0	2M-H	C6H20N3O7+3	-5.477127757	2863.893472	2824.692362	2844.638106	2316.049338	1677.171912	1862.619823	2178.39496	2192.380232	1914.361396
8.09_261.1708m/z	261.1707646	8.087016667	neg	(R)-3-Hydroxydodecanoic acid	HMDB0010728	Organic acids and derivatives	Hydroxy acids and derivatives	Medium-chain hydroxy acids and derivatives	*		37.4	0	M+FA-H	C12H24O3	0.078955042	143.2474973	174.1765541	154.3320652	85.07327277	98.9405268	196.8350311	102.5808074	131.407795	186.9672623
8.10_607.3492m/z	607.3491855	8.101016667	neg	Baliospermin	67398	Unclassified	Unclassified	Unclassified			37.4	0	M+FA-H	C32H50O8	0.736322892	3840.270244	4426.266978	4738.060743	5100.096301	4273.542143	4343.864305	2983.405052	5301.886044	3597.677161
8.13_713.2386m/z	713.2386211	8.129733333	neg	Gravacridonetriol	86194	Organoheterocyclic compounds	Quinolines and derivatives	Benzoquinolines	*		37.4	0	2M-H	C19H19NO6	4.792324542	1639.828703	1338.913795	1590.815826	3091.495948	3135.850046	3273.513197	1613.073669	2026.744253	1568.064761
8.81_475.1951m/z	475.1951034	8.814016667	neg	4'-METHOXYCHALCONE	44091	Phenylpropanoids and polyketides	Linear 1,3-diarylpropanoids	Chalcones and dihydrochalcones	*		37.4	0	2M-H	C16H14O2	7.602910715	2148.855916	2680.575203	2157.1331	1038.896875	976.435307	924.7417491	1437.9285	1689.708584	2157.904319
9.40_257.1760m/z	257.176015	9.396466667	neg	(±)-(E)-3-Methyl-4-decen-1-yl acetate	88715	Lipids and lipid-like molecules	Fatty Acyls	Fatty alcohol esters	*		37.4	0	M+FA-H	C13H24O2	0.858484114	1158.372522	1039.268074	1187.66637	961.1103325	988.6213799	1102.973144	886.2904422	795.6664814	965.5280759
9.57_377.1584m/z	377.1583679	9.565266667	neg	S-Prenyl-L-cysteine	HMDB0012286	Organic acids and derivatives	Carboxylic acids and derivatives	Amino acids, peptides, and analogues	*		37.4	0	2M-H	C8H15NO2S	2.498583079	3242.3033	3351.695604	3101.141156	3113.166163	3157.662489	3464.575932	3051.48742	2933.76161	3222.905656
0.74_223.9944m/z	223.9943695	0.7359	neg	Cyanophos	HMDB0250614	Organic acids and derivatives	Organic phosphoric acids and derivatives	Phosphate esters		C14430	37.3	0	M-H2O-H	C9H10NO3PS	1.274886764	915.9198567	439.8645514	818.9802896	1899.603868	3579.75966	2607.281573	0.001403199	2529.685909	2431.46377
0.74_272.0868m/z	272.0867634	0.7359	neg	HA-1077	45280	Unclassified	Unclassified	Unclassified			37.3	0	M-H2O-H	C14H17N3O2S	1.568801298	1494.732375	494.6716167	2325.18456	407.8147291	5823.348993	1619.20543	492.5381303	5466.036907	5000.320056
0.74_313.1136m/z	313.1135514	0.7359	neg	Histidinyl-Hydroxyproline	85792	Organic acids and derivatives	Carboxylic acids and derivatives	Amino acids, peptides, and analogues	*		37.3	0	M+FA-H	C11H16N4O4	-6.737494596	591.7283697	209.8136258	534.4122877	0.001403199	1356.825114	762.9989438	0.001403199	1912.633949	1370.270491
0.80_220.0285m/z	220.0285447	0.800366667	neg	S-(3-Oxo-3-carboxy-n-propyl)cysteine	HMDB0002135	Organic acids and derivatives	Carboxylic acids and derivatives	Amino acids, peptides, and analogues	*		37.3	0	M-H	C7H11NO5S	0.125142615	1299.867509	1412.062024	1560.442167	1873.568421	1754.908533	1899.494558	1318.801126	1971.332107	1589.098638
0.80_265.0736m/z	265.0735861	0.800366667	neg	Psilocybin	66689	Unclassified	Unclassified	Unclassified			37.3	0	M-H2O-H	C12H17N2O4P	-4.10587162	20945.95123	13383.35585	13682.47359	7434.482025	7920.65031	8148.292193	16460.70203	18481.35207	18226.08682
0.82_272.9923m/z	272.9922869	0.819566667	neg	L-Iduronate 2-sulfate	65910	Unclassified	Unclassified	Unclassified			37.3	0	M-H	C6H10O10S	0.349639461	2346.468369	2266.575356	2982.656022	5017.697673	3863.860253	4221.394726	2482.796849	2521.825662	2305.724054
0.86_323.0171m/z	323.0171152	0.86335	neg	5-O-(1-Carboxyvinyl)-3-phosphoshikimate	3454	Unclassified	Unclassified	Unclassified			37.3	0	M-H	C10H13O10P	-0.745627081	1667.728077	1518.1759	1994.203797	1666.486477	1087.303967	1664.635955	1546.928403	1243.440681	302.3600156
1.18_188.0025m/z	188.002468	1.180366667	neg	2-Pyridyl hydroxymethane sulfonic acid	68992	Unclassified	Unclassified	Unclassified			37.3	0	M-H	C6H7NO4S	0.876807779	8901.713578	8419.68956	8071.612363	2426.375409	2724.520039	2627.402629	3868.568278	4217.213179	4175.608697
1.49_201.0382m/z	201.0382361	1.492933333	neg	2-(2-Thienylmethylene)-1,6-dioxaspiro[4.4]non-3-ene	HMDB0034857	Organic oxygen compounds	Organooxygen compounds	Ethers			37.3	1.08	M-H2O-H	C12H12O2S	1.257108453	2617.36924	2669.742519	2994.530507	2106.242478	2041.487016	1894.634286	2498.138279	2448.166507	2493.736014
1.58_338.0730n	383.0712071	1.577833333	neg	6-(2-Amino-2-carboxyethyl)-7,8-dioxo-1,2,3,4,7,8-hexahydroquinoline-2,4-dicarl	70976	Unclassified	Unclassified	Unclassified			37.3	0	M-H, M+FA-H	C14H14N2O8	-6.009199896	5465.67795	4542.808211	4104.037416	5443.012566	5095.176458	5296.135707	3961.682208	5819.562852	4408.876479
1.59_339.0271m/z	339.027058	1.5934	neg	Diethylthiophosphate	HMDB0001460	Organic acids and derivatives	Organic thiophosphoric acids and derivatives	Thiophosphoric acid esters	*	C06607	37.3	0	2M-H	C4H11O3PS	3.031270337	817.4565195	815.015566	844.3695264	1126.966975	905.8232599	914.2529046	799.8922375	903.0332397	1018.371642
1.77_297.1193m/z	297.1192694	1.769966667	neg	Histidinyl-Proline	HMDB0304781	Organic acids and derivatives	Carboxylic acids and derivatives	Amino acids, peptides, and analogues	*		37.3	0	M+FA-H	C11H16N4O3	-4.655750474	1221.073269	1301.440514	1223.670705	741.5515005	656.8979552	912.5214457	1303.297399	1649.613338	1506.166262
1.82_320.0924m/z	320.0924199	1.820883333	neg	L-L-Homoglutathione	HMDB0029395	Organic acids and derivatives	Peptidomimetics	Hybrid peptides	*		37.3	0	M-H	C11H19N3O6S	0.747295085	1625.467412	1511.887653	1742.387553	4032.151374	4134.328438	3834.520824	1772.536246	2023.320696	1799.529679
12.40_271.2280m/z	271.2280175	12.40346667	neg	9-pentadecen-1-ol	LMFA05000050	Lipids and lipid-like molecules	Fatty Acyls	Fatty alcohols			37.3	0	M+FA-H	C15H30O	0.658778855	477.3535157	1101.262333	636.5719135	764.1289138	457.4167285	486.6740046	1715.295407	728.0330753	597.1388426
12.47_327.1271m/z	327.1271014	12.46835	neg	L-Rhamnulose	61546	Lipids and lipid-like molecules	Fatty Acyls	Fatty alcohols	**	C00861	37.3	0	2M-H	C6H12O5	-7.829455391	1740.000896	2441.426844	1934.15465	1090.99622	1718.558792	1552.578326	2054.774642	2165.625951	1690.708308
14.96_150.0018m/z	150.0017556	14.96435	neg	4-Thiocyanatophenol	95063	Organosulfur compounds	Thioethers	Aryl thioethers			37.3	0	M-H	C7H5NOS	-1.011443495	3374.531766	3180.733205	3914.32325	2738.000864	3584.577827	3437.179731	3646.533507	3961.048692	4009.826473
2.00_283.0913m/z	283.0912518	2.004766667	neg	3''-Deamino-3''-oxonicotianamine	70942	Unclassified	Unclassified	Unclassified			37.3	0	M-H2O-H	C12H18N2O7	-7.63953235	47676.88545	44320.12463	47254.35424	30501.46582	27793.0931	29560.71338	32051.76976	38178.2829	32799.54786
2.07_267.0963m/z	267.0963376	2.070366667	neg	2-p-Tolyl-5,6,7,8-tetrahydrobenzo[d]imidazo[2,1-b]thiazole	69104	Unclassified	Unclassified	Unclassified			37.3	0	M-H	C16H16N2S	0.725543376	6989.157583	7539.302325	7399.329142	13926.84306	15612.15554	15077.50902	10556.21106	9669.609934	10162.18988
2.07_335.0840m/z	335.0839543	2.070366667	neg	Flumioxazin	68790	Organoheterocyclic compounds	Benzoxazines	Benzoxazinones		C11035	37.3	0	M-H2O-H	C19H15FN2O4	0.593802813	3783.816413	4170.940466	3797.69475	7402.457244	8944.842024	8269.814136	5719.949636	5408.330591	5845.049296
2.63_368.9875m/z	368.9874671	2.634083333	neg	2'-Deoxyuridine 5'-diphosphate (dUDP)	3515	Unclassified	Unclassified	Unclassified			37.3	0	M-H2O-H	C9H14N2O11P2	-5.088251476	2314.871028	2651.094688	2337.188774	95.16854643	0.001403199	54.50907845	3226.918747	3425.748948	3380.192437
2.67_227.0539m/z	227.0538643	2.6698	neg	(S)-5-Hydroxymethyl-2[5H]-furanone	265050	Unclassified	Unclassified	Unclassified			37.3	0	2M-H	C5H6O3	-9.854000425	1336.183444	1058.60863	1243.644038	1846.889942	1579.294185	1364.493078	1399.846327	1426.277335	1460.45961
2.72_385.0381m/z	385.0380927	2.72215	neg	ERYSOLIN	43501	Unclassified	Unclassified	Unclassified			37.3	0	2M-H	C6H11NO2S2	-2.258974644	2782.101413	3177.627576	2939.056173	3121.604614	3231.47219	2767.286502	2810.82089	3129.534626	3207.484346
2.92_208.0651m/z	208.0651178	2.9156	neg	S-(2-carboxypropyl)-Cysteamine	LMFA01020395	Organic acids and derivatives	Carboxylic acids and derivatives	Amino acids, peptides, and analogues	*		37.3	0	M+FA-H	C6H13NO2S	1.320136822	358.608707	304.8892326	421.9689386	541.4664909	502.8858477	639.1387118	368.4855219	471.8010203	411.0073633
3.24_373.0308m/z	373.0307996	3.23635	neg	Fluorodifen	68807	Unclassified	Unclassified	Unclassified			37.3	0	M+FA-H	C13H7F3N2O5	5.763821632	1507.359369	1423.567317	1369.301507	0.001403199	0.001403199	54.31494177	676.8027443	715.4812927	798.1642427
3.31_261.0074m/z	261.0074002	3.31425	neg	3-hydroxy-3-(3-hydroxyphenyl)propanoic acid-O-sulphate	HMDB0059967	Organic acids and derivatives	Hydroxy acids and derivatives	Beta hydroxy acids and derivatives	*		37.3	0	M-H	C9H10O7S	-0.179314521	3957.92545	4001.296244	3743.204012	5544.264847	5357.383731	5391.494908	3675.955295	3785.486962	3493.947226
3.46_347.0111m/z	347.0110735	3.455516667	neg	Phenolic phosphate	65822	Unclassified	Unclassified	Unclassified			37.3	0	2M-H	C6H7O4P	5.629701795	2172.399976	2328.281974	2395.568707	1530.771246	1304.132581	1510.550825	2496.81972	2546.189813	2199.890409
3.62_354.1200m/z	354.1200398	3.624166667	neg	Tranylcypromine glucuronide	2897	Unclassified	Unclassified	Unclassified			37.3	0	M+FA-H	C15H19NO6	1.939691504	378.3148946	442.0792536	312.0972195	1572.419477	1401.895341	1655.532603	434.5741491	332.6346823	429.2059397
3.80_311.0188m/z	311.0187984	3.801716667	neg	3',5'-Cyclic Inosine monophosphate (cIMP)	3460	Unclassified	Unclassified	Unclassified			37.3	0	M-H2O-H	C10H11N4O7P	0.315756296	371.4265853	198.197109	266.7287536	520.2893606	338.8906801	434.3132698	300.4505935	385.0094528	304.2946566
3.89_361.0908m/z	361.090758	3.8906	neg	Homoferreirin	48006	Phenylpropanoids and polyketides	Isoflavonoids	O-methylated isoflavonoids	*	C10457	37.3	0	M+FA-H	C17H16O6	-6.748276512	405.6015985	504.1666543	519.4046785	332.6540643	302.0860193	270.1826699	609.4401109	704.9845314	975.2527039
3.91_304.9789m/z	304.9789263	3.912566667	neg	Thiocysteine	58162	Organic acids and derivatives	Carboxylic acids and derivatives	Amino acids, peptides, and analogues	**	C01962	37.3	0	2M-H	C3H7NO2S2	8.372489003	1610.767035	1510.677288	1635.960329	2453.620285	2121.286924	2224.090018	1881.486133	1954.159603	2241.293648
3.94_319.0049m/z	319.004941	3.9351	neg	D-Glucuronic acid 1-phosphate	58195	Organic oxygen compounds	Organooxygen compounds	Carbohydrates and carbohydrate conjugates	**	C05385	37.3	0	M+FA-H	C6H11O10P	-8.193312639	5117.988308	5428.61477	5534.132069	4795.519194	4442.877607	4992.335322	4017.050092	3910.996087	3939.739526
3.98_229.1559m/z	229.1559039	3.97985	neg	Isoleucyl-Valine	HMDB0028920	Organic acids and derivatives	Carboxylic acids and derivatives	Amino acids, peptides, and analogues	*		37.3	0	M-H	C11H22N2O3	0.598671666	51.24005224	68.18044357	81.88847202	98.89379733	37.09492472	92.91532718	46.50449215	66.28120041	72.40675296
4.05_187.0145m/z	187.0144627	4.046666667	neg	4-Nitro-3-(trifluoromethyl)aniline	676	Unclassified	Unclassified	Unclassified			37.3	0	M-H2O-H	C7H5F3N2O2	9.667299573	185.1057832	230.3044109	275.7958533	267.8690388	454.0720055	375.427695	353.0031189	329.5096703	395.029106
4.14_190.0544m/z	190.0544303	4.136366667	neg	N-Acetyl-DL-methionine	62466	Unclassified	Unclassified	Unclassified			37.3	0	M-H	C7H13NO3S	0.484140857	95.41826738	77.63012587	64.70915089	78.95010161	102.5062691	125.0747005	141.8218153	114.6200765	88.81207843
4.18_279.0486m/z	279.0486268	4.181333333	neg	Benzo[b]naphtho[2,1-d]thiophene	73542	Unclassified	Unclassified	Unclassified			37.3	0	M+FA-H	C16H10S	0.438405743	5888.695591	5222.620566	5212.196982	2079.182764	1859.953196	2314.572962	4242.297825	3603.554091	3450.197863
4.21_318.1019m/z	318.1019047	4.212116667	neg	Salbutamol 4-O-sulfate	HMDB0060604	Organic acids and derivatives	Organic sulfuric acids and derivatives	Arylsulfates	*		37.3	0	M-H	C13H21NO6S	0.698037964	1259.557728	1661.93665	1561.1848	1931.64558	2016.454991	2070.599962	1651.119308	1832.760695	1770.938851
4.23_258.0782m/z	258.0781938	4.234483333	neg	Fenamic acid	69644	Unclassified	Unclassified	Unclassified			37.3	0	M+FA-H	C13H11NO2	4.751152757	150.5471972	640.906735	904.5170134	1497.61309	1356.954187	46.732688	132.9455719	542.5483971	454.2089327
4.28_519.1419m/z	519.1419431	4.278183333	neg	JZL 184	45471	Unclassified	Unclassified	Unclassified			37.3	0	M-H	C27H24N2O9	1.997818966	2759.803746	2839.531296	3076.17181	266.7750351	434.934211	252.0711418	2515.289485	3063.665134	2776.441861
4.32_421.1651m/z	421.1650836	4.3224	neg	6-Maleimidocaproic acid	24061	Unclassified	Unclassified	Unclassified			37.3	0	2M-H	C10H13NO4	8.158514452	2035.570335	2025.704666	2241.111931	3066.510975	2845.835226	2933.171815	1774.25318	2159.094229	2178.929938
4.34_275.1069m/z	275.1069124	4.344733333	neg	(2-Butylbenzofuran-3-yl)(4-hydroxyphenyl)ketone	70543	Unclassified	Unclassified	Unclassified			37.3	0	M-H2O-H	C19H18O3	-2.858957042	674.1914448	1017.936465	943.6541669	1061.262509	834.7803596	909.5128194	594.7803606	443.9621353	579.9232081
4.41_353.0990m/z	353.098959	4.41115	neg	Fluprostenol Lactone Diol	63038	Unclassified	Unclassified	Unclassified			37.3	0	M-H2O-H	C18H19F3O5	-4.456324054	2137.350575	1911.319563	1606.024296	1891.71115	1249.102656	1703.984435	2427.22996	2384.327841	2093.843499
4.43_399.0039m/z	399.0038639	4.432683333	neg	3,5-DINITROCATECHOL (OR-486)	44188	Unclassified	Unclassified	Unclassified			37.3	0	2M-H	C6H4N2O6	-6.828259672	4173.503857	4158.534988	4492.575885	2355.017797	2678.367387	2956.540783	4180.62969	3152.276889	3577.517426
4.45_368.9881m/z	368.9880507	4.454616667	neg	dUDP	HMDB0001000	Nucleosides, nucleotides, and analogues	Pyrimidine nucleotides	Pyrimidine deoxyribonucleotides	**	C01346	37.3	0	M-H2O-H	C9H14N2O11P2	-3.584134115	2143.608737	2362.627523	2142.26806	224.9445113	237.4302996	235.3116763	2141.928363	2377.832299	2422.639492
4.45_535.1424m/z	535.1424434	4.454616667	neg	Trigraecum	LMPK12110062	Phenylpropanoids and polyketides	Flavonoids	O-methylated flavonoids	*		37.3	0	2M-H	C16H12O4	4.853366065	1223.536164	1915.771529	1092.499301	360.7430129	210.6488612	182.4238422	366.2033881	645.406303	558.1888133
4.50_353.0340m/z	353.033997	4.49925	neg	cis-Resveratrol 3-sulfate	HMDB0041712	Phenylpropanoids and polyketides	Stilbenes	Unclassified	*		37.3	0	M+FA-H	C14H12O6S	1.08758887	4142.581804	4709.454256	4684.745322	2420.381842	2342.557479	2116.775777	2759.327486	3972.363555	3906.145319
4.50_366.1538m/z	366.1538449	4.49925	neg	6'-Dehydro-6'-oxoparomamine	71792	Unclassified	Unclassified	Unclassified			37.3	0	M+FA-H	C12H23N3O7	6.358197087	455.7594866	439.6713052	630.9461195	235.7153713	210.5671449	201.9932207	410.1760065	508.7981674	433.7294449
4.57_249.0377m/z	249.0377071	4.5668	neg	L-Homocystine	HMDB0000676	Organic acids and derivatives	Carboxylic acids and derivatives	Amino acids, peptides, and analogues	**	C01817	37.3	0.689	M-H2O-H	C8H16N2O4S2	1.489168343	2324.903539	2376.169001	2455.775438	2813.43688	2740.841698	2696.263792	2049.28707	2237.359724	2308.386914
4.57_301.0391m/z	301.0390937	4.5668	neg	5-(3',5'-Dihydroxyphenyl)-gamma-valerolactone-O-sulphate-O-methyl	HMDB0060031	Organic acids and derivatives	Organic sulfuric acids and derivatives	Arylsulfates	*		37.3	0	M-H	C12H14O7S	1.14682379	889.5934142	688.8861233	1707.727181	1002.174391	831.0824435	927.2436618	1379.217522	1617.457359	1694.812388
4.57_349.1269m/z	349.126906	4.5668	neg	2'-Deoxymugineic acid	70941	Organic acids and derivatives	Carboxylic acids and derivatives	Amino acids, peptides, and analogues			37.3	0	M+FA-H	C12H20N2O7	5.432528333	604.1952523	537.4657928	600.4220112	824.193899	685.1480859	583.2282701	479.608524	527.4359185	325.9848134
4.70_373.0493m/z	373.0492893	4.703166667	neg	Sodium 6-hydroxy-5-(phenylazo)-2-naphthalenesulfoniate	HMDB0032902	Benzenoids	Naphthalenes	Unclassified			37.3	0	M+FA-H	C16H12N2O4S	-2.107771156	344.6935436	689.9217173	569.9104119	1339.592346	1283.062234	946.5995269	735.1267266	756.6033357	500.7473482
4.75_293.0394m/z	293.0393606	4.747466667	neg	(E)-2-(2-Furyl)-3-(5-nitro-2-furyl)acrylamide	66225	Unclassified	Unclassified	Unclassified			37.3	0	M+FA-H	C11H8N2O5	-8.722867743	5195.868815	6087.492851	5520.415893	2809.024491	2591.23273	2815.514667	4865.525876	5528.225166	5074.411598
4.75_344.0366m/z	344.0366067	4.747466667	neg	Guanosine monophosphate	HMDB0001397	Nucleosides, nucleotides, and analogues	Purine nucleotides	Purine ribonucleotides	***	C00144	37.3	0	M-H2O-H	C10H14N5O8P	-9.781593881	21296.23103	21674.46033	21530.4878	10536.33381	10576.20218	10100.4186	19132.84746	21476.43986	20660.87777
4.84_385.0578m/z	385.0577776	4.836566667	neg	Pyrazosulfuron	72650	Unclassified	Unclassified	Unclassified			37.3	0	M-H	C12H14N6O7S	1.51832785	3290.754652	3363.054122	3496.123434	3082.294612	3082.465625	2926.247472	2591.386476	3245.315445	3003.635834
4.84_537.0560m/z	537.056048	4.836566667	neg	(±)-Mephosfolan	HMDB0031801	Organic acids and derivatives	Organic phosphoric acids and derivatives	Phosphate esters	*		37.3	0	2M-H	C8H16NO3PS2	2.751462092	674.4450912	397.2033112	385.5858296	1708.093107	1137.1678	1403.152885	572.0535288	1024.952759	656.7946827
4.90_319.0048m/z	319.0047589	4.902316667	neg	6-Phospho-2-dehydro-D-gluconate	63129	Unclassified	Unclassified	Unclassified			37.3	0	M+FA-H	C6H11O10P	-8.858022371	998.0602695	1094.698601	967.4611759	1037.197066	1096.676925	1030.457571	945.4380389	1012.655229	956.2033227
4.95_725.1937m/z	725.1936687	4.94705	neg	Pelargonidin 3-sambubioside-5-glucoside	46792	Unclassified	Unclassified	Unclassified			37.3	0	M-H	C32H38O19	0.297586449	1861.732821	2016.598957	1828.065001	0.001403199	0.001403199	0.001403199	151.9145288	0.001403199	170.4451399
5.06_310.1079m/z	310.1078997	5.05975	neg	Launobine	HMDB0030217	Organoheterocyclic compounds	Indoles and derivatives	Carbazoles	*		37.3	0	M-H	C18H17NO4	-1.87025092	488.061798	325.7511402	524.3014879	643.2809192	441.5672372	823.1594424	413.567046	405.1339645	487.2307305
5.08_629.1193m/z	629.1192871	5.080566667	neg	Cordeauxione	89840	Organic acids and derivatives	Vinylogous acids	Unclassified			37.3	0	M+FA-H	C28H24O14	7.66770427	8746.027867	7792.64644	8351.535052	13160.25798	10806.21882	12063.59774	11370.81923	10430.96273	10359.71147
5.13_227.0561n	272.0542471	5.132616667	neg	Lansine	86807	Organoheterocyclic compounds	Indoles and derivatives	Carbazoles	*		37.3	0	M-H, M+FA-H	C13H9NO3	-9.510639349	13586.82772	14403.05969	11379.06898	21453.88926	20196.67902	20148.2492	19225.59878	20070.27339	25679.61777
5.13_261.1345m/z	261.1344639	5.132616667	neg	(R)-Lisofylline	45571	Unclassified	Unclassified	Unclassified			37.3	0	M-H2O-H	C13H20N4O3	-4.410102191	2950.580294	3398.748947	2675.673899	1697.083566	1709.427825	1816.231245	1821.0602	1715.433622	1786.456027
5.18_301.1658m/z	301.165779	5.1777	neg	N3-Metyladenine	66285	Unclassified	Unclassified	Unclassified			37.3	0	2M-H	C6H9N5	4.847720492	637.2805702	793.6389788	538.1856355	538.7834573	549.4175743	497.3497606	550.2649867	332.2777944	582.5944298
5.30_259.1550m/z	259.1549656	5.302883333	neg	9-keto lauric acid	LMFA01060040	Lipids and lipid-like molecules	Fatty Acyls	Fatty Acids and Conjugates			37.3	0	M+FA-H	C12H22O3	-0.615505918	446.1267397	619.6058397	632.2643548	617.2575658	668.4957631	797.1825238	508.848173	549.2540298	523.691201
5.36_319.1301m/z	319.1300579	5.361783333	neg	Nicotine glucuronide	6127	Organic oxygen compounds	Organooxygen compounds	Carbohydrates and carbohydrate conjugates	*		37.3	0	M-H2O-H	C16H22N2O6	0.332813791	910.6093295	1079.649108	1261.639681	1288.734271	1514.915141	1346.892525	1013.228358	1426.031015	1519.222716
5.38_455.1542m/z	455.1542348	5.384216667	neg	XANTHYLETIN	43779	Unclassified	Unclassified	Unclassified			37.3	0	2M-H	C14H12O3	9.257316685	15607.85759	14715.07697	13937.6772	16930.64057	19492.71217	17387.06438	13361.10975	12142.83386	12893.31535
5.43_571.1436m/z	571.1435736	5.428733333	neg	6-Desmethoxy hormothamnione triacetate	65419	Unclassified	Unclassified	Unclassified			37.3	0	M+FA-H	C27H26O11	-4.069140456	2251.093972	2685.029558	2445.007532	0.001403199	0.001403199	0.001403199	0.001403199	0.001403199	122.471048
5.49_548.3078m/z	548.3077818	5.489016667	neg	Pederin	71091	Unclassified	Unclassified	Unclassified			37.3	0	M+FA-H	C25H45NO9	0.29195735	1748.512335	1500.499615	1450.320395	981.4647279	765.0582725	1632.411685	2215.91083	2365.293556	2345.522888
5.53_439.0709m/z	439.0708704	5.525416667	neg	Dracunculin	87197	Phenylpropanoids and polyketides	Coumarins and derivatives	Unclassified	*		37.3	0	2M-H	C11H8O5	8.635202964	1623.181299	1686.938319	1694.878795	1803.442147	2375.645717	1790.934109	1473.590924	1526.410301	1497.789423
5.53_769.1931m/z	769.1931395	5.525416667	neg	(S)-2,3-Dihydro-3,5-dihydroxy-2-oxo-3-indoleacetic acid 5-glucoside	90128	Organic oxygen compounds	Organooxygen compounds	Carbohydrates and carbohydrate conjugates			37.3	0	2M-H	C16H19NO10	-1.786092152	1612.691795	2060.190703	2062.993473	0.001403199	0.001403199	0.001403199	674.5582485	1316.247169	1316.221875
5.61_448.1614m/z	448.1614268	5.610733333	neg	O-Ethyl-4-[(2',3',4'-tri-O-acetyl-alpha-L-rhamnosyloxy)benzyl]carbamate	HMDB0031949	Organic oxygen compounds	Organooxygen compounds	Carbohydrates and carbohydrate conjugates	*		37.3	0	M-H2O-H	C22H29NO10	0.260708179	1808.552184	2130.769321	2310.419688	3141.914717	3498.907025	3598.822039	3759.142267	3341.212814	3501.311691
5.65_238.0818n	219.0639248	5.650833333	neg	1-(2,4,5-Trimethoxyphenyl)-1,2-propanedione	87928	Benzenoids	Benzene and substituted derivatives	Phenylpropanes	*		37.3	0	M-H2O-H, 2M-H	C12H14O5	-9.902477769	3870.211534	4463.353237	5649.092017	5436.75201	4192.646693	4448.517666	3921.698146	3029.70013	4352.505325
5.65_271.1917m/z	271.191736	5.650833333	neg	Phenelzine	HMDB0014918	Benzenoids	Benzene and substituted derivatives	Unclassified		C07430	37.3	0	2M-H	C8H12N2	-3.983775761	331.4306525	324.1933412	371.7794369	0.001403199	0.001403199	0.001403199	0.001403199	65.01300526	0.001403199
5.65_310.0312m/z	310.0312154	5.650833333	neg	Nitisinone	85368	Organic oxygen compounds	Organooxygen compounds	Carbonyl compounds			37.3	0	M-H2O-H	C14H10F3NO5	-6.231659281	3509.403362	3783.740612	4713.21219	6405.825468	5568.902254	5427.078892	4258.251254	3403.422165	4399.95712
5.65_425.1446m/z	425.1446128	5.650833333	neg	(S)-Bitalin A 12-glucoside	93049	Organic oxygen compounds	Organooxygen compounds	Carbohydrates and carbohydrate conjugates			37.3	0	M+FA-H	C19H24O8	-1.861880772	4539.878145	4571.781424	5205.801767	6764.643312	6708.737633	6725.595362	3700.841214	4026.310573	4719.812199
5.70_428.2413n	473.2331272	5.701283333	neg	Nummularine F	68090	Unclassified	Unclassified	Unclassified			37.3	0	M-H2O-H, M+FA-H	C23H32N4O4	-2.525887652	2548.154978	2339.699019	2088.963344	2623.714786	3100.631954	3218.338412	1873.73128	1987.852691	2370.979586
5.71_811.2001m/z	811.200074	5.709933333	neg	Hoslundin	48588	Lipids and lipid-like molecules	Polyketides	Flavonoids			37.3	0	2M-H	C23H18O7	-3.884967413	15711.28273	16054.95786	12210.49671	15164.69406	19065.07585	19086.65761	12060.30629	17342.50656	20665.30509
5.74_364.9975m/z	364.9974915	5.744566667	neg	Luteolin 4'-sulfate	49166	Phenylpropanoids and polyketides	Flavonoids	Flavones	*		37.3	0	M-H	C15H10O9S	0.587392802	0.001403199	0.001403199	75.14479432	1363.982465	1593.136995	1352.221219	0.001403199	0.001403199	75.86117844
5.83_340.1111n	385.1119181	5.825	neg	Diphenadione	72476	Unclassified	Unclassified	Unclassified			37.3	0	M-H2O-H, M+FA-H	C23H16O3	3.37990329	18826.92762	20916.77523	19503.71765	55675.03866	44593.23748	44820.85999	34790.35012	27649.96536	29457.06853
5.87_795.1270m/z	795.1270139	5.866533333	neg	Sulfasalazine	2551	Organoheterocyclic compounds	Azobenzenes	Unclassified		C07316	37.3	0	2M-H	C18H14N4O5S	-3.379807415	10026.27842	10520.14867	10735.208	9057.436889	7579.36771	7649.919095	14008.29007	12073.24531	14177.71551
5.88_386.1256m/z	386.1256121	5.88025	neg	Cassythine	67647	Unclassified	Unclassified	Unclassified			37.3	0	M+FA-H	C19H19NO5	3.184992925	5368.807403	5550.480674	6129.616508	5491.618334	5383.159373	4815.876106	6395.332926	6876.490069	7963.91516
5.92_479.1898m/z	479.1897557	5.9168	neg	Isopropyl 3-(3,4-dihydroxyphenyl)-2-hydroxypropanoate	96114	Benzenoids	Phenols	Benzenediols			37.3	0	2M-H	C12H16O5	-5.237672911	3073.461882	3378.643638	3213.931252	929.2912332	988.5619867	1147.362691	2139.951673	2732.89165	3457.724359
5.95_359.2079m/z	359.2079064	5.95155	neg	1-(p-Fluorophenyl) piperazine	96616	Unclassified	Unclassified	Unclassified			37.3	0	2M-H	C10H13FN2	7.299965031	620.1712727	693.1307522	956.4735657	1031.681717	1636.349864	1557.441889	514.6897519	392.1281749	869.4121844
6.07_422.1803m/z	422.1803359	6.07	neg	Oxprenolol glucuronide	1682	Unclassified	Unclassified	Unclassified			37.3	0	M-H2O-H	C21H31NO9	-3.863435156	5373.393414	5993.98233	6354.498675	12476.91868	12230.94641	10503.39393	7104.363686	7458.863189	7427.4286
6.16_230.1400m/z	230.1399576	6.161866667	neg	N(alpha)-t-Butoxycarbonyl-L-leucine	66157	Unclassified	Unclassified	Unclassified			37.3	0	M-H	C11H21NO4	0.7608553	413.5395201	622.875309	489.8789412	428.5272454	360.0888402	490.7433081	463.307762	752.1840027	634.6363382
6.16_299.1490m/z	299.1489807	6.161866667	neg	3-methyl Benzamideoxime	45442	Unclassified	Unclassified	Unclassified			37.3	0	2M-H	C8H10N2O	-7.891712776	1397.226449	1662.269761	1600.184331	2277.032617	2486.250408	3021.685572	1140.767311	1327.018078	1241.714681
6.25_685.2072m/z	685.2072433	6.248083333	neg	Caffeoyl tyrosine	86166	Organic acids and derivatives	Carboxylic acids and derivatives	Amino acids, peptides, and analogues	*		37.3	0	2M-H	C18H17NO6	4.874868672	22154.63762	16892.41034	20164.57427	23394.75117	19004.23811	23810.72149	19226.0074	21996.58615	19903.59557
6.26_261.1708m/z	261.1708321	6.261966667	neg	Dodecanoic acid, 3-hydroxy-, (R)-; Dodecanoic acid, 3-hydroxy-, D-(-)- (8CI); (3I	74616	Unclassified	Unclassified	Unclassified			37.3	0	M+FA-H	C12H24O3	0.391391319	576.4588926	515.3652404	505.6986466	543.2587425	470.4553477	686.0826941	470.8270509	599.4206316	450.1006768
6.37_393.1170m/z	393.1170114	6.373833333	neg	Machaerol C	48287	Lipids and lipid-like molecules	Polyketides	Flavonoids			37.3	0	M+FA-H	C18H20O7	-6.016307438	5014.588891	5915.963782	6652.810199	2410.531658	2461.473361	2445.237136	5372.084314	5870.312829	5749.263076
6.40_295.1553m/z	295.15531	6.39835	neg	Procurcumadiol	HMDB0034721	Lipids and lipid-like molecules	Prenol lipids	Sesquiterpenoids	*		37.3	0	M+FA-H	C15H22O3	0.849918949	1934.162471	2109.856593	2358.979344	3298.326175	3256.274075	3139.488624	2109.958167	1980.081704	1868.51096
6.41_639.1678m/z	639.1678394	6.408833333	neg	3-p-Coumaroyl-1,5-quinolactone	86163	Phenylpropanoids and polyketides	Cinnamic acids and derivatives	Hydroxycinnamic acids and derivatives	*		37.3	0	2M-H	C16H16O7	-6.388636006	2604.94643	3403.118779	3921.205909	709.9237575	631.427943	933.7805386	2455.722065	3063.889834	2503.33091
6.44_547.2166m/z	547.2165822	6.442716667	neg	Cyclocalamin	HMDB0035863	Benzenoids	Benzene and substituted derivatives	Benzoic acids and derivatives	*		37.3	0	M+FA-H	C27H34O9	-3.789805977	7191.748895	6254.925887	6774.001906	7446.972295	7078.15485	6832.120283	8187.540998	7983.392789	8212.104547
6.64_531.2216m/z	531.2216393	6.644916667	neg	Nevirapine	43275	Organic nitrogen compounds	Organonitrogen compounds	Amines		C07263	37.3	0	2M-H	C15H14N4O	-8.654910744	2475.808914	3193.136634	2729.335233	4613.043233	4193.271169	4312.246032	3075.795445	3871.714778	4009.298456
6.69_223.1341m/z	223.1341407	6.69475	neg	(6S,9R)-Vomifoliol	65649	Unclassified	Unclassified	Unclassified			37.3	0	M-H	C13H20O3	0.770300657	343.357914	324.1891805	338.7661455	367.6117471	376.8504217	382.4076623	264.8446854	371.423365	409.3495381
6.71_437.1798m/z	437.179773	6.70695	neg	Picrasin G	71529	Unclassified	Unclassified	Unclassified			37.3	0	M+FA-H	C21H28O7	-4.929091989	11131.15255	12630.62374	12345.35567	14846.23564	14099.96013	13035.53184	10757.32073	13310.14922	13236.68642
6.75_247.0953m/z	247.0952845	6.747166667	neg	1-(2-Furanyl)-2-propanone	88822	Organoheterocyclic compounds	Heteroaromatic compounds	Unclassified			37.3	0	2M-H	C7H8O2	-9.262359877	588.4003062	597.8463609	740.5875738	1005.811353	981.5742706	1237.833901	650.3486823	686.641701	682.2202967
6.94_407.2076m/z	407.2075821	6.939266667	neg	4,5β-Dihydrocortisone	41870	Lipids and lipid-like molecules	Steroids and steroid derivatives	Hydroxysteroids	**	C05469	37.3	0	M+FA-H	C21H30O5	0.152338927	1333.845496	1140.447973	1603.349375	1229.644696	763.6501906	971.2692956	702.7088598	990.2896385	1163.033416

6.95_261.1708m/z	261.1708294	6.951516667	neg	Dodecanoic acid, 3-hydroxy-, (S)-; L-3-Hydroxydodecanoic acid	74617	Unclassified	Unclassified	Unclassified			37.3	0	M+FA-H	C12H24O3	0.378941427	409.040339	380.2661924	483.7127376	437.9159613	350.6485829	443.1290169	256.2338269	344.3953869	355.6434535
7.18_441.1206m/z	441.120579	7.178516667	neg	6,8-Di-O-methylaverufin	HMDB0035465	Benzenoids	Anthracenes	Anthraquinones			37.3	0	M+FA-H	C22H20O7	3.718868507	7652.697728	9312.351253	8600.423597	2748.576194	2563.929372	2595.361847	5778.61179	7696.197951	5976.063664
7.37_475.2189m/z	475.2188849	7.372733333	neg	Ustiloxin D	95487	Organic acids and derivatives	Carboxylic acids and derivatives	Amino acids, peptides, and analogues			37.3	0	M-H2O-H	C23H34N4O8	-1.897980972	2533.138538	2673.860048	1778.349312	2096.73615	2361.312972	2370.163447	633.3109241	851.8022588	652.6827477
7.44_261.1709m/z	261.1708707	7.440866667	neg	α-hydroxy lauric acid	35417	Unclassified	Unclassified	Unclassified			37.3	0	M+FA-H	C12H24O3	0.569972382	255.1948284	111.7342966	246.43864	29.98977758	115.5051495	73.06078851	155.4595265	160.221014	91.16754877
7.44_719.2879m/z	719.2879381	7.440866667	neg	Trichilin A	67203	Unclassified	Unclassified	Unclassified			37.3	0	M+FA-H	C35H46O13	-6.089764204	56651.80366	53285.49375	67053.66611	9497.729864	9790.273132	9045.467728	27334.42485	30484.10107	24704.24196
7.44_777.2463m/z	777.2462718	7.440866667	neg	Epoxy Fluor 7	64657	Unclassified	Unclassified	Unclassified			37.3	0	2M-H	C23H19NO5	1.160005864	3775.917609	3334.968029	3897.078759	0.001403199	0.001403199	167.3122104	1123.55422	2088.88575	1475.478694
7.45_446.2916m/z	446.2916426	7.453016667	neg	Sodium glycocholate	88551	Unclassified	Unclassified	Unclassified			37.3	0	M-H2O-H	C26H43NO6	0.957649541	2733.073238	2835.16328	3584.284751	2295.735536	3005.702401	2862.626739	2512.566731	2508.50185	2280.774387
7.47_415.2155m/z	415.2154611	7.46525	neg	Benzyl (2R,3S)-2-methyl-3-hydroxybutanoate	66209	Unclassified	Unclassified	Unclassified			37.3	0	2M-H	C12H16O3	6.844363566	2281.249644	2230.894743	2726.107167	2980.53108	3506.224478	2546.624804	2191.003133	2150.070925	2239.066615
7.58_261.1708m/z	261.170833	7.5758	neg	alpha-hydroxy lauric acid	LMFA01050036	Lipids and lipid-like molecules	Fatty Acyls	Fatty Acids and Conjugates			37.3	0	M+FA-H	C12H24O3	0.395632326	1513.270703	1883.041243	1855.440508	1290.953928	1175.182424	1343.129357	1191.469587	1365.203164	1087.950538
7.58_610.3602m/z	610.3601934	7.5758	neg	JSTX-3	69746	Unclassified	Unclassified	Unclassified			37.3	0	M+FA-H	C27H47N7O6	5.674840267	3722.885904	4937.957158	4979.335668	0.001403199	101.1858968	416.7131687	3386.132817	4920.710934	2879.027526
7.65_179.1443m/z	179.1442982	7.65305	neg	Allyl nonanoate	HMDB0029763	Lipids and lipid-like molecules	Fatty Acyls	Fatty acid esters	*		37.3	0	M-H2O-H	C12H22O2	0.804174621	381.9195621	354.2667271	333.3928968	203.4675885	174.9461731	228.1794594	280.2999065	293.912248	281.7653204
8.05_366.1723m/z	366.1722942	8.053916667	neg	Carboxyterbinafine	2750	Unclassified	Unclassified	Unclassified			37.3	0	M+FA-H	C21H23NO2	3.774671229	2239.640168	2805.745223	2562.125146	4270.766814	4074.402802	3802.442438	1614.296164	2207.441616	2037.364408
8.07_441.2414m/z	441.2413708	8.066183333	neg	1-(8Z,11Z,14Z-eicosatrienoyl)-glycero-3-phosphate	HMDB0062313	Lipids and lipid-like molecules	Glycerophospholipids	Glycerophosphates	*		37.3	0	M-H2O-H	C23H41O7P	0.481610792	11298.7648	13947.50441	12037.72673	1567.598114	1759.4898	1462.013838	4757.501967	6747.036943	6452.904662
8.09_525.1746m/z	525.1746246	8.087016667	neg	Physalin E	89851	Lipids and lipid-like molecules	Steroids and steroid derivatives	Physalins and derivatives	*		37.3	0	M-H2O-H	C28H32O11	-3.668088037	10475.61409	12314.49861	11380.40878	4506.521015	3597.911273	3847.017031	7057.840009	8077.260206	8193.424348
8.24_497.2398m/z	497.2397769	8.244766667	neg	b-D-Glucopyranosiduronic acid, (3a,5b)-17-oxoestran-3-yl	1440	Unclassified	Unclassified	Unclassified			37.3	0	M+FA-H	C24H36O8	1.22915727	4488.725525	5023.406451	5399.449161	828.9048952	410.8730643	810.0404916	1849.352698	2549.913091	2293.295872
8.61_569.2113m/z	569.2112518	8.606866667	neg	Arabinoside	87702	Unclassified	Unclassified	Unclassified			37.3	0	2M-H	C10H15N5O5	6.823766614	1490.384454	1084.157434	1571.750366	1604.037833	1450.590036	1598.321115	1379.823333	2115.80428	1336.7665
8.61_647.2279m/z	647.2278892	8.606866667	neg	PtdIns-(3)-P1 (1,2-dioctanoyl) (sodium salt)	45022	Unclassified	Unclassified	Unclassified			37.3	0	M-H2O-H	C25H48O16P2	5.961188124	9180.792454	10639.29344	10229.64285	12930.03249	12514.22943	12033.1346	11354.0844	12024.86495	11275.11978
8.93_397.2235m/z	397.2235238	8.925416667	neg	Prostaglandin D2	HMDB0001403	Lipids and lipid-like molecules	Fatty Acyls	Eicosanoids	**	C00696	37.3	0	M+FA-H	C20H32O5	0.984558786	5900.853997	6034.423515	6457.852748	2474.370381	2361.309186	2638.982467	3278.323249	3921.329128	3664.055176
9.31_473.2759m/z	473.2759166	9.311316667	neg	1(3)-glyceryl-PGF2α	36228	Unclassified	Unclassified	Unclassified			37.3	0	M+FA-H	C23H40O7	0.724051837	701.5852464	895.5698263	771.599433	1615.540568	1556.520847	1841.710122	520.4421992	791.6192398	580.569432
9.32_429.1509m/z	429.150866	9.321783333	neg	Kinetin	HMDB0012245	Organoheterocyclic compounds	Imidazopyrimidines	Purines and purine derivatives	***	C08272	37.3	0	2M-H	C10H9N5O	-7.619046819	12731.78925	12704.32876	8741.095294	12281.74135	15472.71893	12574.79823	10128.35278	10944.91319	14203.37082
9.48_593.1307m/z	593.1307262	9.482033333	neg	Pelargonidin 3-(6"-caffeylglucoside)	46799	Unclassified	Unclassified	Unclassified			37.3	0	M-H	C30H26O13	1.113800718	4986.294889	4518.681963	4038.435977	4025.22777	4201.52051	4161.744761	4354.75759	4220.677077	4435.851565
0.54_885.5499m/z	885.5499488	0.535283333	neg	PI(16:0/22:4(10Z,13Z,16Z,19Z))	HMDB0009793	Lipids and lipid-like molecules	Glycerophospholipids	Glycerophosphoinositols	*		37.2	0	M-H	C47H83O13P	0.108200152	753.729934	560.5730307	1996.695085	0.001403199	0.001403199	0.001403199	660.8721306	0.001403199	203.0950826
0.67_693.1745m/z	693.1744621	0.6701	neg	D-Arabino-hexos-2-ulose	86597	Organic oxygen compounds	Organooxygen compounds	Carbohydrates and carbohydrate conjugates	*		37.2	0	M-H2O-H	C24H40O24	1.897123896	1129.50145	1125.667545	1303.901227	997.6668257	588.6792587	791.356643	565.5851252	335.7923489	447.0824605
0.80_234.0546n	279.0528521	0.800366667	neg	cis-3-(6-Hydroxy-7-methoxy-5-benzofuranyl)acrylic acid	1203	Unclassified	Unclassified	Unclassified			37.2	0	M+FA-H, 2M-H	C12H10O5	7.800665069	453983.2649	406882.0473	370317.5358	285127.9497	332854.495	309852.4022	484904.9816	447737.5897	438855.815
0.80_339.1099m/z	339.1099356	0.800366667	neg	3,4-bis(methylene)-Hexanedioic acid	3791	Unclassified	Unclassified	Unclassified			37.2	0	2M-H	C8H10O4	4.100026078	751.0905325	1085.095385	1347.897642	865.8558855	0.001403199	1134.58665	0.001403199	360.1320038	412.911495
0.84_249.0424m/z	249.0424422	0.841733333	neg	AG-183	45250	Unclassified	Unclassified	Unclassified			37.2	0	M-H2O-H	C13H8N4O3	2.39943492	7886.616661	6836.77489	7291.69018	9369.741552	10708.24881	8723.640487	9165.152846	9941.399248	10268.10074
1.10_379.0374m/z	379.0374488	1.10065	neg	3-(trifluoromethyl)-Benzoic acid	1959	Unclassified	Unclassified	Unclassified			37.2	0	2M-H	C8H5F3O2	-9.479948926	3340.066814	2574.331143	3594.274128	2693.029197	1798.499433	2898.326346	3387.426582	2465.261284	2040.621148
1.16_324.9783m/z	324.978278	1.160166667	neg	Acesulfame-K	73544	Unclassified	Unclassified	Unclassified			37.2	0	2M-H	C4H5NO4S	-7.064290101	4887.517845	3958.399613	3360.307026	5393.738062	4191.493365	3491.020599	5101.715168	3717.044666	3078.031254
1.34_184.0001m/z	184.00013	1.335933333	neg	Phospho-L-serine	297	Unclassified	Unclassified	Unclassified			37.2	0	M-H	C3H8NO6P	-8.200501129	51.96422636	29.27064506	50.22974301	64.02189381	50.40711136	84.80058285	26.73013658	20.93092632	48.49669609
1.46_300.9863m/z	300.9863003	1.459966667	neg	Ascorbate 2-sulfate	65843	Unclassified	Unclassified	Unclassified			37.2	0	M+FA-H	C6H8O9S	-3.146051234	2156.767304	2851.509225	2501.792764	3813.098126	3277.475007	3664.118358	3523.158459	2528.915926	3145.520569
1.53_248.0403m/z	248.0402913	1.534483333	neg	2-Amino-4-oxo-6-(1,2-dioxoprolyl)-7,8-dihydroxypteridine	6226	Unclassified	Unclassified	Unclassified			37.2	0	M-H2O-H	C9H9N5O5	-8.372542449	6609.57129	5116.175272	5202.116713	6656.065994	6663.036681	6593.446649	5384.683606	6805.392122	5566.070542
1.55_188.0178m/z	188.0178015	1.553483333	neg	BAY-11-7082	45239	Unclassified	Unclassified	Unclassified			37.2	0	M-H2O-H	C10H9NO2S	1.174159196	1079.578411	1033.442145	904.6842166	2023.657864	1927.595458	1990.914458	1214.720293	1710.397541	1322.097126
1.96_340.0676m/z	340.067576	1.95955	neg	HBOA glucuronide	HMDB0240378	Organic oxygen compounds	Organooxygen compounds	Carbohydrates and carbohydrate conjugates			37.2	0	M-H	C14H15NO9	0.502334515	1470.96265	1280.422751	1144.655513	0.001403199	42.09580176	0.001403199	1392.20865	1351.370762	969.2098393
11.91_269.2123m/z	269.212343	11.91278333	neg	3S,7,11-Trimethyl-6E,10-dodecadien-1-ol	46103	Lipids and lipid-like molecules	Fatty Acyls	Fatty alcohols			37.2	0	M+FA-H	C15H28O	0.555674465	1606.919519	1510.341407	1682.084467	1563.63018	1817.915944	1152.782028	1589.466703	1204.097324	1380.065517
15.10_299.0265m/z	299.0264654	15.09626667	neg	3-carboxy-2,3-dihydroxypropanoate	HMDB0062642	Organic oxygen compounds	Organooxygen compounds	Carbohydrates and carbohydrate conjugates			37.2	0	2M-H	C4H6O6	2.886432544	35153.37731	17660.69337	9515.903031	2745.960873	3445.158205	2474.934802	7332.162326	5266.483467	4433.252584
2.36_592.9683m/z	592.9682668	2.36465	neg	5-Hydroxythiabendazole sulfate	2826	Unclassified	Unclassified	Unclassified			37.2	0	2M-H	C10H7N3O4S2	-0.087964353	4564.692184	4773.105103	4401.345515	7671.019558	8720.795536	8085.127036	5836.496463	7164.463417	7040.393501
2.73_219.0407m/z	219.0407176	2.734316667	neg	Quinazoline acetic acid (3(2H)-Quinazolineacetic acid, 1,4-dihydro-2,4-dioxo-)	893	Unclassified	Unclassified	Unclassified			37.2	0	M-H	C10H8N2O4	-1.875376002	613.0864629	575.6793723	716.8298257	961.8113784	883.2939484	861.0750791	728.765562	760.8279181	811.30284
3.06_286.0910m/z	286.0909607	3.05735	neg	Tetraphyllin B	86584	Organic oxygen compounds	Organooxygen compounds	Carbohydrates and carbohydrate conjugates	*		37.2	0	M-H	C12H17NO7	-7.888451168	15027.82093	15194.05321	17116.06752	35029.35905	36902.60213	33177.98593	24888.9529	25638.80989	24776.8463
3.48_346.0926m/z	346.0926104	3.484883333	neg	UK-121,265	HMDB0060893	Benzenoids	Benzene and substituted derivatives	Phenylpropanes	*		37.2	0	M-H2O-H	C16H14F3N5O2	1.348018908	1251.744369	1608.147312	1546.748357	2222.003078	1853.045133	1685.103494	1449.561547	1422.022483	1634.951964
3.56_472.0249m/z	472.0249218	3.564783333	neg	ADP	HMDB0001341	Nucleosides, nucleotides, and analogues	Purine nucleotides	Purine ribonucleotides	***	C00008	37.2	0	M+FA-H	C10H15N5O10P2	-6.313468098	894.9550162	1187.595099	1267.342126	1625.443514	1947.260193	1600.412943	1324.25515	1347.981332	1056.080981
3.75_356.0479m/z	356.0479233	3.747833333	neg	Glucocochlearin	HMDB0302326	Unclassified	Unclassified	Unclassified			37.2	0	M-H2O-H	C11H21NO9S2	-0.023425591	1691.173637	1339.863139	1312.984047	2419.475554	2782.623544	1649.09708	1541.66668	1562.489866	1783.089607
3.77_327.0641m/z	327.0641169	3.770416667	neg	3'-Amino-3'-deoxy-AMP	64574	Unclassified	Unclassified	Unclassified			37.2	0	M-H2O-H	C10H15N6O6P	8.348134302	2743.21407	2404.844376	2226.416617	750.520884	501.9296347	434.826318	1873.200643	1306.572294	1539.977562
3.78_401.1093m/z	401.1092513	3.779133333	neg	Veranisatin B	HMDB0040664	Lipids and lipid-like molecules	Prenol lipids	Terpene lactones	*		37.2	0	M+FA-H	C16H20O9	0.887877457	512.1356459	658.998434	709.5369402	109.7653917	190.0952392	114.204728	762.5351664	904.6467571	698.3682993
3.98_405.0488m/z	405.0488217	3.97985	neg	1-O-p-Coumaroyl-(b-D-glucose 6-O-sulfate)	HMDB0041174	Phenylpropanoids and polyketides	Cinnamic acids and derivatives	Hydroxycinnamic acids and derivatives	*		37.2	0.62	M-H	C15H18O11S	-2.177650156	3620.393405	4432.588929	5484.889216	1158.645976	888.1816108	892.8401844	3313.557354	4528.565122	3693.171698
4.00_173.0552m/z	173.0551672	4.002233333	neg	2-Aminoacrylic acid	58164	Organic acids and derivatives	Carboxylic acids and derivatives	Amino acids, peptides, and analogues	**	C02218	37.2	0	2M-H	C3H5NO2	-9.267860815	289.83503	337.8239253	370.376328	18.55597564	54.84768481	70.46357714	230.0654605	310.5235596	313.7480376
4.05_251.1038m/z	251.1038244	4.046666667	neg	Alanyltyrosine	HMDB0028699	Organic acids and derivatives	Carboxylic acids and derivatives	Amino acids, peptides, and analogues	*		37.2	0	M-H	C12H16N2O4	0.372044063	302.9362742	266.402242	319.1360773	66.738018	109.7773851	157.0285341	181.1062097	164.5937731	195.9866945
4.18_333.1194m/z	333.1194432	4.181333333	neg	Mitomycin	590	Organoheterocyclic compounds	Indoles and derivatives	Indolequinones		C06681	37.2	0	M-H	C15H18N4O5	-2.993141813	1895.021584	1930.282391	2300.64943	3715.588757	3189.696164	3413.395351	3082.86611	2586.786486	2551.920014
4.23_177.0171m/z	177.0170583	4.234483333	neg	Oxamate	63389	Unclassified	Unclassified	Unclassified			37.2	0	2M-H	C2H3NO3	9.82370798	387.398388	84.5008354	114.3871905	25.92979478	163.1064524	435.4909261	272.5893144	268.0192817	275.9387028
4.26_350.1724m/z	350.1723517	4.2568	neg	PC(7:0/0:0)[U]	40331	Unclassified	Unclassified	Unclassified			37.2	0	M-H2O-H	C15H32NO7P	-3.917113817	263.8495496	344.1444196	524.0382573	556.3635766	647.7090821	537.5748889	796.2149081	1026.868521	759.6466654
4.26_363.0699m/z	363.0699382	4.2568	neg	Cibarian	67004	Unclassified	Unclassified	Unclassified			37.2	0	M-H2O-H	C12H18N2O12	4.724771629	2029.425703	2167.42621	2373.534016	379.414243	222.9559468	258.7666337	856.0731806	1151.881986	957.8154712
4.28_352.1379m/z	352.1379274	4.278183333	neg	Convoline	68633	Unclassified	Unclassified	Unclassified			37.2	0	M+FA-H	C16H21NO5	-7.319856422	11151.45822	11511.09245	11433.00226	6823.037762	6297.480497	6473.286133	10502.22211	11470.56532	11054.31948
4.32_255.0855m/z	255.0855069	4.3224	neg	Glutamyl-Glutamine	85731	Organic acids and derivatives	Carboxylic acids and derivatives	Amino acids, peptides, and analogues	*		37.2	0	M-H2O-H	C10H16N3O6-	-2.050989917	1509.782705	1363.437584	1548.065635	1701.470968	1455.510963	1528.754632	1324.933742	1551.751532	1499.058885
4.45_368.1229m/z	368.1229347	4.454616667	neg	Bialaphos	63603	Unclassified	Unclassified	Unclassified			37.2	0	M+FA-H	C11H22N3O6P	0.339844367	105.8881682	209.6575183	94.44721485	233.4122695	265.3831728	227.7170604	131.3897198	231.9797221	177.1649337
4.54_300.0889m/z	300.0889401	4.54425	neg	1-Hydroxy-3-methoxy-10-methylacridone	86186	Organoheterocyclic compounds	Quinolines and derivatives	Benzoquinolines	*		37.2	0	M+FA-H	C15H13NO3	4.680545867	902.8511815	1268.366656	926.1767015	1219.932567	1234.407951	1189.188341	625.9592659	1088.224026	801.373241
4.61_338.0784m/z	338.0783786	4.611883333	neg	Balsalazide	85458	Organoheterocyclic compounds	Azobenzenes	Unclassified			37.2	0	M-H2O-H	C17H15N3O6	0.376677311	558.7470721	227.7201524	493.953228	748.0182418	776.4841995	507.2094702	671.0781376	488.5611916	515.117955
4.64_367.0132m/z	367.0132075	4.641416667	neg	2,4-DINITROPHENOL	43994	Unclassified	Unclassified	Unclassified			37.2	0	2M-H	C6H4N2O5	-9.66940344	913.4215486	1148.588235	1041.560622	1656.318349	1419.971708	1255.054871	867.1706576	873.2892952	820.4712551
4.68_465.1980m/z	465.1980487	4.682483333	neg	Serinyl-Gamma-glutamate	85946	Organic acids and derivatives	Carboxylic acids and derivatives	Amino acids, peptides, and analogues	*		37.2	0	2M-H	C8H15N3O5	6.400523229	2611.21681	2806.3106	3212.895166	3554.865058	2995.936473	3595.778102	5217.403328	4474.494495	5247.08291
4.73_439.1122m/z	439.112238	4.725416667	neg	(R)-(Homo)2-citrate	71244	Unclassified	Unclassified	Unclassified			37.2	0	2M-H	C8H12O7	6.609622086	3084.612933	3118.628321	3371.350639	356.5968334	440.4031612	149.3609252	1886.837512	2361.625143	2397.252625
4.77_347.1350m/z	347.1349625	4.769883333	neg	Phenylalanylhistidine	HMDB0028997	Organic acids and derivatives	Carboxylic acids and derivatives	Amino acids, peptides, and analogues	*		37.2	0	M+FA-H	C15H18N4O3	-3.742701378	7078.025494	8032.992383	8536.013067	11525.20975	10846.53062	11283.93552	9454.84557	9351.085171	9078.336966
4.79_507.0735m/z	507.0734978	4.792316667	neg	Benzoyl meso-tartaric acid	94080	Benzenoids	Benzene and substituted derivatives	Benzoic acids and derivatives			37.2	0	2M-H	C11H10O7	-8.917875402	2337.908676	2377.461827	2168.21642	347.5158779	547.8153153	461.8708374	1866.017923	1886.549927	2314.587686
4.84_549.1619m/z	549.161921	4.836566667	neg	Urdamycinone F	63761	Unclassified	Unclassified	Unclassified			37.2	0	M+FA-H	C25H28O11	1.103576398	1569.58916	1492.259654	1410.274226	1202.972928	1075.127479	840.3812592	2061.554184	2110.40534	2142.397484
4.86_271.0648m/z	271.0647676	4.858383333	neg	Erythronic acid	45855	Organic oxygen compounds	Organooxygen compounds	Carbohydrates and carbohydrate conjugates	*		37.2	0	2M-H	C4H8O5	-8.463421989	1163.57559	1127.35746	1358.634074	320.2412085	483.2782216	381.3473406	902.2762796	961.0929094	1030.190571
4.86_673.0935m/z	673.0935417	4.858383333	neg	Guanosine diphosphate adenosine	HMDB0001501	Nucleosides, nucleotides, and analogues	(3'->5')-dinucleotides and analogues	(3'->5')-dinucleotides	*		37.2	0	M-H2O-H	C20H26N10O14P2	1.248357868	4376.101219	4123.782053	4718.552429	5403.396463	5445.231655	5371.283254	6121.261821	6342.749606	5986.794789
4.90_231.0407m/z	231.0407313	4.902316667	neg	AG-18	45241	Unclassified	Unclassified	Unclassified			37.2	0	M+FA-H	C10H6N2O2	-2.144575014	597.1719848	586.2830349	582.5968458	538.2458302	548.6988248	619.2057109	412.8795485	500.783284	183.1486039
4.92_370.0779m/z	370.0779161	4.92485	neg	Pancratistatin	67051	Unclassified	Unclassified	Unclassified			37.2	0	M+FA-H	C14H15NO8	-0.163598221	290.0010201	453.7411437	488.645622	646.4518083	521.9286092	480.9063962	499.4899595	746.3618904	493.1129303
5.06_325.0813m/z	325.0813145	5.05975	neg	AG-494	45230	Unclassified	Unclassified	Unclassified			37.2	0	M+FA-H	C16H12N2O3	-6.000531562	288.5862733	203.5604463	429.6513932	524.5628436	267.23092	636.5568218	499.0837407	349.7737102	553.292645
5.08_549.1618m/z	549.1617767	5.080566667	neg	Sergeolide	67199	Unclassified	Unclassified	Unclassified			37.2	0	M+FA-H	C25H28O11	0.817346561	1576.821283	1522.165765	1672.580184	259.0615916	165.6139783	218.3719591	1195.580632	781.8735123	692.9616956
5.16_465.1980m/z	465.1980333	5.155116667	neg	Threoninyl-Asparagine	85949	Organic acids and derivatives	Carboxylic acids and derivatives	Amino acids, peptides, and analogues	*		37.2	0	2M-H	C8H15N3O5	6.367545307	5118.575377	5947.826512	4417.988637	1366.761353	1045.980872	1293.340269	2740.663398	2698.191122	2217.5295
5.18_509.1942m/z	509.1942301	5.1777	neg	Karwinskione	68031	Unclassified	Unclassified	Unclassified			37.2	0	M-H2O-H	C32H32O7	-5.172350698	21688.35395	23620.55173	22541.68744	16353.05347	16230.87724	15734.1344	24299.01892	22371.72895	22654.24069
5.18_713.1568m/z	713.1568465	5.1777	neg	Delphinidin 3-glucoside-5-(6-acetylglucoside)	47110	Unclassified	Unclassified	Unclassified			37.2	0	M+FA-H	C29H32O18	-0.33004644	2260.834032	1707.324014	2161.039614	1187.621766	957.066712	875.3604087	1384.446113	1475.972305	1650.178437
5.20_489.1982m/z	489.1981876	5.20005	neg	6-O-Oleuropeoylsucrose	HMDB0038117	Lipids and lipid-like molecules	Saccharolipids	Unclassified	*		37.2	0	M-H2O-H	C22H36O13	0.860947347	2480.644319	3105.752261	2856.734948	0.001403199	0.001403199	94.78681561	961.9576462	1018.490982	1130.427553
5.20_549.2323m/z	549.2323034	5.20005	neg	16a-Hydroxydydrogesterone glucoronide	2520	Unclassified	Unclassified	Unclassified			37.2	0	M+FA-H	C27H36O9	-3.633699035	899.0764449	872.1119261	1045.24879	927.3421946	1445.435365	1138.794032	862.4179395	933.6766742	1110.828064
5.22_490.1549m/z	490.1548865	5.222166667	neg	Lucuminamide	87873	Organic oxygen compounds	Organooxygen compounds	Carbohydrates and carbohydrate conjugates	*		37.2	0	M+FA-H	C19H27NO11	-3.879743891	4726.318886	4181.093585	3690.988999	3898.785643	3840.983148	3795.833436	4977.035044	4977.264824	5151.08901
5.41_597.1181m/z	597.1180632	5.406433333	neg	8-Anilino-1-naphthalene sulfonate	HMDB0061854	Benzenoids	Naphthalenes	Naphthalene sulfonic acids and derivatives		C11326	37.2	0	2M-H	C16H13NO3S	3.529418242	2757.069557	2113.418307	2075.40822	3182.307358	3533.24937	3060.236606	3242.816033	2819.210872	1847.466692
5.53_341.1584m/z	341.1584436	5.525416667	neg	alhpa-tocopheronic acid	53843	Lipids and lipid-like molecules	Prenol lipids	Quinones and hydroquinones			37.2	0	M+FA-H	C16H24O5	-7.202474337	13798.85632	12806.14385	13971.93176	9415.204621	10939.62276	9030.947024	10991.83448	11080.44651	10935.40226
5.59_332.1538m/z	332.1538374	5.5933	neg	Pirenzepine	1973	Organoheterocyclic compounds	Benzodiazepines	1,4-benzodiazepines		C07508	37.2	0	M-H2O-H	C19H21N5O2	6.132501148	883.6250993	668.4010602	825.6715276	287.7330452	516.9329191	290.6232097	575.0073247	662.1966248	787.7116488
5.67_439.0709m/z	439.070869	5.666566667	neg	PURPUROGALLIN	43934	Unclassified	Unclassified	Unclassified			37.2	0	2M-H	C11H8O5	8.632053727	4786.522126	4807.289116	4906.777104	5543.920477	6376.162013	5336.891854	5651.660891	5999.717212	7075.133268
5.81_273.1345m/z	273.1345225	5.806016667	neg	(-)-12-hydroxy-9,10-dihydrojasmonic acid	45945	Lipids and lipid-like molecules	Fatty Acyls	Octadecanoids			37.2	0	M+FA-H	C12H20O4	0.703600322	1011.837503	1123.119548	1081.33378	1638.38234	1542.591461	1459.090361	2016.471986	1566.73039	1704.499341
5.81_301.1270m/z	301.1269832	5.806016667	neg	Decarbamoylsaxitoxin	HMDB0038319	Phenylpropanoids and polyketides	Saxitoxins, gonyautoxins, and derivatives	Unclassified			37.2	0	M+FA-H	C9H16N6O3	1.530177683	949.2782637	849.8382747	746.3544013	1131.82136	1153.046897	1091.01187	1492.255221	1400.902344	1187.581226
5.85_583.3127m/z	583.3126587	5.84575	neg	16-Epivellosimine	64338	Unclassified	Unclassified	Unclassified			37.2	0	2M-H	C19H20N2O	8.229382543	3698.290154	3936.713069	4220.173887	1466.277844	631.082807	1383.23122	5853.623448	5457.845072	5403.644664
5.92_379.1375m/z	379.1375288	5.9168	neg	Pseudaminic acid	73533	Unclassified	Unclassified	Unclassified			37.2	0	M+FA-H	C13H22N2O8	5.118475737	1117.612307	1115.735575	1289.993621	1319.192305	1506.083671	1548.286877	866.8592985	1220.168632	1463.898627
6.01_423.1666m/z	423.1665769	6.007266667	neg	Niveusin C	HMDB0034474	Lipids and lipid-like molecules	Prenol lipids	Terpene lactones	*	C09517	37.2	0	M+FA-H	C20H26O7	1.377394129	1412.530201	1681.245405	1362.06915	1219.429284	751.4431993	1021.887042	2118.470181	2365.524097	2251.670521
6.01_539.2502m/z	539.2501601	6.007266667	neg	flumethasone pivalate	4030	Lipids and lipid-like molecules	Steroids and steroid derivatives	Pregnane steroids			37.2	0	M+FA-H	C27H36F2O6	8.016110291	179831.3406	185733.8161	192888.7476	185407.3557	184408.3465	188645.9906	151833.5394	156750.9266	154368.0673
6.03_507.2086m/z	507.2085607	6.028183333	neg	Valerosidatum	71536	Unclassified	Unclassified	Unclassified			37.2	0	M+FA-H	C21H34O11	0.532114889	1249.143977	1329.453435	1475.827666	2670.299966	2395.028698	2454.376212	1148.214082	1132.561564	1262.258111
6.07_511.2551m/z	511.2551047	6.07	neg	Cinncassiol D2 glucoside	HMDB0034679	Lipids and lipid-like molecules	Prenol lipids	Terpene glycosides	*		37.2	0	M-H2O-H	C26H42O11	0.440595161	1780.786435	2535.937591	1984.493581	976.2579955	1286.540714	975.1889956	716.0136136	636.8412876	685.1564265
6.12_453.1384m/z	453.1384449	6.123483333	neg	Nodakenin	67571	Unclassified	Unclassified	Unclassified			37.2	0	M+FA-H	C20H24O9	-4.386417571	19742.36605	21967.50874	22110.08682	25682.34564	27360.02363	26522.36912	18114.31936	19582.4917	20278.7836
6.15_345.0985m/z	345.0984642	6.154666667	neg	2',4-Dihydroxy-4',6'-dimethoxychalcone	HMDB0030875	Phenylpropanoids and polyketides	Linear 1,3-diarylpropanoids	Chalcones and dihydrochalcones	*		37.2	0	M+FA-H	C17H16O5	1.625197907	1289.35385	1226.439079	1280.464349	34.41733999	0.001403199	40.55804382	40.25954732	53.91246033	25.49280597
6.16_737.0522m/z	737.0521732	6.161866667	neg	(-)-Epicatechin sulfate	HMDB0012467	Phenylpropanoids and polyketides	Flavonoids	Sulfated flavonoids	*		37.2	0	2M-H	C15H13O9S-	4.598151156	2421.83541	3080.231233	2525.728633	1640.339938	1678.952334	2348.299601	1023.734931	1688.751106	1449.606657
6.17_409.1120m/z	409.1119841	6.171316667	neg	1-Isothiocyanato-6-(methylsulfinyl)hexane	HMDB0038461	Organosulfur compounds	Sulfoxides	Unclassified			37.2	0	2M-H	C8H15NOS2	0.605461022	77760.05947	83094.51428	85674.58981	72873.26302	74874.349	72377.96689	71189.60835	75246.07218	78892.4503
6.20_255.1240m/z	255.123972	6.195716667	neg	(3S,7R)-iso-jasmonic acid	36072	Lipids and lipid-like molecules	Fatty Acyls	Octadecanoids			37.2	0	M+FA-H	C12H18O3	0.831534765	451.1129961	333.8345928	483.2177049	580.8436799	502.0175685	671.2637939	422.5925375	551.4758606	507.8863765
6.33_624.3395m/z	624.3395229	6.331216667	neg	dihydro-α-ergocryptine	4042	Unclassified	Unclassified	Unclassified			37.2	0	M+FA-H	C32H45N5O5	-1.293752736	8741.340616	11773.2002	10775.44722	2974.111996	3463.373504	2362.795923	6870.859632	6335.828222	8023.019507
6.43_241.1448m/z	241.1447753	6.433933333	neg	p-Menth-1-en-9-ol acetate	HMDB0037301	Lipids and lipid-like molecules	Prenol lipids	Monoterpenoids	*		37.2	0	M+FA-H	C12H20O2	1.236777039	504.7731803	460.5469958	477.8099499	385.3119481	438.237908	328.3205871	519.310383	312.5109284	357.8413285
6.44_483.0913m/z	483.0913386	6.442716667	neg	Riboflavin cyclic-4',5'-phosphate	HMDB0059614	Organoheterocyclic compounds	Pteridines and derivatives	Alloxazines and isoalloxazines		C16071	37.2	0	M+FA-H	C17H19N4O8P	-2.087305445	1704.955549	1243.971114	1449.841233	0.001403199	0.001403199	0.001403199	0.001403199	0.001403199	0.001403199
6.48_258.0879m/z	258.0879148	6.477433333	neg	CAY10572	45627	Unclassified	Unclassified	Unclassified			37.2	0	M+FA-H	C12H11N3O	-2.346456288	819.3183597	930.0579058	1075.141561	1892.506912	1709.448228	1466.67761	1350.326859	1496.789621	1471.81268
6.86_547.1027m/z	547.1026727	6.86375	neg	Isoginkgetin	HMDB0033937	Phenylpropanoids and polyketides	Flavonoids	Biflavonoids and polyflavonoids			37.2	4.38	M-H2O-H	C32H22O10	-1.38327973	37030.24886	39397.23787	35001.02955	39706.60768	42603.14383	38331.15517	32867.02248	36626.79891	32397.72957
6.88_345.1457m/z	345.1456674	6.8759	neg	coumarin-SAHA	64949	Unclassified	Unclassified	Unclassified			37.2	0	M-H	C18H22N2O5	0.208170332	6306.126794	6881.600906	6170.963908	5339.970384	6793.965993	5332.910103	5347.70519	6274.432681	5676.146089
7.06_323.1613m/z	323.1612682	7.055233333	neg	Leucyl-Phenylalanine	85839	Organic acids and derivatives	Carboxylic acids and derivatives	Amino acids, peptides, and analogues	*		37.2	0	M+FA-H	C15H22N2O3	0.081975233	966.3670967	968.8057549	933.2975189	1296.570618	1182.985842	992.0774913	846.6507907	950.2209091	686.8186065
7.15_290.1012m/z	290.1012271	7.147366667	neg	N-Hydroxynorcocaine	1906	Unclassified	Unclassified	Unclassified			37.2	0	M-H	C15H17NO5	-7.451093907	10000.20948	10773.1953	10406.83517	16622.2025	17331.91426	16073.77383	11196.26085	13490.71421	12767.9175
7.30_751.3140m/z	751.3139531	7.29525	neg	XE991	69700	Unclassified	Unclassified	Unclassified			37.2	0	2M-H	C26H20N2O	8.112303727	20267.34574	23214.41661	24737.00263	2999.328367	3736.009898	3535.496068	10804.9265	11927.63785	14711.0756
7.31_531.2216m/z	531.2216443	7.314366667	neg	Austalide I	86761	Organoheterocyclic compounds	Benzopyrans	1-benzopyrans	*		37.2	0	M+FA-H	C27H34O8	-3.962400691	6128.961985	6386.95584	5742.271836	7370.714844	8541.500268	7335.921494	6323.553014	6721.608727	8064.146451
7.42_251.0901m/z	251.0901386	7.41815	neg	Threoninyl-Serine	85963	Organic acids and derivatives	Carboxylic acids and derivatives	Amino acids, peptides, and analogues	*		37.2	0	M+FA-H	C7H14N2O5	8.074840956	1354.17637	1248.857535	1393.174039	671.9634702	741.7215582	664.9791452	1686.111313	1655.767322	1727.126032
7.42_362.1202m/z	362.1201972	7.41815	neg	Ceranib-2	96481	Unclassified	Unclassified	Unclassified			37.2	0	M-H2O-H	C25H19NO3	4.053295942	928.4325259	1066.620994	1089.043663	1314.667926	1594.444516	1317.56356	703.3394306	980.6536563	1301.843907
7.51_381.1327m/z	381.132699	7.50615	neg	Carbendazim	68667	Organoheterocyclic compounds	Benzimidazoles	2-benzimidazolylcarbamic acid esters	*	C10897	37.2	0	2M-H	C9H9N3O2	2.675369852	340.9490881	517.7595781	418.839106	316.9347448	470.4179597	336.4266251	302.0090927	462.9451654	486.956748
7.53_533.1258m/z	533.1257876	7.527033333	neg	Sulfamoxole	85585	Benzenoids	Benzene and substituted derivatives	Benzenesulfonamides			37.2	0	2M-H	C11H13N3O3S	-4.60651933	8525.251465	9072.398109	9435.493888	16050.12947	15257.36362	15311.43039	11503.93398	12926.42785	12714.98381
7.56_457.1698m/z	457.1698	7.556516667	neg	Deoxynivalenol 3-glucoside	94399	Lipids and lipid-like molecules	Prenol lipids	Sesquiterpenoids	*		37.2	0	M-H	C21H30O11	-3.787444515	2003.152927	2413.61153	2609.52468	4158.876768	3808.515342	4120.767831	1539.93559	2005.559414	1508.869309
7.65_509.2370m/z	509.2370375	7.6458	neg	Dehydroepiandrosterone 3-glucuronide	57963	Lipids and lipid-like molecules	Steroids and steroid derivatives	Steroidal glycosides	*		37.2	0	M+FA-H	C25H36O8	-4.70337051	11201.99792	13075.09535	11745.28173	11894.52961	10647.96512	11424.85992	8693.031983	9995.862483	10277.38556
7.92_501.2261m/z	501.2261497	7.91555	neg	1-(5Z,8Z,11Z,14Z,17Z-eicosapentaenoyl)-glycero-3-phosphate	HMDB0062308	Lipids and lipid-like molecules	Glycerophospholipids	Glycerophosphates	*		37.2	0	M+FA-H	C23H37O7P	0.562553709	3105.802933	2987.617436	3669.51538	4964.846989	4833.733559	5295.888345	3030.58729	4380.892845	3944.038054
7.97_415.1590m/z	415.1589585	7.9723	neg	Allobarbital	85061	Unclassified	Unclassified	Unclassified			37.2	0	2M-H	C10H12N2O3	-8.048696848	9388.02123	9017.02108	9705.888173	8729.412972	8742.408386	9534.836538	6021.479173	6817.817831	6527.250241
8.13_645.2511m/z	645.2511381	8.129733333	neg	Gliclazide	HMDB0015252	Benzenoids	Benzene and substituted derivatives	Benzenesulfonamides			37.2	0	2M-H	C15H21N3O3S	-3.575241227	4752.215703	4504.076748	4972.94916	7383.825846	6945.420755	6909.515116	4173.307135	5216.928485	4887.426705
8.64_439.1588m/z	439.15885	8.635	neg	Glaucarubolone	67183	Unclassified	Unclassified	Unclassified			37.2	0	M+FA-H	C20H26O8	-5.380236621	6827.183047	7505.021852	6788.978451	10689.40998	9994.629056	9702.023746	4837.238798	4595.804665	4592.584968

8.65_383.1715m/z	383.1715459	8.654333333	neg	N'-Hydroxymethylnorcotinine	58037	Organoheterocyclic compounds	Pyridines and derivatives	Pyrrolidinylpyridines	*		37.2	0	2M-H	C10H12N2O2	-2.42830946	1548.959197	1544.703052	1505.916948	2449.33231	1916.511069	2048.955607	1036.694668	1212.956623	1173.515453
8.78_512.2691m/z	512.2690865	8.782416667	neg	Sulfolithocholylglycine	HMDB0002639	Lipids and lipid-like molecules	Steroids and steroid derivatives	Bile acids, alcohols and derivatives	*	C11301	37.2	0	M-H	C26H43NO7S	0.660928309	2253.623986	2352.174709	2896.844756	82.51268974	79.33031563	224.9791902	2380.991365	2328.548187	2219.188655
8.85_369.1744m/z	369.1743679	8.851966667	neg	Piperonyl sulfoxide	HMDB0258607	Unclassified	Unclassified	Unclassified			37.2	0	M+FA-H	C18H28O3S	0.769014096	2102.850278	2622.239193	2696.60723	2520.316363	2108.682674	2329.959426	1938.731207	2385.508338	2669.577986
8.94_277.0331m/z	277.0330551	8.944866667	neg	N-Acetyldjenkolic acid	HMDB0029421	Organic acids and derivatives	Carboxylic acids and derivatives	Amino acids, peptides, and analogues	*		37.2	0	M-H2O-H	C9H16N2O5S2	2.812488774	723.8241849	619.2525547	603.7729608	566.3214955	707.0278212	819.6747828	678.2649151	691.1009541	698.89363
9.10_453.1769m/z	453.1768962	9.095016667	neg	Vernoflexuoside	67821	Unclassified	Unclassified	Unclassified			37.2	0	M+FA-H	C21H28O8	0.674810169	2103.966792	1522.408932	2145.021982	1807.206356	1698.317492	1552.076253	1605.199719	1137.732395	1618.694055
9.97_515.2266m/z	515.2265718	9.969833333	neg	PI(12:0/0:0)	LMGP06050007	Lipids and lipid-like molecules	Glycerophospholipids	Glycerophosphoinositols			37.2	0	M-H	C21H41O12P	0.551867076	2127.136458	1994.979441	2039.359848	1902.508847	1955.994107	1709.905933	1502.630456	1648.849655	1212.488586
0.69_375.1386m/z	375.138641	0.692166667	neg	N-(2,5-Dihydroxyphenyl)pyridinium(1+)	95233	Benzenoids	Phenols	Benzenediols			37.1	0	2M-H	C11H10NO2+	9.598165557	1207.675403	1711.641606	1320.775358	1740.79236	1894.787196	1461.632435	1002.380306	981.5285187	1276.871594
0.80_199.9693m/z	199.9693138	0.800366667	neg	Cysteine-S-sulfate	HMDB0000731	Organic acids and derivatives	Carboxylic acids and derivatives	Amino acids, peptides, and analogues	**	C05824	37.1	0	M-H	C3H7NO5S2	0.128907284	8330.952892	7956.003129	9612.065543	9517.696504	10187.15848	10649.63084	10627.90839	11137.40161	9391.935064
0.80_411.0589m/z	411.0588855	0.800366667	neg	1-(5'-Phosphoribosyl)-5-formamido-4-imidazolecarboxamide	3506	Nucleosides, nucleotides, and analogues	Imidazole ribonucleosides and ribonucleotides	1-ribosyl-imidazolecarboxamides	**	C04734	37.1	0	M+FA-H	C10H15N4O9P	8.244346535	11329.76468	13910.31462	13205.78959	23064.10765	24766.86152	22971.60268	16620.12342	15575.9644	17866.78696
0.82_335.0428m/z	335.0428383	0.819566667	neg	Methylthiobenzoic acid	2538	Unclassified	Unclassified	Unclassified			37.1	0	2M-H	C8H8O2S	3.314080877	8532.99522	7527.956662	8467.500659	8854.157915	12794.95546	12247.42551	9698.433012	9201.826355	11210.95589
0.84_269.0546m/z	269.0546316	0.841733333	neg	A771726	HMDB0060945	Benzenoids	Benzene and substituted derivatives	Trifluoromethylbenzenes	*		37.1	0	M-H	C12H9F3N2O2	1.095543329	4770.911306	5285.256246	5616.171648	3982.548125	4746.913377	4006.244461	5106.064646	4900.358261	5677.386244
0.84_307.0843m/z	307.0843468	0.841733333	neg	1-(2-Thienyl)-1-butanone	HMDB0032935	Organic oxygen compounds	Organooxygen compounds	Carbonyl compounds			37.1	0.142	2M-H	C8H10OS	3.737067727	15991.87398	15761.02353	16626.09336	16479.95761	17866.03386	17295.50138	15319.75628	19312.08929	17554.15773
0.88_343.0456m/z	343.0455778	0.884633333	neg	8-Methoxycoumestrol	48340	Phenylpropanoids and polyketides	Isoflavonoids	Coumestans	*		37.1	0	M+FA-H	C16H10O6	-1.2182332	9075.704115	9763.098696	9773.291388	14234.96043	7461.874035	10341.94419	9975.501169	8370.524364	5247.980518
1.16_217.0161m/z	217.0160848	1.160166667	neg	Diphenyl disulfide	87947	Benzenoids	Benzene and substituted derivatives	Unclassified	*		37.1	0	M-H	C12H10S2	4.4442581	13806.53329	20431.00753	20238.2485	21583.29604	26152.50593	22212.89225	23178.0953	19312.44378	22854.34145
1.16_549.0588m/z	549.0587736	1.160166667	neg	Phosphophosphinate	69259	Unclassified	Unclassified	Unclassified			37.1	0	2M-H	C6H15NO7P2	2.363077986	12573.19775	11530.91537	23624.45276	15776.64825	21005.54538	18573.00092	13562.75431	13948.44238	19063.99564
1.25_208.0452m/z	208.0451692	1.246333333	neg	3-Hydroxy-L-glutamate	65897	Unclassified	Unclassified	Unclassified			37.1	0	M+FA-H	C5H9NO5	-6.783941928	226.5125215	249.815195	264.0371111	109.6108088	122.4962972	105.8748639	134.4096184	159.3220482	113.3691925
1.34_315.0838m/z	315.0838336	1.335933333	neg	(R)(-)-Allantoin	63395	Unclassified	Unclassified	Unclassified			37.1	0	2M-H	C4H6N4O3	9.901835283	620.5872887	906.3631105	729.3525684	1096.069689	760.4562997	936.9714467	730.2784107	823.5562376	772.9263247
1.59_212.0542m/z	212.0542262	1.5934	neg	N-Acetyl-L-2-amino-6-oxopimelate	63452	Unclassified	Unclassified	Unclassified			37.1	0	M-H2O-H	C9H13NO6	-9.606378827	15813.59758	14243.01222	14579.93114	13560.23287	12994.03176	13283.64417	14522.71617	15215.32526	14914.72113
1.63_202.0183m/z	202.0183071	1.631516667	neg	4-methylthiazole-5-acetic-acid	1877	Unclassified	Unclassified	Unclassified			37.1	0	M+FA-H	C6H7NO2S	2.259068824	680.1501165	627.0449508	640.4434467	243.4230786	240.9147618	177.3550062	547.1968789	703.6292738	577.0872442
1.63_252.1468m/z	252.1468456	1.631516667	neg	Prolyl-Arginine	HMDB0029011	Organic acids and derivatives	Carboxylic acids and derivatives	Amino acids, peptides, and analogues	*		37.1	0	M-H2O-H	C11H21N5O3	0.911462534	399.594156	415.1609296	442.7624119	551.4318925	532.0787753	642.1619883	721.8680898	640.790863	554.2263806
13.34_433.3450m/z	433.3450018	13.3426	neg	2',3'-Dihydro-phytomenadione	90237	Lipids and lipid-like molecules	Prenol lipids	Quinone and hydroquinone lipids	*		37.1	0	M-H2O-H	C31H48O2	-5.720746724	397.8021326	630.6677468	1005.123559	742.0901155	664.9153538	218.6332536	266.0723619	504.5772306	1807.725477
2.03_367.1412m/z	367.1412348	2.025516667	neg	cis-3-(1-Carboxy-ethyl)-3,5-cyclo-hexadiene-1,2-diol	69046	Unclassified	Unclassified	Unclassified			37.1	0	2M-H	C9H12O4	3.785247373	531.8951919	641.7062382	630.4862837	1049.303239	956.0957543	971.4618966	586.1063872	766.990782	803.8332705
2.07_243.0195n	288.0178319	2.070366667	neg	N-acetyl-2-carboxy Benzenesulfonamide	45011	Unclassified	Unclassified	Unclassified			37.1	0	M-H, M+FA-H	C9H9NO5S	-2.528870089	4795.521902	5017.048173	4981.745398	4491.342065	4648.359962	4231.423742	5193.522806	4470.48694	4504.126455
2.20_179.0186m/z	179.0186023	2.203283333	neg	Malic acid	HMDB0000156	Organic acids and derivatives	Hydroxy acids and derivatives	Beta hydroxy acids and derivatives	***	C00149	37.1	0	M+FA-H	C4H6O5	-8.385539969	1298.190849	1153.396621	1256.844729	1768.650818	1521.748597	1662.609984	1217.078052	1481.125815	1261.038288
2.28_310.0023m/z	310.0022842	2.2828	neg	Flurochloridone	HMDB0252372	Unclassified	Unclassified	Unclassified			37.1	30.8	M-H	C12H10Cl2F3NO	1.30759125	9289.297873	9028.587807	8701.556208	13018.56493	11607.05438	11699.25907	10936.97729	11715.16509	10582.6832
2.52_230.9969m/z	230.9969151	2.5212	neg	3,4-Dihydroxyphenylglycol O-sulfate	HMDB0001474	Organic acids and derivatives	Organic sulfuric acids and derivatives	Arylsulfates	*		37.1	0	M-H2O-H	C8H10O7S	0.130317466	657.7864365	536.8861596	702.6477416	340.5475514	233.9233615	249.108463	477.7463657	409.6348674	506.2650283
2.63_436.9749m/z	436.9749281	2.634083333	neg	Nitrophenylphosphate	6144	Organic acids and derivatives	Organic phosphoric acids and derivatives	Phosphate esters		C03360	37.1	0	2M-H	C6H6NO6P	-9.914773419	1719.97485	1459.598343	1556.763035	0.001403199	0.001403199	0.001403199	2327.907831	2408.155273	2629.769229
2.93_167.0211m/z	167.0211322	2.926066667	neg	Glyphosate-monoammonium	72370	Unclassified	Unclassified	Unclassified			37.1	0	M-H2O-H	C3H11N2O5P	-8.518499173	155.1493559	141.5430173	137.5378112	229.3681469	354.837744	225.2255964	144.211736	160.3157015	101.4403127
3.36_265.0831m/z	265.0830848	3.362333333	neg	5-(3,4-Dihydroxy-1,5-cyclohexadien-1-yl)-5-ethylbarbituric acid	1818	Unclassified	Unclassified	Unclassified			37.1	0	M-H	C12H14N2O5	0.336955302	700.5299452	848.1851781	735.5062025	869.9660613	1032.661584	905.1616961	783.0340461	715.4784616	568.4884056
3.47_277.1194m/z	277.1194345	3.47105	neg	Aminoglutethimide	HMDB0014501	Benzenoids	Benzene and substituted derivatives	Aniline and substituted anilines		C07617	37.1	0	M+FA-H	C13H16N2O2	0.231984062	278.0366743	290.5040202	218.7678642	879.1635901	504.7017898	516.8435867	238.1402253	535.5645182	559.2585514
3.77_257.1033m/z	257.1032997	3.770416667	neg	4'-Hydroxytrimethoprim	2962	Unclassified	Unclassified	Unclassified			37.1	0	M-H2O-H	C13H16N4O3	-3.982180204	648.4860213	576.0139375	680.8332381	980.1634798	757.2830607	425.4143932	559.9563996	670.2472239	677.3764462
3.80_267.0964m/z	267.0963899	3.801716667	neg	3-Hydroxynorpromazine	2143	Unclassified	Unclassified	Unclassified			37.1	0	M-H2O-H	C16H18N2OS	0.862767926	1325.542284	1288.8812	1243.675777	1851.920269	1890.421364	1929.092871	1280.477703	1271.999474	1197.24409
3.80_312.0800n	357.0830397	3.801716667	neg	Epithienamycin B	71661	Unclassified	Unclassified	Unclassified			37.1	0	M-H, M+FA-H	C13H16N2O5S	6.591717511	18574.48279	19466.59712	19835.59457	13845.33482	13365.42328	12740.51616	10811.38187	12055.35073	12269.0335
3.82_257.0044m/z	257.0044399	3.82415	neg	Ethoxzolamide	85362	Organoheterocyclic compounds	Benzothiazoles	Unclassified			37.1	0	M-H	C9H10N2O3S2	-6.076614467	856.0823797	860.8148213	1139.544545	911.6351334	863.6103244	639.1846668	958.5304507	652.8061231	793.7625266
3.89_406.0877n	451.0860662	3.8906	neg	5,7,3',5'-Tetrahydroxy-3,6,8,4'-tetramethoxyflavone	51770	Unclassified	Unclassified	Unclassified			37.1	0	M-H, M+FA-H	C19H18O10	-5.679130709	1036.159989	912.9616269	1140.512141	311.7868952	416.056594	792.80648	4312.778251	4704.813571	4618.09746
4.11_323.1113m/z	323.1113482	4.113883333	neg	Artecanin	67588	Unclassified	Unclassified	Unclassified			37.1	0	M+FA-H	C15H18O5	-8.192253631	2066.915126	2312.153672	2068.4643	2448.275475	2022.628556	2271.634087	2276.83538	2287.121007	2194.417519
4.16_405.0417m/z	405.041667	4.15895	neg	4,4',6,6'-Tetranitro-2,2'-azoxytoluene	71192	Unclassified	Unclassified	Unclassified			37.1	0	M-H	C14H10N6O9	-4.882378559	2400.9566	2078.14752	2038.252534	2170.832636	1564.3359	1813.393878	3868.104185	2561.693301	2676.189648
4.18_401.1072m/z	401.1071529	4.181333333	neg	Gentiopicrin	41154	Lipids and lipid-like molecules	Prenol lipids	Isoprenoids		C09782	37.1	0	M+FA-H	C16H20O9	-5.004663578	454.6970042	696.1537087	504.1277801	713.3542382	824.9752722	1048.10292	865.2691729	763.9528613	711.3310434
4.21_327.0713m/z	327.0712866	4.212116667	neg	BERGENIN	43945	Unclassified	Unclassified	Unclassified			37.1	0	M-H	C14H16O9	-2.648980846	486.6585353	623.740219	602.9160296	553.9180632	437.3583139	624.7895576	523.5701216	454.5144691	199.0126676
4.21_497.1302m/z	497.1301517	4.212116667	neg	Aspalathin	52331	Phenylpropanoids and polyketides	Flavonoids	Flavones	*		37.1	0	M+FA-H	C21H24O11	0.192976102	1222.865449	1674.676773	1951.78533	207.1746545	234.3539348	163.007948	1517.46212	1492.650172	1580.241203
4.34_459.0524m/z	459.0523864	4.344733333	neg	4-Methoxyglucobrassicin	HMDB0303668	Unclassified	Unclassified	Unclassified			37.1	3.58	M-H2O-H	C17H22N2O10S2	-2.843490526	7854.119701	8382.756665	8067.328959	2717.676013	2488.946734	2352.038594	6582.786814	6716.276364	7141.564202
4.39_333.0968m/z	333.0968317	4.3893	neg	1-(3-methoxy-4-hydroxy)-phenyl-6,7-dihydroxy-isochroman	86173	Benzenoids	Phenols	Methoxyphenols	*		37.1	0	M+FA-H	C16H16O5	-3.973475317	5370.419925	5308.189601	4655.446594	4415.369752	4892.689946	4136.335432	6248.450618	5823.805977	6439.440437
4.41_661.1743m/z	661.1743269	4.41115	neg	Vitexin 3''',4'''-Di-O-acetyl 2''-O-rhamnoside	LMPK12110281	Lipids and lipid-like molecules	Polyketides	Flavonoids			37.1	0	M-H	C31H34O16	-4.653740351	2410.571989	2276.655865	2450.127419	1275.336042	488.086122	1274.769862	2752.757273	1276.266431	2035.366555
4.57_137.0608m/z	137.0608227	4.5668	neg	3-Methoxybenzyl alcohol	65961	Unclassified	Unclassified	Unclassified			37.1	0	M-H	C8H10O2	0.141501841	103.4271155	75.73603717	109.2417111	89.40878541	119.9610893	67.68739822	94.12167245	71.98683936	95.23351592
4.57_439.1225m/z	439.122521	4.5668	neg	6-Hydroxymusizin 8-O-b-D-glucopyranoside	87213	Organic oxygen compounds	Organooxygen compounds	Carbohydrates and carbohydrate conjugates	*		37.1	0	M+FA-H	C19H22O9	-5.23722854	4468.238959	4941.26051	4566.078673	2252.819339	2202.162544	1962.71962	3883.905639	3897.880244	4154.05688
4.59_609.0392m/z	609.039208	4.589283333	neg	UDP-3-ketoglucose	63727	Unclassified	Unclassified	Unclassified			37.1	0	M+FA-H	C15H22N2O17P2	2.89801433	1758.378099	1778.140437	1230.029008	1713.221637	1842.684346	1817.453518	1719.897883	1343.964547	1371.409276
4.73_315.1067m/z	315.1067459	4.725416667	neg	Laurencione monoacetate	65392	Unclassified	Unclassified	Unclassified			37.1	0	2M-H	C7H10O4	-5.679077962	1010.045205	841.8548273	813.2307802	676.9351255	877.0299298	988.0271742	514.5010327	605.5295369	544.2992131
4.73_583.0730m/z	583.0730362	4.725416667	neg	5-Carboxypyranocyanidin 3-O-(6''-O-malonyl-beta-glucopyranoside)	47200	Unclassified	Unclassified	Unclassified			37.1	0	M-H2O-H	C27H22O16	0.153978344	1082.115396	881.4298515	1103.102275	3913.067262	3395.412826	3439.726095	1813.208737	1644.904936	1747.482475
4.79_277.0330m/z	277.0330209	4.792316667	neg	2-O-Feruloyltartronic acid	88856	Phenylpropanoids and polyketides	Cinnamic acids and derivatives	Hydroxycinnamic acids and derivatives			37.1	0	M-H2O-H	C13H12O8	-7.955847181	33194.39752	31937.97719	30086.44859	32332.90445	36380.25038	35713.59837	40091.15493	33469.6349	36009.95185
4.79_625.2481m/z	625.2481	4.792316667	neg	PC(O-1:0/2:0)[U]	40035	Unclassified	Unclassified	Unclassified			37.1	0	2M-H	C11H24NO7P	-4.313015251	2564.94756	2582.174827	2717.944726	3431.628589	2963.128513	3639.886318	2459.928718	2406.498453	2554.157221
4.81_189.0030m/z	189.0029598	4.814166667	neg	HYDROXYCITRIC ACID	84968	Organic acids and derivatives	Carboxylic acids and derivatives	Tricarboxylic acids and derivatives	*		37.1	0	M-H2O-H	C6H8O8	-5.366073746	8203.301031	9245.952334	9032.512224	3608.162182	3499.345864	3719.161382	4316.00742	4978.923608	4739.549919
4.86_537.1183m/z	537.118346	4.858383333	neg	Piretanide glucuronide	1980	Unclassified	Unclassified	Unclassified			37.1	0	M-H	C23H26N2O11S	-0.201202637	9965.780697	10931.39524	11707.12981	12985.86564	12881.881	13082.51148	14124.872	14681.46996	13219.8474
4.99_373.0498m/z	373.0497634	4.99225	neg	Orange I	HMDB0032886	Benzenoids	Naphthalenes	Naphthols and derivatives			37.1	0	M+FA-H	C16H12N2O4S	-0.662625019	581.4999693	603.0850065	534.5477905	911.5183621	755.5419013	772.4835066	752.284531	813.0912119	362.003468
5.16_363.0185m/z	363.0184595	5.155116667	neg	6-Thioinosine-5'-monophosphate	HMDB0060416	Nucleosides, nucleotides, and analogues	Purine nucleotides	Purine ribonucleotides	**	C04646	37.1	0	M-H	C10H13N4O7PS	4.064235739	134.7770201	152.9193804	353.5007258	956.8039767	1061.463269	1194.714993	0.253282097	0.370444565	0.873119994
5.18_289.0754m/z	289.0753672	5.1777	neg	Mecarbinzid	72702	Unclassified	Unclassified	Unclassified			37.1	0	M-H2O-H	C13H16N4O3S	-3.580220782	324.6860575	484.0942079	532.2595046	521.0646921	600.4247862	448.2981031	339.3464944	384.0736427	492.0095443
5.18_499.1656m/z	499.1656092	5.1777	neg	2-(5'-Methylthio)pentylmalic acid	64501	Unclassified	Unclassified	Unclassified			37.1	0	2M-H	C10H18O5S	-4.205869352	1145.657069	1833.803246	1065.254448	689.7984337	729.2413892	944.0800409	971.092572	380.062778	917.3403733
5.24_464.0968m/z	464.0967586	5.244033333	neg	6-Hydroxycyanidin 3-glucoside	LMPK12010425	Unclassified	Unclassified	Unclassified			37.1	0	M-H	C21H21O12+	1.578018767	510.4883509	585.0765991	606.9524626	344.8806757	388.2368331	400.22813	721.3782735	447.7136841	336.4719031
5.24_539.1361m/z	539.1361478	5.244033333	neg	D-Lombricine	63423	Unclassified	Unclassified	Unclassified			37.1	0	2M-H	C6H15N4O6P	-4.475635052	1458.491342	1667.223219	1424.958639	1598.33294	1698.984935	1176.395841	1866.253524	1554.31022	2135.331865
5.29_233.0565m/z	233.0564768	5.28555	neg	5-Hydroxy-N-formylkynurenine	58207	Organic oxygen compounds	Organooxygen compounds	Carbonyl compounds	**	C05648	37.1	0	M-H2O-H	C11H12N2O5	-1.204329794	133.3415221	90.68655324	122.0058073	0.001403199	93.84144139	105.73577	176.2332972	86.62369728	105.7221035
5.29_336.1430m/z	336.1430463	5.28555	neg	(S)-Edulinine	86773	Organoheterocyclic compounds	Quinolines and derivatives	Quinolones and derivatives	*		37.1	0	M+FA-H	C16H21NO4	-7.6070204	10382.00459	7858.944215	7519.813972	4319.609254	5690.536164	6121.02431	7513.085676	5113.832623	7148.75646
5.32_315.1430m/z	315.1429944	5.320233333	neg	10-Hydroxy-8-nor-2-fenchanone glucoside	89442	Lipids and lipid-like molecules	Prenol lipids	Terpene glycosides	*		37.1	0	M-H	C15H24O7	-6.111802417	10460.55471	10984.25812	10894.8421	10800.33053	12537.63924	11669.78928	7769.655559	7354.135609	8224.402901
5.36_401.0808m/z	401.0807826	5.361783333	neg	4'-Phosphopantothenoylcysteine	HMDB0001117	Organic acids and derivatives	Peptidomimetics	Hybrid peptides	**	C04352	37.1	0	M-H	C12H23N2O9PS	4.653405424	4467.163771	5119.851823	5564.756382	4316.292705	4569.214493	3959.803126	3317.98778	3886.058613	4032.749464
5.45_373.1871m/z	373.187112	5.451166667	neg	Granisetron metabolite 3	3147	Unclassified	Unclassified	Unclassified			37.1	0	M+FA-H	C18H24N4O2	-3.098595143	866.7465449	828.775739	908.2795906	2429.582917	2260.864024	2237.494727	658.1766789	721.5464111	843.9651943
5.47_286.1151m/z	286.1150515	5.471616667	neg	tetrahydro-L-Biopterin	64878	Unclassified	Unclassified	Unclassified			37.1	0	M+FA-H	C9H15N5O3	-2.657508551	1350.107235	1215.22377	1088.098739	1505.42308	1373.997726	1505.835804	1210.21341	1132.571398	1321.998021
5.49_421.1511m/z	421.1510983	5.489016667	neg	Carissanol	HMDB0030081	Lignans, neolignans and related compounds	Furanoid lignans	Tetrahydrofuran lignans	*		37.1	0	M+FA-H	C20H24O7	1.840445259	328.9819612	413.6254872	313.3703243	411.6199555	343.5681144	395.2486459	1157.433507	726.1730317	847.8899212
5.58_363.1783m/z	363.1783484	5.584533333	neg	Cohulupone	86701	Organic oxygen compounds	Organooxygen compounds	Carbonyl compounds	*		37.1	0	M+FA-H	C19H26O4	-9.314560174	3984.991318	2830.668052	3316.8257	3666.670518	4057.383859	3740.25889	4672.597859	4555.282012	3822.761963
5.61_365.0678m/z	365.067758	5.610733333	neg	19-Hydroxytetrangulol	63757	Unclassified	Unclassified	Unclassified			37.1	0	M+FA-H	C19H12O5	3.379455321	2792.243035	3005.28972	4019.199034	3779.916702	3667.017216	3520.124371	3144.232458	2845.986341	2851.392265
5.61_377.0857m/z	377.0856511	5.610733333	neg	5,2',5'-Trihydroxy-7,8-dimethoxyflavanone	53123	Unclassified	Unclassified	Unclassified			37.1	0	M+FA-H	C17H16O7	-6.488209086	8084.760076	8206.335562	10177.02239	3904.18197	4104.435183	3515.3526	7173.969327	6335.279122	5990.054061
5.76_300.0854m/z	300.0853688	5.7632	neg	Tetrodotoxin	69159	Unclassified	Unclassified	Unclassified			37.1	0	M-H2O-H	C11H17N3O8	5.156489377	1052.982153	1090.937508	818.7235272	675.4360157	1226.72013	646.4571894	2312.310362	2392.487175	1825.647747
5.83_306.0573m/z	306.0573085	5.825	neg	Aminoparathion	6284	Organic acids and derivatives	Organic thiophosphoric acids and derivatives	Thiophosphoric acid esters	*	C06605	37.1	0	M+FA-H	C10H16NO3PS	0.975504799	1584.937278	1218.189508	1976.740007	1943.470641	1412.583954	1424.650951	921.5178734	1134.599335	1152.527702
5.88_477.1353m/z	477.1353456	5.88025	neg	albendazole (IV) 1H-Benzimidazol-2-amine	797	Unclassified	Unclassified	Unclassified			37.1	0	2M-H	C10H13N3O2S	-6.427565185	3130.003854	2947.150842	3671.10057	3164.475735	3442.751199	2696.46511	2198.978463	2307.684624	2552.639799
5.91_459.1046m/z	459.1045604	5.908066667	neg	Wyeronic acid	93738	Lipids and lipid-like molecules	Fatty Acyls	Fatty acids and conjugates	*		37.1	0	2M-H	C13H10O4	-8.651616309	1481.346586	1727.000709	1638.431721	332.9158404	349.5296345	259.1915144	746.3275122	1023.578586	1360.208885
5.92_375.1816m/z	375.1815615	5.9168	neg	antipyrine	3931	Organoheterocyclic compounds	Azoles	Pyrazoles		C13244	37.1	0	2M-H	C11H12N2O	-2.892309296	1305.445902	1837.812189	1920.491496	1244.372171	1342.309473	1069.336099	981.0778156	1339.5612	1210.579025
6.07_593.1451m/z	593.1450537	6.07	neg	albendazole sulfoxide	795	Organoheterocyclic compounds	Benzimidazoles	Unclassified	*	C02809	37.1	0	2M-H	C12H15N3O4S	-7.277260268	3692.288668	4371.694247	3786.512842	4576.206299	4498.952799	3932.871182	2392.968414	2703.08259	2895.068677
6.16_784.8295m/z	784.8295226	6.161866667	neg	5-Diphosphoinositol pentakisphosphate	HMDB0006229	Organic oxygen compounds	Organooxygen compounds	Alcohols and polyols	**	C11526	37.1	0	M+FA-H	C6H19O27P7	4.890296434	4836.814771	4451.74908	2834.14154	4616.453117	4208.484294	5411.478223	5304.537033	4084.479028	4015.355031
6.25_617.2197m/z	617.2196568	6.248083333	neg	5alpha-Ethoxy-6beta-hydroxy-5,6-dihydrophysalin B	86386	Lipids and lipid-like molecules	Steroids and steroid derivatives	Physalins and derivatives	*		37.1	0	M+FA-H	C30H36O11	-7.528555342	37183.04067	27826.08283	35804.58099	40632.12496	31498.55086	37860.52222	32235.43317	33631.61629	31650.23255
6.30_457.1699m/z	457.1699096	6.302033333	neg	Kanokoside A	71684	Organic acids and derivatives	Carboxylic acids and derivatives	Amino acids, peptides, and analogues	*	C09993	37.1	0	M-H2O-H	C21H32O12	-3.41403583	8053.544705	7717.363957	8681.074939	10879.5023	10221.4886	10720.00437	6508.504144	7050.470361	7730.703549
6.39_571.1800m/z	571.1799639	6.386066667	neg	Physalin A	HMDB0033773	Lipids and lipid-like molecules	Prenol lipids	Triterpenoids	*		37.1	0	M+FA-H	C28H30O10	-4.059580302	6883.136416	7597.673664	8519.389727	2013.929089	2578.358625	2176.777429	7355.216724	6745.262244	6748.59679
6.43_586.0909m/z	586.0909014	6.433933333	neg	CMP-3-deoxy-D-manno-octulosonate	HMDB0304292	Unclassified	Unclassified	Unclassified			37.1	0	M+FA-H	C17H24N3O15P-2	-3.336205026	1093.945757	1266.585574	1203.538142	1902.206025	2114.095614	2120.376106	1507.278922	633.0783234	1288.478084
6.56_425.1820m/z	425.1819902	6.55905	neg	Chaparrin	67175	Unclassified	Unclassified	Unclassified			37.1	0	M+FA-H	C20H28O7	0.747348976	4698.822786	4242.780492	5020.870033	3270.004968	2872.327986	2877.419919	5033.928781	5408.745675	4931.482505
6.68_683.1209m/z	683.1209206	6.680966667	neg	Oxamflatin	64801	Unclassified	Unclassified	Unclassified			37.1	0	2M-H	C17H14N2O4S	-9.733288188	5059.775931	7405.591746	6997.024171	8262.704461	6894.661057	6507.783407	3994.504851	5212.390646	5855.784547
6.69_547.0063m/z	547.0062723	6.69475	neg	3-Dehydro-L-gulonate 6-phosphate	63132	Unclassified	Unclassified	Unclassified			37.1	0	2M-H	C6H11O10P	-8.061032896	2741.050689	4017.874719	2741.902601	3636.294471	3371.079039	2941.288809	115.6799951	298.9942055	91.86089642
6.76_275.0903m/z	275.0902825	6.759433333	neg	Aspartyl-Proline	85675	Organic acids and derivatives	Carboxylic acids and derivatives	Amino acids, peptides, and analogues	*		37.1	0	M+FA-H	C9H14N2O5	7.858200418	1379.207153	1518.959205	1537.951761	1568.36694	1224.473725	1206.945524	1415.334146	1555.576504	1519.186333
6.79_411.1658m/z	411.1657769	6.7874	neg	Pyrantel	2191	Unclassified	Unclassified	Unclassified			37.1	0	2M-H	C11H14N2S	-6.030466968	3113.489801	2761.699899	3250.795114	3102.383094	2566.997638	2799.292128	1674.865383	1915.833435	1738.587868
6.83_249.0745m/z	249.0745243	6.832183333	neg	Aspartyl-Alanine	85661	Organic acids and derivatives	Carboxylic acids and derivatives	Amino acids, peptides, and analogues	*		37.1	0	M+FA-H	C7H12N2O5	8.32991825	1908.411461	1727.037459	1855.43092	1046.775731	809.056108	911.8668627	1763.966726	1702.303046	1367.262587
6.90_571.1025m/z	571.1025092	6.896766667	neg	Phosphoribosylglycinamide	376	Unclassified	Unclassified	Unclassified			37.1	0	2M-H	C7H15N2O8P	-5.97532526	6486.160917	7495.185047	6611.80789	9890.420133	9748.02206	10116.94282	5285.566537	6402.37559	6467.302704
6.94_257.1760m/z	257.1759865	6.939266667	neg	Ethyl 10-undecenoate	HMDB0034286	Lipids and lipid-like molecules	Fatty Acyls	Fatty acid esters	*		37.1	0	M+FA-H	C13H24O2	0.723952955	635.042556	673.8417883	736.2366401	625.4316165	641.9325647	635.1185844	457.1071464	693.6189525	503.6269872
7.09_369.1455m/z	369.145484	7.086816667	neg	p-Hydroxyphenylbutazone	1840	Unclassified	Unclassified	Unclassified			37.1	0	M+FA-H	C19H20N2O3	-0.343724778	2264.555444	2544.725918	2553.275514	3029.536629	4051.353674	2831.613654	1615.045164	2296.69676	1886.362008
7.09_457.2067m/z	457.2066612	7.086816667	neg	AFP 07 (free acid)	96511	Unclassified	Unclassified	Unclassified			37.1	0	M+FA-H	C22H30F2O5	5.647192999	1929.87528	1863.621388	1711.656512	2469.858702	2678.293413	1959.603956	1881.565904	1860.983845	2386.281087
7.10_571.1026m/z	571.1026063	7.097333333	neg	5'-Phosphoribosylglycinamide (GAR)	3411	Unclassified	Unclassified	Unclassified			37.1	0	2M-H	C7H15N2O8P	-5.805477516	12668.62807	14590.61827	12613.13055	15652.85741	17491.98888	16753.33037	10529.23412	10754.81159	10739.15996
7.26_245.0672m/z	245.0671986	7.262266667	neg	3-Hydroxy-4-butanolide	HMDB0034681	Organic oxygen compounds	Organooxygen compounds	Carbohydrates and carbohydrate conjugates			37.1	0	M-H2O-H	C10H16O8	1.977522436	836.353533	960.1112623	989.506905	1134.663871	930.7085341	994.5268108	1037.290016	1041.342609	1055.148698
7.51_753.0808m/z	753.0808168	7.50615	neg	Glucosisymbrin	71621	Unclassified	Unclassified	Unclassified			37.1	0	2M-H	C10H19NO10S2	-2.628904673	4462.664689	5521.270766	5204.690093	4268.178909	4679.171252	4290.370587	3746.397434	4084.201203	4035.366412
7.56_397.2057m/z	397.2056548	7.556516667	neg	Pregnanolone sulfate	HMDB0240590	Lipids and lipid-like molecules	Steroids and steroid derivatives	Sulfated steroids	*		37.1	0	M-H	C21H34O5S	0.592710911	3245.335324	2850.769422	3285.284281	2316.844765	2405.756526	3044.854086	1558.694372	1860.450444	1721.983041
7.60_624.3392m/z	624.3392094	7.600283333	neg	dihydro-β-ergocryptine	4043	Unclassified	Unclassified	Unclassified			37.1	0	M+FA-H	C32H45N5O5	-1.834848624	1565.096081	2114.368378	2404.874653	182.7751077	306.824018	422.0747277	1696.918457	2641.721957	1521.288309
7.77_659.1835m/z	659.1834977	7.770583333	neg	3,5,7,3',4'-Pentahydroxy-6-methoxyflavan 3-O-[glucopyranosyl-(4->1)-alpha-D-x	LMPK12020149	Unclassified	Unclassified	Unclassified			37.1	0	M+FA-H	C27H34O16	0.992947783	1037.667822	1720.693261	1578.474526	2812.445864	2431.935893	2590.853607	1715.757419	1775.773457	1865.722214
7.78_445.2088m/z	445.2088295	7.7828	neg	Linalool 3,6-oxide primeveroside	HMDB0035489	Organic oxygen compounds	Organooxygen compounds	Carbohydrates and carbohydrate conjugates	*		37.1	0	M-H2O-H	C21H36O11	1.957347583	1080.449262	1032.56985	1141.62524	1539.211324	1524.326312	1826.845049	497.3681948	618.1319072	807.6668032
7.83_475.2684m/z	475.2683769	7.8311	neg	17-phenyl-trinor-PGF2alpha isopropyl ester	LMFA03010124	Lipids and lipid-like molecules	Fatty Acyls	Eicosanoids			37.1	0	M+FA-H	C26H38O5	-4.067858556	2180.162159	2279.988195	2758.317248	227.892353	155.285706	150.5503128	631.3193317	1110.82727	945.0238903
7.96_597.2274m/z	597.2273785	7.961816667	neg	2,3,9,10-Tetrahydroxyberbine	69431	Unclassified	Unclassified	Unclassified			37.1	0	2M-H	C17H17NO4	5.246905902	1866.544378	1710.966949	1994.677859	1332.921684	883.1885089	1300.500292	784.0604452	1290.050274	1336.964335
7.97_445.2082m/z	445.208226	7.9723	neg	Linalool 3,7-oxide beta-primeveroside	91645	Organic oxygen compounds	Organooxygen compounds	Carbohydrates and carbohydrate conjugates	*		37.1	0	M-H2O-H	C21H36O11	0.657421481	1618.623225	1401.356293	1263.431346	2825.283839	1875.160601	2555.645003	889.1960476	1438.823096	1016.388062
8.29_579.2403m/z	579.2403371	8.294766667	neg	N2-(3-Hydroxysuccinoyl)arginine	HMDB0032765	Organic acids and derivatives	Carboxylic acids and derivatives	Amino acids, peptides, and analogues			37.1	0	2M-H	C10H18N4O6	4.041254695	17107.11803	19210.36513	21648.05367	17911.64566	16729.53008	17173.3448	14607.22354	18495.177	18426.41439
8.34_427.1588m/z	427.1587983	8.338466667	neg	1-(alpha-Methyl-4-(2-methylpropyl)benzeneacetate)-beta-D-Glucopyranuronic a	61662	Organic oxygen compounds	Organooxygen compounds	Carbohydrates and carbohydrate conjugates	*		37.1	0	M+FA-H	C19H26O8	-5.684330058	7484.900241	8359.791993	8557.791377	9846.565275	9107.90307	9769.929021	6012.977117	6721.331923	6276.499961
8.38_383.1715m/z	383.1715205	8.383816667	neg	Hydroxycotinine	HMDB0001390	Organoheterocyclic compounds	Pyridines and derivatives	Pyrrolidinylpyridines	*		37.1	0	2M-H	C10H12N2O2	-2.494459904	274.2359914	482.7814213	430.6110053	174.6132249	426.8576888	474.3810627	109.2915709	142.2645626	148.9931115
8.61_579.2403m/z	579.2403322	8.606866667	neg	N2-(3-Carboxy-2-hydroxy-1-oxopropyl)arginine	93983	Organic acids and derivatives	Carboxylic acids and derivatives	Amino acids, peptides, and analogues			37.1	0	2M-H	C10H18N4O6	4.032759009	17360.78352	17620.16724	18913.77834	22456.8647	20616.70051	21247.75599	18745.03085	20722.39185	19216.3923
8.86_667.1580m/z	667.158	8.860733333	neg	19-Hydroxy-8-O-methyltetrangulol	63758	Unclassified	Unclassified	Unclassified			37.1	0	2M-H	C20H14O5	-4.445921484	4268.309735	5816.250808	4435.72638	2117.480442	1031.004621	1149.285852	1985.017038	2817.442251	3031.076356
9.02_492.2586m/z	492.2586303	9.01635	neg	1-Pyrrolidinepropanol, a-(4-hydroxycyclohexyl)-a-phenyl-, cis- glucuronide	2123	Unclassified	Unclassified	Unclassified			37.1	0	M-H	C26H39NO8	-3.366332506	4419.41642	7817.921451	6488.654709	920.199875	706.6207401	450.6337661	1815.071197	2093.031943	2338.17294
9.08_385.1872m/z	385.1872376	9.077466667	neg	11-Dehydro-2,3-dinor-txb2	HMDB0244346	Lipids and lipid-like molecules	Fatty Acyls	Eicosanoids			37.1	0	M+FA-H	C18H28O6	1.311431265	1237.4378	1209.940379	1437.521662	2720.106021	2202.258739	2588.968548	1146.835488	1156.290969	1016.5875
9.24_465.1746m/z	465.1745725	9.2377	neg	Castaneiolide	HMDB0034876	Lipids and lipid-like molecules	Fatty Acyls	Fatty acid esters	*		37.1	0	M+FA-H	C22H28O8	-4.874536987	4162.968719	4297.584783	5107.079097	7537.197123	8221.885528	7253.549499	2521.899857	3289.26429	3138.083786
9.32_351.1346m/z	351.1346237	9.321783333	neg	3-Oxo-14,15-dehydrorhazinilam	93706	Benzenoids	Unclassified	Unclassified			37.1	0	M+FA-H	C19H18N2O2	-1.329357241	1798.379346	1768.23069	1061.445051	1540.604546	2224.470055	1767.463855	1203.809467	1430.871515	1871.057909
9.84_286.0516m/z	286.0515634	9.835916667	neg	Atherospermidine	67620	Unclassified	Unclassified	Unclassified			37.1	0	M-H2O-H	C18H11NO4	1.955809857	4989.527047	3741.867088	3352.527647	1643.028561	1669.931388	1310.317451	2919.633067	2142.591	1843.076783
0.76_275.1252m/z	275.1252017	0.757316667	neg	Saccharopine	383	Organic acids and derivatives	Carboxylic acids and derivatives	Amino acids, peptides, and analogues	**	C00449	37	0	M-H	C11H20N2O6	1.237528174	356.3133164	573.3508504	606.7785038	412.3438772	400.9911824	451.1005087	313.157849	310.4925971	361.1977914

0.78_247.0404m/z	247.0404279	0.778533333	neg	Phenanthrene-4,5-dicarboxylate	72096	Unclassified	Unclassified	Unclassified			37	0	M-H2O-H	C16H10O4	1.353784041	9428.094568	10068.74262	12047.1136	6195.596018	8058.248342	7008.497437	8913.216489	10920.23761	12814.23057
0.78_377.0858m/z	377.0857594	0.778533333	neg	5,7,4'-Trihydroxy-8,3'-dimethoxyflavanone	53149	Unclassified	Unclassified	Unclassified			37	0	M+FA-H	C17H16O7	-6.161846724	189887.0465	210688.2753	228440.1158	64293.84771	63423.55236	64723.17724	175417.6561	149956.1187	152970.8289
0.80_307.0345m/z	307.0345018	0.800366667	neg	dUMP	HMDB0001409	Nucleosides, nucleotides, and analogues	Pyrimidine nucleotides	Pyrimidine deoxyribonucleotides	**	C00365	37	0	M-H	C9H13N2O8P	2.682083629	1944.992957	3423.066546	5161.989526	8057.119201	7062.364694	7846.819468	3175.884026	4116.709373	3654.584585
0.82_193.0135m/z	193.0134682	0.819566667	neg	2-Hydroxy-6-ketononatrienedioate	63525	Unclassified	Unclassified	Unclassified			37	0	M-H2O-H	C9H8O6	-3.672151018	1630.997286	936.3290536	1322.780652	521.5721602	184.2556329	484.4865612	929.9342612	1160.527527	755.3986718
0.84_293.0687m/z	293.0686836	0.841733333	neg	Coriandrone E	86628	Organoheterocyclic compounds	Benzopyrans	2-benzopyrans	*		37	0	M+FA-H	C13H12O5	8.091332227	67661.96598	62300.10502	65344.38498	51925.01464	62834.6261	51883.53873	69925.00753	76402.97902	75073.07727
0.84_320.0659m/z	320.0659476	0.841733333	neg	Deoxy-5-methylcytidylate	65987	Unclassified	Unclassified	Unclassified			37	0	M-H	C10H16N3O7P	1.985653727	7923.564284	7778.81143	8633.589732	8774.70573	7378.809939	8753.242003	8791.553573	10248.09425	8054.55914
0.84_338.1015n	337.0948835	0.841733333	neg	4-p-Coumaroylquinic acid	HMDB0301710	Unclassified	Unclassified	Unclassified			37	0	M-H2O-H, M-H	C16H18O8	3.800789006	20436.40231	18252.0085	23869.97133	19846.67268	24424.29482	19370.91443	17787.92414	21254.67651	23705.64905
0.86_203.0507m/z	203.0506671	0.86335	neg	Isoflavone skeleton	47523	Unclassified	Unclassified	Unclassified			37	0	M-H2O-H	C15H10O2	1.930461343	1690.806884	1524.962391	2008.651278	923.9955313	793.6098831	659.4691888	1278.821366	1420.675541	1147.249308
0.93_287.0636m/z	287.0635978	0.925866667	neg	2-Deoxystreptamine 4-phosphate	66090	Unclassified	Unclassified	Unclassified			37	0	M+FA-H	C6H15N2O6P	-5.692193618	2557.872395	2452.443275	3233.526089	3021.7222	1643.817534	3372.421628	2469.995855	2119.929077	1077.641497
1.14_261.0594m/z	261.059395	1.1382	neg	2-Thiopheneacetic acid, 5-(hydroxyphenylmethyl)-α-methyl-	2846	Unclassified	Unclassified	Unclassified			37	0	M-H	C14H14O3S	1.168115348	1800.766297	629.4122551	553.4669931	3931.393407	2783.835962	4672.629847	599.5738061	2036.284901	1455.732622
1.14_365.1202m/z	365.1202102	1.1382	neg	3-Epihydroxy-2'-deoxymugineic acid	70950	Unclassified	Unclassified	Unclassified			37	0	M+FA-H	C12H20N2O8	0.130216276	362.3893969	140.1724106	312.8244218	708.521987	415.2395803	973.2464587	173.9779768	454.6895561	255.7956999
1.20_245.0393m/z	245.0392504	1.202333333	neg	Mono-N-hydroxydapsone	1931	Unclassified	Unclassified	Unclassified			37	0	M-H2O-H	C12H12N2O3S	0.864431433	1556.808336	1582.194285	1795.404747	2268.818119	2120.491991	2564.523591	1962.312159	2037.870537	2130.21865
1.27_401.0510m/z	401.0510064	1.26875	neg	Xerocomic acid	86285	Benzenoids	Phenols	Benzenediols	*		37	0	M+FA-H	C18H12O8	-1.162278237	1335.467784	1419.748897	1544.73913	1782.414532	1663.397979	1780.943145	1171.784034	2001.800302	1644.097173
1.36_285.1459m/z	285.1458863	1.358266667	neg	1-Isothiocyanatohexane	93123	Organosulfur compounds	Isothiocyanates	Unclassified			37	0	2M-H	C7H13NS	-2.02039796	206.2629634	208.7393287	317.0851051	256.0074218	219.8340216	317.2953526	239.5890152	203.2927265	265.6535374
1.46_245.9964m/z	245.9964352	1.459966667	neg	N-Phosphotaurocyamine	63574	Unclassified	Unclassified	Unclassified			37	0	M-H	C3H10N3O6PS	3.720555544	269.9863658	393.0653219	345.9274074	0.001403199	0.001403199	0.001403199	515.6795079	397.5948296	439.0244763
1.52_253.0809m/z	253.0809118	1.515416667	neg	p-Hydroxyfelbamate	2656	Benzenoids	Phenols	1-hydroxy-2-unsubstituted benzenoids	**	C16584	37	0	M-H	C11H14N2O5	-8.199077992	586.4280593	465.7798878	568.3532577	824.9435536	842.2246834	790.138763	428.0913153	551.6736527	544.5398447
1.53_283.0914m/z	283.0913694	1.534483333	neg	Bicozamycin	68945	Unclassified	Unclassified	Unclassified			37	0	M-H2O-H	C12H18N2O7	-7.25015332	5446.219336	4641.236077	4425.764597	4294.269459	3907.492447	4182.423385	4612.801643	5101.829658	4979.558367
1.84_220.0452m/z	220.0451759	1.841666667	neg	D-N-(Carboxyacetyl)alanine	93757	Organic acids and derivatives	Carboxylic acids and derivatives	Amino acids, peptides, and analogues			37	0	M+FA-H	C6H9NO5	-6.280198813	2019.137396	1958.849628	2456.666708	1628.190204	1686.966934	1554.339344	2780.713294	2463.339996	2524.149104
1.95_249.0074m/z	249.0073843	1.945716667	neg	Hydroxymethoxyphenylcarboxylic acid-O-sulphate	HMDB0060000	Benzenoids	Phenols	Methoxyphenols	*		37	0	M-H	C8H10O7S	-0.251618994	887.5816153	882.2229024	479.2010844	313.7970964	251.6411816	354.1688517	330.059984	397.9547501	287.3712319
11.42_1145.1613m/z	1145.161306	11.42398333	neg	GDP-3,6-dideoxy-D-galactose	66293	Unclassified	Unclassified	Unclassified			37	0	2M-H	C16H25N5O14P2	-5.291992341	696.8739116	2208.232655	1733.803185	2894.172736	2133.160696	2497.874051	3526.451767	2840.33291	1322.786286
11.58_249.1497m/z	249.1497433	11.58276667	neg	2-Benzylidene-1-heptanol	HMDB0036170	Phenylpropanoids and polyketides	Cinnamyl alcohols	Unclassified			37	0	M+FA-H	C14H20O	0.613097101	4287.207069	3566.241038	4578.068637	4417.685379	4656.146383	4275.601569	4981.825743	3901.608303	5310.984271
12.44_761.5342m/z	761.5342121	12.4444	neg	PA(15:0/22:1(13Z))	HMDB0114825	Lipids and lipid-like molecules	Glycerophospholipids	Glycerophosphates			37	0	M+FA-H	C40H77O8P	0.562729433	348361.159	347861.302	332096.3157	288211.4134	315889.2609	272237.2863	378797.6554	333878.9526	334547.5226
14.71_197.0203m/z	197.0202981	14.7064	neg	Allyl isothiocyanate	HMDB0005843	Organosulfur compounds	Isothiocyanates	Unclassified	**	C19317	37	0	2M-H	C4H5NS	-4.877271283	831.7937524	572.0458363	628.6218682	530.0885626	502.0142681	480.65529	684.6617526	574.8953562	476.1994007
2.00_425.0231m/z	425.0230567	2.004766667	neg	5-Hydroxyriluzole glucuronide	2325	Unclassified	Unclassified	Unclassified			37	0	M-H	C14H13F3N2O8S	-9.712709968	1913.547905	1728.444333	2188.055121	1763.383051	1106.197245	1603.519903	1113.925314	2246.554125	1685.153163
2.07_398.0116m/z	398.0116283	2.070366667	neg	5'-Hydroxytenoxicam	HMDB0014098	Organoheterocyclic compounds	Thienothiazines	Unclassified	*		37	0	M+FA-H	C13H11N3O5S2	-1.662840656	1107.725263	793.1858866	916.1268789	1274.062505	1030.971224	1114.070914	1628.427314	1365.80549	1316.013766
2.09_317.0661m/z	317.0661115	2.092433333	neg	Naringenin	HMDB0002670	Phenylpropanoids and polyketides	Flavonoids	Flavans	***	C00509	37	0	M+FA-H	C15H12O5	-2.076195152	1502.611826	2019.926745	1890.832024	2594.628816	3043.253153	3042.591587	1997.264212	2051.844738	1851.688078
2.19_343.0527m/z	343.0526983	2.194566667	neg	Monomethyl phenylphosphonate	87986	Benzenoids	Benzene and substituted derivatives	Unclassified	*		37	0	2M-H	C7H9O3P	6.141791781	917.3797768	823.7838974	863.8030435	1263.397282	1293.193546	1354.369324	736.853191	1175.213623	1038.413028
2.28_232.0592m/z	232.0592226	2.2828	neg	N-Carboxyacetyl-D-phenylalanine	93703	Organic acids and derivatives	Carboxylic acids and derivatives	Amino acids, peptides, and analogues			37	0	M-H2O-H	C12H13NO5	-9.195645325	17091.6342	17654.16801	16619.44338	26435.18244	25346.99519	25502.38294	21842.16302	23852.33926	22651.50676
2.78_293.0758m/z	293.0758304	2.776216667	neg	5-Hydroxyindoleacetylglycine	58225	Organic acids and derivatives	Carboxylic acids and derivatives	Amino acids, peptides, and analogues	**	C05832	37	0	M+FA-H	C12H12N2O4	-8.38170324	10219.5298	10120.69909	10663.65433	13128.13378	12928.30335	13106.21991	10665.7749	12350.48548	11485.93673
2.92_236.0178m/z	236.0177914	2.9156	neg	5-(3'-Carboxy-3'-oxopropyl)-4,6-dihydroxypicolinate	63551	Unclassified	Unclassified	Unclassified			37	0	M-H2O-H	C10H9NO7	-8.897262771	18290.94579	18080.12541	18836.4236	13943.07082	13329.86113	13904.73652	12542.95667	13893.67186	12527.47832
3.11_357.0830m/z	357.0830225	3.112733333	neg	N1-(5-Phospho-a-D-ribosyl)-5,6-dimethylbenzimidazole	HMDB0003882	Nucleosides, nucleotides, and analogues	Benzimidazole ribonucleosides and ribonucleotides	Unclassified	***	C04778	37	0	M-H	C14H19N2O7P	-7.508291097	5328.558179	5674.031786	5963.267305	5460.966733	4655.952267	4548.412688	5072.314956	5717.846143	5413.097834
3.44_326.0615n	371.0598173	3.440016667	neg	Fertaric acid	86082	Phenylpropanoids and polyketides	Cinnamic acids and derivatives	Hydroxycinnamic acids and derivatives			37	0	M-H, M+FA-H	C14H14O9	-6.961132479	20753.73035	24106.058	21632.2062	10955.38887	10692.62418	10379.49561	12429.08584	12326.95436	11772.60321
3.73_275.1139m/z	275.1138788	3.730516667	neg	Ethyl (S)-3-hydroxybutyrate glucoside	87869	Lipids and lipid-like molecules	Fatty Acyls	Fatty acyl glycosides	*		37	0	M-H2O-H	C12H22O8	0.857547372	320.9889266	415.9672813	359.8762612	1045.9832	910.4967208	652.8239266	365.9716856	499.7860839	324.0635442
3.78_277.1295m/z	277.129507	3.779133333	neg	N-Benzoyl-D-arginine	65879	Unclassified	Unclassified	Unclassified			37	0	M-H	C13H18N4O3	-3.980203689	479.9759353	441.3267185	439.3404456	643.8900658	764.7965149	932.4350587	464.0310435	464.687534	428.4715264
3.85_283.0104m/z	283.0104279	3.84605	neg	4-(1,2-dithiin-3-yl)-2-(prop-1-en-2-yloxy)but-3-yn-1-yl acetate	LMFA12000363	Lipids and lipid-like molecules	Fatty Acyls	Oxygenated hydrocarbons			37	0	M-H	C12H12O4S2	0.012333275	6018.239408	5949.448778	6753.754748	5461.693041	6923.353639	6790.051784	7664.260778	6434.980726	7449.001608
3.85_326.0319m/z	326.0319416	3.84605	neg	2',3'-Cyclic GMP	3487	Lipids and lipid-like molecules	Sphingolipids	Glycosphingolipids	*		37	0	M-H2O-H	C10H12N5O7P	6.805839682	63.50014312	83.76070919	40.25193806	1926.132251	1907.870367	2039.374736	125.0604253	103.9801216	96.28189773
3.89_337.0906m/z	337.0906095	3.8906	neg	Phosphodimethylethanolamine	63348	Organic acids and derivatives	Organic phosphoric acids and derivatives	Phosphate esters	**	C13482	37	0	2M-H	C4H12NO4P	-8.586341987	414.1076312	356.8901469	410.3146024	894.5355549	690.3434767	796.739671	621.9096882	715.5475388	663.0652968
4.00_313.0752n	294.0573707	4.002233333	neg	Albendazole-beta-hydroxysulphone	71270	Unclassified	Unclassified	Unclassified			37	0	M-H2O-H, M-H	C12H15N3O5S	6.293275945	4936.226978	5367.359968	5797.913229	7457.265812	7129.795792	7342.488675	6604.039966	7192.870338	6841.573497
4.14_233.0198m/z	233.0197745	4.136366667	neg	Orellanine	73453	Unclassified	Unclassified	Unclassified			37	0	M-H2O-H	C10H8N2O6	-2.461296329	109.5231935	216.7439014	208.6646595	353.4298067	295.5710673	245.1949723	200.1073959	131.9830017	125.2393702
4.32_232.0950n	213.0771331	4.3224	neg	TODRALAZINE	43492	Unclassified	Unclassified	Unclassified			37	0	M-H2O-H, M-H	C11H12N4O2	-4.529950253	2143.642071	2833.225432	2467.03431	3350.275454	3120.74547	2834.70048	1891.38715	2173.945012	2209.854705
4.41_733.1720m/z	733.1720234	4.41115	neg	Xanthurenate-8-O-beta-D-glucoside	HMDB0013118	Lipids and lipid-like molecules	Glycerophospholipids	Glycerophosphocholines	*		37	0	2M-H	C16H17NO9	-1.855634791	9155.697538	8939.748345	7540.760316	1041.643155	1615.983096	1638.923095	4446.633262	6333.862305	4998.091294
4.45_349.0028m/z	349.002776	4.454616667	neg	Genistein 7-sulfate	HMDB0240522	Phenylpropanoids and polyketides	Isoflavonoids	Isoflav-2-enes			37	0	M-H	C15H10O8S	1.183396482	70.25243993	43.67014535	39.7639614	3130.28368	2995.575476	3139.721082	0.001403199	0.001403199	0.001403199
4.52_263.0232m/z	263.0232295	4.521733333	neg	4,6-Dihydroxysulfadiazine	2440	Unclassified	Unclassified	Unclassified			37	0	M-H2O-H	C10H10N4O4S	-4.272947172	2680.595594	2350.283844	2468.929231	1446.082198	1349.734354	1447.235554	2027.799968	2525.432661	2023.249368
4.54_480.1550m/z	480.1549851	4.54425	neg	Penilloate	1226	Unclassified	Unclassified	Unclassified			37	0	M+FA-H	C19H25N5O5S	-1.971096603	831.2773069	667.7363891	812.100106	1378.769692	1067.672782	1379.305023	983.8124744	1001.274897	710.0715097
4.57_219.0276m/z	219.0276264	4.5668	neg	Benzoylmalic acid	94064	Benzenoids	Benzene and substituted derivatives	Benzoic acids and derivatives			37	0	M-H2O-H	C11H10O6	-9.538211023	7431.801952	7911.518161	7866.885403	3486.68205	3635.728228	3389.381081	5222.603706	5712.604478	5421.888231
4.59_327.0600m/z	327.060018	4.589283333	neg	Niflumic Acid	HMDB0015573	Benzenoids	Benzene and substituted derivatives	Trifluoromethylbenzenes		C13698	37	0	M+FA-H	C13H9F3N2O2	0.719587417	488.6599661	685.3238823	628.6147932	725.6615116	633.8458302	382.2769209	529.9994635	679.9881653	748.1837676
4.75_301.0385m/z	301.038514	4.747466667	neg	{2-methoxy-4-[(5-oxooxolan-2-yl)methyl]phenyl}oxidanesulfonic acid	HMDB0127769	Organic acids and derivatives	Organic sulfuric acids and derivatives	Arylsulfates	*		37	0	M-H	C12H14O7S	-0.772477975	2324.774473	2249.687381	2255.200081	1459.530829	1318.850646	1274.775674	2352.296024	2664.541408	2347.253255
4.79_295.1300m/z	295.1300277	4.792316667	neg	3-Hydroxymelatonin	HMDB0060742	Organoheterocyclic compounds	Indoles and derivatives	Indolines	*		37	0	M+FA-H	C13H18N2O3	0.329327006	1782.475845	2242.679208	2400.695114	1761.836526	1644.165048	1561.314703	1719.818828	1877.987302	1933.320217
4.86_256.1193m/z	256.1193489	4.858383333	neg	O-Glutarylcarnitine	HMDB0240779	Lipids and lipid-like molecules	Fatty Acyls	Fatty esters			37	0	M-H2O-H	C12H21NO6	1.099977262	306.4277225	353.7483961	398.6274732	731.5274069	949.4933001	858.5510132	509.243959	535.0573046	489.1952257
4.86_441.1767m/z	441.1767002	4.858383333	neg	Pyrimidodiazepine	63638	Unclassified	Unclassified	Unclassified			37	0	2M-H	C9H11N5O2	3.227993989	1153.871301	1586.172007	1277.031187	812.898721	586.2762064	952.9689224	1238.420715	1469.620589	1113.253821
5.06_308.0732m/z	308.073235	5.05975	neg	Glymidine	3133	Benzenoids	Benzene and substituted derivatives	Benzenesulfonamides			37	0	M-H	C13H15N3O4S	7.067494565	378.5468319	296.5632847	621.7762086	689.4500048	386.2576353	828.4743986	391.8398257	334.2599255	443.7782593
5.06_348.1486m/z	348.148555	5.05975	neg	Isatidine	68253	Unclassified	Unclassified	Unclassified			37	0	M-H2O-H	C18H25NO7	8.971319897	693.2622374	942.1014076	942.2364153	2155.253167	1729.629439	1646.378012	996.8535062	1050.686796	1034.458918
5.08_372.0680m/z	372.0679531	5.080566667	neg	O-Desmethyloxotolrestat	2883	Unclassified	Unclassified	Unclassified			37	0	M+FA-H	C15H12F3NO4	-6.397155069	1328.93748	1403.559338	1345.023123	1288.552607	1139.549078	1228.581321	2083.356852	1486.861317	1604.401489
5.16_216.9979m/z	216.9978716	5.155116667	neg	TETROQUINONE	44110	Unclassified	Unclassified	Unclassified			37	0	M+FA-H	C6H4O6	-6.506412513	2428.217788	2820.312307	2247.401349	0.001403199	0.001403199	0.001403199	1935.632745	2247.719836	2169.91413
5.16_475.2186m/z	475.2185786	5.155116667	neg	SU 5416	45544	Unclassified	Unclassified	Unclassified			37	0	2M-H	C15H14N2O	9.719933255	1516.982278	1815.975462	1296.728259	1465.098981	1194.75231	1220.46086	1792.257221	1578.079581	1871.15171
5.20_219.0696m/z	219.0696345	5.20005	neg	Isopropyl β-D-ThiogalactoPyranoside	44762	Unclassified	Unclassified	Unclassified			37	0	M-H2O-H	C9H18O5S	-0.079941631	194.02542	176.6593292	117.8360393	47.69977171	120.5566558	96.10783186	181.1086252	128.0067923	154.8923237
5.24_640.3341m/z	640.334127	5.244033333	neg	(3a,5b,7a,12a)-24-[(carboxymethyl)amino]-1,12-dihydroxy-24-oxocholan-3-yl-b-l	6693	Unclassified	Unclassified	Unclassified			37	0	M-H	C32H51NO12	0.432431647	1327.105266	869.2276594	1379.065602	608.3273287	292.4594214	504.8975552	2118.379476	2123.970918	2574.921267
5.30_431.1542m/z	431.1542048	5.302883333	neg	6-(3,4-Methylenedioxyphenyl)-3,5-hexadien-2-one	88137	Organoheterocyclic compounds	Benzodioxoles	Unclassified	*		37	0	2M-H	C13H12O3	9.702008816	683.8474676	470.0583344	753.0461566	886.9385205	737.0508693	1212.682326	688.3744612	773.2800685	1339.081105
5.34_330.1378m/z	330.1377621	5.3394	neg	Pretazettine	67052	Unclassified	Unclassified	Unclassified			37	0	M-H	C18H21NO5	9.258093619	1554.9183	1991.595864	2113.646545	2854.773064	2622.890447	2922.156499	1919.808772	1959.018202	2499.477232
5.34_429.1953m/z	429.1952633	5.3394	neg	Ptilosteroid B	LMST02030213	Lipids and lipid-like molecules	Sterol Lipids	Steroids			37	0	M-H	C21H34O7S	0.035602002	8539.605016	9347.134358	10253.6062	13936.96458	14557.52741	13221.70457	8797.861736	11004.76939	11342.87353
5.34_542.1401n	587.1379629	5.3394	neg	Resveratrol 4'-(6-galloylglucoside)	HMDB0034687	Phenylpropanoids and polyketides	Stilbenes	Stilbene glycosides			37	0	M-H, M+FA-H	C27H26O12	-4.24990143	13207.45613	15473.44362	17748.14525	181.5043084	174.7932232	305.3940779	129.4148422	424.0943298	417.7940779
5.36_347.1613m/z	347.1612786	5.361783333	neg	Trichostatin A	46755	Unclassified	Unclassified	Unclassified			37	0	M+FA-H	C17H22N2O3	0.109723193	4929.320989	4773.242571	5245.260325	1546.04438	2204.138185	1883.879668	4674.348366	5094.938805	5499.516363
5.41_267.1223m/z	267.1222871	5.406433333	neg	Hydroxyibuprofen	691	Phenylpropanoids and polyketides	Phenylpropanoic acids	Unclassified	*		37	0	M+FA-H	C13H18O3	-6.798850156	3795.523487	4114.709163	3857.780986	4121.911396	3743.315217	4388.995022	3637.217199	4342.084588	3557.129457
5.41_354.1284m/z	354.1284274	5.406433333	neg	Tyrosyl-Glutamine	85995	Organic acids and derivatives	Carboxylic acids and derivatives	Amino acids, peptides, and analogues	*		37	0	M+FA-H	C14H19N3O5	-7.266095228	6011.002053	6516.243459	6304.468108	11955.33231	11187.50077	10601.98004	8248.38099	9062.589055	6950.157319
5.49_273.0781m/z	273.0780695	5.489016667	neg	Benzylparaben	70024	Unclassified	Unclassified	Unclassified			37	0	M+FA-H	C14H12O3	5.359412807	1153.730745	384.4817706	370.572948	165.9580978	178.9674088	137.789726	558.0414068	78.30866623	273.1041283
5.53_452.1317n	451.1227737	5.525416667	neg	Precarthamin	88079	Phenylpropanoids and polyketides	Flavonoids	Flavonoid glycosides	*		37	0	M-H2O-H, M-H	C21H24O11	-0.413980453	6181.520653	5780.033979	5402.527532	5285.302838	6728.599876	5381.41556	5999.173347	5769.418554	5909.507018
5.65_673.0958m/z	673.0957704	5.650833333	neg	Cyclic guanosine monophosphate-adenosine monophosphate	HMDB0245558	Organic oxygen compounds	Organooxygen compounds	Carbohydrates and carbohydrate conjugates			37	0	M-H	C20H24N10O13P2	4.587930097	4291.770007	5990.406042	6402.522163	1321.525869	1621.186122	1218.29791	4570.825789	3369.743834	4689.602009
5.88_645.2512m/z	645.2512205	5.88025	neg	2',7-Dihydroxy-4'-methoxy-8-prenylflavan 2',7-diglucoside	91542	Phenylpropanoids and polyketides	Flavonoids	Flavonoid glycosides			37	0	M-H2O-H	C33H44O14	-6.088505478	1224.733792	953.6617611	1548.867571	1675.708409	1289.011531	1703.270935	486.2131751	1826.309884	1234.526105
5.89_215.0386m/z	215.0385564	5.894166667	neg	S-(2-Furanylmethyl) propanethioate	HMDB0037732	Organoheterocyclic compounds	Heteroaromatic compounds	Unclassified			37	0	M+FA-H	C8H10O2S	1.193942178	563.0647186	614.2887926	571.2095361	274.5457658	401.0483304	350.0670249	358.9102493	672.0072765	607.0866188
6.04_289.1060m/z	289.1059862	6.040433333	neg	(2S,4S)-Pinnatanine	86268	Organic acids and derivatives	Carboxylic acids and derivatives	Amino acids, peptides, and analogues	*		37	0	M+FA-H	C10H16N2O5	7.62661027	973.5497668	1132.405347	1162.488707	1709.409892	1508.867585	1475.576638	1301.169509	1083.353843	946.1856429
6.04_503.2503m/z	503.2503494	6.040433333	neg	[6]-Gingerdiol 4'-O-beta-D-glucopyranoside	91279	Organic oxygen compounds	Organooxygen compounds	Carbohydrates and carbohydrate conjugates	*		37	0	M+FA-H	C23H38O9	1.230283089	1021.47706	1110.288263	1415.036373	1447.843955	1016.68592	1117.151115	982.3555587	1056.416435	1194.507886
6.06_217.0905m/z	217.0904802	6.056116667	neg	S-(3-Methyl-2-butenyl) 2-methylpropanethioate	87875	Organic acids and derivatives	Thiocarboxylic acids and derivatives	Thioesters	*		37	0	M+FA-H	C9H16OS	0.529770226	347.129189	329.3401476	166.4970488	233.1407442	159.5323165	286.1334018	93.31951352	189.1928215	123.5051039
6.06_402.1770m/z	402.1770198	6.056116667	neg	Uplandicine	68316	Unclassified	Unclassified	Unclassified			37	0	M+FA-H	C17H27NO7	0.181291908	2187.057922	2173.268635	2346.156817	4634.007024	4644.78727	3671.814902	1942.256204	2104.776293	2260.169222
6.10_269.1011m/z	269.1011381	6.101316667	neg	Aspidinol	68482	Unclassified	Unclassified	Unclassified			37	0	M+FA-H	C12H16O4	-8.584410189	14291.08814	14727.26178	15323.95169	10771.11894	10717.49673	9765.768073	14721.12146	15559.594	16419.62391
6.20_387.1279m/z	387.1278999	6.195716667	neg	4-Hydroxy-3-(2-hydroxyethyl)acetophenone 4-glucoside	92409	Organic oxygen compounds	Organooxygen compounds	Carbohydrates and carbohydrate conjugates			37	0	M+FA-H	C16H22O8	-5.175330849	3451.143102	2940.872772	3667.478293	2851.263262	3088.31284	3447.465863	4167.525235	4009.349446	4288.536025
6.25_675.1799m/z	675.1798949	6.248083333	neg	Leucodelphinidin 3-O-(beta-D-glucopyranosyl-(1->4)-alpha-L-rhamnopyranoside	LMPK12020207	Lipids and lipid-like molecules	Polyketides	Flavonoids			37	0	M+FA-H	C27H34O17	3.320406982	4331.638577	2746.112445	4224.074482	3775.165454	2940.157331	3759.910681	3078.977052	4710.888797	3271.689191
6.42_326.1048m/z	326.1047594	6.41995	neg	Balsoxine	68395	Unclassified	Unclassified	Unclassified			37	0	M+FA-H	C17H15NO3	4.849390576	2663.572346	3077.23751	3038.998803	5064.132376	4863.030936	5192.430844	3261.559227	3158.301294	3162.199967
6.43_395.1630m/z	395.1630301	6.433933333	neg	strychnine N-oxide	34472	Unclassified	Unclassified	Unclassified			37	0	M+FA-H	C21H22N2O3	5.09670314	1386.510899	1656.779242	1343.567784	1536.108619	1231.875224	1365.939449	2063.106029	1865.656461	1733.736176
6.46_317.1540m/z	317.1539569	6.458366667	neg	1,1,2-Triphenylpropane	69935	Unclassified	Unclassified	Unclassified			37	0	M+FA-H	C21H20	-2.743297083	446.4535033	603.0436405	705.7315534	0.001403199	96.13085183	12.29524088	281.7199853	326.3350925	476.2120127
6.51_479.1904m/z	479.190412	6.509966667	neg	Iridodial glucoside tetraacetate	LMPR0102070026	Lipids and lipid-like molecules	Prenol lipids	Isoprenoids		C11657	37	1.06	M-H2O-H	C24H34O11	-3.730957201	22562.94019	23413.04705	24224.65043	11604.70886	11854.93981	11387.14539	19975.90633	21942.27641	21920.65553
6.69_583.3129m/z	583.312923	6.69475	neg	Vellosimine	64339	Unclassified	Unclassified	Unclassified			37	0	2M-H	C19H20N2O	8.68169316	4742.032776	5953.154764	5915.001909	1286.63656	1536.996889	1231.664603	2795.470521	4453.904662	4318.894654
6.96_365.1696m/z	365.1695844	6.96195	neg	Bufotenine O-glucoside	86346	Organic oxygen compounds	Organooxygen compounds	Carbohydrates and carbohydrate conjugates	*		37	0	M-H	C18H26N2O6	-6.078320162	8061.126438	7355.964385	7467.523321	13896.99118	12331.85584	14520.21425	7227.30621	8770.281757	8133.798974
6.96_501.1451m/z	501.1451306	6.96195	neg	alpha-L-Rhamnopyranosyl-(1->2)-beta-D-galactopyranosyl-(1->2)-beta-D-glucur	69297	Unclassified	Unclassified	Unclassified			37	0	M-H	C18H30O16	-1.947216933	1542.866866	1953.64752	1451.881029	3597.087946	3073.164858	4147.990245	1443.616903	2277.942551	1977.073328
7.03_441.1205m/z	441.1205487	7.02545	neg	Methabenzthiazuron	68678	Unclassified	Unclassified	Unclassified			37	0	2M-H	C10H11N3OS	7.371523832	3294.547144	3496.348932	2990.537135	1113.077122	1172.852586	1043.963034	2299.268488	2192.066665	1720.294265
7.18_470.2377m/z	470.2377097	7.178516667	neg	Nafoxidine	69880	Unclassified	Unclassified	Unclassified			37	0	M+FA-H	C29H31NO2	9.471354483	14523.78705	18474.91099	16963.73347	20866.57586	19587.10876	20726.40087	13248.14709	16699.36265	14208.51355
7.20_601.2141m/z	601.2141309	7.201166667	neg	Glaucarubolone 15-O-beta-D-glucopyranoside	90393	Lipids and lipid-like molecules	Prenol lipids	Terpene lactones	*		37	0	M+FA-H	C26H36O13	0.605478303	1159.525278	1238.602572	1669.159894	899.5153574	810.9133257	767.708584	764.5901295	890.3729136	467.4982791
7.26_483.1858m/z	483.1858021	7.262266667	neg	Equol	82413	Phenylpropanoids and polyketides	Isoflavonoids	Isoflavans	*	C14131	37	0	2M-H	C15H14O3	9.27301383	3800.729068	4725.551274	4624.518485	7303.452863	6817.744133	6541.961214	2714.394469	3144.838065	3415.687952
7.36_402.1957m/z	402.1957218	7.36395	neg	Laudanosine	1154	Organoheterocyclic compounds	Isoquinolines and derivatives	Benzylisoquinolines	*		37	0	M+FA-H	C21H27NO4	9.828249973	1414.0313	1584.892581	1324.682182	2114.508585	2229.6671	1953.675257	1048.627644	1477.39793	1799.270573
7.55_569.2501m/z	569.2500759	7.547766667	neg	Leucomethylene blue (Reduced Methylene Blue)	1223	Unclassified	Unclassified	Unclassified			37	0	2M-H	C16H19N3S	-4.532956911	2122.656637	2143.184075	2864.72574	3530.903294	3175.067549	4157.988226	2123.187435	1833.951745	2373.380562
7.55_673.2824m/z	673.2824117	7.547766667	neg	Zolpidem Metabolite I	3072	Unclassified	Unclassified	Unclassified			37	0	2M-H	C19H19N3O3	6.533101831	7467.32078	7831.787664	7528.39868	449.0375915	0.001403199	285.6853819	2294.800124	2131.705376	2306.036292
7.62_283.1551m/z	283.1551412	7.621333333	neg	(9S,13S)-1a,1b-dihomo-jasmonic acid	36063	Lipids and lipid-like molecules	Fatty Acyls	Octadecanoids			37	0	M+FA-H	C14H22O3	0.183786847	566.0091181	576.9019312	490.3033872	780.0697197	563.4259925	514.5489364	360.6963397	483.9993817	349.8353446
7.78_285.1710m/z	285.1709771	7.7828	neg	CPX	44518	Unclassified	Unclassified	Unclassified			37	0	M-H2O-H	C16H24N4O2	-3.641818624	770.1252335	871.3665732	987.0506703	1765.855619	1693.133769	1732.230209	903.6900373	895.2632168	832.0074331
7.92_601.2249m/z	601.2248701	7.91555	neg	5,4'-Dihydroxy-6-C-prenylflavanone 4'-xylosyl-(1->2)-rhamnoside	52591	Unclassified	Unclassified	Unclassified			37	0	M-H	C31H38O12	-6.940995397	54299.18109	54835.64112	56739.56389	71414.60536	71315.52777	72915.80023	55741.216	69062.96782	64223.38347
8.07_439.1587m/z	439.1586967	8.066183333	neg	Specionin	67943	Unclassified	Unclassified	Unclassified			37	0	M+FA-H	C20H26O8	-5.76909576	8931.193524	10308.8056	10077.63783	15806.74683	14909.10658	13812.36271	5055.150192	6426.575458	6235.43097
8.68_657.2873m/z	657.2873403	8.68065	neg	Cypendazole	72678	Unclassified	Unclassified	Unclassified			37	0	2M-H	C16H19N5O3	-4.499887044	10880.49959	13109.41409	11480.79436	716.5181896	726.8936352	739.368864	4790.090581	5264.904838	4830.927896
0.74_269.1235m/z	269.1235242	0.7359	neg	2-[3-Carboxy-3-(methylammonio)propyl]-L-histidine	3400	Unclassified	Unclassified	Unclassified			36.9	0	M-H	C11H18N4O4	-7.420326232	530.0331977	111.586489	1092.239147	190.6456413	2300.395359	371.9352832	450.0170987	1476.006062	2060.776901
0.80_470.1516m/z	470.1515591	0.800366667	neg	Hyaluronic acid	58088	Organic oxygen compounds	Organooxygen compounds	Carbohydrates and carbohydrate conjugates	**	C00518	36.9	0	M+FA-H	C16H27NO12	0.0728113	5806.169505	7248.230931	6385.623304	4587.410684	5128.772112	5728.222145	5535.824708	4666.388199	6843.33577
1.14_187.0226m/z	187.0226405	1.1382	neg	Methyl (Z)-5-(1-propynyl)-2-thienylacrylate	90250	Lipids and lipid-like molecules	Fatty Acyls	Fatty acid esters	*		36.9	0	M-H2O-H	C11H10O2S	1.606733695	6742.457354	4898.418519	4002.077591	7586.645063	6409.66467	8551.990518	3989.931213	5902.994596	5226.608548
1.14_547.0418m/z	547.0418384	1.1382	neg	UDP-4-keto-rhamnose	63210	Unclassified	Unclassified	Unclassified			36.9	0	M-H	C15H22N2O16P2	8.500986714	8854.308638	14678.41554	17655.13274	6032.40915	9976.188673	6650.234597	13884.61505	7428.524711	11267.47996
1.16_180.0337m/z	180.0337367	1.160166667	neg	Methionine sulfone	HMDB0062174	Organic acids and derivatives	Carboxylic acids and derivatives	Amino acids, peptides, and analogues			36.9	0	M-H	C5H11NO4S	0.741538275	1339.322541	1439.051688	1973.237712	1463.637268	2091.081268	2267.187694	1358.326432	1971.084076	2116.203346
1.18_194.0683m/z	194.0682956	1.180366667	neg	Glucosaminic acid	3334	Unclassified	Unclassified	Unclassified			36.9	0	M-H	C6H13NO6	6.586447516	720.5133534	411.601351	582.0022055	525.0621635	679.4967495	641.3217955	721.6932241	694.8865068	620.146136
1.18_279.0600m/z	279.0599608	1.180366667	neg	Mefenacet	HMDB0254408	Unclassified	Unclassified	Unclassified			36.9	0	M-H2O-H	C16H14N2O2S	0.681614633	4057.454163	3675.960758	3631.526685	3376.813296	2472.057749	3946.200694	2519.182906	4279.52758	3398.902184
1.20_280.0111m/z	280.0110886	1.202333333	neg	Menazon	72757	Unclassified	Unclassified	Unclassified			36.9	0	M-H	C6H12N5O2PS2	4.845646372	6922.127858	7319.369001	7233.644808	8837.908757	8440.440379	9509.635495	7279.43715	8363.383893	8255.983488
1.22_198.0385m/z	198.0384722	1.224233333	neg	Thiolanilide	96349	Unclassified	Unclassified	Unclassified			36.9	0	M-H2O-H	C12H11NOS	0.821732483	1265.804838	1205.193104	1444.427182	989.0080761	1081.397332	1176.506453	1410.344336	1347.493942	1393.406797
1.36_331.0674m/z	331.067441	1.358266667	neg	Pyrocatechol glucuronide	1826	Unclassified	Unclassified	Unclassified			36.9	0	M+FA-H	C12H14O8	1.296009406	502.6936355	467.1363749	689.6953091	234.8708885	285.3689232	59.97496613	489.0540253	576.8577576	328.6715386
1.63_235.0226m/z	235.0225843	1.631516667	neg	L-Djenkolic acid	44506	Unclassified	Unclassified	Unclassified			36.9	0	M-H2O-H	C7H14N2O4S2	3.647090554	5613.530833	6280.325649	7332.148044	6876.065389	6821.755086	7145.382185	8856.912989	7304.029371	7713.679224
1.84_276.0080m/z	276.0079864	1.841666667	neg	N-Phosphohypotaurocyamine	66013	Unclassified	Unclassified	Unclassified			36.9	0	M+FA-H	C3H10N3O5PS	8.248607865	4652.577668	4911.723284	6174.782751	3890.619853	3768.57233	3871.003653	6402.422081	5751.079322	5741.618148
1.93_215.0538m/z	215.0538187	1.933583333	neg	(2Z,4'Z)-2-(5-Methylthio-4-penten-2-ynylidene)-1,6-dioxaspiro[4.4]non-3-ene	88601	Organic oxygen compounds	Organooxygen compounds	Ethers	*		36.9	0	M-H2O-H	C13H14O2S	0.893568236	3046.784478	3117.577576	2362.513437	2681.898945	2949.129772	3236.848742	2817.837164	2988.485531	2569.461108
1.93_284.0753m/z	284.0753367	1.933583333	neg	3-Hydroxy-N-methylpyridinium glucuronide	2201	Unclassified	Unclassified	Unclassified			36.9	0	M-H	C12H15NO7	-7.852649886	8936.274226	8754.954334	6581.224846	8537.968836	9268.317917	9519.677643	8176.918371	8058.58437	7263.115602
1.95_697.1790m/z	697.1789939	1.945716667	neg	10-Hydroxydihydrosanguinarine	64377	Unclassified	Unclassified	Unclassified			36.9	0	2M-H	C20H15NO5	-5.406540299	2253.977464	2883.377423	3165.966616	0.001403199	0.001403199	0.001403199	1980.163381	2650.15213	2155.300943

13.56_355.1584m/z	355.1583931	13.55923333	neg	L-(-)Sorbose	3317	Unclassified	Unclassified	Unclassified			36.9	0	2M-H	C7H14O5	-7.236899739	10181.56214	11444.00482	12702.85936	7833.338978	7655.92298	10256.49066	11440.95192	12391.29986	9207.567501
14.65_383.1895m/z	383.1895222	14.6516	neg	5(S),6(R)-7-trihydroxymethyl Heptanoate	44893	Unclassified	Unclassified	Unclassified			36.9	0	2M-H	C8H16O5	-7.154093734	3902.883574	3633.882223	4199.620116	2707.27267	3552.184855	3388.585693	3898.912026	3176.772901	4181.857452
14.66_412.1922m/z	412.192167	14.6619	neg	JWH145	85145	Unclassified	Unclassified	Unclassified			36.9	0	M+FA-H	C26H25NO	0.952460397	655.6071829	1762.077923	1814.425874	1151.91603	2014.868683	1931.858924	2042.948815	2257.392726	1791.080726
2.54_375.0656n	420.0640513	2.5421	neg	2-Methylpropyl glucosinolate	71614	Organic oxygen compounds	Organooxygen compounds	Carbohydrates and carbohydrate conjugates			36.9	0	M-H, M+FA-H	C11H21NO9S2	-0.350402832	4854.830393	6271.211991	6314.482282	7124.352562	7230.2439	7745.731191	5855.514913	5862.26084	6854.416052
2.78_361.0634m/z	361.0633872	2.776216667	neg	O-Acetylserine	6809	Organic acids and derivatives	Carboxylic acids and derivatives	Amino acids, peptides, and analogues	**	C00979	36.9	0	M-H2O-H	C13H21N2O7PS	1.367368602	4798.930436	4913.58388	4730.737671	5553.362316	6016.99034	5647.403002	4706.503067	5583.616734	5641.943189
2.84_288.1455m/z	288.1454761	2.83525	neg	4-Amino-6,7-dimethoxy-2-(1-piperazinyl)quinazoline	2460	Unclassified	Unclassified	Unclassified			36.9	0	M-H	C14H19N5O2	-3.881345884	472.197743	612.434131	851.917794	815.6066239	559.2304712	861.3204778	468.0268272	706.3119486	544.2428294
3.10_124.0403m/z	124.0403397	3.10055	neg	3-Hydroxyaminophenol	70194	Unclassified	Unclassified	Unclassified			36.9	0	M-H	C6H7NO2	-0.498319658	258.0690821	235.275874	303.4053095	253.9455112	241.272407	209.2727501	246.4863962	246.8363274	310.7829143
3.47_395.0291m/z	395.0291209	3.47105	neg	4,5-Dihydroxyphthalate	65938	Unclassified	Unclassified	Unclassified			36.9	0	2M-H	C8H6O6	8.891847728	1711.685925	2382.971206	2519.403902	320.1596391	282.8016153	428.5153252	2948.194057	2696.313805	2785.914127
3.48_582.0658m/z	582.0657886	3.484883333	neg	O-acetyl-ADP-ribose	HMDB0059656	Nucleosides, nucleotides, and analogues	Purine nucleotides	Purine nucleotide sugars			36.9	0	M-H2O-H	C17H25N5O15P2	2.314676197	4297.840979	5510.827437	5687.105045	7877.725754	6343.651899	6504.265326	5424.366184	5500.393396	5482.000742
3.56_267.0963m/z	267.0963395	3.564783333	neg	N-Demethylpromethazine sulfoxide	2148	Unclassified	Unclassified	Unclassified			36.9	0	M-H2O-H	C16H18N2OS	0.686515455	5910.285565	6408.726981	6348.410021	8744.771718	8208.005557	8288.065562	5991.959906	6640.70829	6739.293603
3.75_963.3165m/z	963.3165141	3.747833333	neg	Dukunolide A	90946	Organoheterocyclic compounds	Naphthopyrans	Unclassified	*		36.9	0	2M-H	C26H26O9	8.737479988	1964.426559	1902.750918	1287.940168	1703.612032	3074.177169	1445.087515	1423.125665	1762.222359	1798.51811
3.94_258.9919m/z	258.9918751	3.9351	neg	Caffeic acid 4-O-sulfate	HMDB0041708	Phenylpropanoids and polyketides	Cinnamic acids and derivatives	Hydroxycinnamic acids and derivatives	*		36.9	0	M-H	C9H8O7S	0.299709735	536.2072936	300.984785	613.2770558	95.24740564	308.2650697	356.1324539	393.5252696	495.8071098	352.0308785
3.94_391.0262m/z	391.026219	3.9351	neg	Epithienamycin E	69198	Unclassified	Unclassified	Unclassified			36.9	0	M-H	C13H16N2O8S2	-3.346667305	21946.80846	20934.70164	23478.48669	798.0035635	929.0022349	870.2189718	18041.53307	20003.87643	19542.70023
4.26_421.0754m/z	421.0753867	4.2568	neg	α,α'-Trehalose 6-phosphate	3529	Unclassified	Unclassified	Unclassified			36.9	0	M-H	C12H23O14P	0.286880607	285.2396685	346.3675768	173.992349	52.82407969	0.001403199	76.46415728	480.7841841	222.0213161	390.8220885
4.32_312.0912m/z	312.0912455	4.3224	neg	Miserotoxin	67029	Unclassified	Unclassified	Unclassified			36.9	0	M+FA-H	C9H17NO8	-8.887882048	517.0586075	486.8021356	643.0544348	302.8221682	314.6071137	493.3494335	537.8858401	614.3248415	607.544981
4.34_288.0491m/z	288.0490573	4.344733333	neg	GW 610	45012	Unclassified	Unclassified	Unclassified			36.9	0	M-H	C15H12FNO2S	-3.266363659	1197.099237	1318.60636	1485.794671	611.2313128	464.5550277	486.5792757	1040.66358	990.5317574	1131.486702
4.34_342.1017m/z	342.1017082	4.344733333	neg	Niazinin	HMDB0031942	Organic oxygen compounds	Organooxygen compounds	Carbohydrates and carbohydrate conjugates	*		36.9	0	M-H	C15H21NO6S	0.076447994	2904.978627	3087.945158	3506.905932	3134.745536	2570.009434	2601.47399	2665.36583	3112.155767	2944.511231
4.37_374.9819m/z	374.9818576	4.367166667	neg	Isorhamnetin 3-sulfate	HMDB0240537	Phenylpropanoids and polyketides	Flavonoids	O-methylated flavonoids			36.9	0	M-H2O-H	C16H10O10S-2	0.586803828	927.2188695	943.5348584	784.590651	1896.268617	1228.826593	773.4697263	768.0135033	930.3423462	1012.09553
4.37_447.1508m/z	447.1507853	4.367166667	neg	L-3-Hydroxykynurenine	HMDB0011631	Organic oxygen compounds	Organooxygen compounds	Carbonyl compounds	**	C03227	36.9	0	2M-H	C10H12N2O4	-3.01670851	95.77345818	0.001403199	181.5437686	552.4558116	288.6466526	420.3569874	0.001403199	0.001403199	122.4544676
4.45_269.0467m/z	269.0466898	4.454616667	neg	Eriodictyol	HMDB0005810	Phenylpropanoids and polyketides	Flavonoids	Flavans	***	C05631	36.9	0	M-H2O-H	C15H12O6	3.967340797	502.8625486	714.8929912	686.3014161	898.7922565	904.3236087	730.6696982	722.9799345	364.0842934	380.5108942
4.48_246.0161m/z	246.0161309	4.477166667	neg	Pyridoxal 5'-phosphate	HMDB0001491	Organoheterocyclic compounds	Pyridines and derivatives	Pyridine carboxaldehydes	**	C00018	36.9	0	M-H	C8H10NO6P	-4.721693728	62.6122086	49.36297712	37.9810688	36.87932098	94.83262857	103.9394933	38.38861091	51.66503334	0.001403199
4.52_467.0291m/z	467.0291157	4.521733333	neg	phenylglycol 3-O-sulfate	734	Unclassified	Unclassified	Unclassified			36.9	0	2M-H	C8H10O6S	-6.892561737	2737.929625	361.312885	9594.683896	714.4125009	292.6617979	627.879258	475.6782025	482.1355387	1587.714471
4.64_258.1713m/z	258.1712608	4.641416667	neg	Hexanoylcarnitine	LMFA07070070	Lipids and lipid-like molecules	Fatty Acyls	Fatty acid esters	*		36.9	0	M-H	C13H25NO4	0.690338924	333.1506485	201.8187515	260.7001714	380.9292106	365.7966264	445.6598413	431.9518671	357.3137001	248.0930541
4.70_458.0637m/z	458.0637464	4.703166667	neg	Dermatan	5605	Unclassified	Unclassified	Unclassified			36.9	0	M-H2O-H	C14H23NO15S	5.759047717	11342.58886	10550.36503	10360.69766	12410.45416	13219.58293	13742.42765	14153.88979	11061.45895	12035.73182
4.73_330.1404n	311.1225606	4.725416667	neg	Desmethyl methotrimeprazine 5-sulfoxide	1194	Unclassified	Unclassified	Unclassified			36.9	0	M-H2O-H, M-H	C18H22N2O2S	0.614106207	12754.72472	12853.44115	17969.43838	25773.36303	25170.96102	24357.77511	12955.85332	14186.21577	14547.77409
4.75_286.0910m/z	286.0909516	4.747466667	neg	Volkenin	66922	Unclassified	Unclassified	Unclassified			36.9	0	M-H	C12H17NO7	-7.920119335	3629.314222	4097.286655	3676.088996	3411.157952	3608.444499	3151.258571	3358.256477	3978.156189	3913.605854
4.84_389.1289m/z	389.1288801	4.836566667	neg	4-(2-Benzofuranyl)pyridine	70186	Unclassified	Unclassified	Unclassified			36.9	0	2M-H	C13H9NO	-1.720700389	1481.419418	1066.816504	1369.317066	797.3183966	488.2437184	759.3179054	1404.380196	1796.921392	902.8141454
4.95_645.1468m/z	645.1468064	4.94705	neg	Pelargonidin 3-O-[2-O-(beta-D-xylopyranosyl)-6-O-(methyl-malonyl)-bata-D-gala	46780	Unclassified	Unclassified	Unclassified			36.9	0	M-H2O-H	C30H32O17	1.0509059	1091.990301	753.8701022	698.913316	1196.760189	930.8347242	1453.417277	1079.307333	877.3982849	1518.204108
4.97_334.1608m/z	334.1608371	4.969683333	neg	Almotriptan	HMDB0015054	Organoheterocyclic compounds	Indoles and derivatives	Tryptamines and derivatives			36.9	0	M-H	C17H25N3O2S	4.073912657	1630.553945	1890.050326	2017.033796	1294.578648	902.7767583	1148.261625	1408.489918	1489.926303	1645.307433
4.99_355.1013m/z	355.101349	4.99225	neg	trans-Cinnamoyl beta-D-glucoside	66124	Unclassified	Unclassified	Unclassified			36.9	0	M+FA-H	C15H18O7	-6.793634908	7469.915135	7847.392929	7985.051563	6783.692613	6792.190972	6754.677605	8490.336341	7144.39608	7777.407187
5.01_277.0826m/z	277.0825737	5.014716667	neg	Nalidixic Acid	HMDB0014917	Organoheterocyclic compounds	Diazanaphthalenes	Naphthyridines		C05079	36.9	0	M+FA-H	C12H12N2O3	-1.816017806	673.1215862	726.8452542	702.4474703	567.552213	526.0876621	690.1644491	807.7985098	613.8007505	388.1579938
5.01_367.0025m/z	367.0024867	5.014716667	neg	6-Thiourate	HMDB0060417	Organoheterocyclic compounds	Imidazopyrimidines	Purines and purine derivatives	**	C16613	36.9	0	2M-H	C5H4N4O2S	-3.341287776	848.7107928	901.0419869	786.2625333	417.5055744	488.3500622	399.3150829	426.553793	530.9970284	248.0724693
5.18_441.2068m/z	441.2068299	5.1777	neg	QUININE ETHYL CARBONATE	44610	Unclassified	Unclassified	Unclassified			36.9	0	M+FA-H	C23H28N2O4	9.388103254	102296.2384	105233.9512	107657.3689	73678.71759	67684.53539	69471.16421	105470.1794	94819.10249	100971.3746
5.22_385.1146m/z	385.11464	5.222166667	neg	Toxoflavine	71343	Unclassified	Unclassified	Unclassified			36.9	0	2M-H	C7H7N5O2	5.09538532	154.5649439	217.9236958	305.6144747	180.5991901	0.001403199	0.001403199	0.001403199	0.001403199	313.3577707
5.22_469.0754m/z	469.0753745	5.222166667	neg	6-(3,4-Dihydroxyphenyl)-6a,12b-dihydro-3,10,11,12-tetrahydroxy-[2]benzopyran	47226	Unclassified	Unclassified	Unclassified			36.9	0	M+FA-H	C22H16O9	-5.330298948	5126.251607	6117.313438	5673.412748	4813.434978	4069.712919	4248.783728	5620.353333	5364.736443	6232.693114
5.22_563.1338m/z	563.1337968	5.222166667	neg	Homocysteine-penicillamine disulfide	1733	Unclassified	Unclassified	Unclassified			36.9	0	2M-H	C9H18N2O4S2	-0.930458873	39290.53486	34912.37543	34689.80633	34477.813	34749.30172	32965.19634	46519.22597	44149.26532	48202.94152
5.38_499.1801m/z	499.1800669	5.384216667	neg	alpha-CEHC glucuronide	HMDB0062445	Organic oxygen compounds	Organooxygen compounds	Carbohydrates and carbohydrate conjugates	*		36.9	0	M+FA-H	C22H30O10	-4.476550843	9726.648386	9466.907402	9816.027845	10589.18515	11104.74736	9664.746667	10441.62739	10775.80972	8908.329613
5.41_501.2199m/z	501.2199303	5.406433333	neg	Ac-Tyr-OEt	44264	Unclassified	Unclassified	Unclassified			36.9	0	2M-H	C13H17NO4	-8.58036846	8165.939167	9297.26074	9649.193773	12291.45935	10252.16317	9917.696221	7800.83771	10386.28849	6550.307058
5.45_302.0648m/z	302.0647818	5.451166667	neg	N-Benzoyl-4-hydroxyanthranilate	66137	Unclassified	Unclassified	Unclassified			36.9	0	M+FA-H	C14H11NO4	-8.670279903	5346.551463	5434.170426	5687.052398	1897.952811	1773.563351	1786.748645	5072.000312	3515.071281	3971.024322
5.50_323.1070m/z	323.1069694	5.497816667	neg	N-FORMYLMETHIONYLPHENYLALANINE	44227	Unclassified	Unclassified	Unclassified			36.9	0	M-H	C15H20N2O4S	-0.408258451	433.1520499	440.8880798	453.5227896	345.3630499	377.6438651	319.0007079	284.6301506	406.0549705	442.9143821
5.53_467.0512m/z	467.0512172	5.525416667	neg	Cyclobrassinin	HMDB0033352	Organoheterocyclic compounds	Indoles and derivatives	Indoles			36.9	0	2M-H	C11H10N2S2	3.018782566	2333.958884	2137.780577	2415.627135	924.8642333	1146.821561	911.7952296	1746.282371	1728.966647	1989.229467
5.60_401.0808m/z	401.0807569	5.602	neg	4-Phospho-N-pantothenoylcysteine	6014	Unclassified	Unclassified	Unclassified			36.9	0	M-H	C12H23N2O9PS	4.58948913	3630.732308	3592.87351	3881.319812	8607.128389	8179.006848	8164.046066	3657.091201	3664.102896	2990.606885
5.70_352.1567m/z	352.1566759	5.701283333	neg	N-[2-(3,4-Dimethoxyphenyl)ethyl]-3,4-dimethoxycinnamic acid amide	88275	Phenylpropanoids and polyketides	Cinnamic acids and derivatives	Cinnamic acid amides	*		36.9	0	M-H2O-H	C21H25NO5	3.351908189	750.1765659	933.9821635	892.9811493	757.1159492	807.5709886	1183.925877	575.8582252	414.6250439	670.7245765
5.94_677.2405m/z	677.2405054	5.937716667	neg	6-Hydroxygliclazide	HMDB0014028	Benzenoids	Benzene and substituted derivatives	Benzenesulfonamides	*		36.9	0	2M-H	C15H21N3O4S	-4.08771945	21776.54991	21539.55428	24286.72587	23339.05075	31331.08829	30239.78974	19465.28563	22898.20228	20747.07053
5.97_285.1457m/z	285.1456502	5.965483333	neg	1-Isothiocyanato-4-methylpentane	HMDB0038435	Organosulfur compounds	Isothiocyanates	Unclassified			36.9	0	2M-H	C7H13NS	-2.845198605	980.6844362	1129.817812	1107.482104	959.1562928	1332.046748	1314.742619	892.6352189	942.928215	831.4830547
6.17_475.0837m/z	475.0837052	6.171316667	neg	DIPYROCETYL	43821	Unclassified	Unclassified	Unclassified			36.9	0	2M-H	C11H10O6	-9.44019562	8959.669532	8882.1476	9324.269743	3354.562959	3364.201165	4081.986712	6511.095977	7756.929864	7851.471888
6.20_371.0702m/z	371.070225	6.195716667	neg	Miraxanthin-II	67061	Unclassified	Unclassified	Unclassified			36.9	0	M+FA-H	C13H14N2O8	-9.17969569	5355.459745	4703.08639	5193.396388	8642.313453	8195.941973	8598.828232	5685.910137	6246.180545	7024.108043
6.28_441.1383m/z	441.1383271	6.2811	neg	Aloesol 7-glucoside	HMDB0040565	Organic oxygen compounds	Organooxygen compounds	Carbohydrates and carbohydrate conjugates			36.9	0	M+FA-H	C19H24O9	-4.816765906	3299.295447	2911.452061	3618.703003	1485.728658	1147.316599	1195.030742	2564.887683	2898.621875	3494.404815
6.32_633.1008m/z	633.1007728	6.320716667	neg	N-Glycolyl-D-mannosamine 6-phosphate	66166	Unclassified	Unclassified	Unclassified			36.9	0	2M-H	C8H16NO10P	8.964756584	2251.109194	3800.225813	3167.929481	3331.933949	3063.312285	3394.506905	2236.576362	1678.422793	3147.561619
6.59_475.2681m/z	475.2681138	6.592933333	neg	(3b,4b,11b,14b)-11-Ethoxy-3,4-epoxy-14-hydroxy-12-cyathen-15-al 14-xyloside	HMDB0034617	Lipids and lipid-like molecules	Prenol lipids	Diterpenoids	*		36.9	0	M-H2O-H	C27H42O8	-4.073206556	1388.697353	1376.731197	1350.405781	0.001403199	0.001403199	20.97649896	191.9640584	182.1312256	76.1269454
6.66_375.1485m/z	375.1485476	6.663683333	neg	MS-275	45517	Unclassified	Unclassified	Unclassified			36.9	0	M-H	C21H20N4O3	6.070671744	11576.93945	14485.75523	14820.25861	5918.31441	5797.40583	4715.070895	8783.205683	10455.85574	11499.78264
6.69_298.1782n	297.170852	6.69475	neg	9'-desmethylgranisetron	HMDB0061061	Organoheterocyclic compounds	Benzopyrazoles	Indazoles	*		36.9	0	M-H2O-H, M-H	C17H22N4O	-3.987442899	2411.352692	3178.438973	3036.985983	3987.034325	4221.818223	3687.977051	1955.82153	2680.357632	2907.997254
6.86_615.0900m/z	615.0899839	6.86375	neg	Nimesulide	44341	Unclassified	Unclassified	Unclassified			36.9	0	2M-H	C13H12N2O5S	6.29019534	24492.33775	24881.62253	21340.14191	25021.29493	26337.85042	26067.76065	22282.95272	23262.0732	21314.10452
7.01_453.1385m/z	453.1384556	7.01105	neg	Torachrysone 8-glucoside	HMDB0038600	Organic oxygen compounds	Organooxygen compounds	Carbohydrates and carbohydrate conjugates			36.9	0	M+FA-H	C20H24O9	-4.360220801	1334.997729	1051.524718	1244.853982	1287.040504	1172.122917	1529.448791	1200.885554	1252.266872	1150.675248
7.30_347.1610m/z	347.1610486	7.29525	neg	Trichostatin	LMPK01000055	Lipids and lipid-like molecules	Polyketides	Linear polyketides			36.9	0	M+FA-H	C17H22N2O3	-0.651484244	1222.384603	1324.763468	1404.2693	1898.53649	2052.402686	1914.01349	956.1884026	800.1119943	1031.554066
7.61_372.2393m/z	372.2393045	7.6091	neg	N-Palmitoyl Glutamine	HMDB0241924	Lipids and lipid-like molecules	Fatty Acyls	Fatty amides			36.9	0	M+FA-H	C18H33NO4	0.437366054	1850.353741	2306.938939	2272.670046	2543.945246	2192.204308	2897.520937	1893.19649	2188.059726	2132.361231
7.67_474.2171m/z	474.2170515	7.66825	neg	Atracurium (Quaternary acid)	1161	Unclassified	Unclassified	Unclassified			36.9	0	M+FA-H	C24H31NO6	8.645720965	16755.87504	18430.37701	18893.14466	21128.3506	21306.86069	21092.86334	12990.93261	13331.12107	13900.90813
7.67_542.2046m/z	542.2046097	7.66825	neg	Validamycin A	69311	Organic oxygen compounds	Organooxygen compounds	Carbohydrates and carbohydrate conjugates	**	C12112	36.9	0	M+FA-H	C20H35NO13	-8.916472518	5313.497312	5951.466509	6503.157302	6666.182964	6924.808519	7614.512229	3951.465432	4322.563889	4273.796635
8.07_626.2182m/z	626.2182002	8.074883333	neg	3'-Deoxystreptomycin 6-phosphate	2500	Unclassified	Unclassified	Unclassified			36.9	0	M-H2O-H	C21H40N7O14P	-1.618524353	1402.094456	1680.439582	1101.845075	0.001403199	0.001403199	0.001403199	740.7309153	663.6208566	717.3220954
8.15_471.2423m/z	471.2422541	8.147366667	neg	Chenodeoxycholic acid 3-sulfate	HMDB0002586	Lipids and lipid-like molecules	Steroids and steroid derivatives	Bile acids, alcohols and derivatives	*		36.9	0	M-H	C24H40O7S	0.118290309	3899.249103	4065.118895	4472.059065	9.495733709	2.185811775	7.117326285	2841.341578	3341.849647	2949.938491
8.18_199.0977m/z	199.0977285	8.181083333	neg	6,9-dioxo-decanoic acid	LMFA01060080	Lipids and lipid-like molecules	Fatty Acyls	Fatty Acids and Conjugates			36.9	0	M-H	C10H16O4	0.7295371	309.2260626	196.5081646	256.9819786	202.091296	270.7750865	191.6237703	265.9818692	113.5890236	252.6635799
8.78_471.2597m/z	471.2597116	8.782416667	neg	Prostaglandin D2-1-glyceryl ester	85243	Lipids and lipid-like molecules	Fatty Acyls	Eicosanoids	*		36.9	0	M+FA-H	C23H38O7	-0.574344335	3099.175285	3384.102286	3787.585594	3298.181932	5287.28736	4996.760059	2139.974493	2265.875827	2059.073543
9.02_547.1985m/z	547.1984923	9.01635	neg	Isocycloheterophyllin	HMDB0029471	Phenylpropanoids and polyketides	Cinnamic acids and derivatives	Hydroxycinnamic acids and derivatives	*		36.9	0	M+FA-H	C30H30O7	2.262370322	4485.538959	6753.382892	6496.61291	94.81500451	229.2335518	267.6196587	1572.567494	2312.603102	2098.806206
9.33_513.2685m/z	513.2684587	9.332316667	neg	18-acetoxy-PGF2α-11-acetate methyl ester	36163	Unclassified	Unclassified	Unclassified			36.9	0	M+FA-H	C25H40O8	-4.404251019	3335.51605	3733.751069	4255.743785	4225.175318	4182.105199	4177.568087	2907.177399	4264.960813	3867.019907
9.92_497.0338m/z	497.0338412	9.922166667	neg	Allura red AC	88798	Benzenoids	Naphthalenes	Naphthalene sulfonic acids and derivatives			36.9	0	M+FA-H	C18H16N2O8S2	1.838041887	2382.353586	2474.60677	1784.710026	2215.018228	2177.865884	2276.98575	2555.385124	2623.539326	2355.087355
0.76_239.0345m/z	239.0344593	0.757316667	neg	Alternariol	71377	Phenylpropanoids and polyketides	Coumarins and derivatives	Unclassified	*	C16838	36.8	0	M-H2O-H	C14H10O5	-2.026670321	2719.443489	3285.427425	3461.215487	3982.930506	3037.016426	3638.737787	2478.682045	3737.480741	2600.876771
0.80_367.0692m/z	367.0692035	0.800366667	neg	4-O-Methyl-gallate	44726	Unclassified	Unclassified	Unclassified			36.8	0	2M-H	C8H8O5	5.795534122	10161.36345	11389.89496	10597.87717	12315.36744	12680.98597	11861.6348	9466.607889	9357.363236	8235.804091
0.88_411.0908m/z	411.0908365	0.884633333	neg	Furocoumarinic acid glucoside	86367	Organic oxygen compounds	Organooxygen compounds	Carbohydrates and carbohydrate conjugates	*		36.8	0	M+FA-H	C17H18O9	-6.68826987	10269.77591	10771.37327	12578.93688	6908.504263	11601.77395	7468.562781	12236.82509	7629.988167	11572.39017
1.20_319.1150m/z	319.1150158	1.202333333	neg	Mugineic acid	70948	Unclassified	Unclassified	Unclassified			36.8	0	M-H	C12H20N2O8	1.020362823	440.7624784	661.4856344	633.8835006	866.8141988	178.7584483	762.7996582	674.9523593	578.140255	261.1410912
1.22_329.9881m/z	329.9880546	1.224233333	neg	AS-604850	45220	Unclassified	Unclassified	Unclassified			36.8	0	M+FA-H	C11H5F2NO4S	-3.099503245	1009.969736	1035.946616	1129.870609	1669.400957	1376.062652	1491.994809	1389.125874	1611.0418	1544.684117
1.36_396.0329m/z	396.0329127	1.358266667	neg	Meloxicam	HMDB0014952	Organoheterocyclic compounds	Benzothiazines	Unclassified		C08169	36.8	0	M+FA-H	C14H13N3O4S2	-0.10858143	3224.159794	2783.511497	3279.692059	3343.43754	3051.031341	2887.116342	3048.275594	3171.177955	3011.582697
1.38_323.0307m/z	323.0306915	1.380283333	neg	Uridine 5'-monophosphate	HMDB0000288	Nucleosides, nucleotides, and analogues	Pyrimidine nucleotides	Pyrimidine ribonucleotides	***	C00105	36.8	0	M-H	C9H13N2O9P	6.484913371	731.8028183	357.914898	601.794238	1059.11199	1052.825182	690.7721894	992.8949989	802.1168137	905.8353716
1.42_193.9846m/z	193.9846499	1.421683333	neg	4-Phospho-L-aspartate	360	Organic acids and derivatives	Carboxylic acids and derivatives	Amino acids, peptides, and analogues	**	C03082	36.8	0	M-H2O-H	C4H8NO7P	-6.324823948	6852.203959	6437.384619	5660.78583	4965.946369	4099.670554	3605.958995	6559.699822	5781.652748	6193.40823
1.49_253.0001m/z	253.0000943	1.492933333	neg	Stipitatonate	65709	Unclassified	Unclassified	Unclassified			36.8	0	M+FA-H	C9H4O6	5.305730017	986.8288602	1353.055905	1456.803116	1040.057239	1075.776753	970.0938909	1289.836034	1300.360667	1465.890137
1.58_369.0030m/z	369.0030102	1.577833333	neg	Sedoheptulose 1,7-bisphosphate	156	Organic oxygen compounds	Organooxygen compounds	Carbohydrates and carbohydrate conjugates	**	C00447	36.8	0	M-H	C7H16O13P2	9.926386562	864.6070404	650.2606737	613.9298142	625.8314927	610.9095283	762.904347	473.9300728	759.4679464	466.9609712
1.59_423.0402m/z	423.0402474	1.5934	neg	6-Methylthiopurine ribonucleotide	719	Unclassified	Unclassified	Unclassified			36.8	0	M+FA-H	C11H15N4O7PS	5.655543612	1688.147702	1758.979633	1723.412484	2017.91096	2154.375246	2060.129695	1665.608574	1737.391628	1777.698218
10.19_263.1654m/z	263.1653962	10.18963333	neg	beta-Sinensal	67912	Lipids and lipid-like molecules	Prenol lipids	Sesquiterpenoids	*	C09730	36.8	0	M+FA-H	C15H22O	0.586725233	748.4880616	657.10895	604.472401	739.8099951	731.6420484	732.3295067	823.4602506	788.4893389	582.3448974
10.47_501.1347m/z	501.1347126	10.46831667	neg	p-Hydroxycarvedilol sulfate	1567	Unclassified	Unclassified	Unclassified			36.8	0	M-H	C24H26N2O8S	1.995888542	4002.401474	3408.323963	3307.449892	3092.148033	4062.911997	3440.255918	3958.613712	3457.298916	3912.129521
10.53_269.2123m/z	269.2123171	10.53493333	neg	8E,10Z-Pentadecadien-1-ol	LMFA05000193	Lipids and lipid-like molecules	Fatty Acyls	Fatty alcohols			36.8	0	M+FA-H	C15H28O	0.44030745	543.748701	591.0341725	496.2485868	485.6642127	494.0438834	431.6416837	681.5131152	896.5893513	497.3194843
11.18_277.1446m/z	277.1445828	11.18458333	neg	alantolactone	LMPR0103190013	Lipids and lipid-like molecules	Prenol lipids	Terpene lactones	*	C09289	36.8	0	M+FA-H	C15H20O2	0.21538654	670.5979287	292.8942692	588.7577712	492.6551536	627.5187253	414.4541851	945.3378893	577.2433693	677.3390917
11.52_271.2280m/z	271.2279995	11.51641667	neg	13-methyltetradecanal	LMFA06000258	Lipids and lipid-like molecules	Fatty Acyls	Fatty aldehydes			36.8	0	M+FA-H	C15H30O	0.579451104	1355.513721	1225.283498	993.0552696	973.0423632	1230.295054	1118.108678	1274.519379	988.3536229	1390.607591
11.60_269.2123m/z	269.2123328	11.6033	neg	10-pentadecenal	LMFA06000084	Lipids and lipid-like molecules	Fatty Acyls	Fatty aldehydes			36.8	0	M+FA-H	C15H28O	0.510500371	1245.248363	1138.341579	1227.009229	1263.524069	1303.113956	1209.224893	1609.896616	1637.446396	1562.36758
12.34_309.1744m/z	309.1743659	12.3359	neg	8-O-Methyloblongine	HMDB0034579	Organoheterocyclic compounds	Isoquinolines and derivatives	Benzylisoquinolines			36.8	0	M-H2O-H	C20H26NO3+	2.858991315	44857.93363	22507.24296	33521.99071	19313.65716	28095.73711	20361.88837	28016.96209	43192.66767	21215.84408
14.02_269.1312m/z	269.1312364	14.02363333	neg	(Z)-Phenylacetaldehyde oxime	63615	Unclassified	Unclassified	Unclassified			36.8	0	2M-H	C8H9NO	6.237673734	2952.173209	3073.888606	3223.149102	3207.432404	3191.819486	3152.761371	3930.384921	3940.496587	3537.840796
14.91_323.2205m/z	323.2205117	14.9138	neg	Gamma-Linolenic acid	HMDB0003073	Lipids and lipid-like molecules	Fatty Acyls	Lineolic acids and derivatives	***	C06426	36.8	0	M+FA-H	C18H30O2	-8.163779223	15383.10913	14551.02443	15357.99321	9930.164648	13098.63393	12260.20795	14543.18717	13613.71439	14005.69531
2.00_360.0678n	341.0499123	2.004766667	neg	4-Methylgallic acid 3-glucuronide	HMDB0240462	Unclassified	Unclassified	Unclassified			36.8	0	M-H2O-H, M-H	C14H16O11	-4.187725208	4926.01462	4831.887208	4880.032116	5005.179568	4531.985598	4869.896075	4386.753645	5040.235683	4187.630351
2.00_453.1970m/z	453.1969755	2.004766667	neg	Butyl (S)-3-hydroxybutyrate [arabinosyl-(1->6)-glucoside]	93805	Lipids and lipid-like molecules	Fatty Acyls	Fatty acyl glycosides	*		36.8	0	M-H	C19H34O12	-1.705391395	1407.192271	1501.778742	1725.303141	311.7903791	217.6079617	135.6890678	239.189541	477.7654343	361.9223591
2.35_480.9563m/z	480.9562959	2.34905	neg	Umbelliferone sulfate	HMDB0240565	Phenylpropanoids and polyketides	Coumarins and derivatives	Unclassified			36.8	0	2M-H	C9H5O6S-	4.574235613	4818.939097	4012.659053	4092.349142	1643.378739	1859.739842	1029.972717	764.1289754	794.3685304	791.3221516
2.72_566.0071m/z	566.007088	2.72215	neg	6-Thioguanosine-5'- O- triphosphate	HMDB0060781	Nucleosides, nucleotides, and analogues	Purine nucleotides	Purine ribonucleotides	*		36.8	0	M+FA-H	C11H18N5O13P3	-4.818372022	2210.417707	2326.589642	2056.941422	2616.866164	2119.104981	2246.596647	3336.531808	3251.226201	3236.556551
2.85_359.1033m/z	359.103302	2.852583333	neg	L-Methionine sulfoximine	65992	Organic acids and derivatives	Carboxylic acids and derivatives	Amino acids, peptides, and analogues	*	C03510	36.8	0	2M-H	C5H12N2O3S	-8.74197821	1017.272007	1049.14315	1388.600682	825.1097147	558.5637042	773.6019829	1270.001192	1186.734947	1102.459827
3.22_405.0416m/z	405.041586	3.22435	neg	2,4',6,6'-Tetranitro-2',4-azoxytoluene	71193	Unclassified	Unclassified	Unclassified			36.8	0	M-H	C14H10N6O9	-5.081742472	11259.34226	11895.41327	12011.80621	759.0774158	395.4026276	369.0657511	7335.338286	7747.881749	7404.578838
3.73_322.1409m/z	322.1409108	3.730516667	neg	PC(0:0/5:0)[U]	40358	Unclassified	Unclassified	Unclassified			36.8	0	M-H2O-H	C13H28NO7P	-4.651618465	962.0769362	930.3685455	951.4906806	1718.928847	1369.474742	749.8919023	1076.619644	1036.956548	1007.250382
3.75_424.0353m/z	424.0353078	3.747833333	neg	6-Thioguanosine monophosphate	722	Nucleosides, nucleotides, and analogues	Purine nucleotides	Purine ribonucleotides	**	C16619	36.8	0	M+FA-H	C10H14N5O7PS	5.143414983	1620.32649	1176.34901	1096.849607	2585.076911	2572.75456	1497.970499	1611.087683	1407.825089	1408.798553
3.75_497.1420m/z	497.1420274	3.747833333	neg	S-(Hydroxymethyl)mycothiol	72081	Unclassified	Unclassified	Unclassified			36.8	0	M-H2O-H	C18H32N2O13S	-5.11776856	1446.925183	1581.832471	1622.729306	3442.317293	2939.830242	1959.625146	1798.169204	1933.596606	1703.987316
3.85_261.0493m/z	261.0493347	3.84605	neg	7-Carboxynalidixic acid	1412	Unclassified	Unclassified	Unclassified			36.8	0	M-H	C12H10N2O5	-9.006609056	5242.906205	5504.065523	5775.018103	6435.491263	6072.17227	6131.287364	5624.929733	6386.558839	6283.013495
3.85_421.0432m/z	421.0432452	3.84605	neg	1-O-Caffeoyl-(b-D-glucose 6-O-sulfate)	HMDB0041173	Phenylpropanoids and polyketides	Cinnamic acids and derivatives	Hydroxycinnamic acids and derivatives	*		36.8	0	M-H	C15H18O12S	-3.258815885	8913.502071	10916.12457	9354.574359	9242.039321	9764.875602	11025.53238	9120.30433	10017.46353	10402.17023
4.07_309.0560m/z	309.0559772	4.069216667	neg	dapsone hydroxylamine	HMDB0060984	Benzenoids	Benzene and substituted derivatives	Benzenesulfonyl compounds	*		36.8	0	M+FA-H	C12H12N2O3S	3.450132369	1148.956242	1396.587427	1202.868394	663.8553311	841.6422992	620.4718947	1252.551933	1431.032768	1300.03122
4.11_242.0495m/z	242.0494503	4.113883333	neg	N-(4-hydroxyphenyl)ethoxycarbothioamide	HMDB0033179	Benzenoids	Phenols	1-hydroxy-2-unsubstituted benzenoids			36.8	0	M+FA-H	C9H11NO2S	1.003935012	1708.979878	1579.559726	1625.494892	1615.194856	1288.878763	1232.861425	1788.043111	1755.712538	1610.538647
4.21_384.0713m/z	384.0713403	4.212116667	neg	6-(alpha-D-Glucosaminyl)-1D-myo-inositol 1,2-cyclic phosphate	71127	Unclassified	Unclassified	Unclassified			36.8	0	M-H2O-H	C12H22NO12P	3.025631805	1059.530644	1255.67911	1375.461608	1309.219562	1323.03346	1653.842921	1302.386666	1199.472646	1338.422914
4.30_223.0588m/z	223.0588494	4.300033333	neg	Elenaic acid	93733	Organic acids and derivatives	Carboxylic acids and derivatives	Dicarboxylic acids and derivatives			36.8	0	M-H2O-H	C11H14O6	-9.697742297	577.8143806	739.0911525	587.8400795	956.6569865	952.6029912	985.0283641	617.3009546	786.4912629	540.1960437
4.32_289.0025m/z	289.0025448	4.3224	neg	p-Coumaric acid sulfate	HMDB0125166	Phenylpropanoids and polyketides	Cinnamic acids and derivatives	Cinnamic acids	*		36.8	0	M+FA-H	C9H8O6S	0.74970122	855.3074861	646.7890799	480.7014282	245.0963802	143.1671316	253.2280549	683.3186286	640.2425581	750.8183662
4.34_268.0817m/z	268.0817055	4.344733333	neg	2,4-DPD	63077	Unclassified	Unclassified	Unclassified			36.8	0	M+FA-H	C11H13NO4	-4.282269818	786.6217733	742.0297196	811.2296022	551.1077518	415.0546419	515.0627135	384.6407828	254.15032	387.5989353
4.37_284.0778n	329.075899	4.367166667	neg	5-(3,4-Dihydroxyphenyl)-5-phenylhydantoin	1855	Organoheterocyclic compounds	Azolidines	Imidazolidines	*		36.8	0	M-H, M+FA-H	C15H12N2O4	-6.77885341	6855.384797	7148.01539	7327.03126	9265.806762	7489.609631	7999.691563	10701.2006	11585.54326	11849.12746
4.43_385.0602m/z	385.0601913	4.432683333	neg	Pyridafenthion	72534	Unclassified	Unclassified	Unclassified			36.8	0	M+FA-H	C14H17N2O4PS	-7.869854805	7652.253055	9373.691892	8236.057952	3715.407255	3820.871121	3106.272588	8885.284752	11013.25901	10516.49723
4.48_537.2684m/z	537.2684206	4.477166667	neg	Erinacine P	91458	Lipids and lipid-like molecules	Prenol lipids	Diterpenoids	*		36.8	0	M+FA-H	C27H40O8	-4.267010345	460.7028407	321.2254328	500.55796	635.126126	721.0267561	497.5089211	451.2021997	524.8371602	493.3723342
4.50_434.1403m/z	434.1403498	4.49925	neg	Hetacillin	HMDB0014877	Organoheterocyclic compounds	Lactams	Beta lactams		C11729	36.8	0	M+FA-H	C19H23N3O4S	3.134264311	414.4049852	371.5258107	662.9436209	387.0622712	274.1353014	256.7339336	395.4319647	428.9516302	427.928016
4.57_373.1403m/z	373.1403272	4.5668	neg	AMRINONE	44655	Organoheterocyclic compounds	Pyridines and derivatives	Bipyridines and oligopyridines		C13594	36.8	0	2M-H	C10H9N3O	-4.063180706	307.3663175	272.6565971	351.4482136	23.81111888	77.46689271	4.023764984	193.8309678	245.3343325	207.0953521
4.59_325.0930m/z	325.093033	4.589283333	neg	Gravolenic acid	HMDB0033884	Phenylpropanoids and polyketides	Cinnamic acids and derivatives	Hydroxycinnamic acids and derivatives			36.8	0	M+FA-H	C14H16O6	0.506651186	1290.943527	1354.434419	1314.061551	1374.490381	1293.123793	1063.789312	1418.117168	1374.930888	1774.034773
4.61_426.0423m/z	426.0423401	4.611883333	neg	Desacetycefapirin	HMDB0037269	Organoheterocyclic compounds	Lactams	Beta lactams	*		36.8	0.864	M+FA-H	C15H15N3O5S2	-3.084655187	4771.857689	4278.331268	4885.220003	8074.870833	8623.456327	6711.641414	6697.820714	4929.924978	7021.862507
4.63_310.1276m/z	310.1275851	4.632666667	neg	ANISOMYCIN	43970	Unclassified	Unclassified	Unclassified			36.8	0	M+FA-H	C14H19NO4	-7.640924682	3364.502203	2577.46341	2368.23077	2216.22241	2207.198397	1841.392952	2959.428174	2053.901758	2764.169578

4.63_661.1416m/z	661.1415541	4.632666667	neg	Palargonidin 3-(6"-malonylsophoroside)	46801	Unclassified	Unclassified	Unclassified			36.8	0	M-H2O-H	C30H32O18	0.780717136	2084.373041	2105.869845	1712.062898	2916.067181	2594.041309	2688.63635	4322.841552	3481.709476	3429.252858
4.73_307.0481m/z	307.048099	4.725416667	neg	Protocatechuic acid	HMDB0001856	Benzenoids	Benzene and substituted derivatives	Benzoic acids and derivatives	***	C00230	36.8	0	2M-H	C7H6O4	7.005664092	332.1686982	308.0004802	452.8357791	817.6670995	700.0000801	498.1244772	627.6340298	374.782261	585.0027643
4.77_301.0471n	601.0871027	4.769883333	neg	N-Acetylgalactosamine 4-sulfate	63649	Organic oxygen compounds	Organooxygen compounds	Carbohydrates and carbohydrate conjugates	*	C16265	36.8	0	M-H, 2M-H	C8H15NO9S	1.150321979	993.3672866	921.1071269	930.5634373	1515.516992	2296.232341	1742.395545	1186.786644	2184.472745	691.4512011
4.81_443.1923m/z	443.1923206	4.814166667	neg	Glycyl-Phenylalanine	HMDB0028848	Organic acids and derivatives	Carboxylic acids and derivatives	Amino acids, peptides, and analogues	*		36.8	0	2M-H	C11H14N2O3	-2.898766281	1150.6388	889.295883	1188.58084	545.6237919	407.5196015	284.2649653	592.9110041	913.4593318	673.0407369
4.95_568.1539n	613.1519297	4.94705	neg	6,8-Dimethoxy-2,3-trans-2-(4-hydroxy-2,3-dimethoxyphenyl)-9-(5-hydroxy-2-met	51478	Unclassified	Unclassified	Unclassified			36.8	0	M-H, M+FA-H	C29H28O12	-7.348269935	19562.56217	19120.49992	20318.12755	110.0209105	185.0016479	87.06264281	13336.5316	11685.01482	12065.63052
4.99_237.0747m/z	237.0746715	4.99225	neg	Propanoic acid, 2-hydroxy-3-[2-(2-propenyloxy)phenoxy]-	1685	Unclassified	Unclassified	Unclassified			36.8	0	M-H	C12H14O5	-9.137955288	1515.444631	1620.871093	1553.686565	1767.503021	1834.899094	2016.70636	2256.785835	1598.510166	1492.688298
5.18_218.1221m/z	218.1221217	5.1777	neg	Pentahomomethionine	64511	Unclassified	Unclassified	Unclassified			36.8	0	M-H	C10H21NO2S	0.448078294	548.5239232	635.6153067	559.8024838	950.5101509	966.1953062	790.2625266	575.07158	543.3588156	419.7674511
5.18_395.1330m/z	395.1330251	5.1777	neg	4-[(2-Furanylmethyl)thio]-2-pentanone	91290	Organoheterocyclic compounds	Heteroaromatic compounds	Unclassified			36.8	0	2M-H	C10H14O2S	-6.562834952	281.1202907	508.717962	260.7241253	1108.428012	832.3495757	942.8977967	1143.99678	801.7625566	942.5373292
5.18_643.1240m/z	643.1239923	5.1777	neg	L-gamma-Glutamyl-S-allylthio-L-cysteine	HMDB0038515	Organic acids and derivatives	Carboxylic acids and derivatives	Amino acids, peptides, and analogues			36.8	0	2M-H	C11H18N2O5S2	-0.246331389	5959.777887	6187.086099	6415.689917	5911.425606	6494.225056	5794.435782	6112.153877	6273.454149	5971.429667
5.22_495.1464m/z	495.1464427	5.222166667	neg	Auriculoside	47465	Lipids and lipid-like molecules	Polyketides	Flavonoids			36.8	0	M+FA-H	C22H26O10	-9.679328746	64431.21832	56071.21375	58701.58326	61200.00633	57478.61401	54409.30558	79751.22805	75965.71869	81945.16843
5.34_324.1566m/z	324.1566117	5.3394	neg	Karbutilate	72240	Unclassified	Unclassified	Unclassified			36.8	0	M+FA-H	C14H21N3O3	0.420006961	0.001403199	184.4848989	334.8193918	282.9611061	551.8865086	411.2521585	487.4363358	366.7104607	543.1453562
5.36_753.0808m/z	753.0807924	5.361783333	neg	2-Hydroxypropyl glucosinolate	93120	Organic oxygen compounds	Organooxygen compounds	Carbohydrates and carbohydrate conjugates			36.8	0	2M-H	C10H19NO10S2	-2.661240498	5241.39088	4618.175588	3927.992339	1552.003236	1979.417147	1375.94111	5075.198797	4378.444651	6359.201987
5.41_609.0885m/z	609.0885189	5.406433333	neg	EPIGALLOCATECHIN 3,5-DIGALLATE	43930	Unclassified	Unclassified	Unclassified			36.8	0	M-H	C29H22O15	-0.122388192	10258.46785	12449.11677	10753.51514	20061.4945	21260.3982	18691.0878	15738.16081	15017.32826	13498.85772
5.41_677.2406m/z	677.2406308	5.406433333	neg	Methylhydroxygliclazide	HMDB0014029	Benzenoids	Benzene and substituted derivatives	Benzenesulfonamides	*		36.8	0	2M-H	C15H21N3O4S	-3.902802518	12317.22575	11667.10085	12269.14319	12928.25914	14861.64509	14754.013	12824.97958	14091.62017	11721.28578
5.50_219.0639m/z	219.0639434	5.497816667	neg	Carbamorph	72684	Unclassified	Unclassified	Unclassified			36.8	0	M-H	C8H16N2OS2	3.702068108	4092.356186	3273.230336	3199.096934	3556.99304	4134.29809	4301.586192	3789.705401	3137.060967	3702.160173
5.57_589.1135m/z	589.1134776	5.57225	neg	Desmethylglymidine	3134	Unclassified	Unclassified	Unclassified			36.8	0	2M-H	C12H13N3O4S	-7.794522787	4814.648826	4067.243711	4887.412354	5412.6541	6233.734605	5052.885852	3864.455071	4159.078806	3936.089454
5.67_605.2223m/z	605.2223492	5.666566667	neg	Labriformidin	67235	Unclassified	Unclassified	Unclassified			36.8	0	M+FA-H	C29H36O11	-2.883861262	2990.463212	4038.600501	3818.998093	3367.01051	3439.459554	3023.672272	3121.489018	3123.096265	3641.574043
5.69_267.0849m/z	267.0849465	5.6875	neg	3-Hydroxy-4,6-heptadiyne-1-yl 1-glucoside	93573	Lipids and lipid-like molecules	Fatty Acyls	Fatty acyl glycosides	*		36.8	0	M-H2O-H	C13H18O7	-8.616578251	676.1424092	678.3934418	513.3858264	539.3059742	513.8122499	448.623126	479.8846035	551.7364298	633.3861294
5.70_282.1325m/z	282.1324533	5.701283333	neg	Gigantine	67703	Unclassified	Unclassified	Unclassified			36.8	0	M+FA-H	C13H19NO3	-9.458811236	2341.336077	2146.710551	1901.282359	1405.667392	1836.23196	1980.027163	2084.401162	1978.421522	2648.01268
5.73_677.2404m/z	677.2404005	5.727283333	neg	7-Hydroxygliclazide	HMDB0014027	Benzenoids	Benzene and substituted derivatives	Benzenesulfonamides	*		36.8	0	2M-H	C15H21N3O4S	-4.24242427	18086.48651	18567.10576	12879.10592	18226.27223	24798.56529	20845.08951	18592.95046	24939.12112	24462.3928
5.73_745.2279m/z	745.2278535	5.727283333	neg	Prasugrel	85557	Organoheterocyclic compounds	Thienopyridines	Unclassified			36.8	0	2M-H	C20H20FNO3S	7.430033464	10026.68131	11024.10012	8928.36085	11459.10086	14657.59225	12793.01694	11234.28585	14464.64602	15634.44589
5.76_507.1483m/z	507.1483421	5.7632	neg	Methylnissolin 3-O-glucoside	48124	Lipids and lipid-like molecules	Polyketides	Flavonoids			36.8	0	M+FA-H	C23H26O10	-5.318187601	3613.867756	3611.802269	3099.708075	4793.738982	5965.915304	4929.472945	4477.61176	5484.966616	4511.571214
5.76_601.1542m/z	601.1542494	5.7632	neg	Epicatechin 5-O-beta-D-glucopyranoside-3-benzoate	47285	Lipids and lipid-like molecules	Polyketides	Flavonoids			36.8	0	M+FA-H	C28H28O12	-3.649723715	5535.195654	5958.01765	6156.809362	0.001403199	0.001403199	0.001403199	0.001403199	203.3556349	0.001403199
6.01_359.0704m/z	359.0703767	6.007266667	neg	Valdecoxib	HMDB0005033	Benzenoids	Benzene and substituted derivatives	Benzenesulfonamides	*		36.8	0	M+FA-H	C16H14N2O3S	-1.081038759	5370.023317	6139.076655	5989.276792	5208.377977	5136.36687	4622.657545	4335.812652	4214.928873	5052.217596
6.04_294.1711m/z	294.1711328	6.040433333	neg	O-Desmethyltramadol	HMDB0060997	Benzenoids	Benzene and substituted derivatives	Cyclohexylphenols	*		36.8	0	M+FA-H	C15H23NO2	0.204508601	880.3847278	1142.340229	1209.438478	562.6230486	554.3398827	543.70376	422.3010381	481.3101902	501.6398175
6.12_124.0404m/z	124.0403733	6.123483333	neg	Aminohydroquinone	70196	Unclassified	Unclassified	Unclassified			36.8	0	M-H	C6H7NO2	-0.229498732	228.4068072	197.0294118	167.7934348	168.9424726	147.468694	219.395172	254.9606412	173.5233459	193.8127604
6.12_589.1130m/z	589.1130102	6.123483333	neg	Acetylsulfamethoxazole	2549	Benzenoids	Benzene and substituted derivatives	Benzenesulfonamides	*	C13061	36.8	0	2M-H	C12H13N3O4S	-8.586605959	2287.51099	2722.543909	2743.007477	3602.188173	3058.452783	2932.091541	2011.503926	2564.961632	2361.776369
6.30_591.1653m/z	591.1653091	6.302033333	neg	PtdIns-(4)-P1 (1,2-dihexanoyl)	45079	Unclassified	Unclassified	Unclassified			36.8	0	M-H2O-H	C21H40O16P2	6.541953635	9109.740675	11997.57599	10886.77069	10653.88623	10918.80722	9453.914021	10904.19994	11261.83509	13460.25332
6.32_551.2491m/z	551.2491437	6.320716667	neg	Trimethylenetetraurea	66393	Unclassified	Unclassified	Unclassified			36.8	0	2M-H	C7H16N8O4	-4.494134759	385.3098303	522.3856116	124.0687826	398.2580473	0.001403199	0.001403199	574.1566221	608.7611542	851.8063784
6.34_413.2181m/z	413.2180976	6.339983333	neg	N-Ethylglycocyamine	65849	Unclassified	Unclassified	Unclassified			36.8	0	2M-H	C8H17NO5	9.726100748	4300.379687	4508.024842	4275.181804	1015.703351	1178.255009	771.6984657	2771.451705	3418.398392	2783.838856
6.37_549.1953m/z	549.195343	6.373833333	neg	Eucommin A	68383	Unclassified	Unclassified	Unclassified			36.8	0	M-H	C27H34O12	-4.374935592	9530.106375	10516.14607	12095.54317	8134.659465	10255.17457	10365.3076	9631.834845	9698.659368	9611.389749
6.46_385.1870m/z	385.1870311	6.458366667	neg	12-hydroxyjasmonic acid 12-O-beta-D-glucoside	LMFA02020207	Lipids and lipid-like molecules	Fatty Acyls	Octadecanoids			36.8	0	M-H	C19H30O8	0.620352155	1105.998026	909.4100615	1177.054564	1237.307808	1250.433707	1405.321424	1203.0318	1420.736516	1425.923735
6.58_358.1677m/z	358.1677315	6.582416667	neg	6-O-Methylcodeine	86231	Alkaloids and derivatives	Morphinans	Unclassified	*		36.8	0	M+FA-H	C19H23NO3	5.540261039	1103.335414	1117.027507	1194.19741	726.1024855	1016.49059	1313.622334	472.1021424	764.7418669	681.7442307
6.62_671.0777m/z	671.0777425	6.61635	neg	3',5'-Cyclic diGMP	71205	Unclassified	Unclassified	Unclassified			36.8	0	M-H2O-H	C20H24N10O14P2	1.035896055	1811.611504	2081.832563	1944.554058	3067.332218	2669.135691	3258.418039	2053.528446	2243.432373	2069.939243
6.74_383.1709m/z	383.1709451	6.739933333	neg	Cotinine N-oxide	HMDB0001411	Organoheterocyclic compounds	Pyridines and derivatives	Pyrrolidinylpyridines	*		36.8	0	2M-H	C10H12N2O2	-3.992236982	902.0076369	754.8206471	1167.375613	0.001403199	0.001403199	0.001403199	213.9956774	741.579507	122.9423523
6.95_741.2700m/z	741.2700486	6.951516667	neg	Galα1-3Galβ1-4[Fucα1-3]GlcNAcβ-Sp	3655	Unclassified	Unclassified	Unclassified			36.8	0	M-H2O-H	C28H48N4O20	2.23573964	3007.896431	3664.159194	4027.318665	1346.647355	506.1151762	1037.581318	1214.328886	2356.908545	2282.340663
7.21_409.2868m/z	409.2868025	7.211683333	neg	2-Ethylethcathinone	96614	Unclassified	Unclassified	Unclassified			36.8	0	2M-H	C13H19NO	1.829102842	2336.89123	2795.747797	2600.240965	2245.728342	1919.799822	1749.445579	1544.48309	1782.00342	1301.250344
7.30_277.0329m/z	277.0329087	7.303966667	neg	cis-Coutaric acid	90083	Phenylpropanoids and polyketides	Cinnamic acids and derivatives	Hydroxycinnamic acids and derivatives			36.8	0	M-H2O-H	C13H12O8	-8.334569417	929.4106352	782.2090419	991.9813008	944.3424079	954.1416762	907.0854325	1059.662541	930.5885353	1253.567263
7.43_923.2498m/z	923.2497932	7.426916667	neg	Artomunoxanthentrione epoxide	95024	Organoheterocyclic compounds	Benzopyrans	1-benzopyrans			36.8	0	2M-H	C26H22O8	-6.346291889	10777.38255	9837.688115	12251.19708	762.078627	674.656206	581.5689624	3993.068359	5314.563437	3314.144529
7.44_709.2591m/z	709.2591241	7.440866667	neg	(5,12,13-Triaza-indeno[1,2-b]anthracen-13-yl)-acetic acid ethyl ester	69101	Unclassified	Unclassified	Unclassified			36.8	0	2M-H	C22H17N3O2	3.16353208	8491.722078	6412.555318	9205.5582	149.8651504	132.9878794	378.8054985	2419.895714	3486.608326	2006.987736
7.79_505.1493m/z	505.1492695	7.793283333	neg	Oxytetracycline	1690	Phenylpropanoids and polyketides	Tetracyclines	Unclassified	**	C06624	36.8	0	M+FA-H	C22H24N2O9	6.272538687	23437.51319	24714.84281	26281.99208	35158.5846	31388.50158	31410.94245	23770.47811	28465.58397	27783.44178
7.82_146.0248m/z	146.024808	7.82385	neg	(R)-2-Hydroxy-2H-1,4-benzoxazin-3(4H)-one	HMDB0038318	Organoheterocyclic compounds	Benzoxazines	Benzoxazinones		C15769	36.8	0	M-H2O-H	C8H7NO3	0.3395976	245.6780993	230.3277627	404.9534971	290.8422363	240.9386554	279.3236601	313.1698156	229.9172935	278.8082754
8.05_269.1390m/z	269.1390076	8.053916667	neg	11-hydroxy-4-methyl-2E,4E,6E-dodecatrienoic acid	LMFA01020413	Lipids and lipid-like molecules	Fatty Acyls	Fatty Acids and Conjugates			36.8	0	M+FA-H	C13H20O3	-1.962068935	938.1243365	1260.977706	1239.819877	1006.908339	1072.508835	765.3831846	1241.894096	1188.565526	1358.924635
8.07_531.2108m/z	531.210752	8.074883333	neg	13H-Dibenzo[a,g]fluorene	73094	Unclassified	Unclassified	Unclassified			36.8	0	2M-H	C21H14	-2.015023423	9405.662832	10599.50073	9873.730186	1247.201366	1013.802201	754.0818623	3709.560575	4635.184133	4456.680365
8.16_463.1954m/z	463.1954295	8.16495	neg	2-(4-Allyl-2,6-dimethoxyphenoxy)-1-(3,4,5-trimethoxyphenyl)-1-propanol	HMDB0030828	Lignans, neolignans and related compounds	Unclassified	Unclassified	*		36.8	0	M+FA-H	C23H30O7	-4.607089825	3100.718155	2770.037314	3311.954425	1134.298443	1068.999279	1393.763956	2233.393139	2326.523795	2228.512757
8.68_589.2998m/z	589.2998399	8.68065	neg	Boc-Phe(NMe)-Pro-Phe(NMe)-Gly-OMe	65475	Unclassified	Unclassified	Unclassified			36.8	0	M-H2O-H	C33H44N4O7	-5.455644779	35852.74721	42850.52031	41109.87357	4578.096602	4377.931323	3566.201022	18358.18043	21014.48101	21082.54846
9.32_433.0938m/z	433.0938333	9.321783333	neg	9,10-Dihydro-10-(3,4-dihydroxyphenyl)-pyrano[2,3-h]catechin-8-one	LMPK12020083	Unclassified	Unclassified	Unclassified			36.8	0	M-H2O-H	C24H20O9	2.084118336	1588.459263	1343.05708	686.667765	1493.55933	1973.029608	1436.562094	824.7654136	1290.483914	1422.318824
9.75_324.1067m/z	324.1066857	9.75225	neg	b-D-Glucopyranosiduronic acid	2024	Unclassified	Unclassified	Unclassified			36.8	0	M-H2O-H	C15H21NO8	-6.381922163	2934.672095	2731.758712	2359.333104	2662.639427	2826.918699	2902.65735	3049.088595	2877.275653	2537.23537
0.54_762.5415n	761.5338068	0.535283333	neg	PG(13:0/22:1(11Z))	78914	Lipids and lipid-like molecules	Glycerophospholipids	Glycerophosphoglycerols			36.7	0	M-H2O-H, M-H	C41H79O10P	0.478764783	2730.837398	2410.61855	3360.505756	0.001403199	477.5768857	0.001403199	2507.916435	0.001403199	158.854677
0.60_269.2125m/z	269.2124637	0.6034	neg	10Z-Pentadecenal	46482	Lipids and lipid-like molecules	Fatty Acyls	Fatty aldehydes			36.7	0	M+FA-H	C15H28O	1.093974695	283.2931043	215.4998271	294.066028	290.2778497	268.5511905	368.3104772	215.4862175	207.6193325	119.6568373
0.67_291.0288m/z	291.0287938	0.6701	neg	6-Demethylsterigmatocystin	41023	Phenylpropanoids and polyketides	Sterigmatocystins	Unclassified		C03683	36.7	0	M-H2O-H	C17H10O6	-3.557963886	829.7671014	839.6788223	655.4573038	2748.765403	2685.604552	2144.116806	1758.263528	1010.95533	1220.580865
0.76_243.0397m/z	243.0397305	0.757316667	neg	Cinoxacin	1771	Organoheterocyclic compounds	Diazanaphthalenes	Benzodiazines		C08052	36.7	0	M-H2O-H	C12H10N2O5	-5.341523902	1441.136464	1308.748547	535.2359796	4179.582178	1780.167187	4089.224787	1825.771895	878.9454346	1456.157527
0.78_519.0150m/z	519.014983	0.778533333	neg	S-Methyl-5-thio-D-ribose 1-phosphate	63428	Organic oxygen compounds	Organooxygen compounds	Carbohydrates and carbohydrate conjugates	**	C04188	36.7	0	2M-H	C6H13O7PS	-3.194806176	6528.258136	7579.838606	7415.965362	6912.872623	4783.704704	9238.531532	6600.041173	7928.060318	5669.119172
0.82_256.0594m/z	256.0593978	0.819566667	neg	HDBA	44870	Unclassified	Unclassified	Unclassified			36.7	0	M-H2O-H	C14H13NO5	-7.756141852	41757.34164	45228.69394	48759.06142	32884.05468	33116.24183	33722.09297	43105.6246	47861.66723	46341.38896
0.90_193.0718m/z	193.0718202	0.904683333	neg	(-)-Bornesitol	HMDB0031437	Organic oxygen compounds	Organooxygen compounds	Alcohols and polyols	**	C03659	36.7	0	M-H	C7H14O6	0.3010964	956.2348196	897.9517369	972.6882573	505.8246313	166.5411883	305.4993547	586.0368685	282.1172793	75.22986744
1.22_172.0447m/z	172.0446533	1.224233333	neg	2-Ethyl-4-methylthiazole	86451	Organoheterocyclic compounds	Azoles	Thiazoles	*		36.7	0	M+FA-H	C6H9NS	6.92793719	393.4813646	425.6354866	361.9610759	774.5074106	722.9019401	660.8304671	529.9594871	480.7404957	427.7487226
1.22_256.0442m/z	256.0441733	1.224233333	neg	5-(2'-Formylethyl)-4,6-dihydroxypicolinate	63555	Organic oxygen compounds	Organooxygen compounds	Carbohydrates and carbohydrate conjugates	**	C05654	36.7	0	M+FA-H	C9H9NO5	-9.959653287	773.3048288	836.3027299	836.2139758	933.2846383	925.5389532	815.4446792	932.1819678	948.5405985	970.6660651
1.22_440.0117m/z	440.0117098	1.224233333	neg	Molybdopterin	6546	Unclassified	Unclassified	Unclassified			36.7	0	M+FA-H	C10H14N5O6PS2	3.025499205	5934.393354	6238.322661	6915.83218	8464.493794	8061.454234	8868.50917	3143.047505	4668.314289	4529.37497
1.38_197.0681m/z	197.0681445	1.380283333	neg	D-Apiitol	65615	Unclassified	Unclassified	Unclassified			36.7	0	M+FA-H	C5H12O5	9.654270706	22696.80831	20305.12246	21154.96502	33121.5025	29523.97826	29211.89277	28458.20865	29186.56021	27913.22183
1.55_215.0734m/z	215.0734201	1.553483333	neg	Captopril	1474	Organic acids and derivatives	Carboxylic acids and derivatives	Amino acids, peptides, and analogues			36.7	0	M-H	C10H16O3S	-6.103326808	577.5695624	654.9806962	645.3521606	2505.442948	1727.058449	1896.986132	842.1689949	1601.367649	1043.901804
1.55_384.0152m/z	384.0152025	1.553483333	neg	(-)-Epigallocatechin sulfate	HMDB0012468	Phenylpropanoids and polyketides	Flavonoids	Sulfated flavonoids	*		36.7	0	M-H	C15H13O10S-	-1.204350493	934.5233213	917.3789524	711.9542537	1063.331597	883.6961903	1063.036289	749.4885375	914.707354	943.8523613
1.65_194.0494m/z	194.0493926	1.65405	neg	2-(N-morpholino)ethanesulfonic acid	44696	Unclassified	Unclassified	Unclassified			36.7	0	M-H	C6H13NO4S	0.718554661	1671.27016	1996.339652	1979.325454	1670.30679	1492.138839	1383.44159	1405.032971	1417.596196	1524.298416
10.07_289.0873m/z	289.08732	10.06711667	neg	(±)-Flurbiprofen	2922	Unclassified	Unclassified	Unclassified			36.7	0	M+FA-H	C15H13FO2	-3.444331843	929.8958524	761.3431509	524.0023541	734.7803615	886.4824379	1014.277818	860.5876492	664.027437	535.4526504
10.93_661.1460m/z	661.1459924	10.92908333	neg	Pelargonidin 3-(6"-malonylglucoside)-5-glucoside	46805	Unclassified	Unclassified	Unclassified			36.7	0	M-H2O-H	C30H32O18	7.306128368	6283.041511	6308.732472	6631.064308	5362.431583	6002.366861	5885.150545	6893.907187	5800.020462	6488.458901
11.08_333.1357m/z	333.13571	11.07696667	neg	4-Methylumbelliferyl heptanoate	34517	Organic oxygen compounds	Organooxygen compounds	Ethers	*		36.7	0	M+FA-H	C17H20O4	4.678356293	8427.122004	4994.561574	14629.4594	4521.787842	4950.728674	4335.951803	8140.399332	5575.917122	4148.843039
11.42_299.0957m/z	299.0956916	11.42398333	neg	D-Ribulose	312	Organic oxygen compounds	Organooxygen compounds	Carbohydrates and carbohydrate conjugates	**	C00309	36.7	0	2M-H	C5H10O5	-8.926192385	783.9557589	598.9463521	629.4682369	630.5050305	461.566306	544.1535693	997.2143995	1012.661114	585.7510324
11.63_723.4973m/z	723.4973473	11.6333	neg	PA(16:0/22:4(7Z,10Z,13Z,16Z))	82136	Lipids and lipid-like molecules	Glycerophospholipids	Glycerophosphates	*		36.7	0	M-H	C41H73O8P	0.438651782	7773.762839	7783.906415	9123.062399	5840.386877	7343.536087	6403.513806	7275.206633	8693.777632	7032.003677
12.28_699.4975m/z	699.4975069	12.28461667	neg	PA(18:1(9Z)/18:1(11Z))	59315	Lipids and lipid-like molecules	Glycerophospholipids	Glycerophosphates	*		36.7	0	M-H	C39H73O8P	0.68154417	4284.765308	5626.43245	5700.398613	4009.335595	5708.516672	3021.129531	6644.230244	7923.71781	6384.955585
12.77_699.4972m/z	699.4972162	12.77263333	neg	PA(18:1(11Z)/18:1(9Z))	HMDB0007863	Lipids and lipid-like molecules	Glycerophospholipids	Glycerophosphates	*		36.7	0	M-H	C39H73O8P	0.266534983	4379.673575	6473.920869	5561.036213	4633.110138	4994.934837	1558.574828	4839.995742	5163.447896	9897.297155
13.27_465.1018m/z	465.1018126	13.26855	neg	5,3',5'-Trihydroxy-3,6,7,8,4'-pentamethoxyflavone	LMPK12113361	Unclassified	Unclassified	Unclassified			36.7	0	M+FA-H	C20H20O10	-4.848983391	6824.451718	3092.913403	3816.201057	2935.43196	1896.014715	2431.839425	4071.309438	7995.290462	3176.077728
2.50_214.9650m/z	214.9649961	2.49675	neg	Protocatechuic acid 4-O-sulfate	HMDB0240382	Organic acids and derivatives	Organic sulfuric acids and derivatives	Arylsulfates			36.7	0	M-H2O-H	C7H6O7S	-2.505578446	679.5969635	697.6057452	662.9542861	961.5849051	632.5857572	700.8548539	782.4744762	566.2604046	572.758124
3.06_422.0660m/z	422.0660317	3.05735	neg	5'-Hydroxysulfapyridine glucuronide	2557	Unclassified	Unclassified	Unclassified			36.7	0	M-H2O-H	C17H19N3O9S	-0.74212535	5162.720507	6201.396219	6964.949524	14136.04782	13899.95666	13045.65396	10022.04231	9941.797661	9614.510942
3.36_329.0814m/z	329.0814161	3.362333333	neg	9-Hydroxybenzo[a]pyrene-4,5-oxide	70359	Benzenoids	Phenanthrenes and derivatives	Chrysenes	**	C14854	36.7	0	M+FA-H	C20H12O2	-1.817890023	3757.59257	4297.254313	3912.782724	296.9816667	235.822145	363.2628265	328.8027579	243.0271301	99.42739908
3.56_333.1071m/z	333.1070894	3.564783333	neg	Pyridoxal (Vitamin B6)	2203	Unclassified	Unclassified	Unclassified			36.7	0	2M-H	C8H9NO3	-6.346469579	193.6491506	303.170322	206.4634071	498.0090592	279.3860439	317.7990056	366.0564928	373.3246225	290.1782975
3.69_263.0398m/z	263.0397605	3.685866667	neg	(1R,2R)-3-[(1,2-Dihydro-2-hydroxy-1-naphthalenyl)thio]-2-oxopropanoic acid	70331	Unclassified	Unclassified	Unclassified			36.7	0	M-H	C13H12O4S	5.328791895	32310.44767	34376.81777	34846.85218	53792.92007	53704.666	40195.55922	41737.35429	44343.79127	45371.28198
3.75_701.1049m/z	701.1048518	3.747833333	neg	3'-O-Methyl-(-)-epicatechin-5-O-sulphate	86064	Phenylpropanoids and polyketides	Flavonoids	O-methylated flavonoids	*		36.7	0	2M-H	C16H15O7S-	6.310421049	351.3112227	827.7455259	618.4645448	2359.104145	2170.907447	1046.948157	802.4107609	1282.500885	714.5111237
3.78_324.1178m/z	324.1178374	3.779133333	neg	Asparaginyl-Phenylalanine	85653	Organic acids and derivatives	Carboxylic acids and derivatives	Amino acids, peptides, and analogues	*		36.7	0	M+FA-H	C13H17N3O4	-8.137963887	3874.216336	3540.504257	3681.771068	8162.225533	7932.630654	6348.944258	3451.530441	3839.257051	3783.023869
3.85_181.0063m/z	181.0063085	3.84605	neg	2-(4-Chlorophenoxy)propionic acid	HMDB0244931	Unclassified	Unclassified	Unclassified			36.7	0	M-H2O-H	C9H9ClO3	0.638708045	2186.911042	2383.324959	2788.04374	2750.272044	3025.981545	2882.218073	3154.420321	2602.330313	3339.538866
3.89_240.0855m/z	240.0855131	3.8906	neg	Osmaronin	88696	Lipids and lipid-like molecules	Fatty Acyls	Fatty acyl glycosides	*		36.7	0	M-H2O-H	C11H17NO6	-8.618125098	704.4642703	605.4210004	746.4946793	73.15289593	63.01687214	25.81924614	357.0688873	467.4492261	439.2264125
3.89_290.9635m/z	290.9635338	3.8906	neg	4-Mercapto-5-methyl-3(2H)-thiophenone	HMDB0039828	Organic acids and derivatives	Vinylogous thioesters	Unclassified			36.7	0	2M-H	C5H6OS2	-4.121433948	1272.637985	860.2503353	1105.719368	1334.320781	1045.05849	1345.003125	1303.914791	1193.447311	1401.218213
3.96_353.1334m/z	353.1334169	3.957583333	neg	Dinocton 6	72683	Unclassified	Unclassified	Unclassified			36.7	0	M-H	C16H22N2O7	-5.669110419	365.6556518	287.3610455	389.3905588	830.655805	774.7059659	785.5031242	308.3328056	379.0930455	329.4973149
4.00_243.0851m/z	243.0850704	4.002233333	neg	threo-Syringoylglycerol	87513	Benzenoids	Phenols	Methoxyphenols	*		36.7	0	M-H	C11H16O6	-9.592212502	2135.515892	2427.47593	2676.2218	2429.216571	2390.716235	2422.639836	1903.807615	2592.051657	2355.604306
4.05_327.0159m/z	327.0159006	4.046666667	neg	2-Naphthaleneacetic acid, 6-(sulfooxy)-	1381	Unclassified	Unclassified	Unclassified			36.7	0	M+FA-H	C12H10O6S	-7.486310094	3442.237523	3373.136293	3503.411799	2280.250992	2083.011047	1770.84939	5513.898669	6422.202618	6037.767673
4.19_399.0393m/z	399.0392745	4.189783333	neg	Cyclovariegatin	86422	Organoheterocyclic compounds	Benzofurans	Unclassified	*		36.7	0	M+FA-H	C18H10O8	9.898367412	26563.56467	25081.95217	24909.51599	15856.83509	13894.28374	17126.76937	21823.12752	20318.37966	19498.64206
4.26_283.1166m/z	283.1165587	4.2568	neg	4-Oxocyclohexanecarboxylate	66041	Unclassified	Unclassified	Unclassified			36.7	0	2M-H	C7H10O3	-7.578495927	127.7859326	282.732279	299.9201546	1178.215562	1051.089117	818.9888937	211.3432263	399.0974792	346.8091364
4.34_442.9693m/z	442.9693217	4.344733333	neg	Acetazolamide	HMDB0014957	Organoheterocyclic compounds	Azoles	Thiadiazoles		C06805	36.7	0	2M-H	C4H6N4O3S2	0.752173006	562.1698129	691.6058979	778.7018401	1394.009704	884.34085	757.4568317	573.7942806	676.6016922	456.3924938
4.37_324.1567m/z	324.1567034	4.367166667	neg	Oxamniquine	HMDB0015228	Organoheterocyclic compounds	Quinolines and derivatives	Nitroquinolines and derivatives		C07341	36.7	0	M+FA-H	C14H21N3O3	0.748685055	205.7660999	440.3913899	332.3393955	994.9602947	564.5065993	741.9812875	120.7453483	110.0778923	145.6314385
4.37_385.0109m/z	385.0108837	4.367166667	neg	Bis(4-nitrophenyl)phosphate	66043	Unclassified	Unclassified	Unclassified			36.7	0	M+FA-H	C12H9N2O8P	8.908532365	4539.607132	5282.104301	5621.334441	9086.348044	5998.97945	5996.129255	4151.636519	4109.181345	4312.256181
4.39_389.0500m/z	389.0500344	4.3893	neg	Aflatoxin GM1	87004	Phenylpropanoids and polyketides	Coumarins and derivatives	Furanocoumarins	*		36.7	0	M+FA-H	C17H12O8	-4.027905299	20961.82995	19414.01593	15933.27753	19756.24166	17670.59598	20243.24678	23490.03613	22219.5761	25057.27889
4.54_519.0197m/z	519.0196676	4.54425	neg	5-Methylthioribulose 1-phosphate	6143	Organic oxygen compounds	Organooxygen compounds	Carbonyl compounds	**	C04582	36.7	0	2M-H	C6H13O7PS	5.813669649	3107.866892	3172.487189	3222.573602	1748.684714	1721.34894	1918.981126	2391.687805	2639.1092	2612.825413
4.59_311.0211m/z	311.0210823	4.589283333	neg	2,3-Di-O-methylellagic acid	HMDB0041395	Phenylpropanoids and polyketides	Tannins	Hydrolyzable tannins			36.7	0	M-H2O-H	C16H10O8	4.108921069	783.7674288	858.1184063	957.6546417	443.7605508	523.859256	509.3940396	777.9504329	976.0579574	868.0624184
4.61_383.0962m/z	383.096166	4.611883333	neg	alpha-Hydrojuglone 4-O-b-D-glucoside	89797	Organic oxygen compounds	Organooxygen compounds	Carbohydrates and carbohydrate conjugates			36.7	0	M+FA-H	C16H18O8	-6.520033828	2723.356571	2070.343861	2237.228794	4883.933176	4477.342698	4011.138651	4759.60658	4109.654286	4690.259313
4.61_457.1556m/z	457.1556246	4.611883333	neg	a-L-Fucopyranosyl-(1->2)-b-D-galactopyranosyl-(1->2)-D-xylose	HMDB0041222	Organic oxygen compounds	Organooxygen compounds	Carbohydrates and carbohydrate conjugates			36.7	0	M-H	C17H30O14	-1.428692115	1647.57155	2061.400125	2113.087302	1973.748688	1292.938603	1108.436816	1803.131688	1691.336117	1899.654911
4.68_391.1262m/z	391.1262348	4.682483333	neg	Protionamide sulfoxide	2180	Unclassified	Unclassified	Unclassified			36.7	0	2M-H	C9H12N2OS	-1.419980122	693.8656702	930.0322167	1291.501791	1666.012376	1114.084493	1727.700803	1081.337245	1090.145744	915.3801189
4.70_273.0440m/z	273.0440175	4.703166667	neg	1-phenyl-1H-pyrazolo[3,4-d]pyrimidine-4-thiol	96301	Unclassified	Unclassified	Unclassified			36.7	0	M+FA-H	C11H8N4S	-5.054212193	2108.831878	2269.095197	2437.953753	1003.437426	923.0853471	846.9189225	1790.614399	1813.837902	1923.844244
4.70_369.1182m/z	369.1181506	4.703166667	neg	Geniposide	HMDB0252679	Unclassified	Unclassified	Unclassified			36.7	0	M-H2O-H	C17H24O10	-2.461043117	1464.445441	1803.320327	2015.341203	2212.089549	1827.654318	1579.022841	1878.052522	1973.244525	1885.211679
4.73_246.0693m/z	246.069284	4.725416667	neg	H-8	45237	Unclassified	Unclassified	Unclassified			36.7	0	M-H2O-H	C12H15N3O2S	-5.177950834	974.830376	946.2224908	799.6212896	763.1735115	954.2657882	847.1168397	883.8912943	841.5026893	898.3392005
4.75_294.1127m/z	294.1126982	4.747466667	neg	Cysteinyl-Lysine	HMDB0028780	Organic acids and derivatives	Carboxylic acids and derivatives	Amino acids, peptides, and analogues	*		36.7	0	M+FA-H	C9H19N3O3S	-0.871499368	343.7626075	188.7816587	218.3127171	565.5050443	385.1449873	367.1995725	334.3962091	282.4344673	244.3693738
4.75_561.1909m/z	561.190871	4.747466667	neg	N2-(gamma-Glutamyl)-4-carboxyphenylhydrazine	95168	Organic acids and derivatives	Carboxylic acids and derivatives	Amino acids, peptides, and analogues			36.7	0	2M-H	C12H15N3O5	-7.45954576	3306.459011	4602.43351	5208.948873	3172.710687	3385.94654	2216.345711	2710.853182	3460.339499	3159.926358
4.84_267.0851m/z	267.085131	4.836566667	neg	Gastrodin	71466	Unclassified	Unclassified	Unclassified			36.7	0	M-H2O-H	C13H18O7	-7.971794417	5345.765381	5416.17224	5927.413673	5805.395853	5755.038021	5581.887271	5094.466941	5697.674048	5014.03555
4.84_309.1324m/z	309.1324059	4.836566667	neg	Michelenolide	67758	Organoheterocyclic compounds	Pyrans	Pyranones and derivatives	*	C09506	36.7	0	M+FA-H	C15H20O4	-7.405635382	2973.368587	3172.299092	3277.064872	4926.055205	5352.987523	4968.850207	3425.865842	3631.861853	3454.045189
4.84_547.1464m/z	547.1463574	4.836566667	neg	Daidzein 7-O-glucoside-4'-O-apioside	47544	Lipids and lipid-like molecules	Polyketides	Flavonoids			36.7	0	M-H	C26H28O13	1.172867849	755.8785717	1276.000494	985.7446226	981.1986878	929.319855	981.0884166	837.5129161	1152.880369	1081.374408
4.90_273.0075m/z	273.0074756	4.902316667	neg	Isoferulic acid 3-sulfate	HMDB0041748	Phenylpropanoids and polyketides	Cinnamic acids and derivatives	Hydroxycinnamic acids and derivatives	*		36.7	0	M-H	C10H10O7S	0.103394211	2570.141675	2624.858747	2622.026523	1607.633644	1400.526144	1284.062504	1604.636118	1644.326099	1573.393937
4.90_307.0482m/z	307.0481772	4.902316667	neg	2,6-Dihydroxybenzoic acid	HMDB0013676	Benzenoids	Benzene and substituted derivatives	Benzoic acids and derivatives	***		36.7	0	2M-H	C7H6O4	7.259486507	2292.209814	2709.058822	2518.378613	2050.224778	2151.498206	2582.613261	2481.353532	2632.516003	2343.632227
4.90_553.0652m/z	553.0652484	4.902316667	neg	Azathioprine	721	Organosulfur compounds	Thioethers	Aryl thioethers	**	C06837	36.7	0	2M-H	C9H7N7O2S	-6.970511117	785.2179374	642.4551234	1043.364874	1184.449352	1348.568562	1161.131156	986.0942524	1056.715773	583.4929814
4.92_416.1453n	461.1433441	4.92485	neg	3,7-Dihydroxy-2-[4-hydroxy-3-(4-hydroxy-3-methylbutyl)phenyl]-5,6-dimethoxy-4	51269	Unclassified	Unclassified	Unclassified			36.7	0	M-H2O-H, M+FA-H	C22H24O8	-4.468787751	3365.808992	3177.82571	3152.526132	3273.730813	2964.149469	3357.409564	4165.913047	3687.824471	4065.98404
4.99_603.1701m/z	603.1700855	4.99225	neg	Physalin K	89866	Lipids and lipid-like molecules	Steroids and steroid derivatives	Physalins and derivatives	*		36.7	0	M+FA-H	C28H30O12	-3.303249927	1734.423042	2363.562935	2092.565491	688.5916868	377.5879029	645.7854976	1691.537637	1622.940647	1016.136625
5.01_274.0699m/z	274.0698989	5.014716667	neg	FENAMISAL	43838	Unclassified	Unclassified	Unclassified			36.7	0	M+FA-H	C13H11NO3	-9.591683506	945.2880645	925.4723438	992.8697648	714.411107	729.8451357	846.0127088	1225.435013	989.5032076	671.7506475
5.18_645.1695m/z	645.1694826	5.1777	neg	Leucodelphinidin 3-[galactosyl-(1->4)-glucoside]	88689	Organoheterocyclic compounds	Indoles and derivatives	Tryptamines and derivatives			36.7	0	M-H	C27H34O18	3.473934247	5281.368086	5010.526123	5308.915872	3164.711528	3093.600293	2759.885366	4464.182005	5363.762248	5561.783544
5.41_449.1099m/z	449.1099462	5.406433333	neg	cis-Resveratrol 3-O-glucuronide	HMDB0041711	Phenylpropanoids and polyketides	Stilbenes	Stilbene glycosides	*		36.7	0	M+FA-H	C20H20O9	2.502021033	2099.197721	2191.123821	2178.289777	1073.653087	1372.684676	661.5983531	1348.793491	763.7361867	801.8696762

5.43_466.1671m/z	466.1671119	5.428733333	neg	Pitavastatin	HMDB0041991	Organoheterocyclic compounds	Quinolines and derivatives	Phenylquinolines		C13334	36.7	0	M+FA-H	C25H24FNO4	-0.065087133	1683.608908	1778.815965	2131.440952	5566.256751	4732.58639	4818.587453	3240.213433	3321.206764	2634.518133
5.43_583.1132m/z	583.1131586	5.428733333	neg	S-methylazathioprine	HMDB0061068	Organoheterocyclic compounds	Imidazopyrimidines	Purines and purine derivatives	*		36.7	0	2M-H	C10H10N7O2S+	-4.968371876	9148.895227	9075.637412	9169.043382	8570.042574	9034.392203	9032.720031	1075.260512	871.5819855	571.5033474
5.53_475.0835m/z	475.0835264	5.525416667	neg	3,5,7,3',4'-Pentahydroxy-6,8-dimethoxyflavone 3-alpha-L-arabinopyranoside	51735	Unclassified	Unclassified	Unclassified			36.7	0	M-H2O-H	C22H22O13	-9.45796775	65681.34926	55115.63391	53170.56442	19568.84362	27771.31539	21946.06534	47545.70085	46602.79332	59696.56255
5.59_273.0780m/z	273.077987	5.5933	neg	3,4-Dihydro-8,9-dihydroxy-1(2H)-anthracenone	70684	Unclassified	Unclassified	Unclassified			36.7	0	M+FA-H	C14H12O3	4.99774701	1227.666842	1138.010534	1232.176115	439.4732578	495.3430925	425.7110093	796.5387313	947.9190382	780.9108863
5.61_281.1508m/z	281.1507603	5.610733333	neg	DROPROPIZINE	44155	Unclassified	Unclassified	Unclassified			36.7	0	M+FA-H	C13H20N2O2	0.336910954	2011.830386	1987.122948	2279.786299	2418.116029	2233.929849	2364.818452	1635.657464	1618.60476	1360.874238
5.68_535.1460m/z	535.1460108	5.678766667	neg	5-Hydroxy-7,8,2'-trimethoxyflavone 5-glucoside	49716	Unclassified	Unclassified	Unclassified			36.7	0	M+FA-H	C24H26O11	0.604542281	2520.804103	1993.417503	1956.289931	0.001403199	0.001403199	0.001403199	497.3871358	601.503352	598.3069394
5.76_379.0084m/z	379.0083863	5.7632	neg	6-Thioxanthine 5'-monophosphate	71265	Organoheterocyclic compounds	Diazines	Pyrimidines and pyrimidine derivatives	**	C16618	36.7	0	M-H	C10H13N4O8PS	-9.231872259	29109.45379	32106.43287	32333.51219	17990.3792	19759.71428	17917.60987	41331.1146	38248.95916	34006.10294
6.20_439.0578m/z	439.0577909	6.195716667	neg	Mirin	44842	Unclassified	Unclassified	Unclassified			36.7	0	2M-H	C10H8N2O2S	8.567625567	3772.312395	3527.292725	4113.460609	6832.478018	6137.93718	6730.739741	3959.423195	4573.406029	4818.105225
6.22_545.1206m/z	545.1205615	6.215	neg	Tolcapone	HMDB0014468	Benzenoids	Benzene and substituted derivatives	Benzophenones		C07949	36.7	0	2M-H	C14H11NO5	0.719731291	1689.971014	1338.62149	1683.460749	1564.367453	1483.594188	1487.806093	1298.803302	1543.483014	2060.684722
6.37_355.0854m/z	355.0854015	6.373833333	neg	Gulonolactone	3314	Organoheterocyclic compounds	Lactones	Gamma butyrolactones	**	C01040	36.7	0	2M-H	C6H10O6	-7.8579073	0.001403199	56.043836	63.25999083	191.2201115	67.21528352	232.5162078	128.7726716	235.866745	141.6209093
6.37_547.1395m/z	547.1394709	6.373833333	neg	m-Hydroxycarvedilol sulfate	1569	Unclassified	Unclassified	Unclassified			36.7	0	M+FA-H	C24H26N2O8S	0.560152206	1748.853386	2271.984569	2276.306601	2192.255667	2325.309952	2288.186018	1413.598247	1111.970075	1455.549385
6.39_481.1881m/z	481.1880673	6.386066667	neg	Isoaustin	HMDB0030043	Organic oxygen compounds	Organooxygen compounds	Alcohols and polyols	*		36.7	0	M-H2O-H	C27H32O9	2.550525088	15738.30983	16515.01051	18357.91345	18004.38007	17087.06524	18634.67875	14824.81927	14528.76957	13876.76998
6.44_400.2344m/z	400.2344153	6.442716667	neg	5-Hydroxybuspirone	1451	Organoheterocyclic compounds	Diazinanes	Piperazines	*		36.7	0	M-H	C21H31N5O3	-2.48764906	953.3227799	959.1141934	661.6193438	1387.893754	1257.429168	1303.520774	1253.116163	1013.44459	1038.271756
6.48_299.1865m/z	299.1865306	6.477433333	neg	Kessyl glycol	HMDB0037197	Organoheterocyclic compounds	Oxepanes	Unclassified	*		36.7	0	M+FA-H	C15H26O3	0.523396623	568.0863731	638.941401	694.1790256	801.3709049	575.2209994	577.5614824	684.1351246	653.5201048	546.0845938
6.48_404.0917m/z	404.0916866	6.477433333	neg	N-Desmethyl-O-hydroxy rosiglitazone	HMDB0060836	Benzenoids	Phenol ethers	Unclassified	*		36.7	0	M+FA-H	C17H17N3O4S	-1.373692291	3400.102852	3245.96612	4264.059592	6462.617293	5983.085949	6008.668171	4851.642081	5534.970973	5647.226711
6.52_355.1034m/z	355.10344	6.520516667	neg	Mellitoxin	67755	Unclassified	Unclassified	Unclassified			36.7	0	M+FA-H	C15H18O7	-0.050943184	592.2709414	878.8955786	687.6852873	713.1935811	674.3751604	651.0075	665.2398443	968.6565307	1070.532934
6.54_583.3130m/z	583.3130139	6.539816667	neg	Cinchoninone	71300	Unclassified	Unclassified	Unclassified			36.7	0	2M-H	C19H20N2O	8.837361609	2986.202044	3112.086446	3018.430294	2348.503602	2374.049121	2575.382858	1826.365698	2355.801859	1949.671916
6.55_356.1839n	401.1820653	6.548566667	neg	Pibutidine	HMDB0041986	Organic nitrogen compounds	Organonitrogen compounds	Amines			36.7	0	M-H, M+FA-H	C19H24N4O3	-2.780078155	2223.296921	2432.608851	2532.833789	3542.330357	3987.384328	3710.415674	1594.528613	2309.268967	1920.984486
7.09_707.0776m/z	707.077558	7.086816667	neg	Quinoline yellow	92458	Benzenoids	Indanes	Indanones			36.7	0	2M-H	C18H12NO5S	-3.392986634	3217.75567	2911.255386	3033.571279	3406.707962	2712.245161	3632.33734	2414.710253	1788.286469	2497.329922
7.25_299.1865m/z	299.1865155	7.248333333	neg	5-Acetoxydihydrotheaespirane	HMDB0040276	Organoheterocyclic compounds	Tetrahydrofurans	Unclassified	*		36.7	0	M+FA-H	C15H26O3	0.464165504	634.6534925	678.3445406	654.886902	946.5529627	955.1868922	662.4240657	675.5757995	743.6170616	732.7911124
7.29_354.1926m/z	354.1925708	7.286583333	neg	Bunazosin	1403	Unclassified	Unclassified	Unclassified			36.7	0	M-H2O-H	C19H27N5O3	-2.620022427	2195.80409	2402.444867	2216.422878	1110.922386	1134.890603	1217.012723	2370.469982	2024.86644	2379.56623
7.35_655.0336m/z	655.0335803	7.353466667	neg	Dapsone N-sulfamate	1932	Unclassified	Unclassified	Unclassified			36.7	0	2M-H	C12H12N2O5S2	5.075495712	5877.896622	5322.8034	4579.951254	3185.874524	3890.824021	3466.16377	2563.178096	3413.135204	3481.214797
7.51_384.1830m/z	384.1830312	7.50615	neg	Dextromoramide M4	1986	Unclassified	Unclassified	Unclassified			36.7	0	M+FA-H	C21H25NO3	4.082388953	1505.355312	1424.804814	1567.765776	1396.820499	1329.918838	967.5349417	622.8916215	852.3528305	923.141042
7.74_285.1263m/z	285.1263055	7.738233333	neg	Prolyl-2-naphthylamide	65952	Unclassified	Unclassified	Unclassified			36.7	0	M+FA-H	C15H16N2O	7.660294226	1263.334664	1530.263568	1307.008445	1598.066419	1848.686336	1393.327999	1253.086007	1299.024315	1426.481437
7.92_659.1835m/z	659.1834643	7.91555	neg	Sucrose octaacetate	86572	Organic oxygen compounds	Organooxygen compounds	Carbohydrates and carbohydrate conjugates	*		36.7	0	M-H2O-H	C28H38O19	0.849969715	4150.166822	4011.463597	5329.599792	6000.933065	6811.099723	6284.629935	3881.149425	6800.811514	5406.603711
8.46_296.1480m/z	296.1480059	8.455083333	neg	Rotigotine	HMDB0015615	Benzenoids	Tetralins	Unclassified			36.7	0	M-H2O-H	C19H25NOS	0.512753919	1346.877429	1852.55806	1666.903908	1931.577829	1835.608679	1974.929214	925.8953966	1434.677878	1029.221965
0.67_293.0869m/z	293.0868859	0.6701	neg	6-O-beta-D-Xylopyranosyl-D-glucose	66861	Unclassified	Unclassified	Unclassified			36.6	0	M-H2O-H	C11H20O10	-2.947162123	459.9395771	537.1703291	719.5247371	525.8372987	627.6251033	465.1188013	777.8061522	588.1027541	1069.002516
0.80_353.0899m/z	353.0898573	0.800366667	neg	Veranisatin C	87922	Lipids and lipid-like molecules	Prenol lipids	Terpene lactones	*		36.6	0	M-H2O-H	C16H20O10	5.513525287	48025.17043	48774.90889	46256.4388	32183.04792	37265.49925	36329.20148	44624.41277	46976.09704	48175.79836
1.06_350.1096m/z	350.1096084	1.055866667	neg	N-Acetyl-7-O-acetylneuraminic acid	HMDB0000785	Organic oxygen compounds	Organooxygen compounds	Carbohydrates and carbohydrate conjugates	*	C04016	36.6	0	M-H	C13H21NO10	0.965329462	88.17523072	186.1727385	293.7078591	223.7765097	285.2715174	310.9203075	252.9860991	305.4971075	229.8981745
1.18_180.0529m/z	180.052857	1.180366667	neg	4-(Hydroxymethyl)benzenediazonium(1+)	88549	Benzenoids	Benzene and substituted derivatives	Benzyl alcohols	*		36.6	0	M+FA-H	C7H7N2O+	-8.764679887	3481.38998	3545.447876	3781.304108	3487.418404	3915.44229	4013.641077	3682.457494	4192.012572	3867.188825
1.40_225.0539m/z	225.0538971	1.3958	neg	2,4-Dimethylthiazole	88873	Organoheterocyclic compounds	Azoles	Thiazoles			36.6	0	2M-H	C5H7NS	5.897051673	44.66706547	120.0115223	223.4420259	158.1602483	22.25330275	21.01636289	304.8900049	188.2731037	220.5343315
1.58_367.0451m/z	367.0451262	1.577833333	neg	STICTIC ACID	43729	Unclassified	Unclassified	Unclassified			36.6	0	M-H2O-H	C19H14O9	-2.110267073	853.676783	898.9034549	953.9706049	951.1694582	1068.16287	868.7773414	863.7949292	976.9473815	945.0275217
1.62_298.1023m/z	298.1023197	1.615883333	neg	Pyricarbate	69497	Unclassified	Unclassified	Unclassified			36.6	0	M+FA-H	C11H15N3O4	-8.45173128	1180.985406	1078.150158	1301.236825	513.3185631	613.2701809	682.9002865	917.2898341	835.3142384	852.9765154
1.84_422.0120m/z	422.011964	1.841666667	neg	5-Amino-4-hydroxy-3-(phenylazo)-2,7-naphthalenedisulfonic acid	HMDB0033381	Benzenoids	Naphthalenes	Naphthalene sulfonic acids and derivatives			36.6	0	M-H	C16H13N3O7S2	-0.594173251	17913.63658	16436.79392	20045.68712	14579.60933	14053.97144	13980.29997	24013.50111	19718.32892	21230.84485
10.83_786.5300m/z	786.5299657	10.83215	neg	PE(18:3(9Z,12Z,15Z)/18:0)	HMDB0009156	Lipids and lipid-like molecules	Glycerophospholipids	Glycerophosphoethanolamines	**	C00350	36.6	0	M+FA-H	C41H76NO8P	1.224226893	308377.4236	255566.3863	217839.6741	121413.4547	116581.6898	99450.89319	223415.0432	169975.6912	151871.2289
10.84_597.1333m/z	597.1332869	10.84081667	neg	fenbendazole	4023	Organoheterocyclic compounds	Benzimidazoles	2-benzimidazolylcarbamic acid esters	*		36.6	0	2M-H	C15H13N3O2S	-8.579853672	3491.767839	4031.024408	3297.798568	3122.957223	4016.735196	4370.133979	4144.163632	3855.812923	3729.660768
10.97_701.5128m/z	701.512774	10.96876667	neg	PA(20:0/16:1(9Z))	HMDB0115065	Lipids and lipid-like molecules	Glycerophospholipids	Glycerophosphates	*		36.6	0	M-H	C39H75O8P	0.134457108	3451.80953	2978.03161	2808.04629	4916.180853	4454.342729	4095.071433	4290.33293	4147.10101	3769.558152
12.61_248.0799m/z	248.0798733	12.61216667	neg	3-Deoxyguanosine	4149	Unclassified	Unclassified	Unclassified			36.6	0	M-H2O-H	C10H13N5O4	3.596059294	287.3885552	172.8106938	381.7302094	224.5806579	424.378996	129.9853867	337.309462	144.1175785	177.8200745
14.63_141.0207m/z	141.0207182	14.63088333	neg	D-2,3-Diketo 4-deoxy-epi-inositol	66561	Unclassified	Unclassified	Unclassified			36.6	0	M-H2O-H	C6H8O5	8.660044654	517.0032282	422.3905955	307.5962609	494.6957002	605.7679097	542.6974929	589.3525029	588.582136	495.8631915
2.36_397.0754m/z	397.0754029	2.36465	neg	Chlorogenoquinone	86232	Organic oxygen compounds	Organooxygen compounds	Alcohols and polyols	*		36.6	0	M+FA-H	C16H16O9	-6.339778617	3021.591207	3413.757794	3568.589065	0.001403199	0.001403199	0.001403199	2724.602115	3367.011848	3281.201064
3.55_321.1070m/z	321.1070045	3.549183333	neg	Binapacryl	72773	Unclassified	Unclassified	Unclassified			36.6	0	M-H	C15H18N2O6	-6.846373362	3863.79665	4005.405556	3812.525812	1951.017695	2569.979708	2226.109032	1529.756892	1856.840034	2074.008745
3.69_217.0340m/z	217.0340195	3.685866667	neg	2-Hydroxyhepta-2,4-dienedioate	63511	Unclassified	Unclassified	Unclassified			36.6	0	M+FA-H	C7H8O5	-7.886050306	303.1942766	427.7442261	296.9047734	695.2373291	616.3907552	437.4258469	504.933892	488.9414834	536.9424981
3.75_873.3475m/z	873.3475242	3.747833333	neg	Fluphenazine	2776	Organoheterocyclic compounds	Benzothiazines	Phenothiazines		C07010	36.6	0	2M-H	C22H26F3N3OS	5.792109968	16602.06413	14780.36484	15036.67577	18965.87396	19584.21697	13839.35841	11348.86831	16240.68019	13630.27829
3.94_353.0832m/z	353.0831685	3.9351	neg	(R)C(S)S-Alliin	HMDB0033592	Organic acids and derivatives	Carboxylic acids and derivatives	Amino acids, peptides, and analogues		C08265	36.6	0	2M-H	C6H11NO3S	-4.189503451	521.8064849	800.3513887	839.122374	489.1718556	526.7868717	667.7991168	395.8534985	499.4629936	740.0742585
3.98_290.0648m/z	290.0647832	3.97985	neg	Inodxyl glucuronide	61642	Unclassified	Unclassified	Unclassified			36.6	0	M-H2O-H	C14H15NO7	-7.20669524	1055.413902	889.1568544	882.9340047	982.9161502	1200.197199	1187.650015	928.0608674	1204.294582	1205.391896
4.16_456.0504m/z	456.0504181	4.15895	neg	celecoxib carboxylic	85021	Unclassified	Unclassified	Unclassified			36.6	0	M+FA-H	C17H12F3N3O4S	5.239390485	589.6695266	574.722751	492.9844958	672.4596686	491.2847846	365.8836737	1065.917085	724.3569069	661.4422617
4.19_401.0550m/z	401.0550234	4.189783333	neg	Shoyuflavone B	90052	Phenylpropanoids and polyketides	Isoflavonoids	Isoflav-2-enes			36.6	0	M-H	C19H14O10	8.961917928	8368.163321	6996.348108	7227.965328	1513.906065	1557.946177	1955.348467	8696.903817	6451.826143	7085.872121
4.37_342.1559m/z	342.1559181	4.367166667	neg	4-(2-Hydroxy-3-isopropylaminoproxy)-benzyloxy acetic acid	1356	Unclassified	Unclassified	Unclassified			36.6	0	M+FA-H	C15H23NO5	0.310932999	1412.54599	1439.438371	1294.782517	429.9194299	231.1572383	193.4561648	322.632494	261.5784264	258.8844031
4.43_268.0536n	535.1024218	4.432683333	neg	Penicillamine cysteine disulfide	1729	Unclassified	Unclassified	Unclassified			36.6	0	M-H, 2M-H	C8H16N2O4S2	-5.868827738	4413.45247	4219.8891	4552.248981	7887.070453	6712.297745	6836.254901	4318.935072	4328.212457	5091.218097
4.48_300.1359m/z	300.1358523	4.477166667	neg	Prolyl-Tryptophan	85921	Organic acids and derivatives	Carboxylic acids and derivatives	Amino acids, peptides, and analogues	*		36.6	0	M-H	C16H19N3O3	1.618165227	427.7175718	500.1375312	453.9674987	542.4168265	571.4651698	595.1368484	528.0255678	606.0779036	595.219168
4.52_299.0435m/z	299.0434533	4.521733333	neg	Mumefural	90506	Organic acids and derivatives	Carboxylic acids and derivatives	Tricarboxylic acids and derivatives			36.6	0	M-H	C12H12O9	8.657841064	1022.707305	108.5874168	4076.787209	236.4813989	163.5542961	158.6941494	194.720357	259.1430721	606.2560502
4.57_575.0973m/z	575.0973413	4.5668	neg	Furamizole	69954	Unclassified	Unclassified	Unclassified			36.6	0	2M-H	C12H8N4O5	9.857642828	1051.864339	1155.237642	832.0268074	405.2614097	270.2050424	355.2627938	860.0530891	912.542778	810.8696352
4.59_441.0443m/z	441.0442564	4.589283333	neg	Cefalotin	HMDB0014599	Organoheterocyclic compounds	Lactams	Beta lactams		C07761	36.6	0	M+FA-H	C16H16N2O6S2	2.715031014	186.6809232	0.001403199	268.9223555	282.6655127	95.05096045	0.001403199	1048.911297	1191.315159	556.2319073
4.64_230.1399m/z	230.1399431	4.641416667	neg	Isobutyryl carnitine	34492	Unclassified	Unclassified	Unclassified			36.6	0	M-H	C11H21NO4	0.697992452	789.7514129	679.495228	603.7683701	901.7908428	1065.855693	1106.670273	1092.796817	887.6619677	663.1324751
4.68_326.1607m/z	326.160721	4.682483333	neg	Benzenepropanoic acid, 4-[2-hydroxy-3-[(1-methylethyl)amino]propoxy]- (9CI) A	2577	Unclassified	Unclassified	Unclassified			36.6	0	M+FA-H	C15H23NO4	-0.675959256	557.5632648	405.3290986	499.8083944	834.4983209	676.2052837	654.4767198	531.3648407	558.6975585	492.4302725
4.73_245.0490m/z	245.0490122	4.725416667	neg	SULFAMERAZINE	44260	Benzenoids	Benzene and substituted derivatives	Benzenesulfonamides			36.6	0	M-H2O-H	C11H12N4O2S	-4.708493008	4744.248997	4749.171146	4969.684303	2444.524652	2465.462658	2501.997976	5002.663489	5951.737941	5254.649204
4.73_345.1195m/z	345.1195137	4.725416667	neg	4-Methoxybenzyl glucoside	HMDB0041405	Organic oxygen compounds	Organooxygen compounds	Carbohydrates and carbohydrate conjugates			36.6	0	M+FA-H	C14H20O7	1.359093026	601.6829684	604.2457639	958.7232693	1168.19505	727.0535279	1147.003209	872.2694585	789.4699391	535.9203624
4.75_405.0757m/z	405.0756877	4.747466667	neg	HPF	64737	Unclassified	Unclassified	Unclassified			36.6	0	M-H2O-H	C26H16O6	-2.733873398	30590.83131	32306.39892	33075.70937	29025.91499	29900.34245	28133.94952	30354.58244	31897.21461	34080.55678
4.75_419.0958m/z	419.0958144	4.747466667	neg	Pebrellin	49908	Phenylpropanoids and polyketides	Flavonoids	O-methylated flavonoids	*		36.6	0	M+FA-H	C19H18O8	-6.832474347	2242.988918	3024.626851	2183.363096	4493.82032	4613.990017	4533.195115	3639.112839	4933.489164	4037.758459
4.75_541.0506m/z	541.0505763	4.747466667	neg	3,5-Di-O-galloyl-1,4-galactarolactone	92171	Organic acids and derivatives	Carboxylic acids and derivatives	Tetracarboxylic acids and derivatives			36.6	0	M+FA-H	C20H16O15	6.962232986	14948.04569	14252.6418	16018.74102	13702.51903	17343.77086	14734.64083	15804.02328	15797.74371	15506.3322
4.79_476.0346m/z	476.0346259	4.792316667	neg	Bensultap	HMDB0248963	Unclassified	Unclassified	Unclassified			36.6	0	M+FA-H	C17H21NO4S4	2.507721443	3220.589559	3267.579164	3941.119179	5803.536317	5905.520078	5739.634118	3331.337937	3295.146362	3069.664543
4.81_521.1671m/z	521.1671104	4.814166667	neg	Oleuropein	67938	Lipids and lipid-like molecules	Prenol lipids	Terpene glycosides	*		36.6	0	M-H2O-H	C25H32O13	1.222623433	1852.256744	1736.726646	1679.354037	218.8852308	0.001403199	0.001403199	910.4350981	993.6156869	256.8053445
4.86_260.0671m/z	260.0670641	4.858383333	neg	NS-102	69665	Unclassified	Unclassified	Unclassified			36.6	0	M-H	C12H11N3O4	-2.356936959	168.6416286	355.2802072	343.7948376	226.214144	102.5234908	187.0449805	278.7019181	333.4134064	462.7462477
4.86_575.1756m/z	575.1755674	4.858383333	neg	Curcumin monoglucoside	64199	Unclassified	Unclassified	Unclassified			36.6	0	M+FA-H	C27H30O11	-2.729805768	906.8704445	1155.421967	1353.699276	0.001403199	0.001403199	0.001403199	774.1616011	959.4122417	373.1581541
4.95_387.1122m/z	387.1121919	4.94705	neg	Piperonyl acetate	88566	Organoheterocyclic compounds	Benzodioxoles	Unclassified	*		36.6	0	2M-H	C10H10O4	9.406340062	1012.212441	956.84502	978.929273	256.3301546	113.3089339	207.97123	997.8543115	847.7090739	576.3111239
4.99_330.1196m/z	330.1195534	4.99225	neg	N'-Hydroxyneosaxitoxin	89462	Phenylpropanoids and polyketides	Saxitoxins, gonyautoxins, and derivatives	Unclassified			36.6	0	M-H	C10H17N7O6	8.45151891	611.5255467	535.2317402	657.2842123	594.3266188	702.9444451	708.1183738	1290.894288	739.9485863	858.8137578
5.01_270.0597m/z	270.0597323	5.014716667	neg	N-Succinyl-2-amino-6-ketopimelate	62901	Organic acids and derivatives	Carboxylic acids and derivatives	Amino acids, peptides, and analogues	**	C04462	36.6	0	M-H2O-H	C11H15NO8	-7.5860716	232.0081906	257.5633813	247.0039801	372.2550353	410.1244191	376.6845585	315.4708181	337.4819889	294.0846167
5.06_249.0130m/z	249.0129859	5.05975	neg	Blue pigment	63889	Unclassified	Unclassified	Unclassified			36.6	0	M-H	C10H6N2O6	-9.293440837	744.4182848	826.9863338	894.2912324	1410.419629	1266.421843	1372.302827	965.1044187	969.4849989	1494.674094
5.13_313.0811m/z	313.081062	5.125666667	neg	2,3-Dihydroxycarbamazepine	71258	Organoheterocyclic compounds	Benzazepines	Dibenzazepines	**	C16603	36.6	0	M+FA-H	C15H12N2O3	-7.210892185	916.4295537	802.9021961	884.8294856	719.1793951	871.5036879	810.1081401	964.6003667	842.5659115	1117.52712
5.13_635.1164m/z	635.1164496	5.125666667	neg	Melanin	7011	Benzenoids	Anthracenes	Unclassified	**	C05606	36.6	0	2M-H	C18H10N2O4	-6.897338274	1036.10875	965.5667639	1203.282238	980.1097122	1196.23755	843.5751344	1152.550734	1039.220098	1462.241587
5.24_443.1748m/z	443.1747919	5.244033333	neg	5'-(3'-methoxy-4'-hydroxyphenyl)-gamma-valerolactone	86105	Benzenoids	Phenols	Methoxyphenols	*		36.6	0	2M-H	C12H14O4	8.218563669	6917.067132	6491.622556	5673.975455	9959.651925	10591.28377	11227.34555	6434.963223	5740.140924	6956.601124
5.26_453.1385m/z	453.1384881	5.263033333	neg	Marmesin galactoside	HMDB0041508	Phenylpropanoids and polyketides	Coumarins and derivatives	Furanocoumarins			36.6	0	M+FA-H	C20H24O9	-4.280757714	6917.940447	7724.091776	8301.975032	13855.93465	15278.39274	13881.91375	10070.70371	9506.658456	9538.524711
5.53_701.2061m/z	701.2060957	5.525416667	neg	Ramontoside	93541	Lignans, neolignans and related compounds	Lignan glycosides	Unclassified			36.6	0	M-H	C34H38O16	-3.721056735	4688.663075	4716.478098	4365.721983	211.1556792	398.518624	166.831765	2119.195676	3044.583041	2924.181932
5.55_447.2239m/z	447.2238666	5.547916667	neg	Neryl arabinofuranosyl-glucoside	HMDB0029348	Lipids and lipid-like molecules	Prenol lipids	Terpene glycosides	*		36.6	0	M-H	C21H36O10	0.659585918	1302.888253	963.5460967	927.8200396	1245.28467	1506.448971	1108.860592	1153.936496	1286.072258	1362.734859
5.56_327.1087m/z	327.1086649	5.56185	neg	Dihydromelilotoside	HMDB0038334	Organic oxygen compounds	Organooxygen compounds	Carbohydrates and carbohydrate conjugates			36.6	0	M-H	C15H20O8	0.377139126	927.0895492	668.5567469	626.1428197	502.8882701	523.2111032	653.336947	774.5089021	781.7219849	782.764403
5.56_541.2657m/z	541.2657411	5.56185	neg	Chaetoglobosin N	88035	Alkaloids and derivatives	Cytochalasans	Chaetoglobosins	*		36.6	0	M-H	C33H38N2O5	-9.321353293	569820.9977	478902.7138	499434.9675	677892.1575	802851.388	750869.0974	504422.3146	520719.9105	420153.2547
5.56_609.2533m/z	609.2533235	5.56185	neg	Resiniferatoxin	67492	Unclassified	Unclassified	Unclassified			36.6	0	M-H2O-H	C37H40O9	6.258168818	160730.2351	133508.4979	140663.0045	196660.9574	233375.4722	208211.8747	141801.4969	150633.06	119089.1841
5.56_949.1902m/z	949.1901695	5.56185	neg	Chondroitin 6-sulfate	5570	Organic oxygen compounds	Organooxygen compounds	Carbohydrates and carbohydrate conjugates	*	C00635	36.6	0	2M-H	C15H25NO14S	-1.794366153	10820.05169	9836.368681	9555.707732	14405.98593	17152.51705	15283.92227	8670.090808	10383.64901	8582.600886
5.61_247.0589m/z	247.0589182	5.610733333	neg	Propanoic acid, 2-hydroxy-3-[(4-hydroxy-1-naphthalenyl)oxy]-	2175	Unclassified	Unclassified	Unclassified			36.6	0	M-H	C13H12O5	-9.186525246	2805.827412	2671.17833	3249.443451	2988.818145	2737.247758	2786.635668	2976.272327	2968.621276	2713.535587
5.61_305.1245m/z	305.1244677	5.610733333	neg	Cardiogenol C	64802	Unclassified	Unclassified	Unclassified			36.6	0	M+FA-H	C13H16N4O2	-4.07848748	928.737901	664.2096354	976.9511545	1213.463167	887.6270659	1136.611766	780.0959121	901.6692371	786.691277
5.61_547.2164m/z	547.2164008	5.610733333	neg	Verrucarin A	67920	Unclassified	Unclassified	Unclassified			36.6	0	M+FA-H	C27H34O9	-4.150953761	2902.976209	2494.575455	2789.060905	4203.949558	3676.297481	3563.5168	3025.872538	2747.460944	2947.796278
5.67_360.1851m/z	360.1851445	5.666566667	neg	Phalaenopsine T	68276	Unclassified	Unclassified	Unclassified			36.6	0	M-H	C20H27NO5	9.684487701	745.6492695	755.4921575	603.8285833	933.7925132	826.8219773	855.8848865	460.4287666	726.8470904	660.9057258
5.81_457.1703m/z	457.1702707	5.806016667	neg	3-Hydroxychavicol 1-[rhamnosyl-(1->6)-glucoside]	HMDB0037108	Organic oxygen compounds	Organooxygen compounds	Carbohydrates and carbohydrate conjugates			36.6	0	M-H	C21H30O11	-2.760088018	2667.699367	2767.689623	2775.02608	5462.223559	4920.232501	5664.119063	1885.671082	2736.117493	2922.887219
5.81_657.0618m/z	657.0617593	5.806016667	neg	NAc-DNP-Cys	69126	Unclassified	Unclassified	Unclassified			36.6	0	2M-H	C11H11N3O7S	8.349226615	1489.99822	2346.259149	1456.428986	6999.321666	5886.199397	5652.309769	4064.702052	3142.514046	3414.265865
5.85_523.1776m/z	523.1776149	5.84575	neg	Deoxyloganin tetraacetate	41164	Lipids and lipid-like molecules	Prenol lipids	Isoprenoids		C11646	36.6	0	M-H2O-H	C25H34O13	-8.272141498	57656.00122	59099.10845	66420.56791	53532.46249	53472.08122	52899.8192	83018.47582	78610.56787	83015.30886
5.92_743.0803m/z	743.0802511	5.9168	neg	3-butenylglucosinolate	HMDB0304115	Organic oxygen compounds	Organooxygen compounds	Carbohydrates and carbohydrate conjugates		C08415	36.6	0	2M-H	C11H18NO9S2-	3.939417715	2562.740611	2584.434285	2757.905685	2049.361621	1867.681542	2776.227747	312.5716538	426.0706329	768.5390157
5.94_503.2502m/z	503.2501511	5.937716667	neg	[6]-Gingerdiol 5-O-beta-D-glucopyranoside	HMDB0036123	Lipids and lipid-like molecules	Fatty Acyls	Fatty acyl glycosides	*		36.6	0	M+FA-H	C23H38O9	0.797429669	1926.139404	1953.145985	2496.984804	1346.048803	1805.103071	2020.379006	1692.132915	2300.088824	2371.880162
6.03_479.1537m/z	479.1537331	6.028183333	neg	CHRYSAROBIN	43940	Unclassified	Unclassified	Unclassified			36.6	0	2M-H	C15H12O3	7.749643194	3078.440864	3981.348199	3389.708239	3682.624182	4170.610881	4242.65795	2476.297027	2998.330609	2718.033228
6.09_245.0796m/z	245.0796294	6.087383333	neg	Dihydroclavaminic acid	64563	Unclassified	Unclassified	Unclassified			36.6	0	M+FA-H	C8H12N2O4	8.595054741	5458.78143	6373.282993	6533.882558	8534.524406	9938.704394	9033.906892	6894.419307	7025.812809	7352.636944
6.44_302.2340m/z	302.2339538	6.442716667	neg	D-erythro-Sphingosine C-15	64626	Unclassified	Unclassified	Unclassified			36.6	0	M+FA-H	C15H31NO2	1.056079432	256.8108586	365.8233525	164.7704701	377.542126	400.3054716	342.5252027	421.562753	388.325737	316.1156654
6.51_437.1796m/z	437.179603	6.509966667	neg	Viguiestenin	67826	Unclassified	Unclassified	Unclassified			36.6	0	M+FA-H	C21H28O7	-5.362426406	4852.214394	5111.561204	5517.394377	5513.136405	5423.220964	5236.064815	3979.802509	5283.887942	5316.767862
6.54_743.3503m/z	743.3502649	6.539816667	neg	Corchorusoside A	HMDB0032823	Lipids and lipid-like molecules	Steroids and steroid derivatives	Steroid lactones	*		36.6	0	M+FA-H	C35H54O14	1.010390633	1530.992332	1634.575298	1694.757998	326.0779319	160.8598345	549.6524693	437.1276587	914.2560123	666.0342066
6.57_439.0577m/z	439.0577241	6.570233333	neg	AG-123	45249	Unclassified	Unclassified	Unclassified			36.6	0	2M-H	C10H8N2O2S	8.415964392	2910.813227	3162.674124	2837.3094	2709.416243	2317.921045	2676.410411	1650.294698	2254.196915	1570.241089
6.63_681.1053m/z	681.1052674	6.63275	neg	ARISTOLOCHIC ACID	44093	Unclassified	Unclassified	Unclassified			36.6	0	2M-H	C17H11NO7	7.975977771	8455.199468	8546.273833	7125.758095	4891.975647	5078.873469	5511.786117	0.001403199	0.001403199	0.001403199
6.84_581.2978m/z	581.297798	6.842166667	neg	Coroglaucigenin-3-o-alpha-L-rhamnopyranoside	71756	Unclassified	Unclassified	Unclassified			36.6	0	M+FA-H	C29H44O9	1.980488014	1621.050187	1843.508592	1785.319038	104.4311483	0.001403199	116.6884494	645.8784377	635.0078231	784.6598419
6.85_384.1102m/z	384.1101632	6.851566667	neg	1-Hydroxyrutacridone epoxide	HMDB0033520	Organoheterocyclic compounds	Quinolines and derivatives	Benzoquinolines			36.6	0	M+FA-H	C19H17NO5	3.797242939	6021.757262	6253.98084	5466.708842	5249.937749	6117.089312	5751.505086	6875.371646	7119.612302	5959.050543
6.85_417.2136m/z	417.2136017	6.851566667	neg	1-dimethylarsinoyl-(3Z,6Z,9Z,12Z,15Z,18Z)-docosahexaene	LMFA11000717	Lipids and lipid-like molecules	Fatty Acyls	Hydrocarbons			36.6	0	M-H	C24H39AsO	-1.935008393	1622.663228	1662.233409	1185.91136	2114.636601	2562.202282	2064.758319	1425.57444	1715.37674	1524.39014
6.95_454.2083m/z	454.208307	6.951516667	neg	Meptazinol glucuronide	1141	Unclassified	Unclassified	Unclassified			36.6	0	M+FA-H	C21H31NO7	0.126644554	3031.938373	4776.994624	2381.297566	2289.40411	1827.575636	2707.049215	1505.504545	1774.636612	1236.018799
7.30_413.2184m/z	413.2183696	7.29525	neg	Δ17-6-keto PGF1α	36198	Unclassified	Unclassified	Unclassified			36.6	0	M+FA-H	C20H32O6	0.755000103	21630.66961	24314.3881	23681.27762	11441.67558	10648.2774	10917.49409	16677.03695	19092.54962	18899.83938
7.36_749.3351m/z	749.3350501	7.36395	neg	25-Acetyl-6,7-didehydrofevicordin F 3-glucoside	91482	Lipids and lipid-like molecules	Steroids and steroid derivatives	Steroidal glycosides	*		36.6	0	M+FA-H	C37H52O13	-5.600211545	7367.498713	9848.806557	8766.495471	7873.724996	7333.068069	6850.541633	7534.217461	8340.863863	8906.980375
7.51_471.1014m/z	471.1014146	7.50615	neg	L-2-Aminoadipate adenylate	HMDB0006941	Nucleosides, nucleotides, and analogues	Purine nucleotides	Purine ribonucleotides	**	C05560	36.6	0	M-H2O-H	C16H23N6O10P	-4.227330944	2456.307513	2566.553525	2543.566009	2269.000304	2479.372533	2255.642008	2430.231144	2591.215268	2735.109326
7.77_805.1874m/z	805.1873526	7.770583333	neg	3-Methylpentyl glucosinolate	HMDB0036355	Organic oxygen compounds	Organooxygen compounds	Carbohydrates and carbohydrate conjugates			36.6	0	2M-H	C13H25NO9S2	0.598151715	5800.976532	6342.094581	7118.558179	10562.97948	10276.04938	9835.681698	6089.151395	6537.408449	7313.655812
7.88_493.2812m/z	493.281205	7.883383333	neg	Ethyl-(5S,12S)-diacetoxy-(6E,8E,10E,14Z)-eicosatetraenoate	LMFA03000017	Lipids and lipid-like molecules	Fatty Acyls	Eicosanoids			36.6	0	M+FA-H	C26H40O6	1.144647977	1014.69061	1067.964222	1041.677201	1468.85113	1435.661318	1428.2503	878.7323933	1065.22562	866.6582522
8.29_647.2279m/z	647.2279259	8.294766667	neg	PtdIns-(4)-P1 (1,2-dioctanoyl)	45076	Unclassified	Unclassified	Unclassified			36.6	0	M-H2O-H	C25H48O16P2	6.01628535	9684.039679	10329.40051	12312.10651	10282.16075	8306.019284	8752.997755	9138.65287	10938.39403	9433.035468
8.33_607.3491m/z	607.3490934	8.3263	neg	Ponasteroside A	89710	Lipids and lipid-like molecules	Steroids and steroid derivatives	Steroidal glycosides	*		36.6	0	M-H2O-H	C33H54O11	0.514072868	2125.676526	2224.136999	1543.471739	3002.392383	2432.371181	1742.068154	1122.62216	2835.066885	2257.462917
9.28_309.1710m/z	309.1710223	9.278033333	neg	5-Hydroxy-1-(4-hydroxyphenyl)-3-decanone	64192	Unclassified	Unclassified	Unclassified			36.6	0	M+FA-H	C16H24O3	1.040239267	5168.683284	4992.07507	4499.330063	5927.341197	4837.481591	4790.358977	5271.300303	5053.522988	4891.435584
0.67_399.1011m/z	399.1010794	0.6701	neg	S-Acetylphosphopantetheine	66032	Unclassified	Unclassified	Unclassified			36.5	0	M-H	C13H25N2O8PS	3.580161491	422.6648755	599.1793353	1142.103387	974.6619059	2516.666338	1702.877252	1566.521572	1926.914181	2333.168064
0.69_349.0607m/z	349.0606846	0.692166667	neg	Idebenone Metabolite (Benzenebutanoic acid, 2-hydroxy-3,4-dimethoxy-6-meth	768	Unclassified	Unclassified	Unclassified			36.5	0	M-H	C13H18O9S	2.307808092	1451.764929	1145.362103	1656.613849	4337.314463	4905.463549	4732.742267	2673.834973	3072.703758	3556.876731
0.76_297.1070m/z	297.106956	0.757316667	neg	7-Methylguanosine	HMDB0001107	Nucleosides, nucleotides, and analogues	Purine nucleosides	Unclassified	*		36.5	0	M-H	C11H16N5O5+	-3.056446456	143.6143004	93.67258382	192.914939	0.001403199	194.9272689	0.001403199	161.802207	179.5096288	197.6127213
0.76_326.1328n	307.1150065	0.757316667	neg	2,2,4-Trimethyl-3-(4-fluorophenyl)-2H-1-benzopyran-7-ol acetate	70550	Unclassified	Unclassified	Unclassified			36.5	0	M-H2O-H, M-H	C20H19FO3	3.142795136	3205.722635	2791.08681	3621.261145	2487.812063	1836.93798	4370.953043	2296.103654	3025.849846	1712.286605

0.78_287.0193m/z	287.0192789	0.778533333	neg	2-Protocatechoylphloroglucinolcarboxylate	66200	Phenylpropanoids and polyketides	Depsides and depsidones	Unclassified		C04524	36.5	0	M-H2O-H	C14H10O8	-1.461510941	1159.426082	2013.297036	2021.890617	5646.699501	3267.533171	4852.315906	2117.719717	2889.193874	1507.143039
0.82_406.0661m/z	406.066066	0.819566667	neg	5,12-Dihydroxanthommatin	65984	Unclassified	Unclassified	Unclassified			36.5	0	M-H2O-H	C20H15N3O8	-4.722083636	55665.43246	65752.80813	64188.4188	107371.0108	95532.04905	95677.61831	63346.29899	69874.18887	70796.98337
0.86_328.0621m/z	328.0620607	0.86335	neg	Hydroxysanguinarine	69373	Unclassified	Unclassified	Unclassified			36.5	0	M-H2O-H	C20H13NO5	1.524926857	2561.431379	2777.563896	2784.273021	1707.478536	2680.443422	2501.113994	3484.06591	2898.146941	3510.487112
0.88_147.0493m/z	147.0493269	0.884633333	neg	Methyl 4-(methylthio)butyrate	92461	Lipids and lipid-like molecules	Fatty Acyls	Fatty acid esters	*		36.5	0	M-H	C6H12O2S	5.421587961	80.6546585	58.70840953	176.3529952	57.71507911	0.001403199	0.001403199	81.22407002	95.13918686	188.4709643
0.97_238.9942m/z	238.9941669	0.967616667	neg	6-Phospho-g-gluconolactone	375	Unclassified	Unclassified	Unclassified			36.5	0	M-H2O-H	C6H11O9P	-7.986233884	489.9399455	347.129483	162.552738	209.4135322	527.2760109	207.905851	330.3884051	86.5892334	118.9466907
1.18_129.0388m/z	129.0387591	1.180366667	neg	Methylthiomethyl butyrate	93023	Lipids and lipid-like molecules	Fatty Acyls	Fatty acid esters	*		36.5	0	M-H2O-H	C6H12O2S	5.400559536	2719.349808	2997.58739	2989.366678	2425.323806	2812.336616	2961.007808	2962.410606	2957.229265	3406.217596
1.20_259.9865m/z	259.9864673	1.202333333	neg	2,4-Dihydroxy-1,4-benzoxazin-3-one sulfate	HMDB0240443	Organoheterocyclic compounds	Benzoxazines	Benzoxazinones			36.5	0	M-H	C8H7NO7S	-2.217667927	668.7085305	564.0903686	566.659714	723.602417	267.9806631	651.0575007	683.4182123	512.5591302	303.2762702
1.25_474.0330m/z	474.0330479	1.246333333	neg	Gonyautoxin 8	73505	Unclassified	Unclassified	Unclassified			36.5	0	M-H	C10H17N7O11S2	-5.098554106	652.3613568	593.2444008	617.0753935	853.5159329	1249.831047	1017.69279	780.8841276	713.6498058	639.6531976
1.49_215.0539m/z	215.0539153	1.492933333	neg	1-Hydroxyhexane-1,2,6-tricarboxylate	71256	Unclassified	Unclassified	Unclassified			36.5	0	M-H2O-H	C9H14O7	-9.38335953	4757.580517	4854.2943	5182.198844	4075.140525	3585.370762	3678.39772	4937.65345	4372.506731	4769.564914
1.72_250.0275m/z	250.0275186	1.721216667	neg	5-Hydroxymethylsulfamethoxazole	2550	Unclassified	Unclassified	Unclassified			36.5	0	M-H2O-H	C10H11N3O4S	-6.19643689	2294.117619	2219.956329	1935.728602	2701.544669	2718.970831	2514.255842	2460.856029	2335.615371	2638.833911
1.74_309.1127m/z	309.112727	1.736816667	neg	CAY10587	45468	Unclassified	Unclassified	Unclassified			36.5	0	M+FA-H	C18H16O2	-1.914557612	2377.354305	2141.554125	1871.475597	1994.951958	2145.960515	1981.152299	1681.651149	2009.584094	1700.10774
1.84_286.0369m/z	286.0368555	1.841666667	neg	Vamidothion	72451	Unclassified	Unclassified	Unclassified			36.5	0	M-H	C8H18NO4PS2	9.215566418	47570.3675	46555.1789	58620.86111	39575.12796	40419.04159	41285.99734	66675.28681	57844.54941	57795.31664
11.60_859.6891m/z	859.6891406	11.6033	neg	4alpha-carboxy-5alpha-cholesta-8-en-3beta-ol	57667	Lipids and lipid-like molecules	Steroids and steroid derivatives	Cholestane steroids	*		36.5	0	2M-H	C28H46O3	8.163646858	1076.340828	1365.289186	1267.906585	1369.145184	651.4962494	0.001403199	2171.255947	691.3079681	1219.071096
13.43_141.0207m/z	141.0206945	13.42951667	neg	2-Hydroxymuconate semialdehyde	65525	Unclassified	Unclassified	Unclassified			36.5	0	M-H	C6H6O4	9.591817793	195.3949717	86.59644956	162.7107559	155.4757599	172.5637052	125.8953934	328.3613645	233.9263466	181.7597432
13.64_678.5295m/z	678.5294738	13.64228333	neg	N-[(4E,8Z)-1,3-dihydroxyoctadeca-4,8-dien-2-yl]hexadecanamide 1-glucoside	HMDB0034623	Lipids and lipid-like molecules	Sphingolipids	Glycosphingolipids	*		36.5	0	M-H2O-H	C40H75NO8	-2.800371164	2140.37615	330.7133516	735.2093487	1323.353597	4174.673298	2353.042829	1033.091118	828.5073307	1977.213901
2.30_298.9536m/z	298.9535784	2.303733333	neg	catechol disulfonate	1832	Unclassified	Unclassified	Unclassified			36.5	0	M+FA-H	C6H6O7S2	-0.468698349	9792.76226	10119.67189	10929.36271	1017.991335	986.5895247	945.3996885	7155.848702	7153.326975	7219.334302
3.73_285.0957m/z	285.0957234	3.730516667	neg	Imicyafos	72275	Unclassified	Unclassified	Unclassified			36.5	0	M-H2O-H	C11H21N4O2PS	4.212022907	2239.407898	1995.412488	2160.854073	2641.385691	2546.759094	1679.451718	1804.549883	1751.614195	1939.049154
3.75_365.2084m/z	365.208423	3.747833333	neg	4-Butyl-5-propylthiazole	HMDB0040067	Organoheterocyclic compounds	Azoles	Thiazoles			36.5	0	2M-H	C10H17NS	-1.752111407	172.963435	65.63088853	181.0764978	273.898341	252.1747987	215.88267	172.0960469	223.4163492	211.0797921
4.11_490.9857m/z	490.9856937	4.113883333	neg	THIOMETON	HMDB0259016	Unclassified	Unclassified	Unclassified			36.5	0	2M-H	C6H15O2PS3	-2.822413403	877.2660297	1140.208003	1109.006065	896.7420854	1207.298523	1331.071143	4906.223404	5426.342388	4346.917003
4.16_333.0081m/z	333.0081155	4.15895	neg	Naringenin 7-sulfate	HMDB0240547	Phenylpropanoids and polyketides	Flavonoids	Flavans			36.5	0	M-H2O-H	C15H12O8S	1.898298841	585.832993	829.1075787	886.2593333	2307.16663	1812.057444	1961.909721	280.0960062	203.405682	169.5123205
4.18_326.1612m/z	326.1611635	4.181333333	neg	Cycloheximide	44124	Unclassified	Unclassified	Unclassified			36.5	0	M+FA-H	C15H23NO4	0.897664691	446.4721496	364.2523195	485.4106711	338.6332324	258.5487099	432.0071553	413.1920939	254.9126892	332.8915924
4.21_316.0842m/z	316.0841601	4.212116667	neg	Mefenamic acid Metabolite (Benzoic acid, 3-[(2-carboxyphenyl)amino]-2-methyl-	1100	Unclassified	Unclassified	Unclassified			36.5	0	M+FA-H	C15H13NO4	5.530756503	2981.264248	3215.93828	3073.999928	3555.246568	2999.220784	3637.581698	3344.944806	3245.767698	3504.89619
4.34_529.0318m/z	529.0317507	4.344733333	neg	UDP-4-dehydro-6-deoxy-D-glucose	62919	Nucleosides, nucleotides, and analogues	Pyrimidine nucleotides	Pyrimidine nucleotide sugars	**	C04089	36.5	0	M-H2O-H	C15H22N2O16P2	9.371329864	630.2819689	568.5990086	791.9054206	1287.697749	973.6924064	939.6244314	601.9453652	1507.222649	945.2291226
4.37_424.0983n	405.0804244	4.367166667	neg	3-Glucosyl-2,3',4,4',6-pentahydroxybenzophenone	88586	Organic oxygen compounds	Organooxygen compounds	Carbohydrates and carbohydrate conjugates	*		36.5	0	M-H2O-H, M-H	C19H20O11	-5.413688815	5816.849238	5432.684583	6024.62366	3346.551062	2509.788322	2350.67648	8102.84472	8067.995925	8946.295464
4.39_685.1720m/z	685.1719994	4.3893	neg	Chartreusin	67494	Unclassified	Unclassified	Unclassified			36.5	0	M+FA-H	C32H32O14	-8.449443699	34432.22181	38724.51995	37186.70909	24144.13239	20156.80302	22417.04285	40454.52517	36944.5603	40257.55384
4.45_261.0439m/z	261.0439388	4.454616667	neg	Sulfamonomethoxine	HMDB0258579	Unclassified	Unclassified	Unclassified			36.5	0	M-H2O-H	C11H12N4O3S	-4.39656714	805.6230206	778.0855356	927.1073796	889.1108916	755.3833765	890.7152921	625.7382721	756.0797882	848.3322548
4.54_415.0323m/z	415.0322778	4.54425	neg	2-Naphthalenesulfonic acid	HMDB0255446	Unclassified	Unclassified	Unclassified			36.5	0	2M-H	C10H8O3S	1.740143447	903.5848629	822.1813647	1029.741311	91.32218619	0.001403199	153.7095456	234.7577564	209.7876511	180.1356313
4.57_422.9898m/z	422.9898272	4.5668	neg	2-Amino-5-(5-nitro-2-furyl)-1,3,4-thiadiazole	72918	Unclassified	Unclassified	Unclassified			36.5	0	2M-H	C6H4N4O3S	-8.769655922	3812.2561	3464.928208	3705.384843	1438.503977	2058.929159	1705.942541	2692.175411	3203.847845	3325.664675
4.61_378.1150m/z	378.1149914	4.611883333	neg	[[[5-methoxy-1-[4-(trifluoromethyl)phenyl]pentylidene]amino]oxy]-, (E)-Acetic aci	2950	Unclassified	Unclassified	Unclassified			36.5	0	M+FA-H	C15H18F3NO4	-6.016433096	1485.258675	1459.599254	1106.521846	982.2850675	1105.623915	1032.890926	1477.358881	956.2922554	1365.067298
4.61_525.1420m/z	525.141965	4.611883333	neg	Premithramycin A1	63776	Unclassified	Unclassified	Unclassified			36.5	0	M-H2O-H	C27H28O12	3.178748406	853.3524388	919.0797588	889.095153	587.6202811	652.2033007	423.3162545	577.4915107	656.6439944	618.3532366
4.61_557.1339m/z	557.1338517	4.611883333	neg	7,8,3',4'-Tetrahydroxyflavanone 7-(2,4,6-triacetylglucoside)	52584	Unclassified	Unclassified	Unclassified			36.5	0	M-H2O-H	C27H28O14	6.573465627	8627.761473	8922.834901	8849.391078	0.001403199	0.001403199	0.001403199	6956.730253	6069.864014	6142.118077
4.61_671.1465m/z	671.1464904	4.611883333	neg	Delphin	47050	Phenylpropanoids and polyketides	Flavonoids	Flavonoid glycosides	*	C16312	36.5	0	M+FA-H	C27H30O17	-0.019090956	1506.820368	2095.430129	2210.864871	1504.729978	1713.140328	1369.59856	2146.771784	1593.55115	2199.792252
4.66_529.0194m/z	529.019403	4.660483333	neg	Tizoxanide	96516	Unclassified	Unclassified	Unclassified			36.5	0	2M-H	C10H7N3O4S	-9.006976559	2560.514353	2029.069946	1794.609158	2609.484785	2978.244179	2952.654395	3027.183787	2869.792636	2102.753285
4.75_237.0900m/z	237.0900213	4.747466667	neg	Falimint	95428	Benzenoids	Benzene and substituted derivatives	Nitrobenzenes			36.5	0	M-H	C11H14N2O4	8.151461944	1007.721499	1171.781624	1039.979637	1026.846874	1026.398915	991.480026	1079.916954	1119.315004	1093.061319
4.79_361.0365m/z	361.036485	4.792316667	neg	Tetracenomycin D3	63754	Unclassified	Unclassified	Unclassified			36.5	0	M-H2O-H	C20H12O8	2.917350859	567.1312244	517.4796612	688.2391498	194.0848814	163.316788	91.18182875	127.3934403	366.3095455	262.8266311
4.81_371.1347m/z	371.1347315	4.814166667	neg	4-(4-Hydroxyphenyl)-2-butanone glucoside	90253	Organic oxygen compounds	Organooxygen compounds	Carbohydrates and carbohydrate conjugates			36.5	0	M+FA-H	C16H22O7	-0.074894277	974.5690867	1126.18297	975.0403335	696.1138003	407.3281299	527.2792367	877.5849421	892.4075851	609.8171562
4.86_486.1210m/z	486.121004	4.858383333	neg	Pyranocyanin B	90668	Phenylpropanoids and polyketides	Flavonoids	Flavonoid glycosides			36.5	0	M-H	C24H23O11+	8.712104283	1575.978623	1463.356485	1343.378536	1865.558454	1968.936334	1722.678863	1559.774883	1706.598824	1658.986961
4.90_399.0264m/z	399.0264058	4.902316667	neg	(R)-2-Hydroxypropyl-CoM	69071	Unclassified	Unclassified	Unclassified			36.5	0	2M-H	C5H12O4S2	-4.29829521	12208.24519	12488.01799	13328.61627	13104.28624	13956.38719	13861.42724	10791.04676	10428.46908	10371.10877
4.92_286.0699m/z	286.0698706	4.92485	neg	Ascorbigen	86542	Organoheterocyclic compounds	Furofurans	Isosorbides	*		36.5	0	M-H2O-H	C15H15NO6	-7.294369777	871.156732	906.9744302	994.5443354	1861.753941	1996.246152	1854.369848	1262.863871	1178.758807	1350.132527
4.95_394.1543m/z	394.1543324	4.94705	neg	Ethyl 2-hydroxy-3-(3-indolyl)propanoate glucoside	90261	Lipids and lipid-like molecules	Fatty Acyls	Fatty acyl glycosides	*		36.5	0	M-H	C19H25NO8	9.090114599	1766.516273	2100.280582	2091.512211	3319.729436	2979.695184	3311.245434	1915.268019	2097.659279	1529.276988
4.99_280.1184m/z	280.1184039	4.99225	neg	Pentylone	HMDB0256297	Unclassified	Unclassified	Unclassified			36.5	0	M+FA-H	C13H17NO3	-2.732188144	366.1708935	518.8481324	270.0352194	387.9868142	436.3014663	409.1177396	473.7737859	510.8552475	450.6798296
5.06_197.1185m/z	197.1184938	5.05975	neg	p-Mentha-1(6),8-dien-3-ol	HMDB0034715	Lipids and lipid-like molecules	Prenol lipids	Monoterpenoids	*		36.5	0	M+FA-H	C10H16O	1.155552416	733.2416735	772.5929341	785.0612638	778.9352065	840.9902276	860.037144	998.9497288	596.7463249	752.8739804
5.20_403.0836m/z	403.0835823	5.20005	neg	5,7-Dihydroxyflavone 7-benzoate	48622	Unclassified	Unclassified	Unclassified			36.5	0	M+FA-H	C22H14O5	3.507203112	1154.768101	1006.789863	934.565388	0.001403199	0.001403199	0.001403199	517.1045541	215.9787445	623.4650173
5.43_486.2322m/z	486.2322323	5.428733333	neg	Mebeverine metabolite (1-Butanol, 4-[ethyl[2-(4-methoxyphenyl)-1-methylethyl]a	1081	Unclassified	Unclassified	Unclassified			36.5	0	M+FA-H	C22H35NO8	-5.07135708	1453.96072	2008.508679	1826.340688	3273.595519	3051.148673	2999.245459	1176.072057	1711.206802	1273.51347
5.47_305.1122m/z	305.1121809	5.471616667	neg	Asparaginyl-Glutamine	85644	Organic acids and derivatives	Carboxylic acids and derivatives	Amino acids, peptides, and analogues	*		36.5	0	M+FA-H	C9H16N4O5	7.336654981	4797.74347	3843.77819	3716.476825	3773.497819	3689.616612	4406.794886	2414.959763	1929.727666	2205.309124
5.57_449.2035m/z	449.2034519	5.57225	neg	1-dimethylarsinoyl-(2Z,5Z, 8Z,11Z,14Z,17Z)-heneicosahexaene	LMFA11000714	Lipids and lipid-like molecules	Fatty Acyls	Hydrocarbons			36.5	0	M+FA-H	C23H37AsO	-1.950140849	3252.329187	2218.862218	2328.936753	2711.307252	3287.335297	3138.24498	3272.458482	3700.673511	2886.961991
5.60_375.1299m/z	375.1299234	5.602	neg	(±)-3-(4-Hydroxyphenyl)-1,2-propanediol 4'-O-glucoside	HMDB0033082	Organic oxygen compounds	Organooxygen compounds	Carbohydrates and carbohydrate conjugates			36.5	0	M+FA-H	C15H22O8	0.765841663	1348.093854	869.8441165	1169.749654	829.0695703	1016.094376	950.8405909	1037.521955	935.6034296	922.0188908
5.65_345.1920m/z	345.1919548	5.650833333	neg	13,14-dihydro-15-keto-tetranor PGF1β	45724	Unclassified	Unclassified	Unclassified			36.5	0	M+FA-H	C16H28O5	0.259646239	723.9840573	819.3883581	751.0715871	1128.37542	1319.116503	1280.492962	780.9445968	692.3090057	575.1623609
5.65_378.0185m/z	378.0185404	5.650833333	neg	Methyl glucosinolate	HMDB0038429	Organic oxygen compounds	Organooxygen compounds	Carbohydrates and carbohydrate conjugates		C08404	36.5	0	M+FA-H	C8H15NO9S2	4.547577431	542.750406	530.3982688	661.8210429	1007.290677	901.1208797	864.5574366	661.9289288	338.089119	554.9457825
5.71_801.1716m/z	801.1716326	5.709933333	neg	6-Hydroxykaempferol 3,6-diglucoside 7-glucuronide	92070	Phenylpropanoids and polyketides	Flavonoids	Flavonoid glycosides	*		36.5	0	M-H	C33H38O23	-1.842981022	2192.982782	1417.230871	1743.219747	2100.947981	2443.561865	1934.252204	1539.042973	2647.869209	2802.443843
5.76_509.0561m/z	509.0561359	5.7632	neg	4-Hydroxyglucobrassicin	HMDB0303667	Unclassified	Unclassified	Unclassified			36.5	0	M+FA-H	C16H20N2O10S2	4.301689916	2489.882269	3019.642686	3339.218941	2384.184497	1769.217876	1674.645362	4263.594802	3649.851634	2590.922809
5.81_268.1305n	313.1271745	5.806016667	neg	Ripazepam	73201	Unclassified	Unclassified	Unclassified			36.5	0	M-H, M+FA-H	C15H16N4O	-7.119751691	7000.550819	7134.662932	6900.188497	7694.002774	7066.318215	6875.933052	8033.427057	8130.075411	8733.524857
5.87_519.2240m/z	519.2239914	5.866533333	neg	Maculosin	68423	Unclassified	Unclassified	Unclassified			36.5	0	2M-H	C14H16N2O3	-1.762499353	145.6425637	379.614077	135.2639249	255.2641432	144.5042875	377.0630776	374.8484457	422.0165749	592.6535428
6.03_364.1252m/z	364.1252386	6.028183333	neg	2-(acetylamino)-1,5-anhydro-2-deoxy-3-O-b-D-galactopyranosyl-D-arabino-Hex-	58113	Organic oxygen compounds	Organooxygen compounds	Carbohydrates and carbohydrate conjugates	*		36.5	0	M-H	C14H23NO10	0.873795688	1266.835153	1083.213578	1166.945506	1908.95887	1610.193747	1534.138726	1290.367934	1225.302933	1287.674358
6.30_319.1222m/z	319.1221937	6.302033333	neg	3-methyl-2-Quinoxalinone	64787	Unclassified	Unclassified	Unclassified			36.5	0	2M-H	C9H8N2O	6.698586482	296.2525073	330.1215619	254.4500958	597.2118928	613.2424153	506.2964859	253.8469037	392.7774018	418.8935151
6.42_541.1699m/z	541.1698692	6.41995	neg	Cappariloside B	90317	Organic oxygen compounds	Organooxygen compounds	Carbohydrates and carbohydrate conjugates			36.5	0	M+FA-H	C22H28N2O11	4.749600808	2038.821781	2031.994512	2191.019993	654.1698499	524.1182873	714.1263117	1741.556003	1147.241611	1638.086157
6.52_391.1356m/z	391.1355672	6.520516667	neg	Mytilin B	HMDB0033442	Organic acids and derivatives	Carboxylic acids and derivatives	Amino acids, peptides, and analogues			36.5	0	M+FA-H	C14H22N2O8	-0.726066955	305.6678242	212.5528638	350.4097472	361.531543	367.3702759	247.3157723	110.4180549	63.84618378	152.7357023
6.55_525.1570m/z	525.1569805	6.548566667	neg	Blestriarene B	68197	Unclassified	Unclassified	Unclassified			36.5	0	M+FA-H	C30H24O6	3.101331676	10881.82357	11959.06367	11754.0415	14367.69288	15177.87729	13862.07001	9128.297075	10629.32992	9159.825517
6.58_280.1168m/z	280.1168436	6.582416667	neg	Northiadene	2430	Unclassified	Unclassified	Unclassified			36.5	0	M-H	C18H19NS	1.065064329	936.3226655	909.5761268	947.5468736	700.1831098	717.4343305	733.874517	667.9639542	734.313553	665.4056112
6.83_527.2479m/z	527.2479217	6.832183333	neg	Canavalioside	91462	Lipids and lipid-like molecules	Prenol lipids	Terpene glycosides	*		36.5	0	M-H2O-H	C26H42O12	-3.412227351	7737.691452	6746.304154	7051.299393	6709.045187	6574.085086	6466.924765	10149.81137	9314.658033	8049.055376
7.18_267.0851m/z	267.0850728	7.178516667	neg	8-Azaadenosine	68949	Unclassified	Unclassified	Unclassified			36.5	0	M-H	C9H12N6O4	1.292055899	460.2136528	571.2766167	525.2957773	574.3716858	395.4981563	384.9767246	414.7902581	560.3569585	393.5895045
7.20_443.2573m/z	443.2573036	7.201166667	neg	sn-3-O-(geranylgeranyl)glycerol 1-phosphate	46751	Lipids and lipid-like molecules	Glycerophospholipids	Glycerophosphates	**	C04590	36.5	0	M-H	C23H41O6P	1.13522155	5538.451081	6416.825196	6699.575241	4776.867722	4066.888699	3999.111559	3427.669177	4043.892321	3334.036604
7.38_297.1711m/z	297.1711373	7.381583333	neg	Zedoarondiol	91582	Lipids and lipid-like molecules	Prenol lipids	Sesquiterpenoids	*		36.5	0	M+FA-H	C15H24O3	1.545821457	675.4002447	671.7010031	638.7105751	924.3684789	917.8389172	867.4358184	408.2492034	454.9656215	427.8088455
7.63_429.2132m/z	429.2132093	7.631783333	neg	L-Linalool 3-[xylosyl-(1->6)-glucoside]	86962	Lipids and lipid-like molecules	Fatty Acyls	Fatty acyl glycosides	*		36.5	0	M-H2O-H	C21H36O10	0.453057595	3150.6688	3506.704219	3808.572868	2093.278222	2062.616376	2505.358372	2237.80171	2499.12136	2520.851367
7.78_591.1961m/z	591.1960523	7.7828	neg	Medinoterb acetate	72816	Unclassified	Unclassified	Unclassified			36.5	0	2M-H	C13H16N2O6	2.796708247	2325.658179	2837.610907	3409.471605	5015.306469	4312.904674	3820.708595	2894.349973	3032.139581	3659.056014
7.94_289.0695m/z	289.0695159	7.9373	neg	Catechin	HMDB0002780	Phenylpropanoids and polyketides	Flavonoids	Flavans	***	C06562	36.5	0	M-H	C15H14O6	-7.742038557	907.9609595	745.7535156	495.8260463	718.3141288	1070.664859	962.8672396	674.8123753	1025.256261	758.9353786
8.09_599.1983m/z	599.198296	8.087016667	neg	Osthenol-7-O-beta-D-gentiobioside	71720	Unclassified	Unclassified	Unclassified			36.5	0	M+FA-H	C26H34O13	0.274178426	6430.973223	7579.14362	6215.059784	390.7398682	287.3929011	324.2904969	2329.388671	3236.475459	2625.657114
8.18_292.1558m/z	292.1557578	8.181083333	neg	Meperidine (pethidine)	1127	Unclassified	Unclassified	Unclassified			36.5	0	M+FA-H	C15H21NO2	1.319225698	958.798667	901.4572809	1042.258142	463.4306603	436.4399688	456.6381604	627.4798742	290.9493523	581.0961942
8.20_441.2119m/z	441.2119229	8.200183333	neg	CAY10509	45170	Unclassified	Unclassified	Unclassified			36.5	0	M-H	C23H35FO5S	0.623825496	3607.058346	4127.673317	4241.023715	4264.315667	4019.748276	3933.726912	3379.457049	3514.921458	3926.259346
8.99_471.2429m/z	471.2428607	8.9919	neg	Ursodeoxycholic acid 3-sulfate	LMST05020033	Lipids and lipid-like molecules	Steroids and steroid derivatives	Bile acids, alcohols and derivatives	*		36.5	0	M-H	C24H40O7S	1.402782718	14757.14308	15489.16338	16901.34409	4182.857705	3493.497512	3934.454861	9586.34119	9007.533502	9817.001924
9.02_601.2145m/z	601.2145141	9.01635	neg	11-Hydroxyiridodial glucoside pentaacetate	41176	Lipids and lipid-like molecules	Prenol lipids	Isoprenoids		C11666	36.5	0	M+FA-H	C26H36O13	1.294496383	6905.81568	9322.369337	8516.26571	202.4396907	91.88005469	335.6966482	2428.134178	2790.890173	2938.538678
9.03_513.2686m/z	513.2686233	9.03025	neg	3α-androstanediol glucuronide	43166	Unclassified	Unclassified	Unclassified			36.5	0	M+FA-H	C25H40O8	-4.052715227	2729.168727	3556.358019	2918.578872	3538.694393	3018.354539	2938.818833	2269.613922	3006.628978	2725.917357
9.06_493.2425m/z	493.2425451	9.056483333	neg	TETRAHYDROCORTISONE-3,21-DIACETATE	44530	Unclassified	Unclassified	Unclassified			36.5	0	M+FA-H	C25H36O7	-3.929230147	2676.322775	2666.453955	3295.689605	4044.507217	3778.414983	3892.225251	1726.173878	1208.133293	1526.647322
9.10_465.1747m/z	465.174723	9.095016667	neg	Citreoviridinol A1	HMDB0035369	Organoheterocyclic compounds	Dioxepanes	1,4-dioxepanes	*		36.5	0	M+FA-H	C22H28O8	-4.516387924	1749.951512	2299.466434	1871.398575	3573.507931	2528.626416	3577.034109	870.567921	1127.78455	1017.580799
9.33_329.1528m/z	329.152784	9.332316667	neg	2-amino-4'-hydroxy-Propiophenone	2018	Unclassified	Unclassified	Unclassified			36.5	0	2M-H	C9H11NO2	6.370403104	463.9546532	485.7537077	202.3867935	529.4739259	789.9816195	596.2393475	456.1168994	364.1460323	607.2441907
9.78_591.3543m/z	591.3543109	9.7801	neg	Hovenidulcigenin B	95930	Lipids and lipid-like molecules	Prenol lipids	Terpene lactones	*		36.5	0	M+FA-H	C32H50O7	0.831081877	1190.155589	2261.821249	1837.94814	1322.053516	1106.102222	1516.985605	1351.145057	1526.746067	1070.597888
0.76_146.0653m/z	146.0653312	0.757316667	neg	5-Methylthiopentanaldoxime	64513	Unclassified	Unclassified	Unclassified			36.4	0	M-H	C6H13NOS	5.59329669	1385.987402	1262.117983	1044.549647	979.7316342	1170.976696	1073.085926	1383.959922	1380.022319	1331.377342
0.78_385.1159m/z	385.1158896	0.778533333	neg	Oleoside 11-methyl ester	HMDB0041550	Lipids and lipid-like molecules	Prenol lipids	Terpene glycosides	*		36.4	14.6	M-H2O-H	C17H24O11	4.625121026	43547.95609	36721.27172	36257.24557	31895.32081	36323.10683	42960.37404	33349.5147	45896.10495	35577.59532
0.80_159.0244m/z	159.0243593	0.800366667	neg	L-Cys-Gly	3305	Organic acids and derivatives	Carboxylic acids and derivatives	Amino acids, peptides, and analogues	**	C01419	36.4	0	M-H2O-H	C5H10N2O3S	5.54503752	1645.477735	1728.80918	1886.776699	1445.815252	1070.050767	1804.450207	1912.049015	2056.921274	1307.499767
0.80_278.0565m/z	278.0564989	0.800366667	neg	Cidofovir	66562	Organoheterocyclic compounds	Diazines	Pyrimidines and pyrimidine derivatives		C06909	36.4	0	M-H	C8H14N3O6P	6.283758036	98371.23928	89598.22825	78278.45761	64348.7374	73733.83623	68739.59861	108673.9195	95721.02745	95480.39616
0.97_217.0161m/z	217.0161248	0.967616667	neg	4,4'-Biphenyldithiol	70164	Unclassified	Unclassified	Unclassified			36.4	0	M-H	C12H10S2	4.627692407	1684.596514	3388.283748	5595.06916	24135.42973	19048.54896	19804.53953	4352.797494	11557.13414	8713.729324
1.20_201.0382m/z	201.0382113	1.202333333	neg	Chrycorin	HMDB0035149	Organoheterocyclic compounds	Oxanes	Unclassified	*		36.4	0.27	M-H2O-H	C12H12O2S	1.144257967	2366.228132	2244.310003	2152.839313	1602.335977	1753.086852	1631.174636	1579.534874	1619.901832	1587.424429
1.27_377.0268m/z	377.0267851	1.26875	neg	PQQH2	63124	Unclassified	Unclassified	Unclassified			36.4	0	M+FA-H	C14H8N2O8	1.557113808	1317.521096	1837.017167	1826.57038	1133.175032	1044.061998	848.7093936	1122.447308	806.5186568	894.1900821
1.27_418.1109m/z	418.1108856	1.26875	neg	N-Didesethylquinagolide sulfate	2215	Unclassified	Unclassified	Unclassified			36.4	0	M-H	C16H25N3O6S2	-0.752851885	4291.530725	5804.278998	5073.383485	6316.270155	5788.621223	6027.542013	2930.791456	3863.239862	3598.466061
1.72_279.0042m/z	279.004233	1.721216667	neg	Riluzole	2321	Organoheterocyclic compounds	Benzothiazoles	Unclassified		C07937	36.4	0	M+FA-H	C8H5F3N2OS	-6.146381795	2937.071429	3516.699258	3194.367981	4271.444011	4203.87308	4126.286864	3854.099319	3709.716119	4369.975481
12.28_672.7821m/z	672.7821139	12.28461667	neg	Metrizoic acid	HMDB0254687	Unclassified	Unclassified	Unclassified			36.4	0	M+FA-H	C12H11I3N2O4	-2.213861126	526833.9994	497959.9703	486941.3565	520740.3323	528914.4157	530834.9406	557583.0946	509392.1785	553407.105
14.75_141.0207m/z	141.0207244	14.75111667	neg	1,2,3,5-Tetrahydroxybenzene	66034	Unclassified	Unclassified	Unclassified			36.4	0	M-H	C6H6O4	9.802224441	388.3391994	552.4752903	527.5997105	414.4038409	565.5007945	474.284537	661.6782613	488.1992164	460.5287077
2.07_197.0069m/z	197.0068778	2.070366667	neg	1,2-Dihydroxydibenzothiophene	66504	Unclassified	Unclassified	Unclassified			36.4	0	M-H2O-H	C12H8O2S	1.011119484	440.2729018	395.0221066	480.8975	394.1822673	313.3478068	371.4552765	452.4757389	343.8717868	323.1135375
2.09_319.0629m/z	319.0628938	2.092433333	neg	2-Naphthalenethiol	86427	Benzenoids	Naphthalenes	Unclassified	*		36.4	0	2M-H	C10H8S	2.586012007	332.6373042	382.1806962	406.7673414	627.2594395	469.6668261	797.5506313	258.865866	411.264962	434.5996077
2.29_453.0117m/z	453.0117485	2.294983333	neg	Primisulfuron	72644	Unclassified	Unclassified	Unclassified			36.4	0	M-H	C14H10F4N4O7S	-3.540875299	595.6456898	583.6534543	398.3913392	1343.95128	749.8200885	858.3732017	705.9699978	789.0130196	1028.624739
2.67_477.0888m/z	477.0888039	2.6698	neg	17-Beta-Estradiol-3,17-beta-sulfate	LMST05020041	Lipids and lipid-like molecules	Steroids and steroid derivatives	Sulfated steroids	*		36.4	0	M+FA-H	C18H24O8S2	-1.524537411	6655.222107	6103.442019	6233.419426	77.39493666	0.001403199	77.6740655	5542.550628	5419.22273	5202.318114
3.04_330.9743m/z	330.974314	3.041866667	neg	Alpha-T	LMFA12000345	Organohalogen compounds	Organochlorides	Unclassified		C18246	36.4	0	2M-H	C8H6S2	-1.789074372	3748.854112	3451.847713	4569.877609	589.0218163	565.4768947	520.6291885	3587.973403	3687.709292	3751.355047
3.26_344.1329m/z	344.1328749	3.260183333	neg	4,5-DIDEMETHYLSIMMONDSIN	84952	Unclassified	Unclassified	Unclassified			36.4	0	M-H	C15H23NO8	-6.418745894	635.2821053	405.2964293	223.2295358	1880.450121	1671.321289	1971.626371	1135.344711	1036.881958	962.8927885
3.85_299.0458m/z	299.0458069	3.84605	neg	Tazobactam	HMDB0015544	Organic acids and derivatives	Carboxylic acids and derivatives	Amino acids, peptides, and analogues		C07771	36.4	0	M-H	C10H12N4O5S	0.80924055	486.9281587	415.4387058	371.8018859	565.0100279	668.098465	676.0910842	498.9394077	562.0454663	660.0319011
3.87_317.0256m/z	317.0256321	3.868533333	neg	(E)-4-Isothiocyanato-1-(methylthio)-1-butene	87780	Organosulfur compounds	Isothiocyanates	Unclassified	*		36.4	0	2M-H	C6H9NS2	-7.464643952	12569.54044	13535.28937	13771.5523	3956.84246	3833.663832	4385.042055	12723.75581	13346.07556	14020.72445
3.89_383.0729m/z	383.0729023	3.8906	neg	N-Nitrosothialdine	87478	Organoheterocyclic compounds	Azacyclic compounds	Dithiazinanes	*		36.4	0	2M-H	C6H12N2OS2	5.125691722	380.6796521	487.1976736	400.7282585	120.9365664	54.28753735	381.5479861	634.1998506	786.3180947	681.2193629
4.02_545.0615m/z	545.0614686	4.024283333	neg	dTDP-4-dehydro-6-deoxy-L-mannose	63825	Nucleosides, nucleotides, and analogues	Pyrimidine nucleotides	Pyrimidine nucleotide sugars	**	C11907	36.4	0	M-H	C16H24N2O15P2	6.507825806	2453.162174	3087.721328	2895.383771	1589.016457	1389.684113	1713.187483	2543.617018	2910.839397	2414.439022
4.05_315.0215m/z	315.0214684	4.046666667	neg	Sulfamethizole	HMDB0014715	Benzenoids	Benzene and substituted derivatives	Benzenesulfonamides		C08050	36.4	0	M+FA-H	C9H10N4O2S2	-4.636979124	3188.920403	3418.146936	3492.832253	5894.490992	5537.693742	5446.360447	4072.887644	3935.899269	4514.191472
4.07_169.0871m/z	169.0870882	4.069216667	neg	3-Methylsuberic acid	3800	Lipids and lipid-like molecules	Fatty Acyls	Fatty acids and conjugates	*		36.4	0	M-H2O-H	C9H16O4	0.373895631	546.9564269	514.6018135	527.9216033	480.8455783	530.7519868	489.1802873	593.6173007	515.1604728	483.8540015
4.26_340.9951m/z	340.995139	4.2568	neg	L-Homocysteine sulfonic acid	6566	Organic acids and derivatives	Organic sulfonic acids and derivatives	Organosulfonic acids and derivatives	*		36.4	0	2M-H	C3H9NO3S2	-6.885353901	21620.58728	21307.25508	20781.68108	11766.29062	10589.02768	10752.38373	16800.35263	17403.73503	17129.74956
4.43_181.0585m/z	181.0584588	4.432683333	neg	Diphenylmethylphosphine	518	Unclassified	Unclassified	Unclassified			36.4	0	M-H2O-H	C13H13P	4.062874412	148.2205043	0.001403199	97.05974912	0.001403199	0.001403199	0.001403199	104.6608636	194.0709686	34.42073247
4.52_321.0532m/z	321.0532345	4.521733333	neg	Asparenomycin A	71665	Unclassified	Unclassified	Unclassified			36.4	0	M-H2O-H	C14H16N2O6S	-5.385989573	564.7454719	360.5758379	229.5917868	652.7592328	527.3376048	549.0274078	458.9049408	581.1311019	590.4659588
4.52_469.1692m/z	469.1692017	4.521733333	neg	Loxoprofen Metabolite trans-glucuronide	1043	Unclassified	Unclassified	Unclassified			36.4	0	M+FA-H	C21H28O9	-5.501531636	3585.169404	4152.040937	3714.49161	3920.26905	3671.637893	3745.094462	3012.024674	2730.893962	3401.411901
4.57_317.0255m/z	317.0254829	4.5668	neg	(E)-Raphanusanin	95516	Organoheterocyclic compounds	Thiolactams	Unclassified			36.4	0	2M-H	C6H9NS2	-7.933939904	3307.960715	3158.751413	3025.289239	3393.458478	3486.242384	3245.23828	2676.293096	3017.466067	2981.449755
4.63_218.0436m/z	218.0435831	4.632666667	neg	Azaserine	72922	Unclassified	Unclassified	Unclassified			36.4	0	M+FA-H	C5H7N3O4	9.966047706	583.2343656	599.6605939	475.9462168	0.001403199	0.001403199	0.001403199	38.50396873	54.81192303	28.63550998
4.66_631.1327m/z	631.132678	4.660483333	neg	Prunin 6''-O-gallate	HMDB0037582	Phenylpropanoids and polyketides	Flavonoids	Flavonoid glycosides			36.4	0	M+FA-H	C28H26O14	3.78686642	1285.75781	1027.099406	1312.903266	700.742666	440.8754754	405.0259714	1809.683433	2426.398749	1542.752903
4.75_365.2082m/z	365.2082446	4.747466667	neg	2,4-Dimethyl-5-pentylthiazole	94624	Organoheterocyclic compounds	Azoles	Thiazoles			36.4	0	2M-H	C10H17NS	-2.239373599	211.5551615	88.60142952	223.5956574	65.82504326	0.001403199	0.001403199	162.0625084	95.64243698	0.001403199
4.75_410.2163m/z	410.2163316	4.747466667	neg	Lasiocarpine	68261	Unclassified	Unclassified	Unclassified			36.4	0	M-H	C21H33NO7	-5.092886687	1308.323538	1499.48411	1436.915247	872.502099	1121.133604	807.1669592	1139.115836	1224.640184	1434.017399
4.77_401.1068m/z	401.1067848	4.769883333	neg	Secogalioside	69151	Unclassified	Unclassified	Unclassified			36.4	0	M-H2O-H	C17H24O12	-5.118297783	1596.358699	1803.019981	1903.38275	2267.059957	2668.753113	2254.250359	1715.872846	1696.850809	1018.466536
4.79_410.0439m/z	410.0438907	4.792316667	neg	Carboxycelecoxib	HMDB0060571	Organoheterocyclic compounds	Azoles	Pyrazoles	*		36.4	0	M-H	C17H12F3N3O4S	2.689757343	1442.226606	1290.987746	1425.862748	2431.571116	2209.277816	2015.735882	1235.802633	1097.761009	1308.693364

4.92_325.1279m/z	325.1279197	4.92485	neg	S-Methylpenicillamine	1731	Unclassified	Unclassified	Unclassified			36.4	0	2M-H	C6H13NO2S	5.50954358	234.5660018	323.9565905	325.1622775	483.2010084	717.2202961	409.2369017	283.2825774	318.5060525	328.1782353
4.92_443.1176m/z	443.1176494	4.92485	neg	5'-(3',4'-dihydroxyphenyl)-gamma-valerolactone glucuronide	86077	Unclassified	Unclassified	Unclassified			36.4	0	M+FA-H	C18H22O10	-4.647829155	2629.331305	2944.549853	2962.928046	2243.514977	2165.297207	2375.27372	2467.557922	2506.527946	2491.611977
4.95_295.1165m/z	295.1164692	4.94705	neg	Helinorbisabone	88199	Lipids and lipid-like molecules	Prenol lipids	Monoterpenoids	*		36.4	0	M+FA-H	C14H18O4	-8.966730009	4091.349632	4232.323995	4544.54696	5181.211249	5405.928416	5269.991202	3778.286474	4126.849406	3754.09147
4.97_345.0934m/z	345.0933824	4.969683333	neg	Coronene	73078	Unclassified	Unclassified	Unclassified			36.4	0	M+FA-H	C24H12	4.262613258	622.645807	671.767031	868.2148888	1015.64136	1151.996167	840.8821648	526.5461567	410.8439349	211.6717606
5.01_275.0516m/z	275.0516037	5.014716667	neg	6-Methoxy-3-(2-thiazolyl)-1H-indole	93293	Organoheterocyclic compounds	Indoles and derivatives	Indoles			36.4	0	M+FA-H	C12H10N2OS	8.767149154	4848.586602	5121.906398	5004.990634	7289.733834	8390.3028	8293.723205	7743.567604	6121.077742	5843.763456
5.06_431.0575m/z	431.05754	5.05975	neg	Myricetin 3-arabinoside	95999	Phenylpropanoids and polyketides	Flavonoids	Flavonoid glycosides			36.4	0	M-H2O-H	C20H18O12	-9.875715211	3429.541439	3683.17633	3775.791352	576.1803883	846.5118872	519.9402903	4636.964909	4390.622837	6078.168644
5.08_354.0836m/z	354.0836009	5.080566667	neg	Isotan B	67019	Unclassified	Unclassified	Unclassified			36.4	0	M+FA-H	C14H15NO7	1.767276108	712.2329905	690.1455074	778.9748443	1152.985609	654.9491579	1377.636598	1190.615533	767.2646252	1080.212748
5.20_339.1428m/z	339.1428166	5.20005	neg	Tocopheronic acid	87057	Lipids and lipid-like molecules	Prenol lipids	Sesquiterpenoids	*		36.4	0	M+FA-H	C16H22O5	-7.173403126	4522.16921	4534.668072	4244.593531	3724.021667	4152.609	3791.130643	3464.683068	3181.457162	3896.487087
5.27_311.1505m/z	311.1504536	5.271716667	neg	Blennin D	HMDB0031902	Lipids and lipid-like molecules	Prenol lipids	Terpene lactones	*		36.4	0	M+FA-H	C15H22O4	1.658869961	725.6278429	372.7653549	812.6559553	658.9985564	752.1573448	984.3677121	569.6875799	691.6150122	834.312187
5.32_452.1273n	497.1258708	5.320233333	neg	Viniferifuran	89445	Phenylpropanoids and polyketides	2-arylbenzofuran flavonoids	Unclassified			36.4	0	M-H, M+FA-H	C28H20O6	2.917686893	8444.404224	7908.447514	10195.42009	9608.323261	12020.73104	11713.60445	8110.622829	8532.556369	12451.81441
5.34_479.0606m/z	479.0606435	5.3394	neg	11-O-Demethylpradinone II	66533	Unclassified	Unclassified	Unclassified			36.4	0	M-H	C24H16O11	-2.794264832	3692.06902	4540.450451	4820.98148	3879.037903	4215.605552	3643.762619	3852.152174	3840.438236	3622.109943
5.36_233.1396m/z	233.1395995	5.361783333	neg	7-Methyl-3-methylene-1,6,7-octanetriol	HMDB0033640	Lipids and lipid-like molecules	Fatty Acyls	Fatty alcohols	*		36.4	0	M+FA-H	C10H20O3	0.808513756	349.5355272	385.001422	266.5339959	385.2702575	436.1635822	305.207233	360.1697145	236.3853521	312.0645861
5.36_341.1969m/z	341.1968858	5.361783333	neg	3beta-Acetoxy-4-oplopanone	LMPR0103350001	Lipids and lipid-like molecules	Prenol lipids	Isoprenoids			36.4	0	M+FA-H	C17H28O4	-0.25799213	265.3247135	606.1751381	523.885502	620.335933	584.7967814	463.9298211	242.0046749	612.5659729	552.4991657
5.36_393.0759m/z	393.0758819	5.361783333	neg	Zileuton O-glucuronide	HMDB0060914	Organic oxygen compounds	Organooxygen compounds	Carbohydrates and carbohydrate conjugates	*		36.4	0	M-H2O-H	C17H20N2O8S	-0.760967635	1249.07485	1452.619439	1499.813976	1297.015831	1605.200713	1222.441342	1672.662678	2186.248718	2070.890394
5.36_469.0680m/z	469.0680085	5.361783333	neg	Cefuroxime	HMDB0015244	Organoheterocyclic compounds	Lactams	Beta lactams		C06894	36.4	0	M+FA-H	C16H16N4O8S	2.172177922	3310.469356	3764.474808	3590.747714	3038.003276	2776.361775	2423.136705	2230.388129	2783.498599	2125.836342
5.41_329.1065m/z	329.1065033	5.406433333	neg	Chelerythrine	96495	Unclassified	Unclassified	Unclassified			36.4	0	M-H2O-H	C21H18NO4+	2.187176736	2869.887962	2954.717105	2701.537105	4772.505496	3996.673684	4381.221988	3595.152681	4083.830537	3119.736835
5.45_445.1904m/z	445.190443	5.451166667	neg	Ptilosteroid A	84302	Lipids and lipid-like molecules	Sterol Lipids	Steroids			36.4	0	M-H	C21H34O8S	0.628433994	4186.597087	4217.028119	4126.407789	5620.36361	4575.149002	4873.937766	4975.488397	4640.355304	4824.088183
5.50_365.1585m/z	365.1585317	5.497816667	neg	Bis(2-methylpropanoyloxy)-9,10-epoxy-p-mentha-1,3,5-triene	88635	Benzenoids	Phenol esters	Unclassified	*		36.4	0	M+FA-H	C18H24O5	-6.387563548	1052.396198	820.8126071	789.1555606	752.1827649	990.7275463	1327.268859	826.9889174	734.9996602	1115.060229
5.52_402.1045m/z	402.1044579	5.516816667	neg	N-Arachidonoyl Glycine	HMDB0241918	Organic acids and derivatives	Carboxylic acids and derivatives	Amino acids, peptides, and analogues	*		36.4	0	M+FA-H	C15H19NO9	0.766910077	1196.877498	1257.852118	1199.824296	446.6720374	286.9538425	410.90127	1105.412998	998.779808	1201.096112
5.64_304.1053m/z	304.1053227	5.6386	neg	N-Acetyl-8-O-methyl-Neuraminic acid	5736	Unclassified	Unclassified	Unclassified			36.4	0	M-H2O-H	C12H21NO9	4.742953883	945.7280459	1153.869535	1263.68925	1198.499905	1019.120963	850.7988315	1042.916916	925.3447639	972.754094
5.70_330.1748m/z	330.1747681	5.701283333	neg	1-NM-PP1	45538	Unclassified	Unclassified	Unclassified			36.4	0	M-H	C20H21N5	7.092303365	1783.713809	1812.92939	1615.708366	1861.954872	2088.775218	2325.799355	1126.653769	1641.429474	1851.17448
5.79_679.2725m/z	679.2725132	5.785316667	neg	p,γ-Dihydroxyphenylbutazone	1842	Unclassified	Unclassified	Unclassified			36.4	0	2M-H	C19H20N2O4	-7.092110515	1243.642398	637.1056358	788.7627047	510.908236	1024.610742	1024.575309	1630.644564	1666.585228	1207.844734
5.87_412.1960m/z	412.1960031	5.866533333	neg	CAY10591	45193	Unclassified	Unclassified	Unclassified			36.4	0	M+FA-H	C20H25N5O2	-8.237462642	4038.430362	4350.430612	5096.003435	3829.056023	3160.267046	3348.40583	5572.552197	4489.724439	4728.258456
5.95_286.0698m/z	286.0697832	5.95155	neg	Mukonidine	86803	Organoheterocyclic compounds	Indoles and derivatives	Carbazoles	*		36.4	0	M+FA-H	C14H11NO3	-9.594122742	1027.337632	1139.494838	1260.047771	991.9251981	1486.506727	1493.034779	832.3700366	1209.936345	1070.391366
5.99_729.2333m/z	729.2333074	5.9916	neg	Galabiose	86577	Organic oxygen compounds	Organooxygen compounds	Carbohydrates and carbohydrate conjugates	*	C00760	36.4	0	M+FA-H	C24H44O22	3.919008877	2297.965091	1677.838045	2658.564811	2575.414955	2959.448927	2559.985368	1385.879575	1418.876334	856.0937145
6.25_539.2046m/z	539.2045818	6.248083333	neg	Olmesartan medoxomil	HMDB0255956	Benzenoids	Benzene and substituted derivatives	Biphenyls and derivatives			36.4	0	M-H2O-H	C29H30N6O6	-0.465337794	9808.559775	7390.286228	9478.320231	9282.640467	7109.770846	8773.100987	7801.981716	10080.26206	7928.479336
6.25_607.1923m/z	607.1922853	6.248083333	neg	Diazinon	HMDB0032943	Organic acids and derivatives	Organic thiophosphoric acids and derivatives	Thiophosphoric acid esters		C14324	36.4	0	2M-H	C12H21N2O3PS	-4.173933208	6445.100872	4673.911911	5490.125929	5978.073924	3735.681737	4796.959467	4997.532207	5173.454091	4761.000889
6.56_213.1496m/z	213.1495769	6.55905	neg	11-oxo-dodecanoic acid	LMFA01060088	Lipids and lipid-like molecules	Fatty Acyls	Fatty Acids and Conjugates			36.4	0	M-H	C12H22O3	-0.192557977	1162.625113	1083.207719	1124.538297	1096.913025	1254.895821	1333.066931	1074.024917	1076.386525	1088.995452
6.62_548.2715m/z	548.2715141	6.61635	neg	Dihydrodeoxystreptomycin	2507	Unclassified	Unclassified	Unclassified			36.4	0	M-H2O-H	C21H41N7O11	5.200298034	2670.876095	3124.60693	2874.715654	4241.129737	4580.682789	4792.157018	2961.592835	3589.327392	3687.780875
6.86_401.0988m/z	401.0987876	6.86375	neg	doripenem	96162	Benzenoids	Phenols	Benzenediols			36.4	0	M-H2O-H	C15H24N4O6S2	6.90804783	12290.58472	12545.81922	11441.58306	12052.49608	12814.60431	12338.98858	11599.09799	12215.69462	10232.65992
6.92_657.1501m/z	657.1500569	6.915966667	neg	Oxidized glutathione	HMDB0003337	Organic acids and derivatives	Carboxylic acids and derivatives	Amino acids, peptides, and analogues	***	C00127	36.4	0	M+FA-H	C20H32N6O12S2	-0.17721949	22978.94359	4022.642486	70543.93128	4754.347079	3114.984101	3215.139566	5144.446405	4796.771801	11273.75865
7.29_605.3107m/z	605.3106576	7.286583333	neg	PG(22:4(7Z,10Z,13Z,16Z)/0:0)	LMGP04050017	Lipids and lipid-like molecules	Glycerophospholipids	Glycerophosphoglycerols			36.4	0	M+FA-H	C28H49O9P	1.847046252	9518.169418	11409.84853	10923.85182	998.2998222	1237.890137	938.7853179	5152.760338	5492.096884	6387.97735
7.31_408.1438m/z	408.1437871	7.314366667	neg	CORALYNE	43948	Unclassified	Unclassified	Unclassified			36.4	0	M+FA-H	C22H21NO4	-4.058827328	1286.835274	1622.495746	1568.469035	2208.780047	2193.921253	1715.147011	827.8141751	929.9211744	1495.573696
7.42_323.1865m/z	323.1864955	7.41815	neg	6-Paradol	68355	Benzenoids	Phenols	Methoxyphenols	*	C10482	36.4	0	M+FA-H	C17H26O3	0.351935834	392.783868	272.2648847	326.1425969	457.2690214	332.2476899	445.3941473	205.9747892	138.4631195	348.6291479
7.51_403.1142m/z	403.1142338	7.50615	neg	Melithiazol A	71030	Unclassified	Unclassified	Unclassified			36.4	0	M-H2O-H	C20H26N2O4S2	-3.137505665	5510.913638	5566.205491	5309.39396	5680.434013	6473.012655	5632.47891	4585.99846	6086.330069	5631.271726
7.55_715.2543m/z	715.2542602	7.547766667	neg	202-791	69691	Unclassified	Unclassified	Unclassified			36.4	0	2M-H	C17H18N4O5	8.512708068	9201.062429	9309.401618	8401.968948	11019.98633	11866.29346	10909.88797	6829.244742	7352.250478	8065.836476
7.74_695.3618m/z	695.3618071	7.738233333	neg	Acrivastine	HMDB0240231	Benzenoids	Benzene and substituted derivatives	Styrenes			36.4	0	2M-H	C22H24N2O2	2.193531989	1371.459385	1564.634199	2399.513972	1695.927487	1880.490061	1643.692617	1659.027662	2302.86248	1926.63875
8.69_725.2750m/z	725.2750105	8.6894	neg	Fucα1-2Galβ1-4[Fucα1-3]GlcNAcβ-Sp	3645	Unclassified	Unclassified	Unclassified			36.4	0	M-H2O-H	C28H48N4O19	2.117844497	3864.257753	5163.068293	4243.263285	324.2222049	0.001403199	0.001403199	1226.892661	1955.701355	1902.714845
8.96_473.2525m/z	473.2524794	8.962016667	neg	Lucidenic acid L	HMDB0035975	Lipids and lipid-like molecules	Prenol lipids	Triterpenoids	*		36.4	0	M-H	C27H38O7	-4.212253028	3615.68605	3786.521815	4184.442827	299.9534958	19.11580226	178.4842748	1470.70019	1596.37136	1674.395221
9.02_815.5685m/z	815.5685192	9.01635	neg	1,3,12-Trihydroxycholan-24-oic acid	5360	Lipids and lipid-like molecules	Steroids and steroid derivatives	Bile acids, alcohols and derivatives	*		36.4	0	2M-H	C24H40O5	0.792211703	1950.253858	3697.296934	3122.711532	0.001403199	0.001403199	0.001403199	0.001403199	0.001403199	0.001403199
9.83_382.2062m/z	382.2062295	9.825466667	neg	Methylmibefradil Metabolite (Acetic acid, methoxy-, 6-fluoro-1,2,3,4-tetrahydro-2	1281	Unclassified	Unclassified	Unclassified			36.4	0	M+FA-H	C19H28FNO3	8.020854333	1085.914871	1221.594631	1509.03086	1622.673485	1710.953483	1425.377619	1261.058895	1440.032189	916.8666993
0.69_223.0530m/z	223.0530134	0.692166667	neg	2-Amino-5-nitrobenzophenone	1570	Unclassified	Unclassified	Unclassified			36.3	0	M-H2O-H	C13H10N2O3	7.073899911	3253.676753	3273.931529	3658.857584	5458.732015	5413.731616	5164.316373	5105.083262	4927.970122	5604.01356
0.69_553.0233m/z	553.0232759	0.692166667	neg	Fenitrothion	70064	Organic acids and derivatives	Organic thiophosphoric acids and derivatives	Thiophosphoric acid esters		C14442	36.3	0	2M-H	C9H12NO5PS	-7.595055432	2440.764527	2165.05384	2667.74902	4745.678356	5425.418872	5343.666503	4055.847007	5169.179109	4637.341995
0.71_673.3610m/z	673.3610042	0.714216667	neg	Istamycin AO	71978	Unclassified	Unclassified	Unclassified			36.3	0	2M-H	C13H27N3O7	-2.253690241	6073.279722	3815.227018	4585.732634	1661.526535	1178.196659	2339.222539	5585.685948	2692.014282	3415.661349
0.78_261.0286m/z	261.0285722	0.778533333	neg	Nithiazide	73162	Unclassified	Unclassified	Unclassified			36.3	0	M+FA-H	C6H8N4O3S	-6.211283696	15304.10157	14837.35627	16937.74319	25214.6696	27193.59546	26374.18041	15637.4023	19505.89272	17626.26035
0.78_268.0861m/z	268.0861044	0.778533333	neg	Rutaecarpine	67545	Organoheterocyclic compounds	Indoles and derivatives	Pyridoindoles		C09238	36.3	0	M-H2O-H	C18H13N3O	-6.675394387	682.3862604	1374.520186	908.0592514	175.1766757	0.001403199	0.001403199	1458.473374	1844.643967	576.5069517
0.80_294.0880m/z	294.0880379	0.800366667	neg	Mebendazole	1074	Benzenoids	Benzene and substituted derivatives	Benzophenones			36.3	0.711	M-H	C16H13N3O3	-1.277359857	5496.365775	3565.396932	5404.515498	1513.894019	1214.968274	2270.528225	4215.42893	8041.083051	1455.118654
0.84_283.0784m/z	283.0784363	0.841733333	neg	Naphthalene epoxide	58364	Benzenoids	Naphthalenes	Unclassified	*		36.3	0	2M-H	C10H6O	6.980561608	1882.728148	1816.088493	1638.055227	1214.917856	654.2224495	913.8673541	1644.290788	1347.919366	340.8893676
0.86_262.0618m/z	262.0617551	0.86335	neg	CAY10550	45294	Unclassified	Unclassified	Unclassified			36.3	0	M-H2O-H	C15H11N3O3	-1.583335603	2038.576106	2419.372728	2242.472347	3679.087294	2185.493568	2757.657882	2340.625497	2751.815038	1595.094902
1.06_192.9425m/z	192.942474	1.055866667	neg	Thiotropocin	69309	Unclassified	Unclassified	Unclassified			36.3	0	M-H2O-H	C8H4O3S2	0.609054623	1060.221266	1000.640297	835.6983129	942.3419492	958.6025659	1019.262918	1192.234863	942.0942268	1010.526319
1.16_191.0939m/z	191.0938968	1.160166667	neg	Methyl DL-Leucate	91377	Lipids and lipid-like molecules	Fatty Acyls	Fatty acid esters	*		36.3	0	M+FA-H	C7H14O3	9.580154443	500.9671208	743.102185	374.1253567	1381.412794	70.53682678	216.6632989	109.501211	146.0495988	97.23589517
1.27_304.0908n	303.0837092	1.26875	neg	4-amino-6,7-dihydro-5H-cyclopenta[b]pyridin-2-yl 4-methylbenzenesulfonate	96300	Unclassified	Unclassified	Unclassified			36.3	2.77	M-H2O-H, M-H	C15H16N2O3S	8.768894653	8703.239725	9349.922564	9351.366179	9719.10122	9671.702242	9371.200925	8345.125666	9685.135587	9399.789734
1.31_411.0463m/z	411.0462795	1.3134	neg	CMP-2-aminoethylphosphonate	63350	Organic oxygen compounds	Organooxygen compounds	Carbohydrates and carbohydrate conjugates	**	C05673	36.3	0	M-H2O-H	C11H20N4O10P2	-3.128519037	995.4595505	1084.170604	1257.061063	64.78165201	97.72133654	236.4539269	560.2946198	533.6177368	503.7438436
1.38_283.0218m/z	283.0218354	1.380283333	neg	Quercetin	HMDB0005794	Phenylpropanoids and polyketides	Flavonoids	Flavones	***	C00389	36.3	0	M-H2O-H	C15H10O7	-9.853404012	1793.602822	1546.542333	1763.082561	2965.645503	2878.215039	2930.620304	2411.717086	2455.709938	2692.620422
1.38_293.0507m/z	293.0506606	1.380283333	neg	2-Butanone, 4-[6-(sulfooxy)-2-naphthalenyl]-	1378	Unclassified	Unclassified	Unclassified			36.3	0	M-H	C14H14O5S	5.925566797	11175.82099	8645.250578	8759.412684	14834.4736	15009.23395	12898.35776	16490.27354	13678.55701	13596.18269
1.62_289.0568m/z	289.0568199	1.615883333	neg	Isoprothiolane	HMDB0031779	Organic acids and derivatives	Carboxylic acids and derivatives	Dicarboxylic acids and derivatives	*	C11111	36.3	0	M-H	C12H18O4S2	-1.912519788	2681.766229	2749.876226	2917.748848	2528.440104	2404.170146	2614.623397	2814.02275	2757.670417	2933.473143
1.65_124.0404m/z	124.0403891	1.65405	neg	4-Aminocatechol	72171	Unclassified	Unclassified	Unclassified			36.3	0	M-H	C6H7NO2	-0.103446636	624.247826	504.4275851	554.8181964	501.855757	545.4414167	523.1651877	606.3326391	497.5346918	513.4505718
1.72_240.9926m/z	240.9925519	1.721216667	neg	Methylthioribosyl phosphate	6458	Unclassified	Unclassified	Unclassified			36.3	0	M-H2O-H	C6H13O7PS	-6.028008199	1354.784557	1520.568279	1178.172777	1599.445963	1471.694482	1248.825647	1452.243702	1394.454628	2007.071747
1.72_265.0930m/z	265.0930352	1.721216667	neg	Histidylglutamic acid	HMDB0028884	Organic acids and derivatives	Carboxylic acids and derivatives	Amino acids, peptides, and analogues	*		36.3	0	M-H2O-H	C11H16N4O5	-4.200101716	1630.158474	1625.117728	1283.302549	599.1165556	564.6401404	689.4364942	776.7140846	875.7376028	670.4471528
1.80_310.1024m/z	310.102413	1.798266667	neg	Omeprazole sulfide	1635	Unclassified	Unclassified	Unclassified			36.3	0	M-H2O-H	C17H19N3O2S	1.386160146	2150.337863	2377.132631	2367.958124	4330.059989	4685.187064	5223.666009	2054.158646	2249.12902	2109.910611
1.84_303.0271m/z	303.0270695	1.841666667	neg	6-Mercaptopurine	503	Organoheterocyclic compounds	Imidazopyrimidines	Purines and purine derivatives	**	C02380	36.3	0	2M-H	C5H4N4S	9.90554784	2481.805578	2584.684525	3334.541598	2028.254144	1885.213843	2063.373185	3498.046964	3119.910172	3274.691824
1.91_332.0771m/z	332.0770939	1.911116667	neg	Grandisine III	HMDB0040683	Organoheterocyclic compounds	Quinolines and derivatives	Benzoquinolines			36.3	0	M+FA-H	C15H13NO5	-1.677077621	766.3100662	472.016265	469.6079858	541.7177966	614.1737871	541.6982915	505.566948	443.1736619	195.0728533
11.00_723.4976m/z	723.4975514	11.00455	neg	PA(18:0/20:4(5Z,8Z,11Z,14Z))	3884	Lipids and lipid-like molecules	Glycerophospholipids	Glycerophosphates	*		36.3	0	M-H	C41H73O8P	0.720418895	26062.87654	23399.42804	24764.12807	21025.48362	23695.46079	21499.56988	25893.89699	21700.33782	22898.38132
13.56_759.5175m/z	759.5175331	13.55923333	neg	PG(15:0/20:2(11Z,14Z))	78977	Lipids and lipid-like molecules	Glycerophospholipids	Glycerophosphoglycerols			36.3	0	M-H	C41H77O10P	-0.822780737	2411.105097	1717.976981	1914.783568	517.1568192	434.1623676	0.001403199	2145.456326	1973.686192	2235.742027
2.07_248.0486m/z	248.0486241	2.070366667	neg	Cysteinyl-Glutamate	85688	Organic acids and derivatives	Carboxylic acids and derivatives	Amino acids, peptides, and analogues	*		36.3	0	M-H	C8H13N2O5S-	5.553070002	1115.671094	1331.407151	1322.292105	1278.319186	1092.942393	1070.278107	1433.465721	1170.187266	1224.817528
2.19_275.0651m/z	275.0650784	2.194566667	neg	4-Phthalimidoglutaramic acid	2814	Unclassified	Unclassified	Unclassified			36.3	0	M-H	C13H12N2O5	-8.210470185	1810.183365	1591.928969	1874.391036	2560.094837	2543.629294	2695.541229	2187.829729	2657.757854	2016.557579
2.36_253.0307m/z	253.0307068	2.36465	neg	7-Aminocephalosporanic acid	64538	Unclassified	Unclassified	Unclassified			36.3	0	M-H2O-H	C10H12N2O5S	6.820341652	8049.053282	8425.598591	9158.862216	15619.58457	15595.46834	15127.06147	11467.84077	13360.14209	13075.19796
2.61_435.1145m/z	435.1145277	2.6102	neg	Monotropein	HMDB0035608	Lipids and lipid-like molecules	Prenol lipids	Terpene glycosides	*	C09788	36.3	0	M+FA-H	C16H22O11	0.290431875	637.3378918	594.2269162	597.324536	0.001403199	0.001403199	0.001403199	0.001403199	0.001403199	0.001403199
2.72_317.0505m/z	317.0505156	2.72215	neg	1-Phenyl-3-(phenylsulfonyl)-2-propen-1-one	70570	Unclassified	Unclassified	Unclassified			36.3	0	M+FA-H	C15H12O3S	5.871836756	6682.748582	7362.415779	7006.677913	7194.581102	6751.327605	7322.510947	6566.80816	7826.770196	6907.68258
2.82_236.0541m/z	236.0541489	2.817666667	neg	Methyl 2,3-dihydro-3,5-dihydroxy-2-oxo-3-indoleacetic acid	93553	Organoheterocyclic compounds	Indoles and derivatives	Indolyl carboxylic acids and derivatives			36.3	0	M-H	C11H11NO5	-9.689701577	800.2590096	816.3694802	759.2353847	706.3662101	554.8678664	619.3418842	784.1819084	1195.784898	1055.938193
2.88_399.0149m/z	399.0148936	2.877083333	neg	Rosacyanin B	47206	Lipids and lipid-like molecules	Polyketides	Flavonoids			36.3	0	M-H2O-H	C22H10O9	0.604737156	3911.387919	4085.740395	4305.812278	6581.224035	6372.918874	6175.266161	5173.861058	5503.914598	4373.637088
3.44_511.9857m/z	511.9856843	3.440016667	neg	dCTP	HMDB0000998	Nucleosides, nucleotides, and analogues	Pyrimidine nucleotides	Pyrimidine deoxyribonucleotides	**	C00458	36.3	0	M+FA-H	C9H16N3O13P3	-4.531300692	1631.935481	2580.547505	2618.510283	0.001403199	0.001403199	0.001403199	1021.138956	1926.56028	1463.929804
3.48_514.0782m/z	514.0781952	3.484883333	neg	Tolrestat glucuronide	2879	Unclassified	Unclassified	Unclassified			36.3	0	M-H2O-H	C22H22F3NO9S	-1.314310924	6462.9462	7543.975145	7312.096115	11393.25932	9443.694878	9808.720765	8104.271712	8003.556196	7693.539381
3.53_335.0446m/z	335.0445871	3.529966667	neg	4-Hydroxy-5-(3'-hydroxyphenyl)-valeric acid-3'-O-sulphate	HMDB0059975	Lipids and lipid-like molecules	Fatty Acyls	Fatty acids and conjugates	*		36.3	0	M+FA-H	C11H14O7S	1.242609085	930.4328943	867.5328244	715.3843437	151.2795817	201.516747	134.4495522	264.2209825	206.5478435	159.4317526
3.56_404.0375m/z	404.0375278	3.564783333	neg	Cefapirin	86987	Organic acids and derivatives	Carboxylic acids and derivatives	Amino acids, peptides, and analogues	*	C06896	36.3	0	M-H2O-H	C17H17N3O6S2	-1.201664414	1616.618748	1906.294364	1635.334538	2538.11143	2691.715886	2318.906428	2318.883827	1936.551935	1998.33384
3.87_465.0119m/z	465.0118594	3.868533333	neg	(2,6-dihydroxy-4-{4-hydroxy-7-methyl-11-oxo-2,8-dioxatricyclo[7.3.1.0^{5,13}]trideca	HMDB0124751	Unclassified	Unclassified	Unclassified	*		36.3	0	M+FA-H	C18H12O10S	-3.478448934	955.0451905	991.9713155	1188.365677	745.3438203	798.1406685	1367.479644	1243.031074	1470.372704	1384.861632
3.87_467.0356m/z	467.0356447	3.868533333	neg	Ajoene	HMDB0033566	Organosulfur compounds	Sulfoxides	Unclassified		C16757	36.3	0	2M-H	C9H14OS3	3.343723701	992.8705425	1140.093497	1009.294199	462.3719104	429.3545711	1225.687202	798.2567332	741.7267639	914.188121
4.09_454.0971m/z	454.0971223	4.091783333	neg	1-(5-Phosphoribosyl)-4-(N-succinocarboxamide)-5-aminoimidazole	66260	Unclassified	Unclassified	Unclassified			36.3	0	M+FA-H	C12H20N5O9P	-2.308583548	1015.8428	952.0133688	990.4273311	274.9955997	250.6586309	315.1892541	266.4350904	480.1675072	256.9058502
4.23_326.0260m/z	326.0259718	4.234483333	neg	Sulfamoyldapsone	72196	Unclassified	Unclassified	Unclassified			36.3	0	M-H	C12H13N3O4S2	-4.585749328	7248.466315	7777.982261	7620.917519	8851.81062	8456.487255	7811.457849	7897.042538	8894.835727	7997.846045
4.30_525.1024m/z	525.1023679	4.300033333	neg	2,3-Dehydrosilybin	HMDB0040513	Lignans, neolignans and related compounds	Flavonolignans	Unclassified			36.3	0	M+FA-H	C25H20O10	-3.086382533	1064.683143	1056.420963	1063.469934	1309.851388	1318.315658	1167.042512	1090.054368	952.0011077	1605.100941
4.32_569.1854m/z	569.1854383	4.3224	neg	Ligustroside	90177	Organic oxygen compounds	Organooxygen compounds	Carbohydrates and carbohydrate conjugates	*		36.3	0	M+FA-H	C25H32O12	-4.084414625	1270.720429	1752.072798	1683.917106	1615.940572	1855.392952	1611.766235	1511.180268	1150.906677	1891.647841
4.39_1039.2510m/z	1039.250988	4.3893	neg	Calcein AM	68943	Unclassified	Unclassified	Unclassified			36.3	0	M+FA-H	C46H46N2O23	3.670222261	3367.18161	3626.891937	6923.153698	1053.310196	1556.217451	0.001403199	5958.086084	4753.087158	6021.583558
4.39_323.0596m/z	323.0596297	4.3893	neg	1,2-Dihydroxy-3-keto-5-methylthiopentene	62805	Organic oxygen compounds	Organooxygen compounds	Carbonyl compounds	**	C15606	36.3	0	2M-H	C6H10O3S	-9.949142064	10953.89417	10508.73751	9863.512746	9144.85827	8448.807761	8663.538098	12265.92073	12253.63479	13004.61698
4.39_607.1558m/z	607.1558401	4.3893	neg	Demonomethylchlorpromazine	HMDB0061135	Organoheterocyclic compounds	Benzothiazines	Phenothiazines	*		36.3	0	2M-H	C16H17ClN2S	4.804750291	13614.53509	19577.56794	17691.36076	8675.78559	6913.362208	7979.459112	18729.2667	14722.02448	21675.34674
4.43_216.0491m/z	216.0490644	4.432683333	neg	Carboxin	HMDB0249648	Unclassified	Unclassified	Unclassified			36.3	0	M-H2O-H	C12H13NO2S	0.875842135	352.4587047	193.9663205	222.194998	925.513097	423.8424847	932.8014631	578.112291	851.6832733	522.5781961
4.45_399.0935m/z	399.0934724	4.454616667	neg	1-O-Caffeoylquinic acid	HMDB0030652	Organic oxygen compounds	Organooxygen compounds	Alcohols and polyols	*		36.3	0	M+FA-H	C16H18O9	0.529192071	597.9593666	808.6882678	768.3582235	120.970309	210.2031005	207.4518335	332.5808102	593.5564627	549.8210714
4.57_436.2205m/z	436.2204654	4.5668	neg	FK-866	45518	Unclassified	Unclassified	Unclassified			36.3	0	M+FA-H	C24H29N3O2	-9.49502522	5265.608781	6028.504115	5996.904447	7310.140409	7215.704158	6734.425571	6949.005461	7141.989674	7591.649558
4.70_613.1241m/z	613.124114	4.703166667	neg	N-Acetyl-5-hydroxysulfapyridine	2554	Unclassified	Unclassified	Unclassified			36.3	0	2M-H	C13H13N3O4S	9.829593053	960.3966576	349.4809503	946.8599385	1494.643489	1450.02281	1119.550555	0.001403199	150.9467239	0.001403199
4.79_451.0651m/z	451.0650653	4.792316667	neg	3,5,7-Tris(acetyloxy)-2-[4-(acetyloxy)-3-hydroxyphenyl]-4H-1-benzopyran-4-one	50719	Unclassified	Unclassified	Unclassified			36.3	0	M-H2O-H	C23H18O11	-4.265132354	865.1775768	659.9891733	609.7267779	2253.069409	2475.457186	2633.335021	92.53232155	6.592851263	0.001403199
4.79_723.1660m/z	723.1660363	4.792316667	neg	DAF-2	64883	Unclassified	Unclassified	Unclassified			36.3	0	2M-H	C20H14N2O5	-9.984254031	870.3096701	1547.904405	1810.519298	3522.259122	3210.555267	3477.543495	568.4981965	920.384625	1046.831996
4.88_403.1976m/z	403.1975577	4.880083333	neg	Flunarizine	HMDB0015589	Benzenoids	Benzene and substituted derivatives	Diphenylmethanes			36.3	0	M-H	C26H26F2N2	-3.886960208	3140.016347	3617.532307	3658.896684	1226.991164	1049.401956	1353.74351	2222.038337	2491.985596	2764.465459
4.88_452.1293n	451.1220486	4.880083333	neg	Catechin-4-ol 3-O-alpha-L-rhamnopyranoside	47417	Unclassified	Unclassified	Unclassified			36.3	0	M-H, M+FA-H	C21H24O11	-5.61011957	16955.71877	17324.71808	17890.07338	23126.11028	23391.72851	22835.21108	20191.02058	20140.07289	19081.98873
4.90_349.1021m/z	349.102065	4.902316667	neg	N-Acetyldemethylphosphinothricin tripeptide	63601	Unclassified	Unclassified	Unclassified			36.3	0	M-H	C12H21N3O7P	-6.769946941	5399.192009	5421.497248	5824.670961	6507.301185	7412.982967	7029.152004	5161.34675	6129.369293	5933.780589
4.92_558.1354n	603.1314424	4.92485	neg	Piceatannol 4'-galloylglucoside	95318	Phenylpropanoids and polyketides	Stilbenes	Stilbene glycosides			36.3	0	M-H, M+FA-H	C27H26O13	-3.553379109	8706.533142	9035.463774	9138.771245	0.001403199	0.001403199	0.001403199	969.4692682	1033.688499	1404.792038
4.95_529.1300m/z	529.1300317	4.94705	neg	Isoniazid alpha-ketoglutaric acid	HMDB0060663	Organoheterocyclic compounds	Pyridines and derivatives	Pyridinecarboxylic acids and derivatives	**	C16625	36.3	0	2M-H	C11H11N3O5	-4.589058076	627.628165	704.3877774	995.6447938	474.4867492	578.4047041	475.1730314	666.4527534	995.7880863	828.0838913
5.01_484.0621n	483.0547804	5.014716667	neg	alpha,beta-Dihydroxyethyl-TPP	69564	Unclassified	Unclassified	Unclassified			36.3	0	M-H, M+FA-H	C14H22N4O9P2S	7.817872346	909.7315772	1110.535469	634.0939551	5271.253033	6989.456345	6721.521154	1798.401701	1625.774199	1037.697673
5.06_232.0594m/z	232.0594109	5.05975	neg	N-Phenylacetylaspartic acid	86216	Organic acids and derivatives	Carboxylic acids and derivatives	Amino acids, peptides, and analogues	*		36.3	0	M-H2O-H	C12H13NO5	-8.445537955	1302.594648	1376.694105	1353.00974	1689.769486	1635.958425	2055.352728	1634.549699	1432.105544	1405.485016
5.18_489.1520m/z	489.1520179	5.1777	neg	5'-Deoxy-5-fluorocytidine	71275	Nucleosides, nucleotides, and analogues	5'-deoxyribonucleosides	Unclassified	**	C16635	36.3	0	2M-H	C9H12FN3O4	-6.271170834	1038.168895	1302.482594	1439.018098	1745.0423	2068.195869	1668.7654	1171.448448	1658.208068	1334.436252
5.22_512.1363m/z	512.1363134	5.222166667	neg	Neoacrimarine F	87415	Organoheterocyclic compounds	Quinolines and derivatives	Benzoquinolines	*		36.3	0	M-H2O-H	C29H25NO9	2.302853665	5230.352355	4125.066135	5378.726864	4899.969035	4603.752598	4607.119602	6685.00252	6099.088015	6576.912707
5.22_699.1087m/z	699.1087127	5.222166667	neg	Tricetin 7,3'-diglucuronide	49271	Lipids and lipid-like molecules	Polyketides	Flavonoids			36.3	0	M+FA-H	C27H26O19	5.627850371	16519.7513	15134.27152	13846.16328	16039.9902	13889.5806	13002.1966	19603.07406	18202.86205	21367.23844
5.30_534.1144m/z	534.1143783	5.302883333	neg	Citicoline	HMDB0001413	Nucleosides, nucleotides, and analogues	Pyrimidine nucleotides	Pyrimidine ribonucleotides	**	C00307	36.3	0	M+FA-H	C14H27N4O11P2+	2.084325955	1328.732393	1910.80128	2724.684665	1633.552913	2227.694414	2103.502996	1301.729217	1237.562596	2332.798562
5.36_481.1309m/z	481.1308786	5.361783333	neg	Epiafzelechin 3-O-beta-D-allopyranoside	47242	Lipids and lipid-like molecules	Polyketides	Flavonoids			36.3	0	M+FA-H	C21H24O10	-9.793378709	49247.41253	48094.76061	48474.21341	19986.06173	22520.29541	21181.69062	51456.81634	46424.49859	53646.70147
5.45_437.0916m/z	437.0916289	5.451166667	neg	Hosloppin	48619	Lipids and lipid-like molecules	Polyketides	Flavonoids			36.3	0	M+FA-H	C22H16O7	9.750734941	13570.60202	12031.11029	13020.39751	14781.24388	17725.93627	17462.11715	2400.58354	1802.331493	2545.359497
5.45_597.0929m/z	597.0928778	5.451166667	neg	Epitheaflagallin 3-O-gallate	89146	Benzenoids	Benzene and substituted derivatives	Benzoic acids and derivatives			36.3	0	M+FA-H	C27H20O13	7.759980343	23470.57439	20256.91867	22430.81946	16693.15335	17141.64601	17205.23902	2283.229055	1357.799581	1399.733087
5.56_813.2157m/z	813.2157463	5.56185	neg	Kaempferol 3-[2''-glucosyl-6''-acetyl-galactoside] 7-glucoside	92175	Phenylpropanoids and polyketides	Flavonoids	Flavonoid glycosides			36.3	0	M-H	C35H42O22	7.675828826	24062.41158	19629.59615	21596.20102	30500.95606	39940.35765	32857.93119	22601.30965	25025.63078	19977.7652
5.60_333.0935m/z	333.0934892	5.602	neg	Methitural	66681	Unclassified	Unclassified	Unclassified			36.3	0	M+FA-H	C12H20N2O2S2	-4.628810127	5397.051588	5209.244194	6436.434825	12132.80005	12602.37231	12002.82062	5751.988171	5840.042419	4801.02767
5.65_547.1455m/z	547.1455417	5.650833333	neg	5'-Hydroxycastavinol	86519	Phenylpropanoids and polyketides	Flavonoids	Flavans	*		36.3	0	M-H2O-H	C26H30O14	-0.305302791	4513.0049	4055.220917	4860.084403	2141.527674	1955.822228	2027.469204	721.4539687	577.4947964	730.9865491
5.69_456.1032n	501.1020942	5.6875	neg	Flavin mononucleotide	HMDB0001520	Nucleosides, nucleotides, and analogues	Flavin nucleotides	Unclassified	**	C00061	36.3	0	M-H, M+FA-H	C17H21N4O9P	-3.010426706	4194.985595	3781.912595	3639.185593	0.001403199	0.001403199	0.001403199	0.001403199	0.001403199	0.001403199
6.01_597.2091m/z	597.2091179	6.007266667	neg	Chenodeoxycholic acid disulfate	57976	Lipids and lipid-like molecules	Steroids and steroid derivatives	Bile acids, alcohols and derivatives	*		36.3	0	M+FA-H	C24H40O10S2	8.376559877	4249.454423	4304.558504	4582.388829	4041.426379	4354.249556	4590.825331	3951.247596	3554.149893	3480.877903
6.03_425.1821m/z	425.18207	6.028183333	neg	Cinncassiol C1	71839	Unclassified	Unclassified	Unclassified			36.3	0	M+FA-H	C20H28O7	0.957204384	16279.08699	18421.18969	17605.33822	10527.6052	11870.01369	10892.55945	14150.84539	15726.69868	15866.02595

6.14_193.0905m/z	193.0904913	6.143466667	neg	Burimamide	66645	Unclassified	Unclassified	Unclassified			36.3	0	M-H2O-H	C9H16N4S	-5.823029972	624.5979608	807.955922	624.7899298	652.3721157	731.8868927	779.0278415	751.0927265	691.6565023	725.0308281
6.15_533.0894m/z	533.0894003	6.154666667	neg	Bilobetin	87220	Phenylpropanoids and polyketides	Flavonoids	Biflavonoids and polyflavonoids	*		36.3	0	M-H2O-H	C31H20O10	2.888125286	15126.66165	16384.35551	15993.47063	9307.050554	10451.07844	11643.84976	9596.885927	10869.72899	11964.13913
6.17_261.1167m/z	261.1167308	6.171316667	neg	N-HISTIDYL-2-AMINONAPHTHALENE (βNA)	44316	Unclassified	Unclassified	Unclassified			36.3	0	M-H2O-H	C16H16N4O	7.713489772	404.7173176	396.8079826	464.5094065	191.1720158	326.2894998	382.6041875	421.5701799	413.1280327	477.81076
6.32_497.1260m/z	497.1259722	6.320716667	neg	7-Hydroxybutylidenephthalide 7-(6-malonylglucoside)	90179	Organic oxygen compounds	Organooxygen compounds	Carbohydrates and carbohydrate conjugates			36.3	0	M+FA-H	C21H24O11	-9.051075455	8392.362427	9573.352066	10460.80088	12657.00855	12752.91052	11145.26456	7232.570072	7745.57952	9369.591189
6.35_323.1481m/z	323.1480581	6.348783333	neg	Diisobutyl phthalate	70692	Benzenoids	Benzene and substituted derivatives	Benzoic acids and derivatives	*	C15205	36.3	0	M+FA-H	C16H22O4	-7.024601405	1293.830809	1549.900345	1355.091821	2359.315245	2363.569201	2348.54398	1169.560883	1083.054338	1355.097955
6.37_356.0755m/z	356.0755037	6.373833333	neg	Furoparadine	95520	Organoheterocyclic compounds	Quinolines and derivatives	Benzoquinolines			36.3	0	M+FA-H	C17H13NO5	-6.659647088	1472.647963	1670.326461	2001.890917	1962.569046	2849.272337	2535.492	2871.6405	3311.920504	3105.958037
6.43_450.1162m/z	450.1162386	6.433933333	neg	Bucladesine	HMDB0249421	Unclassified	Unclassified	Unclassified			36.3	0	M-H2O-H	C18H24N5O8P	-4.624760898	2020.96471	1709.199349	1473.079845	3182.46296	2719.491027	2892.284219	2026.281027	2192.689244	1939.276195
6.51_320.1298n	365.1229824	6.509966667	neg	Enoxacin	HMDB0014610	Organoheterocyclic compounds	Diazines	Pyrimidines and pyrimidine derivatives		C06979	36.3	0	M-H, M+FA-H	C15H17FN4O3	4.012391609	3792.497681	3052.288739	3955.904603	4510.418432	4686.613159	4742.430001	4009.387789	4501.811874	4577.15124
6.58_477.0994m/z	477.0993664	6.582416667	neg	Naphthofluorescein	45440	Unclassified	Unclassified	Unclassified			36.3	0	M+FA-H	C28H16O5	3.216558124	5625.575672	5928.696007	5497.000179	11594.37912	12136.68486	11508.82356	6669.971762	8496.236987	7749.979585
6.62_467.1149m/z	467.1148811	6.61635	neg	MDL 73492 sulfate	2370	Unclassified	Unclassified	Unclassified			36.3	0	M+FA-H	C19H22N2O7S	4.515753531	5390.59093	5185.629686	4931.814474	10147.16176	10024.40831	10368.93186	8186.98095	9734.172687	9732.315686
6.86_479.1151m/z	479.1151357	6.86375	neg	3'-O-Methyl-(-)-epicatechin 7-O-glucuronide	96020	Phenylpropanoids and polyketides	Flavonoids	Flavonoid glycosides	*		36.3	0	M-H	C22H24O12	-9.089326735	58890.78749	60323.18088	53762.4862	63215.58195	65437.78532	60891.42962	53884.4856	57473.26358	53278.96272
6.93_511.1458m/z	511.145838	6.926366667	neg	3-(4-Hydroxy-3-methoxyphenyl)-1,2-propanediol 2-O-(galloyl-glucoside)	91519	Benzenoids	Benzene and substituted derivatives	Benzoic acids and derivatives			36.3	0	M-H	C23H28O13	0.241027074	7033.21478	445.8779104	23427.32172	1076.998201	1185.618892	786.3950701	863.2720896	707.7776375	3776.992632
6.99_420.1078m/z	420.1077906	6.993516667	neg	3-Hydroxymethyltriazolopthalazinone glucuronide	3168	Unclassified	Unclassified	Unclassified			36.3	0	M+FA-H	C17H17N3O7	7.8319746	930.9727713	884.0226608	856.9138702	1711.947663	1552.856351	1749.350151	1298.182756	1309.280786	1100.284843
7.07_155.0715m/z	155.07148	7.0658	neg	(E,Z)-2,4-Heptadienal	HMDB0061891	Organic oxygen compounds	Organooxygen compounds	Carbonyl compounds			36.3	0	M+FA-H	C7H10O	1.019032632	983.6246442	1061.15551	1087.074727	1032.226449	982.1994735	821.4991708	983.2405146	914.7678563	988.3522036
7.16_378.1511m/z	378.1511236	7.1647	neg	Tiaprost	96518	Unclassified	Unclassified	Unclassified			36.3	0	M-H2O-H	C20H29O6S	1.208718333	1711.334693	1829.735101	1604.437592	2246.030287	1731.820928	1780.36679	1510.689134	2002.191174	1394.884277
7.18_452.1362m/z	452.1362142	7.178516667	neg	8-Carboxymethyldihydrochelerythrine	HMDB0029832	Alkaloids and derivatives	Benzophenanthridine alkaloids	Dihydrobenzophenanthridine alkaloids	*		36.3	0	M+FA-H	C23H21NO6	2.760548838	4169.251764	4891.010653	4540.502669	5251.265703	5350.3627	5956.14309	3571.921674	4972.749392	3666.337034
7.20_533.2267m/z	533.2267417	7.201166667	neg	Acarbose (M7)	762	Unclassified	Unclassified	Unclassified			36.3	0	M-H2O-H	C24H40O14	5.028266667	2886.987204	2687.222671	2720.925371	1602.152214	1854.299092	1782.51992	1536.425093	1734.106041	1369.013686
7.44_651.3006m/z	651.3005645	7.440866667	neg	(+)-Galeon	87743	Phenylpropanoids and polyketides	Diarylheptanoids	Cyclic diarylheptanoids	*		36.3	0	2M-H	C20H22O4	6.473367759	122495.0576	116630.4159	143468.1302	24248.26204	23372.2706	20929.44156	58941.19249	68395.98312	59467.77337
7.92_805.1871m/z	805.1871242	7.91555	neg	4-Methylpentyl glucosinolate	HMDB0038420	Organic oxygen compounds	Organooxygen compounds	Carbohydrates and carbohydrate conjugates			36.3	0	2M-H	C13H25NO9S2	0.314876882	12989.11859	13042.39098	14085.12818	17356.47012	17019.23227	17520.93202	12761.53565	17348.92098	15318.62791
8.07_813.1897m/z	813.1897374	8.074883333	neg	S-Succinylglutathione	65921	Unclassified	Unclassified	Unclassified			36.3	0	2M-H	C14H21N3O9S	-3.299855414	9541.603452	10279.66706	9506.236858	1276.799843	721.3501869	149.5764989	3984.616201	4671.486609	4714.787203
8.55_388.2342m/z	388.2342215	8.55215	neg	Geranylgeranylcysteine	62417	Lipids and lipid-like molecules	Prenol lipids	Diterpenoids	*		36.3	0	M-H2O-H	C23H37NO3S	6.501206806	1545.097904	1277.437028	1130.496772	1273.969041	1438.952566	1679.43669	1315.388617	1174.096831	1191.237865
9.02_443.2573m/z	443.2572543	9.01635	neg	1-(11Z,14Z-eicosadienoyl)-glycero-3-phosphate	HMDB0062303	Lipids and lipid-like molecules	Glycerophospholipids	Glycerophosphates	*		36.3	0	M-H2O-H	C23H43O7P	0.984437831	18786.35599	24973.58971	24477.54967	1520.54901	1239.831583	1520.882993	9236.819578	10096.56466	9361.884458
0.54_759.5182m/z	759.5181869	0.535283333	neg	PG(13:0/22:2(13Z,16Z))	LMGP04010090	Lipids and lipid-like molecules	Glycerophospholipids	Glycerophosphoglycerols			36.2	0	M-H	C41H77O10P	0.036913488	1243.480772	776.983049	1717.847597	0.001403199	0.001403199	0.001403199	1085.80816	0.001403199	307.0178196
0.67_222.9850m/z	222.9849558	0.6701	neg	2,3-Diketo-5-methylthio-1-phosphopentane	5947	Organic acids and derivatives	Organic phosphoric acids and derivatives	Phosphate esters	**	C15650	36.2	0	M-H2O-H	C6H11O6PS	5.790081159	929.1195523	905.959424	716.6777631	1035.900274	546.1459959	932.7205593	924.3477421	636.1822448	605.4467263
0.71_867.2912m/z	867.2911739	0.714216667	neg	Epimedin C	LMPK12112015	Lipids and lipid-like molecules	Polyketides	Flavonoids			36.2	0	M+FA-H	C39H50O19	-2.016785548	4852.303238	4497.821189	4798.141093	1977.908559	7881.032592	5300.588727	3837.749326	1120.031067	4445.72407
0.74_977.2583m/z	977.2583145	0.7359	neg	Pelargonidin 3-O-[2-O-(6-O-(E)-feruloyl-beta-D-glucopyranosyl)-beta-D-glucopy	46838	Unclassified	Unclassified	Unclassified			36.2	0	M+FA-H	C43H48O23	1.580956212	5814.222422	4108.693504	6450.39841	1570.879696	5647.839706	3435.175125	3640.715434	2297.65033	3071.652917
0.78_217.0484m/z	217.0483651	0.778533333	neg	Dillapional	87076	Organoheterocyclic compounds	Benzodioxoles	Unclassified	*		36.2	0	M-H2O-H	C12H12O5	-9.60437849	67100.02656	72357.38927	91100.41716	33285.01722	44480.09552	34280.25016	51750.45483	70709.8388	64639.40283
0.82_353.0532m/z	353.0532489	0.819566667	neg	2-Caffeoylisocitrate	65864	Unclassified	Unclassified	Unclassified			36.2	0	M-H	C15H14O10	5.165019711	2147.055394	2562.786426	2068.89633	1473.399311	1102.519654	953.8296566	1268.200808	1846.251228	895.2079358
0.82_410.0728n	409.0654975	0.819566667	neg	Zeanic acid	88862	Organoheterocyclic compounds	Quinolines and derivatives	Quinoline carboxylic acids			36.2	0	M-H, M+FA-H	C20H14N2O8	-5.46603209	27260.5869	28274.85379	27279.3153	45012.33766	41798.90031	40745.18589	35301.05448	32997.36126	31054.72052
0.88_194.0274m/z	194.0273825	0.884633333	neg	2-Methylbenzothiazole	88895	Organoheterocyclic compounds	Benzothiazoles	Unclassified			36.2	0	M+FA-H	C8H7NS	-4.96931224	0.001403199	204.0744442	206.5894716	177.7650496	327.5443055	226.6110696	0.001403199	112.660723	68.82521557
0.90_287.9778n	286.9705278	0.904683333	neg	2,6-Naphthalenedisulfonic acid	71139	Unclassified	Unclassified	Unclassified			36.2	0	M-H, 2M-H	C10H8O6S2	5.466745732	156070.1323	164432.5808	167915.0548	163929.6605	196850.689	170191.6107	177056.3275	147927.0677	183356.4182
0.93_188.9997m/z	188.9997236	0.925866667	neg	1-Naphthalenesulfonic acid	71143	Unclassified	Unclassified	Unclassified			36.2	0	M-H2O-H	C10H8O3S	-8.895291161	91.1170697	132.1684445	240.0178687	0.001403199	0.001403199	0.001403199	0.001403199	78.88114144	1.02653693
0.97_219.0118m/z	219.0118355	0.967616667	neg	Phenylmethanesulfonyl fluoride	66517	Unclassified	Unclassified	Unclassified			36.2	0	M+FA-H	C7H7FO2S	-8.309767079	0.001403199	901.7731491	1472.137914	8584.247791	4915.206541	7681.867905	1393.893777	4127.691623	3151.813726
1.16_286.9705m/z	286.9704733	1.160166667	neg	1,6-Naphthalenedisulfonic acid	71138	Unclassified	Unclassified	Unclassified			36.2	0	M-H	C10H8O6S2	5.277428706	87325.02269	128107.3554	114826.5274	132075.301	103419.881	105292.2674	122058.0393	92166.25652	102962.14
1.68_331.0785m/z	331.0785244	1.68365	neg	Ethyl phenothiazine-2-carbamate	1352	Unclassified	Unclassified	Unclassified			36.2	0	M+FA-H	C15H14N2O2S	9.517827914	621.524724	693.7492065	701.0762459	837.4273905	791.2861401	706.8103962	683.068648	779.4647865	728.4991357
1.76_446.1421m/z	446.1420945	1.7577	neg	N-Desethylquinagolide sulfate	2214	Unclassified	Unclassified	Unclassified			36.2	0	M-H	C18H29N3O6S2	-0.909550861	1444.128922	1481.885001	1530.361751	1283.220665	1389.151848	1536.457138	1005.079095	1552.211439	1144.338668
1.79_215.0539m/z	215.0538994	1.7861	neg	1-Isopropyl citrate	88461	Organic acids and derivatives	Carboxylic acids and derivatives	Tricarboxylic acids and derivatives			36.2	0	M-H2O-H	C9H14O7	-9.451239024	708.5693684	700.2056757	709.6487262	710.3205663	524.131308	829.8875647	782.0907321	652.8963885	634.9515472
10.43_701.5128m/z	701.5127622	10.42685	neg	PA(20:1(11Z)/16:0)	LMGP10010532	Lipids and lipid-like molecules	Glycerophospholipids	Glycerophosphates	*		36.2	0	M-H	C39H75O8P	0.11758458	14427.87737	12466.03835	11061.21464	15256.90423	16659.86121	15843.10691	13282.98409	15464.26958	15410.76078
11.05_481.0389m/z	481.0389302	11.05113333	neg	C.I. Food Red 1	HMDB0033383	Benzenoids	Naphthalenes	Naphthalene sulfonic acids and derivatives			36.2	0	M+FA-H	C18H16N2O7S2	1.913811727	6910.375176	7534.241455	6604.606498	6440.119483	8202.629774	8213.509998	7963.824232	8095.45637	7600.385697
2.00_300.0814m/z	300.0813847	2.004766667	neg	Ro 19-4603	69655	Unclassified	Unclassified	Unclassified			36.2	0	M-H2O-H	C15H17N3O3S	0.512080768	4353.283558	4223.983816	4138.118316	2847.845988	2360.265974	2494.983544	2875.449409	3222.26313	2540.806624
2.00_409.0370m/z	409.0370002	2.004766667	neg	XMP	3505	Nucleosides, nucleotides, and analogues	Purine nucleotides	Purine ribonucleotides	**	C00655	36.2	0	M+FA-H	C10H13N4O9P	-8.837730873	2835.8111	2689.178856	2585.658812	1733.544265	1627.581555	1698.284292	1718.019474	2218.85504	2017.903628
2.28_300.0468m/z	300.0467522	2.2828	neg	N-Acetyl-D-mannosamine 6-phosphate	63213	Organic oxygen compounds	Organooxygen compounds	Carbohydrates and carbohydrate conjugates	**	C04257	36.2	0	M-H	C8H16NO9P	-7.437391842	7467.312651	7036.540046	6537.641977	11944.89804	11106.18703	11167.714	10072.6481	10486.73642	9524.738531
2.28_368.0343m/z	368.0342665	2.2828	neg	EPN	70057	Unclassified	Unclassified	Unclassified			36.2	0	M+FA-H	C14H14NO4PS	-6.351897285	9723.3553	9987.206563	9385.519272	15638.01745	13818.89696	14514.58161	12150.81418	12948.9396	12744.07557
3.06_354.0785m/z	354.0784798	3.05735	neg	Deacetylcephalosporin C	64542	Unclassified	Unclassified	Unclassified			36.2	0	M-H2O-H	C14H19N3O7S	5.226363186	7999.806246	7916.155094	8934.016716	18902.72625	19935.64818	17836.64644	13882.13182	13684.32674	12777.48522
3.29_227.9706n	272.9687792	3.293883333	neg	Ammonium peroxydisulfate	92479	Unclassified	Unclassified	Unclassified			36.2	0	M+FA-H, 2M-H, M-H	H8N2O8S2	-7.153979098	132981.8471	134493.4894	135610.7971	25028.22213	24435.93866	25050.20931	107011.6046	117665.3062	113002.6111
3.35_450.0882m/z	450.0882458	3.350266667	neg	4'-Desmethylpapaverine sulfate	1707	Unclassified	Unclassified	Unclassified			36.2	0	M+FA-H	C19H19NO7S	4.492732492	11506.76628	13891.35242	11781.89458	16488.32267	15732.68565	15934.6715	12569.54663	15165.1478	12579.51399
3.66_248.9962m/z	248.9962188	3.6635	neg	4-Hydroxyriluzole	2323	Unclassified	Unclassified	Unclassified			36.2	0	M-H	C8H5F3N2O2S	4.448578582	145.8562212	121.0800773	341.5635321	0.001403199	0.001403199	0.001403199	98.33276461	107.8632431	128.405294
3.66_329.0865m/z	329.0864813	3.6635	neg	Musca-aurin-VII	67066	Unclassified	Unclassified	Unclassified			36.2	0	M-H2O-H	C15H16N4O6	-7.64657666	2486.94325	2555.123271	2951.944505	4060.40768	4259.439129	3268.799866	2569.342625	2711.437849	2937.29134
3.69_198.0602m/z	198.0602289	3.685866667	neg	1,3,7-Trimethyluric Acid - 13C4,15N3 (2,4,5,6-13C4, 1,3,9-15N3)	103469	Unclassified	Unclassified	Unclassified			36.2	0	M-H2O-H	C[13]4C[12]4H10N[15]3N[14]O3	-8.034378481	32.90167972	0.001403199	0.001403199	65.55196139	57.40044798	56.46736448	33.39540072	28.71578121	0.001403199
3.85_355.0627m/z	355.0627215	3.84605	neg	Neodiospyrin	86326	Benzenoids	Naphthalenes	Naphthoquinones	*		36.2	0	M-H2O-H	C22H14O6	4.075113228	501.5417248	521.9485607	673.9072444	721.7013743	513.7164758	758.2974005	496.3693818	325.2773628	720.8443767
3.89_265.0136m/z	265.0135758	3.8906	neg	Cassic acid	LMPK13040015	Benzenoids	Anthracenes	Anthracenecarboxylic acids and derivatives		C10401	36.2	0	M-H2O-H	C15H8O6	-2.362661645	39.3087015	0.001403199	45.83021693	87.99344664	89.84216615	64.08006499	61.22222215	68.97674488	143.9362048
3.91_374.1574m/z	374.1573926	3.912566667	neg	AM2201 6-hydroxyindole metabolite	96610	Unclassified	Unclassified	Unclassified			36.2	0	M-H	C24H22FNO2	3.230354145	556.4639121	653.0461969	499.5893314	608.4767543	680.3907769	617.5385092	465.7132246	523.4375256	534.979306
3.91_450.9826m/z	450.9826463	3.912566667	neg	2,2'-Dithenyl sulfide	HMDB0032982	Organoheterocyclic compounds	Heteroaromatic compounds	Unclassified			36.2	0	2M-H	C10H10S3	2.203845979	4812.841301	4740.852333	5168.599355	6567.370288	5920.328066	5469.318042	5655.772324	5886.135093	6072.073738
4.02_214.0335m/z	214.0335326	4.024283333	neg	Brassicanal B	93257	Organoheterocyclic compounds	Indoles and derivatives	Indoles			36.2	0	M-H2O-H	C12H11NO2S	1.390673239	173.9492105	233.2502625	233.7426077	454.0653693	564.8525644	441.557808	357.5235934	412.4619232	418.229798
4.05_450.9963m/z	450.9963378	4.046666667	neg	Sanguisorbic acid dilactone	HMDB0029274	Phenylpropanoids and polyketides	Tannins	Hydrolyzable tannins			36.2	0	M-H2O-H	C21H10O13	4.337223935	2627.528568	2792.899418	3003.532587	4824.757104	4398.544675	4294.224124	3133.799985	3857.391321	3584.109556
4.07_385.1139m/z	385.1139182	4.069216667	neg	4-O-beta-D-Glucosyl-sinapate	65860	Unclassified	Unclassified	Unclassified			36.2	0	M-H	C17H22O10	-0.264848078	152.4525431	384.6150232	385.1822025	125.9702625	1.163223778	58.5885272	808.5048321	922.396463	687.0451691
4.11_451.0714m/z	451.0714023	4.113883333	neg	(Z)-Resveratrol 3-(6''-sulfoglucopyranoside)	HMDB0037077	Phenylpropanoids and polyketides	Stilbenes	Stilbene glycosides			36.2	0	M-H2O-H	C20H22O11S	2.044085867	1133.973809	1284.570036	1346.65889	1791.263189	1384.451343	1550.449117	1586.499881	1438.248287	1514.107768
4.14_285.0958m/z	285.0957606	4.136366667	neg	Isosalicin	89665	Organic oxygen compounds	Organooxygen compounds	Carbohydrates and carbohydrate conjugates			36.2	0	M-H	C13H18O7	-7.744932595	3182.100049	3359.438542	3715.323951	2901.11393	2507.266028	2549.781423	2730.547699	2710.111407	2503.543974
4.14_371.0444m/z	371.0444172	4.136366667	neg	Dothistromin	68254	Unclassified	Unclassified	Unclassified			36.2	0	M-H	C18H12O9	9.573212218	1089.32601	1118.914878	1282.15935	169.3328546	138.936115	162.7440875	2073.760199	1858.782956	2217.611009
4.16_183.0493m/z	183.0492578	4.15895	neg	2-Phenylethanethiol	88580	Benzenoids	Benzene and substituted derivatives	Unclassified	*		36.2	0	M+FA-H	C8H10S	5.314390816	69.15536669	31.05632032	50.9936853	83.93115506	74.19421756	110.1683412	77.42784291	76.41923018	53.52988935
4.21_452.0587m/z	452.0587351	4.212116667	neg	o-O-sulfate rosiglitazone	HMDB0060858	Organic acids and derivatives	Organic sulfuric acids and derivatives	Arylsulfates	*		36.2	0	M-H	C18H19N3O7S2	-0.949982322	552.7367631	583.6257468	496.0787846	860.1728555	760.3623086	1000.640763	675.6449098	851.6037771	678.6289968
4.52_240.0863m/z	240.0863424	4.521733333	neg	Proacacipetalin	66917	Unclassified	Unclassified	Unclassified			36.2	0	M-H2O-H	C11H17NO6	-5.417716932	1065.07237	731.9695448	407.743788	475.8344047	665.6911389	753.4822887	931.4561497	927.0759505	983.1829
4.70_328.0417m/z	328.0417379	4.703166667	neg	N2-Acetyl-L-aminoadipyl-δ-phosphate	63445	Unclassified	Unclassified	Unclassified			36.2	0	M+FA-H	C8H14NO8P	-7.659685739	1182.494555	1055.262484	1265.328725	275.4014007	347.196718	348.3249011	1304.53959	1334.187127	1544.940594
4.81_481.1319m/z	481.131942	4.814166667	neg	Arthromerin B	47243	Lipids and lipid-like molecules	Polyketides	Flavonoids			36.2	0	M+FA-H	C21H24O10	-7.355096077	7699.513676	8058.870979	7753.983872	5791.286849	5299.826381	5663.431701	8062.0529	8889.948625	8047.138714
4.88_474.2527m/z	474.2527463	4.880083333	neg	Mebeverine	1078	Unclassified	Unclassified	Unclassified			36.2	0	M+FA-H	C25H35NO5	7.035975444	2243.807727	2995.945899	2490.539693	2506.283983	2570.097048	2307.763041	2997.496093	3513.545821	3073.005184
4.90_615.1035m/z	615.1035293	4.902316667	neg	Delphinidin 3-(2''-galloylgalactoside)	47032	Unclassified	Unclassified	Unclassified			36.2	0	M-H	C28H24O16	7.094705204	14565.01548	15175.74487	16513.51658	14697.28468	13655.23566	14708.66084	14623.24462	14702.93123	10982.65769
4.95_425.0920m/z	425.0919629	4.94705	neg	Formononetin 7-O-glucuronide	96093	Phenylpropanoids and polyketides	Isoflavonoids	Isoflavonoid O-glycosides	*		36.2	0	M-H2O-H	C22H20O10	9.360878138	8868.717679	9568.411648	9534.558657	2742.895212	2735.33001	2702.739073	1752.528872	1405.28836	1464.551572
4.97_410.1800m/z	410.1799584	4.969683333	neg	Abiraterone sulfate	HMDB0060584	Lipids and lipid-like molecules	Steroids and steroid derivatives	Sulfated steroids	*		36.2	0	M-H2O-H	C24H31NO4S	0.978632108	1674.641833	2788.667289	3054.215249	1492.421589	1899.69018	1275.505419	1843.358482	1482.039217	1951.099986
4.99_252.0855m/z	252.0855086	4.99225	neg	Deidaclin	66908	Unclassified	Unclassified	Unclassified			36.2	0	M-H2O-H	C12H17NO6	-8.253290862	795.1656397	875.5141266	907.2637401	229.1737602	205.132162	308.5367798	1070.948905	853.7327181	786.8357309
4.99_341.0234m/z	341.023389	4.99225	neg	Glucoputranjivin	HMDB0301840	Unclassified	Unclassified	Unclassified			36.2	0	M-H2O-H	C10H18NO9S2-	-2.96638151	973.1221441	1294.744414	1150.816187	1026.15636	1259.244614	1215.337685	951.7927228	653.0293104	551.6193722
4.99_343.0844m/z	343.0843929	4.99225	neg	8-Demethylsideroxylin	49445	Phenylpropanoids and polyketides	Flavonoids	O-methylated flavonoids	*		36.2	0	M+FA-H	C17H14O5	6.932490896	834.9301954	801.8053547	805.359416	759.6015457	1098.570224	967.1462432	1314.489332	1023.244879	1039.390422
5.01_471.0976m/z	471.0975767	5.014716667	neg	Cyanidin 3-(6-acetylgalactoside)	46876	Phenylpropanoids and polyketides	Flavonoids	Flavonoid glycosides	*		36.2	0	M-H2O-H	C23H22O12	8.756563738	820.3123391	730.3913519	1035.53197	2124.195359	2399.739427	2279.045649	433.99643	849.0960524	565.6913253
5.08_347.1715m/z	347.1715065	5.080566667	neg	(1S,2S,4R)-p-Menth-8-ene-1,2,10-triol 2-glucoside	94041	Lipids and lipid-like molecules	Prenol lipids	Terpene glycosides	*		36.2	0	M-H	C16H28O8	1.048516684	762.3629356	853.2236716	881.4491107	734.1248969	589.2226286	911.4376201	827.8399321	1089.022644	775.8364962
5.18_217.0484m/z	217.0483541	5.1777	neg	Methoxsalen Metabolite	1209	Unclassified	Unclassified	Unclassified			36.2	0	M-H2O-H	C12H12O5	-9.650675217	585.8918973	670.1779924	550.4160962	1183.068971	1239.710197	1027.386407	699.3914698	721.0453918	835.7458076
5.18_247.0824m/z	247.0823583	5.1777	neg	3-Deazaadenosine	HMDB0245849	Nucleosides, nucleotides, and analogues	Purine nucleosides	Unclassified	*	C01736	36.2	0	M-H2O-H	C11H14N4O4	-4.906196193	205.7218713	478.3866273	247.0895521	0.001403199	0.001403199	0.001403199	155.3516709	46.34148216	368.7483087
5.24_297.1322m/z	297.1321821	5.244033333	neg	Sesamex	72887	Unclassified	Unclassified	Unclassified			36.2	0	M-H	C15H22O6	-7.311624763	1565.013214	1508.928654	1418.45795	2335.687304	2247.390055	2130.266916	1200.859391	1217.346617	1516.71496
5.29_365.0858m/z	365.0857697	5.28555	neg	4-p-Coumaroyl-1,5-quinolactone	86164	Phenylpropanoids and polyketides	Cinnamic acids and derivatives	Hydroxycinnamic acids and derivatives	*		36.2	0	M+FA-H	C16H16O7	-6.360664635	0.001403199	132.4677974	127.0342625	1430.74057	1674.594703	1807.06386	2971.505757	2458.869674	2865.725571
5.47_267.0851m/z	267.0851446	5.471616667	neg	Methylarbutin	71805	Unclassified	Unclassified	Unclassified			36.2	0	M-H2O-H	C13H18O7	-7.924208634	1619.360411	1628.664171	1745.788986	1543.052608	1341.250156	1738.602857	1588.763008	1435.762076	1196.144283
5.56_735.1997m/z	735.1997218	5.56185	neg	Penicilloic acid V	1740	Unclassified	Unclassified	Unclassified			36.2	0	2M-H	C16H20N2O6S	-1.924083422	6610.074979	5572.742603	4838.746552	8816.386822	10662.28981	9021.127507	5069.988909	5670.235522	4697.556786
5.56_803.1871m/z	803.1870719	5.56185	neg	Pelargonidin 3-arabinoside	46856	Unclassified	Unclassified	Unclassified			36.2	0	2M-H	C20H18O9	5.202720492	4746.775056	4467.660515	3921.504027	5758.236828	7176.65531	5832.712963	3474.727583	3695.673371	2505.63828
5.92_377.0804m/z	377.0804396	5.9168	neg	N-Acetylisatin	63543	Unclassified	Unclassified	Unclassified			36.2	0	2M-H	C10H7NO3	6.691154022	280.4385148	123.0505472	49.11959182	333.5597722	242.7947143	298.3344954	131.4473721	288.516721	194.072784
6.03_259.0589m/z	259.0588722	6.028183333	neg	(S)-ACPA	69626	Unclassified	Unclassified	Unclassified			36.2	0	M+FA-H	C8H10N2O5	7.931747545	373.4784102	518.3461788	455.1312534	1114.473035	1024.435372	858.4265005	516.0495513	643.0894222	599.6853858
6.17_320.1531m/z	320.1530941	6.171316667	neg	4,5-Dihydropiperlonguminine	95874	Organoheterocyclic compounds	Benzodioxoles	Unclassified	*		36.2	0	M+FA-H	C16H21NO3	9.986000123	144.105643	47.08749231	78.17916177	998.25915	1079.22455	1195.624681	213.0591604	286.8272407	229.8209798
6.20_527.2477m/z	527.2476882	6.195716667	neg	ent-6R,16bOH,17-Trihydroxy-7-oxo-6,7-seco-19,6-kauranolide 6-O-glucoside	HMDB0038546	Lipids and lipid-like molecules	Prenol lipids	Terpene glycosides	*		36.2	0	M-H	C26H40O11	-3.970536038	3269.633996	2563.334537	3606.128948	2803.562838	2275.33826	2988.971783	3190.084657	3951.165472	4541.897653
6.27_279.1214m/z	279.1213733	6.272366667	neg	2,6-Diamino-7-hydroxy-azelaic acid	69262	Unclassified	Unclassified	Unclassified			36.2	0	M+FA-H	C9H18N2O5	6.82859784	377.5087996	329.5473368	288.2998774	760.1329098	825.7302845	596.0288348	328.9722335	456.7163637	461.3853375
6.41_613.1180m/z	613.1180264	6.408833333	neg	Chrysophanol 8-(6-galloylglucoside)	92063	Benzenoids	Anthracenes	Anthraquinones			36.2	0	M+FA-H	C28H24O13	-3.28684254	4057.734998	4822.774085	5229.628843	2075.220374	1362.709927	2174.939511	599.9132572	521.6678925	312.7885514
6.49_345.1453m/z	345.1453449	6.494283333	neg	Bifenazate	72390	Unclassified	Unclassified	Unclassified			36.2	0	M+FA-H	C17H20N2O3	-0.834659492	2062.292367	2040.309313	2220.840378	2626.97517	2464.687596	2583.921347	2455.350968	2486.943669	2105.881694
6.62_333.1452m/z	333.1451981	6.61635	neg	N-(3-Indolylacetyl)-L-isoleucine	34533	Unclassified	Unclassified	Unclassified			36.2	0	M+FA-H	C16H20N2O3	-1.378656588	349.472704	546.8717463	517.0698461	1112.749042	956.3900761	978.7693278	919.257291	973.8999096	1073.736253
7.04_293.1376m/z	293.1376144	7.044716667	neg	Gamma-CEHC	58067	Organoheterocyclic compounds	Benzopyrans	1-benzopyrans	*		36.2	0	M+FA-H	C15H20O3	-7.386995172	2595.604704	2520.278503	2597.377636	4294.325786	4101.153665	3990.192562	2559.466409	2671.64024	2473.069698
7.11_259.0951m/z	259.0950712	7.114116667	neg	Oxypinnatanine	86246	Organic acids and derivatives	Carboxylic acids and derivatives	Amino acids, peptides, and analogues	*		36.2	0	M-H	C10H16N2O6	5.81094653	569.8498328	576.7033894	574.8213672	515.6271382	695.0037524	505.109955	443.6900795	443.1473757	474.9782984
7.15_358.0889m/z	358.0888517	7.147366667	neg	PI3-Kinase α Inhibitor 2	45221	Unclassified	Unclassified	Unclassified			36.2	0	M+FA-H	C16H15N3O2S	6.870570275	4284.307187	4850.498984	4801.754221	7409.513433	7729.497642	7507.90995	5093.926198	6288.121176	5928.97316
7.32_249.1109m/z	249.1108582	7.324733333	neg	N6-Acetyl-N6-hydroxy-L-lysine	63465	Unclassified	Unclassified	Unclassified			36.2	0	M+FA-H	C8H16N2O4	8.075626452	514.7958041	460.9794906	468.3628177	657.6132107	527.0741063	547.4913801	337.1864214	366.3866488	451.011443
7.39_329.2336m/z	329.2336156	7.3938	neg	Tanikolide	65441	Unclassified	Unclassified	Unclassified			36.2	0	M+FA-H	C17H32O3	0.942237038	2392.522319	2068.247545	1334.622636	1532.327813	954.050006	1189.866629	2045.163079	2732.180786	1361.637148
7.86_370.2235m/z	370.2234779	7.86405	neg	N-3-oxo-tetradecanoyl-L-Homoserine lactone	64792	Unclassified	Unclassified	Unclassified			36.2	0	M+FA-H	C18H31NO4	-0.10298667	361.1945173	367.4717128	185.0187524	167.3574088	507.52668	234.6926089	332.813776	112.9226456	418.2149753
8.02_289.0697m/z	289.0696535	8.024383333	neg	3,3',4'5-Tetrahydroxystilbene	HMDB0004215	Phenylpropanoids and polyketides	Stilbenes	Unclassified	***	C05901	36.2	0	M+FA-H	C14H12O4	-8.637783539	1473.622098	994.7365039	1313.724282	1560.089434	1686.268793	1764.166259	1518.687226	1258.631864	1355.864779
9.02_669.2018m/z	669.2017668	9.01635	neg	3,4',6'-Trihydroxy-4,2'-dimethoxychalcone 4'-O-rutinoside	LMPK12120304	Unclassified	Unclassified	Unclassified			36.2	0	M+FA-H	C29H36O15	-2.974154615	3762.077467	4712.051166	4624.54359	0.001403199	0.001403199	0.001403199	1541.591278	1451.692532	1275.748786
9.92_361.1636m/z	361.1635708	9.922166667	neg	Gibberellin A9	41232	Lipids and lipid-like molecules	Prenol lipids	Isoprenoids	**	C11863	36.2	0	M+FA-H	C19H24O4	-6.614666048	2034.857625	1745.36191	1393.495263	1280.653962	1005.33684	1720.307806	1363.704884	1236.409004	1875.479023
0.69_417.0483m/z	417.0482644	0.692166667	neg	NORSTICTIC ACID	43603	Unclassified	Unclassified	Unclassified			36.1	0	M+FA-H	C18H12O9	5.186346135	2069.965703	1256.92239	1874.082457	5159.113932	5147.796405	4642.737052	3125.39483	3494.400306	4199.568663
0.71_777.3575m/z	777.3575156	0.714216667	neg	MAM2201 N-(4-hydroxypentyl) metabolite	85212	Unclassified	Unclassified	Unclassified			36.1	0	2M-H	C25H24FNO2	8.450533992	4082.197139	3984.987601	3039.634099	3638.312249	4188.360485	3419.131087	5318.721183	2166.19273	3747.421814
0.82_322.0828m/z	322.0827879	0.819566667	neg	Indirubin-3'-monoxime	45531	Unclassified	Unclassified	Unclassified			36.1	0	M+FA-H	C16H11N3O2	-1.954700395	31878.25384	30369.18378	29381.14334	16883.95543	15736.35595	17823.5946	29278.38988	25771.06497	22493.77361
0.82_350.0416m/z	350.041579	0.819566667	neg	2',3' cyclic CMP	62429	Organic acids and derivatives	Carboxylic acids and derivatives	Amino acids, peptides, and analogues	**	C00127	36.1	0	M+FA-H	C9H12N3O7P	6.850718221	3579.214319	3535.567128	3977.60291	7407.479324	3972.888568	5588.38369	4370.818616	4649.21849	3294.600554
0.88_368.0078m/z	368.0078402	0.884633333	neg	2'-Deoxycytidine diphosphate (dCDP)	3514	Unclassified	Unclassified	Unclassified			36.1	0	M-H2O-H	C9H15N3O10P2	6.238390685	268.3921443	0.001403199	0.001403199	2108.033557	578.715143	858.5175606	956.8714861	1127.799812	355.8953437
1.14_217.0527m/z	217.0526542	1.1382	neg	2-(4'-Methylthio)butylmalic acid	64497	Unclassified	Unclassified	Unclassified			36.1	0	M-H2O-H	C9H16O5S	-5.715588253	661.0773019	1652.881711	1270.384925	2209.787829	1248.108734	1459.77812	2271.498844	1109.762596	1024.063711
1.18_240.0498m/z	240.0497636	1.180366667	neg	2-Hydroxy-6,7-dimethoxybenzoxazole	87153	Organoheterocyclic compounds	Benzoxazoles	Benzoxazolones	*		36.1	0	M+FA-H	C9H9NO4	-8.187740938	1388.950099	1974.521319	1770.625758	1469.608589	1598.71032	1324.266385	1486.769539	1309.331589	1263.57444
1.34_308.0812m/z	308.0811745	1.335933333	neg	Bisindolylmaleimide IV	45524	Unclassified	Unclassified	Unclassified			36.1	0	M-H2O-H	C20H13N3O2	-5.3839357	833.5880228	944.1257847	1040.499796	1147.314934	1276.011323	1155.506108	660.8335182	710.9402307	822.2312165
1.36_135.0313m/z	135.031303	1.358266667	neg	1-Pentanesulfenothioic acid	87453	Organosulfur compounds	Sulfenyl compounds	Unclassified	*		36.1	0	M-H	C5H12S2	3.947746584	243.7175037	224.0550948	170.6903067	162.3889842	192.079815	158.9058493	181.1683627	159.5461798	212.0928237
1.40_226.0335m/z	226.0334808	1.3958	neg	Stizolobate	63513	Unclassified	Unclassified	Unclassified			36.1	0	M-H	C9H9NO6	-9.820789393	1170.095102	612.4969131	616.3952245	804.2094048	768.4032163	743.0408048	808.8500733	744.2117615	770.5585529
1.55_224.9855m/z	224.9854548	1.553483333	neg	O-Coumaric acid sulfate	HMDB0240549	Phenylpropanoids and polyketides	Cinnamic acids and derivatives	Hydroxycinnamic acids and derivatives			36.1	0	M-H2O-H	C9H8O6S	-3.537046073	31.83770615	32.07990728	60.86454723	144.7398043	63.73795427	119.3153885	69.85826123	72.79097271	86.27846934
1.84_354.0244m/z	354.0244209	1.841666667	neg	SUCCINYLSULFATHIAZOLE	44631	Unclassified	Unclassified	Unclassified			36.1	0.0199	M-H	C13H13N3O5S2	5.731343955	22829.50443	22675.47101	28911.16568	19210.96576	18337.16001	19104.55887	32119.84744	27589.18357	27844.8562
10.01_311.1858m/z	311.1857655	10.00813333	neg	5-Hexyl-2-furanhexanoic acid	HMDB0112088	Lipids and lipid-like molecules	Fatty Acyls	Fatty acids and conjugates			36.1	0	M+FA-H	C16H26O3	-2.374498035	531.3515054	330.3981249	202.0018596	348.5250969	360.5066969	499.057137	349.8168801	315.1923506	464.040134

10.69_838.5603m/z	838.5603051	10.6877	neg	PC(22:5(7Z,10Z,13Z,16Z,19Z)/15:0)	HMDB0008691	Lipids and lipid-like molecules	Glycerophospholipids	Glycerophosphocholines	**	C00157	36.1	0	M+FA-H	C45H80NO8P	-0.066671377	13028.32078	11970.68475	11840.79809	8231.859384	5491.14463	6353.648934	10250.56585	11229.25683	7952.804608
11.14_593.1611m/z	593.1610764	11.13858333	neg	3,4-Dihydroxyrottlerin	52072	Lipids and lipid-like molecules	Polyketides	Flavonoids			36.1	0	M+FA-H	C30H28O10	-9.802680002	7397.098855	5752.84239	5325.298718	5944.835209	6656.246423	6161.321026	6805.105343	5470.293394	5302.836471
13.29_759.5179m/z	759.5178658	13.29488333	neg	PA(15:1(9Z)/22:1(11Z))	LMGP10010186	Lipids and lipid-like molecules	Glycerophospholipids	Glycerophosphates			36.1	0	M+FA-H	C40H75O8P	-0.4100879	1585.495237	2470.881923	2794.036803	0.001403199	0.001403199	0.001403199	1127.731147	1085.493668	2301.370587
2.07_330.0243m/z	330.0243157	2.070366667	neg	CAY10638	64838	Unclassified	Unclassified	Unclassified			36.1	0	M-H	C16H13NO3S2	-6.323180872	734.7047169	897.3212137	745.4552146	912.0363346	895.1806361	862.5019628	1204.711924	991.9526256	936.0627211
2.30_396.0550m/z	396.0549733	2.303733333	neg	5-Taurinomethyl-2-thiouridine	HMDB0011610	Nucleosides, nucleotides, and analogues	Pyrimidine nucleosides	Unclassified	*		36.1	0	M-H	C12H19N3O8S2	2.24950672	1575.808615	1470.220117	1283.49855	3362.703254	2922.549236	2633.020376	2278.35344	2519.057499	2484.661995
3.77_273.0756m/z	273.0755738	3.770416667	neg	Pemirolast	85441	Organoheterocyclic compounds	Pyridopyrimidines	Unclassified			36.1	0	M+FA-H	C10H8N6O	6.190893149	315.889523	217.6954388	253.7531323	447.4174015	465.1170014	320.60057	335.1607608	340.6606789	335.6026009
3.80_139.0401m/z	139.0401352	3.801716667	neg	2,4,5-Trihydroxytoluene	72152	Unclassified	Unclassified	Unclassified			36.1	0	M-H	C7H8O3	0.482408196	306.7135599	227.6814803	175.588682	290.2713859	287.4234105	253.836819	267.1782065	248.0700414	294.9580124
3.82_283.1301m/z	283.1300601	3.82415	neg	Arginyl-Glutamate	85623	Organic acids and derivatives	Carboxylic acids and derivatives	Amino acids, peptides, and analogues	*		36.1	0	M-H2O-H	C11H20N5O5-	4.82357849	543.9488574	679.0105082	691.5630137	1177.99256	995.3349059	1078.176083	687.9773927	787.9915314	809.8517115
3.91_463.0558m/z	463.0558131	3.912566667	neg	Shoyuflavone C	90053	Phenylpropanoids and polyketides	Isoflavonoids	Isoflav-2-enes			36.1	0	M+FA-H	C19H14O11	9.565645433	1890.585368	1816.375478	1884.58763	713.3872562	313.791548	665.9930315	3526.174578	3555.890929	4259.516626
3.96_479.1148m/z	479.1147912	3.957583333	neg	4'-O-Methyl-(-)-epicatechin 3'-O-glucuronide	96034	Phenylpropanoids and polyketides	Flavonoids	Flavonoid glycosides	*		36.1	0	M-H	C22H24O12	-9.806930218	11732.88778	10758.36271	12053.30327	12784.86653	11193.52268	11633.2233	11067.63455	11963.23973	11215.39327
4.14_247.0282m/z	247.0282293	4.136366667	neg	5-Hydroxysulfadiazine	2441	Unclassified	Unclassified	Unclassified			36.1	0	M-H2O-H	C10H10N4O3S	-4.851617752	1544.691751	1387.283632	1543.645187	338.0408503	385.7855565	375.6913097	1662.09766	1418.638265	1294.061865
4.23_249.1345m/z	249.1345113	4.234483333	neg	Histidinyl-Isoleucine	85794	Organic acids and derivatives	Carboxylic acids and derivatives	Amino acids, peptides, and analogues	*		36.1	0	M-H2O-H	C12H20N4O3	-4.430664986	294.3480381	304.4221367	272.5862093	362.5649799	295.5214014	481.7361604	280.4225357	287.6332065	231.055719
4.23_315.9970m/z	315.9969822	4.234483333	neg	AZINPHOS METHYL	44549	Unclassified	Unclassified	Unclassified			36.1	0	M-H	C10H12N3O3PS2	-4.76724291	1057.909079	981.5757113	1009.90356	1175.817563	978.6102498	814.6190602	1191.913196	1133.004328	1018.645809
4.32_391.1369m/z	391.1369099	4.3224	neg	Homoveratric acid	24093	Benzenoids	Benzene and substituted derivatives	Methoxybenzenes	*		36.1	0	2M-H	C10H12O4	-7.475333107	115.6666712	81.91696391	109.6089965	459.3878226	656.2598617	312.7102524	238.1738119	534.1336517	793.7379611
4.34_294.1022m/z	294.1021776	4.344733333	neg	Angustine	67367	Unclassified	Unclassified	Unclassified			36.1	0	M-H2O-H	C20H15N3O	-4.769484386	127.9748644	162.8662661	163.7462814	747.0043684	695.855031	819.7924741	295.2234211	299.5782623	315.9326388
4.48_352.1382m/z	352.1381518	4.477166667	neg	Phenylephrine 3-O-glucuronide	700	Unclassified	Unclassified	Unclassified			36.1	0	M-H2O-H	C17H25NO8	-5.452811194	1133.803592	1231.201144	1214.469216	649.4641897	718.0167083	560.1905068	1200.0812	1043.78697	1012.222248
4.50_415.1910m/z	415.1910392	4.49925	neg	Flunisolide	2736	Lipids and lipid-like molecules	Steroids and steroid derivatives	Hydroxysteroids		C07005	36.1	0	M-H2O-H	C24H31FO6	-3.653974365	897.6167036	642.3434583	810.2492694	461.880042	648.2606246	532.3909941	525.3036413	677.1647148	704.4076963
4.52_435.0908m/z	435.0908413	4.521733333	neg	5,3',5'-Trihydroxy-3,6,7,4'-tetramethoxyflavone	49045	Unclassified	Unclassified	Unclassified			36.1	0	M+FA-H	C19H18O9	-6.264549082	679.6700618	1015.586038	739.7272313	0.001403199	60.59890119	0.001403199	1497.314344	1601.537933	1702.54444
4.57_401.1645m/z	401.1644987	4.5668	neg	BAY-41-8543	64714	Unclassified	Unclassified	Unclassified			36.1	0	M-H2O-H	C21H21FN8O	0.248182987	1656.944125	1551.78937	1459.331018	1835.305906	2043.516537	1851.562597	1624.578279	1477.331649	1564.296507
4.70_314.1308m/z	314.1307598	4.703166667	neg	Pefloxacin	HMDB0014630	Organoheterocyclic compounds	Quinolines and derivatives	Quinoline carboxylic acids			36.1	0	M-H2O-H	C17H20FN3O3	-0.806958544	104.1159981	170.62769	76.70081583	201.6472019	212.9022872	284.2398168	82.57255711	189.1603165	180.5636059
4.75_252.0858m/z	252.0858065	4.747466667	neg	Dihydroferuloylglycine	96083	Benzenoids	Phenols	Methoxyphenols			36.1	0	M-H	C12H15NO5	-7.663742888	1168.727223	892.2747488	1030.215521	1684.897887	2259.416845	1871.510737	1360.296954	1545.555381	1063.949125
4.75_385.1124m/z	385.1124396	4.747466667	neg	Scandoside methyl ester	HMDB0252234	Unclassified	Unclassified	Unclassified			36.1	0	M-H2O-H	C17H24O11	-3.911809607	2165.443391	2506.924676	2655.514573	3173.366937	3112.099391	2910.768029	2096.152961	2343.792249	2690.43469
4.77_561.1590m/z	561.1590148	4.769883333	neg	albendazole sulfone	794	Organoheterocyclic compounds	Benzimidazoles	2-benzimidazolylcarbamic acid esters	*	C16626	36.1	0	2M-H	C12H15N3O3S	-0.948919569	894.9619753	627.3258727	912.4834491	0.001403199	0.001403199	0.001403199	0.001403199	204.0401764	269.1350047
4.81_401.0810m/z	401.0810031	4.814166667	neg	4-Phosphopantothenoylcysteine	58022	Unclassified	Unclassified	Unclassified			36.1	0	M-H	C12H23N2O9PS	5.201758769	1699.366714	1700.981352	1768.632778	3584.58059	2974.908282	3306.183618	1716.068701	1893.279633	2396.534166
4.84_335.0728m/z	335.0728223	4.836566667	neg	Captopril-cysteine disulfide	HMDB0060562	Organic acids and derivatives	Carboxylic acids and derivatives	Amino acids, peptides, and analogues	*		36.1	0	M-H	C12H20N2O5S2	-3.764060359	2263.391439	1848.592227	2195.09493	2171.878079	2413.48349	2163.710101	2231.691833	2260.334547	1952.51248
4.84_482.1706m/z	482.1706393	4.836566667	neg	6-Hydroxy Doxazosin	2455	Unclassified	Unclassified	Unclassified			36.1	0	M+FA-H	C22H23N5O5	5.758761021	2618.986028	3186.182638	3009.994322	3691.7037	4068.01824	3687.329501	2465.739943	3185.087133	2536.42866
4.86_446.1131m/z	446.1130937	4.858383333	neg	Mefenamic acid Metabolite (b-D-Glucopyranuronic acid, 1-[2-[(3-carboxy-2-meth	1101	Unclassified	Unclassified	Unclassified			36.1	0	M-H	C21H21NO10	8.553093297	3682.985559	3832.771852	3858.106765	4848.9455	4825.678224	5012.400925	4667.731809	5643.040467	5372.791126
4.95_295.0761m/z	295.0761484	4.94705	neg	Methoxybrassitin	93298	Organoheterocyclic compounds	Indoles and derivatives	Indoles			36.1	0	M+FA-H	C12H14N2O2S	1.38682325	362.4087224	471.3387362	469.5579229	666.660738	616.8126998	527.2879407	660.2845449	475.2655338	467.6135326
5.01_231.0277m/z	231.0276531	5.014716667	neg	5H-Oxireno[4,5]furo[3,2-g][1]benzopyran-5-one, 1a,8b-dihydro-3-methoxy-	1206	Unclassified	Unclassified	Unclassified			36.1	0	M-H	C12H8O5	-9.670061706	1239.77221	1150.677474	1232.646106	519.1439007	795.683087	685.0318366	610.2073638	844.4018694	737.0157206
5.08_338.0918m/z	338.0918041	5.080566667	neg	Topiramate	44196	Organoheterocyclic compounds	Dioxolopyrans	Unclassified		C07502	36.1	0	M-H	C12H21NO8S	0.863738379	211.3918815	221.6658044	299.9970625	250.7784348	226.6357562	252.8925654	265.2346607	178.4522871	219.8837009
5.27_359.1823m/z	359.182272	5.271716667	neg	trans-Stilbene	96322	Unclassified	Unclassified	Unclassified			36.1	0	2M-H	C14H12	4.851992822	1214.433339	944.5879831	1173.565664	1127.666908	811.7118904	853.0748489	1224.855367	933.2563215	1181.748222
5.34_601.1541m/z	601.1541438	5.3394	neg	Camellianin A	HMDB0029908	Phenylpropanoids and polyketides	Flavonoids	Flavonoid glycosides	*		36.1	0	M-H2O-H	C29H32O15	-3.443283751	982.1066954	1618.628129	1818.633441	111.4775771	162.1167783	0.001403199	0.001403199	282.2454893	157.3255848
5.41_397.0937m/z	397.0936904	5.406433333	neg	3,5,4'-Trihydroxy-6",6"-dimethylpyrano[2,3:7,6]flavone	50409	Unclassified	Unclassified	Unclassified			36.1	0	M+FA-H	C20H16O6	2.270035244	888.1566331	1044.777226	1012.542127	2429.428519	2187.506566	2150.047727	1628.311444	1506.824127	1353.245424
5.43_287.0225m/z	287.0225324	5.428733333	neg	5'-(3',4'-dihydroxyphenyl)-gamma-valerolactone sulfate	86078	Organic acids and derivatives	Organic sulfuric acids and derivatives	Arylsulfates	*		36.1	0	M-H	C11H12O7S	-1.961197063	1831.544355	1357.071073	1120.900139	817.9892909	817.4474504	390.9205637	851.5558728	1209.040956	959.7668555
5.53_321.0438m/z	321.043777	5.525416667	neg	Thiophene-4,5-epoxide	HMDB0060925	Phenylpropanoids and polyketides	Phenylpropanoic acids	Unclassified	*		36.1	0	M+FA-H	C14H12O4S	-0.201729189	482.812697	279.5685844	343.0105293	175.1080829	308.4953907	126.0837453	163.6985927	83.76128006	92.73710229
5.53_492.0739m/z	492.073925	5.525416667	neg	trans-Zeatin riboside diphosphate	64084	Unclassified	Unclassified	Unclassified			36.1	0	M-H2O-H	C15H23N5O11P2	9.462769583	4014.180332	3406.990323	3066.94282	1186.79291	1331.382074	1222.476524	2335.617124	2424.713657	3079.599173
5.83_587.1160m/z	587.1160079	5.825	neg	Rugulosin	71324	Unclassified	Unclassified	Unclassified			36.1	0	M+FA-H	C30H22O10	-6.441077316	1085.165396	555.6167695	1038.692692	1664.688347	1381.3044	768.5682119	1380.275368	1454.416323	1176.579495
6.30_523.1778m/z	523.1777674	6.302033333	neg	Dichotosinin	47489	Lipids and lipid-like molecules	Polyketides	Flavonoids			36.1	0	M+FA-H	C24H30O10	-9.060663277	14875.99876	19547.80595	19147.48292	18738.79421	18973.77008	15199.43521	18543.76161	17657.79153	23617.51865
6.44_415.2151m/z	415.2150647	6.442716667	neg	Coronafacic acid	71096	Unclassified	Unclassified	Unclassified			36.1	0	2M-H	C12H16O3	5.892012334	383.2471045	173.1953295	107.0456641	219.8555687	159.6820563	263.1524175	200.9977353	326.6096999	359.7022492
6.57_303.0828m/z	303.0828025	6.570233333	neg	4-methyl-n-(2-oxo-2,5,6,7-tetrahydro-1h-cyclopenta[b]pyridin-4-yl)benzenesulfor	96299	Unclassified	Unclassified	Unclassified			36.1	0	M-H	C15H16N2O3S	6.299306601	5859.708083	6461.77503	6605.410506	5281.994563	5443.116566	5344.996121	3614.53983	4284.042104	4061.546499
6.65_626.3546m/z	626.3545525	6.653683333	neg	Janthitrem F	87043	Organoheterocyclic compounds	Naphthopyrans	Unclassified	*		36.1	0	M-H2O-H	C39H51NO7	9.050115457	31113.60126	30438.12597	34870.08375	2072.749784	2158.615584	2442.809045	18917.67976	22831.51335	23328.3231
6.79_135.0313m/z	135.0313419	6.7874	neg	Ethyl propyl disulfide	HMDB0033053	Organosulfur compounds	Organic disulfides	Dialkyldisulfides			36.1	0	M-H	C5H12S2	4.233390363	381.3197696	416.0028116	358.5143759	450.0943276	315.3708528	398.1526755	350.0124227	392.9080989	406.3196016
7.04_327.1354m/z	327.1354018	7.035966667	neg	GSK 4716	70995	Unclassified	Unclassified	Unclassified			36.1	0	M+FA-H	C17H18N2O2	1.31533496	676.9108886	627.0569987	514.1688613	623.7586055	528.6393171	705.7554509	476.3407158	820.5475719	617.8056104
7.08_371.1901m/z	371.1901102	7.076316667	neg	(Ac)2-L-Lys-D-Ala-D-Ala	65956	Unclassified	Unclassified	Unclassified			36.1	0	M-H	C16H28N4O6	-9.398028169	1437.673965	1422.32824	1586.519258	2018.371654	1937.864348	1923.033325	1594.853673	2187.693488	1946.255811
7.08_538.2250m/z	538.2250405	7.076316667	neg	Ryanodine	67139	Unclassified	Unclassified	Unclassified			36.1	0	M+FA-H	C25H35NO9	-8.807294355	4021.596512	3968.56666	4157.711124	1734.190388	1941.635922	2107.358215	2816.766211	3680.107914	3757.0741
7.16_300.1349m/z	300.1349211	7.1647	neg	Febrifugine	68486	Unclassified	Unclassified	Unclassified			36.1	0	M-H	C16H19N3O3	-1.474196915	270.0946196	337.5110565	194.5787294	323.0687267	347.1143051	279.2975904	289.5231444	226.181861	207.8549749
7.20_281.1371m/z	281.137075	7.201166667	neg	Artemisinin	532	Unclassified	Unclassified	Unclassified			36.1	0	M-H	C15H22O5	-8.408219419	1046.249558	1016.374486	1062.742967	1784.308093	1642.711863	1911.337765	1025.482028	1169.446438	898.7960361
7.39_370.2236m/z	370.2235942	7.3938	neg	Tumonoic Acid E	65435	Unclassified	Unclassified	Unclassified			36.1	0	M+FA-H	C18H31NO4	0.254793817	229.6914604	241.2962171	0.001403199	152.8092617	140.740649	210.0004305	238.514763	302.4812603	268.3468149
7.69_489.2344m/z	489.2343502	7.687666667	neg	ID14326	HMDB0060828	Nucleosides, nucleotides, and analogues	Nucleoside and nucleotide analogues	1-pyranosylindoles	*		36.1	0	M-H2O-H	C28H36N4O3S	2.713749171	1496.595745	1626.711213	1444.442933	928.7589936	796.5484321	1121.227096	644.0275835	828.1554679	1112.273047
7.74_627.3754m/z	627.3753628	7.738233333	neg	all-trans-4-oxoretinoic acid	2282	Lipids and lipid-like molecules	Prenol lipids	Retinoids	**	C16678	36.1	0	2M-H	C20H26O3	9.946014666	6569.560424	8270.744198	7136.802616	8448.919113	9747.29782	8306.582457	8363.111563	9615.168897	10195.24912
7.75_378.1020m/z	378.1019778	7.747083333	neg	12-Hydroxydihydrochelirubine	64388	Unclassified	Unclassified	Unclassified			36.1	0	M-H	C21H17NO6	9.6726514	6114.34898	6744.497639	6694.550858	8925.301289	8698.45353	7844.444034	8048.059653	8475.929792	8893.547501
8.07_463.2235m/z	463.2234806	8.066183333	neg	1-(4Z,7Z,10Z,13Z,16Z,19Z-docosahexaenoyl)-glycero-3-phosphate	HMDB0062307	Lipids and lipid-like molecules	Glycerophospholipids	Glycerophosphates	*		36.1	0	M-H2O-H	C25H39O7P	-4.185735282	11971.20384	14236.59327	12834.40022	1131.007758	1147.333441	903.8561474	4971.415413	6480.320187	6296.245726
8.14_246.0752m/z	246.0751893	8.138533333	neg	Streptozocin	592	Organic oxygen compounds	Organooxygen compounds	Carbohydrates and carbohydrate conjugates		C07313	36.1	0	M-H2O-H	C8H15N3O7	7.659983896	301.6474736	273.1579442	325.4120971	350.7301761	449.1435585	407.58185	256.3029326	379.2945967	230.975951
8.81_293.1373m/z	293.1372701	8.814016667	neg	(S)-Pterosin A	87177	Benzenoids	Indanes	Indanones	*		36.1	0	M+FA-H	C15H20O3	-8.774495649	252.5196511	354.5820004	303.0977369	770.2890473	464.5114234	776.3788002	165.4534823	196.2573451	134.8088094
9.32_295.1823m/z	295.1822703	9.321783333	neg	Rhazidigenine Nb-oxide	HMDB0030263	Organoheterocyclic compounds	Indoles and derivatives	Indoles	*		36.1	0	M-H2O-H	C19H26N2O2	2.174899486	1287.593419	1284.407536	466.2180243	821.3656706	1170.664422	779.7037479	701.3366351	928.839268	1044.122601
0.54_773.5352m/z	773.535169	0.535283333	neg	PG(18:0/18:2(9Z,12Z))	61878	Lipids and lipid-like molecules	Glycerophospholipids	Glycerophosphoglycerols	*		36	0	M-H	C42H79O10P	1.755964191	1184.841426	811.8781586	1018.020684	0.001403199	0.001403199	0.001403199	224.3817005	0.001403199	0.001403199
0.69_281.0732m/z	281.0732262	0.692166667	neg	Thiacetazone	2827	Unclassified	Unclassified	Unclassified			36	0	M+FA-H	C10H12N4OS	7.799528619	752.7014206	629.0757254	565.4407499	2232.693478	2419.029065	2641.839234	1555.172215	1641.85841	1792.447185
0.74_364.9990m/z	364.9990271	0.7359	neg	Luteolin 7-sulfate	HMDB0038472	Phenylpropanoids and polyketides	Flavonoids	Flavones	*		36	0	M-H	C15H10O9S	4.783079074	1050.460484	811.6733351	1777.216167	1251.702084	2168.200559	1017.546708	1693.170727	774.3678589	2420.316912
0.74_455.1026m/z	455.1026029	0.7359	neg	Nicosulfuron	68714	Unclassified	Unclassified	Unclassified			36	0	M+FA-H	C15H18N6O6S	8.648410836	2600.022216	1233.482182	2932.550781	1053.180811	1231.202996	1043.963637	803.2713693	2252.61111	2713.799879
0.76_333.0595m/z	333.0595031	0.757316667	neg	Aromadendrin	HMDB0030847	Phenylpropanoids and polyketides	Flavonoids	Flavans	***	C00974	36	0	M+FA-H	C15H12O6	-7.248078516	5736.188342	5909.263027	6017.965425	8156.451212	6993.327323	6783.413694	7160.79822	7362.101352	6555.248273
0.82_182.9969m/z	182.9968797	0.819566667	neg	6-Thiouric acid	718	Organoheterocyclic compounds	Imidazopyrimidines	Purines and purine derivatives	**	C16613	36	0	M-H	C5H4N4O2S	-7.283838516	4894.331446	5015.887052	5298.587606	6232.881472	5394.896553	5628.784146	6173.487939	7214.939162	6313.230462
1.22_278.0260m/z	278.0260349	1.224233333	neg	L-Cysteinylglycine disulfide	HMDB0000709	Organic acids and derivatives	Carboxylic acids and derivatives	Amino acids, peptides, and analogues	*		36	0	M-H2O-H	C8H15N3O5S2	-4.836048754	955.9825952	1110.222476	1054.992897	1040.275809	1165.249305	1459.881162	1180.35349	1235.638029	1177.302865
1.38_228.0709m/z	228.0708623	1.380283333	neg	N-(3-oxo-hexanoyl)-homoserine thiolactone	LMFA08020240	Lipids and lipid-like molecules	Fatty Acyls	Fatty amides			36	0	M-H	C10H15NO3S	3.816868332	70.70153935	27.78222201	57.85807037	178.1934416	197.7574508	28.80725856	153.4015714	153.0998568	38.51704036
1.59_551.9916m/z	551.9915911	1.5934	neg	dGTP	HMDB0001440	Nucleosides, nucleotides, and analogues	Purine nucleotides	Purine deoxyribonucleotides	**	C00286	36	0	M+FA-H	C10H16N5O13P3	-4.649540441	1651.929747	1229.476294	1528.421284	1272.619857	1497.508135	1418.252139	1139.778433	1652.063106	1573.233674
1.80_212.0260m/z	212.0259649	1.798266667	neg	Thioguanine	597	Organoheterocyclic compounds	Imidazopyrimidines	Purines and purine derivatives	**	C07648	36	0	M+FA-H	C5H5N5S	7.159587719	57.96643366	99.00916506	79.81349775	23.10921458	21.73865711	74.4572896	57.00697742	52.00756836	26.82686567
10.23_340.2497m/z	340.2496822	10.22736667	neg	Linoleyl hydroxamic acid	LMFA08020211	Lipids and lipid-like molecules	Fatty Acyls	Fatty amides			36	0	M+FA-H	C18H33NO2	1.18544044	53.82119553	63.29263043	66.39317209	154.3707244	247.6794211	121.6827821	56.58464633	0.001403199	348.8173334
10.89_433.3455m/z	433.3455203	10.88813333	neg	Dihydrovitamin K1	HMDB0251338	Lipids and lipid-like molecules	Prenol lipids	Diterpenoids	**	C03313	36	0	M-H2O-H	C31H48O2	-4.574602858	901.7809683	1069.072222	929.6426028	982.7256066	1165.949073	1191.713136	1062.93816	1082.921646	1294.749751
10.89_709.1634m/z	709.1633649	10.88813333	neg	Apigenin 7-(6"-malonylneohesperidoside)	LMPK12110386	Lipids and lipid-like molecules	Polyketides	Flavonoids			36	0	M+FA-H	C30H32O17	1.825486227	14458.56051	16678.21045	14889.4455	12583.64498	15324.86437	13859.69316	16707.94684	15426.12602	16945.47646
13.92_337.2056m/z	337.2056393	13.91825	neg	Lisuride	1010	Organoheterocyclic compounds	Quinolines and derivatives	Indoloquinolines			36	0	M-H	C20H26N4O	6.665235329	55109.40861	27896.22878	30933.88317	22034.98092	27561.43837	23482.00791	29103.39705	51651.17994	26274.68134
14.66_436.2292m/z	436.2291979	14.6619	neg	Tafluprost ethyl amide	96555	Unclassified	Unclassified	Unclassified			36	0	M-H	C24H33F2NO4	-2.951714358	3603.958078	3142.185776	3588.750176	3082.839324	3654.858094	3051.181605	3838.575274	3402.58376	3184.519954
2.15_435.0513m/z	435.0513455	2.154666667	neg	5-Phosphoribosyl-4-(N-succinocarboxamide)-5-aminoimidazole	6010	Unclassified	Unclassified	Unclassified			36	0	M-H2O-H	C13H19N4O12P	-9.958851648	1653.693767	1771.530283	1709.256527	2459.267932	2083.434113	2098.313262	1208.362	1844.766848	1931.258125
2.38_370.1370m/z	370.1369957	2.380333333	neg	Acarbose (M8)	766	Unclassified	Unclassified	Unclassified			36	0	M+FA-H	C12H23NO9	4.648691832	1145.390337	1102.908396	1576.586206	1540.784496	1416.106603	1696.121612	1323.310562	1344.530656	1416.317927
2.72_294.0576m/z	294.0575502	2.72215	neg	Albendazole-gamma-hydroxysulphone	71271	Unclassified	Unclassified	Unclassified			36	0	M-H2O-H	C12H15N3O5S	6.866437155	7908.948952	8348.290664	8844.073812	7627.803254	8506.867366	7972.66404	9609.358007	11230.90927	11151.33259
3.36_540.0578m/z	540.057785	3.362333333	neg	N1-(5-Phospho-D-ribosyl)-AMP	3596	Nucleosides, nucleotides, and analogues	Purine nucleotides	Purine ribonucleotides	**	C02741	36	0	M-H2O-H	C15H23N5O14P2	7.069535751	3503.291563	3635.200844	3148.381058	5314.214846	4892.658499	4945.653291	3162.964643	4177.771259	3186.522701
3.48_205.0641m/z	205.0640608	3.484883333	neg	Glycyl-Methionine	85757	Organic acids and derivatives	Carboxylic acids and derivatives	Amino acids, peptides, and analogues	*		36	0	M-H	C7H14N2O3S	-5.707023139	1003.273322	1001.252074	1081.533285	50.23603575	77.85370858	24.42482398	839.6739477	914.831151	817.4151725
3.75_336.0731m/z	336.0730734	3.747833333	neg	Metaproterenol 3-O-sulfate	1178	Unclassified	Unclassified	Unclassified			36	0	M+FA-H	C11H17NO6S	-9.57737802	592.9732006	421.2782527	491.386668	1324.607314	1217.360503	700.1285511	463.7738092	662.8044193	586.5292456
3.75_769.0919m/z	769.0918559	3.747833333	neg	1,2,3,4-Tetragalloyl-alpha-D-glucose	68194	Unclassified	Unclassified	Unclassified			36	0	M-H2O-H	C34H28O22	3.139819991	559.9206813	309.2592898	428.5438032	1685.279643	1756.682653	772.45075	394.3902736	1051.684692	866.3446005
3.78_460.0935m/z	460.0934695	3.779133333	neg	6-Heptenyl glucosinolate	93118	Organic oxygen compounds	Organooxygen compounds	Carbohydrates and carbohydrate conjugates			36	0	M+FA-H	C14H25NO9S2	-4.352583537	749.4186519	696.6548179	694.5193369	833.2780029	956.2475101	991.9457515	561.4510334	572.2729397	719.5015118
3.89_222.9758m/z	222.9757654	3.8906	neg	Hydroxymethylphosphonate	63590	Unclassified	Unclassified	Unclassified			36	0	2M-H	CH5O4P	-9.14676668	444.4342842	433.077725	573.3389004	693.7962565	578.2832976	690.1541183	528.4273825	464.7321701	564.8056489
4.05_273.0342m/z	273.0341866	4.046666667	neg	ensulizole	4009	Unclassified	Unclassified	Unclassified			36	0	M-H	C13H10N2O3S	0.911706775	127.0580571	153.685328	557.7428715	584.412678	267.7306765	134.7369262	323.5301135	207.9704742	249.5360037
4.11_353.1333m/z	353.1333495	4.113883333	neg	Deacetyldiltiazem	HMDB0002873	Organoheterocyclic compounds	Benzothiazepines	Unclassified	*		36	0	M-H2O-H	C20H24N2O3S	1.147213975	381.9631948	470.461658	414.5023315	1034.644872	800.1278391	863.7866799	403.206594	491.6588879	459.4474469
4.16_265.0306m/z	265.0306477	4.15895	neg	2-(2-pyridyl)-4-methyl-Thiazole-5-Carboxylic Acid	45438	Unclassified	Unclassified	Unclassified			36	0	M+FA-H	C10H8N2O2S	8.164089842	3048.908933	3059.872127	3240.097958	4317.511722	3364.821396	3858.219296	4031.660701	3021.984207	3095.60738
4.16_429.1319m/z	429.1319036	4.15895	neg	Cycloartomunoxanthone	49939	Organoheterocyclic compounds	Benzopyrans	1-benzopyrans	*		36	0	M-H2O-H	C26H24O7	-5.485590481	380.4558374	275.5051176	349.7333079	203.2553893	267.3021384	582.0715053	626.5346685	739.1001028	455.6506027
4.28_376.0651m/z	376.0651493	4.278183333	neg	Gonyautoxin 2	71388	Unclassified	Unclassified	Unclassified			36	0	M-H2O-H	C10H17N7O8S	-7.444299418	1776.653742	2036.845159	1951.942187	135.1499045	0.001403199	44.15931506	1631.726369	1774.386257	1521.890864
4.34_597.0195m/z	597.0195473	4.344733333	neg	Myricatin	86317	Phenylpropanoids and polyketides	Flavonoids	Flavans	*		36	0	M+FA-H	C22H16O15S	0.640543956	1691.109446	1493.976541	1208.345464	2310.5041	1940.803989	1821.385836	2063.901072	2206.111519	1356.001323
4.39_326.0991m/z	326.0990552	4.3893	neg	(S)-Esomeprazole	58285	Unclassified	Unclassified	Unclassified			36	0	M-H2O-H	C17H19N3O3S	6.327899339	5789.467053	8919.606468	9120.780336	6983.997164	7290.939537	6856.707287	6571.372817	8320.330374	8730.997627
4.41_665.1846m/z	665.1846185	4.41115	neg	Ethalfluralin	72592	Unclassified	Unclassified	Unclassified			36	0	2M-H	C13H14F3N3O4	6.925613181	13153.93226	15962.13673	15796.31826	905.9202541	1692.17074	863.4523468	8042.233818	8500.484363	7787.465124
4.59_203.1176m/z	203.1176373	4.589283333	neg	3-Hydroxymonoethylglycylxylidide (3-Hydroxy-MEGX)	1002	Unclassified	Unclassified	Unclassified			36	0	M-H2O-H	C12H18N2O2	-6.074587604	63.44943145	64.55248853	0.001403199	67.73077958	120.3575863	46.96783409	94.69636731	0.001403199	131.2450767
4.79_462.0660m/z	462.0660182	4.792316667	neg	Adenylosuccinate	3551	Benzenoids	Benzene and substituted derivatives	Biphenyls and derivatives	**	C03794	36	0	M-H	C14H18N5O11P	-1.616283514	891.2478285	778.4766702	882.3004434	1320.805572	1061.315421	1390.579328	400.9321596	827.6213684	806.5720849
4.84_463.0842m/z	463.084184	4.836566667	neg	Kaempferol 3-O-arabinoside	86135	Phenylpropanoids and polyketides	Flavonoids	Flavonoid glycosides	*		36	0	M+FA-H	C20H18O10	-9.604625671	2744.59365	2947.424472	2969.325304	3493.640908	3415.340136	3399.428385	4704.649829	4656.551674	5239.444375
5.06_317.0912n	298.0733396	5.05975	neg	3-O-Methylrimiterol sulfate	2339	Unclassified	Unclassified	Unclassified			36	0	M-H2O-H, M-H	C13H19NO6S	-6.709636533	2080.315734	2027.783217	2170.967794	6457.176613	6036.574122	6694.56058	2818.196906	2807.491924	3115.07579
5.20_295.1164m/z	295.1164096	5.20005	neg	Cyclocalopin E	94363	Organoheterocyclic compounds	Furopyrans	Unclassified	*		36	0	M-H	C15H20O6	-7.77471094	4583.903295	4671.224552	4747.753837	5353.044569	5427.391276	5540.022576	4097.159779	3998.983589	4674.21212
5.22_833.3433m/z	833.3432581	5.222166667	neg	Tirandamycin	85096	Unclassified	Unclassified	Unclassified			36	0	2M-H	C22H27NO7	-8.353534244	3117.265935	2529.081417	3080.589106	3224.299946	3097.601644	2622.242754	4668.319269	5179.872742	5015.700568
5.24_412.2098n	411.2025698	5.244033333	neg	16(R)-AFP 07 (free acid)	96476	Unclassified	Unclassified	Unclassified			36	0	M-H, M+FA-H	C22H30F2O5	9.014230088	6057.198271	5995.263433	5769.57264	4562.026115	4747.918629	4357.729725	7723.751341	6846.34648	7734.840256
5.26_511.1416m/z	511.1415805	5.263033333	neg	(2S)-Liquiritigenin	HMDB0029519	Phenylpropanoids and polyketides	Flavonoids	Flavans	***	C09762	36	0	2M-H	C15H12O4	3.395891832	1544.892187	1079.224152	928.5918902	1807.359451	1527.840083	1533.50615	1274.279938	1006.920942	1168.025141
5.30_135.0314m/z	135.0313616	5.302883333	neg	Ethyl isopropyl disulfide	HMDB0033054	Organosulfur compounds	Organic disulfides	Dialkyldisulfides			36	0	M-H	C5H12S2	4.378185536	539.6903496	540.4067929	511.1770455	508.5032467	486.0642007	602.9876007	700.2036172	501.4416819	579.8358984
5.34_270.0984m/z	270.0984211	5.3394	neg	6-Benzylaminopurine	68947	Organoheterocyclic compounds	Imidazopyrimidines	Purines and purine derivatives		C11263	36	0	M+FA-H	C12H11N5	-5.451640127	143.9014683	168.4699257	129.6461569	302.2178626	185.9883938	410.6572492	241.5928984	257.3924186	323.5760714
5.34_408.1515m/z	408.1515478	5.3394	neg	Linustatin	HMDB0303673	Unclassified	Unclassified	Unclassified			36	0	M-H	C16H27NO11	1.010608741	763.3320497	736.6487833	687.1962363	72.93439099	0.001403199	133.386325	541.3519419	756.1067356	851.2489261
5.43_645.1111m/z	645.111082	5.428733333	neg	NORSTICTIC ACID PENTAACETATE	44625	Unclassified	Unclassified	Unclassified			36	0	M+FA-H	C28H24O15	2.264643229	2788.18029	3074.410845	3776.343381	1475.383337	1360.931706	1192.803322	1519.658228	1941.707405	1270.780905
5.71_383.1537m/z	383.1536545	5.709933333	neg	3b,16b-Dihydroxyandrostenone sulfate	5323	Unclassified	Unclassified	Unclassified			36	0	M-H	C19H28O6S	0.706196996	117611.9899	117796.9161	122530.2059	174212.1961	170067.055	173595.9806	132655.7699	144135.6335	151946.3403
5.74_595.2741m/z	595.2741305	5.744566667	neg	Toxin T2 tetrol	HMDB0036159	Lipids and lipid-like molecules	Prenol lipids	Sesquiterpenoids	*		36	0	2M-H	C15H22O6	-3.135955815	781.9838481	839.5538443	902.9579362	684.7705496	959.7321958	817.9720479	1058.817699	1069.304771	1032.154251
5.87_405.0805m/z	405.0804986	5.866533333	neg	Rosmarinate	3504	Phenylpropanoids and polyketides	Cinnamic acids and derivatives	Hydroxycinnamic acids and derivatives	**	C01850	36	0	M+FA-H	C18H16O8	-6.170121923	1588.675859	1306.953165	1511.906853	579.2146815	719.6823043	540.8764831	2641.789115	1847.620565	2176.300894
6.07_490.1679m/z	490.1678874	6.07	neg	Dubiusine	69387	Unclassified	Unclassified	Unclassified			36	0	M+FA-H	C23H27NO8	-8.945533575	3132.086165	3697.20533	3205.779061	7919.975828	7483.232182	6979.019302	4588.958031	4737.309334	4641.148714
6.07_525.1574m/z	525.1573633	6.07	neg	Bruceine B	67170	Unclassified	Unclassified	Unclassified			36	0	M+FA-H	C23H28O11	-8.333192481	5921.443361	6607.546425	6677.47696	7802.029272	7331.804075	5938.490859	4692.364074	5158.61078	4711.325537
6.21_271.1165m/z	271.1164537	6.206233333	neg	12-hydroxyjasmonic acid	45941	Lipids and lipid-like molecules	Fatty Acyls	Octadecanoids			36	0	M+FA-H	C12H18O4	-9.98681031	1011.843715	753.7155797	1006.448306	1584.867966	1296.831746	1357.82341	639.642375	944.8071194	851.6727224
6.22_286.0698m/z	286.0698239	6.215	neg	Koeniginequinone A	88169	Organoheterocyclic compounds	Indoles and derivatives	Carbazoles	*		36	0	M+FA-H	C14H11NO3	-9.425352522	1314.249979	998.1148858	1217.398005	394.7189666	267.6500776	357.2752458	1149.116065	1297.735261	1181.267809
6.26_438.1752m/z	438.1751865	6.261966667	neg	Latifoline	68262	Unclassified	Unclassified	Unclassified			36	0	M+FA-H	C20H27NO7	-4.498244123	2689.06687	1535.527738	2677.024278	3148.027544	2289.299518	3674.468267	2102.91649	2638.323425	2762.957592
6.34_505.2662m/z	505.266192	6.339983333	neg	SLF	64652	Unclassified	Unclassified	Unclassified			36	0	M-H2O-H	C30H40N2O6	-8.781230196	24856.31402	28786.02138	29093.6107	21733.65122	23180.12738	21108.40428	28364.09045	29259.40465	32106.74617
6.39_376.2611m/z	376.2610844	6.386066667	neg	Sphingosine 1-phosphate (d19:1-P)	HMDB0060062	Lipids and lipid-like molecules	Sphingolipids	Phosphosphingolipids	*		36	0	M-H2O-H	C19H42NO5P	-2.870263803	1912.307221	1413.958581	1733.108703	1655.426982	1964.46257	1773.447587	1897.435361	1782.128401	1499.03175
6.40_290.1435m/z	290.1434582	6.39835	neg	Fluperlapine	68729	Unclassified	Unclassified	Unclassified			36	0	M-H2O-H	C19H20FN3	-9.142612017	5818.684644	7229.601365	7237.619415	9151.29999	8824.330724	9057.907316	7067.791853	6893.418989	7250.664734

6.44_479.1153m/z	479.1153136	6.442716667	neg	2-O-Caffeoylarbutin	87601	Organic oxygen compounds	Organooxygen compounds	Carbohydrates and carbohydrate conjugates	*		36	0	M+FA-H	C21H22O10	-9.642750043	18326.95053	18843.25488	19612.6172	21808.52067	22166.11997	22337.7347	20908.7841	21831.04897	20542.72351
6.83_359.1247m/z	359.1246595	6.825	neg	3-Methoxytyramine-βxanthin	64466	Unclassified	Unclassified	Unclassified			36	0	M-H	C18H20N2O6	-0.556661961	654.2023025	550.912826	611.8831857	659.8836768	480.0122199	687.2017457	379.1402404	566.6859739	514.5074387
6.92_579.1336m/z	579.1336216	6.915966667	neg	Monoacetyldapsone(MADDS)	1930	Unclassified	Unclassified	Unclassified			36	0	2M-H	C14H14N2O3S	-7.116481948	4713.551349	563.6505631	15715.81715	634.0590529	546.6447169	388.71813	401.9308951	452.0656586	1828.706433
7.00_426.9406m/z	426.9405569	7.0023	neg	Luteolin 7,3'-disulfate	49167	Lipids and lipid-like molecules	Polyketides	Flavonoids			36	0	M-H2O-H	C15H10O12S2	-6.659073197	10540.83547	8933.231688	8490.056419	9301.276844	8633.659267	9635.206087	11805.32034	10898.87582	10886.75656
7.26_551.2091m/z	551.2091269	7.262266667	neg	13-Hydroxypergolide glucuronide	1794	Unclassified	Unclassified	Unclassified			36	0	M+FA-H	C25H34N2O7S	4.447998932	9059.322954	10357.59671	10737.57769	11786.96472	10449.15697	10761.18948	13413.10046	14384.33897	14623.56177
7.35_297.1321m/z	297.1320786	7.353466667	neg	Neoilludin A	73450	Unclassified	Unclassified	Unclassified			36	0	M-H	C15H22O6	-7.658736723	1090.068945	1177.143017	1247.324633	1570.013505	1493.808657	1492.882617	791.9874638	1129.529949	1053.140708
7.44_991.2370m/z	991.2369678	7.440866667	neg	1-Nitro-5-glutathionyl-6-hydroxy-5,6-dihydronaphthalene	70338	Organic acids and derivatives	Carboxylic acids and derivatives	Amino acids, peptides, and analogues	**	C14806	36	0	2M-H	C20H24N4O9S	-8.62130491	7543.717058	6708.60527	6853.274308	0.001403199	285.5393956	630.7509615	1588.554504	2305.477133	1389.169857
7.87_343.1190m/z	343.119032	7.87455	neg	(+)-Ligballinol	86329	Lignans, neolignans and related compounds	Furanoid lignans	Unclassified	*		36	0	M+FA-H	C18H18O4	1.073725804	682.3817419	686.266583	600.0334914	0.001403199	0.001403199	0.001403199	278.0656332	266.2422161	236.1185661
7.92_727.1709m/z	727.1708911	7.91555	neg	Petunidin 3-glucoside-5-(6''-acetylglucoside)	47133	Unclassified	Unclassified	Unclassified			36	0	M+FA-H	C30H34O18	-2.676815251	2150.26908	2390.880596	2501.917016	2461.030949	3371.26419	2654.18315	1935.896681	2941.946899	2851.586761
7.92_737.1999m/z	737.1998793	7.91555	neg	6-Methoxykaempferol 3,7-bis(3-acetylrhamnoside)	51257	Lipids and lipid-like molecules	Polyketides	Flavonoids			36	0	M+FA-H	C32H36O17	9.284588321	21814.21465	22682.77071	23768.98406	27514.88605	29249.76341	28638.24594	23491.24873	27642.23441	26285.75814
7.93_577.2252m/z	577.2252491	7.9268	neg	Austalide D	87439	Organoheterocyclic compounds	Benzopyrans	1-benzopyrans	*		36	0	M+FA-H	C28H36O10	-7.141780435	5327.039843	6027.295087	6072.761684	6976.698888	6943.067276	7358.538119	5296.775703	6631.408679	6513.560199
8.38_476.2326m/z	476.2326183	8.383816667	neg	Dihydrotetrabenazine glucuronide	2802	Unclassified	Unclassified	Unclassified			36	0	M-H2O-H	C25H37NO9	7.324918346	5351.944181	5623.200379	6282.328876	5746.446843	6642.607968	6804.484198	3833.513761	4631.505238	4037.126952
8.50_514.2787m/z	514.2787495	8.503866667	neg	Atracurium (Quaternary alcohol)	1155	Unclassified	Unclassified	Unclassified			36	0	M-H	C29H41NO7	-4.4182568	2862.037922	2740.140197	3247.188173	69.77168064	0.001403199	77.47991399	1059.109226	1271.569682	1112.2678
8.64_371.1713m/z	371.1713092	8.635	neg	17beta-(Acetylthio)estra-1,3,5(10)-trien-3-ol acetate	70454	Unclassified	Unclassified	Unclassified			36	0	M-H	C22H28O3S	7.173792165	23898.50964	26510.29232	24379.5323	39151.21901	33831.37638	34435.8383	17022.34115	16896.24673	16690.3079
9.33_220.1468m/z	220.1467801	9.332316667	neg	Alchorneine	68389	Unclassified	Unclassified	Unclassified			36	0	M-H	C12H19N3O	5.626528599	1240.457654	1150.835775	725.3757654	1010.146601	1416.660645	1093.317149	942.5533019	1035.232298	1154.461987
0.51_118.9926m/z	118.9925542	0.511216667	neg													1136.307577	1335.602052	1054.739815	1250.517554	1753.041347	1627.727202	1474.358799	1323.510808	1275.541355
0.51_210.8841m/z	210.884142	0.511216667	neg													971.4962217	935.9769526	636.734438	517.1439479	633.3094794	508.6467648	881.3873743	654.1660829	681.5381444
0.51_212.8820m/z	212.8819532	0.511216667	neg													626.1411372	574.7786815	412.2687161	363.4794974	434.6807135	316.7347093	585.3477544	456.1948183	430.2683284
0.51_226.9786m/z	226.9786067	0.511216667	neg													546.0937922	746.748589	560.7471776	649.7281986	910.229627	846.5929598	880.5888482	675.6148691	737.579351
0.51_276.9753m/z	276.9753189	0.511216667	neg													321.2586822	407.353347	292.3702451	385.0947552	512.176399	500.2402291	513.083579	383.663753	349.296956
0.51_288.9004m/z	288.9003745	0.511216667	neg													2092.905357	1534.142871	922.1128134	864.0593503	837.8375596	712.6439633	1472.077017	1074.333458	891.2906433
0.51_290.8986m/z	290.8985931	0.511216667	neg													887.1189777	708.3206389	441.9888512	342.5597826	418.1319368	325.9005303	704.19119	529.0820261	520.6993291
0.51_338.8972m/z	338.8971669	0.511216667	neg													896.9048093	677.8803184	379.3738682	353.5774386	349.0276751	284.8537819	583.9381268	483.9825449	335.1977938
0.51_340.8954m/z	340.8953517	0.511216667	neg													411.6109782	330.3941776	181.3607843	142.0599763	150.3539116	125.0150473	280.3269399	0.001403199	152.7678599
0.51_348.9543m/z	348.9542577	0.511216667	neg													298.3898991	349.9824933	393.584139	440.8689508	575.6286278	361.9170866	403.0628063	474.8551004	440.9417751
0.51_354.8922m/z	354.8922381	0.511216667	neg													638.7649926	515.2082284	277.0547122	280.1083036	329.5877214	243.528616	385.0291527	351.2424307	397.9896793
0.52_1093.2400m/z	1093.239983	0.51805	neg													0.001403199	0.001403199	0.001403199	0.001403199	407.3975541	10692.28983	0.001403199	0.001403199	0.001403199
0.52_1137.9640m/z	1137.964011	0.51805	neg													872.1948043	242.1407829	229.6750497	3461.060733	2730.624943	1171.364604	1804.401354	2675.226852	4180.194087
0.52_157.9552m/z	157.9551881	0.51805	neg													72.85020199	67.05070454	90.05676062	80.85185905	83.82065597	74.89481088	75.9651877	36.85580254	64.95625225
0.52_292.9704m/z	292.9704267	0.51805	neg													214.6444874	289.3315252	213.8795232	255.2000046	358.3873321	394.0538291	338.2403158	288.1331282	256.2620706
0.52_298.9573m/z	298.9573426	0.51805	neg													951.5750698	1068.06708	1092.30399	1549.772963	1881.627437	1502.902251	1517.079141	1527.906876	1549.92141
0.52_314.9524m/z	314.9523989	0.51805	neg													548.0132059	623.4032732	557.1919799	929.7000231	1170.649366	792.5151296	934.8535991	842.0300679	849.6014357
0.52_951.2349m/z	951.2348882	0.51805	neg													0.001403199	0.001403199	0.001403199	0.001403199	0.001403199	0.001403199	0.001403199	10757.17306	61.62378728
0.53_130.9442m/z	130.9441905	0.529533333	neg													205.3201806	212.5287091	219.609568	325.0024848	216.2518952	275.2743107	276.2299025	223.4125702	250.7288548
0.53_149.9946m/z	149.9945879	0.529533333	neg													365.4670567	437.9401484	344.2758978	391.3993402	321.5200265	311.6273043	469.6315193	416.0996771	374.7570184
0.53_183.0048m/z	183.0048205	0.529533333	neg													232.9421514	252.7247275	192.7668729	250.7170948	225.4678744	216.8767303	242.09844	190.9762352	222.7447295
0.53_248.9733m/z	248.9733467	0.529533333	neg													1526.004635	4814.133416	928.2755843	1697.13584	2505.712545	1508.877187	1495.710065	1192.504143	1805.927123
0.53_373.0454m/z	373.0454355	0.529533333	neg													1590.640085	277.2943735	93.04391798	12.14074122	36.25427413	0.001403199	159.1427205	69.53607988	57.50629494
0.53_431.0328m/z	431.0328475	0.529533333	neg													552.2939361	217.5610985	26.7995417	36.12313608	21.49294315	0.001403199	67.81350365	24.09444523	36.18650553
0.53_447.0642m/z	447.0642496	0.529533333	neg													1152.503523	113.9921933	14.11824803	0.001403199	0.001403199	0.001403199	17.85327182	0.001403199	10.76714749
0.53_498.7845m/z	498.7844597	0.529533333	neg													191.0766179	530.8730607	53.89585227	1281.37152	536.7306405	557.1892438	683.5037427	554.6643066	763.7478843
0.54_129.9364m/z	129.936415	0.535283333	neg													111.6192752	124.6868519	100.0078535	38.83328417	63.34140546	97.95999756	112.5153814	60.1907382	79.83565786
0.54_136.8946m/z	136.8946032	0.535283333	neg													193.0543876	219.5166844	284.958166	54.46011759	221.4498503	105.3871095	202.2161341	137.3082919	247.7533852
0.54_146.0248m/z	146.0248475	0.535283333	neg													132.6619733	144.5985418	125.3375276	91.2967205	210.3534817	166.627354	123.9644334	154.379221	190.2610516
0.54_148.9189m/z	148.9188915	0.535283333	neg													440.4374746	330.9009538	502.9039911	226.6771751	407.508622	521.0518074	404.0844998	199.2924309	278.3942135
0.54_163.9084m/z	163.9083799	0.535283333	neg													137.7056456	109.2145318	219.5382121	65.95656302	136.3444907	61.80612557	91.5284625	105.6958914	121.2767752
0.54_165.8944m/z	165.8944452	0.535283333	neg													65.93556392	80.32744519	56.33887521	12.634248	27.1437012	18.38549315	90.40908403	16.25955093	24.4046743
0.54_172.8636m/z	172.863583	0.535283333	neg													211.1042443	273.6412294	280.6414415	285.3099495	199.0428254	273.0843889	232.1402607	162.7114119	220.2837801
0.54_174.8643m/z	174.8642949	0.535283333	neg													334.1921003	355.8825719	429.6157405	206.7275979	335.5652151	464.5762913	478.4677412	153.0236633	379.4803041
0.54_176.8593m/z	176.8593059	0.535283333	neg													15.56107501	118.7410202	128.6609966	55.222421	64.14571789	145.1503396	175.7492245	46.56927278	66.72623057
0.54_178.9045m/z	178.9044651	0.535283333	neg													715.5535407	773.6468874	768.2234622	383.1247152	669.6451521	616.607438	800.1517539	517.6529384	688.7373511
0.54_180.8998m/z	180.8998116	0.535283333	neg													15612.75967	14894.50146	14897.75796	12060.11938	12191.31996	13545.96641	15541.34894	11195.26037	11573.55126
0.54_186.9341m/z	186.9341095	0.535283333	neg													18.81106775	67.14977469	90.13060693	1.605524719	109.0531457	0.292319784	0.001403199	86.9282806	106.7148576
0.54_187.8890m/z	187.888984	0.535283333	neg													786.5520481	1025.080653	1006.710228	867.0767588	989.5269856	1307.751139	1230.847689	784.6432278	964.643884
0.54_203.9369m/z	203.9369138	0.535283333	neg													1564.185653	1740.440053	2157.124307	1155.655736	2038.703255	1440.467358	1627.171404	1539.341571	1705.040817
0.54_206.8247m/z	206.8247349	0.535283333	neg													209.9979868	264.2902865	255.6793968	96.59824752	139.7835192	119.2877837	238.0658021	107.75774	121.6965265
0.54_217.9893m/z	217.9892826	0.535283333	neg													0.001403199	60.89153427	164.1016628	177.4100908	117.6565605	102.0395004	206.4850208	87.57322979	177.8273004
0.54_218.9864m/z	218.9863941	0.535283333	neg													72.6543715	144.5121088	77.93811782	137.0513389	58.75867832	96.20007763	120.613671	101.4531193	109.4055436
0.54_240.9112m/z	240.9111769	0.535283333	neg													1000.633657	999.3562423	1340.089014	959.4471195	1183.242288	1119.721596	1129.737848	787.560647	1018.055898
0.54_248.0802m/z	248.0801837	0.535283333	neg													158.7364693	127.8803984	464.5212589	69.52188676	189.0793752	119.7972819	235.228889	104.5441006	178.772059
0.54_285.8484n	284.8410825	0.535283333	neg													1405.006005	1524.362339	1663.29124	1248.694796	1223.596618	1661.39704	1529.292442	1116.69553	1203.658996
0.54_290.8541m/z	290.8540541	0.535283333	neg													1046.578302	1097.573813	1315.221535	931.3908609	1243.426902	1624.40608	1213.638075	785.0304473	954.5291262
0.54_297.0474m/z	297.047405	0.535283333	neg													2516.047254	403.0745602	187.490662	357.1739679	223.0798806	1444.562585	308.732691	438.5255489	151.2314243
0.54_311.1691m/z	311.169144	0.535283333	neg													251.3816041	320.1472427	302.9460136	210.2782699	86.26802139	213.7285344	279.3379019	83.88888359	130.81295
0.54_313.0789m/z	313.0788581	0.535283333	neg													17475.41133	702.8837825	565.5393524	615.3424377	1467.612278	13365.56616	678.760342	2886.696976	418.4989366
0.54_316.7788m/z	316.7787754	0.535283333	neg													82.15848964	218.1862437	175.5223277	96.75990419	73.42116518	277.8732937	137.7833759	46.6440258	39.99002438
0.54_320.8715m/z	320.8714555	0.535283333	neg													1091.216453	814.3584426	948.1898557	359.8702281	654.5739223	569.6678579	932.5349242	469.1403847	708.3014732
0.54_325.1848m/z	325.1847644	0.535283333	neg													466.0992894	537.5232583	288.6542803	271.6260608	178.1842836	341.4801194	475.7025105	97.7843473	208.0123241
0.54_339.2004m/z	339.2003944	0.535283333	neg													436.5210375	526.0411676	306.9278212	310.5419411	251.6480974	345.3646123	468.8494526	112.1093978	238.3794959
0.54_348.8128m/z	348.8128086	0.535283333	neg													1003.51924	707.1900895	1091.156358	463.9033568	632.3750146	584.7186341	1188.467936	555.072506	626.5538315
0.54_352.8286m/z	352.8285678	0.535283333	neg													3352.681746	2885.852128	3635.088246	2607.254713	2908.56729	3045.458211	3370.124717	2389.061531	3264.602721
0.54_354.8289m/z	354.8289199	0.535283333	neg													691.2843036	624.1229188	768.8113395	418.5011425	461.7829151	720.8559872	578.6809961	297.3088207	402.7563064
0.54_363.7915n	362.7842216	0.535283333	neg													405.1059522	428.1061496	342.9751643	1147.23786	359.3627462	1590.284615	494.2162118	267.1146507	237.5784386
0.54_382.8671m/z	382.8670696	0.535283333	neg													1623.551286	1368.163225	1577.337263	884.5094289	1243.3504	1232.016694	1425.320302	1019.444765	1331.720803
0.54_481.8699m/z	481.8698794	0.535283333	neg													353.112298	318.7890787	418.0459374	227.1162921	803.0227201	375.6612901	346.3669159	359.6222924	600.8356091
0.54_494.8169m/z	494.8169105	0.535283333	neg													1838.214615	1933.197476	2261.144496	1608.66989	1870.82707	2030.177337	2312.571946	1252.486022	1374.840578
0.54_590.8226m/z	590.8226165	0.535283333	neg													2754.007069	2464.460484	2507.013989	1402.368554	2349.154824	2230.54829	2565.814895	1834.105537	2190.73387
0.54_620.7669m/z	620.7668667	0.535283333	neg													1112.000519	1187.466063	1107.906799	1027.963039	579.3317588	975.9833504	1081.279912	525.6053925	641.1709907
0.54_668.7668m/z	668.7667881	0.535283333	neg													1550.682061	1386.558632	1526.405933	550.3265762	755.135236	1005.577353	961.1188205	534.8967979	421.3758137
0.54_724.8036m/z	724.8035836	0.535283333	neg													4628.371652	5028.663014	4677.027364	2756.590743	3049.980746	4038.46072	4711.263368	3290.71664	4517.471563
0.54_729.1785m/z	729.1784595	0.535283333	neg													108437.3276	321.0110751	79.91819953	107.6668967	0.001403199	0.001403199	147.3125541	251.9201069	645.9039581
0.54_736.7542m/z	736.754244	0.535283333	neg													733.9707365	603.8701228	606.1279719	316.8877286	336.902019	691.3383176	813.0609043	298.9089279	711.0130909
0.54_870.8036m/z	870.8035545	0.535283333	neg													3087.554301	3164.029463	2995.415739	2778.468977	2101.033789	3630.174965	2602.511742	1603.935829	2117.489879
0.56_132.8679m/z	132.8679467	0.557883333	neg													16851.50247	14549.06321	15755.47278	7875.07107	10932.48017	9415.581829	13512.07045	8420.17766	9605.353323
0.56_134.8654m/z	134.8653622	0.557883333	neg													15142.93404	14345.55706	15594.1653	6663.494636	10719.04957	8201.985204	13033.40969	7656.057027	8652.133601
0.56_136.8632m/z	136.8632114	0.557883333	neg													5381.638199	4969.137816	3368.359064	2445.386106	1687.968487	2075.571489	4511.65614	2792.638381	1810.893902
0.56_138.8603m/z	138.8602637	0.557883333	neg													640.2357282	613.4538882	447.6855651	290.9129876	202.432838	285.0330218	576.9408567	384.2292596	211.7049331
0.56_144.9434m/z	144.9433918	0.557883333	neg													1594.403235	1358.047023	1213.819861	1128.456934	1043.810726	1135.569513	1140.453887	952.5331304	1111.500097
0.56_182.8967m/z	182.8967348	0.557883333	neg													3158.579859	3073.199784	3654.4341	1769.981023	3045.174432	2859.749171	3246.947038	2293.077215	2713.710509
0.56_184.8900m/z	184.8899602	0.557883333	neg													556.0965166	623.5425774	546.0231521	579.9248977	533.1274125	804.9596898	581.7752558	454.2823607	515.4958511
0.56_223.7985m/z	223.7985265	0.557883333	neg													1644.185116	1477.666123	1273.696387	1010.997655	1021.586538	1325.740424	1280.431016	798.4065733	801.0715914
0.56_230.7665m/z	230.7664603	0.557883333	neg													743.7866671	407.6152361	215.1368659	282.2882344	70.2196899	137.724191	295.6256796	184.1117978	63.60352485
0.56_232.7641m/z	232.7641016	0.557883333	neg													1636.442196	1098.191631	733.4316741	541.020015	326.535809	512.1092807	778.9711468	643.4470987	332.4835264
0.56_234.7617m/z	234.761656	0.557883333	neg													1255.50077	831.1767782	515.3259172	365.1589602	234.641274	323.6414707	538.8886462	390.6649692	259.9702004
0.56_248.8873m/z	248.8872915	0.557883333	neg													3472.918435	3435.29828	3793.767824	2298.179133	3284.948864	3404.934799	3279.550715	2516.810414	2853.693342
0.56_294.9137n	588.8251376	0.557883333	neg													7891.940993	7529.804302	8308.012177	5068.37568	7812.218423	6738.002908	7591.2963	6247.594681	7802.417738
0.56_305.8407m/z	305.8407158	0.557883333	neg													1932.412452	1483.261561	1689.649881	1423.055896	1322.995986	1601.105883	1437.045376	1255.282434	1223.553573
0.56_314.8798m/z	314.8798189	0.557883333	neg													569.5322457	453.0219702	520.1768115	285.7871809	328.6575312	392.2160951	427.7440043	192.0838225	345.9199575
0.56_316.8749m/z	316.8748622	0.557883333	neg													11364.46575	9787.584413	10613.8053	7085.738715	8813.032124	8743.309649	10418.08982	6756.714781	8014.366575
0.56_318.8719m/z	318.8719121	0.557883333	neg													3600.764132	3035.347853	3947.177065	2083.117657	3005.260436	2841.824752	3298.758839	2507.960123	2768.348531
0.56_327.8231m/z	327.8230848	0.557883333	neg													979.811152	739.8860384	928.2394841	627.8082866	856.2952055	624.836681	773.070015	674.1140666	796.9978279
0.56_358.8415m/z	358.8414769	0.557883333	neg													2529.032456	2379.636127	2632.277908	2073.407356	2516.005656	2864.244174	2441.911744	1657.888808	2314.006569
0.56_369.8309m/z	369.8308649	0.557883333	neg													540.2145997	461.7896542	800.8862759	384.7006406	559.7133742	465.0452812	648.7898509	464.8665237	611.1400739
0.56_376.8863m/z	376.886276	0.557883333	neg													305.7221723	330.5955553	912.9176624	503.5561122	644.5498751	591.3666446	555.1511638	456.1496544	480.3735676
0.56_384.8625m/z	384.8624849	0.557883333	neg													24273.07352	20982.77427	21329.08255	15435.31163	18162.82011	17524.23035	21699.69703	14305.88447	16005.31276
0.56_395.8102m/z	395.8102316	0.557883333	neg													780.5072243	1031.056386	1158.975889	526.5277121	756.0975728	619.3149046	963.1658766	876.1459888	788.3689475
0.56_420.8162m/z	420.816206	0.557883333	neg													2294.719678	1843.572876	2664.298329	1691.667388	1753.141364	2339.931196	2172.403616	1709.055025	1829.820133
0.56_426.8290m/z	426.8289816	0.557883333	neg													2156.399737	2138.721554	2141.812002	1727.661811	1651.905932	2400.037563	2453.146613	1570.842873	1690.065957
0.56_452.8500m/z	452.8500252	0.557883333	neg													8210.466943	8066.859337	7716.161767	5549.790174	6693.601149	6569.126665	8200.236732	5583.370832	6384.264036
0.56_454.8480m/z	454.8479944	0.557883333	neg													4123.509515	3564.662168	4012.04572	2244.614905	3880.120368	3377.74122	3715.780209	2985.586751	3259.268426

0.56_476.8308m/z	476.8308219	0.557883333	neg	940.9549705	931.3090835	956.8832051	507.1019888	500.668473	1040.805612	1011.140414	361.3357925	467.4840541
0.56_488.8041m/z	488.8041335	0.557883333	neg	1724.726847	1592.597904	1602.952544	1716.759896	1835.507892	1628.220794	1441.010832	1487.786134	1643.352192
0.56_503.7884m/z	503.7884101	0.557883333	neg	817.2579763	738.62794	807.284211	606.2141925	572.1397399	685.1445296	671.6001851	265.414701	888.4184937
0.56_515.8159m/z	515.8158598	0.557883333	neg	4632.918803	3785.392026	3207.225134	2556.179109	7756.024922	5605.673541	2971.852779	3239.379928	4413.936415
0.56_520.8373m/z	520.8373249	0.557883333	neg	6688.315959	6363.622679	6150.577322	4489.273061	5123.052221	5230.943181	6589.157114	4303.565492	5120.503721
0.56_529.8698m/z	529.8697512	0.557883333	neg	693.1825552	738.645038	463.5859046	691.7894109	635.5703277	968.0485161	809.8379202	301.3197021	567.9520446
0.56_530.7927m/z	530.792746	0.557883333	neg	6452.842482	4836.495691	3859.832107	3365.023261	3307.422776	3025.380114	4517.048091	2996.642754	3198.876884
0.56_538.8256m/z	538.8256143	0.557883333	neg	5696.174471	4085.597382	3900.008578	2700.288033	3209.452554	3040.778475	4287.064148	2729.922379	4000.678844
0.56_598.7802m/z	598.7802029	0.557883333	neg	5529.684979	4743.9275	5560.458727	3309.455284	2885.26512	3296.466816	4605.604986	2747.857006	2804.504174
0.56_606.8134m/z	606.8133529	0.557883333	neg	4681.113495	4205.432786	3486.397265	2633.134858	2141.83768	1976.096958	4051.834217	2221.436442	3023.509101
0.56_666.7682m/z	666.7681567	0.557883333	neg	2947.315852	2912.493462	2992.074634	1825.782907	1867.916444	2320.709899	2732.994049	1584.333365	1772.196266
0.56_734.7564m/z	734.7564229	0.557883333	neg	1849.041944	2184.530781	1605.305615	1536.934334	1428.292734	1551.803409	2221.222161	859.4424438	1387.036655
0.56_803.8188m/z	803.8188463	0.557883333	neg	375.7282892	706.4916641	462.163243	545.1108096	356.086681	596.8305426	541.7566969	162.5031959	202.8160642
0.56_812.7723m/z	812.7722827	0.557883333	neg	6509.218484	5674.117014	4798.837456	3539.433046	5307.764747	4677.689312	5603.736611	2475.109494	3277.060298
0.58_102.9487m/z	102.9487493	0.58075	neg	945.4376954	731.8192023	684.0077513	780.8534599	693.4359965	865.8631323	828.5995781	635.3326891	604.6121986
0.58_103.9203m/z	103.9203034	0.58075	neg	566.8261446	558.7562859	606.7009244	531.2224742	511.2969526	597.5763092	601.3749851	587.7170893	518.7195714
0.58_104.9539m/z	104.9538778	0.58075	neg	371.896961	418.7002355	345.3580482	281.2505169	260.2346764	292.3652887	415.0376913	322.8347397	266.4191655
0.58_119.9470m/z	119.9470278	0.58075	neg	265.7709445	265.3424733	257.4420936	216.3276586	192.254277	217.6938661	316.6764592	250.2016799	211.9063397
0.58_140.9807m/z	140.9806598	0.58075	neg	254.9038874	283.9428607	240.2146615	237.4273571	247.1162109	257.5195774	296.7976073	269.1712914	242.7170804
0.58_146.9660m/z	146.965986	0.58075	neg	2971.983125	3066.418652	3621.687032	3203.002584	3412.794791	3219.405008	3480.646226	3014.538949	3200.011173
0.58_152.8951m/z	152.8951488	0.58075	neg	1133.675633	1119.487325	1174.779242	1143.250025	1087.73957	1078.220348	951.8609254	933.1386841	1074.308727
0.58_156.8949m/z	156.8949297	0.58075	neg	1356.280022	1244.457717	1244.949094	1258.609081	1353.291167	1273.120873	1422.676667	1238.646397	1337.250076
0.58_158.8933m/z	158.8932565	0.58075	neg	835.0768561	777.6636841	913.0301386	769.0693986	870.3380886	912.0295169	847.6770823	849.9342681	851.1787656
0.58_163.9290m/z	163.9289561	0.58075	neg	3868.020467	4699.704719	3847.565123	3432.31915	3507.858472	3724.38866	4568.908853	3872.17675	4476.49585
0.58_164.8956m/z	164.8955504	0.58075	neg	793.6783449	874.5815611	975.5268282	865.1369426	940.170719	911.175544	962.5147383	925.2447104	945.443936
0.58_177.9208m/z	177.9207918	0.58075	neg	422.0567384	348.9380877	267.9451968	387.2211748	270.6716914	251.6115109	391.1662707	263.1889763	226.7231668
0.58_178.8155m/z	178.8154944	0.58075	neg	348.0918043	324.1782645	226.9276001	277.1426646	65.97337661	157.6621251	345.2015498	184.1669502	89.45221721
0.58_179.9365m/z	179.9364516	0.58075	neg	4498.790302	4303.487345	4847.352123	4128.886393	4202.343456	4199.087134	4489.739786	4122.192955	4668.194048
0.58_187.9290m/z	187.9290378	0.58075	neg	694.4546626	674.8584089	591.9731546	591.7285887	870.8731342	814.4102394	548.712476	601.3427012	818.4980722
0.58_188.9130m/z	188.9129526	0.58075	neg	720.7445435	684.5290125	587.3465184	753.6789715	665.4575137	654.5739152	698.9838957	601.3676688	708.9859881
0.58_188.9333m/z	188.933288	0.58075	neg	3917.087987	3213.661437	3292.313874	2637.19313	3709.566881	3550.074384	3196.111372	2866.176358	3581.995053
0.58_190.9286m/z	190.9286158	0.58075	neg	88605.45229	75488.27944	75770.04382	68785.67774	73113.0286	80562.6682	76641.64422	62724.83161	67999.57835
0.58_196.8850m/z	196.88504	0.58075	neg	1895.43304	1643.464424	1939.366412	1860.336226	2544.292104	2581.125079	1549.045223	1799.945711	2209.044044
0.58_198.9365m/z	198.9365124	0.58075	neg	604.6593247	641.5372146	670.4333607	671.6741688	452.5706428	596.1613292	672.7703797	634.821764	519.8628189
0.58_205.9157m/z	205.9157089	0.58075	neg	5989.186021	5880.437669	6642.936853	5638.737237	6458.466039	5834.844428	6171.662188	5913.944253	6526.957863
0.58_207.9313m/z	207.9313427	0.58075	neg	32225.2054	31549.07276	32201.22563	29386.76568	30748.6305	30766.46165	33281.86748	29991.76389	31985.88594
0.58_213.9185m/z	213.9185126	0.58075	neg	685.3618354	729.5386526	716.8577233	543.2768814	610.4695005	590.8293312	523.4900304	528.2337689	587.3246661
0.58_221.8007m/z	221.8007196	0.58075	neg	1079.58365	794.8845777	660.7593568	712.0567844	411.9940747	449.8704121	837.5695729	578.1959686	367.0258968
0.58_223.8819m/z	223.8819275	0.58075	neg	340.6274172	317.5222463	359.6097477	233.1053918	348.9150949	260.8958399	324.647026	300.3842117	247.1821242
0.58_225.7962m/z	225.7961992	0.58075	neg	858.9135139	705.6486269	803.0635861	549.0276221	558.2915256	641.5104365	547.6421288	453.5466357	461.6361594
0.58_232.9057n	464.802768	0.58075	neg	2276.284371	1792.355052	1253.09152	1106.227754	1117.770386	915.4240983	1896.789729	1228.650827	1018.185803
0.58_247.9239m/z	247.9238962	0.58075	neg	1848.666873	1695.595612	1731.609711	1719.948336	1366.93611	1714.254097	1843.746032	1810.235091	1499.636514
0.58_250.8839m/z	250.8839491	0.58075	neg	862.527727	622.2834502	497.458698	600.3915603	540.7870527	640.6432581	638.8257801	505.1599426	454.1025909
0.58_256.9208m/z	256.920808	0.58075	neg	1347.837969	1309.7476	1292.250189	1014.489151	1486.160062	1487.234522	1366.347502	1221.333059	1421.046228
0.58_258.9159m/z	258.9159447	0.58075	neg	41286.28021	35171.34339	37067.09814	32479.09335	34835.35247	37211.24501	36514.12492	29516.65851	33100.11913
0.58_261.8511m/z	261.8511167	0.58075	neg	689.2495682	667.32471	616.0976599	408.2415408	472.3378848	413.4057058	579.8380633	569.3460159	417.7054856
0.58_262.8584m/z	262.8584338	0.58075	neg	2117.979577	1640.185238	1323.381272	1687.574373	1103.033521	1569.11297	1495.508	1169.478252	909.2879652
0.58_264.8725m/z	264.8725238	0.58075	neg	3535.824146	2874.906261	2894.801945	2859.009639	2793.561399	3413.278646	3029.836581	2494.410215	2714.131723
0.58_273.9034m/z	273.9033749	0.58075	neg	933.7891373	1067.784986	1037.889758	942.2393553	700.1067521	1130.196083	1056.628811	1171.025439	949.9336604
0.58_296.8905m/z	296.8904849	0.58075	neg	1099.520179	1203.430715	820.6709837	751.9291162	464.4329761	692.0537497	1341.651607	808.8415293	588.1538776
0.58_297.8275m/z	297.8274753	0.58075	neg	1094.970639	1171.156295	888.1402521	1104.459888	529.6077242	967.0481845	1094.105638	751.4469193	755.5187702
0.58_297.9008m/z	297.9008423	0.58075	neg	5673.316631	6152.498256	6472.949789	5782.985323	6747.690878	5867.379033	6430.499893	6266.519757	6778.129635
0.58_307.8562m/z	307.8562467	0.58075	neg	10319.13314	8365.236654	7020.790745	7674.829036	6396.972744	6887.962273	7572.385622	6581.417202	6362.225002
0.58_323.9104m/z	323.910368	0.58075	neg	507.1914018	659.1934912	500.0124517	686.8216019	275.9828127	554.6606015	622.3418086	528.6133814	290.4489103
0.58_326.9036m/z	326.9035845	0.58075	neg	97838.62544	83277.93723	85513.15932	72775.04644	78635.72561	82388.15014	85538.74405	69142.1939	74528.2202
0.58_332.8602m/z	332.8602039	0.58075	neg	6569.75858	5215.398144	4905.404416	4900.837144	4405.542954	4861.631305	4514.948375	3629.365669	3966.620851
0.58_341.8906m/z	341.8905753	0.58075	neg	2708.691451	2909.486418	3352.452055	2633.711633	3255.45246	2567.030487	2828.898045	2610.110517	2879.622229
0.58_355.8596m/z	355.8596414	0.58075	neg	1734.821715	1810.098887	1609.035681	1328.878681	1159.415492	1392.60583	1758.345977	1426.522875	1131.37485
0.58_365.8883m/z	365.8882973	0.58075	neg	12138.48778	12923.61653	14730.97677	11724.89241	13886.19006	12184.83767	13145.56118	11410.79506	14380.37127
0.58_371.9012m/z	371.9011592	0.58075	neg	7389.386747	7766.625793	7737.067738	6133.782502	6632.641479	6351.495062	7583.350354	5701.388838	6765.884761
0.58_373.8281m/z	373.8281213	0.58075	neg	5046.949163	4575.347642	4473.649669	3445.302489	4176.47387	4202.548264	3789.053606	3466.520225	3635.141551
0.58_375.8436m/z	375.8435837	0.58075	neg	9104.759211	7258.90746	6535.234437	6669.991865	5471.678042	6711.192696	7365.951077	5915.436473	5172.879153
0.58_376.8516m/z	376.8515835	0.58075	neg	8774.812023	6118.726934	6488.740466	5914.764706	5322.277883	6853.142967	5843.218324	4874.561647	5391.386092
0.58_392.8959m/z	392.8958614	0.58075	neg	5338.959191	5059.107578	4785.706767	4376.760272	5091.395869	5303.671586	4931.976199	4147.388516	5505.620327
0.58_393.8513n	374.8334265	0.58075	neg	3733.535963	3296.598478	3207.304716	2164.322405	2642.752836	2477.494107	3244.433539	2387.95836	2414.369204
0.58_400.8473m/z	400.8472846	0.58075	neg	4400.562379	4334.637492	5015.712137	3618.920709	5232.129741	4515.063848	4508.327443	3908.84019	4166.461212
0.58_404.8460m/z	404.8459618	0.58075	neg	5663.064378	3973.764138	3739.294239	3965.352788	3440.630598	3901.576274	3930.575366	3274.637223	2947.068837
0.58_409.8776m/z	409.8776199	0.58075	neg	4160.922076	4380.845009	3829.490457	3684.004759	2900.611621	4105.358809	3983.486654	3809.944309	3254.055141
0.58_411.8814m/z	411.8814018	0.58075	neg	27657.45447	25429.55455	27866.30673	26768.7039	29630.84175	28287.43313	29172.25154	27763.76859	29599.04773
0.58_418.8260m/z	418.8260282	0.58075	neg	1092.454913	856.6993794	684.4650891	931.9669833	646.5540956	857.6836916	881.6680504	837.5775852	723.6884832
0.58_423.8473m/z	423.8472526	0.58075	neg	1123.277279	1416.737266	1657.64373	1148.90117	1530.529533	1137.142715	1332.42083	1162.083697	1330.658612
0.58_433.8023m/z	433.8022984	0.58075	neg	2941.326951	2866.445191	3559.261516	1949.676208	2727.43703	2097.662859	2445.18869	2424.528273	2524.818246
0.58_433.8758m/z	433.8757998	0.58075	neg	8256.007458	8759.876382	9955.213401	7298.597602	12124.39606	10664.57154	9624.795634	9303.451913	13641.007
0.58_441.8156m/z	441.8156337	0.58075	neg	6464.83314	5614.266834	5842.372824	4362.428427	5420.594888	4660.09654	4757.440243	4483.168991	5146.240491
0.58_443.8312m/z	443.8311647	0.58075	neg	16208.8155	13595.59033	13343.96141	11309.93668	12241.07325	11867.97112	11812.72733	10212.33658	11194.33291
0.58_444.8378m/z	444.837771	0.58075	neg	5517.209004	4083.847205	5230.978579	3497.27545	5606.223019	4314.219599	4118.578552	3699.289277	5459.144834
0.58_447.8282m/z	447.8282022	0.58075	neg	1048.250498	941.547379	1343.94695	734.2670431	1048.496444	1477.270146	939.9078926	932.3118838	1095.22061
0.58_460.8832m/z	460.8832326	0.58075	neg	2009.270914	1679.117126	2068.497442	1322.826589	2430.312325	1994.090462	1869.395683	1572.802468	2241.397756
0.58_462.8055m/z	462.8054568	0.58075	neg	5997.641378	4952.088046	2720.231695	3034.626964	2431.281218	2831.848554	4317.193137	2676.202499	2091.265267
0.58_462.8787m/z	462.8786888	0.58075	neg	73764.05275	64586.97536	66561.55633	54403.37359	57244.90796	61309.27927	68137.90396	54202.96918	58964.12542
0.58_468.8348m/z	468.8348174	0.58075	neg	2574.583347	2513.023349	2976.880755	2280.817669	2769.397543	2624.792814	2595.899181	2248.974653	2652.387152
0.58_470.8378m/z	470.8378022	0.58075	neg	5862.57113	4283.317758	4449.453032	3837.78199	3409.741944	3886.824016	3746.622126	3364.553815	4537.144508
0.58_472.8340m/z	472.8339852	0.58075	neg	34686.42458	25753.63998	25179.54808	22459.71171	22103.78335	24736.04044	24579.62908	18058.33417	19914.31582
0.58_476.7760m/z	476.7760454	0.58075	neg	5615.455067	3196.111605	3154.50569	3388.218325	2817.61941	3654.509235	2956.563085	2123.522646	1945.018478
0.58_479.8688m/z	479.8687867	0.58075	neg	6717.520824	6848.407514	6887.062085	6098.042429	6803.277544	6113.693578	7185.062612	6190.344129	6426.316702
0.58_486.8135m/z	486.8134734	0.58075	neg	1461.589315	1693.05992	1972.112014	1356.070394	1672.180777	1891.549027	1717.674365	1330.500565	1327.980798
0.58_488.8289m/z	488.8288641	0.58075	neg	540.785756	643.429113	670.3662726	481.7836911	498.741652	285.2270027	391.1846251	192.7565498	529.8069812
0.58_501.7899m/z	501.7899435	0.58075	neg	1214.323017	971.3308561	1337.875769	900.3001777	1087.193179	966.4904178	1134.122489	1095.763454	894.1591322
0.58_501.8634m/z	501.8634134	0.58075	neg	5071.746851	4966.986973	5515.663259	4231.671654	5446.901728	5170.711414	4965.238757	4171.270409	6450.368491
0.58_509.8033m/z	509.8033165	0.58075	neg	6022.279161	5211.266118	5605.449699	4359.49437	5431.376083	4724.063494	5176.576074	5044.612102	5484.234574
0.58_511.8190m/z	511.8189705	0.58075	neg	10203.00559	8942.2337	9682.898857	7446.673488	9574.206068	7775.053778	9668.869477	7673.793335	9453.229461
0.58_519.7590m/z	519.7589877	0.58075	neg	981.9944253	533.5474222	437.5797539	490.1523666	293.5667495	451.3986274	436.9621713	308.5975216	336.0989195
0.58_521.7741m/z	521.7741226	0.58075	neg	10486.03878	7940.800799	6029.162895	5253.822233	6094.672851	5609.821311	7125.681729	4824.666803	4709.895251
0.58_536.8250m/z	536.8249722	0.58075	neg	3199.669645	4870.22875	3253.529618	2917.828595	2633.556795	3396.795542	3883.999265	3501.072296	3931.424126
0.58_540.8216m/z	540.8216102	0.58075	neg	20944.03384	15402.46848	16965.70393	12086.7683	15595.97185	15416.35069	14310.63557	11259.28859	13693.06445
0.58_545.8535m/z	545.8534806	0.58075	neg	1897.13827	2582.827919	2426.455783	2122.544235	1856.11667	2301.556165	2201.574252	2023.557251	2007.238423
0.58_554.7999m/z	554.7999088	0.58075	neg	1566.538992	1581.890054	853.9384324	1082.086001	838.7292306	1125.812902	1283.396445	734.3326797	956.3923008
0.58_569.7777m/z	569.777678	0.58075	neg	1063.934137	1156.717889	1286.613593	950.3537929	917.6626913	873.0431026	1012.997206	760.9958458	732.1605127
0.58_570.8582n	615.8394531	0.58075	neg	6635.382404	7359.30478	6090.724111	6425.332834	7320.906072	7263.785242	6843.350382	5675.899574	6075.393501
0.58_577.7909m/z	577.7909361	0.58075	neg	5833.970261	5362.143045	5246.217797	3702.946628	4470.54341	4376.388353	4610.288315	4216.293799	4260.4657
0.58_579.8071m/z	579.8070935	0.58075	neg	10796.85253	8979.233962	9135.459894	7057.199198	8424.647329	7388.666997	8843.778174	7141.411847	8663.27469
0.58_587.7471m/z	587.7471126	0.58075	neg	1220.070369	808.2447344	557.3867007	451.1552078	450.5204414	679.3258908	1223.916902	760.9300919	550.9643592
0.58_608.8094m/z	608.8093825	0.58075	neg	20113.1463	15902.8195	16177.525	12128.95715	15804.42802	15034.58941	15171.66728	9850.158023	12531.84615
0.58_612.7527m/z	612.7526579	0.58075	neg	1838.806667	743.1637294	574.8282112	793.7228752	206.0404449	0.001403199	363.7645469	523.8854271	515.1899277
0.58_622.7868m/z	622.7867809	0.58075	neg	1376.496872	1373.195358	1504.695079	710.8837182	739.2343383	1480.872864	526.755287	801.469925	1068.576409
0.58_645.7776m/z	645.7775984	0.58075	neg	4245.308214	3566.968514	4492.503233	3731.811994	4133.116589	4265.498558	4510.581358	3925.285298	3509.882804
0.58_647.7947m/z	647.7946822	0.58075	neg	7715.484573	7091.4873	7253.559075	5393.757786	4908.206573	5295.760543	6100.475996	5364.352515	6070.912197
0.58_663.8416m/z	663.8415574	0.58075	neg	542.5366482	458.5149201	372.7966786	950.752404	1153.218455	339.5414303	1185.780974	806.6098874	810.1496024
0.58_676.7969m/z	676.7968523	0.58075	neg	14547.39812	11848.57431	12950.60004	9287.653115	11122.83892	11988.92505	11919.75317	8704.621318	10089.4746
0.58_683.8308m/z	683.8307832	0.58075	neg	1804.909497	1657.869528	1705.350645	1998.129535	1350.447805	1476.590162	1885.489912	1471.990898	1194.806184

0.58_709.8233m/z	709.8233217	0.58075	neg	1960.660074	2697.949297	2524.698619	1830.429313	3995.284944	3014.173765	2613.50738	2221.080548	2592.654518
0.58_713.7645m/z	713.7645078	0.58075	neg	2499.213071	1920.116629	2837.315878	1497.897748	1574.077342	1950.832029	1690.3137	1764.356147	2252.477421
0.58_715.7816m/z	715.7816149	0.58075	neg	4398.778866	4299.942565	3818.027727	2270.91868	2683.547737	4097.541823	2781.053924	3188.059895	2795.697929
0.58_744.7846m/z	744.784552	0.58075	neg	9185.116867	8414.314219	8019.624878	6433.688856	6055.257783	7866.178178	8488.114887	4467.442933	6014.53613
0.60_105.9359m/z	105.9359283	0.6034	neg	152.1129565	153.3552576	158.8807784	162.09326	107.4302896	161.3865352	165.2496652	170.1406985	150.0197622
0.60_142.9963m/z	142.9962606	0.6034	neg	227.2086362	247.8955079	286.5325098	270.5626958	207.47388	303.2753343	311.1597365	231.2214279	232.1666379
0.60_187.9419m/z	187.9419393	0.6034	neg	472.0709367	501.9458076	323.0992377	374.8240289	266.542107	242.0514443	387.9549484	229.8949566	104.0231447
0.60_190.8886m/z	190.8886119	0.6034	neg	421.3862841	426.5335682	434.0996243	511.789771	325.8837269	379.264796	551.509395	424.6381607	438.564087
0.60_239.9033m/z	239.9033147	0.6034	neg	535.2988098	499.6458719	441.6665483	486.2456618	336.3451885	475.280612	476.9215862	278.0088463	327.0961345
0.60_447.9198m/z	447.9197707	0.6034	neg	1122.158209	867.4225432	1080.915357	887.5617084	886.4912044	398.3230709	1815.232719	255.1326447	703.1545213
0.60_528.8693m/z	528.869319	0.6034	neg	3292.743008	3882.343498	4743.633925	3902.49049	4065.770784	4306.452278	4378.097754	3590.375447	3838.831769
0.60_567.8375n	612.8296931	0.6034	neg	3787.724519	3807.29747	3035.129153	1920.217215	2905.74274	2454.259083	4632.483801	3432.976872	2600.640601
0.60_593.8499m/z	593.8499006	0.6034	neg	1300.293409	1411.582908	1513.459258	806.0522038	1608.306295	150.9068188	1667.77469	1588.12945	758.3807973
0.60_596.8561m/z	596.8561344	0.6034	neg	3639.871459	3035.808318	4556.105387	2766.863477	3737.018585	3302.039538	4542.744407	2856.489475	5069.008874
0.60_623.7844m/z	623.7843758	0.6034	neg	2056.94303	1872.210861	1665.956102	1610.671722	2109.163102	1749.096077	2121.891934	2094.075408	1346.995543
0.60_625.8007m/z	625.8006547	0.6034	neg	2564.428154	2171.024362	1516.276404	1804.091861	2253.777923	1837.418264	1892.024118	972.2287827	920.792493
0.60_664.8428m/z	664.8428261	0.6034	neg	1254.878536	2106.344734	1859.689477	1177.557693	1332.130688	543.5412573	1056.666323	1645.111088	1195.229387
0.60_674.8006m/z	674.8006357	0.6034	neg	2961.3001	2101.497785	1185.487851	1611.818136	2012.134085	1066.434573	1733.374358	2567.072656	2216.822292
0.60_693.7875m/z	693.7874685	0.6034	neg	2157.717159	2223.351622	933.3744873	1470.41197	1455.275598	1513.967831	1640.059239	1402.25901	781.4680825
0.60_728.8114m/z	728.8113996	0.6034	neg	3004.345344	2944.303389	2880.908697	1291.975127	1967.220785	1284.881426	3333.348553	1379.594894	2219.385229
0.63_114.9887m/z	114.9886631	0.62565	neg	924.0473192	913.7378092	1187.161233	1328.555021	1734.001368	1364.856364	1414.288233	1788.861293	2051.566041
0.63_118.9663m/z	118.9663052	0.62565	neg	292.5874973	403.4679447	306.7543752	263.4544707	246.5543109	367.6984057	413.8440602	377.7885662	417.3197873
0.63_120.9549m/z	120.9549102	0.62565	neg	321.2558071	358.0385185	371.9015479	402.4779794	370.2276771	441.5963509	419.1800706	365.0125705	368.2305197
0.63_122.8943m/z	122.8942694	0.62565	neg	768.2813748	868.6502794	771.8433618	831.5335569	721.7834299	821.9258292	1082.819285	897.711999	828.7461528
0.63_123.9021m/z	123.9020551	0.62565	neg	726.6245859	719.3555923	689.9323293	792.9558178	571.8264095	691.6152206	786.2405704	756.6549038	551.8900282
0.63_127.8703m/z	127.8703499	0.62565	neg	411.0938724	515.7683286	574.2207026	546.9411339	373.4044233	598.0692127	688.8200818	615.3599027	407.5213568
0.63_130.9837m/z	130.9836927	0.62565	neg	5925.507004	6567.063123	6864.791172	8176.911355	8583.329511	8590.870119	8990.044944	9098.088377	9457.470099
0.63_146.9614m/z	146.9613531	0.62565	neg	4102.394399	5915.355548	4509.967942	3835.511432	4200.203528	4246.553466	5615.190352	5531.540681	6188.525534
0.63_148.9498m/z	148.9498021	0.62565	neg	2846.627027	3217.312388	3284.891727	3792.87507	4233.660658	3722.808586	4111.416827	4024.595937	4163.258599
0.63_150.9470m/z	150.9470166	0.62565	neg	1093.356258	1117.228204	1316.791391	1375.451341	1507.84004	1366.942779	1699.819781	1485.284419	1428.3724
0.63_152.9445m/z	152.9445025	0.62565	neg	59.85270518	76.50885958	142.0709542	64.58793432	75.70706159	79.567622	69.24854529	179.088595	134.8238599
0.63_158.9786m/z	158.97864	0.62565	neg	67462.70795	72897.0504	76999.13992	91202.85981	94687.74378	92571.14317	100901.1814	105918.6267	107209.3802
0.63_160.9828m/z	160.9827772	0.62565	neg	776.5628942	911.6766168	1000.033409	1077.050936	1135.326304	1163.895847	1239.592786	1189.015163	1310.145953
0.63_164.9350m/z	164.934979	0.62565	neg	799.2608049	1013.657086	1098.063401	1181.664678	1311.280226	1227.16316	1281.287296	1428.964897	1430.186242
0.63_164.9487n	145.9308274	0.62565	neg	2696.107635	2753.111972	2608.30622	2561.023172	2273.175243	2732.712797	2889.762434	2642.791949	2278.538996
0.63_168.8364m/z	168.8364499	0.62565	neg	186.5168566	202.432429	241.3293442	155.4569534	207.4424792	135.5893542	148.2865558	251.7556789	236.3248088
0.63_175.9688m/z	175.9687967	0.62565	neg	3888.778298	4059.427606	4487.220936	4886.791845	5152.462752	5353.510727	5827.937549	5580.759913	5302.644499
0.63_180.9127m/z	180.9127076	0.62565	neg	394.3349926	647.0180016	438.6656854	300.8940766	312.1164846	415.4054173	596.8579508	478.9601418	406.4034242
0.63_189.8708m/z	189.8707905	0.62565	neg	1229.901363	1528.47697	1773.026757	1572.50072	1824.667448	1638.631735	1922.377121	2103.93283	1890.057602
0.63_193.9740n	174.9561836	0.62565	neg	68719.24777	96178.53856	76620.10832	82568.2938	87747.42059	88639.36563	106182.362	110650.0608	123105.4421
0.63_197.9025m/z	197.9025394	0.62565	neg	6770.425088	7968.146011	8598.743679	7900.430568	8613.513022	7707.494661	9140.830669	9479.441307	9739.162235
0.63_199.8996m/z	199.8996385	0.62565	neg	2068.528006	2550.520673	2375.933323	2543.930263	2447.112921	2566.956444	2844.247229	3023.254361	2764.495692
0.63_207.8369m/z	207.8368873	0.62565	neg	683.7008109	930.0323637	1161.750948	1053.04664	680.479707	1089.127147	1313.928012	1316.974777	692.6522814
0.63_215.8687m/z	215.868655	0.62565	neg	5035.227503	6476.131144	7417.010144	5244.48126	6983.584861	5835.030467	8054.822532	6770.233947	7226.986637
0.63_216.9373m/z	216.9373081	0.62565	neg	1992.550284	1896.531145	2290.953971	2465.574488	2663.080707	2231.031655	3075.075189	2378.216564	2614.181821
0.63_216.9692m/z	216.9691952	0.62565	neg	100.9132121	70.6903442	106.0589791	227.4077898	125.2913955	361.7321848	208.3820456	350.7195601	175.138107
0.63_217.8656m/z	217.8656298	0.62565	neg	3826.607384	5252.747874	5636.221357	4165.788809	5646.027446	4492.993137	6422.610872	5608.729463	6138.286261
0.63_225.8976m/z	225.8975528	0.62565	neg	5361.378475	6736.470859	6548.446471	5948.695857	6540.086592	6189.734813	7624.531679	7163.74373	7521.265491
0.63_226.9662m/z	226.9661985	0.62565	neg	42666.69693	43842.00465	49302.94018	52686.95466	57306.87524	54409.21064	60200.76541	58609.07066	59404.66879
0.63_228.9701m/z	228.9701211	0.62565	neg	476.9139155	625.51623	504.5766498	379.6177089	633.9907786	343.6025897	592.7934174	514.7149641	648.1568761
0.63_231.9162n	276.9140597	0.62565	neg	20453.90541	19301.59911	18143.91303	22080.12617	23770.56023	23245.06069	22728.96218	23815.50514	23276.53439
0.63_232.9229m/z	232.9229271	0.62565	neg	1606.791983	1593.017111	1752.665357	2137.644656	1897.80716	2132.79265	1893.654971	1936.249699	1869.371628
0.63_234.9353m/z	234.9353157	0.62565	neg	588.0592238	787.8273621	964.9426207	1422.688133	1549.422583	1294.587416	1361.957359	1838.636404	1647.745403
0.63_236.9319m/z	236.931924	0.62565	neg	607.372836	624.2068521	669.2765103	841.2093313	1030.517699	1014.835419	764.8556972	1310.244617	1018.645822
0.63_238.9642m/z	238.9642048	0.62565	neg	2221.507829	1515.534776	2184.630794	2091.263582	1678.699921	2767.187052	2307.469686	2358.733795	2399.549878
0.63_244.9510n	289.9492292	0.62565	neg	9391.274719	12839.73188	13785.51575	23007.00879	21133.01986	22232.01514	22947.00875	27164.72834	24018.33481
0.63_244.9641m/z	244.9640893	0.62565	neg	12731.16565	15282.69114	15547.22254	23861.9967	23569.36611	23041.37809	24958.82574	31058.84757	28612.6673
0.63_261.9543m/z	261.9543368	0.62565	neg	1413.376894	1496.075477	1950.508821	2829.875586	2985.467738	3096.872956	3106.149859	3323.398857	3390.336922
0.63_262.9304m/z	262.9303504	0.62565	neg	3111.231174	4625.036669	4126.838161	6745.428061	6261.141242	6472.349743	7463.882	8110.23416	8114.258196
0.63_265.9350n	530.866128	0.62565	neg	77888.51882	76077.01087	72880.46982	58264.06707	66362.40956	67065.15458	79520.10122	67606.77972	76136.09098
0.63_266.8854m/z	266.8853677	0.62565	neg	4273.032034	4322.000879	4075.92617	5465.533941	5881.528407	5346.537641	5394.187822	5416.68293	5198.783272
0.63_268.8824m/z	268.8824395	0.62565	neg	1911.915648	1750.254703	1717.578878	2096.129	2190.464663	2229.011979	2185.983389	2105.134349	2368.842862
0.63_272.9592m/z	272.9592073	0.62565	neg	72773.67752	89899.598	91073.00463	136154.4778	137718.2779	138502.6094	144311.1458	176724.97	166122.4306
0.63_275.9063m/z	275.9062596	0.62565	neg	7583.828795	6955.858153	6898.287915	7796.325893	7317.52979	8162.222615	8187.932145	8508.523995	7940.61884
0.63_278.9085m/z	278.908482	0.62565	neg	2365.384692	4217.134837	3133.745599	3206.714666	3328.449738	2937.770731	4287.968407	4657.344429	4220.446606
0.63_283.8754m/z	283.8754266	0.62565	neg	445.9203827	388.0375916	353.5305929	529.4482314	209.33804	340.696934	459.5027833	530.656847	265.5218855
0.63_284.9249m/z	284.9249467	0.62565	neg	2541.052319	2022.118041	2887.781575	2850.34952	3188.027645	2626.805826	3559.215032	2598.026286	2684.023439
0.63_291.8840m/z	291.8839835	0.62565	neg	2613.394586	3272.455546	2184.787194	2166.292884	1798.898423	2335.357354	2446.074618	2876.002577	2931.19115
0.63_293.9045m/z	293.9044604	0.62565	neg	1848.055094	1745.739691	1611.168101	2436.187425	2837.159273	2361.782544	2167.390299	2005.416131	2398.157064
0.63_293.9369n	274.9190675	0.62565	neg	2985.256095	3672.558535	2899.360626	3806.944041	2904.761295	3442.139671	4265.57265	3538.25018	3973.126758
0.63_294.8801m/z	294.8800561	0.62565	neg	8507.220179	8764.329924	7786.698878	10640.4446	10281.93005	10492.96061	10171.0546	10946.83454	10585.41843
0.63_294.9536m/z	294.9535723	0.62565	neg	39863.20436	40924.44722	45739.53533	45699.36209	49917.17143	47096.13263	54016.0994	48108.6863	52389.70475
0.63_296.8772m/z	296.8772076	0.62565	neg	3501.33294	3607.717053	3743.696535	5008.880189	5232.86436	4774.880403	4724.901114	4966.292827	4935.751248
0.63_300.8325m/z	300.832523	0.62565	neg	994.133683	729.8242263	601.6103325	761.2235226	237.9569656	642.0885988	639.2432855	558.2176135	206.9161461
0.63_301.9201n	300.9102186	0.62565	neg	772.8245411	869.4318541	791.4799102	712.9990049	932.8416269	953.1392526	1274.943732	644.9850748	938.8608012
0.63_302.9140m/z	302.9139639	0.62565	neg	3701.212983	3149.674766	2907.111773	3338.593044	3472.246272	3020.475895	3686.909096	3745.074884	3915.875564
0.63_304.9087m/z	304.9086871	0.62565	neg	37754.00946	33277.46594	32333.48971	42625.67675	44115.16432	43085.61625	41159.71695	42744.81572	40007.19873
0.63_305.9266m/z	305.9266093	0.62565	neg	2582.367349	5055.851927	3722.968678	3948.688694	2559.652732	4248.8952	5785.425031	5372.052628	4571.016705
0.63_306.9391m/z	306.9391215	0.62565	neg	383.8838852	572.1160275	492.6849441	1036.227942	1128.832528	1353.040085	973.6930842	1335.166333	873.2463652
0.63_307.9545n	288.9366914	0.62565	neg	53745.52213	85278.02037	63127.51504	73417.62772	70640.18833	75584.31641	96523.45872	103366.6596	112400.3724
0.63_312.9520m/z	312.9520197	0.62565	neg	2877.913125	3553.595244	3954.604306	5517.210504	6136.014861	5661.11032	5739.327592	6896.13185	6280.868518
0.63_318.8909m/z	318.8909436	0.62565	neg	1042.974225	1381.428994	816.4690521	768.5060698	1047.534585	950.1134828	1059.088545	949.8891593	1108.28474
0.63_319.8964m/z	319.8964104	0.62565	neg	585.4575319	785.3997027	565.2023782	674.076531	722.9891496	785.5581741	796.2741703	366.2168694	727.9475701
0.63_320.8870m/z	320.88702	0.62565	neg	20341.46369	25247.10824	17722.65425	16449.09126	17640.61377	19593.06967	21820.15907	19169.6371	21711.88677
0.63_323.8971m/z	323.8970652	0.62565	neg	452.5313152	293.2519895	156.2432557	389.8310862	282.1310601	336.6154084	372.0924784	627.0248642	282.4921086
0.63_330.9177m/z	330.9176666	0.62565	neg	673.9427064	607.469676	1404.917016	1520.973527	1426.83204	1653.651228	1542.26244	1631.495845	1896.167631
0.63_335.9423n	380.9391492	0.62565	neg	5876.407692	5524.050729	8375.785003	9238.53644	11502.05293	9308.628407	10163.292	11461.0314	10914.1806
0.63_338.8888m/z	338.8888353	0.62565	neg	757.3922054	248.0012284	477.2945393	519.0156431	331.4118911	589.7026222	497.4402434	492.9274961	407.6862542
0.63_341.9590n	340.9466772	0.62565	neg	14999.99627	16749.31657	20487.09314	27289.03202	28990.98765	26760.5927	27273.01528	32170.20551	31877.9932
0.63_343.8940m/z	343.8940155	0.62565	neg	8484.359916	7013.546185	7757.008166	8145.773299	8172.026389	8044.173064	8503.493115	7326.059943	7955.083894
0.63_344.9019m/z	344.9018925	0.62565	neg	5940.740932	5257.934972	5416.798729	5953.26335	6543.671142	6795.06024	6822.904989	5918.873515	6259.176165
0.63_346.8951m/z	346.8950613	0.62565	neg	1929.850164	2002.815598	1741.546817	1555.808747	1618.167278	1487.346067	2233.491624	2106.235279	2389.186208
0.63_356.9243m/z	356.9242953	0.62565	neg	26187.78576	39851.79301	30439.99849	33415.77712	34865.16633	35427.91325	44446.71752	45838.5949	49806.76464
0.63_358.9450m/z	358.9450102	0.62565	neg	747.826633	971.4336242	1026.578292	2512.976053	2356.042264	2338.628381	2244.781495	3786.89739	2349.904418
0.63_373.9057n	418.8932265	0.62565	neg	27412.68451	39988.47097	26405.04695	32030.21292	33870.21153	33830.62521	39356.84212	39743.93797	45618.14937
0.63_374.9261m/z	374.9260953	0.62565	neg	647.2752622	289.4846793	556.0202689	1251.629035	805.3443746	1717.439941	1103.101641	1024.579704	855.5480625
0.63_398.9043m/z	398.9042915	0.62565	neg	831.3445955	986.860798	1663.973457	1044.405591	2349.624309	1736.691978	1809.795185	1878.49132	2006.171012
0.63_403.9244n	402.9171321	0.62565	neg	17035.95484	30900.64993	24413.01446	35207.89481	35912.02138	35690.93823	45870.90198	52515.74088	57136.92394
0.63_408.9342m/z	408.9341632	0.62565	neg	24172.39694	24318.87639	31998.21487	35469.52191	42986.5898	38574.68807	41468.12622	44707.72605	41803.94928
0.63_424.9119m/z	424.9118821	0.62565	neg	22183.43876	33062.77953	28280.5869	26453.00201	29263.82743	28992.0679	34443.84084	35109.04526	40121.03026
0.63_426.9317m/z	426.9317254	0.62565	neg	4801.107428	5891.120744	6657.498234	13198.58416	13274.91206	13174.6359	12356.84131	17102.48446	14348.13617
0.63_435.7994m/z	435.7993647	0.62565	neg	798.6998099	838.9946691	130.3985219	402.1237129	235.8386746	282.8470256	370.819463	0.001403199	229.9338773
0.63_435.8745n	434.8672015	0.62565	neg	13778.01725	18222.75118	12473.94714	15484.56123	13682.05409	15786.43666	18815.52003	18445.27355	18997.32543
0.63_440.8846m/z	440.8845866	0.62565	neg	32634.92684	34924.46739	32042.0456	30156.75847	34767.8525	33791.99031	36575.28766	31913.95423	33308.76277
0.63_442.9099m/z	442.909916	0.62565	neg	3087.395327	4785.397255	4374.372248	6067.883225	4959.427993	5982.99344	5860.719988	8264.4524	8072.749114
0.63_444.8267m/z	444.8266982	0.62565	neg	11317.88379	8461.994213	5924.978321	7047.251855	7258.561483	7448.550879	7695.708039	5505.896324	4450.017202
0.63_455.9282m/z	455.9281736	0.62565	neg	994.8576356	1259.985603	1220.015574	3377.441881	2622.388066	2859.250413	2290.39095	3950.584394	3536.236987
0.63_457.8754m/z	457.8753875	0.62565	neg	5041.292713	7739.643613	7024.958582	8413.323366	9163.031971	8387.548684	9671.136345	9658.056408	11088.28117
0.63_466.8923m/z	466.8922884	0.62565	neg	543.5431839	886.5481863	1189.040962	1035.704225	1679.811004	643.1324631	1414.305888	1204.295407	1423.766893
0.63_471.9121n	470.9048728	0.62565	neg	19360.50751	30532.41704	23101.30345	34508.68232	33157.03837	34354.02731	42587.63888	49515.19931	51358.78168

0.63_472.9252m/z	472.9251817	0.62565	neg	4.130501395	0.001403199	0.162638072	2194.236392	2119.608361	1666.266911	813.3513599	2600.390866	1571.002473
0.63_474.8607m/z	474.8607186	0.62565	neg	2723.345365	3965.370308	2806.973259	2968.36736	2520.545113	4015.080187	4192.476721	4617.795402	3562.389092
0.63_477.8633n	522.8519796	0.62565	neg	2821.453696	3600.599254	2384.600733	2112.292953	1540.677473	2243.214683	3442.032698	3364.768577	2015.29225
0.63_489.8243m/z	489.8242806	0.62565	neg	8676.140464	7282.833589	6436.11797	7295.479255	13823.93282	8448.759918	8027.349728	8897.842506	12010.03113
0.63_492.8259m/z	492.825944	0.62565	neg	2592.684424	3037.397973	1961.957364	2355.653613	2765.198095	2962.139003	3644.133227	2650.296997	3187.255183
0.63_495.9394n	476.9215698	0.62565	neg	16059.39646	18470.908	23126.39645	27663.37508	31402.13788	26804.63284	29105.67392	33718.89836	30717.30034
0.63_501.9213m/z	501.9212577	0.62565	neg	159.5124325	275.8911597	186.8280284	1019.219573	889.387119	869.2215021	698.9621657	1746.274904	1257.422653
0.63_505.8999n	486.8820897	0.62565	neg	12012.19621	24664.59112	13588.03692	16380.45511	19956.14866	16900.05811	23320.49607	24765.13934	33448.30295
0.63_506.8749m/z	506.8749293	0.62565	neg	1830.674618	1579.946013	1305.777933	1366.124851	2105.698407	849.6092218	1629.590082	2263.74521	1662.353138
0.63_511.9171n	492.8992988	0.62565	neg	19092.10287	25907.83203	24813.21242	21617.58163	24496.1771	24067.0542	27979.05771	29241.39978	33243.31151
0.63_518.8321m/z	518.8320672	0.62565	neg	3070.923691	5594.86439	2992.011195	2592.208988	2268.258076	3150.328672	4169.206116	3226.373969	3140.274022
0.63_521.8732n	502.8553549	0.62565	neg	19473.3717	24300.85379	17257.2247	20039.04993	22952.89708	22099.94475	22368.07326	22588.10353	26035.01205
0.63_523.9159m/z	523.9158786	0.62565	neg	2065.781568	2350.231437	3004.7167	4699.52259	5757.545134	5543.897665	4507.55182	5820.793187	4174.59671
0.63_525.8633m/z	525.8632829	0.62565	neg	1548.975442	1153.297886	2079.936295	1517.54065	1946.352049	1869.334638	2395.826944	1868.576649	1754.812501
0.63_533.8758m/z	533.8758277	0.62565	neg	859.4752287	2534.638457	1690.437481	2215.000658	1789.322848	1903.870703	3076.131779	3719.866235	3036.057584
0.63_539.8937m/z	539.8936627	0.62565	neg	4395.186824	7171.323902	6167.483362	8732.14766	8703.566144	8002.322977	10594.08595	12579.09124	11959.78416
0.63_540.9128m/z	540.9127729	0.62565	neg	332.8321467	73.64740319	183.5290126	3432.330523	4082.321883	3580.861563	2789.275178	5184.826996	3126.463043
0.63_548.8510m/z	548.8510087	0.62565	neg	4253.046326	7438.524558	5146.374908	5759.348908	4557.376349	5920.742826	7256.821933	6057.80577	8297.24344
0.63_556.8896m/z	556.8895781	0.62565	neg	4661.443149	8020.61539	5504.125885	11595.63564	9548.925685	11910.02635	12899.43315	16221.60302	15970.23789
0.63_557.8126m/z	557.8125975	0.62565	neg	5032.286759	4753.307768	2952.000404	4526.279332	3818.145424	4349.349158	3868.454459	3509.329498	4087.663123
0.63_560.8865m/z	560.8865481	0.62565	neg	10849.77466	11887.6585	13547.84235	11999.08373	12693.40408	11437.00164	16222.69529	14605.47708	15636.63137
0.63_563.9272n	544.9093239	0.62565	neg	9946.562626	8694.549241	15013.17079	13899.42242	18917.97068	13188.63999	17940.3407	15679.34181	17069.82593
0.63_572.8668m/z	572.8667931	0.62565	neg	4766.221218	8456.433238	4618.842842	7959.330163	4093.598817	7602.675423	8949.372652	9867.530926	8679.51279
0.63_584.8850m/z	584.8849634	0.62565	neg	550.9791728	710.9437927	408.2966859	1308.404383	2064.087768	1518.105928	2908.333499	3002.143872	2824.002
0.63_589.8610n	570.8431618	0.62565	neg	13003.32897	19548.22756	14806.46817	12768.20529	23005.63119	18062.38191	16869.05316	19085.13008	22023.51812
0.63_590.8397m/z	590.8396663	0.62565	neg	2149.62831	3481.778858	2982.455873	3513.817469	2879.973585	2840.167536	3148.231462	2628.798959	3939.170419
0.63_591.9036m/z	591.9036107	0.62565	neg	1545.338932	1415.995159	2811.140741	4077.951893	5427.322543	4260.954777	3182.569375	5353.329218	5044.17578
0.63_601.8643m/z	601.8642871	0.62565	neg	785.0029131	2401.57456	1240.538243	1935.888657	2019.89032	2104.454775	2662.790358	2776.246948	2768.010821
0.63_604.8153m/z	604.8153094	0.62565	neg	3884.174787	4626.239985	2356.797617	2831.498064	3002.507317	3645.941305	3761.804199	4274.939428	4786.254234
0.63_607.8813m/z	607.8812937	0.62565	neg	4103.161956	5591.303143	5628.357314	7155.787267	8433.61431	6998.756879	6588.907968	8707.188001	8963.090506
0.63_612.8969m/z	612.8969228	0.62565	neg	4395.061102	3612.604009	6398.769493	5379.607096	6675.443211	4503.957279	8026.134291	4029.246588	5577.966911
0.63_616.8404m/z	616.8404396	0.62565	neg	2102.438243	6115.376939	2542.742375	3727.890678	2857.787558	4010.30262	7510.212113	5236.274953	7112.047353
0.63_624.8752m/z	624.8751664	0.62565	neg	3835.139054	5431.964709	5140.951969	8850.499292	7473.382729	8376.583281	6911.51814	11984.54027	9058.026447
0.63_628.8742m/z	628.8741711	0.62565	neg	4125.870603	6955.948776	6019.705711	3992.218203	5468.64424	3985.174327	7295.24577	4072.555957	7291.041021
0.63_639.8380n	638.8306842	0.62565	neg	11231.22475	16902.99693	12329.17433	11162.47833	16891.32703	14375.78988	15512.65938	14247.17133	20665.67095
0.63_640.8537m/z	640.8537369	0.62565	neg	1593.060016	2672.976028	2257.981788	2289.489644	2767.716404	2789.789877	2662.517308	3999.594971	5230.927527
0.63_652.8731m/z	652.8731262	0.62565	neg	1461.30478	2643.167836	2040.994026	4412.457178	3610.526276	3596.814345	4077.35606	6711.309412	4886.690068
0.63_656.8192m/z	656.8191925	0.62565	neg	7772.489909	8143.269846	6581.089012	5782.310577	7666.007479	6623.439664	7928.090705	6199.322411	8618.800474
0.63_658.8273m/z	658.8273388	0.62565	neg	1637.861556	2176.464701	1474.496611	1974.001664	2172.361993	2715.136161	2413.958267	2369.086569	2452.504735
0.63_659.8912m/z	659.8911629	0.62565	neg	0.001403199	882.4653756	1022.164932	2275.509897	2384.482417	1871.177129	1844.133206	1899.888251	2195.496698
0.63_672.8004m/z	672.8004048	0.62565	neg	1993.1399	3030.023266	2463.754874	1950.18258	2732.353129	2436.454409	2911.969616	2262.285668	3278.461089
0.63_675.8689m/z	675.8688862	0.62565	neg	1249.182924	1207.395682	2763.317283	1145.96317	3673.215311	3027.051227	3273.650619	2874.179764	3487.102963
0.63_680.8845m/z	680.8844786	0.62565	neg	1474.202495	1442.694088	2279.345279	1350.906754	901.9296165	738.5053576	1783.229326	1520.584244	513.9387016
0.63_686.8478m/z	686.8478296	0.62565	neg	344.6364001	1270.290841	1353.113446	1002.139737	771.1908482	1365.670992	1749.916862	2169.720887	2093.933956
0.63_692.8624m/z	692.8624056	0.62565	neg	738.3934884	1126.012937	1414.175888	2489.453681	2193.202889	3171.464661	3152.456491	3071.780007	3247.866216
0.63_702.8222m/z	702.8222282	0.62565	neg	698.0549785	673.5885396	702.7144322	901.6544354	956.1319824	905.2262074	1639.151687	1719.250031	1401.97772
0.63_720.8608m/z	720.8607526	0.62565	neg	1053.630898	2501.674797	2776.546668	4283.759862	3041.947787	2242.877958	2858.649895	4024.420123	4253.438993
0.63_743.8553m/z	743.8553319	0.62565	neg	354.0863596	0.001403199	657.3137693	843.0516624	1421.280374	547.0139901	777.7131588	564.5496826	1073.371437
0.63_752.8130m/z	752.812952	0.62565	neg	1825.061625	3346.988699	2406.420694	1129.014579	3237.995769	3225.338076	3192.611208	4342.976807	4795.825418
0.63_768.7888m/z	768.788785	0.62565	neg	1346.86962	3800.032215	1783.780226	1773.235138	1990.186446	1248.245476	2912.166957	2178.340302	3488.300278
0.63_770.8093m/z	770.8093334	0.62565	neg	755.5782813	843.5767336	501.257191	1765.038609	939.2598502	598.1902459	1730.174927	426.2332679	2256.440057
0.65_124.9537n	123.9464713	0.647833333	neg	3070.552891	3123.46923	2519.413511	2786.56405	2524.99059	2780.353259	3110.792592	2599.524094	2434.45908
0.65_125.8732m/z	125.8732484	0.647833333	neg	730.4451295	1013.873853	939.8794229	1033.93409	1029.966464	959.1297865	1166.491732	1167.878443	1064.00947
0.65_126.8811m/z	126.8810919	0.647833333	neg	2372.20442	2270.895972	2052.06559	2479.591956	2033.803296	2556.005522	2399.031635	2430.710092	2113.315126
0.65_128.8782m/z	128.8781724	0.647833333	neg	2146.752842	2506.318587	2586.434942	2106.516986	2086.143298	2194.351628	2732.08388	1983.507635	1956.164097
0.65_130.8752m/z	130.8752094	0.647833333	neg	198.1597435	163.3065472	157.3871309	234.8489117	73.11865906	142.6344899	240.1067231	175.3471045	76.8552792
0.65_130.9662m/z	130.9662082	0.647833333	neg	166.8366913	248.1641015	132.3733226	103.9215091	40.46902341	106.6218189	189.2112533	167.9831742	64.92216344
0.65_134.9881m/z	134.9880801	0.647833333	neg	374.0638007	405.1962666	358.8447542	460.6535097	246.7707612	515.7176263	393.6957237	374.213741	372.8361627
0.65_135.9020m/z	135.9020404	0.647833333	neg	280.3013223	373.1371036	300.9040786	342.4450463	220.1526526	360.8970147	357.6000047	445.6967465	316.5538717
0.65_136.9099m/z	136.9098508	0.647833333	neg	3568.206449	3522.776643	3426.755059	2701.497578	2828.9352	3087.71584	3482.418972	2561.875071	2653.196207
0.65_136.9325m/z	136.9324986	0.647833333	neg	177.8767652	308.2733797	139.7854865	137.3395582	49.58497623	116.4295409	178.6008714	143.8514603	84.19430084
0.65_136.9837m/z	136.9836659	0.647833333	neg	485.7396248	336.2566456	346.5838451	429.5455145	126.990901	391.1068568	393.4404758	398.3270522	209.2781572
0.65_138.9070m/z	138.906951	0.647833333	neg	1130.313249	1119.540479	863.7497439	895.4625376	671.3600296	902.7180291	1143.423691	924.103575	740.0605403
0.65_141.9126m/z	141.9125986	0.647833333	neg	408.4111717	411.8330068	379.5110221	522.3837527	311.6412208	439.2058566	444.457855	501.2608526	325.9834471
0.65_146.9387m/z	146.9387262	0.647833333	neg	23442.21137	19721.33416	19903.48184	18461.58444	19479.03029	21394.47599	19904.57965	16822.32961	18385.50275
0.65_150.9829m/z	150.9829226	0.647833333	neg	5103.275801	4504.473382	4769.437964	5702.592261	4888.6845	6111.393787	5407.024869	4947.393286	5689.42663
0.65_152.9787m/z	152.9786501	0.647833333	neg	28288.00634	19279.7903	22417.77192	25911.04323	18091.97125	30851.7346	24549.38316	23129.2809	24696.47116
0.65_156.9630m/z	156.9630189	0.647833333	neg	281.706063	277.8215597	312.3147188	409.6474471	232.9685402	312.5361861	439.036378	362.9334984	213.2993868
0.65_157.8631m/z	157.8630658	0.647833333	neg	511.6342093	666.7901721	740.6467829	832.1881403	497.5507853	761.9755075	822.0099823	982.805689	675.7982262
0.65_160.8421m/z	160.842122	0.647833333	neg	3295.989598	3944.771981	4600.993667	2891.599791	3164.348465	2795.649958	4382.565103	3051.290844	2991.304114
0.65_160.8860n	141.8681894	0.647833333	neg	517.1601791	537.4430069	477.7779817	691.9270911	333.3561587	628.0096605	559.12381	600.3945428	381.2447164
0.65_162.8392m/z	162.8392249	0.647833333	neg	3304.171489	3849.409994	4573.631197	2804.793454	3241.273753	2870.914181	4281.604483	2939.602442	3019.66629
0.65_164.8362m/z	164.8362053	0.647833333	neg	601.8216157	646.4077656	594.2951278	700.7336661	380.2132949	585.936099	729.1248208	640.8342718	410.6131425
0.65_166.9245m/z	166.9244635	0.647833333	neg	842.5425115	1177.728251	852.5906078	737.1180619	651.6370803	798.2048624	1196.62769	897.7700448	828.4497051
0.65_170.8710m/z	170.870989	0.647833333	neg	3724.466977	3597.168877	3190.673356	4423.009825	3210.328633	3748.385907	3887.699957	3688.285494	3146.004564
0.65_174.8651m/z	174.8651223	0.647833333	neg	320.066563	262.1368026	220.1008409	330.5241051	123.4924182	260.3485357	293.186905	274.2418365	106.8989884
0.65_178.9492m/z	178.9492	0.647833333	neg	1171.233534	1638.081871	1302.549775	1186.429814	853.0009847	1328.063946	1629.189307	1622.652397	1361.440435
0.65_187.8612m/z	187.8611794	0.647833333	neg	1702.614653	1585.223613	1799.543167	1478.833176	1659.919866	1855.767857	1752.876112	1745.244187	1770.066045
0.65_187.8738m/z	187.8737548	0.647833333	neg	1780.894238	2108.32535	2382.449822	2329.000063	2427.142769	2292.721488	2534.243111	2873.224123	2852.558407
0.65_189.8583m/z	189.8583091	0.647833333	neg	1017.372016	1005.64775	1186.026864	977.5900503	1144.690691	1050.701068	1067.489059	1094.977606	1063.191761
0.65_191.9464m/z	191.9464286	0.647833333	neg	2806.110763	3860.932087	3154.887878	2591.571187	1899.275913	2788.514108	3628.959677	3066.798624	2770.058887
0.65_197.8900m/z	197.8900003	0.647833333	neg	3403.729196	2981.463664	3160.096285	3389.285368	3027.390984	3395.822138	3144.88386	3126.28249	3076.750237
0.65_197.9454n	394.8909426	0.647833333	neg	231555.6254	203495.9803	202864.372	167658.7464	175037.6318	182715.4319	210142.1087	170020.8674	183532.186
0.65_197.9957n	178.9779026	0.647833333	neg	34780.16544	32476.55931	33807.95715	39265.4065	34079.72622	42231.17162	38771.40527	34794.22287	38600.5136
0.65_199.8871m/z	199.8871077	0.647833333	neg	1020.267071	792.9548312	656.6887779	955.8930737	766.9018581	854.2696682	785.480062	864.2120686	853.8069464
0.65_205.8398m/z	205.8398429	0.647833333	neg	752.9108892	818.2885394	1159.561107	957.9129044	601.3581603	1069.506074	1174.344521	1329.391553	793.8266001
0.65_206.9729m/z	206.9728537	0.647833333	neg	7817.094614	7811.242658	7431.464897	8267.390756	7640.685704	8999.821623	8618.320552	7262.20992	8578.420757
0.65_207.9188m/z	207.9188255	0.647833333	neg	6530.945674	5431.42785	5367.354143	5305.657751	5197.014843	5821.946899	5315.69643	5092.624539	5019.902917
0.65_218.9346m/z	218.9345851	0.647833333	neg	1035.734031	927.3510696	979.3260036	1026.713435	675.6821055	959.4180305	1169.193193	963.9375518	654.2787537
0.65_224.9089m/z	224.9089195	0.647833333	neg	589.0808962	556.7861297	438.564439	508.9225181	281.0862084	610.677666	676.6491224	417.1879496	419.2964594
0.65_227.8947m/z	227.8947042	0.647833333	neg	1538.377609	1533.925461	1754.39943	1868.064244	1604.900177	1758.051477	1904.97713	2094.280624	1863.134332
0.65_232.9150m/z	232.9150386	0.647833333	neg	836.1438924	1140.570491	932.2556612	663.505423	692.3939472	754.516885	1178.337209	762.8045039	1024.024385
0.65_246.8865m/z	246.8865371	0.647833333	neg	693.2956309	1189.766946	762.2177669	831.4590821	683.4627229	805.4969671	1223.217084	1087.581087	992.4067178
0.65_254.9441n	508.8727052	0.647833333	neg	37241.61578	35926.57855	35786.94107	31128.0861	35960.56875	33910.94004	37917.93395	33989.78205	38228.29139
0.65_259.9338m/z	259.9338068	0.647833333	neg	1027.410172	1496.706268	1041.525969	839.7920787	641.4225891	916.6225185	1209.40255	949.7672281	691.506793
0.65_263.8889m/z	263.8889164	0.647833333	neg	432.1849675	385.4959961	549.5587685	435.6635075	423.6306746	334.8186323	358.4184831	374.0253503	687.5314825
0.65_286.9218m/z	286.9218017	0.647833333	neg	1242.025255	1414.53872	1386.872913	1384.487209	1586.407854	1219.083814	1870.290806	1783.243198	1496.498295
0.65_298.8353m/z	298.8353016	0.647833333	neg	4983.765563	3618.690008	2908.800695	3448.19972	3081.891028	3187.97576	3121.435123	3197.213007	2805.149377
0.65_302.8999m/z	302.8998578	0.647833333	neg	138.6235212	709.6047628	313.2227767	192.9054718	234.1097357	154.2290271	364.346446	152.561758	233.6407953
0.65_306.8689m/z	306.8689312	0.647833333	neg	2146.580834	1649.841121	1531.568286	1649.624356	1411.309875	1783.913587	1415.9102	1241.497604	1417.328749
0.65_308.8642m/z	308.8642262	0.647833333	neg	18093.82947	13609.27279	12105.67701	13621.26921	12283.79151	16072.19943	12229.13679	11032.81288	10802.55435
0.65_310.8577m/z	310.8576964	0.647833333	neg	2178.060987	2559.649762	1508.726467	2007.334648	706.4560882	1454.003512	1984.634729	1479.421198	662.8978324
0.65_311.8704m/z	311.8704147	0.647833333	neg	779.5339016	575.8268463	757.3590015	915.6313822	764.2472908	909.3106004	1118.274009	912.6143181	574.9101751
0.65_325.8544m/z	325.8543825	0.647833333	neg	1133.442631	896.8872845	649.4427719	748.3347293	528.7563161	737.1583153	803.7527403	516.1154785	237.8554083
0.65_326.8302m/z	326.8301933	0.647833333	neg	2771.105183	2178.663467	1781.160409	2111.050438	2038.787513	2202.657765	1858.235486	1847.558946	1307.913573
0.65_327.9213m/z	327.9212516	0.647833333	neg	675.9977029	1114.011845	473.3054012	313.6249594	331.6075705	633.2245313	1037.848987	633.788214	470.1843601
0.65_336.8590m/z	336.859035	0.647833333	neg	12613.27082	9937.069849	8668.510384	10275.08759	8580.899849	12446.53663	9501.35502	7019.884853	6848.435901
0.65_353.9196n	706.8181985	0.647833333	neg	9456.550485	11179.73215	9478.472075	7033.630438	13277.28312	9488.644809	12344.09042	7991.807233	12162.73672
0.65_354.9092m/z	354.9091575	0.647833333	neg	1345.726765	1903.607338	1779.798708	1638.063393	1777.872364	1538.835413	2404.39119	1663.186151	1663.910663
0.65_359.8715m/z	359.8715293	0.647833333	neg	2052.181359	2575.817976	2281.631614	1376.313581	1043.875661	1488.467275	2144.442719	1885.814777	1746.169199

0.65_362.8687m/z	362.8686608	0.647833333	neg	1361.94505	1502.638904	2328.776467	1834.125631	1727.323615	1363.185175	1252.047573	861.0228221	1343.476761
0.65_362.9413m/z	362.94131	0.647833333	neg	46496.50055	48606.05793	53764.0179	51561.06554	56214.96982	51620.10181	65883.43506	54581.93576	59817.00285
0.65_378.9185m/z	378.9184632	0.647833333	neg	18864.69152	24620.48644	20661.85086	17680.94555	17045.91367	18305.97428	24697.5563	19107.60159	21611.60692
0.65_379.9316m/z	379.9315832	0.647833333	neg	4040.415678	3629.003515	3785.379996	4545.875835	4116.832807	4850.112789	5885.877606	5230.668184	4531.085662
0.65_386.8791m/z	386.8790929	0.647833333	neg	823.6220229	1284.80107	979.1734084	635.9592647	505.1137222	395.9780481	505.5423148	581.304985	319.5594119
0.65_387.8843m/z	387.8842501	0.647833333	neg	1212.852773	1042.521497	1319.957204	1458.157345	1621.30524	1503.415058	1523.455134	1359.191207	1401.810599
0.65_388.8746m/z	388.8745836	0.647833333	neg	14603.82113	16134.35721	12462.54997	11232.93112	11653.26963	12658.26866	14209.60378	13063.02654	13259.0501
0.65_421.8373m/z	421.8373236	0.647833333	neg	1184.485225	1283.61461	956.1481112	941.1898159	482.6363949	962.475036	1342.875307	1125.749542	783.3646539
0.65_427.8585m/z	427.8585468	0.647833333	neg	2515.528417	2945.466459	1808.833553	1497.838656	1622.858153	1807.036458	2396.221322	1756.398322	2375.564914
0.65_430.8548m/z	430.8548002	0.647833333	neg	6130.824879	5969.078547	6148.77104	6175.157492	17431.87403	10917.73064	6306.437413	6277.066126	10263.0849
0.65_450.8439m/z	450.8439433	0.647833333	neg	3387.618098	4645.527904	2487.368934	2801.2825	3224.898818	2890.528717	3936.999295	3446.580967	3861.602979
0.65_454.8670m/z	454.8670178	0.647833333	neg	790.9555708	1315.558062	845.6432388	878.6588835	399.8819374	747.0410343	756.9219105	632.3221261	213.9733404
0.65_456.8617m/z	456.8617165	0.647833333	neg	12321.52259	13658.62093	10412.25179	8530.76112	7731.50541	8844.936094	12447.55874	9429.547867	7833.492631
0.65_467.8242n	512.8136869	0.647833333	neg	4390.903131	3567.786241	1980.678805	2949.764654	1828.938904	2013.551624	2774.319931	2216.947054	1507.337917
0.65_482.8713m/z	482.8712577	0.647833333	neg	2672.402205	2676.354564	3446.209551	2921.584351	2768.685959	3164.343618	3724.851165	3184.648381	3438.725979
0.65_498.8424m/z	498.8423964	0.647833333	neg	3487.319663	3701.720184	2866.065197	2258.593816	2090.688026	2588.443027	2984.395563	1518.5431	2157.536637
0.65_505.8017m/z	505.8016861	0.647833333	neg	1360.830635	1698.011847	1516.512454	1015.411528	858.9261519	817.7560646	1632.324548	917.7870941	541.849376
0.65_514.8202m/z	514.8201828	0.647833333	neg	1070.424532	780.4730034	607.2162518	540.0829598	752.5128779	556.3825427	828.9502671	558.3411771	582.1959491
0.65_524.8489m/z	524.8489386	0.647833333	neg	9819.153137	10012.34682	8011.803058	6343.994998	5432.929125	6152.72277	9333.342136	6326.247304	5681.355013
0.65_534.8049m/z	534.8048808	0.647833333	neg	3023.841495	3282.233972	1375.964752	1864.838436	951.0964203	1661.583964	1755.195058	1742.035339	1077.743385
0.65_555.7972m/z	555.7972268	0.647833333	neg	2537.667893	2640.067492	2531.618191	2463.369285	1698.500182	1874.823362	2079.540604	1840.516128	1784.084249
0.65_560.8142m/z	560.8142274	0.647833333	neg	1738.273988	2033.878167	1287.186645	1142.195934	4052.909757	1654.847073	2095.49379	971.604643	3295.065607
0.65_563.8341m/z	563.8340745	0.647833333	neg	967.1789817	967.2190572	384.6582093	511.74355	0.001403199	337.7204827	896.6686334	552.5778342	193.3702885
0.65_574.7971n	573.7898096	0.647833333	neg	3999.664704	5087.334466	3131.870693	3110.489874	2424.010592	3841.718922	4201.503254	2602.784057	3564.24821
0.65_576.8607m/z	576.8607138	0.647833333	neg	11666.9734	12975.84422	11041.05737	10415.61089	11301.49819	9431.307331	14101.41493	11026.06259	10408.12692
0.65_580.8039m/z	580.8038885	0.647833333	neg	1202.675275	742.2544205	286.2672755	497.3091974	429.9039407	623.3784811	0.001403199	736.1522675	142.6556099
0.65_587.8262n	586.8189401	0.647833333	neg	5695.20553	12138.60162	6534.737578	5998.061281	8204.507935	7882.178743	7964.188879	7099.23687	7195.130991
0.65_592.8364m/z	592.8363594	0.647833333	neg	6650.569504	7765.566264	5235.767116	4651.36275	5153.918729	4406.488764	6812.89391	4854.773346	5582.968184
0.65_628.8028m/z	628.8028025	0.647833333	neg	2079.380229	1364.488983	2594.775689	999.846495	3728.975692	2763.908667	2442.718927	2514.765865	3766.965974
0.65_644.8481m/z	644.8480877	0.647833333	neg	7582.660612	8496.649521	7323.252027	6845.231479	6665.741928	5753.567431	8705.808316	6702.110015	5987.11976
0.65_655.8132n	654.8059392	0.647833333	neg	6827.894579	10999.17282	6941.083355	6168.054784	11848.99716	8413.990301	8050.16536	7536.068172	9383.931719
0.65_660.8239m/z	660.8238885	0.647833333	neg	4762.111202	5363.09091	4358.452448	3962.941113	4180.703617	2834.608112	4854.954182	3653.754377	3795.164104
0.65_696.8616m/z	696.861647	0.647833333	neg	1015.826498	2575.255285	2212.970937	1146.021632	2583.871086	1176.307752	3224.233809	2061.480484	2247.145536
0.65_712.8347m/z	712.8347227	0.647833333	neg	4372.13751	3391.369772	3935.470387	3457.740643	4050.692442	4259.972419	3873.34144	4175.16153	2716.064863
0.65_722.7931m/z	722.7931081	0.647833333	neg	2793.298842	3201.960768	2136.031636	2375.811405	3220.401796	2614.763357	3114.518415	2122.395716	2293.834821
0.67_108.9887m/z	108.9887034	0.6701	neg	213.1362212	126.8868346	149.6270574	189.1179336	80.44591861	166.63872	171.3950047	145.9754024	74.78637815
0.67_149.9917n	194.9900143	0.6701	neg	2490.896696	2068.442804	2090.456064	2646.077479	1748.144928	2889.619743	2346.752592	1779.734939	2095.809335
0.67_321.9745m/z	321.9745433	0.6701	neg	1185.262102	1256.720432	1070.433446	1292.323473	2303.056084	1405.684722	2018.297949	1537.426101	1988.173267
0.67_336.0305m/z	336.0305116	0.6701	neg	378.9427191	385.8370482	484.5246785	711.0330659	797.7319836	445.5501377	1147.466672	528.7686615	1011.607943
0.67_368.8892m/z	368.8891534	0.6701	neg	1028.780514	1415.445218	1038.212258	788.7578792	863.6854697	482.8880837	1134.63992	535.2595604	749.05869
0.67_389.9624m/z	389.9623524	0.6701	neg	3091.47369	2915.08405	2920.518397	3975.851685	6262.00786	4914.406572	4112.649874	3631.051468	6501.861878
0.67_391.1338m/z	391.1338117	0.6701	neg	1270.656982	1489.732431	1305.150622	2013.476744	1509.692214	1515.613707	1303.121812	1035.017706	1060.226329
0.67_404.0180m/z	404.018019	0.6701	neg	258.4161376	304.0614285	331.466458	663.9123833	805.610981	351.110795	618.3055182	735.0531006	873.2009938
0.67_430.9285m/z	430.9285291	0.6701	neg	15540.59473	15812.14526	18871.69778	17088.48854	17920.20602	18636.44886	22340.9444	19306.88943	18946.99618
0.67_446.9059m/z	446.9058538	0.6701	neg	12527.7801	14211.84464	12663.54979	9880.831788	10022.02287	10242.41379	15239.46718	11608.14696	13320.99586
0.67_453.8479m/z	453.8479247	0.6701	neg	330.6030715	0.001403199	463.7637645	345.9538786	326.0671869	982.0456871	350.7231077	368.6728821	207.9975731
0.67_459.1211m/z	459.121124	0.6701	neg	961.0700804	552.8089237	1141.218718	760.0848717	1024.212004	1101.303849	702.3442357	673.6317139	744.2824646
0.67_467.0884m/z	467.0884046	0.6701	neg	651.6007862	871.2831016	1559.367809	1286.418992	2038.147823	1856.188822	1803.417894	2315.292362	2547.191385
0.67_472.0047m/z	472.0047116	0.6701	neg	258.2178408	436.3187987	775.4391054	1526.551269	1401.464803	1633.916837	1218.112861	1413.064082	1913.596199
0.67_485.0357m/z	485.0356578	0.6701	neg	1809.120453	1824.342098	2381.961641	4902.026539	6012.340435	7000.159912	3577.310308	4532.441879	5187.239308
0.67_498.9163m/z	498.9162526	0.6701	neg	10959.17196	10455.3854	11969.00433	11679.11467	11832.45969	11615.48204	13782.26421	12998.58112	12861.617
0.67_514.8930m/z	514.8930049	0.6701	neg	10710.71818	13339.03324	12256.57057	9170.352378	8177.988038	9531.079255	11766.72608	11454.8847	11387.45852
0.67_525.9369m/z	525.9369076	0.6701	neg	2295.359555	1819.986113	3901.882726	2226.39531	8005.11892	5719.906073	3369.941249	4533.723257	6569.658275
0.67_535.0763m/z	535.0763469	0.6701	neg	764.4268383	1038.454834	1282.626893	1759.071518	2591.937812	1559.13067	2032.690281	3049.671894	3220.956912
0.67_539.9925m/z	539.9924555	0.6701	neg	501.6087715	321.6061104	487.9342221	1426.071862	896.6058461	928.9944354	837.0709521	774.4543228	1726.09751
0.67_553.1870m/z	553.1870188	0.6701	neg	3904.630792	3777.991207	4316.945017	4215.363161	4011.03723	4568.371885	3416.606525	3841.201097	3970.505138
0.67_566.9036m/z	566.9035542	0.6701	neg	6342.362683	6478.942824	8401.499599	6699.980693	6793.141544	6769.647361	10033.00624	9346.753235	9751.031936
0.67_594.0999m/z	594.0998642	0.6701	neg	194.2555639	188.7269477	538.0800857	1057.186607	928.6972742	553.548517	875.4153498	795.0493774	1459.69158
0.67_634.8909m/z	634.8908772	0.6701	neg	4579.156175	4771.114151	5321.818424	4716.762679	3513.827193	5329.952812	7122.268443	6248.407766	5078.046629
0.67_662.0877m/z	662.0876891	0.6701	neg	0.001403199	0.001403199	727.5094092	1031.405757	773.3826255	1086.750204	430.1016702	831.8375624	1325.845454
0.67_675.9670m/z	675.9670434	0.6701	neg	0.001403199	197.6581176	398.7909484	301.461057	890.1464833	688.4554425	391.7592528	823.7841949	953.1260729
0.67_689.1619m/z	689.161887	0.6701	neg	756.5443993	685.407313	1426.150151	1033.916289	1113.854404	961.0129319	1537.761794	1250.799171	833.9179145
0.67_691.9443m/z	691.9443165	0.6701	neg	0.001403199	452.1606515	883.8595499	716.6561551	1126.726964	825.2918544	1106.970641	903.1574402	1270.008593
0.67_702.8786m/z	702.8785621	0.6701	neg	1508.623458	1914.480426	1953.94	1150.411231	1392.071398	1261.838636	1839.477405	3237.303146	3424.029802
0.67_730.0747m/z	730.0747137	0.6701	neg	0.001403199	0.001403199	591.7935175	1027.81185	1653.22269	1299.048023	0.001403199	1101.175598	999.6167771
0.67_739.0391m/z	739.0391378	0.6701	neg	932.4388216	774.7946395	563.3131979	1751.323875	1038.760212	1008.391433	1497.910561	3274.597397	817.2873336
0.69_1000.3378n	981.3199738	0.692166667	neg	8831.086775	9502.392865	8130.89343	0.001403199	7266.619352	7220.373162	15614.19794	5644.123413	11616.1592
0.69_1001.8580m/z	1001.85797	0.692166667	neg	4995.249546	6214.116926	5556.734077	5227.220352	4553.541502	5879.443552	8026.058147	5954.260437	5072.163286
0.69_1081.2841m/z	1081.284096	0.692166667	neg	0.001403199	4747.266322	1444.704386	0.001403199	0.001403199	0.001403199	0.001403199	2599.439331	1654.970272
0.69_1117.2937m/z	1117.29367	0.692166667	neg	0.001403199	2800.844374	2877.658058	0.001403199	4090.290684	0.001403199	5692.680111	5041.759188	3530.876052
0.69_112.9825m/z	112.9825187	0.692166667	neg	58.55830243	77.80220148	2.473267927	58.91986647	108.3006427	112.2210326	51.31897319	20.03140891	44.42252121
0.69_126.9793m/z	126.9792686	0.692166667	neg	448.1288858	172.965435	342.5104664	411.0918188	124.3260544	510.5953812	509.1108029	341.5322319	310.9273663
0.69_155.0656m/z	155.0656354	0.692166667	neg	2759.523154	2710.232946	2985.670764	4298.208611	4584.380304	4125.870512	4222.813439	4531.898064	4246.204997
0.69_171.0201m/z	171.0200867	0.692166667	neg	955.2761241	698.8172076	832.8771236	832.2573112	839.5837292	853.4916583	1107.751619	580.2879982	797.1780857
0.69_177.0514n	222.0497102	0.692166667	neg	44476.11994	43878.75847	50240.76745	68386.93531	68926.06246	66931.82168	67658.4859	67467.37227	75137.43544
0.69_209.9976m/z	209.9975668	0.692166667	neg	326.9190085	499.3290833	408.9872555	478.375109	580.3945014	270.1180707	802.3065894	355.7537825	391.5363931
0.69_213.0858m/z	213.0858022	0.692166667	neg	1360.970195	1248.695272	1398.177202	5082.842894	4803.447153	4983.959401	3077.286897	3165.971105	3459.966804
0.69_245.0388n	290.0372483	0.692166667	neg	24462.08065	24569.06539	26318.19193	32241.3318	34009.80123	33373.87086	35589.95951	34053.36261	38949.51363
0.69_253.9870m/z	253.9870449	0.692166667	neg	3599.288765	3382.178099	3337.738218	3963.881631	4619.02778	3743.243624	4808.590914	3541.516714	3863.557842
0.69_313.0265n	358.024717	0.692166667	neg	42360.35379	38783.88022	45108.71304	49368.03895	51138.41928	52018.8336	53807.4277	56804.99953	59007.05142
0.69_331.1138m/z	331.1137741	0.692166667	neg	1521.136433	1635.262636	2727.469632	4852.68256	6080.610464	4801.832094	4186.738304	4912.596333	4917.67038
0.69_381.0138n	426.0122231	0.692166667	neg	49454.84492	46392.20192	51188.72297	50818.04757	49657.20002	54587.13148	62535.93844	61910.23799	64596.34776
0.69_392.8960m/z	392.8959922	0.692166667	neg	6745.303611	4579.444054	4930.242803	3764.036673	3850.335211	4147.503661	5377.060907	4042.293732	4237.387398
0.69_439.8887m/z	439.8887231	0.692166667	neg	3463.750224	3507.617986	3486.395744	2980.061533	2506.197739	2992.621631	3668.959181	3502.696305	2823.396249
0.69_449.0013n	493.999736	0.692166667	neg	50325.29836	46258.29308	48410.71274	43310.01187	43029.98006	44254.32279	57858.25116	58265.27997	59004.2458
0.69_457.9492m/z	457.9492201	0.692166667	neg	3204.685845	2869.406199	4391.383235	3985.280533	5751.394697	4083.748954	5171.354999	3456.887284	5457.64351
0.69_516.9884n	561.9871931	0.692166667	neg	46109.19781	43257.24375	44013.81517	40193.16667	35534.76471	40170.31315	61217.56348	55702.72139	57043.16255
0.69_527.1086m/z	527.1086101	0.692166667	neg	574.7903777	591.3573118	627.6821988	683.0026866	614.5955546	744.2072591	277.2932017	468.1742249	552.6157997
0.69_582.8800m/z	582.8800266	0.692166667	neg	7065.560673	8453.900859	8652.715041	5147.125389	4948.783216	7048.692062	8457.558034	8167.080776	8647.535012
0.69_584.9761n	629.9747345	0.692166667	neg	45005.92654	37401.18413	38013.84068	31548.01278	35085.53463	37052.45804	54025.90317	54383.71611	50518.09761
0.69_598.8536m/z	598.8536417	0.692166667	neg	51079.31848	43808.64881	47023.80754	35515.07429	35947.69828	39091.68548	44500.86369	38765.99554	41239.59186
0.69_603.0640m/z	603.0639713	0.692166667	neg	873.7305388	1020.922673	1291.566018	1960.391173	1363.393231	1817.912515	2165.697137	3465.841315	2853.382147
0.69_621.0105m/z	621.0104671	0.692166667	neg	1490.592	1849.046554	3365.7801	5903.982086	4513.393306	5903.695714	6113.949301	6572.997647	6454.493723
0.69_636.9845m/z	636.9844838	0.692166667	neg	1847.709954	539.9675622	1011.123209	4277.569652	2610.027517	5060.501732	3553.508074	3322.312017	2259.339988
0.69_650.8678m/z	650.8677835	0.692166667	neg	5027.548878	5598.707596	5685.331323	5094.463011	4109.886368	4726.455574	6608.371274	6630.074409	5938.815889
0.69_652.9632n	697.9619588	0.692166667	neg	37448.62395	33661.09028	36219.55216	27954.70478	28036.10035	30559.30551	43655.85923	43983.48829	42059.08017
0.69_662.8338m/z	662.8337643	0.692166667	neg	11158.5544	12688.12361	10903.1006	15183.80244	8114.881047	9522.478019	13795.1375	16753.76767	14488.56126
0.69_666.8413m/z	666.8412896	0.692166667	neg	40233.96363	34868.93587	37414.04751	27465.53745	27197.12108	29797.54781	37082.92025	30488.2583	35482.2268
0.69_668.9377n	713.9359953	0.692166667	neg	42942.05631	39117.36217	35602.5056	35449.23556	35992.95572	38349.29757	53096.57654	41609.25367	45955.90889
0.69_671.0516m/z	671.0515531	0.692166667	neg	1076.4822	1501.293924	1109.79843	1818.095411	1072.465971	2005.993193	1615.795143	2268.429291	1646.357834
0.69_688.9980m/z	688.99802	0.692166667	neg	2181.397588	1838.611952	3260.085229	5768.797678	3611.272423	6093.035108	3571.632792	6823.221459	5201.825321
0.69_691.3811m/z	691.3810664	0.692166667	neg	2804.557666	1275.483735	1324.365446	0.001403199	0.001403199	0.001403199	1423.933181	1550.20745	1029.754266
0.69_693.3974m/z	693.3973841	0.692166667	neg	1191.224154	1613.759178	3310.868118	2053.657994	2574.917261	2810.864431	3044.41013	2171.182785	1505.688089
0.69_720.9513n	765.9494086	0.692166667	neg	23703.88307	22221.95376	21837.16109	18939.14108	16591.59646	22052.15699	30714.30878	30150.98098	27851.88618
0.69_734.8285m/z	734.8285277	0.692166667	neg	29649.88937	28064.32	29403.04966	19533.35005	18558.95749	18825.82696	26860.57373	25767.40186	23862.43445
0.69_736.9236n	781.9230423	0.692166667	neg	30097.30531	25357.60989	25769.59196	27190.00274	26400.70279	27029.35945	39534.51275	30431.33543	34412.37312
0.69_756.9855m/z	756.9854504	0.692166667	neg	1621.777002	1390.131279	2197.012143	3303.648904	2752.4663	2706.417413	3185.050232	2191.064819	3976.174798
0.69_759.3682m/z	759.3682187	0.692166667	neg	1338.948968	2320.341217	3205.307318	0.001403199	112.8256371	66.40624254	2205.801007	1046.347149	3713.282994
0.69_772.9586m/z	772.9586033	0.692166667	neg	1388.616093	1045.727342	1690.810476	4262.735085	3643.141143	3579.866651	4226.654554	3361.852417	1739.190138
0.69_786.8422m/z	786.8422167	0.692166667	neg	1661.077396	2689.604143	3203.019526	2128.703972	1280.626611	1097.67523	2848.704861	3282.476306	1737.47001

0.69_802.8154m/z	802.8154186	0.692166667	neg	11896.77747	11284.39377	10477.41131	6487.1767	4722.595205	6115.604585	7733.672899	10640.04597	10779.03083
0.69_805.8253m/z	805.8252857	0.692166667	neg	4449.203288	5730.592479	3379.996127	4040.113564	876.5534664	1005.297886	4592.254346	2856.632111	2591.500774
0.69_813.8688m/z	813.8687914	0.692166667	neg	3755.077726	3058.576639	4911.062903	6387.637508	8534.208106	7747.71276	4565.947784	4502.084778	4412.875319
0.69_821.8865m/z	821.8865486	0.692166667	neg	4351.394448	1337.625687	1530.639326	0.001403199	0.001403199	0.001403199	3686.905559	1192.49448	1441.709919
0.69_833.9372m/z	833.9371899	0.692166667	neg	13354.33112	10898.40999	13396.68726	8069.978375	6798.147224	7640.902872	18836.21268	17637.30481	14082.44202
0.69_849.9103m/z	849.9103346	0.692166667	neg	20534.77367	19580.84234	18535.1	14894.1858	14191.05761	17253.34826	22501.85606	20891.20484	20206.40092
0.69_870.8029m/z	870.8029499	0.692166667	neg	9246.74742	5934.406629	6451.888839	4820.972395	68.74015158	820.3363519	3525.326284	2616.812155	2068.311398
0.69_881.8566m/z	881.8565869	0.692166667	neg	3651.885929	0.001403199	2694.083712	5602.600484	6947.485341	5390.217237	1997.844902	1944.329687	4284.122871
0.69_889.3718m/z	889.3718361	0.692166667	neg	5502.295879	3424.265796	3676.445139	0.001403199	976.7824744	1640.870531	5941.862124	4352.453094	3649.505683
0.69_897.8604m/z	897.8604005	0.692166667	neg	422.3105278	227.9775209	0.001403199	1351.730355	837.2492714	65.92217351	4182.929835	677.4970329	1815.948498
0.69_901.9243m/z	901.9242638	0.692166667	neg	5430.203833	5925.447836	3833.361504	4916.609896	3137.936277	2816.830795	7849.4741	8097.993062	8501.357561
0.69_965.3457m/z	965.3457315	0.692166667	neg	4949.222064	5043.216366	6073.266749	2283.453903	1970.932194	0.001403199	6883.882441	4421.462372	3272.861839
0.69_965.8452m/z	965.8451977	0.692166667	neg	1740.147715	38.60461539	0.001403199	0.001403199	0.001403199	480.5291512	3211.959776	1495.891541	2474.496406
0.69_969.9110m/z	969.9110192	0.692166667	neg	3735.144692	2504.519527	2601.468082	2351.362484	1022.237086	2708.799017	2505.353007	3915.192071	2870.707198
0.69_985.8852m/z	985.8852245	0.692166667	neg	7747.11843	7008.786449	5410.05833	5901.358607	4142.486928	7099.328746	8449.353132	7822.968811	4297.970936
0.71_1003.2647m/z	1003.264687	0.714216667	neg	2464.753891	2584.823976	3753.823138	2976.595543	2068.507315	0.001403199	5276.525227	1671.436276	0.001403199
0.71_1011.7476n	992.7297931	0.714216667	neg	17243.3157	13725.18817	22563.60512	23291.08484	44874.07044	24036.5276	23143.41703	18766.42749	22303.0582
0.71_1012.7964m/z	1012.796372	0.714216667	neg	59544.4225	54692.34349	50650.69522	14188.3382	15944.95525	18211.97615	47500.34171	31561.9681	42152.46254
0.71_1014.2955n	995.2776768	0.714216667	neg	10337.84807	13263.75724	19075.09286	5003.102606	1766.58836	4470.555439	14171.96044	4189.844636	7618.980627
0.71_1015.3123m/z	1015.312332	0.714216667	neg	4672.525538	6006.523633	2475.156303	0.001403199	2437.952247	1827.912825	6828.67373	2791.199707	4559.169
0.71_1018.7448m/z	1018.744847	0.714216667	neg	2328.156681	7954.031408	8522.945797	7290.683886	8931.068884	7053.697291	3135.061656	11979.87538	4832.836988
0.71_1021.2847m/z	1021.284748	0.714216667	neg	3295.3606	4377.606612	4179.983995	765.3747282	0.001403199	1430.998929	6686.465663	5531.072754	7727.305753
0.71_1021.7824n	1002.76452	0.714216667	neg	39468.23689	43637.63917	40265.89786	13845.0176	15428.44632	18988.32574	45264.76742	28847.19838	39278.85613
0.71_1047.7882n	1028.770314	0.714216667	neg	77058.66705	80638.62646	91340.76129	34271.75241	42919.73391	39192.72399	81360.63177	63288.7641	66097.74069
0.71_1055.7759n	1054.777447	0.714216667	neg	36577.70576	36036.36976	32722.60119	7313.30874	11667.26649	9396.584942	34090.48547	25706.33814	27239.44075
0.71_1056.2756n	1055.277982	0.714216667	neg	6576.008113	7835.547123	10496.1716	2984.476247	2993.278608	3891.385787	6110.883201	2780.260148	5678.763896
0.71_1063.2648m/z	1063.264799	0.714216667	neg	5270.983526	0.001403199	4201.596452	2822.08499	4039.999668	0.001403199	5782.320313	1467.454773	3480.569565
0.71_1063.7612n	1044.743336	0.714216667	neg	53784.05645	41408.87	64098.21009	24510.88149	34004.96647	25671.68811	65110.39927	46385.60074	55898.85187
0.71_1075.3138m/z	1075.313779	0.714216667	neg	2241.880421	5229.628271	4979.737568	0.001403199	0.001403199	0.001403199	6939.804469	3846.276978	0.001403199
0.71_1079.7355n	1060.717636	0.714216667	neg	18186.15385	7997.507115	24366.49108	13881.4692	32095.82695	21959.81287	10918.4957	13745.17999	14407.11768
0.71_1080.7832m/z	1080.783236	0.714216667	neg	40486.76101	33476.37867	36552.27979	6476.671203	8512.8973	6932.199181	28088.23048	15819.01276	18831.32337
0.71_1086.7300m/z	1086.730032	0.714216667	neg	4138.876744	3821.337955	7046.232316	6231.261781	7013.140775	1243.09236	2450.185419	2914.425659	7999.764554
0.71_1089.7692n	1070.751313	0.714216667	neg	25236.63757	27339.93968	40554.89673	5788.40115	7844.610076	12344.25432	29337.69861	20699.5528	18476.5778
0.71_1115.7753n	1096.757471	0.714216667	neg	56667.93512	59738.38278	61910.67884	17365.33566	23530.61341	22032.94233	52994.04561	34602.47249	44496.69132
0.71_1123.7628n	1104.744912	0.714216667	neg	20575.29099	18820.58656	38144.74728	6498.071156	6850.137691	4729.253716	25799.6042	17603.28595	18924.49094
0.71_1128.7055m/z	1128.705471	0.714216667	neg	4805.094364	5532.72058	5396.051	5696.484226	14108.39594	9252.628835	4598.997395	7041.988281	7669.299083
0.71_1131.7481n	1112.730309	0.714216667	neg	47707.54009	39153.99167	50153.88898	14157.64666	25227.23695	23607.96426	38296.92136	27207.02476	31305.63838
0.71_1132.2480n	1131.252189	0.714216667	neg	3925.004231	2196.830577	7992.066485	1532.578294	3005.226998	2759.084217	3523.088421	2475.046631	3037.359871
0.71_1148.7720m/z	1148.771989	0.714216667	neg	23490.25609	14965.07393	17636.6796	5037.01048	0.001403199	2937.642376	18633.69845	7617.22394	9531.832293
0.71_1157.2576m/z	1157.257592	0.714216667	neg	0.001403199	5319.520928	0.001403199	0.001403199	0.001403199	0.001403199	1630.730397	0.001403199	1592.850595
0.71_1157.7547n	1138.736875	0.714216667	neg	26408.51598	23403.36074	32360.82657	4051.89127	6980.982978	11243.16986	24900.77821	18754.41899	19413.69073
0.71_1164.7448m/z	1164.744777	0.714216667	neg	31592.911	19027.02239	36007.1424	6689.114658	11650.73167	6198.637561	25841.05638	26080.57353	23446.98651
0.71_1191.7484n	1172.730534	0.714216667	neg	25381.62891	22339.39771	35521.15181	2447.319788	2551.571972	3864.308933	27884.40327	13595.20397	8255.80056
0.71_1199.7361n	1180.718285	0.714216667	neg	33332.22106	24048.97821	43940.02075	6040.192248	13449.56481	9365.045329	22787.72261	16491.80202	14489.84335
0.71_128.9596m/z	128.9596146	0.714216667	neg	5270.929351	5499.099297	5858.405217	7581.247587	8309.909368	7426.591302	6055.830848	6395.204361	6386.186981
0.71_130.9577m/z	130.9576619	0.714216667	neg	326.6032027	455.6073389	538.0908591	521.1557279	681.4938854	551.9430535	342.5503384	451.1175182	438.1273283
0.71_180.9732m/z	180.9732194	0.714216667	neg	131650.613	121202.8069	128633.7262	112485.2904	106541.2051	122661.1506	129371.0335	121488.3954	128447.5928
0.71_196.9470m/z	196.9470076	0.714216667	neg	5043.836189	5393.463134	5992.189086	7661.508641	7486.280386	7487.577982	5992.787571	6055.288105	6826.479258
0.71_215.0099m/z	215.0098926	0.714216667	neg	983.1096876	621.9785496	411.3509585	700.3771766	756.6401266	425.0539055	774.1567866	659.5888034	438.6588655
0.71_236.0653m/z	236.0653437	0.714216667	neg	1351.969939	1590.422439	1698.723087	1860.290572	2044.51856	1979.437049	1837.799331	1644.015381	1925.427496
0.71_238.0236m/z	238.0236408	0.714216667	neg	533.0738591	710.9132743	821.5719575	1805.420389	1670.160831	1988.214803	1453.586256	1108.86351	1487.498655
0.71_248.9605m/z	248.9605199	0.714216667	neg	353889.6818	336998.6412	348129.8474	267160.086	263359.6119	271057.3116	352880.2815	325788.3443	337200.2682
0.71_264.9344m/z	264.9344201	0.714216667	neg	57300.74521	54823.51098	61631.12876	64455.76739	69465.34264	65432.87888	59242.23651	57376.87353	59995.38484
0.71_281.9149n	280.9075755	0.714216667	neg	8243.420403	8697.779835	7955.265181	13378.48095	13061.91818	12140.51639	9576.535285	8988.631657	9699.397143
0.71_306.0111m/z	306.0110887	0.714216667	neg	1020.726947	1219.590782	857.3430442	2112.811039	1266.708857	1615.320274	1667.085107	1475.950531	684.214796
0.71_329.0003n	373.9986093	0.714216667	neg	8711.776087	8448.608597	8199.26183	15231.99017	14907.89399	16473.73697	11842.3322	10318.89456	12898.07075
0.71_332.9220m/z	332.9220255	0.714216667	neg	161706.9852	155749.1331	164067.512	155791.4606	172210.6118	160723.3229	161646.8962	158398.8458	163174.366
0.71_348.8959m/z	348.8959459	0.714216667	neg	21858.44375	21999.57618	25079.64481	33865.21336	41653.72264	33924.92513	25056.66579	26308.41349	27942.36249
0.71_350.8936m/z	350.8936047	0.714216667	neg	3323.602892	2900.758524	3076.546747	3662.594967	3159.820546	4242.494538	3028.682079	3366.670962	2326.354009
0.71_355.9112n	400.90938	0.714216667	neg	250118.2789	235299.1776	247240.4032	203363.0855	219937.7704	214719.1394	252559.8653	224162.7553	236166.2677
0.71_384.9355m/z	384.9355472	0.714216667	neg	501682.5627	475599.8624	479048.3476	292171.4018	284982.7405	294875.9643	488885.2292	422273.4651	426344.0779
0.71_396.9876n	441.9861571	0.714216667	neg	17439.99965	16745.54725	17307.81035	27950.03001	24439.53029	29702.88143	23654.88874	22482.82033	23002.96656
0.71_406.8658m/z	406.8658262	0.714216667	neg	2575.311718	9616.079884	10672.51195	29999.71965	12452.53868	14508.16221	6085.149763	22655.98748	11314.34037
0.71_411.9543m/z	411.9543368	0.714216667	neg	443.130069	747.449388	587.0726219	1813.019297	702.6663983	1416.019004	427.5579529	11.8708658	0.001403199
0.71_416.8834m/z	416.8834273	0.714216667	neg	53729.78358	47207.28206	55190.62809	62904.92586	74261.16286	62760.749	53252.12834	52865.08542	55386.36613
0.71_418.8813m/z	418.8812943	0.714216667	neg	5479.276871	5968.488761	7643.988758	9132.713867	7005.15211	8560.665654	7645.72625	6861.66633	5838.186137
0.71_422.8397m/z	422.8396599	0.714216667	neg	420.8763006	1864.795327	3333.217202	10752.80344	5411.470366	5875.844863	1037.658876	6010.85391	3894.471498
0.71_440.0277m/z	440.0276793	0.714216667	neg	2641.559816	2069.862713	2689.52892	2603.944668	927.1848261	1351.181407	2242.782488	2176.751786	1405.507255
0.71_452.9230m/z	452.9230211	0.714216667	neg	545090.3012	505201.2607	505947.7982	279024.6581	279262.4715	286969.2417	523812.4878	443105.9891	450313.8797
0.71_464.9752n	509.9735556	0.714216667	neg	21738.09464	20347.25822	23765.06459	31735.82416	29079.16535	34984.77359	32824.30063	29002.02421	33511.15358
0.71_468.8967m/z	468.8967181	0.714216667	neg	386923.2531	375331.3768	386847.6749	284915.6113	320479.9072	301079.56	392827.8955	348741.4614	367084.7828
0.71_474.8536m/z	474.8536039	0.714216667	neg	2301.67001	9769.904192	12967.45652	30836.06985	12145.60512	15624.50902	7354.359318	24497.45491	13388.92222
0.71_484.8708m/z	484.8708064	0.714216667	neg	111225.9358	101265.0332	116060.1745	115155.1048	146974.6217	122929.377	119359.9172	109492.7933	113992.2643
0.71_486.8689m/z	486.8689448	0.714216667	neg	14187.45406	15411.72789	16041.60087	16255.85484	22650.2902	18851.14883	17008.68467	16785.9292	17882.47082
0.71_501.8522n	500.8449102	0.714216667	neg	32878.03989	29296.17305	34004.71658	44539.45454	49392.82635	43796.39106	34688.96057	31996.6161	30520.15302
0.71_508.0150m/z	508.0149992	0.714216667	neg	3203.123014	2727.269359	2177.081561	2555.34394	1115.696045	2611.869421	2385.037854	2562.716185	1260.528005
0.71_520.9104m/z	520.9103924	0.714216667	neg	483503.4848	448089.6495	442654.6951	227904.5377	217293.8148	230070.2799	443407.6639	381562.3426	371643.3944
0.71_532.9623n	577.9612553	0.714216667	neg	34767.5054	31727.34781	30672.64042	37567.0354	36923.87622	41335.06146	40989.61925	39473.57698	41724.97812
0.71_536.8844m/z	536.8843886	0.714216667	neg	455395.5641	425303.9293	448470.7823	293247.8774	343821.9636	311759.9607	453365.5437	385450.6846	409780.8307
0.71_542.8420m/z	542.8419876	0.714216667	neg	4451.678601	14703.86901	15670.97504	37079.31	13490.83996	16592.16029	8323.937797	26902.95352	15807.81935
0.71_552.8585m/z	552.8584535	0.714216667	neg	147328.8039	146146.621	165738.8433	141799.3516	181312.6068	152590.5672	164864.5955	142745.464	150032.1166
0.71_554.8611m/z	554.8610867	0.714216667	neg	33828.41327	39873.75676	38108.98925	35425.0605	42962.20147	38285.91336	44839.17534	42997.19435	45219.18135
0.71_568.8323m/z	568.8323347	0.714216667	neg	30925.66949	28451.47339	37970.99774	39221.46466	56932.4134	43594.94685	37766.0121	34131.59863	35226.98501
0.71_576.0030m/z	576.0029983	0.714216667	neg	3700.424091	3058.327987	2098.701708	2076.239845	1323.93826	2144.883192	1705.715574	1019.980988	1254.570878
0.71_577.8491n	622.8473368	0.714216667	neg	34876.15455	41598.75626	45116.04975	34955.47636	52192.16781	44161.03575	43266.52971	43164.55104	49047.86805
0.71_588.8980m/z	588.8979795	0.714216667	neg	452727.3312	412575.4697	406481.7585	181889.2387	174551.2128	193406.9137	410644.892	336170.979	332120.293
0.71_593.9353m/z	593.9353344	0.714216667	neg	6174.835775	5884.914162	5148.916906	9861.661738	5059.249155	7045.863125	8571.464584	7006.021393	2579.928211
0.71_594.8509m/z	594.8509308	0.714216667	neg	10761.40688	11997.29814	14104.54403	18707.09486	7833.345734	11144.90914	8769.2459	19993.48142	11973.21171
0.71_600.9502n	645.9486534	0.714216667	neg	37887.2742	37025.42396	33890.27446	41884.25883	39508.15118	41700.74898	51289.60416	45679.6669	45305.88937
0.71_604.8720m/z	604.8719688	0.714216667	neg	467909.3324	442239.0271	448951.7636	273140.0475	316065.7417	286887.2247	459509.0209	381414.2205	401299.296
0.71_610.8297m/z	610.8297399	0.714216667	neg	5601.222937	16013.40067	16523.2162	38104.60486	13029.47333	18791.00597	11527.65193	30486.31276	17915.20475
0.71_630.8791m/z	630.8791316	0.714216667	neg	11101.97986	9127.036376	11972.16405	2464.092379	4125.965953	4412.644037	10413.4433	8600.554867	10708.01487
0.71_631.3806m/z	631.3805901	0.714216667	neg	0.001403199	2041.640981	1112.099739	1028.453297	703.4289922	0.001403199	2296.106643	2031.915011	696.1543536
0.71_638.8182m/z	638.8182429	0.714216667	neg	7920.695514	8520.984159	12700.67119	13351.57989	14809.45107	13424.53389	10526.64104	11220.65376	7478.172492
0.71_639.3676m/z	639.3676099	0.714216667	neg	2363.46462	2173.292019	2089.982231	0.001403199	0.001403199	601.0150028	1513.198722	2200.85271	972.9377741
0.71_639.8197m/z	639.8197144	0.714216667	neg	0.001403199	0.001403199	0.001403199	1217.506564	1166.149204	0.001403199	0.001403199	783.3357228	0.001403199
0.71_639.8635n	620.8456463	0.714216667	neg	193451.6622	185381.5884	200353.0548	162422.6747	204010.0492	177907.1021	199498.7229	193709.4078	189733.8412
0.71_643.9902m/z	643.9902073	0.714216667	neg	4115.988701	3988.589969	2472.092017	1746.294155	2379.867117	2048.491875	3414.227154	3967.51571	1887.655025
0.71_647.8541m/z	647.8540621	0.714216667	neg	1765.388062	860.980989	2983.084604	386.6893434	1508.505057	944.0691724	2131.878024	1841.052352	850.4885833
0.71_653.8005n	652.7931772	0.714216667	neg	12692.0497	12457.19038	11581.66177	22307.06337	35891.40028	19198.90537	12043.68506	10658.85645	16167.31762
0.71_655.8375n	636.8196486	0.714216667	neg	45162.62118	50420.88635	60228.04367	59551.51185	93306.73632	68722.61079	55648.12671	54412.13907	51196.51553
0.71_656.8856m/z	656.8856049	0.714216667	neg	278276.5549	250667.9559	237752.2817	102789.4152	96472.45308	106525.6521	247449.5824	196359.7987	196077.7748
0.71_661.9217m/z	661.9217266	0.714216667	neg	9166.876332	10952.21205	11053.04123	16896.91459	20240.61597	19558.14264	16289.61818	15258.68014	18032.11548
0.71_664.8720m/z	664.8719651	0.714216667	neg	23358.74795	21725.0109	26711.24028	7829.439935	7775.006966	9270.687726	25180.74988	18508.38863	15963.30913
0.71_666.3727n	665.3745135	0.714216667	neg	4641.826867	4041.113636	8703.52725	1448.574466	2265.693695	3383.84653	3268.853304	3004.588325	3000.088055
0.71_684.9111n	729.909091	0.714216667	neg	17340.54006	14765.34832	16058.74049	21834.00963	26075.36511	22642.31399	21586.09787	19122.86979	17876.66681
0.71_685.4109m/z	685.4108747	0.714216667	neg	3093.904476	2697.940765	1657.516112	1018.866032	1677.132029	1592.610235	3757.410027	3374.331261	2969.166377
0.71_691.8775n	672.8596286	0.714216667	neg	385858.7875	352383.1695	366637.4498	201123.2363	227252.69	204088.9704	354152.3899	295850.3789	312832.8129
0.71_694.7896m/z	694.7895968	0.714216667	neg	731.9718187	5553.634812	8538.471589	23503.09543	9897.426305	9822.054812	4229.838193	17391.93311	8758.930644
0.71_697.8336n	678.8157364	0.714216667	neg	7445.451137	14697.73733	16831.84152	30845.92064	21143.85704	23808.02343	12161.84947	27689.03013	21057.53239

0.71_699.8637n	698.8663198	0.714216667	neg	54729.2579	53684.2511	59442.00397	20084.38248	27538.62811	26029.59968	50419.74291	39985.41784	51401.28453
0.71_700.3661n	699.3679057	0.714216667	neg	11414.52747	9904.73232	12863.36372	2728.005737	6950.697637	4801.17318	15576.31098	7110.524536	7747.76255
0.71_701.8434m/z	701.8434085	0.714216667	neg	607.229504	2565.967079	3534.335695	374.7305442	1244.614335	1.318030913	0.001403199	8688.760315	3521.017816
0.71_707.8510n	688.8331838	0.714216667	neg	260783.905	241523.4746	274884.3218	184368.8446	226884.6119	192571.65	258774.222	223455.9226	226679.8369
0.71_708.3528n	707.3553753	0.714216667	neg	10798.3568	9670.743139	10467.78535	2909.410402	6435.354896	5195.210749	13283.61614	9082.103429	10015.2334
0.71_717.8178m/z	717.817772	0.714216667	neg	0.001403199	0.001403199	6786.62757	4320.147559	2946.193021	1973.346876	0.045037307	4491.436279	4037.884179
0.71_723.8252n	704.8073115	0.714216667	neg	87660.2753	79561.48127	104563.0883	85470.07476	124230.4356	96833.44692	93438.45112	82447.39241	88856.59829
0.71_724.8729m/z	724.8728528	0.714216667	neg	183256.4301	170404.5016	153292.6451	60009.29034	50890.02542	61820.48757	152588.7888	126327.4629	107256.5987
0.71_727.3916m/z	727.3915819	0.714216667	neg	6277.76274	4434.773847	7111.760656	2250.60808	3225.277814	3456.773545	6183.34861	3393.499096	6076.630478
0.71_730.8188m/z	730.8188121	0.714216667	neg	17929.54822	17202.04175	17797.253	16775.82133	23363.01963	16844.67491	19171.73086	22309.72643	21388.7027
0.71_733.8589n	714.8410176	0.714216667	neg	76427.69561	77903.3331	80036.0047	29558.75543	39125.70708	34251.32008	78129.06878	56654.96365	63537.73239
0.71_734.3609n	715.3430116	0.714216667	neg	16956.30134	16733.82586	13433.18226	4955.24627	8151.969851	5769.240942	17333.24954	11253.75677	13660.60679
0.71_737.8379m/z	737.8379253	0.714216667	neg	4119.547982	4522.870816	3857.722697	2426.092444	2223.625199	2878.009873	3760.523917	2244.514832	4253.44899
0.71_739.7990n	720.7811276	0.714216667	neg	14723.53096	11579.45606	19285.68828	27364.35722	39745.84288	26708.81934	13586.32029	14763.83475	14923.90429
0.71_740.8469m/z	740.8469371	0.714216667	neg	302663.6011	287595.8179	292034.0918	138199.8776	157332.5996	152343.9142	285809.5364	230247.9416	246581.4618
0.71_742.3478n	741.34927	0.714216667	neg	17661.87072	11226.60758	16635.18207	6711.156714	10056.70963	10817.67343	13313.84709	13662.48558	12946.87116
0.71_744.8204n	725.8026077	0.714216667	neg	3816.525202	5137.314755	7276.485436	11709.2989	4907.352411	6368.2424	2427.568167	15487.74298	12707.51183
0.71_745.8778m/z	745.8778318	0.714216667	neg	4298.372345	3727.753863	4092.829814	6718.89764	6459.802789	6285.474221	4837.775758	5310.836487	2778.835614
0.71_746.8025m/z	746.8025213	0.714216667	neg	6562.611934	11138.46173	13554.02088	23293.24008	10402.27042	11723.17376	8359.227671	24511.39246	15399.69633
0.71_754.3952n	753.3978714	0.714216667	neg	8653.564795	9198.727944	11578.1821	5670.583531	5912.867142	7585.230407	12826.07119	8824.632926	6929.717444
0.71_761.3846m/z	761.384629	0.714216667	neg	6281.715414	7363.455964	7634.314056	4696.780042	6189.760956	5767.351168	8211.239538	7542.104614	9938.389835
0.71_762.7765m/z	762.7765326	0.714216667	neg	1550.713252	7035.232304	12712.75033	23318.25294	10150.92708	11047.35898	4309.051617	16862.15723	12128.51968
0.71_764.8071m/z	764.8071176	0.714216667	neg	9283.071826	13681.60831	15943.02712	14376.75621	23912.91344	15590.4839	12777.76395	12556.7712	13134.58089
0.71_767.8510n	748.8331445	0.714216667	neg	95184.07605	73245.90627	83507.09483	33402.19788	44257.1964	40605.52922	85870.6479	58583.44105	63153.54745
0.71_768.3539n	749.3361021	0.714216667	neg	24050.20303	14518.60927	24380.4865	4478.993924	9568.439735	3684.080171	22318.11551	11610.58846	16852.98021
0.71_769.8297m/z	769.8297068	0.714216667	neg	714.156805	1698.788011	3772.705326	2486.957363	108.504901	0.001403199	0.001403199	4787.600644	3130.629959
0.71_775.8385n	756.8206838	0.714216667	neg	257324.6111	245505.7117	257771.3058	161921.1886	197930.4492	168369.1885	266225.1441	203218.1964	213764.0518
0.71_776.3402n	775.3429972	0.714216667	neg	20416.00705	21039.11437	19932.64715	6800.78062	10596.88927	8509.367689	20493.4869	14027.00935	16982.22443
0.71_777.8159m/z	777.8158847	0.714216667	neg	14616.67731	10482.90521	15662.00474	8244.415083	5874.416944	6083.794955	11011.45893	12546.99197	9454.139587
0.71_778.7510m/z	778.7510417	0.714216667	neg	0.001403199	1521.099302	3007.448351	10847.51349	5399.985618	3520.739974	697.903439	9778.351242	5052.2577
0.71_785.8062m/z	785.8061743	0.714216667	neg	17.36100048	3015.763793	5813.235074	8509.020874	4973.698961	6231.970121	3955.400272	10830.42147	4983.970239
0.71_788.3890n	769.3711354	0.714216667	neg	12488.26581	10582.90257	15169.24489	8682.104007	7326.258451	8849.183631	17139.09782	12480.71818	15362.55607
0.71_791.8127n	772.7948592	0.714216667	neg	104229.7981	103012.2924	122156.4639	103863.4403	137880.2063	108179.4399	110773.2776	98131.77857	113044.7876
0.71_792.8603m/z	792.8602582	0.714216667	neg	107961.6201	104164.2642	96261.30641	31505.72452	28721.58679	29223.77332	90131.33573	70453.55166	67408.35464
0.71_795.3783m/z	795.3783161	0.714216667	neg	7277.67249	7933.323559	6338.105013	4270.084466	6361.753463	3941.463302	12346.35218	4965.196411	5662.912935
0.71_797.8966m/z	797.8966103	0.714216667	neg	15975.61647	13916.45273	15670.89432	17673.76853	18102.83912	20276.20816	18441.0264	17004.98312	19611.59485
0.71_798.8041m/z	798.8041412	0.714216667	neg	23264.55249	20931.30374	27496.10489	17960.79437	31075.85569	17028.27371	26516.02592	22155.75334	16728.45616
0.71_801.8460n	782.8281849	0.714216667	neg	92974.34245	88724.26815	86113.44356	31514.9198	40455.21862	35999.93813	85142.3127	67467.48181	68585.97815
0.71_802.3475n	783.3297015	0.714216667	neg	22793.08482	20206.61958	24705.17325	5041.986376	6866.962762	7109.520166	15405.73847	14448.14639	14944.82047
0.71_803.8641m/z	803.8640961	0.714216667	neg	0.36224189	2355.878992	0.119735276	2229.227634	2138.492773	31.70929643	3329.159351	532.7976892	10.85179309
0.71_807.2918m/z	807.2918217	0.714216667	neg	0.001403199	0.001403199	3779.132373	0.001403199	3448.618491	1819.89	0.001403199	937.0758057	2895.248583
0.71_807.7865n	788.768667	0.714216667	neg	19697.20954	17486.02551	26403.49177	25813.51845	51238.917	34680.31359	22790.79977	17663.27679	22304.0942
0.71_808.8342m/z	808.8342126	0.714216667	neg	217536.0593	203298.0585	200741.3611	89631.55016	96354.05266	94965.60234	201730.3667	157707.4604	174876.1454
0.71_810.3346n	809.3357744	0.714216667	neg	19852.23194	21192.97655	20063.71382	8660.162608	10611.23923	8661.004275	15334.30545	14446.73916	19213.25503
0.71_812.8086n	793.7907254	0.714216667	neg	627.9815817	3440.163555	9199.138564	12087.40526	9366.187847	4401.304587	2336.048893	14590.01684	10280.32011
0.71_818.3217n	817.3232701	0.714216667	neg	11300.61306	12411.14847	13135.74997	13567.54706	17231.66406	11329.00981	16120.52376	14720.17934	15446.81091
0.71_820.7976n	819.7968649	0.714216667	neg	4488.186886	8279.097598	10580.35955	16993.34162	10756.36615	6892.781849	6962.934654	20224.77001	9171.228753
0.71_822.3826n	803.3647888	0.714216667	neg	16895.46495	12862.7626	10557.34098	6355.17837	7599.83651	9698.725746	12916.10968	10848.92237	14439.84292
0.71_830.3693n	829.3719004	0.714216667	neg	15189.0443	13923.27776	11280.0465	6867.106433	11596.24636	8758.931162	14119.78937	9927.539758	13940.20323
0.71_830.7637m/z	830.763694	0.714216667	neg	0.001403199	10011.39942	9573.576153	23937.69991	9617.324264	10617.31369	5613.012905	26816.32331	11646.51895
0.71_833.8069n	832.7951369	0.714216667	neg	24604.05315	25745.06188	34812.77297	36173.50152	38833.28492	33625.26587	31825.73443	47095.01296	34132.77013
0.71_835.3428m/z	835.3427541	0.714216667	neg	6875.32919	5753.883837	5008.445474	587.8176215	1134.043722	660.3831192	5276.434366	2354.567871	6210.659886
0.71_835.8387n	816.8208248	0.714216667	neg	79025.59147	66894.71983	75680.11892	18049.10756	34396.34413	33486.72847	80639.04334	49385.57364	68828.17988
0.71_843.8262n	824.8084057	0.714216667	neg	213411.8711	202122.3476	218750.0096	115700.0302	143908.5078	126320.8413	226126.5415	176848.5108	180842.5414
0.71_844.3284n	843.3297017	0.714216667	neg	18073.47346	20361.25681	24129.34157	7127.228755	10316.16494	6494.450775	20991.3957	14085.67453	20642.33275
0.71_846.8040n	845.8042063	0.714216667	neg	2718.866133	4280.101528	6465.636212	9340.375436	4429.400626	2424.258811	6024.384848	12948.96838	10196.57696
0.71_847.8059m/z	847.8059335	0.714216667	neg	6776.036665	5304.361877	5593.496503	2393.602407	2224.837108	4710.094745	4950.459817	3036.653564	3600.897219
0.71_854.7908n	853.7930931	0.714216667	neg	6968.089086	3887.005912	12122.61434	11526.96639	7315.840327	2561.923187	5374.222071	13607.76549	10978.39813
0.71_856.3756n	837.3577464	0.714216667	neg	13148.00947	13503.15283	10016.66801	5063.097351	8001.002395	6157.762175	18554.03467	10725.60008	15439.10642
0.71_859.7998n	840.7819489	0.714216667	neg	101491.8758	84443.64821	111605.7646	79886.67204	127365.6808	86252.76454	103544.7238	89497.73687	83954.31682
0.71_860.8472m/z	860.8471584	0.714216667	neg	64991.35153	56390.34548	50835.87829	12059.06849	14919.57513	16821.93163	47319.54479	35607.36223	35294.12332
0.71_861.7780m/z	861.7780021	0.714216667	neg	2283.494006	2059.06883	3112.455389	7292.789905	3687.493251	8054.356479	0.001403199	12982.59312	5893.141903
0.71_864.3621n	863.3657727	0.714216667	neg	14076.23575	15789.58414	7371.341436	6615.228031	12065.32838	10042.02268	17893.58457	12053.24853	15477.14519
0.71_865.8834m/z	865.8834438	0.714216667	neg	10528.86706	10148.49055	10553.14146	13906.70507	15925.47268	13813.75917	15002.95674	13156.37254	17394.41726
0.71_866.7904m/z	866.7904486	0.714216667	neg	21966.4432	21501.2375	27140.24092	15342.81171	25300.80077	18774.20986	24098.70587	22550.72462	23629.1814
0.71_869.8329n	850.8151036	0.714216667	neg	69516.36183	64204.91097	71591.64106	19536.0242	29493.27612	18312.88989	57753.90843	47967.49095	52291.57596
0.71_870.3351n	851.3172371	0.714216667	neg	12108.97046	22710.08664	18632.05776	7164.093737	8986.85993	7337.245372	25924.42427	11066.23804	15636.85465
0.71_870.7725n	915.7940163	0.714216667	neg	6914.348105	4571.562231	4408.338234	6158.395554	4606.306484	3222.990704	4576.09957	4198.222705	2399.202458
0.71_873.8130m/z	873.8129678	0.714216667	neg	4301.812043	4147.764803	4258.664578	592.0748559	0.001403199	712.7535973	6238.615365	0.001403199	0.001403199
0.71_875.7739n	856.7561047	0.714216667	neg	19064.68741	16557.8841	21736.68126	23547.80142	54778.46104	30335.2914	26393.91658	20388.67969	19669.19748
0.71_876.8215m/z	876.8215049	0.714216667	neg	145780.58	135785.2013	128612.3585	50049.3521	55518.12254	52248.62538	128384.9284	100480.4452	112476.3247
0.71_878.3225n	859.3046289	0.714216667	neg	16911.12976	11589.32269	19505.09157	5990.224057	9913.975586	5633.180255	19562.80981	13470.81943	15676.9255
0.71_898.3567n	897.3587528	0.714216667	neg	12327.11815	12535.00305	9288.929877	5622.223202	6996.479415	4682.858422	18685.97627	7992.91404	10951.46301
0.71_898.7514m/z	898.7513624	0.714216667	neg	1620.738212	10404.88962	10419.7842	25237.49136	10864.52111	12871.17176	5202.142158	27876.95007	13935.0614
0.71_901.2855m/z	901.2855033	0.714216667	neg	3288.303956	4955.793909	8551.844832	1295.852689	5305.463045	4313.868624	6153.396655	0.001403199	5013.598075
0.71_901.7930n	900.7825684	0.714216667	neg	15974.23026	25526.16044	35812.28396	30767.13018	35541.84143	25693.28107	28676.79216	40652.68109	38020.2558
0.71_903.8264n	884.8085639	0.714216667	neg	71652.51774	56993.23396	63715.20646	13553.37503	18460.43928	18516.8016	58910.81839	39286.28837	45109.29314
0.71_904.3285n	885.3106215	0.714216667	neg	15170.9682	15158.39121	14424.52498	7254.264426	9490.954001	8958.041778	22862.83343	10950.20314	16364.58627
0.71_905.8471m/z	905.8470625	0.714216667	neg	5698.156154	3330.452098	1415.864691	640.3146318	1406.43815	2580.876069	3564.839239	2312.543864	4168.518579
0.71_911.8135n	892.7956427	0.714216667	neg	172218.6941	158410.5392	152060.6949	74546.42241	97439.07105	82751.89443	158792.5554	121129.1128	135166.3345
0.71_912.3160n	893.2981639	0.714216667	neg	17893.80806	15404.43336	22596.56643	7028.091457	5616.982738	4827.227874	17712.7433	11033.65612	15282.6289
0.71_917.8977m/z	917.8977043	0.714216667	neg	11134.01146	12090.82195	10752.17019	10586.20148	7247.233099	10952.65746	14794.58682	15294.80496	11258.26655
0.71_921.7799m/z	921.7798618	0.714216667	neg	3132.115113	5398.107957	7090.738536	3303.099803	0.001403199	1296.852212	2368.560369	8351.392425	6188.476903
0.71_924.2851m/z	924.2850818	0.714216667	neg	0.001403199	1257.781049	1435.046746	0.001403199	216.0578407	0.001403199	0.001403199	0.001403199	0.001403199
0.71_924.3638n	905.3459633	0.714216667	neg	14841.19657	13062.08289	11348.43533	3454.922463	4429.392169	7062.236112	11457.66071	7131.006104	13980.46859
0.71_927.7875n	908.7696267	0.714216667	neg	83532.38551	81891.06509	105796.9744	58112.31013	100310.9157	65685.11572	93024.5335	79287.31342	83827.25663
0.71_928.8341m/z	928.8341226	0.714216667	neg	35872.61198	28137.25754	27927.9374	7422.528609	4343.77833	7974.376118	24694.65775	22664.73974	17248.6601
0.71_931.7680m/z	931.7680358	0.714216667	neg	3575.027518	1393.489058	0.001403199	0.001403199	3699.334055	2115.123999	3324.288686	1823.97998	0.001403199
0.71_932.3510n	913.3331649	0.714216667	neg	13487.70089	9765.477123	12398.76211	4891.583056	6564.026029	7088.901036	16186.39184	12079.7875	13482.74801
0.71_933.8701m/z	933.8700876	0.714216667	neg	7182.413333	7820.254455	7771.120455	8870.725236	10790.84443	9719.415172	9815.166516	8851.780818	8428.283707
0.71_934.7781m/z	934.7780804	0.714216667	neg	18640.48538	23366.28353	29914.50327	13302.24983	25546.41512	15418.22505	21595.55929	23362.40499	23503.32889
0.71_937.8207n	918.8028765	0.714216667	neg	60678.11467	55610.72099	62992.64487	15028.56125	15393.34455	14676.61132	59724.9588	41725.32239	43515.31637
0.71_938.3231n	919.3052169	0.714216667	neg	18338.2618	13763.64219	18730.52832	7141.824583	8918.01734	5523.405685	18465.57888	6915.39093	10036.49223
0.71_939.3391m/z	939.3390883	0.714216667	neg	7889.625106	7252.326653	7028.868683	2408.914258	5669.142789	4610.417939	8787.230934	5584.003845	5221.379731
0.71_939.8398m/z	939.839822	0.714216667	neg	1599.478597	1354.101027	0.001403199	2030.398266	0.001403199	0.001403199	1563.50244	2342.518157	1299.717624
0.71_943.7610n	924.7431098	0.714216667	neg	22827.63818	15980.38172	23159.72253	30435.25053	47663.33711	34828.21397	23451.40454	20710.3903	23250.84841
0.71_944.8089m/z	944.8089314	0.714216667	neg	96886.90033	88011.42465	95963.5655	24823.52916	32480.10624	30647.95335	82691.99199	59031.99649	67039.54179
0.71_946.3096n	927.2917569	0.714216667	neg	16829.38178	15704.08619	18539.92661	3124.684621	5683.333722	7558.244584	10727.65251	9712.120791	12077.81797
0.71_947.3253m/z	947.3253233	0.714216667	neg	6142.834324	2742.948399	2203.940417	3571.098063	3564.485841	3330.847455	8703.737913	5046.290543	6617.259573
0.71_950.7605m/z	950.7604735	0.714216667	neg	1639.256986	9611.246962	9588.720764	16922.64136	13988.65234	11841.65586	7079.595775	20091.37858	13000.19934
0.71_954.2972n	953.2977168	0.714216667	neg	13325.61034	12612.56246	18261.11468	2326.066391	5873.077484	2912.343899	8945.241848	6225.002323	11099.66368
0.71_955.7707m/z	955.7707461	0.714216667	neg	805.4006571	7915.729842	7981.424883	3635.321184	0.001403199	2894.215064	3127.308995	6041.553603	1776.400924
0.71_966.7374m/z	966.7373943	0.714216667	neg	2375.58087	9180.898115	11111.69681	17923.82	11459.04922	11369.00655	5004.574258	17862.86827	11601.15246
0.71_971.8137n	952.7958793	0.714216667	neg	54619.61809	48472.10268	47969.06735	8665.697184	10714.84241	8626.33728	49242.66885	29444.64651	29766.12906
0.71_973.3332m/z	973.3332182	0.714216667	neg	8242.478096	7518.354676	5886.087771	2136.674203	1452.809832	1458.549661	5478.973698	5139.2995	1738.292241
0.71_973.8349m/z	973.834851	0.714216667	neg	4693.582327	3133.228406	1026.675312	0.001403199	0.001403199	1993.189945	4350.102397	1403.412038	0.001403199
0.71_979.8004n	960.7825741	0.714216667	neg	116501.1576	116947.8036	116447.2652	47475.77155	65055.86647	53117.97152	112128.1642	80456.83524	96448.10817
0.71_980.3020n	961.2841329	0.714216667	neg	9884.602759	9299.72749	16858.33647	2935.409528	2396.941673	3363.590829	12206.48561	9734.515574	9907.634692
0.71_987.7880n	986.7901562	0.714216667	neg	45988.70048	45889.93652	52253.77985	10441.19844	25475.00315	15739.01241	44817.12619	37465.85558	43228.24977
0.71_988.2879n	987.2908695	0.714216667	neg	8326.291832	10941.78765	15936.36228	6524.690719	3437.899304	3138.543295	16690.23568	6854.837489	12240.69426

0.71_995.7744n	976.7566051	0.714216667	neg	75260.91284	60773.28079	69983.50514	46126.45836	60145.40472	50918.9443	68460.12957	59260.31863	62609.94015
0.71_996.8222m/z	996.822153	0.714216667	neg	19719.03751	15042.09332	14809.40694	3341.808228	1737.539381	3526.894554	15004.92533	10956.43555	10254.8052
0.74_1123.2654m/z	1123.265434	0.7359	neg	5152.922958	0.001403199	1663.254563	0.001403199	0.001403199	0.001403199	4800.957555	0.001403199	2506.294777
0.74_113.0197m/z	113.019673	0.7359	neg	216.764497	102.2376454	152.9521337	175.2806605	79.40214132	174.747673	216.5118445	127.1335836	233.9105988
0.74_115.0513m/z	115.0513491	0.7359	neg	289.3321929	281.967206	261.1746484	184.8180225	265.4982743	349.4510538	287.9597389	603.1221079	420.4679055
0.74_124.0629m/z	124.0629002	0.7359	neg	388.371274	286.6600945	565.9355979	495.8175963	595.6648181	289.438435	506.6893532	368.5480537	531.5629696
0.74_131.0715m/z	131.0714791	0.7359	neg	253.5054015	171.8020713	92.20016445	132.1645038	102.7650382	216.9627568	169.6229322	123.640289	105.635301
0.74_164.9571m/z	164.9570928	0.7359	neg	2754.73577	1424.86264	2558.310763	4061.264521	5880.730135	5071.051227	1350.649943	4488.53956	4025.154449
0.74_172.0229m/z	172.0229054	0.7359	neg	6507.219467	3932.159037	13412.02308	4211.34151	14261.59325	4329.008668	9724.910603	11051.61619	17279.17474
0.74_186.0386m/z	186.0386158	0.7359	neg	749.5924771	235.013896	653.6578069	164.1210258	799.3669199	356.5258393	163.5027854	1743.759644	1236.334625
0.74_186.9295m/z	186.9295336	0.7359	neg	3885.583306	7917.898449	9988.508792	20447.10966	11280.61565	12248.85836	6276.542555	17687.66586	13064.18564
0.74_191.9951m/z	191.9951496	0.7359	neg	1038.880526	421.5928927	1072.473233	742.2822055	2733.041197	1506.368011	268.6659277	1733.795895	1540.751154
0.74_240.0105m/z	240.0104923	0.7359	neg	3017.998909	1978.586315	4106.386646	2032.893457	4183.963364	2875.393854	3662.317476	2657.326844	4951.200198
0.74_243.0029n	223.9850187	0.7359	neg	2792.108749	2090.246014	5875.450811	3487.177	4694.926144	3344.328848	4654.886631	4201.413944	6107.535107
0.74_248.9499m/z	248.9498515	0.7359	neg	280.0714049	318.1834195	360.2988766	133.5791743	130.0810532	228.752801	368.1585694	151.9490051	160.6417751
0.74_254.0262m/z	254.0261968	0.7359	neg	144.0300263	0.001403199	70.61096117	0.001403199	232.0376067	0.001403199	0.001403199	347.8104172	246.9076389
0.74_254.9171m/z	254.9170594	0.7359	neg	8737.914463	17761.14561	22654.9203	40585.38509	22376.35853	24269.837	14382.32673	36923.06743	26973.04756
0.74_254.9266m/z	254.9266399	0.7359	neg	1016.882969	549.7415733	4261.255985	447.281421	5009.318246	1336.537317	1141.503248	4094.277513	5598.588124
0.74_259.9824m/z	259.9824446	0.7359	neg	929.5061654	1011.558029	786.4101412	0.001403199	706.9009069	402.4699354	405.5088006	452.7323914	894.6151582
0.74_267.0214m/z	267.0213738	0.7359	neg	490.2022829	457.4901403	1114.386964	257.2535055	814.6398948	172.4103213	1299.177368	705.0579408	1459.818437
0.74_270.8911m/z	270.891063	0.7359	neg	1423.622903	5213.597787	6863.981742	20928.64178	10094.51201	10655.91517	4420.491713	12924.02492	9214.783407
0.74_274.0119m/z	274.0118778	0.7359	neg	507.5232436	1148.405597	1094.677253	2834.82438	2797.937732	1938.848127	723.2011078	2346.918796	2247.209736
0.74_282.1073m/z	282.1073296	0.7359	neg	642.1455951	516.3369019	605.4758659	720.0521712	820.6849241	613.9624995	485.3058449	464.5433128	430.8388715
0.74_291.9820m/z	291.9820221	0.7359	neg	377.9603722	250.9434079	638.8766964	525.2606741	2018.28342	919.2742458	1.650441576	1331.113995	1351.689545
0.74_300.0549n	281.0370525	0.7359	neg	1921.038325	909.5141285	4669.570391	648.0483421	4031.293372	837.4318042	2807.428438	3349.075687	5805.200639
0.74_304.0657m/z	304.0656554	0.7359	neg	1679.805599	1325.708458	2852.129865	959.8457009	1476.639926	585.062781	1994.880244	1738.579681	2694.974509
0.74_307.9980m/z	307.9979625	0.7359	neg	8780.406097	8050.206764	11353.07975	4615.907664	7106.683519	6652.991555	12258.43856	7681.703664	12177.72705
0.74_322.9044m/z	322.9043602	0.7359	neg	8300.361271	18029.79384	21335.13874	38126.2538	18106.63051	21467.43354	12853.91524	32523.46323	24932.19934
0.74_328.9863m/z	328.9863116	0.7359	neg	3470.108197	2748.64175	2674.185032	3644.256354	3894.805171	3896.200261	4602.242653	2755.918765	3415.849221
0.74_335.0091m/z	335.0090501	0.7359	neg	1114.039622	748.4520592	1955.417067	607.7243336	1038.543142	368.0619191	2206.073106	923.367825	2120.573439
0.74_338.8784m/z	338.8783594	0.7359	neg	2034.838085	6882.058747	8880.491342	23753.9165	9137.428503	12121.99244	4159.30093	14590.26897	11298.54973
0.74_349.0246m/z	349.0246211	0.7359	neg	3169.148377	1521.647005	6672.791272	393.384677	3425.884797	706.363163	4299.588089	2875.391158	6950.905275
0.74_358.9669m/z	358.9669237	0.7359	neg	387.9300608	680.6156762	874.4969995	2205.968241	3272.885373	2165.226515	414.6819137	2183.786695	1918.12496
0.74_375.9854m/z	375.9853674	0.7359	neg	7721.060748	5714.521783	8578.49013	2669.339543	2787.954879	2399.263928	11038.75594	2960.036938	7219.77575
0.74_391.9596m/z	391.9596114	0.7359	neg	3005.509555	2146.985892	2758.417153	1535.849935	0.001403199	0.001403199	4564.372992	866.6216151	0.001403199
0.74_396.9737m/z	396.9736806	0.7359	neg	3054.084862	3018.950276	2483.712098	3302.223549	795.5451519	3244.175193	3478.395591	2470.291669	1253.981294
0.74_422.9687m/z	422.9687312	0.7359	neg	50.28694026	0.001403199	1016.900889	0.001403199	961.7063568	0.001403199	284.3627956	1352.730386	1492.207964
0.74_426.8386m/z	426.8386305	0.7359	neg	934.5714457	1121.293135	1268.871684	2377.765646	1452.735265	1615.221017	1598.310684	1338.150539	0.001403199
0.74_432.9867m/z	432.9866572	0.7359	neg	1464.236857	1371.085414	2722.099482	1039.071065	2060.063001	1109.646247	2846.485633	1423.105791	2766.110979
0.74_443.9731m/z	443.9731295	0.7359	neg	6690.208614	5866.204315	6768.361628	2170.977645	1978.894053	2423.573597	9762.717838	1741.680084	5284.409129
0.74_459.9470m/z	459.9470399	0.7359	neg	4737.578604	3225.856296	3605.332555	2089.568098	1369.503098	1852.229169	5565.421966	498.4441834	2833.534619
0.74_500.9740m/z	500.9740173	0.7359	neg	1941.495849	1313.1613	2684.753252	931.3325367	885.7944665	1057.216195	3025.30818	1112.870706	2428.387612
0.74_527.9352m/z	527.9351878	0.7359	neg	5806.869272	3975.843154	4756.248814	2721.12258	1726.663799	2359.536216	6899.30516	1204.833397	3086.231603
0.74_532.8239m/z	532.8238944	0.7359	neg	0.001403199	0.001403199	297.7659843	5149.780291	4124.904184	5866.982363	0.001403199	0.001403199	0.001403199
0.74_552.9872m/z	552.9872171	0.7359	neg	2056.215031	1013.905995	3397.401082	492.6655367	835.750573	0.001403199	2901.164832	609.3292556	2572.388434
0.74_558.8153m/z	558.8153464	0.7359	neg	448.8471458	4203.833911	5968.894156	17070.93196	9153.884086	9134.307549	857.8506768	9785.444247	6799.225432
0.74_595.9217m/z	595.9217035	0.7359	neg	6662.196434	4384.069813	3047.610754	3445.135372	516.1027753	2424.206051	8419.048441	181.9203359	2352.632751
0.74_626.8032m/z	626.8031571	0.7359	neg	2321.661518	6537.917077	7899.462292	22246.20435	11238.30488	11856.57718	2916.389607	13570.15698	9021.82583
0.74_630.8013m/z	630.8012793	0.7359	neg	3841.541563	3508.366233	2514.990497	4872.95237	3186.081514	4127.421557	3753.484812	2556.002411	1832.494182
0.74_642.7764m/z	642.7764406	0.7359	neg	643.2474183	906.4303985	1364.950445	7993.098616	3852.774885	4457.05026	651.4134866	5308.981491	2177.084453
0.74_753.8977m/z	753.8977332	0.7359	neg	5845.218085	2164.999555	1618.078882	549.0339301	0.001403199	1114.739318	5178.507716	1346.303467	1144.326386
0.74_763.8464m/z	763.8464453	0.7359	neg	3424.116106	4598.488732	1513.989279	454.9792245	660.6560322	1281.67096	1824.543268	2218.216431	1526.424092
0.74_766.7760m/z	766.7760145	0.7359	neg	3025.994872	3443.148053	1897.217049	4752.868031	0.001403199	1508.073776	2974.700088	2608.392295	0.001403199
0.74_769.8722m/z	769.8721602	0.7359	neg	6658.936421	5608.175279	2516.965379	2189.108733	4721.786854	3437.401011	7123.153457	1582.299402	535.0526324
0.74_837.8599m/z	837.8599345	0.7359	neg	2657.645197	3785.315931	891.0082276	988.171919	2055.799321	7.461003056	5213.906395	32.10480926	2256.389314
0.74_840.3211m/z	840.3211378	0.7359	neg	3075.51682	1095.200336	0.001403199	0.001403199	0.001403199	0.001403199	0.001403199	0.001403199	0.001403199
0.74_841.2839m/z	841.2839244	0.7359	neg	16661.38995	10662.52615	14737.05667	10917.54511	16922.57821	10497.86956	14319.27487	13230.3293	15212.00424
0.74_909.2719m/z	909.2719221	0.7359	neg	9726.491352	7415.295904	6447.95168	5246.640777	8091.811019	3944.004439	13486.9529	5176.702293	11796.83187
0.74_914.7257m/z	914.7257396	0.7359	neg	0.001403199	2626.578034	6082.336997	17397.42791	8976.319106	11389.66307	739.9205533	14215.59418	7007.800281
0.74_925.2448m/z	925.24482	0.7359	neg	0.001403199	1289.338257	894.5003398	925.2935627	2012.958233	0.001403199	1888.40036	0.001403199	931.5984581
0.76_124.0037m/z	124.0037088	0.757316667	neg	27.07565574	13.95830031	0.001403199	110.4560485	0.001403199	135.8133555	0.001403199	0.001403199	0.001403199
0.76_124.0074m/z	124.0073713	0.757316667	neg	25762.08657	21664.44636	20485.33412	44381.31782	35617.14304	57147.4247	19274.16509	19863.42036	18966.89582
0.76_125.0108m/z	125.0107588	0.757316667	neg	442.4719835	425.6696668	302.9830169	825.9996578	540.8586679	1021.024703	224.7892804	344.8505698	280.4252356
0.76_126.0032m/z	126.0031976	0.757316667	neg	1092.542831	690.3943322	841.0229954	1787.781335	1066.259929	2209.912804	767.1744507	616.6008243	455.198507
0.76_142.0511m/z	142.0511118	0.757316667	neg	95.57943062	81.71330685	60.02262898	106.5461054	44.60574623	89.67333491	109.2498881	76.73412418	233.817238
0.76_143.0352m/z	143.0351983	0.757316667	neg	135.3968115	91.36117876	22.14173986	93.98235342	103.6887503	104.5523744	0.001403199	53.20381507	0.001403199
0.76_161.9942m/z	161.9941654	0.757316667	neg	1746.789298	946.4729882	265.3410724	214.3742834	0.001403199	232.0363946	500.0560714	1953.987211	0.001403199
0.76_164.9501m/z	164.9501108	0.757316667	neg	0.001403199	40.10697528	38.11499646	181.6241218	0.001403199	117.8348299	0.001403199	128.0013068	0.001403199
0.76_195.8877m/z	195.8877404	0.757316667	neg	67.92447836	353.1978485	235.5094741	0.001403199	0.001403199	0.001403199	301.2219991	329.8450775	252.2875543
0.76_196.8949m/z	196.8949364	0.757316667	neg	385.6361818	212.2336864	246.0647832	1633.777747	1459.451325	1317.499326	266.5571401	268.6515838	199.0980512
0.76_205.0177m/z	205.0177401	0.757316667	neg	1504.712227	1212.318708	406.7668525	151.0065997	0.001403199	155.4731417	574.1945866	1313.700304	198.2305013
0.76_221.9749m/z	221.9748535	0.757316667	neg	2707.258302	3232.230666	3604.396279	14390.91047	14075.10708	13809.59854	3431.553531	3643.373804	3085.1855
0.76_232.9022m/z	232.9022378	0.757316667	neg	271.449344	243.0424058	666.0747092	1811.39397	743.5845372	2034.435383	413.8931306	803.8728409	722.6185274
0.76_239.0167m/z	239.0167377	0.757316667	neg	8008.630689	8300.910355	7982.193245	13079.88958	13211.29728	14568.20265	9530.855118	8994.800539	9910.050488
0.76_243.9567m/z	243.956711	0.757316667	neg	15278.34234	19011.4833	21614.78301	46347.85762	34033.20779	45149.55755	21803.08007	21110.22104	20179.67814
0.76_243.9661m/z	243.9660689	0.757316667	neg	6391.791249	4929.190559	5290.821521	11223.0148	5406.165426	11705.48692	5285.673858	4869.32064	4590.863468
0.76_259.9306m/z	259.9306269	0.757316667	neg	960.9630073	1283.207809	969.0440547	4331.38467	2467.098383	3751.392903	1618.606803	1355.294557	1141.557498
0.76_265.0115m/z	265.0114955	0.757316667	neg	1741.591383	2458.715897	2331.474922	5278.752091	4597.818678	5502.278043	2069.722387	5284.554382	4172.549253
0.76_276.8472m/z	276.8471597	0.757316667	neg	373.8739549	822.4329474	1170.543987	1758.385374	0.001403199	974.3179144	779.2000592	1867.835185	853.2755542
0.76_285.1061n	284.0992401	0.757316667	neg	3389.005441	2487.376361	3229.755722	3811.758422	5031.730146	5593.413404	1428.698745	5362.39661	2902.343691
0.76_311.9448m/z	311.9448333	0.757316667	neg	20.97542915	238.5727595	33.62193694	557.982989	0.001403199	602.789052	0.001403199	150.3594705	24.30931971
0.76_311.9541m/z	311.9540895	0.757316667	neg	645.4895132	374.0199842	1389.75933	1696.257654	2777.630734	2143.939592	466.3658924	2147.067461	2415.947395
0.76_323.8973m/z	323.8973026	0.757316667	neg	1421.516265	1760.831812	2142.931645	926.4797459	0.001403199	1.810426132	1906.334605	1349.628459	806.0580061
0.76_334.1259m/z	334.1259361	0.757316667	neg	3097.861304	3485.390328	4583.293003	2234.47456	3528.323875	3068.848426	3200.213316	3326.448209	3604.377982
0.76_375.0999m/z	375.0998786	0.757316667	neg	410.2411474	329.7807781	963.0550058	293.8850765	230.236482	214.4948144	232.6222888	453.7040265	1012.606562
0.76_466.1239m/z	466.1239193	0.757316667	neg	4096.754698	4298.179051	5554.56661	3629.348421	1719.315094	5896.877675	3870.335462	2444.247411	1764.070032
0.76_831.2545m/z	831.2545189	0.757316667	neg	9730.721157	10847.34565	14217.18276	2499.976228	2614.236564	3350.437344	14393.61547	9053.642944	10315.09958
0.78_132.9463m/z	132.9463162	0.778533333	neg	8736.378909	8573.805409	9776.988109	14936.95489	11090.94339	14241.91994	11001.90275	10663.21634	7258.832691
0.78_134.9434m/z	134.9434127	0.778533333	neg	2897.628699	2851.773849	3574.828972	5310.993702	3691.277341	5045.704833	3885.46867	3803.614144	2625.459226
0.78_149.0357n	148.0283846	0.778533333	neg	3796.016602	4171.133084	4634.808163	1873.773037	389.6512722	976.1742433	3864.428276	4572.355692	1707.635848
0.78_150.8866m/z	150.8866196	0.778533333	neg	2395.523613	2415.674563	2656.184111	0.001403199	452.1513547	131.5028499	2122.047623	2241.192698	1418.902309
0.78_152.8837m/z	152.883703	0.778533333	neg	2365.276888	2345.119862	2603.217217	185.5735553	316.4715078	132.203347	2087.402924	2109.0887	1491.21737
0.78_154.8807m/z	154.8806919	0.778533333	neg	481.9363084	716.0131546	258.4914923	0.001403199	0.001403199	0.001403199	194.8775807	177.5322037	0.001403199
0.78_154.9282m/z	154.928244	0.778533333	neg	21650.18925	19203.3316	20418.92617	14013.88314	15017.85891	13722.52936	22392.85959	21076.76357	20449.49852
0.78_156.9253m/z	156.9252704	0.778533333	neg	7123.756233	6240.233698	6739.232678	4863.182247	3087.441253	4338.769347	6920.020242	6523.138451	4092.622472
0.78_160.8421m/z	160.8420772	0.778533333	neg	267091.3074	252733.4283	267185.0424	99982.8411	122090.4749	104066.0701	304861.5749	231639.1117	255786.6834
0.78_162.8392m/z	162.839167	0.778533333	neg	273863.4789	259206.3919	273293.8535	104380.7976	124491.5967	107205.8632	311233.6456	237551.3738	258227.8304
0.78_164.8361m/z	164.8361441	0.778533333	neg	86168.52994	81193.55299	87430.42567	30647.91083	38480.36634	32414.92619	97341.18813	74383.5179	82856.63161
0.78_172.8424m/z	172.842429	0.778533333	neg	3123.963983	2510.255161	5156.546422	3053.006391	4114.228904	2886.205494	4689.897461	3961.640045	6244.682228
0.78_174.0520m/z	174.0519672	0.778533333	neg	2654.098844	2725.714823	2305.268722	1800.60718	1590.53021	2047.922026	2503.159787	2435.198336	2488.855963
0.78_174.8395m/z	174.8395207	0.778533333	neg	2760.282622	2762.1945	6087.562402	3014.73603	4302.703702	2677.508578	5215.311823	3038.67041	6842.978028
0.78_186.8655m/z	186.865503	0.778533333	neg	1523.493544	1233.834613	1382.158381	1598.143219	1702.89755	1584.270889	1742.864008	781.5260525	962.2166446
0.78_186.9650m/z	186.9649702	0.778533333	neg	0.001403199	0.001403199	0.001403199	1082.356275	508.2854834	742.4740983	139.3058192	0.001403199	0.001403199
0.78_195.8110m/z	195.8110023	0.778533333	neg	527899.457	486965.9062	583474.1389	138797.4362	163561.2636	154000.731	566304.1655	540847.1858	587052.0976
0.78_195.9538n	194.9463637	0.778533333	neg	9480.055274	8857.986691	9884.290118	52039.80998	52071.91252	52051.06743	12468.51835	12185.53354	12252.04775
0.78_197.8080m/z	197.8080169	0.778533333	neg	685039.2174	641684.8076	757296.0253	184482.2204	220988.8169	206736.1375	732336.0347	692620.2664	748769.4348
0.78_199.8051m/z	199.8050664	0.778533333	neg	340641.9516	306837.3572	369364.4261	89900.22084	107096.461	101611.381	357964.812	342301.3414	365078.2391
0.78_201.8021m/z	201.8021383	0.778533333	neg	80157.53062	73444.63567	84075.25742	20265.27147	23337.84286	22986.24147	82097.94254	76599.93776	81524.69155
0.78_212.8868m/z	212.8867815	0.778533333	neg	3200.670876	3618.360268	3532.191233	726.9467711	721.0482276	0.001403199	4018.747818	3610.707669	1663.277905
0.78_217.9262n	216.9185182	0.778533333	neg	94139.38354	91578.34009	101288.4765	174382.0207	168246.405	182220.1386	122393.8215	121149.5402	122202.1663

0.78_217.9361n	216.9280025	0.778533333	neg	12929.85266	8826.671383	9603.850828	24363.01072	20077.47727	27279.63612	13770.77768	14080.16493	13159.55729
0.78_218.0245n	435.044852	0.778533333	neg	23584.04087	26409.69197	26927.15645	14332.11968	15693.76151	16583.15781	21363.30772	19196.43565	18726.67039
0.78_221.8345m/z	221.8345028	0.778533333	neg	72.71006263	224.2936526	378.3431072	0.001403199	638.1993173	855.1096562	337.0572331	314.6985912	0.001403199
0.78_232.8925m/z	232.8925097	0.778533333	neg	2244.307537	2794.539495	2625.297139	8318.459553	6911.897841	7886.454314	2776.872519	5235.378391	4062.522517
0.78_250.8333m/z	250.8332945	0.778533333	neg	2657.783019	3131.526233	2965.511323	0.001403199	0.001403199	0.001403199	3159.337654	2272.526741	1099.223559
0.78_252.8303m/z	252.830255	0.778533333	neg	1229.897894	1405.712848	1046.067876	0.001403199	0.001403199	0.001403199	770.7791844	594.2160416	203.0986991
0.78_252.9911m/z	252.9911298	0.778533333	neg	2018.890582	2145.834616	1876.609814	0.001403199	0.001403199	0.001403199	1096.733691	825.1443176	680.8907746
0.78_268.8008m/z	268.8007868	0.778533333	neg	1174.915731	1484.183171	961.00207	0.001403199	0.001403199	0.001403199	1043.373445	654.3903809	1267.722384
0.78_272.0194m/z	272.0194382	0.778533333	neg	954.8635724	1421.044464	1532.308695	1791.055227	1186.06227	1619.081776	559.1171768	1332.706856	1107.110789
0.78_272.0274n	253.0095596	0.778533333	neg	11542.20961	11400.90915	11774.91707	7242.039738	4972.938469	4736.896903	10429.21523	9273.4101	7902.20605
0.78_272.8427m/z	272.8426631	0.778533333	neg	1858.18959	2321.745289	2050.087418	0.001403199	0.001403199	0.001403199	1667.331269	1181.569641	721.2919471
0.78_275.0543m/z	275.054285	0.778533333	neg	20551.19641	22267.91988	25274.06567	18907.41137	20164.86036	18537.5673	23885.2305	27462.53133	27075.45368
0.78_286.0606m/z	286.0606438	0.778533333	neg	1091.966697	1072.314743	459.3615108	1466.020399	0.001403199	207.7617564	702.1817656	0.001403199	0.001403199
0.78_292.8182m/z	292.8182296	0.778533333	neg	4072.120979	4281.537025	4290.531796	0.001403199	173.0969919	0.001403199	4030.000164	3267.22567	2638.379918
0.78_292.9235m/z	292.92346	0.778533333	neg	0.001403199	72.97929313	277.66039	4626.699924	1099.685031	3034.457312	178.9975596	0.001403199	0.001403199
0.78_311.9817m/z	311.9816631	0.778533333	neg	0.001403199	275.2003258	0.001403199	1627.556912	175.2072892	0.001403199	0.001403199	0.001403199	187.1585684
0.78_312.9852m/z	312.985215	0.778533333	neg	12198.19976	13966.28115	13808.78087	8453.059025	9788.503214	10674.22895	11154.89175	12572.40606	12051.2692
0.78_314.8959m/z	314.8959012	0.778533333	neg	9632.381345	8529.311977	10659.81947	44376.74424	27979.68924	39989.41178	15237.35012	14091.43964	7244.727064
0.78_330.9827m/z	330.982726	0.778533333	neg	2473.731057	2942.341066	3021.388008	1057.780138	285.2945881	1260.320989	2577.157311	2301.194986	2056.540964
0.78_363.9066m/z	363.9065599	0.778533333	neg	4513.166275	7003.383703	7284.230432	15514.84698	14107.54378	15040.04863	5621.295654	7726.930438	9451.037884
0.78_368.0882m/z	368.0881554	0.778533333	neg	15955.44354	17435.74738	17970.58012	16954.01239	32948.63075	18753.277	18673.71613	21595.42123	21406.8148
0.78_437.0412m/z	437.0411545	0.778533333	neg	9107.955234	10411.47378	10092.86042	643.4367259	1.311720342	0.001403199	5532.924739	4332.859009	3109.221094
0.78_439.9009m/z	439.9009365	0.778533333	neg	0.001403199	245.6327931	817.0477693	7257.063311	2686.446481	6873.756265	663.7692428	1926.794434	0.001403199
0.78_701.1910m/z	701.1909975	0.778533333	neg	10921.52157	8589.978467	16785.32736	4053.642354	7561.101895	5912.861191	7774.842032	5746.125031	2323.033255
0.78_752.1546m/z	752.1545632	0.778533333	neg	7255.7683	7125.246357	5389.566356	4112.196295	3138.14677	4724.47023	2488.55994	4835.058716	8223.245432
0.80_124.9876m/z	124.9875809	0.800366667	neg	0.001403199	0.001403199	0.001403199	129.8582866	84.10272453	0.001403199	0.001403199	0.001403199	0.001403199
0.80_125.9947m/z	125.9947051	0.800366667	neg	1677.390677	2001.825537	1978.785926	3048.069552	3306.021962	2957.769718	2138.901137	1865.99103	2042.08756
0.80_126.9872m/z	126.9872055	0.800366667	neg	3804.51235	3958.448551	4571.675314	6165.275352	6584.888621	6974.263623	4792.9473	4160.901865	4385.39968
0.80_154.9188m/z	154.9188014	0.800366667	neg	81464.95706	84443.63855	85896.94488	55168.02254	57088.50906	56911.80425	95672.50616	93421.53112	93617.07356
0.80_156.9158m/z	156.9158112	0.800366667	neg	25639.40068	26954.77229	27078.86302	16029.42078	15714.9021	16421.11103	30039.56225	29563.35025	25922.67715
0.80_157.0273m/z	157.0273005	0.800366667	neg	6527.341801	5425.532761	5904.312558	4959.708214	3265.108802	4725.903306	5624.14509	6002.810112	3840.069999
0.80_177.8646n	158.846752	0.800366667	neg	15937.96184	15142.55061	16043.75563	5985.744669	6319.935121	6319.687743	18337.74522	13738.42599	12326.95419
0.80_182.9500m/z	182.9499898	0.800366667	neg	1030.893227	766.9494509	1354.744723	191.041285	0.001403199	392.8799564	948.7666872	882.2051277	300.5739988
0.80_187.0379m/z	187.037932	0.800366667	neg	20075.04045	23027.67284	27009.19007	19756.04067	20100.59594	20525.15068	20195.36241	24289.06961	26658.94673
0.80_188.1041m/z	188.1040889	0.800366667	neg	1394.900268	1268.514963	1320.164307	632.6622649	393.5799125	583.9303364	1023.490326	1530.366974	971.3394528
0.80_190.0422n	379.0824114	0.800366667	neg	82352.77288	84556.68108	94671.9897	48149.47292	64067.787	47841.4837	71837.94995	65624.712	71517.1327
0.80_194.9368m/z	194.9368304	0.800366667	neg	24741.77878	25484.16439	27880.61934	103978.3599	112591.6997	99156.91647	35919.77238	32347.66292	35302.1164
0.80_196.9411m/z	196.9411171	0.800366667	neg	0.001403199	0.001403199	119.3122699	1372.572847	376.4367841	396.051374	0.001403199	0.001403199	0.001403199
0.80_212.8774m/z	212.877449	0.800366667	neg	10679.24905	11868.34784	12348.49754	2842.770835	3406.842228	3312.208714	12720.00803	12084.19084	9754.597817
0.80_213.8529m/z	213.8529407	0.800366667	neg	4543.609342	4251.845364	5088.112274	1296.247622	1271.072875	2162.564602	5235.688302	4504.991773	3383.451466
0.80_215.8499m/z	215.8499037	0.800366667	neg	2230.869211	2602.201987	3033.713051	1063.876341	254.2992069	924.757604	2970.587327	2694.146873	2052.101258
0.80_222.9990m/z	222.9990283	0.800366667	neg	2042.101149	2160.895644	2421.631482	336.9826693	0.001403199	259.3994183	1487.709828	846.2389832	591.8877707
0.80_233.0065m/z	233.0065075	0.800366667	neg	4607.482593	4701.67149	5905.03324	7726.710234	7966.834763	7677.041989	5510.406971	5441.144538	4082.761648
0.80_233.8923n	232.883372	0.800366667	neg	30029.69962	36008.16536	36916.16996	51538.71939	53217.08553	50597.52562	41698.95444	47016.19173	43858.32563
0.80_234.8593m/z	234.8593186	0.800366667	neg	17917.64188	19525.40945	18699.01092	3327.666429	3977.095891	4304.941641	19459.5306	16456.01568	13153.31953
0.80_257.8113m/z	257.8112522	0.800366667	neg	4990.623032	3759.389087	4138.999882	4094.734814	4074.100616	4187.551141	5086.570624	4245.726144	5108.186827
0.80_259.0934m/z	259.0934357	0.800366667	neg	2523.075085	2650.52263	1733.781903	1995.898071	1879.010668	1776.401458	2057.262569	2266.330201	1566.621942
0.80_277.0236m/z	277.0235722	0.800366667	neg	10703.99162	12128.63794	13943.55033	39108.97389	34891.45024	37833.34126	17703.99152	15079.45724	16184.68201
0.80_282.0653m/z	282.0652579	0.800366667	neg	2519.483661	2667.949207	3879.027132	1890.577747	1042.723004	1500.827693	3732.494638	2892.826385	1553.996097
0.80_284.9901m/z	284.9900612	0.800366667	neg	5692.189189	7266.201333	6933.784682	2494.160028	2194.472662	2629.600001	5313.458245	4434.643831	2879.524265
0.80_289.0698m/z	289.0697628	0.800366667	neg	19784.16176	20679.47333	21934.7345	15234.89349	15401.27403	14608.09451	21994.04554	25194.39691	22896.86685
0.80_290.8424m/z	290.8423702	0.800366667	neg	3450.22566	3791.012911	4293.697029	634.2592344	2348.287086	1163.316716	4348.468676	4969.115478	3479.863205
0.80_291.0392m/z	291.0392323	0.800366667	neg	5275.72701	4639.724945	3685.461561	6556.834202	5042.681086	5556.200988	6437.339134	5228.941935	5939.028857
0.80_293.0187m/z	293.0187232	0.800366667	neg	27560.39797	29722.21535	33146.30779	69704.47449	74749.12921	71497.78688	37251.83997	35020.18756	39269.60841
0.80_313.8328n	312.8243599	0.800366667	neg	7126.081483	8566.578467	8337.311825	124.3864908	0.001403199	205.5159781	9529.230986	8789.040758	6990.856298
0.80_315.0007m/z	315.0007432	0.800366667	neg	26266.71703	30305.27714	28843.31936	15729.59893	18555.01207	16042.38142	27841.65078	26493.64475	27566.3585
0.80_319.9184m/z	319.9183674	0.800366667	neg	2315.038036	2311.994697	2136.872735	2273.980601	1432.861422	1621.604599	2276.869477	2120.792809	1883.765352
0.80_333.8025m/z	333.8024647	0.800366667	neg	1443.523918	254.9295492	884.6077452	0.001403199	0.001403199	0.001403199	958.182498	489.2315063	742.5941951
0.80_338.0778m/z	338.0778396	0.800366667	neg	34790.43469	34134.15876	32164.14995	25955.35124	10948.05427	26615.8035	33056.44185	43816.12229	18323.9014
0.80_352.0934m/z	352.0933571	0.800366667	neg	9259.003151	10547.44137	10921.36428	7044.022005	7559.165498	8507.783442	10337.07336	9361.935362	10906.90875
0.80_372.9592m/z	372.9591989	0.800366667	neg	3388.390205	3539.707204	2504.178995	0.001403199	0.001403199	0.001403199	4415.134803	2161.100906	527.6022231
0.80_382.0535m/z	382.0535248	0.800366667	neg	4755.617264	5508.034833	4358.863536	7369.275739	4502.354029	6561.319192	6913.877075	8353.057377	6280.962292
0.80_382.1039m/z	382.1039446	0.800366667	neg	9836.636974	12820.98878	12704.29007	13289.19446	10307.68241	18036.77518	12780.56804	11665.08673	9880.016461
0.80_431.9554m/z	431.9554422	0.800366667	neg	863.0076438	767.6230289	1086.687104	4176.621979	2302.376726	3773.788874	2616.856019	3254.017242	1146.525213
0.80_453.0553m/z	453.0553139	0.800366667	neg	8793.788424	9004.664321	10481.49668	21334.51637	24243.51194	20531.75137	8485.438586	8668.017179	10519.86264
0.80_461.8731m/z	461.8731327	0.800366667	neg	879.3975985	623.39495	2247.397856	7773.832704	4686.632513	8178.447818	1320.827332	1974.682465	543.8874771
0.80_523.1436m/z	523.1435968	0.800366667	neg	18842.78844	19374.84824	24038.32061	1758.294782	1914.291456	1547.87241	13940.56033	14768.70639	15102.11229
0.80_540.1459n	539.1386447	0.800366667	neg	59447.4353	70871.1228	74286.17672	22584.98913	24106.6772	22114.26313	31283.98547	37195.65787	33541.52089
0.80_597.0969m/z	597.0969452	0.800366667	neg	2243.711484	4634.225155	3674.030898	0.001403199	0.001403199	0.001403199	0.001403199	0.001403199	0.001403199
0.80_638.9622m/z	638.9622454	0.800366667	neg	5866.460282	7941.778332	9240.688448	15215.72614	15883.76311	17402.34983	5874.66106	9242.343835	8007.205242
0.80_730.1723m/z	730.1723279	0.800366667	neg	25046.57467	29215.73189	25080.71664	45185.24236	50980.44351	56640.24706	25480.06087	27581.17412	26040.11713
0.80_758.9113m/z	758.9113031	0.800366667	neg	1936.742607	940.3264329	3615.83638	8423.146535	6334.528081	7341.731352	4574.508224	953.0221252	3437.392996
0.82_125.8731m/z	125.8731484	0.819566667	neg	5838.213659	5317.790415	5253.944961	2040.028654	2097.872536	2120.007083	5931.436216	4731.151855	4563.895542
0.82_134.8947m/z	134.8947041	0.819566667	neg	3602.898715	4306.019814	4438.829828	5143.608647	4571.084826	4604.833005	5130.341341	4366.231722	4067.116886
0.82_135.0257m/z	135.0257089	0.819566667	neg	328.2549955	0.001403199	308.1267397	305.9241482	246.5885544	303.8741899	425.0724071	79.86822096	0.001403199
0.82_136.8632m/z	136.8631649	0.819566667	neg	6373.40369	4885.51089	4738.072609	3991.413914	4141.185829	3894.981174	5411.769056	4425.199974	4797.784626
0.82_140.9666m/z	140.9665835	0.819566667	neg	586.8868254	348.0294427	644.9583483	1045.699461	635.8122208	1028.117207	752.7168914	683.3706865	384.1722279
0.82_153.8686m/z	153.8686345	0.819566667	neg	5260.782138	5794.957899	5846.865787	5137.276786	4175.955778	5854.01776	6998.130063	6021.264792	5845.962295
0.82_155.8657m/z	155.8656673	0.819566667	neg	2832.225358	3420.31705	3464.890281	3267.127944	3413.756794	3631.746754	4461.001543	3477.416527	3663.315232
0.82_157.8630m/z	157.8629626	0.819566667	neg	4486.144783	4098.140297	3874.838113	1596.431251	1909.807697	1955.45909	4892.814649	3804.963523	3948.264632
0.82_160.0042n	204.9989693	0.819566667	neg	8966.814439	10026.64744	11287.22841	396.3763219	455.3476621	575.9935558	6027.545053	6263.316163	5472.293437
0.82_174.8609m/z	174.8609349	0.819566667	neg	999.9311855	1309.468402	955.8033581	614.1548185	773.076431	1047.458802	1147.651387	1230.474011	1089.947977
0.82_174.9884m/z	174.9884106	0.819566667	neg	1837.904424	2208.635963	1987.941817	5498.66803	5152.062437	5586.010277	2575.950632	3605.458246	3288.617569
0.82_177.8600m/z	177.8600211	0.819566667	neg	4770.893132	3924.740993	4818.033154	2609.040139	2763.381831	2747.537791	5052.861839	4703.011885	4713.911688
0.82_192.9885m/z	192.9885311	0.819566667	neg	3565.229144	4107.725737	4264.805462	1177.922852	1003.482143	1150.965976	3447.225041	4006.107262	2682.43979
0.82_194.9856m/z	194.9855853	0.819566667	neg	1426.640376	1065.487643	1519.697031	302.5607393	0.001403199	0.001403199	827.8351083	853.9172821	1050.370864
0.82_196.9233m/z	196.9233236	0.819566667	neg	24439.7298	26987.88824	29191.9549	72444.62027	66320.35108	69214.04215	37467.71698	40121.72725	39265.62613
0.82_211.0283m/z	211.0283381	0.819566667	neg	7408.534172	8223.078118	8679.674868	5484.454022	2714.112695	4079.202777	6545.275258	6071.791538	4320.14374
0.82_216.9095m/z	216.9094927	0.819566667	neg	1077112.04	1172453.647	1218050.246	1722819.586	1737788.624	1724703.271	1434229.026	1500452.937	1526573.562
0.82_218.9052m/z	218.9052375	0.819566667	neg	55388.9354	56962.58564	61116.41255	81859.76548	99403.98747	92211.11117	74562.59218	77702.03614	88687.65882
0.82_222.8330m/z	222.8330152	0.819566667	neg	5611.332089	5211.545411	5405.278514	3332.19715	3554.655025	3660.932437	6372.026528	5986.799181	5888.229398
0.82_222.9589m/z	222.9588723	0.819566667	neg	2593.899089	3199.627637	3414.242646	11960.0443	9699.615034	11753.32919	5325.271458	4005.13364	3844.678345
0.82_222.9676m/z	222.9676086	0.819566667	neg	0.001403199	317.6099315	421.7316509	2748.670121	1157.213585	2481.294304	586.6441434	0.001403199	354.948959
0.82_223.8220m/z	223.8219735	0.819566667	neg	4355.190892	4437.674893	4975.180847	2457.330736	2939.956091	2198.430483	4664.292901	4953.606116	4711.098356
0.82_224.8296m/z	224.8295693	0.819566667	neg	4407.000106	4282.620731	4074.191944	1549.954292	2419.421077	1476.590308	4729.467751	3847.380789	4548.489345
0.82_232.9973m/z	232.9973382	0.819566667	neg	3315.172667	3117.094232	2926.135869	6710.961459	2284.933414	4334.885684	4386.901685	3065.406855	2021.04807
0.82_234.8800m/z	234.8799882	0.819566667	neg	1592.731532	1614.413354	2627.32216	5856.795887	4298.348494	4018.935107	2706.683723	3316.579743	2712.647351
0.82_243.0180m/z	243.0180248	0.819566667	neg	2671.815718	3716.300415	3259.440198	5823.791657	4636.452138	5407.076044	3449.183333	3720.731604	3328.640969
0.82_244.0938m/z	244.0938393	0.819566667	neg	606.0167519	1122.372599	550.8570539	0.001403199	0.001403199	0.001403199	593.7965389	773.9117966	264.1097358
0.82_249.8295m/z	249.8295012	0.819566667	neg	414.3410711	1086.388294	44.77138455	267.0868013	20.48837674	47.94728197	24.06110522	349.3152232	503.0701815
0.82_251.8267m/z	251.8267348	0.819566667	neg	1088.505093	0.001403199	0.001403199	267.994161	0.001403199	0.001403199	653.1145409	314.6149368	187.1071731
0.82_255.0052m/z	255.0052101	0.819566667	neg	4613.721196	4820.986813	4961.844893	1559.314582	2244.292644	1430.649804	4085.193237	3612.923584	3415.471211
0.82_259.8946m/z	259.8945944	0.819566667	neg	3172.177403	2619.068662	2471.293501	0.001403199	114.0377751	0.001403199	1491.240404	2250.233363	1263.015474
0.82_261.8918m/z	261.8917907	0.819566667	neg	2108.732282	1289.120781	1450.07218	0.001403199	148.9966287	0.001403199	1039.037094	1036.796152	901.2937149
0.82_263.0078m/z	263.007845	0.819566667	neg	1921.050229	2873.801317	2500.656701	6998.530504	3334.652742	5622.059955	3844.783201	3208.885089	2711.078777
0.82_263.0691n	308.0672855	0.819566667	neg	35464.42887	32586.60974	29189.65093	22276.09146	12455.9655	20780.60267	30187.96371	34672.46539	21878.69747
0.82_264.0772m/z	264.0771697	0.819566667	neg	4036.173905	3888.989008	2609.659316	1358.736458	202.5436634	550.8994152	3916.904076	1735.905202	0.001403199
0.82_271.0518m/z	271.0517704	0.819566667	neg	1596.960648	1825.792385	1965.625107	840.6734007	847.4228896	624.2957086	2077.548255	1646.864284	1536.617438
0.82_274.8683m/z	274.8682678	0.819566667	neg	55610.28888	57249.57502	58355.06919	30290.06845	33299.07053	33740.09907	69882.63419	65890.55859	63067.11668
0.82_275.0080m/z	275.0080298	0.819566667	neg	7707.375522	8307.142264	9563.119903	14382.28064	13684.95303	14788.09543	8370.082901	8249.387588	8774.995424

0.82_276.8650m/z	276.8649767	0.819566667	neg	22320.5342	22878.36805	22270.87612	10733.89694	12639.02073	11879.51804	26444.7882	25855.87638	24444.77824
0.82_304.9821m/z	304.9820795	0.819566667	neg	14450.94442	16339.81314	16831.57516	63172.29877	56934.09993	58744.10321	20652.12518	23656.17636	23473.54627
0.82_305.0550m/z	305.0550299	0.819566667	neg	3589.877704	2654.652901	3690.532284	4448.848283	1901.393489	4399.617007	3863.100318	3976.973398	2073.423321
0.82_314.8771m/z	314.8771313	0.819566667	neg	45507.19995	50649.99783	55999.88254	85792.89026	92271.96741	92272.91715	66263.53943	72125.76599	72100.13357
0.82_316.8730m/z	316.8730441	0.819566667	neg	9937.259294	10509.41179	8838.947028	15195.62879	16139.68697	17167.27392	12059.32921	12768.86528	13111.85925
0.82_319.0574n	300.0395376	0.819566667	neg	83833.28618	90048.34334	102684.2779	116294.3953	113334.9822	111424.0373	93243.48082	96913.31852	95818.80443
0.82_327.0329m/z	327.0328921	0.819566667	neg	2368.970882	2501.955899	2022.084984	1393.891959	1087.861505	1422.875354	2128.855121	1695.921542	625.0621287
0.82_333.0137m/z	333.013698	0.819566667	neg	4601.721678	4930.928748	5885.234727	11661.57669	4763.969447	7124.362313	6866.137441	5872.756187	1847.828067
0.82_337.0580m/z	337.0579994	0.819566667	neg	18117.51889	16862.49673	17085.1178	14526.12977	9585.634835	13390.83863	16772.14457	17992.63424	14820.78088
0.82_346.0815m/z	346.0814867	0.819566667	neg	4777.570358	4400.513006	4287.431068	3332.184671	2467.722969	2703.762466	3622.166433	4363.515442	2079.661832
0.82_350.9931n	331.9752697	0.819566667	neg	8455.900176	6886.987546	11645.48993	7787.650973	8931.103467	7112.280531	4987.503552	6103.367078	5865.667889
0.82_354.8290m/z	354.8290076	0.819566667	neg	3174.60119	3053.73691	3433.566362	5135.184689	5053.707422	5256.304926	4157.03815	4110.807641	4505.057209
0.82_374.8149m/z	374.8148629	0.819566667	neg	10263.96999	9955.995321	11071.27125	8843.697102	6816.593096	8379.038137	12874.00282	14337.87142	12327.37189
0.82_381.0482m/z	381.0481898	0.819566667	neg	3838.518023	3988.954082	3871.971549	8007.535087	6809.626416	9606.735005	5394.648267	4683.332734	4674.852697
0.82_385.0196m/z	385.0196468	0.819566667	neg	5837.735979	7145.53107	7121.358484	10643.66664	10287.12512	10250.26049	8166.41802	9441.540161	8340.526875
0.82_395.0637m/z	395.0636762	0.819566667	neg	14018.92833	17930.44113	17703.07216	34362.14435	39378.4898	32854.17499	19483.44426	20216.73578	23357.70274
0.82_418.1123m/z	418.1122755	0.819566667	neg	5871.895972	6109.143915	5490.609235	4193.819145	2827.448699	2513.195744	5707.003794	4592.702478	3593.310907
0.82_423.0228m/z	423.0228126	0.819566667	neg	400.8666743	1048.799753	1092.83784	3478.154282	2452.696614	2613.152131	1239.132867	1290.295756	1036.185254
0.82_427.9360m/z	427.9359705	0.819566667	neg	546.745079	357.3677353	0.001403199	4057.943758	3587.294506	3602.840787	1824.727812	1883.385565	2653.739526
0.82_428.0477m/z	428.0477156	0.819566667	neg	8526.785923	10071.84938	8769.263662	6505.185868	6512.449436	6893.407148	9379.070972	8004.367363	8809.182596
0.82_434.0974m/z	434.0973866	0.819566667	neg	6708.940265	7307.369778	7487.482983	6416.912858	2255.918651	5214.912597	3724.954442	5812.809021	4056.55795
0.82_472.7816m/z	472.7815822	0.819566667	neg	16566.01457	15432.71414	17333.25909	27863.13873	24686.29265	29241.11637	23555.85977	29374.11793	25121.25033
0.82_481.1105n	480.102375	0.819566667	neg	9270.390204	8325.81891	9971.336689	13584.5747	10083.30717	10084.34353	6082.891298	9121.265913	6790.7783
0.82_497.0340m/z	497.0339923	0.819566667	neg	7466.378105	7806.340431	6208.340346	1498.912691	0.001403199	0.001403199	5863.182164	3269.813324	1828.204447
0.82_525.9026m/z	525.9026455	0.819566667	neg	2469.743611	4887.782488	4582.08429	11637.45258	7363.777684	7636.887263	7508.886101	6681.795502	6061.36503
0.82_526.0154m/z	526.0153625	0.819566667	neg	3069.956633	3920.183723	4085.80876	5887.163087	2272.854246	2857.484833	4086.919111	3941.519455	1526.868889
0.82_547.8852m/z	547.8852291	0.819566667	neg	17142.88811	21633.32396	20600.05815	29150.04881	31993.16874	31162.48376	22251.7176	28622.64807	25886.84834
0.82_551.9047m/z	551.9047067	0.819566667	neg	2320.908937	3627.429612	4030.816743	9095.493387	8091.936794	10482.55207	5663.510999	7387.246773	5708.057238
0.82_559.0307m/z	559.0306931	0.819566667	neg	13837.67949	16991.77461	18778.21944	8399.968715	2629.322965	4746.253732	14487.24625	12422.38105	5788.799063
0.82_581.0080m/z	581.0080436	0.819566667	neg	8236.092316	9247.000521	6877.214972	1130.747526	0.001403199	0.001403199	6953.43071	4559.607715	5872.743003
0.82_594.7299m/z	594.7299311	0.819566667	neg	650.5861333	0.001403199	0.001403199	457.3201597	1766.692837	0.001403199	1785.782394	538.6279602	2176.44131
0.82_645.8611m/z	645.8610961	0.819566667	neg	4820.985509	4130.174713	4579.17617	15536.2415	14792.43841	17835.32009	7640.594474	11071.21636	6301.760907
0.82_678.9754m/z	678.9753922	0.819566667	neg	7629.742142	9400.179381	11510.16793	2582.686864	0.001403199	0.001403199	9278.317293	4552.43868	6963.830439
0.82_721.0793m/z	721.0793243	0.819566667	neg	10835.25058	11390.56658	13256.30868	12173.79958	15630.55069	13751.5385	4548.839598	4820.852248	6747.334575
0.84_152.9864m/z	152.9863547	0.841733333	neg	2770.89408	2670.901239	3315.482143	2718.277138	2530.232417	2728.29274	3425.983637	3856.524931	3498.220621
0.84_158.9116m/z	158.9115531	0.841733333	neg	379.160459	666.4465344	625.2866988	126.1552465	175.5226028	109.1068344	313.686183	485.3472813	284.6663162
0.84_158.9503m/z	158.9503202	0.841733333	neg	535.2584248	422.2573898	380.5769412	634.7131331	214.1477	382.8528923	680.223739	524.5981739	287.0854116
0.84_159.8601m/z	159.8600956	0.841733333	neg	2164.456805	2072.721949	1951.157908	466.0299197	565.6522393	769.9380382	2169.980313	1915.801188	1686.523846
0.84_176.9170m/z	176.9169915	0.841733333	neg	1050.640569	1528.627124	1486.332129	4820.828259	4338.605437	4812.226481	2422.189778	2269.606729	2303.765205
0.84_180.8596m/z	180.8595601	0.841733333	neg	2687.512125	2802.052763	2782.403377	1650.293975	2431.331933	2373.517089	3071.896273	3191.41919	2435.974156
0.84_182.8613m/z	182.861279	0.841733333	neg	491.3520099	691.365569	667.8316517	286.2119294	475.3196179	349.6092081	317.9044548	369.6806854	598.2713621
0.84_185.9023m/z	185.9022989	0.841733333	neg	0.001403199	91.11194876	68.75411798	1192.614587	991.1750987	697.8604099	147.2968055	204.6374781	169.2041792
0.84_186.8561m/z	186.8561443	0.841733333	neg	91308.87091	78363.97983	75155.70065	61560.28684	62222.39223	61305.14813	88784.57199	79895.25695	79657.48839
0.84_189.4946m/z	189.4946407	0.841733333	neg	4133.776881	3824.548861	4522.199918	4958.94844	4216.744318	4211.701021	4163.253941	4577.489252	4396.689156
0.84_219.8666m/z	219.8666221	0.841733333	neg	5911.757509	4866.166607	4374.760063	1872.90787	1868.602386	2080.503972	4214.21908	4670.823635	4260.705512
0.84_221.8250m/z	221.8249654	0.841733333	neg	7244.234639	7549.500584	7146.063399	3950.933965	4508.282315	4086.148855	8031.485646	7845.914956	7303.081582
0.84_221.8637m/z	221.8636958	0.841733333	neg	5376.884109	5512.821029	4795.829739	1926.904225	1239.477013	2092.280383	4773.762015	4441.454559	4158.296057
0.84_222.0442m/z	222.0442481	0.841733333	neg	2267.871439	1967.781427	1681.97889	2038.007911	870.4691839	1243.647924	773.4905553	932.3980079	940.6958716
0.84_223.8607m/z	223.8607202	0.841733333	neg	1287.116513	1558.925807	1127.604751	155.8864404	123.1572364	289.0995741	1330.410361	1077.238782	837.017414
0.84_236.0235m/z	236.023525	0.841733333	neg	8595.821695	8867.840651	11280.12346	8107.209177	8824.699504	8340.062125	7811.001393	7932.614405	8661.913978
0.84_242.0519m/z	242.0519208	0.841733333	neg	1270.426374	1237.622348	947.1669149	1152.610293	308.3245452	675.6851868	1059.471781	584.2102687	0.001403199
0.84_251.9797m/z	251.9796505	0.841733333	neg	1579.303136	1334.120958	1981.353854	1429.283532	879.4207612	1649.100182	1712.114555	1926.060196	842.7766743
0.84_252.9428m/z	252.9427825	0.841733333	neg	381.8119441	316.3350114	790.702897	1300.506156	1127.502636	756.4546073	369.4403847	308.9260674	775.7929009
0.84_258.0748m/z	258.0747991	0.841733333	neg	8219.008227	8493.675352	9484.741261	8649.049704	8056.028959	8430.18947	8356.204747	9018.325638	8892.426335
0.84_260.0721m/z	260.0721226	0.841733333	neg	4048.677572	3741.101749	5267.942358	2960.035239	3709.53759	4973.903718	3665.168095	4123.261144	4527.876391
0.84_268.9950m/z	268.995003	0.841733333	neg	14150.66334	13321.237	14554.56645	9795.859988	9806.307839	10944.19672	11608.47734	12526.74749	13528.74804
0.84_279.0192m/z	279.0191931	0.841733333	neg	0.001403199	0.001403199	0.001403199	326.8949953	202.2527027	578.1408973	0.001403199	195.2485733	211.1866067
0.84_283.8158m/z	283.8157663	0.841733333	neg	5624.899314	5579.512262	5380.681764	4673.530555	4555.090893	4616.038844	6754.57306	6152.224252	7064.521925
0.84_284.8237m/z	284.8236904	0.841733333	neg	27564.58436	23078.71198	23783.34613	22465.67645	22733.00954	22846.03425	28222.19916	26329.13583	25279.16766
0.84_285.0833m/z	285.0833329	0.841733333	neg	0.001403199	520.0551145	284.733629	278.22746	0.001403199	62.71408795	0.001403199	702.3753546	537.0048374
0.84_285.8129m/z	285.8129062	0.841733333	neg	1351.128559	1510.822309	777.7762682	624.3491061	682.4746673	1119.979648	1348.315806	1380.822171	799.5594093
0.84_286.8296m/z	286.8296328	0.841733333	neg	1640.032731	120.329097	764.6671514	3346.688218	3874.42373	4836.084644	868.1669886	1577.17281	1620.90879
0.84_290.0996m/z	290.0995593	0.841733333	neg	780.2378437	1015.881138	1275.910667	1984.175398	597.222294	1834.352113	601.8810121	633.8729361	0.001403199
0.84_292.0723m/z	292.072348	0.841733333	neg	16068.58891	12742.41332	15716.99307	11192.84641	14469.0754	11260.94653	16096.87476	15762.76262	16740.71381
0.84_296.8502m/z	296.8501521	0.841733333	neg	30650.33442	29770.48294	29229.30477	13059.23483	14111.8393	14538.21906	33582.3833	30852.97847	31330.04693
0.84_305.7978m/z	305.7977767	0.841733333	neg	13348.79402	12697.39595	11624.59301	7601.906019	7988.417336	7993.535936	14230.88164	13579.57234	13689.03886
0.84_321.0634m/z	321.063373	0.841733333	neg	9033.333182	10365.80403	10471.2525	7717.909633	10110.47137	8062.280406	11521.86765	10940.24918	9081.120537
0.84_331.0092m/z	331.0091823	0.841733333	neg	1047.617742	980.5372313	1325.651104	7661.028584	6371.745497	7187.717106	1946.204976	1714.247498	1434.722025
0.84_333.9910m/z	333.9909983	0.841733333	neg	3387.65725	3653.938677	4228.910574	4158.018965	3510.296026	3119.518031	2879.970724	3205.550346	3530.649821
0.84_334.0464m/z	334.0463939	0.841733333	neg	4847.62896	5526.998045	5615.866779	9238.989322	6898.391544	9486.54007	5822.988308	4670.815635	4512.377436
0.84_334.0831m/z	334.0830749	0.841733333	neg	1796.099262	1978.220873	1638.651662	2387.928682	213.1850162	1184.006036	2930.213814	1130.037264	1087.68937
0.84_336.8591m/z	336.8591084	0.841733333	neg	514179.4564	561028.7429	584449.4176	816473.3993	840948.4449	823735.0284	709721.4441	741504.2063	756007.9845
0.84_355.9730m/z	355.9730009	0.841733333	neg	2355.845369	2342.443403	2623.860431	2042.907432	1405.220503	2206.631979	2504.615391	2111.181499	1319.165932
0.84_359.0766m/z	359.0766325	0.841733333	neg	1510.160228	2218.060384	1762.924261	398.3292653	0.001403199	291.8832828	487.9882837	772.2335348	300.8922341
0.84_365.0531m/z	365.0530682	0.841733333	neg	8128.088101	10032.77313	10388.84497	11875.96474	11830.07508	11986.71626	9596.588842	9886.448646	11670.97973
0.84_367.1146n	366.1094935	0.841733333	neg	5141.825084	4732.317298	4849.226654	4298.818669	4291.24663	4126.041489	5154.080469	3054.869484	3315.310626
0.84_379.0693m/z	379.0693035	0.841733333	neg	5272.250028	6389.899477	6381.679475	17032.38055	12066.84728	13908.85884	7279.622279	8219.617658	6419.483519
0.84_394.0674m/z	394.067434	0.841733333	neg	2016.283553	1898.405474	3032.315999	6764.852629	6297.340455	7431.869619	3698.832852	3672.140356	4156.297574
0.84_412.8458m/z	412.8457732	0.841733333	neg	2433.315498	2742.010718	3485.757971	8302.588986	6374.529336	7106.894723	3921.573794	4297.254768	5205.216773
0.84_419.9889m/z	419.9888795	0.841733333	neg	5801.670894	6622.775463	6210.583227	7842.561779	6421.057675	7143.335092	6112.643164	6869.541857	5453.040313
0.84_434.8250m/z	434.8249653	0.841733333	neg	33997.2112	38326.19659	42652.64227	65759.31856	56949.28189	61759.10796	55500.6711	56635.82521	51202.27488
0.84_443.7838m/z	443.7838	0.841733333	neg	1490.051349	0.001403199	422.8873242	4547.376857	5755.642379	4250.563958	1595.459165	1590.777232	1776.412666
0.84_450.8006m/z	450.800568	0.841733333	neg	1175.307558	838.8308956	2293.296728	2638.196762	1892.731337	2338.692851	1459.570036	2326.207214	2185.906182
0.84_456.8076m/z	456.8075505	0.841733333	neg	211684.14	241766.6126	239510.7425	368259.117	358127.3793	354452.3326	304579.6523	334824.0443	325126.9684
0.84_458.8029m/z	458.8029487	0.841733333	neg	35031.45763	42043.84122	40497.41488	60019.24191	59325.43215	58061.6532	50469.59262	56383.15552	55435.81185
0.84_474.0858m/z	474.0857955	0.841733333	neg	5026.622752	2411.470068	2674.135654	3750.630515	2638.833277	3356.184469	2321.322835	3495.964054	2779.480167
0.84_481.0396m/z	481.0396483	0.841733333	neg	899.0201397	445.9172136	807.8967689	0.001403199	0.001403199	0.001403199	1370.219603	330.2583466	530.1623152
0.84_494.7642m/z	494.7641638	0.841733333	neg	2277.857009	2128.426373	1219.095821	883.5343252	353.2692691	623.7962502	2263.006929	4063.742769	2788.347397
0.84_549.1675m/z	549.1675223	0.841733333	neg	2353.494022	1561.984114	2290.12683	3420.691121	2637.807933	5082.995423	0.001403199	0.001403199	1573.54589
0.84_585.7137m/z	585.7136807	0.841733333	neg	438.7368905	721.6304489	1130.105771	2825.212619	3106.440954	4406.983896	2345.703173	1463.160399	1072.347257
0.84_592.7315m/z	592.7315333	0.841733333	neg	7106.794852	6995.506073	8811.061197	10893.75093	8700.216444	10140.53809	13113.298	12259.41416	9166.751383
0.84_599.1943m/z	599.194286	0.841733333	neg	3646.675977	3353.627025	4132.70713	4176.473487	2561.230069	3062.866684	3123.132979	1901.208191	1504.246837
0.84_626.9572m/z	626.9571805	0.841733333	neg	979.6985663	1430.668481	696.5394593	705.1278371	0.001403199	602.3597837	2270.509045	3434.464294	715.2242638
0.84_659.0885m/z	659.088457	0.841733333	neg	2623.652667	2913.428411	2881.607482	686.6623767	0.001403199	1083.808667	957.7512925	1039.002014	0.001403199
0.84_667.8348m/z	667.8348463	0.841733333	neg	10676.74092	10543.22321	9990.446234	11151.23943	10828.3861	11636.28673	11796.19767	11692.75388	10713.45423
0.84_697.1621m/z	697.1620811	0.841733333	neg	803.5763788	1909.522257	3290.856029	3205.655244	1543.038346	3127.24507	907.7442855	2133.501007	1119.141458
0.84_702.9435m/z	702.9435099	0.841733333	neg	3130.488166	2586.976107	4226.607815	4070.574824	6199.287821	5515.036698	3803.580722	6418.012688	4087.294417
0.84_763.1824m/z	763.1823534	0.841733333	neg	11292.66703	12982.93536	16227.72271	21619.88515	22568.83318	25658.30968	1833.879834	4045.043365	7830.843605
0.84_787.7840m/z	787.7840429	0.841733333	neg	1914.334196	1961.138379	5093.705952	6073.882294	4827.415976	6522.492576	6045.066359	6427.486653	3842.285896
0.84_883.1329m/z	883.1329071	0.841733333	neg	1060.392864	990.3282484	3446.172036	2593.856787	3325.887969	5561.665449	0.001403199	0.001403199	0.001403199
0.84_891.1479m/z	891.1478919	0.841733333	neg	2677.336761	3469.535438	3336.211613	4006.169691	4194.183203	6427.586577	3439.473075	5615.773506	2792.366052
0.86_119.9470m/z	119.947002	0.86335	neg	218.9848977	113.9638368	149.5034203	101.2313917	106.2138994	106.0088235	311.1577635	186.4463062	278.1436654
0.86_154.9657m/z	154.9657103	0.86335	neg	370.6151702	475.3228257	597.6854095	890.6088538	611.1450413	825.7986608	723.2038108	648.1193643	426.0266494
0.86_166.8332m/z	166.8331892	0.86335	neg	8410.459832	8417.785251	9119.952352	3200.144265	3111.658834	3296.111717	10312.79227	7422.201562	6740.153355
0.86_168.0138m/z	168.0137946	0.86335	neg	443.4339414	672.4704386	839.3805202	1071.753952	744.1052514	736.7900454	482.4452438	617.7659217	559.1052355
0.86_184.8608m/z	184.8607901	0.86335	neg	1982.756408	1398.599484	1347.159642	878.6302241	900.15613	1303.912109	2224.974906	1825.714571	1528.381052
0.86_186.0521m/z	186.0521133	0.86335	neg	1857.54081	1520.67869	1663.680499	1483.433077	1891.985345	1919.228178	1911.347704	2081.591946	1799.618571
0.86_188.8531m/z	188.8531398	0.86335	neg	38816.05577	33015.04078	30535.02903	23470.15446	24617.83293	24256.42337	36866.31243	32900.21879	32601.06883
0.86_207.0042m/z	207.0042339	0.86335	neg	2693.616571	2117.537258	2838.583921	595.3918664	615.0386971	1127.740713	1585.842205	1937.012419	1445.095083
0.86_210.0079m/z	210.0078919	0.86335	neg	420.8376168	525.4921921	536.4721596	1044.375821	0.001403199	184.9740341	576.3815934	413.5020727	116.9446745

0.86_220.9011m/z	220.9010515	0.86335	neg	534.0463535	315.9794451	659.088719	1423.259212	656.8465978	1133.675386	936.4102052	846.7340259	502.2250344
0.86_237.0147m/z	237.0146861	0.86335	neg	1359.705169	1146.292474	1225.85242	324.0419623	272.9719344	128.4480961	466.1549264	1087.364779	738.1602489
0.86_244.8148m/z	244.8147636	0.86335	neg	6700.862467	6106.845367	5163.152761	975.5197621	1559.578417	2074.818008	5398.916488	5501.605717	5054.087791
0.86_246.8116m/z	246.8115618	0.86335	neg	3277.251286	2652.145997	2169.106661	1002.644679	791.5364196	1386.932616	3126.985728	3068.371449	2536.317887
0.86_247.0131m/z	247.0131186	0.86335	neg	2336.557022	2366.138318	2285.876673	2992.640754	2013.259339	2923.000662	1804.801968	2353.057803	1613.0649
0.86_247.0270m/z	247.0270321	0.86335	neg	415.2296712	732.0234382	1608.387241	1158.630569	1964.595889	1757.189936	1921.495735	1994.411077	1904.767787
0.86_247.8390m/z	247.8389834	0.86335	neg	20450.91496	21145.75368	20306.76971	28513.24128	28394.7445	29333.17881	26110.19475	25191.77171	25701.36029
0.86_256.9860m/z	256.9859746	0.86335	neg	2061.122209	2253.217969	2173.469308	6771.044336	6475.923242	6820.745348	2691.290831	3946.675188	3531.815966
0.86_257.8017m/z	257.8016768	0.86335	neg	766.3984713	1760.886642	479.3642084	0.001403199	248.1884022	0.001403199	1773.390479	1654.797524	1019.20709
0.86_259.7989m/z	259.7989154	0.86335	neg	2055.284039	1446.74874	1200.949247	229.4861949	762.2365139	664.978546	2291.630502	1416.47109	866.5026644
0.86_270.9564m/z	270.9563754	0.86335	neg	2816.626437	2654.406734	2223.708522	2513.176015	2591.55922	2240.193181	4990.554723	4674.654446	5258.776639
0.86_275.8720m/z	275.8720101	0.86335	neg	2414.127962	1365.89625	1797.709338	524.8852911	501.7953933	993.7479818	4045.205742	2626.44483	2068.75294
0.86_278.8609m/z	278.860942	0.86335	neg	382.9453635	372.6715111	588.7417826	0.001403199	137.2817109	0.001403199	541.0185394	352.3661499	362.6176057
0.86_284.0195m/z	284.0195273	0.86335	neg	4238.974313	4197.043289	4471.306536	6349.640222	6897.094542	6758.448104	4428.970056	5556.656459	6076.729504
0.86_291.0531m/z	291.0531057	0.86335	neg	1326.305339	1364.262291	1057.168623	2548.40008	1096.627837	1321.754781	967.5837527	1560.837379	1061.735447
0.86_296.0339m/z	296.0338848	0.86335	neg	167.6230043	0.001403199	158.7062593	0.001403199	289.3507206	0.001403199	0.001403199	0.001403199	336.7632707
0.86_296.9899m/z	296.9899144	0.86335	neg	3955.313044	4396.011295	4226.054773	3218.414927	2607.162432	2946.11059	3750.291307	3317.411282	2282.957169
0.86_297.8256m/z	297.8256245	0.86335	neg	3157.117798	2539.624725	2192.028556	931.6972627	1140.501267	1273.712008	2853.147167	2080.010751	2504.017346
0.86_299.0056m/z	299.0055573	0.86335	neg	8678.403644	8061.945341	8454.265029	4982.801351	4508.009264	5839.769057	7406.3335	8736.935077	6349.430675
0.86_303.0029m/z	303.0028659	0.86335	neg	712.291998	747.9424256	1177.372981	2370.63747	1270.44556	2230.840678	1473.990536	1740.333432	970.7883184
0.86_306.0016m/z	306.0015775	0.86335	neg	1391.243991	696.761358	988.9610595	1399.884962	575.0857963	1636.21268	1502.85881	1246.436417	433.7002092
0.86_306.0881m/z	306.08806	0.86335	neg	3112.08901	3227.422406	3157.085826	3164.396349	3225.184331	3453.75338	4064.277152	4156.751312	3220.731264
0.86_307.7946m/z	307.7946441	0.86335	neg	3758.563548	4113.334435	3754.892681	1674.196379	2136.224647	1663.54992	4885.224007	3364.583027	4502.658486
0.86_308.8026m/z	308.8025589	0.86335	neg	8212.399339	6649.358232	6852.688639	4412.62662	4235.134009	4530.315981	8198.419195	7666.259345	6836.632526
0.86_309.9675m/z	309.9674601	0.86335	neg	1444.50653	1912.442602	1711.57394	2074.478217	1831.42213	1297.856079	2580.501972	2520.627451	2724.227438
0.86_311.0063m/z	311.0062538	0.86335	neg	1452.542272	1918.564245	1932.030399	5234.380796	1242.529681	1700.380662	3251.867095	1977.66095	1641.445489
0.86_320.8288m/z	320.8288079	0.86335	neg	7751.886953	6841.69377	8563.637492	3078.825318	3719.363562	3609.457053	8214.492742	7926.844792	8042.215842
0.86_321.0999m/z	321.0998966	0.86335	neg	2807.911496	1975.158877	1648.472524	1727.936681	1514.018752	1519.411248	1497.137174	2068.51313	1720.292882
0.86_344.0569m/z	344.056883	0.86335	neg	2351.458308	2257.98013	2362.234518	2641.035645	522.4995608	1757.77483	2631.216312	2796.68955	840.2267013
0.86_344.8190m/z	344.8190449	0.86335	neg	272.2574615	1296.73077	754.4608535	4188.718445	4062.921202	4876.041398	1684.360067	1839.439566	1867.41948
0.86_349.0586m/z	349.0585779	0.86335	neg	3995.843983	4143.794772	5152.423026	13605.03049	9733.044476	13684.9899	6135.553167	7972.512362	6646.085381
0.86_365.0776m/z	365.0775537	0.86335	neg	25447.24513	24327.74978	27340.76414	24757.20327	26699.96815	28037.4936	26815.59108	28360.20952	26303.79712
0.86_374.9303m/z	374.9303288	0.86335	neg	12828.94633	12641.42957	12399.35005	8870.927563	8908.543434	10570.84655	11881.28407	11494.41121	9661.755356
0.86_376.1187m/z	376.1186646	0.86335	neg	2281.929375	2556.576106	2490.201106	1697.087714	1366.688344	2234.512862	2519.027338	2693.332195	1885.570326
0.86_387.0594m/z	387.0594318	0.86335	neg	7772.934065	6963.493277	4530.869853	5214.993815	657.0821007	1830.289544	5245.967871	3044.627372	1246.160547
0.86_388.0558m/z	388.0558008	0.86335	neg	54110.21559	50489.93203	64308.02828	75578.54663	76904.10903	73229.87297	60585.5033	60409.40546	61620.2533
0.86_394.8172m/z	394.8172242	0.86335	neg	2739.537	1992.56199	2668.070213	835.2265624	273.9049822	840.8295166	4201.282149	2335.491714	1283.81645
0.86_410.0375m/z	410.0374533	0.86335	neg	6796.265844	5808.666219	6744.684471	4189.580403	2317.505631	4437.846669	4933.54127	4542.763037	2738.293525
0.86_441.0206m/z	441.0205571	0.86335	neg	5353.115841	4399.404848	6889.9135	6287.05813	7179.322162	8038.119525	5990.539399	4959.327296	4971.732862
0.86_449.0246m/z	449.0246121	0.86335	neg	3586.982245	3867.130666	4083.168105	12065.39244	12424.23643	11506.09588	4486.430306	4396.841705	4870.760395
0.86_471.0068m/z	471.0068004	0.86335	neg	808.6806112	1003.182582	1773.297977	3012.288758	2443.74075	1647.700648	1048.578858	700.1299133	1106.379851
0.86_474.7790m/z	474.7789659	0.86335	neg	1067.102315	1128.461156	414.177219	2868.195986	1493.636186	2876.405191	2873.116636	3681.366209	1752.390988
0.86_476.0716m/z	476.0715894	0.86335	neg	484.8339796	384.6581082	0.001403199	1436.960244	948.8554259	423.8364506	0.001403199	667.145355	277.9809088
0.86_507.0081m/z	507.0080686	0.86335	neg	4222.485706	3659.572664	3611.836422	2618.517834	1069.774066	1564.246418	3451.996883	4154.30365	1143.555177
0.86_582.9942m/z	582.9941503	0.86335	neg	3102.35087	2844.34438	3583.560558	4776.857945	1575.661809	5261.705066	3460.016854	5559.863937	1430.51819
0.86_674.7348m/z	674.7347528	0.86335	neg	1326.699395	0.001403199	1751.736735	4481.492493	6772.782614	3655.940951	3781.577702	2890.069107	2958.445398
0.88_122.8942m/z	122.8941941	0.884633333	neg	2593.690097	2392.516371	2332.241928	685.915682	1275.344355	992.8080139	2793.502233	2353.573806	2196.291964
0.88_126.9051m/z	126.9050655	0.884633333	neg	3134.812237	3882.27955	4585.572101	3407.598331	1981.889045	3145.076309	4510.899898	4921.470267	2280.027821
0.88_127.8702m/z	127.8702475	0.884633333	neg	3724.22074	3812.632893	3663.409151	933.010232	1250.313765	1400.537171	4214.112346	3145.009749	2960.030207
0.88_175.0361m/z	175.0360589	0.884633333	neg	1184.303331	1405.52927	824.9286102	844.5422054	659.3476169	671.3543152	1009.27939	1404.698984	1300.464076
0.88_199.8737m/z	199.8737065	0.884633333	neg	1292.731253	1047.196841	1033.119947	85.39246956	0.001403199	0.001403199	601.9354338	555.292564	374.6607659
0.88_200.9358m/z	200.93577	0.884633333	neg	157.4598242	95.45320739	311.4805368	430.6120805	353.378721	512.4361186	449.0729922	397.3792184	294.2257126
0.88_231.8538m/z	231.8537897	0.884633333	neg	1800.993994	1654.98459	1510.641542	928.6151457	554.4250628	1206.21507	1931.860041	1668.064449	1263.563492
0.88_232.0512m/z	232.0511666	0.884633333	neg	1545.941689	1809.547356	2230.081668	1344.584794	1700.209656	836.5331375	1338.319977	1353.977203	1378.504794
0.88_240.0008m/z	240.0007758	0.884633333	neg	13438.83388	14028.75348	15776.58754	12040.10324	13466.52524	13179.80084	14569.66065	14431.43829	13828.21744
0.88_241.9972m/z	241.9971848	0.884633333	neg	1495.763639	1766.723239	1821.100209	1657.318698	1492.454612	2072.232579	1779.847161	1698.133839	1846.076925
0.88_254.0341m/z	254.0341375	0.884633333	neg	1804.003376	1600.081132	1816.411756	1703.869115	1146.166697	1322.027955	1475.504809	1638.1651	1476.521918
0.88_261.0425m/z	261.0424644	0.884633333	neg	2020.765734	2496.304786	2981.959574	4733.910455	3666.827547	4011.762122	2568.477792	2986.211673	3544.551096
0.88_272.0889m/z	272.0889248	0.884633333	neg	0.001403199	0.001403199	0.001403199	0.001403199	118.1386492	165.993667	139.065476	391.9344609	107.4881903
0.88_273.9104m/z	273.9104487	0.884633333	neg	3012.640382	2227.228906	2233.516655	291.1012285	0.001403199	386.9283887	1881.977405	1189.986049	1547.055847
0.88_275.9074m/z	275.9074422	0.884633333	neg	1317.221521	1680.060752	1286.92565	0.001403199	0.001403199	4.881977631	526.7966804	655.215493	11.64826301
0.88_276.1088m/z	276.1088156	0.884633333	neg	1915.699014	1886.486451	1930.341202	1018.131382	1751.466124	1181.403596	1546.694776	1937.561274	2629.708821
0.88_282.0581m/z	282.0581209	0.884633333	neg	617.2634445	467.9494711	980.5969656	509.5493044	440.3537836	602.2198407	786.4694352	479.3752551	610.9929028
0.88_283.0517m/z	283.0517352	0.884633333	neg	3446.831721	3267.22054	3304.365029	2433.990063	2582.911402	1810.787529	4165.024089	2915.509319	3252.172282
0.88_285.9674m/z	285.9674056	0.884633333	neg	2205.94094	1931.11159	2060.687062	1529.015039	1563.040192	1796.500102	1983.735569	1683.067253	1690.405521
0.88_305.0687m/z	305.0687454	0.884633333	neg	1104.591792	2050.241951	2489.820787	4994.2509	4005.589334	4142.348359	1694.27333	3029.164017	2462.879935
0.88_311.9436m/z	311.9436285	0.884633333	neg	10149.38476	9661.584177	9977.506647	9985.784419	7943.733232	11599.47069	12092.52683	11999.80324	13562.48259
0.88_316.7392m/z	316.7391805	0.884633333	neg	968.6606507	1132.817287	433.0565521	399.2299024	0.001403199	0.001403199	649.3970744	924.1812009	606.7884044
0.88_326.9639m/z	326.9638646	0.884633333	neg	1199.90744	2199.003243	2002.519674	1937.286279	2782.882828	1734.307709	1910.353326	1240.158636	1198.6496
0.88_329.8599m/z	329.8599009	0.884633333	neg	696.0941024	498.4839704	0.001403199	0.001403199	0.001403199	0.001403199	358.2068428	0.001403199	625.8798681
0.88_337.9683m/z	337.9682987	0.884633333	neg	7117.49781	9105.835772	10678.03253	10037.39136	10688.2724	11817.59529	10420.13751	10847.34611	9046.961345
0.88_340.8506m/z	340.8505549	0.884633333	neg	1133.275365	1001.71159	855.0800953	1808.780839	1105.037787	1753.109444	1216.179226	950.0067854	841.1404442
0.88_353.9316m/z	353.9315943	0.884633333	neg	913.1044871	528.0930012	746.8603464	376.5043054	332.7160826	668.9932796	1137.029032	1093.002047	1702.036299
0.88_357.0317m/z	357.0317362	0.884633333	neg	14087.04899	15089.06185	21651.56659	9090.273471	21091.37585	10973.76692	22016.36382	14304.26941	23391.86528
0.88_359.9501m/z	359.9501343	0.884633333	neg	6205.261141	5330.805671	5243.413326	3747.926788	5698.742397	5578.220581	5804.979777	5478.494961	4947.089877
0.88_363.0119m/z	363.0119347	0.884633333	neg	47719.57867	49878.99033	47380.05824	50379.9328	45077.27624	54691.89391	47656.27706	45144.72486	47993.1115
0.88_364.0573m/z	364.0572688	0.884633333	neg	4097.201196	3690.942783	4922.649328	4873.286006	4043.293085	4901.922015	4346.740897	2684.109741	4365.063091
0.88_373.0389m/z	373.0389417	0.884633333	neg	270.6212976	515.4037069	271.1685662	888.1295233	751.7749933	1072.186436	507.150813	323.392395	569.0354503
0.88_378.0725m/z	378.0724797	0.884633333	neg	974.1701396	1237.049847	1136.394275	2609.772675	1796.133909	3224.160179	566.0845725	1205.113683	915.0174757
0.88_379.9966m/z	379.9966011	0.884633333	neg	1044.143159	1346.01672	1739.796035	1524.428682	1998.917514	2067.432619	1512.611591	1127.988838	1307.054637
0.88_396.9121m/z	396.9121286	0.884633333	neg	828.9662957	1090.814335	514.0684369	688.482017	525.1885957	178.0215861	1150.961176	591.4613724	887.9105273
0.88_402.0105m/z	402.0105144	0.884633333	neg	2835.966939	2312.60153	4563.495397	1284.731119	1793.028573	1945.248006	3455.140862	2775.288666	5266.07856
0.88_409.9107m/z	409.9106769	0.884633333	neg	3254.990746	2150.995369	3025.1299	3389.529748	1417.054981	2409.377649	3059.732571	3067.950499	3103.033578
0.88_433.0165m/z	433.0164947	0.884633333	neg	1678.843321	2340.533254	3097.753792	3064.684311	7213.95273	4548.281793	4850.418974	3907.84318	3484.472335
0.88_467.0912m/z	467.0912446	0.884633333	neg	959.5519217	365.4866366	924.6765169	491.2221911	998.193084	0.001403199	702.6136452	562.4327393	1355.969705
0.88_552.9657m/z	552.965653	0.884633333	neg	4530.74718	5154.178427	9504.593314	9080.160455	11572.87731	6715.515315	9209.752194	8477.019185	8613.208502
0.88_623.0277m/z	623.0277198	0.884633333	neg	2114.783032	1556.919714	6398.500342	1982.615436	6800.791035	3266.819338	3301.808278	3857.16272	5245.652275
0.90_124.0150m/z	124.0149577	0.904683333	neg	253.1526555	376.136999	149.0135578	433.7359504	106.8537814	126.6139692	235.8784298	79.51469421	46.78287268
0.90_124.8913m/z	124.8912618	0.904683333	neg	440.3315377	281.9835024	284.5570227	0.001403199	0.001403199	30.0760692	460.642809	235.8210563	328.850979
0.90_177.8448m/z	177.844808	0.904683333	neg	3625.899027	3216.438668	3105.591213	762.9597976	619.8442558	768.260383	4574.017117	3154.16018	2821.505568
0.90_179.8418m/z	179.8418188	0.904683333	neg	4067.150005	3051.158524	2757.06682	678.4396093	396.2214155	300.7304008	3522.286305	2541.146839	2604.764573
0.90_201.8708m/z	201.8707987	0.904683333	neg	752.3723102	576.0551523	395.6669402	0.001403199	0.001403199	0.001403199	379.9599031	88.34134251	150.4861605
0.90_202.9979m/z	202.9978957	0.904683333	neg	666.8191865	677.581847	274.6706647	460.2262892	0.001403199	55.42113399	419.0573192	155.3311568	57.9164537
0.90_204.0337m/z	204.0337133	0.904683333	neg	1572.491095	1829.437353	1266.703707	1940.301923	1652.017115	2008.608394	1364.128976	1822.380012	1116.756291
0.90_205.8398m/z	205.8397683	0.904683333	neg	12147.17567	10624.46044	9791.300657	2523.726093	3371.598812	3036.82186	12829.16782	10134.25239	11115.22941
0.90_207.8368m/z	207.8368308	0.904683333	neg	10940.52471	9190.621492	7681.079645	1777.35445	2847.369268	2315.583303	10263.07127	7553.300415	8748.135286
0.90_208.9760m/z	208.9759835	0.904683333	neg	14891.44862	17198.10684	18048.72889	18112.0453	20181.92949	18269.66322	18341.64534	17780.02899	19496.41442
0.90_209.8339m/z	209.8339342	0.904683333	neg	3714.399952	3249.407113	2921.770103	495.2851134	692.9107112	673.2410886	3946.792815	2831.859419	3045.580808
0.90_224.8036m/z	224.803638	0.904683333	neg	3630.1988	3627.525607	3407.968353	2356.612422	760.3049182	1805.938709	3037.423864	3535.847816	1830.867341
0.90_224.9798m/z	224.9797889	0.904683333	neg	10875.02756	10597.2019	9981.745814	4327.604549	5120.894693	5147.542283	11734.32649	8462.693844	10124.56609
0.90_226.8014m/z	226.8013669	0.904683333	neg	2412.459279	2646.926103	2251.64111	1193.359907	556.6436587	1110.428284	2795.921954	2015.776955	1660.927175
0.90_226.9769m/z	226.9768812	0.904683333	neg	3184.697813	3165.184875	3310.75842	1092.056263	1541.084877	1390.426009	3185.003438	2092.435684	3657.889409
0.90_230.0168m/z	230.0167684	0.904683333	neg	1351.022301	1743.338361	1573.32177	1643.313207	2011.795584	1666.821794	1528.402243	1384.533549	1381.663232
0.90_231.9414m/z	231.9414064	0.904683333	neg	1936.788083	1934.993887	2028.905805	2307.27167	1697.690087	2736.608618	2230.02851	2762.629418	1568.41219
0.90_252.0185m/z	252.0184886	0.904683333	neg	2171.005292	2804.906918	2616.799169	4159.246152	4523.525033	3740.032911	2386.198188	3820.541675	3652.037212
0.90_257.0167m/z	257.0166508	0.904683333	neg	2842.478187	2900.262456	2336.893394	2851.794407	1991.706093	2966.965349	2450.369715	2281.077221	1450.495374
0.90_263.9797m/z	263.9796579	0.904683333	neg	7855.331543	7397.139968	6995.479398	6237.053719	5025.598002	6585.926356	7015.346665	7251.134807	5709.975829
0.90_264.9885m/z	264.9884992	0.904683333	neg	236083.6677	275207.0243	276795.3925	425589.9096	436732.4046	397964.4165	298762.7281	280653.163	327835.2206
0.90_266.9464m/z	266.946413	0.904683333	neg	3847.050601	3599.778111	3455.743589	3464.558468	1362.854857	3001.478009	4050.667112	2747.017549	1754.658849
0.90_266.9843m/z	266.9843136	0.904683333	neg	10984.54277	13953.00468	13390.97577	20783.6276	19536.86411	19213.46996	14855.02133	13485.81059	15561.40438

0.90_272.0585m/z	272.0584585	0.904683333	neg	1131.550622	1892.177139	1680.287494	4712.157142	1381.379912	2208.873897	1323.402253	1585.243896	471.7534161
0.90_275.0218m/z	275.0217803	0.904683333	neg	765.6863108	1817.365317	1575.143044	4537.310441	2871.618601	3495.067573	413.6728093	1953.663483	1050.498558
0.90_276.0773m/z	276.0772537	0.904683333	neg	708.7112312	946.4572265	802.5133582	2379.786544	997.7982082	2281.627832	531.2581281	1285.944703	487.2271878
0.90_279.9878m/z	279.9878383	0.904683333	neg	13679.64857	11368.1213	11370.48661	9506.09622	8757.258765	12346.97226	14684.49197	11321.65999	14359.03709
0.90_281.0272m/z	281.0271502	0.904683333	neg	1847.218854	2069.640409	1529.72541	972.7592817	535.4234695	741.4529755	1686.020335	1263.880774	554.4079413
0.90_288.0030m/z	288.0030366	0.904683333	neg	235.3326923	997.4075622	601.1865214	914.3486732	529.5061633	730.4270149	447.63675	144.6584483	266.6573385
0.90_294.8907m/z	294.8906999	0.904683333	neg	2266.615255	2266.981776	1665.746982	503.3846911	280.729235	766.9300465	2049.69113	1125.442059	424.6869919
0.90_302.9225m/z	302.9224767	0.904683333	neg	1415.828014	1236.610136	838.1702275	585.8793012	167.6940169	547.5411821	1263.897384	773.0133382	396.3672247
0.90_309.9600n	354.9579896	0.904683333	neg	8541.944239	8273.60022	8248.951883	7338.194498	6185.377312	8081.956721	9365.770434	7090.180231	5625.019568
0.90_317.8816m/z	317.8815842	0.904683333	neg	691.8908049	564.2491147	810.358008	0.001403199	170.4582336	0.001403199	145.8731343	871.8185043	269.7141994
0.90_319.9151n	364.9134466	0.904683333	neg	5342.75507	5539.519359	3519.351958	5642.474035	3700.242694	5304.858273	4343.239027	3464.06522	1725.104151
0.90_332.9248m/z	332.9247717	0.904683333	neg	3426.526879	3899.339037	3452.878948	4197.7568	4451.408398	4328.918043	4129.039104	4257.39851	4191.612613
0.90_344.9291m/z	344.9291431	0.904683333	neg	10893.25776	11877.25139	9035.941224	4051.50773	5396.476684	4288.725116	10021.94306	7464.392776	9878.03715
0.90_346.9260m/z	346.9259943	0.904683333	neg	1652.786317	2427.346067	1032.023377	702.8371331	137.2091682	0.001403199	1293.191025	671.2602067	184.3238267
0.90_348.0623m/z	348.0622813	0.904683333	neg	508.2399373	0.001403199	250.5385661	3153.709335	2422.478977	1898.698616	0.001403199	675.7178268	0.001403199
0.90_351.8614m/z	351.8613808	0.904683333	neg	147.9385526	1058.030732	925.5708061	163.6860822	0.001403199	311.4748639	280.2232598	155.9786758	329.3054767
0.90_353.8762m/z	353.8762115	0.904683333	neg	415.3690476	836.8674639	369.7150395	329.335489	0.001403199	0.001403199	222.8512025	190.56884	0.001403199
0.90_354.8848m/z	354.8848077	0.904683333	neg	7344.337848	6996.195324	6685.374335	3990.195803	1600.323403	3735.099084	6256.924394	5798.241535	1780.816065
0.90_366.9109m/z	366.9109305	0.904683333	neg	3391.690769	2942.12216	1757.801514	1023.701931	287.4501804	231.1343603	2282.266889	1197.690093	522.9431937
0.90_367.0275m/z	367.0274981	0.904683333	neg	5367.863976	6730.036761	7130.682142	6758.47271	7818.190217	6557.435057	5485.130827	6443.505924	6923.011374
0.90_376.8664m/z	376.866443	0.904683333	neg	2355.338393	2296.890535	1384.020387	1132.176856	156.3727155	371.8993614	1643.564176	600.1465225	380.2886519
0.90_381.0305m/z	381.0304704	0.904683333	neg	4428.415919	4400.456368	5091.781239	3539.731704	5119.04389	4218.864383	5159.448516	4072.602605	4949.890646
0.90_383.9289m/z	383.9289314	0.904683333	neg	2684.310306	2530.975537	1984.719666	1132.344096	1031.164848	1293.756596	2891.295301	1659.009605	1556.973449
0.90_406.9196m/z	406.919565	0.904683333	neg	45329.21057	48556.21017	44080.11022	45699.00171	53834.14089	48597.77422	47021.00693	41588.68248	50289.02325
0.90_411.9596m/z	411.9596321	0.904683333	neg	1362.551948	1741.939105	1842.092356	2801.177448	1879.544449	1874.586679	2062.139692	2189.814094	1297.887007
0.90_415.8670m/z	415.8669777	0.904683333	neg	3198.708343	2757.037114	3692.40158	4038.069311	1925.764629	3787.487359	3249.283697	2936.114447	1298.281188
0.90_416.8751m/z	416.8751055	0.904683333	neg	6458.002548	7278.657167	7848.547072	10836.83056	11292.95581	10581.84671	7626.782856	6565.505707	9014.198417
0.90_428.9014m/z	428.9013945	0.904683333	neg	13944.29063	14132.41864	11636.16834	10413.89286	12884.72835	12451.24069	14366.92197	11106.15759	13808.0609
0.90_437.8494m/z	437.8494071	0.904683333	neg	2602.000564	2146.968477	2592.587083	2612.443416	1343.081226	1473.83011	1675.646868	1618.369995	765.543019
0.90_438.8564m/z	438.8564274	0.904683333	neg	6321.846855	6427.331536	4192.198371	5146.143108	4977.83412	5508.698121	4646.467324	3825.530423	3592.840123
0.90_476.9803m/z	476.9802654	0.904683333	neg	4421.172631	5384.649116	7104.696556	3645.832963	1047.058495	3858.541577	6248.397947	6024.216999	1080.077972
0.90_504.8866m/z	504.8865716	0.904683333	neg	22405.7053	29737.89316	25302.19854	45008.19844	39131.12839	37351.8511	30019.69344	29721.74703	32496.06285
0.90_506.8824m/z	506.8824086	0.904683333	neg	2000.664922	2740.577911	2607.28796	4016.292495	2222.743169	2756.522448	2894.194906	4148.477081	1313.202145
0.90_548.8511m/z	548.8510781	0.904683333	neg	5459.800132	4714.834424	3003.279616	2259.066683	2680.347022	2556.244592	4611.641692	3550.154734	3735.483941
0.90_558.8072m/z	558.8071749	0.904683333	neg	2657.022935	3110.604541	753.2667023	1907.160891	1983.501572	2316.635976	1023.714321	2235.660507	2231.215941
0.90_624.8368m/z	624.8367912	0.904683333	neg	4989.46542	5031.657451	3606.379623	9907.599427	4394.28717	6576.307819	4930.860837	4676.48555	2181.367595
0.90_646.8187m/z	646.8186672	0.904683333	neg	2827.562196	4013.702333	1679.528019	3199.889896	1015.828706	1287.100358	2065.244575	629.4874878	1162.334953
0.93_161.8500m/z	161.8500412	0.925866667	neg	377.4474439	170.3724678	159.5829395	0.001403199	0.001403199	0.001403199	277.287925	163.2941471	92.24450835
0.93_181.8383m/z	181.8382683	0.925866667	neg	749.7911641	392.6348127	568.9140282	0.001403199	0.001403199	0.001403199	713.7499791	330.9439316	400.0333513
0.93_207.9231m/z	207.9231318	0.925866667	neg	342.1874446	435.9787367	146.002303	0.001403199	0.001403199	87.71980448	178.5980668	0.001403199	1.507673627
0.93_207.9653n	206.9580493	0.925866667	neg	1596.338726	1481.104721	1382.056372	1816.332349	1427.041276	863.0443762	2081.318166	1205.572928	1355.887148
0.93_221.8519m/z	221.8519343	0.925866667	neg	1539.99137	1192.091805	1014.506557	97.07445239	241.1821556	95.81309086	1451.940819	1056.90445	975.6051441
0.93_222.9063m/z	222.9063037	0.925866667	neg	4438.962423	3728.166737	3601.952852	2168.657045	2394.215713	2608.329447	4209.044439	3504.026631	3981.974281
0.93_225.0440m/z	225.0439863	0.925866667	neg	1124.388784	957.6919288	955.0604294	1327.655233	829.2121257	822.8139352	1064.1997	673.2512481	670.6079139
0.93_233.9823m/z	233.9822995	0.925866667	neg	1111.486857	785.1218677	888.3772996	713.7106098	74.98169707	472.9488587	1019.241595	585.2506218	713.2463384
0.93_243.8998m/z	243.8998247	0.925866667	neg	9905.821041	9478.61249	9715.151037	2948.435672	2950.545901	2791.492667	10262.87859	8744.030396	6898.794798
0.93_245.8969m/z	245.8969192	0.925866667	neg	6522.360144	6559.421685	6213.77228	1603.615323	1695.358964	2115.588813	6926.074101	5947.47665	4467.723565
0.93_247.8940m/z	247.8940221	0.925866667	neg	594.1439176	644.9709394	538.35698	113.7453203	90.7118676	0.001403199	630.645651	641.3548398	481.5427574
0.93_248.0234m/z	248.0234438	0.925866667	neg	763.7193245	811.1323404	1215.083425	1336.367746	550.8185453	863.9195372	920.4506789	771.2845412	489.1931273
0.93_264.9640m/z	264.9640397	0.925866667	neg	4337.696762	4420.051367	4897.927972	3728.52442	6411.351504	3974.464009	5585.410831	5312.369846	6958.877862
0.93_279.9080m/z	279.9080126	0.925866667	neg	7756.072409	7594.10752	8454.316657	5646.137506	5256.011681	4746.719106	8682.48265	8966.108055	8136.640843
0.93_307.0219m/z	307.0219316	0.925866667	neg	1441.010765	1412.29306	1207.557378	1315.009824	608.9903828	874.9228097	1345.563691	1503.880107	1225.815605
0.93_309.9851m/z	309.9851013	0.925866667	neg	813.4348427	912.330175	690.2574333	499.6921545	430.8116651	114.4101768	394.062203	82.37326311	255.7534021
0.93_312.8025m/z	312.802492	0.925866667	neg	937.928548	626.319197	495.1311397	0.001403199	0.001403199	0.001403199	416.2173774	337.8922684	440.6388515
0.93_317.0175m/z	317.0175193	0.925866667	neg	9238.780685	10357.09861	9073.649825	13170.41009	12624.79117	12600.64565	11934.78779	9958.322362	12203.89105
0.93_320.8851m/z	320.8851321	0.925866667	neg	550.5455103	1016.200067	1074.246308	155.6769038	518.7042643	531.7467688	760.4023232	636.689786	486.1011271
0.93_328.7877m/z	328.7877111	0.925866667	neg	20559.87704	17473.86768	17034.48876	11816.69818	12871.11425	13639.93325	21323.01414	18133.61479	18558.71998
0.93_340.8975m/z	340.8975335	0.925866667	neg	2113.940678	3252.492834	2934.581456	2196.560151	2520.766723	2062.917855	3701.554588	3740.866002	2661.098407
0.93_346.8144m/z	346.8144476	0.925866667	neg	34457.46788	35477.15184	36135.15165	74159.64585	54683.76262	59535.08619	44455.8716	52125.99943	42876.79723
0.93_386.7463m/z	386.7463082	0.925866667	neg	1453.493982	1009.256075	949.8030938	0.001403199	0.001403199	0.001403199	1027.906656	522.5012958	400.4598125
0.93_424.0492m/z	424.049191	0.925866667	neg	2739.997923	3684.059442	3318.054726	2891.023051	2928.505286	3022.981571	1884.527561	2284.875506	2741.881022
0.93_431.8930m/z	431.8929599	0.925866667	neg	1489.206073	880.3399821	1427.726295	1551.722221	889.9829384	560.2453917	1339.872736	1639.889014	542.4877268
0.95_178.9907m/z	178.9907169	0.946033333	neg	890.0216335	640.307438	728.8090544	415.9152023	258.1529022	211.6254987	658.4435163	391.899828	487.7893184
0.95_208.9313m/z	208.9312847	0.946033333	neg	2699.206347	2699.868518	1977.37108	669.8707397	1167.116501	806.2228099	1834.215826	1576.595174	1830.664735
0.95_216.9008m/z	216.9007939	0.946033333	neg	297.6579536	685.1315191	654.9660739	786.3530814	352.4444684	84.42419977	910.7589267	913.577877	380.737791
0.95_223.8490m/z	223.8489994	0.946033333	neg	1131.929229	1242.731616	856.8663772	88.75950946	143.4111483	166.270899	1478.990177	994.8545959	918.8293421
0.95_239.8508m/z	239.8508231	0.946033333	neg	6068.604985	6222.156475	7085.444402	10834.30314	10516.39593	10928.11671	8226.830832	9191.827082	9303.734783
0.95_242.8504m/z	242.8503797	0.946033333	neg	3790.061296	4434.374721	4717.337503	7004.638355	7070.004811	6513.132882	5669.74679	5573.850692	5459.823557
0.95_244.9077m/z	244.9077453	0.946033333	neg	783.8531605	878.9657654	673.8803579	189.4138041	295.8004055	191.027927	519.3846042	451.9496326	477.1186593
0.95_246.0668m/z	246.0667759	0.946033333	neg	1566.949015	2818.960788	3483.714785	12295.0938	12642.50878	12517.95228	2812.946359	6492.022911	5452.121596
0.95_249.8541n	248.8468695	0.946033333	neg	54274.1928	50517.94898	50482.58242	71062.04278	72494.51085	72626.84565	63615.5556	61074.88065	63480.53578
0.95_250.9754m/z	250.9754071	0.946033333	neg	772.0394885	855.8218308	755.7195253	168.5349859	129.5886972	106.3459941	538.4470642	771.147885	299.3018504
0.95_255.8223m/z	255.8222978	0.946033333	neg	4623.134967	3695.702374	3284.265751	772.9243504	1270.946587	1003.005425	4454.40002	3510.573453	3202.205795
0.95_256.8406m/z	256.8405631	0.946033333	neg	3051.089044	2594.575398	2243.152416	1476.179856	945.486256	1288.546964	2470.843215	2201.540238	2059.760865
0.95_259.8394m/z	259.8394086	0.946033333	neg	392.8966374	977.1430086	663.8212783	2386.89222	2161.194249	2082.223601	851.4092542	1472.6395	920.3250135
0.95_281.9052m/z	281.9052322	0.946033333	neg	1757.773541	2073.692851	1728.559195	421.1788616	1106.486941	731.1425436	1818.0234	1684.045527	1741.935383
0.95_289.9944n	288.9873208	0.946033333	neg	20366.86044	25635.90483	29441.53411	57483.82808	52450.25886	52846.44935	27968.77314	35882.23211	34176.81822
0.95_294.8618m/z	294.8618226	0.946033333	neg	128.8738965	297.2086163	312.1754882	1278.960505	1521.06298	1019.183864	627.2840342	749.7343566	386.6617064
0.95_296.9431m/z	296.9431459	0.946033333	neg	3428.322871	3434.52043	3886.626215	2067.769513	2340.46834	2551.999444	3240.36012	3571.835581	3129.770859
0.95_330.7843m/z	330.784258	0.946033333	neg	6590.541687	5255.931173	4792.396272	2769.885733	3448.17801	2688.66303	6007.299251	4667.78451	5136.590139
0.95_341.8988m/z	341.8988118	0.946033333	neg	38478.18627	44608.0207	43303.84712	60192.18635	57349.67952	57554.49873	48417.28974	53133.45699	50670.33937
0.95_348.8096m/z	348.8096461	0.946033333	neg	3297.844557	3716.167211	3839.209374	8670.311474	9575.382617	9256.451219	5694.889571	7453.725508	6655.616909
0.95_358.8410m/z	358.8409879	0.946033333	neg	142401.8068	146435.4062	144841.9424	179131.083	180452.6804	182761.0697	179794.0313	174226.5714	173118.9437
0.95_359.8167m/z	359.8166851	0.946033333	neg	12841.82093	10986.8312	11574.43612	14899.57644	15840.14689	16952.877	14084.72836	13860.50043	14255.77532
0.95_361.0580m/z	361.0580294	0.946033333	neg	0.001403199	0.001403199	0.001403199	1227.707196	451.7567854	521.897917	0.001403199	160.1988602	0.001403199
0.95_366.8008m/z	366.8007749	0.946033333	neg	1546.415972	1647.720932	1330.253535	2672.871335	2199.328301	1794.087202	2258.643499	2246.789992	2039.933549
0.95_367.7884m/z	367.7884162	0.946033333	neg	23502.53834	24777.60719	26480.86755	42158.33467	39009.22293	40206.6001	32189.54228	32533.12693	33470.24231
0.95_368.7962m/z	368.796226	0.946033333	neg	42159.83746	42237.18238	40213.75504	62225.52794	56777.57884	55220.0757	51064.61309	53678.23739	51715.23202
0.95_370.7914m/z	370.7913975	0.946033333	neg	4273.660502	4462.502229	3558.694964	5622.476763	6032.098102	5552.71657	4741.615727	5233.928531	5137.840082
0.95_389.7703m/z	389.7703225	0.946033333	neg	23276.57822	24643.00463	23283.93339	27939.47888	30149.27435	28274.24693	27982.86976	27191.49687	26702.59781
0.95_390.0367m/z	390.0366829	0.946033333	neg	0.001403199	0.001403199	0.001403199	2484.064149	1063.860136	1579.851717	0.001403199	0.001403199	0.001403199
0.95_391.0329m/z	391.0328664	0.946033333	neg	0.001403199	0.001403199	0.001403199	11992.04291	3340.927871	4226.616455	0.001403199	907.5927582	379.1879868
0.95_407.0275m/z	407.0275089	0.946033333	neg	0.001403199	0.001403199	0.001403199	2169.554375	342.7041355	741.7738133	0.001403199	0.001403199	0.001403199
0.95_418.7958m/z	418.7957801	0.946033333	neg	808.6906835	1548.637704	1513.740447	0.001403199	131.002095	633.9291667	1753.102775	449.6139984	1654.766782
0.95_426.7541m/z	426.7540594	0.946033333	neg	8905.217323	7058.923904	6904.637021	4049.271804	4344.875509	4759.983892	8734.156057	9987.770191	9211.372839
0.95_436.8210m/z	436.8209979	0.946033333	neg	4344.722021	5954.268618	5290.672286	7557.99344	7452.81514	8000.361816	6352.85141	8687.24197	8233.316595
0.95_438.0345m/z	438.0344829	0.946033333	neg	821.9768	1474.21433	1824.494159	0.001403199	0.001403199	0.001403199	1207.304127	2186.256984	1344.886694
0.95_439.8653m/z	439.8653238	0.946033333	neg	6481.987989	9263.985338	11023.72729	18458.85382	14897.53204	15299.89862	10014.02941	13829.69916	12377.49214
0.95_448.7362m/z	448.7361971	0.946033333	neg	5882.840898	5960.266295	5215.274043	3347.159999	3429.52311	3747.450625	6048.804076	5317.982317	6102.430011
0.95_461.8477m/z	461.8476949	0.946033333	neg	16861.12096	22323.73573	21239.39854	30875.19624	29139.99703	27622.56256	24553.40802	26955.07556	26689.00955
0.95_463.8432m/z	463.8432059	0.946033333	neg	195.3788785	382.0486822	923.0284279	1384.521596	949.3405727	890.1455415	421.958216	884.7202638	688.9039091
0.95_466.7624m/z	466.7624058	0.946033333	neg	27777.02217	30674.37704	28979.08564	62650.54844	52926.81396	51561.4848	38574.75337	42273.08703	41259.84683
0.95_468.7592m/z	468.7591594	0.946033333	neg	6290.897589	5006.437176	5665.527021	11163.67764	9531.860415	10242.33114	6137.726402	8081.300559	6400.424313
0.95_478.7892m/z	478.789213	0.946033333	neg	84776.72377	88634.42234	88354.25584	89765.6717	96898.74079	96792.18668	113039.5017	105662.3009	104522.8574
0.95_483.8297m/z	483.829732	0.946033333	neg	11619.78121	13931.32776	13585.99276	16498.59059	17562.96898	17607.64859	17183.71888	18842.25826	18309.52917
0.95_487.7367m/z	487.7366639	0.946033333	neg	7767.167709	9582.34309	10463.88388	13027.14544	13823.53169	14195.91552	12685.56768	13643.65817	12729.75194
0.95_488.7445m/z	488.7445007	0.946033333	neg	24588.00474	28194.40693	24976.81264	46144.68786	40400.95591	41795.38763	33697.8765	36421.79956	32217.6986
0.95_509.7192m/z	509.7191552	0.946033333	neg	12710.84601	12743.30865	13871.60071	17138.57101	17608.84494	17987.96344	17389.59232	16762.02575	16307.16607
0.95_559.8150m/z	559.81499	0.946033333	neg	2114.250287	3758.812157	4719.536004	10957.07351	7171.079014	8525.645595	5429.463062	7348.43673	6347.389853
0.95_576.7570m/z	576.7569954	0.946033333	neg	77006.08427	85605.62656	87669.34578	136899.8289	137140.7037	134286.3828	114180.358	127717.5984	123602.1832

0.95_578.7530m/z	578.7529661	0.946033333	neg	14206.03755	17852.04362	17034.04198	30490.30593	26386.03172	27753.98363	21881.95587	26799.93901	19913.93376
0.95_581.7974m/z	581.7974297	0.946033333	neg	9204.151004	12047.72112	11010.6103	19476.14472	15806.55084	15326.09736	11926.28691	16356.42096	12647.33776
0.95_585.7066m/z	585.7066345	0.946033333	neg	0.001403199	1747.15535	878.09976	5670.737885	2967.998921	4036.751356	2111.753944	3109.464061	2354.533249
0.95_586.7123m/z	586.7123081	0.946033333	neg	6829.711607	10203.03295	9402.653988	26802.84385	21199.20539	21503.22371	13223.47768	16075.66469	13863.97179
0.95_588.7094m/z	588.7094436	0.946033333	neg	1342.113574	532.7245165	1667.271269	4376.561131	4214.513485	4106.21142	1581.048444	3763.504463	2501.116665
0.95_598.7392m/z	598.739156	0.946033333	neg	23210.04952	25621.88282	26233.467	36504.38003	34048.61533	35097.0256	32957.28116	36626.21726	31350.3501
0.95_608.6947m/z	608.694679	0.946033333	neg	12692.01275	13971.1893	15195.2502	25441.22698	24861.2265	23575.22166	17817.10333	21559.51401	17151.26716
0.95_639.6249m/z	639.6248515	0.946033333	neg	386.3274836	0.001403199	0.001403199	560.2422039	0.001403199	958.8316403	764.0513843	944.2521973	0.001403199
0.95_674.7290m/z	674.7289749	0.946033333	neg	983.7615337	1825.036307	2579.989783	6352.752252	3310.581829	4300.508766	1797.440425	3008.864944	1217.513968
0.95_696.7069m/z	696.7069256	0.946033333	neg	23620.96552	26537.35218	29394.28876	52712.38439	47958.71468	51048.82529	38639.98945	44880.00724	40789.66412
0.95_706.6633m/z	706.663272	0.946033333	neg	2686.21771	5956.414554	5452.030258	15428.60392	12378.22398	11704.66681	5967.192825	8990.826752	8838.692445
0.95_718.6894m/z	718.6894491	0.946033333	neg	11475.93695	12000.58601	14901.42268	15158.78777	16869.69614	18327.91102	18659.63229	18865.78659	15981.85708
0.95_727.6394m/z	727.6393597	0.946033333	neg	1743.163391	2521.346037	3244.95256	6394.034622	5441.246647	6621.263806	2431.355658	3717.160558	4483.388062
0.95_749.6193m/z	749.6193306	0.946033333	neg	1804.343911	2552.632189	2245.725758	4232.84254	4378.96466	4253.16174	3868.10751	3631.891437	2934.854544
0.95_816.6569m/z	816.6569445	0.946033333	neg	4394.661333	8740.282127	7175.593437	19422.26503	16481.57031	20811.91163	11056.32552	15188.7794	13439.66137
0.95_936.6057m/z	936.6056962	0.946033333	neg	0.001403199	0.001403199	455.3934358	6907.665066	1692.993925	2124.401398	1264.544205	2045.52684	541.1374232
0.97_195.8772m/z	195.8772487	0.967616667	neg	2038.47648	2071.422097	1917.201181	2585.633913	2304.755846	2624.768007	2546.655037	2320.564231	2093.064312
0.97_216.0198m/z	216.0198255	0.967616667	neg	0.001403199	494.2144261	1105.604515	4839.146469	3587.332407	3834.388668	434.608903	2193.803736	1494.515298
0.97_238.9844m/z	238.9844397	0.967616667	neg	70436.14658	68417.78811	65571.87427	101961.9666	99031.80909	105204.1138	70180.35954	78717.97524	78740.75615
0.97_244.8523m/z	244.8522553	0.967616667	neg	886.5798236	976.9152264	1003.551097	2052.048493	1750.904995	1682.631683	1315.60749	1450.224612	1290.476018
0.97_245.9469m/z	245.9469234	0.967616667	neg	3360.713918	3210.3681	3071.701755	3207.766943	2884.167004	3600.576003	3780.144237	2967.207069	2996.285929
0.97_246.8517m/z	246.8516625	0.967616667	neg	1697.733813	1706.842964	1426.690465	2636.562074	2192.634466	2332.27449	2419.841309	1951.503573	2006.364236
0.97_248.8560m/z	248.8560493	0.967616667	neg	2526.518764	1609.811328	2164.919038	4047.982504	4372.146356	4374.188013	2431.764279	2593.936314	2331.078567
0.97_252.9106n	297.9089109	0.967616667	neg	5069.037294	6125.790608	5911.445244	8447.141644	7527.883269	7541.480046	6516.373415	7830.572547	7190.797576
0.97_253.9191m/z	253.9191276	0.967616667	neg	985.687501	1420.885742	1324.710142	1873.262514	1710.035495	1906.883563	1439.655084	1568.133735	1562.328661
0.97_259.8162m/z	259.816194	0.967616667	neg	1580.648833	914.9225724	993.1801547	244.1099011	134.6192617	133.776773	1202.74592	941.8426002	722.9668032
0.97_261.9418m/z	261.9418464	0.967616667	neg	18630.17479	18188.18024	17258.37941	20034.95308	19975.46577	20562.46742	19260.57907	17942.11062	19201.65559
0.97_263.8337m/z	263.833651	0.967616667	neg	2382.366804	1898.447264	2156.12523	2747.810734	2840.222643	2658.239437	2155.188398	2820.821553	2393.54504
0.97_270.9219m/z	270.9218581	0.967616667	neg	5402.666085	5321.544226	4633.958251	8377.053146	7556.285559	7357.153788	4215.006828	5334.338447	5211.163338
0.97_278.8801m/z	278.8801204	0.967616667	neg	1652.286607	2133.747501	1704.720856	1710.713477	2466.364464	2058.009844	2389.336353	1762.629147	1925.260818
0.97_289.9369m/z	289.9369148	0.967616667	neg	32232.21673	36015.00852	34486.3488	39114.26835	41177.42046	40741.40605	41612.04329	38265.81584	41082.47204
0.97_290.9835m/z	290.9835151	0.967616667	neg	1337.948072	1334.420468	1575.526926	2162.566383	1395.14771	1804.536096	1299.206466	1270.676948	1149.93413
0.97_293.9904m/z	293.9903865	0.967616667	neg	3958.804008	4432.080553	4974.369753	7778.11686	7303.572442	6919.923989	4780.405021	6941.378517	6112.321187
0.97_310.9693m/z	310.9693368	0.967616667	neg	84234.77921	95539.71845	103026.2443	130013.7927	129392.3514	131510.312	100833.7217	107861.5466	111224.586
0.97_315.8385n	360.836689	0.967616667	neg	32095.5339	32519.01289	33425.81646	40196.06028	42529.87513	40988.44016	41434.351	42202.48711	39677.32563
0.97_318.9250m/z	318.9249747	0.967616667	neg	2085.651438	2357.264291	2106.230438	804.3890158	1840.333687	1434.923015	2006.630874	2020.792324	1864.452362
0.97_333.9547m/z	333.9547303	0.967616667	neg	1138.835	1111.016133	1352.065436	1269.104313	1601.141449	1414.217895	851.1294602	1313.872148	1291.112046
0.97_345.0440m/z	345.044016	0.967616667	neg	0.001403199	0.001403199	0.001403199	304.016663	227.0379431	332.135889	0.001403199	0.001403199	141.9048679
0.97_358.9341m/z	358.9340562	0.967616667	neg	22189.138	21105.62642	21445.09938	34416.91073	32971.75666	35284.72811	21860.60461	26136.493	25802.57595
0.97_360.9296m/z	360.9295931	0.967616667	neg	1041.544993	1591.979945	1571.0761	1977.218504	1554.350595	1898.76399	1438.879831	1192.43492	1117.86813
0.97_361.8855m/z	361.8855036	0.967616667	neg	1159.75248	1295.128173	858.978973	1325.819001	917.3366207	1083.229849	1270.178508	1151.591012	955.745757
0.97_363.8809m/z	363.88092	0.967616667	neg	46885.75377	47526.84961	48304.81543	57346.75869	55462.65175	56081.99498	54104.70895	51862.71763	53150.91613
0.97_363.9738m/z	363.9738332	0.967616667	neg	961.7726384	1523.027879	1409.73964	1906.00733	1615.227061	1848.012495	1046.741117	1898.087187	1325.2591
0.97_368.8892m/z	368.8892235	0.967616667	neg	3185.533145	3053.866012	2514.167783	5763.84206	4969.812352	5242.362485	3042.612665	3480.369279	3411.584467
0.97_421.8389m/z	421.8389261	0.967616667	neg	1553.013843	1282.631274	1436.472474	171.0997049	441.3890585	377.6547927	1190.435825	1120.423891	1051.313066
0.97_428.7510m/z	428.7509975	0.967616667	neg	2027.342336	1986.246979	1948.646405	951.0166922	813.8689041	953.2382938	1624.193318	1579.91252	1081.052072
0.97_430.9182m/z	430.9182217	0.967616667	neg	28409.12441	34981.36662	36139.80567	48809.43228	45476.59204	45771.54529	35714.70123	41891.76376	39548.28532
0.97_435.7867n	480.7848608	0.967616667	neg	12735.11253	11828.57956	13448.74937	15902.60051	17445.64611	17473.06821	18511.63741	17627.26702	16072.66385
0.97_435.9584m/z	435.9584065	0.967616667	neg	1770.251583	1845.637438	2779.330106	4432.386981	3557.969029	3659.480016	2373.838239	3017.356564	2464.753202
0.97_440.8735m/z	440.8735138	0.967616667	neg	4497.078194	5469.240305	5913.417284	10385.10769	8063.660212	7773.927901	5653.198675	6242.634975	6262.561705
0.97_478.8826m/z	478.8825904	0.967616667	neg	1465.091585	1941.868643	1427.404344	2295.165015	3146.514752	2544.017997	1474.830888	2220.42485	2063.79054
0.97_490.7408m/z	490.7408226	0.967616667	neg	3416.576748	4267.521723	4106.003334	7291.75924	6770.404934	7389.290229	4491.596066	6048.682358	5399.474547
0.97_500.8648m/z	500.8647667	0.967616667	neg	4716.524736	4512.525652	4339.147062	6742.025456	7388.317628	7022.829733	4424.615717	4958.356793	5147.955621
0.97_505.8117m/z	505.8117322	0.967616667	neg	8644.075439	8725.191904	8172.10638	6081.900879	8930.763587	7726.189153	9449.317984	9118.415313	9230.48829
0.97_510.7266m/z	510.7265569	0.967616667	neg	24434.55101	24122.29107	22644.11377	28757.42612	30855.02887	32876.88244	30454.13925	28114.41188	30289.34752
0.97_512.7238m/z	512.7238033	0.967616667	neg	2944.734795	3454.381519	2654.208695	4932.182258	4098.40729	4585.774248	4385.084092	4523.236294	3437.239414
0.97_550.8676m/z	550.8675948	0.967616667	neg	9489.908789	9305.851202	11167.89548	15720.12895	13125.91036	14103.09494	11876.83108	13429.9648	11965.81756
0.97_555.9077m/z	555.9076614	0.967616667	neg	1001.854787	721.2182104	2267.811486	3020.137387	1305.698726	2095.842022	1365.074938	1425.305112	699.4602063
0.97_600.7357m/z	600.7356725	0.967616667	neg	4772.722147	4590.436158	6646.771017	7610.633425	6323.98462	6771.084363	7670.594648	6860.991559	5624.769516
0.97_603.7793m/z	603.7793488	0.967616667	neg	5442.481883	6655.907173	7670.960796	9327.602537	10347.19193	9698.888512	7592.952686	8986.571724	8292.725379
0.97_607.6872m/z	607.6872251	0.967616667	neg	2171.396882	3276.768271	4525.871771	9779.36831	7912.64286	7795.339968	4248.510584	6789.697003	4833.691433
0.97_610.6918m/z	610.6918134	0.967616667	neg	1537.164589	2794.652417	2259.361393	3643.851874	2402.473288	2801.562495	2795.269825	2850.623289	2146.878976
0.97_620.7214m/z	620.7213791	0.967616667	neg	15098.40896	15542.56086	16089.56715	17554.06041	17432.41929	18300.53444	19247.70016	19263.73082	19653.51162
0.97_629.6691m/z	629.6690947	0.967616667	neg	4194.298472	5496.344656	4937.489341	8918.7609	8159.95646	8499.258383	7935.886924	8634.643767	7639.173354
0.97_640.6324m/z	640.6324046	0.967616667	neg	904.8506646	1132.890522	1052.681543	1316.793432	1435.35485	2252.96803	1370.488551	1043.777395	1183.996435
0.97_642.7042m/z	642.7042308	0.967616667	neg	4070.183003	4910.398873	4508.168842	5606.184816	5485.166337	4938.144973	6565.57022	5746.025161	5419.681396
0.97_698.7037m/z	698.7036712	0.967616667	neg	4178.991221	5362.318139	4224.447081	12644.58981	11719.24269	10457.34702	5684.425609	9923.06179	7750.564449
0.97_701.7478m/z	701.7477565	0.967616667	neg	1087.489659	3577.973945	2710.795195	5342.495713	4886.365925	4423.371335	2293.930572	5169.643195	3862.413898
0.97_750.6268m/z	750.6267789	0.967616667	neg	4600.991407	5888.807029	5301.730064	8609.21413	9729.037893	9172.977638	7152.196298	8293.725665	7582.854846
0.99_168.8900m/z	168.8900081	0.989233333	neg	14407.77903	13516.08618	12201.65955	15342.17148	15708.88113	15446.11365	15710.45649	13818.60608	14743.29298
0.99_178.8841m/z	178.8841076	0.989233333	neg	780.8011799	698.376641	489.5686471	298.1962682	326.3866833	174.502214	723.0018548	517.5507841	525.1400755
0.99_188.8618m/z	188.861783	0.989233333	neg	82.97337625	0.001403199	84.95210123	0.001403199	0.001403199	61.04269191	0.001403199	48.54649734	107.6775267
0.99_190.9452m/z	190.9451589	0.989233333	neg	174.8606695	134.5922421	59.39375126	469.9288413	200.6807159	164.3597545	0.001403199	174.2921391	150.4770892
0.99_248.9784m/z	248.9783915	0.989233333	neg	2833.845357	2363.164961	2994.841883	1450.309067	1087.826616	1532.003868	2350.259636	2401.946422	2040.150661
0.99_269.9141m/z	269.91407	0.989233333	neg	988.2453115	1173.460762	1009.223111	1553.13617	1349.977393	1224.409491	1084.890188	961.2465856	887.5072919
0.99_270.9610m/z	270.9609785	0.989233333	neg	1340.218517	1016.51113	1303.919603	609.7744234	453.7024138	526.5315608	1189.133159	924.7861391	860.1281525
0.99_390.8716m/z	390.8716103	0.989233333	neg	1262.305651	1939.260405	1365.087719	2963.482081	2673.410759	2191.212951	1797.723733	1746.171273	1489.58028
0.99_412.8538m/z	412.8538376	0.989233333	neg	3194.942502	2551.945384	2428.291285	3624.466512	4111.696933	4054.420321	2173.168062	2907.733459	2291.807361
0.99_452.9003m/z	452.900334	0.989233333	neg	15779.4008	15726.23682	17002.63338	16394.8185	18613.49583	16128.27485	18695.19094	17893.3103	19509.77319
0.99_474.8822m/z	474.8822344	0.989233333	neg	9780.916154	8958.247049	9930.578529	10410.86429	11011.19033	10449.40154	10617.49912	10095.26218	10343.47801
0.99_484.8375m/z	484.8375245	0.989233333	neg	3509.038823	3741.321698	3763.778811	4837.600489	4179.372105	3929.986871	4925.088449	3249.489502	3561.733007
0.99_494.7633m/z	494.76331	0.989233333	neg	1187.464432	1634.377727	1725.490267	1799.887071	2479.089666	2717.645512	2093.316605	2602.988357	1473.227282
0.99_501.7477m/z	501.7476678	0.989233333	neg	7875.228328	8898.800178	8542.282259	9192.774533	9958.310504	10112.91841	10328.49941	9806.654979	9507.468548
0.99_522.8469m/z	522.8469195	0.989233333	neg	2537.376458	1736.894849	2174.253003	2153.816038	2353.793924	2191.331966	1734.347429	1829.094505	1753.732624
0.99_572.8497m/z	572.84972	0.989233333	neg	5176.722625	7523.730724	7330.737395	8646.468119	7442.931945	7525.164577	8730.808103	8461.12227	6456.808082
0.99_594.8321m/z	594.8321451	0.989233333	neg	1369.455891	1781.329622	1616.64007	373.0444816	1046.199637	2227.396366	2032.398461	1562.40375	1680.217407
0.99_625.7614m/z	625.7613529	0.989233333	neg	3144.943065	3233.951902	2886.215952	1939.19127	2307.96503	2976.652595	3173.042372	3523.129759	3137.147981
1.01_103.9203m/z	103.9202574	1.0111	neg	9220.24932	8644.073205	8345.444967	10111.90901	10410.26977	10242.74217	10634.53794	9057.125479	9617.702806
1.01_151.8871m/z	151.8871107	1.0111	neg	502.4057312	752.2940602	603.5176191	762.015984	926.3779264	809.1535968	946.5712817	910.0832034	934.8245588
1.01_183.8771m/z	183.8771261	1.0111	neg	11553.65935	11054.77875	10717.44334	11935.65883	12502.90889	12467.56605	13315.80506	12121.97679	12524.06757
1.01_187.8734m/z	187.873412	1.0111	neg	1036.589593	925.3107221	564.950864	39.1183134	171.7981216	264.3159859	1103.244272	579.4097633	661.9265824
1.01_198.9006n	243.8997894	1.0111	neg	3692.746239	3930.263925	4054.501331	3497.280667	2984.492449	2756.668355	4380.488746	4176.939912	3173.269663
1.01_239.8671m/z	239.8670925	1.0111	neg	14095.45968	12818.92763	13531.48251	15883.34145	17998.42126	17248.74314	16438.19787	14938.35377	15644.91248
1.01_253.0003m/z	253.0003111	1.0111	neg	747.6305713	806.5363156	674.9274696	458.6523102	691.2195108	571.2936051	721.6865155	952.0497488	606.2972794
1.01_274.8249m/z	274.8249364	1.0111	neg	1631.328379	994.6854187	833.0704641	941.920848	979.9259531	1212.368118	1408.635784	1049.85468	1137.134361
1.01_276.9633m/z	276.9632943	1.0111	neg	1895.448559	1462.979827	1688.069128	1210.565027	954.2224879	1228.181405	1145.754608	1181.684814	1184.559752
1.01_286.8929m/z	286.8929338	1.0111	neg	1390.787886	1666.09678	1354.80882	1145.032684	1706.982069	1586.068749	2066.841621	1637.120009	1394.999843
1.01_311.8325m/z	311.832501	1.0111	neg	2320.826253	1532.887449	1406.380421	1649.975763	1537.407848	1462.272172	1860.274732	1614.214953	1472.070184
1.01_392.7739m/z	392.7739356	1.0111	neg	4689.648906	4157.305096	4058.620002	5412.898928	4699.079134	5028.772835	5320.04748	4832.24653	4387.200443
1.01_470.7185m/z	470.7185207	1.0111	neg	5077.376427	3937.59054	3937.182126	2700.640734	2990.805203	2609.494282	4654.942176	4170.432274	4323.555377
1.01_496.8652m/z	496.8651957	1.0111	neg	2292.909022	2054.570597	1955.174315	1605.630202	2218.750405	2419.658381	1699.389637	2440.471198	2390.518201
1.01_500.7714m/z	500.7713711	1.0111	neg	29175.9461	30053.26462	28990.22241	29004.24283	33070.73863	31084.77394	37351.85721	34529.11641	35111.82743
1.01_510.8202m/z	510.8201623	1.0111	neg	564.4258151	752.4789393	656.9172885	929.0702957	630.0743288	654.174769	503.4751107	546.7955399	462.1539815
1.01_652.6595m/z	652.6594696	1.0111	neg	6741.470044	6962.650554	7263.079436	7797.164864	9530.803808	8902.577743	9459.749521	7935.549916	6920.161198
1.03_119.9494n	164.9475739	1.033516667	neg	383293.0086	371595.5726	355161.9406	441151.4046	457345.8566	448159.5243	461203.0493	417743.3304	433950.1514
1.03_162.8830n	161.8758673	1.033516667	neg	3577.801193	2953.51002	2901.059687	1528.522695	1380.039242	1589.216671	3207.991771	2731.819462	2656.375112
1.03_166.9434m/z	166.9434161	1.033516667	neg	9775.41349	9116.189545	8456.994926	10248.33842	10474.82877	10645.04372	10782.42078	10193.88573	10402.16583
1.03_168.8893n	213.8878782	1.033516667	neg	13643.2897	11993.33388	10511.41873	12453.55236	12719.60885	12645.58042	14265.77293	11692.08505	11926.69076
1.03_173.9657m/z	173.9656606	1.033516667	neg	492.6132808	509.1897672	376.7886225	474.1957526	412.4162431	530.3178513	371.5159971	341.7522204	335.191918
1.03_180.9127m/z	180.9126633	1.033516667	neg	686.3189863	660.4105918	610.2367799	562.9505941	627.0139844	624.1343492	811.196961	619.8321449	736.4720098

1.03_185.8658m/z	185.8658254	1.033516667	neg	931.1030685	676.5752202	722.4991429	458.600523	426.2695794	425.2790182	946.313728	717.0965962	702.0754383
1.03_185.8928m/z	185.892829	1.033516667	neg	12839.81337	11661.04116	11058.6858	12982.0892	13726.98403	13827.6973	13866.26579	12670.89594	13076.86274
1.03_191.8553m/z	191.8553471	1.033516667	neg	1948.272121	1639.510515	1416.45892	839.3019858	903.7113499	846.7347977	1724.907797	1589.062845	1477.452469
1.03_194.8897m/z	194.8896746	1.033516667	neg	4626.287462	3914.129181	3915.475375	3579.219591	3811.757737	3850.614839	4557.050521	3809.766987	4159.210329
1.03_195.8892n	240.8873851	1.033516667	neg	78193.24034	72249.37532	68965.12562	85988.01533	89140.53438	86285.08724	86294.29583	80454.64042	82720.79156
1.03_212.0179m/z	212.0178708	1.033516667	neg	336.1141708	257.6078936	240.3061016	97.44839991	37.18470696	80.92455848	298.9835559	179.2038028	304.5517983
1.03_237.8883m/z	237.8883154	1.033516667	neg	6814.396639	4971.533164	4488.729377	4492.535828	4574.728483	4465.064499	5027.312886	4538.072448	4535.880465
1.03_250.9956m/z	250.99564	1.033516667	neg	2788.257377	2871.624608	2482.44249	1876.595028	1825.681375	1637.957017	3032.837457	2097.949211	1730.386054
1.03_261.8327m/z	261.832703	1.033516667	neg	987.6339462	762.1397456	1055.104367	1075.509343	1101.979538	983.7334168	1206.891844	924.6996254	1189.371655
1.03_268.8335m/z	268.8335078	1.033516667	neg	4146.757502	3411.437648	3112.908809	3479.901971	3761.992986	3667.52658	4108.33561	3683.232222	3547.549018
1.03_270.8288m/z	270.828828	1.033516667	neg	70705.87726	64763.48454	62546.63989	75180.92831	82384.71189	79125.75069	77326.52913	75292.40631	75033.55989
1.03_287.8317m/z	287.831686	1.033516667	neg	2627.554897	2437.289085	2327.182133	3031.994379	2968.116907	2774.604153	2909.827081	2760.912757	2644.009371
1.03_344.7892m/z	344.7891899	1.033516667	neg	683.0059706	596.1092606	837.6394949	1627.154611	1236.257211	945.9176575	1239.718181	1194.945647	1297.315359
1.03_380.8228m/z	380.8227748	1.033516667	neg	54274.63981	52304.63357	50707.14895	57276.69318	61759.68332	62290.53278	67078.38682	60795.42318	64133.25065
1.03_381.7985m/z	381.7985203	1.033516667	neg	21964.80614	18934.15613	19449.64747	19596.6863	21340.09857	21414.72986	23460.61321	19234.55892	20839.75618
1.03_431.8682m/z	431.8682123	1.033516667	neg	3894.335154	3855.979541	3406.441976	4375.460819	3915.488095	4309.415514	4818.881843	4102.25279	4353.594792
1.03_440.7787m/z	440.7786996	1.033516667	neg	1314.10124	1026.05795	953.1833642	612.223815	176.1306668	740.6797353	1424.304664	1669.870226	1263.467466
1.03_523.7302m/z	523.730153	1.033516667	neg	3240.307593	2929.983112	2456.406848	2769.454813	2946.327059	3476.430211	3513.952951	2813.831284	2820.063511
1.03_524.7501m/z	524.7501446	1.033516667	neg	282.8891425	561.6173103	528.7646879	1144.195847	797.6196341	704.8064614	969.5618245	1074.543439	958.4617021
1.03_532.7090m/z	532.7090013	1.033516667	neg	16071.25683	15623.06226	15630.32877	18160.42003	20498.25227	20608.40362	21153.26374	19067.60056	17744.73085
1.06_150.8998m/z	150.8997746	1.055866667	neg	905.2841227	1010.891168	770.076527	758.534709	718.8051235	858.4635854	932.0880538	726.1654429	741.2829622
1.06_152.8951m/z	152.8951082	1.055866667	neg	19261.66467	16846.83818	16119.62764	17624.9851	19881.37881	18880.06983	19483.09285	17672.96756	18625.49888
1.06_154.8910m/z	154.8910092	1.055866667	neg	495.7318605	285.9703903	291.3113516	251.1844173	283.9812925	435.6540393	290.1646675	401.405806	202.2530741
1.06_154.8956m/z	154.8955507	1.055866667	neg	1264.323709	706.9812064	727.4043124	703.0912177	822.7793451	758.1453663	1053.746739	848.3354237	992.9139523
1.06_157.8722m/z	157.8721952	1.055866667	neg	498.7012152	447.3872119	672.5266044	260.0154125	233.9056652	294.3648677	766.6063359	681.6638727	512.5344093
1.06_160.8859n	159.8787515	1.055866667	neg	6218.36162	4650.510028	4784.529582	2200.376351	2819.278756	2536.505123	5500.58764	4891.986674	4888.182676
1.06_166.9963m/z	166.9963369	1.055866667	neg	877.8464978	722.1818573	624.281175	630.1987935	621.1272978	413.8314505	909.4103823	808.5389664	774.6410424
1.06_171.0664m/z	171.0664113	1.055866667	neg	570.509671	508.8850504	442.0790437	539.5405277	947.9238572	699.8422941	1086.320989	509.6792669	1297.897046
1.06_174.8921n	348.7718031	1.055866667	neg	3106.551111	2812.655744	2749.634662	2267.519433	2223.886954	2183.123183	3515.51797	2767.792859	2478.783605
1.06_183.8485m/z	183.8484545	1.055866667	neg	1600.434163	1583.639196	3059.579394	1074.496376	1094.304639	1046.105154	2978.804687	3294.18877	2605.437755
1.06_185.8456m/z	185.845557	1.055866667	neg	887.8287121	1038.589908	1978.598045	681.6256097	704.3211556	705.9438911	1845.887185	2049.444877	1619.562608
1.06_196.8850m/z	196.8850111	1.055866667	neg	71204.55291	61779.89127	58739.12557	66700.25651	75715.61537	72112.93785	72706.44472	66597.26166	71759.56403
1.06_200.8810m/z	200.8809689	1.055866667	neg	488.6087378	273.9744069	207.5209348	273.9855099	295.8226185	309.6613802	365.5077917	225.8963947	244.2323395
1.06_215.8686m/z	215.8686038	1.055866667	neg	3082.233508	2588.434412	2366.498209	675.1034023	836.0599892	802.3139725	2853.858422	2187.782366	2156.154782
1.06_217.8656m/z	217.8656055	1.055866667	neg	1393.084975	1086.901676	856.141438	479.1525489	338.7607565	474.2592232	1218.129342	955.4702497	835.0023839
1.06_234.9838m/z	234.9837675	1.055866667	neg	1018.687611	597.9537261	847.0580235	1379.238908	1238.726495	1171.62279	1224.304655	1369.518484	1370.066299
1.06_236.8780m/z	236.8780201	1.055866667	neg	3669.535828	3373.433687	2878.185786	3055.221057	2900.792625	3267.061197	4022.50701	3736.816693	3598.811372
1.06_261.8806n	260.873356	1.055866667	neg	115014.747	107600.3791	105997.8111	104175.5426	122011.4493	119051.9533	132894.6447	119167.1714	124837.0163
1.06_261.8912n	306.8883179	1.055866667	neg	4344.290995	2833.30189	3485.497587	3812.456884	4273.803074	4471.694635	4316.659098	3422.236531	3694.474889
1.06_262.8693m/z	262.8692968	1.055866667	neg	8987.945171	8329.580361	8008.630049	7430.466882	9559.098755	8738.07525	10442.57952	9121.766127	9687.314856
1.06_265.9672m/z	265.9671663	1.055866667	neg	96.07342617	46.05859826	142.9628128	243.5361938	52.14438229	115.0102966	101.7349444	134.8537025	170.2655677
1.06_286.9461m/z	286.9460681	1.055866667	neg	6255.337818	5317.377544	6343.827458	5795.076533	6594.783066	5799.585172	7555.336096	7135.560095	7431.213079
1.06_298.9068m/z	298.9068209	1.055866667	neg	1255.887308	1181.361427	1151.549139	968.1164397	1393.545307	1277.51633	1577.645099	1167.023704	1206.745878
1.06_306.0583m/z	306.0582714	1.055866667	neg	404.4044538	542.3148612	371.5535336	397.2551033	384.1939063	518.1558082	709.8676306	554.2043055	323.5042583
1.06_313.8127m/z	313.8126613	1.055866667	neg	1062.521405	895.4592128	674.8087826	559.6064379	560.155366	819.2817386	906.4569523	535.3644774	688.6233698
1.06_315.8267m/z	315.8266714	1.055866667	neg	11132.43689	9983.735268	8076.708794	9987.959962	11178.93617	10523.27498	10796.30993	10227.60555	10950.8273
1.06_336.8217m/z	336.8216508	1.055866667	neg	1437.118571	1303.434724	901.1182252	1009.418694	900.5978615	632.2772742	1612.074068	1502.70562	1192.174475
1.06_337.8198n	382.8180274	1.055866667	neg	9492.701958	10528.3383	9739.085585	11187.31156	11465.78743	12950.27129	14142.27294	12495.96599	12223.63598
1.06_349.8624m/z	349.8624117	1.055866667	neg	2706.930773	1827.281611	1716.823565	1057.519763	1516.24539	1657.192434	2135.829691	1860.518836	1601.552766
1.06_384.9133m/z	384.9132776	1.055866667	neg	2890.811383	3231.302055	4153.492523	4471.512549	3352.227855	3302.330537	4493.705506	4409.351422	3626.499379
1.06_402.8047m/z	402.8046661	1.055866667	neg	18360.09751	16717.20989	17199.95129	16770.1329	20060.69253	19907.6824	22813.73195	20272.6235	20793.14385
1.06_406.8956m/z	406.8956021	1.055866667	neg	1827.883659	1741.627254	1783.353393	2522.298961	1903.504943	2001.020943	2380.811297	2677.623417	1660.666514
1.06_412.7600m/z	412.7599694	1.055866667	neg	28002.76365	26466.0689	24655.72522	29955.29283	30059.71804	30021.78981	31263.24933	29247.33256	31440.17074
1.06_464.7620m/z	464.7620088	1.055866667	neg	2966.138118	2599.204594	2494.268537	1428.749598	1608.755607	1512.994125	3090.618516	2286.517386	2017.192526
1.06_490.7031m/z	490.7031211	1.055866667	neg	2049.211552	1845.206102	1389.165517	1715.949849	1668.389287	1715.091117	1939.192129	1450.248192	1222.657907
1.06_525.7453m/z	525.7453272	1.055866667	neg	1156.9299	1246.024666	1304.971618	1022.151982	1121.649786	1393.828	1217.445077	1172.572318	1423.204009
1.06_534.7065m/z	534.7064793	1.055866667	neg	2433.639505	2207.66949	1973.931825	2590.896464	2594.419035	2450.002027	3400.294234	2638.17868	2268.8871
1.06_544.7358m/z	544.7357978	1.055866667	neg	8573.037291	7294.210555	7742.53259	8312.308255	8790.22483	9255.945449	10152.04592	9138.857635	9402.786054
1.08_104.9539m/z	104.953871	1.078266667	neg	470.1126336	386.0038944	343.3527627	167.0738615	190.0817952	202.7336252	413.4364595	351.681093	322.6900362
1.08_126.8811m/z	126.8810644	1.078266667	neg	5362.611849	3818.58221	3607.283724	1938.762526	2340.209866	2218.41837	4356.284074	3457.628748	3744.322273
1.08_128.8782m/z	128.8781611	1.078266667	neg	4140.903548	2943.854031	2852.702563	1266.818736	1562.463118	1554.381464	3394.201449	2644.610829	2691.259801
1.08_165.9509m/z	165.950922	1.078266667	neg	2112.919336	1840.514603	1980.642172	2085.943652	2521.237845	2191.945375	2618.72209	2305.299616	2557.662047
1.08_170.8710m/z	170.8709527	1.078266667	neg	10663.43119	7737.636773	7063.46971	3029.636053	3841.121852	3888.992455	8694.597735	6252.363487	7075.702964
1.08_174.8650m/z	174.864989	1.078266667	neg	758.3782997	617.5432887	614.2944685	281.0638156	284.2127883	262.9322012	659.4336816	583.4603837	598.8173077
1.08_181.8819m/z	181.8818631	1.078266667	neg	477.1809526	408.3535153	355.9620619	413.288067	367.6725427	419.2565067	576.6625668	459.333684	444.7088188
1.08_181.9379m/z	181.937863	1.078266667	neg	23406.82716	22141.9857	21477.57772	24378.25467	24922.5133	25372.14761	27447.41714	23490.76485	24224.34082
1.08_187.8888m/z	187.8887885	1.078266667	neg	1588.537796	1537.833094	1640.614789	1463.139282	1591.828243	1630.447434	2113.5341	1511.429257	1764.634984
1.08_223.8819m/z	223.8819267	1.078266667	neg	526.2760111	326.1356653	252.4573821	228.6621492	249.6973067	159.8218093	451.2068158	292.1900101	331.5639625
1.08_225.8853m/z	225.8853205	1.078266667	neg	1851.660163	1595.733863	1427.524346	1418.391923	1317.520907	1434.375282	1952.504987	1538.069841	1655.561368
1.08_226.9048n	271.9072843	1.078266667	neg	5677.67108	4402.500485	4226.444989	2987.319439	3108.639614	3871.37233	5073.928356	3909.962022	4139.842122
1.08_245.0672m/z	245.0671514	1.078266667	neg	744.5979607	1459.571266	1247.743198	5793.554755	2043.047833	3243.121169	1138.233959	1790.281706	875.5952447
1.08_262.8773m/z	262.8772869	1.078266667	neg	3684.366442	2640.760933	2840.410048	2759.775511	2792.007387	2488.649262	3840.64435	2696.241886	2923.607252
1.08_277.8546n	554.6912342	1.078266667	neg	11414.6993	10594.92107	9060.432544	10535.70438	10969.94162	11059.17879	13805.57332	10868.14041	10232.96599
1.08_281.8629m/z	281.8629221	1.078266667	neg	1186.825314	739.2621981	824.469045	503.0033658	704.2944665	801.9016227	805.8197418	786.3913847	728.5387563
1.08_292.9870m/z	292.9869998	1.078266667	neg	424.0362075	347.0432182	301.1350122	63.77060316	0.001403199	0.001403199	263.3615718	291.6267756	285.798132
1.08_301.0211m/z	301.0211023	1.078266667	neg	1731.69242	1515.477856	1697.962112	1205.54911	852.5299548	943.2381648	1600.295203	1641.484681	1296.327495
1.08_301.8875m/z	301.8875374	1.078266667	neg	1285.326949	1219.362349	1054.657072	1210.659532	1004.462669	999.1510736	1243.394811	1407.685912	1307.68161
1.08_306.8793m/z	306.8793152	1.078266667	neg	1637.128865	1808.613298	1326.499291	1515.124482	1825.715765	1604.658201	2298.329375	1396.720688	1516.71854
1.08_314.9406m/z	314.9406449	1.078266667	neg	1864.472989	2209.070535	2217.380234	1742.686704	1255.00531	1301.95406	1679.192654	1429.104134	1367.138494
1.08_316.8439m/z	316.8439034	1.078266667	neg	1903.115117	1526.049434	1601.759365	2346.586669	2132.005526	2287.096521	2089.352886	1134.641535	1747.393854
1.08_328.8611m/z	328.8611076	1.078266667	neg	35920.9113	30110.67885	31156.07009	29482.84511	34673.82634	36402.31789	38387.41755	32458.06872	37006.05856
1.08_336.8682m/z	336.8682277	1.078266667	neg	2392.133546	977.2599543	832.6538669	1184.154201	2545.144256	695.2474893	1959.628616	1272.633972	1168.676847
1.08_338.8164m/z	338.8164294	1.078266667	neg	40149.87881	34843.05256	32144.49381	34309.89183	38873.64917	37470.30712	41328.72997	36049.24025	39532.52001
1.08_365.0076m/z	365.0076339	1.078266667	neg	824.9280108	482.7497947	606.5674468	649.148229	517.8394916	744.3759557	792.8884797	504.4976165	533.0331129
1.08_368.9357m/z	368.9356776	1.078266667	neg	233.4388861	360.5761628	82.77070785	0.001403199	106.905737	163.4435445	204.4616109	192.9987602	195.6175757
1.08_383.8140m/z	383.814026	1.078266667	neg	7736.022221	6600.797807	5930.534149	5814.92477	6829.694271	6449.915238	7445.414848	6440.205074	6845.090747
1.08_398.7716m/z	398.7716469	1.078266667	neg	1371.744525	1172.813298	907.2569334	503.0957423	655.1988076	854.6928794	1466.72334	947.017128	849.4145716
1.08_449.7858m/z	449.7858079	1.078266667	neg	7491.857322	6383.161576	6150.542833	5836.026414	6892.86096	7213.382568	8557.160266	6481.438748	7185.867411
1.08_457.7578m/z	457.7578005	1.078266667	neg	2766.701417	2440.065184	2285.288959	2321.801716	2417.942599	3255.014212	3228.183254	2877.490059	2355.52556
1.08_460.7633m/z	460.7633353	1.078266667	neg	5845.550684	5117.503694	5424.42098	1983.329622	2792.69378	3198.926302	5963.227879	5008.077329	4678.710889
1.08_470.7922m/z	470.7922278	1.078266667	neg	8787.612895	8094.700877	8372.532997	8296.686366	8870.499268	9814.769881	10044.99254	8040.600856	8764.183173
1.08_480.7474m/z	480.7474306	1.078266667	neg	12807.40488	10743.34234	9828.622953	10384.32748	10284.88399	10131.23164	13390.47589	10449.4906	11102.07378
1.08_484.7465m/z	484.7464609	1.078266667	neg	3762.942173	2761.178077	2692.108688	2578.195129	2931.231332	2569.086509	3386.294645	2816.193192	3017.205091
1.08_504.9434m/z	504.9434298	1.078266667	neg	3032.789609	2671.345189	2986.988086	3567.543445	2862.065538	3480.640165	3048.726601	2984.201052	2614.28649
1.08_548.7351m/z	548.7351483	1.078266667	neg	5834.62521	4747.689316	4319.50974	4281.187239	4122.683609	4994.555089	5483.393973	4539.562927	4358.89041
1.08_616.7229m/z	616.722935	1.078266667	neg	3262.396862	3497.562709	2391.247452	2304.320058	2262.684428	2230.585499	2951.221447	1841.727864	2623.899117
1.10_130.8752m/z	130.8751912	1.10065	neg	433.6494572	316.5706576	317.6960763	164.7593579	163.7243969	169.8680733	394.3955933	330.8034026	285.883627
1.10_138.9070m/z	138.9069796	1.10065	neg	432.1464684	300.9290926	235.6493917	163.3682493	180.6848456	152.5341786	334.0817961	250.5716182	192.3039416
1.10_141.9126m/z	141.9126344	1.10065	neg	447.0381564	340.7170931	316.0859444	207.2550573	216.4322501	177.2239312	381.8862397	267.0364421	294.1075457
1.10_154.0340m/z	154.0340331	1.10065	neg	45.04505091	62.80979708	94.73310688	26.39969356	23.92877374	82.57318188	46.26278753	59.05705166	19.23469494
1.10_162.9206n	324.8281535	1.10065	neg	4411.555891	2839.929534	2743.614642	1331.647552	1866.06093	1996.367674	2999.673568	2509.688838	2575.31381
1.10_178.9778m/z	178.9778218	1.10065	neg	713.8385265	526.4095068	465.8412034	454.3845607	431.6394456	506.6468269	574.9935911	436.112483	554.8948735
1.10_182.8968m/z	182.896781	1.10065	neg	3800.056856	2619.72671	2415.285185	1312.368358	1747.163043	1590.67335	2816.433406	2068.996162	2386.752231
1.10_186.9296m/z	186.9296056	1.10065	neg	70393.8937	60523.66692	61073.35054	57389.85798	72232.39367	68933.83396	75606.69168	66504.17371	75447.90564
1.10_188.9130m/z	188.913024	1.10065	neg	1016.31416	740.9312802	693.6331887	675.196456	662.852104	707.5973172	984.5200573	654.2020139	748.6962207
1.10_193.8773m/z	193.8773204	1.10065	neg	543.7190251	294.5783022	621.4716461	180.5330903	118.9926509	164.2032127	766.1882751	455.5662003	403.9085251
1.10_197.9025m/z	197.90251	1.10065	neg	1107.332526	839.2786262	857.8908325	474.3357845	449.7332145	335.1199831	882.9647335	706.0958748	666.6369768
1.10_198.9365m/z	198.936546	1.10065	neg	1041.340991	870.1819999	833.4550426	1190.79369	1327.519047	906.5757533	1419.454716	1223.577158	908.9018075
1.10_203.9199m/z	203.9198551	1.10065	neg	543.6898641	441.7488484	392.8132206	374.1715705	288.9093086	320.2858756	483.2228804	308.0673097	276.2816117
1.10_205.9062n	410.8025253	1.10065	neg	3046.796556	2260.14089	1813.769902	1440.591543	1475.114451	1976.79837	2564.808257	1677.531245	1590.904179

1.10_213.8754m/z	213.8753642	1.10065	neg	489.3987724	288.6543445	372.8646976	275.8708014	327.3992946	352.1275733	389.6167907	272.0787945	406.0715065
1.10_220.8826m/z	220.8825592	1.10065	neg	751.2693904	635.7130263	524.0474169	542.5577373	474.0561917	557.1039007	713.1087928	548.5684736	494.7713654
1.10_226.9182m/z	226.9181785	1.10065	neg	963.1717516	826.3155097	663.2431032	778.8101315	731.2107786	919.4954059	1046.456497	950.6424564	760.5661956
1.10_232.9439m/z	232.9439245	1.10065	neg	1273.22441	632.2383612	761.0705323	930.2379606	927.9067501	1041.305073	1051.795471	899.7003949	931.7185605
1.10_262.8575m/z	262.8575247	1.10065	neg	1552.523826	990.8315457	1172.649938	1036.848183	1442.202921	1311.452744	1479.63705	1265.770006	1265.980518
1.10_271.8984n	542.7784642	1.10065	neg	7742.953126	6574.677636	5639.436436	4104.691024	4701.079546	5690.397278	7528.319919	5583.221824	6318.528684
1.10_281.8749m/z	281.8749231	1.10065	neg	2220.096706	2136.751198	1591.687274	1277.404027	1117.004034	1041.288478	2157.664333	1531.65836	1613.653406
1.10_283.8903n	264.8724293	1.10065	neg	45860.82006	36782.41214	34209.49798	33984.10899	40219.46086	37798.20342	44364.44324	37538.40013	41391.15061
1.10_308.8643m/z	308.8642586	1.10065	neg	1548.870151	1054.979084	837.4360535	478.0417706	617.662485	722.727536	1055.189774	851.7787552	802.1932276
1.10_311.0500m/z	311.0500486	1.10065	neg	10512.8473	9849.211861	11479.65282	8519.396221	8460.889742	10951.75568	11099.21302	9811.304598	9691.068748
1.10_323.8694m/z	323.8693692	1.10065	neg	4145.630427	4157.127677	3721.081028	3106.740017	3230.43218	3448.295115	4768.855471	4265.855185	3895.559077
1.10_328.0401m/z	328.0401479	1.10065	neg	1120.710181	765.0535148	919.6754242	946.1569864	466.3948144	861.7819663	782.3586813	708.4656479	455.5656818
1.10_330.8649m/z	330.8648707	1.10065	neg	4231.750132	3373.135424	2895.33273	2459.437533	2927.162649	2933.351968	4139.08818	3042.910425	3229.646539
1.10_342.8153m/z	342.8152639	1.10065	neg	4260.672128	2925.699012	2564.089818	2147.495027	2664.39471	2728.967954	3370.092413	2731.441109	3029.903253
1.10_345.8512m/z	345.8511783	1.10065	neg	2756.651859	2825.045752	2285.517728	1676.624231	2190.359324	2044.14379	2944.078411	2620.858324	2506.592368
1.10_351.8780n	332.8601563	1.10065	neg	37377.58254	28579.18479	27341.02075	21776.39667	28593.12696	27811.01852	33541.36477	26744.33399	30296.04525
1.10_368.0310m/z	368.0309671	1.10065	neg	73.43517104	235.5732887	76.624638	236.0364022	176.0408566	79.46407379	162.354134	145.4595394	162.9206249
1.10_380.8631m/z	380.863089	1.10065	neg	4234.160227	2884.080195	3368.357345	1498.761627	1940.634431	2241.673495	3939.916043	3534.02697	3280.902988
1.10_390.8190m/z	390.8189872	1.10065	neg	4972.833785	3820.072217	3403.029673	1999.437387	1986.729341	2291.460629	4196.372629	3403.409446	2974.01815
1.10_396.7750m/z	396.7750436	1.10065	neg	6510.532034	5597.977895	4899.639505	2850.840132	3147.627218	3354.211593	6586.0126	5031.030797	5467.724403
1.10_396.8482m/z	396.8482213	1.10065	neg	16693.64904	14814.71976	14458.00415	12366.84037	15699.76859	15548.76065	18038.12977	15837.79864	16767.39766
1.10_400.8473m/z	400.8472718	1.10065	neg	22168.7149	15439.42296	14198.65408	10780.42744	14390.34059	15056.30207	17911.89299	14107.31786	16707.54226
1.10_406.8036m/z	406.8036325	1.10065	neg	21069.55339	16602.43653	14942.26218	15284.68577	17856.74807	17651.25482	21693.13478	16250.10545	17692.92321
1.10_407.8827m/z	407.882744	1.10065	neg	1759.234519	1262.049909	937.2254482	985.1005782	1463.454911	1082.833059	1795.952422	1144.004567	953.6754665
1.10_416.7591m/z	416.7590669	1.10065	neg	5058.884702	3693.321307	3262.080662	3119.006544	3341.97962	3719.538414	4056.677958	3196.598305	3579.274308
1.10_443.8303m/z	443.8303091	1.10065	neg	5528.439482	3972.144691	4089.820921	2769.553988	3669.817066	3782.067844	5181.557052	4062.955456	4320.338376
1.10_451.8021m/z	451.8020741	1.10065	neg	2417.195244	1678.991433	1740.026965	1642.454021	1180.16391	1761.526169	2218.923339	1933.194947	1486.472181
1.10_452.9814m/z	452.9813952	1.10065	neg	2095.018645	1456.417756	1900.430831	2088.195791	1163.753697	1852.284904	2002.34982	1931.120972	1326.673116
1.10_454.8072m/z	454.8071919	1.10065	neg	2317.866959	1954.120311	1575.948258	694.4789616	1271.050511	993.7717776	2151.053914	1585.455246	1732.573778
1.10_464.8356m/z	464.8356132	1.10065	neg	8732.483245	7369.149145	6931.553621	5755.177296	7930.7542	6954.520654	9442.850747	7198.656871	8266.113972
1.10_468.8347m/z	468.8347139	1.10065	neg	12344.17946	8774.679724	8771.962607	6838.691207	7973.742752	8413.734904	11435.78184	7829.45233	9923.6155
1.10_472.8339m/z	472.8339017	1.10065	neg	2236.795569	1724.186882	1161.045464	1006.714031	782.8995291	830.9225225	1608.955832	1148.549863	1050.570563
1.10_474.7911m/z	474.7911214	1.10065	neg	11665.75644	9450.392693	8587.486328	6798.344068	8123.670225	7483.410642	10485.70499	8040.199642	7809.358686
1.10_511.8175m/z	511.8174633	1.10065	neg	2328.390115	2031.917599	1497.372084	1362.327898	1869.085434	1593.220567	2100.050233	1037.546188	2190.456878
1.10_517.7737m/z	517.773679	1.10065	neg	3117.296785	2250.16718	2010.034419	1379.648128	1348.948005	1689.634499	2614.834912	1908.449524	1508.720901
1.10_526.7929m/z	526.7929382	1.10065	neg	1273.911668	1429.776717	1216.302724	563.9497919	562.4286468	874.2188783	1502.038869	1227.33988	834.9842315
1.10_532.8229m/z	532.8228758	1.10065	neg	4666.062775	3218.415614	3524.063198	3362.923808	3117.170744	3672.69859	4650.722037	2850.362032	3671.061685
1.10_538.7799m/z	538.7799141	1.10065	neg	3386.251497	2961.528971	3058.743328	2684.175482	2944.915333	3464.29026	3802.425118	3544.720992	3044.301742
1.10_604.8100m/z	604.8100109	1.10065	neg	4173.97552	2756.249842	2563.165458	1891.281456	1838.030723	1972.970114	3121.132869	1794.499312	2414.185255
1.10_610.7668m/z	610.7668063	1.10065	neg	3366.510938	2924.757914	2101.051601	1646.560171	1396.62468	2500.369283	2487.660015	1923.694746	1883.585512
1.12_102.9488m/z	102.9487723	1.11925	neg	175.8401145	129.0288185	108.9372151	84.10373696	103.4310039	112.0692816	117.9405128	114.2985567	93.90245326
1.12_158.9346m/z	158.9346411	1.11925	neg	232.6778772	182.70591	156.9169103	188.5214241	127.4773166	202.8194986	190.7687606	191.8829193	144.4529198
1.12_179.9365m/z	179.9364749	1.11925	neg	603.8168271	371.0588664	383.9106636	326.1718638	256.292595	367.477074	506.6251398	325.7350115	236.0966636
1.12_244.0262m/z	244.0261691	1.11925	neg	516.5461805	393.2739618	466.7523491	99.55427454	269.0060603	208.3235257	407.7910834	488.3664571	504.5768223
1.12_248.9000m/z	248.8999999	1.11925	neg	1180.252299	953.5250997	983.9775005	978.0796078	864.3088634	932.3340653	1302.806177	1015.582576	955.8955575
1.12_255.0100m/z	255.0100074	1.11925	neg	2379.349526	1296.962195	1272.341772	1971.840943	2017.714744	2598.156389	1361.877423	1760.7828	1627.80617
1.12_260.9664m/z	260.9663607	1.11925	neg	3061.589908	2566.420008	2362.568233	3524.19368	3570.314299	4011.937875	2621.504501	2937.233448	2911.575182
1.12_267.9291m/z	267.9290685	1.11925	neg	312.4470751	250.9021041	370.1310771	337.7686977	286.2819224	248.5057453	414.2277817	370.5539227	513.8239165
1.12_283.0509m/z	283.0509007	1.11925	neg	476.2789791	377.9390146	419.7450079	380.4407527	402.5406793	493.5305352	297.4874941	554.6423721	389.2859951
1.12_292.9038m/z	292.9038487	1.11925	neg	3944.95282	3117.96498	2563.320797	4327.218581	3719.43831	4149.895695	2548.869112	2949.120336	2827.597221
1.12_295.9882m/z	295.9881881	1.11925	neg	856.044021	823.5433752	741.2699894	368.43827	507.0167829	485.1164228	812.2688943	703.3033547	896.7480082
1.12_305.8760m/z	305.8759708	1.11925	neg	1347.956987	990.7012095	851.0768456	591.2530711	631.6458995	787.4897368	1086.855011	779.7936273	651.0024273
1.12_306.9721m/z	306.9721352	1.11925	neg	2836.270833	1824.33207	1746.518703	3677.540038	3556.923547	4472.111711	1444.683826	2947.32605	2441.517568
1.12_307.0222m/z	307.0221872	1.11925	neg	1058.923378	1284.510897	1205.887777	1204.942808	999.8946507	1355.416765	1307.595754	1407.233475	1316.434734
1.12_322.9411m/z	322.9410945	1.11925	neg	1878.200122	1172.641541	1359.338321	850.9187709	1402.92251	1549.32517	1239.421651	1716.48061	1951.00193
1.12_322.9976m/z	322.9975847	1.11925	neg	1821.785913	1078.794747	840.3414374	1755.208427	1891.182794	2026.726313	1055.453946	1495.864814	1610.069562
1.12_328.9540m/z	328.9540267	1.11925	neg	5001.810911	3731.0664	3419.408617	5826.190539	5753.192626	6851.545918	3213.812151	4879.480639	4685.729448
1.12_332.9697m/z	332.9697396	1.11925	neg	4028.835348	2580.445459	2414.959289	858.566669	2680.446229	1584.194725	2737.339606	2591.274975	3557.063613
1.12_339.8949m/z	339.8949146	1.11925	neg	3270.502503	3180.400517	2349.724739	2843.777265	2583.275796	2689.318705	3598.582642	3091.326418	3056.055174
1.12_342.9985m/z	342.9985467	1.11925	neg	2960.33341	2116.216267	1652.07684	1079.916525	1730.81667	1770.735458	1571.36836	1806.105267	2349.679565
1.12_345.0026m/z	345.0026078	1.11925	neg	308.0909188	110.4806118	186.6614973	71.09053392	162.1089127	83.23144016	72.36621409	329.3343673	163.582939
1.12_349.9601m/z	349.960113	1.11925	neg	403.1421023	158.7818306	260.320834	238.5616714	318.9399351	310.9136693	188.7612759	361.8754059	372.6272784
1.12_377.8577m/z	377.8576942	1.11925	neg	2702.452587	2257.344692	1997.307626	1647.014183	1570.007125	1478.372557	2015.991607	1720.08735	1572.236303
1.12_380.9156m/z	380.9156434	1.11925	neg	12504.34324	10917.36514	10917.81019	14371.76262	16530.77801	15968.26929	12120.1156	12744.77494	12625.11664
1.12_385.0051m/z	385.0050914	1.11925	neg	1573.284765	806.5551915	780.5640473	1593.910562	1556.006961	2076.481266	1256.648336	1920.625036	1716.234957
1.12_396.9413m/z	396.9413188	1.11925	neg	2374.175851	1658.973291	1446.551014	2076.208326	2583.989963	3016.150703	1445.622701	2300.719448	2400.517271
1.12_402.8979m/z	402.8979426	1.11925	neg	2533.996935	1786.905141	1655.679087	3377.33731	2748.048636	3376.387179	1409.678902	2287.390141	2040.555604
1.12_453.8504m/z	453.8503807	1.11925	neg	1145.184212	515.7341771	805.5695529	636.4874113	722.5400398	711.2866647	660.7534602	369.4388046	659.6794629
1.12_600.8110m/z	600.8109801	1.11925	neg	1789.417007	1238.25083	1297.038949	915.9228001	1002.552089	1124.173088	1207.004773	995.3941803	1168.08104
1.14_119.0315m/z	119.0315087	1.1382	neg	6.477579376	0.001403199	0.001403199	18.99778639	15.91642037	16.70828227	0.001403199	11.57125521	0.001403199
1.14_159.0145n	317.0175509	1.1382	neg	9589.549932	7789.570971	6573.793175	12004.71468	10419.94062	13014.20152	6920.216891	10555.37524	9451.324214
1.14_218.9599m/z	218.9598833	1.1382	neg	3491.691397	2471.533787	1876.158568	3297.633437	2803.146759	3463.485142	2470.140205	2593.26765	2288.328871
1.14_231.0484m/z	231.0483839	1.1382	neg	141.8792192	146.9686106	78.71074524	513.010474	211.2656929	411.9835495	271.1492657	174.2105289	143.540186
1.14_248.0666m/z	248.0665897	1.1382	neg	0.001403199	0.001403199	0.001403199	840.6602528	56.82337203	283.4887403	0.001403199	0.001403199	0.001403199
1.14_261.9258m/z	261.9257985	1.1382	neg	0.001403199	0.001403199	0.001403199	144.1144685	113.7802481	122.5170293	0.001403199	105.3841534	0.001403199
1.14_271.9257m/z	271.9257498	1.1382	neg	69.99940908	0.001403199	0.001403199	70.47264393	164.8997497	69.83721383	0.001403199	124.1626434	169.1793343
1.14_309.0455m/z	309.0455093	1.1382	neg	5283.794382	8851.786182	6784.956846	14171.16604	2959.413644	4903.402156	7951.347607	3330.582645	2439.809268
1.14_317.1105m/z	317.1105165	1.1382	neg	796.8526601	329.2655962	182.4274786	5095.870453	1452.996065	3733.146567	67.74635114	1555.642097	710.6497408
1.14_333.0439m/z	333.0439218	1.1382	neg	5030.506535	4587.658645	5225.842468	6052.281044	5461.281754	6223.667121	4838.831181	4863.562101	4665.638939
1.14_360.8916m/z	360.8915846	1.1382	neg	1157.86604	527.8243668	831.6587189	1020.46842	1003.813316	1211.828857	529.0834007	960.6052362	722.5749143
1.14_360.9299m/z	360.9298618	1.1382	neg	632.1827543	529.9323548	308.0365145	467.32764	641.4746292	795.0336147	891.9278972	627.8874413	693.9852787
1.14_363.9741m/z	363.974094	1.1382	neg	656.1847974	617.4936124	824.8813884	943.1852438	379.7510356	819.8096	640.7441621	548.433122	406.5246907
1.14_476.9806m/z	476.9805526	1.1382	neg	9798.204183	22478.11695	16015.2097	14570.76817	10505.68472	11416.6488	18097.09816	8792.350864	11803.32805
1.14_486.9363m/z	486.9363237	1.1382	neg	8646.160368	10488.06598	14153.49722	9992.817442	12446.80181	11431.8235	11547.61745	7417.870146	13533.90317
1.14_525.0593m/z	525.0593127	1.1382	neg	2352.602409	5793.319736	6699.389702	4488.85023	5434.688465	4026.16023	3928.076562	3378.572212	3938.448884
1.14_553.8913m/z	553.8913071	1.1382	neg	676.7177545	2427.506483	1848.399078	729.5680849	588.0352104	312.4171326	3065.540513	954.5772301	0.001403199
1.14_556.9973m/z	556.9972861	1.1382	neg	3389.731848	4735.345889	4622.96448	1522.643008	3603.160443	3984.285837	4241.003321	1328.210205	3252.278065
1.14_620.9288m/z	620.9287624	1.1382	neg	2095.66083	4834.273204	2632.161377	4739.817712	4173.798698	3409.849418	6672.699566	2298.131378	2366.633726
1.14_676.9468m/z	676.9468108	1.1382	neg	2186.627478	4006.783194	3660.822394	1911.697993	3166.640785	3333.42057	3583.802993	2127.575684	1963.008822
1.14_717.0871m/z	717.0870745	1.1382	neg	1590.409122	755.909314	4033.689666	4342.576853	7690.43649	3274.557204	2484.658663	0.001403199	3059.52279
1.16_124.0149m/z	124.0149485	1.160166667	neg	782.35593	1196.54888	1115.510432	984.2853008	197.0593883	657.7350492	336.2528217	736.4033966	156.2178617
1.16_152.0296m/z	152.0295653	1.160166667	neg	454.4835752	419.4859092	528.0065742	464.0057167	777.1302667	745.9211586	467.1306065	513.2967098	667.3116835
1.16_168.0244m/z	168.0244194	1.160166667	neg	20796.28992	29205.39144	30426.82239	28134.63821	25958.213	25111.13357	28147.64915	21448.94982	26506.02328
1.16_168.0284n	335.0495788	1.160166667	neg	745762.9207	1037440.218	1164607.407	894120.3099	1004188.928	849252.7977	1023730.824	757359.5037	977863.0891
1.16_203.9973m/z	203.9972742	1.160166667	neg	1983.62478	2006.727743	2134.142093	251.5599047	626.9939778	864.0649472	751.2444683	551.2577648	689.8693392
1.16_214.0092n	427.0106527	1.160166667	neg	39372.97544	39972.36767	45628.24312	43256.37378	47734.80675	45413.05549	46277.81177	43718.437	47040.18291
1.16_224.9797m/z	224.979702	1.160166667	neg	2228.30514	2585.898418	1944.881504	2086.425072	930.9889773	1293.461745	2556.512732	1753.218205	1576.202057
1.16_230.0169m/z	230.0169182	1.160166667	neg	1093.936184	2001.559084	2097.32724	1910.052168	817.2923625	1227.470145	1379.83535	1186.737106	743.1838193
1.16_235.0086m/z	235.0086123	1.160166667	neg	11135.10708	14836.12825	12587.07197	13349.75445	9332.627031	10266.63608	14155.83499	8925.03119	10010.06241
1.16_243.9890n	288.9873051	1.160166667	neg	16841.00709	19621.07982	22728.42537	26587.96858	26305.36049	25279.35041	23532.98588	26405.72782	27477.69629
1.16_251.9987m/z	251.9987421	1.160166667	neg	5476.510752	6345.873363	5896.497557	6545.435156	5266.223485	5839.999621	6145.970404	5532.246788	5356.864127
1.16_257.9980n	302.9965088	1.160166667	neg	7178.760311	7756.789485	6955.157092	7834.865076	5220.001183	5062.777652	7579.657418	5015.788029	5197.811592
1.16_264.9885m/z	264.9884727	1.160166667	neg	111725.3774	152043.3483	187217.2711	200831.652	199444.4097	171069.9835	165301.0477	154616.555	185480.035
1.16_266.9844m/z	266.9843814	1.160166667	neg	5142.566642	6103.452703	8561.115687	8689.47063	8401.135669	7814.823391	8463.691743	7133.138466	7853.721858
1.16_267.9536n	312.9517514	1.160166667	neg	7822.66143	10073.8778	8993.383905	9316.440949	2454.602608	5404.115207	3861.618678	5792.639633	1873.769618
1.16_268.9974m/z	268.9974293	1.160166667	neg	2788.737984	2939.315543	2798.488413	4144.210089	4613.127594	4694.969975	3002.705031	3173.940928	3088.245803
1.16_276.9290m/z	276.9289714	1.160166667	neg	521.6070814	466.0122504	356.7557383	50.95493562	335.4929727	182.7844692	474.6258956	249.6249097	358.8096227
1.16_293.0064m/z	293.0063669	1.160166667	neg	2227.909958	2570.079728	2100.214662	3846.067019	3930.233564	3155.039254	2981.089646	3164.14711	2968.565751
1.16_309.9598n	354.9578499	1.160166667	neg	9976.958427	11634.0782	9531.362187	13711.88994	6504.578489	10003.21076	11929.25128	7723.367319	7381.298538
1.16_310.9693m/z	310.9693246	1.160166667	neg	120585.915	142380.9523	148939.8337	183869.2613	170698.6484	172045.7097	157422.4275	154778.7284	167584.899

1.16_315.8996m/z	315.8995608	1.160166667	neg	461.9456258	269.3622104	1136.486504	628.7711885	201.7775651	1408.677878	236.4421502	511.8783766	134.7697462
1.16_319.0477m/z	319.0477164	1.160166667	neg	183.1781196	380.4125578	1746.775634	3782.629345	3431.848125	4154.26251	1494.992788	2553.036719	2113.896407
1.16_332.9514m/z	332.9513546	1.160166667	neg	44591.1908	52541.42245	50157.66656	57806.92812	59294.4566	58701.5387	58272.26343	52774.77683	55755.54989
1.16_334.9337m/z	334.93368	1.160166667	neg	2092.843252	2569.378589	1410.449655	1817.814704	303.6572739	1130.336118	1156.71978	603.6186644	424.3987527
1.16_346.9406m/z	346.9405817	1.160166667	neg	1937.291687	2016.502436	1596.373045	1702.087475	2482.267889	2336.536076	2081.686575	2665.599786	1990.515233
1.16_350.1097m/z	350.1097104	1.160166667	neg	1322.003591	1273.366124	1576.661683	1531.266936	1650.844368	1164.113973	1343.421326	1714.798345	933.6702437
1.16_357.0315m/z	357.0315417	1.160166667	neg	66861.47153	102100.4014	99165.77826	63787.51436	70501.74172	58498.53654	86473.09823	56465.88767	81054.43131
1.16_364.8889m/z	364.8888709	1.160166667	neg	20502.39193	20030.24609	18164.90584	18254.86624	18300.10494	18815.07834	20273.0798	18334.69879	18590.30502
1.16_376.9399m/z	376.9399224	1.160166667	neg	3352.714439	4294.282844	2022.276316	4230.339365	2639.127736	3053.635789	3624.345083	3086.511123	2702.432524
1.16_386.0333m/z	386.0332614	1.160166667	neg	1265.289837	2141.106777	1516.111205	624.2583336	1176.604534	826.6628497	1386.758194	791.5138874	1088.664946
1.16_387.0351m/z	387.0350978	1.160166667	neg	809.4187449	1537.14097	1887.236168	1388.986932	1642.54765	2412.839944	1500.289499	1572.324816	1922.450076
1.16_402.9208m/z	402.9207787	1.160166667	neg	2079.053288	1109.362805	1015.650968	1320.165602	540.046155	518.7926637	985.1879285	313.3807373	639.1591961
1.16_406.9196m/z	406.9195935	1.160166667	neg	11923.04917	16493.54172	12809.38354	20824.72613	13560.17552	15049.17017	15807.37858	13826.25675	13195.75533
1.16_409.0179m/z	409.0179134	1.160166667	neg	1133.235473	1539.582164	1155.879625	1270.233541	637.8253747	1050.477165	654.3711783	870.3342285	0.001403199
1.16_411.9598m/z	411.9597997	1.160166667	neg	18231.60491	15999.19417	23998.44911	20375.33634	19328.36288	18140.42451	19385.01122	19009.33517	21538.66368
1.16_412.9676m/z	412.9676305	1.160166667	neg	7845.422059	6878.201447	9861.62641	6451.631596	7070.532029	7121.244253	7488.496463	7618.476611	9056.830714
1.16_416.8751m/z	416.8751394	1.160166667	neg	2742.186138	4060.229096	3917.342684	4625.094602	5088.94503	3280.06197	3105.725654	3337.236565	4578.034567
1.16_424.0493m/z	424.049331	1.160166667	neg	9326.132428	9862.452966	11589.95834	10260.38783	10816.85263	11631.95695	8072.051329	9102.443359	7488.958724
1.16_428.9017m/z	428.901703	1.160166667	neg	4402.204574	4967.403376	3588.749832	8057.34601	3171.115714	3892.81231	5172.779161	4243.243468	4174.254453
1.16_430.9182m/z	430.9181667	1.160166667	neg	20029.82607	25646.90077	22087.49162	31653.40501	30213.43365	27804.6191	28782.40207	26810.38377	27353.99353
1.16_433.0165m/z	433.0165299	1.160166667	neg	3559.115718	4773.61467	11816.6295	7974.730005	11128.03115	9484.58905	7517.218919	8225.372421	6821.37559
1.16_435.9586m/z	435.9585928	1.160166667	neg	14075.64288	12574.64437	15520.7222	19994.72586	16338.19617	21254.70366	13476.45332	16340.84883	20844.99658
1.16_448.9926m/z	448.9926212	1.160166667	neg	5939.709093	7393.432498	7788.086342	6256.424985	8787.89558	7130.082122	8646.206025	7703.996967	7601.258473
1.16_452.9005m/z	452.9004555	1.160166667	neg	10857.66121	12897.60814	11605.35091	17895.00497	16200.85802	15140.00706	14379.58071	13034.92618	14696.5817
1.16_455.9833n	500.9794903	1.160166667	neg	28401.32111	32171.53645	35446.33867	35277.34299	37961.97175	36305.07098	35726.35726	29852.92609	36378.13526
1.16_457.9407m/z	457.9407357	1.160166667	neg	5227.935955	5643.08916	5746.972489	4439.57836	3125.268707	5770.647338	4828.967037	5540.698569	2705.414212
1.16_477.9656n	522.9613354	1.160166667	neg	13445.68042	18544.78056	16914.7327	12619.64966	7330.614028	12519.02141	9446.629993	11509.2631	5482.500254
1.16_478.9971m/z	478.9970619	1.160166667	neg	8245.046743	9277.247435	11835.1143	12817.62298	12443.43354	10199.21965	10492.46732	12551.91583	13250.89803
1.16_524.9780m/z	524.9780399	1.160166667	neg	10403.73085	14937.8498	15096.40161	18266.47728	17285.18652	18979.18109	17555.11019	16794.64574	16474.79557
1.16_529.9530n	574.9478132	1.160166667	neg	12634.46575	19264.3139	20078.87074	20278.6005	22363.79486	18206.91566	19570.16465	15609.63647	17481.5797
1.16_531.9084m/z	531.9084268	1.160166667	neg	3951.193997	5281.091717	7165.855289	6611.883496	7681.571914	5591.235864	5444.631925	6759.897569	5914.821176
1.16_532.9170m/z	532.9169673	1.160166667	neg	7114.242543	7845.724852	7137.292567	10430.62512	7110.226937	8131.539084	10174.29233	7611.381775	7123.090123
1.16_546.9603m/z	546.960269	1.160166667	neg	7763.993671	7351.667501	8755.602409	8386.960536	10510.84444	8964.135935	10334.56826	7494.015152	10225.25213
1.16_551.8714m/z	551.8714317	1.160166667	neg	0.001403199	219.458907	0.001403199	0.001403199	378.5360957	0.001403199	0.001403199	274.1828613	373.9287233
1.16_552.9660m/z	552.9660162	1.160166667	neg	5412.014642	6759.497089	8628.401099	10096.04743	11262.21349	9762.003008	7090.084071	7696.84668	8859.390686
1.16_555.9074m/z	555.9073648	1.160166667	neg	3186.513993	5136.73003	3842.477932	6212.329694	2581.700069	5598.270071	4820.423969	4639.361975	2360.77352
1.16_577.8897m/z	577.8897012	1.160166667	neg	3637.524902	3020.76522	4842.150973	4412.147256	3863.921305	5098.052934	3664.279168	5741.356622	5203.030707
1.16_593.0225m/z	593.0224857	1.160166667	neg	5128.355549	5160.722719	5587.86991	2546.860823	2125.277752	2744.542593	6089.764044	2003.387146	3632.501219
1.16_595.0392m/z	595.0392317	1.160166667	neg	10751.90877	10031.05853	14045.44911	15003.53248	17304.69601	15001.08472	9292.977911	14512.79958	18025.54262
1.16_598.9471m/z	598.9471125	1.160166667	neg	7504.6322	6986.010672	6606.261052	8842.534114	10450.33217	9659.288889	9805.056011	8354.300324	6070.198999
1.16_599.8721m/z	599.8720951	1.160166667	neg	440.3229699	1036.832775	569.7570038	460.9149662	1118.589996	373.8043465	957.4360524	1276.619622	492.3364528
1.16_600.0135n	645.0093746	1.160166667	neg	11772.26283	16119.54501	18483.37784	9795.05947	15545.07935	12459.95284	17705.50374	11481.01135	16274.62987
1.16_602.9783m/z	602.9782888	1.160166667	neg	3573.653617	4011.476581	5288.957813	3369.071533	3069.418324	5174.885599	4184.478376	2708.641235	4159.344832
1.16_603.9866m/z	603.9866324	1.160166667	neg	7739.820918	3900.280774	8475.412334	12223.91908	15211.19375	10373.85928	7067.07113	12060.36003	11274.13788
1.16_623.0272m/z	623.0272215	1.160166667	neg	20125.29648	25808.87296	46918.95038	28423.68565	52856.16791	34831.93935	32880.9083	32663.3045	33681.08197
1.16_625.9683m/z	625.9682821	1.160166667	neg	6590.279203	4440.47645	10374.45598	9586.333451	9989.268432	10109.45134	7867.252568	6731.30542	9217.891373
1.16_647.0266m/z	647.0265663	1.160166667	neg	1500.28857	2379.812372	5016.962576	5135.445221	0.001403199	4402.313534	755.4062795	5778.83783	0.001403199
1.16_669.0081m/z	669.0080793	1.160166667	neg	10316.26821	10740.23618	11339.43133	14405.08896	16911.56075	13756.74747	11811.80167	10236.90624	17724.01522
1.16_723.9358m/z	723.9358062	1.160166667	neg	2116.58639	948.1277054	1961.401047	1616.055325	0.001403199	3585.663944	2522.332863	3139.614868	2218.328204
1.18_140.9959n	280.9892426	1.180366667	neg	62146.71969	62513.66526	60950.21599	68720.46571	72493.6186	73414.23547	74967.32931	65076.63447	68859.36414
1.18_144.9594m/z	144.9593547	1.180366667	neg	168.2252952	204.5048759	155.398797	114.8888646	73.41534885	163.361911	67.78523471	191.4279824	104.7005722
1.18_173.0199m/z	173.0198626	1.180366667	neg	548.4886655	777.2527918	969.4837975	1027.376737	1694.079088	2371.545771	0.001403199	460.9688721	204.4884516
1.18_181.0561m/z	181.0561224	1.180366667	neg	140.5059325	111.0152236	183.8329156	132.59471	200.867661	164.4080287	185.2483385	179.5349461	173.5580691
1.18_189.9982m/z	189.998194	1.180366667	neg	233.6969465	118.536887	113.0926031	0.001403199	0.001403199	57.87474786	0.001403199	59.69253731	0.001403199
1.18_192.9813m/z	192.9813158	1.180366667	neg	548.589485	455.7117992	288.3330111	151.5119679	0.001403199	26.17610291	105.2518967	70.074036	0.001403199
1.18_196.0228m/z	196.0228317	1.180366667	neg	12066.60444	11715.4806	10593.43135	9653.105741	10028.09018	10727.56422	11109.48964	10608.54323	10602.88729
1.18_227.9602m/z	227.960239	1.180366667	neg	1927.708469	1862.415064	1637.351017	1340.578457	596.59992	1418.84918	948.1163651	1370.494404	616.2398993
1.18_241.0444m/z	241.0444316	1.180366667	neg	1411.168134	1313.276961	1074.90636	1156.001715	1637.436856	1360.256186	1580.038307	1419.767525	1428.494691
1.18_247.9848m/z	247.9847727	1.180366667	neg	23215.00395	24237.21939	24162.37859	25760.2719	26977.01136	26468.17411	24025.61304	27975.82486	27350.57747
1.18_248.9783m/z	248.9782863	1.180366667	neg	1238.304603	2199.123413	1657.778872	1712.909226	1846.710258	1364.121467	2417.137794	1801.766109	2158.466654
1.18_250.9756m/z	250.9756227	1.180366667	neg	424.6621157	212.3854517	540.8237877	222.8579004	443.3322087	428.2818062	693.4034666	666.3231155	410.437437
1.18_264.0103m/z	264.0102687	1.180366667	neg	3490.336579	3657.652605	3371.626325	2964.787513	3554.501879	3222.480648	3184.391823	2843.777523	3493.326765
1.18_270.0597m/z	270.059677	1.180366667	neg	1545.52878	1314.003947	1820.23387	1286.535136	753.0591038	1429.120754	975.7909825	1670.823006	572.5759022
1.18_270.9605m/z	270.9605019	1.180366667	neg	5307.854854	4499.631921	4648.682666	4349.206571	4366.406323	4560.605892	5621.890636	4949.807038	4457.375176
1.18_270.9740n	315.9723336	1.180366667	neg	5042.671209	5706.936582	4942.553732	4997.070802	5120.860476	5845.39753	5366.479375	4952.067116	5121.340419
1.18_275.9974m/z	275.9974399	1.180366667	neg	2144.260491	2032.019588	2068.056664	1665.271177	1682.702026	1963.017945	2412.839484	1721.615778	1850.592405
1.18_279.9221m/z	279.9221274	1.180366667	neg	1564.826879	1629.773232	1562.655627	1727.74072	686.0249356	1585.612534	767.7870668	1782.396019	585.0854355
1.18_295.9477m/z	295.9477257	1.180366667	neg	2064.47334	1871.473956	1591.703527	1173.208087	1545.222443	1497.508374	1747.915082	1478.152853	1576.571273
1.18_312.9268m/z	312.9268293	1.180366667	neg	5405.336141	5437.890466	4813.114989	5304.816046	5279.613844	5363.567188	5558.792638	4646.152851	5878.925367
1.18_331.9981m/z	331.9980646	1.180366667	neg	2707.730666	2076.553088	2114.592502	1916.958577	2151.404056	2117.848972	2075.552765	1843.99649	2016.716227
1.18_342.9069m/z	342.9069154	1.180366667	neg	5730.266203	7242.298679	7495.957597	7630.65379	7826.526175	6401.367258	7183.550303	6578.865454	7850.831333
1.18_343.0519n	342.0444993	1.180366667	neg	18454.06687	21671.00583	22825.72329	22116.81698	22368.59	21594.96619	17662.7153	18335.85272	18883.59432
1.18_348.9767m/z	348.9766888	1.180366667	neg	6971.039117	7139.815918	7009.513551	5915.943472	7012.819673	6876.997964	8473.62762	7032.678693	6955.677688
1.18_354.9334m/z	354.9334023	1.180366667	neg	16362.95015	17133.40856	14368.93199	19056.84254	19603.31546	18272.47659	18719.81487	16918.55383	17814.53456
1.18_355.9088m/z	355.9088273	1.180366667	neg	2716.162383	1999.42791	2280.46279	1950.069065	1375.377654	2261.644202	1505.389477	2063.506525	1200.022222
1.18_357.9245m/z	357.9244759	1.180366667	neg	12855.4792	11916.80711	11567.23371	11750.6842	12482.85294	12690.65416	12990.29817	10893.93047	11990.24356
1.18_364.0260m/z	364.0259924	1.180366667	neg	5683.054688	5247.186761	5939.043309	5176.146934	6062.715453	5687.484499	6067.71392	4886.416654	5551.639645
1.18_367.9341m/z	367.934066	1.180366667	neg	4139.430442	4073.317525	3864.913575	4606.752406	4974.57055	4643.957869	3955.693536	4390.377289	3818.859767
1.18_389.9159m/z	389.9159128	1.180366667	neg	2215.502868	2064.130169	1328.692652	2460.844457	197.6881905	797.3257839	1286.40953	518.5113525	124.4937448
1.18_405.9413m/z	405.9412735	1.180366667	neg	2056.782617	1492.102952	1518.608627	1846.16765	2164.549738	2127.184753	1582.019408	1516.985509	1964.7746
1.18_421.8534m/z	421.8534447	1.180366667	neg	1151.939595	725.4542414	695.0041213	819.5239955	665.4930129	1165.66876	1158.995245	1127.450734	787.8730147
1.18_432.0133m/z	432.0132562	1.180366667	neg	2313.238147	2329.22217	2432.512483	2249.168952	2584.615069	2710.644245	3014.150988	2348.70431	2402.153195
1.18_528.9500m/z	528.9500209	1.180366667	neg	645.2377532	1192.061663	1448.413935	1282.20303	1279.068953	872.7649707	1211.500704	427.8769073	1470.77159
1.20_151.0013m/z	151.0012693	1.202333333	neg	329.7269961	341.2968831	442.4697801	298.5428201	294.8091834	320.1942146	320.4505424	344.4256697	397.3170793
1.20_173.0031m/z	173.0030919	1.202333333	neg	326.6119433	231.7649031	282.2609481	398.3829587	287.0119249	230.7522547	263.6578147	414.3922544	283.6170605
1.20_198.8849m/z	198.8849262	1.202333333	neg	132.3590837	312.730391	114.7570718	83.55153813	390.4251526	222.3352248	299.0821044	216.0410469	404.6300746
1.20_213.0494m/z	213.0494287	1.202333333	neg	382.8238648	682.3745099	606.4957985	663.4505286	679.3846542	1008.921571	544.2712439	598.6315117	652.7846656
1.20_218.0283m/z	218.0282675	1.202333333	neg	620.9128332	856.3466397	877.9919296	1197.694171	686.1517652	1095.319651	565.7979209	719.5956484	406.9886238
1.20_218.9889m/z	218.9889271	1.202333333	neg	3769.765769	4061.536033	4216.731946	2904.526119	3491.466487	3473.588375	3793.873514	3657.985145	3613.307547
1.20_226.9285m/z	226.9285479	1.202333333	neg	497.625551	437.2009937	254.4697902	290.0030055	181.5925715	188.8908428	287.4716667	248.5995379	87.53299304
1.20_228.0492m/z	228.049178	1.202333333	neg	4198.16954	4851.410227	4313.064144	5221.100728	5301.468259	5617.269887	5155.896075	4669.503769	5636.479174
1.20_239.0352n	238.0255262	1.202333333	neg	832.2989302	1039.845271	900.6106761	933.3711216	1120.05705	1019.139862	1205.220579	1212.983249	1271.31913
1.20_240.9969m/z	240.9968745	1.202333333	neg	3333.39336	3409.269799	3827.126791	2691.09622	2991.487513	2867.510265	3114.782979	3187.72143	3259.200625
1.20_248.0542m/z	248.0541958	1.202333333	neg	10970.19823	11028.84082	10663.45767	13014.45587	12779.09207	14025.36284	13593.59142	13220.97426	13707.75887
1.20_254.0038m/z	254.0037565	1.202333333	neg	0.001403199	59.43064632	41.09254845	106.057078	0.001403199	43.73985092	41.26586037	0.001403199	51.57784197
1.20_262.9787m/z	262.9787057	1.202333333	neg	9823.782811	10294.36715	10468.7073	8003.667579	7992.323517	9263.707404	10341.48697	9659.662697	9833.200144
1.20_271.0435n	316.0417331	1.202333333	neg	10011.18075	11100.73279	12165.17244	10237.04174	6306.540915	10258.25438	7030.404394	9786.912523	6580.539363
1.20_284.9871m/z	284.987101	1.202333333	neg	604.5641972	682.3084119	669.1344242	524.8690871	201.0043877	490.9672418	485.9121982	491.3038902	389.3492429
1.20_285.9641m/z	285.9641406	1.202333333	neg	1377.484359	1877.167997	1489.93501	2047.87252	2106.114539	2104.149516	1454.653758	1622.442925	1591.218977
1.20_292.9589m/z	292.958854	1.202333333	neg	18410.31755	21240.33016	21280.41543	20178.31413	18908.63403	19248.78608	18928.51743	21159.25946	20326.19382
1.20_296.0367m/z	296.0366677	1.202333333	neg	1964.23058	1858.706594	1493.003077	1812.470554	2262.033742	2138.121733	1757.690713	1702.811216	2167.02318
1.20_297.9796m/z	297.9795615	1.202333333	neg	1250.919507	1555.691243	1582.086344	1420.74176	1178.616193	1580.970316	1442.354045	1768.967394	1170.974103
1.20_300.0163m/z	300.0162914	1.202333333	neg	2897.796667	3333.140042	3192.444457	5276.591018	4991.931223	5312.988174	4235.736057	4213.46455	4971.231373
1.20_303.0003n	347.9985477	1.202333333	neg	1238.072684	1586.738	1657.26638	1741.57542	965.0226328	1927.831711	1361.894856	1435.159	921.3560243
1.20_314.9409m/z	314.9408717	1.202333333	neg	19876.576	19790.53885	20165.23331	18338.96274	17575.61013	18011.16218	19545.92391	19556.60585	19800.90682
1.20_320.9878m/z	320.9877769	1.202333333	neg	241.6088192	338.9521522	404.412829	225.2738555	405.7548731	296.9053026	330.9958035	234.0795214	380.1160188
1.20_323.0055n	368.0036571	1.202333333	neg	3474.598338	3158.364437	3452.551515	3912.696295	4552.768117	4395.609987	4328.560736	4229.327527	4796.252782
1.20_336.8682m/z	336.8682003	1.202333333	neg	0.001403199	0.001403199	346.2241174	1414.622627	755.574088	1006.813159	0.001403199	471.5773621	304.9419046
1.20_336.9228m/z	336.9228381	1.202333333	neg	8302.578868	8738.537282	9052.991842	7217.712754	7960.211247	7899.249957	8362.755517	8933.95947	8473.677107
1.20_337.9546m/z	337.9545984	1.202333333	neg	4133.184907	4423.787556	3428.046865	4038.140965	4117.31855	3920.927098	3650.450046	3966.31266	3764.430727

1.20_346.8782m/z	346.8781923	1.202333333	neg	3567.899543	3611.909532	3434.88061	2688.594219	3246.019443	2901.519777	3013.692376	3433.050883	3504.130994
1.20_356.0604m/z	356.0603504	1.202333333	neg	1391.213063	1605.626449	1331.864857	1503.887375	1200.208997	1390.488326	1106.048203	1568.990603	1186.276882
1.20_370.9586m/z	370.9586138	1.202333333	neg	8057.346625	7985.528404	6735.580851	8230.44706	8922.102906	8281.284567	8668.083072	7475.268566	8328.216413
1.20_384.0293m/z	384.0292803	1.202333333	neg	2301.362902	2317.255329	1789.92438	2139.293365	420.9965416	1573.010913	2598.943955	1299.494408	305.4662479
1.20_388.0256n	387.0186613	1.202333333	neg	8792.805929	9600.928981	11227.69528	9746.317704	7766.522178	8629.866833	8824.586869	10912.75358	10105.63837
1.20_389.9857m/z	389.9856739	1.202333333	neg	4145.192137	4303.097804	4587.316366	4315.169379	4062.95211	4469.160586	4684.136948	4233.78741	4319.021439
1.20_399.9411m/z	399.9410728	1.202333333	neg	1403.759608	1047.804376	1410.554349	1072.940712	1430.009298	1585.722357	1303.556354	1688.704783	1339.107003
1.20_404.9099m/z	404.9099403	1.202333333	neg	5542.36734	5502.928867	5146.856438	4779.230895	4417.304353	4438.148208	4734.548614	5080.43895	5253.908565
1.20_409.0006m/z	409.0006	1.202333333	neg	2404.352069	2344.212162	3207.943956	2535.773066	2114.838157	2513.990192	2518.024846	3038.999773	2504.855258
1.20_411.0608m/z	411.060786	1.202333333	neg	1582.408888	1380.850449	1868.278038	1678.199943	1643.064265	1295.762631	1540.282662	1657.253768	1729.898989
1.20_412.9078m/z	412.9077518	1.202333333	neg	4260.725606	4214.95176	5030.400309	4549.698552	4344.294787	4131.944962	4213.826306	4755.364256	5102.994025
1.20_417.0651n	398.0472112	1.202333333	neg	2610.018237	2388.507679	2784.317889	3241.462251	2645.764176	3078.935209	2376.367874	3168.447485	2756.990861
1.20_421.9234m/z	421.9233971	1.202333333	neg	562.6530608	531.0210837	268.1217439	383.786949	442.9622763	604.0048537	521.0598517	906.394825	729.0919355
1.20_422.9206m/z	422.9205834	1.202333333	neg	7155.615236	7313.014675	6570.338531	6917.892515	8048.399491	7987.182827	8475.095079	6746.262802	7595.605173
1.20_434.8898m/z	434.8897937	1.202333333	neg	5305.208582	5500.852538	4882.391765	4324.706804	4716.009618	4568.158304	5086.182284	5165.904624	5098.756374
1.20_441.9472m/z	441.9471884	1.202333333	neg	1341.233949	1321.479823	1346.023948	1780.144215	2306.118807	2051.862527	1608.684676	2073.217325	2205.57281
1.20_442.9232m/z	442.9232172	1.202333333	neg	1114.347458	1518.52399	1347.421703	1671.135188	1306.103405	1677.434404	1014.575499	1141.065685	1271.640179
1.20_444.8452m/z	444.8452114	1.202333333	neg	937.1054355	1219.545043	940.3507251	1056.62872	1482.062744	1214.836838	1094.95802	1308.042168	1482.429882
1.20_451.9027m/z	451.9027291	1.202333333	neg	579.1995204	341.9819276	425.6478873	689.3348572	831.7707676	793.9697852	763.1618906	764.714039	1094.541186
1.20_452.0169m/z	452.0169199	1.202333333	neg	1253.235213	1013.221736	1140.927551	1606.242445	1727.292153	1466.301082	1598.040874	1336.707696	1602.294703
1.20_457.9730m/z	457.9729774	1.202333333	neg	1087.514978	864.6853834	903.3672128	1143.211914	995.2852604	1335.751603	1674.343585	1284.868458	851.9065278
1.20_463.9293m/z	463.9293372	1.202333333	neg	1168.205459	1232.770646	1398.819773	1910.913529	1231.059563	2212.284293	1523.948366	1893.026192	900.3188318
1.20_473.8854m/z	473.8854207	1.202333333	neg	483.5400074	406.423397	501.4703068	561.7745729	886.3275847	553.1181022	584.7236825	796.5820465	642.3276785
1.20_483.9541m/z	483.9541419	1.202333333	neg	837.4802003	1137.396895	591.3975974	963.464182	466.0686304	729.5541061	1152.226942	861.922544	1253.350444
1.20_489.9112m/z	489.9111617	1.202333333	neg	1618.602835	1062.973183	1325.360871	1712.809339	1937.975176	1553.135383	1202.271492	1582.986755	1518.581563
1.22_185.0458m/z	185.0457618	1.224233333	neg	310.3288807	330.6320163	287.9258894	212.7083886	203.3386395	175.6882233	73.53229631	93.39873793	93.08169871
1.22_187.8889m/z	187.8888997	1.224233333	neg	25933.56495	25067.30824	24384.35911	29874.40379	30842.91078	30127.07984	31604.15334	27128.80633	27749.19268
1.22_189.4946m/z	189.4945617	1.224233333	neg	324.80423	301.3045791	295.4459121	357.9470602	232.7452195	247.2644648	209.715547	233.9111309	218.8029989
1.22_215.0289m/z	215.0289193	1.224233333	neg	380.6395965	478.5854068	508.3078405	452.3085107	517.8064081	543.3985477	376.428992	471.1982478	414.8399252
1.22_218.9947m/z	218.994651	1.224233333	neg	215.9284422	283.8378689	290.1275419	379.6623332	266.6448403	304.6626213	296.8952133	268.6655384	256.0960673
1.22_230.9679m/z	230.9678687	1.224233333	neg	866.0308847	752.2217661	821.9492971	654.7807845	307.5351026	579.9207187	667.6652318	555.0628042	287.0987818
1.22_239.0288m/z	239.0287788	1.224233333	neg	3085.181915	2916.006129	3129.539979	5647.454486	5724.410975	5884.309744	4180.541991	4422.957741	4222.951194
1.22_250.0005m/z	250.0005181	1.224233333	neg	738.1160974	1007.490435	861.1884887	845.0559925	834.0923341	850.4513941	922.5026589	866.5010376	853.0743748
1.22_252.9499m/z	252.9499216	1.224233333	neg	1043.931628	871.9092551	891.3575546	773.4261517	233.2204565	544.667264	885.5110476	785.5015755	467.6992318
1.22_269.0257m/z	269.0256685	1.224233333	neg	524.3690825	607.4387548	623.6160007	378.780691	4.433892432	291.9496188	273.7925994	248.2619827	0.001403199
1.22_270.9508m/z	270.9508257	1.224233333	neg	2359.702105	2470.871719	2483.762073	1831.651877	2266.119841	2037.260693	2440.11168	2542.214598	2153.902109
1.22_272.0891m/z	272.0891167	1.224233333	neg	498.3648031	597.72348	420.4634501	574.4826123	280.7758458	342.4842696	478.1132242	563.4812431	394.2072982
1.22_278.9649m/z	278.9649144	1.224233333	neg	217.0961891	298.7039126	184.6918874	111.4626119	307.0636046	168.8879638	199.1436372	325.4817785	214.1883392
1.22_279.9688m/z	279.9688003	1.224233333	neg	665.4112751	569.499472	536.3298437	298.9129782	425.7962601	437.9444774	617.4830114	395.350667	364.0299019
1.22_294.8525m/z	294.8524918	1.224233333	neg	1381.025885	1294.561625	1196.638545	1660.792225	1323.117617	1553.803607	1319.476775	1504.634583	1198.107059
1.22_294.9161m/z	294.916095	1.224233333	neg	3491.483856	3390.247254	2886.710293	1994.629133	2477.14421	2374.354082	2416.831954	2596.687357	2566.770355
1.22_308.0061m/z	308.0061207	1.224233333	neg	1713.477214	2279.320278	2221.92408	3275.944329	3171.346125	3259.902241	2612.043105	3274.85679	3183.291715
1.22_308.0871m/z	308.08711	1.224233333	neg	909.8464406	821.857081	1083.522861	812.8967523	788.3328612	683.0532697	699.58379	847.693077	770.978745
1.22_313.9764m/z	313.9764167	1.224233333	neg	62.67730283	146.8094245	61.74693095	133.0628932	210.121162	66.2762209	133.3525193	86.60693359	106.6876121
1.22_321.0698n	320.0626034	1.224233333	neg	31836.09899	38105.01003	39870.08112	47864.50869	45218.88671	46432.80128	23164.41512	26996.84453	27878.11312
1.22_323.0195m/z	323.0195	1.224233333	neg	641.6484782	924.2682063	656.1833856	1028.8636	924.1374935	932.3169833	751.2254002	807.7809342	499.5719458
1.22_328.9984m/z	328.9983726	1.224233333	neg	1065.688834	1076.769956	1093.111334	1970.789496	2056.0355	2339.434177	1789.743697	1491.674283	1378.015561
1.22_340.9555m/z	340.9554764	1.224233333	neg	1881.234572	1819.005354	1973.267078	2576.202169	2327.880446	2586.146681	2086.031426	2295.760225	2166.056913
1.22_352.9484m/z	352.9483594	1.224233333	neg	2740.590849	3142.351683	3050.177569	2098.672048	1905.688331	2686.87303	3091.981541	2702.199157	2194.491047
1.22_362.9037m/z	362.9037054	1.224233333	neg	2640.513225	2634.58152	2467.324977	1284.693424	1301.78233	1767.601967	2345.717546	1959.612144	1333.849659
1.22_365.0319n	410.0320966	1.224233333	neg	3037.555422	2614.886746	2691.452889	2791.947957	2917.161582	3379.045412	1607.509508	1789.003341	1725.83669
1.22_369.0419m/z	369.0418565	1.224233333	neg	188.7856197	183.2403112	282.1455609	148.0708096	292.3689347	214.5734345	282.1415788	323.7335234	262.112242
1.22_415.9667m/z	415.96666	1.224233333	neg	1181.754521	703.5499103	1091.052991	1257.267952	640.844634	1242.077113	1176.074794	858.2803749	867.5459721
1.22_420.9355m/z	420.9354749	1.224233333	neg	1729.317543	1708.958506	1350.100477	1099.848482	896.5665505	1325.276146	1588.268462	1529.014009	1257.827685
1.22_430.8915m/z	430.8915348	1.224233333	neg	1736.655453	1234.560646	1228.493475	881.4995363	493.2698923	602.1345818	973.4147097	733.2543564	487.4003897
1.22_456.8724m/z	456.8723773	1.224233333	neg	1407.529767	1752.417017	1211.700402	1412.376917	431.3418312	1191.454791	1076.587847	822.0208591	601.170581
1.22_461.9938m/z	461.9938205	1.224233333	neg	3192.485191	3686.891369	4018.2712	5593.818974	5013.315291	4737.220011	2059.787378	2658.227469	2652.497617
1.22_483.9755m/z	483.975457	1.224233333	neg	2146.716115	2213.347505	1852.619073	3328.770488	1699.193364	2394.465151	1175.557622	1476.090683	1012.440554
1.22_505.9568m/z	505.9567696	1.224233333	neg	1616.36789	1257.288732	1550.404559	1974.533613	892.3387357	1435.795865	965.003924	391.8922799	661.4787595
1.22_534.1581m/z	534.1580963	1.224233333	neg	1287.027009	1704.853931	1546.72212	1779.832643	1772.235709	1727.805992	1516.94885	1852.795132	1962.468188
1.25_153.0419m/z	153.0418738	1.246333333	neg	0.001403199	33.25454581	36.09428997	31.26586202	28.29181499	28.34016285	0.001403199	33.93315697	57.7884603
1.25_174.0853m/z	174.0853317	1.246333333	neg	248.003479	260.9149846	273.9982961	287.0772002	328.8185122	366.6335003	286.3821108	373.9068356	369.9903151
1.25_228.0315m/z	228.0315072	1.246333333	neg	5210.59591	5855.391333	5561.181448	3105.672872	3470.519774	3194.442519	3599.18134	3801.554394	4038.505961
1.25_241.0697m/z	241.0696642	1.246333333	neg	780.4143121	952.4025144	813.6634223	1128.419401	1296.298829	1262.348405	1136.097986	1289.521649	1220.442107
1.25_261.0109m/z	261.0109435	1.246333333	neg	1921.23816	2281.080676	2359.562914	3859.290654	3088.853988	3827.821063	2925.384966	2824.195208	2198.493243
1.25_261.0308n	260.0235298	1.246333333	neg	131326.5553	150738.641	150791.6102	212290.7553	213017.4872	205569.0947	148588.5207	166223.5421	161891.5854
1.25_262.0280m/z	262.0280298	1.246333333	neg	728.4058263	1029.111709	909.5274866	1413.54247	1377.291832	1446.457755	832.8395974	959.5556102	1108.471832
1.25_263.0095m/z	263.0094804	1.246333333	neg	712.8299908	1079.27197	1050.506412	1516.938634	1337.740864	1354.254151	1326.111576	1363.487083	1415.515952
1.25_274.8683m/z	274.8683129	1.246333333	neg	1478.114654	1525.375477	1635.198327	1838.822804	1643.404677	1699.590179	1825.415892	1919.381643	1480.302471
1.25_282.0055m/z	282.0055407	1.246333333	neg	27183.88476	28958.39312	28263.79194	39905.10171	41511.14533	39954.02778	29276.8215	30093.82492	30562.04708
1.25_286.8550m/z	286.8550065	1.246333333	neg	405.7911512	525.0955917	378.4743854	480.8344818	601.971176	590.6989238	699.019356	651.3141841	479.0209336
1.25_294.0037m/z	294.0036967	1.246333333	neg	371.4135286	193.0916787	160.9109985	284.7953164	0.001403199	183.476712	295.3445485	111.8251862	51.5319738
1.25_323.8693m/z	323.8692793	1.246333333	neg	1680.157616	1678.494224	1395.680961	2102.699627	1974.765588	1888.338597	2251.858763	1859.325294	1642.080108
1.25_325.9981m/z	325.9981257	1.246333333	neg	5534.416135	5873.501465	5599.460277	4971.137624	5173.609203	4792.833107	4738.999022	5474.241708	5236.512805
1.25_344.9097m/z	344.9097091	1.246333333	neg	518.0613705	396.7750226	420.1942892	819.3828011	706.900013	829.0731566	524.5279765	516.4463348	436.1282792
1.25_347.9801m/z	347.9801377	1.246333333	neg	3474.054315	3714.462767	3512.649791	2748.839671	2725.9995	2766.459321	3160.607926	3112.150085	3240.986772
1.25_349.9931m/z	349.9931076	1.246333333	neg	8185.573146	7649.040663	7820.73074	10562.39432	10684.09464	10982.63886	8861.1215	8632.834445	8254.621614
1.25_357.9913m/z	357.9913071	1.246333333	neg	3849.088323	4049.97087	3676.026567	7475.500566	6545.264708	6545.081228	4335.184925	4291.474758	4439.98713
1.25_359.9487m/z	359.948741	1.246333333	neg	2374.208414	2602.186355	2376.113382	3476.599871	3386.070688	3043.477896	2563.267101	2598.882243	2083.066923
1.25_379.9731m/z	379.9730654	1.246333333	neg	14638.88617	16641.13086	16657.2293	28861.54108	26014.49928	25503.76645	17291.62207	19564.95098	18850.10332
1.25_381.0503m/z	381.0502635	1.246333333	neg	12117.92736	13512.25631	12678.564	10226.36635	10076.63875	9702.390549	9502.514973	11017.39037	11131.88491
1.25_401.9548m/z	401.9548436	1.246333333	neg	7268.575861	8764.58521	8480.877527	13305.90447	12196.14673	13641.27676	8644.353362	9940.352145	9552.761172
1.25_411.9104m/z	411.9104096	1.246333333	neg	567.2810862	698.5239981	693.2473787	906.1432466	834.8536888	921.7845815	493.0840241	578.7042465	843.8946308
1.25_417.9807m/z	417.980677	1.246333333	neg	3367.409077	4236.646793	3727.429479	4316.873076	4669.796764	4296.615302	3436.837443	3182.5205	3591.074392
1.25_423.9372m/z	423.9371622	1.246333333	neg	4122.338677	5187.164847	5313.415218	7129.16405	7510.425479	7430.173513	5225.659908	4950.588473	5191.575943
1.25_427.9359m/z	427.9359197	1.246333333	neg	3410.156854	3155.660065	3041.47347	3502.718428	4209.863148	3110.022788	2899.458459	2891.030514	2737.704904
1.25_433.8920m/z	433.8920267	1.246333333	neg	1253.692251	1622.758817	1093.578799	2409.320207	1838.608701	2188.55647	1652.655729	1737.556816	1469.802251
1.25_449.0376m/z	449.037648	1.246333333	neg	1240.270563	1351.728712	1198.285913	944.0890946	851.8507336	516.0477232	990.6788208	921.7374315	1057.622623
1.25_454.9043m/z	454.9043178	1.246333333	neg	376.2355389	279.3121392	328.7081829	613.9420635	528.4123496	653.993138	448.2246864	500.7388458	442.9220789
1.25_485.9682m/z	485.9682335	1.246333333	neg	1800.024295	1964.830865	1743.096371	2422.400222	2426.937771	3070.98208	1995.238669	1706.624201	1721.556092
1.25_491.9245m/z	491.9245236	1.246333333	neg	1635.458216	2403.964493	2097.116784	3608.77901	3858.895409	3948.693063	2414.207653	2635.705032	2432.341619
1.25_495.9238m/z	495.9238328	1.246333333	neg	1310.888814	1632.97895	1437.905631	1551.134275	1938.390702	1850.714894	1226.228533	1199.744037	1762.794917
1.25_499.9224m/z	499.9223587	1.246333333	neg	1307.164398	1807.579349	1829.139187	3538.01354	3977.589608	3445.528541	2190.39179	2343.090716	2179.477604
1.25_501.8795m/z	501.8795111	1.246333333	neg	937.7014217	988.6058812	1200.395369	2117.167403	729.9379342	1855.374333	1444.893004	1187.244292	396.9171207
1.25_521.9043m/z	521.9043282	1.246333333	neg	933.4629277	1784.759107	1629.633423	2867.432384	3102.365721	3710.545489	2104.820676	2734.632556	2524.924905
1.25_543.0367m/z	543.0367075	1.246333333	neg	509.9909408	435.136454	774.5934291	1551.153464	1487.515546	1621.20636	705.5774881	602.3421918	694.8566892
1.25_559.9117m/z	559.9117146	1.246333333	neg	635.9888169	1053.212582	783.6422246	1107.296806	1435.604565	1476.239047	956.0205006	893.3044205	1382.538368
1.25_565.8684m/z	565.8684277	1.246333333	neg	1009.139927	1301.771986	1111.149839	1120.40281	1387.290216	1505.914185	1408.196646	1087.128398	1226.666572
1.25_575.8240m/z	575.8239523	1.246333333	neg	1153.301738	901.6841355	1100.491001	1736.397804	1600.469122	1033.560898	967.8454391	1114.994008	781.00257
1.27_129.0188n	257.0281781	1.26875	neg	1786.036747	2227.256869	2288.916292	3020.244563	3166.567929	3181.325565	2009.253529	2240.829755	2199.699982
1.27_153.8955m/z	153.8954936	1.26875	neg	136.3993658	97.94066253	64.81307313	118.5504244	157.7441472	79.41680159	128.8692547	212.9497849	117.034438
1.27_179.9897n	358.9671533	1.26875	neg	9133.635156	9692.380739	9419.582027	2644.070359	2556.803633	2497.673097	3176.897844	3131.004051	3199.935393
1.27_222.9920m/z	222.9920189	1.26875	neg	93742.25098	102120.6826	103018.2861	34604.27223	34533.6326	33118.34683	36638.18881	41207.36693	40432.75836
1.27_274.0183m/z	274.0183311	1.26875	neg	366.2739646	612.7913503	720.7006829	990.2330447	912.1427657	949.0808927	573.1721162	587.7548398	490.634987
1.27_280.0675n	325.0656773	1.26875	neg	4175.53386	3908.551238	4062.722727	3765.918213	3815.605461	3995.397951	3743.635338	3612.915094	3871.283947
1.27_296.0188m/z	296.0187848	1.26875	neg	2272.758119	2486.967688	2153.691369	1094.22012	1088.803071	1108.302899	1182.513987	1458.966891	1378.613571
1.27_307.9694m/z	307.9694054	1.26875	neg	838.6189465	924.9110698	647.2522832	160.0342049	164.6285184	64.41313557	217.1210837	143.0314789	162.3858882
1.27_309.9974n	290.979588	1.26875	neg	14554.35484	15566.78072	16111.11996	8973.900369	9298.558293	8426.930763	7910.667596	8337.862111	8251.33916
1.27_314.9829m/z	314.9828953	1.26875	neg	1873.414061	1906.687997	1935.119348	645.2478108	743.3409823	500.6829571	1722.835198	2035.827662	1439.45158
1.27_315.8265m/z	315.8265308	1.26875	neg	1770.268647	1597.489546	1321.934337	1904.391033	1456.77496	1574.575246	1742.55689	1744.321894	1268.913907

1.27_322.9148m/z	322.9147944	1.26875	neg	1672.095075	945.2747951	1141.691175	0.001403199	1091.462401	561.0606153	285.5867129	0.001403199	181.5202295
1.27_325.0154m/z	325.0153689	1.26875	neg	769.4645853	640.4205712	502.3875035	1003.638844	735.5087111	554.2611425	506.5263442	401.2944775	551.2615676
1.27_342.9416m/z	342.9415611	1.26875	neg	6669.859265	8397.346031	7703.000623	2493.699735	2354.949574	2171.953432	2556.59714	2888.235127	2786.956022
1.27_369.9625m/z	369.962474	1.26875	neg	943.9509682	882.4025143	979.4096421	658.7870588	493.9661004	617.8743403	577.1437721	576.7531471	655.6240354
1.27_393.0032m/z	393.0032165	1.26875	neg	746.9454137	890.3751977	744.8150553	1209.169804	1188.565596	1391.709729	844.9687394	854.2504864	860.0263159
1.27_398.9597m/z	398.9596752	1.26875	neg	1012.513988	1093.575351	831.6054862	1560.423127	1154.469535	1175.314977	937.4395217	973.5062662	1076.908729
1.27_437.9498m/z	437.9498181	1.26875	neg	1035.708037	1035.459789	979.5273623	708.2027604	926.549142	888.5726583	1024.71559	1075.423266	1128.441032
1.27_445.0146m/z	445.0146155	1.26875	neg	1458.278982	1127.512009	1105.428302	966.8700033	769.2945049	832.8549201	988.0149658	868.6699142	858.563073
1.27_448.9012m/z	448.9011595	1.26875	neg	1488.647487	1513.731738	1215.263285	1041.900699	974.2884267	837.5204546	1130.330247	983.0857924	874.503907
1.27_449.7863m/z	449.7863386	1.26875	neg	4515.497693	4167.675258	3758.064547	4540.636897	4734.776673	3961.924707	5032.750331	4218.97377	4116.385128
1.27_506.0045m/z	506.0044609	1.26875	neg	1098.626915	1024.553261	1089.951007	283.9894786	217.9466284	233.9035531	130.9425384	113.0801277	281.311176
1.29_178.8979m/z	178.8979322	1.290916667	neg	2965.317117	2676.693118	2685.328417	2967.185093	3129.801953	3105.104288	3559.150185	2956.262296	3189.199383
1.29_203.9018n	406.8035324	1.290916667	neg	19566.26282	18097.27507	15657.36455	22045.92596	21212.04274	20813.85634	21869.66696	18335.80735	18817.15967
1.29_234.9309m/z	234.9308943	1.290916667	neg	517.741686	421.5472951	435.0738812	862.0493618	783.3661122	696.3690463	639.3715662	536.9764881	796.6120568
1.29_260.8829m/z	260.8828691	1.290916667	neg	72.23014919	18.56996327	16.88192832	222.8791318	398.1837509	197.4888714	58.93945606	48.32016563	109.695683
1.29_262.8575m/z	262.8575364	1.290916667	neg	2587.510014	1947.111734	1513.789766	2017.091363	1949.043642	1951.815653	2096.848883	1680.697876	1846.474776
1.29_262.8770m/z	262.8769788	1.290916667	neg	11084.81821	9051.322893	8339.807333	11235.92785	11951.03404	11059.51454	12147.32422	9612.351652	9883.316098
1.29_280.9506m/z	280.9506391	1.290916667	neg	1063.702411	1235.169625	1290.199822	237.5337809	360.558369	199.8983701	443.4467772	465.5323898	402.0262552
1.29_320.8287m/z	320.8287271	1.290916667	neg	1352.719303	1296.06362	1284.382512	1248.282569	1616.894888	1471.976348	1815.032939	1815.034432	1375.178299
1.29_322.8314m/z	322.8313863	1.290916667	neg	6231.481922	5595.738462	4717.06927	4534.407788	5586.441964	4881.170238	6944.437394	6209.612276	5702.584974
1.29_343.0582m/z	343.0582068	1.290916667	neg	2793.173647	3332.452679	2843.191793	436.5380604	472.5966028	500.2314179	1303.234035	1433.237694	1519.900035
1.29_364.9234m/z	364.9233972	1.290916667	neg	2680.029019	3068.429919	3005.624988	851.8044845	1009.011331	752.7086709	963.0574984	1363.959101	1307.043299
1.29_373.0383m/z	373.0383031	1.290916667	neg	851.8012163	998.0712248	1181.917692	159.4204047	180.1168191	118.0052774	605.3312309	728.0040703	664.0534776
1.29_390.8190m/z	390.8189608	1.290916667	neg	4350.786212	4193.732136	3658.681727	3558.440343	3698.09907	4078.011761	4797.062233	3973.028037	3754.857612
1.29_395.0200m/z	395.0199835	1.290916667	neg	1334.997396	1516.086712	1487.740276	246.8891204	283.2465712	212.579197	798.5897367	745.6423264	706.9215614
1.29_396.7751m/z	396.7751076	1.290916667	neg	1492.076594	921.0361875	1098.932568	1487.246484	1382.564915	1519.019215	1834.960537	1775.974393	1329.968648
1.29_396.8482m/z	396.8481744	1.290916667	neg	36655.93569	35094.18437	31567.15916	44015.07513	44927.54141	43964.91624	45448.13717	37004.60316	41378.86187
1.29_400.8470m/z	400.8469741	1.290916667	neg	262151.013	226600.0146	206036.8321	240762.9964	249066.4668	241325.7773	273265.2058	217112.8074	232194.5637
1.29_426.9544m/z	426.9544491	1.290916667	neg	2314.237732	2242.170321	1765.075118	483.3354953	331.9143604	330.3376643	516.8646193	349.5287857	382.5215447
1.29_440.0931m/z	440.0930908	1.290916667	neg	717.9146036	1250.091777	1170.390246	1108.091063	1084.295692	1377.110064	516.236723	564.7087102	704.3864268
1.29_445.8454m/z	445.8454182	1.290916667	neg	3957.216874	3119.272215	2156.002883	2688.479189	2914.161145	2750.869051	3553.969166	3068.429759	2324.265366
1.29_474.7910m/z	474.790953	1.290916667	neg	13863.8433	13034.60888	10746.04658	14947.02811	14449.42411	13270.82988	15528.25853	11706.10627	12537.06574
1.29_492.1479m/z	492.1479414	1.290916667	neg	495.454288	531.1354923	543.2792349	633.0423162	802.3264847	465.4303129	602.74371	454.6547886	568.8058559
1.29_542.7788m/z	542.7787634	1.290916667	neg	9896.03258	8471.875382	7371.277314	9259.750339	10293.23089	9290.646515	11410.22371	8706.628373	9373.935728
1.31_128.8781m/z	128.8781494	1.3134	neg	22705.87052	20292.15903	18985.26542	19231.5226	19662.02287	18661.06475	22691.93469	19703.01856	20290.02855
1.31_146.9467m/z	146.9467476	1.3134	neg	47.81999221	88.90275732	88.03880367	101.4377873	59.17967811	71.54969263	116.0719423	124.8285335	87.13334771
1.31_153.9001m/z	153.9000611	1.3134	neg	2587.631472	1738.658821	1429.143461	1468.685097	1620.792193	1572.438005	1979.994456	1557.785356	1580.616419
1.31_160.0697m/z	160.0697267	1.3134	neg	110.6655706	144.7405572	123.7827912	174.6410577	171.8652752	211.0793295	65.84726806	92.33701706	95.9210774
1.31_170.8710m/z	170.870985	1.3134	neg	4358.290898	3873.344706	3761.653088	2811.021267	3337.507883	2639.956235	3982.711328	3883.871586	3085.138526
1.31_174.8649m/z	174.8648872	1.3134	neg	556.3857135	607.7445725	498.116419	422.5105986	304.4843256	416.8326535	573.0538737	563.652811	395.8777518
1.31_178.9044m/z	178.9043882	1.3134	neg	498.9233236	464.3919184	318.7449303	373.3556774	427.2089263	406.0706345	507.6558394	480.4396739	382.9670681
1.31_179.0021m/z	179.0021487	1.3134	neg	1042.880298	1048.344063	1126.114675	1489.808038	1394.38909	1378.919301	1350.483963	1302.054532	1423.70304
1.31_194.9582m/z	194.9581502	1.3134	neg	503.9649628	617.5146542	628.6416003	590.2368165	526.3275202	557.6341291	538.9610702	546.4240882	420.5925516
1.31_197.8850m/z	197.8850012	1.3134	neg	77.09575166	128.5234923	171.6396303	277.4733624	107.8136793	282.3011266	107.9937245	149.9481334	173.7204044
1.31_200.9840m/z	200.9840284	1.3134	neg	528.0993545	564.7682466	602.4848192	698.6617992	625.4156706	658.935478	627.1170367	656.3261566	684.0980898
1.31_213.8753m/z	213.8752794	1.3134	neg	263.7781997	292.6753477	441.5850728	397.8706232	318.9308558	439.1246911	456.6949837	498.8248333	413.7393647
1.31_242.0649m/z	242.0648971	1.3134	neg	1130.618011	1092.544451	1115.286805	949.6244359	802.2865537	846.3450943	1080.973795	1071.398412	1044.06631
1.31_263.9061n	244.8882322	1.3134	neg	39118.74079	35723.96564	31807.33233	38179.0656	38128.62596	36541.94794	45185.01273	36410.54126	36918.89135
1.31_270.8910m/z	270.8910253	1.3134	neg	4386.165103	4075.161535	3947.076536	4634.665653	4876.663394	4576.635854	4835.919675	4038.894985	4340.309932
1.31_287.0410m/z	287.0409704	1.3134	neg	632.6257621	757.7326523	625.1795037	376.9107504	333.731834	451.2929555	540.1755157	562.4102898	519.9316363
1.31_296.8503m/z	296.8502729	1.3134	neg	2383.16992	2485.210343	2307.391878	3021.579536	3084.792911	3101.832786	3385.040858	2822.135447	2344.179403
1.31_298.9513m/z	298.9512775	1.3134	neg	468.4969063	744.9667073	529.3775455	745.8888684	582.1647694	488.6852502	441.6209597	606.4826634	561.8776754
1.31_303.0296m/z	303.0296011	1.3134	neg	528.2914821	632.0790557	576.2267011	956.9331556	913.723463	990.9260347	689.4602815	602.810463	646.3966214
1.31_306.8880m/z	306.8879657	1.3134	neg	2470.413462	2396.94748	2204.359676	4250.849781	4023.868131	3989.920531	3243.89389	2830.280644	2316.847142
1.31_312.8760m/z	312.8760109	1.3134	neg	93618.17141	90745.28539	87089.53552	110825.7202	114845.8161	112565.4772	116089.7401	97952.61034	106241.7855
1.31_332.8602m/z	332.8601634	1.3134	neg	237635.1558	210806.0393	190678.5583	225750.7687	233643.0763	224811.6647	248172.4052	204245.0491	217330.539
1.31_339.8948m/z	339.8947897	1.3134	neg	5136.562764	5055.843155	4518.23161	5578.825118	6424.730421	6325.493103	6734.366331	5668.851553	5101.726141
1.31_342.0565n	323.0386739	1.3134	neg	3765.846168	4234.751295	3558.628433	1495.446749	1507.476565	1403.921214	1461.999237	1479.916404	1281.995101
1.31_342.8153m/z	342.8153454	1.3134	neg	6638.560667	5853.013376	4993.272285	5832.177174	5464.113547	4899.16139	6391.651494	4885.832156	5032.671014
1.31_345.9567m/z	345.9566901	1.3134	neg	753.4559072	687.0202945	901.7311886	603.2527027	724.3834325	519.6908648	620.0916624	923.3138603	421.6795556
1.31_387.0551m/z	387.055053	1.3134	neg	5776.232266	6229.578623	6070.323918	2762.08416	2642.411355	2542.469398	2323.599342	2667.661918	2380.862174
1.31_390.8919m/z	390.8919311	1.3134	neg	297144.1458	286194.332	268171.3702	340659.5292	364992.1864	358368.424	360227.9746	298365.0617	326268.7403
1.31_405.0112m/z	405.0111646	1.3134	neg	1057.930693	1085.344344	1239.583446	621.5333381	451.9464073	451.5443285	719.6547528	1108.576874	1025.158261
1.31_410.0440n	455.0423992	1.3134	neg	2670.025734	3030.977987	2029.922258	1014.437821	709.8848406	708.4998743	910.9913919	874.4794326	670.6567957
1.31_410.8024m/z	410.8024375	1.3134	neg	6327.698681	5555.03602	4341.337207	5117.711733	4405.14148	4555.903418	5555.398967	4070.63276	4259.934204
1.31_417.0348m/z	417.0348421	1.3134	neg	3273.778117	3860.136339	3376.593405	2012.516398	994.3305794	1405.339373	1375.451703	1210.391552	849.4101927
1.31_421.0368m/z	421.0367711	1.3134	neg	788.0636055	843.4515069	698.6853672	318.6909006	344.8310642	357.7508853	503.9952982	632.2813531	615.1172007
1.31_439.0168m/z	439.0168029	1.3134	neg	5039.97196	5551.401195	4886.330432	2703.006317	1513.504133	2275.130708	2170.781593	2035.718923	1486.104188
1.31_464.8353m/z	464.835291	1.3134	neg	17383.46913	16986.88352	15345.26754	23589.64061	22542.7643	22373.85044	25710.8788	19088.8886	21566.2894
1.31_468.8343m/z	468.8343188	1.3134	neg	247319.0839	215462.577	189226.0221	221852.0143	230600.2364	222789.0866	255364.8291	201015.8688	216040.8221
1.31_470.7921m/z	470.7920533	1.3134	neg	1319.548641	1644.337377	1514.834095	2087.765019	2078.334191	1929.590467	1725.149464	1807.911219	1769.909463
1.31_479.0334m/z	479.0334457	1.3134	neg	318.8444112	571.7949816	454.4928094	0.001403199	0.001403199	75.88475016	297.4491851	0.001403199	243.3806852
1.31_523.0297m/z	523.0297127	1.3134	neg	1685.228703	1996.099451	1500.393027	306.0144517	390.9256559	532.8272432	547.2535563	257.6571236	423.6553276
1.31_538.7797m/z	538.779659	1.3134	neg	824.7213697	951.1593867	707.8370104	1080.450914	901.2549955	1242.904332	984.6510826	857.6446162	656.0543941
1.31_546.7782m/z	546.7781986	1.3134	neg	4854.061921	3754.048168	2446.96907	2922.671486	3581.470903	2905.663178	4621.092675	3931.517624	3128.842852
1.31_610.7665m/z	610.766548	1.3134	neg	3864.831697	3249.432701	2615.81177	3697.641588	4313.761303	3381.050617	4754.713488	3792.648758	2976.00857
1.31_668.7988m/z	668.7987922	1.3134	neg	1006.070643	1640.273796	1089.429136	1959.708417	1711.835773	1475.64278	1669.998656	1481.336248	1726.706764
1.34_122.8942m/z	122.8941985	1.335933333	neg	303.6809965	263.1237202	237.898873	246.9095282	207.4194858	267.41556	275.5469344	290.2467897	214.8062675
1.34_183.0049m/z	183.0048772	1.335933333	neg	422.5782597	449.8484628	377.2955293	329.9446698	300.6109103	271.7272745	455.1349925	385.6901339	378.9377621
1.34_199.0225m/z	199.0225229	1.335933333	neg	898.6227836	930.6613695	805.5401234	809.5290686	603.2691007	578.5635287	695.1359541	637.5363379	572.7007222
1.34_204.9885m/z	204.9885079	1.335933333	neg	180.9432023	214.7033023	221.9798571	104.1687596	141.0727718	111.8701646	149.0409796	185.9589882	128.8812773
1.34_225.8854m/z	225.8853982	1.335933333	neg	1298.101134	1121.505078	1170.649584	1329.583039	1302.791171	1268.36344	1411.319689	1155.384092	1033.877711
1.34_232.0077m/z	232.0077337	1.335933333	neg	205.9126324	287.376183	309.2564154	213.5043536	188.1313337	174.2812531	223.9557813	172.048172	192.9845517
1.34_236.9995m/z	236.9994716	1.335933333	neg	3336.783579	3253.443025	3434.074087	2824.546131	3056.487699	3005.425352	2599.192747	2935.425687	2599.959434
1.34_245.9469m/z	245.9468696	1.335933333	neg	550.3610027	686.8801598	507.4658499	663.0322352	644.0129854	502.6150175	450.8265386	438.2012777	417.4892333
1.34_246.9549m/z	246.9548536	1.335933333	neg	543.7738155	545.8172975	573.2253322	329.9128099	331.26499	264.6655122	405.5252776	311.2460479	290.4183462
1.34_247.0397m/z	247.0396908	1.335933333	neg	1148.705595	1473.787011	1331.037296	1312.961765	1218.824296	1251.050812	1246.908775	1560.768375	1385.459139
1.34_247.9600m/z	247.9599899	1.335933333	neg	224.7623254	288.6085675	214.3623465	355.4504058	305.8711994	250.1661183	157.5310398	281.3456799	242.5368727
1.34_266.9795m/z	266.9794502	1.335933333	neg	3039.546243	3272.588326	3768.579621	3275.0905	3172.92945	2949.414659	2361.187166	2972.49239	2803.766651
1.34_271.9073m/z	271.9073093	1.335933333	neg	44012.24113	41595.19084	40502.47066	53482.28528	55901.44533	56707.67154	53175.25037	46226.40531	48668.18287
1.34_304.9871m/z	304.9870921	1.335933333	neg	2421.758414	2085.345906	2105.994145	1633.799712	1895.861119	1708.603344	1681.045453	1730.77258	1331.217226
1.34_310.9434m/z	310.9434077	1.335933333	neg	1104.992735	1071.879524	1313.353488	1169.722797	1073.123039	1036.319142	1022.834435	1149.543058	1174.616028
1.34_345.8512m/z	345.8511736	1.335933333	neg	1485.12738	1364.246621	1227.605272	1747.783899	1845.845762	1615.563054	1654.862011	1540.056187	1426.348681
1.34_372.9742m/z	372.97424	1.335933333	neg	1095.881732	861.4153535	771.9388255	840.2839676	552.8127118	346.5154413	618.7395213	341.6207314	678.5377345
1.34_458.8058m/z	458.8057623	1.335933333	neg	22928.53577	20523.71552	18033.31557	19264.96073	20095.08197	20235.72015	24936.73676	18556.43843	21259.5738
1.34_532.8231m/z	532.8230876	1.335933333	neg	11183.06169	10294.29107	9431.152907	14849.04968	15500.57738	14561.50043	16326.44444	11805.81465	13705.63514
1.36_185.0069m/z	185.0068808	1.358266667	neg	458.074841	570.3199593	605.8431992	654.1341571	720.2432935	1162.405226	522.0763619	558.6738224	620.2797055
1.36_206.9156m/z	206.9156219	1.358266667	neg	106.2087483	100.6858967	98.7381821	68.96807257	80.73246153	127.3503779	30.6732202	88.96886308	0.001403199
1.36_206.9889m/z	206.9889128	1.358266667	neg	1295.058698	1337.564834	1401.876301	1507.935082	1802.414576	2888.998036	1269.353577	1288.404182	1493.478349
1.36_234.8594m/z	234.8593885	1.358266667	neg	1491.520871	1300.29789	1200.675097	1351.591397	1336.188309	1326.108464	1662.04516	1386.01483	1345.18357
1.36_258.9506m/z	258.9505677	1.358266667	neg	568.167582	777.2733217	793.5206589	898.6165302	1082.198066	2000.387417	789.5126107	867.2798992	881.1500877
1.36_265.0191m/z	265.0190592	1.358266667	neg	1106.539506	1136.173256	1046.201169	1357.153808	974.4166313	949.1816377	974.4428364	1116.219685	1162.560006
1.36_270.9766m/z	270.9765565	1.358266667	neg	439.3964036	595.4121747	592.1131728	487.3115293	365.4509365	393.9614317	545.1728302	594.8354038	553.7686762
1.36_274.9764m/z	274.9764254	1.358266667	neg	1021.310696	1175.324216	1208.069731	1277.667779	1195.110016	2321.201374	1028.115833	1119.621591	1207.098066
1.36_280.9327m/z	280.9327342	1.358266667	neg	670.683774	814.6223565	686.1763396	915.6072617	949.1650419	1598.834943	689.6151561	690.5976105	766.9142974
1.36_329.8660m/z	329.8659863	1.358266667	neg	14917.65155	14098.15901	12690.56415	16824.92275	17386.04668	15104.17018	18300.48099	15085.54632	15728.54677
1.36_331.0109m/z	331.010887	1.358266667	neg	4224.743172	3976.764168	4143.100116	3987.604525	4295.827382	4242.638723	3943.600001	3987.293648	3744.257682
1.36_335.0614m/z	335.061409	1.358266667	neg	964.3648956	1023.188767	1072.383626	938.2201032	1161.598318	981.7095005	891.007906	857.9421257	811.9790437
1.36_342.9637m/z	342.9637298	1.358266667	neg	565.898332	880.8578935	654.3745319	636.772835	834.7895936	1499.002457	594.3502689	679.383873	632.4178007
1.36_345.1783m/z	345.178277	1.358266667	neg	521.556437	480.6145277	562.7414652	895.7027776	822.91727	795.1994019	718.9147939	867.2973518	838.061625

1.36_398.9983m/z	398.9983096	1.358266667	neg	1254.898471	1223.601582	1088.903408	1118.499027	1182.665161	890.3353245	1032.686473	1267.333124	1145.286357
1.36_447.9947m/z	447.9946552	1.358266667	neg	520.3980748	588.1418785	826.1595898	574.9003955	716.0656957	682.7479062	600.2109645	972.1305555	911.3493917
1.36_464.0205m/z	464.0205041	1.358266667	neg	1193.151722	1141.630267	1124.116395	1076.53739	1004.813516	1182.557513	1164.534809	1089.717308	1231.7235
1.38_151.0013m/z	151.0012733	1.380283333	neg	519.2371175	295.6612418	423.7386337	406.0263207	294.2645921	323.6594428	431.8624657	336.4547383	489.4479981
1.38_154.0623m/z	154.0622981	1.380283333	neg	232.9571231	229.978492	263.6462265	288.1287401	352.3802818	347.2680174	389.802501	391.0724721	353.8521843
1.38_194.9911m/z	194.9911474	1.380283333	neg	3589.764138	3141.567922	3085.576815	2790.943374	2763.985628	2668.089307	3010.83532	3078.525545	2783.646478
1.38_198.0718m/z	198.0717631	1.380283333	neg	2340.239243	2172.94732	2208.829739	3665.617386	3171.166568	3127.564524	2811.605789	2754.629531	2801.480173
1.38_226.0663m/z	226.0663339	1.380283333	neg	20628.44003	16917.35544	18188.32526	30679.01949	29440.46084	26406.40639	27578.20873	27441.07635	25465.58768
1.38_232.0286m/z	232.0286136	1.380283333	neg	1789.504728	1888.125712	2008.953468	1561.971068	1461.560226	1445.456621	1574.6617	1739.128645	1619.119452
1.38_235.0725m/z	235.0724965	1.380283333	neg	672.0454362	645.2938856	473.4028613	449.7917285	551.415267	464.8934376	791.6130764	487.7470556	613.1354004
1.38_236.9688m/z	236.9688341	1.380283333	neg	1121.630792	1444.293635	1270.616964	1792.940018	1881.790814	3178.545113	1307.392269	1412.335239	1523.210719
1.38_252.0185m/z	252.0185311	1.380283333	neg	49.2169365	33.72963079	121.2650563	0.001403199	50.48383714	55.83864761	0.001403199	53.05636316	78.72698687
1.38_252.9498m/z	252.9497681	1.380283333	neg	1145.636222	573.2393096	801.3825285	621.1106439	517.8054059	357.2512624	612.4716599	659.3026018	887.5205765
1.38_262.9787m/z	262.9787135	1.380283333	neg	11092.69518	9287.066612	8600.919196	7701.572283	7254.562583	6657.138841	9123.378265	8613.828879	8525.910387
1.38_272.9342m/z	272.9341737	1.380283333	neg	2170.334719	1842.721938	1662.249034	1301.518624	1315.535818	1126.633006	1530.886299	1658.938375	1620.983505
1.38_293.9623m/z	293.9622686	1.380283333	neg	185.3529261	191.7523905	149.6445755	228.1347972	118.2499509	111.2903842	139.1543619	267.2500206	197.4583287
1.38_308.0366m/z	308.0366168	1.380283333	neg	878.9617861	769.2410451	902.4731141	587.0649126	610.7320391	538.0269937	830.5825292	759.12635	887.2171936
1.38_310.0409m/z	310.0409102	1.380283333	neg	3136.742502	2854.486753	2604.007786	4480.315925	4312.695063	4052.42856	4516.018761	4184.316758	3903.886042
1.38_315.0328m/z	315.0328256	1.380283333	neg	220.0802873	301.1623212	384.5318484	225.3226043	422.9652677	329.5814165	594.8197395	493.8722645	553.998352
1.38_319.0025m/z	319.0025182	1.380283333	neg	485.5006091	335.9523436	583.8623681	605.1983156	711.3999798	567.2127292	576.4682862	437.7814079	504.2408994
1.38_322.9138m/z	322.9137878	1.380283333	neg	206.917582	263.4482784	161.9473895	388.0555832	203.7612239	0.001403199	0.001403199	0.001403199	142.1788651
1.38_324.9878m/z	324.9878202	1.380283333	neg	3945.414825	2454.549349	2710.259227	4699.536997	4168.372175	3635.07126	4734.921067	3987.80725	3883.820021
1.38_330.9664m/z	330.9663534	1.380283333	neg	5761.253452	4554.816855	4570.081016	3970.189376	3721.056079	3220.435166	4665.272253	4602.15163	4346.320686
1.38_345.0126m/z	345.0126109	1.380283333	neg	7072.04928	5719.854868	5968.447315	11140.28372	10211.45619	9487.330879	11982.14471	10423.57254	9844.776248
1.38_361.0381m/z	361.0381133	1.380283333	neg	5585.93402	4657.451088	4833.254104	7741.939315	7461.393921	6825.552725	8288.127503	7353.448136	7361.458888
1.38_362.9038m/z	362.9037795	1.380283333	neg	2289.725905	1965.973859	1868.077685	1245.490679	1420.470999	1378.309536	1757.972184	1624.497314	1557.00523
1.38_371.9627m/z	371.9626695	1.380283333	neg	63.19718791	158.7055086	200.1643603	56.3835804	79.2227175	0.001403199	102.5390097	72.79444122	214.4891636
1.38_392.9757m/z	392.9756515	1.380283333	neg	3361.175536	2165.064501	2110.925386	3659.023209	3204.360368	2886.29811	4207.507167	3593.978645	3357.51592
1.38_412.9999m/z	412.9998986	1.380283333	neg	2332.329604	1863.183207	2190.316163	4138.372081	3997.192101	3093.223791	3870.46793	3463.99008	3460.531301
1.38_420.9357m/z	420.9356723	1.380283333	neg	2235.883032	1989.404904	1983.923368	1371.252039	1391.336797	1397.587251	1960.342941	1599.776278	1700.684148
1.38_430.8912m/z	430.8912445	1.380283333	neg	2092.66	1646.369709	1436.339688	1147.762632	1287.213811	856.0610671	1251.970195	1346.856445	1650.299081
1.38_434.9820m/z	434.9819683	1.380283333	neg	2277.701441	1750.302454	1550.999947	3082.623056	3084.736551	2602.042842	3029.258553	3132.163271	3117.135445
1.38_460.9633m/z	460.9633216	1.380283333	neg	2160.894579	1267.364959	1460.360185	2167.758868	1814.028093	2277.108102	2500.546436	1859.963139	2284.925062
1.38_600.8111m/z	600.8111302	1.380283333	neg	1617.370171	1889.720125	1584.385084	3433.004426	3153.659193	2601.825677	2534.7458	1619.990546	2695.378334
1.38_604.8107m/z	604.8107416	1.380283333	neg	179858.1858	149014.5979	137247.097	164215.1594	177334.6291	169807.3003	186382.8502	149937.7023	161124.0233
1.40_204.0515m/z	204.0514747	1.3958	neg	1698.438429	1090.165898	1063.768349	1302.744909	1156.888228	1082.496264	1326.381257	1327.590782	1194.490542
1.40_281.8750m/z	281.8750391	1.3958	neg	1013.53073	895.9670851	807.7381537	987.3081462	1012.185292	756.2600959	1012.91356	558.9739094	1137.403251
1.40_314.9591m/z	314.9590602	1.3958	neg	1196.26722	783.4879794	727.5243418	1204.434518	1363.097915	723.4849389	1506.671425	1517.754687	1255.18921
1.40_322.9615m/z	322.9614781	1.3958	neg	1127.654674	875.5681215	893.2374631	1458.622795	1147.56548	1042.703107	1317.738359	1459.833288	1199.169536
1.40_324.9592m/z	324.9592365	1.3958	neg	1204.430424	581.2360572	851.3216435	1512.998464	1257.700347	972.8223257	1260.07673	1087.190376	1138.336986
1.40_384.0273n	429.02539	1.3958	neg	3284.419789	2319.409109	2403.085242	4252.937442	3829.918553	4003.669407	5013.617672	4306.733529	4338.421071
1.40_536.8226m/z	536.8225761	1.3958	neg	205705.3697	182935.2061	160658.1461	192527.9808	199736.2525	192008.6379	215053.7883	170163.6899	180792.69
1.41_232.9440m/z	232.9440109	1.40785	neg	2188.993802	2088.111169	1835.687807	2917.776953	3201.621457	3023.932553	2294.250317	2180.060928	2007.083677
1.41_278.9710m/z	278.9709931	1.40785	neg	1310.31095	1134.372961	1030.266031	1079.354466	1062.338381	895.0108918	1390.845618	1062.859077	1019.132848
1.41_281.8627m/z	281.8627448	1.40785	neg	1958.946496	1974.153948	1258.842954	2038.585902	1951.048356	1776.713812	2000.06792	1723.495765	1646.272986
1.41_379.0512m/z	379.0512326	1.40785	neg	520.1672339	688.1734369	507.2024918	950.737467	1133.854782	918.5044744	657.8596968	765.246595	561.7515697
1.41_470.8869m/z	470.8869338	1.40785	neg	987.514918	1088.25634	1178.974998	1114.746212	1068.329109	858.7066314	1271.588825	1327.935647	1327.96532
1.42_143.0350m/z	143.0349909	1.421683333	neg	397.9180909	397.242738	431.451998	551.1849255	515.3078869	438.9283015	478.4028648	437.5608768	315.6543416
1.42_190.9657m/z	190.9657466	1.421683333	neg	7898.834448	7996.348991	6949.130292	5182.73194	4512.13168	4161.842859	6974.756049	5831.753073	6575.88407
1.42_192.9813m/z	192.9812783	1.421683333	neg	106247.3601	99590.71998	82298.27094	72992.12068	63874.28073	57495.67564	100764.8941	84925.93806	94552.84083
1.42_194.9771m/z	194.9771411	1.421683333	neg	4574.469218	4428.521364	3713.168073	3324.473896	2781.89268	2447.576929	4479.962116	3613.314075	3916.059734
1.42_250.9401m/z	250.9400719	1.421683333	neg	1927.904839	1807.689552	1251.520256	1197.418099	901.5340344	918.1109505	1581.136014	1191.778042	1658.235234
1.42_256.9207m/z	256.9206577	1.421683333	neg	56466.22665	49340.10732	45704.0791	51299.31326	54663.70383	52337.06518	58886.81909	48186.0817	50279.36348
1.42_260.9689m/z	260.9688988	1.421683333	neg	13010.55751	12055.74838	9945.577626	8929.404406	7867.520398	7256.303382	12168.91003	10003.74894	11367.82257
1.42_277.9589m/z	277.9589488	1.421683333	neg	3372.14942	3561.90849	2990.891156	2355.126488	2007.574582	1625.763531	3158.171947	2633.574135	2814.568869
1.42_283.9581n	328.9566099	1.421683333	neg	9439.913643	9105.482136	7386.541097	6645.491324	5788.106787	5696.493403	8870.037432	7088.897144	8226.344386
1.42_292.9063m/z	292.9063058	1.421683333	neg	3638.112832	2664.701214	1766.643724	1644.657138	1441.987595	1429.689215	2491.51668	1833.36681	2429.504908
1.42_312.9313m/z	312.931328	1.421683333	neg	5107.023281	5209.076221	4330.647141	4489.395553	3585.18139	3355.719799	5416.746579	4742.926897	5313.490177
1.42_314.0997m/z	314.0997463	1.421683333	neg	2004.255242	591.3440813	472.075361	1404.089212	798.9525268	987.0944345	401.6109484	492.9188766	1183.034708
1.42_314.8728m/z	314.8727828	1.421683333	neg	11827.09611	11793.49577	10323.80693	13756.48657	13435.44228	13305.14557	14861.20288	10773.9036	12399.01366
1.42_335.9203n	334.912981	1.421683333	neg	6496.000129	5570.902598	5394.632728	6453.811727	5568.295606	5423.064957	6456.924593	5133.254828	6401.650693
1.42_345.9466m/z	345.9466145	1.421683333	neg	264.796517	380.4884441	203.02079	167.1848574	209.3599376	277.9441895	302.2666891	213.604394	335.4383794
1.42_349.8622m/z	349.8621591	1.421683333	neg	4642.570866	3544.503237	2888.88664	3797.383141	3661.864604	3311.953283	3756.999834	3091.482671	3148.418229
1.42_350.8676m/z	350.8676354	1.421683333	neg	735.2652468	483.9738221	578.5890889	778.4006125	445.8403546	663.8777323	606.245388	354.9780994	718.0664982
1.42_351.9453n	350.9380482	1.421683333	neg	6468.484022	5495.731777	5221.077503	5088.811309	4206.835251	3725.772061	6825.375186	5921.36072	5549.568854
1.42_360.8937m/z	360.8937241	1.421683333	neg	1993.878691	1859.137392	1138.917181	1236.956158	943.2259876	932.6845466	1731.283362	1300.863998	1490.753811
1.42_373.1003m/z	373.1003297	1.421683333	neg	1351.000092	1244.600601	729.290607	1833.607394	1741.15661	1844.133269	1374.210277	944.5150686	1193.475331
1.42_380.8631m/z	380.8631195	1.421683333	neg	32057.67343	30273.76363	27450.90225	33586.53929	34229.9816	35550.89552	39522.60601	31848.87513	32413.01607
1.42_381.0136m/z	381.0135994	1.421683333	neg	2593.541211	2171.637009	1705.654901	203.2969673	171.0007058	68.00162806	2025.019521	1405.527931	1783.620658
1.42_402.9001m/z	402.9001259	1.421683333	neg	3087.069786	3349.826753	2636.467091	2525.709528	2353.329333	2089.730269	3483.360679	2983.854861	3145.695101
1.42_460.0478m/z	460.0477827	1.421683333	neg	2809.101175	1898.537108	1861.256526	0.001403199	0.001403199	0.001403199	1750.307907	1381.818505	1354.993406
1.42_464.9312m/z	464.9311933	1.421683333	neg	2372.264031	2415.589763	2208.450162	1711.952155	1595.006036	1876.255855	2629.339479	2372.665917	2186.117662
1.42_479.9803m/z	479.9803086	1.421683333	neg	1058.759597	958.6303512	841.3651848	1783.839725	1677.287778	1240.691939	1199.297859	1198.333173	1137.651679
1.44_141.9126m/z	141.9125517	1.43735	neg	971.0918742	773.215068	585.6131905	765.3961535	719.0383529	723.6709068	922.4325063	669.1969605	748.9939391
1.44_164.9864m/z	164.9863982	1.43735	neg	149.7501168	139.2044702	60.12756636	73.08867446	67.71909592	112.7212878	131.4579551	99.38237	165.0932273
1.44_198.9365m/z	198.9364961	1.43735	neg	3195.519265	2816.403644	2288.53868	2979.017476	2720.641972	2580.519229	3272.026788	2585.695812	2500.764418
1.44_220.0651m/z	220.0650639	1.43735	neg	2186.984839	2012.132251	1507.694654	3726.764475	3473.796108	3229.980434	1846.912111	1471.679245	1881.723061
1.44_250.0393m/z	250.0392784	1.43735	neg	261.3823983	218.7740516	136.496251	1663.558959	1185.03271	1435.776536	985.7118208	581.1939937	1071.837681
1.44_258.0594m/z	258.0594395	1.43735	neg	534.2343989	536.0837576	285.1265973	650.0527916	517.3665689	447.1676008	449.6663799	333.32374	568.6151488
1.44_273.9033m/z	273.9033032	1.43735	neg	2422.77428	1802.291997	1456.797833	1998.722021	2075.948399	1947.396206	2376.50519	1770.174032	1778.523102
1.44_361.9967m/z	361.9967239	1.43735	neg	323.480703	624.6015706	451.2742148	1282.548862	932.3304125	1312.514884	546.7377881	389.1792541	682.2317929
1.44_407.8821m/z	407.882098	1.43735	neg	4975.009166	4619.975361	2973.298575	5219.969741	4728.594583	3839.347865	5582.95247	3277.278703	4037.495662
1.45_215.8686m/z	215.8685696	1.4461	neg	913.56135	757.4581712	522.519535	773.0542416	616.3883072	629.9883501	872.3865664	573.6324352	620.6689951
1.45_240.0491m/z	240.0490989	1.4461	neg	880.545986	724.4237145	531.5674295	830.186247	737.9299694	672.8446738	681.8084589	393.0536013	576.5881732
1.45_256.8406m/z	256.8405628	1.4461	neg	810.7556794	785.1438835	472.7685381	1100.30104	680.752385	746.3328959	937.7199368	602.5312372	710.0785102
1.45_258.0442m/z	258.0442075	1.4461	neg	1171.667285	1158.811084	906.267175	968.7333406	903.7758309	773.7096564	1047.691411	709.0387473	971.7025679
1.45_298.8352m/z	298.8351944	1.4461	neg	3987.061661	2769.941396	2139.343092	2304.17876	2025.296224	2063.193163	2854.977861	2368.768021	2250.996886
1.45_339.9118m/z	339.9117719	1.4461	neg	1784.315428	764.2463436	705.9511634	325.0742881	470.9779504	422.4258727	660.4442068	337.6938878	678.2909656
1.45_672.7981m/z	672.7981297	1.4461	neg	85150.95865	69815.22213	58931.00775	77409.52926	79977.61509	79115.14966	89076.69079	69746.66642	76128.41173
1.46_163.9289m/z	163.9289368	1.459966667	neg	3602.960684	2886.293829	2675.445232	2775.48771	2857.297885	2697.913113	3597.352893	2650.678718	2843.118251
1.46_176.9864m/z	176.9863778	1.459966667	neg	421.5887126	492.1163665	355.7027918	526.2451125	338.5165811	455.5412614	418.5646133	363.2192307	409.6683176
1.46_182.0401m/z	182.0401243	1.459966667	neg	1102.436567	1450.559116	1207.108132	1527.144479	1390.019709	1682.016579	1628.023722	1168.09845	1459.276973
1.46_266.0144m/z	266.0144283	1.459966667	neg	1577.457238	2020.903459	1825.485939	1941.844393	1654.359199	2033.758845	2252.159312	1607.221065	2020.765085
1.46_272.0135n	317.0117226	1.459966667	neg	1695.346116	2095.749404	1627.918824	2642.593055	2290.581201	2441.007319	2638.143745	1779.300389	2110.609399
1.46_279.0042m/z	279.0041534	1.459966667	neg	657.6786324	1038.925368	836.0724199	1243.598928	1095.415406	1204.815354	1472.061918	873.1425781	1153.191382
1.46_318.1041n	317.0991693	1.459966667	neg	2437.117311	3299.750119	2407.915369	2832.288146	2012.474402	2366.831726	2668.334911	1658.699471	2535.289534
1.46_331.0786m/z	331.0785771	1.459966667	neg	1878.078022	2031.079603	1848.92026	2167.854593	1769.680937	2131.08702	1674.991091	1364.876626	1624.556941
1.46_338.9938m/z	338.9937513	1.459966667	neg	1848.177692	2546.278724	2453.747596	3253.473028	2853.303947	2737.81231	3232.276943	2146.114882	2570.658409
1.46_342.0565n	387.0548935	1.459966667	neg	22716.08402	28764.76112	22984.36995	8728.102695	7595.771166	8460.558354	9167.165607	7045.973334	7792.027255
1.46_368.9733m/z	368.9733231	1.459966667	neg	554.032	746.4986327	609.3333851	1098.769241	692.4822325	806.3050724	864.8860986	531.3621127	777.0056353
1.46_377.0260m/z	377.0260074	1.459966667	neg	2709.862412	3598.606153	2907.964821	2061.852446	1635.341204	1820.823224	1419.851373	834.7578887	1233.072628
1.46_411.9043m/z	411.9043078	1.459966667	neg	495.386667	804.7848794	672.33577	0.001403199	0.001403199	0.001403199	948.7278396	676.4642128	887.6913952
1.46_417.0345m/z	417.0344663	1.459966667	neg	2154.972778	3005.398329	2585.086696	855.6511108	617.7673903	757.1121752	850.3093813	526.3997269	838.3434778
1.46_439.0164m/z	439.0163956	1.459966667	neg	4875.87083	7228.044864	5777.042601	1902.402476	1483.901436	1857.960444	1928.185215	1569.38097	1859.736634
1.46_448.8501m/z	448.8501037	1.459966667	neg	51090.82927	47764.34738	45740.25616	57388.72287	63150.42154	61584.33767	67044.0158	55620.96207	58740.32118
1.46_455.0424m/z	455.0424232	1.459966667	neg	9170.86227	12773.02368	9317.409349	3031.71519	2573.325355	2822.263157	3756.001182	2596.046565	2893.902591
1.46_460.9989m/z	460.9988876	1.459966667	neg	1556.125046	1978.211242	1911.854463	473.5993152	374.4768951	581.2529921	629.5630843	322.4578133	574.0142088
1.46_523.0297m/z	523.0297428	1.459966667	neg	5534.81351	6847.56103	5656.373164	1568.034238	1412.003152	1877.904608	1903.323189	1418.266208	1317.150354
1.46_536.1131m/z	536.1130997	1.459966667	neg	1023.654052	1432.108	1449.964784	1530.70062	1244.121796	1704.34393	1214.049163	705.0000466	1356.726606
1.46_554.9670m/z	554.967045	1.459966667	neg	1458.73195	2741.154663	1677.743184	418.4851498	313.3025901	455.5768273	276.0817191	281.3258972	685.0607905

1.46_591.0174m/z	591.0174249	1.459966667	neg	2504.78361	3414.731253	2754.703323	785.514865	580.7667622	739.728081	1086.825949	607.1537323	663.6967891
1.46_594.7808m/z	594.7807732	1.459966667	neg	3644.644551	3208.293439	1793.19806	3452.807774	2511.836756	2658.806678	3690.383981	2638.020177	2695.873566
1.46_594.8553m/z	594.8553125	1.459966667	neg	96177.05427	98120.01006	88091.16937	116334.7183	129603.0426	126449.9209	124173.8981	102801.7385	111182.8634
1.46_614.7654m/z	614.7654063	1.459966667	neg	9269.193827	6770.10966	5210.858277	6655.450601	7144.276878	5850.61624	8492.38725	5711.697048	6417.322376
1.46_808.7719m/z	808.7719179	1.459966667	neg	6039.378565	5267.284022	3867.52117	5151.587071	5388.009112	4469.530109	6231.044364	3906.902466	5123.042637
1.47_146.9615m/z	146.9614792	1.472133333	neg	0.001403199	0.001403199	0.001403199	75.27388991	68.09767828	28.82992407	29.81170049	0.001403199	22.67007298
1.47_152.9046m/z	152.904639	1.472133333	neg	1134.310703	865.6649936	879.4186974	1151.41134	1160.603125	1144.635594	886.8440092	776.1680927	835.269706
1.47_183.0275m/z	183.0275149	1.472133333	neg	1140.528536	1228.596748	1240.396401	1412.333948	1197.249202	1242.483933	1271.749187	1109.553552	1237.742654
1.47_195.8870m/z	195.8870285	1.472133333	neg	1968.359469	1770.757192	1491.319843	1840.693891	1998.434814	1717.549556	2340.066966	1570.805729	1830.203249
1.47_263.1169m/z	263.1168764	1.472133333	neg	41.0172478	133.0060633	132.5465797	137.2212892	41.19132479	78.07873318	116.7831438	66.58373586	105.4412861
1.47_273.1458m/z	273.1458408	1.472133333	neg	2327.931151	2451.140005	2284.725025	3240.51577	2596.820222	2643.081312	2698.637947	1737.438221	2526.530039
1.47_311.9799m/z	311.9798749	1.472133333	neg	11021.81783	12897.99923	11482.73151	888.9444804	997.0335297	658.1645001	14556.66794	11398.72467	12286.92799
1.47_324.8279m/z	324.8278874	1.472133333	neg	3175.767108	2940.107681	2301.761939	2903.328715	2804.054868	2838.438792	3592.617588	2793.160358	2736.219533
1.47_326.9672m/z	326.9672424	1.472133333	neg	872.929334	1028.796896	673.7065647	1202.046004	950.2139007	964.3095391	976.5282514	761.5670553	1253.002485
1.47_328.9699m/z	328.9699237	1.472133333	neg	487.8886506	845.0213391	724.3678465	74.48500765	19.09974505	4.229624413	806.8970193	625.3638871	753.0546326
1.47_363.9417m/z	363.9417054	1.472133333	neg	1453.504632	1939.574718	2012.376672	119.9478122	0.001403199	134.9371149	2368.463019	1943.333737	2240.51729
1.47_379.9673m/z	379.9672697	1.472133333	neg	2921.372472	3746.826387	3110.414329	215.9212005	0.001403199	218.5852248	4216.748359	3119.809783	3857.612054
1.47_385.9240m/z	385.9239805	1.472133333	neg	561.9621184	806.7933563	479.2828547	0.001403199	0.001403199	0.001403199	1135.367899	607.4948938	945.7920017
1.47_418.9923m/z	418.9923101	1.472133333	neg	2146.004656	2906.557619	2036.387768	825.0474427	660.5220158	678.9546299	613.1692645	405.2182846	497.1604325
1.47_445.0135m/z	445.0135415	1.472133333	neg	971.3291737	1259.402689	1079.173187	293.2080301	160.6911373	299.7799092	248.9497144	182.0489807	367.1200737
1.47_447.9549m/z	447.954904	1.472133333	neg	1549.391559	2142.706387	1647.011196	0.001403199	0.001403199	0.001403199	2374.290345	1683.674201	2124.079711
1.47_486.9797m/z	486.9796813	1.472133333	neg	2263.508964	2657.820308	1694.701538	522.176928	294.8757754	476.8342198	662.9724724	422.9294472	571.3809828
1.47_584.8262m/z	584.8262421	1.472133333	neg	2506.681647	1897.566699	1613.885538	2472.261513	2317.750841	2280.585836	2647.349926	2308.958797	2153.926489
1.49_162.0596m/z	162.0595501	1.492933333	neg	400.046926	370.0812572	392.1797408	313.9219225	366.482983	357.5349879	330.2322864	350.4749623	350.6211028
1.49_219.0042m/z	219.0042219	1.492933333	neg	582.4524915	538.0953641	719.5541177	602.5064523	473.9532344	573.3394646	623.1576009	548.7516573	607.6580989
1.49_240.9864m/z	240.986385	1.492933333	neg	471.2887358	482.1207886	635.0442282	505.6225919	515.8277513	492.9769067	549.6705015	456.6786526	510.6744252
1.49_250.8841m/z	250.8840595	1.492933333	neg	1133.567494	661.5766397	697.3816661	721.208145	616.2968135	718.4049738	845.7712681	629.2386126	876.2610032
1.49_270.0329n	251.0150755	1.492933333	neg	5147.734492	5904.185816	5803.861539	5393.109445	5211.701772	5328.070455	5545.848694	4979.071082	5699.799811
1.49_274.9764m/z	274.9763627	1.492933333	neg	718.8917129	764.4306679	783.01837	768.0892656	991.8103782	919.0726018	950.9973544	938.4441452	881.0953916
1.49_275.9576m/z	275.9575569	1.492933333	neg	175.093867	183.5395742	105.6651656	178.5801473	88.46000298	152.2233499	106.8412865	115.214125	92.65387877
1.49_283.0412m/z	283.0412143	1.492933333	neg	1061.257883	1268.179093	1123.988651	713.0615032	660.1939345	786.1141394	1079.476814	995.9353867	939.9221982
1.49_301.9509m/z	301.9509045	1.492933333	neg	510.1687885	605.9036751	474.3830103	0.001403199	0.001403199	0.001403199	808.5030038	601.2768846	859.2025739
1.49_302.9773m/z	302.9772718	1.492933333	neg	1156.434334	1724.758043	1953.355689	1722.820688	1708.80727	1813.378914	2011.785616	1595.534798	1960.243967
1.49_308.9737m/z	308.9737128	1.492933333	neg	309.6418464	587.6715666	443.3157193	520.1753678	342.6200079	345.354142	416.6231223	362.4041198	426.6050846
1.49_324.9591m/z	324.9590644	1.492933333	neg	1077.67104	1254.12124	1191.247878	1251.226459	1321.939109	1199.369145	1297.677871	1059.229051	922.7221735
1.49_338.0205n	319.0026546	1.492933333	neg	3995.046029	4357.874707	4539.791406	3854.824683	3895.837028	4118.359642	4580.940549	3876.722998	4105.68599
1.49_386.9901m/z	386.9901484	1.492933333	neg	1560.699343	1894.589676	1612.689405	1850.206606	1991.753533	1738.849158	2050.695342	1352.980884	1895.268593
1.49_392.9465m/z	392.9465456	1.492933333	neg	478.8617069	740.6631245	592.6632581	854.8055551	689.8902556	899.2716784	777.0800497	553.3151684	761.5640216
1.49_454.9773m/z	454.9773309	1.492933333	neg	1089.802279	1187.724354	1008.194288	1108.137847	1188.060338	1215.559315	853.4753928	981.1241331	1194.357195
1.52_162.0231m/z	162.0230958	1.515416667	neg	103.8150851	96.71115144	206.1925356	214.3807506	177.0469127	195.1731759	118.9402507	79.68433248	130.5189872
1.52_187.0282m/z	187.0281535	1.515416667	neg	142.557546	205.8841261	190.4641757	672.8794778	631.1166253	584.9059765	208.6451891	219.7273161	250.4296417
1.52_230.0469m/z	230.0469129	1.515416667	neg	351.3531549	471.8459707	589.5258665	621.0601604	468.8269186	604.0072241	525.7641993	479.1894564	456.604601
1.52_233.0067m/z	233.0067114	1.515416667	neg	344.6280323	378.7599746	387.213583	402.4009106	428.2359538	368.6813385	260.7917798	432.4758608	418.8319195
1.52_241.8925m/z	241.8925025	1.515416667	neg	270.8445118	166.0411847	162.8006264	297.9680644	266.6907536	177.3788841	188.0270836	153.3521439	234.5351074
1.52_276.9554m/z	276.9553943	1.515416667	neg	695.982629	597.4541024	554.9062048	687.8520816	468.8963055	542.5666553	717.0009279	571.8722252	599.6390558
1.52_278.0705m/z	278.0704529	1.515416667	neg	1181.453699	1105.726684	1079.117214	1723.495368	1313.135338	1490.941084	1078.99974	1090.330437	1028.020399
1.52_297.1193m/z	297.1192962	1.515416667	neg	847.5374636	548.9757341	689.7267076	394.2349461	433.2827459	447.5309006	368.7472748	505.0247675	494.8534565
1.53_174.9337m/z	174.9336731	1.534483333	neg	200.969138	156.1993283	128.9537032	152.0559581	111.0895813	109.3050895	121.409171	57.96738625	170.4002088
1.53_225.8854m/z	225.8854165	1.534483333	neg	297.0767934	281.6260732	255.7180815	278.169151	321.4884986	321.146992	305.0681088	295.0316678	295.2142171
1.53_238.0115m/z	238.011519	1.534483333	neg	767.0798554	634.38201	538.2743078	811.4514742	651.6131869	689.3710805	701.7108061	739.0979195	771.8246001
1.53_240.0087m/z	240.0086787	1.534483333	neg	113.6701559	69.1058788	121.4404093	100.9113676	70.89929183	195.1597469	136.8385628	194.7831133	151.7605362
1.53_265.0556m/z	265.0556376	1.534483333	neg	1165.211386	1193.501052	1175.451241	1528.92388	1519.77658	1405.035936	1161.540961	1570.269224	1457.826811
1.53_325.9832m/z	325.9832209	1.534483333	neg	2195.704351	1755.912343	1652.357763	1646.87035	1967.915754	1754.298433	1599.468962	2107.578622	1752.179341
1.53_335.0536m/z	335.053552	1.534483333	neg	1080.636429	902.1929144	1101.468076	1117.254528	1003.344493	1177.649044	1065.284512	1418.427316	1299.840508
1.53_393.9704m/z	393.9703742	1.534483333	neg	739.9731968	601.636355	702.7944055	619.4261073	613.2522189	582.2920395	523.9665062	559.1289572	775.6722101
1.53_419.0664m/z	419.0663786	1.534483333	neg	1627.461225	1229.622381	1148.226502	877.4293951	881.444665	1047.292648	993.7536151	1442.531128	1131.674213
1.53_461.9584m/z	461.9584241	1.534483333	neg	635.6228905	553.9157443	585.0994406	439.9371225	594.817621	532.2112566	560.165399	514.5580699	606.6464124
1.53_487.0537m/z	487.053673	1.534483333	neg	860.6544925	591.3751817	683.9673994	429.907631	467.2435522	640.5253889	343.6293751	664.4443207	832.9916686
1.53_529.9460m/z	529.9459885	1.534483333	neg	712.9759808	705.4485191	815.9997348	646.3867294	612.692288	657.3683762	695.2708968	703.650882	493.6933052
1.55_159.9824n	204.981367	1.553483333	neg	7233.385835	5908.822774	5996.923599	16608.40832	15806.38772	15798.83473	5622.931453	6926.246767	6096.887891
1.55_160.9988m/z	160.9988234	1.553483333	neg	1029.117985	831.1514521	838.0876636	1396.90838	1326.470943	1369.91048	1041.426982	1379.751332	1089.6712
1.55_171.0276m/z	171.0276059	1.553483333	neg	18487.02763	17185.50862	17023.94077	29394.89153	28004.15377	26250.55399	20086.41282	23795.49705	21243.59016
1.55_202.9651m/z	202.9650608	1.553483333	neg	3499.711403	2756.960352	2497.261868	4104.665268	3759.8811	3776.443199	2961.639889	4106.371183	3011.009347
1.55_223.9931m/z	223.9931247	1.553483333	neg	30.87524687	23.79676115	47.22427338	100.8795394	99.10055838	72.40987611	42.82803394	101.9782896	32.593052
1.55_227.9051m/z	227.9051497	1.553483333	neg	55.35616337	136.5572258	171.0363195	217.0963826	280.2896353	197.8548951	228.1454417	256.5852879	174.9247079
1.55_228.9129m/z	228.9128574	1.553483333	neg	81.58598529	127.8739911	75.61242922	203.377259	142.6579883	179.5555871	158.3361699	181.0406799	88.86054052
1.55_229.9020m/z	229.9019824	1.553483333	neg	104.3571189	43.30791256	107.3714743	131.4551658	144.9126973	145.1782529	100.3766949	135.3573142	131.4416684
1.55_230.9098m/z	230.9097952	1.553483333	neg	43.54009605	0.001403199	51.69176065	104.0952332	113.0777338	119.3568128	44.04195284	35.87134552	86.54897027
1.55_239.0151m/z	239.0151067	1.553483333	neg	3983.565592	3511.363791	3096.253722	5783.456285	5570.314742	4981.447351	3766.682556	5534.366353	4101.256931
1.55_244.9715m/z	244.9715072	1.553483333	neg	275.5608486	177.9054077	246.3240393	601.9316799	468.1761758	551.6760671	330.1500582	537.2870983	358.6544527
1.55_245.9470m/z	245.946993	1.553483333	neg	1882.882775	1521.712964	1475.536392	2185.039995	1907.021402	1919.574715	1529.196243	2067.162546	1594.706558
1.55_254.9262m/z	254.9262107	1.553483333	neg	2503.679955	2103.460295	2027.846367	3409.187004	2900.810503	2679.055124	1943.896239	2130.34241	2870.183233
1.55_262.0756m/z	262.0755522	1.553483333	neg	1379.744032	1134.777895	1041.352647	1120.86031	1058.62235	1082.985577	909.5483678	1466.51428	1102.512382
1.55_265.0305m/z	265.0304562	1.553483333	neg	1038.054803	856.4845101	880.8222323	1174.697454	1246.906668	1233.887199	879.0067467	1290.94461	879.4530556
1.55_271.0938m/z	271.0937691	1.553483333	neg	291.7214523	202.5379913	248.7758805	1004.285163	930.2875319	1010.375927	450.0526748	685.5130787	460.9944078
1.55_271.9599n	542.9024005	1.553483333	neg	4313.399533	3100.486497	2799.598893	4229.00943	4304.182987	4166.242812	3724.895484	4693.656234	3230.048938
1.55_276.9087m/z	276.9087191	1.553483333	neg	543.7554042	539.7134313	522.9831426	940.3157183	964.8985317	861.876763	588.8414801	774.2450724	600.2513977
1.55_300.0023m/z	300.0022926	1.553483333	neg	1261.757135	1187.137904	1190.342126	1641.616149	1457.81937	1482.165316	789.5548489	1525.824021	1350.001664
1.55_307.0026m/z	307.0026139	1.553483333	neg	8452.985623	8002.989646	8107.279643	12859.24014	12498.15876	11171.24661	8924.773303	10667.49391	9690.37212
1.55_312.9591m/z	312.9591494	1.553483333	neg	788.8398957	591.5154793	583.4606189	1561.053688	1208.205955	1373.933677	812.4552967	1128.28998	852.8191181
1.55_313.9348m/z	313.9347679	1.553483333	neg	495.323367	396.7581263	422.6089401	738.3028499	611.9044911	717.6933991	443.9001482	672.8423386	477.6409879
1.55_315.9543m/z	315.9542747	1.553483333	neg	1086.060305	740.5198223	533.3593252	802.7542807	1042.149282	691.6917783	596.8249569	616.0470854	693.264942
1.55_316.0278m/z	316.0277629	1.553483333	neg	1873.778733	1572.893112	1374.415672	1629.858627	1597.300803	1704.341753	1511.572275	1802.088154	1415.305208
1.55_317.0517m/z	317.0516767	1.553483333	neg	1896.307358	1390.188237	1321.444325	479.6975715	552.5831159	515.5725719	204.1072699	350.9288826	310.7754767
1.55_338.9398m/z	338.9397955	1.553483333	neg	4699.278064	3355.022704	2762.396183	4897.064985	4963.794962	4564.434196	3629.425232	4838.685065	3534.117844
1.55_348.8954m/z	348.8953775	1.553483333	neg	853.1862274	466.1349211	332.0287948	640.8603979	557.3920689	490.8501294	385.9902676	479.6545258	217.7332567
1.55_355.9299m/z	355.9299227	1.553483333	neg	225.3730329	126.9502423	105.4640109	226.7473151	181.9148496	210.015469	150.611985	243.6153603	172.921838
1.55_364.9611m/z	364.9610997	1.553483333	neg	948.1941538	936.3481377	601.7351285	1225.327284	1179.014758	1113.035018	881.6026919	1259.142426	915.3495107
1.55_365.0445m/z	365.0444915	1.553483333	neg	259.8828224	254.8897271	151.7603495	685.6101799	540.1407952	543.6053644	295.0835178	468.5537548	213.3285158
1.55_374.9901m/z	374.9900547	1.553483333	neg	5325.282357	4406.625968	4150.835793	7581.964571	7468.831911	6996.044037	5001.092111	6811.843596	5891.989383
1.55_380.9464m/z	380.9464241	1.553483333	neg	887.7002171	998.1354121	542.4455385	1416.435535	1468.419946	1605.737571	1082.50984	1401.840774	1162.817845
1.55_381.9221m/z	381.9221478	1.553483333	neg	1472.897117	1069.741993	1081.37099	1759.026033	1885.641803	1695.867611	1272.00425	1631.945046	1522.060536
1.55_406.9273m/z	406.9272561	1.553483333	neg	3464.488443	2326.667611	2034.160536	3741.663195	3299.119545	3478.201527	2559.494196	3770.578585	2940.532503
1.55_442.9774m/z	442.9774443	1.553483333	neg	5723.700058	5724.782151	5554.505101	9176.074222	9216.734645	8039.683028	6388.999074	7402.360819	7128.933667
1.55_448.9337m/z	448.9336965	1.553483333	neg	1180.291366	904.9007602	1070.523281	2200.521312	1601.036509	1922.790463	1434.028198	1687.701767	1178.697518
1.55_449.9097m/z	449.9096669	1.553483333	neg	1439.338077	931.2894837	869.1620119	1204.986218	1223.82553	1467.52651	1165.458375	1416.643802	1138.967682
1.55_474.9146m/z	474.9146167	1.553483333	neg	3048.563031	1914.721349	2152.37704	3113.69394	2695.040488	2420.716205	2161.991537	2754.424301	2397.384743
1.55_480.8712m/z	480.8711708	1.553483333	neg	885.8546617	400.0108309	354.7744974	471.7627967	676.3304584	710.2422666	531.4655986	680.9120761	342.3761538
1.55_510.9647m/z	510.9647487	1.553483333	neg	3268.878225	2643.629359	2291.197904	3985.01134	4078.575978	3947.933058	3191.697172	4019.447379	3024.511611
1.55_516.9211m/z	516.9210834	1.553483333	neg	1084.202099	871.8854453	750.0408466	1604.297508	1586.873755	1823.008047	846.1865354	1469.853614	931.7154078
1.55_517.8972m/z	517.8971603	1.553483333	neg	747.7288117	482.6986995	588.2410373	574.8810452	997.0668912	908.11765	756.1737895	1010.72157	816.5921711
1.55_578.9528m/z	578.9527574	1.553483333	neg	2309.28415	1664.492506	1588.392267	3254.259334	3534.627232	3391.174137	2374.404618	2745.951621	2072.763063
1.56_162.9959m/z	162.995872	1.56225	neg	301.8921669	253.3452318	220.4193116	398.5320562	397.9568365	358.2793925	287.0652707	365.2458935	302.8752021
1.56_192.9362m/z	192.9362134	1.56225	neg	1016.423552	802.9099719	723.4319498	1164.995837	1144.204826	1124.377814	935.9761186	1229.093049	910.5288705
1.56_237.9341m/z	237.9340788	1.56225	neg	663.0468598	412.7966354	301.9753382	443.0904242	696.5855149	458.8587508	447.1847866	434.3916454	525.0785841
1.56_247.9627m/z	247.9627463	1.56225	neg	2304.378341	1502.933933	1479.777456	2247.472327	2154.191215	1999.426533	1912.9962	1914.128682	1839.93422
1.56_272.9689m/z	272.9689126	1.56225	neg	896.745059	664.0127908	601.2554106	2556.049873	2523.411177	2355.970929	518.876652	748.3045396	671.7475829
1.56_296.9736m/z	296.9735581	1.56225	neg	741.3761852	542.668641	539.8083359	867.6046035	888.0616509	1053.788391	782.5011973	1121.493034	764.0564667
1.56_298.0617m/z	298.061731	1.56225	neg	801.539304	562.4645935	595.8725302	747.8728285	711.1236861	665.7425888	487.3134549	820.3928165	568.8690783
1.56_306.0584m/z	306.0583519	1.56225	neg	107.0100203	86.69941197	129.0100289	175.0148517	128.4890084	175.154339	91.06583046	236.2846031	200.494065

1.56_340.9561m/z	340.9560886	1.56225	neg	121.7169596	146.6202788	265.6927373	1320.34112	1085.371168	1206.948833	262.559211	304.5362434	147.1366155
1.56_381.1114m/z	381.1114139	1.56225	neg	0.001403199	0.001403199	53.62626614	151.0933044	143.0858296	76.71813941	120.0079529	169.926829	0.001403199
1.56_412.8837m/z	412.883674	1.56225	neg	835.627515	641.8785953	370.1455218	597.2815443	558.825958	590.960226	634.0694049	623.0501797	581.5728057
1.56_417.0113m/z	417.0112821	1.56225	neg	951.6213361	653.6409926	678.9597869	162.7401958	97.47839714	41.1576671	347.3545916	453.487587	421.0947835
1.56_451.0586m/z	451.0586244	1.56225	neg	970.8285343	629.955288	962.8813377	888.3660108	725.8710804	867.9080008	646.8039619	866.1542816	786.7285631
1.56_646.9404m/z	646.9404401	1.56225	neg	1652.424309	1455.130416	1458.046517	2025.190327	2432.711951	2414.485767	1635.023871	1955.499222	1646.180008
1.58_250.0335m/z	250.0334918	1.577833333	neg	440.2517695	398.2332015	405.6617159	900.5951114	872.352084	805.9413247	275.1529189	413.017473	332.8587349
1.58_270.0962m/z	270.0961797	1.577833333	neg	146.7302535	204.8689527	278.8412917	484.4108328	256.1426439	441.0886781	307.7056144	380.9978676	309.7003047
1.58_332.0740m/z	332.0739807	1.577833333	neg	1506.955797	1019.968381	1077.356285	1360.077074	1392.089893	1761.764346	1065.643759	1680.278253	1237.877152
1.58_854.3264n	899.3256572	1.577833333	neg	21650.21583	16670.1534	14989.50801	12862.64737	12755.75278	12867.51577	13039.57779	21124.96762	17130.49644
1.59_202.0254m/z	202.0254076	1.5934	neg	1620.141407	1350.143656	1293.92181	1362.974159	1146.908733	1351.842224	1394.47808	1483.199962	1482.566937
1.59_204.0224m/z	204.0223745	1.5934	neg	572.4707494	542.7427424	489.0419492	410.998479	430.934371	455.1161857	540.4846819	611.5284633	544.1518406
1.59_264.0161m/z	264.0161172	1.5934	neg	2302.974574	2264.778669	2302.061572	2349.084448	2278.823523	2104.290472	2312.681142	2637.591427	2534.546444
1.59_279.9683m/z	279.9682873	1.5934	neg	1812.212607	1509.910482	1564.633727	1253.608177	1244.004948	1124.207274	1367.914728	1535.989577	1448.156049
1.59_280.0417m/z	280.0416754	1.5934	neg	6087.302609	5370.924445	5519.291847	5615.147377	5811.372569	5753.39564	5771.526999	5812.054826	6237.53527
1.59_289.9972m/z	289.9971625	1.5934	neg	4402.903313	3550.394557	3654.171802	2894.578042	2885.919281	3119.817509	3737.616676	3739.353821	3278.101736
1.59_348.0293m/z	348.0292504	1.5934	neg	4237.757689	3738.341442	3874.821689	3463.77932	3865.827001	3718.736413	3941.486467	3808.092411	3899.830981
1.59_355.0527m/z	355.0527359	1.5934	neg	2636.469377	2306.473508	2588.585969	2943.483483	3027.409385	3028.108422	2655.470367	2664.258468	2744.744263
1.59_357.9847m/z	357.9846761	1.5934	neg	3028.541092	2383.617429	2754.970308	1850.482492	2035.682273	2224.522479	2512.327331	2477.432703	2689.548999
1.59_397.0755m/z	397.075469	1.5934	neg	641.9825453	630.23989	659.7077129	488.9086968	573.8553517	506.3108188	613.5717691	611.7755896	540.7970054
1.59_414.9459m/z	414.9459165	1.5934	neg	651.0788318	580.5035983	402.8040166	485.2292377	693.6402452	641.6304486	665.1219724	606.2116904	631.1008777
1.59_415.9436m/z	415.9435551	1.5934	neg	365.749168	227.9163337	162.480155	181.5199485	166.2383277	123.8446417	191.8980214	279.537728	428.223545
1.59_416.0167m/z	416.0167104	1.5934	neg	3401.988183	2934.190072	2803.254467	2613.284197	2743.145115	2787.388153	3206.605968	3358.944786	2765.682889
1.59_425.9723m/z	425.9722945	1.5934	neg	3434.164607	2515.393603	2616.655237	1809.874317	1907.674846	1742.707566	2074.558828	2390.704632	2679.396721
1.59_484.0043m/z	484.00425	1.5934	neg	2079.730791	1757.072111	2024.626931	1519.832529	1766.109243	2152.509328	2242.221688	2158.658119	2371.000968
1.59_493.9597m/z	493.9597019	1.5934	neg	3226.334776	2678.085145	2389.255883	1899.326556	2222.181411	2047.871809	2458.099244	2275.688103	2729.216002
1.62_136.9099m/z	136.9098511	1.615883333	neg	3831.842891	3166.645553	2879.382539	3003.073704	3159.374831	2793.728644	3698.823714	3074.912927	3205.90194
1.62_141.9125m/z	141.9125155	1.615883333	neg	532.4352395	451.0522291	441.6033147	437.9599445	441.8527018	433.3044391	557.0603041	441.5738101	519.7563555
1.62_180.8998m/z	180.8997956	1.615883333	neg	36800.55703	29229.39015	25121.91427	27932.39737	26983.41479	24825.61599	33922.42403	25696.17387	28202.75272
1.62_190.9823m/z	190.9822688	1.615883333	neg	453.041559	475.7973147	471.1775247	240.9535358	205.0327096	159.2185163	367.8507645	347.3352177	396.9999737
1.62_224.9136m/z	224.9135641	1.615883333	neg	852.5526256	705.9582008	569.9490549	1049.846837	1039.636079	963.8331205	1544.518652	1331.08067	1391.195386
1.62_226.9114m/z	226.9113838	1.615883333	neg	355.9667367	316.2430347	397.1510028	471.1139399	573.8028847	517.5560518	657.414739	695.8228498	788.8614928
1.62_256.9740m/z	256.9740047	1.615883333	neg	1565.255279	1642.959745	1680.374788	866.6083964	714.8920862	774.5604589	1361.488449	1384.624281	1454.253419
1.62_346.9586m/z	346.9585845	1.615883333	neg	1762.481826	1442.683805	1844.938006	1554.601452	1742.501559	1634.779539	1745.341327	1488.160369	1728.179529
1.63_215.8686m/z	215.8685738	1.631516667	neg	681.2778422	550.7405937	643.3173258	401.0513053	548.5686199	603.86449	714.520292	563.3436296	552.9187268
1.63_224.9940m/z	224.9939724	1.631516667	neg	938.603282	932.2021139	1203.771782	902.7191696	1013.114022	979.1537412	1199.237667	1031.281583	1139.580839
1.63_260.0034m/z	260.0033866	1.631516667	neg	87.3852545	0.001403199	105.7230106	24.9595101	0.001403199	0.001403199	74.60002862	102.9828491	113.2231006
1.63_286.9846m/z	286.9846193	1.631516667	neg	857.3736653	1222.92514	1644.058475	1561.337849	1543.221141	1666.201645	1842.019733	1595.541345	1505.68732
1.63_298.0427m/z	298.042704	1.631516667	neg	7210.209528	7966.539399	8875.172619	11547.48568	11358.26623	11883.56741	12866.93995	11954.64993	11474.6956
1.63_338.8595m/z	338.8595286	1.631516667	neg	760.1817897	189.4295795	142.8638255	155.8158309	185.0148194	148.1466935	216.0252426	223.5426598	233.1500226
1.63_366.0302m/z	366.030237	1.631516667	neg	2084.145671	2446.771747	2486.52729	3465.664196	3613.085917	3750.495684	4338.592882	3890.073625	3480.844287
1.63_370.9975m/z	370.9975187	1.631516667	neg	1950.183561	2407.227887	2621.341202	2688.845501	2350.374901	2254.479953	3140.0957	2739.265174	2455.88838
1.63_434.0178m/z	434.0178152	1.631516667	neg	1110.380339	1021.160877	1211.899557	1497.009518	1719.783064	1500.025161	1732.479246	1751.205394	1494.120963
1.63_462.8056m/z	462.8055722	1.631516667	neg	3393.083829	2980.018905	3203.174351	2249.068561	2534.682996	2336.438405	3085.068667	2254.212952	2215.988634
1.63_526.7930m/z	526.792994	1.631516667	neg	7474.06493	6138.538036	5826.053541	5585.439813	5971.822945	6032.830324	7510.298192	6445.170361	7034.743614
1.65_177.0102m/z	177.0102484	1.65405	neg	1356.415155	1380.860891	1667.383652	430.9330766	331.8967271	329.9672699	1728.934069	1259.338114	1354.516504
1.65_218.0494m/z	218.0493945	1.65405	neg	9353.954412	9878.42698	11324.76329	2700.677546	2552.074567	2623.029928	10099.2002	8736.895184	9273.938593
1.65_247.0396m/z	247.0396008	1.65405	neg	1146.813642	1431.884508	1865.023485	1501.254984	1566.365652	1388.843923	1585.830011	1384.694059	1398.457396
1.65_252.0128m/z	252.0127602	1.65405	neg	491.8159094	658.2370495	774.4821787	727.4229883	743.3127584	828.5750326	1070.634475	778.074934	814.0179611
1.65_266.9600m/z	266.9599958	1.65405	neg	638.491383	675.3320801	753.259655	700.4182947	687.8075503	658.8065531	875.9306847	723.71198	667.0028665
1.65_278.9712m/z	278.9711994	1.65405	neg	1219.197759	886.226017	1081.97361	1217.393498	1300.055189	1064.540515	1238.56708	1075.112549	1274.812326
1.65_286.0369m/z	286.036915	1.65405	neg	6799.723242	7684.185228	8616.142014	1905.456366	1737.877594	1867.499781	7527.847808	6403.63447	6693.795092
1.65_302.0520m/z	302.0520381	1.65405	neg	5885.003217	6149.827529	5917.84081	7382.106295	6684.364022	5781.510922	5454.367935	6473.995391	5510.406499
1.65_324.0339m/z	324.033907	1.65405	neg	1385.989486	1494.99887	1653.514016	1752.163316	1739.46175	1599.017806	1596.290566	1601.362405	1557.189132
1.65_337.9993m/z	337.9992681	1.65405	neg	1436.340639	1656.09527	1674.309674	542.5573993	795.9732576	465.3454882	1609.195977	1299.752257	1610.028844
1.65_342.9985m/z	342.9984986	1.65405	neg	1681.391349	1592.375152	1643.385018	1662.45412	1549.786872	1323.550733	1790.431187	1667.78694	1722.257214
1.65_354.0245m/z	354.0244588	1.65405	neg	3664.963905	4061.685315	4454.810562	879.847252	993.8959239	954.5825538	4012.753984	3275.788707	3671.014362
1.65_374.0780m/z	374.0779847	1.65405	neg	1587.440322	2057.964307	2069.035226	2529.540074	1891.598055	1789.129196	1617.786351	1865.954314	1532.704134
1.65_422.0122m/z	422.0121999	1.65405	neg	2470.818827	2845.528806	3236.444045	452.6793429	612.342782	645.9521882	2498.712614	2460.13601	2458.927597
1.65_438.9852m/z	438.9852037	1.65405	neg	1667.293758	1530.422792	2034.621927	1763.929759	1824.788258	1860.645187	2500.05599	2032.243271	2132.263208
1.65_442.0654m/z	442.0653913	1.65405	neg	291.3883194	528.0349148	220.8627348	539.5101281	485.0311484	479.2829242	331.6051947	494.7711011	332.6142266
1.65_489.9993m/z	489.9992995	1.65405	neg	1705.042274	1681.365137	2121.481614	298.7925756	315.0318487	323.0619455	1816.623494	1526.896326	1592.127203
1.65_808.7722m/z	808.7722299	1.65405	neg	5020.530574	5373.405034	4796.314808	5535.335893	5092.494946	4972.295678	6051.27193	5850.696201	5354.674492
1.67_213.0382m/z	213.0382397	1.66975	neg	516.4949136	495.2782477	915.3111995	459.3261314	368.8615215	356.6869779	581.6869094	307.3184013	409.5069441
1.67_226.9908m/z	226.9908445	1.66975	neg	619.7843939	821.5657226	1021.317071	240.7303083	268.9705476	243.4737662	373.3350211	296.3140992	310.8014841
1.67_229.0695m/z	229.0695145	1.66975	neg	383.1584677	543.8609815	642.9364429	945.5209656	735.7307682	689.1936719	471.9326575	551.695624	718.3069196
1.67_242.0649m/z	242.0649144	1.66975	neg	465.0231644	411.2790931	569.4292199	335.3904715	412.2018553	292.7404612	473.9355828	479.998414	430.8807595
1.67_269.0215m/z	269.0214652	1.66975	neg	1074.954807	1320.488439	1374.721335	1051.106531	1183.393521	1013.779627	1237.909954	1036.847744	1044.068954
1.68_104.0435m/z	104.0434673	1.68365	neg	35.14313928	84.26767915	58.36464764	139.6869885	128.4871231	88.41164719	62.30098248	70.64990761	79.46360196
1.68_160.9988m/z	160.9987801	1.68365	neg	643.5402986	748.38626	877.2435651	1277.78708	1154.932462	895.6930542	944.3980876	741.9212629	862.2877075
1.68_188.0178m/z	188.0177891	1.68365	neg	703.6298585	917.8858887	1072.157408	1601.030059	1370.107917	1179.790634	1106.697979	974.352232	1184.973127
1.68_192.9362m/z	192.9361565	1.68365	neg	173.6914706	254.1812224	204.8149937	528.0967928	396.2178433	311.3915961	197.0413977	251.5212872	268.3721359
1.68_270.9527m/z	270.952686	1.68365	neg	862.7095485	847.8107066	980.208357	1475.747838	1120.245654	896.5869567	855.8129861	751.4459451	891.755732
1.68_296.9736m/z	296.9736203	1.68365	neg	261.5053033	505.0736846	246.2083452	564.9815731	474.4911376	251.2427289	420.5304245	163.8029633	548.4657995
1.68_312.9590m/z	312.9590161	1.68365	neg	648.4429346	730.0072086	915.0966983	1416.245576	1147.089103	944.7655126	958.7845159	747.4376592	880.0754216
1.68_364.9613m/z	364.9612723	1.68365	neg	308.8113411	612.5629403	642.7321307	853.9609036	1004.90852	748.4199913	566.6261357	594.1569154	823.9585525
1.68_375.9937m/z	375.9936802	1.68365	neg	0.001403199	189.2571496	61.52943968	264.2869631	353.2299103	226.681574	102.3824624	180.7851563	156.3259224
1.68_380.9464m/z	380.9464374	1.68365	neg	439.0448928	662.6848219	603.6027362	1478.286533	1177.033657	637.0833728	641.7743051	553.7065619	732.9174109
1.68_510.9646m/z	510.9646493	1.68365	neg	1351.953535	2012.069247	1719.745591	2900.369155	2230.442174	1644.473452	1702.92878	1405.387959	2070.144996
1.70_138.0308m/z	138.0308381	1.6992	neg	186.6878864	248.1172032	200.394382	220.8084146	256.6359631	261.2719313	239.9433005	175.5035563	324.2673907
1.70_245.9469m/z	245.9469395	1.6992	neg	250.5459685	306.7171181	148.4979745	587.1284191	358.5601476	198.4328881	536.3915048	194.7595987	576.9413174
1.70_419.0437m/z	419.0437493	1.6992	neg	3093.719411	2953.365386	3296.987544	2200.324988	2332.385609	2026.612641	2352.471761	2282.230006	2773.719271
1.70_453.0505m/z	453.0505397	1.6992	neg	650.1088125	784.7914338	1164.321203	1548.446783	1510.094342	1360.150577	991.1603506	1413.898551	1460.168769
1.72_181.0302m/z	181.0301879	1.721216667	neg	63.54536853	153.2306522	83.4474739	16.00856759	0.001403199	0.001403199	85.57785823	61.25987656	0.001403199
1.72_182.0401m/z	182.0400885	1.721216667	neg	9021.034568	9336.244403	7991.396236	9985.574278	10002.08733	10311.19718	9488.435211	9314.870105	11289.14621
1.72_234.9919m/z	234.9918714	1.721216667	neg	1223.323051	1278.370822	937.2090837	268.0120975	193.8513002	133.6066768	130.1751056	169.9789934	177.9924848
1.72_249.0242m/z	249.0242369	1.721216667	neg	27419.94676	27512.72348	24624.1062	29626.81666	29949.14962	29592.01881	28838.18612	27977.28718	33449.56127
1.72_261.9846n	306.9831275	1.721216667	neg	2104.250962	2255.686617	2018.076165	2175.962309	2410.097539	2288.640423	2216.811983	2011.658667	3073.822798
1.72_266.0144m/z	266.0144429	1.721216667	neg	8304.800171	8708.040695	7506.944528	9256.775294	9453.573711	9191.004194	8402.395948	8896.887533	10637.33884
1.72_272.0136n	317.011647	1.721216667	neg	19668.45123	20359.50045	18369.5396	21869.78263	22450.97379	22403.63214	20512.7061	20550.46136	24626.26275
1.72_280.9618m/z	280.9618179	1.721216667	neg	626.9327396	751.9648284	449.9828025	805.4177735	659.5802079	561.4135954	733.6453556	602.2371574	1021.179055
1.72_293.0143m/z	293.0142733	1.721216667	neg	760.352474	892.7798691	343.2890297	910.0158531	891.922547	472.3266831	834.2086059	524.4209557	1040.727782
1.72_323.9756n	368.973536	1.721216667	neg	4239.006236	4980.305572	3902.202647	5998.718125	5727.3969	5470.43017	5516.445833	5052.981389	6807.43897
1.72_326.9672m/z	326.9672226	1.721216667	neg	8290.069562	7835.214593	7376.923101	8224.92494	8363.042491	8203.089257	8090.746606	7505.953752	9102.581615
1.72_328.9650m/z	328.9649582	1.721216667	neg	3152.949023	3194.192329	2716.139594	3126.534747	3280.47056	3221.043291	3092.2058	2942.94504	3891.91198
1.72_338.9201m/z	338.9201337	1.721216667	neg	827.7779682	735.9319504	410.9494531	361.9074287	527.4392188	562.809962	785.0362689	305.0703139	571.1025231
1.72_341.0271m/z	341.0270849	1.721216667	neg	668.4611678	629.844356	263.6366226	683.4670267	311.5171532	205.79685	616.5647902	169.9504519	569.0483104
1.72_349.9564n	394.9546055	1.721216667	neg	14890.41339	14440.85772	12299.59723	13928.78036	14629.9195	13223.58628	14321.31726	12969.4196	16555.60257
1.72_355.9839m/z	355.9838884	1.721216667	neg	1016.100363	989.9388006	548.6921359	945.273728	1134.193033	677.5273013	1049.877455	596.6206173	1457.332271
1.72_358.0115n	338.9936894	1.721216667	neg	22189.84066	23530.69363	19575.30169	23581.17211	25000.26257	24727.71046	23210.93163	22171.60818	27406.20007
1.72_385.0630m/z	385.0630148	1.721216667	neg	3459.651559	4077.971092	4083.292694	5353.90343	5353.016965	5259.526585	4181.780922	5121.772442	5873.843178
1.72_390.9557m/z	390.9556976	1.721216667	neg	3697.334931	3820.718968	3484.960231	4761.346667	4902.894872	4857.445926	4242.39426	4366.66838	5531.323787
1.72_406.9078m/z	406.9077779	1.721216667	neg	1376.86946	1287.015439	1241.358605	1258.374781	1241.325944	1129.267216	1351.71563	1466.752276	1639.578942
1.72_407.9884n	452.9866066	1.721216667	neg	11483.84341	12049.11986	10226.08558	12154.26907	13183.23168	12552.7233	12141.64119	12513.2509	14128.03051
1.72_416.9367m/z	416.9367323	1.721216667	neg	10990.5364	9879.287188	8431.57155	9361.333553	9442.388103	9336.838074	10291.07921	9402.154289	11882.87357
1.72_452.1678m/z	452.1677629	1.721216667	neg	800.576746	612.3232624	668.1651898	880.6709355	693.0702713	622.5461516	505.0412082	620.3604298	498.789343
1.72_458.9428m/z	458.9427848	1.721216667	neg	2381.045125	2163.143703	1980.642562	2875.64649	2673.827369	2686.973459	2455.735754	2663.071287	3145.927695
1.72_465.8763m/z	465.8762855	1.721216667	neg	0.001403199	111.5548495	182.1070772	123.0658387	0.001403199	106.1323639	175.4401928	85.27598953	174.1570365
1.72_474.9686m/z	474.9685764	1.721216667	neg	4617.565685	4645.759162	4108.631312	4997.762913	5465.424188	5185.517253	4515.355587	5135.923844	5941.999206
1.72_485.9314n	484.9241184	1.721216667	neg	12912.53738	11763.56611	9435.261901	10951.75226	11545.1756	10746.91115	11238.20026	11022.59752	13687.21354

1.72_487.0311m/z	487.0311128	1.721216667	neg	1320.238388	1458.6166	914.1585388	906.32174	820.4822703	636.3819296	934.9823149	812.8165063	1064.891555
1.72_520.9749m/z	520.9748956	1.721216667	neg	2595.836843	2454.09451	1725.626443	2356.611806	2705.047448	2546.457011	2370.379777	2577.980127	3183.560098
1.72_542.9564m/z	542.9563581	1.721216667	neg	2615.010046	3350.707475	2603.349868	3308.139911	3232.421913	3277.5379	3383.924673	3341.390627	3934.797901
1.72_552.9117m/z	552.9117219	1.721216667	neg	4283.483525	4479.670011	3609.726711	4197.434233	4126.920354	4087.873597	4212.466591	4003.043922	4869.908351
1.72_620.8992m/z	620.8991726	1.721216667	neg	3188.20939	3782.930771	3038.673488	3219.781757	2832.46828	2736.484819	3000.671755	2429.664156	3372.541178
1.74_206.9970m/z	206.996957	1.736816667	neg	792.2888624	994.0129183	771.7100534	204.5249841	218.1937395	273.0238803	396.212034	347.2076939	432.5556356
1.74_246.0443m/z	246.0443249	1.736816667	neg	1431.843845	1204.347252	1026.242019	1154.242446	1244.52338	1526.300126	1180.07974	1423.822125	1244.387252
1.74_377.1003m/z	377.1002952	1.736816667	neg	199.8556863	385.3092075	240.911604	304.9426561	161.9567823	83.70738455	260.1015934	135.1464276	0.001403199
1.74_384.9988m/z	384.9987932	1.736816667	neg	674.0949394	980.8947036	219.620371	910.1820294	866.0893551	521.833074	949.7975144	498.9169951	1054.241567
1.74_604.8722m/z	604.8721624	1.736816667	neg	1902.208615	2087.518409	1093.656782	1879.755238	1692.451272	1481.144869	2596.251115	1694.100922	2887.714312
1.75_272.0754m/z	272.0754462	1.74555	neg	782.54011	852.6852673	684.785321	499.9206825	452.379502	518.0689751	757.8807108	898.950346	962.5187138
1.75_376.8518m/z	376.8518025	1.74555	neg	58297.48942	46290.879	40639.9132	42790.99121	44440.81272	41511.96681	50980.70127	40478.23178	44213.84797
1.75_516.8375m/z	516.8375156	1.74555	neg	17010.18832	18724.90296	16100.14952	21198.72031	22446.67124	21784.81551	24880.31862	18018.45631	20558.78971
1.75_594.7810m/z	594.7809859	1.74555	neg	10919.1601	10880.71587	7949.788002	9956.427687	9547.261799	10763.08827	13461.52217	8980.518477	10775.79739
1.76_301.0568m/z	301.0567607	1.7577	neg	1668.922994	1989.151796	1471.794874	1473.604229	1227.765755	1538.743161	1548.901129	1656.135414	1774.502501
1.76_304.9035m/z	304.9034531	1.7577	neg	662.5665165	770.6529129	553.7230089	373.5399481	612.161392	685.0601051	938.2669543	631.975605	556.9289541
1.76_577.7905m/z	577.7905062	1.7577	neg	12940.88443	10004.45255	8618.301141	8743.016451	9408.480706	10633.96389	11852.96299	9175.585784	10912.66123
1.76_584.8262m/z	584.8262055	1.7577	neg	1949.414121	2301.196301	2585.763257	1713.634145	2772.375346	2784.409892	3223.445499	2539.48805	2531.631005
1.76_662.8427m/z	662.8427158	1.7577	neg	134758.6518	127477.9829	123362.0109	155288.9627	170324.2466	169683.7025	169624.5913	136904.6027	147852.6703
1.77_190.9286m/z	190.9285717	1.769966667	neg	1404221.493	1202622	1122605.792	1204261.645	1264529.651	1214328.911	1392077.577	1153217.14	1214550.495
1.77_205.9157m/z	205.9156629	1.769966667	neg	4026.780082	3815.123273	3308.098687	3171.102172	3769.670657	4522.752126	4091.113241	3997.169084	3882.556021
1.77_225.8854m/z	225.8854216	1.769966667	neg	140.041866	250.1543729	93.43934214	157.3385675	133.9146317	382.8627271	366.5626021	394.4817021	213.9027164
1.77_275.9062m/z	275.9062026	1.769966667	neg	3441.653503	3214.322731	2543.743136	1879.258778	2702.340685	3343.177361	3450.216523	3115.364297	3264.665683
1.77_511.8183m/z	511.8182597	1.769966667	neg	131722.0302	106323.4897	93043.21058	97247.90478	105968.4756	97674.32308	119987.9905	94333.87675	99426.69589
1.77_658.8102m/z	658.8102023	1.769966667	neg	8325.870632	6405.602556	6798.23066	5783.329121	5963.231039	5748.758291	8104.398814	6266.901166	7299.417644
1.77_730.8298m/z	730.8298206	1.769966667	neg	45620.30495	43584.23987	40510.09832	55473.08627	60737.12424	58295.04259	61373.27502	45566.95285	52943.95603
1.77_798.8171m/z	798.817136	1.769966667	neg	9400.344977	10022.76375	8432.514996	8478.350886	10777.59476	11506.43704	11225.69766	9489.989952	11120.62813
1.77_808.7727m/z	808.7727444	1.769966667	neg	10841.25093	11298.99815	8767.955334	9866.677377	11929.65089	12215.23798	12332.32382	9435.51115	10214.43463
1.78_472.8338m/z	472.833786	1.778766667	neg	343881.1595	271200.4157	236654.135	239900.9275	247558.3544	235325.9962	297730.3344	232681.0691	243429.3669
1.78_713.7654m/z	713.7654306	1.778766667	neg	2932.462621	2145.485184	2235.371059	872.4700881	1307.211287	1778.931633	1820.953842	2164.157217	1505.092796
1.78_740.7853m/z	740.7853296	1.778766667	neg	45635.55786	38179.85366	31429.44065	38400.29119	44212.2584	41537.16133	49602.14536	34170.75181	39141.90123
1.78_812.7718m/z	812.7717999	1.778766667	neg	6244.48063	4822.503929	4595.086135	3150.072382	4201.169969	4285.323273	5545.376886	5027.581978	5167.567647
1.79_264.8819m/z	264.8819203	1.7861	neg	4222.116821	3778.780833	3806.765243	7020.890699	7536.76731	6939.982093	5306.006654	4546.889436	4190.101614
1.79_337.0810n	336.0729529	1.7861	neg	1388.240411	1147.887812	1520.259646	1378.651778	1806.777784	2006.357748	1537.47437	1780.667366	1478.929635
1.79_404.8461m/z	404.8461094	1.7861	neg	7343.877788	5818.726751	4690.088294	4976.552889	5287.798393	5184.835917	6110.873521	5111.008495	5813.526911
1.79_433.8018m/z	433.8017753	1.7861	neg	2373.072466	2109.944057	2001.481086	1890.079064	1910.195097	2279.2307	2362.557549	1813.200874	2017.590907
1.79_540.8215m/z	540.8215064	1.7861	neg	318417.3363	253855.7022	225149.394	226750.7991	230830.2389	220943.0751	278248.3377	218435.6527	227282.304
1.80_224.0542m/z	224.0542064	1.798266667	neg	1464.492738	1385.966659	1576.567796	1535.711125	1508.553485	1732.342675	1613.002906	1717.613202	1623.125956
1.80_239.0539m/z	239.0539209	1.798266667	neg	1252.785255	1148.140616	1410.906355	1310.913457	1421.793296	1531.162155	1381.755289	1767.896403	1652.274738
1.80_264.0549m/z	264.0548584	1.798266667	neg	5199.48187	5131.006071	5431.567913	10645.15118	11488.13502	11842.30989	9757.747561	10370.75599	10114.50095
1.80_289.1277n	288.1203767	1.798266667	neg	5856.408586	5555.244841	5867.53824	10685.67418	11095.17004	12197.83108	4756.400043	5657.262187	5058.538755
1.80_292.0417m/z	292.0417401	1.798266667	neg	1136.34049	903.2471013	1154.079349	1024.965942	966.1840424	1166.623246	917.4937354	966.8044747	832.4733741
1.80_308.8642m/z	308.8641898	1.798266667	neg	93146.21481	75012.73395	65953.40729	68756.72242	69316.27611	65662.2766	81841.76954	65840.89856	68261.70565
1.80_390.1278n	435.1260002	1.798266667	neg	2374.476396	2012.949839	2637.623606	2413.634135	2661.686038	2723.479811	2415.528418	2557.786755	2344.053515
1.81_200.1161n	245.1143483	1.808733333	neg	851.2877958	974.9165594	1146.37972	1157.252357	1109.199303	1401.83415	824.5193307	934.5847864	918.9339913
1.81_279.0599m/z	279.0599107	1.808733333	neg	657.5988169	668.2339539	807.8822967	669.4047223	625.9829465	880.0869515	663.082093	691.853447	685.5303176
1.81_378.0898m/z	378.0897592	1.808733333	neg	1229.704498	877.2147801	926.5975797	2006.381324	2179.500446	2298.533215	876.4601862	964.2908701	882.0665279
1.82_207.9187m/z	207.9187417	1.820883333	neg	4475.289046	3847.72066	3642.651342	4394.674756	3867.986259	4244.387575	3675.617732	3851.944754	3943.115039
1.82_332.9699m/z	332.9698662	1.820883333	neg	770.8404717	584.1820614	637.7414215	959.7420111	763.4146947	906.1283111	885.5970404	747.4367523	946.869326
1.82_342.0744m/z	342.074374	1.820883333	neg	544.5321486	622.3100785	707.0615531	1233.197641	1454.200832	1604.980505	635.1040878	739.9991713	752.3719291
1.82_345.9923m/z	345.9923226	1.820883333	neg	775.3527088	667.2950692	861.7806855	612.9209941	457.5887972	547.6188819	1088.266929	935.0526982	1117.359932
1.82_441.8154m/z	441.8154279	1.820883333	neg	82725.65388	72461.64882	66178.715	70179.57524	72624.28136	71578.10135	82722.405	68236.36935	72993.61074
1.82_459.0882m/z	459.0882238	1.820883333	neg	797.0155972	996.8335159	1516.626137	479.2397584	357.0099154	350.9029524	1264.177459	1395.987202	1291.673042
1.84_144.9433m/z	144.9433438	1.841666667	neg	4499.319645	3709.476683	3285.844966	3457.997632	3570.171289	3645.298862	4183.861833	3255.512294	3534.343243
1.84_146.9387m/z	146.9386931	1.841666667	neg	343871.5451	294356.227	274225.4732	296339.6606	308308.8042	295553.2415	339524.6533	283286.976	296991.8644
1.84_163.9289m/z	163.9288847	1.841666667	neg	18220.15469	16766.48945	15386.07577	18100.93922	18262.40236	18327.3009	19772.81237	17068.39574	17540.64339
1.84_179.9364m/z	179.9364274	1.841666667	neg	41435.94418	34975.76396	32579.17013	35979.7727	37887.32125	36774.90223	40376.67148	33853.09659	35709.91356
1.84_182.8967m/z	182.8967424	1.841666667	neg	25620.00793	21399.56609	19251.40494	19394.08553	20731.4546	19947.02102	25175.03079	19985.18982	21356.25968
1.84_218.0493m/z	218.0493481	1.841666667	neg	52578.80714	50506.71383	64999.92203	41374.65285	41892.82541	42631.79309	70868.17957	63140.37878	63645.60858
1.84_234.9393m/z	234.9392821	1.841666667	neg	458.0531376	469.8936153	563.9353638	220.8008044	221.3054424	291.1127904	53.56720808	122.2994739	39.86319337
1.84_278.0050m/z	278.0049781	1.841666667	neg	2938.823928	2729.557555	3432.055327	3913.73498	3461.286344	3464.250905	3585.239998	3233.445105	3659.381203
1.84_318.9814n	317.9741362	1.841666667	neg	6066.21262	5195.682859	6202.329357	3972.412109	3653.210595	3731.233319	6956.894384	5735.163986	6031.197815
1.84_337.9991m/z	337.999103	1.841666667	neg	5183.336634	4930.579215	6846.354585	4820.467721	4219.687323	4586.291696	7824.855331	6366.161687	6760.383963
1.84_343.0583m/z	343.0583179	1.841666667	neg	2498.484831	2275.958118	2793.259591	285.2826506	379.7223483	409.9201667	1173.560943	979.3643112	1282.887279
1.84_343.9953m/z	343.9952798	1.841666667	neg	2538.260067	2465.921479	3101.320018	1986.912552	1698.277791	1916.701063	3858.63605	3044.525019	3103.881766
1.84_371.0145m/z	371.0145294	1.841666667	neg	924.7824887	850.6520037	1308.869976	774.7833601	827.3825872	846.5353144	999.248447	994.1763656	1204.582601
1.84_405.9858m/z	405.9858265	1.841666667	neg	1893.123692	2174.25239	2827.54539	1955.077401	1703.983055	2107.517784	3199.655713	2808.313496	2916.830885
1.84_411.9830m/z	411.9830153	1.841666667	neg	2764.072297	2510.002256	3318.766279	2051.616828	2201.176452	2176.335341	3721.204041	3135.061223	3236.459227
1.84_413.9800m/z	413.9800268	1.841666667	neg	549.8265267	586.2862511	1198.996238	371.1924455	375.7677004	563.7430197	1153.487856	877.6788268	945.2983365
1.84_427.9680m/z	427.9680463	1.841666667	neg	2271.275226	2099.838889	2971.153595	2021.126292	1891.896079	2032.264595	3029.755051	2870.877151	3153.951717
1.84_431.9671m/z	431.9671171	1.841666667	neg	5282.891656	4794.327	5887.471361	3091.611063	2935.487144	3468.292645	6655.141628	5507.407805	5448.939394
1.84_454.9564n	453.9491009	1.841666667	neg	5488.320357	4472.039025	6247.815949	3062.536302	2851.289478	2914.998537	6357.853604	4824.188951	5609.489589
1.84_479.9704m/z	479.9703956	1.841666667	neg	2383.071154	2196.586264	2543.013235	1729.586784	1498.609087	1676.19952	2583.110817	2737.942915	2508.502144
1.84_489.9994m/z	489.9994001	1.841666667	neg	11212.60825	11062.66029	14267.37523	9815.321343	9463.188491	9228.398892	17008.02336	14162.15645	13896.59824
1.84_495.9556m/z	495.955605	1.841666667	neg	1312.754761	1179.873014	1711.907933	1441.439627	1122.153735	946.1223378	1779.454161	1401.404202	1712.397079
1.84_557.9868m/z	557.9867933	1.841666667	neg	7540.270642	7266.161028	8201.654205	6010.040376	5624.19748	6378.601565	10073.08173	8790.496614	9012.445286
1.84_625.9744m/z	625.9743809	1.841666667	neg	4945.500194	4457.998982	6016.744282	3612.885553	4011.008315	4676.829216	6857.822478	6295.773413	6529.751995
1.84_693.9617m/z	693.9616847	1.841666667	neg	3265.659689	3014.172451	3890.078264	2792.69165	2656.610636	2824.563767	4709.716948	3815.097486	4464.162147
1.86_176.9807m/z	176.9806584	1.86075	neg	6872.048798	6607.494981	7484.928812	4126.048796	4095.280008	3760.300639	2171.946546	1922.855879	2054.523678
1.86_178.9043m/z	178.9043487	1.86075	neg	1263.877906	788.5206318	1003.008177	845.7657015	956.6920244	1007.695845	1251.540831	929.6099442	947.2009293
1.86_210.0384m/z	210.0384202	1.86075	neg	625.2531965	794.0504153	772.4171006	647.1691487	710.4540873	690.351558	533.6543316	665.4479923	691.7969547
1.86_244.9680m/z	244.9680377	1.86075	neg	3543.465743	3500.619794	4232.197127	2063.856677	2243.268794	1871.574452	1225.31921	1259.355856	1085.077071
1.86_315.0725m/z	315.0725373	1.86075	neg	8941.722587	9140.184046	10906.88417	1588.029283	1663.75223	1493.520619	4362.034456	3436.623642	3825.958992
1.86_380.9429m/z	380.9429473	1.86075	neg	1799.749874	1720.816473	2228.778321	1014.330615	1089.720667	784.4317044	310.498515	101.741291	344.0610405
1.86_383.0599m/z	383.0598589	1.86075	neg	2344.826962	2387.235869	2773.050537	263.8685628	416.7394891	176.4224615	917.7489282	769.4523697	985.7411082
1.86_475.8710m/z	475.8709988	1.86075	neg	2842.76729	2173.473739	2614.929871	3717.095601	4431.105928	3098.85849	3546.21876	1986.395679	3642.969175
1.86_608.8092m/z	608.8092078	1.86075	neg	281491.1919	223975.8162	197076.3918	198006.3627	207760.8408	195868.9319	246108.6786	186336.0648	198420.8164
1.87_123.9465m/z	123.9464604	1.872916667	neg	17623.83766	15612.99637	14204.46065	16640.10144	17963.84945	17606.63414	20074.0684	14998.28462	16496.3059
1.87_163.0614m/z	163.0613531	1.872916667	neg	723.4397842	664.4955299	538.1531966	732.9767508	782.5468174	653.9640829	767.3804871	616.1096151	678.6328831
1.87_312.9557m/z	312.9556816	1.872916667	neg	3069.1878	3378.058286	4140.431115	2063.502999	1934.945563	2033.822234	1058.009799	786.6061798	1002.911687
1.87_645.7776m/z	645.7776044	1.872916667	neg	16633.62442	13366.34232	12871.04545	13821.74651	15859.82703	13815.3498	16347.18477	11708.70262	14149.8863
1.89_167.0326m/z	167.0325793	1.885116667	neg	315.442572	249.9049667	320.7760319	843.9220053	686.374793	631.3609792	425.2801942	352.9617995	421.2849892
1.89_211.0226m/z	211.0225536	1.885116667	neg	2743.715228	2605.979734	2763.619877	5808.423437	5361.925145	4940.368422	3230.187038	3179.574732	3355.4895
1.89_339.9119m/z	339.9118674	1.885116667	neg	1075.060787	736.2378828	670.8188743	779.5804904	634.6925817	525.5338444	1009.737912	449.4816246	483.4039344
1.89_346.9974m/z	346.9974488	1.885116667	neg	1397.742803	1349.54534	1352.436231	3413.732911	3071.971334	2751.299467	1849.155136	1697.073572	1621.05647
1.89_414.9849m/z	414.9848634	1.885116667	neg	1094.936525	894.011729	1000.636165	2659.092518	2549.791981	2128.441655	1417.007778	1148.145691	1369.620883
1.90_191.0350m/z	191.0350043	1.899016667	neg	1214.587193	1150.567377	1108.144008	1204.323747	1085.530285	857.7012398	1367.711374	1071.325354	1133.87615
1.90_331.0109m/z	331.0108721	1.899016667	neg	1710.642662	1894.174759	1690.884891	1924.196703	1864.735921	1453.705055	1922.980585	1614.081043	1583.561271
1.90_364.0879m/z	364.0878837	1.899016667	neg	5668.669556	6337.166234	5456.132695	6241.005978	6603.815044	6077.750665	5947.277912	6171.8572	5598.521984
[truncated: 4,107,008 more chars]
